# Conformational Effects in Intramolecular C(sp^3^)–H Bond Functionalization: Gold(I)-Catalyzed Cycloisomerization of Aliphatic 1‑Bromoalkynes as Benchmark Reaction

**DOI:** 10.1021/acs.orglett.5c03430

**Published:** 2025-09-22

**Authors:** Rubén Miguélez, Omar Arto, Hannah Siera, Jan Schulte, Isabel Merino, Gebhard Haberhauer, Pablo Barrio

**Affiliations:** † Department of Organic and Inorganic Chemistry, 16763 Universidad de Oviedo, Julian Clavería 8, 33006 Oviedo, Spain; ‡ Institut für Organische Chemie, Universität Duisburg-Essen, Universitätstraße 7, 45117 Essen, Germany; § Servicios Científico Técnicos, 16763 Universidad de Oviedo, Fernando Bonguera s/n, 30006 Oviedo, Spain

## Abstract

The use of cyclohexane derivatives displaying all possible substitution patterns has allowed us to study the influence of the conformational effects in the recently reported gold­(I)-catalyzed cycloisomerization reaction of aliphatic 1-bromoalkynes that entails an unusual functionalization of a nonactivated C­(sp^3^)–H bond. Some clear trends in terms of reactivity, regio- and stereoselectivity may be drawn from the experimental results and, in most cases, rationalized using intuitive conformational analysis arguments (i.e., equatorial/axial preferences). Occasionally, some unexpected results were also obtained, but they have also found a rational explanation. DFT calculations have been used to help us understand the underlying effects that dictate the reactivity or selectivity differences.

## Graphical abstract

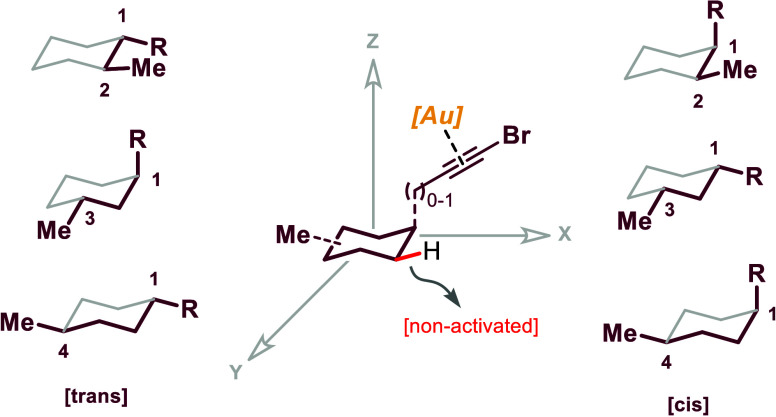

The influence exerted by conformational effects on the reactivity can hardly be overstressed. Dramatic changes in reactivity, chemo-, regio- or stereoselectivity may be found in the organic synthesis literature.^1–4^ Fortunately, a deep understanding of the underlying factors that dictate the observed conformational effects has allowed the synthetic community to use them to develop new or more selective methodologies. Although such effects have been invoked in other C—H bond activation/functionalization in the past, no systematic study has been carried out, to the best of our knowledge.^5–7^ Perhaps, the most comprehensive work in this sense was reported by Lee in 2009.^5a,5b^ However, it is focused on stereoelectronic rather than conformational effects, and only a limited number of substitution patterns were mapped (Figure 1). Our current work presented here aims to completely map how conformational effects dictate the selectivity of the functionalization of unactivated C­(sp^3^)–H bonds (Figure 1). To tackle this goal, we have selected as a model reaction our recently developed gold­(I)-catalyzed cycloisomerization of aliphatic 1-bromoalkynes,^8–11^ using the cyclohexane ring as the classical model structure in conformational analysis.^12^ In order to make our study completely comprehensive, all substitution patterns have been included with total control over the relative stereochemistry. We anticipate that our conclusions may be echoed by the related transformations.

**Figure 1:**
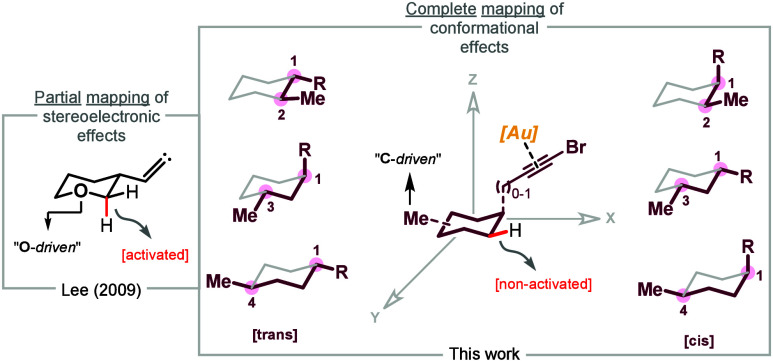
Complete mapping of conformational effects.

The significant synthetic effort behind the synthesis of the starting materials is not detailed in this article to focus the discussion on the study of the conformational effects as the main topic (see Supporting Information (SI) Experimental Procedures and Characterization for details on the stereoselective synthesis of the substrates). To show graphically both issues using a single graphical representation, the ratio of regioisomers will be represented by different colors (i.e., red and blue), while the ratio of diastereoisomers for each regioisomer will be denoted by different tones of each color (i.e., dark blue for the major diastereoisomer, generally cis, and light blue for the minor). All reactions are carried out without any deviation from the originally reported reaction conditions: [IPrAu­(NCMe)]­[SbF_6_] (2.5 mol %), CH_2_Cl_2_, 80 °C, 2 h.^8,13^ In this way the conformational effect may be studied aside from any other experimental variable.

We began our study with the so-called bridged series, bearing the bromoethynyl directly attached to the ring (Scheme 1). The complete set of results is shown in Scheme 1. Rather than comment on specific results, the main trends that may be extracted from them will be rationalized. The reactivity of model substrate **1a** was described in our previous report and will be used for comparison.^8a^ The presence of a methyl substituent in positions 1, 2-*cis*, 3-*trans* and 4-*cis* favors the reactive conformation with the bromoethynyl substituent occupying the axial position, with the A-value of methyl exceeding in more than 1 kcal/mol that of the ethynyl group.^12^ Most of these substitution patterns (*cis*-**1c**, *trans*-**1d**, ^Me^ *cis*-**1e**) resulted in improved yields (57%, 57% and 56%, respectively). However, 1-methyl substituted derivative **1b** afforded the expected product **2b** with a similar yield as model substrate **1a**. The formation of byproduct **5** in roughly 10% yield is observed for **1b** which justifies the lower yield in this case. The presence of a quaternary carbon at the propargylic position is the main differential structural feature of this substrate. Hence, we suggest that this must be key for rationalizing this unexpected result. The following preliminary mechanism has been streamlined:
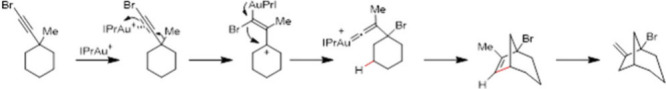
This result allowed us to surmise that new reaction pathways might be triggered by conformational effects.^14^ Conversely, the complementary substitution patterns, which favor the conformation with both substituents occupying equatorial positions (in Scheme 1 the expected reactive diaxial conformation is depicted (*trans*-**1c**, *cis*-**1d**, ^Me^ *trans*-**1e**)), result in poor yields (27%, 3% and 14%, respectively). We assume that the diminished reactivity may be attributed to the required diaxial arrangement in the corresponding reactive conformations. The almost complete shutdown of the reactivity observed for *cis*-**1d** may be rationalized because it is doubly hampered by the unfavorable *cis*-diaxial reactive conformation and the 1,3-diaxial strain imposed by the 3-methyl substituent, which shields the reactive C­(sp^3^)–H bond. DFT calculations support this hypothesis (Figures S7 and S8, SI computational details). The seemingly simple rationale used to explain the observed reactivity thus far prompted us to synthesize derivatives with increasing steric bulk to confirm our hypothesis. As expected, an increase in the steric bulk of the substituent in position 4 resulted in the complete shutdown of the reactivity in the *trans* series (Scheme 1, ^iPr^ *trans*-**1e**). More intriguing results were obtained in the *cis* series. Now increasing the size of the substituent in position 4 should reinforce its conformational lock ability, and hence a beneficial effect was anticipated. However, diminished reactivity was observed for the series ^Me^ *cis*-**1e**, ^iPr^ *cis*-**1e**, and ^tBu^ *cis*-**1e**. Unfavorable *syn*-pentane interactions may be invoked to rationalize this trend, in analogy to the 1,3-diaxial strain claimed for the lack of reactivity for *cis*-**1d**. Regarding regioselectivity in nonsymmetrical arrangements, for 2-methyl derivatives **1c** the CH_2_ contiguous to the methyl substituent proved to be more reactive in both cases, although to a different extent (2.9:1 vs 1.8:1). DFT calculations predicted this selectivity for the cis isomer (Figure S3 and SI computational details). A much more pronounced effect was observed when using the 3-methyl substituted derivative *trans*-**1d**, affording *trans*-**2d** as a single regioisomer (Scheme 1). This exquisite selectivity must be compared with the 7:1 CH vs CH_2_ selectivity obtained for a linear example in our previous report,^8^ and with a similar result reported by Zhang in a related cyclic substrate,^11j^ showcasing that conformational effects may be invoked to rationalize enhanced selectivity. A good agreement between this experimental result and its theoretical survey was achieved (Figure S5 and SI computational details).

**Scheme 1:**
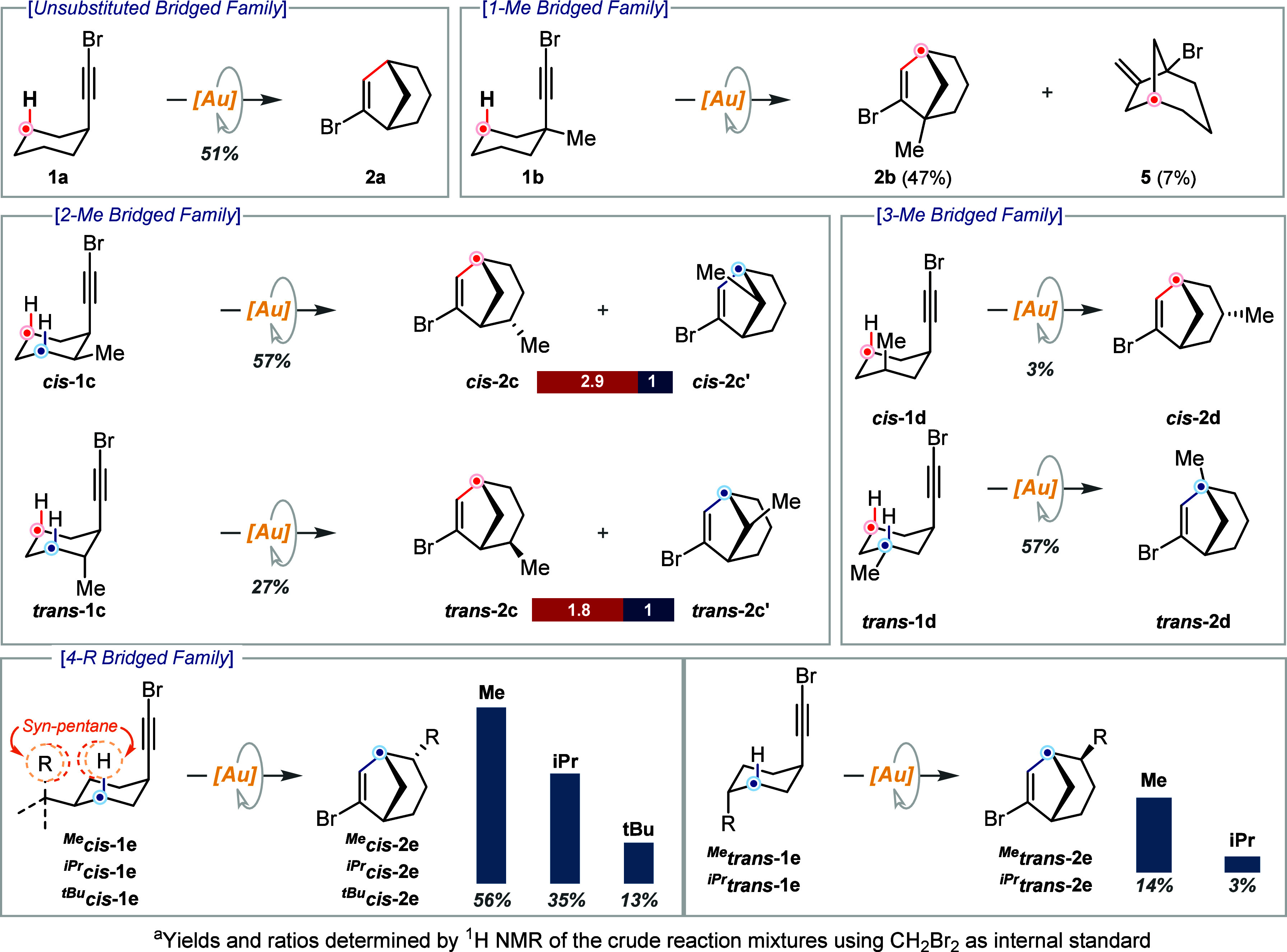
Complete Mapping of Conformational Effects*^a^*

A more complex scenario was addressed when the starting bromoalkyne and the cyclohexane were tethered by a CH_2_ moiety in the fused bicyclic series (Scheme 2). Now, in addition to regioselectivity issues, stereochemistry issues come into play. Gratifyingly, introducing a 1-methyl substituent in **3b** proved sufficient to raise the diastereoselectivity described in our previous report for the unsubstituted cyclohexane **3a** from 3:1 to >20:1 (Scheme 2).^8^ Introducing a 1-methyl substituent favors the conformation with the bromopropargyl unit in the axial position, from which only the *syn* C­(sp^3^)–H bond is available (, SI computational details). Similarly, all substitution patterns that favor the conformation with the bromopropargyl unit occupying the axial position (*cis*-**3c**, *trans*-**3d**, and ^Me^ *cis*-**3e**) resulted in improved diastereoselectivities (4.4–7.5:1). Both 2-methyl derivatives **3c** led to comparable overall yields (66% and 50%, respectively) and selectivities, favoring the insertion at the CH over the CH_2_ in a 2:1 and 1.1:1 ratio, respectively. The minor regioisomers were obtained as 4.4:1 and 3.2:1 diastereomeric mixtures (Scheme 2). The observed increased regioselectivity and decreased diastereoselectivity for the *trans* isomer may be rationalized using a quite simplistic explanation. For the *cis* isomer a conformational equilibrium is expected, increasing the ratio of bromoalkyne in the axial position, and this will favor cyclization at the syn C­(sp^3^)–H bond, as discussed above. In this case, this factor plays in favor of diastereoselectivity but against regioselectivity, since the tertiary C­(sp^3^)–H bond is available only from the opposite chair conformation. Conversely, from the preferred diequatorial conformation for the *cis* isomer the most reactive tertiary C­(sp^3^)–H bond occupies the most reactive axial position resulting in an increased regioselectivity. When the gold-activated bromoalkyne reacts with the neighboring CH_2_ from the equatorial position, both C­(sp^3^)–H bonds are available; hence, the diastereoselectivity drops. In derivatives **3d**, the presence of the methyl substituent in the adjacent position barely has any effect on the regio- or the diastereoselectivity of the process obtaining for both examples a complex mixture of the four possible products in variable ratios (Scheme 2). The regioselectivity and diastereoselectivity of this reaction (and other complex reaction mixtures) were established by integration of the characteristic olefinic signals in the ^1^H NMR spectrum, which were unambiguously assigned by selective 1D TOCSY and 1D NOESY experiments, together with careful analysis of 2D NMR experiments (see ). Finally, for *trans*-**3d**, unexpected new product **7d** was formed. The presence of this new product was inferred after examination of the ^13^C NMR spectrum, which exhibited an additional pattern of peaks in lower intensity, featuring inverse CH and C chemical shifts compared to the shifts expected for compounds **4**. In all the 5-endo cyclization products obtained so far, the quaternary carbon attached to the bromine atom appears at higher fields than the CH (typically, δ­(CH) = 135 ppm and δ­(CBr) = 120 ppm). However, in this new product, a higher field CH (δ­(CH) = 96 ppm) and a lower field quaternary center (δ­(C) = 156 ppm) suggest an exocyclic bromomethylene moiety (=CHBr). This motif must arise from a hitherto unprecedented 5-exo C­(sp^3^)–H insertion.^14^ In view of the clear trends observed for the effect of 4-substitution on compounds in the **1** series, we decided to carry out a similar study with propargyl derivatives **3**. Introducing a *cis*-4-methyl substituent in ^Me^ *cis*-**3e** stabilizes the conformation with the bromopropargyl unit in an axial conformation, affording an improved selectivity, at the expense of chemical yield (Scheme 2). Moreover, the more this conformation is fixed by using bulkier substituents, the greater the increase in reactivity and selectivity, with ^tBu^ *cis*-**4e** obtained in a remarkable 91% yield as a single diastereoisomer. Conversely, following our rationale, it was not surprising that ^Me^ *trans*-**3e** afforded ^Me^ *trans*-**4e** in poor diastereoselectivity, like unsubstituted example **3a**. For both examples, the major conformer places the bromopropargyl reactive unit in an equatorial position, from which both vicinal methylene C­(sp^3^)–H bonds are accessible. Again, this trend may be exacerbated by using bulkier conformational locks (Scheme 2, ^iPr^ *trans*-**4e**, and ^tBu^ *trans*-**4e**). While the observed trends for diastereoselectivity may be rationalized using simple conformational analysis reasoning (the more favored the axial conformation, the higher the d.r.), the seemingly clear trend in chemical yield (the more favored the axial conformation, the higher the yield) cannot be easily rationalized.

**Scheme 2:**
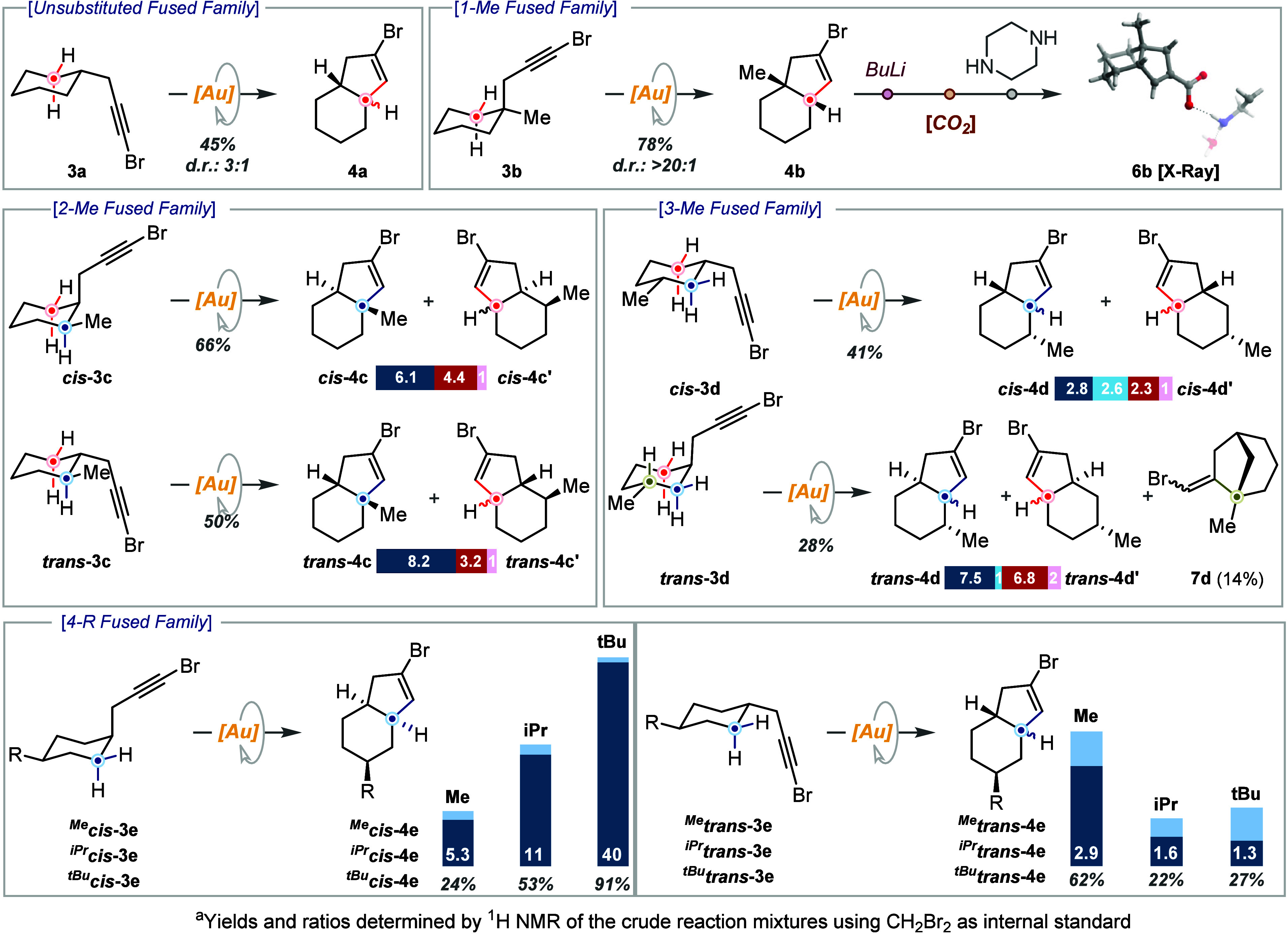
Complete Mapping of Conformational Effects*^a^*

In conclusion, a systematic study of the influence of every possible disubstitution pattern for a cyclohexane ring has allowed us to catch a glimpse of how conformational effects dictate the reactivity and hence the selectivity of C­(sp^3^)—H bond functionalizations, using the gold­(I)-catalyzed cycloisomerization of aliphatic 1-bromoalkynes as a benchmark reaction. To the best of our knowledge, no systematic study of this kind has been hitherto disclosed, especially for intramolecular reactivity. We hope that the present study may inspire related ones paving the way to a better understanding of the conformational factors governing reactivity in related settings. Multivariable regression with the aid of Machine Learning (ML) methods represents an ideal methodology to rationalize the complex interplay of countless underlying effects that account for all the experimental observations. Currently, an ambitious study aimed at elaborating a predictive mathematical model is underway.

## Supplementary Material







## Data Availability

The data underlying this study are available in the published article and its .

## Notes

Experimental procedures and characterization of new compounds (PDF)
Computational details (PDF)
NMR spectra (PDF)
