# Supplementary material for: Conformational Effects in Intramolecular C(sp3)–H Bond Functionalization: Gold(I)-Catalyzed Cycloisomerization of Aliphatic 1‑Bromoalkynes as Benchmark Reaction
Source: Org Lett. 2025 Sep 22;27(39):11065–70. doi: 10.1021/acs.orglett.5c03430 (PMC12501934; doi:10.1021/acs.orglett.5c03430)
Supplement: Supplementary file 1 [file ol5c03430_si_001.pdf]

Supporting Information

Conformational Effects in Intramolecular C(sp^3)—H bond Functionalization: Gold(I)-Catalyzed Cycloisomerization of Aliphatic 1-Bromoalkynes as Benchmark Reaction Rubén

Rubén Miguélez,[a,‡] Omar Arto,[a‡] Hannah Siera,[b] Jan Schulte,[b] Isabel Merino,[c] Gebhard Haberhauer,[b] Pablo Barrio[a*]

[a] Department of Organic and Inorganic Chemistry
Universidad de Oviedo
Julian Clavería 8 33006 Oviedo (Spain)

[b] Institut für Organische Chemie
Universität Duisburg-Essen
Universitätstraße 7, 45117 Essen (Germany)

[c] Servicios Científico Técnicos
Universidad de Oviedo
Fernando Bonguera s/n, 30006 Oviedo (Spain)

Correspondence to: barriopablo@uniovi.es

This PDF file includes:

Materials and Methods

X-ray data
References

Table of Contents

1 EXPERIMENTAL SECTION S3

1.1 MATERIAL AND METHODS S3

1.1.1 Equipment and Instruments S3

1.1.2 Methods S4

1.1.3 Chemicals S4

1.2 EXPERIMENTAL PROCEDURES S5

1.3 CHARACTERIZATION S11

2. APPENDIX S58

2.1 X-RAY DATA S58

3. LITERATURE REFERENCES S59

1. Experimental section

1.1. Material and Methods

1.1.1. Equipment and Instruments

Nuclear Magnetic Resonance (NMR) spectroscopy:

NMR spectra were recorded on a Bruker AV 600 spectrometer operating at 600.15 (^{1}H), 150.91 MHz (^{13}C), using a 5 mm PATXI ^{1}H/D-^{13}C/^{15}N inverse probe with a z-gradient coil, or on a Bruker AV 400 spectrometer operating at 400.54 (^{1}H) and 100.72 MHz (^{13}C), using a 5 mm PABBI 1H/D-BB inverse probe with a z-gradient coil, or on a Bruker AV 300 spectrometer operating at 300.13 (^{1}H), 75.46 MHz (^{13}C), using a 5 mm QNP ^{1}H/^{13}C/^{19}F/^{31}P/D probe with a z-gradient coil and equipped with an automatic sample changer. The NMR samples were prepared in $CDCl_3$ and measured at 298K (unless otherwise stated). Data are reported as follows: chemical shift (δ) in parts per million (ppm) relative to tetramethylsilane (TMS), multiplicity (s: singlet, d: doublet, t: triplet, q: quartet, sep: septet, non: nonet, dd: double doublet, dt: double triplet, m: multiplet), coupling constants (J) in Hertz (Hz) and integration. ^{13}C multiplicities were assigned by DEPT experiments. The residual solvent signals of deuterated solvents were used as internal references. All the experiments were acquired with the TOPSPIN 2.1 Bruker NMR software and the spectra analysis was conducted via the NMR processing softwares TOPSPIN 2.1 or MestReNova v.14.2.1-27684. Assignment of the NMR peaks in the ^{1}H and ^{13}C spectra was accomplished with the aid of additional 2D NMR experiments (gsCOSY, gsHSQCEd, gsHMBC, gsTOCSY, gsNOESY) and their selective 1D versions when needed (sel-1D-gsNOESY, sel-1D-gsTOCSY) which were recorded on the AV600 or AV400 spectrometers. The atom numbering of the signal assignment does not correspond to IUPAC rules.

Mass Spectrometry (MS):

High resolution mass spectra (HRMS) were measured on a high-resolution mass spectrometer IMPACT II, BRUKER (Servicios Ciefntifico Técnicos, Universidad de Oviedo) with a quadrupole and a Time-Of-Flight (TOF) tube as analyzer, using conventional Electrospray Ion Source (ESI), in full scan mode (4 eV) and positive ion polarity. The equipment employes N_2 at the nebulization step (2.4 Bar), and as drying gas (250 ºC, 6.0 L/min). Alternatively, and due to the low polarity of these compounds, some HRMS spectra were determined on an Agilent equipment using APCI(+) ionization (6545 Q-TOF, AGILENT, MS Spectrometer, Servicios Cientifico Técnicos). In some cases, HRMAS could not be obtained, since the molecules failed to ionize under any of these conditions.

1.1.2. Methods

All reactions discussed as results of this work were carried out using oven dried glassware under an atmosphere of argon (99.999%) using standard Schlenk techniques or young sealed tubes. Glassware was evacuated and further dried by heating with a heat-gun. Electric heating-stirring plates with oil baths were used for reactions at elevated temperatures. For reactions below room temperature, the reaction vessel was cooled using a JULABO FT902-Cryostat. Reaction temperatures refer to the external bath temperature. Cannulas and syringes were used for the transfer of reagents and solvents, which were flooded with inert gas (3×) before use. Purification by column chromatography was performed using manual air pressure on Geduran© Si60 silica gel (40-63 µm) from Merck KGaA. Silica gel F254 TLC plates from Merck KGaA were used for monitoring reactions, analyzing fractions of column chromatography, and measuring Rf values. To visualize the analytes, TLC plates were treated with appropriate staining solutions followed by subsequent heating.

1.1.3. Chemicals

Commercial reagents were purchased with the best quality affordable from Sigma Aldrich, TCI, Alfa Aesar and Acros Organics. Solvents purchased in technical grade quality were distilled under reduced pressure and used for purification procedures. and used without further purification unless otherwise stated.1,2-Dichloroethane was distilled from CaH_2 and THF from sodium/benzophenone. Other anhydrous solvents were purchased from commercial sources. TLC was performed on aluminum-backed plates coated with silica gel 60, with F245 indicator, and developed with phosphomolybdic acid or potassium permanganate stains. Solvents used in column chromatography were obtained from commercial suppliers and used without further purification.

1.2. *Experimental Procedures*

<u>General Procedure A</u>: *Synthesis of cyclohexanecarboxylic acids*

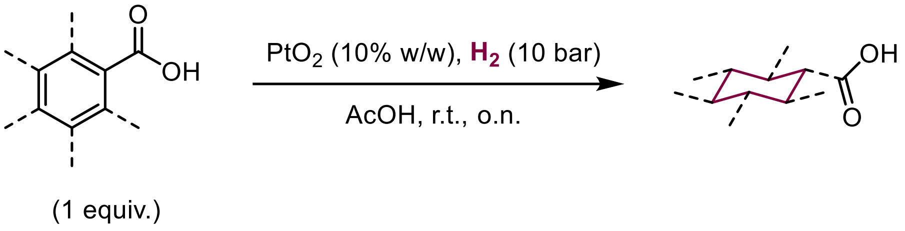

Using a slight modification of the procedure in the literature,[1] the corresponding aromatic carboxylic acid (1 equiv.) was dissolved in acetic acid (0.67 M). Then PtO_2 was added (10% w/w). The glass container was placed in a high-pressure hydrogenation apparatus. The vessel was slowly pressurized to 10 bar with hydrogen gas, then allowed to vent; and this process was repeated three times. After repressurization to 10 bar, the mixture was stirred overnight at room temperature. After this time, the vessel was slowly depressurized and opened. The mixture was filtered over a pad of celite,dissolved in Et_2O and washed five times with water to remove AcOH. The crude product was used without any purification.

<u>General Procedure B</u>: *Arnd-Eistert homologation*

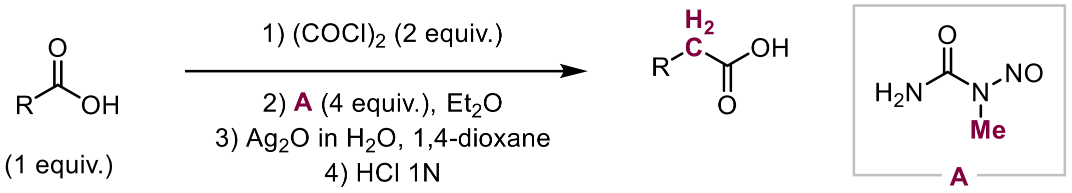

The procedure described in the literature was followed.[2]

<u>General Procedure C</u>: *Knoevenagel reaction*[3]

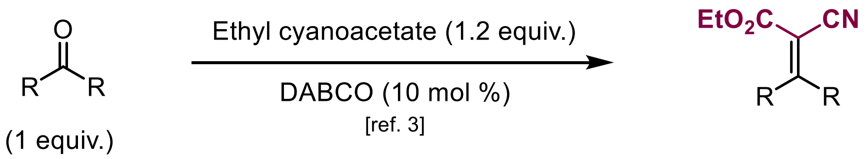

To a round bottom flask with a magnetic stir bar, the corresponding ketone (1 equiv.), ethyl cyanoacetate (1.2 equiv.), and 1,4-diazabicyclo[2.2.2]octane (DABCO, 10 mol%) were added. The resulting reaction mixture was stirred overnight at room temperature. After completion of the reaction the mixture was diluted with EtOAc and washed with water and brine. The combined organic layer was dried over anhydrous Na_2SO_4, filtered, and concentrated in vacuo. The crude product was purified by flash column chromatography using Hexane/EtOAc (10:1 to 5:1) as the eluent to yield the corresponding products.

General Procedure C': *Knoevenagel reaction*[4]

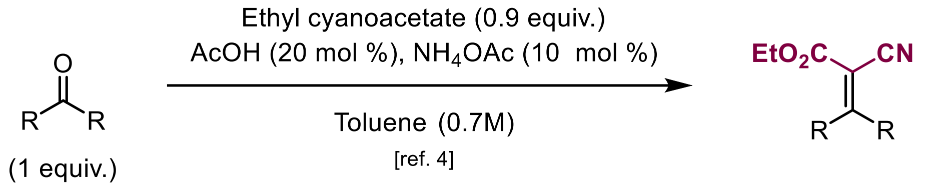

A mixture of ethyl cyanoacetate (0.9 equiv.), the corresponding ketone (1 equiv.), ammonium acetate (10 mol %), and glacial acetic acid (20 mol %) in toluene (0.7M) was refluxed for 4 h using a Dean-Stark water separator. The mixture was washed with water, dried over anhydrous sodium sulfate, and concentrated. The crude product was purified by flash column chromatography using Hexane/EtOAc (10:1 to 5:1) as the eluent to yield the corresponding products.

General Procedure D: 1,4-addition to α,β-unsaturated compounds[3]

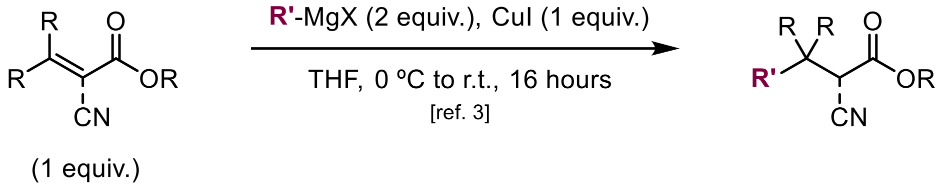

To a suspension of copper iodide (1.0 equiv.) in THF (0.5M) the corresponding alkyl magnesium bromide (2.0 equiv.) in THF was added dropwise at 0 ºC. After stirring for 2 h at the same temperature, ethyl 3-methylcrotonate (1.0 equiv.) was added. The reaction mixture was slowly warmed to room temperature and stirred overnight. The reaction was quenched with sat. $NaHCO_3$ (aq.) and filtered through a pad of celite. After removing copper salts from the mixture, the reaction mixture was diluted with EtOAc and washed with water and brine. The combined organic layers were dried over anhydrous Na_2SO_4, filtered, and concentrated in vacuo. In most cases, esters synthesized through this procedure were pure enough for the next step.

General Procedure E: Hydrolysis of cyanoacetate derivatives[3]

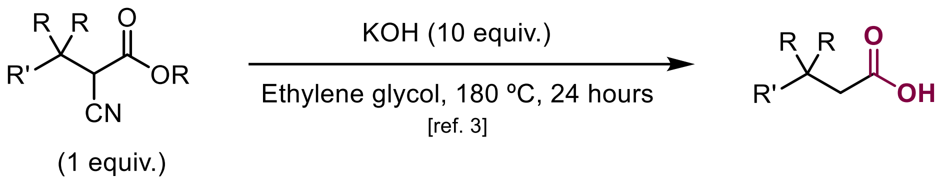

A clean, oven-dried screw cap reaction tube with a magnetic stir–bar was charged with the cyanoacetate derivative (1 equiv.) and KOH (10.0 equiv.), followed by the addition of ethylene glycol (1M). The reaction mixture was sealed tightly and was vigorously stirred for 24 h in a preheated oil bath at 180 °C. After the stipulated time, the reaction mixture was cooled to room temperature and diluted with water. The diluted reaction mixture was acidified with 2N HCl to pH 3 and then extracted with EtOAc three times. The combined organic layers were dried over anhydrous Na_2SO_4 and concentrated in vacuo. The crude product was purified through flash column chromatography using Hexane/EtOAc as the eluent to provide corresponding acid.

General Procedure F: *Horner-Wadsworth-Emmons reaction*

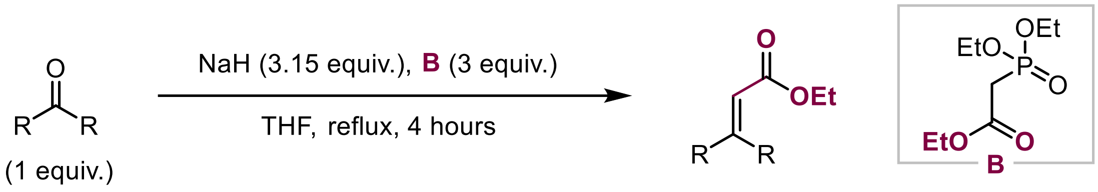

The procedure was followed as described in the literature, with a slide modification.[5] In a round bottom flask with a magnetic stir bar under Ar, the corresponding phosphonate reagent (B) in THF (0.8M) was added dropwise to a suspension of NaH in THF (2,46M). The mixture was stirred for 30 minutes. Then the corresponding ketone (1 equiv.) in THF (0.33M) was added dropwise at 0 ºC. The reaction mixture was stirred for 4 hours at reflux. After completion of the reaction, water was added to the mixture after it was allowed to reach room temperature. The crude reaction mixture was extracted with Et_2O (x3) and washed with brine. The combined organic layers were dried over Na_2SO_4, filtered and the solvent removed in vacuum. The crude mixture was purified by flash column chromatography on silica gel using Hexane/EtOAc as eluents to afford the corresponding conjugated esters.

General Procedure G: *Hydrogenation of alkenes*

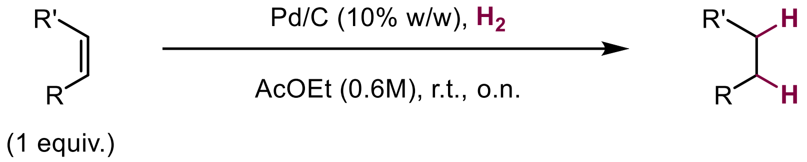

A round bottom flask with a magnetic stir bar and Pd/C (10 w/w%) was evacuated to vacuum and filled with Ar (x3). The corresponding alkene (1 equiv.) diluted in AcOEt (0.6 M) was added and hydrogen was bubbled for 15 minutes. The reaction mixture was maintained on a hydrogen atmosphere and stirred overnight at room temperature. The day after, the solids were filtered through a pad of celite. Removal of solvents under vacuum afforded the products in sufficient purity to be used in the next step without further purification.

General Procedure H: *Synthesis of alcohols*

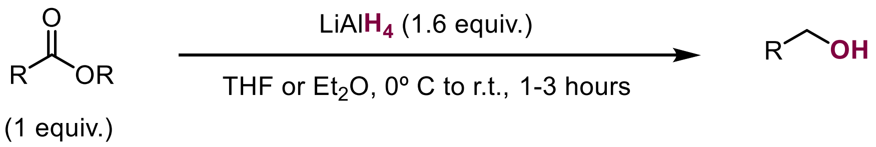

At 0 ºC, a solution of the corresponding carboxylic acid or ester (1 equiv.) in dry THF (1 M) was added dropwise to a suspension of $LiAlH_4$ (1.6 equiv.) in dry THF (0.4 M). After the addition was complete the reaction mixture was stirred for 1-3 hours at room temperature. Then, the reaction was carefully quenched by dropwise addition of water (1mL per gram of LAH), NaOH 15% (1mL per gram of LAH) and water (3mL per gram of LAH) at 0 ºC. Solids were filtered through alternate pads of celite /silica gel/ celite and removing of the solvents by rotary evaporation afforded the crude alcohol that was used in the next step without further purification.

General Procedure I: *Synthesis of 1-bromoalkanes*[6]

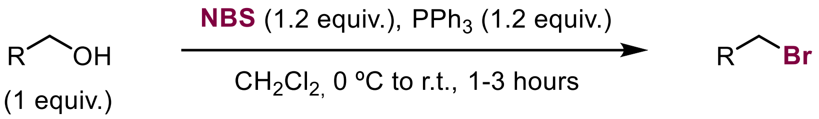

In a Schlenk flask under argon, PPh_3 (1.2 equiv.) was added to a solution of the corresponding alcohol (1 equiv.) in anhydrous DCM (0.5M). The mixture was taken to 0 ºC and NBS (1.2 equiv.) was added portion wise. Then, the reaction was taken to room temperature and upon finishing (1-3 hours, monitored by TLC) the solvent was removed under vacuum and the crude reaction mixture purified by means of flash column chromatography on silica gel using n-hexane or n-pentane as eluents affording the corresponding primary bromide.

General Procedure J: *Synthesis of 1-alkynes*

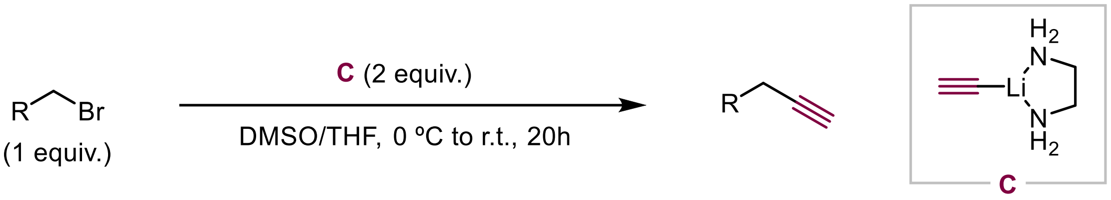

The procedure described in the literature was followed, with a slide modification.[6] Lithium acetylide (C) (2 equiv.) was weighed in a flame dried Schlenk flask under argon. Anhydrous DMSO (1 mL per mmol of C) and THF (0.5 mL per mmol of C) were added, and the reaction mixture was taken to 0 ºC. A solution of the corresponding primary bromide (1 equiv.) in THF (0.5 mL per mmol) was added at once. The reaction mixture was then stirred overnight at room temperature. The day after, the reaction was quenched with saturated NH_4Cl solution, extracted with Et_2O (x3) and washed with brine (x5). The organic layer was dried over Na_2SO_4 and filtered by a pad of silica gel using n-hexane as eluent.

General Procedure K: *Synthesis of 1-bromoalkynes*[6]

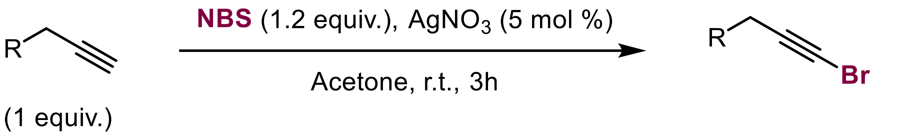

In a Schlenk flask under Ar, NBS (1.2 equiv.) was added to a solution of terminal alkyne (typically 1 mmol, 1 equiv.) in Acetone (0,5M). Then, $AgNO_3$ (5 mol %) was added and the reaction mixture was stirred for 3h in the dark. Acetone was removed in a rotatory evaporator and the crude reaction mixture purified by means of flash column chromatography (typically hexane was used as eluent).

General Procedure L: *Mesylation of alcohols*[7]

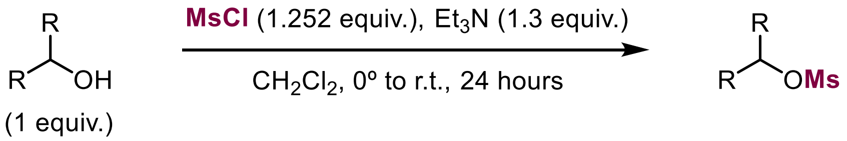

A solution of the corresponding alcohol (1 equiv.) in anhydrous DCM (0.5M), was cooled to 0ºC. At this temperature, MsCl (1.252 equiv.) was added. After stirring for 20 minutes triethylamine (1.3 equiv.) was added dropwise and the reaction was allowed to reach room temperature. The reaction was stirred 24 h and then it was diluted with DCM. The crude reaction mixture was washed with water (two times), saturated aqueous $NaHCO_3$ and saturated aqueous NaCl. The combined organic layers were dried over Na_2SO_4 and concentrated under vacuum. The crude mesylates were used in the following step without further purification.

General Procedure M: *S_N2 reaction. Cyanation*[7]

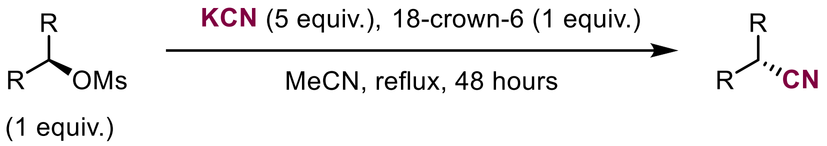

In a round bottom flask with a magnetic stir bar under Ar, the corresponding mesylate (1 equiv.) was dissolved in MeCN (0.3M), followed by the addition of KCN (5 equiv.) and 18-crown-6 (1 equiv.). The resulting reaction mixture was stirred for 48 hours at reflux. After completion of the reaction, the mixture was allowed to reach room temperature and concentrated under vacuum. The resulting mixture was diluted with DCM, washed with water (x3) and brine (x3). The organic layer was dried over Na_2SO_4, filtered through alternate pads of celite /silica gel/ celite and the solvent was removed in vacuum. The crude cyanides were obtained in sufficient purity to be used in the next step without further purification or they were purified by flash column chromatography on silica gel using n-hexane/EtOAc mixtures as eluents.

General Procedure N: *Synthesis of aldehydes (Cyanide reduction)*[8]

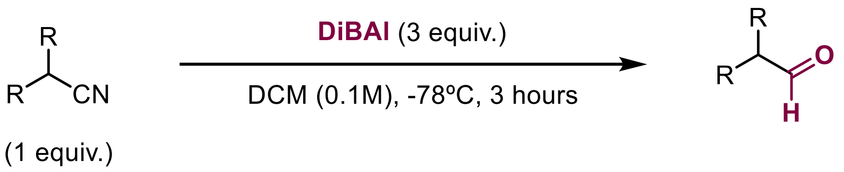

In a Schlenk flask under argon, DiBAl (1.2M in toluene, 2.5 equiv.) was added dropwise to a solution of the corresponding cyanide (1 equiv.) in DCM (0.1M), at -78 ºC. After completion of the reaction an aqueous solution of Rochelle's salt (0.13M) was added dropwise. Upon completion of the addition, the reaction mixture was allowed to warm to room temperature, extracted with DCM (x3) and washed with brine. The combined organic layers were dried over Na_2SO_4, filtered and the solvent removed in vacuum. The aldehydes obtained were used in the next step without further purification.

General Procedure O: *Synthesis of aldehydes (Homologation reaction)*[9]

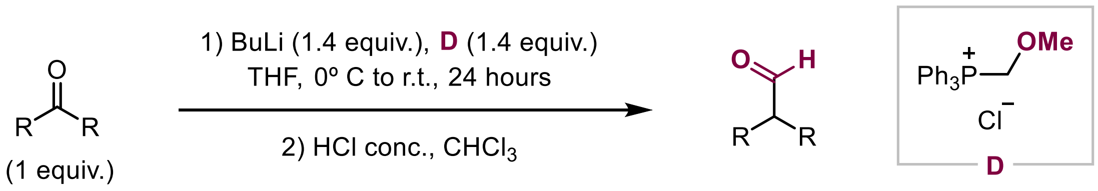

To a round bottom flask with a magnetic stir bar, BuLi (1.4 equiv., 1.6M or 2.5M in hexane) was added dropwise to a solution of methoxymethyltriphenylphosphonium chloride (C) in THF (0.4M) at 0 ºC. The mixture was stirred for 45 minutes and the corresponding ketone (1 equiv.) was added dropwise at the same temperature. Then, the resulting reaction mixture was stirred for 24 hours at room temperature. After completion of the reaction the mixture was quenched with HCl 1N at 0 ºC and it was extracted with diluted with Et_2O three times and washed with brine. The combined organic layers were dried over anhydrous Na_2SO_4, filtered, concentrated until almost dryness and hexane was added to precipitate the solids. The crude product was filtered through a pad of celite. The mixture was concentrated, redissolved in $CHCl_3$ and concentrated aqueous HCl was added at 0 ºC. The mixture was stirred at room temperature, the evolution of the reaction was followed by TLC chromatography (ca. 4 hours). Then, the solvent was removed, diluted with water and extracted with Et_2O three times. The crude was used without any purification, or the aldehyde was distilled under reduced pressure.

General Procedure P: *Synthesis of aldehydes "Swern Oxidation"*

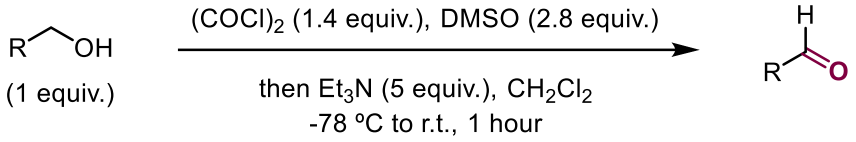

The procedure described in the literature was followed.[6] In a Schlenk flask under Ar, DMSO (2.8 equiv.) was added dropwise to a solution of oxalyl chloride (1.4 equiv.) in dry DCM (0.5M) at -78 ºC. Then, the corresponding alcohol (1 equiv.) dissolved in dry DCM (1M) was added dropwise to the reaction mixture at the same temperature. After stirring for 15 minutes triethylamine (5 equiv.) was added dropwise and the reaction was allowed to reach room temperature. Then, water was added, and the crude reaction mixture was extracted with Et_2O (x3), the combined organic layers were washed with 1N HCl, saturated aqueous $NaHCO_3$ and saturated aqueous NaCl. The combined organic layers were dried over Na_2SO_4 and concentrated under vacuum. The crude aldehydes were used in the following step without further purification.

General Procedure Q: *Synthesis of 1,1-dibromoalkenes "Corey-Fuch Reaction"*

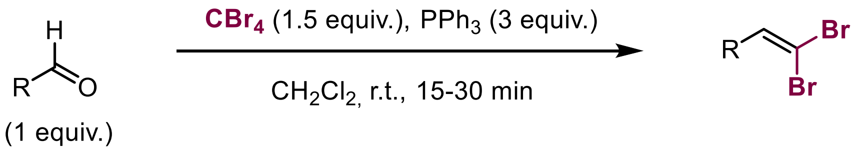

The procedure described in the literature was followed.[6] To a solution of the corresponding aldehyde (1 equiv.) in anhydrous dichloromethane (0.1M), CBr_4 (1.5 equiv.) was added followed by PPh_3 (3 equiv.). The reaction mixture was stirred for 15-30 minutes and then hexane was added. Solids were filtered through alternate pads of celite /silica gel/ celite. Removal of solvents under vacuum afforded crude 1,1-dibromoalkenes in sufficient purity to be used in the next step without further purification or they were purified by flash column chromatography on silica gel using n-hexane as eluent.

General Procedure R: *Synthesis of 1-bromoalkynes*

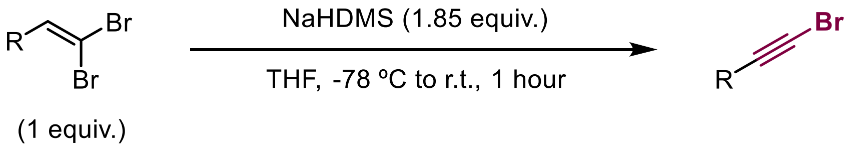

The procedure described in the literature was followed.[6] In a Schlenk flask under argon, NaHMDS (1M in THF, 1.85 equiv.) was added dropwise to a solution of the corresponding dibromoolefin (1 equiv.) in THF (0.4M), at -78 ºC. When the addition was complete, the cooling bath was removed, and the reaction mixture was stirred for one further hour. After this time, the reaction was quenched with NH_4Cl sat. extracted with Et_2O (x3) and the combined organic layers washed with water and brine. The organic layer was dried over Na_2SO_4, filtered and the solvent removed in vacuum. The final 1-bromoalkynes were purified by flash column chromatography on silica gel using n-hexane or n-pentane as eluents.

General Procedure S: Cycloisomerization of 1-bromoalkynes

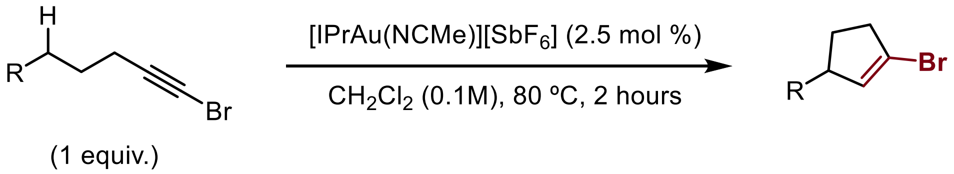

The procedure was followed as described in the literature.[6] [IPrAu(CNMe)][SbF_6] (2.5 mol%) was weighted in a flamed pressure Schlenk under Ar and dissolved in the least amount of dry DCM. Then, the 1-bromoalkyne (1 equiv.) was added. Finally, the remaining dry DCM (to achieve a final 0.1M concentration) was added and the reaction mixture was stirred at 80 ºC for 2 hours. After that time, it was cooled down to room temperature and concentrated on a rotary evaporator under reduced pressure. The crude was purified by flash chromatography to yield the pure compound using hexane or pentane as eluents.

1.3. Characterization

1.3.1. 1-Methyl Family Bridged

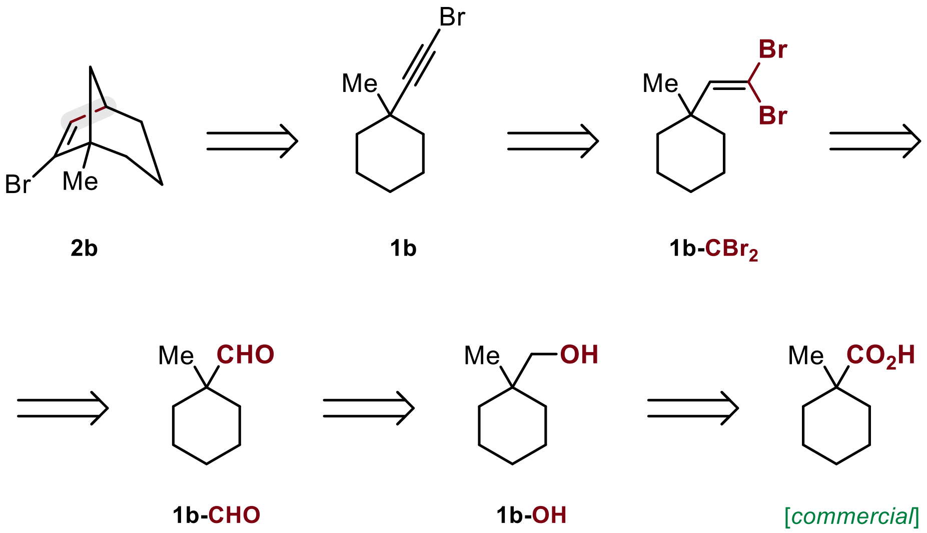

(1-methylcyclohexyl)methanol **(*1b-OH*)**

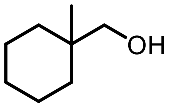

The title compound was synthesized using General Procedure H, starting from 5 mmol (711 mg) of *1-methylcyclohexane-1-carboxylic acid*, yielding the crude alcohol (***1b-OH***) as a colorless liquid (636 mg, 5 mmol, 100%). This compound was used without further purification.

^{1}H NMR (300 MHz, $CDCl_3$) δ 3.32 (d, J = 4.9 Hz, 2H), 1.94 – 1.65 (m, 1H), 1.44 (tt, J = 7.8, 3.8 Hz, 5H), 1.27 (dt, J = 10.6, 4.4 Hz, 5H), 0.89 (d, J = 3.6 Hz, 3H).

^{13}C NMR (75 MHz, $CDCl_3$) δ 72.6 (CH_2), 35.1 (C), 34.2 (CH_2), 26.6 (CH_2), 22.1 (CH_3), 21.9 (CH_2).

HRMS not purified

1-methylcyclohexane-1-carbaldehyde **(*1b-CHO*)**

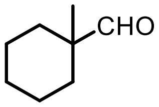

The title compound was synthesized using General Procedure P, starting from 5 mmol (636 mg) of the alcohol (***1b-OH***), yielding the crude aldehyde (***1b-CHO***) as a colorless liquid (384 mg, 3 mmol, 61%). This compound was used without purification. The ^{1}H NMR spectrum matches the one described in the literature.[10]

^{1}H NMR (300 MHz, $CDCl_3$) δ 9.43 (s, 1H), 1.94 – 1.73 (m, 2H), 1.65 – 1.14 (m, 9H), 1.00 (s, 3H).

HRMS not purified

1-(2,2-dibromovinyl)-1-methylcyclohexane **(*1b-CBr₂*)**

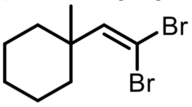

The title compound was synthesized using General Procedure Q, starting from 3 mmol (384 mg) of the aldehyde (***1b-CHO***), yielding the dibromoolefin (***1b-CBr_2***) as a colorless liquid (538 mg, 1.9 mmol, 63%). The crude was purified by flash column chromatography using n-hexane as eluent.

^{1}H NMR (300 MHz, $CDCl_3$) δ 6.54 (s, 1H), 2.01 – 1.87 (m, 2H), 1.63 – 1.47 (m, 3H), 1.17 (s, 9H).

^{13}C NMR (75 MHz, CDCl3) 146.1 (CH), 85.4 (C), 39.9 (C), 38.2 (CH_2), 25.8 (CH_2), 22.9 (CH_3).

HRMS unstable

1-(bromoethynyl)-1-methylcyclohexane **(*1b*)**

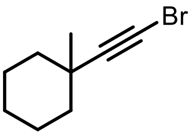

The title compound was synthesized using General Procedure R, starting from 1.9 mmol (538 mg) of dibromoolefin ***1b-CBr_2***, yielding bromoalkyne ***1b*** as a colorless liquid (282 mg, 1.4 mmol, 73%). The crude was purified by flash column chromatography using pentane as eluent.

^{1}H NMR (300 MHz, $CDCl_3$) δ 1.57 (dt, J = 9.4, 3.5 Hz, 7H), 1.19 (s, 6H).
^{13}C NMR (75 MHz, $CDCl_3$) δ 86.8 (C), 39.3 (CH_2), 38.7 (C), 34.2 (C), 30.1 (CH_3), 25.9 (CH_2), 23.4 (CH_2).
HRMS (GC-Q-TOF) m/z: $[M]^+$ calcd for $C_9H_{13}Br$: 200.0201; found: 200.0199.

***(1R,5R)-7-bromo-1-methylbicyclo[3.2.1]oct-6-ene* (*2b*)**

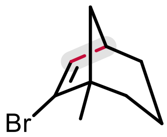

The title compound was synthesized using General Procedure S, starting from 0.2 mmol (49 mg) of bromoalkyne ***1b***), yielding the bromocyclopentene (***2b***) in *47% yield by ^{1}H NMR analysis of the crude reaction mixture using CH_2Br_2 as internal standardstandard* along with byproduct **5** (7%). The crude was purified by column chromatography using n-hexanehexanes as eluent to afford the title compound as a colorless liquid (22 mg, 0.09 mmol, 48%).
^{1}H NMR (300 MHz, $CDCl_3$) δ 5.5 (dp, J = 2.9, 1.5 Hz, 1H), 2.6 (dddd, J = 18.6, 8.7, 5.1, 2.5 Hz, 2H), 2.0 (ddt, J = 10.3, 6.6, 3.3 Hz, 3H), 1.8 (s, 3H), 1.6 – 1.2 (m, 4H).
^{13}C NMR (75 MHz, $CDCl_3$) δ = 142.3 (C), 126.5 (CH), 73.7 (C), 56.2 (CH_2), 40.3 (CH), 36.4 (CH_2), 24.1 (CH_2), 22.1 (CH_2), 14.3 (CH_3).
HRMS (GC-Q-TOF) m/z: $[M]^+$ calcd for $C_9H_{13}Br$: 200.0201; found: 200.0199.

***(1S,5S)-1-bromo-7-methylenebicyclo[3.2.1]octane* (*5*)**

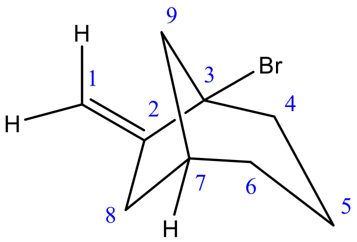

1H NMR (298K, 600 MHz, $CDCl_3$, δ ppm) δ 5.36 [t, J = 2.7 Hz, 1H], 5.14 [t, J = 2.3 Hz, 1H], 2.57 [ddt, J = 16.5, 4.7, 2.7 Hz, 1H, H8ec (J_{8gem}=16.5, J_{8ec-7}=4.7 Hz, J_{8-1}=$J_{8-1'}$= 2.7Hz)], 2.38 – 2.31 [m, 2H, H7, H9], 2.29 [dq, 1H, H8ax (J_{8gem}=16.5, J_{8-1}=$J_{8-1'}$=J_{8ax7}= 2.2Hz)], 2.16 [td, J = 12.2, 5.5 Hz, 1H, H4ax (J_{gem}=$J_{4ax-5ax}$=12.2 Hz, $J_{4ax-5ec}$=5.5 Hz)], 2.11 [ddd, J = 12.2, 5.7, 2.7 Hz, 1H, H4ec (J_{gem}=12.2 Hz, $J_{4ec-5ax}$=5.5 Hz, $J_{4ec-5ec}$=2.7 Hz)], 2.03 [m, 1H, H9' (J_{gem}= 10.2 Hz, $J_{9'-7}$=2.9 Hz) + impurity], 1.72 – 1.55 [m, 3H, H6ax +H6ec+H5ec], 1.51 [m, 1H, H5ax].
^{13}C NMR (298K, 600 MHz, $CDCl_3$, δ ppm) δ = 153.7 (C, C2), 108.7 (CH_2, C1), 68.4 (C, C3), 50.4 (CH_2, C9), 46.0 (CH_2, C4), 35.6 (CH_2, C8), 33.8 (CH, C7), 29.9 (CH_2, C5), 21.8 (CH_2), C6).
HRMS by-product obtained in 10% yield

1.3.2. (cis)-2-methyl Family Bridged

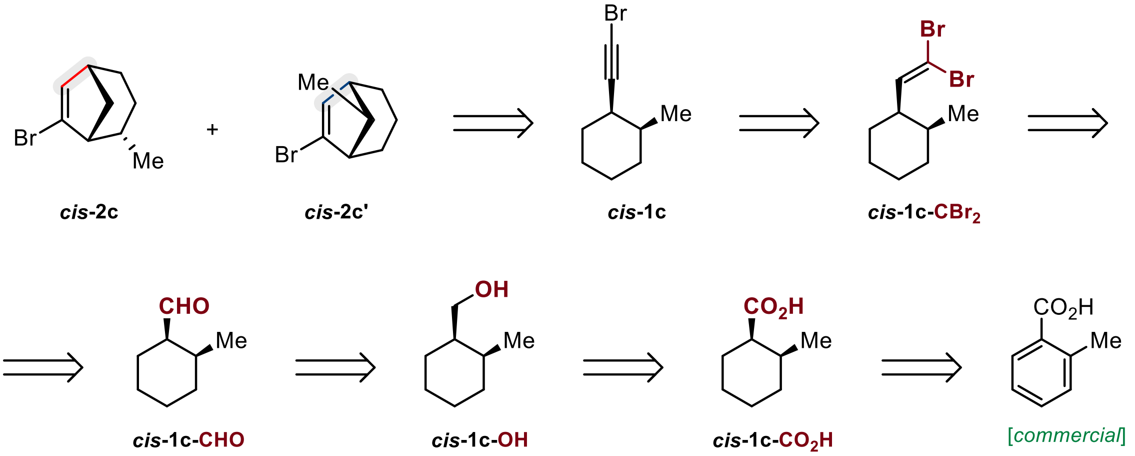

(*1R,2S)-2-methylcyclohexane-1-carboxylic acid* (*cis-1c-CO_2H*)

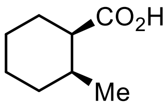

The title compound was synthesized using General Procedure A, starting from 10 mmol (1.4 g) of *o-toluic acid*, yielding acid ***cis-1c-CO_2H*** as a colorless liquid (1.4 g, 10 mmol, quantitative). The crude acid was used in the next step without further purification.
^{1}H NMR (300 MHz, $CDCl_3$) δ 11.14 (s, 1H), 2.55 (dt, J = 9.1, 4.5 Hz, 1H), 2.27 – 2.14 (m, 1H), 1.80 – 1.59 (m, 4H), 1.59 – 1.46 (m, 2H), 1.46 – 1.35 (m, 1H), 1.28 (dd, J = 8.9, 4.4 Hz, 1H), 0.96 (d, J = 7.1 Hz, 3H).
HRMS not purified

(*(1R,2S)-2-methylcyclohexyl)methanol* (*cis-1c-OH*)

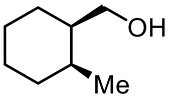

The title compound was synthesized using General Procedure H, starting from 2 mmol (284 mg) of carboxylic acid ***cis-1c-CO_2H***, yielding alcohol ***cis-1c-OH*** as a colorless liquid (208 mg, 1.6 mmol, 81%). The crude alcohol was used in the next step without further purification. The ^{1}H NMR spectrum matches the one previously reported.[11]
^{1}H NMR (300 MHz, $CDCl_3$) δ 3.62 – 3.43 (m, 2H), 1.97 (dt, J = 7.3, 3.7 Hz, 1H), 1.80 – 1.59 (m, 2H), 1.57 – 1.36 (m, 4H), 1.27 (dd, J = 17.7, 8.2 Hz, 3H), 0.89 (d, J = 7.2 Hz, 3H).

(*1R,2S)-2-methylcyclohexane-1-carbaldehyde* (*cis-1c-CHO*)

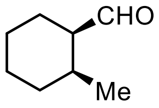

The title compound was synthesized using General Procedure P, starting from 1.6 mmol (208 mg) of alcohol ***cis-1c-OH***, yielding aldehyde ***cis-1c-CHO*** as a colorless liquid (201 mg, 1.6 mmol, 99%). The crude aldehyde was used in the next step without further purification. NMR spectra match those previously reported.[11]
^{1}H NMR (300 MHz, $CDCl_3$) δ 9.73 (d, J = 1.1 Hz, 1H), 2.36 (dtd, J = 8.3, 4.1, 1.2 Hz, 1H), 2.20 (d, J = 8.0 Hz, 1H), 1.71 (s, 1H), 1.65 – 1.43 (m, 6H), 1.37 (ddd, J = 6.8, 4.9,

2.8 Hz, 2H), 0.97 (d, J = 7.1 Hz, 3H).**^{13}C NMR** (75 MHz, $CDCl_3$) δ 205.9 (CH), 53.1 (CH), 32.3 (CH_2), 30.5 (CH_2), 24.2 (CH_2), 22.6 (CH_2), 19.7 (CH), 16.0 (CH_3).

(1R,2S)-1-(2,2-dibromovinyl)-2-methylcyclohexane (*cis-1c-CBr$_2$*)

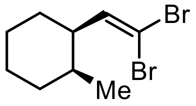

The title compound was synthesized using General Procedure Q, starting from 1.6 mmol (201 mg) of aldehyde ***cis-1c-CHO***, yielding dibromoolefin ***cis-1c-CBr$_2$*** as a colorless liquid (294 mg, 1.1 mmol, 69%, dr = 17:1 (*cis*:*trans*)). The crude was purified by column chromatography using n-hexanehexanes as eluent.
^{1}H NMR (300 MHz, $CDCl_3$) δ 6.51 (d, J = 9.8 Hz, 1H), 2.57 (ddt, J = 9.7, 5.9, 4.1 Hz, 1H), 1.87 – 1.71 (m, 1H), 1.62 (d, J = 4.4 Hz, 2H), 1.57 – 1.38 (m, 4H), 1.38 – 1.18 (m, 3H), 0.87 (d, J = 7.0 Hz, 4H).
^{13}C NMR (75 MHz, $CDCl_3$) δ 140.2 (CH), 87.8 (C), 44.7 (CH), 33.8 (CH), 31.1 (CH_2), 29.2 (CH_2), 24.4 (CH_2), 22.9 (CH_2), 18.1 (CH_3).
HRMS (GC-Q-TOF) m/z: $[M]^+$ calcd for $C_9H_{14}Br_2$: 279.9462; found: 279.9459.

(1R,2S)-1-(bromoethynyl)-2-methylcyclohexane (*cis-1c*)

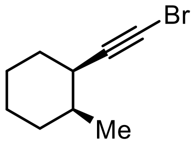

The title compound was synthesized using General Procedure R, starting from 1.1 mmol (294 mg) of dibromoolefin ***cis-1c-CBr$_2$***, yielding bromoalkyne ***cis-1c*** as a colorless liquid (143 mg, 0.7 mmol, 68%). The crude was purified by column chromatography using n-hexanehexanes as eluent.
^{1}H NMR (300 MHz, $CDCl_3$) δ 2.65 (q, J = 4.0 Hz, 1H), 1.89 – 1.75 (m, 1H), 1.75 – 1.58 (m, 2H), 1.58 – 1.13 (m, 6H), 0.97 (dd, J = 6.7, 0.6 Hz, 3H).
^{13}C NMR (75 MHz, $CDCl_3$) δ 81.9 (C), 39.4 (C), 35.5 (CH), 35.0 (CH), 31.2 (CH_2), 30.5 (CH_2), 25.7 (CH_2), 22.0 (CH_2), 20.2 (CH_3).
HRMS (GC-Q-TOF) m/z: $[M]^+$ calcd for $C_9H_{13}Br$: 200.0201; found: 200.0201.

(1R,2S,5R)-7-bromo-2-methylbicyclo[3.2.1]oct-6-ene (cis-2c) and (1S,5R,8S)-6-bromo-8-methylbicyclo[3.2.1]oct-6-ene (cis-2c')

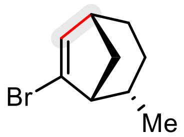

***cis*-2c**

minor

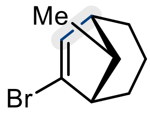

***cis*-2c'**

major

The title compounds were synthesized using General Procedure S, starting from 0.2 mmol (40 mg) of bromoalkyne ***cis-1c***, yielding bromocyclopentenes ***cis-2c'*** and ***cis-2c*** in *57% combined yield by ^{1}H NMR analysis of the crude reaction mixture using CH_2Br_2 as Internal standard* as a mixture of regioisomers [2.9 (***cis-2c'***):1(***cis-2c***].The crude was purified by column chromatography using n-hexanehexanes as eluent to afford the title compounds as a colorless liquid (23.6 mg, 0.12 mmol, 59%).

^{1}H NMR (400 MHz, $CDCl_3$) δ = 6.0 (dd, *J*=3.1, 1.0 Hz, 1H, ***minor***), 5.8 (dd, *J*=3.0, 1.2 Hz, 1H, ***major***), 2.5 (dt, *J*=5.9, 3.0 Hz, 1H, ***minor***), 2.4 (dd, *J*=5.6, 1.9 Hz, 1H, ***minor***), 2.3 (q, *J*=3.1 Hz, 2H, ***major***), 2.3 (t, *J*=2.9 Hz, 2H, ***major***), 1.8 – 1.7 (m, 1H), 1.6 – 1.3 (m, 8H), 1.0 (d, *J*=6.8 Hz, 1H), 1.0 (d, *J*=6.6 Hz, 3H).

^{13}C NMR (101 MHz, $CDCl_3$, ***major***) δ = 130.4 (CH), 121.9 (C), 54.4 (CH), 50.6 (CH), 47.7 (CH), 26.4 (CH_2), 24.8 (CH_2), 18.2 (CH_3), 18.0 (CH_2).

^{13}C NMR (101 MHz, $CDCl_3$, ***minor***) δ = 133.4 (CH), 121.3 (C), 52.4 (CH), 46.4 (CH_2), 40.3 (CH), 32.3 (CH), 28.1 (CH_2), 25.4 (CH_2), 21.2 (CH_3).

HRMS (GC-Q-TOF) m/z: $[M]^+$ calcd for $C_9H_{13}Br$: 200.0201; found: 200.0201.

1.3.3. (trans)-2-methyl Family Bridged

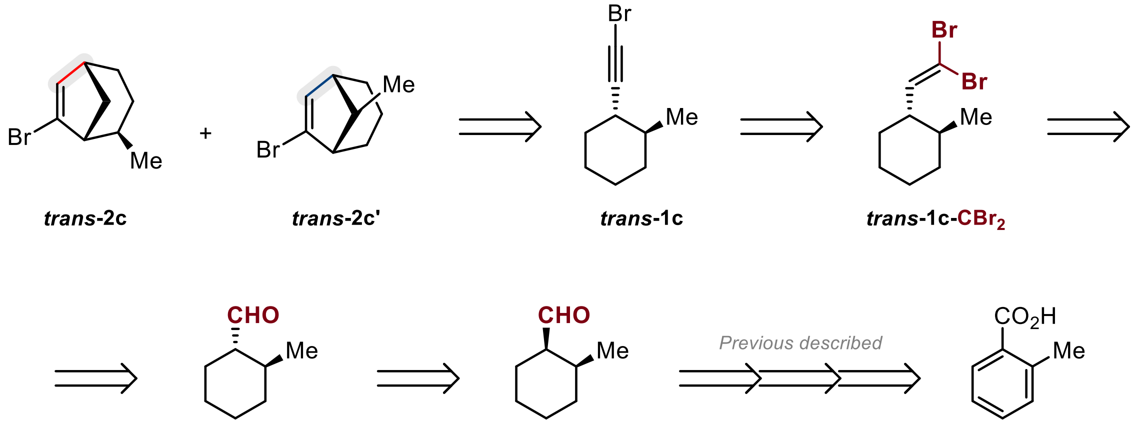

(1S,2S)-2-methylcyclohexane-1-carbaldehyde (trans-1c-CHO)

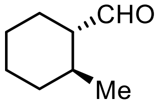

In a round bottom flask 5 mmol (631 mg, 1 equiv.) of aldehyde ***cis-1c-CHO*** was stirred with 0.3 mL (0.24 equiv.) of HCl (4M in dioxane) overnight at room temperature. Then, the reaction was quenched with water, extracted with Et_2O three times, and washed with brine. The crude was dried with Na_2SO_4, filtered and the solvents were removed under vacuum, yielding aldehyde ***trans-1c-CHO***as a colorless liquid (581 mg, 4.6 mmol, 92%, dr = 10:1). The crude aldehyde was used in the next step without further purification. The ^{1}H NMR spectrum matches the one previously reported.[12]
^{1}H NMR (300 MHz, $CDCl_3$) δ 9.56 (d, *J* = 3.8 Hz, 1H), 2.00 – 1.83 (m, 1H), 1.85 – 1.56 (m, 5H), 1.38 – 1.18 (m, 3H), 0.93 (d, *J* = 6.4 Hz, 3H).
HRMS not purified

(1S,2S)-1-(2,2-dibromovinyl)-2-methylcyclohexane (trans-1c-CBr_2)

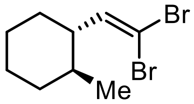

The title compound was synthesized using General Procedure Q, starting from 4.6 mmol (581 mg) of aldehyde ***trans-1c-CHO***, yielding dibromoolefin ***trans-1c-CBr_2*** as a colorless liquid (850 mg, 3 mmol, 66%, d.r. = 9:1). The crude was purified by flash column chromatography using n-hexanehexanes as eluent.

^{1}H NMR (300 MHz, $CDCl_3$) δ 6.16 (d, J = 9.5 Hz, 1H), 1.95 (dtd, J = 11.3, 9.9, 3.4 Hz, 1H), 1.82 – 1.63 (m, 5H), 1.36 – 1.16 (m, 4H), 1.12 – 0.94 (m, 2H), 0.88 (d, J = 6.5 Hz, 4H).
^{13}C NMR (75 MHz, $CDCl_3$) δ 143.7 (CH), 87.6 (C), 49.7 (CH), 37.0 (CH), 34.8 (CH_2), 31.4 (CH_2), 26.2 (CH_2), 25.8 (CH_2), 20.7 (CH_3).
HRMS unstable

***(1S,2S)-1-(bromoethynyl)-2-methylcyclohexane* (*trans-1c*)**

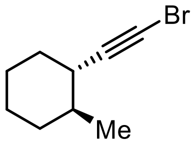

The title compound was synthesized using General Procedure R, starting from 2.9 mmol (807 mg) of dibromoolefin ***trans-1c-CBr₂***, yielding bromoalkyne ***trans-1c*** as a colorless liquid (350 mg, 1.7 mmol, 60%, d.r. = 9:1). The crude was purified by column chromatography using n-hexanehexanes as eluent.
^{1}H NMR (300 MHz, $CDCl_3$) δ 2.00 – 1.90 (m, 1H), 1.89 – 1.79 (m, 1H), 1.76 – 1.59 (m, 3H), 1.43 – 1.28 (m, 3H), 1.28 – 1.10 (m, 2H), 1.03 (d, J = 6.5 Hz, 3H).

^{13}C NMR (75 MHz, $CDCl_3$) δ 84.1 (C), 38.4 (CH), 38.4 (CH), 37.5 (C), 34.6 (CH_2), 33.0 (CH_2), 26.0 (CH_2), 25.8 (CH_2), 21.2 (CH_3).

HRMS (GC-Q-TOF) m/z: $[M]^+$ calcd for $C_9H_{13}Br$: 200.0201; found: 200.0200.

***Catalytic reaction of (1S,2S)-1-(bromoethynyl)-2-methylcyclohexane* (*trans-1c*)**

The catalytic reaction was performed using General Procedure S, starting from 0.2 mmol (40 mg) of bromoalkyne ***trans-1c***, affording a mixture of starting material and bromocyclopentenes ***trans-2c'*** and ***trans-2c*** in *27%* combine *yield by NMR analysis of the crude reaction mixture using CH_2Br_2 as Internal standard* as a mixture of regioisomers [1.8 (***trans-2c'***):1(***trans-2c***)].

1.3.4. (cis)-3-methyl Family Bridged

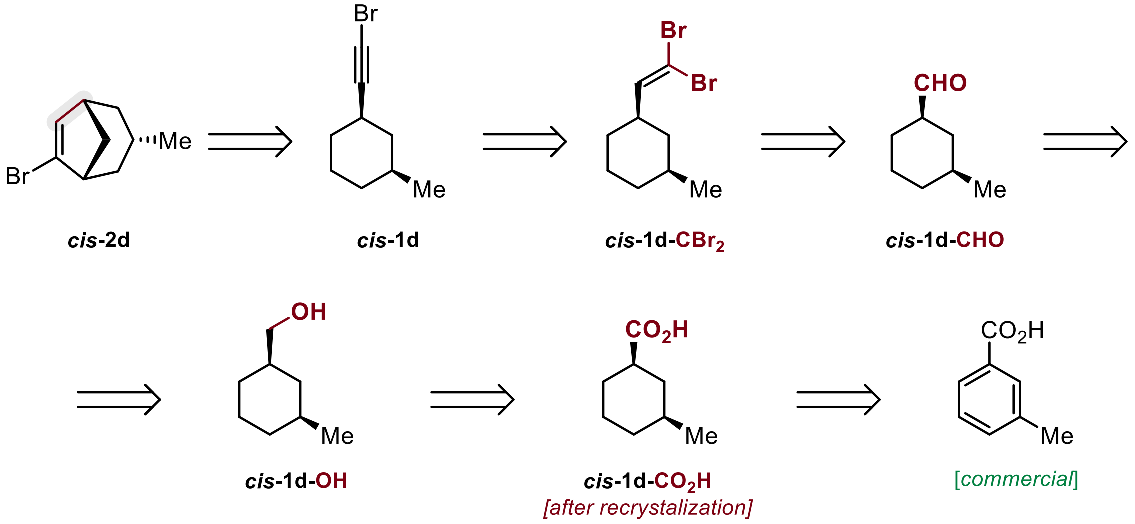

(1R,3S)-3-methylcyclohexane-1-carboxylic acid (cis-1d-CO₂H)

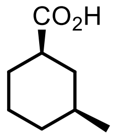

The title compound was synthesized using General Procedure A starting from 10 mmol (1.36 g) of *3-methylbenzoic acid*, yielding carboxylic acid ***cis-1d-CO₂H*** as a colorless liquid (1.42 g, 10 mmol, quantitative) as a 3:1 mixture of diastereoisomers. The crude was dissolved in acetone (0.25M) and *piperazine* (1 equiv., 10 mmol, 861 mg) was added under vigorous stirring. The mixture was allowed to crystallize slowly overnight. Then the liquid was decanted and the solid was dissolved in diethyether. The mixture was acidified with HCl 2M until pH 2. The resulting mixture was extracted with Et_2O (x2) and washed with Brine. The combined organic layers were dried over Na_2SO_4 and concentrated under vacuum to afford the acid (***cis-1d-CO₂H***) in an improved diastereomeric ratio (10:1 to 13:1).[13]

^{1}H NMR (300 MHz, $CDCl_3$) δ 11.67 (s, 1H), 2.32 (tq, J = 10.9, 3.5 Hz, 1H), 2.05 – 1.86 (m, 2H), 1.79 (dq, J = 9.7, 3.1 Hz, 1H), 1.67 (dtt, J = 11.8, 3.5, 1.7 Hz, 1H), 1.60 – 1.48 (m, 1H), 1.47 – 1.34 (m, 1H), 1.29 (td, J = 9.8, 2.5 Hz, 2H), 1.15 – 0.97 (m, 2H), 0.91 (dd, J = 6.6, 3.4 Hz, 4H).

^{13}C NMR (75 MHz, $CDCl_3$) δ 183.0 (C), 43.5 (CH), 37.3 (CH_2), 34.52 (CH_2), 32.2 (CH), 28.6 (CH_2), 25.6 (CH_2), 22.7 (CH_3).

***((1S,3R)-3-methylcyclohexyl)methanol* (*cis-1d-OH*)**

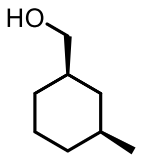

The title compound was synthesized using General Procedure H, starting from 3.6 mmol (512 mg) of carboxylic acid ***cis-1d-CO₂H*** as a 10:1 mixture of diastereoisomers, yielding alcohol ***cis-1d-OH*** as a colorless liquid (423 mg, 3.3 mmol, 92%, d.r. = 10:1). The crude alcohol was used in the next step without further purification.

^{1}H NMR (300 MHz, $CDCl_3$) δ 3.39 (d, J = 6.4 Hz, 2H), 2.10 (s, 1H), 1.81 – 1.59 (m, 4H), 1.56 – 1.29 (m, 3H), 1.29 – 1.13 (m, 1H), 0.86 (d, J = 6.6 Hz, 5H), 0.55 (q, J = 12.0 Hz, 1H).

^{13}C NMR (75 MHz, $CDCl_3$) δ 68.8 (CH_2), 40.7 (CH), 38.5 (CH_2), 35.4 (CH_2), 32.4 (CH), 29.3 (CH_2), 25.9 (CH_2), 22.9 (CH_3).

HRMS not purified

***(1S,3R)-3-methylcyclohexane-1-carbaldehyde* (*cis-1d-CHO*)**

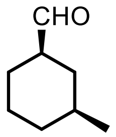

The title compound was synthesized using General Procedure P, starting from 3.3 mmol (422 mg) of alcohol ***cis-1d-OH***, yielding aldehyde ***cis-1d-CHO*** as a colorless liquid (417 mg, 3.3 mmol, 100%, d.r. = 10:1). The crude aldehyde was used in the next step without further purification.

^{1}H NMR (300 MHz, $CDCl_3$) δ 9.57 (d, J = 1.7 Hz, 1H), 2.22 (ttd, J = 12.2, 3.5, 1.5 Hz, 1H), 2.03 – 1.75 (m, 4H), 1.69 (dtt, J = 12.4, 3.4, 1.7 Hz, 1H), 1.50 – 1.34 (m, 2H), 1.27 (tt, J = 12.7, 3.4 Hz, 1H), 1.11 (qd, J = 12.7, 3.4 Hz, 1H), 0.92 (d, J = 6.5 Hz, 6H).

^{13}C NMR (75 MHz, $CDCl_3$) δ 204.7 (CH), 50.7 (CH), 34.7 (CH_2), 34.5 (CH_2), 31.9 (CH), 25.8 (CH_2), 25.3 (CH_2), 22.8 (CH_3).

HRMS not purified

***(1S,3R)-1-(2,2-dibromovinyl)-3-methylcyclohexane* (*cis-1d-CBr₂*)**

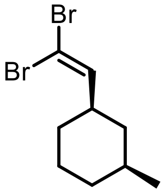

The title compound was synthesized using General Procedure Q, starting from 3.3 mmol (422 mg) of aldehyde ***cis-1d-CHO***, yielding dibromoolefin ***cis-1d-CBr$_2$*** as a colorless liquid (614 mg, 2.2 mmol, 66%, d.r. = 10:1). The crude was purified by flash column chromatography using n-hexanehexanes as eluent.

^{1}H NMR (300 MHz, $CDCl_3$) δ 6.18 (d, J = 9.1 Hz, 1H), 2.29 (tdt, J = 12.2, 9.0, 3.5 Hz, 1H), 1.81 – 1.60 (m, 4H), 1.41 (ttd, J = 11.6, 6.1, 5.6, 3.3 Hz, 1H), 1.35 – 1.21 (m, 1H), 1.08 – 0.92 (m, 1H), 0.89 (d, J = 6.6 Hz, 3H), 0.85 – 0.67 (m, 2H).

^{13}C NMR (75 MHz, $CDCl_3$) δ 143.9 (CH), 87.2 (C), 42.8 (CH), 39.9 (CH_2), 34.6 (CH_2), 32.2 (CH), 30.9 (CH_2), 25.7 (CH_2), 22.8 (CH_3).

HRMS (GC-Q-TOF) m/z: $[M]^+$ calcd for $C_9H_{14}Br_2$: 279.9462; found: 279.9459.

***(1S,3R)-1-(bromoethynyl)-3-methylcyclohexane* (*cis-1d*)**

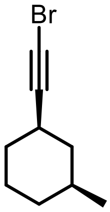

The title compound was synthesized using General Procedure R, starting from 2.2 mmol (614 mg) of dibromoolefin ***cis-1d-CBr$_2$***, yielding bromoalkyne ***cis-1d*** as a colorless liquid (321 mg, 1.6 mmol, 73%, d.r. = 10:1). The crude was purified by flash column chromatography using n-hexanes as eluent.

^{1}H NMR (300 MHz, $CDCl_3$) δ 2.25 (tt, J = 11.8, 3.3 Hz, 1H), 1.91 (ddp, J = 9.2, 3.5, 1.8 Hz, 2H), 1.80 – 1.56 (m, 2H), 1.42 – 1.12 (m, 3H), 0.99 (dt, J = 13.0, 11.8 Hz, 1H), 0.88 (d, J = 6.5 Hz, 4H).

^{13}C NMR (75 MHz, $CDCl_3$) δ 84.7 (C), 41.4 (CH_2), 37.6 (C), 34.4 (CH_2), 32.5 (CH_2), 32.4 (CH), 30.9 (CH), 25.8 (CH_2), 22.6 (CH_3).

HRMS (GC-Q-TOF) m/z: $[M]^+$ calcd for $C_9H_{13}Br$: 200.0201; found: 200.0199.

***Catalytic reaction of (1S,3R)-1-(bromoethynyl)-3-methylcyclohexane* (*cis-1d*)**

The catalytic reaction was performed using General Procedure S, starting from 0.2 mmol (40 mg) of bromoalkyne ***cis-1d***, affording a mixture of starting material and bromocyclopentene ***cis-2d*** in 3% *yield by ^{1}H NMR analysis of the crude reaction mixture using CH_2Br_2 as Internal standard.*

1.3.5. (trans)-3-methyl Family Bridged

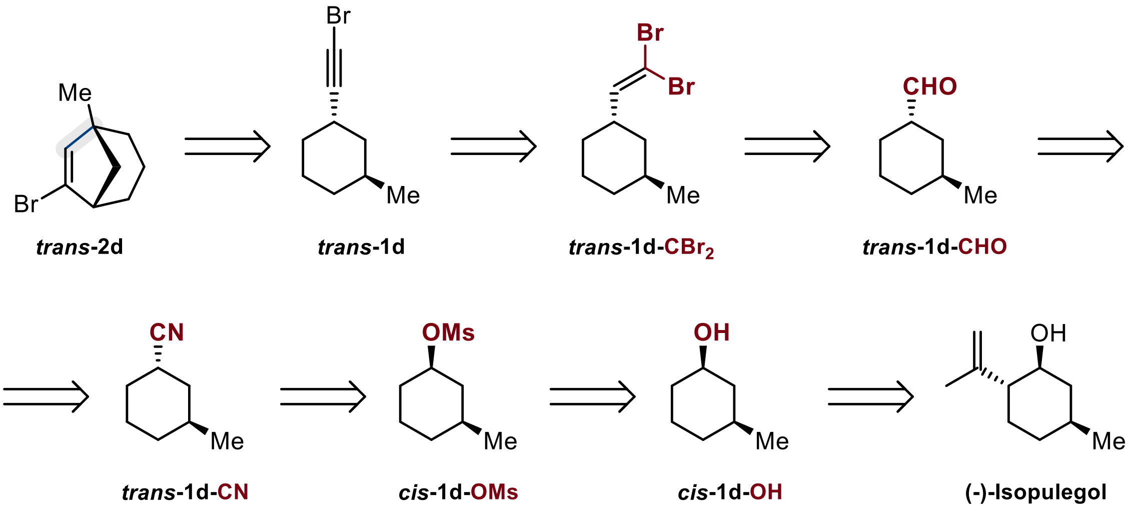

***(1S,3R)-3-methylcyclohexan-1-ol* (*cis-1d-OH*)**

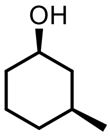

The title compound was synthesized using the procedure described in the literature,[14] starting from 50 mmol (7.71 g) of *(-)-Isopulegol* yielding alcohol ***cis-1d-OH*** as a colorless liquid (3.67 g, 32.2 mmol, 64%). The spectroscopic data match the previously reported.
^{1}H NMR (300 MHz, $CDCl_3$) δ 3.56 (tt, J = 10.8, 4.3 Hz, 1H), 2.00 – 1.88 (m, 2H), 1.74 (dp, J = 12.9, 3.3 Hz, 1H), 1.60 (dtq, J = 11.5, 3.5, 1.7 Hz, 1H), 1.52 – 1.32 (m, 2H), 1.12 (ddd, J = 12.3, 10.6, 3.5 Hz, 1H), 0.92 (d, J = 6.5 Hz, 4H), 0.89 – 0.69 (m, 2H).

***(1S,3R)-3-methylcyclohexyl methanesulfonate* (*cis-1d-OMs*)**

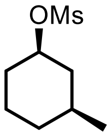

The title compound was synthesized using General Procedure L, starting from 32 mmol (3.67 g) of alcohol ***cis-1d-OH***, yielding mesylate ***cis-1d-OMs*** as a yellow liquid (5.38 g, 28 mmol, 87%). The crude mesylate was used in the next step without further purification.
^{1}H NMR (300 MHz, $CDCl_3$) δ 4.60 (tt, J = 11.1, 4.5 Hz, 1H), 3.00 (s, 3H), 2.12 (t, J = 7.2 Hz, 2H), 1.81 (dq, J = 13.5, 3.5, 3.1 Hz, 1H), 1.68 – 1.55 (m, 2H), 1.54 – 1.42 (m, 2H), 1.42 – 1.32 (m, 1H), 1.25 (s, 1H), 1.23 – 1.11 (m, 1H), 0.95 (d, J = 6.5 Hz, 3H), 0.83 (qd, J = 12.6, 3.8 Hz, 1H).
HRMS not purified

***(1R,3R)-3-methylcyclohexane-1-carbonitrile* (*trans-1d-CN*)**

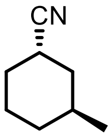

The title compound was synthesized using General Procedure M, starting from 28 mmol (5.38 g) of mesylate ***cis-1d-OMs***, yielding nitrile ***trans-1d-CN*** as a colorless liquid (1.08 g, 8.7 mmol, 31%). The crude nitrile was used in the next step without further purification.

^{1}H NMR (300 MHz, $CDCl_3$) δ 2.92 (p, J = 3.9 Hz, 1H), 1.93 – 1.82 (m, 2H), 1.81 – 1.54 (m, 4H), 1.42 (ddt, J = 13.3, 11.6, 4.5 Hz, 1H), 1.16 (ddd, J = 13.2, 11.3, 4.3 Hz, 1H), 0.89 (d, J = 6.4 Hz, 4H).
^{13}C NMR (75 MHz, $CDCl_3$) δ 122.4 (CH_2), 36.4 (CH_2), 34.0 (CH_2), 28.5 (CH), 28.3 (CH_2), 27.2 (CH), 22.2 (CH_2), 21.8 (CH_3).
HRMS (ESI-TOF) m/z: $[M+H]^+$ calcd for $C_8H_{14}N$: 124.1121; found: 124.1123.

***(1R,3R)-3-methylcyclohexane-1-carbaldehyde* (*trans-1d-CHO*)**

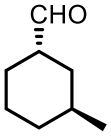

The title compound was synthesized using General Procedure N, starting from 8.7 mmol (1.08 g) of nitrile ***trans-1d-CN***, yielding aldehyde ***trans-1d-CHO*** as a colorless liquid (1.1 g, 8.7 mmol). The crude aldehyde was used in the next step without further purification.
^{1}H NMR (300 MHz, $CDCl_3$) δ 9.56 (t, J = 1.8 Hz, 1H), 2.21 (ddddd, J = 13.6, 8.3, 6.6, 4.2, 2.4 Hz, 1H), 2.05 – 1.50 (m, 5H), 1.50 – 1.02 (m, 4H), 0.96 – 0.60 (m, 3H).
^{13}C NMR (75 MHz, $CDCl_3$) δ 204.7 (C), 50.7 (CH), 34.7 (CH_2), 34.5 (CH_2), 31.9 (CH), 25.8 (CH_2), 25.3 (CH_2), 22.8 (CH_3).
HRMS not purified

***(1R,3R)-1-(2,2-dibromovinyl)-3-methylcyclohexane* (*trans-1d-CBr₂*)**

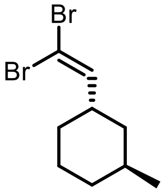

The title compound was synthesized using General Procedure Q, starting from 8.7 mmol (1.1 g) of aldehyde ***trans-1d-CHO***, yielding dibromoolefin ***trans-1d-CBr_2*** as a colorless liquid (588 mg, 2.1 mmol, 24% over two steps, dr > 20:1). The crude was purified by flash column chromatography using n-hexanehexanes as eluent.
^{1}H NMR (300 MHz, $CDCl_3$) δ 6.51 (d, J = 9.1 Hz, 1H), 2.75 – 2.54 (m, 1H), 1.79 – 1.35 (m, 7H), 1.27 (ddd, J = 13.1, 8.5, 4.2 Hz, 1H), 1.16 – 1.01 (m, 1H), 0.92 (d, J = 6.7 Hz, 3H).
^{13}C NMR (75 MHz, $CDCl_3$) δ 142.6 (CH), 87.4 (C), 37.9 (CH), 37.9 (CH_2), 33.6 (CH_2), 30.2 (CH_2), 27.8 (CH), 21.4 (CH_2), 21.2 (CH_3).
HRMS (GC-Q-TOF) m/z: $[M]^+$ calcd for $C_9H_{14}Br_2$: 279.9462; found: 279.9458.

***(1R,3R)-1-(bromoethynyl)-3-methylcyclohexane* (*trans-1d*)**

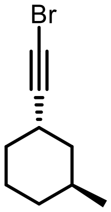

The title compound was synthesized using General Procedure R, starting from 2.1 mmol (588 mg) of dibromoolefin ***trans-1d-CBr_2***, yielding bromoalkyne ***trans-1d*** as a colorless liquid (357 mg, 1.8 mmol, 86%). The crude was purified by flash column chromatography using n-hexanehexanes as eluent.
^{1}H NMR (300 MHz, $CDCl_3$) δ 2.82 (t, J = 3.9 Hz, 1H), 1.87 – 1.62 (m, 5H), 1.62 – 1.49 (m, 2H), 1.47 – 1.31 (m, 1H), 1.26 (s, 1H), 1.18 – 1.06 (m, 1H), 0.87 (d, J = 6.4 Hz, 3H).

^{13}C NMR (75 MHz, $CDCl_3$) δ 84.2 (C), 39.2 (CH_2), 38.3 (C), 34.8 (CH_2), 30.8 (CH_2), 28.4 (CH), 28.1 (CH), 22.2 (CH_3), 22.0 (CH_2).
HRMS (GC-Q-TOF) m/z: $[M]^+$ calcd for $C_9H_{13}Br$: 184.9966; found: 184.9970.

***(1R,5S)-6-bromo-1-methylbicyclo[3.2.1]oct-6-ene* (*trans-2d*)**

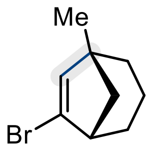

The title compounds were synthesized using General Procedure S, starting from 0.2 mmol (40 mg) of bromoalkyne ***trans-1d***, yielding bromocyclopentene ***trans-2d*** in *57% by NMR analysis of the crude reaction mixture using CH_2Br_2 as Internal standard*. The crude was purified by column chromatography using n-hexane as eluent to afford the title compound as a colorless liquid (24.0 mg, 0.12 mmol, 60%).

^{1}H NMR (400 MHz, $CDCl_3$) δ = 5.68 (d, J = 1.2 Hz, 1H), 2.61 (dt, J = 5.5, 2.7 Hz, 1H), 1.92 (ddt, J = 9.9, 5.0, 2.3 Hz, 1H), 1.65 – 1.45 (m, 4H), 1.35 – 1.12 (m, 4H), 1.05 (s, 3H), 0.95 – 0.79 (m, 1H).

^{13}C NMR (101 MHz, $CDCl_3$) δ = 137.2 (C), 122.2 (CH), 50.7 (CH), 48.6 (CH_2), 46.1 (C), 32.8 (CH_2), 25.2 (CH_2), 22.7 (CH_3), 19.5 (CH_2).

HRMS (GC-Q-TOF) m/z: $[M]^+$ calcd for $C_9H_{13}Br$: 184.9966; found: 184.9968.

1.3.6. *(cis)-4-methyl Family Bridged*

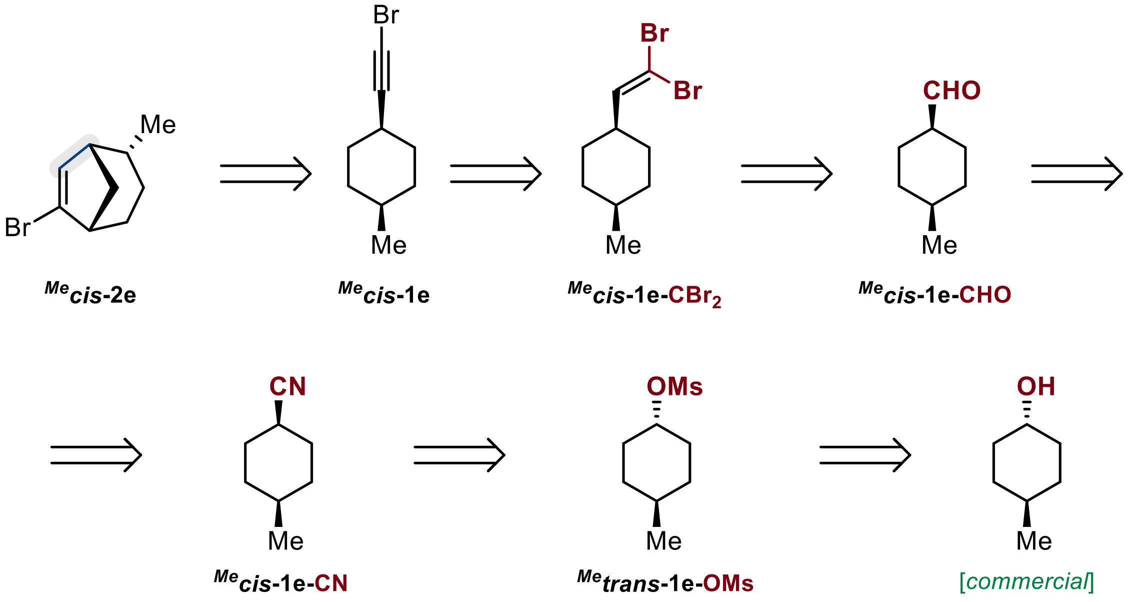

***(1r,4r)-4-methylcyclohexyl methanesulfonate* (*^{Me}trans-1e-OMs*)**

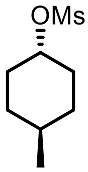

The title compound was synthesized using General Procedure L, starting from 20 mmol (2.28 g) of *(1r,4r)-4-methylcyclohexan-1-ol*, yielding mesylate ***^{Me}trans-1e-OMs*** as a

yellow liquid (3.8 g, 19.8 mmol, 99%). The crude mesylate was used in the next step without further purification.
^{1}H NMR (300 MHz, $CDCl_3$) δ 4.54 (td, J = 10.9, 5.5 Hz, 1H), 2.97 (d, J = 0.8 Hz, 3H), 2.16 – 2.03 (m, 2H), 1.76 (dt, J = 13.6, 3.3 Hz, 2H), 1.62 – 1.46 (m, 2H), 1.35 (dqq, J = 13.2, 6.4, 3.5 Hz, 1H), 1.11 – 0.93 (m, 2H), 0.87 (dd, J = 6.5, 0.8 Hz, 3H).
^{13}C NMR (75 MHz, $CDCl_3$) δ 82.1 (CH), 38.8 (CH_3), 32.9 (CH_2), 32.8 (CH_2), 31.3 (CH), 21.6 (CH_3).
HRMS not purified

(1s,4s)-4-methylcyclohexane-1-carbonitrile **(*^{Me}cis-1e-CN*)**

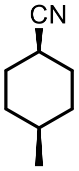

The title compound was synthesized using General Procedure M, starting from 19.8 mmol (3.8 g) of mesylate ***^{Me}trans-1e-OMs***, yielding nitrile ***^{Me}cis-1e-CN*** as a colorless liquid (671 mg, 5.5 mmol, 28%). The crude nitrile was used in the next step without further purification.
^{1}H NMR (300 MHz, $CDCl_3$) δ 2.91 – 2.82 (m, 1H), 1.99 – 1.88 (m, 2H), 1.71 – 1.47 (m, 4H), 1.42 – 1.18 (m, 3H), 0.91 (d, J = 6.0 Hz, 3H).
^{13}C NMR (75 MHz, $CDCl_3$) δ 122.4 (C), 31.8 (CH), 30.8 (CH_2), 28.3 (CH_2), 26.9 (CH), 22.1 (CH_3).
HRMS (ESI-TOF) m/z: $[M+H]^+$ calcd for $C_8H_{14}N$: 124.1121; found: 124.1121.

(1s,4s)-4-methylcyclohexane-1-carbaldehyde **(*^{Me}cis-1e-CHO*)**

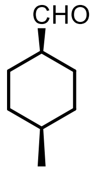

The title compound was synthesized using General Procedure N, starting from 5.5 mmol (671 mg) of nitrile ***^{Me}cis-1e-CN***, yielding aldehyde ***^{Me}cis-1e-CHO*** as a colorless liquid (562 g, 4.5 mmol, 82%). The crude aldehyde was used in the next step without further purification.
^{1}H NMR (300 MHz, $CDCl_3$) δ 9.66 (s, 1H), 2.39 – 2.28 (m, 1H), 2.11 – 1.98 (m, 2H), 1.64 – 1.37 (m, 8H), 1.00 (ddt, J = 14.3, 8.2, 3.4 Hz, 2H), 0.93 – 0.87 (m, 2H), 0.83 (d, J = 6.5 Hz, 3H).
^{13}C NMR (75 MHz, $CDCl_3$) δ 206.0 (CH), 47.3 (CH), 31.5 (CH_2), 31.3 (CH), 24.1 (CH_2), 21.6 (CH_3).
HRMS not purified

(1s,4s)-1-(2,2-dibromovinyl)-4-methylcyclohexane **(*^{Me}cis-1e-CBr_2*)**

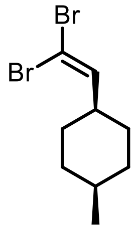

The title compound was synthesized using General Procedure Q, starting from 4.5 mmol (562 mg) of aldehyde ***^{Me}cis-1e-CHO***, yielding dibromoolefin ***^{Me}cis-1e-CBr_2*** as a colorless liquid (469 mg, 1.7 mmol, 37%). The crude was purified by flash column chromatography using n-hexanehexanes as eluent.

^{1}H NMR (300 MHz, $CDCl_3$) δ 6.52 (d, J = 9.0 Hz, 1H), 2.61 – 2.39 (m, 1H), 1.56 (d, J = 10.4 Hz, 6H), 1.20 (q, J = 9.8, 9.2 Hz, 2H), 0.92 (d, J = 5.9 Hz, 4H).
^{13}C NMR (75 MHz, $CDCl_3$) δ 142.3 (CH), 87.4 (C), 39.5 (CH), 30.9 (CH_2), 30.4 (CH), 28.2 (CH_2), 20.9 (CH_3).
HRMS (GC-Q-TOF) m/z: $[M]^+$ calcd for $C_9H_{14}Br_2$: 279.9462; found: 279.9451.

(1s,4s)-1-(bromoethynyl)-4-methylcyclohexane (^{Me}cis-1e)

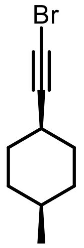

The title compound was synthesized using General Procedure R, starting from 1.7 mmol (469 mg) of dibromoolefin ***^{Me}cis-1e-CBr$_2$***, yielding bromoalkyne ***^{Me}cis-1e*** as a colorless liquid (163 mg, 0.8 mmol, 50%). The crude was purified by flash column chromatography using n-hexanehexanes as eluent.
^{1}H NMR (300 MHz, $CDCl_3$) δ 2.78 – 2.67 (m, 1H), 1.82 – 1.70 (m, 2H), 1.58 – 1.46 (m, 3H), 1.46 – 1.41 (m, 1H), 1.41 – 1.31 (m, 3H), 0.96 – 0.88 (m, 3H).
^{13}C NMR (75 MHz, $CDCl_3$) δ 83.9 (C), 38.3 (C), 32.1 (CH), 30.7 (CH_2), 30.5 (CH_2), 28.3 (CH), 22.3 (CH_3).
HRMS (GC-Q-TOF) m/z: $[M]^+$ calcd for $C_9H_{13}Br$: 200.0201; found: 200.0201.

(1R,2S,5R)-6-bromo-2-methylbicyclo[3.2.1]oct-6-ene (^{Me}cis-2e)

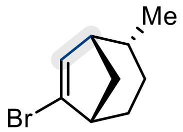

The title compound was synthesized using General Procedure S, starting from 0.2 mmol (40.2 mg) of bromoalkyne ***^{Me}cis-1e***, yielding bromocyclopentene ***^{Me}cis-2e*** in *56% yield by NMR analysis of the crude reaction mixture using CH_2Br_2 as Internal standard*). The crude was purified by column chromatography using n-hexanehexanes as eluent to afford the named product as a colorless liquid (27 mg, 0.14 mmol, 68%).
^{1}H NMR (400 MHz, $CDCl_3$) δ = 5.98 (dd, *J* = 3.0, 1.0 Hz, 1H), 2.51 (dt, *J* = 5.7, 2.8 Hz, 1H), 2.36 (dt, *J* = 5.2, 2.4 Hz, 1H), 2.19 (dtd, *J* = 10.4, 5.3, 2.2 Hz, 1H), 1.53 (dddd, *J* = 13.1, 6.9, 5.6, 3.5 Hz, 4H), 1.44 (dd, *J* = 10.0, 0.9 Hz, 1H), 1.32 (tdd, *J* = 13.0, 5.6, 2.4 Hz, 2H), 1.23 – 1.11 (m, 1H), 1.04 – 0.81 (m, 1H), 0.77 (d, *J* = 6.5 Hz, 3H).
^{13}C NMR (101 MHz, $CDCl_3$) δ = 131.0 (CH), 124.3 (C), 46.9 (CH), 46.8 (CH), 44.6 (CH_2), 31.6 (CH), 27.7 (CH_2), 23.2 (CH_2), 21.5 (CH_3).
HRMS (GC-Q-TOF) m/z: $[M]^+$ calcd for $C_9H_{13}Br$: 200.0201; found: 200.0199.

1.3.7. *(cis)-4-isopropyl/tertbutyl Family Bridged*

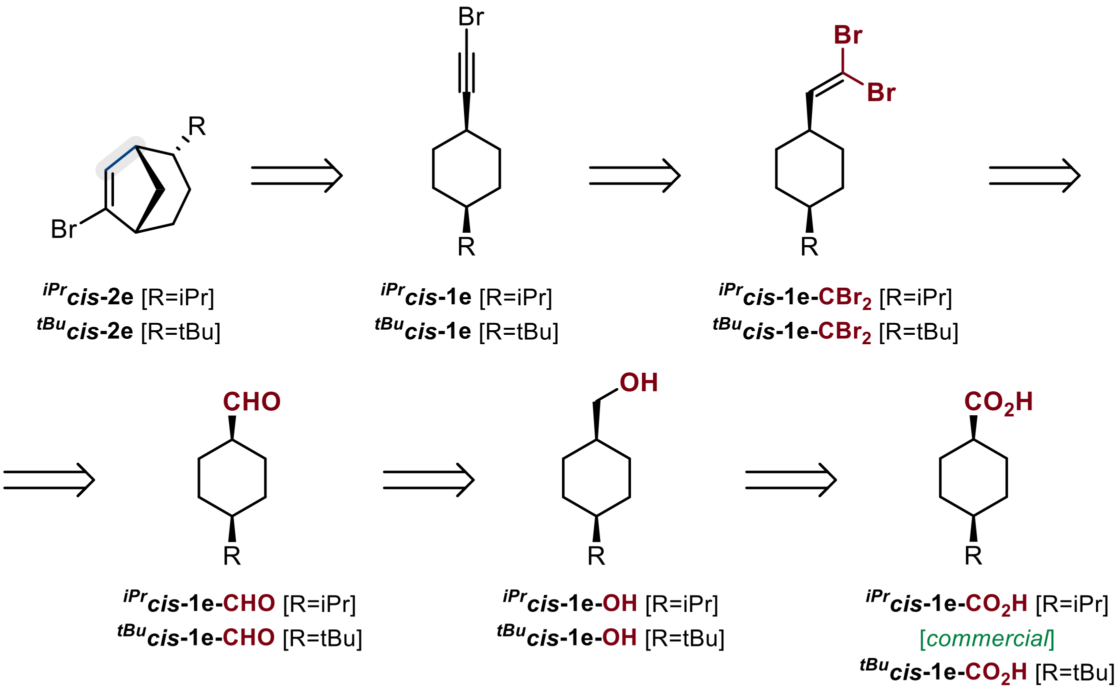

((1s,4s)-4-isopropylcyclohexyl)methanol (^{iPr}cis-1e-OH)

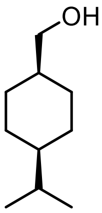

The title compound was synthesized using General Procedure H, starting from 5.9 mmol (1 g) of *(1s,4s)-4-methylcyclohexane-1-carboxylic acid*, yielding alcohol ***^{iPr}cis-1e-OH*** as a colorless liquid (932 mg, 5.9 mmol, quantitative). The crude alcohol was used in the next step without further purification. NMR spectra match those previously reported.[15]
^{1}H NMR (300 MHz, $CDCl_3$) δ 3.54 (d, J = 7.1 Hz, 2H), 1.71 (qd, J = 6.9, 4.7 Hz, 2H), 1.58 – 1.23 (m, 9H), 1.08 (dt, J = 7.8, 3.9 Hz, 1H), 0.84 (d, J = 6.7 Hz, 6H).
^{13}C NMR (75 MHz, $CDCl_3$) δ 65.6 (CH_2), 42.9 (CH), 37.5 (CH), 30.4 (CH), 26.2 (CH_2), 25.7 (CH_2), 20.4 (CH_3).
HRMS not purified

(1s,4s)-4-isopropylcyclohexane-1-carbaldehyde (^{iPr}cis-1e-CHO)

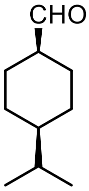

The title compound was synthesized using General Procedure P, starting from 2.2 mmol (375 mg) of alcohol ***^{iPr}cis-1e-OH***, yielding aldehyde ***^{iPr}cis-1e-CHO*** as a colorless liquid (318 mg, 2 mmol, 91%). The crude aldehyde was used in the next step without further purification.

^{1}H NMR (300 MHz, $CDCl_3$) δ 9.80 – 9.61 (m, 1H), 2.38 (ddt, J = 5.0, 3.3, 1.6 Hz, 1H), 2.23 – 2.07 (m, 2H), 1.56 (d, J = 6.8 Hz, 5H), 1.45 – 1.30 (m, 1H), 1.13 – 0.92 (m, 4H), 0.80 (dd, J = 6.8, 1.3 Hz, 6H).
^{13}C NMR (75 MHz, $CDCl_3$) δ 206.0 (CH), 47.2 (CH), 43.3 (CH), 32.1 (CH), 26.6 (CH_2), 24.8 (CH_2), 19.9 (CH_3).
HRMS not purified

(1s,4s)-1-(2,2-dibromovinyl)-4-isopropylcyclohexane (^{iPr}cis-1e-CBr$_2$)

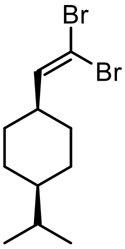

The title compound was synthesized using General Procedure Q, starting from 1.9 mmol (293 mg) of aldehyde ***^{iPr}cis-1e-CHO***, yielding dibromoolefin ***^{iPr}cis-1e-CBr$_2$*** as a colorless liquid (356 mg, 1.2 mmol, 60%, d.r = 15:1). The crude was purified by flash column chromatography using n-hexane as eluent.
^{1}H NMR (300 MHz, $CDCl_3$) δ 6.57 (d, J = 9.1 Hz, 1H), 2.58 (dt, J = 9.0, 4.5 Hz, 1H), 1.71 – 1.38 (m, 8H), 1.32 – 1.18 (m, 2H), 1.09 (ddd, J = 9.6, 6.5, 3.2 Hz, 1H), 0.87 (d, J = 6.7 Hz, 6H).
^{13}C NMR (75 MHz, $CDCl_3$) δ 141.9 (CH), 87.4 (C), 43.0 (CH), 39.0 (CH), 31.4 (CH), 29.1 (CH_2), 25.9 (CH_2), 20.3 (CH_3).
HRMS unstable

(1s,4s)-1-(bromoethynyl)-4-isopropylcyclohexane (^{iPr}cis-1e)

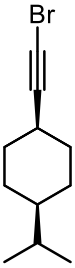

The title compound was synthesized using General Procedure R, starting from 1.2 mmol (363 mg) of dibromoolefin ***^{iPr}cis-1e-CBr$_2$***, yielding bromoalkyne ***^{iPr}cis-1e*** as a colorless liquid (243 mg, 1.1 mmol, 91%, d.r > 20:1). The crude was purified by flash column chromatography using n-hexane as eluent.
^{1}H NMR (300 MHz, $CDCl_3$) δ 2.76 (q, J = 3.5 Hz, 1H), 1.86 – 1.75 (m, 2H), 1.61 – 1.18 (m, 7H), 0.87 (d, J = 6.8 Hz, 7H).

^{13}C NMR (75 MHz, $CDCl_3$) δ 83.9 (C), 43.9 (CH), 38.4 (C), 32.6 (CH), 30.9 (CH_2), 28.5 (CH), 25.6 (CH_2), 20.0 (CH_3).

HRMS (GC-Q-TOF) m/z: $[M-Br]^+$ calcd for $C_{11}H_{17}$: 149.1330; found: 149.1329.

(1R,2R,5R)-6-bromo-2-isopropylbicyclo[3.2.1]oct-6-ene (^{iPr}cis-2e)

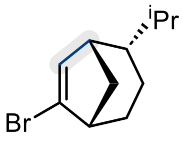

The title compound was synthesized using General Procedure S, starting from 0.1 mmol (22,9 mg) of bromoalkyne ***iPr*cis-1e**, yielding bromocyclopentene ***iPr*cis-2e** in *35% yield by ^{1}H NMR analysis of the crude reaction mixture using CH_2Br_2 as Internal standard.* The crude was purified by flash column chromatography using n-hexane as eluent to afford an inseparable mixture of the title compound and starting material.

^{1}H NMR (400 MHz, $CDCl_3$) δ = 5.92 (dd, J = 3.0, 1.0 Hz, 1H), 2.66 (dt, J = 5.3, 2.4 Hz, 1H), 2.55 – 2.45 (m, 1H), 2.21 (dtd, J = 10.7, 5.5, 2.4 Hz, 1H), 1.74 – 1.61 (m, 1H), 1.61 – 1.51 (m, 2H), 1.40 (s, 2H), 1.36 (s, 1H), 1.31 (dd, J = 5.5, 2.1 Hz, 1H), 1.29 – 1.21 (m, 3H), 1.21 – 1.11 (m, 2H), 1.04 (ddt, J = 12.1, 8.1, 2.9 Hz, 1H), 0.88 (d, J = 6.5 Hz, 6H), 0.82 (d, J = 6.5 Hz, 3H).

^{13}C NMR (101 MHz, $CDCl_3$) δ = 131.4 (CH), 123.6 (C), 47.1 (CH), 44.6 (CH_2), 43.6 (CH), 42.8 (CH), 32.7 (CH), 24.0 (CH_2), 23.3 (CH_2), 21.0 (CH_3), 20.8 (CH_3).

HRMS (GC-Q-TOF) m/z: $[M]^+$ calcd for $C_{11}H_{17}Br$: 228.0514; found: 228.0519

((1s,4s)-4-(tert-butyl)cyclohexyl)methanol (tBucis-1e-CO_2H)

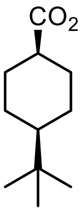

The title compound was purified as a pure diastereoisomer from the commercial mixture of diastereoisomers by flash chromatography using DCM as eluent. [16]

^{1}H NMR (300 MHz, $CDCl_3$) δ = 2.69 (dt, J = 5.6, 2.9 Hz, 1H), 2.30 – 2.18 (m, 2H), 1.66 (d, J = 12.7 Hz, 2H), 1.46 (tt, J = 13.4, 4.6 Hz, 2H), 1.15 (qd, J = 12.9, 3.2 Hz, 2H), 1.00 (dt, J = 11.8, 2.8 Hz, 1H), 0.83 (s, 9H).

^{13}C NMR (75 MHz, $CDCl_3$) δ = 182.0 (C), 48.1 (CH), 39.1 (CH), 32.7 (C), 27.9 (CH_2), 27.6 (CH_3), 24.0 (CH_2).

HRMS commercially available

((1s,4s)-4-(tert-butyl)cyclohexyl)methanol (tBucis-1e-OH)

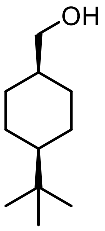

The title compound was synthesized using General Procedure H, starting from 2.7 mmol (489 mg) of carboxylic acid ***tBu*cis-1e-CO_2H**, yielding alcohol ***tBu*cis-1e-OH** as a colorless liquid (443 mg, 2.6 mmol, 97%). The crude alcohol was used in the next step without further purification.

^{1}H NMR (300 MHz, $CDCl_3$) δ 3.64 (d, *J* = 7.6 Hz, 2H), 1.90 – 1.72 (m, 3H), 1.53 (dd, *J* = 29.4, 16.9 Hz, 7H), 1.02 (d, *J* = 7.1 Hz, 3H), 0.83 (s, 9H).

HRMS not purified

(1s,4s)-4-(tert-butyl)cyclohexane-1-carbaldehyde (tBucis-1e-CHO)

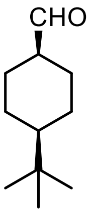

The title compound was synthesized using General Procedure P, starting from 2.6 mmol (469 mg) of alcohol ***^{tBu}cis-1e-OH***, yielding aldehyde ***^{tBu}cis-1e-CHO*** as a yellow liquid (432 mg, 2.56 mmol, 99%). The crude aldehyde was used in the next step without further purification.

^{1}H NMR (300 MHz, $CDCl_3$) δ 9.71 (d, *J* = 0.8 Hz, 1H), 2.41 (ddd, *J* = 8.3, 5.2, 3.6 Hz, 1H), 2.28 (dd, *J* = 14.5, 2.8 Hz, 2H), 1.67 (d, *J* = 11.4 Hz, 3H), 1.52 (d, *J* = 5.2 Hz, 3H), 1.04 – 0.86 (m, 4H), 0.80 (d, *J* = 0.8 Hz, 10H).

HRMS not purified

(1s,4s)-1-(tert-butyl)-4-(2,2-dibromovinyl)cyclohexane (^{tBu}cis-1e-CBr_2)

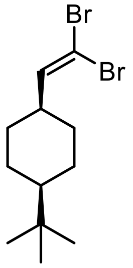

The title compound was synthesized using General Procedure Q, starting from 2.6 mmol (469 mg) of aldehyde ***^{tBu}cis-1e-CHO***, yielding dibromoolefin ***^{tBu}cis-1e-CBr_2*** as a colorless liquid (594 mg, 1.8 mmol, 72%). The crude was purified by flash column chromatography using n-hexane as eluent.

^{1}H NMR (300 MHz, $CDCl_3$) δ 6.67 (d, *J* = 9.1 Hz, 1H), 2.65 (dq, *J* = 7.1, 2.3 Hz, 1H), 1.85 – 1.38 (m, 7H), 1.20 – 0.93 (m, 3H), 0.85 (s, 10H).

^{13}C NMR (75 MHz, $CDCl_3$) δ 141.3 (CH), 87.5 (C), 48.2 (CH), 37.6 (CH), 32.7 (C), 30.4 (CH_2), 27.6 (CH_3), 22.9 (CH_2).

HRMS unstable

(1s,4s)-1-(bromoethynyl)-4-(tert-butyl)cyclohexane (^{tBu}cis-1e)

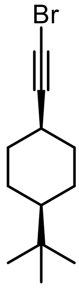

The title compound was synthesized using General Procedure R, starting from 1.7 mmol (590 mg) of dibromoolefin ***^{tBu}cis-1e-CBr_2***, yielding bromoalkyne ***^{tBu}cis-1e*** as a colorless liquid (373 mg, 1.5 mmol, 93%). The crude was purified by flash column chromatography using n-hexane as eluent.

^{1}H NMR (300 MHz, $CDCl_3$) δ 2.83 – 2.73 (m, 1H), 1.93 – 1.81 (m, 2H), 1.65 – 1.53 (m, 2H), 1.48 – 1.31 (m, 4H), 1.00 – 0.89 (m, 1H), 0.86 (s, 10H).

^{13}C NMR (75 MHz, $CDCl_3$) δ 83.8 (C), 48.2 (CH), 38.4 (C), 32.7 (C), 31.4 (CH_2), 28.2 (CH), 27.6 (CH_3), 23.1 (CH_2).

HRMS (GC-Q-TOF) m/z: $[M]^+$ calcd for $C_{12}H_{19}Br$: 227.0435; found: 227.0427.

Catalytic reaction of (1s,4s)-1-(bromoethynyl)-4-(tert-butyl)cyclohexane (^{tBu}cis-1e)

The catalytic reaction was performed using General Procedure S, starting from 0.2 mmol (48 mg) of bromoalkyne ***^{tBu}cis-1e***, affording a mixture of starting material and bromocyclopentene (***^{tBu}cis-2e***) as a single diastereoisomer in *13% yield by ^{1}H NMR analysis of the crude reaction mixture using CH_2Br_2 as Internal standard.*

1.3.8. *(trans)-4-methyl Family Bridged*

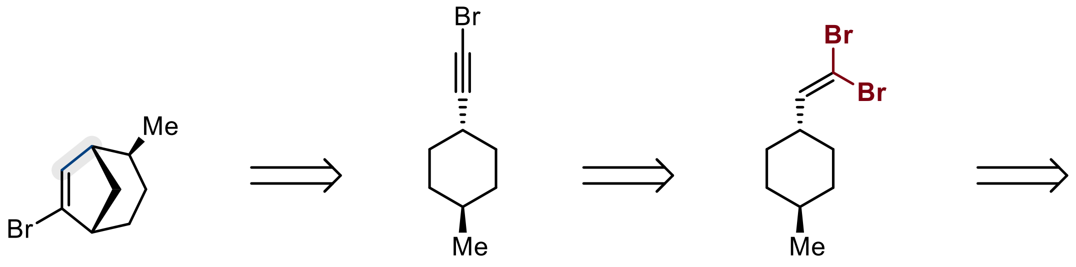

^{Me}***trans*-2e** | ^{Me}***trans*-1e** | ^{Me}***trans*-1e-CBr$_2$**

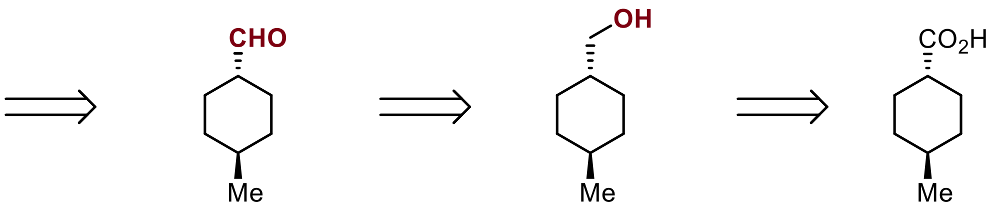

^{Me}***trans*-1e-CHO** | ^{Me}***trans*-1e-OH** | [*commercial*]

((1r,4r)-4-methylcyclohexyl)methanol (^{Me}*trans-1e-OH*)

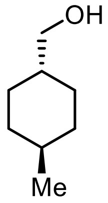

The title compound was synthesized using General Procedure H, starting from 15 mmol (2.1 g) of *trans-4-methylcyclohexanecarboxylic acid*, yielding alcohol ^{Me}***trans-1e-OH*** as a colorless liquid (1.8 g, 14 mmol, 94%). The crude alcohol was used in the next step without further purification. The ^{1}H NMR spectrum matches the one previously reported.[17]

^{1}H NMR (300 MHz, $CDCl_3$) δ 3.46 (d, *J* = 6.3 Hz, 2H), 1.84 – 1.65 (m, 3H), 1.51 – 1.27 (m, 2H), 1.01 – 0.81 (m, 5H).

HRMS not purified

(1r,4r)-4-methylcyclohexane-1-carbaldehyde (^{Me}*trans-1e-CHO*)

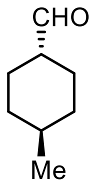

The title compound was synthesized using General Procedure P, starting from 5.8 mmol (746 mg) of alcohol ^{Me}***trans-1e-OH***, yielding aldehyde ^{Me}***trans-1e-CHO*** as a colorless liquid (740 mg, 5.8 mmol, quantitative). The crude aldehyde was used in the next step without further purification.

^{1}H NMR (300 MHz, $CDCl_3$) δ 9.61 (d, *J* = 1.7 Hz, 1H), 2.22 – 2.07 (m, 1H), 2.03 – 1.89 (m, 2H), 1.80 (dd, *J* = 13.3, 3.5 Hz, 2H), 1.43 – 1.12 (m, 4H), 0.99 (dd, *J* = 13.0, 3.2 Hz, 2H), 0.91 (t, *J* = 7.2 Hz, 4H).

HRMS not purified

(1r,4r)-1-(2,2-dibromovinyl)-4-methylcyclohexane (^{Me}***trans-1e-CBr*** $_2$ ***)***

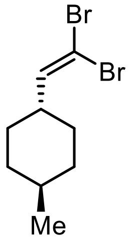

The title compound was synthesized using General Procedure Q, starting from 5.8 mmol (740 mg) of aldehyde ^{Me}***trans-1e-CHO*** yielding dibromoolefin ^{Me}***trans-1e-CBr*** $_2$ as a colorless liquid (1.2 g, 4.4 mmol, 75%). The crude was purified by flash column chromatography using n-hexane as eluent.

^{1}H NMR (300 MHz, $CDCl_3$) δ 6.19 (d, J = 9.0 Hz, 1H), 2.29 – 2.08 (m, 1H), 1.72 (td, J = 14.4, 3.6 Hz, 4H), 1.30 (dd, J = 6.7, 3.3 Hz, 1H), 1.21 – 1.05 (m, 2H), 1.03 (s, 2H), 0.88 (d, J = 6.5 Hz, 4H).

^{13}C NMR (75 MHz, $CDCl_3$) δ 144.0 (CH), 87.2 (C), 42.5 (CH), 34.5 (CH_2), 32.1 (CH), 31.4 (CH_2), 22.8 (CH_3).

HRMS unstable

(1r,4r)-1-(bromoethynyl)-4-methylcyclohexane (^{Me}***trans-1e)***

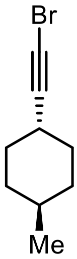

The title compound was synthesized using General Procedure R, starting from 4.2 mmol (1.2 g) of dibromoolefin ^{Me}***trans-1e-CBr*** $_2$, yielding bromoalkyne ^{Me}***trans-1e*** as a colorless liquid (757 mg, 3.8 mmol, 90%). The crude was purified by flash column chromatography using n-hexane as eluent.

^{1}H NMR (300 MHz, $CDCl_3$) δ 2.17 (tt, J = 11.9, 3.6 Hz, 1H), 2.00 – 1.86 (m, 2H), 1.68 (dd, J = 13.6, 3.7 Hz, 2H), 1.45 – 1.22 (m, 3H), 0.97 – 0.77 (m, 5H).

^{13}C NMR (75 MHz, $CDCl_3$) δ 84.7 (C), 37.7 (C), 34.5 (CH_2), 32.8 (CH_2), 31.9 (CH), 30.6 (CH), 22.6 (CH_3).

HRMS (GC-Q-TOF) m/z: $[M]^+$ calcd for $C_9H_{13}Br$: 200.0201; found: 200.0194.

Catalytic reaction of (1r,4r)-1-(bromoethynyl)-4-methylcyclohexane (^{Me}***trans-1e)***

The catalytic reaction was performed using General Procedure S, starting from 0.2 mmol (40 mg) of bromoalkyne ^{Me}***trans-1e***, affording a mixture of starting material and bromocyclopentene ^{Me}***trans-2e*** in *14% yield by* ^{1}*H NMR analysis of the crude reaction mixture using* CH_2Br_2 *as Internal standard.*

1.3.9. *(trans)-4-isopropyl Family Bridged*

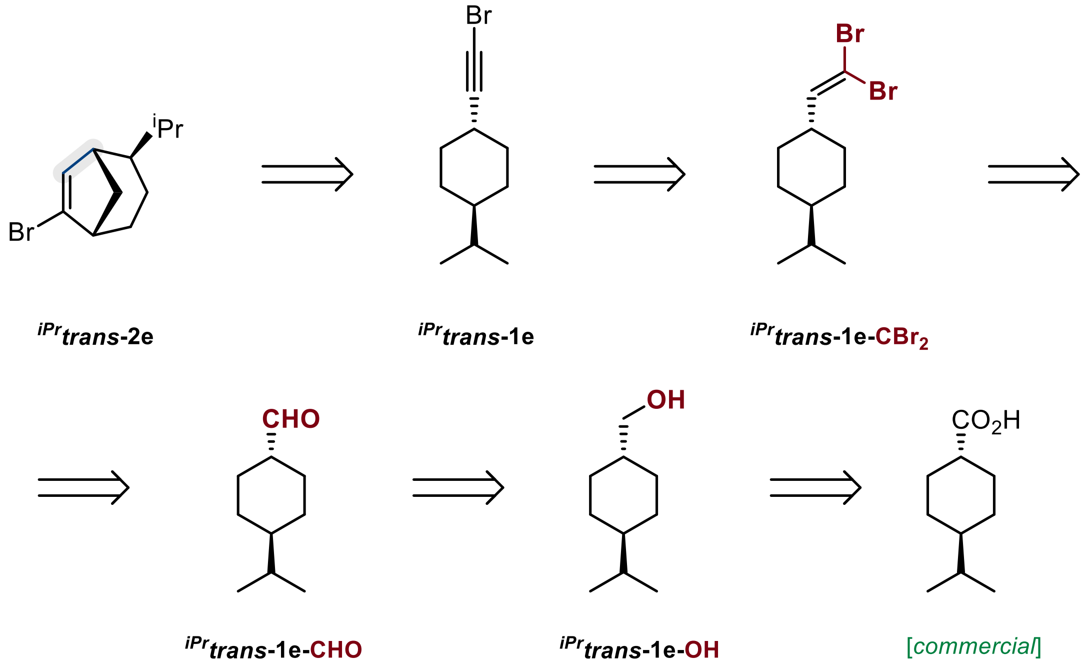

((1r,4r)-4-isopropylcyclohexyl)methanol (iPrtrans-1e-OH)

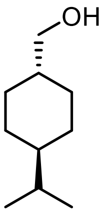

The title compound was synthesized using General Procedure H, starting from 7.7 mmol (1.3 g) of *(1r,4r)-4-isopropylcyclohexane-1-carboxylic acid*, yielding alcohol iPr***trans-1e-OH*** as a colorless liquid (1.2 g, 7.7 mmol, quantitative). The crude alcohol was used in the next step without further purification. NMR spectra match those previously reported.[15]

^{1}H NMR (300 MHz, $CDCl_3$) δ 3.42 (d, *J* = 6.4 Hz, 2H), 1.87 – 1.69 (m, 4H), 1.58 (s, 1H), 1.49 – 1.29 (m, 2H), 1.07 – 0.89 (m, 4H), 0.84 (dd, *J* = 6.8, 0.9 Hz, 7H).

^{13}C NMR (75 MHz, $CDCl_3$) δ 68.9 (CH_2), 44.3 (CH), 40.8 (CH), 33.0 (CH), 29.8 (CH_2), 29.2 (CH_2), 20.0 (CH_3).

HRMS not purified

(1r,4r)-4-isopropylcyclohexane-1-carbaldehyde (iPrtrans-1e-CHO)

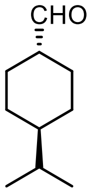

The title compound was synthesized using General Procedure P, starting from 7.7 mmol (1.2 g) of alcohol iPr***trans-1e-OH***, yielding aldehyde iPr***trans-1e-CHO*** as a yellow liquid

(954 mg, 6.2 mmol, 80%). The crude aldehyde was used in the next step without further purification.
^{1}H NMR (300 MHz, $CDCl_3$) δ 9.60 (d, J = 1.7 Hz, 1H), 2.23 – 2.07 (m, 1H), 2.07 – 1.91 (m, 2H), 1.82 (dt, J = 10.1, 3.6 Hz, 2H), 1.43 (td, J = 6.8, 4.1 Hz, 1H), 1.34 – 1.13 (m, 3H), 1.01 (s, 4H), 0.86 (d, J = 6.8 Hz, 7H).
HRMS not purified

(1r,4r)-1-(2,2-dibromovinyl)-4-isopropylcyclohexane (^{iPr}trans-1e-CBr$_2$)

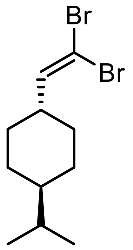

The title compound was synthesized using General Procedure Q, starting from 6.2 mmol (854 mg) of aldehyde ^{iPr}***trans-1e-CHO***, yielding dibromoolefin ^{iPr}***trans-1e-CBr$_2$*** as a colorless liquid (1.5 g, 5 mmol, 80%). The crude was purified by flash column chromatography using n-hexane as eluent.
^{1}H NMR (300 MHz, $CDCl_3$) δ 6.19 (d, J = 9.0 Hz, 1H), 2.19 (ddd, J = 9.0, 3.7, 1.9 Hz, 1H), 1.88 – 1.76 (m, 2H), 1.76 – 1.65 (m, 2H), 1.41 (dd, J = 6.9, 3.9 Hz, 1H), 1.19 – 0.94 (m, 6H), 0.86 (d, J = 6.8 Hz, 7H).
^{13}C NMR (75 MHz, $CDCl_3$) δ 144.0 (CH), 87.2 (C), 43.4 (CH), 43.0 (CH), 33.0 (CH), 31.6 (CH_2), 29.1 (CH_2), 19.9 (CH_3).
HRMS unstable

(1r,4r)-1-(bromoethynyl)-4-isopropylcyclohexane (^{iPr}trans-1e)

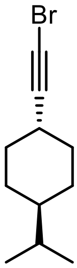

The title compound was synthesized using General Procedure R, starting from 3 mmol (930 mg) of dibromoolefin ^{iPr}***trans-1e-CBr$_2$***, yielding bromoalkyne ^{iPr}***trans-1e*** as a colorless liquid (606 mg, 2.6 mmol, 88%). The crude was purified by flash column chromatography using n-hexane as eluent.
^{1}H NMR (300 MHz, $CDCl_3$) δ 2.16 (tt, J = 11.9, 3.7 Hz, 1H), 2.05 – 1.91 (m, 2H), 1.71 (dd, J = 13.2, 3.0 Hz, 2H), 1.47 – 1.24 (m, 3H), 1.09 – 0.88 (m, 3H), 0.84 (d, J = 6.8 Hz, 7H).
^{13}C NMR (75 MHz, $CDCl_3$) δ 84.8 (C), 43.3 (CH), 37.7 (C), 33.1 (CH_2), 32.9 (CH), 31.1 (CH), 29.2 (CH_2), 19.9 (CH_3).
HRMS (GC-Q-TOF) m/z: $[M-Br]^+$ calcd for $C_{11}H_{17}$: 149.1330; found: 149.1328.

Catalytic reaction of (1r,4r)-1-(bromoethynyl)-4-isopropylcyclohexane (^{iPr}***trans-1e***)

The catalytic reaction was performed using General Procedure S, starting from 0.2 mmol (46 mg) of bromoalkyne ^{iPr}***trans-1e***, affording a mixture of starting material and bromocyclopentene ^{iPr}***trans-2e*** in *3% yield by ^{1}H NMR analysis of the crude reaction mixture using CH_2Br_2 as Internal standard.*

1.3.10. 1-methyl Family Fused

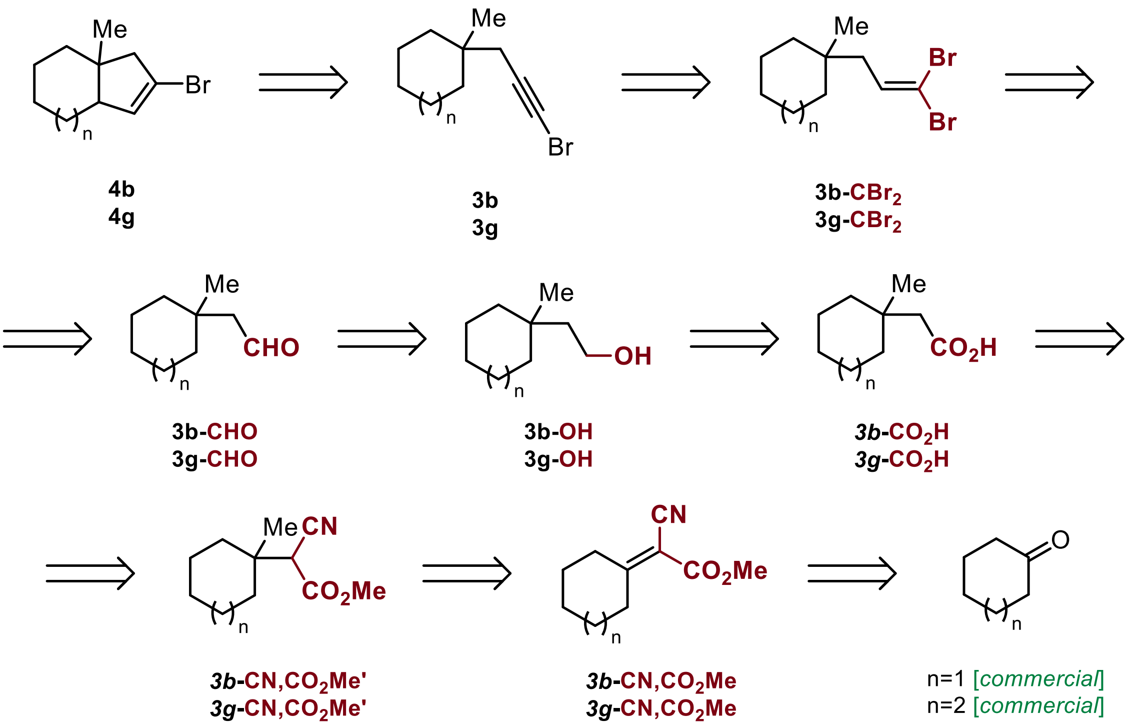

methyl 2-cyano-2-cyclohexylideneacetate (***3b-CN, CO₂Me***)

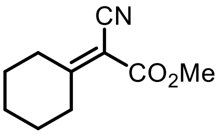

The title compound was synthesized using General Procedure C, starting from 120 mmol (11.78 g) of *cyclohexanone* yielding the cyanoester (***3b-CN, CO₂Me***) as a colorless liquid (17.96 g, 92.9 mmol, 77%). The crude was purified by column chromatography using hexane/EtOAc (10:1 to 5:1) as eluent. NMR spectra match those previously reported.[18]

^{1}H NMR (300 MHz, $CDCl_3$) δ 4.27 (q, J = 7.1 Hz, 2H), 3.05 – 2.91 (m, 2H), 2.73 – 2.59 (m, 2H), 1.86 – 1.60 (m, 6H), 1.34 (t, J = 7.1 Hz, 3H).

methyl 2-cyano-2-(1-methylcyclohexyl)acetate (***3b-CN, CO₂Me'***)

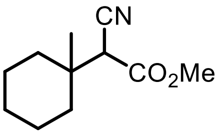

The title compound was synthesized using General Procedure D, starting from 5 mmol (896 mg) of cyanoacrylate ***3b-CN, CO₂Me***, yielding saturated cyanoester ***3b-CN, CO₂Me'*** as an orange liquid (436 mg, 2.2 mmol, 45%). The crude ester was used in the next step without further purification. The ^{1}H NMR spectrum matches the one previously reported.[18]

^{1}H NMR (300 MHz, $CDCl_3$) δ 3.79 (s, 3H), 3.48 (s, 1H), 1.52 (t, *J* = 7.2 Hz, 11H), 1.14 (s, 3H).

***2-(1-methylcyclohexyl)acetic acid* (3*b*-*CO₂H*)**

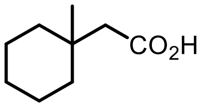

The title compound was synthesized using General Procedure E, starting from 2.2 mmol (436 mg) of cyanoester ***3b-CN, CO₂Me'***, yielding acid ***3b-CO₂H*** as a slightly red liquid (296 mg, 1.9 mmol, 85%). The crude acid was used in the next step without further purification. The ^{1}H NMR spectrum matches the one previously reported.[3]

^{1}H NMR (300 MHz, $CDCl_3$) δ 2.27 (s, 2H), 1.62 – 1.28 (m, 11H), 1.05 (s, 3H).

HRMS not purified

***2-(1-methylcyclohexyl)ethan-1-ol* (3*b*-*OH*)**

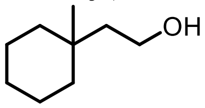

The title compound was synthesized using General Procedure H, starting from 1.9 mmol (436 mg) of acid ***3b-CO₂H***, yielding alcohol **3*b*-*OH*** as a colorless liquid (205 mg, 1.4 mmol, 76%). The crude alcohol was used in the next step without further purification. The ^{1}H NMR spectrum matches the one previously reported.[18]

^{1}H NMR (300 MHz, $CDCl_3$) δ 3.74 – 3.63 (m, 2H), 1.55 (dd, *J* = 8.4, 7.1 Hz, 2H), 1.50 – 1.16 (m, 11H), 0.90 (s, 3H).

HRMS not purified

***2-(1-methylcyclohexyl)acetaldehyde* (3*b*-*CHO*)**

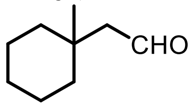

The title compound was synthesized using General Procedure P, starting from 1.4 mmol (205 mg) of alcohol **3*b*-*OH***, yielding aldehyde ***3b-CHO*** as a colorless liquid (157 mg, 1.1 mmol, 80%). The crude aldehyde was used in the next step without further purification. The ^{1}H NMR spectrum matches the one previously reported.[18]

^{1}H NMR (300 MHz, $CDCl_3$) δ 9.86 (t, *J* = 3.3 Hz, 1H), 2.29 (d, *J* = 3.3 Hz, 2H), 1.60 – 1.24 (m, 11H), 1.08 (s, 3H).

HRMS not purified

***1-(3,3-dibromoallyl)-1-methylcyclohexane* (3b-*CBr₂*)**

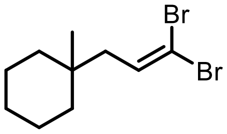

The title compound was synthesized using General Procedure Q, starting from 1.1 mmol (157 mg) of aldehyde ***3b-CHO***, yielding dibromoolefin ***3b-CBr₂*** as a colorless liquid (229 mg, 0.8 mmol, 69%). The crude was purified by flash column chromatography using n-hexane as eluent.

^{1}H NMR (300 MHz, $CDCl_3$) δ 6.43 (t, *J* = 7.6 Hz, 1H), 2.04 (d, *J* = 7.5 Hz, 2H), 1.45 (d, *J* = 5.6 Hz, 5H), 1.35 – 1.17 (m, 5H), 0.91 (s, 4H).

^{13}C NMR (75 MHz, $CDCl_3$) δ 136.3 (CH), 89.1 (C), 46.9 (C), 37.7 (2xCH_2), 34.2 (C), 26.4 (CH_2), 25.2 (CH_3), 22.1 (2xCH_2).

HRMS unstable

***1-(3-bromoprop-2-yn-1-yl)-1-methylcyclohexane* (3b)**

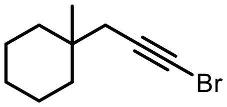

The title compound was synthesized using General Procedure R, starting from 0.8 mmol (229 mg) of dibromoolefin **3b-*CBr₂*** , yielding bromoalkyne **3b** as a colorless liquid (143 mg, 0.7 mmol, 83%). The crude was purified by flash column chromatography using n-hexane as eluent.

^{1}H NMR (300 MHz, $CDCl_3$) δ 2.12 (s, 2H), 1.43 (td, *J* = 6.3, 2.6 Hz, 6H), 1.32 (q, *J* = 5.7 Hz, 5H), 0.97 (s, 3H).

^{13}C NMR (75 MHz, $CDCl_3$) δ 78.7 (C), 38.7 (C), 37.1 (CH_2), 33.7 (C), 32.9 (CH_2), 26.3 (CH_2), 25.3 (CH_3), 22.2 (CH_2).

HRMS (GC-Q-TOF) m/z: $[M]^+$ calcd for $C_{10}H_{15}Br$: 214.0357; found: 214.0355.

(3aS,7aR)-2-bromo-7a-methyl-3a,4,5,6,7,7a-hexahydro-1H-indene (4b)

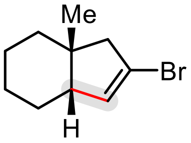

The title compound was synthesized using General Procedure S, starting from 0.2 mmol (43.0 mg) of bromoalkyne **3b**, yielding bromocyclopentene (**4b**) in *78% yield by NMR analysis of the crude reaction mixture using CH_2Br_2 as Internal standard*. The crude was purified by column chromatography using n-hexane as eluent to afford the title compound as a colorless liquid (37.9 mg, 0.18 mmol, 88%, dr > 20:1). In the same way, the reaction was carried out on a 2 mmol scale of starting material (**3b**) (1 equiv., 430 mg), using the same catalyst loading (2.5 mol%, 45 mg) and solvent dilution (0.1M, 20 mL).

^{1}H NMR (300 MHz, $CDCl_3$) δ = 5.75 (d, *J* = 1.7 Hz, 1H), 2.32 (ddd, *J* = 7.2, 3.5, 2.2 Hz, 3H), 1.58 (s, 3H), 1.51 – 1.21 (m, 10H), 1.11 (s, 3H).

^{13}C NMR (75 MHz, $CDCl_3$) δ = 136.2 (CH), 120.0 (C), 53.6 (CH_2), 50.9 (CH), 42.2 (C), 35.0 (CH_2), 26.9 (CH_2), 26.4 (CH_3), 22.5 (CH_2), 22.0 (CH_2).

HRMS (GC-Q-TOF) m/z: $[M]^+$ calcd for $C_{10}H_{15}Br$: 214.0357; found: 214.0354.

(3aS,7aR)-7a-methyl-3a,4,5,6,7,7a-hexahydro-1H-indene-2-carboxylic acid (4b-CO_2H)

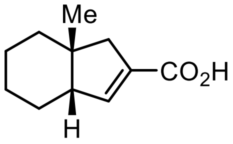

The procedure was adapted from the literature, with a slight modification.[6] Bromocyclopentene **4b** (165 mg, 0.77 mmol, 1 equiv.) was dissolved in THF (0.1M) in a flamed Schlenk flask under argon. The solution was cooled to -78 ºC and *tert*-BuLi 1.7 M (2 equiv., 0.9 mL) was added dropwise. The reaction mixture was stirred for 1 hour. After completion of the time CO_2 was bubbled into the solution. The reaction was allowed to warm to room temperature and the reaction was quenched carefully with water and then acidify with HCl 2N until pH ≈ 2. The crude was purified through flash column chromatography using AcOEt/Hexanes (10:1 to 3:1) as eluent, affording carboxylic acid ***4b-CO_2H*** as a white solid (122 mg, 0.67 mmol, 88%).

^{1}H NMR (300 MHz, $CDCl_3$) δ = 11.46 (s, 1H), 6.85 (d, *J* = 1.9 Hz, 1H), 2.44 (ddd, *J* = 12.1, 6.1, 2.2 Hz, 1H), 2.35 (d, *J* = 1.6 Hz, 1H), 2.31 – 2.21 (m, 1H), 1.73 – 1.23 (m, 9H), 1.10 (s, 3H). **^{13}C NMR** (75 MHz, $CDCl_3$) δ = 171.6 (C), 152.2 (CH), 134.3 (C), 51.7 (CH_3), 44.6 (CH_2), 41.8 (C), 34.7 (CH_2), 26.5 (CH_2), 26.3 (CH_3), 23.3 (CH_2), 22.0 (CH_2).

HRMS (GC-Q-TOF) m/z: $[M]^+$ calcd for $C_{11}H_{16}O_2$: 180.1150; found: 180.1155.

Mp = 145 ºC

(piperazin-1-ium 7a-methyl-3a,4,5,6,7,7a-hexahydro-1H-indene-2-carboxylate (6b)

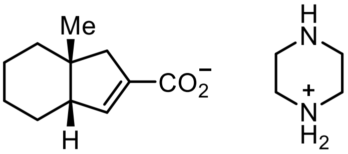

The title compound was synthesized by dissolving carboxylic acid ***4b-CO₂H*** (1 equiv.)in acetone (0.25M) and adding piperazine (1 equiv., 1 mmol, 86.1 mg) under vigorous stirring. The solid was filtered and re-dissolved in dichloromethane, then subjected to liquid–liquid difusion crystallization from a 1:1 DCM/diethyl ether mixture, affording the desired compound (***6b***) after 1 week.[13]
Mp = 184 ºC

1.3.11. *(cis)-2-methyl Family Fused*

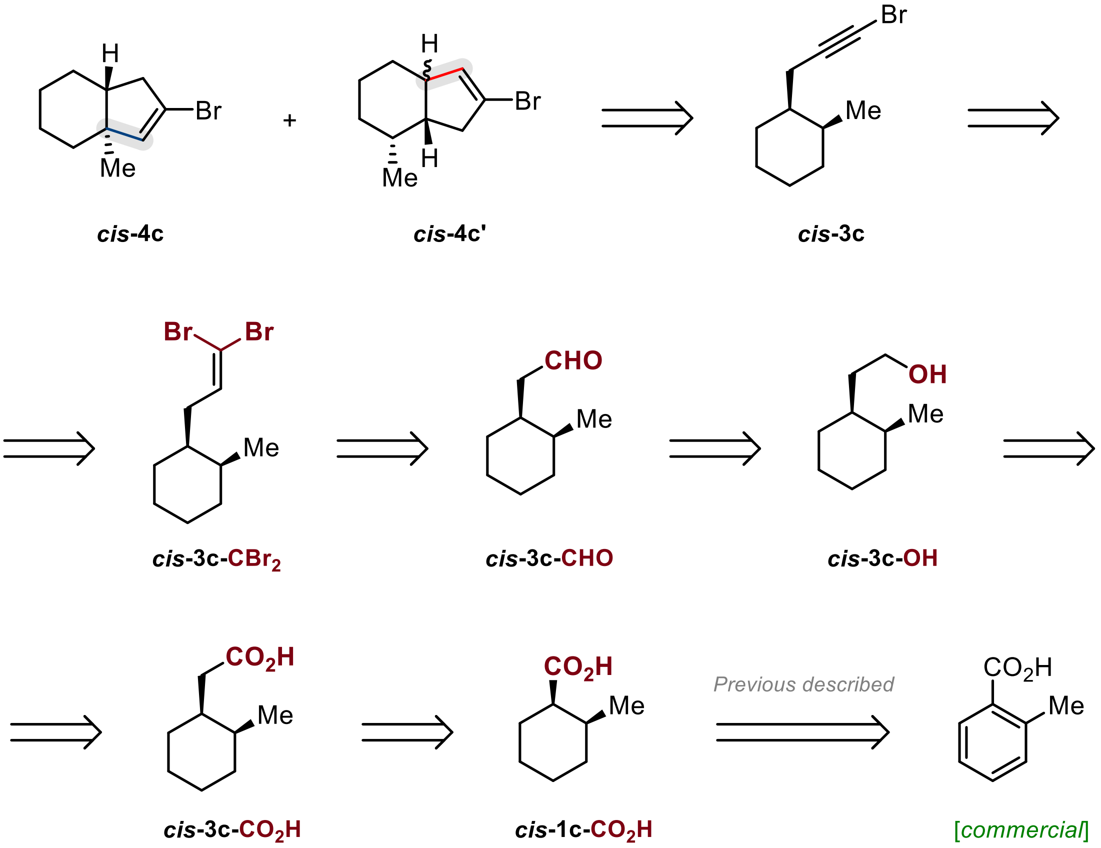

***2-((1S,2S)-2-methylcyclohexyl)acetic acid* (*cis-3c-CO₂H*)**

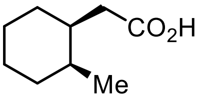

The title compound was synthesized using General Procedure B, starting from 5.5 mmol (768 mg) of acid ***cis-1c-CO₂H***, yielding acid ***cis-3c-CO₂H*** as a yellow liquid (756 mg, 4.8 mmol, 88%). The crude acid was used in the next step without further purification.
^{1}H NMR (300 MHz, $CDCl_3$) δ 11.05 (s, 1H), 2.28 – 2.21 (m, 2H), 2.06 (ddd, J = 8.2, 6.1, 3.6 Hz, 1H), 1.89 – 1.78 (m, 1H), 1.61 – 1.26 (m, 8H), 0.86 (d, J = 7.1 Hz, 3H).

^{13}C NMR (75 MHz, $CDCl_3$) δ 180.5 (C), 37.0 (CH), 36.8 (CH_2), 32.6 (CH), 32.1 (CH_2), 28.1 (CH_2), 24.4 (CH_2), 22.3 (CH_2), 14.9 (CH_3).
HRMS (ESI-TOF) m/z: $[M+H]^+$ calcd for $C_9H_{17}O_2$: 157.1223; found: 157.1222.

***2-((1S,2S)-2-methylcyclohexyl)ethan-1-ol* (*cis-3c-OH*)**

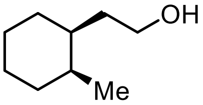

The title compound was synthesized using General Procedure H, starting from 4.8 mmol (756 mg) of acid ***cis-3c-CO₂H***, yielding alcohol ***cis-3c-OH*** as a colorless liquid (575 mg, 4 mmol, 83%). The crude alcohol was used in the next step without further purification.
^{1}H NMR (300 MHz, $CDCl_3$) δ 3.62 (ddd, *J* = 7.0, 6.1, 3.9 Hz, 2H), 1.89 (d, *J* = 20.3 Hz, 1H), 1.74 (dd, *J* = 7.4, 3.9 Hz, 1H), 1.64 – 1.50 (m, 2H), 1.50 – 1.19 (m, 9H), 0.82 (dd, *J* = 7.1, 1.0 Hz, 3H). **^{13}C NMR** (75 MHz, $CDCl_3$) δ 61.4 (CH_2), 36.4 (CH), 34.9 (CH_2), 32.6 (CH), 32.5 (CH_2), 27.8 (CH_2), 25.0 (CH_2), 22.3 (CH_2), 14.3 (CH_3). **HRMS** not purified

***2-((1S,2S)-2-methylcyclohexyl)acetaldehyde* (*cis-3c-CHO*)**

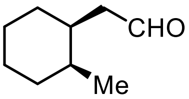

The title compound was synthesized using General Procedure P, starting from 4 mmol (575 mg) of alcohol ***cis-3c-OH***, yielding aldehyde ***cis-3c-CHO*** as a colorless liquid (570 mg, 4 mmol, quantitative). The crude aldehyde was used in the next step without further purification.
^{1}H NMR (300 MHz, $CDCl_3$) δ 9.72 (q, *J* = 2.4 Hz, 1H), 2.32 – 2.23 (m, 2H), 2.21 – 2.11 (m, 1H), 1.84 – 1.71 (m, 1H), 1.60 – 1.25 (m, 9H), 0.82 (d, *J* = 7.1 Hz, 3H).
^{13}C NMR (75 MHz, $CDCl_3$) δ 203.3 (CH), 45.9 (CH_2), 34.6 (CH), 32.9 (CH), 31.9 (CH_2), 28.5 (CH_2), 24.1 (CH_2), 22.5 (CH_2), 17.4 (CH_2), 15.3 (CH_3).
HRMS not purified

***(1S,2S)-1-(3,3-dibromoallyl)-2-methylcyclohexane* (*cis-3c-CBr₂*)**

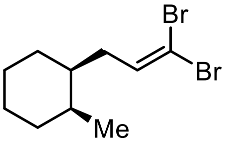

The title compound was synthesized using General Procedure Q, starting from 4 mmol (570 mg) of aldehyde ***cis-3c-CHO***, yielding dibromoolefin ***cis-3c-CBr₂*** as a colorless liquid (836 mg, 2.8 mmol, 71%, dr = 12:1). The crude was purified by flash column chromatography using n-hexane as eluent.
^{1}H NMR (300 MHz, $CDCl_3$) δ 6.38 (t, *J* = 7.2 Hz, 1H), 2.01 (t, *J* = 7.0 Hz, 2H), 1.78 (t, *J* = 3.7 Hz, 1H), 1.70 – 1.50 (m, 3H), 1.50 – 1.18 (m, 7H), 0.87 (d, *J* = 7.1 Hz, 3H).
^{13}C NMR (75 MHz, $CDCl_3$) δ 138.6 (CH), 88.5 (C), 39.6 (CH_2), 35.5 (CH), 32.6 (CH), 32.4 (CH_2), 27.7 (CH_2), 24.9 (CH_2), 22.2 (CH_2), 14.6 (CH_3).
HRMS (GC-Q-TOF) m/z: $[M]^+$ calcd for $C_{10}H_{16}Br_2$: 293.9619; found: 293.9617.

***(1S,2S)-1-(3-bromoprop-2-yn-1-yl)-2-methylcyclohexane* (*cis-3c*)**

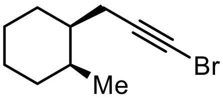

The title compound was synthesized using General Procedure R, starting from 2.8 mmol (829 mg) of dibromoolefin ***cis-3c-CBr₂***, yielding bromoalkyne ***cis-3c*** as a colorless liquid (492 mg, 2.3 mmol, 82%, dr = 12:1). The crude was purified by flash column chromatography using n-hexane as eluent.

^{1}H NMR (300 MHz, $CDCl_3$) δ 2.09 (d, *J* = 7.7 Hz, 2H), 1.92 (dh, *J* = 11.2, 3.2 Hz, 1H), 1.73 (ddd, *J* = 7.8, 5.3, 3.9 Hz, 1H), 1.63 – 1.52 (m, 1H), 1.52 – 1.18 (m, 8H), 0.83 (d, *J* = 7.1 Hz, 3H).
^{13}C NMR (75 MHz, $CDCl_3$) δ 79.9 (C), 39.6 (CH), 37.7 (C), 32.3 (CH), 32.0 (CH_2), 27.5 (CH_2), 24.8 (CH_2), 22.7 (CH_2), 22.0 (CH_2), 14.0 (CH_3).
HRMS (GC-Q-TOF) m/z: $[M-CH_3]^+$ calcd for $C_9H_{12}Br$: 199.0122; found: 199.0122.

(3aR,7aS)-2-bromo-3a-methyl-3a,4,5,6,7,7a-hexahydro-1H-indene (cis-4c), (3aR,7S,7aS)-2-bromo-7-methyl-3a,4,5,6,7,7a-hexahydro-1H-indene (cis-4c'-cis) and (3aS,7S,7aS)-2-bromo-7-methyl-3a,4,5,6,7,7a-hexahydro-1H-indene (cis-4c'-trans)

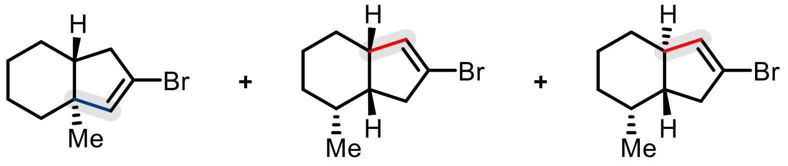

major	*minorM*	*minorm*
**cis-4c**	**cis-4c'-cis**	**cis-4c'-trans**

The title compounds were synthesized using General Procedure S, starting from 0.2 mmol (43.0 mg) of bromoalkyne ***cis-3c***, yielding bromocyclopentenes ***cis-4c***, ***cis-4c'-cis*** *and* ***cis-4c'-trans*** *in 66% combined yield by ^{1}H NMR analysis of the crude reaction mixture using CH_2Br_2 as Internal standard as a mixture of regio- and diastereoisomers [6.1(**cis-4c**):4.4(**cis-4c'-cis**):1(**cis-4c'-trans**)].* The crude was purified by column chromatography using n-hexane as eluent to afford a mixture of isomers as a colorless liquid [28.4 mg, 0.13 mmol, 66%).
^{1}H NMR (400 MHz, $CDCl_3$) δ = 6.00 (dt, J = 2.5, 1.6 Hz, 1H, ***minorM***), 5.98 – 5.93 (m, 1H, ***major***), 5.84 (s, ***minorm***), 2.47 (dq, J = 10.6, 2.9 Hz, 1H), 2.42 (s, 1H), 2.42 – 2.31 (m, 3H), 2.28 (dd, J = 6.9, 0.9 Hz, 1H), 2.25 (dd, J = 6.8, 1.0 Hz, 1H), 1.87 (tdd, J = 12.0, 6.8, 2.9 Hz, 1H), 1.80 – 1.66 (m, 3H), 1.70 – 1.58 (m, 3H), 1.53 (d, J = 9.6 Hz, 6H), 1.40 (ddd, J = 15.6, 8.2, 3.4 Hz, 3H), 1.37 – 1.20 (m, 2H), 1.13 (dt, J = 12.8, 3.0 Hz, 1H), 1.00 (dd, J = 12.6, 3.1 Hz, 1H), 0.94 (d, J = 7.0 Hz, 2H, ***minorm***), 0.90 (d, J = 6.9 Hz, 3H, ***minorM***), 0.86 (s, 4H, ***major***).

^{13}C NMR (101 MHz, $CDCl_3$, ***major***) δ = 143.4 (CH), 121.6 (C), 51.3 (CH), 47.1 (C) 42.3 (CH_2), 35.8 (CH_2), 26.8 (CH_2), 23.8 (CH_2), 21.4 (CH_2), 16.1 (CH_3).

^{13}C NMR (101 MHz, $CDCl_3$, ***minorM***) δ = 137.8 (CH), 121.1 (C), 45.1 (CH), 44.5 (CH), 37.6 (CH_2), 32.6 (CH), 29.8 (CH_2), 29.2 (CH_2), 24.4 (CH_2), 20.5 (CH_3).

^{13}C NMR (101 MHz, $CDCl_3$, ***minorm***) δ = 135.8 (CH), 53.3 (CH), 43.9 (CH), 42.0 (CH_2), 33.2 (CH_2), 31.1 (CH_2), 29.5 (CH), 21.3 (CH_2), 13.6 (CH_3).

HRMS (GC-Q-TOF) m/z: $[M]^+$ calcd for $C_{10}H_{15}Br$: 214.0357; found: 214.0357.

1.3.12. (trans)-2-methyl Family Fused

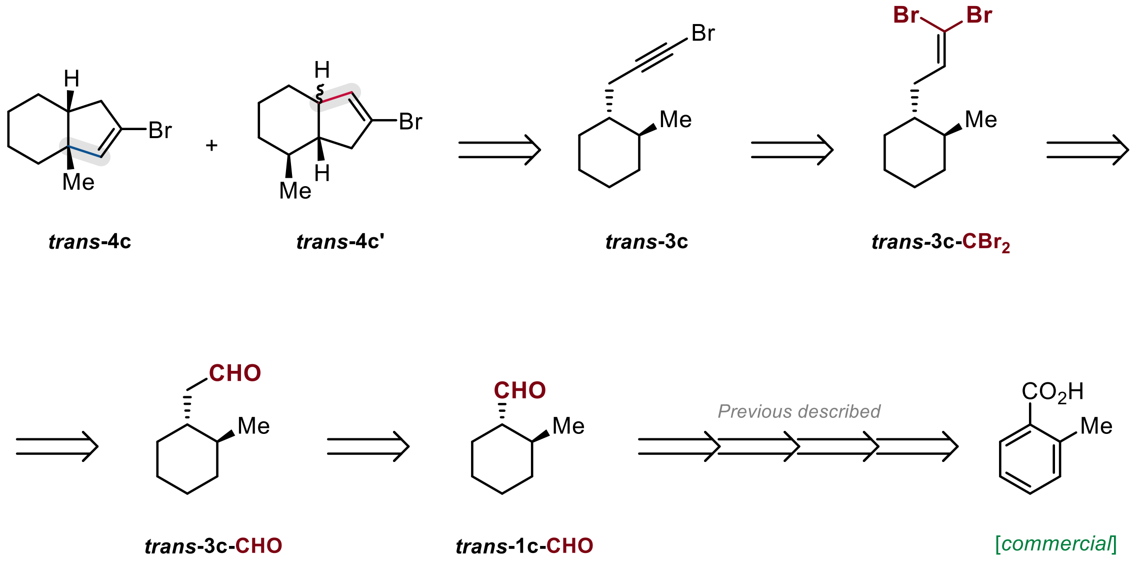

***2-((1R,2S)-2-methylcyclohexyl)acetaldehyde* (*trans-3c-CHO*)**

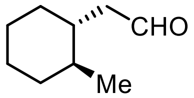

The procedure was adapted from the literature, with a slight modification.[19] In a flamed Schlenk containing *(methoxymethyl)triphenylphosphonium chloride* (4 equiv., 7.4g) in THF (0.4M) ^{t}BuOK (4 equiv., 2.4g) was added portion wise at 0 ºC. This mixture was stirred at room temperature for 15 minutes. Then, it was taken to 0 ºC again, and a solution of aldehyde ***trans-1c-CHO*** (1 equiv., 683mg, 5.4 mmol) in THF (0.3M) was added. The reaction was stirred at room temperature for 30 minutes. Then, it was quenched with brine and extracted with Et_2O three times. The crude was filtered by a pad of celite using HxH:Et_2O (10:1) as eluent. The intermediate was dissolved in $CHCl_3$ (0.05M) and trated with p-TsOH (10 mol%, 75 mg) overnight. Then, the solvent was removed, the residue diluted with water and extracted with Et_2O three times. The crude was purified by column chromatography using Hex:Et_2O (40:1 to 20:1) as the eluent to yield aldehyde ***trans-3c-CHO*** as a colorless liquid (191 mg, 1.4 mmol, 25%).

^{1}H NMR (300 MHz, $CDCl_3$) δ 9.75 (dd, J = 3.2, 1.8 Hz, 1H), 2.56 (ddd, J = 16.1, 4.3, 1.8 Hz, 1H), 2.14 (ddd, J = 16.1, 8.3, 3.1 Hz, 1H), 1.80 – 1.60 (m, 4H), 1.51 (ddt, J = 7.8, 2.7, 1.4 Hz, 1H), 1.29 – 0.94 (m, 6H), 0.88 (d, J = 6.3 Hz, 3H).

^{13}C NMR (75 MHz, $CDCl_3$) δ 203.5 (CH), 49.0 (CH_2), 39.5 (CH), 37.3 (CH), 35.7 (CH_2), 33.3 (CH_2), 26.4 (2xCH_2), 20.5 (CH_3).

***(1R,2S)-1-(3,3-dibromoallyl)-2-methylcyclohexane* (*trans-3c-CBr₂*)**

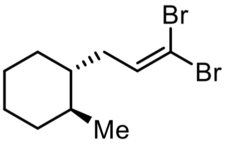

The title compound was synthesized using General Procedure Q, starting from 1.4 mmol (191 mg) of aldehyde ***trans-3c-CHO***, yielding dibromoolefin ***trans-3c-CBr_2*** as a colorless liquid (304 mg, 1.1 mmol, 75%, dr =12:1). The crude was purified by flash column chromatography using n-hexane as eluent.

^{1}H NMR (300 MHz, $CDCl_3$) δ 6.40 (dd, J = 8.0, 6.8 Hz, 1H), 2.30 (ddd, J = 14.8, 6.8, 2.9 Hz, 1H), 2.04 – 1.88 (m, 1H), 1.77 – 1.58 (m, 4H), 1.35 – 0.95 (m, 6H), 0.92 (d, J =

5.9 Hz, 3H).[13]**C NMR** (75 MHz, $CDCl_3$) δ 137.9 (CH), 88.7 (C), 43.7 (CH_2), 37.5 (CH), 36.9 (CH), 35.8 (CH_2), 32.2 (CH_2), 26.6 (CH_2), 26.5 (CH_2), 20.4 (CH_3).
HRMS (GC-Q-TOF) m/z: $[M]^+$ calcd for $C_{10}H_{16}Br_2$: 296.9619; found: 296.9622.

***(1R,2S)-1-(3-bromoprop-2-yn-1-yl)-2-methylcyclohexane* (*trans-3c*)**

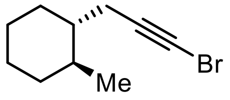

The title compound was synthesized using General Procedure R, starting from 1.1 mmol (300 mg) of dibromoolefin ***trans-3c-CBr₂***, yielding bromoalkyne ***trans-3c*** as a colorless liquid (168 mg, 0.8 mmol, 73%, dr = 12:1). The crude was purified by flash column chromatography using n-hexane as eluent.
^{1}**H NMR** (300 MHz, $CDCl_3$) δ 2.34 (dd, *J* = 16.7, 3.1 Hz, 1H), 2.16 (dd, *J* = 16.8, 6.8 Hz, 1H), 1.86 – 1.76 (m, 1H), 1.76 – 1.58 (m, 3H), 1.30 – 1.14 (m, 4H), 1.14 – 1.03 (m, 2H), 1.02 – 0.91 (m, 1H), 0.89 (d, *J* = 6.3 Hz, 3H).
^{13}**C NMR** (75 MHz, $CDCl_3$) δ 79.2 (C), 43.2 (CH), 38.1 (C), 36.2 (CH), 35.6 (CH_2), 32.2 (CH_2), 26.5 (2xCH_2), 24.5 (CH_2), 20.2 (CH_3).
HRMS (GC-Q-TOF) m/z: $[M-CH_3]^+$ calcd for $C_9H_{12}Br$: 199.0122; found: 199.0122.

(3aR,7aS)-2-bromo-3a-methyl-3a,4,5,6,7,7a-hexahydro-1H-indene (trans-4c), (3aR,7S,7aS)-2-bromo-7-methyl-3a,4,5,6,7,7a-hexahydro-1H-indene (trans-4c'-cis) and (3aS,7S,7aS)-2-bromo-7-methyl-3a,4,5,6,7,7a-hexahydro-1H-indene (trans-4c'-trans)

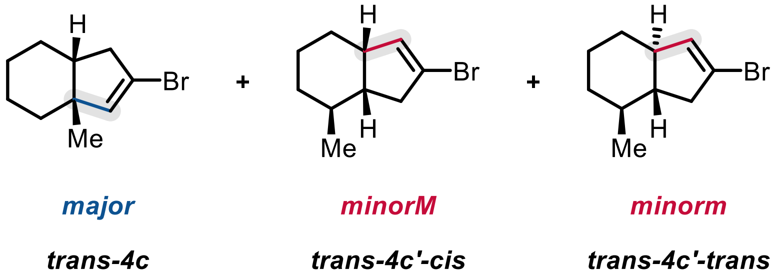

The title compounds were synthesized using General Procedure S, starting from 0.2 mmol (43.0 mg) of bromoalkyne ***trans-3c***, yielding bromocyclopentenes ***trans-4c***, ***trans-4c'-cis*** and ***trans-4c'-trans*** in *50% combined yield by ^{1}H NMR analysis of the crude reaction mixture using CH_2Br_2 as Internal standard as a mixture of regio- and diastereoisomers [8.2(**trans-4c**):3.2(**trans-4c'-cis**):1(**trans-4c'-trans**)]*. The crude was purified by column chromatography using n-hexane as eluent to afford an inseparable mixture of isomers as a colorless liquid (19.2 mg, 0.09 mmol, 45%).
^{1}**H NMR** (400 MHz, $CDCl_3$) δ = 5.91 (d, J = 2.5 Hz, ***minorM***), 5.72 (s, ***minorm***), 5.71 (t, J = 1.7 Hz, 1H, ***major***), 2.60 (ddd, J = 15.6, 7.5, 1.6 Hz, 1H), 2.47 (dd, J = 14.8, 6.8 Hz, 1H), 2.38 (ddd, J = 15.5, 7.2, 1.9 Hz, 1H), 2.34 – 2.24 (m, 1H), 2.22 – 2.03 (m, 1H), 2.01 – 1.86 (m, 2H), 1.85 – 1.63 (m, 3H), 1.63 – 1.52 (m, 2H), 1.51 – 1.29 (m, 10H), 1.27 – 1.20 (m, 1H), 1.15 – 1.09 (m, 1H), 1.06 (s, 3H, ***major***), 0.88 (dd, J = 7.9, 6.4 Hz, 4H).
^{13}**C NMR** (101 MHz, $CDCl_3$) δ = 142.1 (CH, ***major***), 136.2, 119.7 (C, ***major***), 56.8, 51.6, 46.8, 45.0 (CH, ***major***), 44.4 (CH_2, ***major***), 43.4, 43.2, 36.2, 35.9, 35.8, 35.6, 35.2 (CH_2, ***major***), 32.2, 30.0, 26.7, 26.5, 26.5 (CH_2, ***major***), 25.5 (CH, ***major***), 22.5 (CH_2, ***major***), 21.5 (CH_2, ***major***), 20.5, 20.2.
HRMS mixture of isomers

1.3.13. *(cis)-3-methyl Family Fused*

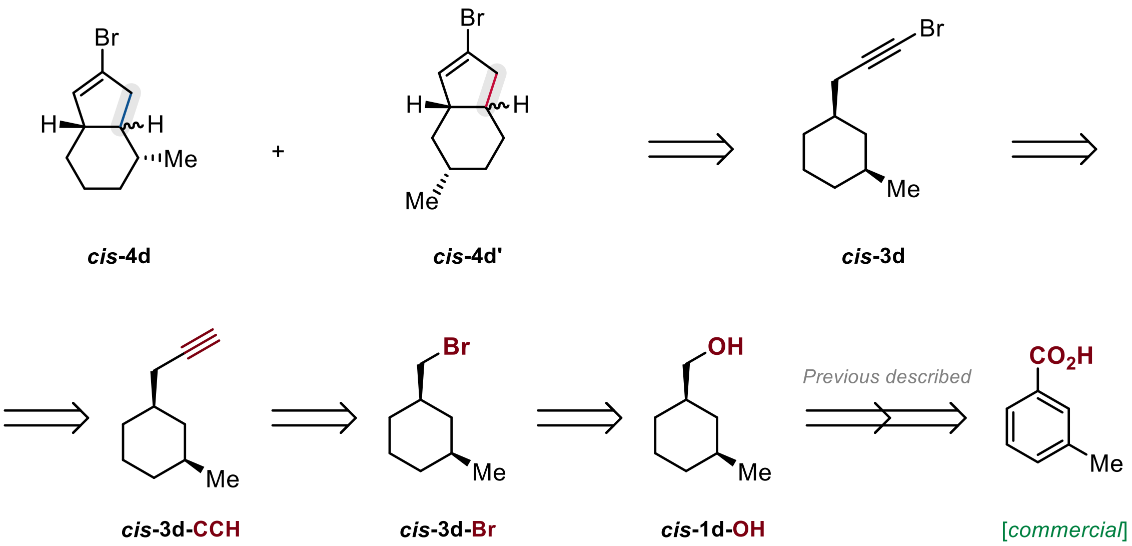

***(1R,3S)-1-(bromomethyl)-3-methylcyclohexane* (*cis-3d-Br*)**

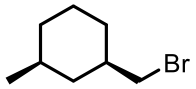

The title compound was synthesized using General Procedure I, starting from 0.63 mmol (121 mg) of alcohol ***cis-1d-OH*** as a 13:1 mixture of diastereoisomers, yielding bromoalkane ***cis-3d-Br*** as a colorless liquid (53 mg, 0.4 mmol, 63%, d.r. = 13:1). The crude was purified by flash column chromatography using n-hexane as eluent.
^{1}H NMR (300 MHz, $CDCl_3$) δ 3.26 (d, J = 6.3 Hz, 2H), 1.92 – 1.71 (m, 3H), 1.71 – 1.56 (m, 2H), 1.51 – 1.36 (m, 1H), 1.28 (qt, J = 13.1, 3.4 Hz, 1H), 0.90 (d, J = 6.5 Hz, 4H), 0.88 – 0.73 (m, 2H), 0.64 (q, J = 12.0 Hz, 1H).
^{13}C NMR (75 MHz, $CDCl_3$) δ 40.9 (CH_2), 40.5 (CH_2), 40.3 (CH), 35.0 (CH_2), 32.5 (CH), 31.5 (CH_2), 25.9 (CH_2), 22.8 (CH_3).

HRMS unstable

***(1S,3R)-1-methyl-3-(prop-2-yn-1-yl)cyclohexane* (*cis-3d-CCH*)**

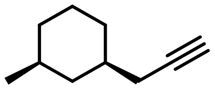

The title compound was synthesized using General Procedure J, starting from 0.4 mmol (54 mg) of bromoalkane ***cis-3d-Br***, yielding alkyne ***cis-3d-CCH*** as a colorless liquid (16 mg, 0.12 mmol, 30%). The crude was filtered through a pad of silica using pentane and the resulting alkyne was used in the next step without further purification.
HRMS not purified

***(1R,3S)-1-(3-bromoprop-2-yn-1-yl)-3-methylcyclohexane* (*cis-3d*)**

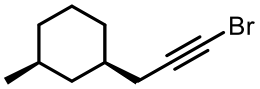

The title compound was synthesized using General Procedure K, starting from 0.12 mmol (16 mg) of alkyne ***cis-3d-CCH***, yielding bromoalkyne ***cis-3d*** as a colorless liquid (24 mg, 0.11 mmol, 94%, d.r. = 17:1). The crude was purified by flash column chromatography using pentane as eluent.
^{1}H NMR (300 MHz, $CDCl_3$) δ 2.10 (d, *J* = 6.6 Hz, 2H), 1.71 (t, *J* = 3.3 Hz, 3H), 1.63 (dt, *J* = 3.2, 1.6 Hz, 1H), 1.57 – 1.46 (m, 1H), 1.46 – 1.31 (m, 1H), 1.25 (d, *J* = 4.4 Hz, 2H), 0.89 (d, *J* = 6.5 Hz, 3H), 0.87 – 0.71 (m, 2H), 0.71 – 0.55 (m, 1H).

^{13}C NMR (75 MHz, $CDCl_3$) δ 79.6 (CH), 41.6 (CH_2), 38.2 (C), 37.3 (CH), 35.0 (CH_2), 32.7 (CH), 32.4 (CH_2), 27.7 (CH_2), 26.2 (CH_2), 22.9 (CH_3).
HRMS (GC-Q-TOF) m/z: $[M-Br]^+$ calcd for $C_{10}H_{15}$: 135.1174; found: 135.1173.

(3aS,7R,7aS)-2-bromo-7-methyl-3a,4,5,6,7,7a-hexahydro-1H-indene (cis-4d-trans), (3aR,4R,7aR)-2-bromo-4-methyl-3a,4,5,6,7,7a-hexahydro-1H-indene (cis-4d-cis), (3aS,5S,7aS)-2-bromo-5-methyl-3a,4,5,6,7,7a-hexahydro-1H-indene (cis-4d'-cis), (3aS,6S,7aR)-2-bromo-6-methyl-3a,4,5,6,7,7a-hexahydro-1H-indene (cis-4d'-trans)

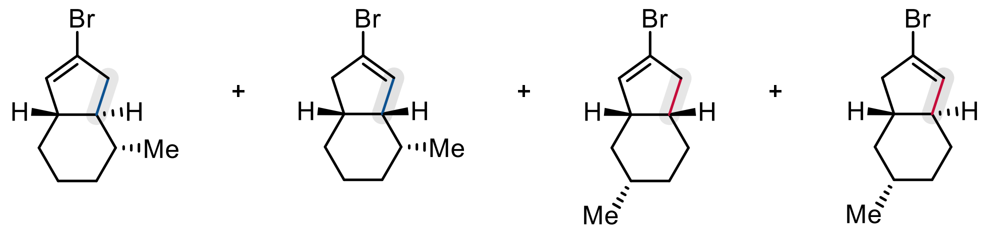

MajorM ***Majorm*** ***minorM*** ***minorm***

cis-4d-trans ***cis-4d-cis*** ***cis-4d'-cis*** ***cis-4d'-trans***

The title compounds were synthesized using General Procedure S, starting from 0.1 mmol (26 mg) of bromoalkyne ***cis-3c***, yielding a mixture of starting material and bromocyclopentenes ***cis-4d-trans***, ***cis-4d-cis***, ***cis-4d'-cis*** *and* ***cis-4d'-trans*** in *41% combined yield by ^{1}H NMR analysis of the crude reaction mixture using CH_2Br_2 as Internal standard as a mixture of regio- and diastereoisomers [2.8(**cis-4d-trans**):2.6(**cis-4d-cis**):2.3 (**cis-4d'-cis**):1(**cis-4d'-trans**)].* The crude was purified by column chromatography using n-hexane as eluent to afford an inseparable mixture of isomers as a colorless liquid (15.2 mg, 0.07 mmol). The determination of the stereochemistry of the major regioisomer has been made by analogy with the corresponding 3,3-dimethyl derivative **4i** that was unambiguously assigned (see below).

^{1}H NMR (600 MHz, CDCl3) δ 6.05 (bs, 1H ***MM***), 5.93 (bs, 1H ***mm***), 5.86 (bs, 1H ***Mm***), 5.67 (bs, 1H ***mM***), 2.87 – 2.74 (m, 2H ***mM***, 1H ***Mm***), 2.72 (m, 1H ***Mm***), 2.45 (m, 1H ***MM***, 1H ***mm***), 2.40 -2.21 (m, 1H ***MM***, 1H ***mm***, 1H ***Mm***, 1H ***mM***), 2.07 (d, J = 16.1 Hz, 1H ***Mm***, d, J = 16.6 Hz 1H ***mM***), 2.01 (m, 1H ***mm***), 1.95 (m, dq, J = 12.3, 3.3 Hz, 1H ***mm***), 1.88 (dq, J = 14.0, 2.8 Hz, 1H ***mM***), 1.85 – 1.63 (m), 1.63 – 1.45 (m), 1.45 – 1.12 (m), 0.98 (d, J = 6.9 Hz, 3H ***Mm***), 0.97 -0.86 (m), 0.84 (d, J = 6.5 Hz, 3H ***mM***).
MajorM **(cis-4d-trans): ^{13}C NMR** (150 MHz, $CDCl_3$) δ 133.9 (CH), 122.2 (C), 58.8 (CH), 49.5 (CH), 45.0 (CH_2), 36.1 (CH), 35.4 (CH_2), 29.2 (CH_2), 26.6 (CH_2), 20.4 (CH_3).
Majorm **(cis-4d-cis): ^{13}C NMR** (150 MHz, $CDCl_3$) δ 130.9 (CH), 121.9 (C), 51.3 (CH), 47.4 (CH_2), 39.1 (CH), 32.7 (CH), 30.6 (CH_2), 28.6 (CH_2), 24.6 (CH_2), 20.7 (CH_3).
MinorM **(cis-4d'-cis): ^{13}C NMR** (150 MHz, $CDCl_3$) δ 135.1 (CH), 121.0 (C), 47.9 (CH_2), 44.1 (CH), 38.4 (CH), 37.9 (CH_2), 32.7 (CH), 30.7 (CH_2), 27.1 (CH_2), 22.8 (CH_3).
Minorm **(cis-4d'-trans): ^{13}C NMR** (150 MHz, $CDCl_3$) δ 135.7 (CH), 122.0 (C), 51.7 (CH), 49.8 (CH), 44.7 (CH_2), 38.1 (CH_2), 35.0 (CH_2), 33.3 (CH), 26.0 (CH_2), 22.5 (CH_3).
HRMS mixture of isomers

1.3.14. *(trans)-3-methyl Family Fused*

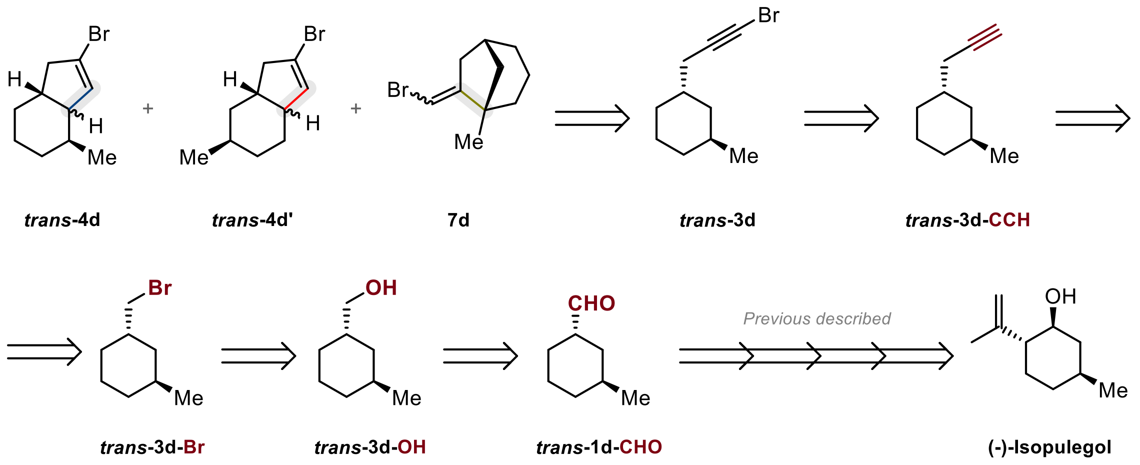

((1R,3R)-3-methylcyclohexyl)methanol (trans-3d-OH)

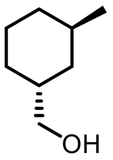

The title compound was synthesized using General Procedure H (using 1 equiv. of $LiAlH_4$), starting from 4.2 mmol (530 mg) of aldehyde ***trans-1d-CHO***, yielding alcohol ***trans-3d-OH*** as a colorless liquid (154.1 mg, 1.2 mmol, 30% yield for two steps from nitrile ***trans-1d-CN***). The crude was purified by column chromatography using hexane/EtOAc (10:1 to 5:1) as the eluent.

^{1}H NMR (300 MHz, $CDCl_3$) δ = 3.51 (dd, *J* = 6.9, 1.7 Hz, 2H), 1.76 (dtd, *J* = 13.9, 7.0, 3.3 Hz, 2H), 1.63 – 1.35 (m, 6H), 1.27 (dd, *J* = 9.3, 6.8 Hz, 3H), 0.90 (d, *J* = 6.9 Hz, 3H).

^{13}C NMR (75 MHz, $CDCl_3$) δ = 66.9 (CH_2), 35.6 (CH_2), 35.5 (CH), 33.7 (CH_2), 28.4 (CH_2), 27.3 (CH), 20.8 (CH_2), 20.6 (CH_3).

(1R,3R)-1-(bromomethyl)-3-methylcyclohexane (trans-3d-Br)

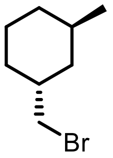

The title compound was synthesized using General Procedure I, starting from 1.2 mmol (154.1 mg) of alcohol ***trans-3d-OH***, yielding bromoalkane ***trans-3d-Br*** as a colorless liquid (160.4 mg, 0.84 mmol, 70%). The crude was purified by flash column chromatography using n-hexane as eluent.

^{1}H NMR (300 MHz, $CDCl_3$) δ = 3.39 (d, *J* = 7.1 Hz, 2H), 2.01 (dt, *J* = 7.2, 3.3 Hz, 1H), 1.88 – 1.63 (m, 2H), 1.48 (ddd, *J* = 16.8, 8.0, 4.7 Hz, 6H), 1.26 – 1.08 (m, 1H), 0.95 (d, *J* = 6.9 Hz, 3H).

^{13}C NMR (75 MHz, $CDCl_3$) δ = 39.5 (CH_2), 37.7 (CH_2), 35.5 (CH), 33.4 (CH_2), 30.5 (CH_2), 27.3 (CH), 20.6 (CH_2), 20.4 (CH_3).

HRMS unstable

(1R,3R)-1-methyl-3-(prop-2-yn-1-yl)cyclohexane (trans-3d-CCH)

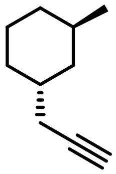

The title compound was synthesized using General Procedure J, starting from 0.84 mmol (160.4 mg) of bromoalkane ***trans-3d-Br***, yielding alkyne ***trans-3d-CCH*** and a small amount of the elimination product as a colorless liquid (72.4 mg, 0.53 mmol, 63%). The crude was purified by flash column chromatography using n-hexane as eluent.
^{1}H NMR (300 MHz, $CDCl_3$) δ = 2.15 (dd, *J* = 7.3, 2.7 Hz, 2H), 1.96 (t, *J* = 2.7 Hz, 1H), 1.85 (td, *J* = 7.3, 3.7 Hz, 1H), 1.75 (dq, *J* = 6.8, 3.5 Hz, 1H), 1.68 – 1.55 (m, 2H), 1.53 – 1.41 (m, 4H), 1.41 – 1.21 (m, 6H), 1.15 (d, *J* = 7.9 Hz, 1H), 0.91 (d, *J* = 6.8 Hz, 7H).
^{13}C NMR (75 MHz, $CDCl_3$) δ = 84.2 (C), 68.9 (CH), 38.5 (CH_2), 33.5 (CH_2), 32.4 (CH), 31.1 (CH_2), 27.3 (CH), 24.1 (CH_2), 20.7 (CH_2), 20.6 (CH), 14.2 (CH_3).
HRMS non-ionizable

(1R,3R)-1-(3-bromoprop-2-yn-1-yl)-3-methylcyclohexane (trans-3d)

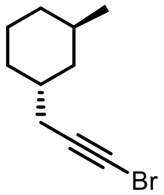

The title compound was synthesized using General Procedure K, starting from 0.48 mmol (65.6 mg) of alkyne ***trans-3d-CCH***, yielding bromoalkyne ***trans-3d*** as a colorless liquid (48.2 mg, 0.22 mmol, 46%). The crude was purified by flash column chromatography using n-hexane as eluent.
^{1}H NMR (400 MHz, $CDCl_3$) δ = 2.16 (d, *J* = 7.3 Hz, 2H), 1.85 (tt, *J* = 7.4, 4.0 Hz, 1H), 1.75 (dtd, *J* = 10.9, 7.0, 3.8 Hz, 1H), 1.55 (s, 5H), 1.48 – 1.38 (m, 3H), 1.38 – 1.22 (m, 4H), 1.20 – 1.10 (m, 1H), 0.91 (d, *J* = 6.9 Hz, 3H), 0.89 – 0.82 (m, 2H).
^{13}C NMR (101 MHz, $CDCl_3$) δ 79.9 (C), 38.5 (CH_2), 38.1 (CH_2), 33.5 (CH_2), 32.3 (CH), 31.1 (CH_2), 27.3 (CH), 25.4 (CH_2), 20.7 (CH_2), 20.6 (CH_3).
HRMS (GC-Q-TOF) m/z: $[M-Br]^+$ calcd for $C_{10}H_{15}$: 135.1174; found: 135.1173.

(1S,5R,E)-7-(bromomethylene)-1-methylbicyclo[3.2.1]octane (7d), (6R,7aR)-2-bromo-6-methyl-3a,4,5,6,7,7a-hexahydro-1H-indene (trans-4d') and (4R,7aS)-2-bromo-4-methyl-3a,4,5,6,7,7a-hexahydro-1H-indene (trans-4d)

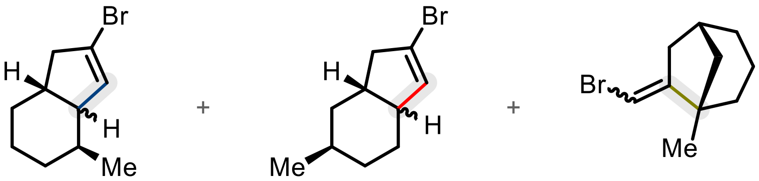

***trans*-4d** ***trans*-4d'** **7d**

major

The title compounds were synthesized using General Procedure S, starting from 0.2 mmol (43.0 mg) of bromoalkyne ***trans-3d***, yielding bromocyclopentenes ***trans-4d-cis***, ***trans-4d'-cis***, ***trans-4d'-trans*** and ***trans-4d-trans*** in *28 % combined yield as a mixture of regio-*

and diastereoisomers[7.5(***trans-4d-cis***):6.8(***trans-4d'-cis***):2(***trans-4d'-trans***):1(***trans-4d-trans***)] and exocyclic bromoolefin ***7d*** in *14% yield* [1]*H by NMR analysis of the crude reaction mixture using* CH_2Br_2 *as Internal standard*. The crude was purified by column chromatography using n-hexane as eluent to afford the title compounds as a colorless liquid (23.3 mg, 0.11 mmol, 54%).

^{1}H NMR (400 MHz, $CDCl_3$) δ = 6.08 (d, *J* = 2.6 Hz, 1H), 5.94 (d, *J* = 2.7 Hz, 1H), 5.90 (d, *J* = 2.3 Hz), 5.85 (s), 5.76 (t, *J* = 2.7 Hz, 1H, ***major***), 2.56 – 2.43 (m, 3H), 2.43 – 2.33 (m, 3H), 2.32 – 2.22 (m, 3H), 1.98 (tdd, *J* = 12.0, 6.2, 3.2 Hz, 2H), 1.83 – 1.59 (m, 6H), 1.58 – 1.41 (m, 14H), 1.40 – 1.32 (m, 4H), 1.26 (s, 10H), 1.08 (s, 4H, ***major***), 1.06 – 0.96 (m, 2H), 0.90 (dd, *J* = 6.5, 2.1 Hz, 14H).

^{13}C NMR (101 MHz, $CDCl_3$) δ = 155.9 (C, ***major***), 137.5, 136.7, 121.8, 120.9, 96.5 (CH, ***major***), 77.5, 76.8, 55.3, 51.6, 47.7 (CH_2, ***major***), 46.5, 45.0, 43.4, 42.8, 42.6, 41.7, 41.5 (CH_2, ***major***), 39.3 (CH_2, ***major***), 39.0, 38.9, 37.5, 35.6, 35.0, 32.5 (CH, ***major***), 32.4 (C, ***major***), 32.2, 32.1, 31.0, 30.8 (CH_2, ***major***), 30.3, 29.9, 29.7, 29.5, 28.9, 27.8, 26.9, 24.4 (CH_3, ***major***), 22.9, 22.4, 21.8, 21.4, 20.9, 20.4, 19.9 (CH_2, ***major***), 19.6, 14.3, 13.3.

HRMS mixture of isomers

1.3.15. *(cis)-4-methyl Family Fused*

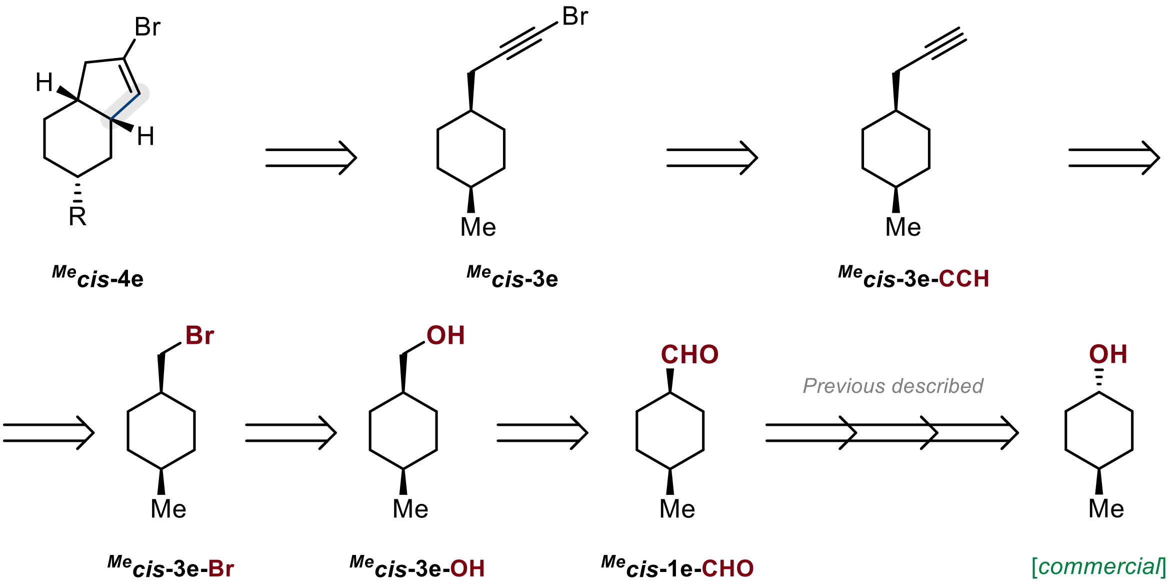

((1s,4s)-4-methylcyclohexyl)methanol (Mecis-3e-OH)

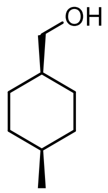

The title compound was synthesized using General Procedure H, starting from 3.77 mmol (476 mg) of aldehyde ***Mecis-1e-CHO***, yielding alcohol ***Mecis-3e-OH*** as a colorless liquid (272 mg, 2.1 mmol, 56%, d.r. = 10:1). The crude alcohol was used in the next step without further purification.

^{1}H NMR (300 MHz, $CDCl_3$) δ = 3.5 (d, *J*=6.9, 2H), 1.9 – 1.6 (m, 3H), 1.6 – 1.3 (m, 6H), 1.3 – 1.2 (m, 2H), 0.9 (d, *J*=6.9, 3H).

^{13}C NMR (75 MHz, $CDCl_3$) δ = 66.4 (CH_2), 38.3 (CH), 30.8 (CH_2), 30.0 (CH), 25.3 (CH_2), 20.1 (CH_3).

HRMS not purified

(1s,4s)-1-(bromomethyl)-4-methylcyclohexane (^{Me}cis-3e-Br)

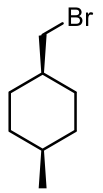

The title compound was synthesized using General Procedure I, starting from 2.1 mmol (272 mg) of alcohol ***Me*****cis-3e-OH**, yielding bromoalkane ***Me*****cis-3e-Br** as a colorless liquid (268 mg, 1.4 mmol, 67%, d.r. = 10:1). The crude was purified by flash column chromatography using n-hexane as eluent.
^{1}H NMR (300 MHz, $CDCl_3$) δ = 3.4 (d, *J*=7.1, 2H), 1.9 – 1.8 (m, 1H), 1.7 – 1.4 (m, 7H), 1.4 – 1.2 (m, 2H), 0.9 (d, *J*=6.9, 3H).
^{13}C NMR (75 MHz, $CDCl_3$) δ = 39.1 (CH_2), 38.2 (CH), 30.6 (CH_2), 29.7 (CH), 27.3 (CH_2), 19.9 (CH_3).
HRMS unstable

(1s,4s)-1-methyl-4-(prop-2-yn-1-yl)cyclohexane (^{Me}cis-3e-CCH)

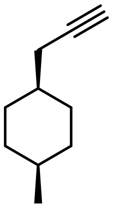

The title compound was synthesized using General Procedure J, starting from 1.4 mmol (268 mg) of bromoalkane ***^{Me}cis-3e-Br***, yielding a mixture of alkyne ***^{Me}cis-3e-CCH*** and a small amount of the elimination product as a colorless liquid (129.4 mg, 0.95 mmol, 68%, d.r. = 7:1). The crude was filtered through a pad of silica using n-hexane as eluent and it was used in the next step without further purification.
^{1}H NMR (300 MHz, $CDCl_3$) δ = 2.2 (dd, *J*=7.2, 2.7, 2H), 2.0 (t, *J*=2.7, 1H), 1.7 – 1.4 (m, 5H), 1.3 (d, *J*=2.5, 4H), 0.9 (d, *J*=6.8, 3H).
HRMS not purified

(1s,4s)-1-(3-bromoprop-2-yn-1-yl)-4-methylcyclohexane (^{Me}cis-3e)

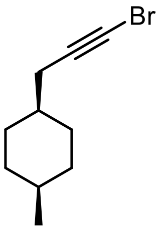

The title compound was synthesized using General Procedure K, starting from 0.86 mmol (117.3 mg) of alkyne ***^{Me}cis-3e-CCH***, yielding bromoalkyne ***^{Me}cis-3e*** as a colorless liquid (79.4 mg, 0.32 mmol, 37%, d.r. = 7:1). The crude was purified by flash column chromatography using n-hexane as eluent.
^{1}H NMR (300 MHz, $CDCl_3$) δ = 2.18 (d, *J* = 7.3 Hz, 2H), 1.66 (dddt, *J* = 17.4, 10.5, 7.0, 3.6 Hz, 2H), 1.57 – 1.38 (m, 6H), 1.33 – 1.18 (m, 3H), 0.90 (d, *J* = 6.9 Hz, 4H).
^{13}C NMR (75 MHz, $CDCl_3$) δ = 79.9 (C), 38.0 (C), 335.1 (CH), 30.6 (CH_2), 29.8 (CH), 28.1 (CH_2), 24.9 (CH_2), 20.2 (CH_3).
HRMS (GC-Q-TOF) m/z: $[M]^+$ calcd for $C_{10}H_{15}Br$: 214.0357; found: 214.0358.

Catalytic reaction of (1s,4s)-1-(3-bromoprop-2-yn-1-yl)-4-methylcyclohexane (^{Me}cis-3e)

The catalytic reaction was performed using General Procedure S, starting from 0.2 mmol (43 mg) of bromoalkyne ^{Me}***cis-3e***, affording a mixture of starting material and bromocyclopentenes ^{Me}***cis-4e-cis*** and ^{Me}***cis-4e-trans*** in *24% yield by* [1]*H NMR analysis of the crude reaction mixture using* CH_2Br_2 *as Internal standard as a mixture of diastereoisomers [5.3(^{Me}**cis-4e-cis**):1(^{Me}**cis-4e-trans**)].*

1.3.16. *(trans)-4-methyl Family Fused*

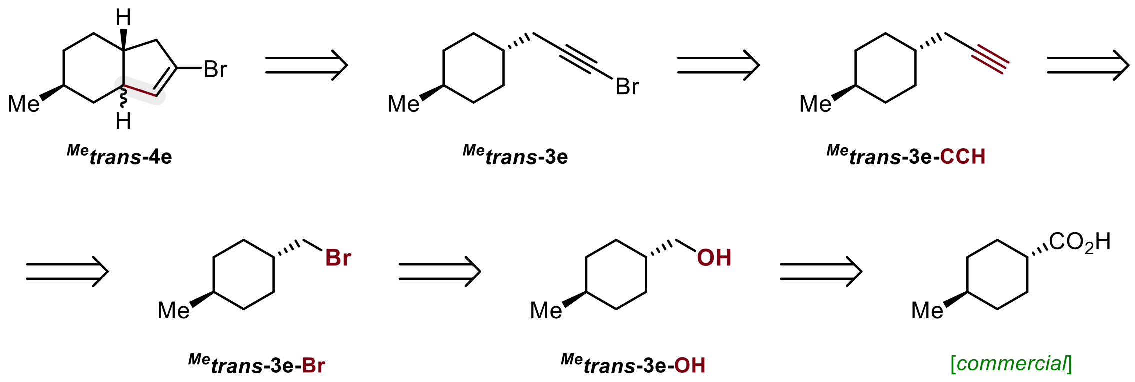

(1r,4r)-1-(bromomethyl)-4-methylcyclohexane (^{Me}trans-3e-Br)

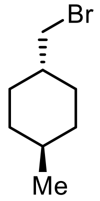

The title compound was synthesized using General Procedure I, starting from 6 mmol (769 mg) of alcohol ^{Me}***trans-3e-OH***, yielding bromoalkane ^{Me}***trans-3e-Br*** as a colorless liquid (892 mg, 4.7 mmol, 78%). The crude was purified by flash column chromatography using n-hexane as eluent.

^{1}H NMR (300 MHz, $CDCl_3$) δ 3.28 (d, *J* = 6.4 Hz, 2H), 1.93 – 1.82 (m, 2H), 1.77 – 1.66 (m, 2H), 1.66 – 1.45 (m, 1H), 1.39 – 1.20 (m, 1H), 1.10 – 0.92 (m, 4H), 0.88 (d, *J* = 6.5 Hz, 4H).

^{13}C NMR (75 MHz, $CDCl_3$) δ 40.9 (CH_2), 40.0 (CH), 34.7 (CH_2), 32.6 (CH), 31.8 (CH_2), 22.5 (CH_3).

HRMS unstable

(1r,4r)-1-methyl-4-(prop-2-yn-1-yl)cyclohexane (^{Me}trans-3e-CCH)

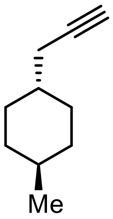

The title compound was synthesized using General Procedure J, starting from 4.7 mmol (892 mg) of bromoalkane ***Me*trans-3e-Br**, yielding alkyne ***Me*trans-3e-CCH** as a colorless liquid (287 mg, 2.1 mmol, 45%). The crude was purified by flash column chromatography using n-hexane as eluent.

^{1}H NMR (300 MHz, $CDCl_3$) δ 2.08 (dd, *J* = 6.6, 2.7 Hz, 2H), 1.95 (t, *J* = 2.7 Hz, 1H), 1.82 (dt, *J* = 10.8, 2.3 Hz, 2H), 1.76 – 1.63 (m, 2H), 1.49 – 1.17 (m, 3H), 1.11 – 0.91 (m, 4H), 0.87 (d, *J* = 6.5 Hz, 5H).

^{13}C NMR (75 MHz, $CDCl_3$) δ 83.8 (C), 69.0 (CH), 37.0 (CH), 35.1 (CH_2), 32.7 (CH_2), 32.6 (CH), 26.2 (CH_2), 22.7 (CH_3).

HRMS non-ionizable

(1r,4r)-1-(3-bromoprop-2-yn-1-yl)-4-methylcyclohexane (*Me*trans-3e)

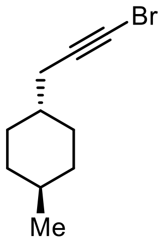

The title compound was synthesized using General Procedure K, starting from 2 mmol (272 mg) of alkyne ***Me*trans-3e-CCH**, yielding bromoalkyne ***Me*trans-3e** as a colorless liquid (409 mg, 1.9 mmol, 95%). The crude was purified by flash column chromatography using n-hexane as eluent.

^{1}H NMR (300 MHz, $CDCl_3$) δ 2.09 (d, *J* = 6.6 Hz, 2H), 1.85 – 1.74 (m, 2H), 1.74 – 1.63 (m, 2H), 1.49 – 1.18 (m, 2H), 1.09 – 0.91 (m, 3H), 0.87 (d, *J* = 6.6 Hz, 4H).

^{13}C NMR (75 MHz, $CDCl_3$) δ 79.6 (C), 38.2 (C), 37.0 (CH), 35.0 (CH_2), 32.7 (CH_2), 32.6 (CH), 27.5 (CH_2), 22.7 (CH_3).

HRMS (GC-Q-TOF) m/z: $[M-CH_3]^+$ calcd for $C_9H_{12}Br$: 199.0122; found: 199.0131.

(3aS,5R,7aS)-2-bromo-5-methyl-3a,4,5,6,7,7a-hexahydro-1H-indene (*Me*trans-4e-cis) and (3aR,6R,7S,7aR)-2-bromo-6,7-dimethyl-3a,4,5,6,7,7a-hexahydro-1H-indene (*Me*trans-4e-trans)

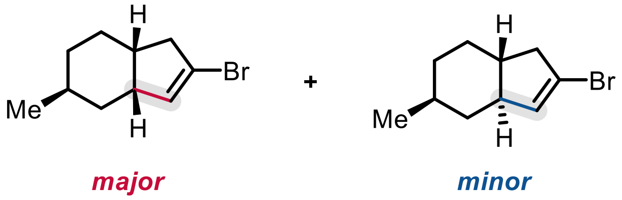

The title compounds were synthesized using General Procedure S, starting from 0.2 mmol (43.0 mg) of bromoalkyne ***Me*trans-3e**, yielding bromocyclopentenes ***Me*trans-4e-cis** and ***Me*trans-4e-trans** in *62 % yield as a mixture of diastereoisomers [2.9(**Me**trans-4e-cis**):1(**Me**trans-4e-trans**)] by ^{1}H NMR analysis of the crude reaction mixture using CH_2Br_2 as Internal standard*. The crude was purified by column chromatography using n-hexane as eluent to afford an inseparable mixture of regioisomers as a colorless liquid (12 mg, 0.06 mmol, 28%).

^{1}H NMR (400 MHz, $CDCl_3$) δ = 5.88 (t, J = 1.8 Hz, 1H), 5.70 (d, J = 2.0 Hz, 3H), 2.80 (dd, J = 6.3, 2.1 Hz, 5H), 2.74 (td, J = 3.7, 1.9 Hz, 2H), 2.47 – 2.24 (m, 4H), 2.21 – 2.07 (m, 7H), 2.04 (d, J = 1.9 Hz, 2H), 1.93 (dt, J = 12.0, 3.2 Hz, 2H), 1.86 – 1.75 (m, 7H), 1.75 – 1.65 (m, 7H), 1.65 – 1.51 (m, 7H), 1.51 – 1.45 (m, 3H), 1.44 – 1.22 (m, 13H), 1.15 (ddd, J = 13.6, 11.6, 5.7 Hz, 4H), 0.92 (d, J = 6.6 Hz, 6H), 0.88 (d, J = 1.2 Hz, 4H), 0.85 (d, J = 6.5 Hz, 14H).

^{13}C NMR (101 MHz, $CDCl_3$, ***major***) δ = 135.1 (CH), 120.5 (C), 47.1 (CH_2), 44.6 (CH), 37.3 (CH), 35.7 (CH_2), 32.5 (CH_2), 28.5 (CH_2), 27.8 (CH), 22.1 (CH_3).
^{13}C NMR (101 MHz, $CDCl_3$, ***minor***) δ = 135.5 (CH_3), 121.5 (C), 51.4 (CH), 49.6 (CH), 44.2 (CH_2), 38.3 (CH_2), 35.2 (CH_2), 32.8 (CH), 28.9 (CH_2), 22.3 (CH_3).
HRMS (GC-Q-TOF) m/z: $[M]^+$ calcd for $C_{10}H_{15}Br$: 214.0357; found: 214.0359.

1.3.17. (cis)-4-isopropyl Family Fused

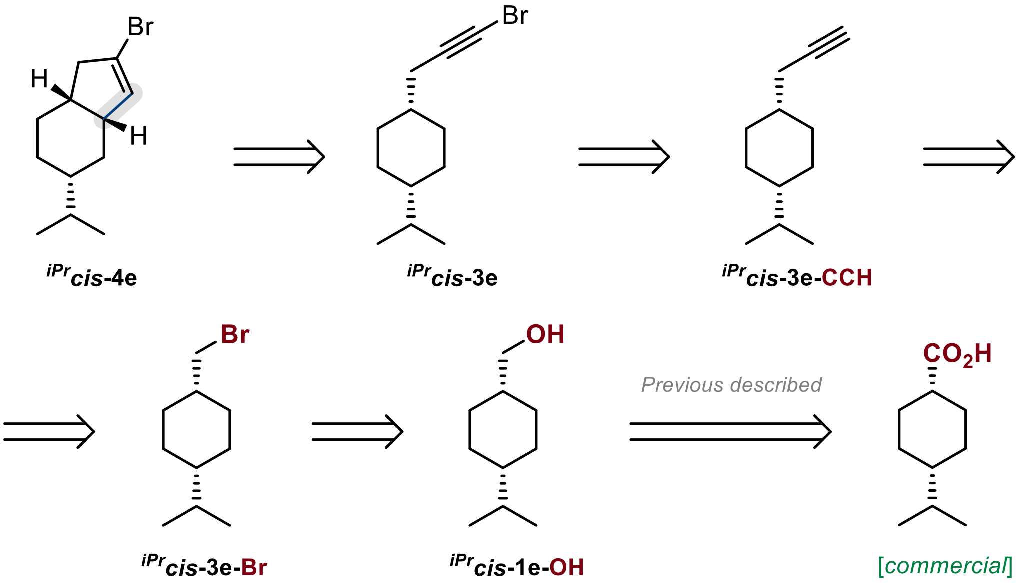

(1s,4s)-1-(bromomethyl)-4-isopropylcyclohexane (^{iPr}cis-3e-Br)

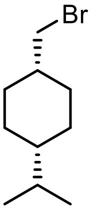

The title compound was synthesized using General Procedure I, starting from 3.2 mmol (500 mg) of alcohol ***^{iPr}cis-1e-OH***, yielding bromoalkane ***^{iPr}cis-3e-Br*** as a colorless liquid (689 mg, 3.1 mmol, 97%, d.r. = 20:1). The crude was purified by flash column chromatography using n-hexane as eluent.
^{1}H NMR (300 MHz, $CDCl_3$) δ 3.41 (d, J = 7.3 Hz, 1H), 1.92 (tt, J = 7.3, 5.0 Hz, 1H), 1.66 – 1.23 (m, 5H), 1.10 (dq, J = 8.0, 4.0 Hz, 1H), 0.86 (d, J = 6.7 Hz, 3H).
^{13}C NMR (75 MHz, $CDCl_3$) δ 42.8 (CH), 38.5 (CH_2), 37.5 (CH), 30.3 (CH), 28.2 (CH_2), 25.5 (CH_2), 20.4 (CH_3).
HRMS unstable

(1s,4s)-1-isopropyl-4-(prop-2-yn-1-yl)cyclohexane (^{iPr}cis-3e-CCH)

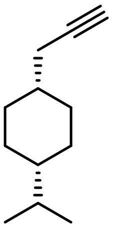

The title compound was synthesized using General Procedure J, starting from 3.1 mmol (689 mg) of bromoalkane ***^{iPr}cis-3e-Br***, yielding alkyne ***^{iPr}cis-3e-CCH*** as a colorless liquid

(378 mg, 2.3 mmol, 74%, d.r. = 10:1). The crude was purified by flash column chromatography using n-hexane as eluent.
^{1}H NMR (300 MHz, Chloroform-d) δ 2.18 (dd, *J* = 7.5, 2.7 Hz, 2H), 1.95 (d, *J* = 2.7 Hz, 1H), 1.79 (ddt, *J* = 10.2, 5.1, 2.6 Hz, 3H), 1.60 – 1.49 (m, 6H), 1.49 – 1.30 (m, 6H), 1.08 (ddt, *J* = 13.3, 10.8, 3.5 Hz, 3H), 0.86 (d, *J* = 6.8 Hz, 13H).

HRMS not purifiednon-ionizable

(1s,4s)-1-(3-bromoprop-2-yn-1-yl)-4-isopropylcyclohexane (^{iPr}cis-3e)

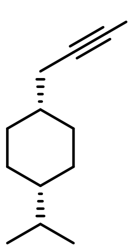

The title compound was synthesized using General Procedure K, starting from 2.29 mmol (377 mg) of alkyne ***^{iPr}cis-3e-CCH***, yielding bromoalkyne ***^{iPr}cis-3e*** as a colorless liquid (462 mg, 1.9 mmol, 83%, d.r. = 10:1). The crude was purified by flash column chromatography using n-hexane as eluent.
^{1}H NMR (300 MHz, $CDCl_3$) δ 2.20 (d, *J* = 7.5 Hz, 2H), 1.87 – 1.69 (m, 1H), 1.58 – 1.17 (m, 10H), 1.07 (tt, *J* = 8.3, 3.6 Hz, 1H), 0.86 (d, *J* = 6.7 Hz, 7H).
^{13}C NMR (75 MHz, $CDCl_3$) δ 80.0 (C), 42.9 (CH), 38.0 (C), 34.4 (CH_2), 30.5 (CH), 28.9 (CH_2), 25.4 (CH_2), 24.0 (CH), 20.4 (CH_3).
HRMS (GC-Q-TOF) m/z: $[M-CH_3]^+$ calcd for $C_{11}H_{16}Br$: 227.0435; found: 227.0432.

(3aS,5S,7aS)-2-bromo-5-isopropyl-3a,4,5,6,7,7a-hexahydro-1H-indene (^{iPr}cis-4e)

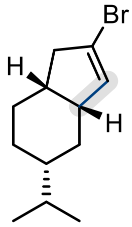

The title compound was synthesized using General Procedure S, starting from 0.1 mmol (24 mg) of bromoalkyne ***^{iPr}cis-3e***, yielding bromocyclopentene ***^{iPr}cis-4e*** in *53% yield as a mixture of diastereoisomers [11.1(**^{iPr}cis-4e-cis**):1(**^{iPr}cis-4e-trans**)]by NMR analysis of the crude reaction mixture using CH_2Br_2 as Internal standard*. The crude was purified by column chromatography using n-hexane as eluent to afford the title compounds as a colorless liquid (19.9 mg, 0.08 mmol, 82%).
^{1}H NMR (300 MHz, $CDCl_3$) δ 5.94 (d, *J* = 2.3 Hz, 1H), 2.55 – 2.28 (m, 3H), 1.71 (d, *J* = 4.7 Hz, 3H), 1.62 – 1.18 (m, 6H), 1.17 – 0.59 (m, 6H).
^{13}C NMR (75 MHz, $CDCl_3$) δ 137.5 (CH), 120.8 (C), 44.6 (CH), 42.3 (CH_2), 41.2 (CH), 38.3 (CH), 32.8 (CH), 32.2 (CH_2), 27.0 (CH_2), 25.1 (CH_2), 19.8 (CH_3).
HRMS (GC-Q-TOF) m/z: $[M-CH_3]^+$ calcd for $C_{11}H_{16}Br$: 227.0435; found: 227.0432.

1.3.18. *(trans)-4-isopropyl Family Fused*

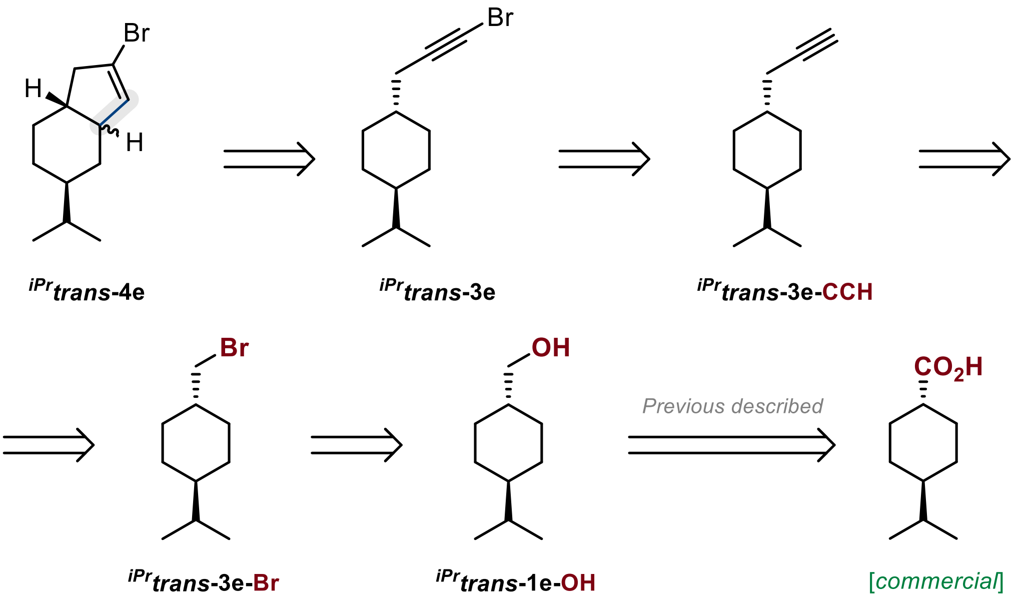

(1r,4r)-1-(bromomethyl)-4-isopropylcyclohexane (iPrtrans-3e-Br)

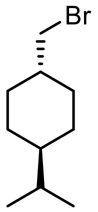

The title compound was synthesized using General Procedure I, starting from 5 mmol (781 g) of alcohol ***iPrtrans-1e-OH***, yielding bromoalkane ***iPrtrans-3e-Br*** as a colorless liquid (986 mg, 4.5 mmol, 90%). The crude was purified by flash column chromatography using n-hexane as eluent.

^{1}H NMR (300 MHz, $CDCl_3$) δ 3.27 (d, J = 6.3 Hz, 2H), 2.03 – 1.83 (m, 2H), 1.75 (q, J = 2.7 Hz, 2H), 1.53 (s, 1H), 1.42 (d, J = 1.9 Hz, 1H), 1.12 – 0.94 (m, 5H), 0.86 (d, J = 6.8 Hz, 7H).

^{13}C NMR (75 MHz, $CDCl_3$) δ 43.9 (CH), 40.9 (CH_2), 40.4 (CH), 32.8 (CH), 32.0 (CH_2), 29.3 (CH_2), 20.0 (CH_3).

HRMS unstable

(1r,4r)-1-isopropyl-4-(prop-2-yn-1-yl)cyclohexane (iPrtrans-3e-CCH)

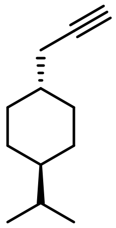

The title compound was synthesized using General Procedure J, starting from 4.4 mmol (954 g) of bromoalkane ***iPrtrans-3e-Br***, yielding alkyne ***iPrtrans-3e-CCH*** as a colorless liquid (534 mg, 3.25 mmol, 74%). The crude was purified by flash column chromatography using n-hexane as eluent.

^{1}H NMR (300 MHz, $CDCl_3$) δ 2.08 (dd, J = 6.6, 2.6 Hz, 2H), 1.95 (t, J = 2.7 Hz, 1H), 1.93 – 1.82 (m, 2H), 1.72 (dd, J = 5.6, 2.5 Hz, 2H), 1.51 – 1.32 (m, 2H), 0.99 (d, J = 2.1 Hz, 5H), 0.85 (d, J = 6.8 Hz, 8H).

^{13}**C NMR** (75 MHz, $CDCl_3$) δ 83.8 (C), 69.0 (CH), 43.9 (CH), 37.4 (CH), 33.0 (CH), 32.8 (CH_2), 29.6 (CH_2), 26.2 (CH_2), 20.0 (CH_3).
HRMS non-ionizable

(1r,4r)-1-(3-bromoprop-2-yn-1-yl)-4-isopropylcyclohexane (^{iPr}trans-3e)

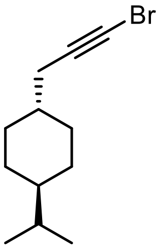

The title compound was synthesized using General Procedure K, starting from 2 mmol (329 mg) of alkyne ***^{iPr}trans-3e-CCH***, yielding bromoalkyne (***^{iPr}trans-3e***) as a colorless liquid (392 mg, 1.6 mmol, 81%). The crude was purified by flash column chromatography using n-hexane as eluent.
^{1}**H NMR** (300 MHz, $CDCl_3$) δ 2.10 (d, J = 6.6 Hz, 2H), 1.94 – 1.78 (m, 2H), 1.72 (dd, J = 5.5, 2.6 Hz, 2H), 1.51 – 1.26 (m, 2H), 0.98 (d, J = 3.2 Hz, 5H), 0.85 (d, J = 6.8 Hz, 6H).
^{13}**C NMR** (75 MHz, $CDCl_3$) δ 79.6 (C), 43.9 (CH), 38.2 (C), 37.4 (CH), 32.9 (CH), 32.9 (CH_2), 29.5 (CH_2), 27.5 (CH_2), 20.0 (CH_3).
HRMS (GC-Q-TOF) m/z: $[M-CH_3]^+$ calcd for $C_{11}H_{16}Br$: 227.0435; found: 227.0433.

Catalytic reaction of (1r,4r)-1-(3-bromoprop-2-yn-1-yl)-4-isopropylcyclohexane (^{iPr}trans-3e)

The catalytic reaction was performed using General Procedure S, starting from 0.2 mmol (49 mg) of bromoalkyne ***^{iPr}trans-3e***, affording a mixture of starting material and bromocyclopentenes ***^{iPr}trans-4e-cis*** and ***^{iPr}trans-4e-trans*** in *22% yield as a mixture of diastereoisomers [1.6(**^{iPr}trans-4e-cis**):1(**^{iPr}trans-4e-trans**)]by ^{1}H NMR analysis of the crude reaction mixture using CH_2Br_2 as internal standard.*

1.3.19. (cis)-4-tertbutyl Family Fused

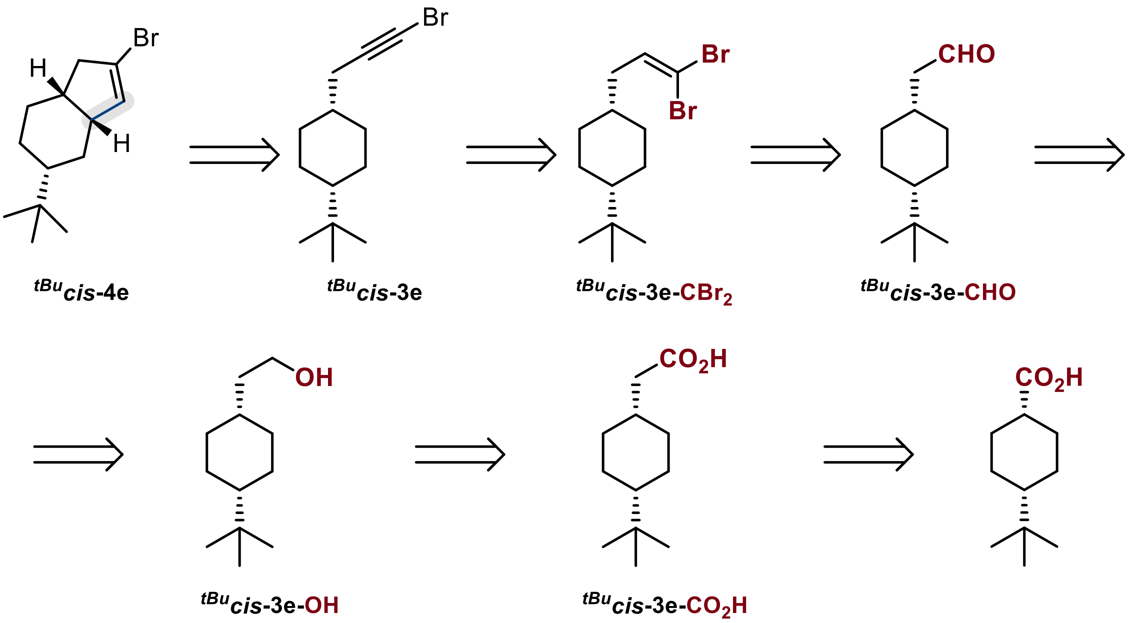

2-((1s,4s)-4-(tert-butyl)cyclohexyl)acetic acid (^{tBu}cis-3e-CO_2H)

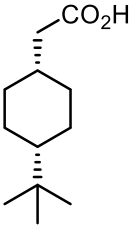

The title compound was synthesized using General Procedure B, starting from 2.9 mmol (543 mg) of carboxylic acid ***^{tBu}cis-1e-CO_2H***, yielding carboxylic acid ***^{tBu}cis-3e-CO_2H*** as a colorless liquid (455 mg, 2.3 mmol, 78%). The crude acid was used in the next step without further purification.
^{1}H NMR (300 MHz, $CDCl_3$) δ 11.19 (s, 1H), 2.40 (d, *J* = 7.6 Hz, 2H), 2.33 – 2.19 (m, 1H), 1.74 – 1.60 (m, 2H), 1.60 – 1.42 (m, 4H), 1.19 – 0.90 (m, 3H), 0.83 (s, 9H).
^{13}C NMR (75 MHz, $CDCl_3$) δ 180.4 (C), 48.4 (CH_2), 36.5 (CH), 32.7 (C), 30.6 (CH_2), 29.7 (CH), 27.6 (CH_3), 21.7 (CH_2).
HRMS (ESI-TOF) m/z: $[M+H]^+$ calcd for $C_{12}H_{23}O_2$: 199.1693; found: 199.1694.

2-((1s,4s)-4-(tert-butyl)cyclohexyl)ethan-1-ol (^{tBu}cis-3e-OH)

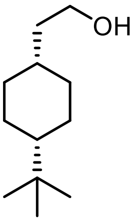

The title compound was synthesized using General Procedure H, starting from 2.3 mmol (455 mg) of carboxylic acid ***^{tBu}cis-3e-CO_2H***, yielding alcohol ***^{tBu}cis-3e-OH*** as a colorless liquid (401.3 mg, 2.2 mmol, 95%). The crude alcohol was used in the next step without further purification.
^{1}H NMR (300 MHz, $CDCl_3$) δ 3.63 (t, *J* = 7.0 Hz, 2H), 1.79 (d, *J* = 2.6 Hz, 1H), 1.60 (q, *J* = 7.1 Hz, 4H), 1.54 – 1.36 (m, 5H), 1.26 – 0.87 (m, 4H), 0.81 (s, 11H).

^{13}C NMR (75 MHz, $CDCl_3$) δ 61.8 (CH_2), 48.6 (CH), 34.1 (CH_2), 32.7 (C), 30.8 (CH_2), 28.9 (CH), 27.6 (CH_3), 21.8 (CH_2).
HRMS not purified

2-((1s,4s)-4-(tert-butyl)cyclohexyl)acetaldehyde (^{tBu}cis-3e-CHO)

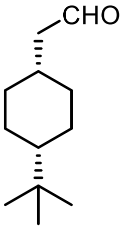

The title compound was synthesized using General Procedure P, starting from 2.2 mmol (401.3 mg) of alcohol ***^{tBu}cis-3e-OH***, yielding aldehyde ***^{tBu}cis-3e-CHO*** as a colorless liquid (386 mg, 2.1 mmol, 96%). The crude aldehyde was used in the next step without further purification.
^{1}H NMR (300 MHz, $CDCl_3$) δ 9.73 (d, J = 2.6 Hz, 1H), 2.54 – 2.25 (m, 3H), 1.54 (d, J = 14.3 Hz, 8H), 1.25 – 0.93 (m, 4H), 0.82 (s, 13H).
^{13}C NMR (75 MHz, $CDCl_3$) δ 203.2 (CH), 48.3 (CH), 45.8 (CH_2), 32.7 (C), 30.8 (CH_2), 27.6 (CH_3), 27.3 (CH), 21.7 (CH_2).
HRMS not purified

(1s,4s)-1-(tert-butyl)-4-(3,3-dibromoallyl)cyclohexane (^{tBu}cis-3e-CBr_2)

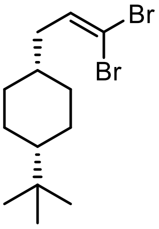

The title compound was synthesized using General Procedure Q, starting from 2.1 mmol (386 mg) of aldehyde ***^{tBu}cis-3e-CHO***, yielding dibromoolefin ***^{tBu}cis-3e-CBr_2*** as a colorless liquid (487 mg, 1.4 mmol, 69%). The crude was purified by flash column chromatography using n-hexane as eluent.
^{1}H NMR (300 MHz, $CDCl_3$) δ 6.37 (t, J = 7.3 Hz, 1H), 2.16 (t, J = 7.6 Hz, 2H), 1.85 (dd, J = 5.2, 2.7 Hz, 1H), 1.68 – 1.37 (m, 7H), 1.37 – 0.95 (m, 4H), 0.85 (s, 10H).**^{13}C NMR** (75 MHz, $CDCl_3$) δ 138.9 (CH), 88.6 (C), 48.4 (CH), 35.0 (CH_2), 32.7 (C), 32.1 (CH), 30.4 (CH_2), 27.6 (CH_3), 21.8 (CH_2).
HRMS unstable

(1s,4s)-1-(3-bromoprop-2-yn-1-yl)-4-(tert-butyl)cyclohexane (^{tBu}cis-3e)

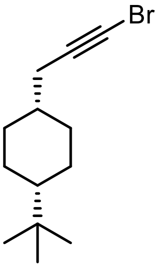

The title compound was synthesized using General Procedure R, starting from 1.4 mmol (487 mg) of dibromoolefin ***^{tBu}cis-3e-CBr_2***, yielding bromoalkyne ***^{tBu}cis-3e*** as a colorless

liquid (346 mg, 1.3 mmol, 93%). The crude was purified by flash column chromatography using n-hexane as eluent.
^{1}H NMR (300 MHz, $CDCl_3$) δ 2.24 (d, *J* = 8.0 Hz, 2H), 1.95 (dtd, *J* = 7.9, 4.9, 2.4 Hz, 1H), 1.85 – 1.70 (m, 2H), 1.60 – 1.41 (m, 4H), 1.15 – 0.90 (m, 3H), 0.84 (s, 9H).
^{13}C NMR (75 MHz, $CDCl_3$) δ 80.2 (C), 48.4 (CH), 38.0 (C), 32.6 (C), 32.4 (CH), 30.0 (CH_2), 27.6 (CH_3), 22.0 (CH_2), 21.6 (CH_2).
HRMS (GC-Q-TOF) m/z: $[M-CH_3]^+$ calcd for $C_{12}H_{18}Br$: 241.0592; found: 241.0592.

(3aS,5S,7aS)-2-bromo-5-(tert-butyl)-3a,4,5,6,7,7a-hexahydro-1H-indene (^{tBu}cis-4e)

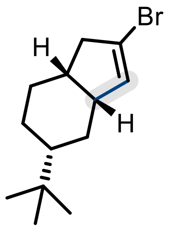

The title compound was synthesized using General Procedure S, starting from 0.2 mmol (51 mg) of bromoalkyne ***^{tBu}cis-3e***, yielding the bromocyclopentene ***^{tBu}cis-4e*** in *91% yield* (dr > 20:1*) by ^{1}H NMR analysis of the crude reaction mixture using CH_2Br_2 as internal standard*). The crude was purified by column chromatography using n-hexane as eluent to afford the title compound as a colorless liquid (43.1 mg, 0.17 mmol, 84%).
^{1}H NMR (300 MHz, $CDCl_3$) δ 5.93 (q, *J* = 1.6, 1.1 Hz, 1H), 2.53 – 2.32 (m, 4H), 1.74 (m, 2H), 1.67 – 1.45 (m, 3H), 1.14 – 1.00 (m, 1H), 0.93 (dt, *J* = 11.0, 2.3 Hz, 1H), 0.83 (s, 10H), 0.77 – 0.60 (m, 1H).
^{13}C NMR (75 MHz, $CDCl_3$) δ 137.3 (CH), 120.8 (C), 45.3 (CH), 45.1 (CH), 42.5 (CH_2), 37.8 (CH), 32.7 (C), 29.9 (CH_2), 27.6 (CH_3), 27.4 (CH_2), 23.0 (CH_2).
HRMS (GC-Q-TOF) m/z: $[M]^+$ calcd for $C_{13}H_{21}Br$: 256.0827; found: 256.0830.

1.3.20. (trans)-4-tert-butyl Family Fused

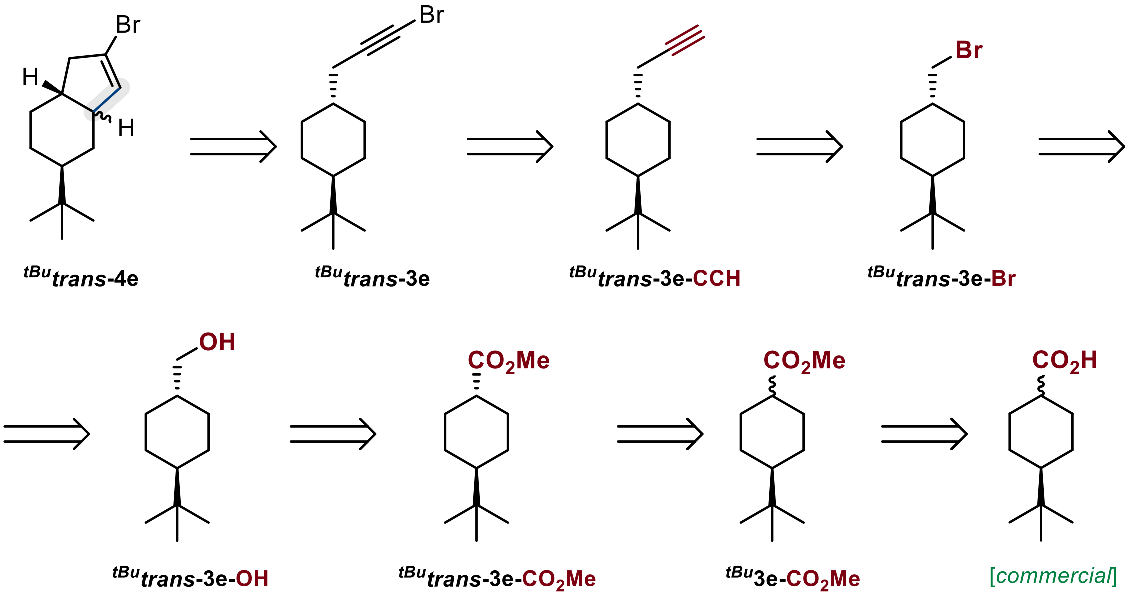

methyl 4-(tert-butyl)cyclohexane-1-carboxylate (tBu3e-CO2Me)

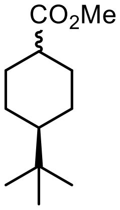

The procedure described in the literature was followed.[20] In a round bottom flask, 6.7 mmol (1.23 g, 1equiv.) of *4-(tert-butyl)cyclohexane-1-carboxylic acid* was dissolved in MeOH (0.5M) and stirred under reflux (75 °C) overnight. After completion of the reaction, HCl 1M was added. The mixture was diluted with Et_2O, washed with $NaHCO_3$ (sat.) and brine. The organic layer was dried over Na_2SO_4, filtered and the solvent removed under vacuum to afford the crude ester ***^{tBu}3e-CO$_2$Me*** as a colorless liquid (1.12 g, 6.3 mmol, 94%). The compound was used in the next step without further purification as a mixture of diastereosiomers 5:1 (***^{tBu}cis-3e-CO$_2$Me***: ***^{tBu}trans-3e-CO$_2$Me***). ^{1}H NMR spectrum matches the one previously reported. [20]

^{1}H NMR (300 MHz, $CDCl_3$) δ 3.66 (d, *J* = 1.4 Hz, 3H), 3.63 (d, *J* = 1.4 Hz, ***minor***), 2.60 (t, *J* = 3.7 Hz, 1H), 2.24 – 2.13 (m, 2H), 1.68 – 1.56 (m, 2H), 1.50 – 1.33 (m, 3H), 1.20 – 1.01 (m, 2H), 1.01 – 0.89 (m, 2H), 0.82 (d, *J* = 1.3 Hz, ***minor***), 0.80 (d, *J* = 1.4 Hz, 10H). **HRMS** commercially available

methyl (1r,4r)-4-(tert-butyl)cyclohexane-1-carboxylate (^{tBu}trans-3e-CO$_2$Me)

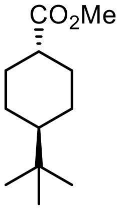

The procedure was followed as described in the literature.[21] In a round bottom flask, ester ***^{tBu}3e-CO$_2$Me*** 6.7 mmol (1.329 g, 1 equiv.) and NaH 60% dispersed in mineral oil (0.1 equiv., 0.67 mmol, 26.8 mg) were stirred at 150 °C monitored by NMR until a 1:5 (***^{tBu}cis-3e-CO$_2$Me***: ***^{tBu}trans-3e-CO$_2$Me***) ratio was approximately observed. After completion of the reaction, the mixture was allowed to cool down to room temperature, and the mixture was diluted with Et_2O and washed carefully with water and brine. The organic layer was dried over Na_2SO_4, filtered and the solvent removed under vacuum to afford the crude ester (***^{tBu}trans-3e-CO$_2$Me***) as a colorless liquid. The compound was used in the next step without further purification as a 5:1 mixture of diastereosiomers (***^{tBu}trans-3e-CO$_2$Me***: ***^{tBu}cis-3e-CO$_2$Me***).

^{1}H NMR (300 MHz, $CDCl_3$) δ 3.68 (s, ***minor***), 3.65 (s, 3H), 2.20 (tt, *J* = 12.3, 3.6 Hz, 2H), 2.01 (d, *J* = 12.8 Hz, 2H), 1.89 – 1.77 (m, 2H), 1.63 (d, *J* = 13.3 Hz, 1H), 1.41 (dd, *J* = 18.1, 8.9 Hz, 3H), 1.14 – 1.04 (m, 1H), 1.01 (d, *J* = 8.1 Hz, 3H), 0.84 (s, 9H), 0.82 (s, ***minor***).

((1r,4r)-4-(tert-butyl)cyclohexyl)methanol (^{tBu}trans-3e-OH)

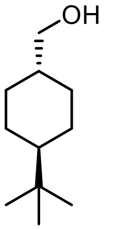

The title compound was synthesized using General Procedure H, starting from 4.1 mmol (747 mg) of ester ***tBu*trans-3e-CO$_2$Me**, to yield alcohol ***tBu*trans-3e-OH** as a colorless liquid (423 mg, 2.5 mmol, 61%, dr 5:1). The crude was purified by column chromatography using hexane/EtOAc (5:1) as eluent.

^{1}H NMR (300 MHz, $CDCl_3$) δ 3.64 (d, J = 7.6 Hz, 1H, ***minor***), 3.44 (d, *J* = 6.3 Hz, 2H), 1.82 (t, *J* = 8.7 Hz, 5H), 1.62 – 1.35 (m, 3H), 1.27 (s, 1H), 1.10 – 0.90 (m, 5H), 0.84 (s, 9H).

^{13}C NMR (75 MHz, $CDCl_3$) δ 68.9 (CH_2), 48.4 (CH), 40.7 (CH), 30.1 (CH_2), 27.7 (CH_3), 27.6 (C), 26.9 (CH_2).

(1r,4r)-1-(bromomethyl)-4-(tert-butyl)cyclohexane (tBu trans-3e-Br)

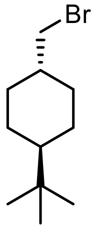

The title compound was synthesized using General Procedure I, starting from 2.4 mmol (416 mg) of alcohol ***tBu*trans-3e-OH**, yielding bromoalkane ***tBu*trans-3e-Br** as a colorless liquid (394 mg, 1.7 mmol, 70%, dr 10:1). The crude was purified by flash column chromatography using n-hexane as eluent.

^{1}H NMR (300 MHz, $CDCl_3$) δ 3.27 (d, *J* = 6.3 Hz, 2H), 1.93 (d, *J* = 9.5 Hz, 2H), 1.80 (d, *J* = 8.3 Hz, 2H), 1.55 (s, 2H), 0.99 (d, *J* = 8.6 Hz, 5H), 0.84 (d, *J* = 1.4 Hz, 9H).

^{13}C NMR (75 MHz, $CDCl_3$) δ 48.0 (CH), 40.8 (CH_2), 40.4 (CH), 32.5 (C), 32.3 (CH_2), 27.7 (CH_3), 27.0 (CH_2).

HRMS unstable

(1r,4r)-1-(tert-butyl)-4-(prop-2-yn-1-yl)cyclohexane (tBu trans-3e-CCH)

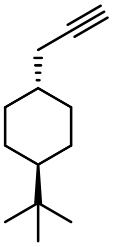

The title compound was synthesized using General Procedure J, starting from 1.7 mmol (394 mg) of bromoalkane ***tBu*trans-3e-Br**, yielding alkyne ***tBu*trans-3e-CCH** as a colorless liquid (198 mg, 1.1 mmol, 66%) and a single diastereoisomer. The crude was purified by flash column chromatography using n-hexane as eluent.

^{1}H NMR (300 MHz, $CDCl_3$) δ 2.08 (dd, *J* = 6.6, 2.7 Hz, 2H), 1.95 (t, *J* = 2.7 Hz, 1H), 1.92 – 1.74 (m, 4H), 1.49 – 1.32 (m, 1H), 0.99 (ddd, *J* = 9.1, 6.2, 2.6 Hz, 5H), 0.84 (s, 9H).

^{13}C NMR (75 MHz, $CDCl_3$) δ 83.8 (C), 69.0 (CH), 48.0 (CH), 37.4 (CH), 33.1 (CH_2), 32.5 (C), 27.7 (CH_3), 27.2 (CH_2), 26.2 (CH_2).

HRMS non-ionizable

(1r,4r)-1-(3-bromoprop-2-yn-1-yl)-4-(tert-butyl)cyclohexane (tBu trans-3e)

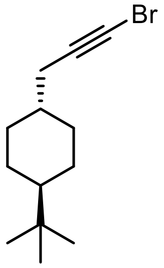

The title compound was synthesized using General Procedure K, starting from 1.0 mmol (180 mg) of the alkyne (***tBu trans-3e-CCH***), yielding the bromoalkyne (***tBu trans-3e***) as a colorless liquid (216 mg, 0.84 mmol, 83%). The crude was purified by flash column chromatography using n-hexane as eluent.
^{1}H NMR (300 MHz, $CDCl_3$) δ 2.09 (d, *J* = 6.5 Hz, 2H), 1.90 – 1.71 (m, 4H), 1.47 – 1.29 (m, 1H), 0.97 (tt, *J* = 6.5, 3.2 Hz, 5H), 0.84 (s, 9H).
^{13}C NMR (75 MHz, $CDCl_3$) δ 77.6 (C), 47.9 (CH), 38.2 (C), 37.3 (CH), 33.1 (CH_2), 32.5 (C), 27.7 (CH_3), 27.4 (CH_2), 27.2 (CH_2).
HRMS (GC-Q-TOF) m/z: $[M\text{-}Br]^+$ calcd for $C_{13}H_{21}$: 177.1643; found: 177.1644.

Catalytic reaction of (1r,4r)-1-(3-bromoprop-2-yn-1-yl)-4-(tert-butyl)cyclohexane (tBu trans-3e)

The catalytic reaction was performed using General Procedure S, starting from 0.2 mmol (51 mg) of bromoalkyne ***tBu trans-3e***, affording a mixture of starting material and bromocyclopentenes ***tBu trans-4e-cis*** *and* ***tBu trans-4e-trans*** in *27% yield as a mixture of diastereoisomers* [1.3(***tBu trans-4e-cis***):1(***tBu trans-4e-trans***)]*by ^{1}H NMR analysis of the crude reaction mixture using CH_2Br_2 as internal standard*).

2. Appendix

X-Ray data

6b (X-ray data)

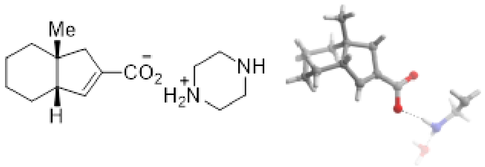

Crystal data	$C_{11}H_{13}O_2{\cdot}0.5(C_4H_{12}N_2){\cdot}H_2O$
Chemical formula	239.31
M_r	Monoclinic, *C*2/*c*
Crystal system, space group	297
Temperature (K)	35.9505 (18), 7.3421 (3), 11.7623 (6)
a, *b*, *c* (Å)	113.175 (6)
β (°)	2854.1 (2)
V (Å^3)	8
Z	Cu *Kα*
Radiation type	0.64
μ (mm^{-1})	0.43 × 0.22 × 0.21
Crystal size (mm)	
Data collection	Xcalibur, Onyx, Nova
Diffractometer	
Absorption correction	Multi-scan *CrysAlis PRO* 1.171.38.43 (Rigaku Oxford Diffraction, 2015) Empirical absorption correction using spherical harmonics, implemented in SCALE3 ABSPACK scaling algorithm.
T_{min}, T_{max}	0.832, 1.000
No. of measured, independent and observed [$I > 2\sigma(I)$] reflections	12361, 2668, 2403
R_{int}	0.024
(sin *θ*/*λ*)$_{max}$ (Å^{-1})	0.608
Refinement	
$R[F^2 > 2\sigma(F^2)]$, $wR(F^2)$, *S*	0.053, 0.172, 1.07
No. of reflections	2668
No. of parameters	280
No. of restraints	295
H-atom treatment	H atoms treated by a mixture of independent and constrained refinement
$\Delta\rho_{max}$, $\Delta\rho_{min}$ (e Å^{-3})	0.31, −0.17

Computer programs: *CrysAlis PRO* 1.171.38.43 (Rigaku OD, 2015), *SHELXT* (Sheldrick, 2015), *SHELXL2019*/2 (Sheldrick, 2019), *PLATON* (Spek, 2009), *enCIFer* (Allen,2004).

Table 2

Hydrogen-bond geometry (Å, °)

D—H···*A*	*D*—H	H···*A*	*D*···*A*	*D*—H···*A*
a_1_555">N41—H41···O1a	a_1_555">0.89	a_1_555">1.83	a_1_555">2.706 (10)	a_1_555">170
N41—H42···O51	0.89	1.81	2.6955 (16)	172
a_7_566">O51—H511···O1a[i]	a_7_566">0.95 (2)	a_7_566">1.80 (2)	a_7_566">2.744 (9)	a_7_566">176 (3)
a_4_555">O51—H512···O2a[ii]	a_4_555">0.93 (2)	a_4_555">1.76 (2)	a_4_555">2.678 (9)	a_4_555">169 (2)
b_7_566">O51—H511···O21b[i]	b_7_566">0.95 (2)	b_7_566">1.74 (3)	b_7_566">2.682 (18)	b_7_566">173 (3)
b_4_555">O51—H512···O22b[ii]	b_4_555">0.93 (2)	b_4_555">1.83 (3)	b_4_555">2.757 (18)	b_4_555">171 (3)

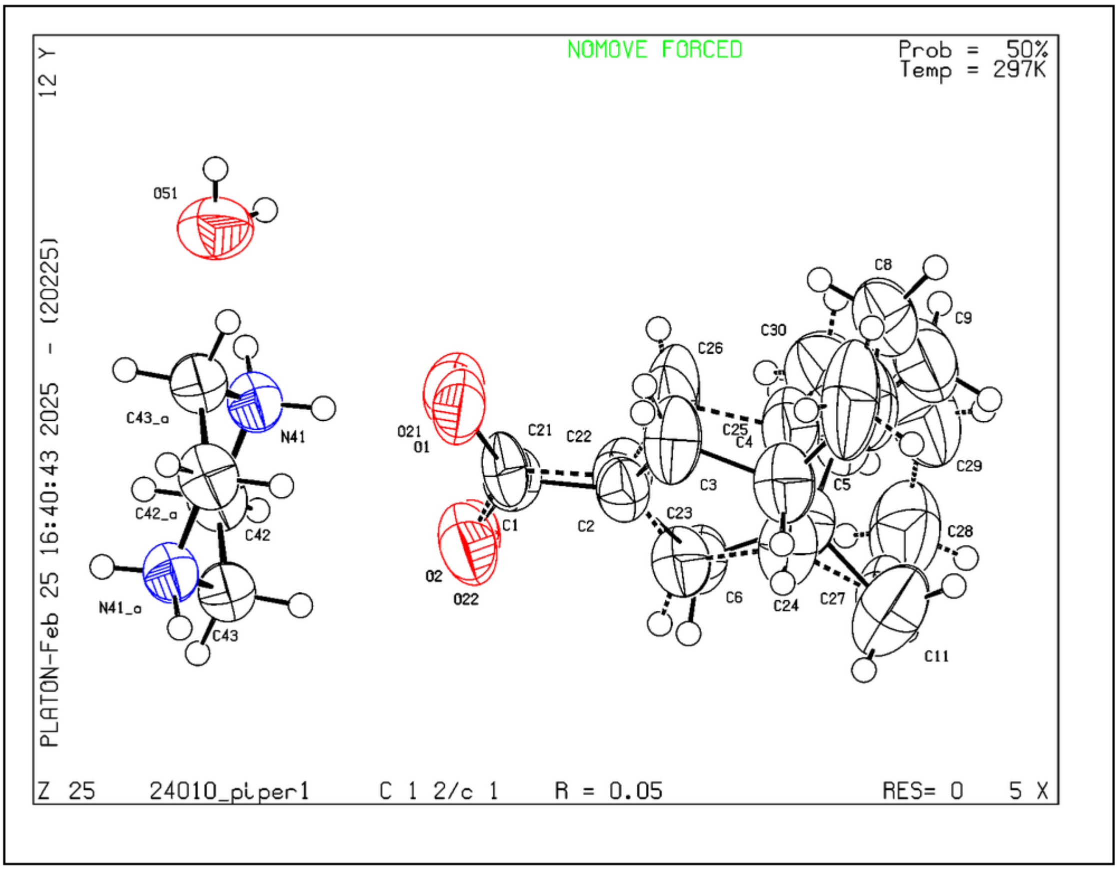

Figura S1. ORTEP diagram for **6b**.

3. References

[1] Call, A., Capocasa, A., Palone, A., Vicens, L., Aparicio, E., Choukairi Afailal, N., Siakavaras, N., López Saló, M. E., Bietti, M., Costas, M., *J. Am. Chem. Soc.*, **2023**, *145*, 32, 18094-18103.

[2] Lohse, C., Detterbeck, R., Acklin, P., Borschberg, H.J., *Helv. Chim. Acta*, **2002**, *85*, 3, 945-961.

[3] Das, J., Ali, W., Ghosh, A., Pal, T., Mandal, A., Teja, C., Dutta, S., Pothikumar, R., Ge, H., Zhang, X., Maiti, D., *Nature Chem.*, **2023**, *15*, 11, 1626–1635.

[4] Menichincheri, M., Bargiotti, A., Berthelsen, J., Bertrand, J. A., Bossi, R., Ciavolella, A., Cirla, A., Cristiani, C., Croci, V., D'Alessio, R., Fasolini, M., Fiorentini, F., Forte, B., Isacchi, A., Martina, K., Molinari, A., Montagnoli, A., Orsini, P., Orzi, F., Vanotti, E., et al., *J. Med. Chem.*, **2009**, *52*, 2, 293–307.

[5] Moebius, H.-J., WO 2004/037234 A2, 2004.

[6] Miguélez, R., Semleit, N., Rodríguez-Arias, C., Mykhailiuk, P., González, J. M., Haberhauer, G., Barrio, P., *Angew. Chem.*, **2023**, *135*, e202305296; Angew. Chem. Int. Ed., **2023**, *62*, e202305296.

[7] Dillner, D. K., *Org. Prep. Proced. Int.*, **2009**, *41*, 2, 147–152.

[8] Lv, N., Han, J.-C., Zhang, P., Huang, Y.-R., Xu, Z.-X., Xu, K., Wang, X.-F., Li, X., Chung, L. W., *Chem.*, **2024**, *10*, 1, 190–198.

[9] Trân, K., Van Den Hauwe, R., X. Sainsily, X., Couvineau, P., Côté, J., L. Simard, L., Echevarria, M., Murza, A., Serre, A., Théroux, L., Saibi, S., Haroune, L., Longpré, J. M., Lesur, O., Auger-Messier, M., Spino, C., Bouvier, M., Sarret, P., Ballet, S., Marsault, É., *J. Med. Chem.*, **2021**, *64*, 5345–5364.

[10] Tomioka, T., Takahashi, Y., Vaughan, T. G., Yanase, T., *Org Lett.*, **2010**, *12*,10, 2171-2173,

[11] Hansen, B. B.; Jepsen, T. H.; Larsen, M.; Sindet, R.; Vifian, T.; Burhardt, M. N.; Larsen, J.; Seitzberg, J. G.; Carnerup, M. A.; Jerre, A.; Molck, C.; Lovato, P.; Rai, S.; Nasipireddy, V. R.; Ritzén, A. *J. Med. Chem.* **2020**, *63*, 13, 7008-7032.

[12] Corey, E. J.; Boger, D. L., *Tetrahedron Lett.*, **1978**, *19*, 1, 9-12.

[13] Macbeth, D. H., Mills, A. K., Simmonds, J. A., *J. Chem. Soc.*, **1949**, 1011–1013.

[14] Smaligo, A. J., Swain, M., Quintana, J. C., Tan, M. F., Kim, D. A., Kwon, O., *Science*, **2019**, *364*, 681–685.

[15] Dauzonne, D., Goasdoue, N., Platzer, N., *Organic Magnetic Resonance* **1981**, *17*, 1, 18-25.

[16] DiLabio, G. A., Ingold, K. U., Roydhouse, M. D., *Org. Lett.*, **2004**, *6*, 4319–4322.

[17] Iwasaki, K., Wan, K. K., Oppedisano, A., Crossley, S. W. M., Shenvi, R. A., *J. Am. Chem. Soc.*, **2014**, *136*, 4, 1300–1303.

[18] Liu, M. T., Ho, J., Liu, J. K., Purakait, R., Morzan, U. N., Ahmed, L., Batista, V. S., Matsunami, H., Ryan, K., *Org. Biomol. Chem.*, **2018**, *16*, 2541–2548.

[19] Chen, C. M., Shiao, H. Y., Uang, B. J., Hsieh, H. P., *Angew. Chem. Int. Ed.*, **2018**, *57*, 47, 15572–15576.

[20] Chan, S. C., Palone, A., Bietti, M., Costas, M., *Angew. Chem. Int. Ed.*, **2024**, *63*, 28, e202402858.

[21] Shinkai, H., Nishikawa, M., Sato, Y., Toi, K., Kumashiro, I., Seto, Y., Fukuma, M, Dan, K., Toyoshima, S., *J. Med. Chem.*, **1989**, *32*, 7, 1436–1441.

[22] Rodríguez, M., Font, G., Nadal-Moradell, J., Hernán-Gómez, A., Costas M., *Adv. Synth. Catal.*, **2020**, *362*, 22, 5116–5123.
