# Supplementary material for: Conformational Effects in Intramolecular C(sp3)–H Bond Functionalization: Gold(I)-Catalyzed Cycloisomerization of Aliphatic 1‑Bromoalkynes as Benchmark Reaction
Source: Org Lett. 2025 Sep 22;27(39):11065–70. doi: 10.1021/acs.orglett.5c03430 (PMC12501934; doi:10.1021/acs.orglett.5c03430)
Supplement: Supplementary file 2 [file ol5c03430_si_002.pdf]

Supporting Information
Conformational Effects in Intramolecular $C(sp^3)$—H bond Func-tionalization: Gold(I)-Catalyzed Cycloisomerization of Aliphatic 1-Bromoalkynes as Benchmark Reaction

Rubén Miguélez,[a,‡] Omar Arto,[a‡] Hannah Siera,[b] Jan Schulte,[b] Isabel Merino,[c] Gebhard Haberhauer,[b] Pablo Barrio[a*]

[a] Department of Organic and Inorganic Chemistry
Universidad de Oviedo
Julian Clavería 8 33006 Oviedo (Spain)
[b] Institut für Organische Chemie
Universität Duisburg-Essen
Universitätstraße 7, 45117 Essen (Germany)
[c] Servicios Científico Técnicos
Universidad de Oviedo
Fernando Bonguera s/n, 30006 Oviedo (Spain)
Correspondence to: barriopablo@uniovi.es

This PDF file includes:

Figures S1-S14
Computational Details
Cartesian coordinates and Absolute Energies
References

1. **Figures** S3

2. **Computational Details** S8

3. **Cartesian Coordinates and Absolute Energies for All Calculated Compounds** S9

4. **References** S127

1. Figures

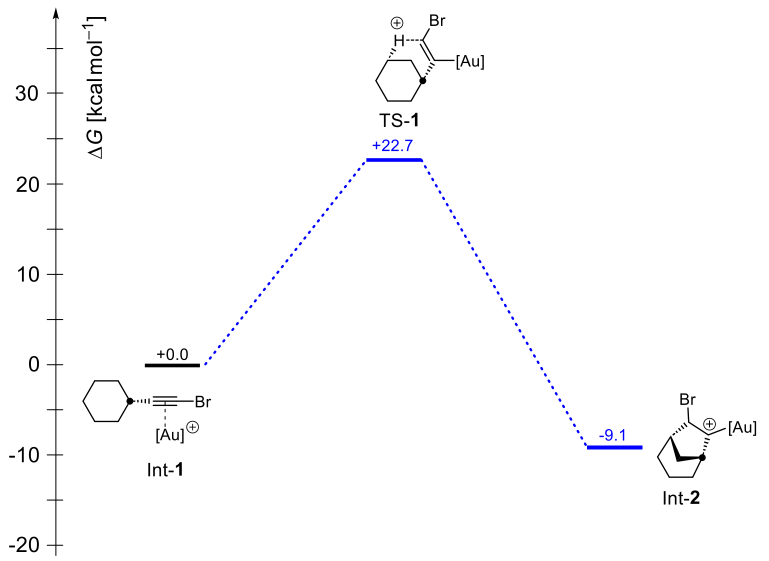

Figure S1. Free energy (Δ*G*) profile of the gold(I)-catalyzed reaction of alkyne **1a** calculated by means of PBE0-D3BJ(SMD)/6-311++G(d,p),def2-TZVP//PBE0-D3BJ/6-31G*,def2-TZVP. $[Au]^+$ = $IPrAu^+$.

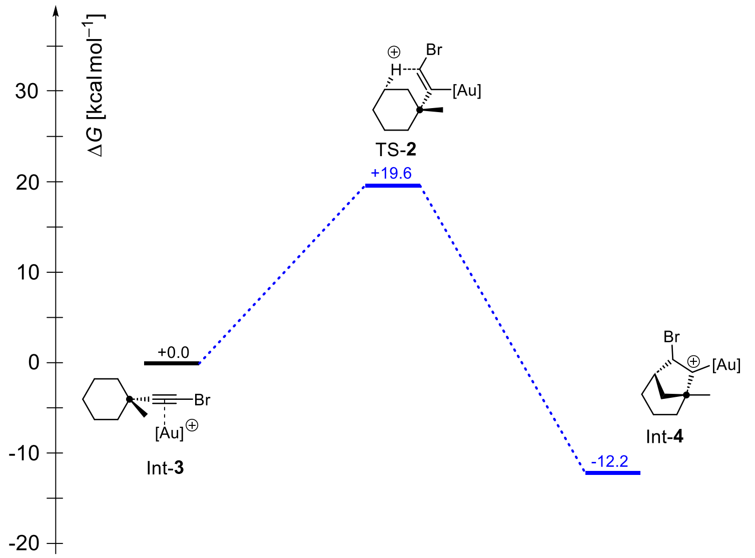

Figure S2. Free energy (Δ*G*) profile of the gold(I)-catalyzed reaction of alkyne **1b** calculated by means of PBE0-D3BJ(SMD)/6-311++G(d,p),def2-TZVP//PBE0-D3BJ/6-31G*,def2-TZVP. $[Au]^+$ = $IPrAu^+$.

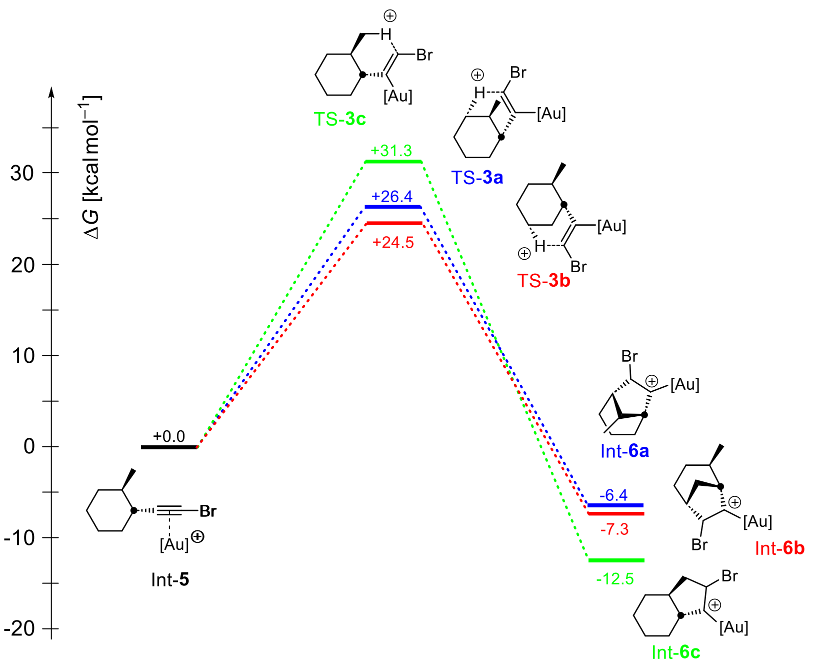

Figure S3. Free energy (Δ*G*) profile of the gold(I)-catalyzed reaction of alkyne *trans*-**1c** calculated by means of PBE0-D3BJ(SMD)/6-311++G(d,p),def2-TZVP//PBE0-D3BJ/6-31G*,def2-TZVP. $[Au]^+ = IPrAu^+$.

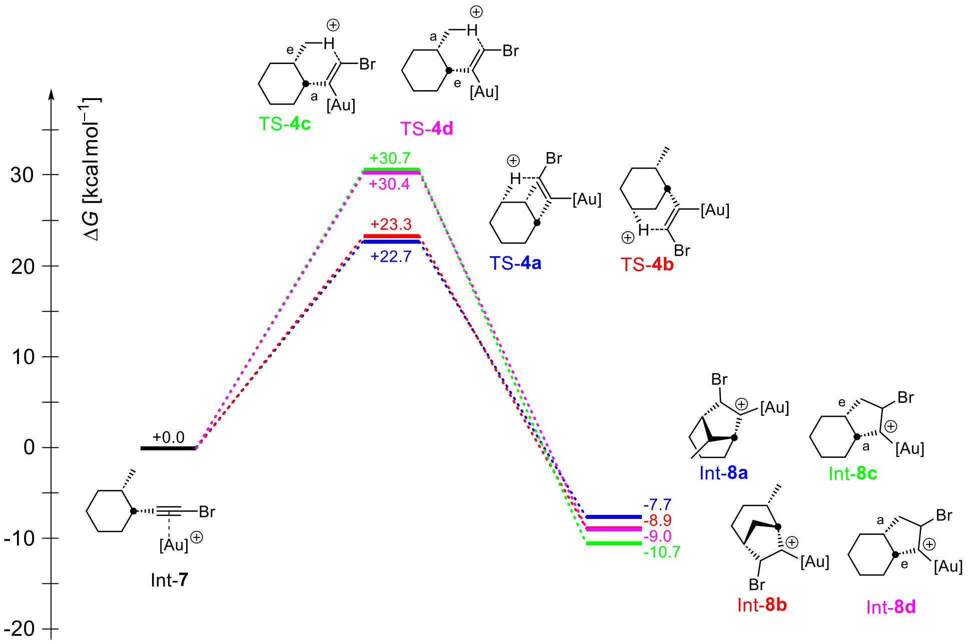

Figure S4. Free energy (Δ*G*) profile of the gold(I)-catalyzed reaction of alkyne *cis*-**1c** calculated by means of PBE0-D3BJ(SMD)/6-311++G(d,p),def2-TZVP//PBE0-D3BJ/6-31G*,def2-TZVP. $[Au]^+ = IPrAu^+$.

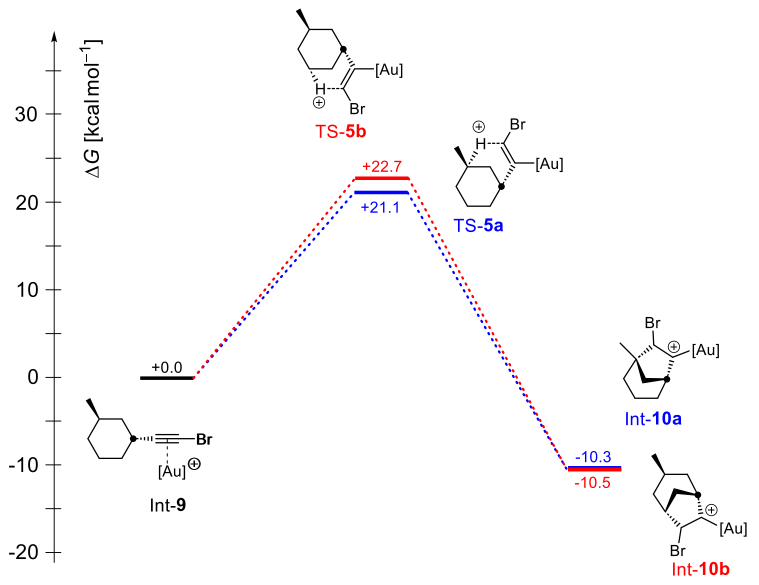

Figure S5. Free energy (Δ*G*) profile of the gold(I)-catalyzed reaction of alkyne *trans*-**1d** calculated by means of PBE0-D3BJ(SMD)/6-311++G(d,p),def2-TZVP//PBE0-D3BJ/6-31G*,def2-TZVP. $[Au]^+$ = $IPrAu^+$.

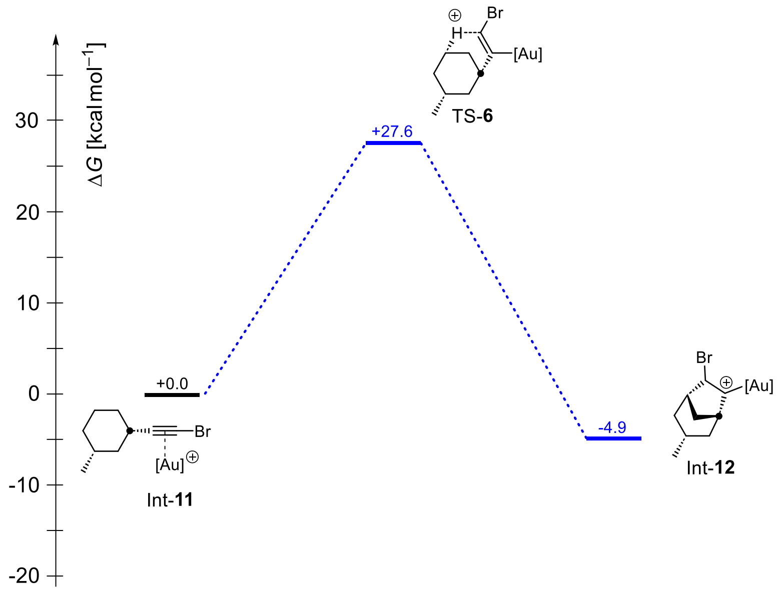

Figure S6. Free energy (Δ*G*) profile of the gold(I)-catalyzed reaction of alkyne *cis*-**1d** calculated by means of PBE0-D3BJ(SMD)/6-311++G(d,p),def2-TZVP//PBE0-D3BJ/6-31G*,def2-TZVP. $[Au]^+$ = $IPrAu^+$.

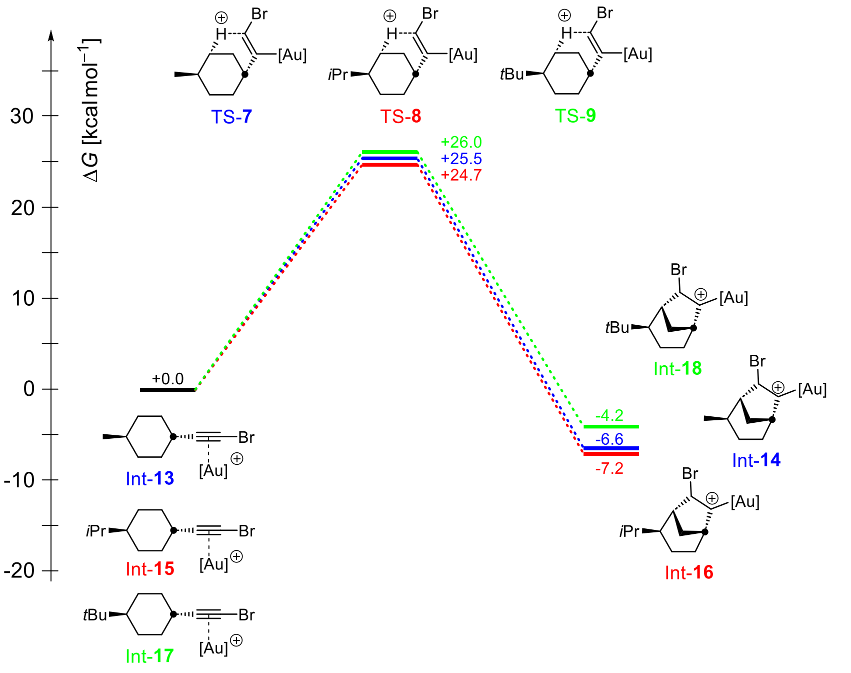

Figure S7. Free energy (Δ*G*) profile of the gold(I)-catalyzed reaction of alkynes *trans*-**1e** calculated by means of PBE0-D3BJ(SMD)/6-311++G(d,p),def2-TZVP//PBE0-D3BJ/6-31G*,def2-TZVP. $[Au]^+$ = $IPrAu^+$.

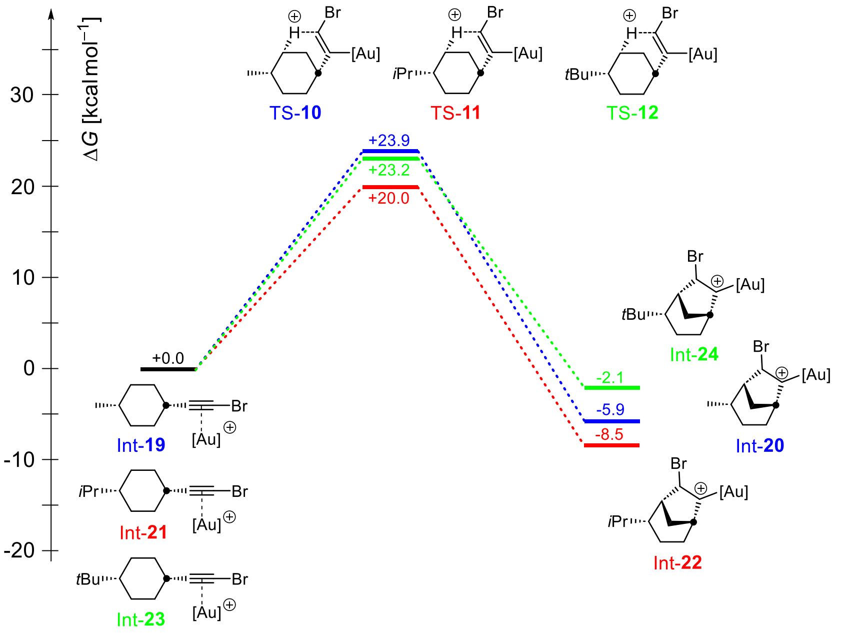

Figure S8. Free energy (Δ*G*) profile of the gold(I)-catalyzed reaction of alkynes *cis*-**1e** calculated by means of PBE0-D3BJ(SMD)/6-311++G(d,p),def2-TZVP//PBE0-D3BJ/6-31G*,def2-TZVP. $[Au]^+$ = $IPrAu^+$.

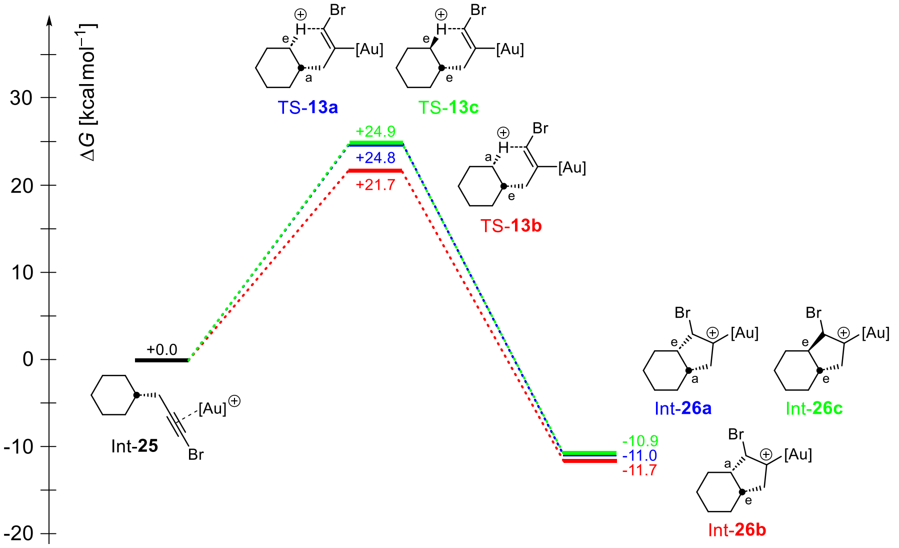

Figure S9. Free energy (Δ*G*) profile of the gold(I)-catalyzed reaction of alkyne **3a** calculated by means of PBE0-D3BJ(SMD)/6-311++G(d,p),def2-TZVP//PBE0-D3BJ/6-31G*,def2-TZVP. $[Au]^+$ = $IPrAu^+$.

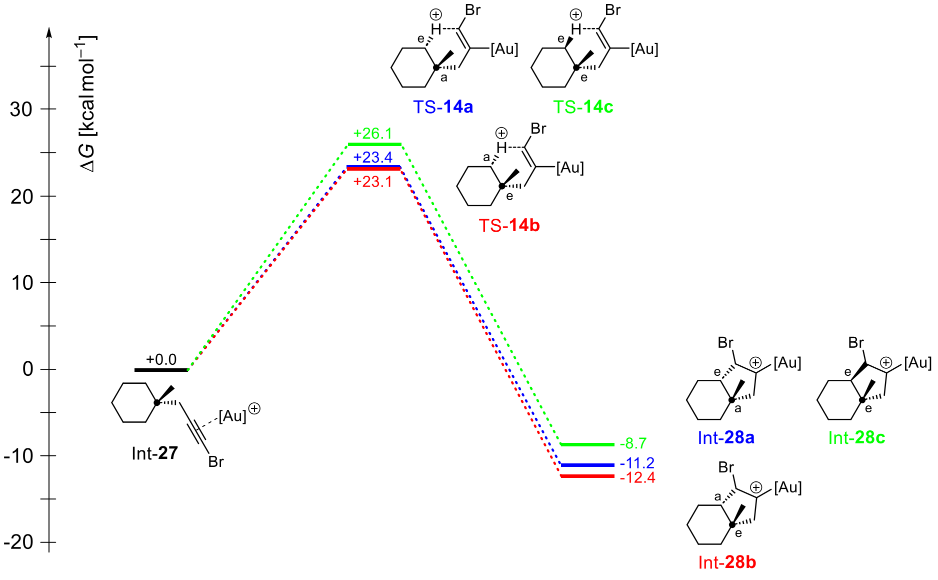

Figure S10. Free energy (Δ*G*) profile of the gold(I)-catalyzed reaction of alkyne **3b** calculated by means of PBE0-D3BJ(SMD)/6-311++G(d,p),def2-TZVP//PBE0-D3BJ/6-31G*,def2-TZVP. $[Au]^+$ = $IPrAu^+$.

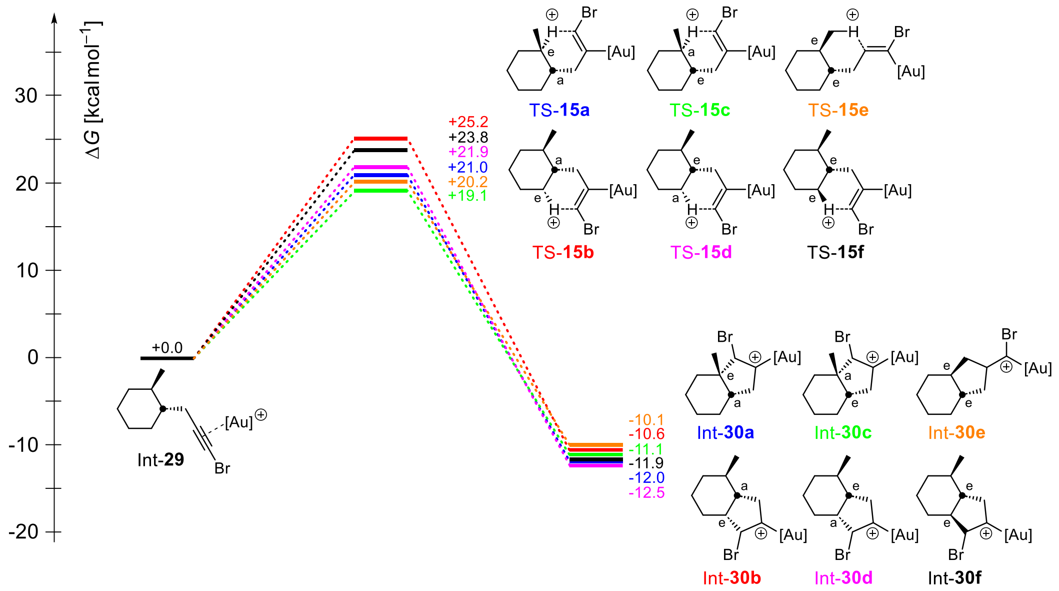

Figure S11. Free energy (ΔG) profile of the gold(I)-catalyzed reaction of alkyne *trans*-**3c** calculated by means of PBE0-D3BJ(SMD)/6-311++G(d,p),def2-TZVP//PBE0-D3BJ/6-31G*,def2-TZVP. $[Au]^+ = IPrAu^+$.

2. Computational Details

All calculations were performed by using the program package Gaussian 16[1]. The geometrical parameters of the stationary points for the gold(I)-catalyzed cyclization were optimized by means of the density functional PBE0[2] and the dispersion correction D3BJ[3]. For all structures C_1 symmetry was applied. For the optimization process two different types of basis sets were used: For the light atoms C, H, N and Br the 6-31G(d)[4-5] basis set was employed. For Au the def2-TZVP[6-7] basis set was used. Frequency calculations were carried out at each of the structures to verify the nature of the stationary point. It turned out that all transition states have exactly one imaginary frequency. All other structures have none.

Furthermore, the energies of the stationary points were calculated using the density functional PBE0, the additional dispersion correction D3BJ and the basis set 6-311++G(d,p)[8-11] for C, H, N and Br. For Au the def2-TZVP basis set was employed. To take solvent effects into account, the solvent model SMD[12] (dichloromethane as solvent) was used for the single point calculations.

3. Cartesian Coordinates and Absolute Energies for All Calculated Compounds

Table S1. Absolute energies [au] calculated by means of different methods.

Compound	*E*[a]	*G*[a]	*E*[b]
**1a**	-2882.122518	-2881.984488	-2884.957613
Int-**1**	-4176.523949	-4175.852485	-4179.703674
TS-**1**	-4176.479114	-4175.809828	-4179.665298
Int-**2**	-4176.542222	-4175.866771	-4179.722207
**1b**	-2921.392576	-2921.227895	-2924.237491
Int-**3**	-4215.795880	-4215.096553	-4218.985185
TS-**2**	-4215.753293	-4215.058376	-4218.949570
Int-**4**	-4215.817283	-4215.115153	-4219.007434
trans-**1c**	-2921.392437	-2921.227960	-2924.237706
Int-**5**	-4215.794310	-4215.096824	-4218.984390
TS-**3a**	-4215.746198	-4215.049321	-4218.941797
Int-**6a**	-4215.810132	-4215.107053	-4219.000191
TS-**3b**	-4215.747564	-4215.051431	-4218.944029
Int-**6b**	-4215.811300	-4215.108299	-4219.001567
TS-**3c**	-4215.736369	-4215.039142	-4218.934180
Int-**6c**	-4215.818153	-4215.116406	-4219.008542
cis-**1c**	-2921.392642	-2921.227840	-2924.237624
Int-**7**	-4215.794468	-4215.095790	-4218.984863
TS-**4a**	-4215.749824	-4215.054257	-4218.945593
Int-**8a**	-4215.811231	-4215.108566	-4219.001087
TS-**4b**	-4215.749094	-4215.051680	-4218.946453
Int-**8b**	-4215.812423	-4215.109229	-4219.003532
TS-**4c**	-4215.738648	-4215.040007	-4218.935915
Int-**8c**	-4215.815835	-4215.113076	-4219.005942
TS-**4d**	-4215.738402	-4215.040736	-4218.935392
Int-**8d**	-4215.811965	-4215.109002	-4219.003421
trans-**1d**	-2921.392174	-2921.227587	-2924.237569
Int-**9**	-4215.794390	-4215.095823	-4218.984673
TS-**5a**	-4215.758217	-4215.062033	-4218.948642
Int-**10a**	-4215.815474	-4215.113132	-4219.004901
TS-**5b**	-4215.749517	-4215.053535	-4218.945847
Int-**10b**	-4215.813494	-4215.112466	-4219.003921
cis-**1d**	-2921.392030	-2921.227631	-2924.237868
Int-**11**	-4215.793793	-4215.096236	-4218.984129
TS-**6**	-4215.743285	-4215.045947	-4218.939997
Int-**12**	-4215.806284	-4215.103553	-4218.997065
^{Me}*trans*-**1e**	-2921.392091	-2921.227665	-2924.237873
Int-**13**	-4215.793821	-4215.096383	-4218.984358
TS-**7**	-4215.747452	-4215.050747	-4218.943031
Int-**14**	-4215.810069	-4215.107278	-4219.000148
^{iPr}*trans*-**1e**	-2999.924840	-2999.707107	-3002.792209
Int-**15**	-4294.327323	-4293.575976	-4297.538932
TS-**8**	-4294.282624	-4293.532253	-4297.498552
Int-**16**	-4294.344672	-4293.587861	-4297.555890
^{tBu}*trans*-**1e**	-3039.191993	-3038.947584	-3042.070222
Int-**17**	-4333.594761	-4332.772192	-4336.817156
TS-**9**	-4333.549188	-4332.824402	-4336.774840
Int-**18**	-4333.608441	-2881.984488	-4336.829991

[a] PBE0-D3BJ/6-31G*,def2-TZVP
[b] PBE0-D3BJ(SMD)/6-311++G(d,p),def2-TZVP//PBE0-D3BJ/6-31G*,def2-TZVP

Table S2. Absolute energies [au] calculated by means of different methods.

Compound	*E*[a]	*G*[a]	*E*[b]
^{Me}*cis*-**1e**	-2921.392241	-2921.227656	-2924.237640
Int-**19**	-4215.793993	-4215.096753	-4218.984516
TS-**10**	-4215.750442	-4215.054028	-4218.945599
Int-**20**	-4215.809292	-4215.106788	-4218.999103
^{iPr}*cis*-**1e**	-2999.925039	-2999.707140	-3002.791981
Int-**21**	-4294.328150	-4293.574091	-4297.538327
TS-**11**	-4294.285747	-4293.536874	-4297.501312
Int-**22**	-4294.344378	-4293.587352	-4297.554873
^{tBu}*cis*-**1e**	-3039.192396	-3038.947792	-3042.070136
Int-**23**	-4333.596459	-4332.816633	-4336.818128
TS-**12**	-4333.553191	-4332.775175	-4336.779366
Int-**24**	-4333.606344	-4332.821190	-4336.826801

[a] PBE0-D3BJ/6-31G*,def2-TZVP

[b] PBE0-D3BJ(SMD)/6-311++G(d,p),def2-TZVP//PBE0-D3BJ/6-31G*,def2-TZVP

Table S3. Absolute energies [au] calculated by means of different methods.

Compound	*E*[a]	*G*[a]	*E*[b]
**3a**	-2921.390604	-2921.225872	-2924.236788
Int-**25**	-4215.793479	-4215.094047	-4218.983899
TS-**13a**	-4215.745253	-4215.048426	-4218.941762
Int-**26a**	-4215.809582	-4215.109092	-4219.002422
TS-**13b**	-4215.748440	-4215.053743	-4218.944538
Int-**26b**	-4215.809854	-4215.109914	-4219.003075
TS-**13c**	-4215.745528	-4215.048806	-4218.941537
Int-**26c**	-4215.808928	-4215.108911	-4219.001924
**3b**	-2960.659128	-2960.467192	-2963.515781
Int-**27**	-4255.058948	-4254.334494	-4258.261412
TS-**14a**	-4255.016418	-4254.293418	-4258.222620
Int-**28a**	-4255.078614	-4254.351902	-4258.281527
TS-**14b**	-4255.018424	-4254.294502	-4258.224006
Int-**28b**	-4255.080566	-4254.353520	-4258.283792
TS-**14c**	-4255.013232	-4254.289489	-4258.219162
Int-**28c**	-4255.074860	-4254.347764	-4258.277973
trans-**3c**	-2960.656832	-2960.465574	-2963.513592
Int-**29**	-4255.062852	-4254.336409	-4258.263258
TS-**15a**	-4255.025446	-4254.302729	-4258.226139
Int-**30a**	-4255.081536	-4254.353752	-4258.283749
TS-**15b**	-4255.013478	-4254.290020	-4258.220130
Int-**30b**	-4255.077388	-4254.350496	-4258.280661
TS-**15c**	-4255.026990	-4254.305010	-4258.228321
Int-**30c**	-4255.080412	-4254.352402	-4258.282553
TS-**15d**	-4255.017782	-4254.295536	-4258.224233
Int-**30d**	-4255.079530	-4254.353149	-4258.283042
TS-**15e**	-4255.022557	-4254.297428	-4258.229780
Int-**30e**	-4255.085152	-4254.354076	-4258.283903
TS-**15f**	-4255.015945	-4254.292735	-4258.222093
Int-**30f**	-4255.079085	-4254.352325	-4258.282489

[a] PBE0-D3BJ/6-31G*,def2-TZVP

[b] PBE0-D3BJ(SMD)/6-311++G(d,p),def2-TZVP//PBE0-D3BJ/6-31G*,def2-TZVP

Cartesian coordinates of the optimized geometry for **1a** at PBE0-D3BJ/6-31G*,def2-TZVP level of theory: (number of imaginary frequencies = 0):

C	1.18006600	0.00007300	0.50869200
C	1.81852000	-1.26446300	-0.09118900
C	1.81862800	1.26451400	-0.09123700
H	1.39022900	0.00008400	1.59050300
C	3.33208000	-1.25939000	0.10433100
H	1.57998900	-1.29904100	-1.16300900
H	1.36884300	-2.15456100	0.36298800
C	3.33218700	1.25930400	0.10430600
H	1.58012400	1.29906300	-1.16306400
H	1.36902800	2.15467500	0.36289100
C	3.96695500	-0.00007700	-0.48081200
H	3.76882900	-2.15620000	-0.35144200
H	3.55792200	-1.31304500	1.17960200
H	3.76901500	2.15606800	-0.35147900
H	3.55802400	1.31295800	1.17957700
H	5.04889700	-0.00012400	-0.30120100
H	3.82855200	-0.00008000	-1.57189100
C	-0.26718500	0.00011100	0.33133500
C	-1.46381600	0.00005700	0.16166700
Br	-3.23211600	-0.00001600	-0.08731500

Cartesian coordinates of the optimized geometry for **1b** at PBE0-D3BJ/6-31G*,def2-TZVP level of theory: (number of imaginary frequencies = 0):

C	1.35649200	0.95769000	0.00002700
C	2.05036600	0.38759600	-1.25553600
C	2.05004100	0.38741700	1.25568500
C	2.09163300	-1.13699500	-1.25988500
H	3.07595100	0.78466900	-1.27023500
H	1.54588400	0.76631900	-2.15259700
C	2.09132200	-1.13717900	1.25981600
H	3.07562400	0.78448100	1.27070700
H	1.54531300	0.76601000	2.15265800
C	2.76534200	-1.67586800	0.00001300
H	2.61615200	-1.49058400	-2.15585200
H	1.06499900	-1.52281900	-1.31995300
H	2.61564700	-1.49086500	2.15586300
H	1.06468600	-1.52304700	1.31961100
C	-0.05530700	0.56310600	-0.00012000
C	-1.22130000	0.24253800	-0.00017000
Br	-2.94406700	-0.22859800	-0.00001700
H	3.82393200	-1.37535500	0.00016400
H	2.75061900	-2.77255400	-0.00006900
C	1.44561500	2.48829200	0.00013600
H	0.96073800	2.90834100	-0.88715500
H	0.96050800	2.90824100	0.88735100
H	2.49706800	2.79853100	0.00029200

Cartesian coordinates of the optimized geometry for *trans*-**1c** at PBE0-D3BJ/6-31G*,def2-TZVP level of theory: (number of imaginary frequencies = 0):

C	-1.08575800	-0.17303100	0.47729700
C	-1.77792200	0.99368000	-0.26257600
C	-1.64878700	-1.53346100	0.02966300
C	-3.29304100	0.89479600	-0.07612900
H	-1.55265900	0.87236200	-1.33320400
C	-3.16010200	-1.59798700	0.21824400
H	-1.39793000	-1.68111400	-1.02958600
H	-1.15018100	-2.33443500	0.58695400
C	-3.85354000	-0.45419500	-0.51496200
H	-3.77800900	1.70918500	-0.62944200
H	-3.52821900	1.05944900	0.98694200

H	-3.53841500	-2.56621400	-0.13129900
H	-3.39514300	-1.53759900	1.29110700
C	0.36291000	-0.13899800	0.30776500
C	1.56042900	-0.12649700	0.14318300
Br	3.32973400	-0.10756100	-0.09760200
H	-4.93622400	-0.48916600	-0.34302300
H	-3.70277800	-0.57465100	-1.59761700
H	-1.30493600	-0.05217200	1.55151900
C	-1.25253000	2.34737000	0.19606000
H	-1.44991400	2.49805500	1.26520500
H	-0.17277000	2.43083600	0.03824700
H	-1.74347200	3.16003800	-0.35100800

Cartesian coordinates of the optimized geometry for *cis*-**1c** at PBE0-D3BJ/6-31G*,def2-TZVP level of theory: (number of imaginary frequencies = 0):

C	-1.29228300	0.20751900	1.12195800
C	-1.92410900	-1.19281800	1.23164400
C	-2.11778400	1.09528000	0.16103200
C	-2.05755700	-1.85547700	-0.13543200
H	-2.91831800	-1.07680000	1.68546900
H	-1.32920500	-1.81484500	1.90976300
C	-2.24960600	0.42150000	-1.20483300
C	-2.85347300	-0.97707300	-1.09783200
H	-2.53435500	-2.83732300	-0.02913800
H	-1.05400100	-2.03216800	-0.54575900
H	-2.86069200	1.05153700	-1.86381800
H	-1.25237400	0.35928300	-1.66385600
C	0.10465800	0.11415000	0.70126900
C	1.25559800	0.02386500	0.34129400
Br	2.95677100	-0.10796700	-0.18493800
H	-3.89034000	-0.89561300	-0.73891800
H	-2.90148700	-1.44526900	-2.08851300
H	-1.30893300	0.67847700	2.11593700
C	-1.54990100	2.50299500	0.05119100
H	-1.47837400	2.98334000	1.03420000
H	-0.54504000	2.48271500	-0.38554000
H	-2.18431800	3.12949600	-0.58576900
H	-3.12282000	1.15636700	0.60700300

Cartesian coordinates of the optimized geometry for *trans*-**1d** at PBE0-D3BJ/6-31G*,def2-TZVP level of theory: (number of imaginary frequencies = 0):

C	-1.01670600	-0.88081700	1.15087900
C	-1.62342500	-1.94016200	0.21245600
C	-1.93721200	0.35135800	1.21869900
C	-1.90462500	-1.36326100	-1.17083500
H	-2.56223200	-2.28891300	0.66507100
H	-0.95404300	-2.80501900	0.14592500
C	-2.22274900	0.94223400	-0.16170500
H	-1.49365200	1.11075300	1.87373000
C	-2.80277200	-0.13155400	-1.08471400
H	-2.36616100	-2.12802700	-1.80706300
H	-0.95203400	-1.08970800	-1.64455400
H	-1.26177100	1.26978000	-0.58657800
C	0.32983000	-0.50749300	0.72092700
C	1.43750000	-0.19376500	0.35146100
Br	3.07517800	0.26817800	-0.19011000
H	-3.79257700	-0.42774700	-0.70271700
H	-2.96836700	0.28896300	-2.08504200
H	-0.93690800	-1.31122800	2.15907600
H	-2.88545100	0.03981400	1.68132900
C	-3.14351800	2.15119700	-0.06087100
H	-3.32899100	2.59110000	-1.04757300

H	-4.11471400	1.86801200	0.36519200
H	-2.71227300	2.92957600	0.57928600

Cartesian coordinates of the optimized geometry for *cis*-**1d** at PBE0-D3BJ/6-31G*,def2-TZVP level of theory: (number of imaginary frequencies = 0):

C	0.82579700	0.36280500	0.53940200
C	1.60327400	-0.88141800	0.07989400
C	1.34191100	1.61883800	-0.18140500
C	3.10929200	-0.72034600	0.28722100
H	1.39657200	-1.05062700	-0.98674300
C	2.84360100	1.78496400	0.02728400
H	1.12150000	1.52038200	-1.25307800
H	0.79677600	2.49960100	0.17605700
C	3.60956000	0.54189000	-0.41664200
H	3.20016000	2.66766700	-0.51712200
H	3.04266500	1.97334500	1.09249900
C	-0.60936600	0.19064100	0.34795600
C	-1.79540400	0.04599300	0.16668300
Br	-3.54799600	-0.16830600	-0.09950000
H	4.68408500	0.66988900	-0.23278100
H	3.49132400	0.41001700	-1.50320300
H	1.01089400	0.49518100	1.61752600
H	1.23570800	-1.76442500	0.61638800
C	3.86205900	-1.95954700	-0.18015400
H	4.93963500	-1.85794000	-0.00730300
H	3.51876900	-2.85784400	0.34571600
H	3.71046700	-2.12333100	-1.25453200
H	3.28696100	-0.59412400	1.36765200

Cartesian coordinates of the optimized geometry for *Me* *trans*-**1e** at PBE0-D3BJ/6-31G*,def2-TZVP level of theory: (number of imaginary frequencies = 0):

C	0.72635900	-0.00001200	0.53630300
C	1.37298900	-1.26218800	-0.05745100
C	1.37294500	1.26215800	-0.05744000
H	0.92496900	-0.00001400	1.62023000
C	2.88370000	-1.25268800	0.15188300
H	1.14329200	-1.29871200	-1.13100500
H	0.92316500	-2.15391800	0.39332600
C	2.88366300	1.25269100	0.15191900
H	1.14329200	1.29869900	-1.13100700
H	0.92312000	2.15390600	0.39330200
C	3.54189700	0.00002100	-0.42837300
H	3.32754800	-2.15110900	-0.29593100
H	3.10347000	-1.30136900	1.22975700
H	3.32751300	2.15114300	-0.29583200
H	3.10339200	1.30131900	1.22980500
H	3.35882700	0.00003700	-1.51503600
C	-0.71869900	-0.00009500	0.34280300
C	-1.91348700	-0.00003400	0.16076800
Br	-3.67901300	0.00001600	-0.10711800
C	5.04674400	0.00003900	-0.19404000
H	5.51888800	-0.88631900	-0.63336700
H	5.51887700	0.88639900	-0.63337500
H	5.27244200	0.00004400	0.88002800

Cartesian coordinates of the optimized geometry for *iPr* *trans*-**1e** at PBE0-D3BJ/6-31G*,def2-TZVP level of theory: (number of imaginary frequencies = 0):

C	-0.10189000	0.00018700	0.64385500
C	0.57500000	-1.25479800	0.07570800
C	0.57506700	1.25514800	0.07577800

H	0.04804200	0.00015100	1.73548600
C	2.07453700	-1.25047200	0.35458500
H	0.39648900	-1.28513600	-1.00780800
H	0.10897500	-2.15212900	0.49824600
C	2.07460900	1.25075200	0.35464800
H	0.39658000	1.28554900	-1.00773800
H	0.10909300	2.15249900	0.49834000
C	2.77807600	0.00012800	-0.18415300
H	2.51367700	-2.15490200	-0.07933600
H	2.24365900	-1.31103500	1.44122600
H	2.51377600	2.15517000	-0.07926400
H	2.24375100	1.31127500	1.44128800
H	2.66807900	0.00015700	-1.28260800
C	-1.53658200	0.00018300	0.38451000
C	-2.72199100	0.00009400	0.14908300
Br	-4.47359200	-0.00009400	-0.19826600
C	4.28702200	-0.00002600	0.12750900
H	4.39419900	0.00018500	1.22429300
C	4.98792200	1.24611800	-0.41453100
H	4.62951400	2.16776800	0.05322800
H	6.06760000	1.18831100	-0.23651800
H	4.83608000	1.33658100	-1.49815900
C	4.98739500	-1.24669800	-0.41398400
H	6.06703900	-1.18953000	-0.23555800
H	4.62823100	-2.16804000	0.05381000
H	4.83592200	-1.33729800	-1.49765300

Cartesian coordinates of the optimized geometry for ^{tBu}*trans*-**1e** at PBE0-D3BJ/6-31G*,def2-TZVP level of theory: (number of imaginary frequencies = 0):

C	0.38511800	-0.00010800	0.61272300
C	-0.28336600	1.25668600	0.03763800
C	-0.28331900	-1.25694900	0.03771600
H	0.22025700	-0.00004700	1.70223900
C	-1.78819100	1.24700100	0.29039000
H	-0.08564300	1.29346200	-1.04230300
H	0.17391200	2.15187200	0.47415000
C	-1.78823500	-1.24751600	0.29008800
H	-0.08544100	-1.29379100	-1.04219900
H	0.17396100	-2.15209300	0.47431500
C	-2.46426600	-0.00016600	-0.29180400
H	-2.22157500	2.15800500	-0.13544700
H	-1.96761100	1.29035500	1.37436400
H	-2.22122700	-2.15832900	-0.13651200
H	-1.96810500	-1.29157900	1.37397100
C	1.82322800	-0.00013900	0.37338000
C	3.01173300	-0.00004700	0.15403600
Br	4.76796200	0.00008100	-0.16941900
H	-2.25534800	-0.00011700	-1.37565100
C	-4.01336500	0.00009400	-0.16248300
C	-4.47428700	0.00252500	1.29837200
H	-4.12393700	0.89098000	1.83525100
H	-5.56975300	0.00120500	1.34730300
H	-4.12163000	-0.88282400	1.83888800
C	-4.59081200	-1.23799400	-0.85936000
H	-5.68546700	-1.18273800	-0.88593500
H	-4.23495800	-1.31152400	-1.89473900
H	-4.32555000	-2.16628600	-0.34317200
C	-4.59031700	1.23614200	-0.86335700
H	-4.23647400	1.30467800	-1.89975700
H	-5.68511400	1.18255500	-0.88743600
H	-4.32248100	2.16620600	-0.35170200

Cartesian coordinates of the optimized geometry for ^{Me}*cis*-**1e** at PBE0-D3BJ/6-31G*,def2-TZVP level of theory: (number of imaginary frequencies = 0):

C	-0.91315300	-1.62439900	-0.00005600
C	-1.71587700	-1.24984600	-1.25953200
C	-1.71598700	-1.25001400	1.25938400
C	-2.11090500	0.22303300	-1.25511700
H	-2.61921600	-1.87540300	-1.27842900
H	-1.13536100	-1.49282100	-2.15648500
C	-2.11103100	0.22286500	1.25512300
H	-2.61931700	-1.87558700	1.27810700
H	-1.13555600	-1.49309700	2.15636400
C	-2.89885600	0.60444600	-0.00001100
H	-2.70073300	0.45363200	-2.15156000
H	-1.20214000	0.83993200	-1.30821200
H	-2.70095000	0.45334300	2.15153700
H	-1.20227400	0.83976000	1.30839400
C	0.39633300	-0.97474600	0.00004900
C	1.47101600	-0.42076100	0.00003900
Br	3.06049200	0.39301500	0.00001500
H	-0.74779600	-2.71094700	-0.00012700
C	-3.26243800	2.08346000	0.00007100
H	-3.84952900	2.35023900	-0.88646000
H	-3.84962400	2.35011600	0.88657600
H	-2.35740900	2.70398500	0.00016300
H	-3.83193500	0.01708900	-0.00009900

Cartesian coordinates of the optimized geometry for ^{iPr}*cis*-**1e** at PBE0-D3BJ/6-31G*,def2-TZVP level of theory: (number of imaginary frequencies = 0):

C	0.07445100	2.01525300	0.00002000
C	-0.79473200	1.81616600	1.25212900
C	-0.79482700	1.81627300	-1.25203600
C	-1.48694800	0.45729700	1.25345000
H	-1.54928400	2.61508400	1.26477200
H	-0.18450300	1.93421200	2.15460100
C	-1.48705400	0.45741300	-1.25342900
H	-1.54937500	2.61519700	-1.26454500
H	-0.18466500	1.93440200	-2.15454300
C	-2.33897300	0.22333900	0.00003700
H	-2.10339700	0.37945600	2.15512200
H	-0.72578600	-0.33390600	1.32055900
H	-2.10359800	0.37967100	-2.15504600
H	-0.72590800	-0.33379300	-1.32069400
C	1.21652700	1.10320600	-0.00007100
C	2.14891100	0.33347500	-0.00005600
Br	3.52912900	-0.79959800	-0.00000200
H	0.46473100	3.04268300	0.00004800
C	-2.99941700	-1.16892900	0.00000900
H	-2.18764100	-1.91386400	0.00004900
H	-3.15028400	0.97285400	0.00010000
C	-3.85349700	-1.40248300	-1.24639200
H	-3.26320600	-1.39825500	-2.16737800
H	-4.36160400	-2.37171200	-1.18876700
H	-4.62728700	-0.62872200	-1.33777400
C	-3.85362600	-1.40246800	1.24632300
H	-4.62746500	-0.62873900	1.33757600
H	-4.36168000	-2.37172500	1.18869400
H	-3.26345200	-1.39816500	2.16738500

Cartesian coordinates of the optimized geometry for ^{tBu}*cis*-**1e** at PBE0-D3BJ/6-31G*,def2-TZVP level of theory: (number of imaginary frequencies = 0):

C	0.31571300	2.12405500	0.00004000
C	-0.55777000	1.95054900	1.25341000

C	-0.55776700	1.95061900	-1.25333500
C	-1.29131900	0.61287100	1.25062200
H	-1.28685500	2.77269700	1.26997800
H	0.05839500	2.04645900	2.15449900
C	-1.29138800	0.61297100	-1.25067700
H	-1.28684100	2.77278000	-1.26985700
H	0.05840000	2.04657900	-2.15441800
C	-2.16198300	0.43472500	-0.00000900
H	-1.90009800	0.54415000	2.15820500
H	-0.54842900	-0.19415600	1.30335100
H	-1.90025600	0.54448100	-2.15821500
H	-0.54857800	-0.19411600	-1.30361800
C	1.42778600	1.17559300	0.00000500
C	2.33199600	0.37293100	-0.00000900
Br	3.66979200	-0.80979700	-0.00001000
H	0.73799000	3.13873700	0.00006500
C	-3.00177600	-0.87357700	0.00000300
H	-2.89540300	1.26039800	0.00001900
C	-3.90701000	-0.90773700	-1.23743700
H	-3.33599400	-1.00925500	-2.16593000
H	-4.59272800	-1.76152500	-1.18288300
H	-4.51379000	0.00380900	-1.30957500
C	-3.90796600	-0.90704600	1.23676200
H	-4.51522500	0.00428100	1.30758900
H	-4.59324200	-1.76120400	1.18244700
H	-3.33765100	-1.00744800	2.16580700
C	-2.12125700	-2.12719200	0.00068200
H	-1.47835500	-2.17466200	-0.88495700
H	-1.47960900	-2.17458300	0.88723000
H	-2.74799700	-3.02709100	0.00028100

Cartesian coordinates of the optimized geometry for **3a** at PBE0-D3BJ/6-31G*,def2-TZVP level of theory: (number of imaginary frequencies = 0):

C	0.83355100	0.90946900	0.23537500
C	1.93414900	0.43040500	0.09549100
Br	3.56137900	-0.27541000	-0.11025800
C	-0.50362700	1.46585600	0.38745200
H	-0.69835200	1.65599700	1.45317300
H	-0.54645300	2.44391600	-0.11174800
C	-1.61202900	0.55969000	-0.16969400
C	-1.64810500	-0.79529700	0.53655500
C	-2.96941600	1.25546700	-0.06493400
H	-1.39646600	0.38373700	-1.23465200
C	-2.77164500	-1.67896400	0.00057600
H	-1.80031200	-0.62655600	1.61426200
H	-0.67784400	-1.29493400	0.43083300
C	-4.09743100	0.37587700	-0.59961600
H	-3.16460000	1.49383100	0.99227600
H	-2.94421200	2.21279500	-0.60253800
C	-4.12502000	-0.97947000	0.10325100
H	-2.79152200	-2.63280400	0.54174400
H	-2.56853500	-1.92099500	-1.05298900
H	-5.06096700	0.88746900	-0.48496300
H	-3.95139100	0.22031300	-1.67857700
H	-4.91609600	-1.61136800	-0.31889000
H	-4.37406500	-0.83023100	1.16434600

Cartesian coordinates of the optimized geometry for **3b** at PBE0-D3BJ/6-31G*,def2-TZVP level of theory: (number of imaginary frequencies = 0):

C	-0.70077000	0.32737800	-0.69374600
C	-1.85153000	0.09971200	-0.40235300
Br	-3.55206500	-0.23640800	0.02548000

C	1.60729700	0.78578100	0.20783300
C	3.04998000	1.01113700	-0.27506100
C	1.56525500	-0.44980600	1.12055500
C	3.69906000	-0.22583000	-0.89445500
H	3.64821000	1.32198400	0.59399300
H	3.07393000	1.85237400	-0.98190200
C	2.19333300	-1.69266600	0.49450600
H	2.11196300	-0.20493000	2.04309900
H	0.52711800	-0.64870300	1.41434900
C	3.62931300	-1.42118000	0.05285200
H	4.74208400	-0.00198700	-1.15018300
H	3.20434500	-0.48200900	-1.84184400
H	2.16668500	-2.51977300	1.21437700
H	1.59692400	-2.02182300	-0.36812400
H	4.06149600	-2.31059700	-0.42189900
H	4.24213200	-1.20960600	0.94174900
C	0.69105700	0.59421100	-1.02632400
H	1.06277200	-0.22471200	-1.65389300
H	0.74687900	1.50120000	-1.64499300
C	1.13307400	2.01189600	0.98468800
H	1.76489400	2.17873300	1.86479900
H	1.17701800	2.91454200	0.36239300
H	0.09942400	1.88576900	1.32529600

Cartesian coordinates of the optimized geometry for *trans*-**3c** at PBE0-D3BJ/6-31G*,def2-TZVP level of theory: (number of imaginary frequencies = 0):

C	0.94522600	0.76807200	0.51249500
C	2.06195000	0.42842400	0.19989200
Br	3.70789500	-0.08691800	-0.26146200
C	-0.40216400	1.19812400	0.85924300
H	-0.58753100	1.00976800	1.92687200
H	-0.45000000	2.28911200	0.73481900
C	-1.53100100	0.55516800	0.02644900
C	-1.92441500	-0.84711600	0.52180600
C	-2.74841600	1.48557300	0.03279000
C	-3.09226600	-1.39208400	-0.30681400
H	-2.28149700	-0.72926800	1.55947200
C	-3.90946400	0.93094300	-0.78485400
H	-3.07359300	1.63618500	1.07414300
H	-2.45652700	2.47323300	-0.34799200
C	-4.29977000	-0.46184400	-0.30346800
H	-3.37293600	-2.38306100	0.07284200
H	-2.74894200	-1.54289900	-1.34197300
H	-4.76696000	1.61315100	-0.73417800
H	-3.61138900	0.87818000	-1.84218900
H	-5.10156600	-0.87406400	-0.92836400
H	-4.70111900	-0.39293700	0.71831500
H	-1.17549700	0.46266500	-1.01125800
C	-0.77022600	-1.84404100	0.52656100
H	-0.33566200	-1.94400500	-0.47556300
H	0.03589500	-1.54790800	1.20336700
H	-1.12570900	-2.83333200	0.83825100

Cartesian coordinates of the optimized geometry for Int-**1** at PBE0-D3BJ/6-31G*,def2-TZVP level of theory: (number of imaginary frequencies = 0):

Au	0.02609600	0.60128000	-0.03462500
C	-1.25751200	2.39164200	-0.20027500
C	0.66347100	-1.30613800	-0.03715900
C	2.01612800	-3.09426500	-0.09429300
C	0.73890900	-3.54704100	0.00224200
N	1.94385300	-1.71593100	-0.11890000

N	-0.07454400	-2.43068900	0.03812400
C	-1.50595400	-2.43716800	0.14992200
C	-2.06859800	-2.43490900	1.43411100
C	-2.26532400	-2.41868700	-1.02871400
C	-3.46243200	-2.43405400	1.51619300
C	-3.65450900	-2.41653400	-0.88846000
C	-4.24663000	-2.43258300	0.36885900
H	-3.93862200	-2.43398500	2.49228200
H	-4.28008000	-2.40581900	-1.77631600
H	-5.32936800	-2.43913200	0.45483600
C	3.04524500	-0.79833700	-0.20559300
C	3.59794300	-0.31523900	0.98862100
C	3.46587300	-0.38850600	-1.47837400
C	4.62561200	0.62270600	0.87617100
C	4.49576800	0.55194400	-1.53205300
C	5.06899000	1.05185700	-0.36891100
H	5.08335100	1.02219500	1.77664700
H	4.85086100	0.89920300	-2.49795300
H	5.87040100	1.78214600	-0.43385700
C	-1.63325500	-2.36976300	-2.40483300
H	-0.54783100	-2.47017500	-2.28968100
C	-1.89914700	-1.01505800	-3.06759600
H	-2.97315000	-0.84815100	-3.20910300
H	-1.50608400	-0.19416500	-2.45572500
H	-1.41791500	-0.96604800	-4.05030600
C	-2.10818000	-3.52386900	-3.28952800
H	-1.58940100	-3.49942100	-4.25356500
H	-1.91201800	-4.49323600	-2.82009000
H	-3.18282200	-3.46138500	-3.49191900
C	2.82412700	-0.90155700	-2.75158600
H	2.10924300	-1.68841200	-2.48368600
C	3.85643500	-1.52498300	-3.69301500
H	3.36024700	-1.94253200	-4.57545500
H	4.58176100	-0.78274000	-4.04329400
H	4.41246600	-2.32923400	-3.20032800
C	2.03886900	0.21341700	-3.44803300
H	1.27209800	0.63185900	-2.78555500
H	2.70043800	1.03164400	-3.75336500
H	1.54288700	-0.17084900	-4.34615600
C	3.10756800	-0.75761100	2.35260900
H	2.34113300	-1.52844900	2.21027000
C	-1.22477700	-2.39056600	2.69168600
H	-0.17297400	-2.50429100	2.40490600
C	-1.36715300	-1.02980800	3.38013600
H	-1.08179400	-0.21296800	2.70717500
H	-2.40071000	-0.85587300	3.70020900
H	-0.72709600	-0.98075600	4.26775900
C	-1.56369300	-3.53596900	3.64705300
H	-0.89575300	-3.51391900	4.51448200
H	-2.59036500	-3.45972300	4.02099700
H	-1.45696500	-4.50966700	3.15793000
C	2.45226000	0.40535200	3.10205300
H	3.16788600	1.21479800	3.28373300
H	1.61230300	0.82202700	2.53407700
H	2.07288100	0.06880200	4.07301500
C	4.23596300	-1.38276800	3.17586300
H	5.02484600	-0.65457600	3.39305600
H	3.84968800	-1.74746100	4.13352800
H	4.69533500	-2.22530300	2.64871800
C	-0.11079900	2.76384800	0.09406800
Br	1.36145800	3.71418200	0.49083500
H	0.33357900	-4.54566900	0.04920800
H	2.95946700	-3.61492700	-0.14696500
C	-2.68363500	2.24710500	-0.51443000
C	-3.37763200	1.22103600	0.39753200
C	-3.36489100	3.62600000	-0.42764300

H	-2.75856000	1.88729300	-1.55187800
C	-4.86055800	1.12278600	0.05610100
H	-3.25040100	1.53646800	1.44207800
H	-2.89722200	0.24133800	0.29449400
C	-4.84765700	3.50211900	-0.77015900
H	-3.24701000	4.01116200	0.59388500
H	-2.86654100	4.33179300	-1.10115800
C	-5.54743000	2.48381400	0.12583700
H	-5.34054900	0.41109500	0.73690500
H	-4.96715100	0.70587900	-0.95607300
H	-5.32228200	4.48542200	-0.67935900
H	-4.95178700	3.19971900	-1.82196400
H	-6.60120500	2.39141000	-0.15955100
H	-5.53334200	2.84420800	1.16426400

Cartesian coordinates of the optimized geometry for Int-**2** at PBE0-D3BJ/6-31G*,def2-TZVP level of theory: (number of imaginary frequencies = 0):

Au	0.06555200	-0.57879900	-0.36387400
C	1.35534500	-1.96961300	-0.91902600
C	-1.15588700	0.92838700	0.25862600
C	-1.82445100	2.94719500	0.98886700
C	-2.93123300	2.16600800	0.86621600
N	-0.75219500	2.16911000	0.61153800
N	-2.49620600	0.93692500	0.42011900
C	-3.33076600	-0.19982600	0.15283800
C	-3.83438200	-0.36057000	-1.14476700
C	-3.56863800	-1.10526500	1.19568400
C	-4.61145500	-1.49467600	-1.38675500
C	-4.35127100	-2.22215300	0.89876100
C	-4.86686300	-2.41484500	-0.37737000
H	-5.02037000	-1.65924100	-2.37942700
H	-4.55925800	-2.95053000	1.67718000
H	-5.47532700	-3.28992300	-0.58665900
C	0.62197100	2.58827200	0.59877700
C	1.13721900	3.13889400	-0.58244200
C	1.38285300	2.39693900	1.75986200
C	2.47975400	3.51977700	-0.57244800
C	2.72053100	2.79347800	1.71490900
C	3.26251100	3.35274600	0.56362600
H	2.91874600	3.94908600	-1.46826800
H	3.34546400	2.66170600	2.59345400
H	4.30444000	3.65983500	0.55121900
C	-2.98301200	-0.91912700	2.58116300
H	-2.52751600	0.07672800	2.63106400
C	-1.87204200	-1.94183800	2.83566300
H	-2.26274600	-2.96510100	2.79556800
H	-1.07879100	-1.85113800	2.08394900
H	-1.42581000	-1.78795100	3.82438700
C	-4.05607200	-0.98367400	3.66922400
H	-3.61375500	-0.77390100	4.64885600
H	-4.85121700	-0.25296600	3.48938600
H	-4.51849500	-1.97508300	3.72327700
C	0.81449800	1.75405200	3.00874700
H	-0.26460500	1.62198900	2.86778400
C	1.00843800	2.63600400	4.24330200
H	0.53173800	2.17607200	5.11543000
H	2.06921000	2.77023900	4.48091000
H	0.57020500	3.62872300	4.09778100
C	1.42149200	0.36381400	3.21630500
H	1.23510300	-0.27770400	2.34737600
H	2.50615100	0.42374000	3.36036100
H	0.98745300	-0.11727800	4.09983700
C	0.30605000	3.28909000	-1.84013600
H	-0.72984200	3.02020800	-1.60286500

C	-3.53399800	0.62129300	-2.25924800
H	-3.01723400	1.48551300	-1.82587700
C	-2.59041200	-0.00675400	-3.28871400
H	-1.65755100	-0.33886500	-2.81757800
H	-3.05335700	-0.87646100	-3.76849300
H	-2.34021900	0.71745100	-4.07185100
C	-4.81193200	1.13995500	-2.92091700
H	-4.56705700	1.89554800	-3.67485800
H	-5.35964200	0.33711600	-3.42606100
H	-5.48566600	1.59446500	-2.18728300
C	0.79368500	2.32063700	-2.92134800
H	1.82815500	2.53877600	-3.20930500
H	0.75639600	1.28431000	-2.56518900
H	0.16834600	2.39952200	-3.81746200
C	0.29831700	4.73199600	-2.34799700
H	1.29875900	5.05740400	-2.65295400
H	-0.35755400	4.82266600	-3.22032800
H	-0.05833900	5.42438500	-1.57845600
C	1.21762000	-3.43412900	-0.95607800
C	2.45665500	-3.96757900	-1.69231800
H	2.33918400	-3.87415000	-2.77844000
C	3.53899300	-3.02057200	-1.16054300
H	2.67280700	-1.45107600	-2.36428000
C	2.76402700	-1.69326900	-1.28848100
Br	3.53527500	-0.11376500	-0.47700400
H	-3.97473300	2.36758600	1.05173700
H	-1.70196600	3.97117900	1.30572600
H	4.44326100	-3.00638900	-1.77368100
C	3.86472800	-3.34978800	0.29644200
C	1.37169600	-3.80164800	0.57167800
C	2.63455500	-3.20358900	1.19500600
H	2.81695500	-3.66842100	2.16920000
H	2.46814700	-2.13541100	1.39599800
H	4.24458500	-4.37834700	0.33161600
H	4.66761600	-2.70048100	0.66073400
H	2.65902900	-5.01790300	-1.46174300
H	1.40517100	-4.89804400	0.58694000
H	0.47748600	-3.48286200	1.11461200
H	0.24109000	-3.78807000	-1.29636700

Cartesian coordinates of the optimized geometry for Int-**3** at PBE0-D3BJ/6-31G*,def2-TZVP level of theory: (number of imaginary frequencies = 0):

Au	-0.07212300	0.42593000	-0.25659000
C	-1.26561400	2.20231100	-0.81647600
C	0.57709300	-1.40617800	0.26542300
C	1.94354900	-3.07888300	0.86666100
C	0.68985300	-3.59132000	0.75481700
N	1.84890400	-1.73549700	0.56081700
N	-0.13225300	-2.54405600	0.38545200
C	-1.54744100	-2.59008500	0.15070700
C	-2.40399400	-2.36917800	1.23842400
C	-1.99290600	-2.78058100	-1.16466700
C	-3.77225800	-2.31803300	0.96568200
C	-3.37143700	-2.72104800	-1.38015800
C	-4.24987500	-2.48538000	-0.32911300
H	-4.47219400	-2.14429700	1.77777900
H	-3.76065500	-2.86354600	-2.38428300
H	-5.31830000	-2.44051000	-0.51938500
C	2.91749600	-0.77592400	0.54153800
C	3.15249000	-0.02420900	1.70133500
C	3.62267600	-0.59540000	-0.65645300
C	4.16055000	0.93927600	1.63804900
C	4.61871800	0.38241100	-0.66409400
C	4.88673000	1.13917800	0.47027200

H	4.37734900	1.54173000	2.51552500
H	5.18944100	0.55457000	-1.57205900
H	5.66902300	1.89236600	0.44383600
C	-1.04346100	-3.04647600	-2.31599500
H	-0.02421200	-3.09683700	-1.91588800
C	-1.07460100	-1.91114400	-3.34136600
H	-2.07119400	-1.79940300	-3.78323200
H	-0.79875300	-0.95423300	-2.88409800
H	-0.36963100	-2.11384100	-4.15480200
C	-1.34302600	-4.39570600	-2.97411600
H	-0.61044500	-4.60438700	-3.76089400
H	-1.30567100	-5.21252600	-2.24598800
H	-2.33645300	-4.40462400	-3.43558600
C	3.30936900	-1.38285300	-1.91248800
H	2.55957800	-2.14325100	-1.66482500
C	4.54287400	-2.11486100	-2.44450200
H	4.27602400	-2.72225000	-3.31575200
H	5.32315200	-1.41321300	-2.75850200
H	4.97359100	-2.77592200	-1.68539900
C	2.70017000	-0.47019400	-2.98069200
H	1.79222400	0.02141700	-2.61198400
H	3.40564800	0.31199700	-3.28215000
H	2.43677700	-1.04790400	-3.87338800
C	2.34787100	-0.20920800	2.97189300
H	1.62759500	-1.01951100	2.80953600
C	-1.88731800	-2.17184600	2.64997700
H	-0.81406400	-2.39475800	2.65585500
C	-2.05294900	-0.71722700	3.09656800
H	-1.51693700	-0.03201600	2.43043500
H	-3.10914900	-0.42445800	3.10235000
H	-1.66116300	-0.58140100	4.11057000
C	-2.55622500	-3.13250800	3.63507100
H	-2.10813900	-3.02539000	4.62844600
H	-3.62764700	-2.92804800	3.73480000
H	-2.44204000	-4.17428000	3.31855500
C	1.54792200	1.05451500	3.29919600
H	2.20966000	1.90528100	3.49532300
H	0.88460600	1.33022600	2.47085100
H	0.93209900	0.89643100	4.19114000
C	3.24257700	-0.61914200	4.14374200
H	3.97082600	0.16319000	4.38361300
H	2.63817400	-0.79559300	5.03982300
H	3.79860600	-1.53544200	3.91997700
C	-0.06797200	2.52600400	-0.77148900
Br	1.50581900	3.39255000	-0.80106800
H	0.30793200	-4.58951800	0.90170200
H	2.88379800	-3.53696900	1.13100400
C	-2.73622300	2.15325300	-0.95448300
C	-3.20804800	3.60649300	-1.19361900
C	-3.37543100	1.61559200	0.34115000
C	-2.96587900	4.51411200	0.00849700
H	-4.28525800	3.54864800	-1.40252300
H	-2.73020200	4.00645600	-2.09571200
C	-3.11606900	2.51564600	1.54330700
H	-4.45596200	1.55227500	0.15304700
H	-3.02961000	0.59039400	0.52293100
C	-3.59307800	3.94165000	1.27754900
H	-3.37251800	5.50936300	-0.20261500
H	-1.88560900	4.64839200	0.15977200
H	-3.61759400	2.10256300	2.42590800
H	-2.04013100	2.52225200	1.77385000
H	-4.68709700	3.93870800	1.16937400
C	-3.10699000	1.25514000	-2.13892500
H	-2.62580800	1.59133200	-3.06287000
H	-2.81982300	0.21587700	-1.94781400
H	-4.19186100	1.28590400	-2.28430400

H	-3.36534000	4.58474900	2.13487600

Cartesian coordinates of the optimized geometry for Int-**4** at PBE0-D3BJ/6-31G*,def2-TZVP level of theory: (number of imaginary frequencies = 0):

Au	0.06193400	-0.48384200	-0.28205600
C	1.25220200	-1.99668000	-0.72977000
C	-1.10258900	1.11030000	0.20901300
C	-1.74118500	3.19434400	0.75379500
C	-2.85950000	2.42032300	0.70526300
N	-0.68031000	2.37065200	0.44624200
N	-2.44199200	1.14991000	0.37208700
C	-3.27546300	-0.00796300	0.21177400
C	-3.74671000	-0.31260800	-1.07214900
C	-3.52201800	-0.80609500	1.33731900
C	-4.50140800	-1.47888100	-1.21092800
C	-4.28037200	-1.96154900	1.14213100
C	-4.76463600	-2.29477700	-0.11744700
H	-4.88543200	-1.75184300	-2.18965000
H	-4.49296600	-2.60944100	1.98755900
H	-5.35444100	-3.19762300	-0.24724200
C	0.70285300	2.75516300	0.38552000
C	1.21791900	3.18507900	-0.84481500
C	1.47579000	2.63231400	1.54772000
C	2.57315800	3.51528400	-0.88526300
C	2.82533600	2.97614700	1.45183700
C	3.36794100	3.41546400	0.25025900
H	3.01270100	3.85023300	-1.82023500
H	3.45947000	2.89544800	2.33002900
H	4.42003600	3.68063400	0.19761500
C	-2.96254400	-0.47369300	2.70603100
H	-2.52521200	0.53057400	2.66442700
C	-1.83801800	-1.44606700	3.07369600
H	-2.21085400	-2.47548700	3.12764400
H	-1.03438500	-1.41352700	2.32840800
H	-1.40980700	-1.18974800	4.04900500
C	-4.05094100	-0.45234300	3.78035900
H	-3.62728600	-0.13973400	4.74057900
H	-4.85591900	0.24218800	3.51933300
H	-4.49668700	-1.44234400	3.92509200
C	0.90807600	2.11335900	2.85311700
H	-0.17388700	1.98491000	2.73197900
C	1.12493100	3.10195700	4.00008500
H	0.65435200	2.72895000	4.91599200
H	2.18993800	3.24589600	4.21179300
H	0.69478900	4.08196500	3.76911700
C	1.49713100	0.73904700	3.18272700
H	1.30248200	0.02565700	2.37359900
H	2.58247400	0.79677200	3.32175800
H	1.05764300	0.34393200	4.10530900
C	0.37255700	3.25501100	-2.10012700
H	-0.66916100	3.05180000	-1.82570500
C	-3.43801400	0.55001000	-2.27890700
H	-2.90025600	1.44162500	-1.93641200
C	-2.51845400	-0.19291800	-3.25185500
H	-1.58849400	-0.49916400	-2.75790700
H	-3.00362000	-1.09340600	-3.64513500
H	-2.26042300	0.44888300	-4.10136000
C	-4.71538500	1.02541100	-2.97396000
H	-4.46773400	1.69256400	-3.80638400
H	-5.28743700	0.18553800	-3.38295400
H	-5.36761300	1.56873000	-2.28248500
C	0.80398800	2.17269600	-3.09381200
H	1.84044400	2.32376900	-3.41563800
H	0.73527400	1.17578700	-2.64235200

H	0.16617700	2.19250000	-3.98444000
C	0.41350900	4.64450700	-2.73831900
H	1.42073400	4.89931100	-3.08516100
H	-0.25286800	4.68200900	-3.60660400
H	0.09771600	5.41829000	-2.03100100
C	0.96833900	-3.44147300	-0.69750500
C	2.18336300	-4.11774400	-1.36410300
H	2.08392300	-4.08870300	-2.45666000
C	3.34009000	-3.23606800	-0.88333700
H	2.60696900	-1.68287400	-2.18964400
C	2.67789000	-1.86080200	-1.09966000
Br	3.57849400	-0.29790000	-0.39252300
H	-3.89915500	2.65344200	0.87485900
H	-1.60479500	4.24124200	0.97598700
H	4.24380300	-3.33601500	-1.48938300
C	3.63449100	-3.49481800	0.59344300
C	1.11315000	-3.69676400	0.86129000
C	2.41879700	-3.17750600	1.46537500
H	2.54690900	-3.59778000	2.46813200
H	2.35162100	-2.08872800	1.60034500
H	2.28875400	-5.16497800	-1.06317500
H	1.04931600	-4.78998300	0.94599800
H	0.24557500	-3.26841600	1.37216800
H	3.91969400	-4.54865900	0.70094500
H	4.49150900	-2.89638800	0.91941400
C	-0.40681000	-3.87785700	-1.17207300
H	-1.19967500	-3.33412700	-0.64743700
H	-0.52115300	-3.69686500	-2.24580600
H	-0.54179900	-4.94955900	-0.99222800

Cartesian coordinates of the optimized geometry for Int-**5** at PBE0-D3BJ/6-31G*,def2-TZVP level of theory: (number of imaginary frequencies = 0):

Au	0.02512100	0.52303200	-0.21339700
C	-1.22717000	2.24383600	-0.80929800
C	0.71760400	-1.33308900	0.14238100
C	2.12497200	-3.05238700	0.45040200
C	0.85903300	-3.52731100	0.58509900
N	2.01233200	-1.70430500	0.17744200
N	0.01225700	-2.45305200	0.39392200
C	-1.42129400	-2.48667700	0.45976100
C	-2.03181100	-2.22078700	1.69319000
C	-2.13369900	-2.75088500	-0.71873200
C	-3.42774900	-2.23346500	1.72461600
C	-3.52652800	-2.75768900	-0.62887900
C	-4.16621400	-2.50530300	0.57935300
H	-3.94142400	-2.03393100	2.66060800
H	-4.11789600	-2.95946300	-1.51709800
H	-5.25135200	-2.52121700	0.62853200
C	3.09076900	-0.77739600	-0.02318000
C	3.59894200	-0.10457700	1.09673200
C	3.53608100	-0.55302600	-1.33304800
C	4.60543800	0.83482700	0.86743500
C	4.54451200	0.39672000	-1.50444200
C	5.07253700	1.08316100	-0.41764600
H	5.02775500	1.37908600	1.70727700
H	4.91867900	0.60266400	-2.50311400
H	5.85718400	1.81780000	-0.57380200
C	-1.44556700	-2.97964400	-2.04930700
H	-0.36764700	-3.06456900	-1.86901600
C	-1.66134700	-1.77863000	-2.97516000
H	-2.72585700	-1.63896800	-3.19579500
H	-1.28610000	-0.85468900	-2.51877300
H	-1.13620800	-1.92575100	-3.92515500
C	-1.90072000	-4.27985800	-2.71386400

H	-1.33463700	-4.45056600	-3.63549600
H	-1.74811800	-5.14027700	-2.05443400
H	-2.96216300	-4.24724800	-2.98209200
C	2.94808300	-1.27549200	-2.52803800
H	2.21996400	-2.00908500	-2.16253800
C	4.02108400	-2.04351200	-3.30264800
H	3.56640500	-2.60232200	-4.12746700
H	4.76662600	-1.36653500	-3.73344600
H	4.54818000	-2.75389800	-2.65740600
C	2.19728600	-0.29934600	-3.43773300
H	1.40712400	0.22606900	-2.88863900
H	2.87373800	0.45514600	-3.85421900
H	1.73542500	-0.83453100	-4.27463800
C	3.08081300	-0.34926000	2.49979200
H	2.35240900	-1.16760600	2.46086300
C	-1.23774000	-1.91377100	2.94668900
H	-0.17279000	-2.03743000	2.71847200
C	-1.44605600	-0.46038500	3.37967300
H	-1.14897000	0.23496800	2.58641400
H	-2.49671200	-0.26447200	3.62119100
H	-0.84863100	-0.23760100	4.27034700
C	-1.57758600	-2.88527400	4.07891300
H	-0.94669000	-2.68458400	4.95117700
H	-2.62100000	-2.78421200	4.39647300
H	-1.41982500	-3.92507300	3.77447300
C	2.35152000	0.88572700	3.03487200
H	3.02651500	1.74604400	3.10381200
H	1.51573000	1.16692700	2.38402500
H	1.95304000	0.69054600	4.03644100
C	4.20342300	-0.78375900	3.44440300
H	4.95182700	0.00620700	3.56941700
H	3.79730800	-1.01359700	4.43513600
H	4.71788200	-1.67481800	3.07010300
C	-0.14683500	2.67455500	-0.37518600
Br	1.22237100	3.71586800	0.14484900
H	0.48249300	-4.51587400	0.79663100
H	3.08491000	-3.53919800	0.52327900
C	-2.57903200	2.06896000	-1.36380500
C	-3.25771600	3.45516300	-1.40435700
C	-3.41351100	1.03998400	-0.56166400
C	-3.58564200	3.96666000	-0.00459700
H	-4.17734500	3.35196000	-1.99251400
H	-2.61977600	4.16370200	-1.94404000
C	-3.73484400	1.59240900	0.83030000
H	-2.80345900	0.13445100	-0.43770600
C	-4.41935300	2.95698100	0.78186300
H	-4.11316300	4.92379200	-0.08128500
H	-2.65194400	4.17034900	0.53890900
H	-4.35438600	0.86469300	1.36786900
H	-2.79989800	1.68234600	1.40239900
H	-5.41147100	2.86312000	0.32099600
H	-4.58869400	3.32323800	1.80061700
H	-2.45551600	1.69490300	-2.38995700
C	-4.66030600	0.63760900	-1.34278300
H	-4.39360400	0.20584300	-2.31429800
H	-5.21997000	-0.11872500	-0.78432500
H	-5.33591200	1.47850900	-1.52576300

Cartesian coordinates of the optimized geometry for Int-**6a** at PBE0-D3BJ/6-31G*,def2-TZVP level of theory: (number of imaginary frequencies = 0):

Au	0.11038600	-0.48004200	-0.28698100
C	1.66347100	-1.63490800	-0.69051800
C	-1.40017700	0.80672600	0.17663000
C	-2.46928700	2.71122600	0.71203100

C	-3.40049900	1.72253600	0.63891300
N	-1.25497500	2.12736500	0.42519500
N	-2.72211700	0.56831500	0.31202800
C	-3.31315100	-0.72714900	0.13154700
C	-3.72675900	-1.09217300	-1.15639800
C	-3.41357800	-1.56947200	1.24713100
C	-4.26151000	-2.37231200	-1.31112600
C	-3.95500500	-2.83845600	1.03607800
C	-4.37401400	-3.23559000	-0.22817000
H	-4.59286600	-2.69683800	-2.29326600
H	-4.04940800	-3.52457800	1.87272000
H	-4.79443600	-4.22699700	-0.37016300
C	0.01262100	2.80309500	0.40103200
C	0.45353000	3.34380400	-0.81430200
C	0.75479800	2.86022400	1.58826700
C	1.69614700	3.97892100	-0.81294200
C	1.99140000	3.50554500	1.53361200
C	2.45557400	4.06284900	0.34790600
H	2.07581900	4.40959800	-1.73484000
H	2.59860800	3.57124000	2.43180400
H	3.41845800	4.56536700	0.32790400
C	-2.92755100	-1.15549800	2.62182500
H	-2.68767900	-0.08620200	2.59390400
C	-1.64004800	-1.90231900	2.98200900
H	-1.81107200	-2.98430100	3.02007100
H	-0.85596100	-1.70786700	2.24047900
H	-1.27044900	-1.58260100	3.96262200
C	-4.00099000	-1.35282000	3.69327100
H	-3.64554000	-0.97465500	4.65760900
H	-4.92347300	-0.82204900	3.43670600
H	-4.24968400	-2.41099600	3.82761300
C	0.27705100	2.22441700	2.87801100
H	-0.74837900	1.86742100	2.72690900
C	0.24490800	3.22913500	4.03093200
H	-0.16399100	2.75880500	4.93148900
H	1.24819900	3.59352600	4.27662800
H	-0.37562200	4.09750900	3.78675500
C	1.13814600	1.00663000	3.22419000
H	1.11718800	0.26942500	2.41339800
H	2.18294100	1.29448900	3.38649200
H	0.77282500	0.52497600	4.13798000
C	-0.34541600	3.22289400	-2.09574900
H	-1.31845200	2.77939100	-1.85456300
C	-3.57970200	-0.16811000	-2.34839600
H	-3.25613000	0.81386600	-1.98409600
C	-2.49463400	-0.68384400	-3.29769000
H	-1.53344100	-0.78485500	-2.77966400
H	-2.76204200	-1.66514900	-3.70567200
H	-2.36047800	0.00612500	-4.13811900
C	-4.90800800	0.03292000	-3.07990300
H	-4.78551100	0.75565300	-3.89351600
H	-5.27103500	-0.90116000	-3.52195300
H	-5.68387700	0.40694000	-2.40399100
C	0.35751700	2.27896400	-3.07535000
H	1.34064400	2.66865800	-3.36210200
H	0.50707800	1.28949400	-2.62724500
H	-0.23859200	2.15806100	-3.98658000
C	-0.61099900	4.58831900	-2.73207100
H	0.31891300	5.07438500	-3.04639800
H	-1.24033500	4.47551900	-3.62121500
H	-1.12102800	5.26134200	-2.03513600
C	1.81291200	-3.09480400	-0.59996200
C	3.14796100	-3.43537600	-1.29902800
H	2.99261900	-3.31062000	-2.38109600
C	4.01324000	-2.25782300	-0.80633800
H	2.90198900	-0.98777900	-2.15109600

C	3.00485600	-1.12126300	-1.05733400
Br	3.42808600	0.64116000	-0.37514300
H	-4.46918300	1.73238700	0.78643300
H	-2.55680300	3.76234000	0.93882900
H	4.91771100	-2.11841700	-1.40455400
C	4.35209600	-2.38412700	0.67777600
C	1.97620900	-3.28197400	0.95837500
C	3.09010200	-2.41099400	1.54526600
H	3.32954000	-2.75470400	2.55669100
H	2.71947000	-1.38172700	1.65586600
H	2.18727300	-4.34806000	1.09518800
H	1.01939800	-3.07151000	1.44430800
H	4.94461900	-3.29389800	0.82433800
H	4.98818500	-1.54637200	0.98224400
H	0.93272200	-3.66053800	-0.91871700
C	3.69223700	-4.83688800	-1.07832400
H	2.97563200	-5.58545600	-1.43301300
H	4.61919300	-4.97463800	-1.64418900
H	3.91190300	-5.05542700	-0.03009200

Cartesian coordinates of the optimized geometry for Int-**6b** at PBE0-D3BJ/6-31G*,def2-TZVP level of theory: (number of imaginary frequencies = 0):

Au	0.08101700	-0.44672800	-0.39766200
C	1.48488400	-1.69725700	-1.01191700
C	-1.26585600	0.91956000	0.28874900
C	-2.10584300	2.83937900	1.10465500
C	-3.14080300	1.97113100	0.94667000
N	-0.97084900	2.17430400	0.69562000
N	-2.60153200	0.80467100	0.44871400
C	-3.33480500	-0.38759700	0.13107800
C	-3.81973700	-0.53806400	-1.17482300
C	-3.49659400	-1.35190700	1.13503500
C	-4.49456200	-1.72448400	-1.46745200
C	-4.17869100	-2.51922900	0.78840600
C	-4.67186700	-2.70378500	-0.49778300
H	-4.88513700	-1.88296800	-2.46847700
H	-4.32511400	-3.29384200	1.53556800
H	-5.20157300	-3.61897400	-0.74609100
C	0.36178400	2.71108700	0.70357700
C	0.82497900	3.35506900	-0.45153400
C	1.13912600	2.53502500	1.85619600
C	2.12974100	3.84944800	-0.42294900
C	2.43762600	3.04659800	1.83052300
C	2.92671300	3.70051700	0.70572600
H	2.52814400	4.35352900	-1.29856600
H	3.07380000	2.93035900	2.70317700
H	3.93840400	4.09619000	0.70826700
C	-2.93200800	-1.17265500	2.53015300
H	-2.57139300	-0.14182400	2.62521900
C	-1.73043700	-2.09749200	2.74424800
H	-2.02381300	-3.14992300	2.65640600
H	-0.94905700	-1.89955000	2.00091100
H	-1.30090000	-1.94702500	3.74089400
C	-3.99430000	-1.38448500	3.60992300
H	-3.57358400	-1.17652900	4.59940600
H	-4.85449900	-0.72445500	3.45844300
H	-4.36168300	-2.41628300	3.61980300
C	0.63045200	1.79261700	3.07528600
H	-0.43324400	1.57328900	2.92620800
C	0.74845100	2.63542100	4.34627500
H	0.31475600	2.09958600	5.19727600
H	1.79385200	2.85141000	4.59165900
H	0.22527900	3.59147600	4.24148000
C	1.35586300	0.45272500	3.22585600

H	1.22390400	-0.16582100	2.33077700
H	2.43155100	0.59989600	3.37438900
H	0.96654500	-0.10044300	4.08781200
C	-0.01874100	3.48712900	-1.70282300
H	-1.02674200	3.11897000	-1.47894600
C	-3.60504300	0.51182900	-2.24627300
H	-3.16463500	1.39837200	-1.77538800
C	-2.61106100	0.01152300	-3.29795700
H	-1.65242800	-0.25638000	-2.83787500
H	-2.99635800	-0.87498700	-3.81424800
H	-2.42583100	0.78639900	-4.05003200
C	-4.92328400	0.94573400	-2.88971600
H	-4.74481100	1.75079600	-3.61036400
H	-5.39983500	0.12065600	-3.42996600
H	-5.63346100	1.30908700	-2.13981800
C	0.54946500	2.61285600	-2.82420800
H	1.55990400	2.93363100	-3.10121100
H	0.60521200	1.56301800	-2.51298000
H	-0.08280000	2.67473000	-3.71680100
C	-0.15304100	4.94464500	-2.14718800
H	0.81484700	5.36905100	-2.43504600
H	-0.81542000	5.01586200	-3.01641400
H	-0.56775400	5.56896400	-1.34902600
C	1.47417600	-3.16538200	-1.09396600
C	2.74135900	-3.55480800	-1.87036700
H	2.59015700	-3.41520800	-2.94751800
C	3.74412400	-2.54048800	-1.31040400
H	2.73695200	-1.01256200	-2.45542600
C	2.85754800	-1.28233200	-1.38977500
Br	3.49661400	0.33080400	-0.52987700
H	-4.19747600	2.07322400	1.13892700
H	-2.07202400	3.85545900	1.46572400
H	4.64107900	-2.42964100	-1.92449000
C	4.10116400	-2.89094000	0.13422000
C	1.64484400	-3.56787600	0.43871300
C	2.86943000	-2.86584700	1.04304600
H	3.10537600	-3.31598700	2.01370900
H	2.61349400	-1.81626600	1.25112200
H	0.74428600	-3.21111400	0.95047300
H	4.56801100	-3.88324700	0.13703800
H	4.84965200	-2.18975200	0.51799400
H	0.52672700	-3.58809000	-1.43926000
H	3.04520900	-4.59178200	-1.71026600
C	1.69820600	-5.08538700	0.57087200
H	1.66156000	-5.34430900	1.63495300
H	0.84765400	-5.56593900	0.07689200
H	2.61849800	-5.51417600	0.16388800

Cartesian coordinates of the optimized geometry for Int-**6c** at PBE0-D3BJ/6-31G*,def2-TZVP level of theory: (number of imaginary frequencies = 0):

Au	0.11418500	0.37035400	-0.29023200
C	1.74516300	1.44159600	-0.69794500
C	-1.41284500	-0.88391900	0.17895700
C	-3.41268800	-1.81653100	0.60908900
C	-2.46129300	-2.75236100	0.86783500
N	-2.74677400	-0.68358500	0.18943300
N	-1.24664000	-2.15879400	0.59718900
C	0.04316700	-2.77264300	0.74237300
C	0.58767100	-3.44404400	-0.36133900
C	0.71100100	-2.63263800	1.96706400
C	1.85517700	-4.00778900	-0.20180100
C	1.97497600	-3.21627500	2.07311300
C	2.53971400	-3.90011800	1.00323500
H	2.31099000	-4.53808400	-1.03304500

H	2.52350700	-3.13202800	3.00689800
H	3.52251300	-4.35090500	1.10819100
C	-3.36287300	0.55814500	-0.18258400
C	-3.72725300	0.74029600	-1.52346300
C	-3.53827100	1.53272500	0.80866200
C	-4.29358600	1.96930700	-1.86486500
C	-4.10944800	2.74339900	0.41271800
C	-4.48308000	2.95935100	-0.90798100
H	-4.58821000	2.15412400	-2.89371800
H	-4.26232700	3.52692300	1.14932600
H	-4.92722500	3.90824500	-1.19461100
C	0.12518600	-1.85905700	3.13057500
H	-0.88249400	-1.52997600	2.85241800
C	0.95336600	-0.60277400	3.41443600
H	1.97418900	-0.86132400	3.71823200
H	1.01700600	0.03737600	2.52676200
H	0.49964900	-0.02093500	4.22424900
C	-0.00644000	-2.73365900	4.37890300
H	-0.48036400	-2.16927300	5.18897900
H	-0.61362400	-3.62309100	4.18114600
H	0.97198600	-3.07025100	4.73870500
C	-3.11231400	1.31672300	2.24678600
H	-2.77460000	0.27927900	2.35330900
C	-4.27784600	1.51830700	3.21705300
H	-3.96015900	1.29767500	4.24171000
H	-4.64178200	2.55131900	3.19994500
H	-5.12009700	0.86323500	2.97137800
C	-1.92911500	2.22091000	2.60219100
H	-1.08121200	2.04656200	1.93009100
H	-2.20418800	3.27906800	2.52755900
H	-1.59795200	2.03109600	3.62935400
C	-3.48401100	-0.32027800	-2.57833500
H	-3.19378300	-1.24788100	-2.07117900
C	-0.12887000	-3.53330800	-1.69320000
H	-1.12809300	-3.09866200	-1.57588000
C	0.60692300	-2.70709700	-2.75202300
H	0.69237800	-1.65896400	-2.44139100
H	1.61787800	-3.09454700	-2.92321500
H	0.06858200	-2.73806500	-3.70559100
C	-0.30927600	-4.98367100	-2.14474800
H	-0.88031800	-5.02120800	-3.07843300
H	0.65417600	-5.47185600	-2.32758300
H	-0.84471700	-5.57224400	-1.39267400
C	-2.31997700	0.08866300	-3.48577700
H	-2.55191000	1.01253800	-4.02778200
H	-1.40728700	0.25938100	-2.90264700
H	-2.11484600	-0.69437100	-4.22436600
C	-4.74263200	-0.62253300	-3.39269600
H	-5.06031000	0.24291400	-3.98406100
H	-4.55114700	-1.44362800	-4.09160600
H	-5.57762400	-0.91131700	-2.74613500
C	3.31380100	3.10885200	-1.56437400
H	1.07780600	3.43938600	-1.42264500
C	1.91747600	2.88567600	-1.00835200
Br	1.87161500	3.21949500	0.95970000
H	-2.53425800	-3.77118500	1.21502300
H	-4.48848900	-1.84914900	0.68287400
H	3.25011000	2.99099000	-2.65671000
H	3.69996100	4.11054800	-1.35994100
C	3.08313700	0.84272400	-0.87151900
C	3.54811800	-0.38716100	-0.11555900
C	4.11778700	1.96822800	-0.94228500
H	2.90515400	0.52617800	-1.93431100
H	2.78939200	-1.17721200	-0.13813600
C	4.86149600	-0.86885700	-0.73899100
H	5.26699800	-1.69905700	-0.15023300

H	4.64627800	-1.27369800	-1.73884600
C	5.38677600	1.47286600	-1.61772300
H	5.17063900	1.20780800	-2.66328100
H	6.15094600	2.25805900	-1.63885800
C	5.90700100	0.24470400	-0.86045200
H	6.21836100	0.55755800	0.14621300
H	6.80385000	-0.14436000	-1.35459100
H	4.36785100	2.24410900	0.09252400
H	3.69689500	-0.11132600	0.93717000

Cartesian coordinates of the optimized geometry for Int-**7** at PBE0-D3BJ/6-31G*,def2-TZVP level of theory: (number of imaginary frequencies = 0):

Au	0.07603600	-0.46070700	-0.13613700
C	1.59868400	-2.00015100	-0.57629200
C	-0.88313100	1.30121700	0.03135400
C	-2.52397200	2.82447200	0.16859000
C	-1.34002200	3.48752700	0.23760700
N	-2.21767400	1.48491100	0.04044000
N	-0.34675700	2.53145500	0.15336100
C	1.06702100	2.77748500	0.19743200
C	1.70570500	2.73717000	1.44475200
C	1.73774000	3.01369500	-1.01107100
C	3.08598900	2.94800400	1.45653700
C	3.11603600	3.22385800	-0.94031900
C	3.78271000	3.19217200	0.27906500
H	3.62006400	2.92524900	2.40208200
H	3.67471300	3.41041500	-1.85275100
H	4.85544800	3.35939500	0.31178300
C	-3.15167200	0.39810800	-0.05338200
C	-3.56016700	-0.22503900	1.13399400
C	-3.55902800	-0.01643900	-1.32876700
C	-4.42411500	-1.31446100	1.01186400
C	-4.42339000	-1.11057100	-1.39223400
C	-4.85093700	-1.75218900	-0.23609700
H	-4.76588000	-1.82651900	1.90679100
H	-4.76323900	-1.46594300	-2.36072700
H	-5.52421800	-2.60140900	-0.30859300
C	1.02759400	3.00011800	-2.34970000
H	-0.05238000	2.96068600	-2.16578700
C	1.40367300	1.74276800	-3.13978400
H	2.47949800	1.71621500	-3.34785000
H	1.14370000	0.83414400	-2.58336600
H	0.87304600	1.71709600	-4.09774500
C	1.30614000	4.26725500	-3.15966300
H	0.72179100	4.25858400	-4.08565000
H	1.04106100	5.16776500	-2.59640600
H	2.36223200	4.34544700	-3.43925700
C	-3.07400600	0.65581400	-2.59707500
H	-2.46224100	1.52100600	-2.31583600
C	-4.24089200	1.17570000	-3.43915900
H	-3.86553900	1.70600200	-4.32075300
H	-4.87712300	0.35700100	-3.79221700
H	-4.87037400	1.86520900	-2.86720100
C	-2.18323500	-0.29125700	-3.40573000
H	-1.32907300	-0.63607500	-2.81115800
H	-2.74046700	-1.17528600	-3.73486600
H	-1.79797800	0.21430900	-4.29800400
C	-3.08150800	0.22744600	2.49887600
H	-2.47462600	1.13123900	2.36947700
C	0.95784500	2.46500800	2.73428900
H	-0.11356600	2.41940600	2.50731200
C	1.35818400	1.10917500	3.32125200
H	1.15825800	0.29697900	2.61322200
H	2.42513900	1.08316200	3.56929900

H	0.79435400	0.90871500	4.23874700
C	1.16120300	3.59168500	3.74947200
H	0.55974500	3.40586100	4.64549900
H	2.20762600	3.66588100	4.06451800
H	0.86708100	4.56142500	3.33494400
C	-2.18698000	-0.83522200	3.14247000
H	-2.73549000	-1.76959100	3.30549500
H	-1.32042900	-1.06155800	2.51056800
H	-1.81883900	-0.48800300	4.11402500
C	-4.25378200	0.59304300	3.41183300
H	-4.88440600	-0.27704000	3.62407300
H	-3.88374000	0.97410700	4.36952400
H	-4.88771700	1.36241600	2.95912900
C	0.60032700	-2.56112100	-0.09738900
Br	-0.57468300	-3.77536300	0.51489000
H	-1.11038500	4.53682500	0.33781500
H	-3.54421700	3.17366200	0.19928800
C	2.90431200	-1.62894300	-1.14477700
C	3.84040900	-2.86429200	-1.13001900
C	3.54142800	-0.44685100	-0.39374500
C	4.23593100	-3.22213500	0.30280000
H	4.74455300	-2.52561800	-1.65796500
C	3.92750900	-0.82588000	1.03033000
H	2.86802400	0.41721900	-0.40208000
C	4.85209900	-2.03969400	1.04739900
H	4.93787200	-4.06405600	0.27855200
H	3.34667700	-3.57866300	0.84386500
H	4.40220300	0.03281700	1.51837300
H	3.01857500	-1.04689700	1.60884400
H	5.80686900	-1.77224100	0.57273300
H	5.08594300	-2.32590100	2.07895600
H	2.72518900	-1.33591100	-2.18901600
H	4.43691500	-0.15311400	-0.95638300
C	3.25785300	-4.05030900	-1.88574700
H	2.37906000	-4.46024600	-1.37390300
H	2.95847300	-3.77358500	-2.90309600
H	3.99657400	-4.85456400	-1.96102800

Cartesian coordinates of the optimized geometry for Int-**8a** at PBE0-D3BJ/6-31G*,def2-TZVP level of theory: (number of imaginary frequencies = 0):

Au	0.08772500	-0.53219200	-0.19825300
C	1.55023500	-1.83480200	-0.46557200
C	-1.32528800	0.90151000	0.11796000
C	-2.24911900	2.91881500	0.48333200
C	-3.25620900	2.00543200	0.44403500
N	-1.07910100	2.22043700	0.28076200
N	-2.66593000	0.78007800	0.22162000
C	-3.35711700	-0.47260600	0.10632000
C	-3.75462500	-0.89400600	-1.16945900
C	-3.56714100	-1.22206900	1.27180400
C	-4.39128700	-2.13321100	-1.25769000
C	-4.20708500	-2.45382300	1.12630900
C	-4.61481800	-2.90429200	-0.12367900
H	-4.71471700	-2.49872600	-2.22800500
H	-4.38791900	-3.06848000	2.00327300
H	-5.11325500	-3.86509100	-0.21438200
C	0.24039800	2.78813500	0.25360200
C	0.75695300	3.20718700	-0.97988300
C	0.95345100	2.86417600	1.45750600
C	2.04819200	3.73649000	-0.98059300
C	2.24119300	3.39990900	1.39994400
C	2.78155200	3.83551100	0.19574400
H	2.48668300	4.07116700	-1.91611600
H	2.82810900	3.47590200	2.31073600

H	3.78358800	4.25446600	0.17352400
C	-3.09149800	-0.75397000	2.63264400
H	-2.76998300	0.29027100	2.54367300
C	-1.87544700	-1.56924200	3.08215700
H	-2.13131700	-2.62987100	3.18707400
H	-1.06008700	-1.48686100	2.35342400
H	-1.50749500	-1.21135600	4.05014000
C	-4.20614100	-0.79881700	3.67878900
H	-3.84981800	-0.38365500	4.62744400
H	-5.07825700	-0.21991700	3.35818500
H	-4.53791100	-1.82421100	3.87404400
C	0.38994700	2.36123500	2.77111100
H	-0.65605200	2.07519400	2.61055700
C	0.40611500	3.44608400	3.84941100
H	-0.06268000	3.07494100	4.76696300
H	1.42814300	3.74921700	4.10109800
H	-0.13714000	4.33960900	3.52500500
C	1.14038000	1.10819800	3.23031700
H	1.08135100	0.31764000	2.47348500
H	2.20028800	1.32394100	3.40597800
H	0.71299200	0.72468700	4.16344200
C	-0.01553500	3.06506800	-2.27535200
H	-1.02969100	2.72451800	-2.03604900
C	-3.48719300	-0.07418400	-2.41550800
H	-3.08566600	0.89833400	-2.10781400
C	-2.42613700	-0.75162900	-3.28711400
H	-1.49518400	-0.90327900	-2.72784400
H	-2.77091300	-1.73063300	-3.63864400
H	-2.20216100	-0.13706700	-4.16603800
C	-4.76893900	0.19230800	-3.20682800
H	-4.55615200	0.83992800	-4.06394900
H	-5.20377500	-0.73495400	-3.59519600
H	-5.52639400	0.68304300	-2.58701200
C	0.62764300	1.99772900	-3.16524400
H	1.64858200	2.28043400	-3.44527100
H	0.67750600	1.03307300	-2.64652900
H	0.04782300	1.86474000	-4.08528900
C	-0.14414800	4.39971400	-3.01151600
H	0.83233400	4.78081800	-3.32933800
H	-0.75837400	4.27849300	-3.91006300
H	-0.61060000	5.16166100	-2.37854500
C	1.58222800	-3.28547600	-0.22889400
C	2.89101100	-3.81759300	-0.84883300
C	3.84034700	-2.65266900	-0.50557900
H	2.84430800	-1.39447700	-1.96036300
C	2.92870200	-1.46275300	-0.86194400
Br	3.49299900	0.30826700	-0.31248200
H	-4.32445000	2.11154600	0.55145800
H	-2.25631900	3.98696600	0.63476700
H	4.74942500	-2.64864500	-1.11337700
C	4.17380700	-2.67213700	0.98699900
C	1.73687400	-3.33354400	1.34274500
C	2.91758700	-2.50013000	1.84475100
H	3.12999100	-2.75963100	2.88685000
H	2.63516200	-1.43783500	1.84926200
H	1.88115300	-4.39761000	1.56689600
H	0.80244700	-3.01325600	1.81213700
H	4.66286900	-3.62821700	1.21066300
H	4.89335300	-1.88203400	1.22556800
H	0.66018200	-3.80891500	-0.49820400
H	3.21068400	-4.72436900	-0.32122000
C	2.77099400	-4.14004200	-2.33372200
H	3.74011900	-4.44323600	-2.74263600
H	2.07079800	-4.96703200	-2.49035700
H	2.40908900	-3.29402600	-2.92965500

Cartesian coordinates of the optimized geometry for Int-**8b** at PBE0-D3BJ/6-31G*,def2-TZVP level of theory: (number of imaginary frequencies = 0):

Au	0.29033100	0.21461300	-0.44323300
C	1.91008500	1.11074200	-1.14583000
C	-1.35818200	-0.75490500	0.27580600
C	-3.45854200	-1.27498600	0.88695900
C	-2.67952200	-2.35981300	1.13529300
N	-2.62672000	-0.30386900	0.36804500
N	-1.39948700	-2.01883900	0.74869800
C	-0.24765200	-2.87019200	0.83657700
C	0.01213700	-3.74429100	-0.22849800
C	0.56892000	-2.76839700	1.97171200
C	1.14406000	-4.55398600	-0.12399400
C	1.68772700	-3.60241800	2.02507000
C	1.97000700	-4.48789800	0.99229300
H	1.38189100	-5.24526000	-0.92707800
H	2.34424800	-3.55765000	2.88932000
H	2.84233800	-5.13202200	1.05661200
C	-3.02871100	1.01050300	-0.04626600
C	-3.28082600	1.22745400	-1.40731600
C	-3.11844700	2.01304400	0.93067300
C	-3.62346500	2.52634600	-1.78901600
C	-3.46298700	3.29220600	0.49345900
C	-3.70956400	3.54684500	-0.85108200
H	-3.83016400	2.73781500	-2.83434200
H	-3.53910100	4.10087600	1.21343800
H	-3.97787500	4.55035200	-1.16854100
C	0.28574800	-1.79429400	3.09762000
H	-0.65782900	-1.28061000	2.88092100
C	1.37869800	-0.72522400	3.17784800
H	2.35664500	-1.17150900	3.39128300
H	1.45872400	-0.16810700	2.23678800
H	1.15490200	-0.00818900	3.97500600
C	0.11650700	-2.51790200	4.43518700
H	-0.14394600	-1.80232700	5.22223600
H	-0.67512200	-3.27253200	4.38338800
H	1.04008100	-3.02239500	4.73930900
C	-2.80648000	1.74040000	2.38938300
H	-2.89680000	0.66098900	2.56066700
C	-3.78338600	2.43063500	3.34083100
H	-3.59049000	2.10985000	4.36978300
H	-3.67470100	3.52024900	3.31727200
H	-4.82291700	2.18916700	3.09652100
C	-1.36013000	2.13394700	2.70442600
H	-0.65040200	1.59443000	2.06869600
H	-1.20495400	3.20601100	2.53753300
H	-1.12177900	1.91363400	3.75133200
C	-3.20531500	0.12093900	-2.44023700
H	-2.93182100	-0.80998600	-1.93034700
C	-0.85664100	-3.78488700	-1.46972800
H	-1.77233500	-3.21800400	-1.26590300
C	-0.14361200	-3.09583200	-2.63739500
H	0.11148500	-2.05936000	-2.38666600
H	0.78361300	-3.62156800	-2.89409300
H	-0.78314600	-3.08618900	-3.52702100
C	-1.27557400	-5.20850500	-1.83815700
H	-1.96446600	-5.18968000	-2.68912000
H	-0.41622300	-5.82305200	-2.12722600
H	-1.77802600	-5.70648500	-1.00263200
C	-2.11948700	0.40576800	-3.47995000
H	-2.33708800	1.31851600	-4.04557400
H	-1.14112100	0.53102700	-3.00033500
H	-2.04773300	-0.42195300	-4.19400500
C	-4.56531700	-0.10451100	-3.10547300

H	-4.88950500	0.78140900	-3.66228800
H	-4.50986900	-0.93969100	-3.81198100
H	-5.33805300	-0.33380300	-2.36441400
C	2.90163300	0.57978800	-2.09312500
C	3.72054300	1.79032000	-2.57176300
H	3.17769000	2.35395600	-3.33987500
C	3.84080500	2.58843700	-1.26992800
H	1.74939300	3.10816600	-1.45451300
C	2.37737300	2.47443000	-0.80079500
Br	1.98684900	3.06248900	1.00106400
H	-2.91073500	-3.33038100	1.54563700
H	-4.51407600	-1.10266600	1.02887000
H	4.12246200	3.63291900	-1.42371600
C	4.80176300	1.89389400	-0.30567800
C	3.86413300	-0.31316400	-1.18733200
C	4.33813300	0.47648700	0.03596700
H	5.14479500	-0.07600300	0.53041700
H	3.51669500	0.52729800	0.76741100
H	5.79106200	1.86580800	-0.77876600
H	4.90527200	2.48104600	0.61268700
H	2.47905400	-0.07057500	-2.86413000
H	4.69055800	1.49916200	-2.98617100
H	4.71931700	-0.47276300	-1.85925900
C	3.27457800	-1.66407500	-0.82220800
H	2.45578400	-1.58232300	-0.10059400
H	2.89768500	-2.19360600	-1.70309000
H	4.05014400	-2.28803800	-0.36448900

Cartesian coordinates of the optimized geometry for Int-**8c** at PBE0-D3BJ/6-31G*,def2-TZVP level of theory: (number of imaginary frequencies = 0):

Au	0.12816100	0.29947100	-0.45133500
C	1.74780700	1.24923300	-1.11987000
C	-1.40552400	-0.80709500	0.29420000
C	-3.38847300	-1.56249200	1.03594700
C	-2.47594000	-2.55129100	1.23039400
N	-2.70947800	-0.50656900	0.46418900
N	-1.27136400	-2.06621400	0.76758500
C	-0.01491900	-2.76059800	0.78257000
C	0.31822900	-3.55380600	-0.32442500
C	0.83567500	-2.56931500	1.88063000
C	1.56423800	-4.18330500	-0.30393500
C	2.06985100	-3.22168200	1.84960900
C	2.42980800	-4.02155600	0.77171200
H	1.86063200	-4.80650000	-1.14267400
H	2.75630800	-3.10187200	2.68293700
H	3.39282500	-4.52430200	0.76995800
C	-3.28385800	0.75733500	0.10016200
C	-3.76830100	0.91372000	-1.20525900
C	-3.30030300	1.77660600	1.06162500
C	-4.28994500	2.16382200	-1.54200600
C	-3.83299300	3.00649000	0.67191600
C	-4.32231600	3.19816500	-0.61450900
H	-4.67480800	2.32904200	-2.54417400
H	-3.86286000	3.82490500	1.38538700
H	-4.73394900	4.16290600	-0.89672900
C	0.46467100	-1.69006800	3.05755700
H	-0.54349300	-1.29528400	2.88823400
C	1.40994400	-0.49104900	3.16668000
H	2.44142100	-0.81253700	3.35078600
H	1.40043800	0.10531400	2.24717200
H	1.10838200	0.15719400	3.99673400
C	0.43052000	-2.49235700	4.36030600
H	0.10967400	-1.85454900	5.19077200
H	-0.26221800	-3.33746200	4.29069400

H	1.41959300	-2.89115800	4.61101900
C	-2.74079500	1.58597000	2.45698700
H	-2.48344600	0.52777200	2.58238300
C	-3.76947800	1.93430500	3.53416300
H	-3.36110200	1.72507600	4.52859800
H	-4.03932100	2.99551400	3.50624700
H	-4.68883500	1.35223500	3.41238600
C	-1.45240900	2.39369900	2.63480200
H	-0.70177900	2.11392600	1.88731800
H	-1.64343400	3.46792200	2.53176100
H	-1.02710700	2.22145100	3.62998700
C	-3.70793100	-0.19935700	-2.23169800
H	-3.37826300	-1.11475100	-1.72655500
C	-0.59651500	-3.69730100	-1.52384700
H	-1.55615700	-3.22624800	-1.28210600
C	-0.01360200	-2.95426900	-2.72955100
H	0.13759900	-1.89262800	-2.50086000
H	0.95281800	-3.37931800	-3.02414800
H	-0.68966600	-3.02585600	-3.58862800
C	-0.87763800	-5.16335500	-1.85677600
H	-1.59328800	-5.23222700	-2.68277400
H	0.03195300	-5.68971800	-2.16572800
H	-1.29631800	-5.69539700	-0.99638100
C	-2.67332700	0.12400000	-3.31313500
H	-2.95081600	1.02900400	-3.86530900
H	-1.68272000	0.28803500	-2.87252600
H	-2.59807800	-0.69996200	-4.03143000
C	-5.08032800	-0.48546900	-2.84358800
H	-5.45476300	0.37145800	-3.41385800
H	-5.01792300	-1.33568900	-3.53110500
H	-5.82007400	-0.72284700	-2.07213700
C	3.27937700	2.92326900	-2.03665700
H	1.11517400	3.31861900	-1.57719600
C	1.97211100	2.71213700	-1.28880300
Br	2.14598800	2.99501600	0.67218700
H	-2.57022100	-3.54100500	1.64928500
H	-4.44490200	-1.51069000	1.24840600
H	3.02035700	3.06371200	-3.09483900
H	3.81289200	3.82129700	-1.71344900
C	2.96034200	0.57747200	-1.61537400
C	3.37817600	-0.77667500	-1.05109500
C	4.07443100	1.62105300	-1.84198700
H	2.48915700	0.39500700	-2.62059300
C	4.32278500	-0.59229200	0.13288600
H	3.89723300	-1.33788100	-1.83783200
H	2.49382100	-1.35846600	-0.76840900
C	5.09300400	1.65506500	-0.69349800
H	4.61511900	1.36671000	-2.76080500
C	5.52769300	0.25606900	-0.26465400
H	4.64019800	-1.57699200	0.49320500
H	3.78421900	-0.11022000	0.96116200
H	5.95746200	2.24856600	-1.01332600
H	4.66595800	2.16979200	0.17290600
H	6.23385300	0.33323400	0.56927800
H	6.06569700	-0.23991700	-1.08544500

Cartesian coordinates of the optimized geometry for Int-**8d** at PBE0-D3BJ/6-31G*,def2-TZVP level of theory: (number of imaginary frequencies = 0):

Au	0.19728700	-0.34986900	-0.32713700
C	1.94875700	-1.13421400	-0.81884000
C	-1.52916700	0.56973100	0.24263900
C	-2.93275000	2.17024900	0.96757900
C	-3.66128000	1.03726100	0.78118800
N	-1.63338400	1.86033600	0.62954400

N	-2.78036200	0.07229300	0.34141300
C	-3.11834500	-1.28544900	0.02179600
C	-3.42763300	-1.59541200	-1.30896500
C	-3.08892800	-2.23433300	1.05313600
C	-3.71382300	-2.93084300	-1.59859500
C	-3.38191000	-3.55442600	0.70820000
C	-3.69054100	-3.89904600	-0.60260100
H	-3.95783300	-3.21477000	-2.61824700
H	-3.36794100	-4.32283600	1.47548600
H	-3.91776900	-4.93219200	-0.84918300
C	-0.51678600	2.76242600	0.68748000
C	-0.23816500	3.54540500	-0.44109600
C	0.25278700	2.79032900	1.85813600
C	0.86063600	4.40200700	-0.36401700
C	1.34024300	3.66544900	1.88214500
C	1.63871400	4.46582900	0.78601600
H	1.11367400	5.02355600	-1.21776700
H	1.96156100	3.71911100	2.77153500
H	2.48667700	5.14360600	0.82701200
C	-2.71318000	-1.86970900	2.47571700
H	-2.71999600	-0.77694000	2.56288800
C	-1.29056700	-2.34327900	2.78857900
H	-1.21527400	-3.43399000	2.70914900
H	-0.56987200	-1.90055800	2.09082700
H	-1.00318800	-2.05593800	3.80618100
C	-3.71401800	-2.41227300	3.49658500
H	-3.45724600	-2.05771700	4.50035500
H	-4.73380100	-2.08561100	3.26902500
H	-3.71119200	-3.50712700	3.52639200
C	-0.04053500	1.90166800	3.04980200
H	-0.95295500	1.33182600	2.83924100
C	-0.29554000	2.72272100	4.31530900
H	-0.55624400	2.06328800	5.15004900
H	0.59214100	3.29337500	4.60905000
H	-1.11632800	3.43294700	4.17127500
C	1.08988900	0.89065300	3.25988500
H	1.24610600	0.28460600	2.35967000
H	2.03517300	1.39416600	3.49089400
H	0.85242600	0.21904200	4.09235100
C	-1.04703400	3.44208100	-1.71803100
H	-1.93914200	2.83904200	-1.51221000
C	-3.43431300	-0.55131200	-2.40701200
H	-3.27106200	0.43102200	-1.94886200
C	-2.28597000	-0.79356000	-3.39000000
H	-1.31888200	-0.79169200	-2.87304600
H	-2.39554400	-1.75951200	-3.89570800
H	-2.26660500	-0.01158400	-4.15704100
C	-4.78224800	-0.49954700	-3.12891300
H	-4.78155600	0.30258100	-3.87457300
H	-4.99449900	-1.43732400	-3.65366400
H	-5.60345000	-0.31500500	-2.42866700
C	-0.23947100	2.71262000	-2.79596300
H	0.67386400	3.26531700	-3.04329000
H	0.05590000	1.71308400	-2.45594800
H	-0.83082200	2.60434400	-3.71201000
C	-1.52519400	4.80832500	-2.21113000
H	-0.68677400	5.45043400	-2.50158700
H	-2.16554300	4.68838800	-3.09135400
H	-2.09830400	5.33371900	-1.44026500
H	2.84036700	-0.14296900	-2.33918400
C	3.10536200	-0.32412100	-1.28062800
Br	3.21188600	1.43711200	-0.47622300
H	-4.71109800	0.82966200	0.91803800
H	-3.21434500	3.15583200	1.30409900
C	2.42642600	-2.51282100	-0.71839300
C	2.58562700	-2.60205100	0.85378800

C	3.79439600	-2.59358400	-1.42974100
H	1.69572600	-3.27575300	-1.00358100
H	1.58658900	-2.64747200	1.29483600
C	3.41309200	-3.83764200	1.20819400
H	3.49684000	-3.88345900	2.30039200
H	2.86724200	-4.73541700	0.88822300
C	4.67552500	-3.74678100	-0.95669400
H	4.24345500	-4.68747700	-1.32333300
H	5.66548900	-3.66122900	-1.41931200
C	4.79227200	-3.81471900	0.56228300
H	5.36327500	-2.95828000	0.94570900
H	5.35414200	-4.70973400	0.84961400
H	3.07413700	-1.70530300	1.25818400
C	4.36311000	-1.18507400	-1.20575900
H	4.82085900	-1.08499600	-0.21644400
H	5.10891800	-0.89391100	-1.94861500
H	3.60061400	-2.71720500	-2.50413300

Cartesian coordinates of the optimized geometry for Int-**9** at PBE0-D3BJ/6-31G*,def2-TZVP level of theory: (number of imaginary frequencies = 0):

Au	0.01446700	0.49669400	-0.23732300
C	-1.20686500	2.21499000	-0.88855200
C	0.73344800	-1.35214800	0.10281500
C	2.18759200	-3.02840100	0.43169900
C	0.93767300	-3.56108000	0.43076600
N	2.03661800	-1.67218500	0.22620200
N	0.06117400	-2.51236000	0.22971400
C	-1.36968300	-2.61209400	0.17252200
C	-2.08515200	-2.51874500	1.37461900
C	-1.97560100	-2.77206900	-1.08137100
C	-3.47574900	-2.60634800	1.29170800
C	-3.36997700	-2.84265400	-1.10701900
C	-4.11133100	-2.76552200	0.06559100
H	-4.06909200	-2.54551000	2.19939800
H	-3.88025800	-2.96456300	-2.05806400
H	-5.19483900	-2.82823500	0.02460000
C	3.08332600	-0.69113000	0.15944400
C	3.45790100	-0.04374200	1.34503900
C	3.62901700	-0.39012800	-1.09617300
C	4.43302100	0.94973200	1.24255400
C	4.59971900	0.61185600	-1.14054800
C	4.99698700	1.27435800	0.01468100
H	4.75357900	1.47591600	2.13718000
H	5.04794000	0.87803300	-2.09343700
H	5.75503400	2.05016800	-0.04263200
C	-1.17848100	-2.84403700	-2.36811500
H	-0.11275500	-2.86741800	-2.11248400
C	-1.41615200	-1.59492600	-3.22110200
H	-2.47057000	-1.50482200	-3.50607600
H	-1.13379800	-0.68710300	-2.67444000
H	-0.82040900	-1.63705200	-4.13930900
C	-1.48739200	-4.11964300	-3.15433700
H	-0.85151800	-4.17867000	-4.04389600
H	-1.31157100	-5.01417000	-2.54819400
H	-2.52918900	-4.14340300	-3.49164200
C	3.18201400	-1.08499300	-2.36638000
H	2.46879000	-1.87228800	-2.09557600
C	4.35555200	-1.75835900	-3.08088500
H	4.00072600	-2.30219800	-3.96271700
H	5.09369200	-1.02391300	-3.42075800
H	4.86873100	-2.46856700	-2.42434700
C	2.45420400	-0.10717500	-3.29285600
H	1.59112200	0.34598900	-2.79116400
H	3.11795500	0.70290800	-3.61453800

H	2.09640600	-0.62438100	-4.18989000
C	2.83333300	-0.37163400	2.68634400
H	2.13997000	-1.20952700	2.54857900
C	-1.40325600	-2.28718800	2.70787800
H	-0.32171400	-2.39111500	2.56307400
C	-1.66362600	-0.85960300	3.19848400
H	-1.33933600	-0.11928100	2.45779000
H	-2.72999600	-0.69677100	3.39066400
H	-1.12131500	-0.67037600	4.13131800
C	-1.82325700	-3.31915000	3.75548700
H	-1.26202400	-3.16413900	4.68286300
H	-2.88785500	-3.23762800	3.99968900
H	-1.63588200	-4.34068600	3.40907000
C	2.01916800	0.81244400	3.21404500
H	2.65450300	1.68994400	3.37782300
H	1.22904900	1.09732400	2.50984300
H	1.54781700	0.55666200	4.16919900
C	3.88939900	-0.81534400	3.70102300
H	4.59784800	-0.00950500	3.92132100
H	3.41218900	-1.10296400	4.64381600
H	4.46263800	-1.67210900	3.33206200
C	-0.16317600	2.65167400	-0.37803600
Br	1.16471900	3.69067900	0.24210700
H	0.58990000	-4.57487300	0.55348200
H	3.15926300	-3.47963400	0.55825500
C	-2.51926300	2.02434800	-1.52763500
C	-3.14575900	3.40949100	-1.78481700
C	-3.45498800	1.15315500	-0.67172900
C	-3.54280500	4.09600300	-0.48264400
H	-4.03668400	3.24183900	-2.40420100
H	-2.45998900	4.03127100	-2.37060800
C	-3.85614600	1.83472600	0.63616300
H	-2.99263400	0.17721700	-0.47564400
C	-4.46279300	3.21092000	0.35367400
H	-4.03299200	5.04954700	-0.70799000
H	-2.64060300	4.34167700	0.09516700
H	-2.94226300	1.98104500	1.23544900
H	-5.41490400	3.07079400	-0.18025600
H	-4.70820600	3.70823000	1.29967700
H	-2.34198400	1.52136900	-2.48750500
H	-4.35475400	0.95985500	-1.27183200
C	-4.81294100	0.95784200	1.43224200
H	-4.37305600	-0.02363700	1.63656600
H	-5.07169900	1.42460200	2.38888200
H	-5.74564700	0.79685200	0.87730800

Cartesian coordinates of the optimized geometry for Int-**10a** at PBE0-D3BJ/6-31G*,def2-TZVP level of theory: (number of imaginary frequencies = 0):

Au	0.04876900	-0.54189100	-0.27235800
C	1.53665700	-1.78468700	-0.65892300
C	-1.38933100	0.83295500	0.16640300
C	-2.34903200	2.79473800	0.70203800
C	-3.33993400	1.87107000	0.57934400
N	-1.16642800	2.13680300	0.44456300
N	-2.72783600	0.68028200	0.25302500
C	-3.39605600	-0.56977100	0.02729100
C	-3.79519400	-0.87998200	-1.27951500
C	-3.58306500	-1.42665900	1.12023000
C	-4.40899200	-2.11796300	-1.47737600
C	-4.20128500	-2.65197600	0.86618600
C	-4.60975000	-2.99383900	-0.41745300
H	-4.73230600	-2.39964600	-2.47528100
H	-4.36442400	-3.34741400	1.68442500
H	-5.09076600	-3.95172900	-0.59313800

C	0.14315300	2.72683100	0.47554000
C	0.66005700	3.25923300	-0.71320300
C	0.84681100	2.71179900	1.68733100
C	1.94165100	3.80895200	-0.65808800
C	2.12527600	3.27219900	1.68617400
C	2.66554700	3.81899900	0.52813200
H	2.37996800	4.23057300	-1.55789500
H	2.70488000	3.27979300	2.60472000
H	3.66006600	4.25552300	0.54998000
C	-3.11117700	-1.07471700	2.51684300
H	-2.78255700	-0.02890500	2.51517300
C	-1.90363200	-1.93174900	2.90705400
H	-2.16595400	-2.99577400	2.92562100
H	-1.08223700	-1.79681700	2.19315900
H	-1.54052900	-1.65491700	3.90300100
C	-4.23450800	-1.19719200	3.54767900
H	-3.88212100	-0.86903200	4.53128300
H	-5.09814300	-0.58364300	3.27154900
H	-4.57825400	-2.23205300	3.65037300
C	0.28359200	2.08739100	2.94789100
H	-0.75904000	1.80739900	2.75746700
C	0.28491300	3.07000900	4.12014000
H	-0.18623500	2.61348000	4.99706200
H	1.30286400	3.35661000	4.40538100
H	-0.26368800	3.98517300	3.87468600
C	1.04443300	0.80488700	3.29499400
H	0.99523900	0.08554800	2.46948900
H	2.10168400	1.01370600	3.49358100
H	0.61769300	0.33461400	4.18786500
C	-0.10177100	3.21827100	-2.02211400
H	-1.10906000	2.83448100	-1.82281900
C	-3.54985700	0.05520500	-2.44635600
H	-3.17418800	1.00633300	-2.05142100
C	-2.47099100	-0.51349700	-3.37224300
H	-1.53598300	-0.68883700	-2.82687500
H	-2.78957900	-1.46659900	-3.80928800
H	-2.26407400	0.18207000	-4.19308200
C	-4.83749100	0.35831500	-3.21447800
H	-4.64122500	1.08732100	-4.00779400
H	-5.24646800	-0.54058200	-3.68823800
H	-5.60880100	0.76950400	-2.55514500
C	0.57004300	2.25174800	-3.00150500
H	1.58632400	2.58033200	-3.24607100
H	0.63739700	1.24430400	-2.57425900
H	-0.00116300	2.19259000	-3.93452800
C	-0.25660800	4.61181800	-2.63365000
H	0.71297500	5.04227600	-2.90640300
H	-0.86195300	4.56017100	-3.54483400
H	-0.74465300	5.30163900	-1.93739100
C	1.59288200	-3.25438700	-0.59372000
C	2.92797500	-3.66332200	-1.22889300
H	2.85883300	-3.66319800	-2.32407100
C	3.86602100	-2.54679700	-0.74039700
H	2.84371700	-1.20545600	-2.08409800
C	2.91188300	-1.35178600	-0.98806400
Br	3.43219800	0.37404000	-0.28081500
H	-4.41010100	1.94905500	0.69156600
H	-2.37530100	3.84536400	0.94582400
C	4.12080300	-2.72620400	0.76215700
C	1.70226600	-3.48992900	0.96155200
C	2.83611200	-2.67976500	1.59004000
H	3.02619100	-3.04032600	2.60612600
H	2.51950200	-1.63183200	1.69523100
H	1.88147200	-4.56721300	1.06575500
H	0.74365700	-3.25854800	1.43441000
H	4.62607100	-3.69140800	0.89611000

H	4.81412800	-1.95422900	1.11431200
H	0.69212100	-3.75763100	-0.95463400
C	5.16357100	-2.42903700	-1.51773000
H	5.72418300	-1.54225300	-1.20374700
H	5.79480700	-3.30656800	-1.34437400
H	4.97780600	-2.35407300	-2.59549400
H	3.25752600	-4.65774200	-0.91144900

Cartesian coordinates of the optimized geometry for Int-**10b** at PBE0-D3BJ/6-31G*,def2-TZVP level of theory: (number of imaginary frequencies = 0):

Au	0.03326800	-0.43715100	-0.50856600
C	1.42936300	-1.60125400	-1.28721000
C	-1.29465600	0.82654600	0.38106900
C	-2.11968200	2.63269300	1.43627600
C	-3.14710100	1.76208400	1.24514600
N	-0.99824000	2.03901600	0.89970800
N	-2.61695400	0.66542000	0.60053200
C	-3.34391600	-0.50782600	0.20649800
C	-3.89733400	-0.54295300	-1.08017800
C	-3.42998200	-1.56908200	1.11790000
C	-4.56276300	-1.71243100	-1.45194400
C	-4.10567900	-2.71455700	0.69449600
C	-4.66539100	-2.78585200	-0.57565700
H	-5.00488600	-1.78289900	-2.44151700
H	-4.19427000	-3.56188800	1.36820500
H	-5.18856200	-3.68619200	-0.88463800
C	0.32451600	2.59982800	0.89447800
C	0.71454100	3.37040400	-0.20900700
C	1.16598100	2.32014900	1.97982400
C	2.01234600	3.88357900	-0.19812400
C	2.45406500	2.85640700	1.93904800
C	2.87210700	3.63274800	0.86452100
H	2.35516100	4.48412100	-1.03555800
H	3.13856800	2.66253600	2.75978000
H	3.87703500	4.04521300	0.85483400
C	-2.79438200	-1.51135800	2.49281900
H	-2.45069200	-0.48559200	2.66995700
C	-1.56384100	-2.42094400	2.55375000
H	-1.83889600	-3.46710300	2.37692100
H	-0.82695900	-2.13017800	1.79572500
H	-1.08611900	-2.35749400	3.53782900
C	-3.79256200	-1.85422900	3.59980000
H	-3.32415900	-1.73140700	4.58206300
H	-4.67359900	-1.20577300	3.55753600
H	-4.13663400	-2.89155600	3.52768500
C	0.73484700	1.44760700	3.14148900
H	-0.33232200	1.22427600	3.02752700
C	0.91088900	2.15884200	4.48418300
H	0.53068700	1.53150600	5.29739700
H	1.96518100	2.36780000	4.69516700
H	0.37070300	3.11091300	4.50549000
C	1.48491700	0.11308100	3.11509100
H	1.30794200	-0.41445000	2.17110000
H	2.56561300	0.26417500	3.21591700
H	1.15365400	-0.53073200	3.93754300
C	-0.19904200	3.61699500	-1.39209200
H	-1.19141400	3.21944500	-1.14948700
C	-3.76399600	0.61125400	-2.05288300
H	-3.31533000	1.45852300	-1.52135500
C	-2.82143300	0.24067100	-3.20120700
H	-1.83374700	-0.04873100	-2.82278700
H	-3.21874300	-0.59955900	-3.78179500
H	-2.69272900	1.09000500	-3.88119800
C	-5.12472900	1.07234800	-2.57813700

H	-5.00305300	1.94764500	-3.22494900
H	-5.61464700	0.29178200	-3.17011300
H	-5.79845100	1.34339800	-1.75877200
C	0.30815100	2.86167200	-2.62368000
H	1.30155500	3.21507900	-2.92181600
H	0.38237200	1.78675000	-2.42112700
H	-0.37211500	3.00678300	-3.47013600
C	-0.36401900	5.10952200	-1.68339900
H	0.58335200	5.56876400	-1.98561900
H	-1.07723900	5.25857200	-2.50097200
H	-0.73252300	5.64974400	-0.80524500
C	1.44718300	-3.06164800	-1.47429700
C	2.70757500	-3.38022300	-2.29262200
H	2.54265700	-3.19315500	-3.36034100
C	3.69806900	-2.37598700	-1.69310200
H	2.62295600	-0.80985200	-2.72116700
C	2.78195800	-1.13524400	-1.67584600
Br	3.41456600	0.43783100	-0.74202400
H	-4.19288500	1.81994300	1.50386100
H	-2.08283700	3.60683500	1.89848000
H	4.57757200	-2.20521900	-2.31856800
C	4.09707200	-2.80018700	-0.27963600
C	1.69705600	-3.55417200	0.00280800
C	2.89352000	-2.86474200	0.66646700
H	2.59990000	-1.82447500	0.88398500
H	4.57664900	-3.78616500	-0.34272400
H	4.84304800	-2.10878900	0.12771600
H	0.50138100	-3.48468200	-1.82268300
H	3.02987800	-4.41917300	-2.17513600
H	1.87194100	-4.63510100	-0.08332400
H	0.78917500	-3.40486900	0.59493000
C	3.23695500	-3.52644300	1.99239800
H	3.52317000	-4.57491700	1.84500000
H	4.07415000	-3.01543200	2.47926800
H	2.38328600	-3.50246800	2.67878400

Cartesian coordinates of the optimized geometry for Int-**11** at PBE0-D3BJ/6-31G*,def2-TZVP level of theory: (number of imaginary frequencies = 0):

Au	0.03232000	0.51620400	-0.01961600
C	-1.60003900	2.00186600	-0.12666400
C	1.05297800	-1.21602500	-0.06280600
C	2.74705400	-2.68306700	-0.15479100
C	1.59143500	-3.39299400	-0.07736100
N	2.39058200	-1.34936600	-0.14625700
N	0.56418500	-2.47065500	-0.01935500
C	-0.83497600	-2.77604400	0.08183600
C	-1.38539000	-2.93857700	1.36109100
C	-1.58237200	-2.86945900	-1.10086300
C	-2.74930900	-3.22824500	1.43286600
C	-2.94229000	-3.15820700	-0.97079700
C	-3.51772200	-3.34395700	0.28057300
H	-3.21488700	-3.36342800	2.40469000
H	-3.55685500	-3.24319000	-1.86239500
H	-4.57594200	-3.57600200	0.35835200
C	3.27866700	-0.22230000	-0.20585100
C	3.72097100	0.33528600	1.00178400
C	3.60563300	0.29542100	-1.46680700
C	4.53514500	1.46585400	0.91589200
C	4.42127100	1.42768000	-1.49390100
C	4.88089100	2.00614000	-0.31680800
H	4.90146500	1.92908400	1.82755900
H	4.69730900	1.86323500	-2.44982100
H	5.51613500	2.88609400	-0.36102100
C	-0.97362200	-2.64667200	-2.47030200

H	0.10674500	-2.50704600	-2.34885600
C	-1.53076400	-1.36983500	-3.10596200
H	-2.61421500	-1.44150100	-3.25503600
H	-1.33259500	-0.49613900	-2.47347700
H	-1.06763000	-1.19308300	-4.08265900
C	-1.17913800	-3.85692000	-3.38343400
H	-0.68006200	-3.69489200	-4.34458500
H	-0.77077500	-4.76857000	-2.93542200
H	-2.24098200	-4.03138500	-3.58795400
C	3.08257800	-0.30830100	-2.75445300
H	2.54355400	-1.23055800	-2.50772800
C	4.22100400	-0.68609600	-3.70417600
H	3.82107400	-1.17546100	-4.59851400
H	4.77987500	0.19644800	-4.03355400
H	4.92910100	-1.37123600	-3.22676900
C	2.08645100	0.63836800	-3.42995800
H	1.24930900	0.87539300	-2.76290200
H	2.56675800	1.58163700	-3.71272000
H	1.68080200	0.18188400	-4.33955300
C	3.32939500	-0.22824400	2.35287800
H	2.73890500	-1.13738300	2.18977700
C	-0.56809600	-2.76529700	2.62504400
H	0.48578600	-2.66147300	2.34206600
C	-0.97659800	-1.47881400	3.34909400
H	-0.85485700	-0.60305000	2.70126400
H	-2.02530500	-1.52029600	3.66454100
H	-0.36144100	-1.33052600	4.24329800
C	-0.67509500	-3.98079900	3.54723200
H	-0.02288700	-3.85235800	4.41737300
H	-1.69604100	-4.11714300	3.91975600
H	-0.38078900	-4.90100400	3.03214600
C	2.44551100	0.75893600	3.11960200
H	2.97929100	1.69341100	3.32498400
H	1.54174300	1.00729100	2.55102500
H	2.13715400	0.33049600	4.07945000
C	4.55961400	-0.62420200	3.17221200
H	5.18028500	0.24628400	3.41060200
H	4.25346900	-1.08118500	4.11919200
H	5.18502300	-1.34233400	2.63190400
C	-0.54758800	2.60113400	0.14431900
Br	0.70429400	3.83396100	0.52031300
H	1.40186200	-4.45479100	-0.05728600
H	3.77789200	-2.99540700	-0.21444400
C	-2.97362400	1.56756100	-0.40638600
C	-3.40930500	0.40233100	0.49650100
C	-3.92181600	2.77183500	-0.26432400
C	-4.85148000	0.01287900	0.19514300
H	-3.31293100	0.71449500	1.54528600
H	-2.74350100	-0.45583500	0.35076500
C	-5.36666100	2.37192300	-0.57144700
H	-3.85774600	3.15331900	0.76438700
C	-5.79533000	1.20477700	0.31859600
H	-5.15664600	-0.79315300	0.87168600
H	-5.40331700	2.03098900	-1.61847900
H	-6.81926200	0.90455200	0.06607900
H	-5.81881700	1.54630600	1.36437800
H	-3.00384700	1.22076600	-1.45040300
H	-3.60042100	3.58140800	-0.93006000
C	-6.29781000	3.56824700	-0.42184500
H	-6.00185000	4.39223300	-1.08042300
H	-7.32895600	3.29423600	-0.66906200
H	-6.29015600	3.94211700	0.60930200
H	-4.90521100	-0.39895100	-0.82309400

Cartesian coordinates of the optimized geometry for Int-**12** at PBE0-D3BJ/6-31G*,def2-TZVP level of theory: (number of imaginary frequencies = 0):

Au	0.06695400	-0.50520100	-0.33689100
C	1.44355300	-1.79981000	-0.91872400
C	-1.27773400	0.91797400	0.22646900
C	-2.14020400	2.90722800	0.82214900
C	-3.16578100	2.01623300	0.75816800
N	-0.99586600	2.21221800	0.49634100
N	-2.61327800	0.80838100	0.39025100
C	-3.33422100	-0.41675800	0.19243700
C	-3.88677200	-0.66110500	-1.07242900
C	-3.41727800	-1.31745700	1.26245900
C	-4.55319400	-1.87446800	-1.24910300
C	-4.09617300	-2.51530500	1.03129500
C	-4.65811900	-2.79062700	-0.20911500
H	-4.99390600	-2.10598700	-2.21421300
H	-4.18576200	-3.24099200	1.83467000
H	-5.18388900	-3.72784000	-0.36727300
C	0.33251500	2.75751500	0.44510700
C	0.83405100	3.17142100	-0.79596600
C	1.06925000	2.81513600	1.63697300
C	2.14042500	3.66296600	-0.82063100
C	2.37214600	3.30759400	1.55362200
C	2.90179200	3.72863500	0.33898700
H	2.56653200	3.99622400	-1.76250200
H	2.97992900	3.36472500	2.45135700
H	3.91633000	4.11472900	0.29770100
C	-2.78778100	-1.04016000	2.61280200
H	-2.36506100	-0.02899400	2.59567300
C	-1.63471300	-2.01110000	2.88048600
H	-1.99016300	-3.04700700	2.92122900
H	-0.87664800	-1.94323900	2.09088800
H	-1.15335300	-1.78127900	3.83748400
C	-3.82413100	-1.08417200	3.73741600
H	-3.35724800	-0.82607100	4.69393400
H	-4.64099500	-0.37860200	3.55415900
H	-4.26230200	-2.08267000	3.84192600
C	0.50770800	2.32105300	2.95571000
H	-0.58217100	2.25028000	2.85830900
C	0.79608700	3.27882200	4.11180800
H	0.28236400	2.93779800	5.01666600
H	1.86515700	3.32621700	4.34503100
H	0.45488700	4.29446800	3.88696200
C	1.03160800	0.91447900	3.25946800
H	0.75687500	0.21128100	2.46622100
H	2.12454000	0.91416300	3.34170000
H	0.61803200	0.54485400	4.20467300
C	0.02295700	3.09688600	-2.07356400
H	-0.96597100	2.69033800	-1.83256700
C	-3.73282800	0.31355400	-2.22299700
H	-3.35521600	1.26132800	-1.82187800
C	-2.69507400	-0.20537300	-3.22314000
H	-1.72607900	-0.37015700	-2.73708100
H	-3.01744600	-1.15579300	-3.66354900
H	-2.55275200	0.51407700	-4.03729200
C	-5.06509600	0.61184800	-2.91175200
H	-4.92730300	1.37920600	-3.68072000
H	-5.47443200	-0.27562400	-3.40616400
H	-5.81264700	0.97316900	-2.19814900
C	0.67300400	2.14527200	-3.08057000
H	1.66538800	2.49886800	-3.38115900
H	0.78906400	1.14321400	-2.65075600
H	0.05651200	2.06350000	-3.98237800
C	-0.18757100	4.48691300	-2.67830400
H	0.76388600	4.94293100	-2.97296200
H	-0.81611700	4.42169100	-3.57291500

H	-0.67447400	5.16157200	-1.96648600
C	1.36110700	-3.25628400	-1.12157400
C	2.61341400	-3.65557600	-1.91937900
H	2.49189200	-3.43312600	-2.98622600
C	3.65565600	-2.74259800	-1.26891500
H	2.69104700	-1.15203800	-2.33733200
C	2.83346300	-1.43986700	-1.27606600
Br	3.58514300	0.14090600	-0.46023900
H	-4.22397000	2.12782500	0.93631700
H	-2.11844500	3.95754800	1.06793900
H	4.56391700	-2.62387400	-1.86505800
C	4.00631300	-3.25271100	0.12943200
C	1.51878800	-3.87247700	0.32157200
C	2.79701300	-3.46154600	1.07272600
H	4.53146200	-4.20479000	-0.01142200
H	4.72272500	-2.57174800	0.60269700
H	0.39460800	-3.58760500	-1.51166200
H	2.84959700	-4.71894800	-1.81620900
H	1.50879200	-4.95250800	0.13296700
H	0.63172400	-3.63484500	0.91713800
C	2.54889300	-2.26427900	1.98777200
H	2.26982000	-1.35739300	1.43990500
H	1.74159900	-2.48023800	2.69630600
H	3.44934600	-2.02218900	2.56181700
H	3.04651400	-4.30253700	1.73114500

Cartesian coordinates of the optimized geometry for Int-**13** at PBE0-D3BJ/6-31G*,def2-TZVP level of theory: (number of imaginary frequencies = 0):

Au	0.12212100	0.57444400	-0.01033700
C	-1.34326700	2.22305600	-0.14470900
C	0.94356400	-1.26056900	-0.05697700
C	2.45939100	-2.90907600	-0.18143200
C	1.23578600	-3.48410200	-0.04818700
N	2.25393000	-1.54413400	-0.18609300
N	0.32038500	-2.45206200	0.02808100
C	-1.09919700	-2.59953400	0.18385700
C	-1.62081600	-2.64326100	1.48443100
C	-1.89115600	-2.66793900	-0.97136800
C	-3.00450800	-2.78359300	1.60865400
C	-3.26847700	-2.80565600	-0.78913700
C	-3.81832300	-2.87167900	0.48572700
H	-3.44892900	-2.82430400	2.59883000
H	-3.91817200	-2.86500800	-1.65747500
H	-4.89173600	-2.98765500	0.60439200
C	3.25977600	-0.52518200	-0.29674400
C	3.81846100	-0.01761500	0.88446500
C	3.58453800	-0.05258900	-1.57577800
C	4.74986100	1.01344400	0.75165600
C	4.52175700	0.97902900	-1.65001900
C	5.09752200	1.50619500	-0.50032300
H	5.20745800	1.43603200	1.64154100
H	4.80156500	1.37612500	-2.62150100
H	5.82538700	2.30834400	-0.58118800
C	-1.30767700	-2.56298600	-2.36581500
H	-0.21463200	-2.57214800	-2.28375600
C	-1.70642800	-1.23321400	-3.01267500
H	-2.79489000	-1.15544700	-3.11637000
H	-1.36126700	-0.38378200	-2.41106200
H	-1.26502200	-1.14295000	-4.01108400
C	-1.70999500	-3.74963600	-3.24321400
H	-1.22045600	-3.67997900	-4.22022500
H	-1.42294100	-4.70102800	-2.78392800
H	-2.79096600	-3.77484300	-3.41799500
C	2.94025500	-0.59882000	-2.83375600

H	2.29384000	-1.43855700	-2.55291000
C	3.98614800	-1.13569400	-3.81305100
H	3.49563700	-1.57710600	-4.68704500
H	4.64640200	-0.33897900	-4.17232500
H	4.61222400	-1.90388000	-3.34753000
C	2.05328900	0.46071900	-3.49324400
H	1.28002400	0.81637200	-2.80223200
H	2.64202700	1.32788900	-3.81217600
H	1.55760300	0.04750400	-4.37855400
C	3.42712900	-0.52806600	2.25656500
H	2.74333700	-1.37517800	2.12720100
C	-0.74927400	-2.50765500	2.71618000
H	0.29921800	-2.49580100	2.39685600
C	-1.02722800	-1.17804900	3.42361700
H	-0.86219800	-0.32848300	2.75087900
H	-2.06250100	-1.12848200	3.77943800
H	-0.36783100	-1.06057700	4.29038600
C	-0.92340200	-3.69195000	3.66863500
H	-0.23958900	-3.59679100	4.51860100
H	-1.94168400	-3.74082400	4.06921700
H	-0.71517900	-4.64260000	3.16696400
C	2.67669500	0.54934000	3.04386500
H	3.30894700	1.42749600	3.21579400
H	1.78041900	0.88175900	2.50736900
H	2.36569600	0.16294300	4.02060000
C	4.64250800	-1.03816900	3.03369700
H	5.35454500	-0.23194300	3.24061300
H	4.32866300	-1.45642200	3.99591800
H	5.17328200	-1.81813400	2.47810900
C	-0.24337900	2.70780600	0.16400500
Br	1.11430500	3.80519800	0.58950900
H	0.93021700	-4.51758800	-0.00045100
H	3.44615700	-3.33550600	-0.27193300
C	-2.74349400	1.92891000	-0.46893700
C	-3.33154500	0.83804000	0.44076200
C	-3.57066400	3.22473200	-0.38772200
H	-2.77368900	1.56196000	-1.50600400
C	-4.79232800	0.58490200	0.08919200
H	-3.24411700	1.16604200	1.48537800
H	-2.75126500	-0.08650500	0.34288500
C	-5.02781400	2.93800100	-0.73861800
H	-3.50086300	3.62236200	0.63331700
H	-3.15001900	3.98033100	-1.06022300
C	-5.64010700	1.85572900	0.15155300
H	-5.19984200	-0.17594800	0.76537100
H	-4.85124100	0.16086100	-0.92513100
H	-5.60785400	3.86502000	-0.65828400
H	-5.09423400	2.61915300	-1.78977200
H	-5.61566500	2.22329000	1.18976300
C	-7.08834000	1.57705600	-0.22753800
H	-7.53234900	0.82023700	0.42860700
H	-7.69980100	2.48302800	-0.15597000
H	-7.15767700	1.20847200	-1.25868100

Cartesian coordinates of the optimized geometry for Int-**14** at PBE0-D3BJ/6-31G*,def2-TZVP level of theory: (number of imaginary frequencies = 0):

Au	0.07110700	-0.46486700	-0.37906300
C	1.62104700	-1.53533300	-0.97549500
C	-1.44062500	0.72880700	0.28604800
C	-2.52211200	2.53787400	1.06951500
C	-3.43458300	1.53795800	0.93590900
N	-1.31133500	2.01953300	0.66584700
N	-2.74889100	0.44270700	0.45712300
C	-3.32015300	-0.84203100	0.16839200

C	-3.79344000	-1.07939800	-1.12888100
C	-3.34238500	-1.80069600	1.19028500
C	-4.30893200	-2.34951300	-1.39266600
C	-3.86813900	-3.05389500	0.87219300
C	-4.34604200	-3.32545200	-0.40422800
H	-4.68478100	-2.57684900	-2.38601900
H	-3.90308800	-3.82709500	1.63423900
H	-4.75288000	-4.30685400	-0.63033400
C	-0.06078700	2.72674300	0.64991200
C	0.30402400	3.40271000	-0.52224100
C	0.74296900	2.67649900	1.79674200
C	1.53450400	4.06109300	-0.51851000
C	1.96430900	3.35052900	1.74602600
C	2.35472500	4.03849000	0.60309700
H	1.85613200	4.59515200	-1.40779900
H	2.61802000	3.33542300	2.61327200
H	3.30698600	4.56097800	0.58606100
C	-2.79408700	-1.52303600	2.57564400
H	-2.55580200	-0.45518700	2.64349500
C	-1.49197900	-2.29630600	2.80263600
H	-1.66139000	-3.37766300	2.74566200
H	-0.74286600	-2.02965500	2.04751000
H	-1.07709200	-2.07012000	3.79111100
C	-3.81972100	-1.83108100	3.66779300
H	-3.42057700	-1.55569800	4.64972400
H	-4.75027200	-1.27660000	3.50905200
H	-4.06723500	-2.89754500	3.70088700
C	0.34567200	1.89892100	3.03517800
H	-0.68702500	1.55357000	2.90854100
C	0.38414600	2.76840600	4.29292400
H	0.02758100	2.19845200	5.15742800
H	1.40094600	3.10685400	4.51929100
H	-0.24749500	3.65605200	4.18431000
C	1.22682400	0.65636100	3.18971100
H	1.15165000	0.01233400	2.30594100
H	2.28016800	0.93208000	3.31345500
H	0.92190300	0.07509000	4.06700500
C	-0.56310500	3.40301100	-1.76465900
H	-1.51615800	2.91981800	-1.51990500
C	-3.72746400	-0.03149000	-2.22151900
H	-3.40299800	0.91414000	-1.77183000
C	-2.68527300	-0.41844900	-3.27441000
H	-1.69645700	-0.55192600	-2.81967100
H	-2.95673300	-1.35670900	-3.77127800
H	-2.60770000	0.35976000	-4.04170000
C	-5.09635900	0.21349600	-2.85871600
H	-5.03071600	1.02025700	-3.59638300
H	-5.46528800	-0.67749400	-3.37809100
H	-5.84176500	0.49595400	-2.10817000
C	0.09468100	2.57991100	-2.87563900
H	1.05812500	3.01156700	-3.16900800
H	0.27508000	1.55008000	-2.54571600
H	-0.54779800	2.54961400	-3.76255500
C	-0.87952400	4.82301300	-2.23733800
H	0.02538200	5.35501900	-2.55018700
H	-1.55743900	4.79324800	-3.09692400
H	-1.35617200	5.41065800	-1.44596700
C	1.79870500	-2.99401500	-1.04160300
C	3.10573300	-3.23368800	-1.81199700
H	2.94999700	-3.12861800	-2.89229500
C	3.97538000	-2.09455000	-1.26671300
H	2.77435900	-0.72612600	-2.42653700
C	2.93273800	-0.96009700	-1.35669700
Br	3.35797200	0.73808900	-0.52884400
H	-4.49462900	1.50421900	1.13365800
H	-2.62076800	3.55687300	1.40988500

H	4.84262500	-1.87633600	-1.89573500
C	4.41315500	-2.35131400	0.18259400
C	2.06722100	-3.34888900	0.47265300
C	3.18042600	-2.49820000	1.08933500
H	3.47321400	-2.92813900	2.05346300
H	2.78371600	-1.49770200	1.31321300
H	4.96372400	-1.46177500	0.51396900
H	3.52174800	-4.22766100	-1.63010100
H	2.33370400	-4.41253000	0.46298700
H	1.13863700	-3.24196700	1.04056800
H	0.91320900	-3.54324700	-1.37162200
C	5.35992100	-3.54496000	0.28385800
H	6.19386200	-3.45127300	-0.42030100
H	5.78138600	-3.60709600	1.29205100
H	4.85927500	-4.49816500	0.08127300

Cartesian coordinates of the optimized geometry for Int-**15** at PBE0-D3BJ/6-31G*,def2-TZVP level of theory: (number of imaginary frequencies = 0):

Au	-0.39047500	-0.54807800	-0.04170000
C	1.19649300	-2.07771400	-0.21627900
C	-1.35516500	1.21627800	-0.03510100
C	-3.00417600	2.73636400	-0.07112200
C	-1.82585700	3.40878100	-0.00204100
N	-2.68925300	1.39245900	-0.09209300
N	-0.82683100	2.45442200	0.02132900
C	0.58234300	2.71734200	0.10220400
C	1.16160000	2.83400000	1.37369400
C	1.30934700	2.81980900	-1.09243700
C	2.53391300	3.08628800	1.42529600
C	2.67860400	3.07021300	-0.98259600
C	3.28267000	3.21072700	0.26113100
H	3.02157200	3.18504100	2.39066600
H	3.27819200	3.15993100	-1.88382700
H	4.34810800	3.41315900	0.32338000
C	-3.61322300	0.29482400	-0.15460000
C	-4.05327600	-0.26807000	1.05142100
C	-3.97714100	-0.19181000	-1.41781800
C	-4.90417900	-1.37091900	0.96156500
C	-4.82928800	-1.29677200	-1.44886300
C	-5.28760700	-1.87958500	-0.27344500
H	-5.26964500	-1.83737900	1.87191700
H	-5.13501300	-1.70752700	-2.40674000
H	-5.95129000	-2.73812400	-0.32073700
C	0.66923700	2.64302600	-2.45427300
H	-0.41274300	2.53586400	-2.31537900
C	1.17337300	1.36076000	-3.12257000
H	2.25601000	1.39999600	-3.28882900
H	0.95729500	0.48237500	-2.50264300
H	0.68842200	1.21744300	-4.09416600
C	0.89729900	3.86253300	-3.34944900
H	0.37471400	3.73475900	-4.30323400
H	0.52810300	4.77868000	-2.87727200
H	1.96014300	4.00583200	-3.57213600
C	-3.45636500	0.41529600	-2.70476900
H	-2.87715800	1.31137100	-2.45288400
C	-4.59811300	0.85397700	-3.62388600
H	-4.19761600	1.34256300	-4.51840500
H	-5.19827100	-0.00017900	-3.95545800
H	-5.26848200	1.55812200	-3.12034700
C	-2.51103600	-0.55595900	-3.41725700
H	-1.67181600	-0.83856600	-2.77092000
H	-3.03309200	-1.47399200	-3.70889700
H	-2.10457000	-0.09761100	-4.32553900
C	-3.62161000	0.26065100	2.40435800

H	-3.00939800	1.15610600	2.24568400
C	0.36488800	2.65071100	2.64929500
H	-0.69660000	2.58025200	2.38508300
C	0.75456800	1.33707100	3.33387000
H	0.59846200	0.48096300	2.66738700
H	1.80985500	1.34471800	3.62937700
H	0.15307100	1.18196200	4.23616400
C	0.52094600	3.84023000	3.59795800
H	-0.11683900	3.70675800	4.47798200
H	1.55225100	3.94196400	3.95256000
H	0.23985500	4.77980300	3.11124500
C	-2.75135300	-0.76390200	3.13691600
H	-3.30717000	-1.68674200	3.33639900
H	-1.86576200	-1.02708100	2.54682600
H	-2.41318600	-0.36122600	4.09788900
C	-4.82485300	0.67557900	3.25375700
H	-5.46473700	-0.18162000	3.48952800
H	-4.48841800	1.10752600	4.20220800
H	-5.44026400	1.41977000	2.73779000
C	0.13589100	-2.65117500	0.07735900
Br	-1.13743300	-3.85584900	0.47255100
H	-1.60292000	4.46368200	0.03305800
H	-4.02569500	3.08126800	-0.10674600
C	2.56950900	-1.66754100	-0.52999100
C	3.05409300	-0.52154700	0.36849800
C	3.50990800	-2.87759200	-0.41096000
H	2.58035700	-1.31611800	-1.57282400
C	4.48922400	-0.14051100	0.02892500
H	2.98628500	-0.84464300	1.41624800
H	2.39937400	0.35031500	0.25696900
C	4.94080300	-2.47304700	-0.75426000
H	3.46368800	-3.25662300	0.61853300
H	3.17101100	-3.68496900	-1.06945600
C	5.46257800	-1.32004000	0.10954500
H	4.79669800	0.66068200	0.70723000
H	4.51865000	0.28407200	-0.98671700
H	5.57923700	-3.35493600	-0.64532300
H	4.99176100	-2.17901300	-1.81373300
H	5.48400900	-1.66670100	1.15697900
C	6.89882500	-0.91368000	-0.27250400
H	6.87792600	-0.59014300	-1.32571600
C	7.87563900	-2.08393200	-0.15670900
H	7.64319900	-2.89913400	-0.84830100
H	8.89618400	-1.75413300	-0.37773300
H	7.87465300	-2.49290600	0.86190000
C	7.40804700	0.25624200	0.56995800
H	6.83846400	1.17612200	0.40387400
H	7.36064000	0.01645800	1.64017700
H	8.45340800	0.47512100	0.32833800

Cartesian coordinates of the optimized geometry for Int-**16** at PBE0-D3BJ/6-31G*,def2-TZVP level of theory: (number of imaginary frequencies = 0):

Au	-0.10496800	-0.35594600	-0.40855800
C	1.64973200	-1.00001700	-1.05224200
C	-1.84616900	0.42038700	0.31040400
C	-3.31620800	1.89814700	1.15414200
C	-3.96188300	0.70934200	1.01318900
N	-2.02516000	1.69670200	0.71778500
N	-3.04198500	-0.17776200	0.49734600
C	-3.29086600	-1.55749400	0.18997300
C	-3.72217200	-1.87997400	-1.10353800
C	-3.05609400	-2.51058400	1.19018100
C	-3.91865700	-3.23257500	-1.38706800
C	-3.26795000	-3.84829700	0.85297000

C	-3.69413000	-4.20547400	-0.42070400
H	-4.25053700	-3.52705400	-2.37842700
H	-3.09628500	-4.62017500	1.59760300
H	-3.85458200	-5.25231900	-0.66188200
C	-0.98363700	2.68623600	0.70299100
C	-0.81491500	3.45587200	-0.45587200
C	-0.17164300	2.80871900	1.83855100
C	0.21611400	4.39638300	-0.44879300
C	0.84581600	3.76323000	1.79170400
C	1.03533500	4.55183700	0.66295800
H	0.38217000	5.01192000	-1.32802100
H	1.49845700	3.88923400	2.65071500
H	1.82959200	5.29281200	0.64887100
C	-2.55951300	-2.13184100	2.57118700
H	-2.59446400	-1.03990200	2.66158000
C	-1.09979300	-2.55790400	2.75170400
H	-0.99440000	-3.64570300	2.66862900
H	-0.46147000	-2.09550000	1.98936700
H	-0.72940300	-2.25577200	3.73757200
C	-3.44630100	-2.70856900	3.67591200
H	-3.10439100	-2.35906800	4.65585600
H	-4.49026200	-2.40414700	3.54931600
H	-3.41609100	-3.80337000	3.68883600
C	-0.34367800	1.92992500	3.06100600
H	-1.25507700	1.33456500	2.93195600
C	-0.51535600	2.75552300	4.33716200
H	-0.70210900	2.09658800	5.19175700
H	0.38250300	3.34078500	4.56312000
H	-1.35582900	3.45202900	4.25222800
C	0.82728700	0.95113600	3.18399900
H	0.91126500	0.32712300	2.28686900
H	1.77652300	1.48398100	3.30995300
H	0.68929700	0.29363800	4.04954500
C	-1.67301200	3.26668200	-1.68998500
H	-2.47370100	2.55828200	-1.44770900
C	-3.93874700	-0.82794400	-2.17249400
H	-3.84906800	0.15983600	-1.70563600
C	-2.85419300	-0.92510600	-3.24899400
H	-1.85433200	-0.81745200	-2.81190700
H	-2.89471100	-1.89227600	-3.76265400
H	-2.98696200	-0.13818100	-3.99967400
C	-5.33798200	-0.91690700	-2.78413000
H	-5.48832400	-0.10433500	-3.50276900
H	-5.48538600	-1.86078500	-3.31993400
H	-6.11519500	-0.84294000	-2.01653900
C	-0.84691200	2.65291000	-2.82384900
H	-0.02661100	3.31696400	-3.11838000
H	-0.40896500	1.69614700	-2.51576000
H	-1.47448200	2.47714600	-3.70451700
C	-2.33649300	4.57316500	-2.12881700
H	-1.59517700	5.31842700	-2.43669500
H	-2.99699700	4.39346900	-2.98368400
H	-2.93340500	5.00909700	-1.32106300
C	2.18371300	-2.36594300	-1.15568400
C	3.49890100	-2.26127200	-1.94270700
H	3.30736000	-2.18435100	-3.01971400
C	4.06660500	-0.95028000	-1.38530300
H	2.54215000	0.09259400	-2.49475300
C	2.77194300	-0.11156100	-1.43139000
Br	2.78012000	1.62680400	-0.57906700
H	-4.97743400	0.41560100	1.22836600
H	-3.65103100	2.85626000	1.51998900
H	4.82540100	-0.50107700	-2.02831000
C	4.59181300	-1.11906300	0.04882100
C	2.55793400	-2.65555400	0.34938900
C	3.43550400	-1.56177200	0.96246400

H	3.81596000	-1.91134800	1.92638900
H	2.81164600	-0.68643700	1.19222300
H	4.92413400	-0.12685200	0.38564600
H	4.15097100	-3.12301600	-1.77750600
H	3.08145400	-3.61879400	0.32208900
H	1.64225200	-2.78871500	0.93248000
H	1.45934100	-3.11580400	-1.48324400
C	5.81738100	-2.05574200	0.13923100
H	5.47081400	-3.09662100	0.04891900
C	6.50560900	-1.90981200	1.49642400
H	5.83478800	-2.10638100	2.33868900
H	7.34390000	-2.60916700	1.57834500
H	6.90605300	-0.89568600	1.61693600
C	6.83549900	-1.79971200	-0.97116500
H	7.14904900	-0.74791200	-0.97899900
H	7.73204600	-2.40732900	-0.81192700
H	6.45054600	-2.04557100	-1.96639500

Cartesian coordinates of the optimized geometry for Int-**17** at PBE0-D3BJ/6-31G*,def2-TZVP level of theory: (number of imaginary frequencies = 0):

Au	-0.52178100	-0.54450000	-0.03524300
C	1.08860600	-2.05179900	-0.19258200
C	-1.50628500	1.20877600	-0.03675900
C	-3.17153300	2.71068500	-0.08706100
C	-2.00116600	3.39609000	-0.01126600
N	-2.84176100	1.37024700	-0.10334500
N	-0.99190200	2.45277200	0.02090300
C	0.41368800	2.73133200	0.11139300
C	0.98077400	2.86436900	1.38680200
C	1.14984100	2.83184100	-1.07781100
C	2.34992700	3.13118300	1.44802700
C	2.51562400	3.09654400	-0.95828700
C	3.10750500	3.25326400	0.28930500
H	2.82830200	3.24269900	2.41663000
H	3.12193200	3.18526800	-1.85515600
H	4.17037800	3.46633100	0.35901400
C	-3.75320400	0.26248800	-0.17083600
C	-4.19409300	-0.30486600	1.03276700
C	-4.10439900	-0.22839300	-1.43601100
C	-5.03273300	-1.41669200	0.93829400
C	-4.94428800	-1.34255200	-1.47170000
C	-5.40334500	-1.92982300	-0.29877600
H	-5.39858700	-1.88674600	1.84664200
H	-5.23979300	-1.75696800	-2.43120400
H	-6.05751100	-2.79543300	-0.34966300
C	0.52299700	2.64086200	-2.44388900
H	-0.55855400	2.52007600	-2.31312100
C	1.04899800	1.36297700	-3.10349900
H	2.13238400	1.41523100	-3.26107300
H	0.83872900	0.48414300	-2.48222800
H	0.57396300	1.21013100	-4.07852200
C	0.74232700	3.86006300	-3.34173300
H	0.22966500	3.72183300	-4.29944200
H	0.35690900	4.77289100	-2.87613200
H	1.80506900	4.01672800	-3.55576700
C	-3.58241700	0.38402400	-2.71996600
H	-3.01674700	1.28783700	-2.46492900
C	-4.72305400	0.80690100	-3.64781500
H	-4.32243500	1.30014400	-4.53971800
H	-5.30937100	-0.05532800	-3.98323500
H	-5.40640000	1.50244200	-3.14979600
C	-2.61948100	-0.57566400	-3.42453200
H	-1.78090100	-0.84615500	-2.77220000
H	-3.12737500	-1.50098500	-3.71814200

H	-2.21304700	-0.11335600	-4.33082600
C	-3.77575200	0.22859900	2.38801100
H	-3.16997800	1.12898400	2.23255200
C	0.17500400	2.68220800	2.65689200
H	-0.88391500	2.60433500	2.38456400
C	0.56697400	1.37379500	3.35023500
H	0.41967800	0.51362300	2.68703500
H	1.62030500	1.38846500	3.65241800
H	-0.03956400	1.21991000	4.24937400
C	0.31631200	3.87655600	3.60174100
H	-0.32890700	3.74350300	4.47638200
H	1.34367600	3.98512700	3.96562400
H	0.03492600	4.81260300	3.10844800
C	-2.90181600	-0.78804800	3.12717600
H	-3.45139200	-1.71508200	3.32445700
H	-2.01055500	-1.04482400	2.54280900
H	-2.57275700	-0.38147700	4.08966500
C	-4.98803800	0.63413600	3.22905200
H	-5.62245700	-0.22817700	3.46089800
H	-4.66157900	1.06929000	4.17952600
H	-5.60600900	1.37301900	2.70852700
C	0.03159600	-2.64152500	0.08090600
Br	-1.23148400	-3.86533500	0.44926300
H	-1.79003500	4.45343300	0.02336600
H	-4.19649700	3.04429900	-0.13040000
C	2.45895200	-1.61551700	-0.48136100
C	2.91268700	-0.47835000	0.44465200
C	3.41753700	-2.81301800	-0.37819400
H	2.47675200	-1.24271800	-1.51663000
C	4.34763300	-0.07186000	0.13334700
H	2.83273500	-0.81940900	1.48583800
H	2.24672900	0.38530900	0.33550000
C	4.84539200	-2.37367800	-0.69281400
H	3.36474300	-3.21878900	0.64072000
H	3.10045200	-3.60840600	-1.06174000
C	5.32549400	-1.24725500	0.22941600
H	4.63134300	0.72792400	0.82355800
H	4.37982700	0.36146300	-0.87641700
H	5.49808600	-3.24804000	-0.61014300
H	4.89331000	-2.04338200	-1.73980000
H	5.26498700	-1.63050800	1.26240900
C	6.81149000	-0.84572800	0.01354500
C	7.20495100	0.23332300	1.02989700
H	6.70007600	1.18688400	0.84088400
H	6.97013900	-0.08033700	2.05476900
H	8.28255900	0.42458000	0.98013600
C	7.71265000	-2.06282300	0.25429700
H	7.57475300	-2.84003800	-0.50436400
H	8.76670700	-1.76560000	0.22203100
H	7.52312000	-2.50916300	1.23836900
C	7.06960300	-0.31061400	-1.39852800
H	6.85557400	-1.06045300	-2.16805900
H	6.47174500	0.58211400	-1.61545700
H	8.12328900	-0.02902100	-1.50488400

Cartesian coordinates of the optimized geometry for Int-**18** at PBE0-D3BJ/6-31G*,def2-TZVP level of theory: (number of imaginary frequencies = 0):

Au	-0.20817000	-0.32193100	-0.42094200
C	1.57016400	-0.86039700	-1.09598700
C	-1.97917800	0.34494200	0.33442400
C	-3.51684200	1.72446900	1.22297000
C	-4.09576600	0.50258000	1.07544900
N	-2.22391000	1.60322100	0.76305200
N	-3.13552400	-0.32328500	0.53211400

C	-3.31157700	-1.71016300	0.20704900
C	-3.74307400	-2.03722500	-1.08528800
C	-3.00988200	-2.66327700	1.18906800
C	-3.86856300	-3.39436900	-1.38685100
C	-3.15224600	-4.00563400	0.83427300
C	-3.57648500	-4.36730000	-0.43877600
H	-4.19802700	-3.69228700	-2.37799500
H	-2.92722200	-4.77756700	1.56448900
H	-3.68223700	-5.41780800	-0.69381800
C	-1.24286100	2.65249100	0.73976100
C	-1.14250500	3.44117200	-0.41419500
C	-0.41738400	2.81240100	1.86083000
C	-0.16877600	4.44093200	-0.41729300
C	0.54098700	3.82568200	1.80427000
C	0.66161400	4.63426900	0.68014300
H	-0.05676900	5.07335800	-1.29309800
H	1.20196600	3.98221800	2.65180100
H	1.41047800	5.42092700	0.65854100
C	-2.51452500	-2.27773000	2.56861100
H	-2.61071600	-1.19115700	2.67720300
C	-1.03005100	-2.62160900	2.72034000
H	-0.86392700	-3.70018400	2.61836800
H	-0.43167800	-2.11201900	1.95571800
H	-0.66182300	-2.31341900	3.70515400
C	-3.34913800	-2.92153400	3.67690100
H	-3.01266200	-2.56763900	4.65714800
H	-4.41080000	-2.67628500	3.57064400
H	-3.25554600	-4.01281400	3.67192800
C	-0.51247500	1.91251600	3.07621300
H	-1.39238400	1.26900300	2.96040500
C	-0.69946200	2.71264000	4.36628700
H	-0.83021600	2.03454000	5.21627600
H	0.17008500	3.34353900	4.57976900
H	-1.57845400	3.36289000	4.30915700
C	0.71349400	0.99900800	3.15985600
H	0.81172500	0.39285800	2.25193200
H	1.63415400	1.58261000	3.27239300
H	0.63195400	0.32341700	4.01860800
C	-2.01329300	3.21389200	-1.63285200
H	-2.76115300	2.45187000	-1.38439200
C	-4.03351000	-0.98359600	-2.13498700
H	-3.99005000	0.00099000	-1.65498400
C	-2.96308000	-1.00635200	-3.22961900
H	-1.96340500	-0.85087700	-2.80674600
H	-2.95971000	-1.96676800	-3.75734400
H	-3.15054700	-0.21707000	-3.96601100
C	-5.43565600	-1.14000100	-2.72615900
H	-5.64145300	-0.32688000	-3.43029600
H	-5.54037600	-2.08296900	-3.27348900
H	-6.20315900	-1.11913300	-1.94562700
C	-1.17533500	2.67129400	-2.79390300
H	-0.40750500	3.39179300	-3.09708300
H	-0.66836800	1.74134700	-2.51074700
H	-1.81057800	2.46683300	-3.66279800
C	-2.76907800	4.48110200	-2.03633700
H	-2.08396100	5.27743200	-2.34661100
H	-3.43481100	4.27215000	-2.88044300
H	-3.37536400	4.86504400	-1.20939200
C	2.17652700	-2.19320300	-1.21854300
C	3.46891000	-2.00702200	-2.02493700
H	3.25085000	-1.91361800	-3.09600200
C	3.98006900	-0.67852300	-1.45548900
H	2.38572800	0.27966000	-2.54700800
C	2.63581600	0.08984900	-1.48565500
Br	2.54389200	1.82624700	-0.63327300
H	-5.08909200	0.14813400	1.30260900

H	-3.89976300	2.65687600	1.60756400
H	4.70183000	-0.18400200	-2.10740900
C	4.51771400	-0.77849600	-0.01964300
C	2.58974100	-2.48225800	0.27595300
C	3.42330100	-1.35770200	0.89811300
H	3.85537200	-1.71476300	1.83807700
H	2.74635300	-0.53925000	1.18187100
H	4.16198900	-2.83894200	-1.89619800
H	3.15317300	-3.42021600	0.22439200
H	1.68912100	-2.66291700	0.87003600
H	1.48896000	-2.97834600	-1.54296500
H	4.66316300	0.26479900	0.29269700
C	5.92834000	-1.42644400	0.15245300
C	5.92646000	-2.95758100	0.23290700
H	5.29978100	-3.32586400	1.05285700
H	5.59957500	-3.43741400	-0.69458700
H	6.94501800	-3.31182300	0.42675000
C	6.51110300	-0.89319800	1.46931100
H	7.50191500	-1.32349700	1.65126100
H	6.61937000	0.19724400	1.44022400
H	5.88252900	-1.14593900	2.33093600
C	6.85759300	-0.99176900	-0.98428200
H	6.87114700	0.09935200	-1.09740100
H	7.88357100	-1.31279800	-0.77424800
H	6.57061300	-1.43343800	-1.94559300

Cartesian coordinates of the optimized geometry for Int-**19** at PBE0-D3BJ/6-31G*,def2-TZVP level of theory: (number of imaginary frequencies = 0):

Au	0.00581800	-0.45139400	-0.33197200
C	1.42728900	-1.90799000	-1.19474600
C	-0.88848600	1.25381400	0.25463700
C	-2.47568200	2.71805400	0.86166400
C	-1.28472000	3.37176200	0.87730200
N	-2.20709700	1.42084200	0.47453000
N	-0.32440000	2.45179400	0.50355500
C	1.08584000	2.69894800	0.39815300
C	1.87989900	2.48336700	1.53337100
C	1.59717800	3.11164700	-0.84039700
C	3.25281600	2.69641600	1.39532600
C	2.97596000	3.31629200	-0.92016000
C	3.79452500	3.11089400	0.18426800
H	3.90472700	2.54004000	2.24986900
H	3.41396100	3.63712200	-1.86080700
H	4.86476300	3.27643300	0.10064600
C	-3.15905000	0.35588800	0.32686100
C	-3.40344600	-0.46942300	1.43328100
C	-3.74772300	0.16449600	-0.93073600
C	-4.29118000	-1.53037700	1.24719200
C	-4.62693400	-0.91173800	-1.06060400
C	-4.89601900	-1.74911600	0.01535000
H	-4.51042100	-2.19381700	2.07892000
H	-5.10450800	-1.09749600	-2.01828900
H	-5.58468000	-2.58003100	-0.10751200
C	0.71940800	3.29688600	-2.06170800
H	-0.32737700	3.19876600	-1.75140500
C	0.99580300	2.19861700	-3.09286300
H	2.03454900	2.23621400	-3.44046400
H	0.81663600	1.20348100	-2.66812800
H	0.34346700	2.31720500	-3.96477800
C	0.88490200	4.68801500	-2.67599800
H	0.18993700	4.81636400	-3.51241700
H	0.68538400	5.47505700	-1.94165700
H	1.89778100	4.84075700	-3.06385400
C	-3.43593300	1.05159200	-2.11887000

H	-2.78178700	1.86349800	-1.78002000
C	-4.70220000	1.69338900	-2.68949700
H	-4.44629100	2.37299400	-3.50922000
H	-5.38985700	0.93968400	-3.08798800
H	-5.23939500	2.26525900	-1.92582100
C	-2.67571400	0.26982300	-3.19366200
H	-1.75110200	-0.16034400	-2.79120100
H	-3.28243300	-0.55189600	-3.58989400
H	-2.41243200	0.92645000	-4.03014100
C	-2.73194200	-0.25611400	2.77498300
H	-2.11834800	0.65028100	2.71386600
C	1.30011600	2.02321500	2.85589500
H	0.20706100	2.03505600	2.77418600
C	1.71909500	0.58321700	3.16295200
H	1.39024300	-0.10148900	2.37318100
H	2.80795700	0.49669000	3.24978900
H	1.27793500	0.25048600	4.10883000
C	1.68114600	2.96739600	3.99793600
H	1.19122100	2.65381800	4.92568900
H	2.76124100	2.96555000	4.17984700
H	1.37955100	3.99770000	3.78302000
C	-1.79456300	-1.41964600	3.10785400
H	-2.34608800	-2.36241800	3.19395400
H	-1.03014900	-1.54858900	2.33292300
H	-1.28628800	-1.24015600	4.06144200
C	-3.76101700	-0.03658300	3.88605400
H	-4.38902700	-0.92216500	4.03160000
H	-3.25611800	0.17075000	4.83549300
H	-4.42106200	0.80670900	3.65798100
C	0.46119100	-2.53261200	-0.72883600
Br	-0.69980600	-3.80571100	-0.21867000
H	-1.02971400	4.39230900	1.11656300
H	-3.47770200	3.04798200	1.08721300
C	2.68759800	-1.47069500	-1.81807200
C	3.59466000	-2.70241800	-2.01310100
C	3.41034300	-0.40045700	-0.98344800
H	2.42884100	-1.04775300	-2.79824600
C	4.10578200	-3.24605400	-0.68288700
H	4.44009500	-2.37501900	-2.63229300
H	3.06554400	-3.47666400	-2.57943400
C	3.91344500	-0.96202400	0.33978300
H	4.25751500	-0.04386800	-1.58318100
H	2.75678300	0.46325700	-0.81895900
C	4.82104100	-2.17778200	0.14751700
H	4.78100000	-4.08927300	-0.87033500
H	3.26452900	-3.64928800	-0.09929200
H	4.44271000	-0.17472000	0.88994400
H	3.05617200	-1.25101700	0.96714700
C	5.29098100	-2.73588300	1.48405100
H	5.96237400	-3.59023200	1.34557000
H	5.82827200	-1.97769300	2.06466300
H	4.43804600	-3.07557000	2.08524200
H	5.70405900	-1.84772200	-0.42269700

Cartesian coordinates of the optimized geometry for Int-**20** at PBE0-D3BJ/6-31G*,def2-TZVP level of theory: (number of imaginary frequencies = 0):

Au	0.01578700	-0.50768200	-0.40432400
C	1.45064300	-1.71444700	-1.03449300
C	-1.36040000	0.81491100	0.30749100
C	-2.24310400	2.70739400	1.14188100
C	-3.25194600	1.80439000	1.01209100
N	-1.09732100	2.07985400	0.70475500
N	-2.68682700	0.65562600	0.50201700
C	-3.38763700	-0.56167900	0.20676800

C	-3.89938000	-0.73368000	-1.08612400
C	-3.49212500	-1.52642600	1.21791000
C	-4.54165700	-1.94301600	-1.35753000
C	-4.14350200	-2.71715000	0.89255900
C	-4.66222200	-2.92336400	-0.38025500
H	-4.95146600	-2.11877400	-2.34792100
H	-4.24521500	-3.49316400	1.64562500
H	-5.16716800	-3.85669600	-0.61203100
C	0.21554000	2.66250400	0.67525400
C	0.62111000	3.32535600	-0.49089400
C	1.03259000	2.51137000	1.80365100
C	1.90747600	3.86656300	-0.49898700
C	2.31023100	3.07036600	1.74193400
C	2.74204500	3.74460200	0.60568200
H	2.26150000	4.38660000	-1.38428700
H	2.97553300	2.97512800	2.59522100
H	3.73816200	4.17727500	0.58018200
C	-2.89733300	-1.32255000	2.59702400
H	-2.57611700	-0.27783300	2.68160400
C	-1.65394200	-2.19805200	2.77785100
H	-1.90783200	-3.26148500	2.69982000
H	-0.90250100	-1.97079900	2.01221100
H	-1.20251600	-2.02838000	3.76166200
C	-3.91941700	-1.57526100	3.70631600
H	-3.47997500	-1.34770600	4.68325200
H	-4.80999800	-0.95159500	3.57821100
H	-4.24339600	-2.62130100	3.72816900
C	0.58837400	1.74786000	3.03478500
H	-0.46643700	1.47666200	2.91011900
C	0.69420200	2.59989800	4.30079600
H	0.30945500	2.04575600	5.16353200
H	1.73302200	2.86990200	4.51928700
H	0.12054300	3.52771000	4.20673400
C	1.38117100	0.44523900	3.17163200
H	1.25992700	-0.18166900	2.28086600
H	2.45149900	0.64425100	3.29656000
H	1.03787700	-0.12348800	4.04292300
C	-0.26252300	3.42790000	-1.71715200
H	-1.25075000	3.02659000	-1.46412200
C	-3.74548600	0.31768200	-2.16654800
H	-3.32359300	1.22061100	-1.70996500
C	-2.76080200	-0.15466300	-3.23971600
H	-1.78311000	-0.38860000	-2.80171000
H	-3.12875000	-1.05593600	-3.74292900
H	-2.61923700	0.62217700	-3.99922300
C	-5.09229600	0.70466600	-2.78003300
H	-4.95775500	1.51167600	-3.50801200
H	-5.55394700	-0.13827900	-3.30542800
H	-5.79611900	1.04798000	-2.01486400
C	0.30188000	2.57125400	-2.85398200
H	1.29263200	2.92508800	-3.16030300
H	0.40167900	1.52427300	-2.54426700
H	-0.35764200	2.61087500	-3.72792200
C	-0.45834800	4.87941500	-2.15818900
H	0.48583000	5.33542100	-2.47498300
H	-1.14810900	4.92703700	-3.00748500
H	-0.86992900	5.49091700	-1.34856200
C	1.47860100	-3.18002200	-1.14426600
C	2.76093500	-3.52797200	-1.91661000
H	2.61936500	-3.38048200	-2.99404700
C	3.74280800	-2.50450800	-1.32752700
H	2.67426500	-1.03957700	-2.47060500
C	2.81321000	-1.26780100	-1.39534900
Br	3.35614400	0.42243600	-0.62036700
H	-4.30611400	1.87124400	1.23163800
H	-2.23427800	3.72501900	1.50006900

H	4.63182300	-2.35242400	-1.94538100
C	4.13152900	-2.94070300	0.09719600
C	1.68697400	-3.59301100	0.36467000
C	2.89782500	-2.91088600	1.00336900
H	3.12364100	-3.39254500	1.96101200
H	2.65639600	-1.86324200	1.23979600
H	3.07945600	-4.56206500	-1.75535800
H	1.82610100	-4.68091500	0.33455500
H	0.77268900	-3.38517300	0.92765500
H	0.54542200	-3.62679500	-1.49649700
C	5.30990300	-2.18576900	0.70056600
H	5.07126700	-1.13681000	0.89160700
H	5.60218200	-2.64489200	1.65110600
H	6.17896600	-2.21868100	0.03474000
H	4.43996200	-3.99204400	-0.00581100

Cartesian coordinates of the optimized geometry for Int-**21** at PBE0-D3BJ/6-31G*,def2-TZVP level of theory: (number of imaginary frequencies = 0):

Au	-0.35721600	-0.57594400	-0.25101300
C	0.55973900	-2.50536200	-0.82478200
C	-0.81317800	1.31001700	0.28596900
C	-2.02807500	3.06729500	0.97076600
C	-0.77905700	3.51635100	0.68231500
N	-2.02537000	1.70999000	0.71812400
N	-0.04949700	2.41838300	0.26615400
C	1.31893200	2.43170500	-0.16685100
C	2.32315800	2.37702800	0.81220500
C	1.57655200	2.48110000	-1.54327200
C	3.64302700	2.38564500	0.36261100
C	2.91718100	2.47730200	-1.93684500
C	3.93713100	2.43552700	-0.99627900
H	4.45375600	2.35242700	1.08281400
H	3.16188000	2.52011500	-2.99426600
H	4.97325900	2.44435800	-1.32080900
C	-3.13563400	0.80782300	0.84590800
C	-3.25672500	0.06861100	2.03075500
C	-4.00115900	0.66841000	-0.24846300
C	-4.30967900	-0.84530100	2.10195700
C	-5.03531900	-0.26048700	-0.12353000
C	-5.18870300	-1.00808300	1.03823100
H	-4.44165400	-1.43671100	3.00344100
H	-5.72831300	-0.40066200	-0.94798100
H	-6.00292500	-1.72291400	1.11624600
C	0.47656400	2.55327800	-2.58311100
H	-0.49023100	2.51474400	-2.06841400
C	0.53168200	1.35711800	-3.53554900
H	1.46715600	1.34020300	-4.10557800
H	0.45437700	0.41089000	-2.98771200
H	-0.29454800	1.40384700	-4.25292500
C	0.53515700	3.87719600	-3.34948800
H	-0.29173800	3.93954600	-4.06495600
H	0.46614800	4.73400000	-2.67116500
H	1.47058800	3.97101000	-3.91184800
C	-3.81660300	1.44772600	-1.53540900
H	-3.04870000	2.21205900	-1.36825300
C	-5.09654900	2.17501300	-1.95096800
H	-4.91013500	2.78825900	-2.83879600
H	-5.89853500	1.47218000	-2.20078100
H	-5.46158900	2.82983600	-1.15313200
C	-3.31278900	0.52960200	-2.65297100
H	-2.35935000	0.06047600	-2.38353400
H	-4.03281500	-0.26956900	-2.86174500
H	-3.16295900	1.09828500	-3.57740000
C	-2.29666600	0.22519200	3.19240100

H	-1.55127700	0.98277000	2.92386200
C	1.99309700	2.25671500	2.28754800
H	0.98700600	2.66396500	2.44495700
C	1.96182900	0.78169800	2.70477300
H	1.24547200	0.20832800	2.10521700
H	2.94975300	0.32467500	2.57957900
H	1.67608100	0.68627300	3.75829300
C	2.94740500	3.04925800	3.17957300
H	2.58989700	3.03240700	4.21411800
H	3.95500000	2.61925500	3.18095800
H	3.02437200	4.09390000	2.86153000
C	-1.54450500	-1.07974100	3.46470800
H	-2.22892000	-1.87973900	3.76742300
H	-1.00437200	-1.42046700	2.57350300
H	-0.81727700	-0.93917500	4.27162300
C	-3.02256400	0.71958900	4.44591700
H	-3.76800400	-0.00663700	4.78784800
H	-2.30931500	0.87340500	5.26265700
H	-3.53986200	1.66662600	4.26031600
C	-0.67456300	-2.63661700	-0.82618600
Br	-2.36846600	-3.22432500	-0.92873800
H	-0.34186600	4.50140000	0.73202000
H	-2.90960900	3.57815100	1.32531500
C	2.01336400	-2.70472900	-0.87319000
C	2.77567300	-1.55110200	-1.53926000
C	2.58533900	-2.98974100	0.52499000
H	2.15224500	-3.60911300	-1.48739600
C	4.26678600	-1.87892700	-1.61741000
H	2.61147400	-0.63158900	-0.96094400
H	2.37638700	-1.36666000	-2.54313500
C	4.06771000	-3.32879000	0.40426800
H	2.43677800	-2.10289200	1.15637700
H	2.03778400	-3.81527800	0.99269600
C	4.88845800	-2.21096300	-0.25062800
H	4.78949800	-1.04195500	-2.09207600
H	4.40103300	-2.74060000	-2.28597100
H	4.47156900	-3.58559800	1.38808700
H	4.16673800	-4.23621100	-0.20825200
C	5.08079300	-0.95143300	0.62408300
H	4.16648300	-0.34257600	0.57073600
H	5.89309600	-2.61975400	-0.43261500
C	5.33994400	-1.27241000	2.09467200
H	6.19939900	-1.94520300	2.20704200
H	5.56967300	-0.35631400	2.65139900
H	4.48009500	-1.74375900	2.58251300
C	6.23535100	-0.10708000	0.08506300
H	7.18146500	-0.65629700	0.16542700
H	6.10023600	0.16773900	-0.96580900
H	6.34710100	0.82030800	0.65830300

Cartesian coordinates of the optimized geometry for Int-**22** at PBE0-D3BJ/6-31G*,def2-TZVP level of theory: (number of imaginary frequencies = 0):

Au	-0.18328900	-0.40899700	-0.48266200
C	1.38831200	-1.33572200	-1.24641800
C	-1.70498400	0.63557000	0.37896100
C	-2.82555900	2.31048800	1.37678600
C	-3.68374600	1.26395500	1.24127300
N	-1.62295500	1.90251200	0.84281900
N	-2.97493300	0.25234700	0.63028300
C	-3.48653600	-1.04705100	0.29894400
C	-4.04692600	-1.23389100	-0.97150000
C	-3.36513600	-2.06754000	1.25182800
C	-4.49874000	-2.51766500	-1.28199000
C	-3.83249900	-3.33170500	0.88923000

C	-4.39254600	-3.55453600	-0.36306500
H	-4.93812500	-2.70726000	-2.25699800
H	-3.75634500	-4.15228100	1.59675500
H	-4.75170300	-4.54571900	-0.62452500
C	-0.42360700	2.69143700	0.78612300
C	-0.19311200	3.46740000	-0.35788100
C	0.46694200	2.62088900	1.86570100
C	0.98737000	4.21087700	-0.39370200
C	1.63276700	3.38371500	1.77809300
C	1.88875100	4.17374900	0.66347400
H	1.20504500	4.82404300	-1.26327900
H	2.34986000	3.35680300	2.59347200
H	2.79961800	4.76389500	0.61730600
C	-2.72638500	-1.84001700	2.60746200
H	-2.56429200	-0.76335200	2.73489000
C	-1.35527200	-2.51848300	2.67577800
H	-1.44657500	-3.60328100	2.54805200
H	-0.69186100	-2.13804400	1.88995500
H	-0.88013500	-2.33028500	3.64493800
C	-3.63117400	-2.30221000	3.75079100
H	-3.17480700	-2.05798600	4.71589000
H	-4.61210200	-1.81834200	3.70403100
H	-3.79089300	-3.38555400	3.72795900
C	0.22054300	1.73279500	3.06837800
H	-0.78401300	1.30302300	2.97932200
C	0.26510500	2.52188600	4.37794700
H	0.02256500	1.86803400	5.22238200
H	1.25978700	2.94204300	4.56200200
H	-0.45114900	3.34994600	4.36982600
C	1.21486500	0.56860700	3.08438800
H	1.14253900	-0.01921800	2.16239000
H	2.24611600	0.92950300	3.16936200
H	1.01616900	-0.09468500	3.93353100
C	-1.14735900	3.48505800	-1.53433100
H	-2.04216600	2.91408000	-1.26056800
C	-4.13702100	-0.11607500	-1.99070300
H	-3.84658100	0.82028600	-1.50014800
C	-3.15272100	-0.35546000	-3.13892700
H	-2.12513500	-0.44153600	-2.76599300
H	-3.39368600	-1.27860600	-3.67803900
H	-3.18941100	0.47309100	-3.85485600
C	-5.56370300	0.06664000	-2.51141300
H	-5.61030100	0.92325300	-3.19211300
H	-5.90699600	-0.81297500	-3.06652600
H	-6.26883600	0.24173600	-1.69236700
C	-0.51474900	2.79201800	-2.74435500
H	0.38656300	3.32013700	-3.07505000
H	-0.22888900	1.76181800	-2.50118700
H	-1.21916600	2.76541800	-3.58302500
C	-1.59944200	4.90503600	-1.87942900
H	-0.76020600	5.52585600	-2.21117600
H	-2.33300000	4.88153900	-2.69225800
H	-2.05997000	5.39834900	-1.01723500
C	1.62879700	-2.76793700	-1.48128400
C	2.93909600	-2.86149700	-2.27651000
H	2.76852400	-2.65925300	-3.34086200
C	3.76389100	-1.74550700	-1.61898100
H	2.46181400	-0.38411800	-2.64339600
C	2.65901100	-0.66368500	-1.58934900
Br	2.97594900	1.02650000	-0.69971200
H	-4.71965700	1.14620200	1.51867900
H	-2.95722700	3.29488300	1.79816500
H	4.60006100	-1.42447800	-2.24174500
C	4.24586000	-2.20638300	-0.22985400
C	1.92711600	-3.27264600	-0.01959000
C	3.03046900	-2.46925000	0.66932100

H	3.33251700	-2.99700200	1.57829600
H	2.62762700	-1.49982200	1.00167300
H	3.41082300	-3.84483000	-2.19020600
H	2.22827400	-4.32006700	-0.14514000
H	1.00523700	-3.25472900	0.56868300
H	0.76509100	-3.31523000	-1.86749400
C	5.29191000	-1.31060800	0.46782500
H	4.77035100	-0.44455600	0.89496600
H	4.74324700	-3.17191200	-0.41801500
C	5.95963400	-2.07304800	1.61289300
H	6.50020900	-2.95005900	1.23468300
H	6.68601800	-1.43402100	2.12538500
H	5.24366100	-2.41790300	2.36581900
C	6.35644400	-0.78747900	-0.49349400
H	7.14633700	-0.27111200	0.06154700
H	6.83004400	-1.60636900	-1.05058800
H	5.94795900	-0.07046800	-1.21316000

Cartesian coordinates of the optimized geometry for Int-**23** at PBE0-D3BJ/6-31G*,def2-TZVP level of theory: (number of imaginary frequencies = 0):

Au	-0.28114300	-0.31044800	-0.51320800
C	1.10398700	-1.52275600	-1.74311300
C	-1.29796000	1.14672800	0.43331500
C	-2.97305900	2.20663900	1.48380700
C	-1.95610900	3.09418900	1.32962000
N	-2.54650800	1.01936600	0.92352200
N	-0.93623400	2.41924800	0.68603100
C	0.34851500	2.95450900	0.33470400
C	1.36283700	2.91974600	1.30280000
C	0.53075800	3.44541900	-0.96540200
C	2.61930800	3.39083500	0.91906700
C	1.80663900	3.90921200	-1.29354400
C	2.83956700	3.87781600	-0.36460100
H	3.43599800	3.37823700	1.63477800
H	1.99183600	4.30004200	-2.28997700
H	3.82478100	4.24229600	-0.64083400
C	-3.27757900	-0.21527500	0.86176100
C	-3.08437400	-1.14885100	1.88964400
C	-4.09437900	-0.44649700	-0.25378600
C	-3.76505700	-2.36251200	1.77851400
C	-4.74847800	-1.67793200	-0.31550800
C	-4.58783600	-2.62417500	0.68965200
H	-3.64732400	-3.11208500	2.55591800
H	-5.39000500	-1.89887400	-1.16363300
H	-5.10987600	-3.57448400	0.62371300
C	-0.58555600	3.47948900	-1.99016100
H	-1.50270700	3.11561500	-1.51271100
C	-0.28397400	2.54866200	-3.16730700
H	0.62856700	2.85246100	-3.69247500
H	-0.15123400	1.51358600	-2.83016800
H	-1.10794500	2.56584000	-3.88872400
C	-0.85463000	4.90807100	-2.46888000
H	-1.70261200	4.92234800	-3.16168600
H	-1.08649600	5.57265900	-1.63031100
H	0.01077200	5.32429500	-2.99585500
C	-4.24695300	0.56280600	-1.37361800
H	-3.72040700	1.47989900	-1.08466000
C	-5.71341700	0.93224100	-1.60527200
H	-5.79159100	1.70610400	-2.37613300
H	-6.29695600	0.06944500	-1.94407500
H	-6.17928200	1.31233800	-0.69024800
C	-3.59416900	0.04557500	-2.65868700
H	-2.53276000	-0.17972300	-2.50159200
H	-4.08254200	-0.87021700	-3.00971700

H	-3.67085900	0.79394600	-3.45508800
C	-2.17039600	-0.88817700	3.07005900
H	-1.74749800	0.11770800	2.96432800
C	1.12975800	2.37399900	2.69816500
H	0.04869600	2.27437000	2.84992600
C	1.74055400	0.97788200	2.84472100
H	1.31842400	0.28004500	2.11317400
H	2.82576700	1.00642800	2.69538700
H	1.54886600	0.57928700	3.84708200
C	1.65265200	3.32000900	3.78016500
H	1.38139900	2.94183100	4.77126800
H	2.74418500	3.40550200	3.75216500
H	1.23364900	4.32550400	3.67106800
C	-1.00054100	-1.87527700	3.08641000
H	-1.35019100	-2.90664900	3.20550700
H	-0.42242800	-1.82307800	2.15653700
H	-0.32522600	-1.65275900	3.91951500
C	-2.94477800	-0.92306500	4.38966600
H	-3.37115500	-1.91462400	4.57679000
H	-2.28032400	-0.68409500	5.22681300
H	-3.76777400	-0.20086000	4.38882000
C	0.24999900	-2.27597400	-1.24873300
Br	-0.77260300	-3.67072200	-0.76276200
H	-1.85905000	4.13014700	1.61461900
H	-3.94850600	2.30507300	1.93388900
C	2.24960000	-0.94831800	-2.47196900
C	3.33892100	-2.02863900	-2.60542700
C	2.83728800	0.29169700	-1.78639400
H	1.87780400	-0.67097800	-3.46807300
C	3.97425900	-2.36118000	-1.25894200
H	4.09784000	-1.62950000	-3.29114800
H	2.92676500	-2.92913500	-3.07416700
C	3.47126800	-0.04702700	-0.44367100
H	3.59729800	0.70167600	-2.46388400
H	2.07335800	1.06779200	-1.66550000
C	4.55450500	-1.12451300	-0.56067100
H	4.75282200	-3.11298100	-1.42034200
H	3.21869900	-2.82992900	-0.61365600
H	3.87937700	0.87362000	-0.01756800
H	2.68629500	-0.38360400	0.24850900
C	5.26159000	-1.44343700	0.78659200
H	5.33733700	-0.71822300	-1.22378700
C	6.43261500	-2.40153500	0.53829000
H	7.00105900	-2.55397700	1.46254200
H	6.09879300	-3.38755700	0.19928300
H	7.12213300	-1.99850800	-0.21360000
C	5.83483300	-0.15349000	1.38638200
H	6.46152100	0.37910200	0.66017100
H	5.05010100	0.53233100	1.72460400
H	6.45868400	-0.38518500	2.25673100
C	4.31033000	-2.08040000	1.80560200
H	3.45118700	-1.43654700	2.02681400
H	3.93054100	-3.04917900	1.46303000
H	4.83757300	-2.25536900	2.75034800

Cartesian coordinates of the optimized geometry for Int-**24** at PBE0-D3BJ/6-31G*,def2-TZVP level of theory: (number of imaginary frequencies = 0):

Au	-0.27626700	-0.37136600	-0.53313300
C	1.31402100	-1.19999400	-1.36717800
C	-1.81061300	0.57290100	0.41729700
C	-2.97043000	2.16025400	1.50895400
C	-3.78125300	1.07643700	1.37484500
N	-1.77178800	1.82854400	0.91639400
N	-3.04896400	0.11948300	0.70619600

C	-3.50679100	-1.19587900	0.35931400
C	-4.10203600	-1.38080800	-0.89538100
C	-3.29939400	-2.23139600	1.28080700
C	-4.49850100	-2.67851200	-1.22332100
C	-3.71406500	-3.50900800	0.90175700
C	-4.30643900	-3.73031700	-0.33593100
H	-4.96241000	-2.86704700	-2.18712100
H	-3.57089000	-4.34145000	1.58455500
H	-4.62351200	-4.73225900	-0.61049300
C	-0.61401900	2.67596700	0.84165400
C	-0.46671700	3.50153500	-0.28113600
C	0.32167700	2.61114000	1.88282000
C	0.67665700	4.29995500	-0.33651200
C	1.44707100	3.43028700	1.77733700
C	1.62148800	4.26865200	0.68244900
H	0.83044300	4.95235400	-1.19102800
H	2.19728100	3.40910500	2.56256500
H	2.50205200	4.90194300	0.62184100
C	-2.62409700	-2.00329400	2.61862800
H	-2.52549500	-0.92261700	2.77410100
C	-1.21113600	-2.59385400	2.61450500
H	-1.23905900	-3.67768300	2.45285900
H	-0.60272100	-2.14654400	1.81947900
H	-0.71255400	-2.40688800	3.57207700
C	-3.45190600	-2.55552800	3.78011600
H	-2.97547900	-2.30737200	4.73444500
H	-4.46353800	-2.13708400	3.78381900
H	-3.54073400	-3.64613000	3.73186700
C	0.16305100	1.67674200	3.06491200
H	-0.81565800	1.18872400	2.98969900
C	0.19566700	2.43332600	4.39400800
H	0.02091100	1.74398400	5.22691600
H	1.16648900	2.91249400	4.56057100
H	-0.57206600	3.21321500	4.42862200
C	1.22612600	0.57596900	3.02349300
H	1.16892700	0.01350500	2.08488600
H	2.23575000	0.99482600	3.09928000
H	1.08650600	-0.12417800	3.85464000
C	-1.46908400	3.51485900	-1.41700800
H	-2.33125400	2.90539100	-1.12222200
C	-4.28658600	-0.24482800	-1.88106000
H	-4.02213700	0.69261200	-1.37806700
C	-3.33884600	-0.40794600	-3.07258600
H	-2.29421000	-0.45323000	-2.74221300
H	-3.55701000	-1.32822300	-3.62612000
H	-3.44345000	0.43511600	-3.76442200
C	-5.74024600	-0.12054500	-2.34088000
H	-5.85551300	0.74814300	-2.99769300
H	-6.06268100	-1.00308800	-2.90381500
H	-6.41947500	0.00076300	-1.49079600
C	-0.86353500	2.87570900	-2.66980000
H	0.00715200	3.44155700	-3.01963100
H	-0.53604300	1.84910400	-2.46762900
H	-1.59943500	2.84914700	-3.48101100
C	-1.98507600	4.92550300	-1.70591700
H	-1.18422500	5.58369000	-2.05973000
H	-2.75349800	4.89449500	-2.48558700
H	-2.42286800	5.38182900	-0.81210300
C	1.60715200	-2.61017500	-1.68096300
C	2.92637000	-2.60732100	-2.46895900
H	2.75564900	-2.34413900	-3.52014800
C	3.70542300	-1.50394600	-1.73168000
H	2.34694800	-0.21939400	-2.75184000
C	2.55193900	-0.46880600	-1.69042600
Br	2.76992700	1.26129000	-0.85870900
H	-4.79809400	0.89906900	1.68871400

H	-3.13292600	3.12431000	1.96516000
H	4.53880300	-1.11750800	-2.31938000
C	4.18259000	-2.07218700	-0.37387100
C	1.90301500	-3.20138700	-0.25448300
C	2.96501600	-2.39796100	0.49576200
H	3.27040700	-2.95352100	1.38869800
H	2.51983400	-1.46015600	0.85932800
H	3.43192800	-3.57674600	-2.44217200
H	2.24549400	-4.22745000	-0.43716000
H	0.97296700	-3.25182700	0.31912500
H	0.76512700	-3.15576400	-2.11544900
C	5.33281200	-1.35112400	0.40763200
H	4.62198900	-3.03734600	-0.66909800
C	6.19853600	-2.45253500	1.03701900
H	6.67774300	-3.07048300	0.26827200
H	6.98986800	-2.01534300	1.65557800
H	5.60563000	-3.11338700	1.68123400
C	6.21358000	-0.52884900	-0.53547100
H	7.08082500	-0.14083600	0.00976400
H	6.59736400	-1.13422200	-1.36617000
H	5.67462400	0.33042200	-0.95028200
C	4.84290200	-0.44628200	1.54462500
H	4.18007700	0.34640500	1.19133300
H	4.31719100	-1.01380300	2.32050800
H	5.70597000	0.02959900	2.02340000

Cartesian coordinates of the optimized geometry for Int-**25** at PBE0-D3BJ/6-31G*,def2-TZVP level of theory: (number of imaginary frequencies = 0):

Au	-0.22065200	-0.64759300	-0.25448300
C	0.54596700	-2.62724500	-0.88903300
C	-0.55090700	1.30710400	0.09145900
C	-1.68695500	3.21953900	0.39012300
C	-0.36198000	3.51710300	0.42050100
N	-1.77789200	1.85835500	0.18479700
N	0.31629600	2.32682800	0.23825800
C	1.74471500	2.18402600	0.23094000
C	2.39458900	2.01760000	1.46260800
C	2.41273200	2.22231600	-1.00068600
C	3.78563000	1.90843600	1.43473600
C	3.80368600	2.10152800	-0.97066800
C	4.48276300	1.95742900	0.23306600
H	4.33045000	1.78376600	2.36574600
H	4.36209700	2.12962300	-1.90188400
H	5.56618200	1.87935100	0.23542200
C	-2.98264100	1.08113300	0.09886700
C	-3.49554900	0.52678300	1.27994600
C	-3.54492400	0.86808400	-1.16748700
C	-4.63247100	-0.27433000	1.16135200
C	-4.67932300	0.05706300	-1.22808600
C	-5.21713200	-0.50748400	-0.07766200
H	-5.06407600	-0.72155700	2.05227200
H	-5.14660300	-0.13472100	-2.18966200
H	-6.10237200	-1.13307200	-0.14753500
C	1.68311600	2.36959300	-2.32059500
H	0.62293300	2.55206000	-2.10976100
C	1.77133100	1.07350600	-3.13148600
H	2.81234100	0.82242700	-3.36523400
H	1.33780300	0.23310800	-2.57594100
H	1.22670800	1.17320400	-4.07662400
C	2.20097200	3.56338500	-3.12501000
H	1.61251200	3.68622300	-4.04029400
H	2.13442600	4.49167200	-2.54852200
H	3.24658600	3.42751200	-3.42139900
C	-2.95628500	1.46281200	-2.43069200

H	-2.10508400	2.09387700	-2.14947600
C	-3.96920700	2.35581300	-3.15072100
H	-3.50871500	2.82029500	-4.02907000
H	-4.83554900	1.78127000	-3.49597800
H	-4.33650100	3.15226600	-2.49529000
C	-2.42606700	0.36541100	-3.35709200
H	-1.67307200	-0.25072100	-2.85176800
H	-3.23207100	-0.29742300	-3.69059700
H	-1.96557600	0.80700900	-4.24762100
C	-2.85172700	0.75027800	2.63356100
H	-2.01433900	1.44634700	2.50705700
C	1.63538800	1.90936900	2.76965400
H	0.59649800	2.20907800	2.58929300
C	1.61539200	0.45415700	3.24844700
H	1.17757700	-0.20690300	2.49161800
H	2.62914000	0.09596700	3.45992200
H	1.02552700	0.36073700	4.16686500
C	2.19542000	2.83952800	3.84624300
H	1.57254900	2.79060000	4.74543100
H	3.21223300	2.55660300	4.13871300
H	2.22104300	3.87928200	3.50433400
C	-2.27862600	-0.55647400	3.18803400
H	-3.06646700	-1.30205900	3.34258000
H	-1.53786600	-0.98783800	2.50503000
H	-1.79007500	-0.38034400	4.15247100
C	-3.83148600	1.38949400	3.61998500
H	-4.67915500	0.72811400	3.82943200
H	-3.33057300	1.59615800	4.57164100
H	-4.23139800	2.33193000	3.23180200
C	-0.58472700	-2.78096300	-0.40395500
Br	-2.15812500	-3.42099000	0.17832700
H	0.16088800	4.45106700	0.55501200
H	-2.56388800	3.83899400	0.49511200
C	1.87345800	-2.74788000	-1.49690700
H	2.08304000	-3.82372800	-1.58323500
H	1.82873500	-2.34866600	-2.51847900
C	3.00170200	-2.06114800	-0.71337500
C	3.10894500	-2.58728300	0.71725300
C	4.32130800	-2.24664100	-1.46368900
H	2.77966500	-0.98276800	-0.66875600
C	4.27122400	-1.93652900	1.46195000
H	3.25638200	-3.67757000	0.68556300
H	2.16870900	-2.41305900	1.25798600
C	5.48813900	-1.60688800	-0.71648700
H	4.51255400	-3.32382200	-1.58397800
H	4.23889100	-1.82702900	-2.47527500
C	5.58797300	-2.12966200	0.71434100
H	4.34052200	-2.34669400	2.47630600
H	4.06819500	-0.86156100	1.56551200
H	6.42239700	-1.79082100	-1.25925400
H	5.34182700	-0.51845600	-0.69495200
H	6.40405600	-1.62604600	1.24572900
H	5.84132400	-3.19926600	0.69445600

Cartesian coordinates of the optimized geometry for Int-**26a** at PBE0-D3BJ/6-31G*,def2-TZVP level of theory: (number of imaginary frequencies = 0):

Au	0.10296900	-0.40086900	-0.36013700
C	1.84813500	-1.16268400	-0.87343400
C	-1.61275100	0.49038900	0.28285900
C	-3.01441300	2.06358900	1.06655300
C	-3.71742800	0.90438600	0.95356000
N	-1.73145200	1.78567600	0.65007400
N	-2.83848200	-0.04212500	0.47421400
C	-3.15368500	-1.41699400	0.20815500

C	-3.59688800	-1.75736100	-1.07684800
C	-2.97085300	-2.34877400	1.23873100
C	-3.86303500	-3.10610500	-1.31842500
C	-3.25229800	-3.68356700	0.94300400
C	-3.69349600	-4.05813500	-0.32040700
H	-4.20638600	-3.41437900	-2.30161600
H	-3.12349200	-4.43915100	1.71264400
H	-3.90857900	-5.10218300	-0.52898200
C	-0.64278500	2.72277000	0.61725200
C	-0.43739100	3.45957800	-0.55703200
C	0.17321700	2.82984300	1.75131300
C	0.63520700	4.35224600	-0.56670500
C	1.23093900	3.73874200	1.68866300
C	1.45744300	4.49507000	0.54479700
H	0.83108400	4.94051800	-1.45832100
H	1.88699800	3.85276400	2.54679400
H	2.28352300	5.20002700	0.51812800
C	-2.46084600	-1.95424100	2.61026200
H	-2.41062300	-0.86023800	2.65730500
C	-1.04185400	-2.48576800	2.83053900
H	-1.02228800	-3.58111700	2.80203000
H	-0.35956100	-2.11465300	2.05640600
H	-0.65946000	-2.16634600	3.80633100
C	-3.40505400	-2.41490500	3.72213200
H	-3.04623800	-2.06041700	4.69423000
H	-4.41849400	-2.02948900	3.57031200
H	-3.46681200	-3.50733400	3.77239100
C	-0.03707000	1.98396100	2.99058300
H	-0.94895600	1.39132000	2.85306600
C	-0.23682000	2.84719300	4.23766700
H	-0.44051100	2.21476200	5.10831200
H	0.65569600	3.44114100	4.46239300
H	-1.07609500	3.53941300	4.11387200
C	1.12297800	1.00130200	3.17290900
H	1.24139200	0.36488000	2.28817500
H	2.06955400	1.53007900	3.33046100
H	0.94612700	0.35756400	4.04166600
C	-1.29900700	3.27966800	-1.79008100
H	-2.14009000	2.62629700	-1.53022800
C	-3.75053300	-0.72985600	-2.18010500
H	-3.62411100	0.26686300	-1.74152900
C	-2.65403400	-0.90807700	-3.23404500
H	-1.65733300	-0.83023700	-2.78358200
H	-2.72912300	-1.88849600	-3.71784400
H	-2.73973500	-0.14037500	-4.01099200
C	-5.14202000	-0.77484600	-2.81357700
H	-5.24436200	0.02328200	-3.55640200
H	-5.32227500	-1.72559700	-3.32666100
H	-5.92775400	-0.64567400	-2.06216500
C	-0.50234600	2.58280400	-2.89687700
H	0.35513000	3.19039100	-3.20711600
H	-0.11896400	1.61419800	-2.55514000
H	-1.13390800	2.41292100	-3.77585600
C	-1.88517900	4.60575500	-2.27720700
H	-1.10184400	5.29651000	-2.60731500
H	-2.55188300	4.43400200	-3.12885900
H	-2.45903800	5.10295200	-1.48836900
C	2.29395400	-2.56587100	-0.88917800
H	1.56708200	-3.25244800	-1.33934800
C	3.73552900	-2.60486300	-1.42372700
H	3.67688700	-2.61949900	-2.52113100
C	4.28093400	-1.22798100	-0.98690000
H	2.89363100	-0.26087800	-2.32837000
C	3.03725500	-0.36119100	-1.23300100
Br	3.04634000	1.45110000	-0.55341200
H	-4.74844500	0.66805700	1.16589900

H	-3.30456900	3.04774100	1.40000500
H	5.10668400	-0.90773000	-1.63036800
H	2.27395900	-2.82439900	0.19072800
C	4.57778200	-3.79216000	-0.96065300
H	4.03512900	-4.72510300	-1.15765900
H	5.48535400	-3.82838400	-1.57679000
C	4.73123900	-1.19922200	0.47550900
C	4.98493500	-3.69557400	0.50676600
C	5.66621600	-2.36132500	0.79388500
H	6.57962300	-2.27787900	0.18819300
H	5.97952600	-2.30594000	1.84200100
H	5.20959800	-0.23657500	0.68329500
H	3.85709500	-1.24917100	1.14259600
H	5.64872400	-4.53042300	0.75633100
H	4.10535600	-3.80302400	1.15969200

Cartesian coordinates of the optimized geometry for Int-**26b** at PBE0-D3BJ/6-31G*,def2-TZVP level of theory: (number of imaginary frequencies = 0):

Au	-0.08148300	-0.47694600	-0.15790100
C	-1.81187500	-1.36663000	-0.48523000
C	1.63790900	0.56577400	0.17203100
C	3.02050700	2.29642600	0.55675300
C	3.78095900	1.17148700	0.47447800
N	1.71474500	1.90063300	0.36979600
N	2.91314800	0.12779200	0.23797900
C	3.28694600	-1.24891000	0.07910100
C	3.32363400	-2.06043400	1.22086200
C	3.56588400	-1.71333900	-1.21320500
C	3.65752600	-3.40267300	1.03332700
C	3.89451500	-3.06365500	-1.34362900
C	3.94012600	-3.89862700	-0.23371800
H	3.69570200	-4.06786000	1.89107400
H	4.11686100	-3.46595700	-2.32773300
H	4.19998100	-4.94602000	-0.35719300
C	0.57095600	2.76991600	0.36426300
C	-0.10166000	2.98965500	1.57392500
C	0.17218000	3.33041600	-0.85643200
C	-1.21668900	3.82788600	1.53691000
C	-0.95058000	4.15983500	-0.83729400
C	-1.63474100	4.41034600	0.34633000
H	-1.76729700	4.02348800	2.45242300
H	-1.29302200	4.61432100	-1.76248000
H	-2.50280600	5.06342000	0.34052800
C	3.48715000	-0.81694000	-2.43256100
H	3.33512800	0.21399200	-2.09233400
C	2.28268100	-1.19384700	-3.29968100
H	2.37576100	-2.21764900	-3.67936000
H	1.35003600	-1.12923700	-2.72655200
H	2.20323000	-0.52031800	-4.16013900
C	4.78415000	-0.84193500	-3.24313500
H	4.72190700	-0.13536500	-4.07753100
H	5.64604900	-0.56768900	-2.62621700
H	4.97731600	-1.83371000	-3.66597000
C	0.88738300	3.03734500	-2.15947800
H	1.77685900	2.43649400	-1.93680900
C	1.36283900	4.31901300	-2.84608800
H	1.92583200	4.07613900	-3.75353300
H	0.52043200	4.95408100	-3.14085600
H	2.01098300	4.90772000	-2.18864100
C	-0.00507500	2.20692700	-3.08591300
H	-0.31483300	1.27327200	-2.60217900
H	-0.91258700	2.75690200	-3.35877900
H	0.52925900	1.95703400	-4.00917700
C	0.31919900	2.32858700	2.87045900

H	1.25662700	1.78857900	2.69338200
C	2.98424500	-1.53433400	2.60096300
H	2.88508300	-0.44438300	2.53843900
C	1.63649200	-2.08969500	3.07000800
H	0.83781900	-1.83446000	2.36326500
H	1.67056900	-3.18134400	3.15996500
H	1.37164700	-1.67789700	4.05017800
C	4.09061700	-1.83286100	3.61410300
H	3.84827800	-1.38113800	4.58184400
H	4.21138800	-2.90947200	3.77538700
H	5.05465200	-1.43395100	3.28218100
C	-0.72583500	1.29896500	3.30926000
H	-1.69144700	1.77690000	3.50873900
H	-0.88093800	0.54178700	2.53156300
H	-0.40325600	0.79113000	4.22488100
C	0.58473600	3.35608700	3.97215600
H	-0.32433000	3.90685700	4.23693100
H	0.94291100	2.85606900	4.87829900
H	1.33975900	4.08640600	3.66333000
C	-2.10168000	-2.68421700	-1.07790600
H	-1.41571600	-3.47888900	-0.76207800
H	-3.26952800	-0.95072600	0.85385900
C	-3.12560200	-0.74397100	-0.22419100
Br	-3.15825900	1.18500800	-0.37030900
H	4.84528700	1.01804100	0.56162400
H	3.28419400	3.32842500	0.72816500
H	-1.83132000	-2.48708700	-2.13934900
C	-4.18099000	-1.51186700	-1.03295000
C	-5.61529500	-1.35867700	-0.54129300
C	-3.61118000	-2.94443300	-0.96332300
H	-4.11074600	-1.15259300	-2.06879300
C	-5.86901800	-2.08811600	0.77440400
H	-6.28051000	-1.77097800	-1.31073100
H	-5.85886000	-0.29277800	-0.45890600
C	-3.96225900	-3.66445000	0.34423500
H	-3.97831100	-3.53787800	-1.80754900
C	-5.44700700	-3.55109000	0.67740000
H	-6.92996200	-2.00951000	1.03507300
H	-5.32150100	-1.60214300	1.59684000
H	-3.65741700	-4.71463200	0.26595500
H	-3.37872600	-3.24362800	1.17769200
H	-5.65780300	-4.07661200	1.61508500
H	-6.03849600	-4.05048500	-0.10290300

Cartesian coordinates of the optimized geometry for Int-**26c** at PBE0-D3BJ/6-31G*,def2-TZVP level of theory: (number of imaginary frequencies = 0):

Au	0.09709400	-0.44480000	-0.08752700
C	1.92157200	-1.18523700	-0.18664600
C	-1.73292900	0.44315900	0.04867100
C	-3.29866600	2.04611200	0.23072100
C	-3.93929200	0.84663000	0.19015300
N	-1.95174100	1.77377800	0.14105000
N	-2.96088700	-0.11700800	0.08013000
C	-3.18737000	-1.53245000	0.00536900
C	-3.34294600	-2.11138800	-1.26116100
C	-3.21101900	-2.26230300	1.20129300
C	-3.52825100	-3.49409500	-1.30693300
C	-3.39978500	-3.64142500	1.09781200
C	-3.55679000	-4.25039700	-0.14142800
H	-3.65107700	-3.98440600	-2.26828800
H	-3.42356600	-4.24584000	1.99998000
H	-3.70445400	-5.32493600	-0.19941500
C	-0.89875200	2.75111200	0.16033900
C	-0.46078700	3.27914700	-1.06168100

C	-0.34611500	3.10313600	1.39888900
C	0.57419100	4.21396600	-1.01437400
C	0.68696700	4.04200500	1.38900300
C	1.13950400	4.59488900	0.19683200
H	0.94527300	4.64590600	-1.93915700
H	1.14295800	4.34254500	2.32791800
H	1.94122400	5.32776700	0.21144800
C	-3.01004600	-1.61046500	2.55453100
H	-2.99666900	-0.52337800	2.41407400
C	-1.65678600	-2.01063600	3.14873800
H	-1.60651600	-3.09163100	3.32161900
H	-0.83556400	-1.73743800	2.47540800
H	-1.49493200	-1.50727300	4.10830800
C	-4.15546700	-1.93117900	3.51633500
H	-4.01721000	-1.39489500	4.46113000
H	-5.12341000	-1.63959500	3.09592000
H	-4.20016600	-3.00065000	3.74863200
C	-0.80650900	2.48275000	2.70222000
H	-1.66488800	1.83443500	2.49105400
C	-1.27116200	3.54407100	3.70126100
H	-1.65121000	3.06754900	4.61116600
H	-0.45017300	4.20730400	3.99434500
H	-2.06906400	4.16554200	3.28170900
C	0.29566900	1.60208700	3.29690500
H	0.60144900	0.82243100	2.58951400
H	1.18431600	2.19305800	3.54484500
H	-0.05512800	1.11764900	4.21485300
C	-1.03916900	2.83950300	-2.39120400
H	-1.90062500	2.19062100	-2.19459300
C	-3.28180200	-1.29754500	-2.53807100
H	-3.25861600	-0.23536000	-2.26788800
C	-1.99330600	-1.60160200	-3.30748700
H	-1.10915600	-1.39971400	-2.69110900
H	-1.95686700	-2.65291700	-3.61458100
H	-1.93026200	-0.98366800	-4.21001400
C	-4.51648400	-1.51734100	-3.41385600
H	-4.47610500	-0.86663700	-4.29378200
H	-4.57909200	-2.55054700	-3.77196500
H	-5.43883500	-1.29631400	-2.86694800
C	-0.01267200	2.01315200	-3.17129700
H	0.87884300	2.60723400	-3.40129300
H	0.30794000	1.13856400	-2.59303100
H	-0.44067700	1.66266800	-4.11698500
C	-1.54022500	4.02623600	-3.21590400
H	-0.72096800	4.69671600	-3.49697300
H	-2.00842200	3.67332300	-4.14087800
H	-2.27868200	4.61460900	-2.66158200
C	2.40753800	-2.54796700	0.11232500
H	1.73917200	-3.34531000	-0.23092700
C	3.88447900	-2.59624200	-0.28935900
H	3.93116800	-2.74666600	-1.38026800
C	4.33034300	-1.16172400	0.00554600
H	3.13702900	-0.40783100	-1.62878900
C	3.12676400	-0.38404200	-0.52115700
Br	3.00891400	1.48622100	-0.03919000
H	-4.98704500	0.59223000	0.22805400
H	-3.67170500	3.05498300	0.31418800
H	2.32417200	-2.56820300	1.22247600
C	4.82094300	-3.59656600	0.36710800
H	4.51885000	-4.62611600	0.14239300
C	6.24605200	-3.33469300	-0.13515800
C	5.71231100	-0.87305000	-0.54476000
C	6.69005500	-1.88270600	0.06863600
H	6.01732500	0.15393000	-0.31487000
H	5.70114200	-0.97277300	-1.63979100
H	7.68889200	-1.73742000	-0.35663200

H	6.77862300	-1.67838000	1.14496700
H	6.94677600	-4.01004000	0.36756200
H	6.29304200	-3.58032500	-1.20555200
H	4.35157200	-1.02883200	1.09913600
H	4.77695800	-3.47734000	1.45938200

Cartesian coordinates of the optimized geometry for Int-**27** at PBE0-D3BJ/6-31G*,def2-TZVP level of theory: (number of imaginary frequencies = 0):

Au	-0.14609100	0.47511700	-0.21349800
C	1.71632000	1.55161000	-0.65235900
C	-1.26933700	-1.15001600	0.16065600
C	-2.94212200	-2.57080400	0.61320700
C	-1.80721400	-3.30516700	0.47671800
N	-2.58631900	-1.25054600	0.41435100
N	-0.79222300	-2.41055000	0.19965900
C	0.59218700	-2.71792100	-0.02580100
C	1.01991600	-2.92007000	-1.34594100
C	1.45582600	-2.73448600	1.07878200
C	2.38152700	-3.15969400	-1.54308900
C	2.80661400	-2.98130100	0.82481200
C	3.26483600	-3.19258200	-0.47081500
H	2.75264400	-3.32589300	-2.55041000
H	3.50863300	-3.00811800	1.65345100
H	4.31982400	-3.38417600	-0.64490700
C	-3.46418500	-0.11510100	0.46318300
C	-4.08549900	0.29105200	-0.72573400
C	-3.61435900	0.55189000	1.68668600
C	-4.89877200	1.42338400	-0.65930400
C	-4.43917900	1.67788100	1.69575700
C	-5.07462800	2.10795100	0.53722100
H	-5.39799300	1.77502000	-1.55746700
H	-4.58309500	2.22606100	2.62230000
H	-5.71273700	2.98637300	0.56682100
C	0.97893000	-2.46650500	2.49171700
H	-0.11142100	-2.35701200	2.47261600
C	1.56089400	-1.15146000	3.01749100
H	2.65510500	-1.19313600	3.06731900
H	1.28294400	-0.30879000	2.37334300
H	1.18822000	-0.94296000	4.02613200
C	1.30663200	-3.63231500	3.42683800
H	0.90142200	-3.44136900	4.42603500
H	0.88103500	-4.57152700	3.05886100
H	2.38802400	-3.77302600	3.53035300
C	-2.89863500	0.11100600	2.94745300
H	-2.38492500	-0.83491800	2.73899000
C	-3.87829900	-0.14603100	4.09406200
H	-3.34265500	-0.52062500	4.97274800
H	-4.39899900	0.77045600	4.39166700
H	-4.63592600	-0.88516700	3.81409600
C	-1.83164600	1.13404100	3.34726700
H	-1.10715600	1.28894000	2.53893100
H	-2.28287700	2.10438900	3.58216700
H	-1.28645300	0.79289900	4.23416800
C	-3.86799100	-0.42645800	-2.04264300
H	-3.29832200	-1.34234700	-1.84568400
C	0.07341000	-2.86646100	-2.52778900
H	-0.93885600	-2.68134600	-2.15064000
C	0.43080900	-1.70819000	-3.46273600
H	0.41128200	-0.74908500	-2.93186300
H	1.43003400	-1.83718900	-3.89369500
H	-0.28398900	-1.65130100	-4.29059400
C	0.04693900	-4.19959200	-3.27896900
H	-0.67954900	-4.15951000	-4.09736300
H	1.02463400	-4.43341100	-3.71427200

H	-0.22973700	-5.02596300	-2.61612400
C	-3.03358100	0.43748600	-2.99277300
H	-3.55381800	1.37034300	-3.23689800
H	-2.06743100	0.70009600	-2.54576000
H	-2.84262600	-0.09703600	-3.92985200
C	-5.19007300	-0.84387300	-2.68904700
H	-5.79582500	0.02547300	-2.96622800
H	-4.99956400	-1.41630400	-3.60297700
H	-5.78651200	-1.46578700	-2.01366400
C	2.93961800	0.75613500	-0.76152800
H	2.90656200	0.18680200	-1.69988900
C	4.22187800	1.62753300	-0.72536900
C	4.28362300	2.46687100	0.55997100
H	3.40408500	3.12092600	0.62408800
C	0.80724200	2.39384700	-0.58069000
Br	-0.12445600	3.93055900	-0.57735900
H	-1.62679700	-4.36625100	0.54967000
H	-3.95936700	-2.85694100	0.83019000
H	5.15291300	3.13370200	0.47562000
H	2.94576900	0.01403900	0.04387700
C	5.43041500	0.67575800	-0.76872400
H	5.35814700	0.02672500	-1.65232700
H	6.32714600	1.29414400	-0.91391100
C	4.42709300	1.62995700	1.82850300
C	5.60777200	-0.15957900	0.49846900
C	5.64859500	0.71758400	1.74746900
H	6.55520500	1.33857000	1.72102800
H	5.72360800	0.09572600	2.64752300
H	4.50531000	2.29366700	2.69718200
H	3.52189900	1.02617400	1.98949100
H	6.52972000	-0.74743900	0.41670100
H	4.79151400	-0.88992000	0.58917900
C	4.23612800	2.54077600	-1.94869000
H	5.15235400	3.14042400	-1.96521700
H	4.19803800	1.96134600	-2.87898100
H	3.38736000	3.23515800	-1.94553500

Cartesian coordinates of the optimized geometry for Int-**28a** at PBE0-D3BJ/6-31G*,def2-TZVP level of theory: (number of imaginary frequencies = 0):

Au	0.07652300	-0.36251600	-0.26283800
C	1.90562300	-0.99925300	-0.63705200
C	-1.74377000	0.41372000	0.22227800
C	-3.31811400	1.90693600	0.81037500
C	-3.91295500	0.68531500	0.74380800
N	-1.99316500	1.71539300	0.48667600
N	-2.92973400	-0.21051800	0.38430600
C	-3.11033200	-1.62305500	0.20415400
C	-3.43683500	-2.09071500	-1.07570400
C	-2.91796600	-2.46028800	1.31127600
C	-3.57134300	-3.47143500	-1.23024100
C	-3.06499200	-3.83230200	1.10090700
C	-3.38819500	-4.33226300	-0.15480600
H	-3.82146200	-3.87722800	-2.20613900
H	-2.92367100	-4.51767800	1.93164200
H	-3.49973200	-5.40338700	-0.29646000
C	-0.98698300	2.74000700	0.44310600
C	-0.77577500	3.41014600	-0.76942000
C	-0.25002600	2.99492500	1.60729500
C	0.21645000	4.39123900	-0.78808900
C	0.72957500	3.98674000	1.53383900
C	0.95769600	4.68084400	0.35147200
H	0.41395500	4.93258400	-1.70863800
H	1.32283700	4.21594700	2.41436900
H	1.72176700	5.45225800	0.31682800

C	-2.53389500	-1.92603000	2.67652300
H	-2.58839100	-0.83163600	2.64546800
C	-1.08825100	-2.30147900	3.01425100
H	-0.96557500	-3.38928700	3.06572500
H	-0.39545300	-1.91814400	2.25551900
H	-0.79966900	-1.88260300	3.98467300
C	-3.49889700	-2.39906800	3.76503700
H	-3.23662100	-1.94481600	4.72640700
H	-4.53240900	-2.12614600	3.52836100
H	-3.46111500	-3.48615600	3.89364200
C	-0.46097200	2.22054500	2.89234300
H	-1.30900300	1.54067300	2.75007400
C	-0.80836500	3.14630900	4.05968200
H	-1.00905200	2.55973000	4.96253200
H	0.01491600	3.83202700	4.28737300
H	-1.69487200	3.75027200	3.84007900
C	0.76557500	1.36039800	3.20925900
H	0.98999800	0.67585400	2.38286800
H	1.65279700	1.98111200	3.37656500
H	0.59270500	0.76640300	4.11352600
C	-1.54523500	3.07069300	-2.02949400
H	-2.33751800	2.35955000	-1.76805900
C	-3.60296800	-1.16073700	-2.26058800
H	-3.59094300	-0.12854700	-1.89149400
C	-2.42743500	-1.31373300	-3.22993800
H	-1.47317500	-1.11693200	-2.72680900
H	-2.38746100	-2.32821100	-3.64245600
H	-2.52516700	-0.61239200	-4.06595900
C	-4.94017400	-1.37379200	-2.97197900
H	-5.05979300	-0.64161600	-3.77751800
H	-5.00455900	-2.37022900	-3.42216000
H	-5.78250400	-1.26329900	-2.28137600
C	-0.62687000	2.38125500	-3.04263600
H	0.18756100	3.04585900	-3.35181400
H	-0.17662600	1.47817400	-2.61404200
H	-1.18952200	2.09462800	-3.93796600
C	-2.21947700	4.30177300	-2.63707700
H	-1.48370800	5.04044900	-2.97281700
H	-2.81738100	4.01404200	-3.50831400
H	-2.88070500	4.79238800	-1.91535700
C	2.47100200	-2.35075600	-0.50787100
H	1.81914900	-3.14181700	-0.89887800
C	3.93083000	-2.32926800	-1.00854700
C	4.33681900	-0.86394200	-0.68320300
H	2.90933700	-0.08451000	-2.12049200
C	3.03287600	-0.12389900	-1.02235800
Br	2.86042500	1.72222300	-0.45678600
H	-4.93149000	0.37396200	0.91579000
H	-3.70991600	2.88203100	1.05452900
H	5.15062200	-0.53451500	-1.33822400
H	2.44117900	-2.49460700	0.59384600
C	4.82142800	-3.37278400	-0.32174600
H	4.35103100	-4.35937100	-0.42713200
H	5.76605500	-3.42600500	-0.87999500
C	4.73378800	-0.63708600	0.77953800
C	5.14048600	-3.07380900	1.13710600
C	5.72957400	-1.67599900	1.27605600
H	6.66063100	-1.60933700	0.69582100
H	5.99286100	-1.46605200	2.31839800
H	5.13403100	0.37681300	0.88126100
H	3.83922100	-0.66841300	1.42049100
H	5.83448700	-3.83058600	1.51890300
H	4.23577000	-3.15222000	1.75949600
C	3.95219100	-2.59548500	-2.51770100
H	4.95878100	-2.44205600	-2.92056600
H	3.66890800	-3.63428300	-2.72063000

H	3.26441300	-1.95782300	-3.08513500

Cartesian coordinates of the optimized geometry for Int-**28b** at PBE0-D3BJ/6-31G*,def2-TZVP level of theory: (number of imaginary frequencies = 0):

Au	-0.05008800	-0.39519300	-0.11328000
C	-1.88259200	-1.09118200	-0.34188700
C	1.78896600	0.44898000	0.12577400
C	3.37840900	2.01743900	0.38672800
C	3.99499200	0.80505200	0.36607400
N	2.03074400	1.77378300	0.24011800
N	3.00254200	-0.13700900	0.20415300
C	3.20131600	-1.55669900	0.13183400
C	3.14706200	-2.29348000	1.32253000
C	3.40724600	-2.13247400	-1.12894200
C	3.30926600	-3.67604000	1.21982300
C	3.56330800	-3.51879000	-1.17424500
C	3.51536100	-4.28181300	-0.01372900
H	3.27280100	-4.28565000	2.11805200
H	3.72345400	-4.00668100	-2.13133900
H	3.64126100	-5.35915700	-0.07113800
C	1.00023500	2.77393600	0.19542600
C	0.39209100	3.15489000	1.39926400
C	0.63723100	3.29414600	-1.05395200
C	-0.61579000	4.11706200	1.32438900
C	-0.37755800	4.25265800	-1.07197300
C	-0.99404900	4.66365900	0.10382900
H	-1.11450100	4.43880700	2.23392400
H	-0.68897800	4.68120400	-2.02022600
H	-1.77803200	5.41480100	0.06858400
C	3.42630100	-1.31110600	-2.40238400
H	3.41718900	-0.25005000	-2.12711900
C	2.16715500	-1.57854400	-3.23175100
H	2.11820500	-2.62740600	-3.54561700
H	1.26164200	-1.35573700	-2.65490500
H	2.16128300	-0.95547600	-4.13294600
C	4.69392300	-1.55770700	-3.22203900
H	4.71083700	-0.90098500	-4.09825000
H	5.59535600	-1.36379900	-2.63171400
H	4.74668600	-2.59002500	-3.58429800
C	1.27571800	2.82898200	-2.34678400
H	2.08809600	2.13554500	-2.10014600
C	1.89015100	3.99311300	-3.12621100
H	2.39275100	3.62324500	-4.02622800
H	1.12674600	4.71061600	-3.44594800
H	2.62534800	4.53463600	-2.52193800
C	0.26214500	2.06057600	-3.19906800
H	-0.15138000	1.20872900	-2.64661700
H	-0.57468800	2.70314600	-3.49439900
H	0.73706800	1.68320700	-4.11142000
C	0.76254900	2.53242500	2.72995100
H	1.63829600	1.89065300	2.57842800
C	2.89243700	-1.64437600	2.66800300
H	2.91216200	-0.55653000	2.53426200
C	1.50131100	-2.01534600	3.18924500
H	0.72221500	-1.71993000	2.47635600
H	1.41669800	-3.09569000	3.35233100
H	1.30217700	-1.51325700	4.14248000
C	3.97905700	-1.99766400	3.68506000
H	3.80506400	-1.46331500	4.62503700
H	3.98636600	-3.06911300	3.91235300
H	4.97399900	-1.72708500	3.31685200
C	-0.37582100	1.64121300	3.23527600
H	-1.28686900	2.22532500	3.40702400
H	-0.61292000	0.85733900	2.50621700

H	-0.09708200	1.16048700	4.17953600
C	1.14470900	3.58837200	3.76836600
H	0.29817400	4.23910600	4.01263900
H	1.46748400	3.10615300	4.69714800
H	1.96198900	4.22299300	3.41051600
C	-2.34501100	-2.38433000	-0.86601100
H	-1.73432000	-3.24361600	-0.56448200
H	-3.20797400	-0.44284200	1.04091900
C	-3.10467800	-0.31416700	-0.05331900
Br	-2.93171800	1.59731700	-0.31024300
H	5.03423800	0.52811100	0.45093200
H	3.76866000	3.01787900	0.49041800
H	-2.11426700	-2.24331700	-1.94744100
C	-4.28099400	-0.99501300	-0.76340800
C	-5.65310600	-0.66727400	-0.18712000
C	-3.87137900	-2.48774600	-0.68315900
H	-4.24408600	-0.68134300	-1.81630400
C	-5.86947400	-1.27462600	1.19558000
H	-6.41366200	-1.05803900	-0.87516300
H	-5.78574500	0.42096000	-0.16672900
C	-4.18026800	-3.07312700	0.70970100
C	-5.60154000	-2.77649300	1.18227500
H	-6.89246900	-1.06936700	1.52894900
H	-5.21254200	-0.79042500	1.93466800
H	-3.99741500	-4.15458900	0.68413400
H	-3.47590600	-2.66921000	1.45286300
H	-5.74943200	-3.20192200	2.18094200
H	-6.32871400	-3.27199100	0.52561500
C	-4.51997600	-3.32151700	-1.78178800
H	-4.31442200	-2.89982800	-2.77253100
H	-4.14248400	-4.35005900	-1.76380900
H	-5.60649200	-3.36771700	-1.66090300

Cartesian coordinates of the optimized geometry for Int-**28c** at PBE0-D3BJ/6-31G*,def2-TZVP level of theory: (number of imaginary frequencies = 0):

Au	0.04878200	-0.37921800	-0.05006800
C	1.92473900	-0.98137800	-0.09793500
C	-1.84591900	0.36876900	0.03153200
C	-3.53179900	1.85228800	0.13831400
C	-4.07837500	0.60651200	0.13504800
N	-2.16685700	1.68107300	0.07293900
N	-3.02778300	-0.28252600	0.07064200
C	-3.14378400	-1.71307900	0.04685100
C	-3.24250100	-2.34831700	-1.19828400
C	-3.12017900	-2.39857100	1.26874200
C	-3.31899000	-3.74198800	-1.19390900
C	-3.20006900	-3.79113000	1.21531200
C	-3.29857800	-4.45525300	-0.00136600
H	-3.39425500	-4.27518600	-2.13711500
H	-3.18408900	-4.36221900	2.13913700
H	-3.36143500	-5.53945600	-0.02043800
C	-1.19108600	2.73565100	0.06801300
C	-0.76999600	3.24439800	-1.16802800
C	-0.68962700	3.17850700	1.29918100
C	0.19054900	4.25631100	-1.14384400
C	0.27043000	4.19145200	1.26600500
C	0.70262700	4.72805400	0.05893300
H	0.54564500	4.67689100	-2.08009100
H	0.68508200	4.56331400	2.19853300
H	1.44693100	5.51928600	0.05534100
C	-2.98197400	-1.68464800	2.59831500
H	-3.04812300	-0.60543200	2.41785500
C	-1.60873900	-1.96068700	3.21671100
H	-1.47949900	-3.02797800	3.42915900

H	-0.80366700	-1.65077100	2.53976400
H	-1.49456200	-1.41259300	4.15846300
C	-4.11014300	-2.05518300	3.56262100
H	-4.02184900	-1.47650900	4.48820500
H	-5.09271400	-1.85195200	3.12451000
H	-4.07763600	-3.11601300	3.83340100
C	-1.12464400	2.57683800	2.61993900
H	-1.93504800	1.86501200	2.42447600
C	-1.67153900	3.63932000	3.57496300
H	-2.03213500	3.17053500	4.49671500
H	-0.90006800	4.36500800	3.85421200
H	-2.50256900	4.19180100	3.12436600
C	0.02671900	1.79410800	3.25713300
H	0.39162800	1.01128900	2.58204200
H	0.87122000	2.45205400	3.49045400
H	-0.30148400	1.32134700	4.18943600
C	-1.28867200	2.70904600	-2.48697200
H	-2.09286400	1.99437700	-2.27652400
C	-3.23011000	-1.57903100	-2.50391100
H	-3.30594700	-0.50994500	-2.27346700
C	-1.90499700	-1.79526400	-3.24038300
H	-1.05421900	-1.49337200	-2.61791700
H	-1.77041100	-2.84990100	-3.50606400
H	-1.87906900	-1.20770700	-4.16480200
C	-4.42300500	-1.93844100	-3.39123900
H	-4.42395500	-1.31610700	-4.29234400
H	-4.38590200	-2.98417200	-3.71490300
H	-5.37186500	-1.78351800	-2.86748300
C	-0.18340500	1.94913100	-3.22570400
H	0.65413600	2.61137500	-3.47181400
H	0.20689500	1.12967100	-2.61069800
H	-0.56730500	1.52573200	-4.16041000
C	-1.87986900	3.81806800	-3.35893100
H	-1.11879500	4.54917600	-3.65220000
H	-2.30036700	3.39423800	-4.27704800
H	-2.67619700	4.35650500	-2.83477800
C	2.50628300	-2.27139600	0.32186600
H	1.88125800	-3.14067800	0.08764400
C	3.97865000	-2.28502900	-0.12741500
C	4.31695500	-0.79057500	0.06005500
H	3.06404100	-0.11660700	-1.59479400
C	3.06950100	-0.11219100	-0.49220500
Br	2.81588200	1.74672200	0.00363500
H	-5.10404000	0.27388600	0.17122300
H	-3.98184700	2.83186400	0.18101700
H	2.45422700	-2.17130100	1.43036100
C	4.95529300	-3.08958000	0.73127800
H	4.77235800	-4.16610700	0.62519100
C	6.40476700	-2.75755100	0.34865900
C	5.70016200	-0.41842500	-0.42937900
C	6.70771600	-1.25465100	0.37182800
H	5.88489500	0.65101600	-0.27686800
H	5.79668300	-0.61002900	-1.50578400
H	7.72277200	-1.07703000	0.00065200
H	6.69318400	-0.90272200	1.41307800
H	7.08400800	-3.27533500	1.03488200
H	6.62292600	-3.16139300	-0.64779300
H	4.29961400	-0.60564800	1.14687800
H	4.79043100	-2.83862900	1.78924200
C	4.02531200	-2.75884700	-1.58629700
H	3.75451400	-3.81946800	-1.63259600
H	3.32291200	-2.22042800	-2.23314700
H	5.01919600	-2.65642300	-2.02706600

Cartesian coordinates of the optimized geometry for Int-**29** at PBE0-D3BJ/6-31G*,def2-TZVP level of theory: (number of imaginary frequencies = 0):

Au	0.25315600	0.59662600	-0.24881100
C	-0.70575200	2.48435300	-0.89841900
C	0.74799400	-1.31231600	0.14919200
C	2.01932400	-3.12696200	0.50133700
C	0.71922400	-3.51859300	0.54758700
N	2.01162200	-1.76932600	0.25374500
N	-0.04292400	-2.38644700	0.33122800
C	-1.47697000	-2.32896000	0.31866300
C	-2.13735000	-2.13230300	1.54001900
C	-2.13674900	-2.45387700	-0.91169700
C	-3.53122700	-2.06918000	1.50219100
C	-3.53119800	-2.38284400	-0.89160500
C	-4.22010700	-2.19862600	0.30149100
H	-4.08463100	-1.92190300	2.42498700
H	-4.08452300	-2.48236700	-1.82104200
H	-5.30555800	-2.15900400	0.29624100
C	3.15540600	-0.90961300	0.12878100
C	3.62988800	-0.26990600	1.28238100
C	3.69357500	-0.70601300	-1.14956500
C	4.70249800	0.60904000	1.12279800
C	4.76344400	0.18461200	-1.25152500
C	5.26262300	0.83422500	-0.12894900
H	5.10272400	1.12418600	1.99137400
H	5.20997600	0.37221400	-2.22374900
H	6.09789300	1.52115500	-0.23073600
C	-1.39260500	-2.64644000	-2.21769100
H	-0.32744100	-2.77006000	-1.99053100
C	-1.52818800	-1.40938500	-3.10941200
H	-2.57270100	-1.23412400	-3.38990200
H	-1.16136400	-0.51174700	-2.59699300
H	-0.94889300	-1.53553000	-4.03045700
C	-1.85302600	-3.90895100	-2.94900600
H	-1.25636200	-4.06007400	-3.85470900
H	-1.74737900	-4.79702600	-2.31759400
H	-2.90265700	-3.83777400	-3.25387000
C	3.14235100	-1.39053600	-2.38387600
H	2.34382500	-2.07337700	-2.07092500
C	4.21409400	-2.23032900	-3.08234100
H	3.78296300	-2.75970900	-3.93860100
H	5.03181900	-1.60533700	-3.45720700
H	4.64567900	-2.97261700	-2.40294200
C	2.52279300	-0.37075300	-3.34294200
H	1.72904500	0.20425200	-2.85184000
H	3.27353000	0.33867900	-3.70809400
H	2.09012400	-0.87776200	-4.21233800
C	3.00863100	-0.48304400	2.64807700
H	2.22992600	-1.24933100	2.55736700
C	-1.38800200	-1.96632600	2.84699600
H	-0.33186400	-2.19799800	2.66761300
C	-1.46002200	-0.51541300	3.33087600
H	-1.08154600	0.17718300	2.57034700
H	-2.49092900	-0.22741400	3.56381800
H	-0.86192400	-0.38585900	4.23949800
C	-1.88891200	-2.93406400	3.92027900
H	-1.28082100	-2.83925700	4.82597100
H	-2.92725700	-2.72588200	4.19981400
H	-1.83528500	-3.97317500	3.57957200
C	2.33314200	0.79790000	3.14476600
H	3.05871300	1.61081500	3.25940800
H	1.55875100	1.13572200	2.44635900
H	1.86203600	0.62860100	4.11905100
C	4.03776200	-0.99550200	3.65791100
H	4.82895500	-0.25878100	3.83403200
H	3.55584200	-1.19898400	4.62000100

H	4.51257800	-1.91893600	3.31068000
C	0.43809800	2.74346900	-0.49584200
Br	1.98950400	3.52238900	-0.03641700
H	0.26618500	-4.48344100	0.71373600
H	2.93886700	-3.67834100	0.62051700
C	-2.07107600	2.47638400	-1.42819600
H	-2.33854200	3.52619100	-1.61840300
H	-2.05666300	1.96878600	-2.39873800
C	-3.11752400	1.83802200	-0.49722600
C	-3.07442500	2.47690900	0.89211200
C	-4.51964600	1.94720700	-1.12308900
C	-4.13272100	1.89406800	1.82071100
H	-3.23375600	3.56131400	0.79082900
H	-2.07627100	2.34887100	1.33205600
C	-5.57581700	1.39243000	-0.16226700
H	-4.73051000	3.01982100	-1.26770400
C	-5.52366300	2.03954900	1.21595900
H	-4.07958900	2.38055200	2.80165400
H	-3.91852300	0.82792100	1.97993900
H	-6.56853800	1.52264300	-0.61020300
H	-5.41719700	0.30893600	-0.06079300
H	-6.27776900	1.59109700	1.87326400
H	-5.77625600	3.10605100	1.13157800
H	-2.87484200	0.76708100	-0.39623200
C	-4.62464300	1.24300900	-2.47290400
H	-3.99712700	1.69781600	-3.24674400
H	-4.33690300	0.18839100	-2.37690100
H	-5.65597500	1.27340300	-2.84004800

Cartesian coordinates of the optimized geometry for Int-**30a** at PBE0-D3BJ/6-31G*,def2-TZVP level of theory: (number of imaginary frequencies = 0):

Au	0.01205100	-0.38807400	-0.34249200
C	1.78875600	-1.11054900	-0.80255700
C	-1.73520000	0.46540900	0.26561000
C	-3.18097900	2.00176400	1.04234200
C	-3.86227900	0.83410900	0.89074700
N	-1.88370000	1.75168100	0.65307100
N	-2.95648500	-0.08968200	0.41687600
C	-3.24207300	-1.46457600	0.11941300
C	-3.64365900	-1.79012000	-1.18293100
C	-3.07213600	-2.41077600	1.13904200
C	-3.88075900	-3.13866200	-1.45417000
C	-3.32317700	-3.74466900	0.81350900
C	-3.72318800	-4.10468200	-0.46774300
H	-4.19144000	-3.43562500	-2.45160700
H	-3.20287400	-4.51117000	1.57366100
H	-3.91503600	-5.14835800	-0.69954900
C	-0.81031500	2.70676000	0.66457000
C	-0.59471000	3.47542000	-0.48721600
C	-0.01862100	2.79988100	1.81704700
C	0.46119800	4.38726300	-0.45327400
C	1.02273100	3.72931900	1.79831400
C	1.25772400	4.51797500	0.67828500
H	0.66440100	5.00039300	-1.32628900
H	1.65964200	3.83369600	2.67195800
H	2.07072200	5.23843900	0.68586100
C	-2.60490800	-2.03141800	2.52999500
H	-2.57873000	-0.93765900	2.59788300
C	-1.18089800	-2.53784300	2.77600700
H	-1.13781800	-3.63181600	2.72720300
H	-0.48759100	-2.13775800	2.02642200
H	-0.82964700	-2.22967900	3.76703700
C	-3.56623600	-2.53156400	3.60961300
H	-3.23931600	-2.18684600	4.59636600

H	-4.58364300	-2.16493800	3.43898600
H	-3.60572200	-3.62575800	3.63970900
C	-0.23668700	1.91940600	3.03063800
H	-1.13028700	1.30849800	2.85772300
C	-0.48617800	2.74753800	4.29248200
H	-0.69361900	2.08992100	5.14337600
H	0.38583800	3.35783000	4.55129700
H	-1.33956200	3.42164000	4.16519900
C	0.94240900	0.96044800	3.21749000
H	1.09661600	0.34849100	2.32100600
H	1.87210900	1.50725800	3.40999500
H	0.76080400	0.29188000	4.06628400
C	-1.42579800	3.30743400	-1.74268900
H	-2.26546800	2.64098700	-1.51324400
C	-3.78267200	-0.74672500	-2.27308100
H	-3.68423700	0.24428600	-1.81485600
C	-2.65435300	-0.89066900	-3.29826900
H	-1.67201100	-0.80591000	-2.81843900
H	-2.70083600	-1.86371100	-3.80021400
H	-2.72979000	-0.11115400	-4.06443200
C	-5.15499300	-0.80231500	-2.94628900
H	-5.24896400	0.00643100	-3.67866500
H	-5.30586200	-1.74701600	-3.47964500
H	-5.96339700	-0.69763200	-2.21539200
C	-0.59539200	2.63703400	-2.84110400
H	0.26279400	3.25857700	-3.12013500
H	-0.20968600	1.66701900	-2.50622100
H	-1.20376500	2.47540300	-3.73783200
C	-2.01520400	4.63461500	-2.22260300
H	-1.23252800	5.33828500	-2.52591100
H	-2.66155900	4.46866200	-3.09091400
H	-2.61116800	5.11410900	-1.43921200
C	2.26489600	-2.50430300	-0.80674900
H	1.58402100	-3.19080600	-1.32535400
C	3.73714100	-2.50852800	-1.24501600
H	3.75476500	-2.53773300	-2.34418000
C	4.23912900	-1.11007300	-0.80038500
H	2.88349100	-0.21164000	-2.21798900
C	2.97096500	-0.28690200	-1.11352800
Br	2.90720000	1.53951300	-0.47476400
H	-4.89433100	0.57784000	1.07274900
H	-3.49506400	2.97496300	1.38608600
H	2.16733600	-2.79336400	0.25983700
C	4.56894300	-3.67212700	-0.70944300
H	4.07825900	-4.61937700	-0.96557200
H	5.53395700	-3.67678100	-1.23237800
C	4.52877600	-1.08118300	0.71182000
C	4.81850800	-3.57697000	0.79275700
C	5.43051900	-2.22870700	1.15832600
H	6.42047600	-2.13632700	0.69266000
H	5.59266000	-2.16138800	2.23970500
H	4.97110200	-0.11225200	0.96788800
H	3.58610400	-1.13521000	1.27716600
H	5.47365600	-4.39632200	1.10804000
H	3.87815500	-3.71333600	1.34825000
C	5.43546400	-0.62125600	-1.60328700
H	5.67049200	0.41773800	-1.34937800
H	6.32353600	-1.22536500	-1.39632200
H	5.23982800	-0.67507900	-2.68066500

Cartesian coordinates of the optimized geometry for Int-**30b** at PBE0-D3BJ/6-31G*,def2-TZVP level of theory: (number of imaginary frequencies = 0):

Au	0.05650500	-0.33105500	-0.31592700
C	1.93268400	-0.78011700	-0.72555300

C	-1.81659300	0.25454300	0.23268000
C	-3.50752800	1.57083700	0.91247900
C	-3.98453200	0.29995700	0.82341400
N	-2.18106300	1.51789900	0.54557200
N	-2.93175100	-0.48578100	0.40796500
C	-2.98246700	-1.90322300	0.18746200
C	-3.30758900	-2.36195600	-1.09600500
C	-2.67240000	-2.75063300	1.25959100
C	-3.31586000	-3.74402200	-1.29135000
C	-2.69620800	-4.12359200	1.00918300
C	-3.01449200	-4.61484700	-0.25119100
H	-3.56027800	-4.14279800	-2.27156300
H	-2.46150200	-4.81652700	1.81204900
H	-3.02895600	-5.68705500	-0.42428400
C	-1.27930000	2.63596100	0.50963600
C	-1.17420300	3.36459200	-0.68293600
C	-0.53175200	2.92125800	1.66000100
C	-0.28012400	4.43599900	-0.69525400
C	0.34566900	4.00505900	1.59405100
C	0.46766700	4.75701400	0.43160900
H	-0.16544300	5.02466200	-1.60059900
H	0.94308600	4.26076800	2.46441500
H	1.15340700	5.59903600	0.40243800
C	-2.29331200	-2.22339400	2.62893700
H	-2.44437300	-1.13767400	2.63375100
C	-0.81041000	-2.47897700	2.91249500
H	-0.59166900	-3.55270100	2.92913300
H	-0.17897300	-2.01667400	2.14426500
H	-0.52806200	-2.06153600	3.88536100
C	-3.17830500	-2.81103800	3.72958100
H	-2.92575600	-2.36431800	4.69707800
H	-4.23859700	-2.62268200	3.53219800
H	-3.04254300	-3.89403300	3.82150100
C	-0.62525300	2.08807100	2.92200900
H	-1.40664900	1.33254800	2.77971200
C	-1.02473300	2.93654000	4.13072800
H	-1.13874000	2.30399100	5.01750900
H	-0.26570300	3.69249700	4.35938500
H	-1.97247600	3.45679100	3.95753900
C	0.68882000	1.34350200	3.17403000
H	0.95276100	0.71144000	2.31800900
H	1.51611000	2.04288800	3.33784000
H	0.60319900	0.70656400	4.06138800
C	-1.94977800	2.99495600	-1.93081900
H	-2.67946400	2.22084300	-1.66603200
C	-3.60254700	-1.41765100	-2.24386900
H	-3.67295100	-0.40002400	-1.84231800
C	-2.45418300	-1.43127000	-3.25679500
H	-1.50442200	-1.15992100	-2.78065100
H	-2.33504600	-2.42509500	-3.70294200
H	-2.64753500	-0.71854200	-4.06609100
C	-4.93940100	-1.73490800	-2.91604500
H	-5.15585800	-0.99468400	-3.69353700
H	-4.92752400	-2.71987400	-3.39500500
H	-5.76251100	-1.72354600	-2.19420200
C	-1.00905400	2.39759000	-2.98133400
H	-0.25190000	3.12659100	-3.29105500
H	-0.48521900	1.51937900	-2.58602900
H	-1.57096500	2.09224500	-3.87093800
C	-2.73049900	4.18299700	-2.49479400
H	-2.06207400	4.97963200	-2.83872800
H	-3.33170800	3.86603000	-3.35348200
H	-3.40384900	4.61120600	-1.74513400
C	2.62862200	-2.07494500	-0.64288600
H	2.06493600	-2.90398600	-1.08734400
C	4.07866000	-1.87089500	-1.11170800

H	4.07801000	-1.94227400	-2.20927500
C	4.33826700	-0.39717500	-0.72829200
H	2.87272800	0.22286000	-2.18714800
C	2.97185600	0.20863800	-1.08241700
Br	2.61288400	2.02384200	-0.51430600
H	-4.96284400	-0.11318300	1.01355300
H	-3.98306000	2.49603400	1.19852700
H	2.59840900	-2.28447900	0.44719300
C	5.09904400	-2.87671600	-0.56312900
H	4.65236500	-3.87769500	-0.65067200
C	4.68503700	-0.21582400	0.75082500
C	5.39624300	-2.61435100	0.91828400
C	5.79611400	-1.16599000	1.18341500
H	6.72365300	-0.92222600	0.64983600
H	6.00801400	-1.02284400	2.24854500
H	4.96235400	0.82823500	0.92886800
H	3.79512800	-0.39914700	1.37230600
H	6.18383500	-3.29942400	1.25248700
H	4.51156000	-2.85437100	1.52752400
C	6.37076700	-2.86845200	-1.41071400
H	7.09490200	-3.59588900	-1.02985300
H	6.15075000	-3.13016900	-2.45155900
H	6.85886000	-1.88768400	-1.41350500
H	5.12383400	0.04563600	-1.34823000

Cartesian coordinates of the optimized geometry for Int-**30c** at PBE0-D3BJ/6-31G*,def2-TZVP level of theory: (number of imaginary frequencies = 0):

Au	-0.01135000	-0.47557100	-0.01880100
C	-1.77285200	-1.34748000	-0.18517300
C	1.74191800	0.55859600	0.07022000
C	3.17656600	2.28805800	0.14305600
C	3.91157400	1.14401200	0.10441100
N	1.85391100	1.90455200	0.12226000
N	3.01203800	0.10138600	0.05866200
C	3.35047800	-1.29233900	0.00136900
C	3.48037900	-1.99191800	1.20847600
C	3.50072500	-1.88485900	-1.25961300
C	3.77668900	-3.35325500	1.12285100
C	3.79726200	-3.24852100	-1.28736300
C	3.93421900	-3.97433800	-0.11023900
H	3.88406600	-3.93424900	2.03420400
H	3.92144100	-3.74814800	-2.24379000
H	4.16692700	-5.03438900	-0.15429200
C	0.72631100	2.79477900	0.13782000
C	0.19080100	3.16432700	1.37911200
C	0.20455100	3.22473200	-1.08949400
C	-0.91198100	4.01937600	1.36549800
C	-0.89805000	4.07978600	-1.04576400
C	-1.44783000	4.47691800	0.16736800
H	-1.35920600	4.32787700	2.30583300
H	-1.33255100	4.43674300	-1.97503200
H	-2.30296600	5.14677500	0.17936600
C	3.31977300	-1.10859500	-2.54859100
H	3.20870200	-0.04713100	-2.29844200
C	2.03808500	-1.54521100	-3.26378600
H	2.08643900	-2.60232500	-3.54869800
H	1.16218000	-1.40828700	-2.61830200
H	1.88742400	-0.95622000	-4.17527900
C	4.53768600	-1.23510000	-3.46541100
H	4.40654200	-0.61213900	-4.35651900
H	5.45420500	-0.91737500	-2.95775900
H	4.68310600	-2.26668200	-3.80367400
C	0.76910900	2.77095500	-2.42021700
H	1.66110700	2.16363800	-2.22747400

C	1.20182900	3.95657800	-3.28475000
H	1.65620500	3.60128700	-4.21575300
H	0.34973800	4.59013100	-3.55393400
H	1.93326200	4.58391000	-2.76478500
C	-0.23785900	1.88277000	-3.15632400
H	-0.51771000	1.01601800	-2.54618000
H	-1.15535700	2.43375500	-3.39091500
H	0.18903200	1.51976100	-4.09780100
C	0.73744100	2.63520200	2.68924000
H	1.66286800	2.08623100	2.47942000
C	3.27573500	-1.32949800	2.55595600
H	3.18278200	-0.24880500	2.39721200
C	1.97128900	-1.81146300	3.19708600
H	1.11325700	-1.61107000	2.54432500
H	2.00275500	-2.88974700	3.39010000
H	1.80171100	-1.30203400	4.15211700
C	4.46768500	-1.55146700	3.48851800
H	4.31998900	-1.00565500	4.42637600
H	4.59186200	-2.61007000	3.74077500
H	5.40108500	-1.20401700	3.03383900
C	-0.25027700	1.64611900	3.31486000
H	-1.20095800	2.13642900	3.55253400
H	-0.46403400	0.81766800	2.62939500
H	0.15849400	1.22977200	4.24219300
C	1.08663400	3.76291400	3.66160600
H	0.19704500	4.32657700	3.96270800
H	1.53799800	3.35167600	4.57070700
H	1.79554000	4.46898100	3.21698000
C	-2.10821700	-2.70922700	-0.64780100
H	-1.49179700	-3.48042800	-0.16682400
H	-3.20737000	-0.89931600	1.13821100
C	-3.06001200	-0.67872400	0.06149900
Br	-3.03679600	1.25330300	-0.03980300
H	4.97736100	0.97660700	0.10594200
H	3.46837000	3.32592300	0.18328800
H	-1.75565600	-2.70641600	-1.69743700
C	-4.15262400	-1.43011700	-0.73539300
C	-5.56027500	-1.19719500	-0.18370100
C	-3.63259600	-2.88351200	-0.55302900
C	-5.81618000	-1.83425100	1.17598000
H	-6.27096600	-1.61792600	-0.90792000
H	-5.75223100	-0.11719400	-0.15720900
C	-4.01253200	-3.52139200	0.79048800
H	-4.01531900	-3.51161400	-1.36530200
C	-5.48028400	-3.32055800	1.14467100
H	-6.86328000	-1.67814300	1.45732100
H	-5.21972400	-1.33742000	1.95726300
H	-3.75972000	-4.58773500	0.75302100
H	-3.39513800	-3.09793000	1.59812500
H	-5.69741500	-3.78936500	2.11057600
H	-6.11402900	-3.82271500	0.40023300
C	-4.11037200	-1.01106100	-2.20563200
H	-4.78355600	-1.64507800	-2.79144300
H	-4.43965500	0.02642800	-2.31716800
H	-3.10872400	-1.08386200	-2.64498300

Cartesian coordinates of the optimized geometry for Int-**30d** at PBE0-D3BJ/6-31G*,def2-TZVP level of theory: (number of imaginary frequencies = 0):

Au	-0.06756400	-0.35191400	-0.18451600
C	-1.91412800	-0.95458600	-0.53156600
C	1.79162800	0.39840200	0.17958700
C	3.43059100	1.87772500	0.60346100
C	3.99907600	0.64449400	0.52265100
N	2.08103600	1.70139200	0.39220700

N	2.97713900	-0.24204500	0.26154300
C	3.12160600	-1.66084500	0.09883600
C	2.98856400	-2.47356600	1.23251800
C	3.35523900	-2.15825000	-1.19018400
C	3.09821300	-3.85177400	1.04018300
C	3.45598700	-3.54379100	-1.32578100
C	3.32942100	-4.38095100	-0.22374500
H	3.00036000	-4.51866200	1.89194600
H	3.63477300	-3.97223700	-2.30770800
H	3.41327800	-5.45639300	-0.35106300
C	1.09286100	2.74439500	0.38213200
C	0.45686500	3.06718900	1.58862900
C	0.79610600	3.36336400	-0.83935100
C	-0.51063100	4.07184400	1.54711900
C	-0.18040700	4.36094500	-0.82462300
C	-0.82360100	4.71491800	0.35542200
H	-1.02904400	4.35105300	2.45968400
H	-0.44031600	4.86578300	-1.75055300
H	-1.57690900	5.49755400	0.34611600
C	3.46003500	-1.25414300	-2.40186000
H	3.48268100	-0.21475700	-2.05424800
C	2.22639500	-1.41014800	-3.29553100
H	2.14786700	-2.43240400	-3.68262500
H	1.30756600	-1.18751500	-2.74004500
H	2.28316600	-0.72821100	-4.15117000
C	4.74931500	-1.49937000	-3.18748000
H	4.82874100	-0.78641100	-4.01493400
H	5.63283600	-1.38474800	-2.55114300
H	4.77516500	-2.50624400	-3.61787600
C	1.46357700	2.96261600	-2.13908800
H	2.24507800	2.22784100	-1.91316900
C	2.14027900	4.15346300	-2.82028500
H	2.66299100	3.82538800	-3.72502800
H	1.41103100	4.91439200	-3.11857700
H	2.86973000	4.63108600	-2.15797100
C	0.45578100	2.28549500	-3.07182700
H	-0.00149700	1.41337400	-2.59001200
H	-0.35097800	2.97300800	-3.34919200
H	0.94872400	1.95327500	-3.99214000
C	0.75784400	2.34330600	2.88519200
H	1.61459700	1.67995200	2.71876900
C	2.70712400	-1.90724000	2.60969200
H	2.76907800	-0.81431200	2.55155700
C	1.28635800	-2.26110800	3.05756000
H	0.54354500	-1.88978900	2.34149000
H	1.15870200	-3.34610200	3.14334600
H	1.07092200	-1.81752300	4.03592900
C	3.74380700	-2.36812300	3.63588700
H	3.55453300	-1.89295100	4.60426600
H	3.70638200	-3.45215500	3.78830300
H	4.75997700	-2.10966900	3.32075600
C	-0.42849500	1.46453000	3.29196800
H	-1.32354900	2.07006400	3.47330500
H	-0.66742500	0.74040600	2.50419200
H	-0.20023600	0.91185800	4.21004200
C	1.14183700	3.31215100	4.00471100
H	0.31122100	3.97657300	4.26605600
H	1.41542900	2.75687600	4.90813200
H	1.99290900	3.93794000	3.71696800
C	-2.40213800	-2.19459400	-1.15672100
H	-1.83021700	-3.08812100	-0.88442400
H	-3.28217500	-0.34662900	0.83156500
C	-3.11675900	-0.14693100	-0.24434600
Br	-2.85588100	1.76722300	-0.36231100
H	5.02251300	0.31910800	0.62577500
H	3.85578100	2.85171400	0.78917600

H	-2.12238600	-2.00016600	-2.21660800
C	-4.28199800	-0.73149700	-1.05499600
C	-5.66943400	-0.37025300	-0.54277900
C	-3.93349600	-2.23565800	-1.02457800
C	-6.01013200	-1.08320100	0.76062700
H	-6.39962700	-0.66007700	-1.30907700
H	-5.74568500	0.71832100	-0.43577700
C	-4.37769200	-2.95315000	0.26326400
H	-4.39051900	-2.74657200	-1.88029300
C	-5.82010300	-2.58853700	0.61718500
H	-7.04205100	-0.85153600	1.04568600
H	-5.38130200	-0.70840600	1.58293100
H	-3.73867700	-2.60851400	1.09416100
H	-6.10497300	-3.10293900	1.54251600
H	-6.48959300	-2.96839300	-0.16910100
C	-4.20427300	-4.46270300	0.13553200
H	-4.48937900	-4.96525300	1.06546600
H	-4.84102800	-4.85761700	-0.66525000
H	-3.17046800	-4.74892700	-0.08854000
H	-4.16666800	-0.36587800	-2.08467400

Cartesian coordinates of the optimized geometry for Int-**30e** at PBE0-D3BJ/6-31G*,def2-TZVP level of theory: (number of imaginary frequencies = 0):

Au	-0.24509100	-0.55746500	-0.32909800
C	2.67111800	-1.60073500	-1.80321400
C	-0.97361500	1.24454100	0.26151200
C	-2.41145600	2.82513500	0.96008900
C	-1.20099300	3.41901600	0.78257700
N	-2.24684200	1.49689800	0.63487600
N	-0.33812100	2.43190200	0.35617600
C	1.05329000	2.61817300	0.05948000
C	1.41902900	2.89220600	-1.26521000
C	1.97321200	2.50336500	1.11088100
C	2.78123800	3.05425800	-1.52603700
C	3.32061700	2.68624900	0.79676100
C	3.72066200	2.95844200	-0.50650800
H	3.10827300	3.26841300	-2.53947500
H	4.06629200	2.61824800	1.58331800
H	4.77466800	3.10076500	-0.72762800
C	-3.26519200	0.48537400	0.67938100
C	-4.03465400	0.26969500	-0.47181900
C	-3.40372700	-0.26121500	1.85732300
C	-4.98723000	-0.74885400	-0.41483100
C	-4.37156300	-1.26687200	1.85945300
C	-5.15448800	-1.50789400	0.73706100
H	-5.60366700	-0.95182300	-1.28571700
H	-4.51151000	-1.87046100	2.75160800
H	-5.90232200	-2.29525000	0.76013200
C	1.54984200	2.16167400	2.52539400
H	0.45603000	2.20679800	2.57761100
C	1.96358800	0.72919100	2.87457200
H	3.05373900	0.61767100	2.84435300
H	1.53059400	0.01008300	2.16929300
H	1.62537500	0.46574700	3.88277700
C	2.09640200	3.16093700	3.54599300
H	1.71309600	2.92766900	4.54487100
H	1.80330500	4.18624700	3.29817800
H	3.18998000	3.12867200	3.59892100
C	-2.53356200	-0.02884500	3.07606800
H	-1.90337700	0.84819300	2.88761500
C	-3.37195800	0.27107900	4.32012500
H	-2.72035600	0.49252900	5.17209800
H	-3.99938100	-0.58270100	4.59781700
H	-4.03016100	1.13124600	4.16000800

C	-1.60054500	-1.22039100	3.30837100
H	-0.97601000	-1.40936300	2.42701300
H	-2.16908500	-2.13327800	3.51788300
H	-0.94120200	-1.03047200	4.16251400
C	-3.83367200	1.06684600	-1.74485700
H	-3.12508100	1.87680100	-1.53556800
C	0.40263900	3.00495800	-2.38364500
H	-0.59962700	2.93577300	-1.94533800
C	0.54866200	1.84553900	-3.37215000
H	0.41459200	0.88088600	-2.86880400
H	1.53803500	1.84776300	-3.84359000
H	-0.20240000	1.92152500	-4.16590600
C	0.49780900	4.35643800	-3.09480400
H	-0.28799700	4.44081500	-3.85284100
H	1.46056900	4.47752200	-3.60296300
H	0.38449200	5.18716300	-2.39068600
C	-3.21532300	0.18895000	-2.83657300
H	-3.88051600	-0.64127200	-3.09895300
H	-2.26045500	-0.23762400	-2.50717300
H	-3.03396500	0.77565900	-3.74390900
C	-5.13534900	1.71287100	-2.22274200
H	-5.87998500	0.96010500	-2.50296600
H	-4.94759000	2.33380200	-3.10505700
H	-5.57608800	2.34621500	-1.44595500
C	3.54167100	-1.19190600	-0.62031600
C	3.74644100	-2.51050100	0.12313200
H	4.36792600	-3.15862800	-0.51600700
C	2.34632000	-3.11019500	0.17337300
H	1.79034600	-3.63448500	-1.88974300
C	1.76206200	-2.73489100	-1.26831700
H	-0.87595000	4.43872400	0.91844800
H	-3.36249000	3.21908300	1.28297400
H	2.94961200	-0.52925900	0.03351500
C	0.40665300	-2.33885100	-0.92106600
Br	-0.82510600	-3.72983800	-0.92798500
H	2.09506900	-0.77051400	-2.22427500
H	3.28819300	-2.01272700	-2.61053500
C	4.86764000	-0.50881200	-0.90888600
H	5.45977500	-1.13744800	-1.59015500
H	4.70697600	0.45077600	-1.41370200
C	5.62887200	-0.30285300	0.40365100
C	4.46153700	-2.30600100	1.44914400
H	3.83321600	-1.68769200	2.10682200
H	4.62357400	-3.26030100	1.96418500
C	5.79850800	-1.60330500	1.19151500
H	6.30254400	-1.40195300	2.14372800
H	6.45522700	-2.28311000	0.63070100
H	5.07469600	0.42022400	1.01884800
H	6.61038600	0.14260000	0.20436500
H	2.28893200	-4.19162800	0.31951100
H	1.77244300	-2.63214000	0.97938600

Cartesian coordinates of the optimized geometry for Int-**30f** at PBE0-D3BJ/6-31G*,def2-TZVP level of theory: (number of imaginary frequencies = 0):

Au	0.06358600	-0.33152900	-0.10038600
C	1.98217700	-0.77151900	-0.21088200
C	-1.88360600	0.25045100	0.05839900
C	-3.68597600	1.57795600	0.26612400
C	-4.12431800	0.29064300	0.22740500
N	-2.31384800	1.52770200	0.16013200
N	-3.00413900	-0.50140100	0.10131500
C	-2.99854800	-1.93458800	0.02193800
C	-3.07866900	-2.52701500	-1.24547700
C	-2.88150200	-2.66256900	1.21347500

C	-3.03640300	-3.92122700	-1.29666200
C	-2.84413500	-4.05369000	1.10460400
C	-2.92098600	-4.67598400	-0.13551900
H	-3.09342300	-4.42176400	-2.25886300
H	-2.75310000	-4.65683800	2.00334600
H	-2.89205800	-5.76000800	-0.19768000
C	-1.43280400	2.66266200	0.16973600
C	-1.09459800	3.25049100	-1.05666800
C	-0.93524000	3.10350700	1.40322700
C	-0.22398200	4.34042000	-1.01934400
C	-0.06722600	4.19663800	1.38325500
C	0.28135100	4.81153400	0.18653700
H	0.06538600	4.82368400	-1.94790100
H	0.34148400	4.56959300	2.31793300
H	0.95446300	5.66413900	0.19345500
C	-2.76651500	-1.99186300	2.56756500
H	-2.92802300	-0.91595100	2.43354800
C	-1.35836000	-2.17417000	3.14056500
H	-1.13436100	-3.23408400	3.30599600
H	-0.60100600	-1.77105800	2.45763300
H	-1.26451200	-1.65526700	4.10091000
C	-3.83228300	-2.49429200	3.54312700
H	-3.76711300	-1.94673900	4.48933000
H	-4.84027800	-2.35841300	3.13795400
H	-3.70323900	-3.55827600	3.76904200
C	-1.27798000	2.41990000	2.71113600
H	-2.03140800	1.64936800	2.51030600
C	-1.88278700	3.39683800	3.72099700
H	-2.17494300	2.86597500	4.63327200
H	-1.16668100	4.17465800	4.00737700
H	-2.77032600	3.89296100	3.31478100
C	-0.04601700	1.71557500	3.28598300
H	0.36271100	0.99194300	2.57116600
H	0.74682200	2.43428300	3.52124200
H	-0.30461900	1.18298000	4.20787200
C	-1.60561200	2.72091900	-2.38091900
H	-2.34032200	1.93336100	-2.17676800
C	-3.16985000	-1.70977100	-2.51841600
H	-3.32253600	-0.65978800	-2.24276800
C	-1.85679300	-1.79033000	-3.30213500
H	-1.01258400	-1.44493800	-2.69355500
H	-1.64774000	-2.81965000	-3.61472600
H	-1.90794200	-1.16762400	-4.20215000
C	-4.35922300	-2.13014000	-3.38351200
H	-4.43871700	-1.47691500	-4.25887600
H	-4.25048800	-3.15691300	-3.74902400
H	-5.29966300	-2.07111700	-2.82604200
C	-0.46302600	2.08498000	-3.17762600
H	0.30765600	2.82477400	-3.42124400
H	0.01397500	1.28087400	-2.60502600
H	-0.83795100	1.66365700	-4.11689600
C	-2.31364400	3.80638400	-3.19344500
H	-1.62560100	4.60932900	-3.47923200
H	-2.72472300	3.38151800	-4.11533500
H	-3.13602000	4.25634800	-2.62774100
C	2.67621500	-2.04457400	0.07135700
H	2.14166900	-2.92846500	-0.29284600
C	4.14616800	-1.85242100	-0.31711900
C	4.35516900	-0.36846600	-0.00610200
H	3.06387000	0.19946100	-1.64061600
C	3.04549700	0.21314500	-0.53281500
Br	2.63207600	2.03788000	-0.03874100
H	-5.11648900	-0.13024000	0.27629400
H	-4.21644200	2.51285200	0.35841200
H	2.58919900	-2.09976500	1.18013700
C	5.22064300	-2.71416300	0.33499400

C	6.58348000	-2.20445800	-0.15798100
C	5.67354200	0.14440400	-0.54696400
C	6.79538200	-0.70424400	0.06128700
H	5.81002400	1.20420700	-0.30478200
H	5.68182000	0.05576800	-1.64295400
H	7.76065800	-0.40135600	-0.35848400
H	6.84842600	-0.49872600	1.13961000
H	7.38232100	-2.76740100	0.33889100
H	6.66869800	-2.43163700	-1.23131900
H	5.16383600	-2.55685200	1.42427500
H	4.22469600	-1.98698800	-1.40916400
H	4.35017300	-0.24373700	1.08852500
C	5.03508800	-4.19745500	0.04227900
H	4.08240500	-4.57495800	0.43117400
H	5.05916200	-4.38870100	-1.03763600
H	5.83501900	-4.78775700	0.50102600

Cartesian coordinates of the optimized geometry for TS-**1** at PBE0-D3BJ/6-31G*,def2-TZVP level of theory: (number of imaginary frequencies = 1):

Au	0.09582900	-0.51360700	-0.36957500
C	1.53143000	-1.84910500	-0.92571800
C	-1.20237700	0.89773700	0.27307500
C	-1.96333500	2.86479700	1.06433000
C	-3.03228200	2.04070500	0.91579800
N	-0.85536800	2.14425600	0.66533500
N	-2.54154700	0.84323800	0.43292800
C	-3.32553100	-0.32008400	0.13483800
C	-3.80627100	-0.47901300	-1.17140000
C	-3.54235900	-1.25249300	1.15865400
C	-4.53553300	-1.63805900	-1.44298400
C	-4.27687500	-2.39364100	0.83328100
C	-4.76815400	-2.58448300	-0.45283700
H	-4.92473200	-1.80095400	-2.44390100
H	-4.46611900	-3.14288700	1.59648100
H	-5.33951300	-3.47875300	-0.68477400
C	0.49711900	2.62462800	0.66373300
C	0.97542100	3.25976400	-0.49048400
C	1.28058400	2.40842900	1.80563400
C	2.29697200	3.70914500	-0.46923100
C	2.59428600	2.87980700	1.77587300
C	3.09620400	3.52869100	0.65367700
H	2.70450800	4.20686100	-1.34439700
H	3.23168600	2.73599000	2.64374800
H	4.11862300	3.89652000	0.65389400
C	-2.97549800	-1.06807000	2.55225500
H	-2.59756300	-0.04246100	2.63503800
C	-1.78879200	-2.01054600	2.77445800
H	-2.10156300	-3.05834700	2.69569800
H	-1.00480100	-1.83105900	2.02918700
H	-1.35774500	-1.85898200	3.77057300
C	-4.03835900	-1.24958800	3.63658400
H	-3.61314200	-1.03039500	4.62178100
H	-4.89089500	-0.58179700	3.47588800
H	-4.41788000	-2.27668200	3.66528700
C	0.76121200	1.66598600	3.01978900
H	-0.29810700	1.43810600	2.85583500
C	0.85744100	2.51595700	4.28788100
H	0.42704800	1.97788200	5.13927300
H	1.89810200	2.75069800	4.53707500

H	0.31950200	3.46291000	4.17484700
C	1.49349300	0.33128700	3.18448300
H	1.38523900	-0.28497000	2.28417500
H	2.56389400	0.48603400	3.36190700
H	1.08676600	-0.22605400	4.03569300
C	0.12869800	3.42283100	-1.73589600
H	-0.89196400	3.09995100	-1.50112200
C	-3.53150300	0.53315000	-2.26492100
H	-3.04888000	1.40597200	-1.81035400
C	-2.55777800	-0.04180500	-3.29720900
H	-1.61789600	-0.34858200	-2.82352500
H	-2.98634900	-0.91779000	-3.79736100
H	-2.32757600	0.70562700	-4.06461600
C	-4.82206100	1.01890600	-2.92702100
H	-4.59837200	1.79565700	-3.66602600
H	-5.33816400	0.20648000	-3.45003500
H	-5.51626400	1.43700600	-2.19068900
C	0.64979100	2.51581700	-2.85435900
H	1.67017300	2.79257400	-3.14299000
H	0.66267900	1.46677000	-2.53688500
H	0.01331700	2.59894400	-3.74230800
C	0.05412600	4.88164300	-2.18932500
H	1.03619200	5.26144400	-2.49155700
H	-0.61387000	4.97522700	-3.05211000
H	-0.32443400	5.52965200	-1.39197400
C	1.33027700	-3.33776500	-0.97897700
C	2.49293800	-3.97264200	-1.77409500
H	2.45357000	-3.73616100	-2.84153500
C	3.75534100	-3.51071000	-1.15780600
H	3.56743300	-2.11556700	-1.58595300
C	2.77224200	-1.40539700	-1.09874200
Br	3.62690000	0.15645500	-0.58220700
H	-4.08397800	2.18925000	1.10448400
H	-1.88622300	3.88358100	1.41054000
H	4.66351700	-3.50433200	-1.76752700
C	3.94062300	-3.72308100	0.30482400
C	1.39517300	-3.87445000	0.46692400
C	2.69397000	-3.45945700	1.15785200
H	2.79658900	-3.97908800	2.11538400
H	2.65454000	-2.38742300	1.38555000
H	4.22337500	-4.78897600	0.37865300
H	4.80857300	-3.16489100	0.67188200
H	2.42951000	-5.06437100	-1.66419100
H	1.30432200	-4.96814600	0.43173600
H	0.53972100	-3.50082000	1.03826500
H	0.36979900	-3.60044500	-1.43086700

Cartesian coordinates of the optimized geometry for TS-**2** at PBE0-D3BJ/6-31G*,def2-TZVP level of theory: (number of imaginary frequencies = 1):

Au	0.09310600	-0.42569500	-0.27966800
C	1.42428200	-1.90110300	-0.72804000
C	-1.15264600	1.07907200	0.22926200
C	-1.89607400	3.11828000	0.82373900
C	-2.97439100	2.29561000	0.74322900
N	-0.79292800	2.35128300	0.50483800
N	-2.49346200	1.05271900	0.38019100
C	-3.26938200	-0.13792400	0.18539400
C	-3.70007300	-0.44541900	-1.11188200
C	-3.50358400	-0.96379300	1.29362400
C	-4.40121500	-1.64067500	-1.28248400
C	-4.20741600	-2.14758200	1.06757600
C	-4.65200200	-2.48263100	-0.20615700
H	-4.75238000	-1.91552700	-2.27301000
H	-4.40782800	-2.81684900	1.89922600

H	-5.19986900	-3.40783100	-0.36057000
C	0.56966900	2.80043700	0.46076500
C	1.06125900	3.31071400	-0.74856400
C	1.35424200	2.65813700	1.61322400
C	2.39886400	3.70875500	-0.77528800
C	2.68463100	3.07371700	1.53351400
C	3.20112000	3.59732000	0.35421800
H	2.81745600	4.10880300	-1.69423100
H	3.32387500	2.98393900	2.40730100
H	4.23745600	3.92133200	0.31475700
C	-2.97872700	-0.62961200	2.67553300
H	-2.60756500	0.40161700	2.66097000
C	-1.79504800	-1.53564400	3.02770300
H	-2.10216100	-2.58784000	3.05378100
H	-0.99193800	-1.43225400	2.28845400
H	-1.39073100	-1.27644800	4.01268900
C	-4.07220200	-0.70490000	3.74184900
H	-3.67631600	-0.38408800	4.71125600
H	-4.92122800	-0.06147800	3.48938300
H	-4.45009600	-1.72572200	3.86416200
C	0.81935300	2.04865000	2.89293900
H	-0.24691700	1.83733500	2.75365700
C	0.94641000	3.01246100	4.07403200
H	0.50663000	2.57019600	4.97440900
H	1.99475000	3.24059000	4.29571400
H	0.43418400	3.95908600	3.87298100
C	1.51377500	0.71508500	3.18272700
H	1.38601700	0.01971100	2.34476800
H	2.58858300	0.85495200	3.34470000
H	1.09390500	0.25268500	4.08298400
C	0.21142800	3.38956500	-2.00014900
H	-0.81967000	3.13296600	-1.73141100
C	-3.40370100	0.44602700	-2.30042900
H	-2.90251200	1.34966100	-1.93518500
C	-2.44379900	-0.24849500	-3.27007400
H	-1.51110100	-0.52829300	-2.76631800
H	-2.89100700	-1.15895200	-3.68526100
H	-2.19667400	0.41587800	-4.10546100
C	-4.68699600	0.88739000	-3.00686700
H	-4.45113700	1.57439700	-3.82659200
H	-5.22418400	0.03413100	-3.43527100
H	-5.36700500	1.39826000	-2.31728400
C	0.68650900	2.35962300	-3.02920700
H	1.71387100	2.56775100	-3.34901800
H	0.66306300	1.34623200	-2.61197000
H	0.04533400	2.38171900	-3.91746900
C	0.19197600	4.80032000	-2.59052000
H	1.18611600	5.10925700	-2.93144000
H	-0.47839300	4.83851900	-3.45571100
H	-0.15393600	5.53613600	-1.85711700
C	1.08347300	-3.37008600	-0.69496900
C	2.21457500	-4.13234700	-1.43019700
H	2.18466600	-3.97025700	-2.51255000
C	3.51185300	-3.72164300	-0.85153000
H	3.43299100	-2.36166900	-1.37174300
C	2.69271900	-1.56419200	-0.93008200
Br	3.66777300	-0.04009600	-0.52159700
H	-4.02544800	2.47440500	0.90781400
H	-1.81060500	4.16430900	1.07330100
H	4.41314500	-3.83366900	-1.46171000
C	3.69279700	-3.84893100	0.62162000
C	1.14614400	-3.79847600	0.79149600
C	2.47440000	-3.43410700	1.45287500
H	2.54104900	-3.89356900	2.44393600
H	2.51542900	-2.34923500	1.60713200
H	2.07503500	-5.20688700	-1.24499300

H	0.97407500	-4.88309300	0.83357300
H	0.32295600	-3.32511300	1.33712900
H	3.89499900	-4.92539600	0.76760300
H	4.60228800	-3.33338600	0.94825000
C	-0.27791500	-3.69000100	-1.29873700
H	-1.07282800	-3.16699800	-0.75671000
H	-0.32600000	-3.37952400	-2.34779100
H	-0.47890800	-4.76612600	-1.24750700

Cartesian coordinates of the optimized geometry for TS-**3a** at PBE0-D3BJ/6-31G*,def2-TZVP level of theory: (number of imaginary frequencies = 1):

Au	0.14179100	-0.41011500	-0.28069700
C	1.83951700	-1.45955500	-0.68563300
C	-1.44583400	0.74990200	0.19547100
C	-2.62919100	2.57674800	0.77682200
C	-3.49891600	1.53871500	0.67751300
N	-1.38006500	2.07055800	0.47769400
N	-2.75167400	0.43229500	0.32326100
C	-3.26621600	-0.88954700	0.11195000
C	-3.63672500	-1.25677800	-1.18822900
C	-3.33997400	-1.75528800	1.21181200
C	-4.09803300	-2.56146500	-1.37255700
C	-3.80723200	-3.04824600	0.97213500
C	-4.18217300	-3.44711500	-0.30548000
H	-4.39375900	-2.88709900	-2.36572500
H	-3.87778100	-3.75222300	1.79614600
H	-4.54540800	-4.45752500	-0.47019300
C	-0.15647000	2.82055200	0.46443000
C	0.23115500	3.43865600	-0.73222300
C	0.60416800	2.87266200	1.64054800
C	1.42911500	4.15535900	-0.72030000
C	1.79260900	3.60380500	1.59918800
C	2.19841800	4.24411800	0.43404500
H	1.76328400	4.64956000	-1.62796500
H	2.40772300	3.67156300	2.49203500
H	3.12206600	4.81632800	0.42516400
C	-2.89385200	-1.33721700	2.59878800
H	-2.75248300	-0.25017700	2.59927800
C	-1.54235500	-1.97323400	2.93793900
H	-1.61604800	-3.06706900	2.94399200
H	-0.78169500	-1.68631500	2.20219500
H	-1.20218600	-1.65026400	3.92841100
C	-3.94126500	-1.65943900	3.66523100
H	-3.62143400	-1.26934900	4.63732900
H	-4.91092800	-1.21406900	3.42031200
H	-4.08715900	-2.73912300	3.77843400
C	0.19695600	2.14504100	2.90547100
H	-0.78515300	1.68852300	2.73812700
C	0.06310900	3.10138200	4.09172800
H	-0.28340400	2.56007400	4.97861300
H	1.02199100	3.56789600	4.34295400
H	-0.65236000	3.90251800	3.87917800
C	1.17987300	1.01097400	3.20958600
H	1.23798200	0.31123400	2.36758000
H	2.18735200	1.39974200	3.39676300
H	0.86087100	0.45592900	4.09884900
C	-0.57391400	3.31014400	-2.00883500
H	-1.51410000	2.79960800	-1.77108900
C	-3.52073200	-0.30707900	-2.36317900
H	-3.24866100	0.68242700	-1.97823300
C	-2.40137700	-0.75365800	-3.30754500
H	-1.44273500	-0.81616500	-2.77939400
H	-2.61715900	-1.73927900	-3.73560200
H	-2.29199700	-0.04340800	-4.13468700

C	-4.84954200	-0.15792000	-3.10610000
H	-4.75314300	0.58187500	-3.90793600
H	-5.16344900	-1.10156500	-3.56540600
H	-5.64919300	0.16900500	-2.43334800
C	0.17546800	2.44082700	-3.02272000
H	1.12630100	2.90300900	-3.31151300
H	0.39544300	1.45107000	-2.60623800
H	-0.42442300	2.30742500	-3.92982100
C	-0.93090600	4.67508700	-2.59931500
H	-0.03692500	5.22768800	-2.90833100
H	-1.56240300	4.54998800	-3.48529900
H	-1.47374900	5.29379300	-1.87713600
C	1.96146200	-2.95760900	-0.59681900
C	3.24673500	-3.39535700	-1.33664100
H	3.15829900	-3.12420500	-2.39651800
C	4.35724000	-2.61105900	-0.73413700
H	3.90928200	-1.33827800	-1.30376300
C	2.96842400	-0.78400000	-0.88638800
Br	3.45723900	0.96671500	-0.51363700
H	-4.56632600	1.48111400	0.82263100
H	-2.77814900	3.61533800	1.02754500
H	5.26747100	-2.47285100	-1.32655700
C	4.54031400	-2.64712000	0.74269900
C	2.07426300	-3.31030700	0.90103000
C	3.24401500	-2.57709400	1.55849100
H	3.42681500	-2.97470900	2.56151800
H	2.97987000	-1.52016400	1.68429100
H	2.17997200	-4.39595500	1.00614000
H	1.14254800	-3.03798600	1.40668000
H	5.04621500	-3.61332400	0.92098500
H	5.25925000	-1.88414500	1.06037600
H	1.08949300	-3.45806200	-1.02954100
C	3.51767500	-4.90722500	-1.26774800
H	2.64051500	-5.44399100	-1.64172400
H	4.37060800	-5.17539000	-1.89771800
H	3.72130300	-5.25490200	-0.25230600

Cartesian coordinates of the optimized geometry for TS-**3b** at PBE0-D3BJ/6-31G*,def2-TZVP level of theory: (number of imaginary frequencies = 1):

Au	0.11441600	-0.37746600	-0.38817400
C	1.68076300	-1.54233800	-0.97716900
C	-1.32254500	0.87057800	0.29466800
C	-2.28142200	2.72288200	1.14561800
C	-3.25932600	1.79682400	0.97253200
N	-1.10585600	2.13396700	0.72453300
N	-2.64827300	0.67207900	0.45317300
C	-3.30716000	-0.55711100	0.11906600
C	-3.77325200	-0.72469900	-1.19143100
C	-3.41965500	-1.53953100	1.11236000
C	-4.37592100	-1.94559100	-1.49986100
C	-4.02964500	-2.74155200	0.75080200
C	-4.50263700	-2.94259200	-0.54058700
H	-4.74902300	-2.11703500	-2.50545300
H	-4.13518700	-3.53054000	1.48976900
H	-4.97643100	-3.88478300	-0.80096400
C	0.18938100	2.75205800	0.73597600
C	0.59377600	3.46897800	-0.39843200
C	0.99674500	2.58275000	1.86918500
C	1.86117500	4.05341600	-0.36451900
C	2.25398800	3.18916200	1.85246200
C	2.68008600	3.92224900	0.75097700
H	2.21039500	4.61814400	-1.22411100
H	2.90725900	3.08498300	2.71415600
H	3.65855200	4.39460600	0.76151400

C	-2.87116200	-1.34023500	2.51120800
H	-2.60161800	-0.28388700	2.62563200
C	-1.59241000	-2.16065800	2.70489400
H	-1.79420300	-3.23237300	2.59230000
H	-0.83325700	-1.87684400	1.96644200
H	-1.17713300	-1.99656500	3.70572500
C	-3.90677200	-1.66459100	3.58858700
H	-3.50396300	-1.43379800	4.58053000
H	-4.82411100	-1.08372000	3.44840800
H	-4.17821400	-2.72578900	3.58437900
C	0.56358600	1.75273200	3.06035100
H	-0.46718600	1.42121500	2.89149600
C	0.57763000	2.56844900	4.35428400
H	0.20967300	1.96253300	5.18922700
H	1.58959100	2.90213100	4.60878800
H	-0.05615100	3.45750400	4.27190800
C	1.43119400	0.49683400	3.18192300
H	1.38073300	-0.10084600	2.26418100
H	2.48107600	0.75640800	3.35945000
H	1.09035100	-0.12419500	4.01787300
C	-0.26960300	3.57970300	-1.63812700
H	-1.24952300	3.14332700	-1.41369400
C	-3.61015300	0.34464100	-2.25241800
H	-3.22683200	1.25080500	-1.76935900
C	-2.57761800	-0.08857300	-3.29671300
H	-1.61046000	-0.30426500	-2.82777300
H	-2.90666900	-0.99097300	-3.82459800
H	-2.42978100	0.70232700	-4.04049100
C	-4.94499100	0.70635000	-2.90600300
H	-4.80645200	1.52623800	-3.61879700
H	-5.36745600	-0.14052800	-3.45761700
H	-5.68260600	1.02187100	-2.16083500
C	0.34126400	2.76743900	-2.78371200
H	1.32537000	3.15981200	-3.06415200
H	0.46705600	1.71669500	-2.49808600
H	-0.30355200	2.80894100	-3.66856600
C	-0.49975900	5.03558200	-2.04633100
H	0.43579400	5.52680100	-2.33526700
H	-1.17562000	5.08430300	-2.90669500
H	-0.94348000	5.61469200	-1.22977500
C	1.63091800	-3.04303400	-1.06019200
C	2.83218900	-3.52796400	-1.90044200
H	2.74570500	-3.24530100	-2.95366900
C	4.05807500	-2.98182400	-1.28596000
H	3.73388900	-1.58750900	-1.63625900
C	2.86433400	-0.96590700	-1.15115900
Br	3.54978900	0.67262900	-0.62050500
H	-4.32009600	1.82941500	1.16602000
H	-2.30950500	3.73327100	1.52251300
H	4.95060000	-2.86623700	-1.90747500
C	4.28040600	-3.22701700	0.16529400
C	1.74908700	-3.60783600	0.38037000
C	3.03040900	-3.08554600	1.04325200
H	3.19940700	-3.60523500	1.99216300
H	2.90569200	-2.02350100	1.28513300
H	0.89950400	-3.20494600	0.94444600
H	4.64915000	-4.26818800	0.20024900
H	5.10697200	-2.61526200	0.54206400
H	0.69389100	-3.38808200	-1.50761200
H	2.87491200	-4.62440500	-1.84991600
C	1.64244000	-5.13032000	0.41659700
H	1.54717600	-5.47559000	1.45103500
H	0.76160500	-5.47973500	-0.13321900
H	2.52106900	-5.63267700	-0.00567700

Cartesian coordinates of the optimized geometry for TS-**3c** at PBE0-D3BJ/6-31G*,def2-TZVP level of theory: (number of imaginary frequencies = 1):

Au	0.14642500	0.32760200	-0.22064000
C	1.40078200	1.88049800	-0.53024500
C	-0.98932000	-1.30082500	0.16574400
C	-2.69997900	-2.69346200	0.60593100
C	-1.55618400	-3.42580700	0.64665400
N	-2.32726400	-1.39743300	0.30981300
N	-0.52289200	-2.55179200	0.37516000
C	0.87444200	-2.87471100	0.32577000
C	1.43234300	-3.22518900	-0.91139600
C	1.62224900	-2.76699100	1.50644400
C	2.80448900	-3.48286500	-0.94128700
C	2.98988000	-3.03458400	1.42116600
C	3.57512600	-3.39019000	0.21178000
H	3.27416300	-3.75985700	-1.88084000
H	3.60339000	-2.96362700	2.31488500
H	4.64023200	-3.59992600	0.16774400
C	-3.21276000	-0.27668700	0.17129200
C	-3.75511400	-0.01407300	-1.09394700
C	-3.45279300	0.52217700	1.29699700
C	-4.58217100	1.10388100	-1.21096300
C	-4.28817000	1.62730400	1.12498700
C	-4.84763800	1.91455300	-0.11383600
H	-5.02012700	1.34504300	-2.17507800
H	-4.50137200	2.27113200	1.97366600
H	-5.49648400	2.77851000	-0.22610900
C	1.00351700	-2.34755900	2.82449200
H	-0.08018300	-2.26528900	2.68408700
C	1.51421900	-0.96700300	3.24552200
H	2.59806100	-0.98030200	3.40994400
H	1.29472900	-0.21678400	2.47684300
H	1.03699600	-0.65024400	4.17949700
C	1.24549200	-3.38872000	3.91887900
H	0.73760600	-3.09211000	4.84272800
H	0.86950900	-4.37357200	3.62307500
H	2.31181700	-3.49294500	4.14738900
C	-2.82490800	0.23839600	2.64631400
H	-2.25093400	-0.69219500	2.56949400
C	-3.88674700	0.03318800	3.72850600
H	-3.41276500	-0.22209700	4.68240000
H	-4.47976600	0.94069200	3.88613600
H	-4.57679600	-0.77428200	3.46243900
C	-1.84384300	1.34896600	3.03060900
H	-1.06782200	1.47005400	2.26585600
H	-2.35777800	2.31027100	3.14319100
H	-1.35591800	1.11526400	3.98346200
C	-3.42696400	-0.86264700	-2.30560900
H	-2.89948700	-1.76036700	-1.96279100
C	0.61094100	-3.29521100	-2.18247700
H	-0.43971400	-3.12481600	-1.92172800
C	1.02594400	-2.18657500	-3.15339900
H	0.91759500	-1.19844200	-2.69089600
H	2.07035600	-2.30393100	-3.46461100
H	0.40232600	-2.21251800	-4.05370000
C	0.70320800	-4.67415100	-2.83859000
H	0.05633500	-4.71742300	-3.72124200
H	1.72450600	-4.89656200	-3.16668400
H	0.39383100	-5.46616900	-2.14877100
C	-2.48069200	-0.10454900	-3.24144900
H	-2.95675500	0.80395900	-3.62756300
H	-1.56387000	0.19232300	-2.71897600
H	-2.20304000	-0.73070200	-4.09682500
C	-4.68399200	-1.32701300	-3.04229500
H	-5.23670700	-0.48423400	-3.47127900

H	-4.41263100	-1.99408000	-3.86739200
H	-5.36263400	-1.86757100	-2.37441100
C	2.78690200	4.15060100	-0.38038700
H	1.76366300	3.85944900	-1.17964600
C	1.02840700	3.16084300	-0.52435100
Br	-0.51380900	4.09947900	-0.13417000
H	-1.38240200	-4.47263800	0.84088400
H	-3.73223600	-2.96839200	0.75640500
H	3.15578300	4.71249300	-1.25219900
H	2.48192900	4.82684600	0.41597500
C	2.89057800	1.71619200	-0.75706800
C	3.52179800	0.40801600	-0.29317600
C	3.56274800	2.90592700	-0.04489900
H	3.05338100	1.78905000	-1.84772200
H	3.04757800	-0.43945500	-0.80030700
C	5.02405000	0.43231400	-0.57238600
H	5.48171000	-0.49202700	-0.20294600
H	5.18558900	0.44668400	-1.65987600
C	5.05665300	2.96408100	-0.36385700
H	5.19447800	3.12665200	-1.44261000
H	5.53211300	3.80514600	0.15343900
C	5.71078000	1.64505500	0.05635400
H	5.66691500	1.56167100	1.15124000
H	6.77245000	1.66105300	-0.21251300
H	3.43501900	2.72568600	1.03182200
H	3.33453800	0.27375600	0.78086300

Cartesian coordinates of the optimized geometry for TS-**4a** at PBE0-D3BJ/6-31G*,def2-TZVP level of theory: (number of imaginary frequencies = 1):

Au	0.11543100	-0.46423800	-0.22892200
C	1.72809300	-1.66920800	-0.54045900
C	-1.37418300	0.84948700	0.15282500
C	-2.40398800	2.79775500	0.62094500
C	-3.35949700	1.83588300	0.54614300
N	-1.19814900	2.17121500	0.37720600
N	-2.70528600	0.65329000	0.26065800
C	-3.32958300	-0.62761300	0.09940500
C	-3.71778500	-1.01909100	-1.18856600
C	-3.48833300	-1.43421800	1.23449700
C	-4.29029200	-2.28531000	-1.32190100
C	-4.06529100	-2.69083800	1.04533600
C	-4.46228600	-3.11175100	-0.21833500
H	-4.60353200	-2.62825200	-2.30379000
H	-4.20478000	-3.34850600	1.89844300
H	-4.91149900	-4.09280700	-0.34372900
C	0.08690900	2.81020200	0.36395600
C	0.55833300	3.32970500	-0.84942900
C	0.81823000	2.85734900	1.55872400
C	1.81398800	3.93959600	-0.83639100
C	2.06700700	3.47999100	1.51755800
C	2.55769100	4.02148800	0.33507100
H	2.21386200	4.35519800	-1.75679700
H	2.66228400	3.54059700	2.42424500
H	3.52798600	4.51043400	0.32600200
C	-3.02307200	-0.99597200	2.60876900
H	-2.75636200	0.06595300	2.55719200
C	-1.76196700	-1.76358800	3.01615100
H	-1.96173600	-2.83966400	3.07941400
H	-0.95881100	-1.60761400	2.28618800
H	-1.40595600	-1.42666500	3.99631200
C	-4.12350100	-1.13811300	3.66117500
H	-3.77962900	-0.74215700	4.62267200
H	-5.02702700	-0.59260700	3.37016300
H	-4.40104200	-2.18594400	3.81876300

C	0.31577300	2.23339200	2.84462400
H	-0.70175300	1.86460200	2.67258800
C	0.24797400	3.25334600	3.98246500
H	-0.17031600	2.79034300	4.88261900
H	1.24144400	3.63676800	4.23963800
H	-0.38093100	4.10861200	3.71436700
C	1.17872700	1.02717400	3.22602800
H	1.18671000	0.28412300	2.41988200
H	2.21484700	1.32750200	3.41958700
H	0.78988600	0.54959700	4.13233700
C	-0.22184600	3.20691100	-2.14201900
H	-1.20816500	2.78989900	-1.90898300
C	-3.50228000	-0.13818100	-2.40240200
H	-3.15833500	0.84337500	-2.05649500
C	-2.40362800	-0.71830800	-3.29740200
H	-1.46487000	-0.83374000	-2.74307600
H	-2.69110800	-1.70234000	-3.68511200
H	-2.21853100	-0.05938900	-4.15309100
C	-4.79846800	0.08228500	-3.18397100
H	-4.62530500	0.77429100	-4.01502500
H	-5.17807900	-0.85315300	-3.60916900
H	-5.58310900	0.50280400	-2.54648300
C	0.47542900	2.22869400	-3.09178900
H	1.47038400	2.59281900	-3.37206600
H	0.59651000	1.24487300	-2.62384200
H	-0.10964400	2.10391900	-4.00973600
C	-0.44508200	4.56661800	-2.80585500
H	0.50062100	5.02572200	-3.11399500
H	-1.06250200	4.45218400	-3.70313700
H	-0.95072800	5.26371100	-2.12955000
C	1.70500700	-3.16659000	-0.41173800
C	2.98279800	-3.76873600	-1.06368200
C	4.13023800	-3.06863500	-0.44204600
H	3.80600500	-1.76222900	-1.09060100
C	2.91920700	-1.10532700	-0.71435600
Br	3.55250500	0.60438500	-0.37222900
H	-4.43066700	1.87824700	0.66666000
H	-2.46613600	3.85600300	0.82086700
H	5.07021500	-3.01972300	-1.00150900
C	4.24356300	-3.05179500	1.04291700
C	1.73064200	-3.50182500	1.09434100
C	2.92154000	-2.84458000	1.79100900
H	3.02134500	-3.22553000	2.81193800
H	2.74008100	-1.76647900	1.87463000
H	1.76841600	-4.59384200	1.20345800
H	0.80021400	-3.16602700	1.56283700
H	4.65672300	-4.04812000	1.28328200
H	5.00753100	-2.33542500	1.36390300
H	0.81203300	-3.59968000	-0.87313700
H	3.03481900	-4.80801500	-0.69985100
C	2.98991800	-3.76481000	-2.58635800
H	3.90613100	-4.21754900	-2.97916500
H	2.14165800	-4.33980100	-2.96837800
H	2.90829000	-2.74881000	-2.98494300

Cartesian coordinates of the optimized geometry for TS-**4b** at PBE0-D3BJ/6-31G*,def2-TZVP level of theory: (number of imaginary frequencies = 1):

Au	-0.28810300	-0.21979500	-0.37863700
C	-2.00388000	-1.14241200	-0.98187100
C	1.39237700	0.73889800	0.22631400
C	3.53181500	1.23175000	0.73017200
C	2.78349300	2.32830600	1.01178300
N	2.65944700	0.27150600	0.25628800
N	1.47905600	2.00446300	0.69262100

C	0.35217100	2.87982700	0.83252500
C	0.07182600	3.77025300	-0.21370200
C	-0.42142400	2.78878400	1.99755300
C	-1.03411500	4.60694800	-0.05807100
C	-1.51572600	3.64993800	2.10294700
C	-1.81680600	4.55122200	1.08959800
H	-1.28617100	5.31121100	-0.84526500
H	-2.13765300	3.61391300	2.99295600
H	-2.66926600	5.21649000	1.19390700
C	3.02598500	-1.05234700	-0.15686100
C	3.22877300	-1.28993600	-1.52225600
C	3.13612600	-2.04519300	0.82750600
C	3.54959200	-2.59572000	-1.89866900
C	3.45700200	-3.33255400	0.39638800
C	3.66023300	-3.60534800	-0.95164600
H	3.71603700	-2.82222100	-2.94802500
H	3.54791400	-4.13320500	1.12390100
H	3.91060000	-4.61469500	-1.26534800
C	-0.11810500	1.79795300	3.10324100
H	0.79256700	1.25087100	2.83470900
C	-1.24423000	0.76924900	3.23550300
H	-2.19089900	1.24898200	3.50949700
H	-1.39602500	0.22934800	2.29308500
H	-1.00198000	0.03541800	4.01195700
C	0.14696000	2.50765200	4.43259500
H	0.41607600	1.77899200	5.20477900
H	0.96591300	3.22890200	4.34291300
H	-0.73866000	3.04954200	4.78238000
C	2.86673900	-1.75553400	2.29130000
H	2.93668500	-0.67188700	2.44286600
C	3.89116800	-2.40788500	3.21929700
H	3.72094600	-2.08311400	4.25114500
H	3.81485000	-3.50050000	3.20743200
H	4.91509600	-2.13855800	2.94053700
C	1.44078800	-2.17652900	2.65966800
H	0.70143400	-1.66175500	2.03659900
H	1.30701900	-3.25512100	2.51578200
H	1.23007700	-1.94370200	3.70991100
C	3.10946400	-0.19903700	-2.56710500
H	2.87304200	0.74184100	-2.05720900
C	0.89146000	3.79444000	-1.48865500
H	1.82122600	3.24316500	-1.30733800
C	0.14285000	3.06844200	-2.61101600
H	-0.09093400	2.03625000	-2.32486800
H	-0.79883200	3.57963300	-2.84434200
H	0.74844500	3.04336000	-3.52396900
C	1.27886300	5.21227400	-1.90883600
H	1.94255900	5.17765000	-2.77914900
H	0.40351800	5.80733800	-2.19095400
H	1.79893900	5.74077400	-1.10333100
C	1.96239900	-0.49115600	-3.53700700
H	2.14252000	-1.41325500	-4.10092100
H	1.01466300	-0.60486500	-2.99752100
H	1.85392600	0.32728900	-4.25741600
C	4.43103400	0.00257200	-3.31188700
H	4.71544300	-0.89465000	-3.87250500
H	4.34265800	0.82713800	-4.02757600
H	5.24692700	0.23611100	-2.61991500
C	-2.97606500	-0.49904200	-1.93577900
C	-3.86370600	-1.60204500	-2.55651100
H	-3.31115500	-2.25714000	-3.23702000
C	-4.46771200	-2.35982800	-1.44072600
H	-3.15298900	-2.96257300	-1.05335700
C	-2.36052100	-2.32844800	-0.49286400
Br	-1.92030000	-3.23528200	1.06466700
H	3.04961500	3.29778200	1.40296600

H	4.59000400	1.04263400	0.82122600
H	-4.78834900	-3.39027300	-1.61872200
C	-5.20540100	-1.60948400	-0.38908700
C	-3.91042400	0.46531600	-1.15207900
C	-4.56899800	-0.27541200	0.01421600
H	-5.33030900	0.36159400	0.47633400
H	-3.81228900	-0.46791800	0.78580000
H	-6.19697400	-1.42756600	-0.84214500
H	-5.39658600	-2.24852800	0.48001500
H	-2.45896200	0.05972500	-2.72206700
H	-4.66553900	-1.11652300	-3.12941700
H	-4.69512700	0.75678600	-1.86690700
C	-3.22054200	1.73913800	-0.68430000
H	-2.45976500	1.53895300	0.07695900
H	-2.72952100	2.25299300	-1.51686500
H	-3.95038600	2.42992300	-0.24899700

Cartesian coordinates of the optimized geometry for TS-**4c** at PBE0-D3BJ/6-31G*,def2-TZVP level of theory: (number of imaginary frequencies = 1):

Au	0.01854400	0.38890300	-0.34420800
C	-0.07393900	2.34624700	-0.85634900
C	0.11136400	-1.55719800	0.19684200
C	-0.47961900	-3.64678100	0.78454900
C	0.87699100	-3.60839500	0.71598300
N	-0.92493400	-2.38052600	0.46169500
N	1.21503400	-2.31821300	0.35831300
C	2.54001100	-1.79469500	0.18912000
C	3.06412500	-1.71789900	-1.10825700
C	3.21455900	-1.32545500	1.32544400
C	4.32324700	-1.13094600	-1.25118200
C	4.46919500	-0.74766600	1.12609900
C	5.01648200	-0.64805300	-0.14808600
H	4.76694500	-1.05591400	-2.23998400
H	5.02439800	-0.36904700	1.97910900
H	5.99581300	-0.19701600	-0.28094800
C	-2.29160800	-1.94557900	0.40911400
C	-2.99097700	-2.10148100	-0.79557700
C	-2.83679800	-1.33446700	1.54600600
C	-4.30009800	-1.61984900	-0.83757500
C	-4.15154000	-0.87407900	1.45127500
C	-4.87523800	-1.01453800	0.27354200
H	-4.87525800	-1.71716600	-1.75365000
H	-4.61299400	-0.40004700	2.31313200
H	-5.89776400	-0.65135300	0.22095300
C	2.60288900	-1.39278300	2.71119400
H	1.74557500	-2.07464400	2.67348500
C	2.07326800	-0.01871200	3.13172800
H	2.88552800	0.71620900	3.17553200
H	1.32117900	0.34839800	2.42433900
H	1.61197200	-0.07294400	4.12426200
C	3.57842100	-1.94978400	3.74862500
H	3.07159000	-2.07365900	4.71138100
H	3.97554700	-2.92329500	3.44341900
H	4.42706200	-1.27683200	3.91218200
C	-2.05210300	-1.14917400	2.82880900
H	-1.05701100	-1.58705700	2.69017900
C	-2.71617800	-1.87882800	3.99844500
H	-2.11052800	-1.77469300	4.90514700
H	-3.70923700	-1.46924900	4.21372200
H	-2.83457200	-2.94664900	3.78688800
C	-1.85596000	0.33696100	3.13778300
H	-1.35331000	0.84751700	2.30809100
H	-2.81530400	0.83806100	3.30920200
H	-1.24667800	0.46201800	4.03962300

C	-2.35367500	-2.71492200	-2.02588800
H	-1.39649900	-3.15974800	-1.73010300
C	2.32217200	-2.24281200	-2.32077900
H	1.36273800	-2.65263000	-1.98568300
C	2.01492000	-1.11969700	-3.31342200
H	1.42105800	-0.32912900	-2.83963400
H	2.93403200	-0.66800100	-3.70357600
H	1.44626300	-1.50863300	-4.16508800
C	3.09934600	-3.37905800	-2.99008600
H	2.52959000	-3.78471200	-3.83298200
H	4.06317100	-3.03018400	-3.37698200
H	3.29763900	-4.19468100	-2.28695500
C	-2.05298900	-1.63057600	-3.06497600
H	-2.97611300	-1.14869000	-3.40678100
H	-1.40257700	-0.85354300	-2.64664700
H	-1.55414800	-2.06423200	-3.93901500
C	-3.21032600	-3.83319300	-2.62113100
H	-4.16512600	-3.45371100	-3.00083400
H	-2.68731700	-4.30196400	-3.46139400
H	-3.42897300	-4.60806800	-1.87913400
C	-0.61509600	4.94559400	-0.90329400
H	-1.25636500	3.97526600	-1.51205200
C	-1.21746100	3.01954100	-0.75953200
Br	-2.91153500	2.70173500	-0.09885100
H	1.62429600	-4.36733800	0.88757300
H	-1.16183400	-4.44610900	1.02840900
H	-0.90704300	5.54660800	-1.77674900
H	-1.14562500	5.28170400	-0.01273900
C	1.07169400	3.19745200	-1.37809500
C	2.47892200	2.66466700	-1.10124300
C	0.85265600	4.61215000	-0.78295100
H	0.94410500	3.22377900	-2.47324900
C	2.86425800	2.74549800	0.36922100
H	3.18557000	3.26001300	-1.69528700
H	2.54856100	1.63371000	-1.46598200
C	1.29361100	4.67382000	0.68343300
H	1.42728000	5.33817600	-1.37497000
C	2.71955100	4.17220000	0.88487200
H	3.88885900	2.38174400	0.50125400
H	2.21991800	2.07374100	0.95321600
H	1.18933600	5.70326300	1.04610200
H	0.60954600	4.05271400	1.28032500
H	2.97816300	4.22901500	1.94790200
H	3.42213800	4.83102000	0.35516400

Cartesian coordinates of the optimized geometry for TS-**4d** at PBE0-D3BJ/6-31G*,def2-TZVP level of theory: (number of imaginary frequencies = 1):

Au	-0.27060100	0.24738800	-0.27229600
C	-2.19738100	0.76785600	-0.66276600
C	1.59268500	-0.40184300	0.17262200
C	3.26673100	-1.80170700	0.72933500
C	3.79669300	-0.55779400	0.60147800
N	1.91776000	-1.68157800	0.46236400
N	2.75325700	0.28110000	0.26271400
C	2.85417700	1.69370600	0.03586200
C	3.06283500	2.14182400	-1.27500800
C	2.70222800	2.55244600	1.13319400
C	3.11459200	3.52277900	-1.47315200
C	2.76098400	3.92350000	0.87921300
C	2.96466800	4.40353800	-0.40918500
H	3.27346200	3.91211200	-2.47469400
H	2.64538100	4.62435800	1.70082900
H	3.00956000	5.47456300	-0.58489800
C	0.96016000	-2.75036800	0.48857000

C	0.73303500	-3.46827300	-0.69369100
C	0.27705100	-3.00073600	1.68644600
C	-0.21316300	-4.49339400	-0.64463200
C	-0.65684500	-4.03864100	1.68216200
C	-0.89594700	-4.78120100	0.53157200
H	-0.41681100	-5.07365900	-1.53994900
H	-1.20289600	-4.26727000	2.59303900
H	-1.61967800	-5.59145600	0.55166900
C	2.44303200	2.03525100	2.53423100
H	2.61628100	0.95284000	2.53703500
C	0.98047000	2.26401400	2.92660400
H	0.73903800	3.33346200	2.92993600
H	0.30359900	1.76376200	2.22432200
H	0.78487800	1.87001200	3.93030200
C	3.39530700	2.65011600	3.56076200
H	3.23479100	2.19456600	4.54366600
H	4.44168400	2.49485200	3.27861100
H	3.23390300	3.72802000	3.66974700
C	0.49735200	-2.17445900	2.93690200
H	1.30052100	-1.45620300	2.73687700
C	0.94017600	-3.04168800	4.11671000
H	1.14362900	-2.41595000	4.99228900
H	0.16537300	-3.76300300	4.39884600
H	1.84938800	-3.60391600	3.87967400
C	-0.76010200	-1.36938000	3.27606900
H	-1.05030900	-0.72456400	2.43822900
H	-1.60480200	-2.03015000	3.50162300
H	-0.58365300	-0.73667100	4.15297800
C	1.44040300	-3.13732000	-1.99170000
H	2.19992500	-2.37517400	-1.78341200
C	3.20048300	1.18993800	-2.44581500
H	3.23008200	0.16673200	-2.05399700
C	1.98335800	1.29051300	-3.36910000
H	1.05660200	1.08155700	-2.82199500
H	1.90070600	2.29270500	-3.80492900
H	2.06613200	0.57126900	-4.19152500
C	4.50266200	1.42231200	-3.21429600
H	4.60693300	0.68167900	-4.01437300
H	4.52531100	2.41412600	-3.67880900
H	5.37455400	1.34093800	-2.55698500
C	0.45402000	-2.53712100	-2.99754300
H	-0.33150000	-3.25613400	-3.25660800
H	-0.02931000	-1.64232400	-2.58856800
H	0.97241600	-2.25647700	-3.92110700
C	2.16096300	-4.35407200	-2.57462200
H	1.45631200	-5.14479200	-2.85439200
H	2.71208100	-4.07026300	-3.47745500
H	2.87270500	-4.77826100	-1.85877000
H	-4.10775700	0.00784700	-1.35950100
C	-3.09833700	-0.21205900	-0.75138500
Br	-3.10946200	-2.00297100	-0.28216200
H	4.80514100	-0.19272300	0.71770500
H	3.71575800	-2.74971200	0.98113200
C	-2.73824800	2.16983300	-0.69920800
C	-2.66632700	2.70515500	0.74788400
C	-4.18698000	2.14045400	-1.24610200
H	-2.11860700	2.81573400	-1.33205800
H	-1.61142500	2.80867700	1.02242000
C	-3.40961900	4.02485500	0.91390500
H	-3.32940900	4.35931400	1.95400800
H	-2.93269000	4.79767100	0.29483200
C	-4.97460600	3.43039200	-0.94420000
H	-4.55715300	4.21377400	-1.58959200
H	-6.01939400	3.30522900	-1.25018700
C	-4.87093600	3.87906000	0.50802300
H	-5.36848100	3.15820800	1.17275100

H	-5.40381800	4.82804600	0.63078600
H	-3.08822000	1.95972800	1.43786800
C	-4.84105800	0.91148900	-0.70030300
H	-4.92933800	0.79879600	0.37906200
H	-5.72382200	0.52416400	-1.22056500
H	-4.14266300	2.02376300	-2.33738000

Cartesian coordinates of the optimized geometry for TS-**5a** at PBE0-D3BJ/6-31G*,def2-TZVP level of theory: (number of imaginary frequencies = 1):

Au	0.09141600	-0.43158500	-0.24524500
C	1.83155000	-1.47995400	-0.56743800
C	-1.52892200	0.69011300	0.17699600
C	-2.76027400	2.48119200	0.76223600
C	-3.60587100	1.42770100	0.62430500
N	-1.49400100	2.00581000	0.48388000
N	-2.82899700	0.34276700	0.26653300
C	-3.30968800	-0.98655700	0.02232400
C	-3.68330900	-1.32564600	-1.28494700
C	-3.34722300	-1.88549500	1.09679100
C	-4.11417200	-2.63538300	-1.50221200
C	-3.78559200	-3.18229900	0.82420400
C	-4.16561300	-3.55304400	-0.46001600
H	-4.41045400	-2.94078800	-2.50148800
H	-3.82863200	-3.91123300	1.62835600
H	-4.50542100	-4.56702200	-0.65047000
C	-0.28311000	2.77588900	0.51324100
C	0.12402100	3.41805300	-0.66439800
C	0.44831900	2.82046900	1.70843500
C	1.30988000	4.15322700	-0.61206500
C	1.62547000	3.57119400	1.70729000
C	2.04877900	4.23670300	0.56254100
H	1.65675300	4.66841500	-1.50318600
H	2.21659800	3.63562000	2.61643200
H	2.96145400	4.82601800	0.58616400
C	-2.90235700	-1.49971100	2.49322700
H	-2.72339000	-0.41840700	2.50996600
C	-1.57930500	-2.18661800	2.84403700
H	-1.69007600	-3.27711600	2.84355000
H	-0.79913900	-1.92369600	2.11950800
H	-1.23989900	-1.88132200	3.84022400
C	-3.97810500	-1.79948600	3.53841500
H	-3.65597800	-1.44771900	4.52432100
H	-4.92334400	-1.30552000	3.29102900
H	-4.17367600	-2.87411000	3.62061900
C	0.02171500	2.06946100	2.95313900
H	-0.94301900	1.59038400	2.75170200
C	-0.17251700	3.01187500	4.14239100
H	-0.53130800	2.45408400	5.01395100
H	0.76621800	3.50034400	4.42586800
H	-0.90170100	3.79634300	3.91481000
C	1.02279500	0.95748400	3.27929800
H	1.12572400	0.26350000	2.43676500
H	2.01387700	1.36859900	3.50243900
H	0.68971200	0.38831000	4.15420600
C	-0.64779900	3.29766400	-1.96224400
H	-1.58448800	2.76740900	-1.75617400
C	-3.58976000	-0.34218900	-2.43397200
H	-3.37323200	0.64929600	-2.01956800
C	-2.42907900	-0.71425900	-3.36118200
H	-1.48047200	-0.74570000	-2.81262800
H	-2.58952500	-1.69888200	-3.81494600
H	-2.33397500	0.01883300	-4.17000300
C	-4.90566200	-0.23532500	-3.20601200
H	-4.82602400	0.53301100	-3.98236200

H	-5.16115600	-1.17756200	-3.70270900
H	-5.73688900	0.03106500	-2.54502000
C	0.14050800	2.45851000	-2.97208500
H	1.09024200	2.94096700	-3.22981200
H	0.36552200	1.46451900	-2.56825200
H	-0.43447300	2.33125600	-3.89590000
C	-1.01350300	4.66636400	-2.53869200
H	-0.12195600	5.23872800	-2.81723200
H	-1.62164600	4.54562700	-3.44139000
H	-1.58367600	5.26381200	-1.81976300
C	1.99355300	-2.97711500	-0.60853000
C	3.26229700	-3.29775900	-1.42005400
H	3.14424300	-3.01562000	-2.47138400
C	4.43757700	-2.59791500	-0.80133600
H	4.00875200	-1.44560000	-0.77113000
C	2.85976200	-0.68948600	-0.60024500
Br	3.46139800	1.00665400	-0.35958500
H	-4.67481400	1.34582500	0.74409200
H	-2.93632300	3.51188800	1.02754600
C	4.65695100	-2.99124600	0.64741600
C	2.16348400	-3.51199700	0.82595800
C	3.39302900	-2.91578900	1.51031200
H	3.57286800	-3.42796800	2.46125100
H	3.19464600	-1.86625600	1.76120400
H	2.25337700	-4.60432700	0.76436400
H	1.26595300	-3.30031600	1.41703400
H	5.02826900	-4.02764500	0.62228300
H	5.46107800	-2.39089700	1.08795200
H	1.12107400	-3.44004600	-1.07709600
C	5.67882200	-2.45202800	-1.64573700
H	6.40813200	-1.78415000	-1.17778600
H	6.15301200	-3.43643300	-1.75408100
H	5.44399400	-2.08123000	-2.64764300
H	3.43351400	-4.38154800	-1.37703700

Cartesian coordinates of the optimized geometry for TS-**5b** at PBE0-D3BJ/6-31G*,def2-TZVP level of theory: (number of imaginary frequencies = 1):

Au	0.06830000	-0.36120800	-0.50323500
C	1.64497900	-1.40245800	-1.26899100
C	-1.36839000	0.75146800	0.38419700
C	-2.33760100	2.46295700	1.48237900
C	-3.29085500	1.51992400	1.26836100
N	-1.17023300	1.97086900	0.93354800
N	-2.67361400	0.48182500	0.59795600
C	-3.30654700	-0.73543500	0.18015600
C	-3.83577500	-0.79746000	-1.11536300
C	-3.33242900	-1.81020000	1.07981600
C	-4.41218800	-2.00676100	-1.50845100
C	-3.91949200	-2.99581100	0.63596100
C	-4.45349200	-3.09324000	-0.64370200
H	-4.83267200	-2.09765800	-2.50584100
H	-3.95858000	-3.85427000	1.30010000
H	-4.90790100	-4.02473900	-0.96893600
C	0.10109000	2.63693800	0.93832900
C	0.40975000	3.48645800	-0.13283800
C	0.98187200	2.38151500	1.99850700
C	1.65641400	4.11438300	-0.10940300
C	2.21411600	3.03708700	1.97509000
C	2.54645600	3.89901400	0.93635400
H	1.93250800	4.78092700	-0.92132500
H	2.92240900	2.86910800	2.78145700
H	3.50704800	4.40683000	0.94154300
C	-2.71452200	-1.72099900	2.46123300
H	-2.49459500	-0.66758800	2.67007800

C	-1.38479500	-2.48048900	2.50014800
H	-1.53665000	-3.54500500	2.28628500
H	-0.68634700	-2.07874600	1.75661500
H	-0.92116400	-2.39521600	3.48965000
C	-3.66497000	-2.21169700	3.55387900
H	-3.21790000	-2.05337200	4.54105200
H	-4.62025200	-1.67791500	3.52163400
H	-3.87567900	-3.28234000	3.45863100
C	0.65228900	1.41226100	3.11559400
H	-0.37736600	1.06405500	2.97584000
C	0.72857100	2.08169500	4.48876400
H	0.43242900	1.37584900	5.27217000
H	1.74546400	2.41995500	4.71603800
H	0.06676300	2.95212000	4.54517200
C	1.56202800	0.18252600	3.04203600
H	1.46446200	-0.31373100	2.06938300
H	2.61389800	0.45928900	3.17697400
H	1.29796000	-0.53694200	3.82530700
C	-0.53395200	3.69537200	-1.29926000
H	-1.48445500	3.20347900	-1.06327100
C	-3.76913300	0.37272100	-2.07547400
H	-3.37926700	1.24057700	-1.53122300
C	-2.79579900	0.07549200	-3.21932200
H	-1.79502100	-0.15375300	-2.83493100
H	-3.13476600	-0.78148200	-3.81248500
H	-2.71684300	0.93901000	-3.88911000
C	-5.15319300	0.75081800	-2.60601400
H	-5.08358700	1.63734900	-3.24533200
H	-5.58893500	-0.05467300	-3.20708500
H	-5.84780700	0.97192900	-1.78897600
C	0.02479800	3.03004600	-2.56034400
H	0.97851300	3.48238700	-2.85509100
H	0.19584200	1.95959300	-2.39849900
H	-0.67540100	3.14417700	-3.39530700
C	-0.83195000	5.17687700	-1.53511600
H	0.06779100	5.72825800	-1.82918400
H	-1.56473600	5.29086000	-2.34095800
H	-1.23657400	5.65194800	-0.63534400
C	1.65858300	-2.89458800	-1.45308000
C	2.86353500	-3.27977700	-2.33842200
H	2.74639600	-2.95598000	-3.37682100
C	4.07034100	-2.69459800	-1.71520500
H	3.67501000	-1.31937300	-1.99526600
C	2.79806300	-0.76606900	-1.43743900
Br	3.44770100	0.84785600	-0.79966200
H	-4.33813300	1.48783100	1.52513400
H	-2.37815400	3.42653600	1.96570900
H	4.94509300	-2.50595900	-2.34455800
C	4.33862200	-3.00906700	-0.28370200
C	1.86355200	-3.53719800	-0.06563300
C	3.11072700	-2.99990200	0.64086800
H	2.92000900	-1.94826600	0.89406000
H	4.75921700	-4.03157200	-0.31483000
H	5.14061000	-2.37598000	0.11226900
H	0.72656300	-3.25542900	-1.89689000
H	2.96132200	-4.37429100	-2.33052700
H	1.93785400	-4.62624700	-0.19478600
H	0.98540800	-3.35093800	0.56146300
C	3.39341800	-3.75026000	1.93548600
H	3.56688800	-4.81703400	1.75045200
H	4.27585400	-3.34771600	2.44468400
H	2.54443900	-3.66347900	2.62171000

Cartesian coordinates of the optimized geometry for TS-**6** at PBE0-D3BJ/6-31G*,def2-TZVP level of theory: (number of imaginary frequencies = 1):

Au	0.09393000	-0.41771200	-0.40148600
C	1.63090000	-1.60204400	-1.02752000
C	-1.32642800	0.85291700	0.27748500
C	-2.28825200	2.73895300	1.04750600
C	-3.25661000	1.79128500	0.95842600
N	-1.11593000	2.14165200	0.62879700
N	-2.64429700	0.64722500	0.48486400
C	-3.29531900	-0.60565700	0.23458400
C	-3.85618200	-0.81908300	-1.03188900
C	-3.30685700	-1.56342400	1.25723100
C	-4.45319200	-2.05965000	-1.26124900
C	-3.91718200	-2.78680400	0.97537500
C	-4.48447800	-3.03226300	-0.26906000
H	-4.89718500	-2.26728800	-2.23040100
H	-3.94807400	-3.55679400	1.74108400
H	-4.95654800	-3.99025600	-0.46754700
C	0.17062500	2.77479200	0.56470900
C	0.57435000	3.33999100	-0.65240600
C	0.97190100	2.76622300	1.71508500
C	1.84033600	3.92733000	-0.69284500
C	2.22966200	3.36387000	1.61981700
C	2.65831800	3.94234300	0.43026100
H	2.18827800	4.37897300	-1.61745700
H	2.88114000	3.37747200	2.48862400
H	3.63717800	4.41123000	0.37949400
C	-2.66410200	-1.31711000	2.60737500
H	-2.33754900	-0.27162700	2.64659400
C	-1.41693200	-2.18942400	2.77553600
H	-1.67353400	-3.25475100	2.74410400
H	-0.69348500	-1.98766500	1.97667700
H	-0.93256100	-1.98608900	3.73736300
C	-3.65226400	-1.53157300	3.75505000
H	-3.18021000	-1.28606300	4.71241400
H	-4.53975200	-0.90089400	3.63973900
H	-3.98725200	-2.57306900	3.81012800
C	0.53233000	2.09185200	2.99891100
H	-0.53908000	1.87364000	2.92113600
C	0.72646700	2.98634800	4.22344100
H	0.32040900	2.49644400	5.11475200
H	1.78580000	3.18747300	4.41642300
H	0.21891200	3.94871000	4.10123800
C	1.26030400	0.75467100	3.16376200
H	1.07085900	0.09891700	2.30675100
H	2.34355200	0.90524600	3.23871700
H	0.92430900	0.24207600	4.07237700
C	-0.29663900	3.31555100	-1.89154000
H	-1.24501800	2.82982400	-1.63550500
C	-3.78443800	0.22104800	-2.13170700
H	-3.44158200	1.16353400	-1.68928100
C	-2.75343100	-0.19114900	-3.18672600
H	-1.76405600	-0.33402700	-2.73662800
H	-3.04386400	-1.13052900	-3.67116000
H	-2.67003600	0.57788100	-3.96293500
C	-5.15077700	0.48715400	-2.76475300
H	-5.07447000	1.29719700	-3.49780100
H	-5.53298300	-0.39436800	-3.29076500
H	-5.89151000	0.77582600	-2.01189800
C	0.35649000	2.48450500	-2.99849700
H	1.30753800	2.92488000	-3.31840100
H	0.55608600	1.46258900	-2.65616100
H	-0.30050600	2.43209200	-3.87363200
C	-0.62061300	4.73140200	-2.37302400
H	0.28474400	5.26813700	-2.67724500
H	-1.29054500	4.69415100	-3.23873500

H	-1.10873500	5.31833000	-1.58789800
C	1.51499200	-3.07971500	-1.27169700
C	2.71576300	-3.54619100	-2.12648000
H	2.68292300	-3.16566400	-3.15124400
C	3.94116400	-3.12843300	-1.42254900
H	3.68803900	-1.67860600	-1.65575900
C	2.84055000	-1.06567500	-1.14030700
Br	3.57109800	0.51620200	-0.50491400
H	-4.31142900	1.82061000	1.18253300
H	-2.32070800	3.76922100	1.36575000
H	4.85640600	-2.97172100	-2.00058900
C	4.12697300	-3.54636100	-0.00885700
C	1.56344400	-3.84474900	0.06322800
C	2.86015800	-3.65206700	0.87812500
H	4.59239000	-4.54405200	-0.11251200
H	4.89855500	-2.93106100	0.46878200
H	0.57847400	-3.32007500	-1.78383400
H	2.69580500	-4.64502600	-2.16481400
H	1.43101400	-4.90836100	-0.17451700
H	0.70648600	-3.55611100	0.68028300
C	2.78632900	-2.50415200	1.88822100
H	2.73273900	-1.52009500	1.41852200
H	1.90084500	-2.61678100	2.52259400
H	3.66489200	-2.51744300	2.54279500
H	2.98182200	-4.56427000	1.47347700

Cartesian coordinates of the optimized geometry for TS-**7** at PBE0-D3BJ/6-31G*,def2-TZVP level of theory: (number of imaginary frequencies = 1):

Au	0.10730600	-0.38041900	-0.38654300
C	1.84600500	-1.26038800	-0.98275800
C	-1.51775700	0.60987500	0.29872200
C	-2.77379100	2.27955000	1.14146200
C	-3.57961900	1.19784900	0.98581400
N	-1.51778500	1.89630400	0.71529100
N	-2.78862600	0.18902600	0.47132300
C	-3.23121300	-1.13779500	0.15449600
C	-3.67130800	-1.39614400	-1.15021800
C	-3.17039000	-2.11373200	1.15866100
C	-4.06278200	-2.70424700	-1.44100000
C	-3.57250800	-3.40504700	0.81457800
C	-4.01399800	-3.69725600	-0.47055300
H	-4.40874600	-2.94740400	-2.44152000
H	-3.53888700	-4.19219900	1.56217100
H	-4.32468800	-4.70849200	-0.71723300
C	-0.34914900	2.72939400	0.70623000
C	-0.08340400	3.48794100	-0.44198600
C	0.48305300	2.72056400	1.83400600
C	1.06375500	4.28334100	-0.42901200
C	1.61603400	3.53531000	1.79629300
C	1.90089100	4.31349400	0.68021500
H	1.30374100	4.88642200	-1.29991300
H	2.28372400	3.55941100	2.65287300
H	2.78287500	4.94808800	0.67439500
C	-2.65229500	-1.80899000	2.55010300
H	-2.56867700	-0.72091800	2.65333000
C	-1.24893900	-2.39369600	2.73641400
H	-1.26379000	-3.48532100	2.63605600
H	-0.55734800	-1.99109300	1.98682100
H	-0.85876600	-2.14943700	3.73096900
C	-3.60578600	-2.29696800	3.64145100
H	-3.24028600	-1.98866700	4.62675800
H	-4.61155800	-1.88626800	3.50601100
H	-3.68808300	-3.38918800	3.65027100
C	0.21029200	1.84391100	3.03915400

H	-0.75506600	1.34682600	2.89093000
C	0.11212900	2.66263700	4.32736400
H	-0.14035300	2.01243200	5.17182000
H	1.06055900	3.15738900	4.56373000
H	-0.65819700	3.43689800	4.25000600
C	1.27379300	0.74795400	3.15291500
H	1.30696100	0.14269600	2.23929300
H	2.26955300	1.17802500	3.31017600
H	1.05324200	0.08634000	3.99808800
C	-0.96365000	3.43014400	-1.67338700
H	-1.84874800	2.82975200	-1.43407800
C	-3.69785700	-0.32680700	-2.22331200
H	-3.46484100	0.63570900	-1.75358900
C	-2.61857200	-0.59499900	-3.27577300
H	-1.62425400	-0.64269700	-2.81654100
H	-2.79914700	-1.54510600	-3.79143700
H	-2.61129700	0.20120300	-4.02849900
C	-5.08078100	-0.19848200	-2.86428500
H	-5.08706600	0.62398400	-3.58748700
H	-5.36320900	-1.11047200	-3.40137600
H	-5.85255900	0.00026800	-2.11343800
C	-0.22954700	2.72656600	-2.81838200
H	0.66628300	3.28425600	-3.11429600
H	0.08359600	1.71817100	-2.52435600
H	-0.88028700	2.64290800	-3.69588300
C	-1.45164100	4.81794300	-2.09190000
H	-0.62031500	5.46391500	-2.39467400
H	-2.13291900	4.73733400	-2.94558100
H	-1.98377100	5.31782600	-1.27582300
C	2.05886500	-2.74623300	-1.06575200
C	3.33542000	-3.02471300	-1.88932600
H	3.21733700	-2.78104900	-2.94931300
C	4.43797600	-2.25787100	-1.27321100
H	3.87093200	-0.94962100	-1.65592300
C	2.91720100	-0.49322600	-1.15493000
Br	3.31861900	1.23412200	-0.61233200
H	-4.62878200	1.05007600	1.18867200
H	-2.97194500	3.27415600	1.50918400
H	5.29572000	-1.98938500	-1.89758800
C	4.72451600	-2.44474700	0.18148600
C	2.29830900	-3.27402000	0.36424400
C	3.45507300	-2.54106000	1.04493000
H	3.70533600	-3.03469500	1.98993000
H	3.13143500	-1.52542500	1.30005200
H	5.33920600	-1.60263700	0.52390900
H	3.56725500	-4.09624100	-1.81223600
H	2.49394900	-4.35247100	0.30369300
H	1.38870800	-3.15107500	0.96062000
H	1.19830000	-3.25313600	-1.51106200
C	5.59685800	-3.71628200	0.29300300
H	6.47831700	-3.67151000	-0.35467200
H	5.94365200	-3.81461700	1.32573700
H	5.02786700	-4.61605300	0.03868500

Cartesian coordinates of the optimized geometry for TS-**8** at PBE0-D3BJ/6-31G*,def2-TZVP level of theory: (number of imaginary frequencies = 1):

Au	-0.08346100	-0.25182500	-0.39153700
C	1.83975900	-0.53961300	-1.00593200
C	-1.92835500	0.17665400	0.31690200
C	-3.62894500	1.36214000	1.19975500
C	-4.06226100	0.08662600	1.03038800
N	-2.32163800	1.39330400	0.75673500
N	-3.00445000	-0.61966000	0.49129800
C	-3.01921600	-2.01317300	0.15333700

C	-3.37956400	-2.37525000	-1.15117100
C	-2.64164300	-2.93752500	1.13689300
C	-3.35168900	-3.73560800	-1.46344300
C	-2.62999100	-4.28447700	0.77191000
C	-2.98139800	-4.67943100	-0.51350500
H	-3.62197400	-4.05853900	-2.46469700
H	-2.34116400	-5.03370600	1.50331000
H	-2.96783900	-5.73326100	-0.77668200
C	-1.46840200	2.54720700	0.75591800
C	-1.47134900	3.37456300	-0.37534900
C	-0.65408400	2.77327400	1.87408400
C	-0.62786200	4.48672900	-0.35366500
C	0.16883200	3.90051600	1.84580300
C	0.17782000	4.75232700	0.74744900
H	-0.60202800	5.15248500	-1.21148500
H	0.81155500	4.11240000	2.69558600
H	0.81900600	5.62954100	0.74877600
C	-2.21987600	-2.50898400	2.52817600
H	-2.46865500	-1.44836900	2.64943900
C	-0.70279600	-2.64300900	2.69085100
H	-0.38749000	-3.68638600	2.57317500
H	-0.17678500	-2.04062600	1.94068400
H	-0.39077400	-2.30400300	3.68523700
C	-2.96487200	-3.27850600	3.61961800
H	-2.69435800	-2.88940000	4.60700700
H	-4.04964600	-3.18859500	3.50211700
H	-2.71379800	-4.34466300	3.60795800
C	-0.62078200	1.83133200	3.06026400
H	-1.37792800	1.05468800	2.90362700
C	-0.96451300	2.55125100	4.36537800
H	-0.98679700	1.83921300	5.19736400
H	-0.22299000	3.32026400	4.60825600
H	-1.94339200	3.03829500	4.30614000
C	0.73796400	1.13062700	3.14950500
H	0.95558600	0.58431000	2.22415400
H	1.54576900	1.85286800	3.31389700
H	0.74674000	0.41794200	3.98183200
C	-2.30891500	3.06969800	-1.60014700
H	-2.96806500	2.22667500	-1.36364500
C	-3.75195200	-1.34990000	-2.20271100
H	-3.82565900	-0.37085200	-1.71545200
C	-2.65495800	-1.24977900	-3.26622400
H	-1.69042100	-0.99200600	-2.81350500
H	-2.53576400	-2.20017100	-3.79897700
H	-2.90531600	-0.47880000	-4.00347300
C	-5.11310400	-1.64903700	-2.83312400
H	-5.38457200	-0.85687100	-3.53902500
H	-5.10278800	-2.59368200	-3.38766500
H	-5.89947300	-1.71510000	-2.07404500
C	-1.41013500	2.63560000	-2.76163200
H	-0.72604600	3.44083900	-3.05270700
H	-0.80565100	1.76353600	-2.48700100
H	-2.01537300	2.37344100	-3.63656500
C	-3.19886000	4.24973500	-1.99335100
H	-2.60601200	5.12016100	-2.29493400
H	-3.83543500	3.97634900	-2.84154800
H	-3.84607300	4.55632500	-1.16500000
C	2.50094700	-1.88590500	-1.09755000
C	3.76904400	-1.77062400	-1.97792200
H	3.53663800	-1.56619900	-3.02681400
C	4.63958900	-0.73377700	-1.39524400
H	3.67199600	0.37319600	-1.66111500
C	2.61435700	0.52210200	-1.18592900
Br	2.44735300	2.29744200	-0.67609400
H	-5.01139900	-0.38195900	1.23842200
H	-4.12033400	2.24084900	1.58718700

H	5.31298000	-0.18778000	-2.05955700
C	5.05232100	-0.85008900	0.03170900
C	2.95064800	-2.30538200	0.31529200
C	3.88357900	-1.27000500	0.94292800
H	4.27541600	-1.65708100	1.88718200
H	3.30582000	-0.37362000	1.19536400
H	5.44838400	0.12198800	0.35597700
H	4.29795500	-2.73342100	-1.92362900
H	3.44193300	-3.28461900	0.24404200
H	2.07400900	-2.43978100	0.95710000
H	1.82487700	-2.63813800	-1.51347600
C	6.25776000	-1.85596700	0.10904500
H	5.83566300	-2.86685200	0.01661100
C	6.94792300	-1.73334300	1.46483600
H	6.26777700	-1.88299900	2.30769200
H	7.74074000	-2.48418100	1.54617300
H	7.41231800	-0.74607800	1.57384100
C	7.28522900	-1.65544200	-1.00489500
H	7.65668300	-0.62296400	-1.01891300
H	8.14747000	-2.30844900	-0.83898300
H	6.89190300	-1.89130600	-2.00029100

Cartesian coordinates of the optimized geometry for TS-**9** at PBE0-D3BJ/6-31G*,def2-TZVP level of theory: (number of imaginary frequencies = 1):

Au	-0.21401400	-0.23610400	-0.40033300
C	1.71290800	-0.46604300	-1.03388500
C	-2.06068900	0.13872300	0.33034100
C	-3.77953600	1.27363300	1.24332300
C	-4.18232800	-0.01116800	1.06891700
N	-2.47897200	1.34161400	0.78461600
N	-3.11373500	-0.68615000	0.51119300
C	-3.09642600	-2.07719500	0.16303600
C	-3.46578200	-2.43937700	-1.13891600
C	-2.67993300	-2.99797800	1.13415400
C	-3.40631300	-3.79624400	-1.46170000
C	-2.63772700	-4.34149300	0.75889800
C	-2.99727200	-4.73654200	-0.52422200
H	-3.68218200	-4.11919000	-2.46141300
H	-2.31817500	-5.08790200	1.48033400
H	-2.95933800	-5.78771100	-0.79549800
C	-1.65460900	2.51633700	0.78357800
C	-1.69494900	3.35524300	-0.33849500
C	-0.82923800	2.75088300	1.89194800
C	-0.87949400	4.48818200	-0.31690800
C	-0.03555400	3.89891100	1.86406200
C	-0.06461300	4.76251100	0.77529400
H	-0.88339900	5.16350600	-1.16759500
H	0.61392000	4.11811000	2.70684100
H	0.55368900	5.65602800	0.77723000
C	-2.24966400	-2.56840800	2.52249900
H	-2.52192900	-1.51481700	2.65421600
C	-0.72781300	-2.66742000	2.66441100
H	-0.38931000	-3.70214700	2.53568000
H	-0.22581300	-2.04813400	1.91154100
H	-0.41091900	-2.32748000	3.65694300
C	-2.96180300	-3.36250800	3.61829500
H	-2.68710600	-2.97401200	4.60477300
H	-4.04985800	-3.29711300	3.51586800
H	-2.68623000	-4.42241300	3.59552900
C	-0.75498200	1.79830900	3.06776500
H	-1.48988900	1.00071600	2.91074100
C	-1.10680100	2.49494700	4.38337100
H	-1.09848300	1.77479600	5.20859500
H	-0.38664200	3.28414100	4.62584400

H	-2.10070600	2.95222300	4.33893000
C	0.62498700	1.13790300	3.13672100
H	0.84927400	0.60626300	2.20438200
H	1.41267300	1.88228300	3.30004400
H	0.66351700	0.41830800	3.96219300
C	-2.54125600	3.04113800	-1.55493500
H	-3.17856500	2.18197300	-1.31677600
C	-3.87944800	-1.41663100	-2.17748900
H	-3.97347100	-0.44368600	-1.68154900
C	-2.79907100	-1.27867500	-3.25373200
H	-1.83616600	-0.99829900	-2.81110600
H	-2.66088800	-2.22148300	-3.79526700
H	-3.07946900	-0.50930100	-3.98179600
C	-5.23981900	-1.74788300	-2.79330900
H	-5.54163700	-0.95792900	-3.48929300
H	-5.21082300	-2.68748400	-3.35568500
H	-6.01444700	-1.84135300	-2.02509200
C	-1.64730400	2.63543800	-2.73040800
H	-0.98489500	3.45787000	-3.02361700
H	-1.02039800	1.77501100	-2.46959700
H	-2.25708400	2.36610000	-3.59996800
C	-3.46194100	4.20374700	-1.92862500
H	-2.89257600	5.08900700	-2.23223300
H	-4.10335000	3.92169700	-2.77029700
H	-4.10489900	4.49061700	-1.08996900
C	2.42344100	-1.78794800	-1.14101700
C	3.66928500	-1.61817300	-2.03826700
H	3.41157500	-1.41838100	-3.08248000
C	4.50373500	-0.54051100	-1.47055300
H	3.53090500	0.48484600	-1.67138200
C	2.43738100	0.62407600	-1.21434000
Br	2.25825000	2.39541100	-0.71641000
H	-5.11661200	-0.50537000	1.28474900
H	-4.28830900	2.13646600	1.64378000
H	5.15959500	0.00582400	-2.15220000
C	4.92793000	-0.58093400	-0.03814600
C	2.91238200	-2.20081900	0.25854000
C	3.82355200	-1.14133500	0.88400300
H	4.27282500	-1.54575000	1.79531500
H	3.20071600	-0.29879200	1.20632400
H	4.23734200	-2.55736700	-1.99844900
H	3.42919000	-3.16339700	0.16637600
H	2.05273300	-2.36523400	0.91668100
H	1.76833300	-2.56109900	-1.55175100
H	5.11844900	0.46381300	0.24825600
C	6.34530900	-1.26676700	0.12830000
C	6.27617000	-2.79251800	0.06699000
H	5.63322900	-3.20709100	0.84979000
H	5.92505400	-3.16109900	-0.90303200
H	7.27921400	-3.20660800	0.21732600
C	6.89281300	-0.84610600	1.49700500
H	7.87519500	-1.30297800	1.65785200
H	7.01530400	0.24133700	1.55759700
H	6.24731100	-1.16235400	2.32234300
C	7.31482900	-0.76118800	-0.94536800
H	7.33105800	0.33485700	-0.99391200
H	8.33186600	-1.09165200	-0.71062700
H	7.07577200	-1.15030400	-1.94229200

Cartesian coordinates of the optimized geometry for TS-**10** at PBE0-D3BJ/6-31G*,def2-TZVP level of theory: (number of imaginary frequencies = 1):

Au	0.05285200	-0.43376800	-0.39223800
C	1.67218600	-1.51026900	-1.00671100
C	-1.43768900	0.73719500	0.31267900
C	-2.48124400	2.53760200	1.17584100
C	-3.40898000	1.55735900	1.02724900
N	-1.28344000	2.01320400	0.73252000

N	-2.74671200	0.46586800	0.49980300
C	-3.34329200	-0.79980800	0.18550700
C	-3.82872400	-0.99993300	-1.11333300
C	-3.37969100	-1.78131300	1.18536300
C	-4.37152100	-2.25323300	-1.40229700
C	-3.93218800	-3.01640800	0.84343800
C	-4.42295700	-3.24995900	-0.43576800
H	-4.75724500	-2.45042000	-2.39837400
H	-3.97787700	-3.80577200	1.58811400
H	-4.85103700	-4.21774800	-0.68095400
C	-0.02610000	2.70496500	0.71179500
C	0.30900000	3.43939300	-0.43402200
C	0.81604200	2.58819000	1.82613200
C	1.54083200	4.09631500	-0.43214600
C	2.03483400	3.26715500	1.77800500
C	2.39174000	4.01848900	0.66431400
H	1.83655100	4.67633000	-1.30152900
H	2.71292900	3.20519800	2.62444100
H	3.34097200	4.54706400	0.65016800
C	-2.81027100	-1.54522800	2.56998600
H	-2.58961000	-0.47634000	2.67148100
C	-1.48935900	-2.30164600	2.73976200
H	-1.64244500	-3.38290200	2.64298300
H	-0.76275300	-1.99013700	1.97994600
H	-1.05835300	-2.10713700	3.72841600
C	-3.80489800	-1.91098300	3.67244900
H	-3.39124600	-1.65536100	4.65379800
H	-4.75145900	-1.37463300	3.55012700
H	-4.02623500	-2.98373600	3.67975300
C	0.46083000	1.73865100	3.02916700
H	-0.55351000	1.34896600	2.88716600
C	0.46182100	2.55699600	4.32145600
H	0.14971000	1.93334200	5.16602300
H	1.45956800	2.94747300	4.54995800
H	-0.22276600	3.40889200	4.25440800
C	1.40103600	0.53404200	3.12951800
H	1.36203500	-0.06677200	2.21323800
H	2.43858400	0.85316400	3.28086900
H	1.11665300	-0.10397600	3.97375700
C	-0.58841500	3.49364900	-1.65319500
H	-1.54037400	3.01333300	-1.39983900
C	-3.74768200	0.07029500	-2.18279100
H	-3.39872900	0.99749000	-1.71381800
C	-2.72163500	-0.31560600	-3.25174200
H	-1.73308200	-0.47952800	-2.80718300
H	-3.01787000	-1.23623900	-3.76743000
H	-2.63333100	0.47783300	-4.00223400
C	-5.11574500	0.35954400	-2.80289200
H	-5.03778600	1.18041700	-3.52373200
H	-5.50888300	-0.51158100	-3.33819900
H	-5.84869400	0.64242800	-2.04020900
C	0.03372400	2.69767400	-2.80418400
H	0.99206900	3.13215100	-3.11061100
H	0.21455400	1.65718700	-2.51068300
H	-0.63193300	2.69927000	-3.67448300
C	-0.89916800	4.93123500	-2.07249100
H	0.00314000	5.46272400	-2.39401700
H	-1.59987100	4.93570500	-2.91413500
H	-1.34779400	5.49961100	-1.25110700
C	1.69291800	-3.00849000	-1.11621300
C	2.91069700	-3.43439900	-1.96947900
H	2.81354500	-3.14472300	-3.01975900
C	4.12014500	-2.85807300	-1.35278000
H	3.72114100	-1.44162600	-1.66973000
C	2.82521500	-0.87451400	-1.18049800
Br	3.39417400	0.82311100	-0.68624400

H	-4.46562400	1.53140400	1.24300300
H	-2.55810300	3.54690600	1.54881900
H	4.99380000	-2.66797600	-1.98404000
C	4.39113800	-3.11896300	0.08937000
C	1.87557100	-3.59941600	0.29558300
C	3.13256900	-3.05435300	0.97001600
H	3.32779100	-3.59360600	1.90264600
H	2.97142100	-2.00342300	1.24363700
H	2.98823600	-4.53014400	-1.92432600
H	1.92289000	-4.69299800	0.20904400
H	0.99996800	-3.36628400	0.90965600
H	0.77073700	-3.39042400	-1.56308600
C	5.53987600	-2.28410400	0.64549100
H	5.27222600	-1.22223400	0.65708500
H	5.76314800	-2.58535500	1.67286800
H	6.45295200	-2.40428600	0.05306600
H	4.71795800	-4.17855300	0.05858900

Cartesian coordinates of the optimized geometry for TS-**11** at PBE0-D3BJ/6-31G*,def2-TZVP level of theory: (number of imaginary frequencies = 1):

Au	-0.16679800	-0.35169700	-0.45403400
C	1.58008000	-1.12007500	-1.17644600
C	-1.78751600	0.52818700	0.37461900
C	-3.05489800	2.09084900	1.38861700
C	-3.82229300	0.98284400	1.22476800
N	-1.81475500	1.78948900	0.86151500
N	-3.02491900	0.04053800	0.60461600
C	-3.42760900	-1.28885900	0.24821800
C	-3.94524900	-1.50358300	-1.03573900
C	-3.25067500	-2.30945600	1.19240600
C	-4.29412000	-2.81363300	-1.36878300
C	-3.61511500	-3.60063000	0.80807800
C	-4.13116600	-3.85018800	-0.45808400
H	-4.69694700	-3.02381400	-2.35537900
H	-3.49235400	-4.42138100	1.50884900
H	-4.41041300	-4.86223800	-0.73693200
C	-0.68606700	2.67532300	0.82746800
C	-0.53664900	3.51832800	-0.28228900
C	0.22451600	2.63463400	1.89230600
C	0.57138700	4.36756900	-0.29313300
C	1.31273300	3.50708100	1.83425900
C	1.48188100	4.36939100	0.75726300
H	0.72179000	5.03617600	-1.13576100
H	2.03743800	3.50932300	2.64355700
H	2.33080400	5.04725100	0.73531300
C	-2.65334900	-2.04928000	2.56094700
H	-2.59831600	-0.96466700	2.70966800
C	-1.22304000	-2.59222200	2.63223500
H	-1.20855900	-3.67815300	2.48252500
H	-0.59386100	-2.13270300	1.86073200
H	-0.77859500	-2.37876000	3.61102000
C	-3.51982400	-2.61897700	3.68490900
H	-3.10091200	-2.34656300	4.65950600
H	-4.54373700	-2.23482200	3.63338600
H	-3.56989900	-3.71242500	3.64394500
C	0.07962800	1.66990300	3.05135800
H	-0.85940500	1.11939400	2.92473400
C	0.00636300	2.40200100	4.39235800
H	-0.15089600	1.68731300	5.20733600
H	0.93284300	2.94689800	4.60439500
H	-0.81653000	3.12415100	4.40771100
C	1.21474300	0.64194900	3.03645600
H	1.23330000	0.09825500	2.08459700
H	2.18928500	1.12583100	3.16880400

H	1.08423100	-0.08379200	3.84700600
C	-1.49590200	3.49184400	-1.45436200
H	-2.34520700	2.85259700	-1.18768200
C	-4.09569800	-0.38441100	-2.04595100
H	-3.88244800	0.56430200	-1.54010700
C	-3.07384300	-0.54014700	-3.17555800
H	-2.05094000	-0.55546900	-2.78167200
H	-3.23767800	-1.47228400	-3.72830000
H	-3.15645800	0.29137500	-3.88433000
C	-5.52154600	-0.29894900	-2.59310300
H	-5.61622300	0.55853700	-3.26783600
H	-5.79051700	-1.19561100	-3.16197000
H	-6.25301600	-0.18219500	-1.78672600
C	-0.81886400	2.86648400	-2.67750200
H	0.04154900	3.46399900	-2.99973200
H	-0.46134900	1.85447600	-2.45534200
H	-1.52211300	2.80693500	-3.51560400
C	-2.05142800	4.88066500	-1.77288000
H	-1.26316100	5.56564600	-2.10373200
H	-2.78913900	4.81583500	-2.57968000
H	-2.53825700	5.32710100	-0.89955600
C	1.84809100	-2.58914500	-1.34888300
C	3.08840300	-2.77261000	-2.25008500
H	2.90853700	-2.47041800	-3.28591100
C	4.20614300	-2.01674500	-1.65210200
H	3.57680800	-0.69699500	-1.87033700
C	2.59887700	-0.29108600	-1.35558300
Br	2.89933400	1.46400600	-0.83284600
H	-4.84828100	0.77608100	1.48613600
H	-3.27050000	3.05443700	1.82305100
H	5.00326000	-1.67695500	-2.31716000
C	4.58126900	-2.26166100	-0.22917600
C	2.18517600	-3.19342600	0.02554900
C	3.36050200	-2.47473900	0.68536300
H	3.67202200	-3.03040900	1.57382500
H	3.02963700	-1.48700100	1.03310100
H	3.35426700	-3.83934700	-2.24793100
H	2.40975900	-4.25926200	-0.11264100
H	1.31134800	-3.13600900	0.68242900
H	0.98711400	-3.10408500	-1.78426600
C	5.58930400	-1.23563300	0.33590900
H	5.06970200	-0.26715000	0.37557100
H	5.11735500	-3.23114700	-0.30724300
C	6.00314400	-1.60967600	1.75805000
H	6.46574700	-2.60448400	1.78426800
H	6.73969200	-0.89375500	2.13554000
H	5.15938100	-1.60719900	2.45367900
C	6.83066900	-1.08353700	-0.54183700
H	7.54807000	-0.41153400	-0.06102300
H	7.33392800	-2.04746700	-0.68928300
H	6.61757500	-0.65443500	-1.52698100

Cartesian coordinates of the optimized geometry for TS-**12** at PBE0-D3BJ/6-31G*,def2-TZVP level of theory: (number of imaginary frequencies = 1):

Au	-0.25292700	-0.30297600	-0.44326800
C	1.58216300	-0.87245000	-1.13387700
C	-1.97237400	0.38249800	0.36878500
C	-3.42309600	1.78668300	1.36905500
C	-4.04880700	0.59171100	1.21490900
N	-2.15473900	1.63491000	0.84464600
N	-3.14104600	-0.25133900	0.60334200
C	-3.37786600	-1.62288400	0.25863600
C	-3.85629900	-1.91101700	-1.02610800
C	-3.08682600	-2.60572700	1.21462200

C	-4.04360200	-3.25667000	-1.34724000
C	-3.29101300	-3.93498000	0.84188800
C	-3.76444100	-4.25715100	-0.42459800
H	-4.41136500	-3.52315500	-2.33394800
H	-3.07578700	-4.72813900	1.55201900
H	-3.91874800	-5.29809200	-0.69413200
C	-1.14386800	2.65262300	0.80053800
C	-1.10184200	3.49777500	-0.31678200
C	-0.23286700	2.73388500	1.86259500
C	-0.10787600	4.47774900	-0.33799500
C	0.73769000	3.73492800	1.79474500
C	0.79667700	4.60213500	0.71014300
H	-0.04221300	5.15185700	-1.18707300
H	1.45830200	3.83359900	2.60168200
H	1.55531300	5.37939000	0.67982000
C	-2.53235800	-2.26181700	2.58273400
H	-2.62189000	-1.17874800	2.72572400
C	-1.04258900	-2.60987300	2.65749700
H	-0.88489700	-3.68537000	2.51428500
H	-0.47925800	-2.07557100	1.88318300
H	-0.63122500	-2.33368000	3.63507400
C	-3.31656200	-2.93517600	3.70964700
H	-2.93996800	-2.60184600	4.68257400
H	-4.38276100	-2.69271300	3.65441700
H	-3.21879200	-4.02569800	3.67711200
C	-0.25279300	1.76710700	3.02882500
H	-1.10863800	1.09440300	2.90280300
C	-0.43103100	2.49391100	4.36289000
H	-0.49234300	1.77185500	5.18426100
H	0.41138700	3.16246400	4.57183900
H	-1.34497000	3.09693000	4.36914100
C	1.01006200	0.90070400	3.02810200
H	1.10908100	0.35853000	2.08041300
H	1.90998400	1.51153600	3.16374100
H	0.97174500	0.16940300	3.84330000
C	-2.05111600	3.34027300	-1.48669100
H	-2.81423800	2.60320300	-1.21185400
C	-4.13197700	-0.82846900	-2.04964300
H	-4.02887800	0.14433200	-1.55532100
C	-3.09731600	-0.88016800	-3.17709400
H	-2.07998100	-0.77695100	-2.78204000
H	-3.15496300	-1.83010800	-3.72077100
H	-3.27122500	-0.07009400	-3.89410700
C	-5.55760800	-0.91327200	-2.59746700
H	-5.74843400	-0.08175200	-3.28427500
H	-5.72215300	-1.84285100	-3.15318200
H	-6.29854700	-0.86898400	-1.79248200
C	-1.30154400	2.79122700	-2.70417700
H	-0.52412000	3.48923400	-3.03507700
H	-0.81801900	1.83566500	-2.47093100
H	-1.99230600	2.63331600	-3.53995400
C	-2.77536200	4.64565400	-1.81863700
H	-2.07848000	5.42022100	-2.15688300
H	-3.49892600	4.48133100	-2.62414800
H	-3.31441700	5.03660700	-0.94937700
C	1.97737600	-2.30400300	-1.34810000
C	3.20385700	-2.35343700	-2.29734800
H	2.97148700	-1.97554600	-3.29650800
C	4.31861100	-1.62048400	-1.67817600
H	3.55145300	-0.26876700	-1.72578800
C	2.52530000	0.04830800	-1.27361300
Br	2.58256700	1.84039200	-0.79057400
H	-5.04205100	0.26302800	1.47785800
H	-3.75563200	2.72040100	1.79483700
H	5.04348400	-1.12965300	-2.33077100
C	4.75488900	-2.00433300	-0.30935400

C	2.38615700	-2.93370200	-0.00638400
C	3.55660400	-2.19818800	0.64169600
H	3.88767000	-2.75467000	1.52176200
H	3.22503800	-1.21214700	0.98947500
H	3.50161000	-3.40795900	-2.38933000
H	2.64780700	-3.98605300	-0.18038300
H	1.53034700	-2.92921800	0.67645300
H	1.15754800	-2.87573300	-1.79249000
C	5.95520300	-1.19770700	0.25816700
H	5.14729900	-3.02629500	-0.50215500
C	6.31113800	-1.73701900	1.64735300
H	6.47730200	-2.82116600	1.62950500
H	7.23470300	-1.26716500	2.00082500
H	5.53222600	-1.52022700	2.38502800
C	7.17375800	-1.38499000	-0.65268100
H	8.03648500	-0.85495000	-0.23630700
H	7.44750400	-2.44299000	-0.74455900
H	7.01901500	-0.98490400	-1.66193600
C	5.62834200	0.29247600	0.36694200
H	5.45486000	0.75167200	-0.61350600
H	4.74526700	0.47791300	0.98698400
H	6.46946400	0.82389600	0.82471100

Cartesian coordinates of the optimized geometry for TS-**13a** at PBE0-D3BJ/6-31G*,def2-TZVP level of theory: (number of imaginary frequencies = 1):

Au	0.14029200	-0.33750900	-0.30140600
C	2.02733700	-0.90953600	-0.80129300
C	-1.66919000	0.37543300	0.25708100
C	-3.24859400	1.83251600	0.93438400
C	-3.83388700	0.61177800	0.82981000
N	-1.92523900	1.66344800	0.57967500
N	-2.84839200	-0.26237100	0.41482900
C	-3.02737700	-1.66487300	0.17480300
C	-3.39036500	-2.07505800	-1.11491300
C	-2.80070100	-2.55304700	1.23458700
C	-3.52695500	-3.44743000	-1.32983500
C	-2.95113200	-3.91440600	0.96566800
C	-3.31065000	-4.35684500	-0.30162100
H	-3.80436000	-3.80804500	-2.31609700
H	-2.78343000	-4.63692300	1.75927200
H	-3.42440500	-5.42066600	-0.48961900
C	-0.93150900	2.69848400	0.55058600
C	-0.74660900	3.40890500	-0.64333800
C	-0.17761400	2.92896500	1.70941700
C	0.22981000	4.40664000	-0.64645000
C	0.78529300	3.93813600	1.65321300
C	0.98314500	4.67415000	0.49064800
H	0.40168600	4.98075000	-1.55239500
H	1.38743900	4.14947500	2.53233200
H	1.73067600	5.46247000	0.47047000
C	-2.37597600	-2.08070300	2.61039200
H	-2.43936900	-0.98668400	2.63170600
C	-0.91685200	-2.45838200	2.88096300
H	-0.78553400	-3.54658800	2.87719000
H	-0.25483800	-2.03322100	2.11726500
H	-0.59884000	-2.08287400	3.86016400
C	-3.30052200	-2.61238800	3.70673600
H	-3.00973400	-2.20098300	4.67926500
H	-4.34336900	-2.33786300	3.51733100
H	-3.25066500	-3.70399300	3.78332300
C	-0.35283600	2.10628900	2.96932400
H	-1.19907300	1.42621100	2.82010200
C	-0.67690900	2.98293200	4.18011300
H	-0.85270900	2.35966600	5.06342500

H	0.14746100	3.66477900	4.41601800
H	-1.57205600	3.58896400	4.00586400
C	0.88686400	1.24303900	3.21963000
H	1.08987900	0.59318000	2.36034600
H	1.77333100	1.86461900	3.38990800
H	0.74142800	0.61218900	4.10371400
C	-1.53123700	3.10030600	-1.90176600
H	-2.28761900	2.34564000	-1.65824600
C	-3.58930900	-1.09123200	-2.25015400
H	-3.58125600	-0.07851900	-1.83078600
C	-2.42926400	-1.18328500	-3.24560400
H	-1.46931400	-0.99825600	-2.74962200
H	-2.38612800	-2.17669600	-3.70668000
H	-2.55068900	-0.44444400	-4.04568300
C	-4.93691100	-1.28433800	-2.94704600
H	-5.07942400	-0.51397900	-3.71235400
H	-4.99888000	-2.25738400	-3.44627100
H	-5.76763400	-1.21875500	-2.23679100
C	-0.61243500	2.49812500	-2.96837100
H	0.16510900	3.21000800	-3.26776900
H	-0.11650800	1.59470900	-2.59529900
H	-1.18753400	2.23124900	-3.86192700
C	-2.26709300	4.33318400	-2.42917400
H	-1.56827900	5.11919800	-2.73575500
H	-2.86978100	4.06852700	-3.30450700
H	-2.93373700	4.75592500	-1.67025900
C	2.50383700	-2.32661000	-0.86979800
H	1.83083500	-2.95722500	-1.45992900
C	3.95293400	-2.41481300	-1.39329000
H	3.92921300	-2.39151400	-2.48910400
C	4.72043200	-1.22825500	-0.90704500
H	3.93402000	-0.23183400	-1.55237900
C	2.95190800	0.03477900	-0.98069500
Br	3.00843800	1.83621300	-0.53707000
H	-4.84617900	0.28414300	1.00763200
H	-3.64273200	2.79428300	1.22310900
H	5.52347900	-0.88334300	-1.57014000
H	2.45541100	-2.71816200	0.15731000
C	4.68265700	-3.70173100	-0.93486800
H	4.02697900	-4.55660000	-1.13566200
H	5.57398800	-3.83642200	-1.55974700
C	5.07043300	-1.14750700	0.54382800
C	5.09955600	-3.66140500	0.53305200
C	5.90850200	-2.40741900	0.84981800
H	6.83545600	-2.39560500	0.26098700
H	6.20304000	-2.38057600	1.90409300
H	5.62506300	-0.23131300	0.76473800
H	4.17120800	-1.16123000	1.16684400
H	5.68635600	-4.55715800	0.76374800
H	4.21636500	-3.69731300	1.18509600

Cartesian coordinates of the optimized geometry for TS-**13b** at PBE0-D3BJ/6-31G*,def2-TZVP level of theory: (number of imaginary frequencies = 1):

Au	-0.07826300	-0.42665400	-0.15085100
C	-1.94059400	-1.19155100	-0.43456100
C	1.70575100	0.48448400	0.13724900
C	3.20640300	2.12826200	0.48803800
C	3.88915200	0.95657600	0.41994400
N	1.87351000	1.81489200	0.31252700
N	2.95053000	-0.03343800	0.20447100
C	3.23455800	-1.43268200	0.07012200
C	3.21908000	-2.22586500	1.22510300
C	3.48543100	-1.93783600	-1.21259300
C	3.47032900	-3.58922500	1.06264300

C	3.73042300	-3.30781200	-1.31893900
C	3.72390300	-4.12429000	-0.19446900
H	3.46568700	-4.23993700	1.93235700
H	3.92722300	-3.74056800	-2.29555600
H	3.91907500	-5.18778000	-0.29883400
C	0.78930400	2.75514700	0.30896400
C	0.14891500	3.03669000	1.52343600
C	0.40736500	3.32348300	-0.91394900
C	-0.90801800	3.94809700	1.49008300
C	-0.65590700	4.22778200	-0.89207500
C	-1.30291000	4.54247400	0.29725900
H	-1.42812500	4.19431800	2.41140500
H	-0.98004100	4.69133900	-1.81947800
H	-2.12140000	5.25715800	0.29457900
C	3.45701100	-1.06008900	-2.44752600
H	3.38729700	-0.01527400	-2.12356900
C	2.21340800	-1.36123700	-3.28880900
H	2.22502300	-2.39612200	-3.64971400
H	1.29867100	-1.21667600	-2.70207100
H	2.17084600	-0.69941800	-4.16118400
C	4.73532200	-1.19571500	-3.27613800
H	4.71357300	-0.49887400	-4.12081900
H	5.62525000	-0.97853500	-2.67640000
H	4.84634500	-2.20525600	-3.68652600
C	1.07831200	2.96013900	-2.22263700
H	1.92638500	2.30205200	-2.00206900
C	1.62946000	4.19361100	-2.93992800
H	2.15758700	3.89678100	-3.85235100
H	0.82834500	4.88157800	-3.23162800
H	2.32928000	4.74600500	-2.30423900
C	0.11404000	2.17717400	-3.11789400
H	-0.24676500	1.27518400	-2.61015500
H	-0.75750800	2.78504200	-3.38623200
H	0.61321000	1.87479700	-4.04523800
C	0.54130800	2.36526400	2.82336400
H	1.43199500	1.75414200	2.63857000
C	2.90808500	-1.65622500	2.59441300
H	2.87135400	-0.56396200	2.51081100
C	1.52940800	-2.12454800	3.06836700
H	0.74990600	-1.83678400	2.35316300
H	1.50164100	-3.21457900	3.17972700
H	1.28612600	-1.67949100	4.03973000
C	3.99386300	-1.99861600	3.61576100
H	3.77523200	-1.51764000	4.57514800
H	4.05418600	-3.07749400	3.79552900
H	4.97942600	-1.65978700	3.28001500
C	-0.56977800	1.42229400	3.29333000
H	-1.49229600	1.97415200	3.50676000
H	-0.79586300	0.67048900	2.52842300
H	-0.26793600	0.90221200	4.20927400
C	0.90182100	3.38620700	3.90377500
H	0.04139600	4.00846200	4.17325700
H	1.23564700	2.87405100	4.81250200
H	1.70514100	4.05226500	3.57202800
C	-2.24848300	-2.60210000	-0.85373800
H	-1.66830600	-3.33051000	-0.27699800
H	-4.03323400	-0.88243500	-0.12137200
C	-2.99266700	-0.38655800	-0.29936200
Br	-3.19450100	1.45319100	-0.20223400
H	4.94128900	0.73359800	0.50346300
H	3.53753500	3.14318900	0.64286900
H	-1.90833100	-2.69610200	-1.89476900
C	-4.50131100	-1.67591600	-1.21106100
C	-5.94490500	-1.47583100	-0.85586900
C	-3.76303100	-2.90020700	-0.77157100
H	-4.20880900	-1.26883600	-2.17982000

C	-6.30056200	-1.98343900	0.53776400
H	-6.49657700	-2.04684100	-1.62244900
H	-6.22999900	-0.42808900	-1.00893000
C	-4.20681900	-3.37242500	0.61746900
H	-3.99700200	-3.67743200	-1.51532300
C	-5.72427700	-3.37580800	0.77885900
H	-7.38854500	-1.98150400	0.65651300
H	-5.90518100	-1.28588200	1.29074000
H	-3.79308900	-4.37068800	0.79530400
H	-3.76106000	-2.71333800	1.37609500
H	-5.98986100	-3.72308500	1.78256600
H	-6.17305700	-4.08833800	0.07237400

Cartesian coordinates of the optimized geometry for TS-**13c** at PBE0-D3BJ/6-31G*,def2-TZVP level of theory: (number of imaginary frequencies = 1):

Au	0.12616000	-0.35668400	-0.09675100
C	2.09077000	-0.85737200	-0.23688300
C	-1.78461600	0.29094600	0.07223500
C	-3.52669600	1.70461800	0.28250200
C	-4.02456200	0.44184300	0.24809100
N	-2.15557700	1.58747100	0.17378700
N	-2.94078400	-0.40446700	0.11939500
C	-3.00750100	-1.83489300	0.04273600
C	-3.12689000	-2.42566100	-1.22227800
C	-2.92054900	-2.56730000	1.23404400
C	-3.15839900	-3.82020100	-1.27147000
C	-2.95697100	-3.95864000	1.12836500
C	-3.07511800	-4.57829200	-0.10977700
H	-3.24727500	-4.31833300	-2.23255700
H	-2.89066700	-4.56398500	2.02786900
H	-3.10360900	-5.66253300	-0.16998800
C	-1.22373800	2.67884400	0.17012100
C	-0.86540400	3.24330800	-1.06163600
C	-0.69809400	3.10732300	1.39663500
C	0.04877600	4.29801900	-1.03726400
C	0.21226500	4.16518300	1.36524800
C	0.57755900	4.75906700	0.16265500
H	0.35006300	4.76312300	-1.97149700
H	0.63990700	4.52766600	2.29571300
H	1.28006900	5.58800800	0.16052500
C	-2.75846600	-1.90020900	2.58495300
H	-2.87663800	-0.81899400	2.44947100
C	-1.35019300	-2.13990100	3.13619400
H	-1.16891500	-3.20840400	3.29997600
H	-0.58887600	-1.76885000	2.43995700
H	-1.22061800	-1.62439600	4.09448300
C	-3.82945400	-2.35502300	3.57773900
H	-3.72760500	-1.80764200	4.52093500
H	-4.83695000	-2.17859900	3.18702000
H	-3.74145800	-3.42275700	3.80619200
C	-1.05528700	2.44170300	2.70968900
H	-1.83751300	1.69899700	2.51608300
C	-1.61472500	3.44306900	3.72151300
H	-1.91747600	2.92608900	4.63838200
H	-0.86853300	4.19578300	3.99864000
H	-2.48771800	3.96941600	3.32173700
C	0.15501200	1.69288200	3.27449400
H	0.52623500	0.95168200	2.55720800
H	0.97558200	2.38304300	3.50150000
H	-0.11494400	1.17273900	4.20037200
C	-1.40329800	2.72571500	-2.37968200
H	-2.15015100	1.95280500	-2.16570100
C	-3.17866700	-1.60573900	-2.49558700
H	-3.29043200	-0.55096500	-2.21908200

C	-1.86417800	-1.73857200	-3.26988900
H	-1.01211900	-1.42666000	-2.65469700
H	-1.69564500	-2.77594300	-3.58106200
H	-1.88457600	-1.11464400	-4.17050700
C	-4.37830200	-1.97672800	-3.36865700
H	-4.42681800	-1.31813800	-4.24238200
H	-4.30791300	-3.00573300	-3.73760400
H	-5.31916800	-1.88252600	-2.81661100
C	-0.28465100	2.06710200	-3.19158200
H	0.49449500	2.79244100	-3.45213400
H	0.18627300	1.25561400	-2.62501800
H	-0.68302600	1.65074200	-4.12346900
C	-2.10001100	3.82760800	-3.17981800
H	-1.40036900	4.61991100	-3.46789300
H	-2.52757300	3.41489900	-4.09982200
H	-2.90893400	4.28919300	-2.60392400
C	2.63897600	-2.25385800	-0.10687800
H	2.07151700	-2.97021000	-0.70906000
C	4.13297200	-2.26912200	-0.43382200
H	4.26898300	-2.25802000	-1.52565700
C	4.74432200	-1.02427000	0.11442100
H	4.06081900	-0.13527800	-0.77344100
C	2.99569900	0.11806800	-0.37398300
Br	2.88699000	1.95788600	-0.13657900
H	-5.03484600	0.06744900	0.30143700
H	-4.01115300	2.66424300	0.37258900
H	2.48752000	-2.54941900	0.94319400
C	4.94922400	-3.44557800	0.13009700
H	4.55977800	-4.38326700	-0.28127900
C	6.42823900	-3.26303600	-0.21665300
C	6.14453000	-0.72561100	-0.31044100
C	6.96708500	-1.91435000	0.25661900
H	6.50878600	0.22368800	0.09186700
H	6.23536800	-0.70928300	-1.40287000
H	8.00904500	-1.77845700	-0.05231000
H	6.95344200	-1.86826700	1.35331100
H	7.01717900	-4.06971700	0.23295300
H	6.55981100	-3.34843100	-1.30367500
H	4.54541400	-0.83092300	1.17027500
H	4.81649900	-3.49175100	1.21952500

Cartesian coordinates of the optimized geometry for TS-**14a** at PBE0-D3BJ/6-31G*,def2-TZVP level of theory: (number of imaginary frequencies = 1):

Au	0.10587400	-0.29110700	-0.21325300
C	2.06042400	-0.70697100	-0.58629500
C	-1.79136000	0.27517300	0.20615600
C	-3.53569400	1.61042700	0.70757500
C	-3.99967600	0.33603100	0.64205700
N	-2.18323200	1.54854900	0.43791100
N	-2.91491900	-0.46184700	0.33491500
C	-2.95120700	-1.88534300	0.16543200
C	-3.18951800	-2.39358000	-1.11843800
C	-2.71808800	-2.69286700	1.28654500
C	-3.19081300	-3.78203200	-1.26166200
C	-2.73061600	-4.07440700	1.08810100
C	-2.96518000	-4.61320600	-0.17117300
H	-3.36935900	-4.21715600	-2.24073300
H	-2.55307400	-4.73630200	1.93089500
H	-2.97292700	-5.69132500	-0.30387300
C	-1.28848100	2.66998400	0.40011900
C	-1.09881100	3.32749700	-0.82306200
C	-0.62850600	3.03439400	1.58160000
C	-0.21822800	4.41063500	-0.83441900
C	0.24095200	4.12461900	1.51581500

C	0.43969700	4.81011200	0.32295800
H	-0.04592700	4.94717800	-1.76303000
H	0.76895100	4.43983900	2.41131400
H	1.11307600	5.66242200	0.29490000
C	-2.42464000	-2.11226700	2.65513500
H	-2.59241700	-1.03001400	2.61118100
C	-0.95495000	-2.33160300	3.02520800
H	-0.72233200	-3.40072000	3.09124200
H	-0.29200500	-1.88509900	2.27462700
H	-0.73181500	-1.87620100	3.99670000
C	-3.35844100	-2.67514400	3.72766300
H	-3.16705300	-2.18738800	4.68946900
H	-4.40945300	-2.51472500	3.46610300
H	-3.20944500	-3.75107100	3.86954400
C	-0.80144800	2.26895900	2.87732100
H	-1.57907900	1.51151800	2.72712100
C	-1.26096000	3.17752800	4.01867800
H	-1.43216200	2.58884700	4.92627900
H	-0.50930600	3.93834400	4.25603600
H	-2.19222100	3.69527400	3.76646400
C	0.49130100	1.53066700	3.23601000
H	0.79338200	0.85667500	2.42577000
H	1.31204000	2.23519700	3.41270600
H	0.35293700	0.93708400	4.14664500
C	-1.77776600	2.87812500	-2.10047100
H	-2.47665700	2.07137900	-1.85203800
C	-3.39890500	-1.49316500	-2.31905900
H	-3.49558600	-0.46175400	-1.96092800
C	-2.17959500	-1.54329400	-3.24415600
H	-1.26778200	-1.25256100	-2.70949400
H	-2.03086600	-2.55322600	-3.64322700
H	-2.31308400	-0.86162300	-4.09165200
C	-4.68441000	-1.83529200	-3.07382000
H	-4.84238300	-1.12433600	-3.89179400
H	-4.64064000	-2.83735200	-3.51414700
H	-5.55752100	-1.79732800	-2.41418300
C	-0.74878200	2.30531700	-3.07907200
H	-0.02004600	3.06747400	-3.37761000
H	-0.19880200	1.47160000	-2.62771300
H	-1.24512700	1.94008000	-3.98501400
C	-2.58888500	4.00653800	-2.73965100
H	-1.94637400	4.83563300	-3.05577300
H	-3.11362200	3.63920800	-3.62802900
H	-3.33357800	4.40722900	-2.04399400
C	2.66626700	-2.07619500	-0.56379700
H	2.07709600	-2.79358400	-1.14571600
C	4.14343200	-2.06266800	-1.03431900
C	4.76248900	-0.79432500	-0.53013700
H	3.93460400	0.10316400	-1.28168900
C	2.90857200	0.30639000	-0.76696600
Br	2.78265900	2.12485500	-0.41522000
H	-4.98681700	-0.07592000	0.78187600
H	-4.03279900	2.54462400	0.91701400
H	5.57854900	-0.40032400	-1.14993700
H	2.60810600	-2.41809000	0.48006800
C	4.93986800	-3.24767200	-0.41994500
H	4.37557300	-4.16581800	-0.62316500
H	5.88692700	-3.33668200	-0.96798500
C	4.99080300	-0.61323100	0.93652900
C	5.23700500	-3.10345900	1.06891900
C	5.91124000	-1.77057500	1.37368300
H	6.87562000	-1.70508600	0.85239000
H	6.12078400	-1.66455300	2.44317900
H	5.44109800	0.36173000	1.14341600
H	4.05174800	-0.67966700	1.49346900
H	5.87730700	-3.93356300	1.38684400

H	4.31381100	-3.18663400	1.65819500
C	4.20532600	-2.13356800	-2.56124600
H	5.23499100	-2.03833900	-2.92325100
H	3.81915000	-3.09769800	-2.90664900
H	3.59991900	-1.34887600	-3.02882100

Cartesian coordinates of the optimized geometry for TS-**14b** at PBE0-D3BJ/6-31G*,def2-TZVP level of theory: (number of imaginary frequencies = 1):

Au	-0.03766200	-0.35383500	-0.08865400
C	-1.97905200	-0.92861800	-0.27302600
C	1.84168600	0.37546900	0.08823500
C	3.51819400	1.86818400	0.28523300
C	4.07206300	0.62864500	0.26776000
N	2.15399700	1.68842200	0.17418000
N	3.02759800	-0.26692100	0.14626600
C	3.15823800	-1.69399400	0.09234300
C	3.10948900	-2.40937800	1.29621100
C	3.29850300	-2.30002100	-1.16317100
C	3.20735900	-3.79927100	1.21338100
C	3.39216600	-3.69240300	-1.18944900
C	3.34769000	-4.43377300	-0.01492700
H	3.17162800	-4.39184000	2.12301100
H	3.49965800	-4.20213900	-2.14256100
H	3.42424500	-5.51653100	-0.05720900
C	1.17433700	2.73676300	0.15143200
C	0.61709500	3.15226400	1.36849000
C	0.80425900	3.27374600	-1.08906400
C	-0.33702100	4.17003200	1.31796000
C	-0.15436900	4.28855600	-1.08389900
C	-0.71436600	4.73747700	0.10649300
H	-0.78993000	4.52162700	2.24060900
H	-0.46519300	4.73222700	-2.02547900
H	-1.45111000	5.53599700	0.08996100
C	3.31033900	-1.50025700	-2.45021700
H	3.36796900	-0.43666700	-2.19156000
C	2.00566700	-1.71233300	-3.22341100
H	1.89001100	-2.76199400	-3.51712100
H	1.13869000	-1.43302600	-2.61324500
H	1.99606900	-1.10323000	-4.13433800
C	4.52878100	-1.82524400	-3.31557600
H	4.54502300	-1.18168200	-4.20164500
H	5.46253300	-1.67231200	-2.76464500
H	4.51211000	-2.86345400	-3.66455000
C	1.37548200	2.76519800	-2.39657500
H	2.15375700	2.02852500	-2.16734800
C	2.02960200	3.88662400	-3.20538600
H	2.48196100	3.48201800	-4.11710600
H	1.29858500	4.64435400	-3.50820500
H	2.81316600	4.38981200	-2.62932900
C	0.29326800	2.04794100	-3.20828200
H	-0.14639000	1.22456700	-2.63341800
H	-0.51423400	2.73557000	-3.48442300
H	0.71697600	1.63691300	-4.13138000
C	0.98903900	2.51529400	2.69156700
H	1.79411000	1.79410500	2.51076900
C	2.92137600	-1.72686100	2.63600000
H	3.00624200	-0.64470000	2.48380300
C	1.51873400	-2.00248700	3.18503500
H	0.74837800	-1.66699700	2.48087500
H	1.37092300	-3.07364100	3.36434000
H	1.36860400	-1.47707300	4.13489400
C	4.00143300	-2.13131200	3.64056600
H	3.87898100	-1.56997700	4.57301500
H	3.94436400	-3.19664800	3.88904100

H	5.00493200	-1.93192100	3.25057900
C	-0.19967500	1.73811700	3.26381300
H	-1.04159200	2.40572900	3.47967700
H	-0.54669500	0.97504700	2.55762800
H	0.08358000	1.23991800	4.19767400
C	1.51422400	3.54817400	3.69015900
H	0.74491200	4.28316300	3.95117800
H	1.82693400	3.05474900	4.61661100
H	2.37373500	4.09330800	3.28627500
C	-2.45235300	-2.31923800	-0.58298300
H	-1.94593000	-3.06733600	0.03771000
H	-4.01622900	-0.37701700	0.05357300
C	-2.93289200	-0.00531900	-0.17770000
Br	-2.94218900	1.84717600	-0.19869300
H	5.09777700	0.30039300	0.32984900
H	3.95898500	2.84949600	0.36534100
H	-2.14192600	-2.52439800	-1.61843500
C	-4.59150800	-1.17453600	-0.95669400
C	-5.98965800	-0.78786300	-0.57388200
C	-3.99209800	-2.46374300	-0.47313200
H	-4.29275300	-0.86890900	-1.96062900
C	-6.31789500	-1.10028100	0.88250900
H	-6.63401300	-1.37731900	-1.24885200
H	-6.17485900	0.26182300	-0.82994500
C	-4.40648100	-2.73552200	0.98591200
C	-5.89466000	-2.51923700	1.25145000
H	-7.38849900	-0.95186400	1.05457200
H	-5.79767700	-0.37963200	1.53106300
H	-4.11289800	-3.76028500	1.24077900
H	-3.82693100	-2.07535900	1.64647100
H	-6.10764000	-2.70942500	2.30850900
H	-6.49417600	-3.24233500	0.68288400
C	-4.46403300	-3.59138300	-1.40621200
H	-4.23708200	-3.36896400	-2.45425800
H	-3.94632200	-4.51786900	-1.13678100
H	-5.53982300	-3.76994600	-1.31924400

Cartesian coordinates of the optimized geometry for TS-**14c** at PBE0-D3BJ/6-31G*,def2-TZVP level of theory: (number of imaginary frequencies = 1):

Au	0.06376100	-0.30550400	-0.05661800
C	2.05652500	-0.67836200	-0.16726900
C	-1.88854900	0.21693600	0.06955500
C	-3.72463000	1.51533800	0.21489400
C	-4.13653800	0.22153700	0.20537300
N	-2.34704700	1.48777900	0.13124700
N	-2.99658700	-0.55306600	0.11617500
C	-2.96682900	-1.98604700	0.07105500
C	-3.02149700	-2.61040300	-1.18228800
C	-2.85537900	-2.68565600	1.27986200
C	-2.96070700	-4.00473500	-1.20052200
C	-2.79863100	-4.07831400	1.20503800
C	-2.85147500	-4.73076900	-0.02068700
H	-2.99763200	-4.52801900	-2.15163100
H	-2.71057300	-4.65863900	2.11903100
H	-2.80791400	-5.81558300	-0.05683400
C	-1.49007800	2.63880800	0.10945800
C	-1.14954600	3.19039000	-1.13317400
C	-1.01430800	3.13623200	1.33022900
C	-0.30648600	4.30297400	-1.12682300
C	-0.17413100	4.24996300	1.28068100
C	0.17175900	4.83149200	0.06642400
H	-0.02070900	4.76015000	-2.06982400
H	0.21380400	4.66624900	2.20599100
H	0.81926800	5.70390900	0.04986300

C	-2.76151200	-1.98049700	2.61776100
H	-2.94863000	-0.91272900	2.45600700
C	-1.34981700	-2.11406600	3.19567700
H	-1.10184700	-3.16427900	3.38798800
H	-0.60304700	-1.71025200	2.50193800
H	-1.27000300	-1.56900000	4.14298600
C	-3.81657800	-2.48421400	3.60388400
H	-3.76735600	-1.91093400	4.53583600
H	-4.82679100	-2.38401600	3.19368200
H	-3.66170000	-3.53846000	3.85800100
C	-1.34847000	2.48552800	2.65672800
H	-2.08417600	1.69398000	2.47454800
C	-1.97665100	3.47782300	3.63650800
H	-2.26083400	2.96694100	4.56268800
H	-1.27827100	4.27865800	3.90315000
H	-2.87322100	3.94320500	3.21394300
C	-0.10335600	1.82294800	3.25281100
H	0.31888400	1.08784400	2.55782800
H	0.67261100	2.56569700	3.47046600
H	-0.35318100	1.31057600	4.18864000
C	-1.63234500	2.60209200	-2.44298900
H	-2.33114600	1.78875100	-2.21689900
C	-3.10196700	-1.82383400	-2.47501500
H	-3.28034600	-0.77160900	-2.22517700
C	-1.77036400	-1.89527600	-3.22782800
H	-0.94802800	-1.51844700	-2.60853600
H	-1.53530800	-2.92743400	-3.51205000
H	-1.81452800	-1.29449500	-4.14320900
C	-4.26420100	-2.28583500	-3.35529600
H	-4.33756800	-1.65304700	-4.24620100
H	-4.12822300	-3.31785900	-3.69642400
H	-5.21737100	-2.23338200	-2.81902000
C	-0.46161600	1.99462700	-3.22055900
H	0.27457900	2.76068900	-3.48921600
H	0.04975600	1.22943800	-2.62537600
H	-0.81809700	1.52985500	-4.14654000
C	-2.38613100	3.63382900	-3.28390900
H	-1.73443000	4.46130400	-3.58513400
H	-2.77224900	3.16888900	-4.19731700
H	-3.23169600	4.05805600	-2.73244100
C	2.69141100	-2.03596700	-0.00172100
H	2.16472500	-2.80129300	-0.58147000
C	4.19343200	-1.98086300	-0.33374700
C	4.68136100	-0.68520600	0.24213800
H	3.98088600	0.15101600	-0.70919900
C	2.90476700	0.34626900	-0.31515900
Br	2.67868400	2.18039700	-0.11138700
H	-5.12043800	-0.21828400	0.25222000
H	-4.27360400	2.44218600	0.27217000
H	2.55475100	-2.30574200	1.05747900
C	5.03624200	-3.04981300	0.40709500
H	4.77406300	-4.03880700	0.01330800
C	6.53674000	-2.77975500	0.26079800
C	6.08850000	-0.26901600	-0.02053500
C	6.91736500	-1.36691700	0.70404500
H	6.31064600	0.71445500	0.40325300
H	6.32880800	-0.25527500	-1.08753800
H	7.97781600	-1.16947400	0.51421000
H	6.76509500	-1.26337400	1.78629400
H	7.09385000	-3.50716200	0.86129000
H	6.84996000	-2.94039100	-0.77747900
H	4.38114600	-0.52348000	1.27904000
H	4.76372900	-3.04592600	1.47112300
C	4.39113700	-2.08337200	-1.85024500
H	4.05096200	-3.06736100	-2.18786300
H	3.79971600	-1.33122000	-2.38321100

H	5.43353000	-1.97514200	-2.15849900

Cartesian coordinates of the optimized geometry for TS-**15a** at PBE0-D3BJ/6-31G*,def2-TZVP level of theory: (number of imaginary frequencies = 1):

Au	0.07124400	-0.28500400	-0.10901300
C	2.09055500	-0.67398600	-0.31020100
C	-1.87091000	0.20855300	0.09985700
C	-3.71121700	1.47738500	0.36292600
C	-4.11070600	0.18134600	0.29786900
N	-2.33581400	1.46979600	0.23938200
N	-2.96680400	-0.57638900	0.13691300
C	-2.92075600	-2.00557700	0.02265700
C	-2.98859700	-2.57011700	-1.25808500
C	-2.77893000	-2.75925000	1.19548500
C	-2.90998300	-3.96112400	-1.34306700
C	-2.70506900	-4.14580900	1.05350400
C	-2.77076200	-4.74021400	-0.20087800
H	-2.95682000	-4.43959000	-2.31705500
H	-2.59294600	-4.76745300	1.93710900
H	-2.71328700	-5.82133300	-0.28910900
C	-1.48589600	2.62625600	0.25277800
C	-1.19791900	3.25287500	-0.96768700
C	-0.96211700	3.05174700	1.48144800
C	-0.35869500	4.36805200	-0.92855900
C	-0.12892000	4.17202100	1.46481000
C	0.16478100	4.82770400	0.27458600
H	-0.11496900	4.88388200	-1.85294700
H	0.29257100	4.53604000	2.39747600
H	0.80497200	5.70571300	0.28499300
C	-2.66948400	-2.11632800	2.56334600
H	-2.88136800	-1.04607600	2.45719300
C	-1.24312800	-2.24854800	3.10470700
H	-0.97000200	-3.30137700	3.23923000
H	-0.51810000	-1.79597900	2.41794600
H	-1.15369200	-1.74966700	4.07622600
C	-3.69242600	-2.68743400	3.54663100
H	-3.63455200	-2.15661500	4.50285000
H	-4.71316100	-2.59030600	3.16264700
H	-3.51033700	-3.74850000	3.74862200
C	-1.24500600	2.32622200	2.78086500
H	-1.96581400	1.52627500	2.57729400
C	-1.87210300	3.25484100	3.82229600
H	-2.11852400	2.69405500	4.73014500
H	-1.18704100	4.06060000	4.10791100
H	-2.79102200	3.71544100	3.44500500
C	0.02922600	1.66679600	3.31553000
H	0.45032700	0.97124400	2.58019400
H	0.79445300	2.41549500	3.54976800
H	-0.18598000	1.10729200	4.23253100
C	-1.73111700	2.74001300	-2.28963900
H	-2.42727600	1.91915900	-2.08341700
C	-3.10466600	-1.72587900	-2.51100500
H	-3.27322200	-0.68562800	-2.20929700
C	-1.79699300	-1.76485300	-3.30680700
H	-0.95452400	-1.41826600	-2.69677000
H	-1.57298800	-2.78271300	-3.64574100
H	-1.86707800	-1.12238100	-4.19164200
C	-4.29411400	-2.14785000	-3.37520400
H	-4.39140800	-1.47868500	-4.23672700
H	-4.17176400	-3.16539400	-3.76184100
H	-5.23084200	-2.11536700	-2.80925300
C	-0.59244000	2.17050700	-3.14033100
H	0.14018200	2.94622200	-3.39045600
H	-0.06743200	1.36815100	-2.60907000

H	-0.98347200	1.76160700	-4.07849200
C	-2.50697400	3.81946400	-3.04637200
H	-1.86044900	4.65726300	-3.32953300
H	-2.93090400	3.40498900	-3.96714600
H	-3.32765800	4.21973100	-2.44207600
C	2.74106600	-2.01539900	-0.39745500
H	2.14134700	-2.69420700	-1.01171800
C	4.16535100	-1.91900000	-0.98899700
H	4.05110300	-1.78090700	-2.07180200
C	4.93947400	-0.73638100	-0.44981500
H	4.11446100	0.13943600	-0.45649400
C	2.73105600	0.44777800	-0.31873100
Br	2.68789500	2.25054600	-0.16148500
H	-5.08856600	-0.27114300	0.34993600
H	-4.26720100	2.39391000	0.48369200
H	2.75177200	-2.44803800	0.61157000
C	4.94861800	-3.22509400	-0.74051700
H	4.32941000	-4.07495000	-1.05129800
H	5.82716800	-3.22532400	-1.39886000
C	5.35881300	-0.88926200	1.00238100
C	5.40594100	-3.38667300	0.70654900
C	6.18844700	-2.16691200	1.18146200
H	7.12705200	-2.08367700	0.61791400
H	6.46725300	-2.27061800	2.23561000
H	5.92393000	-0.00850600	1.32643900
H	4.46650000	-0.95281700	1.63846100
H	6.02114600	-4.28933700	0.79247600
H	4.54141400	-3.54369800	1.36598100
C	6.00588000	-0.19159600	-1.37440500
H	6.43656700	0.73589300	-0.98532300
H	6.82144500	-0.92028200	-1.46678400
H	5.61140200	-0.00689300	-2.37805600

Cartesian coordinates of the optimized geometry for TS-**15b** at PBE0-D3BJ/6-31G*,def2-TZVP level of theory: (number of imaginary frequencies = 1):

Au	0.08801200	-0.24701800	-0.25566300
C	2.07672900	-0.41855700	-0.64725100
C	-1.85728900	0.07738500	0.19730800
C	-3.74399600	1.18049900	0.74506000
C	-4.04478900	-0.14217200	0.68388700
N	-2.40114800	1.29058500	0.44442400
N	-2.87553600	-0.79597000	0.34764600
C	-2.73365500	-2.21279000	0.17762300
C	-2.95217500	-2.75224100	-1.09704000
C	-2.35534100	-2.97914100	1.28796200
C	-2.77754400	-4.12940500	-1.24224600
C	-2.19492600	-4.35122400	1.08799700
C	-2.40416300	-4.92028000	-0.16230000
H	-2.93407000	-4.58759400	-2.21446100
H	-1.90172400	-4.98141500	1.92283400
H	-2.27597600	-5.99060800	-0.29636300
C	-1.65410400	2.51523800	0.40113800
C	-1.56778600	3.19900400	-0.81922600
C	-1.02537800	2.95157600	1.57531800
C	-0.82815200	4.38285100	-0.83537700
C	-0.29940900	4.14185000	1.50526700
C	-0.20625500	4.85384700	0.31499300
H	-0.73858300	4.94216500	-1.76219400
H	0.19923600	4.51490500	2.39536200
H	0.35571100	5.78328400	0.28348000
C	-2.09563100	-2.36303500	2.64780300
H	-2.38140400	-1.30580100	2.60527500
C	-0.60374400	-2.41847900	2.98797900
H	-0.25288800	-3.45491600	3.05215500

H	-0.00928900	-1.90466300	2.22321900
H	-0.41284300	-1.93685100	3.95364800
C	-2.94035400	-3.02053900	3.74044300
H	-2.78066000	-2.51452900	4.69863200
H	-4.00797200	-2.97278600	3.50229300
H	-2.67502600	-4.07464300	3.87638100
C	-1.08302300	2.16425600	2.86829800
H	-1.75823500	1.31357400	2.72213000
C	-1.64319800	3.00182500	4.01907200
H	-1.72687300	2.39286300	4.92558900
H	-0.99326900	3.85247100	4.25182600
H	-2.63619200	3.39605200	3.77933000
C	0.29749400	1.59688400	3.21040200
H	0.67390200	0.96713400	2.39581600
H	1.02384600	2.39999700	3.37950000
H	0.24631200	0.99001400	4.12137300
C	-2.20588000	2.67604400	-2.08956200
H	-2.79909900	1.79018100	-1.83586700
C	-3.31934300	-1.88923600	-2.28725600
H	-3.55423800	-0.88321100	-1.92085600
C	-2.12804300	-1.76205100	-3.24118900
H	-1.25447300	-1.34463900	-2.72712100
H	-1.84599200	-2.73987200	-3.64820500
H	-2.37824000	-1.10540800	-4.08201700
C	-4.56025000	-2.41003200	-3.01371900
H	-4.83967900	-1.72471200	-3.82099300
H	-4.38320500	-3.39236300	-3.46491100
H	-5.41297800	-2.50269400	-2.33317500
C	-1.12809900	2.23508800	-3.08378200
H	-0.50279600	3.08161500	-3.38941100
H	-0.47342000	1.47525500	-2.64184000
H	-1.58778800	1.81250100	-3.98407800
C	-3.15553700	3.70047500	-2.71263400
H	-2.62185200	4.60143300	-3.03447200
H	-3.64491700	3.27483900	-3.59514800
H	-3.93299500	4.00709900	-2.00510200
C	2.84139400	-1.70476000	-0.63526100
H	2.34643500	-2.48054000	-1.22865100
C	4.30308200	-1.50596600	-1.09072600
H	4.32755200	-1.51068300	-2.18729300
C	4.78703800	-0.17808400	-0.61097400
H	3.83687100	0.62038600	-1.33239400
C	2.79135700	0.69322500	-0.82458200
Br	2.44169400	2.47921800	-0.45757800
H	-4.96848600	-0.67577900	0.84434900
H	-4.35006400	2.04394000	0.97057600
H	2.82373100	-2.06666000	0.40362600
C	5.25125500	-2.62759700	-0.57058400
H	4.70121200	-3.57050200	-0.68973400
C	5.02506400	0.01143800	0.85081300
C	5.56968100	-2.44087800	0.91859100
C	6.08265200	-1.04175200	1.24602200
H	7.02938600	-0.83937000	0.73073300
H	6.28382800	-0.94271400	2.31783100
H	5.36602700	1.02732500	1.06837800
H	4.11234700	-0.17410300	1.42490500
H	6.30877900	-3.19240100	1.21855600
H	4.67351900	-2.63848300	1.52203900
C	6.51418600	-2.71215300	-1.42519700
H	7.14315600	-3.54582200	-1.09696100
H	6.26693700	-2.87761900	-2.47916900
H	7.12015500	-1.80076100	-1.36671000
H	5.52961300	0.31796800	-1.24705700

Cartesian coordinates of the optimized geometry for TS-**15c** at PBE0-D3BJ/6-31G*,def2-TZVP level of theory: (number of imaginary frequencies = 1):

Au	-0.00033400	-0.38199800	-0.17614600
C	-1.90264300	-1.11069400	-0.50350100
C	1.80533100	0.44443000	0.16563400
C	3.36281300	2.01007100	0.60082900
C	3.99747700	0.81356400	0.50520100
N	2.02128400	1.75964000	0.38945400
N	3.02365000	-0.12918500	0.23798100
C	3.24791100	-1.53477200	0.05940700
C	3.15449200	-2.36842700	1.18176000
C	3.51698500	-2.00296300	-1.23376500
C	3.34563600	-3.73548300	0.97417000
C	3.69927200	-3.37837800	-1.38529300
C	3.61566500	-4.23486900	-0.29406300
H	3.28063000	-4.41726400	1.81729400
H	3.90690700	-3.78390600	-2.37121000
H	3.76264500	-5.30202400	-0.43360600
C	0.97261800	2.73942100	0.40532800
C	0.32537100	2.99965500	1.62137800
C	0.62777500	3.36394500	-0.80130600
C	-0.69745500	3.94991300	1.60809400
C	-0.40230300	4.30568200	-0.75940900
C	-1.05326700	4.60174500	0.43279200
H	-1.21975400	4.18306500	2.53159500
H	-0.69547700	4.81508200	-1.67295800
H	-1.84306000	5.34799800	0.44647000
C	3.56744400	-1.07929000	-2.43407300
H	3.55315300	-0.04489700	-2.07138100
C	2.32578300	-1.27458200	-3.30912200
H	2.28425200	-2.29439200	-3.70842400
H	1.40823800	-1.09851200	-2.73542900
H	2.33995700	-0.57997500	-4.15652800
C	4.85199000	-1.25939900	-3.24422500
H	4.88931800	-0.52893500	-4.05935900
H	5.74104500	-1.12144500	-2.62027800
H	4.91021300	-2.25606300	-3.69482400
C	1.30182100	3.02316700	-2.11443800
H	2.12147600	2.32554300	-1.90866600
C	1.90825600	4.26027000	-2.77905400
H	2.43743400	3.97660900	-3.69494800
H	1.13752300	4.98796400	-3.05565600
H	2.61938400	4.76252700	-2.11501900
C	0.32040900	2.31096400	-3.04943100
H	-0.07917700	1.40330300	-2.58224800
H	-0.52489600	2.96010500	-3.30461200
H	0.81939100	2.02549900	-3.98202700
C	0.67629300	2.26934800	2.90145200
H	1.54539300	1.63130000	2.70536200
C	2.82881200	-1.83698300	2.56291000
H	2.82449400	-0.74181100	2.51700100
C	1.42846700	-2.28180100	2.99383700
H	0.67126500	-1.94797200	2.27449600
H	1.36712300	-3.37353100	3.06761300
H	1.17803800	-1.86297500	3.97491700
C	3.88377100	-2.24590000	3.59236700
H	3.65750500	-1.79631600	4.56510700
H	3.91284300	-3.33214400	3.73060100
H	4.88438800	-1.92053400	3.28952000
C	-0.47475600	1.35405200	3.32813800
H	-1.37922700	1.93225200	3.54881900
H	-0.71799800	0.63369100	2.53842300
H	-0.20406000	0.79493600	4.23058700
C	1.06248400	3.23873700	4.01996400
H	0.22284500	3.88307300	4.30260600
H	1.36716800	2.68355800	4.91344700

H	1.89351800	3.88515100	3.71926400
C	-2.28724400	-2.53279900	-0.77731400
H	-1.68806600	-3.21261300	-0.16326500
H	-4.04498900	-0.67499000	-0.63328300
C	-2.75431900	-0.13938100	-0.44150100
Br	-3.03382000	1.64182700	-0.25749700
H	5.03767400	0.54399400	0.60002700
H	3.73265800	3.00430400	0.79684100
H	-2.03337400	-2.75946500	-1.82038600
C	-4.63249400	-1.64508400	-1.06519500
C	-6.02980100	-1.46918900	-0.49588000
C	-3.78906100	-2.75890100	-0.48277500
C	-6.10991400	-1.62532700	1.01557800
H	-6.65558000	-2.23782100	-0.97746800
H	-6.43504400	-0.50489400	-0.82529300
C	-3.99335000	-2.95098700	1.02483000
H	-4.08005200	-3.67885500	-1.01219100
C	-5.45545100	-2.93081100	1.44921400
H	-7.15781900	-1.58635300	1.33140600
H	-5.60282800	-0.78005200	1.50284900
H	-3.51364700	-3.89044100	1.32329000
H	-3.45939600	-2.14823700	1.55366100
H	-5.52841200	-3.05627100	2.53486400
H	-5.99042900	-3.77852300	0.99809500
C	-4.58031500	-1.50893600	-2.57162200
H	-5.09332900	-2.36383100	-3.02895100
H	-5.09460600	-0.60111500	-2.90148200
H	-3.55674700	-1.48310400	-2.95425600

Cartesian coordinates of the optimized geometry for TS-**15d** at PBE0-D3BJ/6-31G*,def2-TZVP level of theory: (number of imaginary frequencies = 1):

Au	-0.04522300	-0.31723000	-0.18097100
C	-1.98666600	-0.82444700	-0.51042600
C	1.83797100	0.34159600	0.15669700
C	3.53600600	1.76013000	0.58265100
C	4.06124900	0.51317100	0.46937300
N	2.17551700	1.63060100	0.38756300
N	3.00400600	-0.33648100	0.20865400
C	3.10356100	-1.75431300	0.01826500
C	2.97671200	-2.58401200	1.14028200
C	3.29294100	-2.23661000	-1.28360200
C	3.04719900	-3.96104000	0.92314900
C	3.35608500	-3.62159700	-1.44498600
C	3.23547500	-4.47430300	-0.35439600
H	2.95189600	-4.63962300	1.76597200
H	3.50002200	-4.03707500	-2.43822800
H	3.28960600	-5.54922300	-0.50144800
C	1.22181000	2.70247300	0.42060400
C	0.60982700	3.01032100	1.64340700
C	0.92910700	3.36857600	-0.77721100
C	-0.32180400	4.04987200	1.64578700
C	-0.00961700	4.40028700	-0.71983600
C	-0.62444100	4.74206000	0.47905000
H	-0.81627000	4.31962100	2.57459200
H	-0.26124300	4.94237300	-1.62692500
H	-1.34497900	5.55500500	0.50451500
C	3.38936000	-1.31451700	-2.48214700
H	3.44502300	-0.28267500	-2.11669400
C	2.12973100	-1.42571600	-3.34572900
H	2.01889300	-2.43844000	-3.75005000
H	1.23186500	-1.19358400	-2.76105100
H	2.18052900	-0.72865500	-4.18973500
C	4.65251200	-1.57581700	-3.30396300
H	4.72769200	-0.85143700	-4.12200200

H	5.55414100	-1.49146100	-2.68849200
H	4.64457500	-2.57599400	-3.75064000
C	1.56041900	2.97859300	-2.09783600
H	2.31855800	2.21177500	-1.90230200
C	2.26619100	4.16167800	-2.76246700
H	2.76178800	3.83789400	-3.68392500
H	1.55913500	4.95461300	-3.02982100
H	3.02318700	4.59756500	-2.10220400
C	0.51309400	2.35834100	-3.02677800
H	0.03900500	1.48880300	-2.55689800
H	-0.27426300	3.08018500	-3.27216500
H	0.97769600	2.03350100	-3.96444700
C	0.90133700	2.23942400	2.91442000
H	1.70666500	1.52600700	2.70601600
C	2.73607100	-2.03335200	2.53133100
H	2.84352200	-0.94327900	2.49151100
C	1.30563800	-2.33486700	2.98710900
H	0.57369000	-1.91703800	2.28605500
H	1.13391700	-3.41540700	3.05289900
H	1.11932900	-1.90251800	3.97664200
C	3.76256300	-2.55591900	3.53761700
H	3.60472800	-2.08876100	4.51558100
H	3.67950200	-3.63963500	3.67406200
H	4.78570100	-2.33685300	3.21503500
C	-0.32598200	1.42983100	3.34171500
H	-1.17061200	2.08726100	3.57733100
H	-0.64188100	0.74679500	2.54482800
H	-0.10070000	0.83664800	4.23498700
C	1.38300200	3.15895800	4.03788500
H	0.60877400	3.87641900	4.33119900
H	1.64017100	2.57039900	4.92502600
H	2.26877100	3.72782700	3.73650300
C	-2.46785500	-2.17712200	-0.95465800
H	-1.97566700	-2.97905900	-0.39731900
H	-4.02738400	-0.25231500	-0.24481200
C	-2.92413100	0.10984300	-0.38032400
Br	-2.89550100	1.95738700	-0.25409500
H	5.07460300	0.15186700	0.54854300
H	3.99473900	2.71606400	0.78129000
H	-2.14254600	-2.29327000	-1.99833000
C	-4.57484400	-0.97937300	-1.33149600
C	-5.98142700	-0.57826200	-1.00765400
C	-4.01070400	-2.28981700	-0.88166000
C	-6.41412100	-1.02272700	0.38335000
H	-6.59682200	-1.07478700	-1.77741100
H	-6.11503700	0.49774200	-1.17148500
C	-4.53930300	-2.73147900	0.49694100
H	-4.32615900	-3.02899200	-1.63585400
C	-6.04468800	-2.48183200	0.62225000
H	-7.49131300	-0.86681900	0.49820800
H	-5.92669100	-0.38755500	1.13752500
H	-4.03143100	-2.11563500	1.25578700
H	-6.37760600	-2.79414600	1.61825200
H	-6.57940200	-3.12051600	-0.09681900
C	-4.21285000	-4.19598100	0.76782300
H	-4.56700000	-4.48802000	1.76148200
H	-4.70545700	-4.84645800	0.03466600
H	-3.13841100	-4.40100300	0.73142200
H	-4.20487100	-0.61664900	-2.29154300

Cartesian coordinates of the optimized geometry for TS-**15e** at PBE0-D3BJ/6-31G*,def2-TZVP level of theory: (number of imaginary frequencies = 1):

Au	-0.26293700	-0.48802900	-0.31915200
C	3.15047800	-0.07365300	-0.93691100

C	-1.56814300	0.93906400	0.25879500
C	-3.37352400	2.08258800	0.95975000
C	-2.33515900	2.95614500	0.90421000
N	-2.87938000	0.85599400	0.56194900
N	-1.24042500	2.23350900	0.47294300
C	0.08019200	2.75214500	0.26327100
C	0.39544700	3.27228500	-1.00022200
C	1.00126600	2.67979100	1.31802700
C	1.69307200	3.75292400	-1.18718000
C	2.28259900	3.18287600	1.08067200
C	2.62458400	3.71729400	-0.15645000
H	1.97472200	4.16404400	-2.15228000
H	3.01923900	3.15941300	1.87926700
H	3.62482800	4.10949300	-0.31845500
C	-3.63277200	-0.36302400	0.48365300
C	-4.24988200	-0.68344700	-0.73261200
C	-3.68134000	-1.18389000	1.61828000
C	-4.94672200	-1.89134800	-0.79083700
C	-4.39154600	-2.37991600	1.50536700
C	-5.01746300	-2.72956300	0.31505000
H	-5.43607300	-2.18093700	-1.71616900
H	-4.45115600	-3.04804100	2.35960600
H	-5.56423900	-3.66566200	0.24803100
C	0.65920900	2.06673700	2.66037200
H	-0.38619100	1.74015500	2.63229200
C	1.51548600	0.82470200	2.92301900
H	2.58044500	1.08019100	2.97842100
H	1.38163600	0.08063700	2.12883000
H	1.23355200	0.36072100	3.87436700
C	0.79467000	3.08743500	3.79208900
H	0.49711500	2.63911900	4.74594700
H	0.16360300	3.96457200	3.61536000
H	1.82832900	3.43548400	3.89666100
C	-2.96873600	-0.82818000	2.90757800
H	-2.59135100	0.19740600	2.81929100
C	-3.91292300	-0.86258000	4.11036900
H	-3.38665400	-0.53608200	5.01373500
H	-4.29098600	-1.87329800	4.29803600
H	-4.77561100	-0.20521500	3.96033900
C	-1.76032600	-1.74407900	3.12230500
H	-1.06466000	-1.68445400	2.27714600
H	-2.07262200	-2.78930900	3.22594500
H	-1.22168400	-1.46064200	4.03375600
C	-4.14247500	0.20591800	-1.95462000
H	-3.67654500	1.15081400	-1.65115300
C	-0.59892400	3.28308400	-2.14297900
H	-1.57055200	2.95753500	-1.75503800
C	-0.17945100	2.28194800	-3.22322200
H	-0.08974000	1.27053000	-2.81048300
H	0.78652500	2.55723200	-3.66216200
H	-0.92006000	2.25728900	-4.02998800
C	-0.78421800	4.68609200	-2.72337700
H	-1.55311700	4.67296200	-3.50295300
H	0.13912200	5.06055300	-3.17873200
H	-1.09121100	5.40068900	-1.95265400
C	-3.23223700	-0.43781500	-3.00448600
H	-3.64953600	-1.38789300	-3.35662200
H	-2.23673300	-0.64098300	-2.59291200
H	-3.11851100	0.22311500	-3.87110700
C	-5.51478000	0.54292000	-2.53983700
H	-6.02272500	-0.35032700	-2.91907000
H	-5.40702300	1.23943800	-3.37816500
H	-6.16612800	1.00614100	-1.79145500
C	4.36905700	-0.33428200	-0.05111200
C	5.05924100	-1.57753900	-0.62636700
H	5.47403200	-1.31551700	-1.61145500

C	4.02627500	-2.64631200	-0.81292900
H	3.12098900	-2.21271900	-1.64441300
C	2.42212300	-1.36291900	-1.07286100
H	-2.27230600	4.01009900	1.12549500
H	-4.40661300	2.21420800	1.24104400
H	3.99791800	-0.59463600	0.95297800
C	1.19790300	-1.81259100	-0.79464500
Br	0.84174200	-3.66087200	-0.83341900
H	2.47534800	0.66062900	-0.48666900
H	3.45939900	0.31387400	-1.91737100
C	5.35285500	0.81803900	0.07771900
H	5.70828000	1.10613600	-0.92252300
H	4.84698700	1.69434800	0.49891100
C	6.53527900	0.40260900	0.95404100
C	6.21573700	-2.02278800	0.27682300
H	5.81428100	-2.31428500	1.25751700
H	6.71738100	-2.90288400	-0.14132900
C	7.20779800	-0.86928800	0.43845900
H	8.01235100	-1.17338800	1.11679400
H	7.67889500	-0.66528200	-0.53313600
H	6.17848500	0.23402600	1.98005100
H	7.26706100	1.21599100	1.00827100
H	4.25569800	-3.43780700	-1.53605200
H	3.57957400	-3.06748100	0.08743800

Cartesian coordinates of the optimized geometry for TS-**15f** at PBE0-D3BJ/6-31G*,def2-TZVP level of theory: (number of imaginary frequencies = 1):

Au	0.08270300	-0.24465100	-0.07538600
C	2.10358000	-0.42362900	-0.19563500
C	-1.91006600	0.08532800	0.06416500
C	-3.86222500	1.19807800	0.23407800
C	-4.14735100	-0.12913100	0.20656600
N	-2.48875700	1.30490600	0.14589000
N	-2.93831900	-0.78865600	0.10275200
C	-2.77062300	-2.21141600	0.04014200
C	-2.76552900	-2.82323400	-1.22040900
C	-2.59290700	-2.91133700	1.24090700
C	-2.57113500	-4.20502800	-1.25491200
C	-2.40281200	-4.29111000	1.14988900
C	-2.39267900	-4.93106900	-0.08357900
H	-2.55809100	-4.71816100	-2.21217700
H	-2.25972900	-4.87107500	2.05714300
H	-2.24537800	-6.00622300	-0.13243500
C	-1.74674100	2.53323500	0.14066600
C	-1.47321300	3.13928600	-1.09308100
C	-1.30939100	3.05037900	1.36759500
C	-0.74085500	4.32751300	-1.07071500
C	-0.58066800	4.24059000	1.33415400
C	-0.30393900	4.87620800	0.12931700
H	-0.50963100	4.82829300	-2.00649300
H	-0.22585700	4.67431600	2.26476100
H	0.25649800	5.80710000	0.12550800
C	-2.56763500	-2.21576900	2.58686700
H	-2.86013400	-1.17011100	2.43741900
C	-1.14880000	-2.21471500	3.16275500
H	-0.79703500	-3.23717900	3.34238800
H	-0.44709800	-1.73001500	2.47381000
H	-1.12237300	-1.67577800	4.11654100
C	-3.56601900	-2.83275900	3.56757900
H	-3.57266000	-2.26777200	4.50584600
H	-4.58177100	-2.82884300	3.15883100
H	-3.30688800	-3.86913600	3.80996500
C	-1.56964600	2.34751600	2.68403900
H	-2.21638100	1.48431600	2.49006800

C	-2.29989100	3.25340000	3.67689800
H	-2.52507000	2.70340400	4.59689400
H	-1.69175400	4.12198600	3.95239100
H	-3.24248800	3.62401500	3.26078300
C	-0.26066600	1.81376900	3.27218500
H	0.23743600	1.13741400	2.56789900
H	0.43175100	2.63176500	3.50149500
H	-0.45417200	1.26471000	4.20049400
C	-1.90804200	2.53113400	-2.41052500
H	-2.53170700	1.65609700	-2.19508800
C	-2.92358600	-2.03327900	-2.50382600
H	-3.19546600	-1.00441500	-2.24132000
C	-1.59537000	-1.97504200	-3.26365500
H	-0.80702600	-1.53181800	-2.64414000
H	-1.26898700	-2.97819100	-3.56121800
H	-1.69932400	-1.37056000	-4.17170900
C	-4.04397200	-2.59015700	-3.38351500
H	-4.17896800	-1.95840700	-4.26793700
H	-3.81699400	-3.60252100	-3.73501100
H	-4.99515900	-2.62910200	-2.84257000
C	-0.69066000	2.04129200	-3.19946200
H	-0.02593300	2.87359700	-3.45731500
H	-0.11152800	1.31576300	-2.61685700
H	-1.00721900	1.56065800	-4.13183200
C	-2.75309500	3.50437500	-3.23411500
H	-2.17825700	4.39019100	-3.52570600
H	-3.10055900	3.02003200	-4.15292500
H	-3.63042900	3.84466200	-2.67415200
C	2.86351800	-1.71407100	-0.04297100
H	2.41720100	-2.51232100	-0.64309300
C	4.34762600	-1.50384000	-0.35662600
C	4.75130500	-0.16874100	0.16664500
H	3.93686300	0.58891900	-0.73507500
C	2.84126800	0.68044000	-0.34954600
Br	2.43998400	2.48409900	-0.15325200
H	-5.08397700	-0.66257400	0.24973400
H	-4.49781600	2.06666200	0.30675600
H	2.74891100	-2.02107000	1.00872900
C	5.33030300	-2.54053300	0.23816400
C	6.76051300	-2.11029300	-0.11003800
C	6.08443700	0.34574200	-0.26287800
C	7.08075400	-0.68467700	0.33094000
H	6.28862000	1.34918500	0.12052200
H	6.17679200	0.35352900	-1.35519400
H	8.08960000	-0.39600200	0.01687400
H	7.05855600	-0.61516400	1.42614400
H	7.47020700	-2.80354700	0.35576700
H	6.90451400	-2.20165600	-1.19618500
H	5.21665800	-2.51894400	1.33299000
H	4.49484200	-1.50102000	-1.44772800
H	4.51688700	0.01393400	1.21697900
C	5.02006000	-3.94619600	-0.25960400
H	4.02170100	-4.27530100	0.04489500
H	5.07756300	-3.99539000	-1.35348300
H	5.74271700	-4.66159200	0.14613100

4. Supporting Information References

[1] M. J. Frisch, G. W. Trucks, H. B. Schlegel, G. E. Scuseria, M. A. Robb, J. R. Cheeseman, G. Scalmani, V. Barone, G. A. Petersson, H. Nakatsuji, X. Li, M. Caricato, A. V. Marenich, J. Bloino, B. G. Janesko, R. Gomperts, B. Mennucci, H. P. Hratchian, J. V. Ortiz, A. F. Izmaylov, J. L. Sonnenberg, D. Williams-Young, F. Ding, F. Lipparini, F. Egidi, J. Goings, B. Peng, A. Petrone, T. Henderson, D. Ranasinghe, V. G. Zakrzewski, J. Gao, N. Rega, G. Zheng, W. Liang, M. Hada, M. Ehara, K. Toyota, R. Fukuda, J. Hasegawa, M. Ishida, T. Nakajima, Y. Honda, O. Kitao, H. Nakai, T. Vreven, K. Throssell, J. A. Montgomery, Jr.; , J. E. Peralta, F. Ogliaro, M. J. Bearpark, J. J. Heyd, E. N. Brothers, K. N. Kudin, V. N. Staroverov, T. A. Keith, R. Kobayashi, J. Normand, K. Raghavachari, A. P. Rendell, J. C. Burant, S. S. Iyengar, J. Tomasi, M. Cossi, J. M. Millam, M. Klene, C. Adamo, R. Cammi, J. W. Ochterski, R. L. Martin, K. Morokuma, O. Farkas, J. B. Foresman, D. J. Fox, Gaussian 16, Revision A.03, Wallingford CT, **2016**.

[2] C. Adamo, V. Barone, *J. Chem. Phys.* **1999**, *110*, 6158-6170.

[3] S. Grimme, S. Ehrlich, L. Goerigk, *J. Comp. Chem.* **2011**, *32*, 1456-1465.

[4] R. Ditchfield, W. J. Hehre, J. A. Pople, *J. Chem. Phys.* **1971**, *54*, 724-728.

[5] W. J. Hehre, R. Ditchfield, J. A. Pople, *J. Chem. Phys.* **1972**, *56*, 2257-2261.

[6] F. Weigend, R. Ahlrichs, *Phys. Chem. Chem. Phys.* **2005**, *7*, 3297-3305.

[7] D. Andrae, U. Häußermann, M. Dolg, H. Stoll, H. Preuß, *Theor. Chim. Acta* **1990**, *77*, 123-141.

[8] R. Krishnan, J. S. Binkley, R. Seeger, J. A. Pople, *J. Chem. Phys.* **1980**, *72*, 650-654.

[9] A. D. McLean, G. S. Chandler, *J. Chem. Phys.* **1980**, *72*, 5639-5648.

[10] L. A. Curtiss, M. P. McGrath, J. P. Blaudeau, N. E. Davis, R. C. Binning Jr, L. Radom, *J. Chem. Phys.* **1995**, *103*, 6104-6113.

[11] T. Clark, J. Chandrasekhar, G. W. Spitznagel, P. V. R. Schleyer, *J. Comput. Chem.* **1983**, *4*, 294-301.

[12] A. V. Marenich, C. J. Cramer, D. G. Truhlar, *J. Phys. Chem. B* **2009**, *113*, 6378-6396.
