# Supplementary material for: Conformational Effects in Intramolecular C(sp3)–H Bond Functionalization: Gold(I)-Catalyzed Cycloisomerization of Aliphatic 1‑Bromoalkynes as Benchmark Reaction
Source: Org Lett. 2025 Sep 22;27(39):11065–70. doi: 10.1021/acs.orglett.5c03430 (PMC12501934; doi:10.1021/acs.orglett.5c03430)

Conformational Effects in Intramolecular C(sp^3)—H bond Functionalization: Gold(I)-Catalyzed Cycloisomerization of Aliphatic 1-Bromoalkynes as Benchmark Reaction

Rubén Miguélez,[a,‡] Omar Arto,[a‡] Hannah Siera,[b] Jan Schulte,[b] Isabel Merino,[c] Gebhard Haberhauer,b Pablo Barrio[a*]

[a] Department of Organic and Inorganic Chemistry

Universidad de Oviedo

Julian Clavería 8 33006 Oviedo (Spain)

[b] Institut für Organische Chemie

Universität Duisburg-Essen

Universitätstraße 7, 45117 Essen (Germany)

[c] Servicios Científico Técnicos

Universidad de Oviedo

Fernando Bonguera s/n, 30006 Oviedo (Spain)

Correspondence to: barriopablo@uniovi.es

This PDF file includes:

Copy of NMR spectra

SYNTHESIS OF STARTING MATERIALS

1b

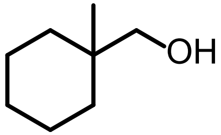

1b-OH

-crude-

^{1}H NMR(300 MHz, $CDCl_3$)

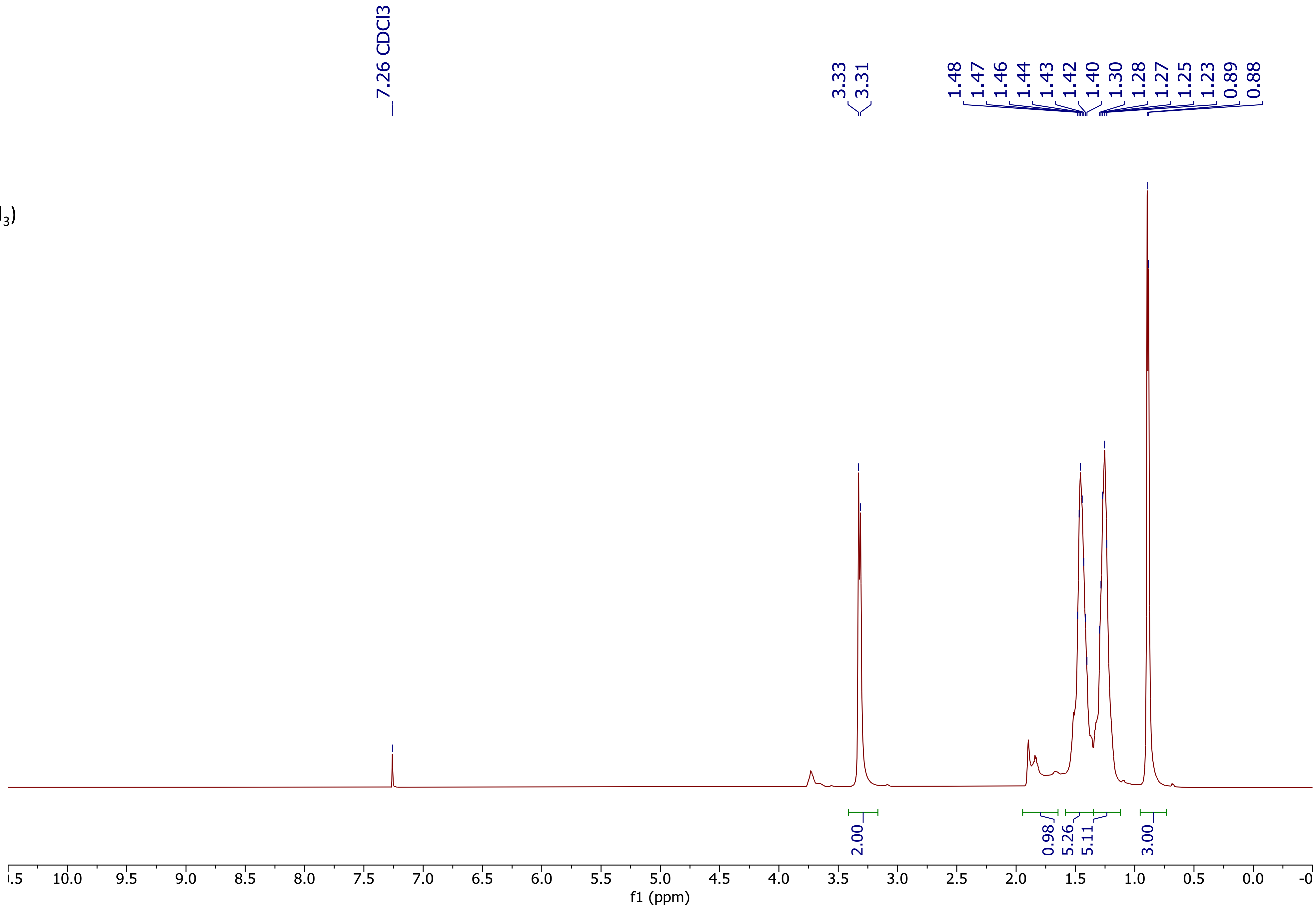

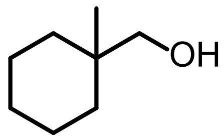

1b-OH

-crude-

^{13}C NMR (75 MHz, $CDCl_3$)

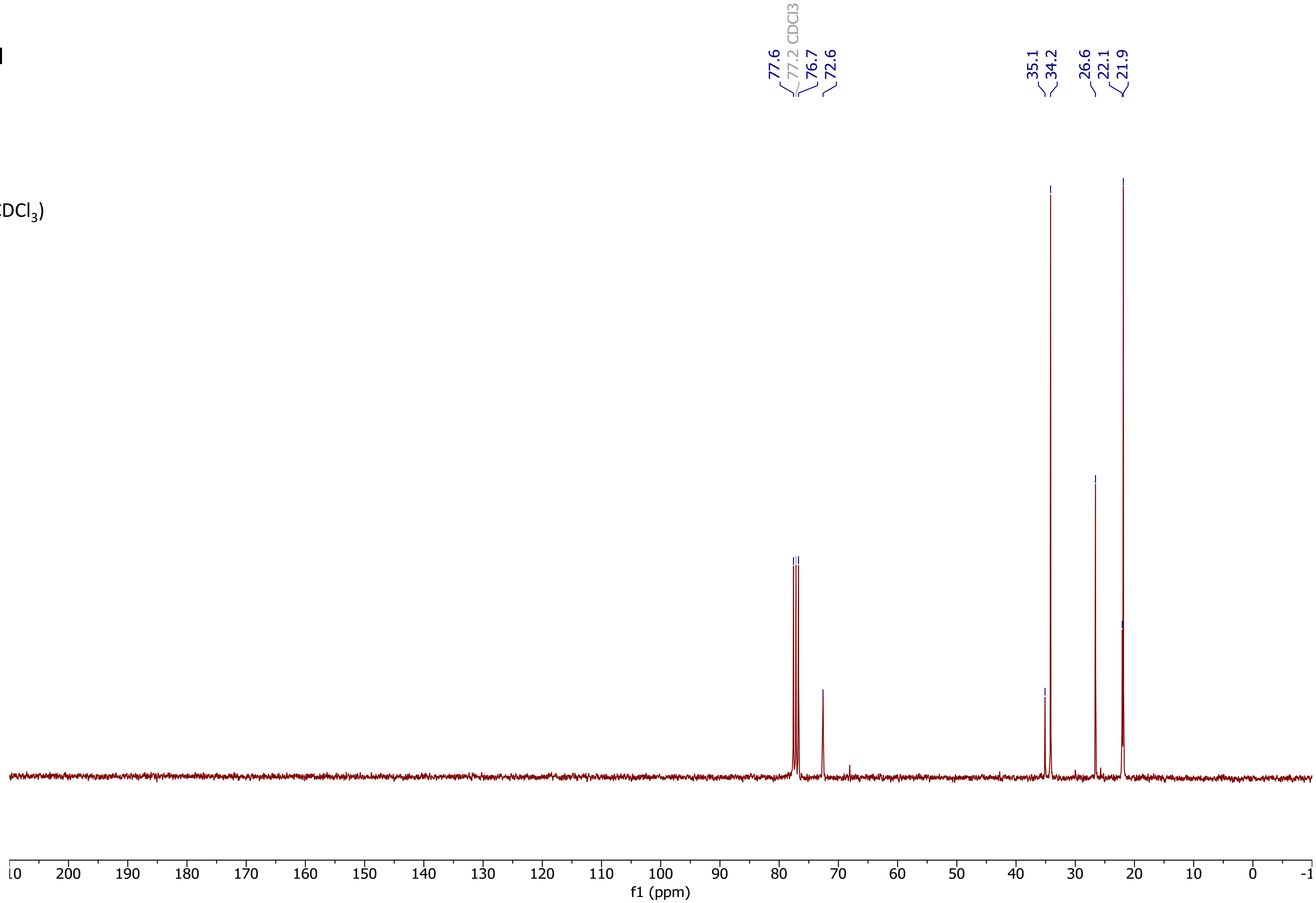

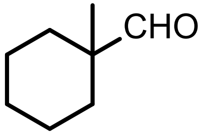

1b-CHO

-crude-

^{1}H NMR(300 MHz, $CDCl_3$)

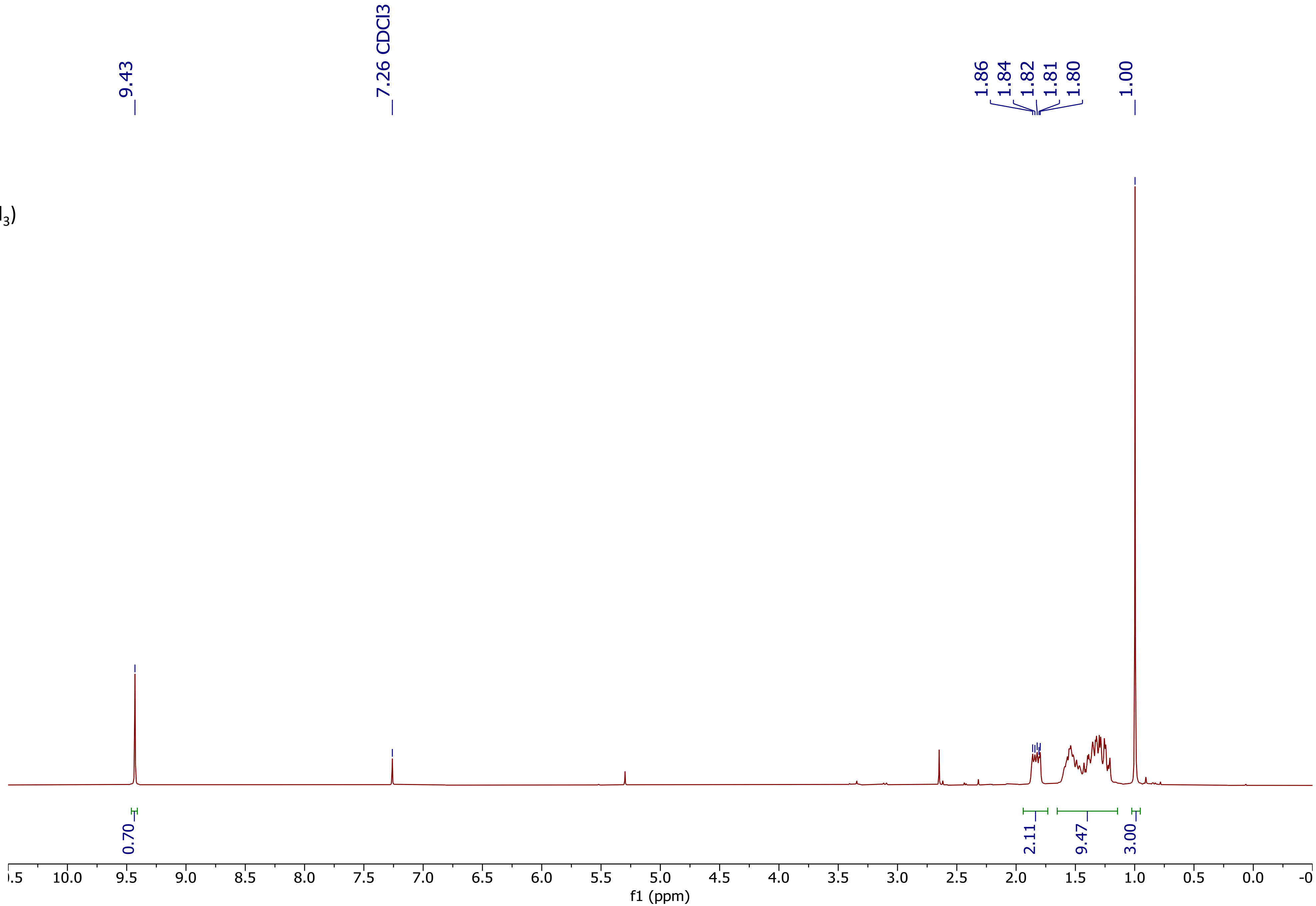

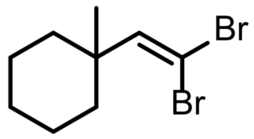

1b-CBr$_2$

^{1}H NMR(300 MHz, $CDCl_3$)

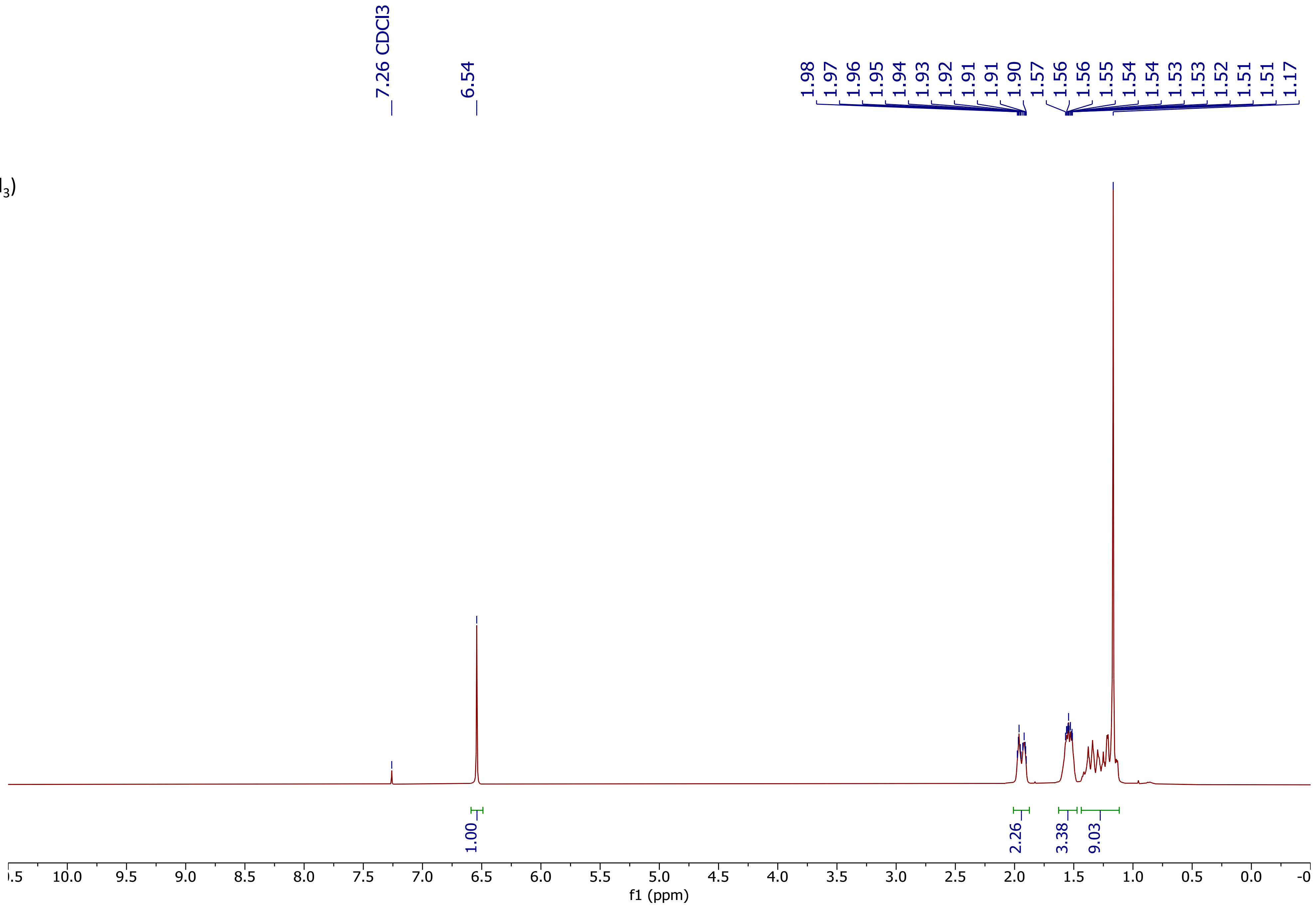

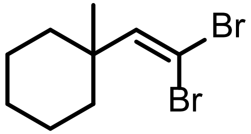

1b-CBr$_2$

^{13}C NMR (75 MHz, $CDCl_3$)

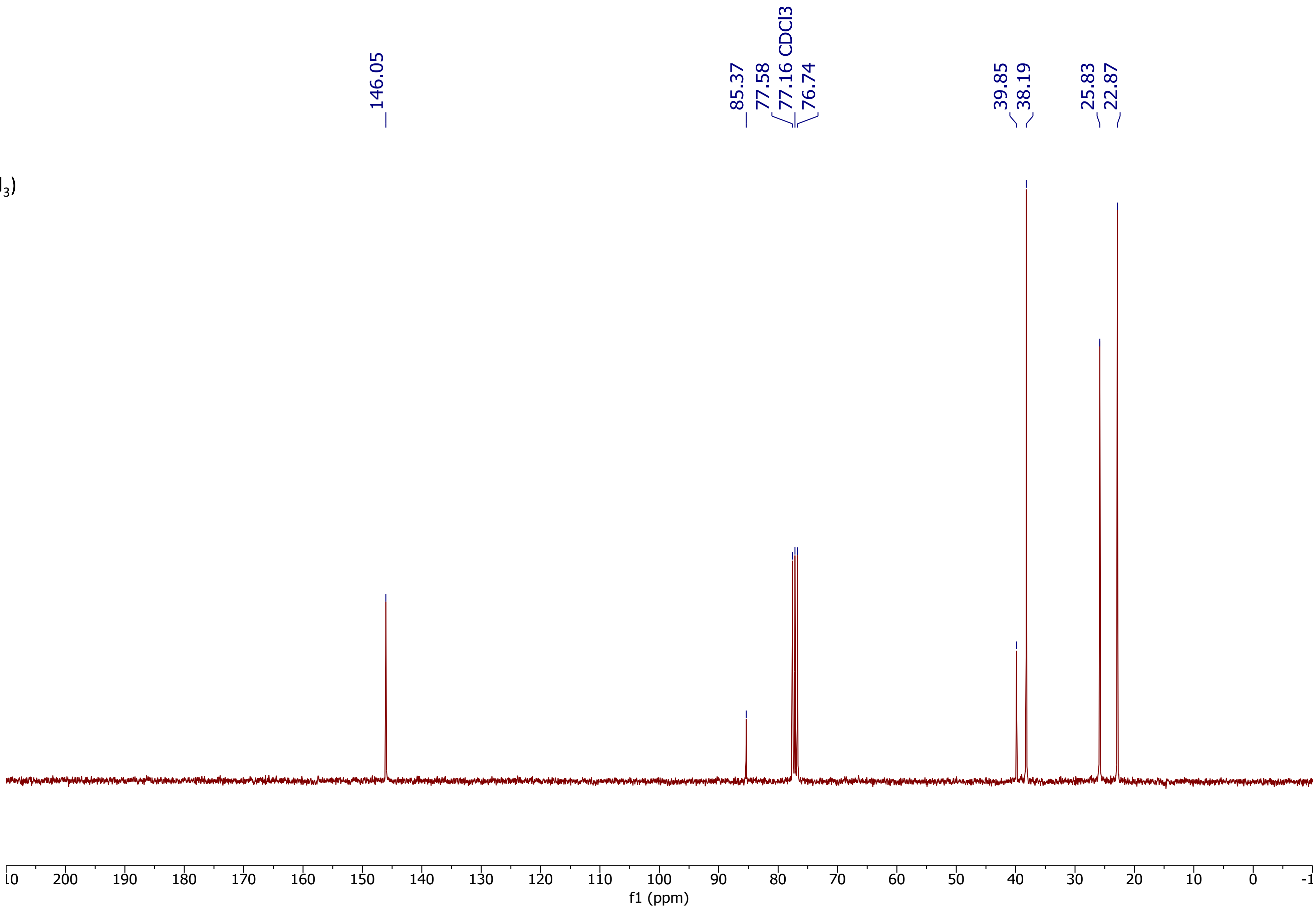

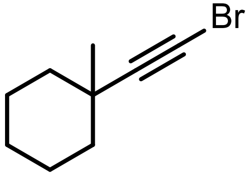

1b

^{1}H NMR(300 MHz, $CDCl_3$)

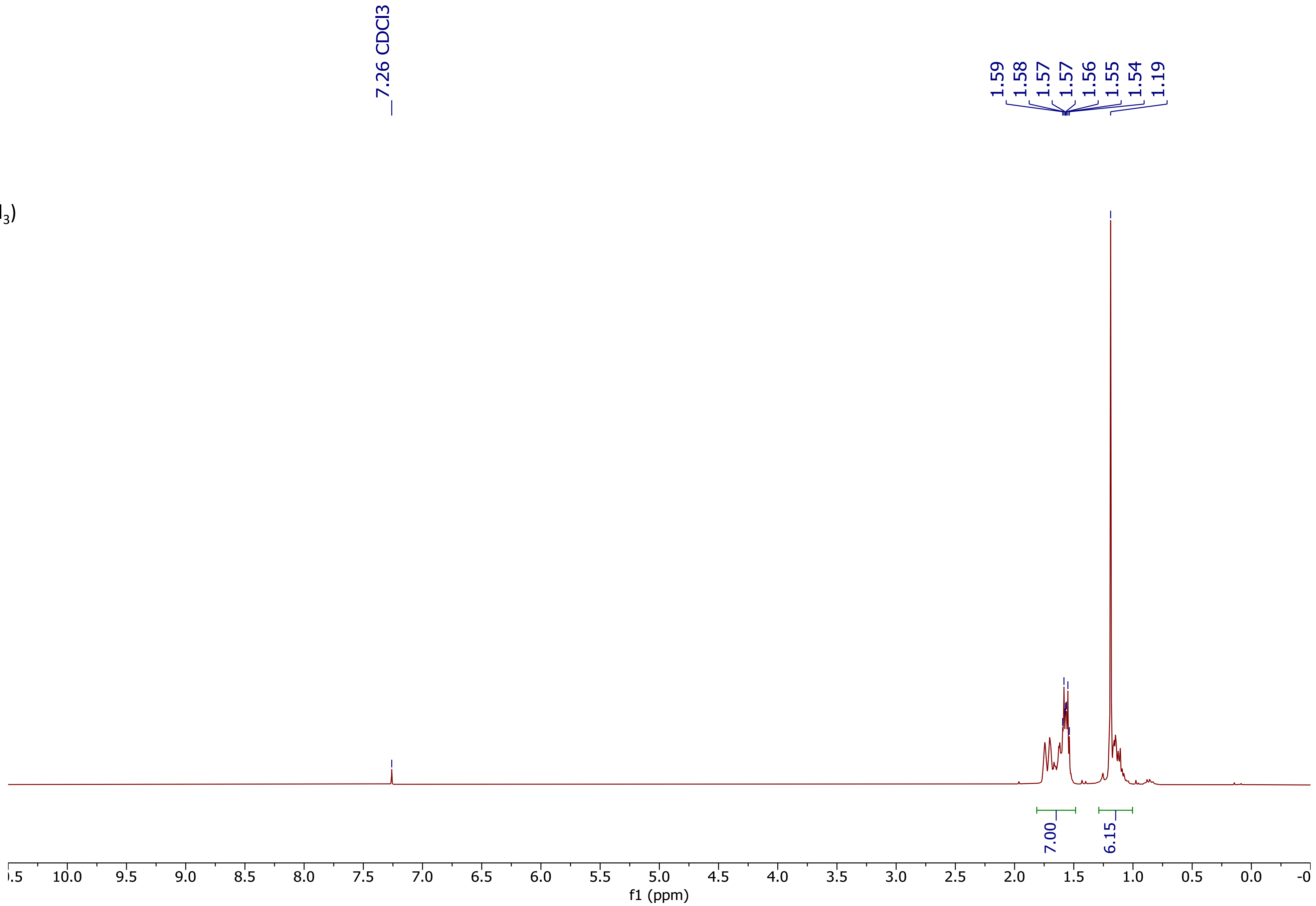

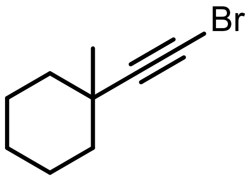

1b

^{13}C NMR (75 MHz, $CDCl_3$)

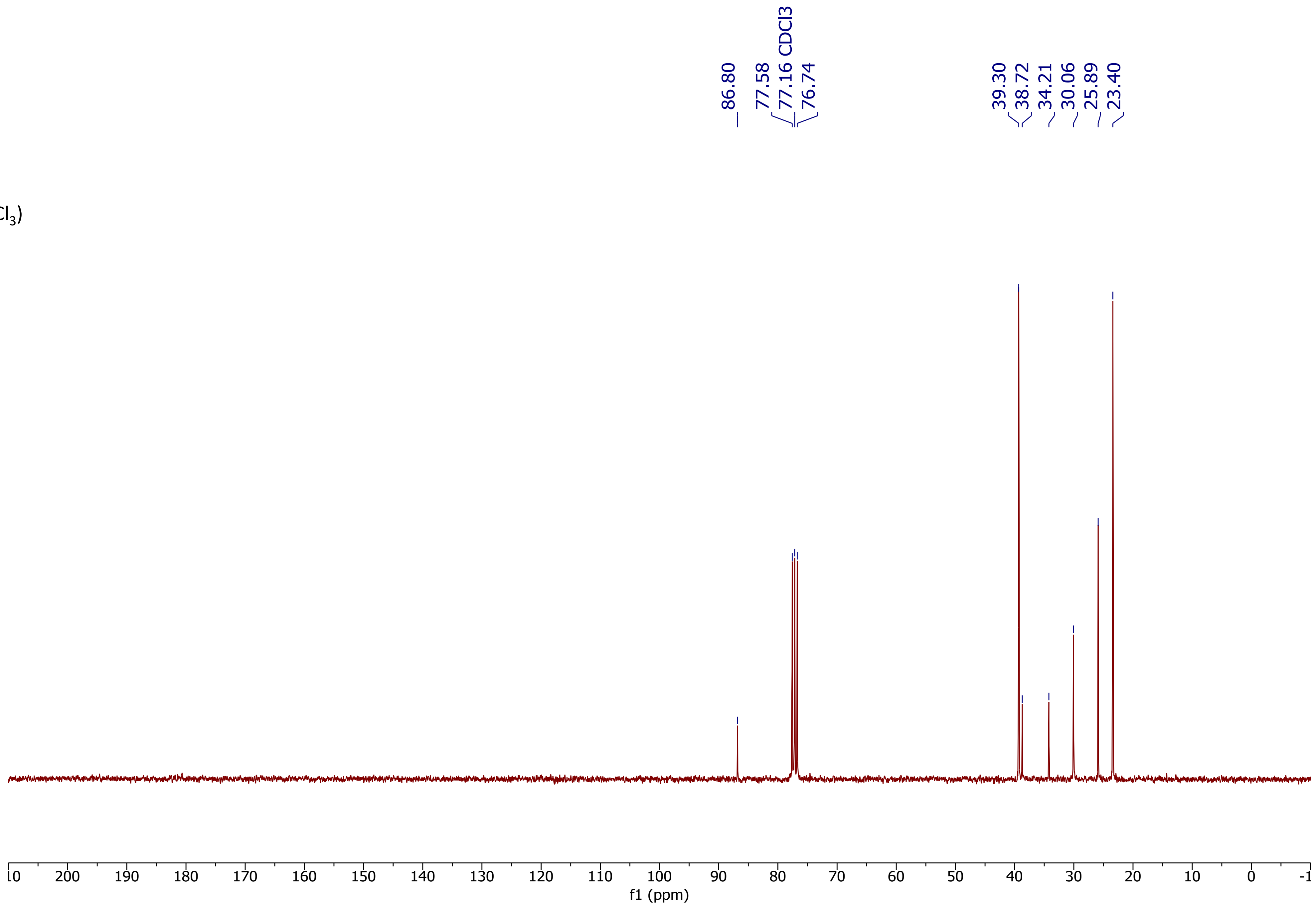

***cis*-1c**

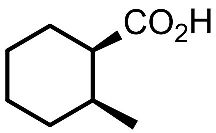

cis-1c-CO_2H

-crude-

^{1}H NMR(300 MHz, $CDCl_3$)

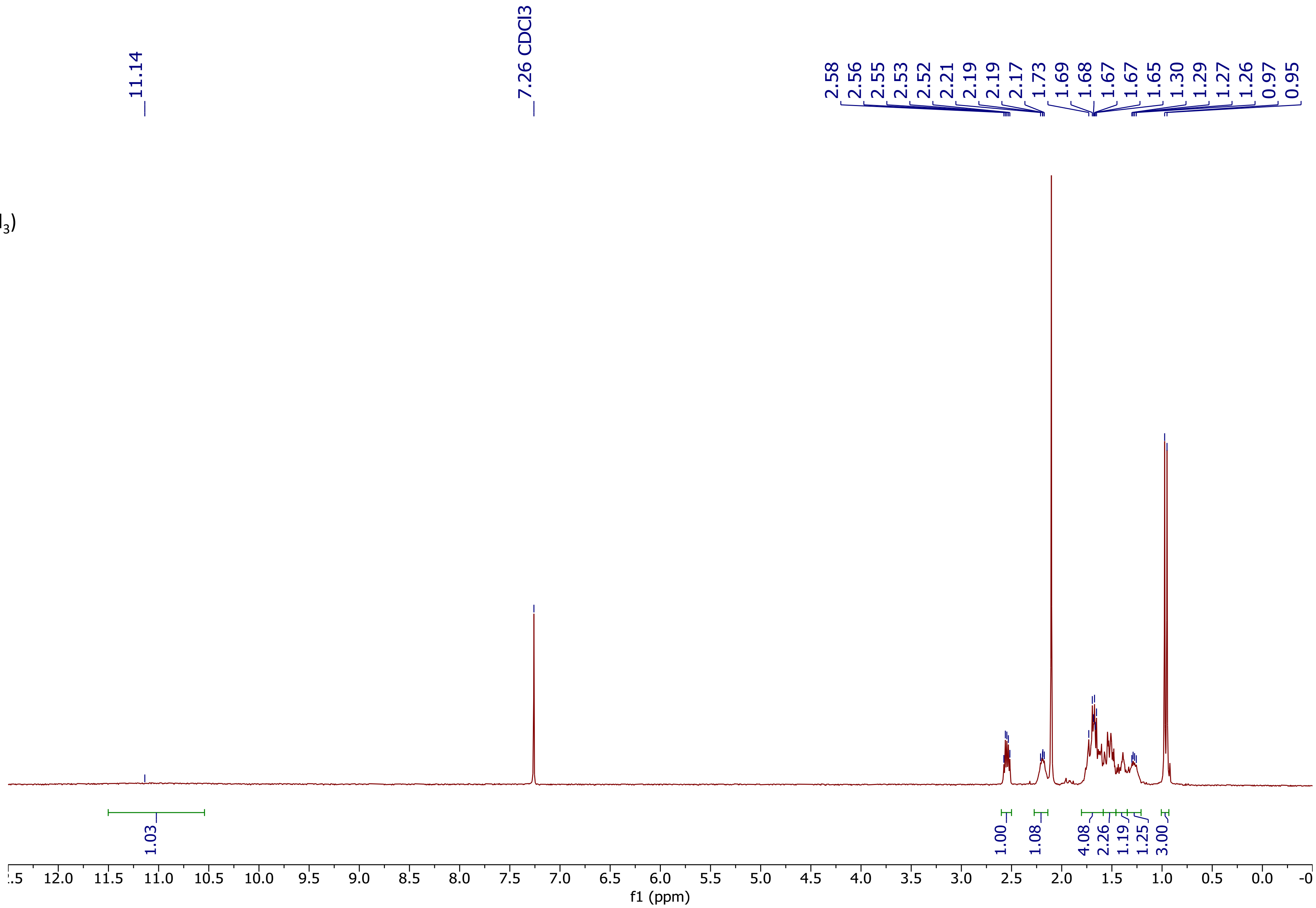

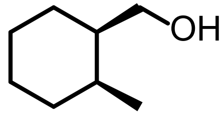

cis-1c-OH

-crude-

^{1}H NMR(300 MHz, $CDCl_3$)

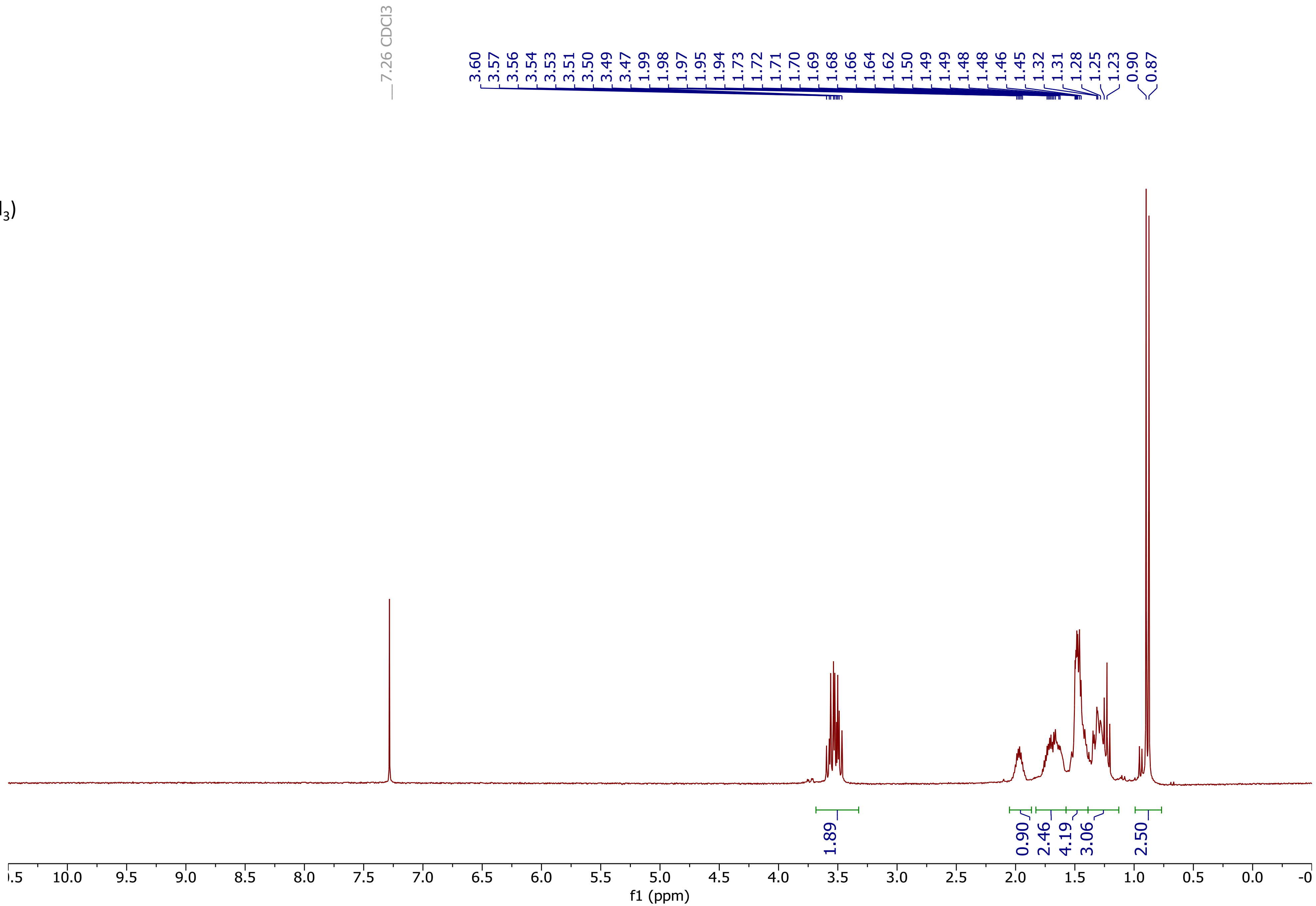

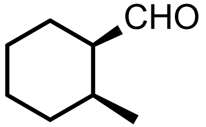

cis-1c-CHO

-crude-

^{1}H NMR(300 MHz, $CDCl_3$)

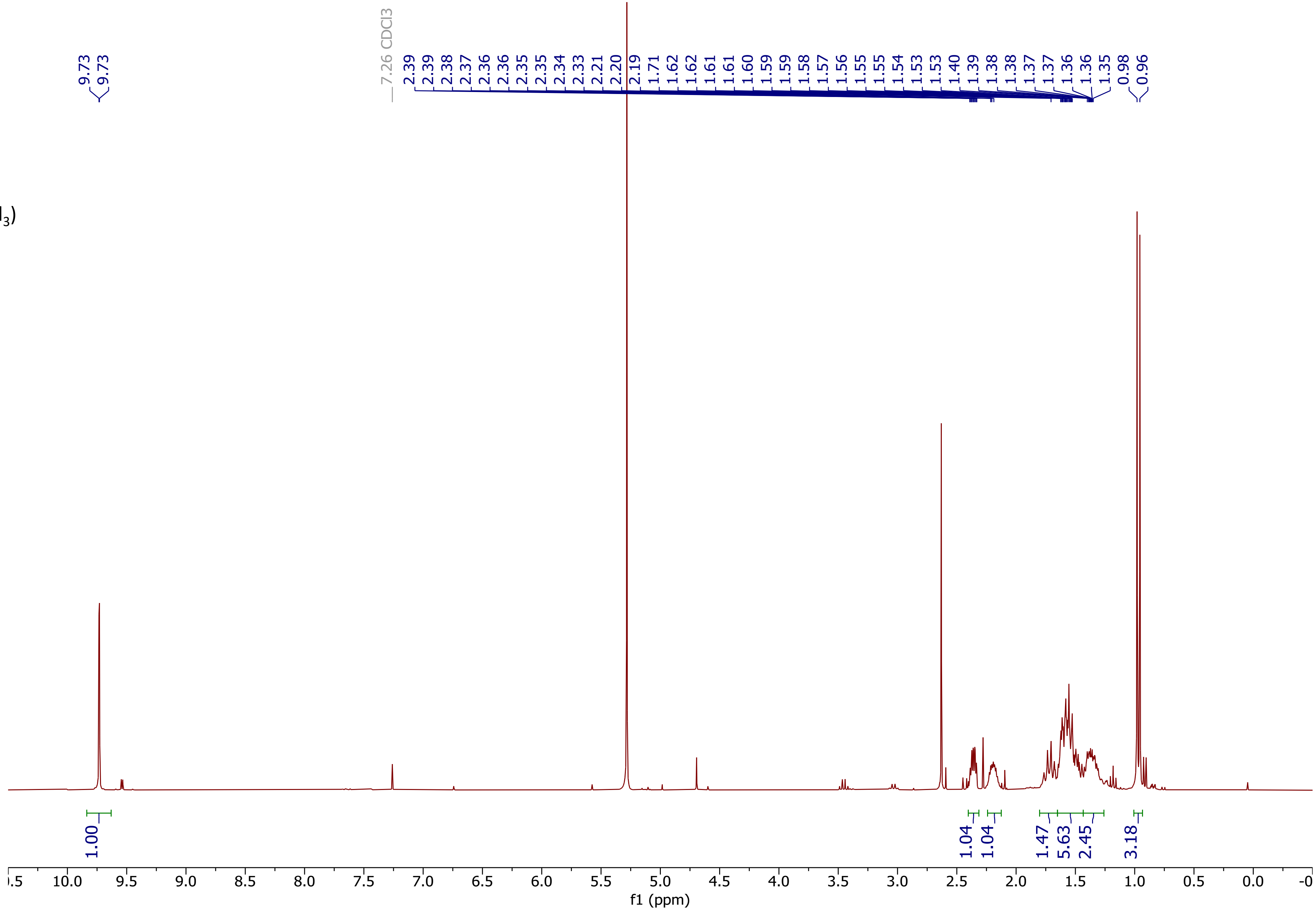

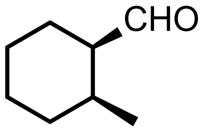

cis-1c-CHO

-crude-

^{13}C NMR (75 MHz, $CDCl_3$)

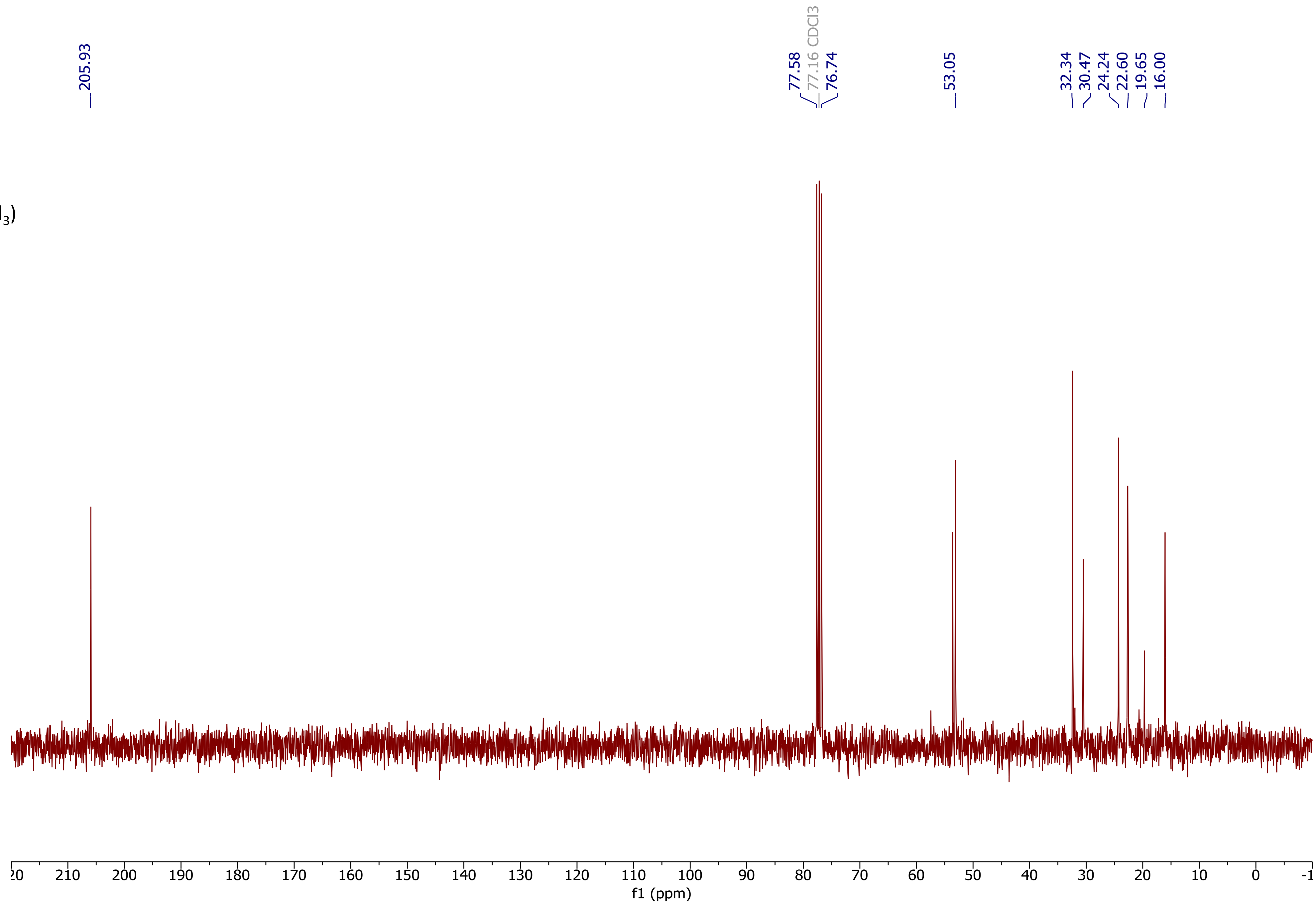

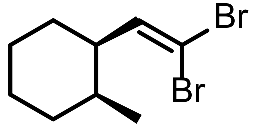

cis-1c-CBr_2

^{1}H NMR(300 MHz, $CDCl_3$)

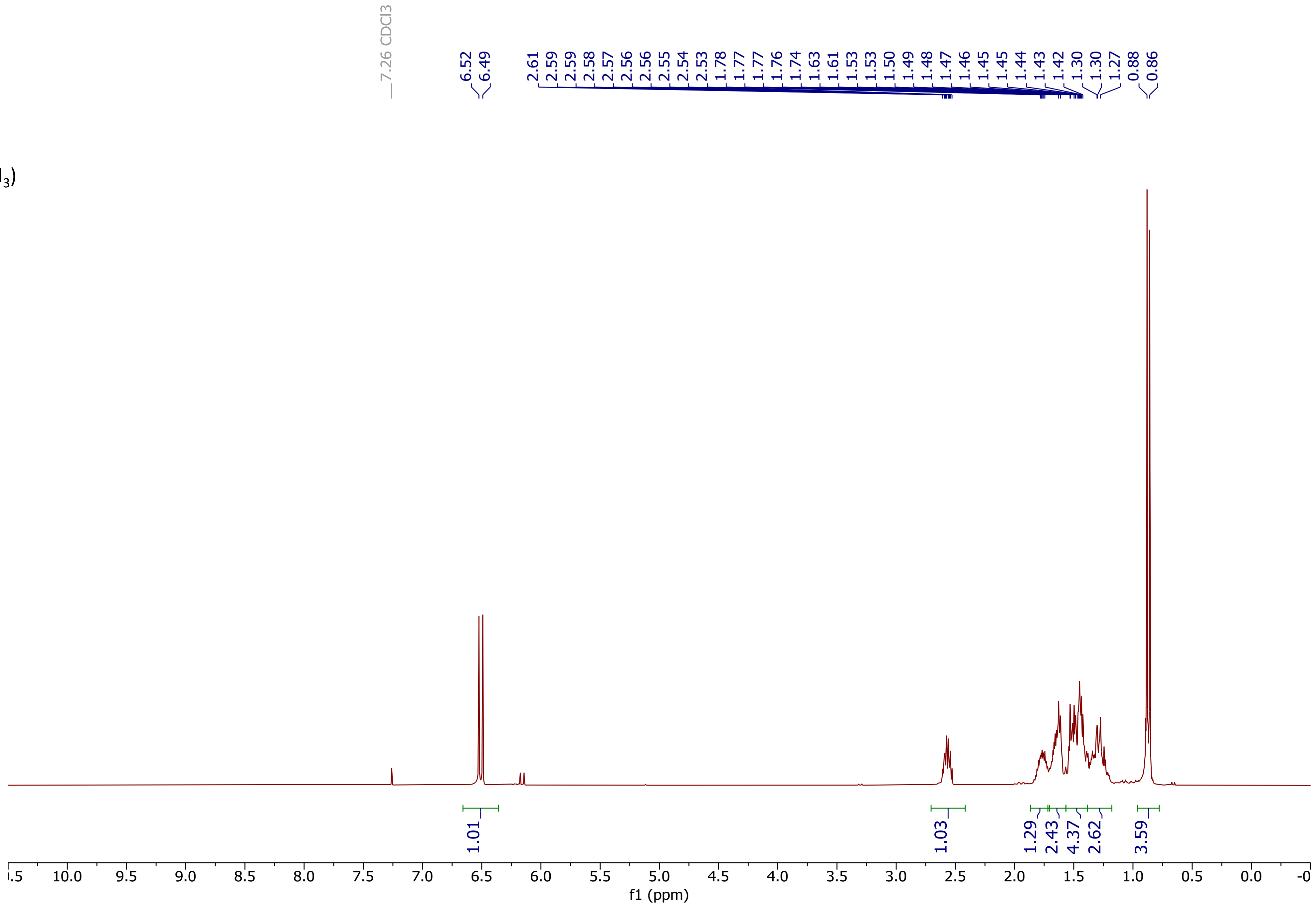

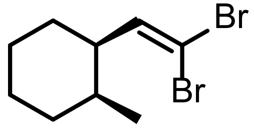

cis-1c-CBr$_2$

^{13}C NMR (75 MHz, $CDCl_3$)

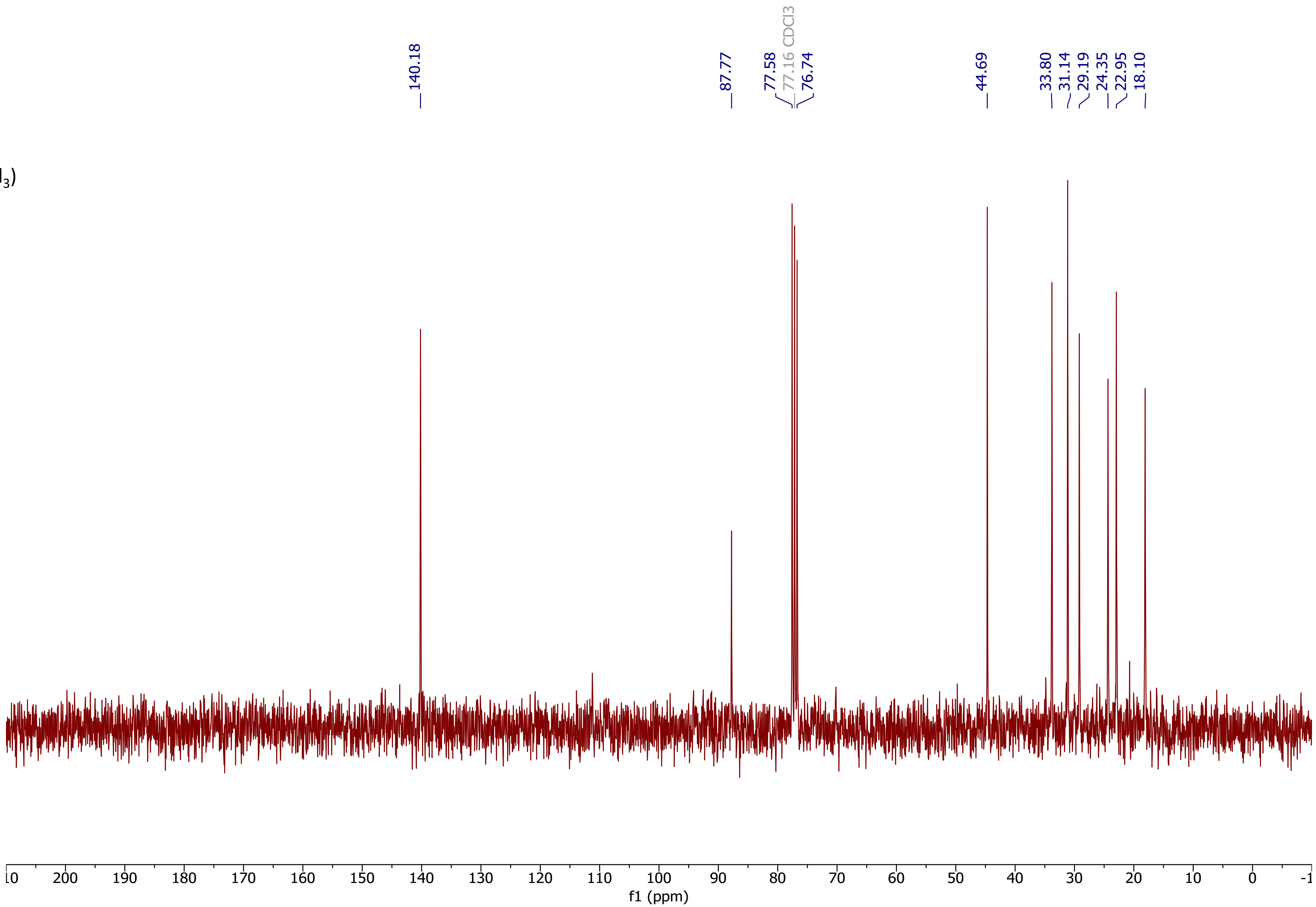

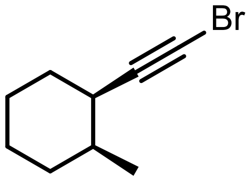

cis-1c

[1]**H NMR**(300 MHz, $CDCl_3$)

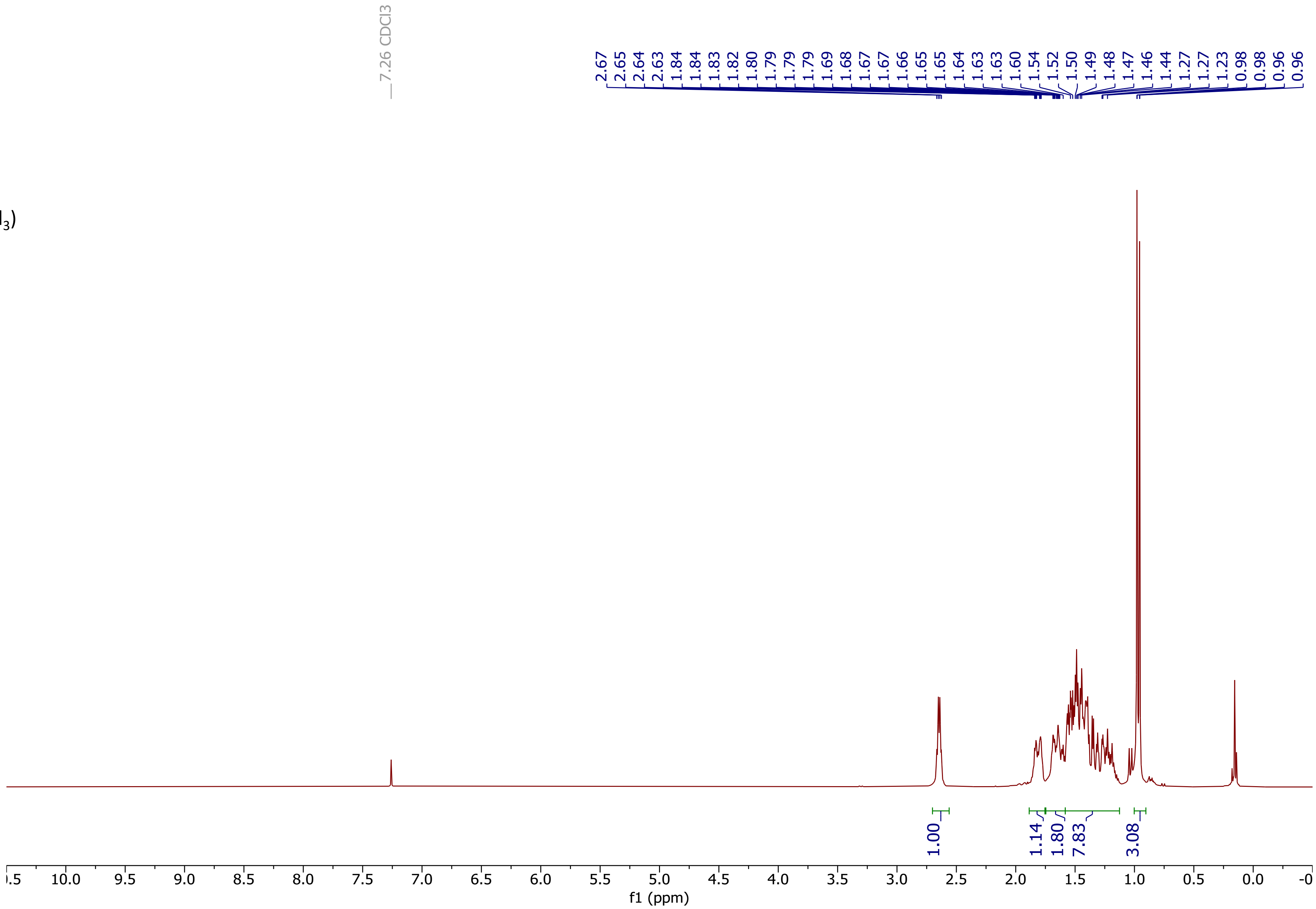

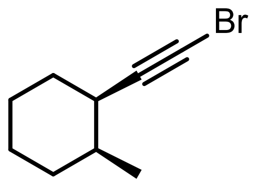

cis-1c

^{13}C NMR (75 MHz, $CDCl_3$)

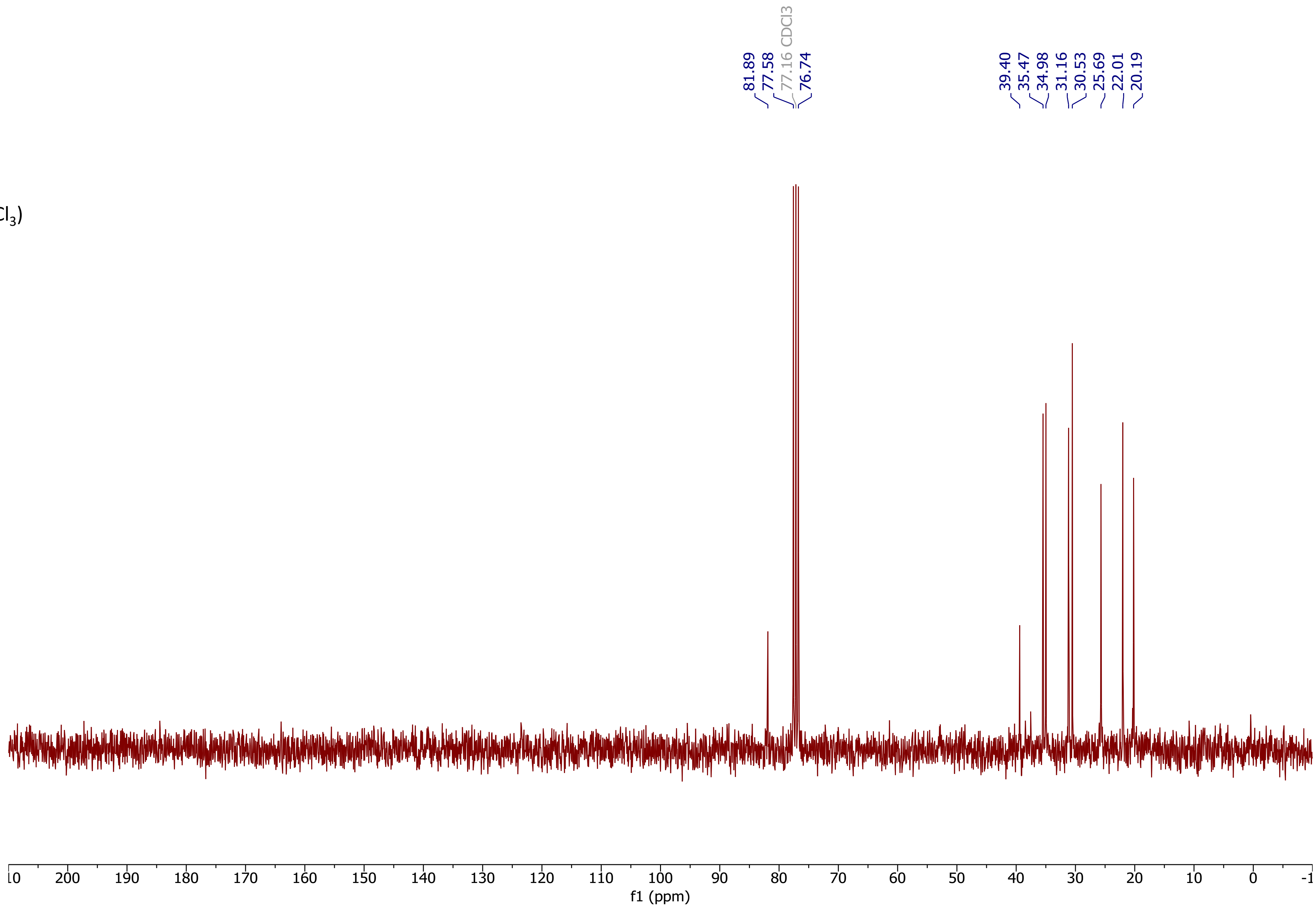

***trans*-1c**

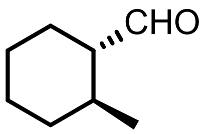

trans-1c-CHO

-crude-

^{1}H NMR(300 MHz, $CDCl_3$)

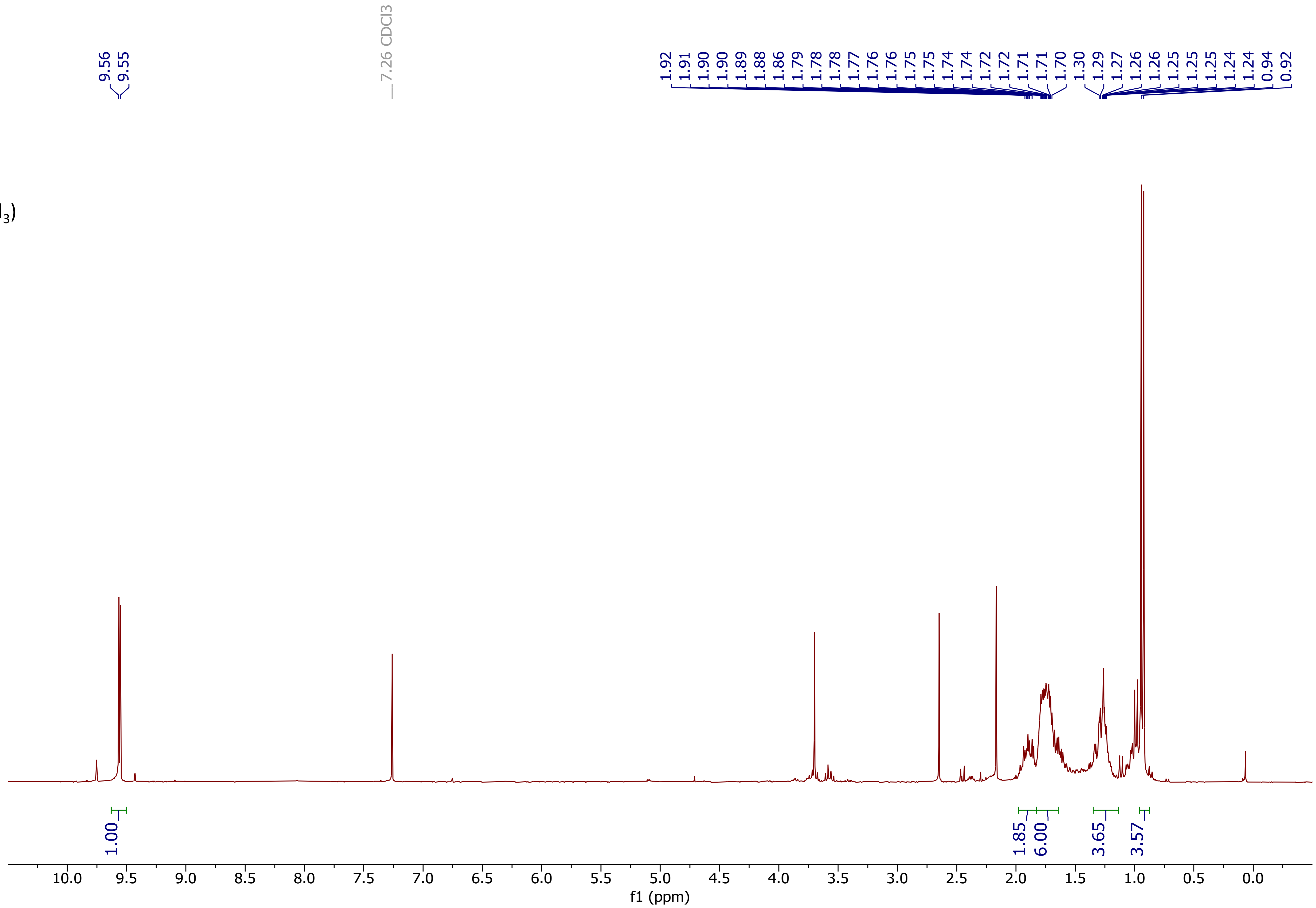

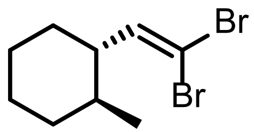

trans-1c-CBr$_2$

^{1}H NMR(300 MHz, $CDCl_3$)

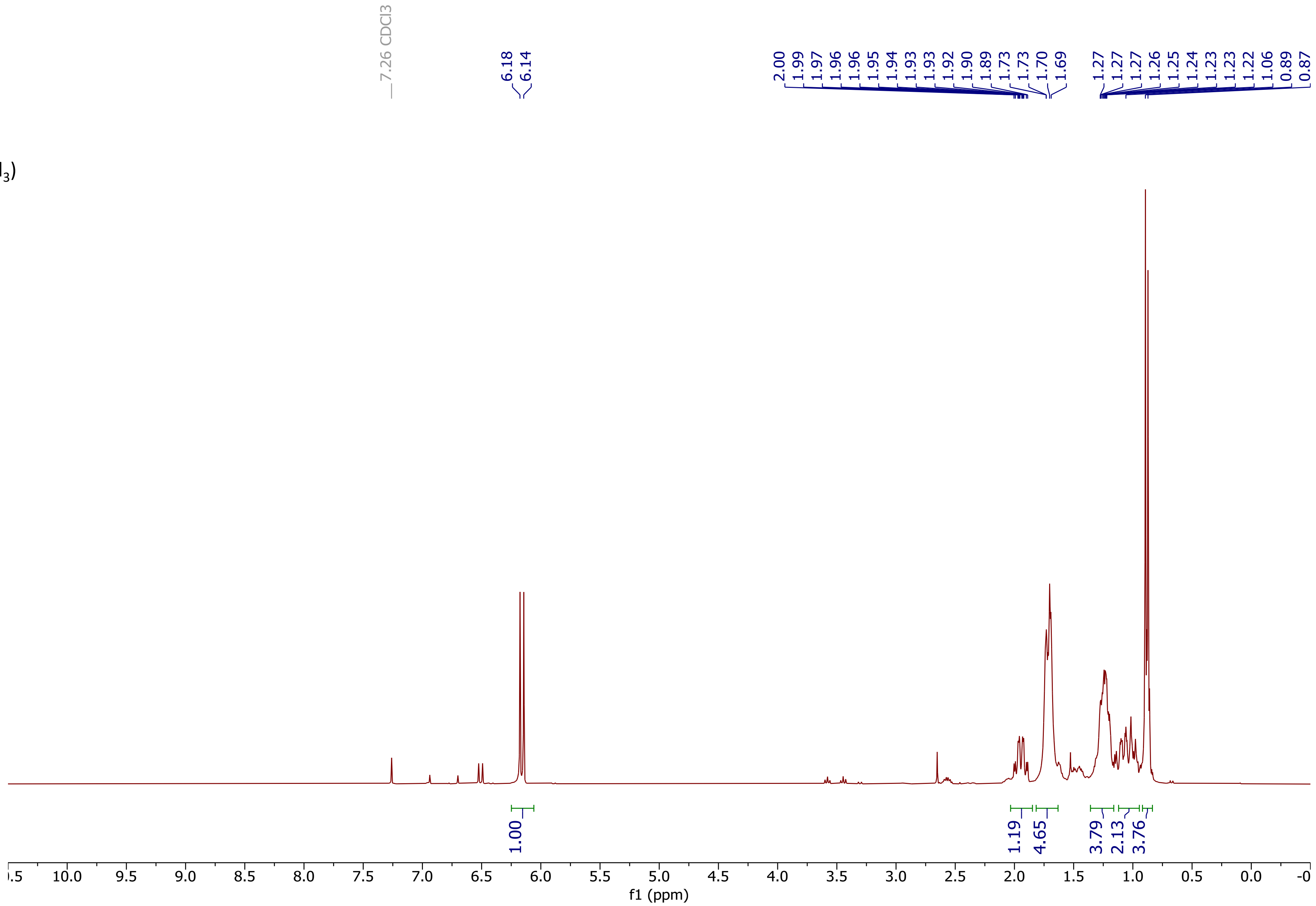

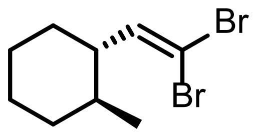

trans-1c-CBr$_2$

^{13}C NMR (75 MHz, $CDCl_3$)

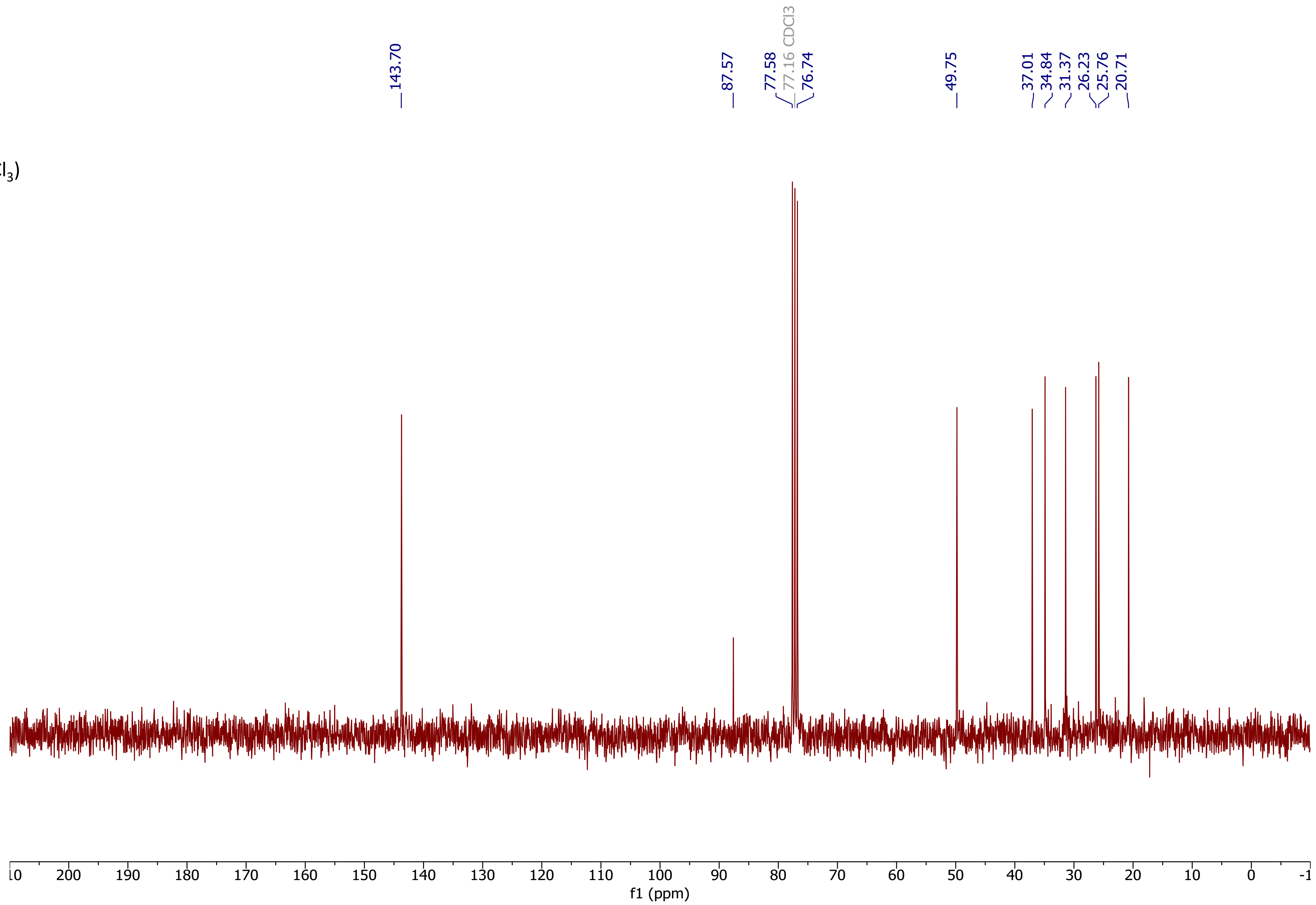

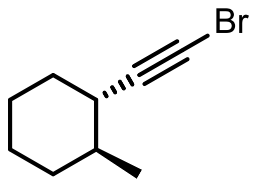

trans-1c

^{1}H NMR(300 MHz, $CDCl_3$)

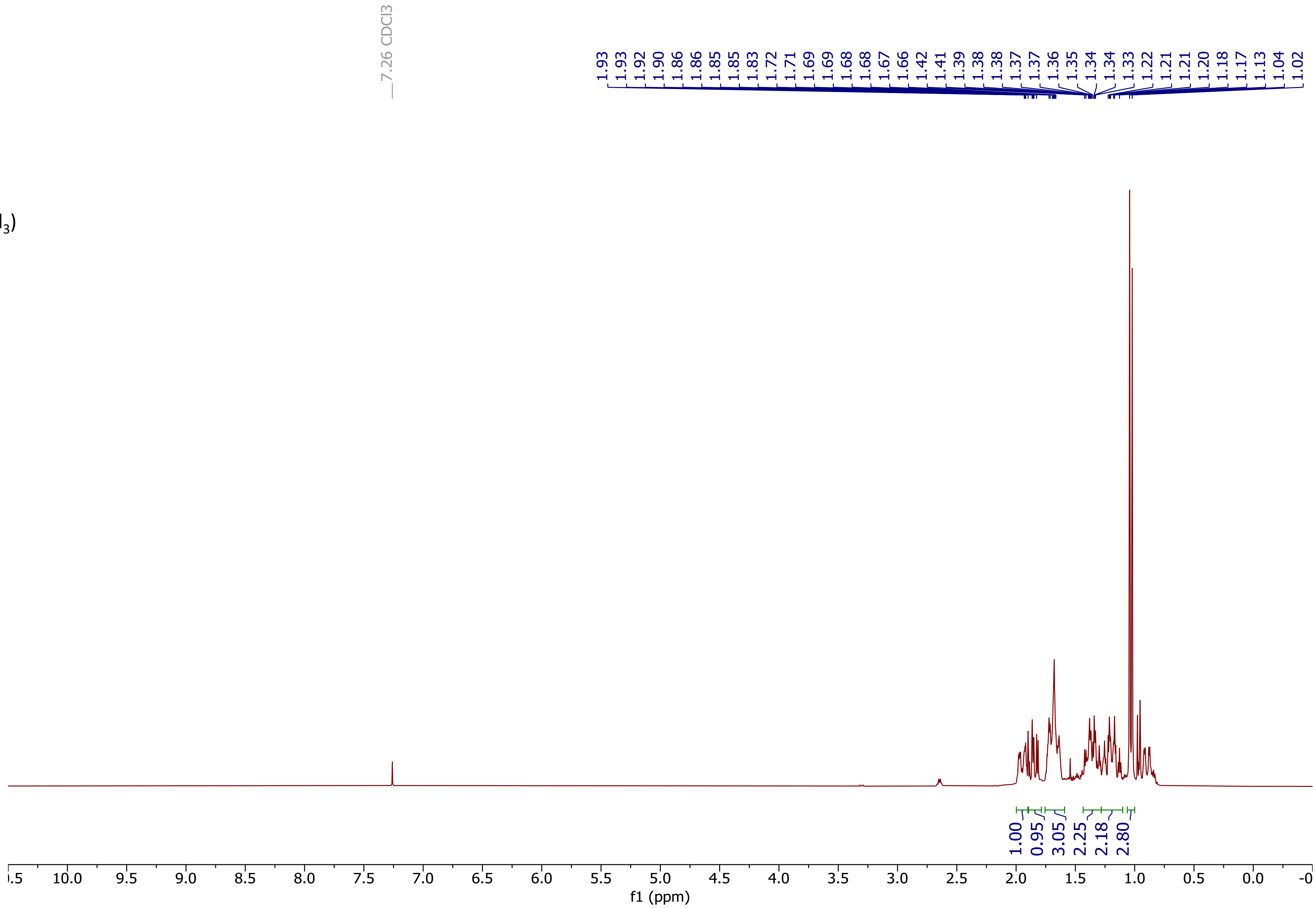

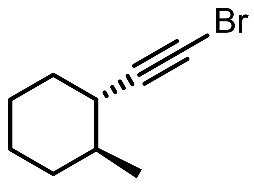

trans-1c

^{13}C NMR (75 MHz, $CDCl_3$)

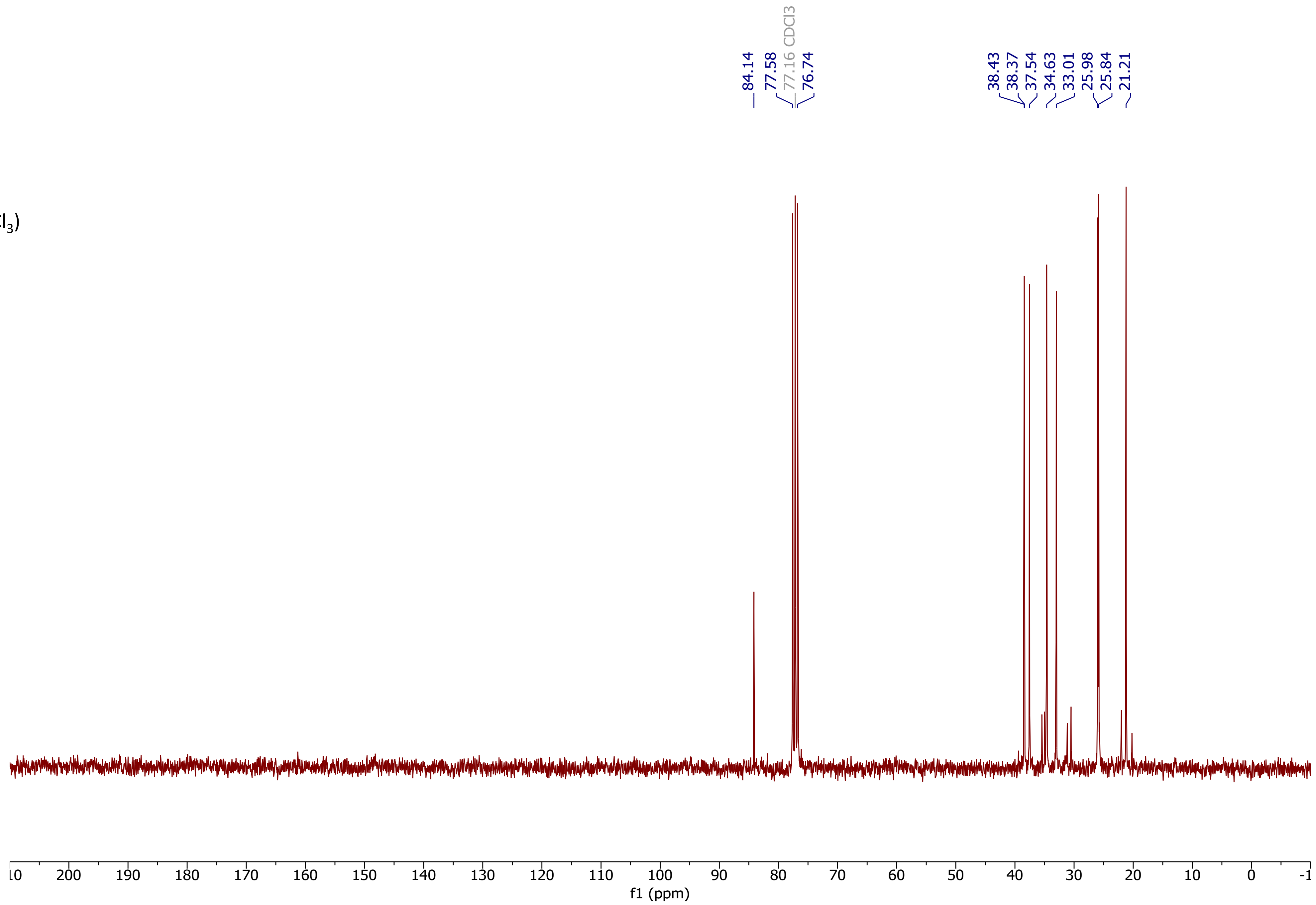

***cis*-1d**

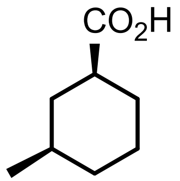

***cis*-1d-CO_2H**

^{1}H NMR(300 MHz, $CDCl_3$)

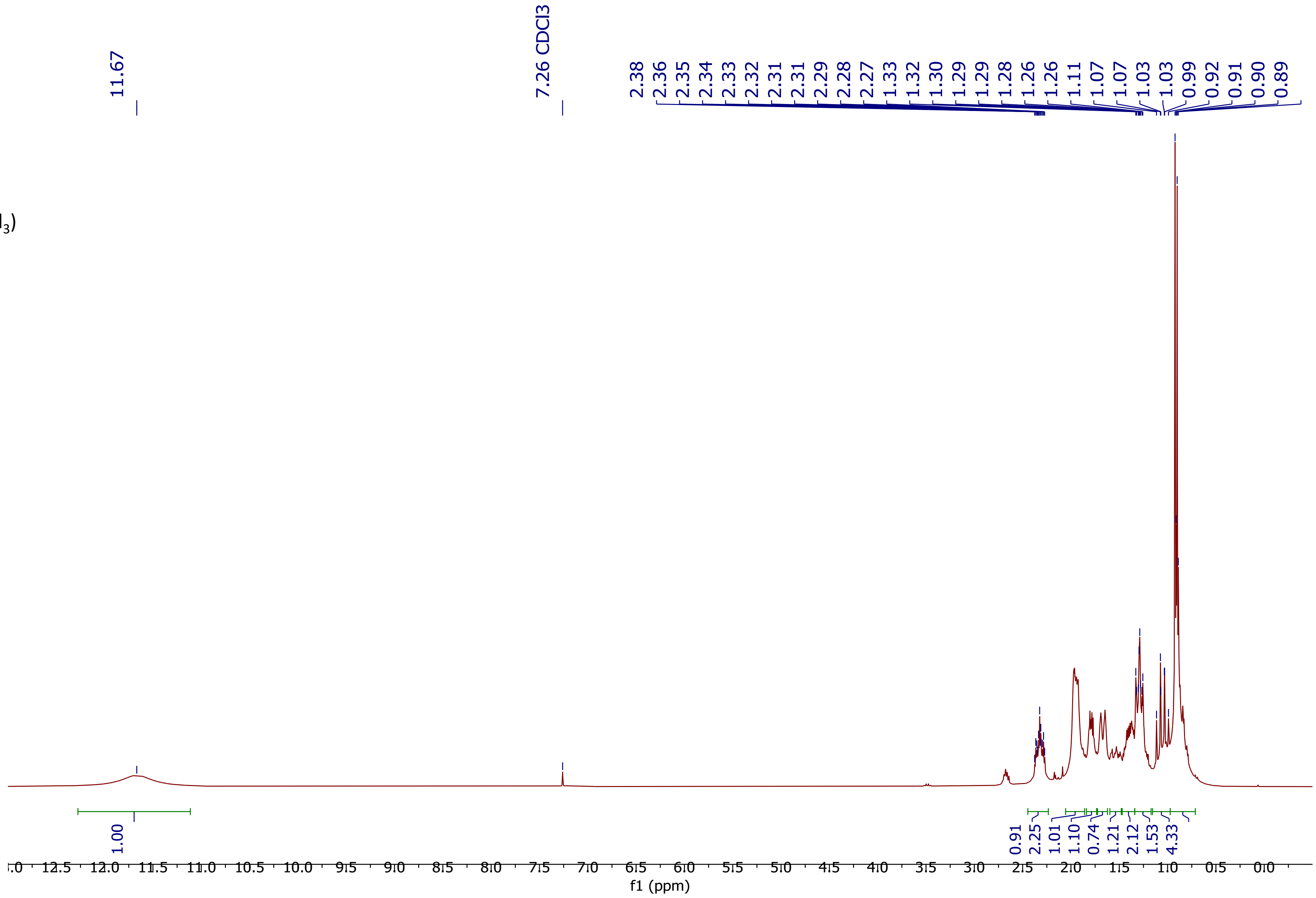

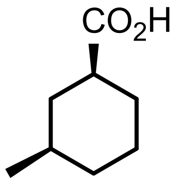

***cis*-1d-CO_2H**

^{13}C NMR (75 MHz, $CDCl_3$)

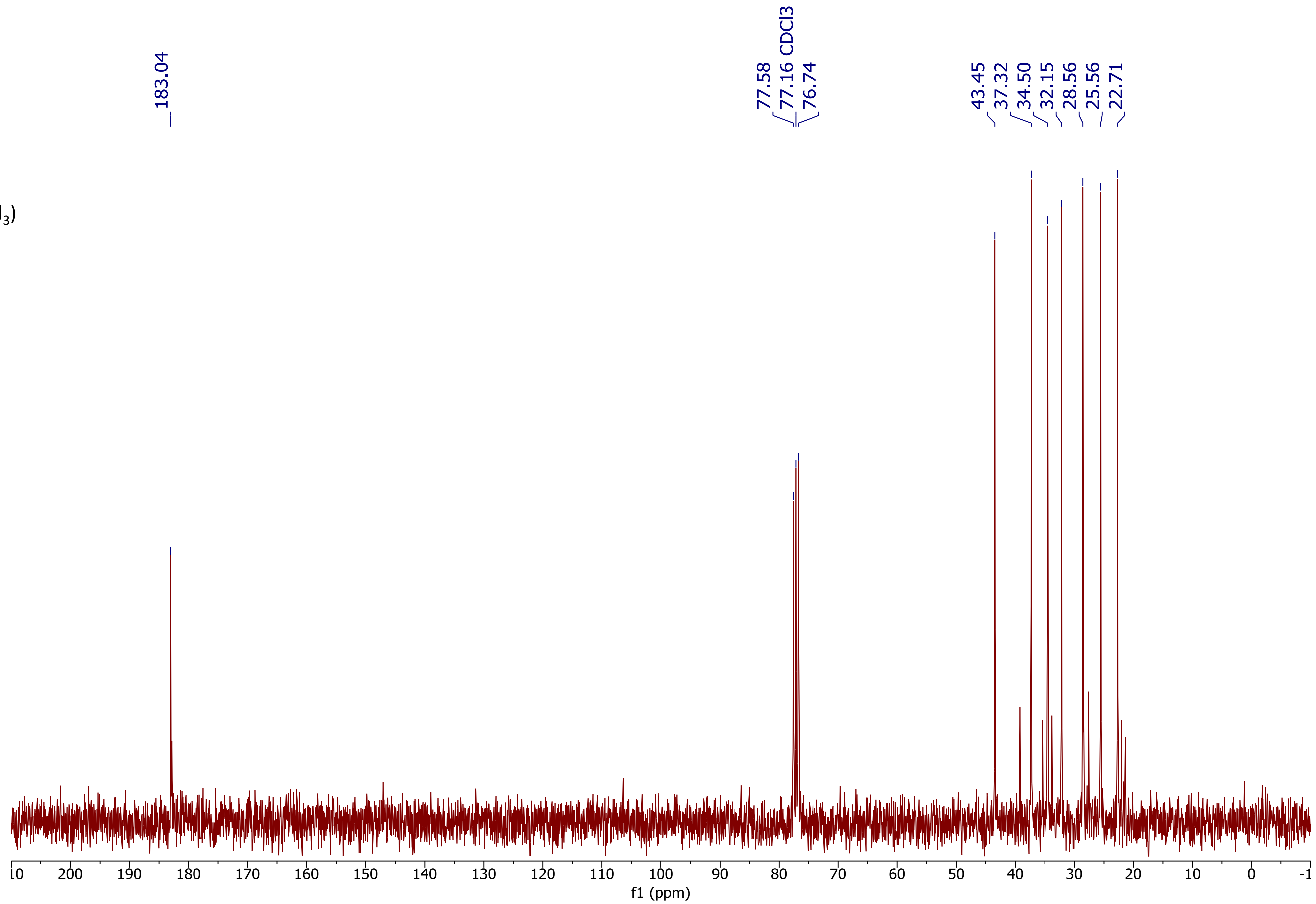

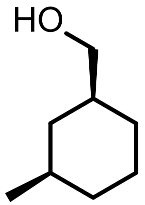

***cis*-1d-OH**

-crude-

^{1}H NMR(300 MHz, $CDCl_3$)

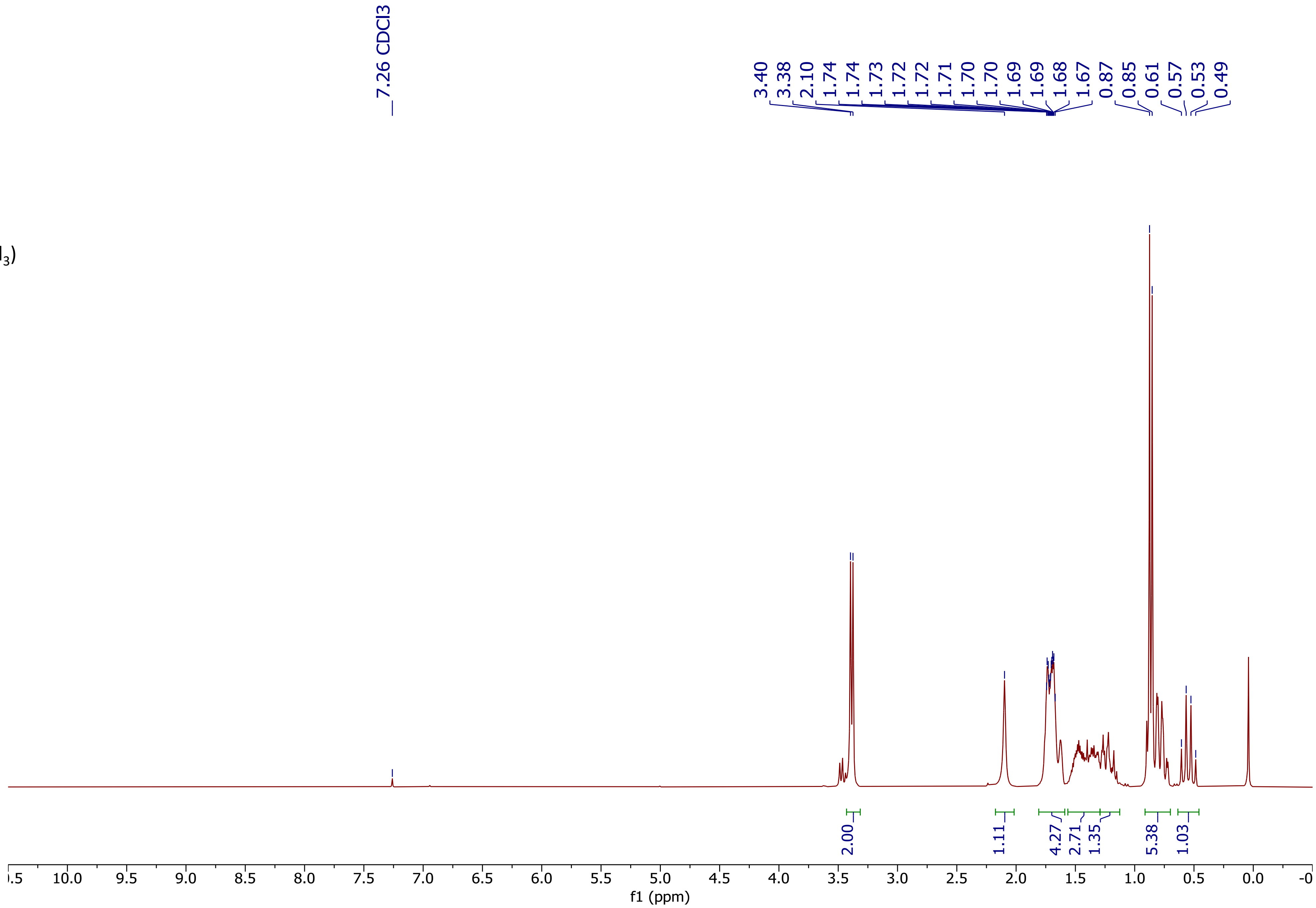

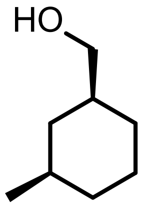

***cis*-1d-OH**

-crude-

^{13}C NMR (75 MHz, $CDCl_3$)

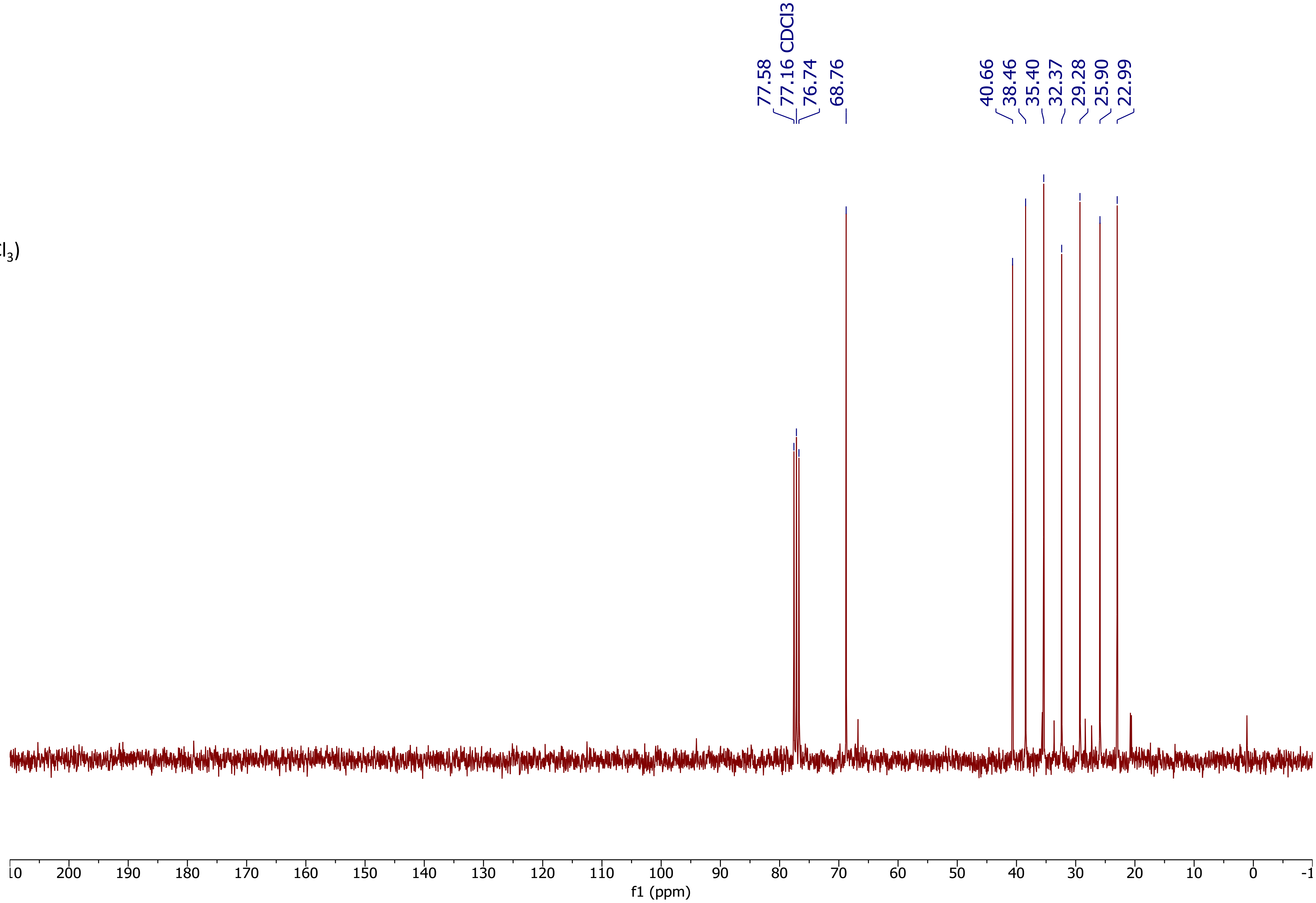

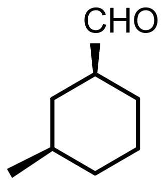

***cis*-1d-CHO**

-crude-

^{1}H NMR(300 MHz, $CDCl_3$)

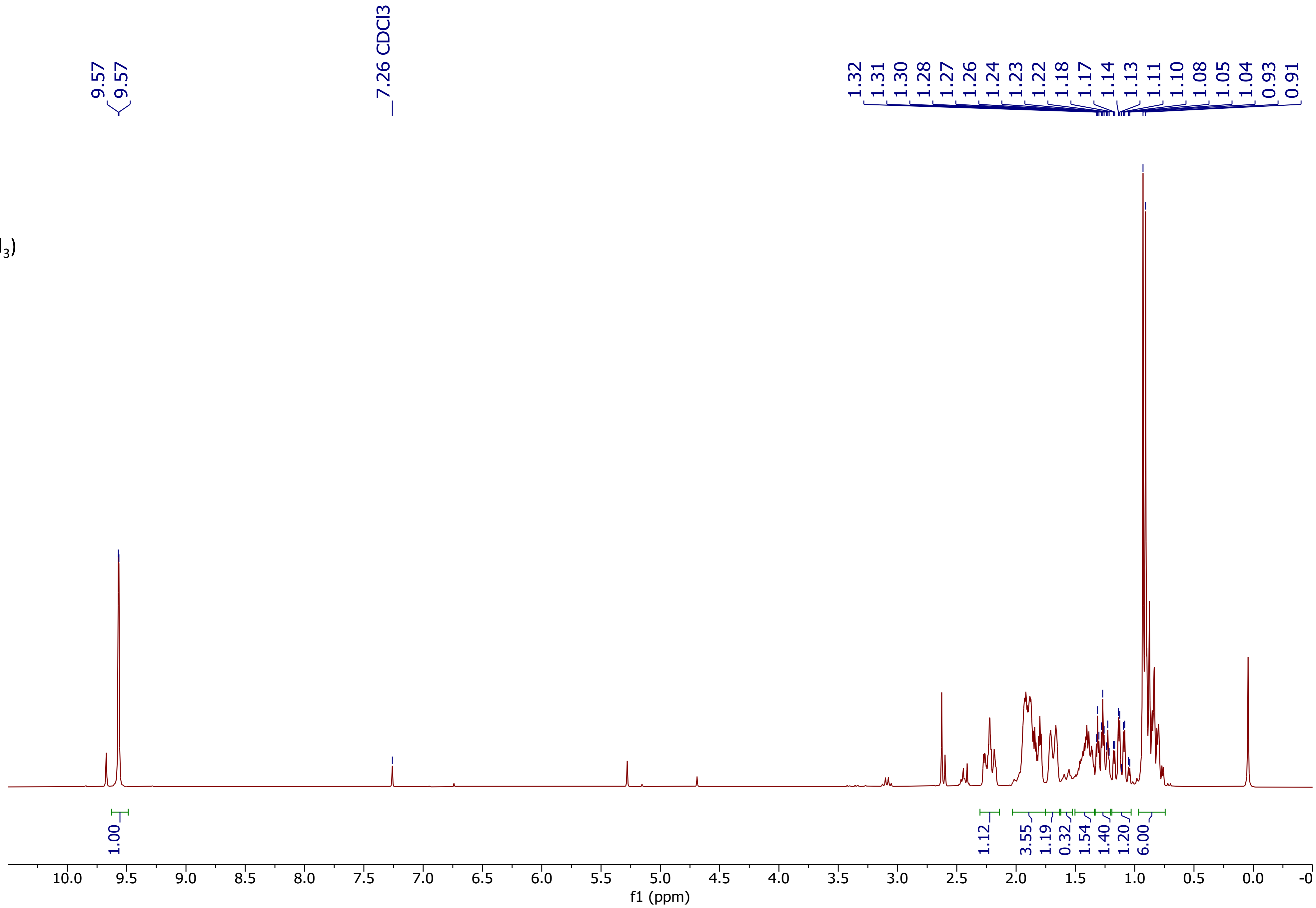

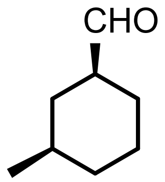

***cis*-1d-CHO**

-crude-

^{13}C NMR (75 MHz, $CDCl_3$)

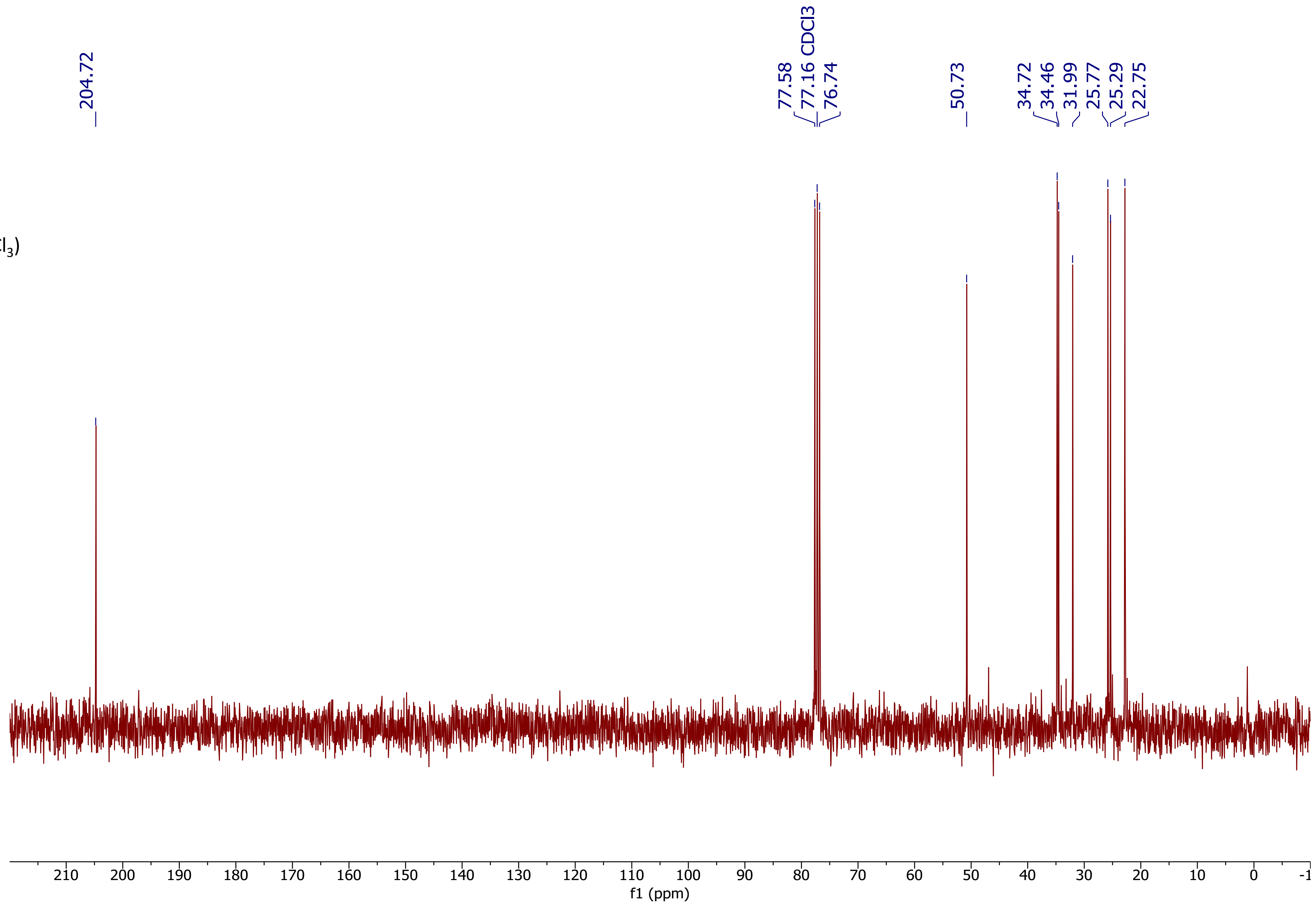

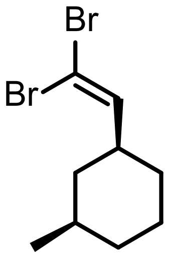

cis-1d-CBr_2

^{1}H NMR(300 MHz, $CDCl_3$)

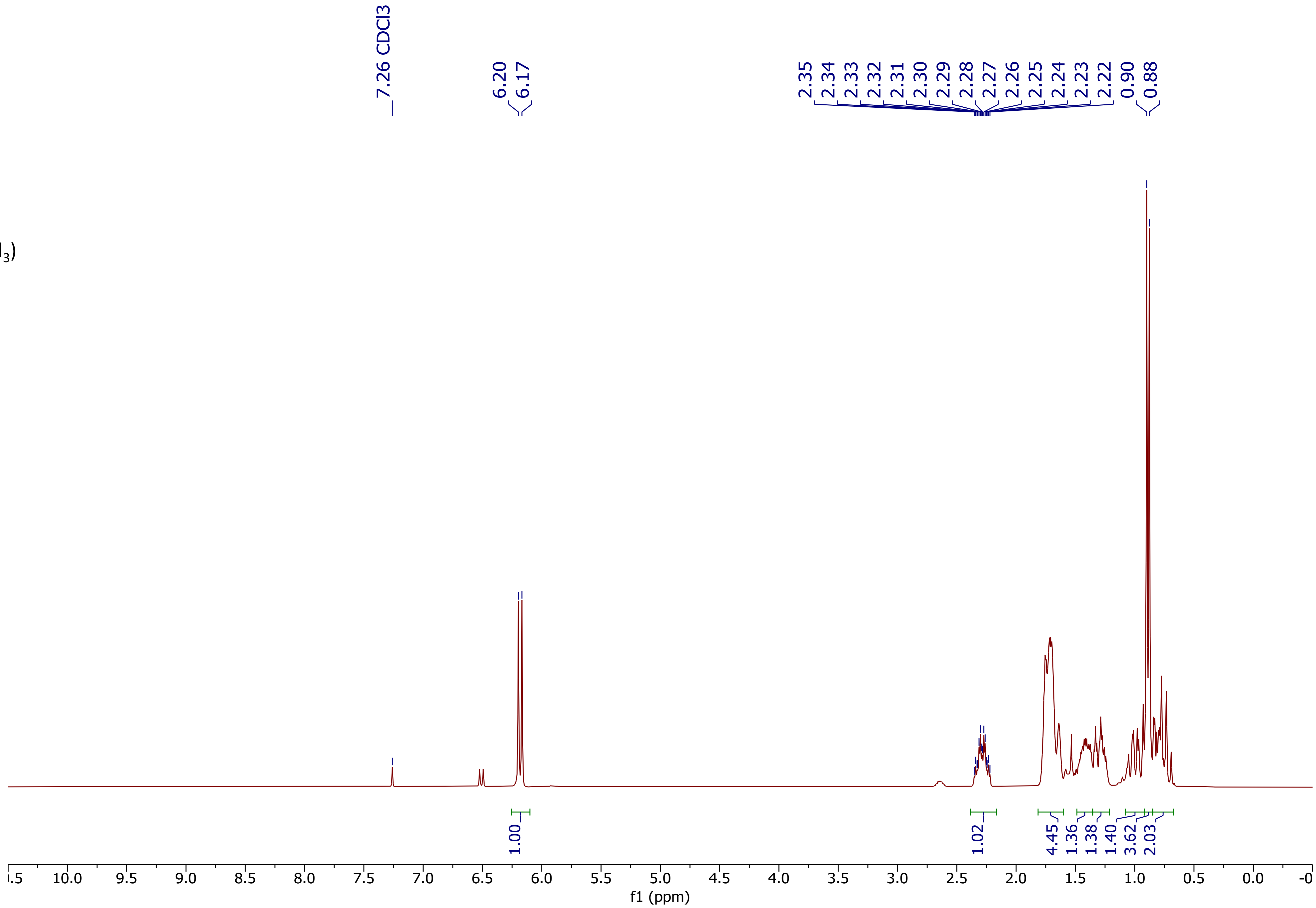

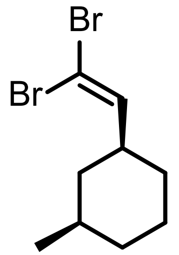

***cis*-1d-CBr$_2$**

^{13}C NMR (75 MHz, $CDCl_3$)

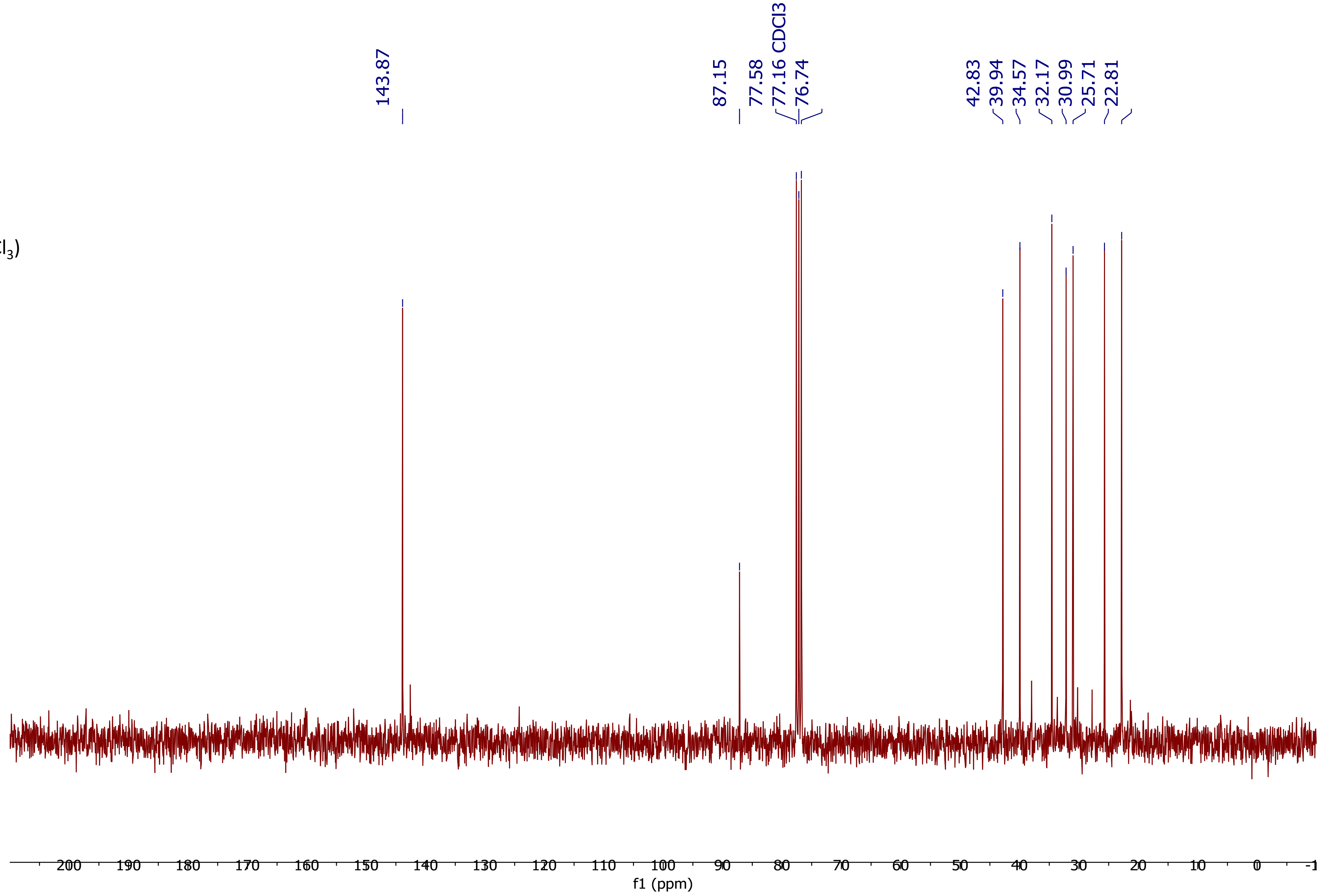

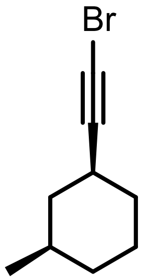

***cis*-1d**

^{1}H NMR(300 MHz, $CDCl_3$)

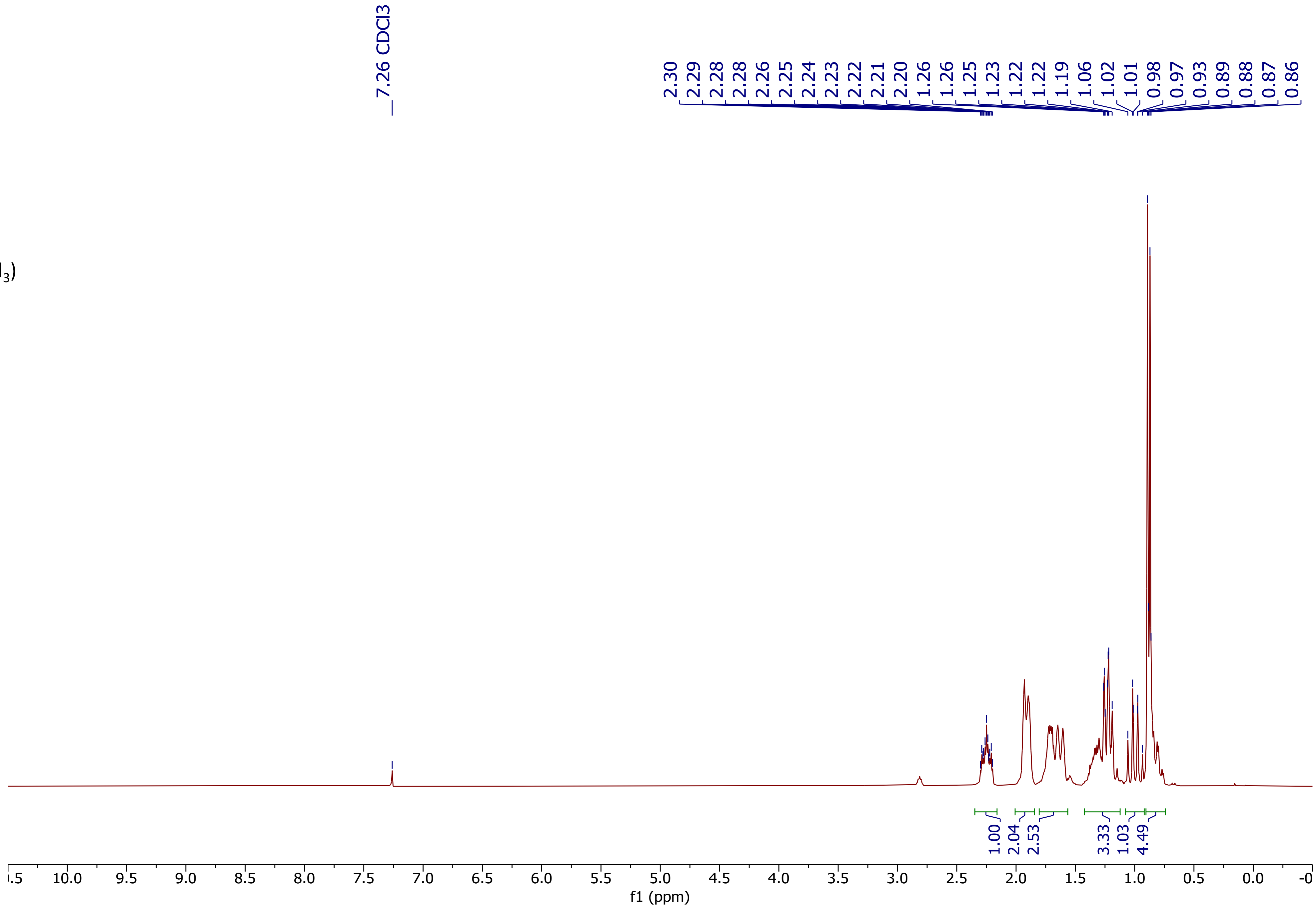

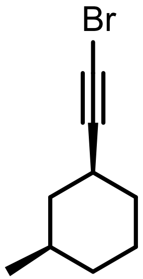

***cis*-1d**

^{13}C NMR (75 MHz, $CDCl_3$)

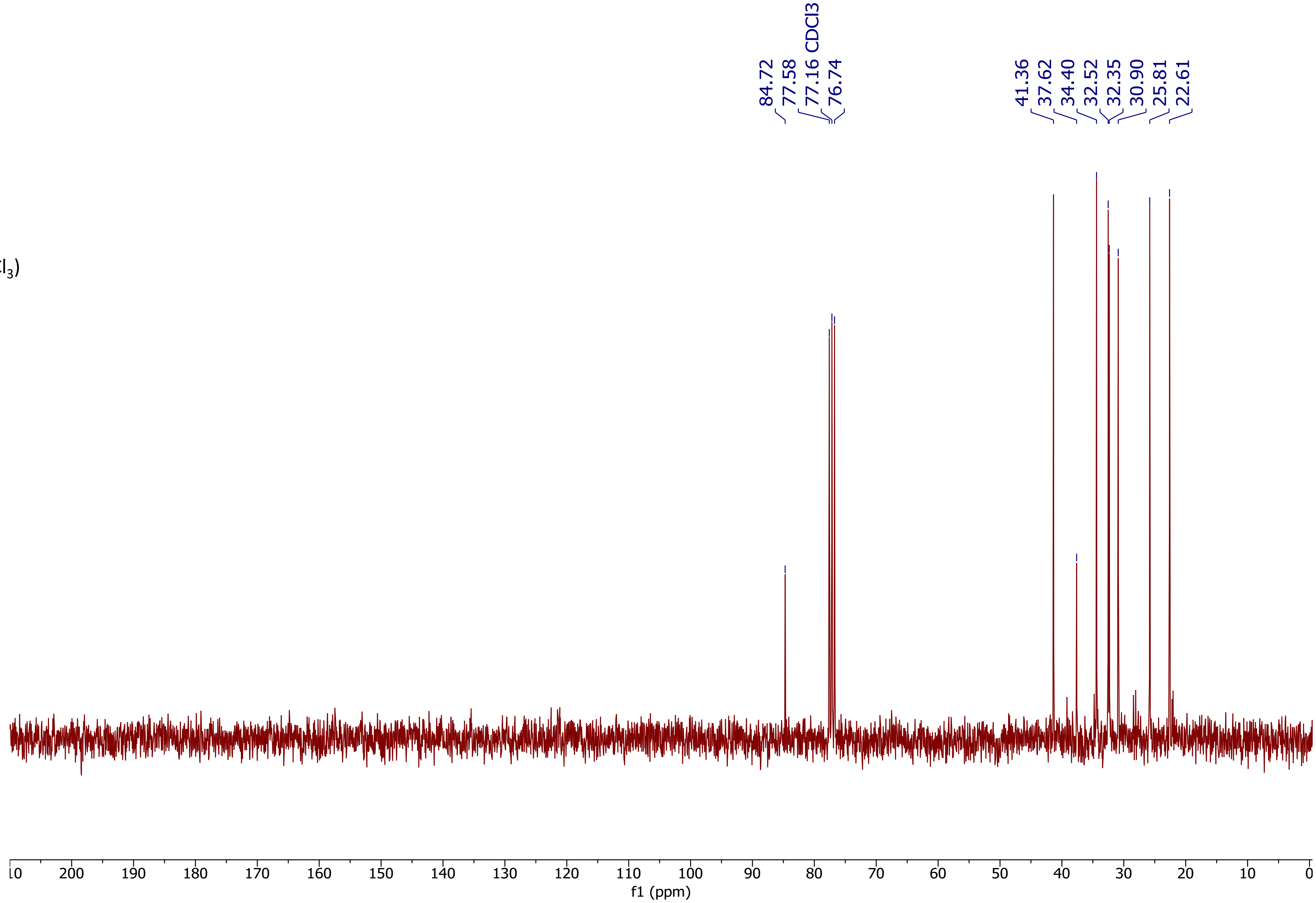

***trans*-1d**

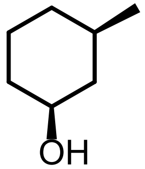

***cis*-1d-OH**

^{1}H NMR(300 MHz, $CDCl_3$)

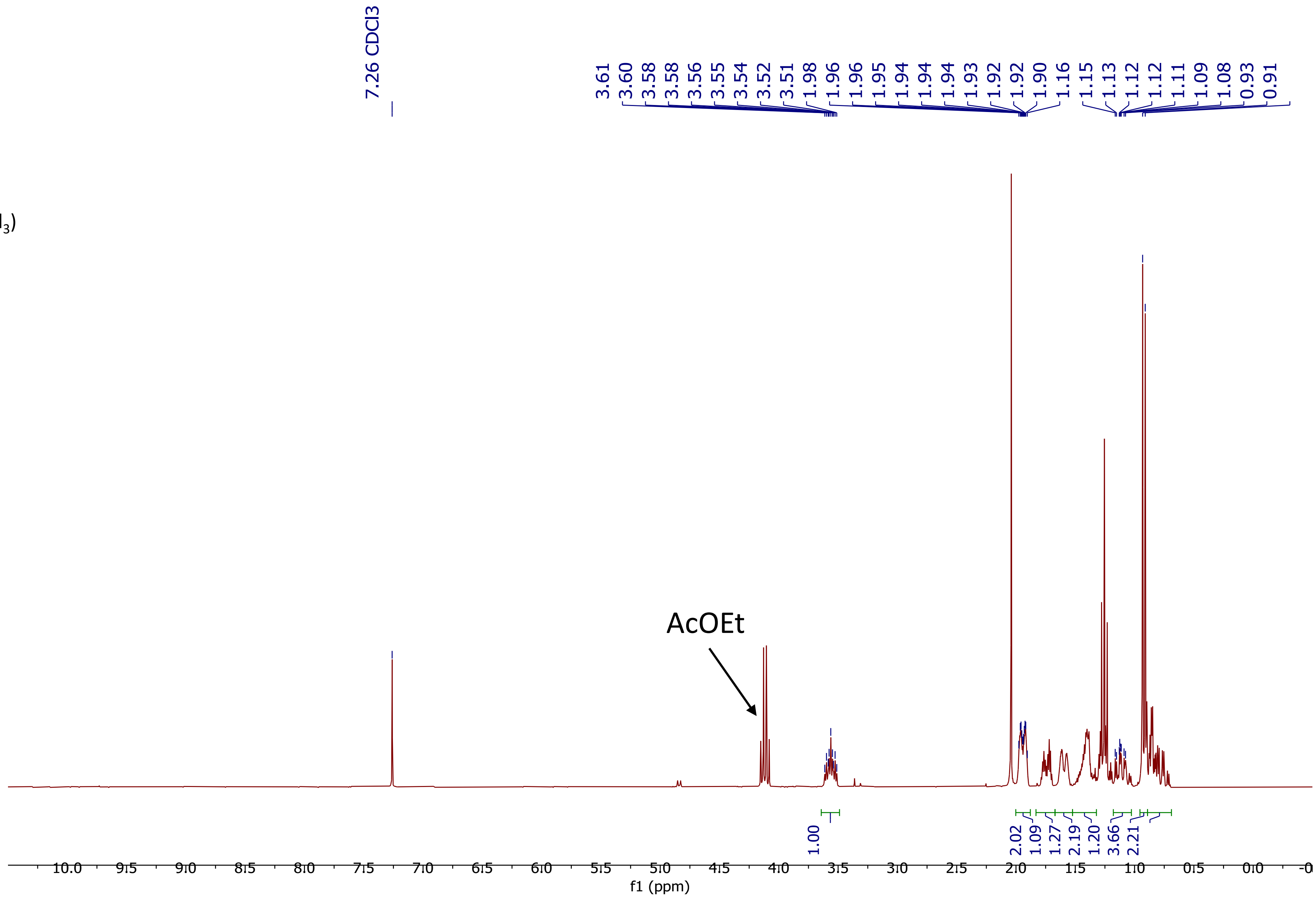

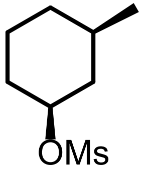

***cis*-1d-OMs**

-crude-

^{1}H NMR(300 MHz, $CDCl_3$)

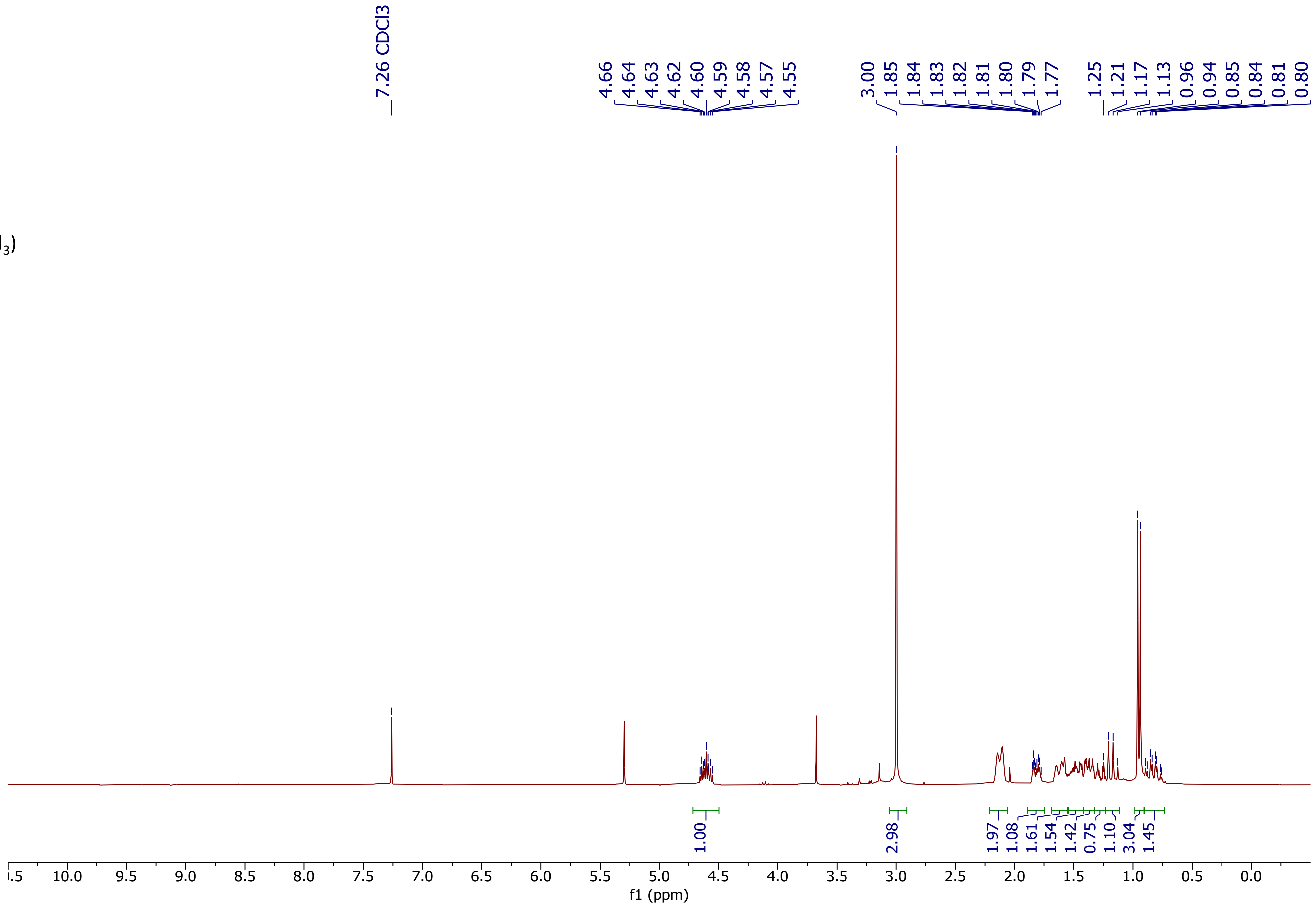

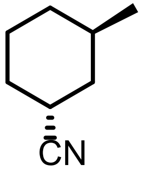

***trans*-1d-CN**

-crude-

^{1}H NMR(300 MHz, $CDCl_3$)

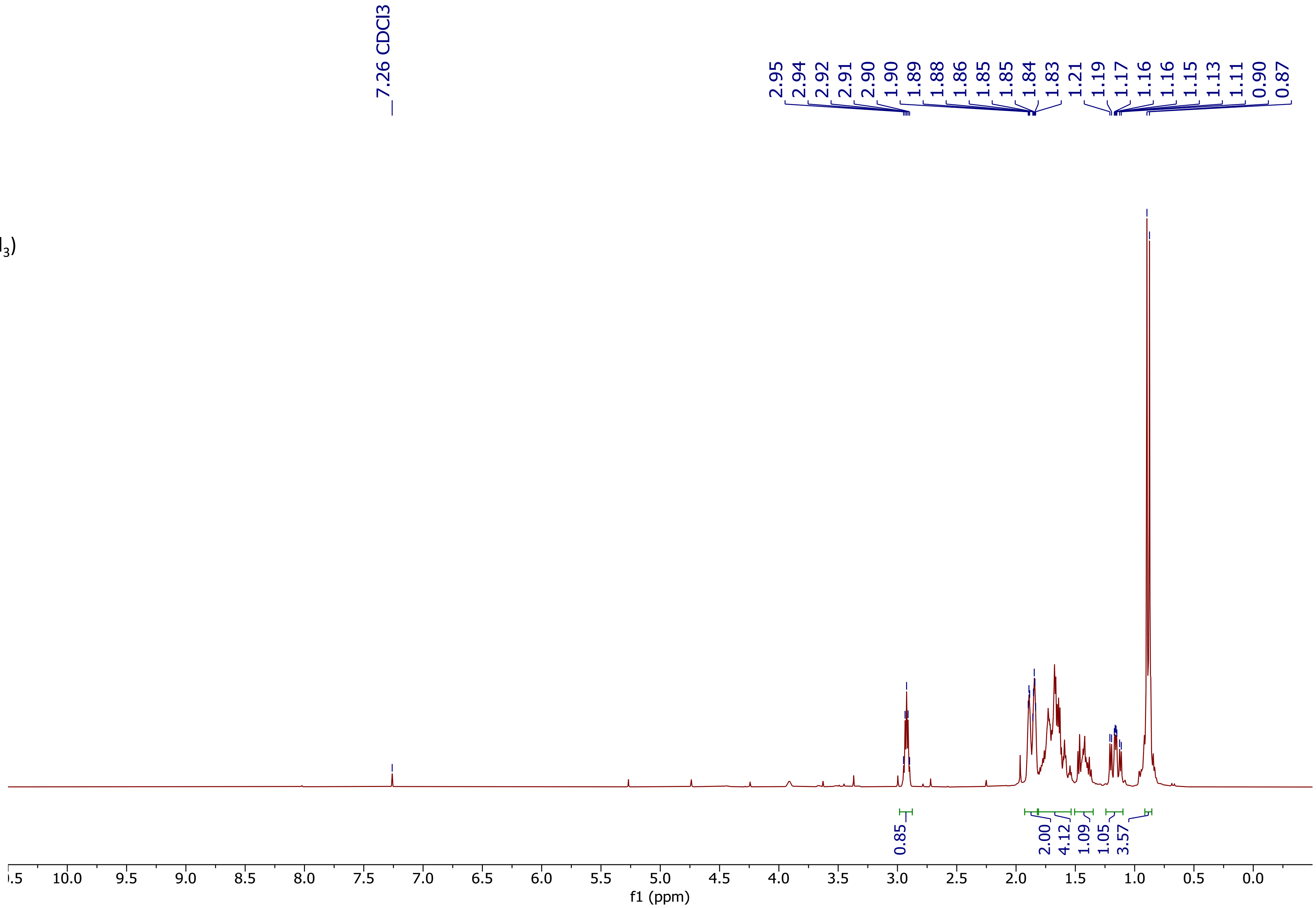

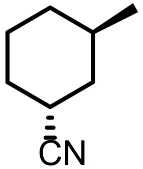

***trans*-1d-CN**

-crude-

^{13}C NMR (75 MHz, $CDCl_3$)

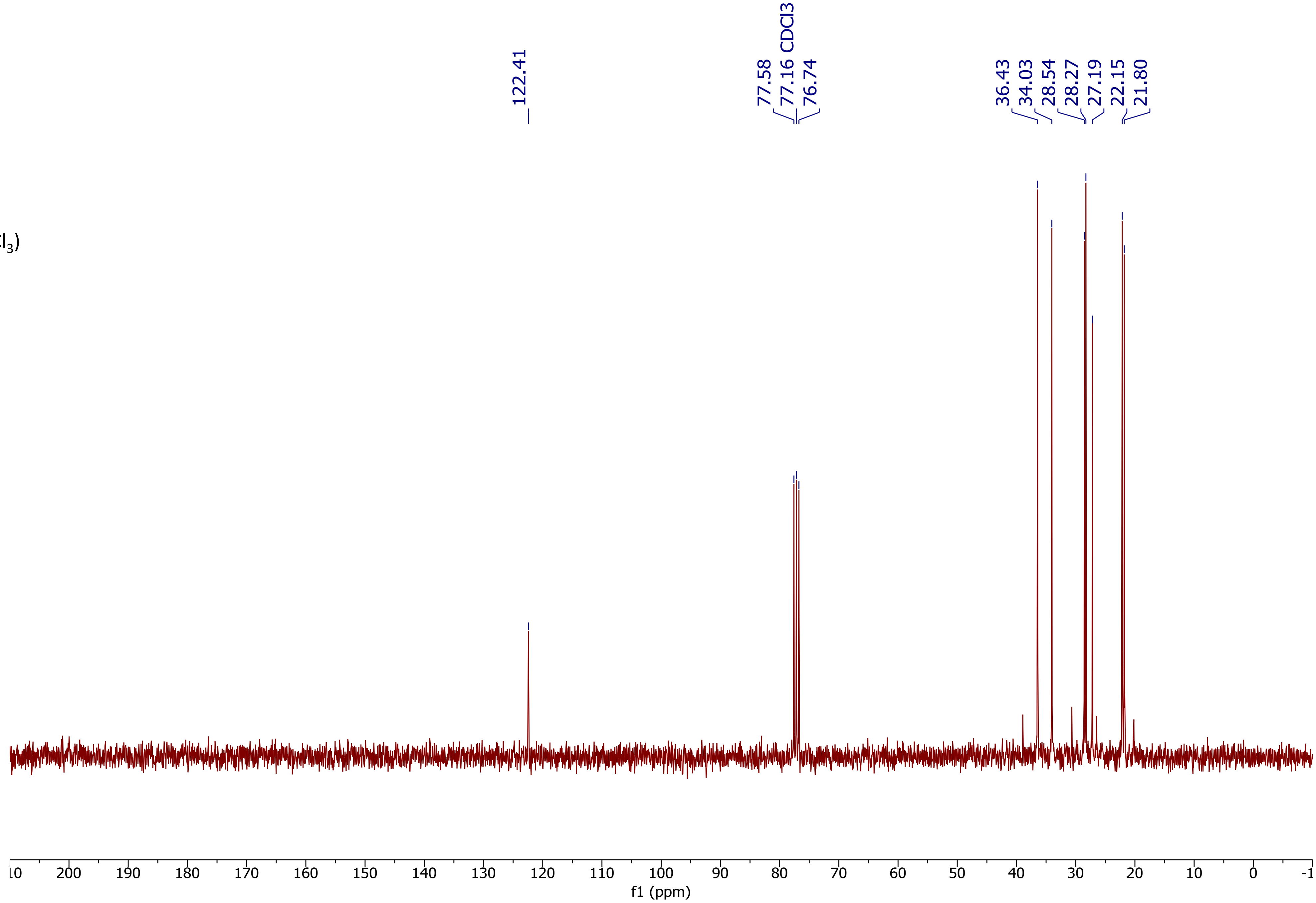

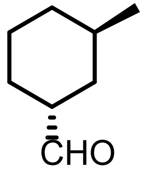

***trans*-1d-CHO**

-crude-

^{1}H NMR(300 MHz, $CDCl_3$)

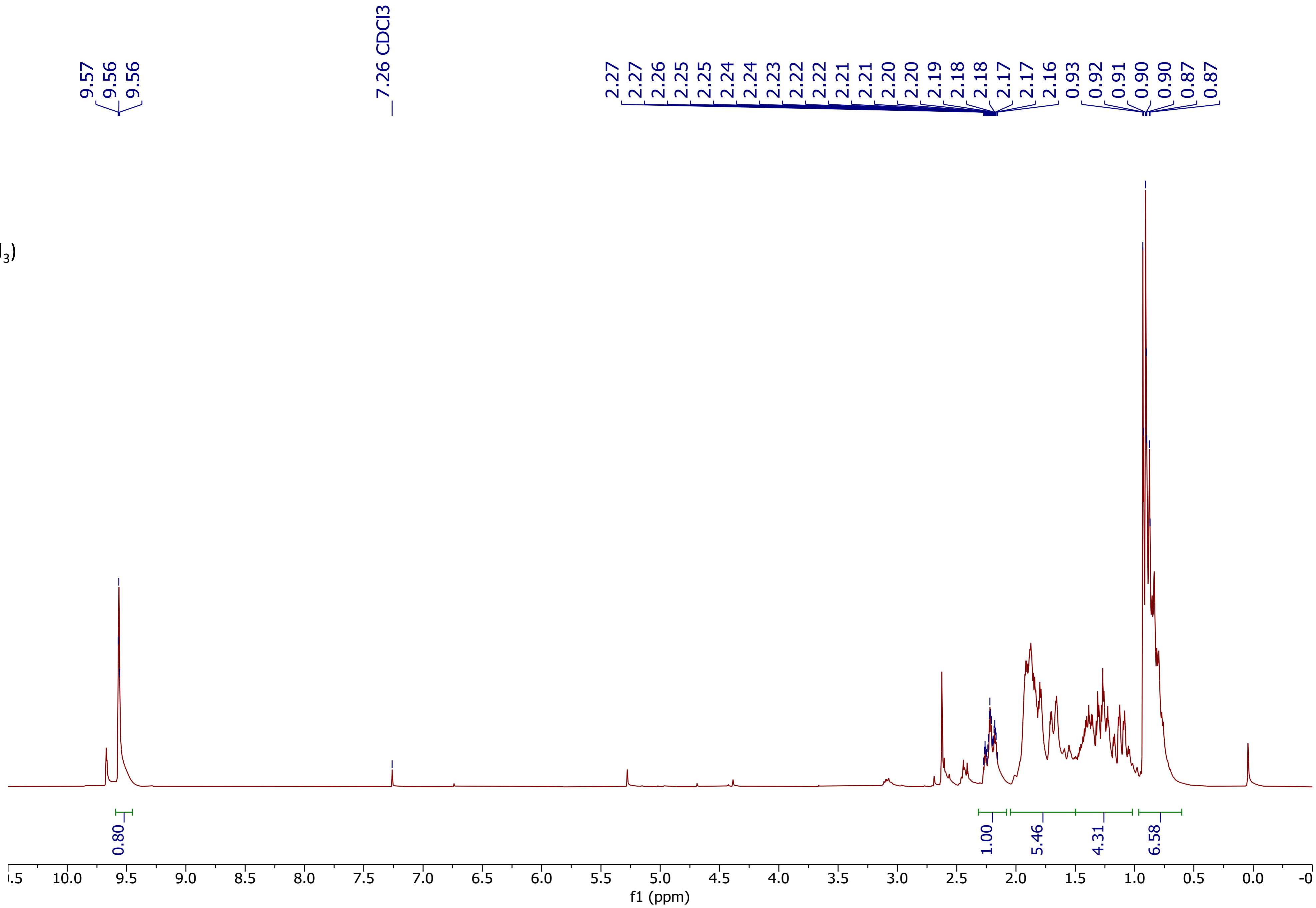

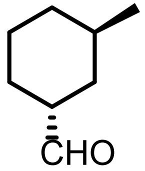

***trans*-1d-CHO**

-crude-

^{13}C NMR (75 MHz, $CDCl_3$)

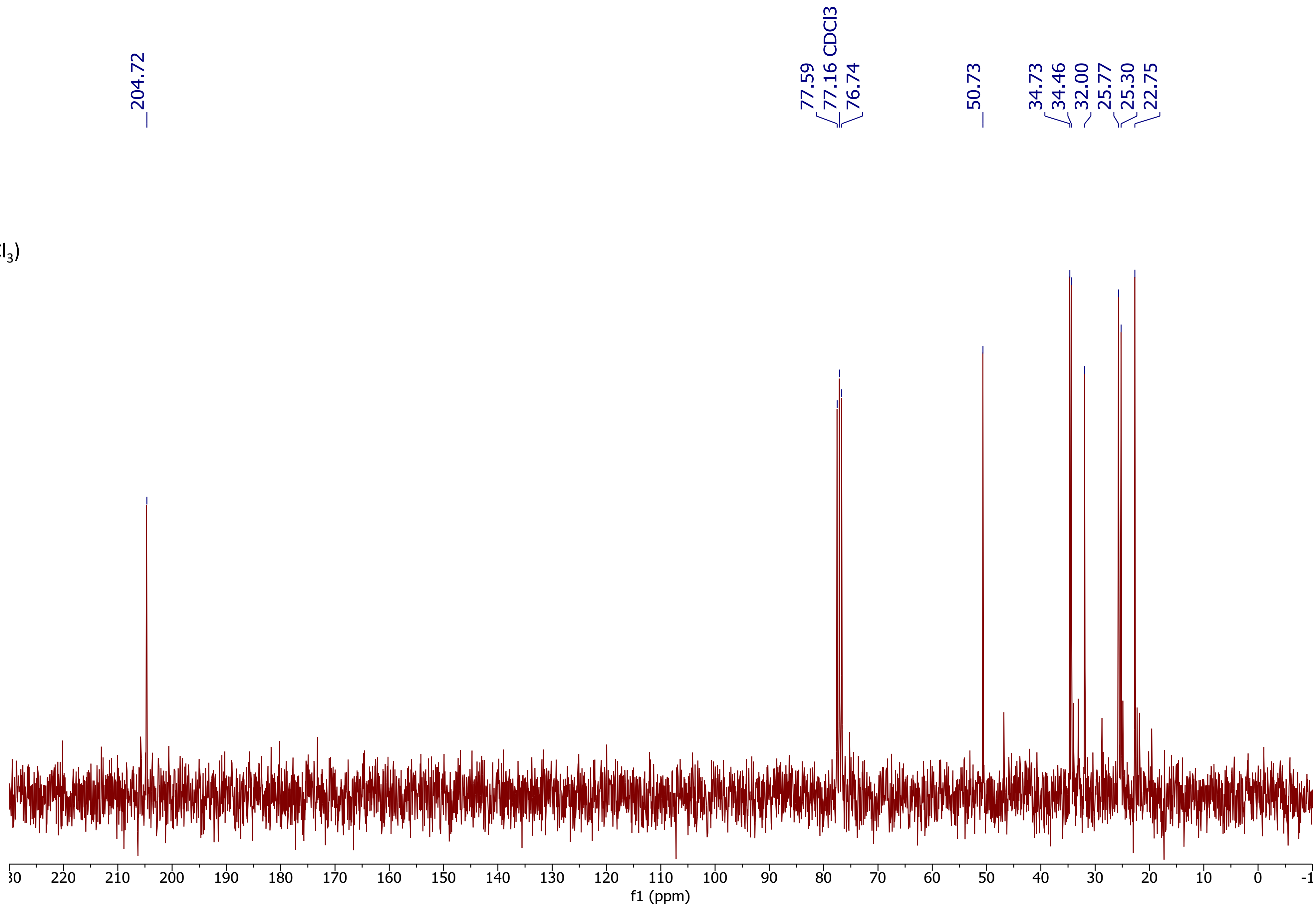

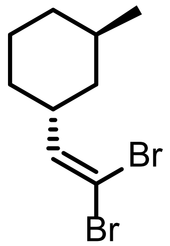

***trans*-1d-CBr$_2$**

^{1}H NMR(300 MHz, $CDCl_3$)

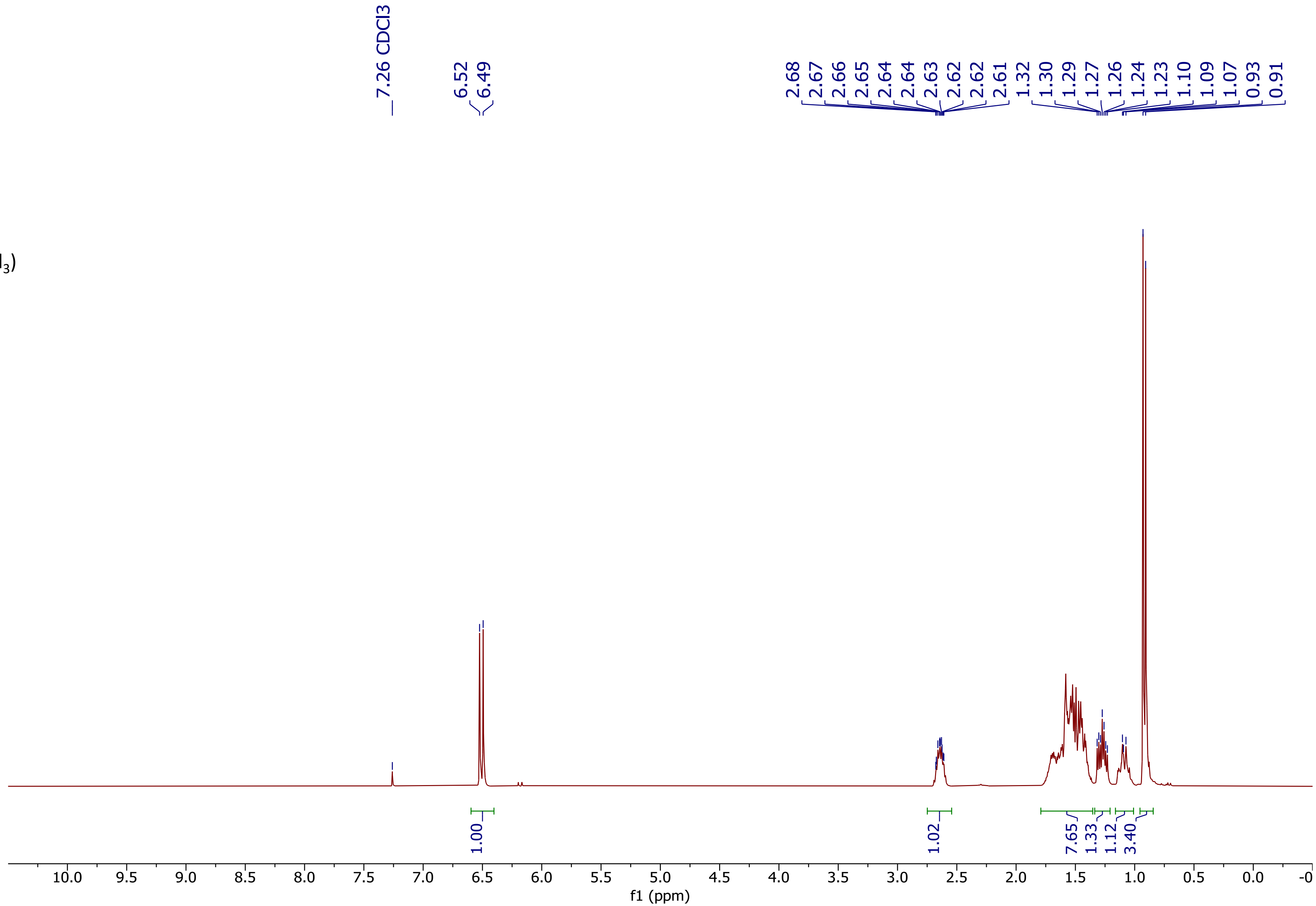

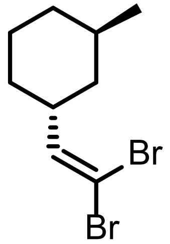

***trans*-1d-CBr$_2$**

^{13}C NMR (75 MHz, $CDCl_3$)

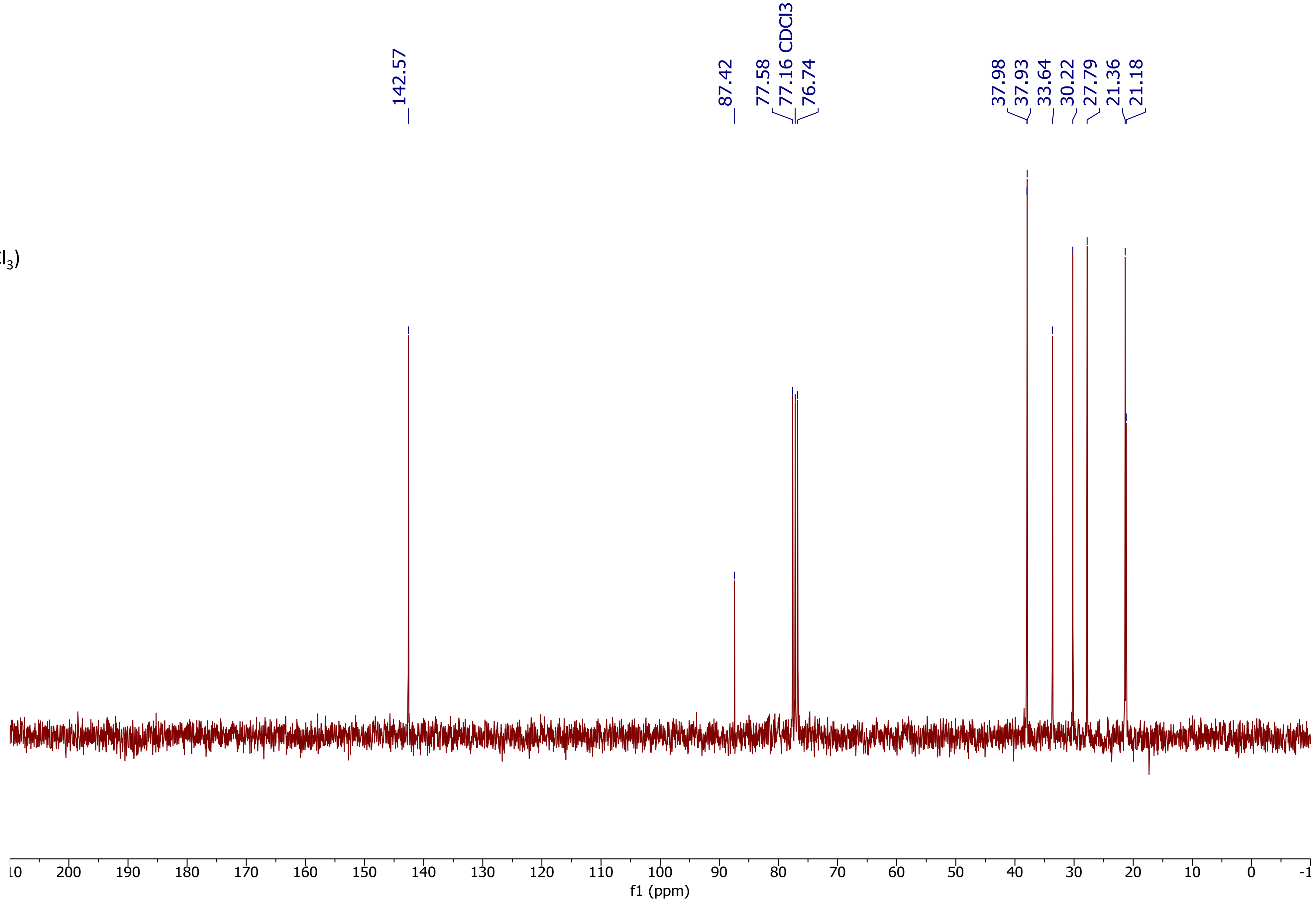

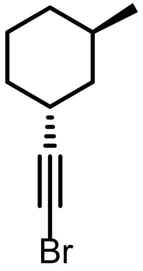

***trans*-1d**

^{1}H NMR(300 MHz, $CDCl_3$)

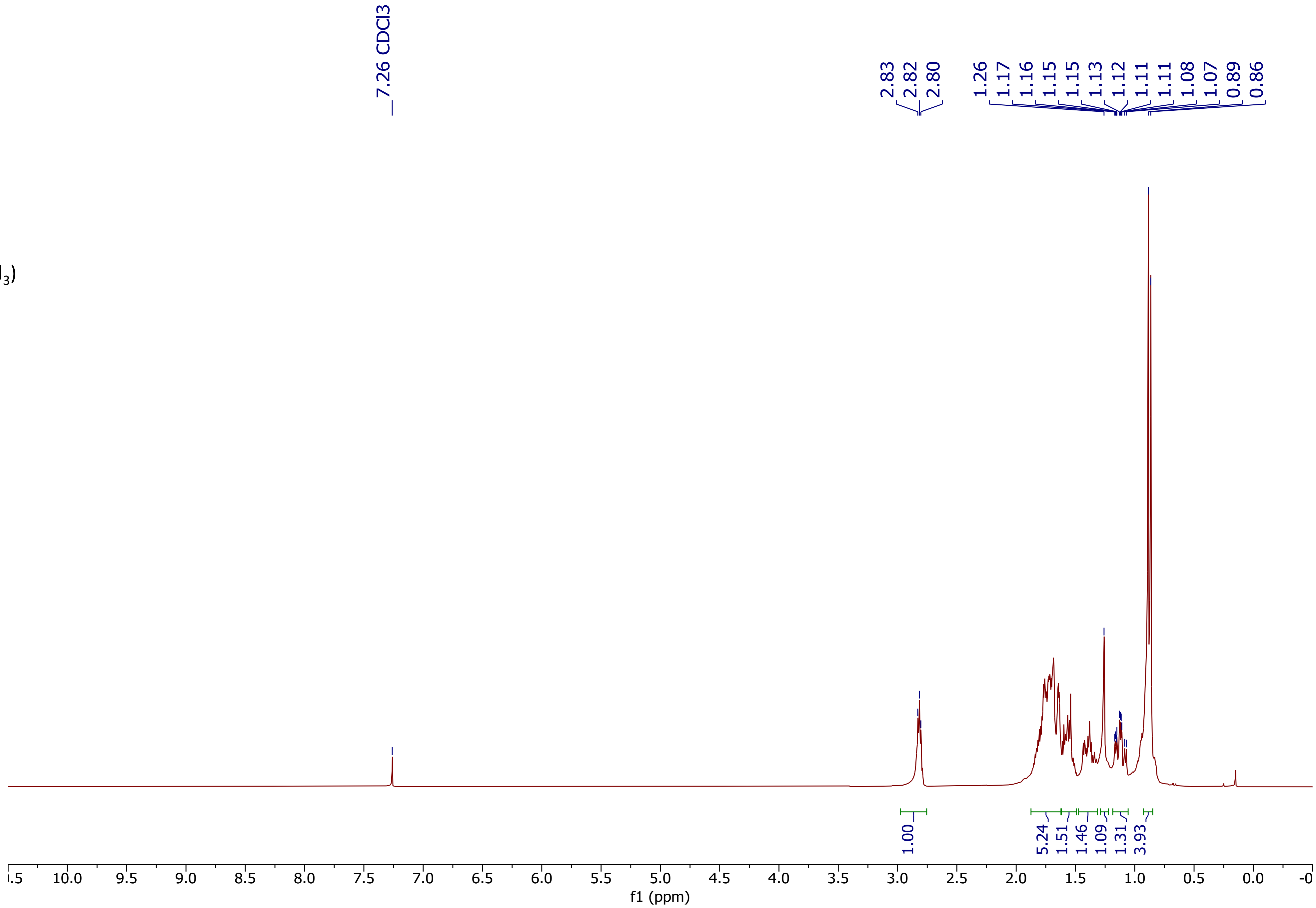

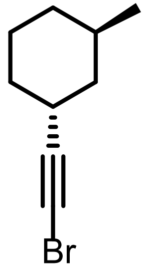

***trans*-1d**

^{13}C NMR (75 MHz, $CDCl_3$)

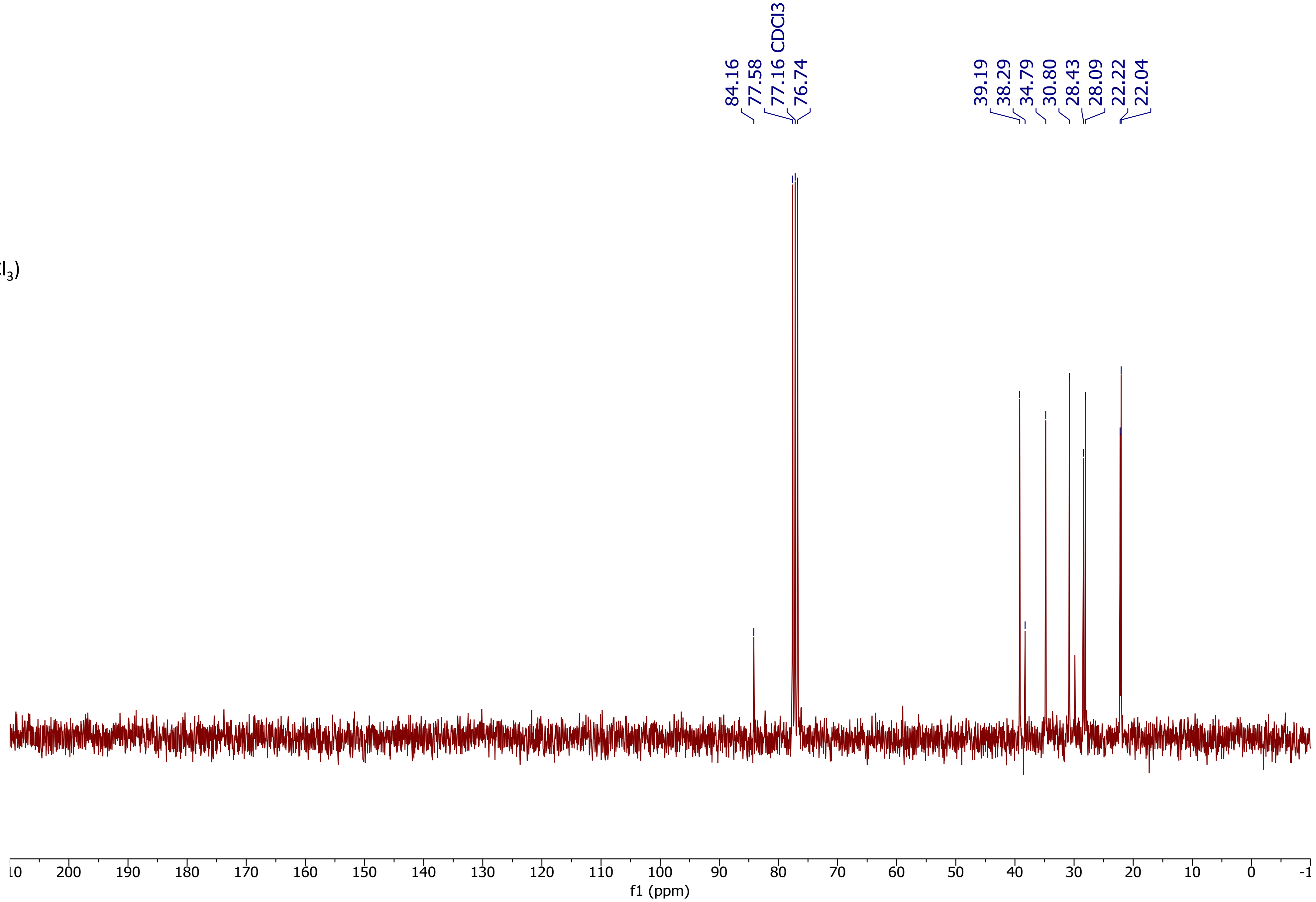

***cis*-1e**

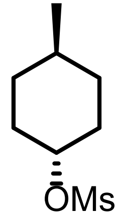

*Me**trans*-1e-OMs**

-crude-

1H NMR(300 MHz, $CDCl_3$)

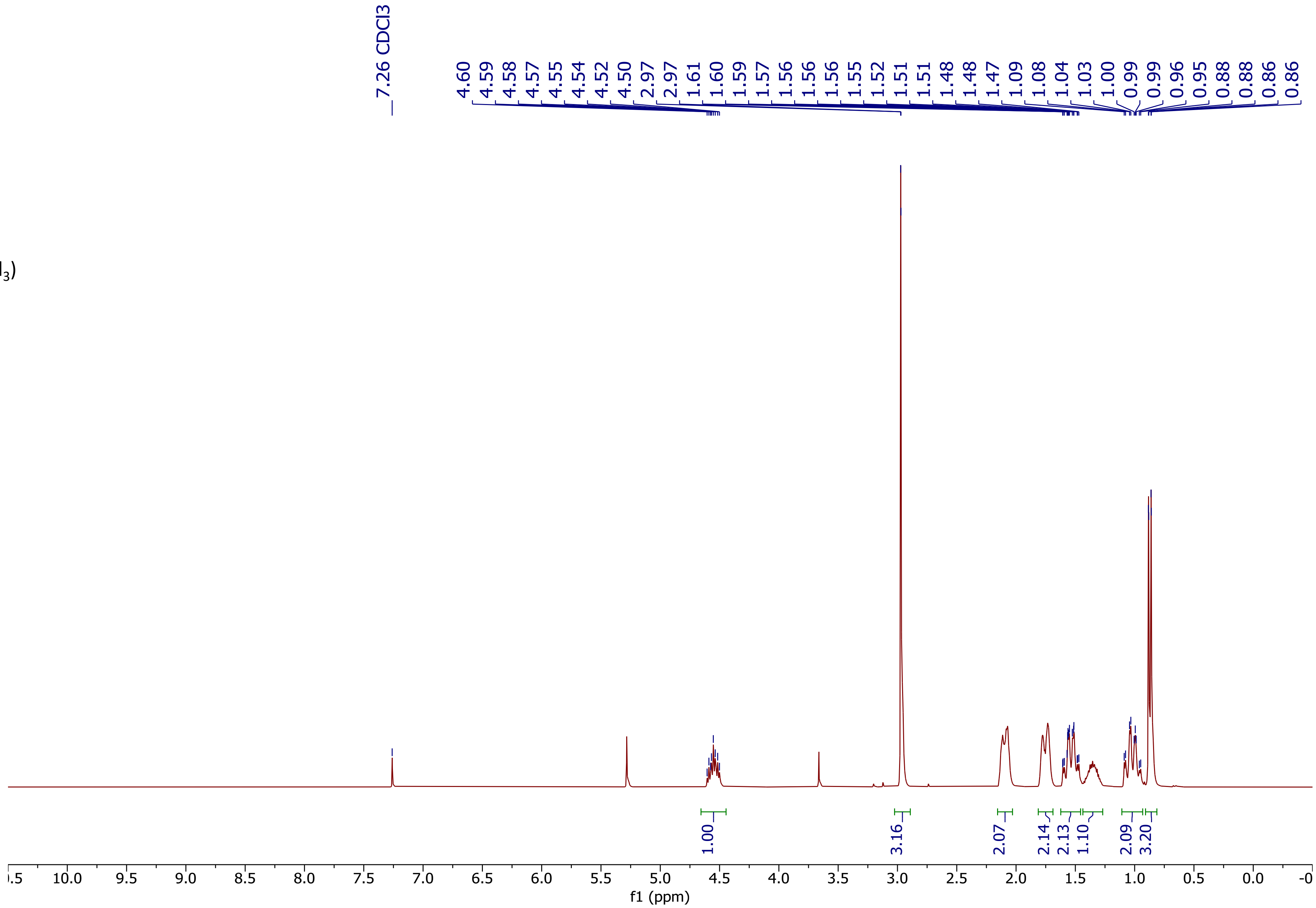

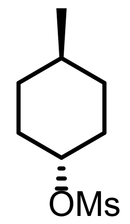

*Me**trans*-**1e-OMs**

-crude-

^{13}C NMR (75 MHz, $CDCl_3$)

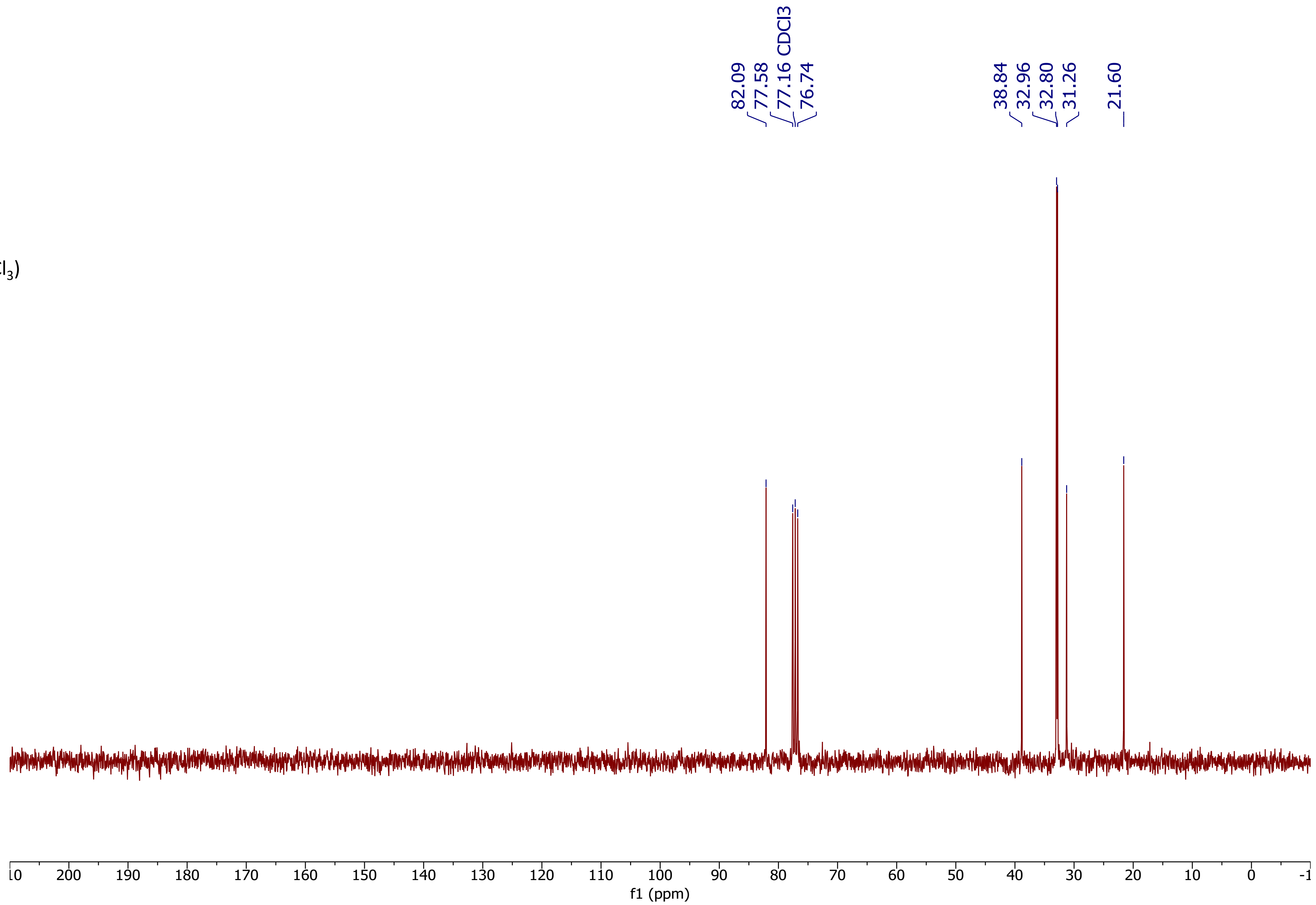

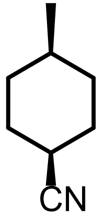

^{Me}***cis*-1e-CN**

-crude-

^{1}H NMR(300 MHz, $CDCl_3$)

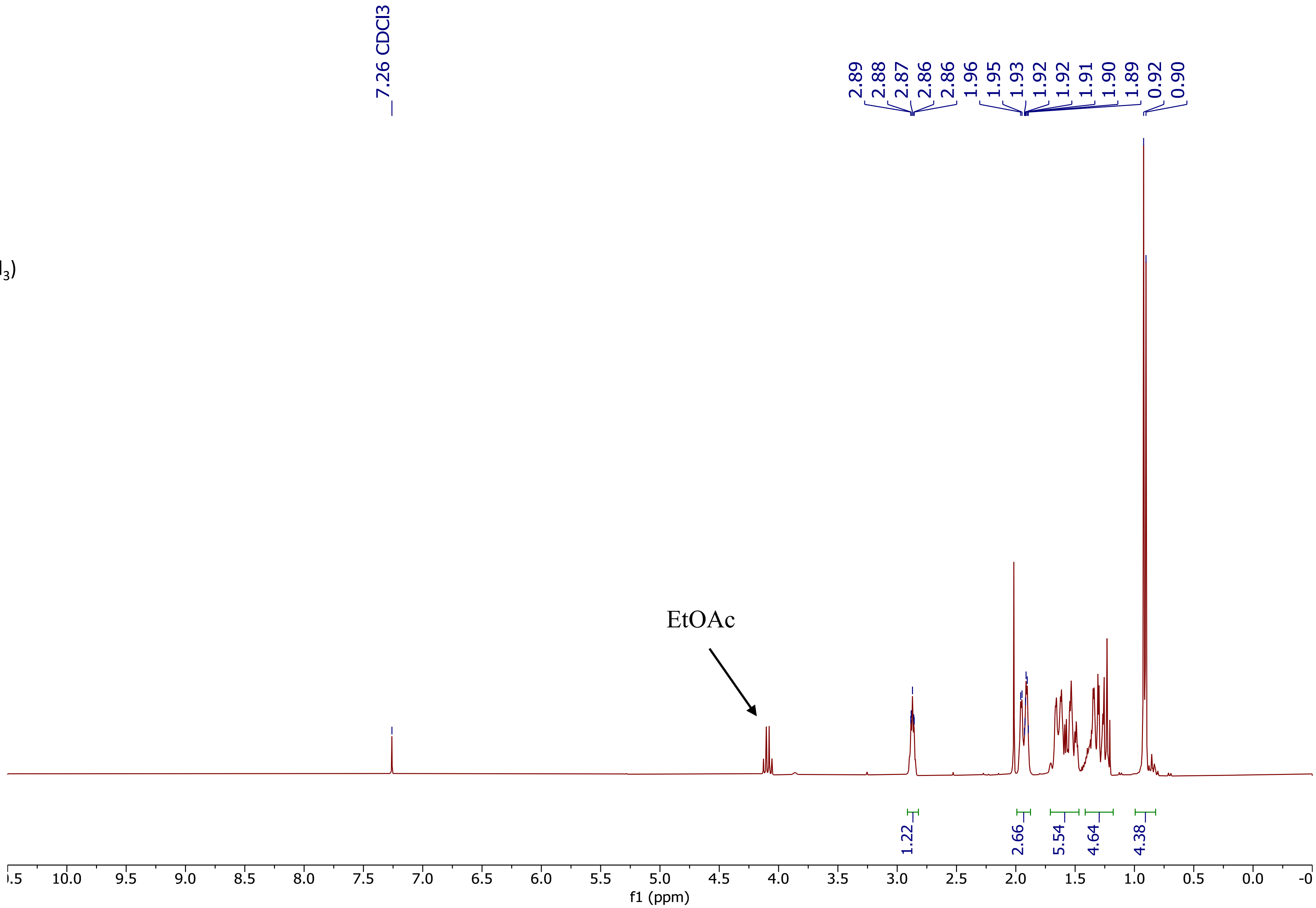

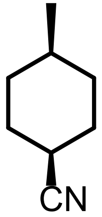

Me**cis-1e-CN**

-crude-

^{13}C NMR (75 MHz, $CDCl_3$)

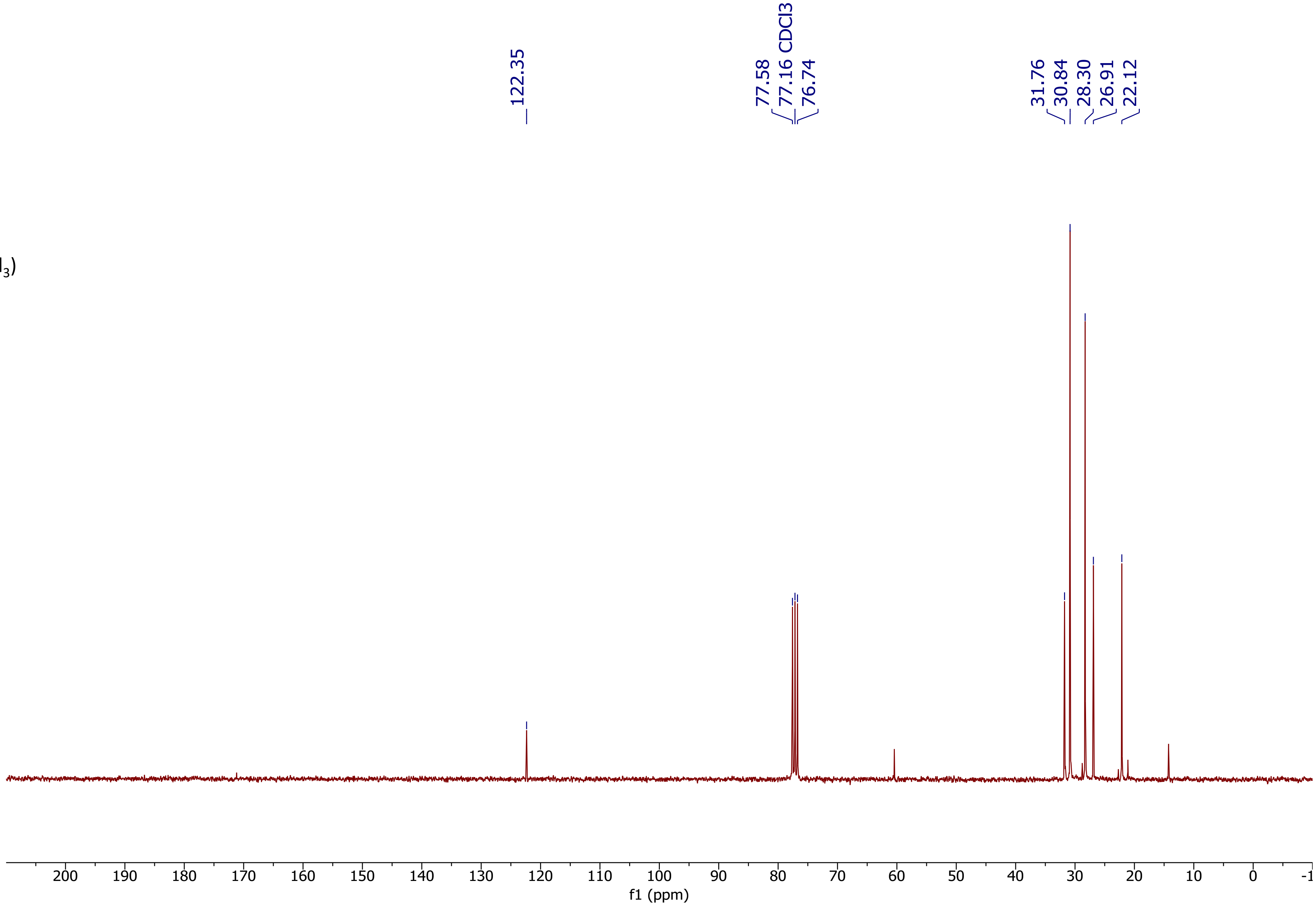

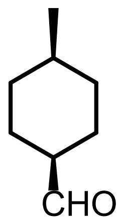

^{Me}*cis*-1e-CHO

-crude-

^{1}H NMR(300 MHz, $CDCl_3$)

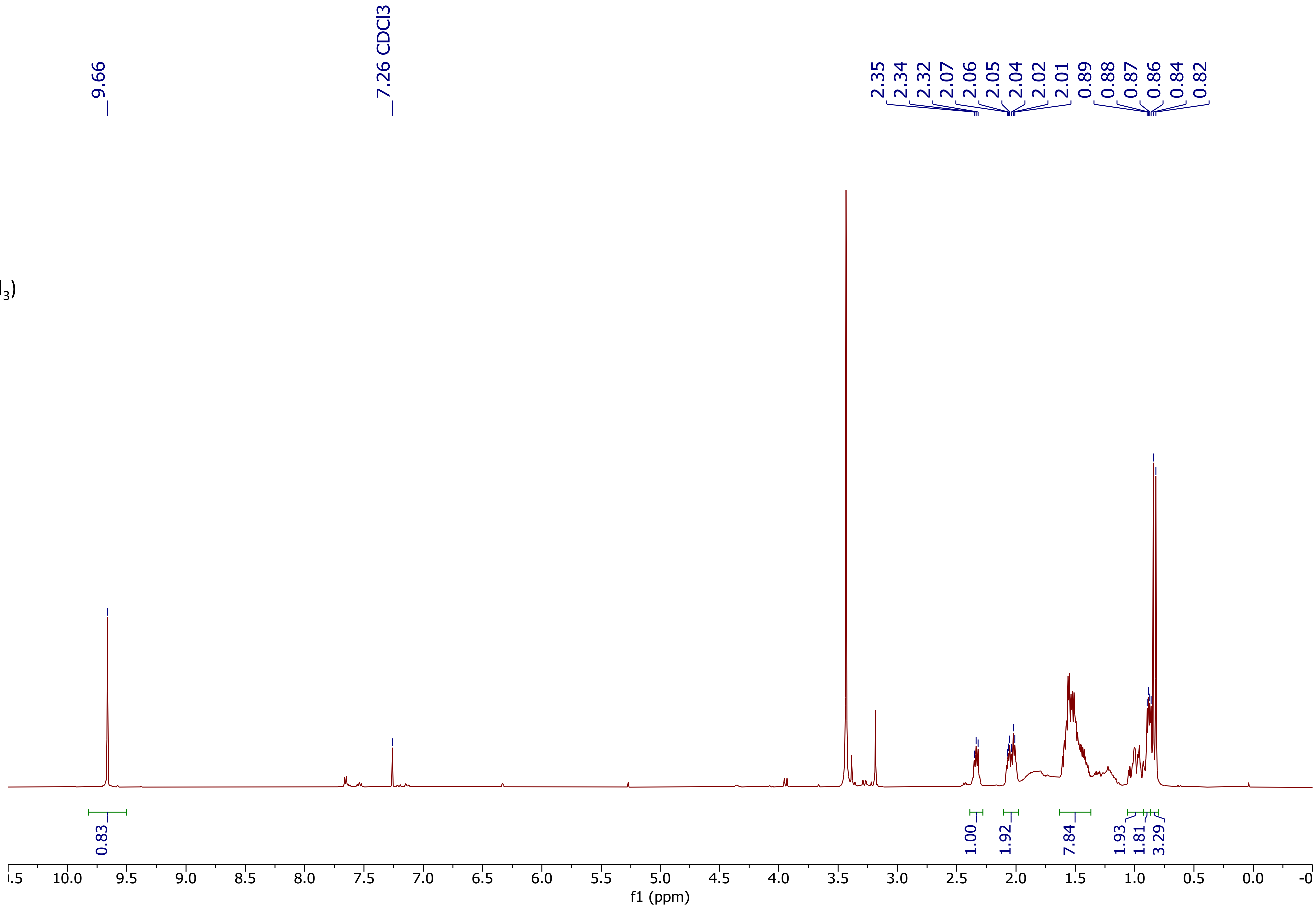

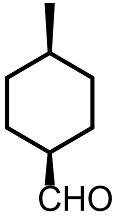

Me ***cis*-1e-CHO**

-crude-

^{13}C NMR (75 MHz, $CDCl_3$)

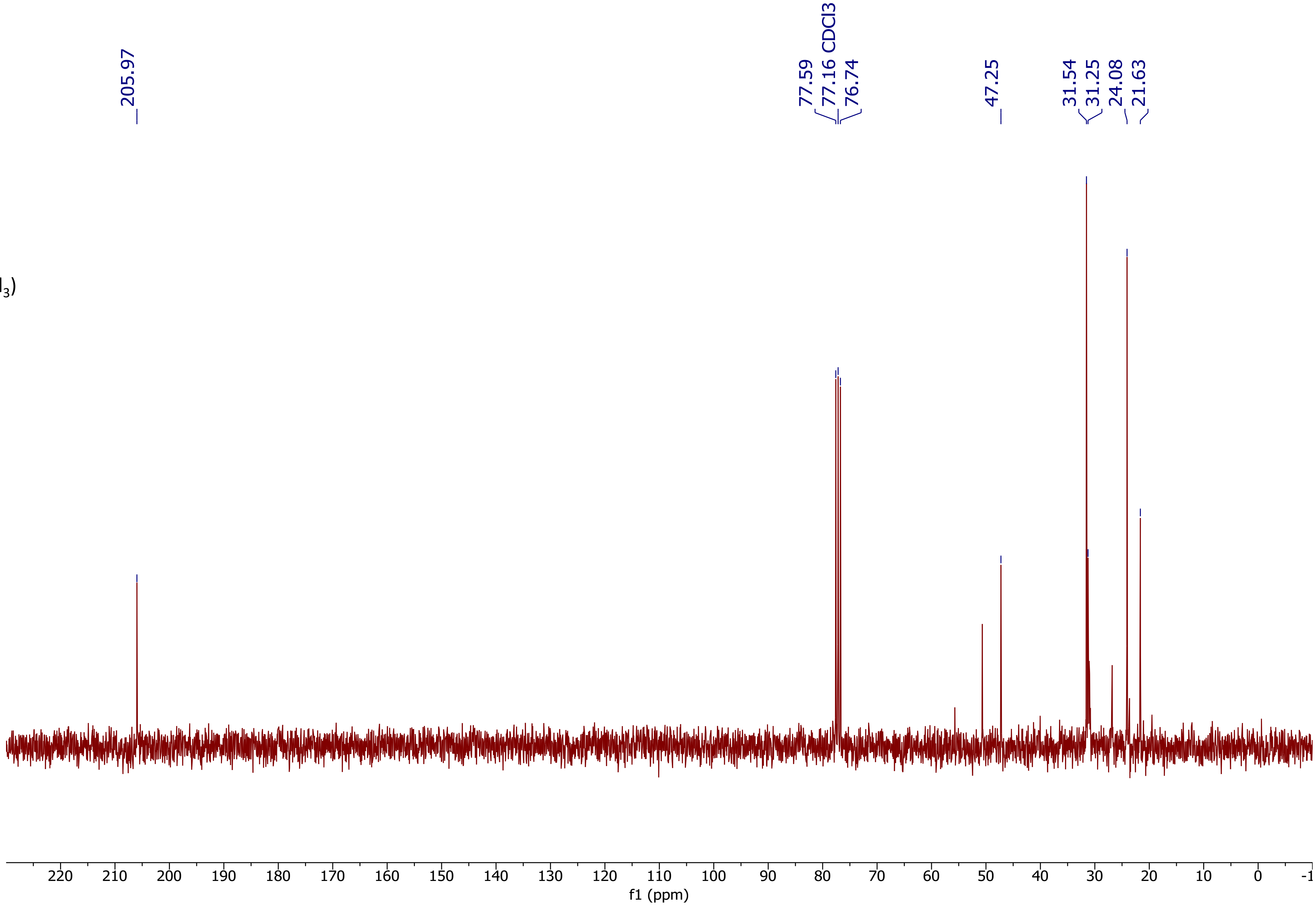

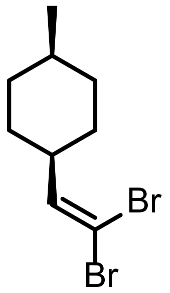

***Me*cis-1e-CBr₂**

¹H NMR(300 MHz, $CDCl_3$)

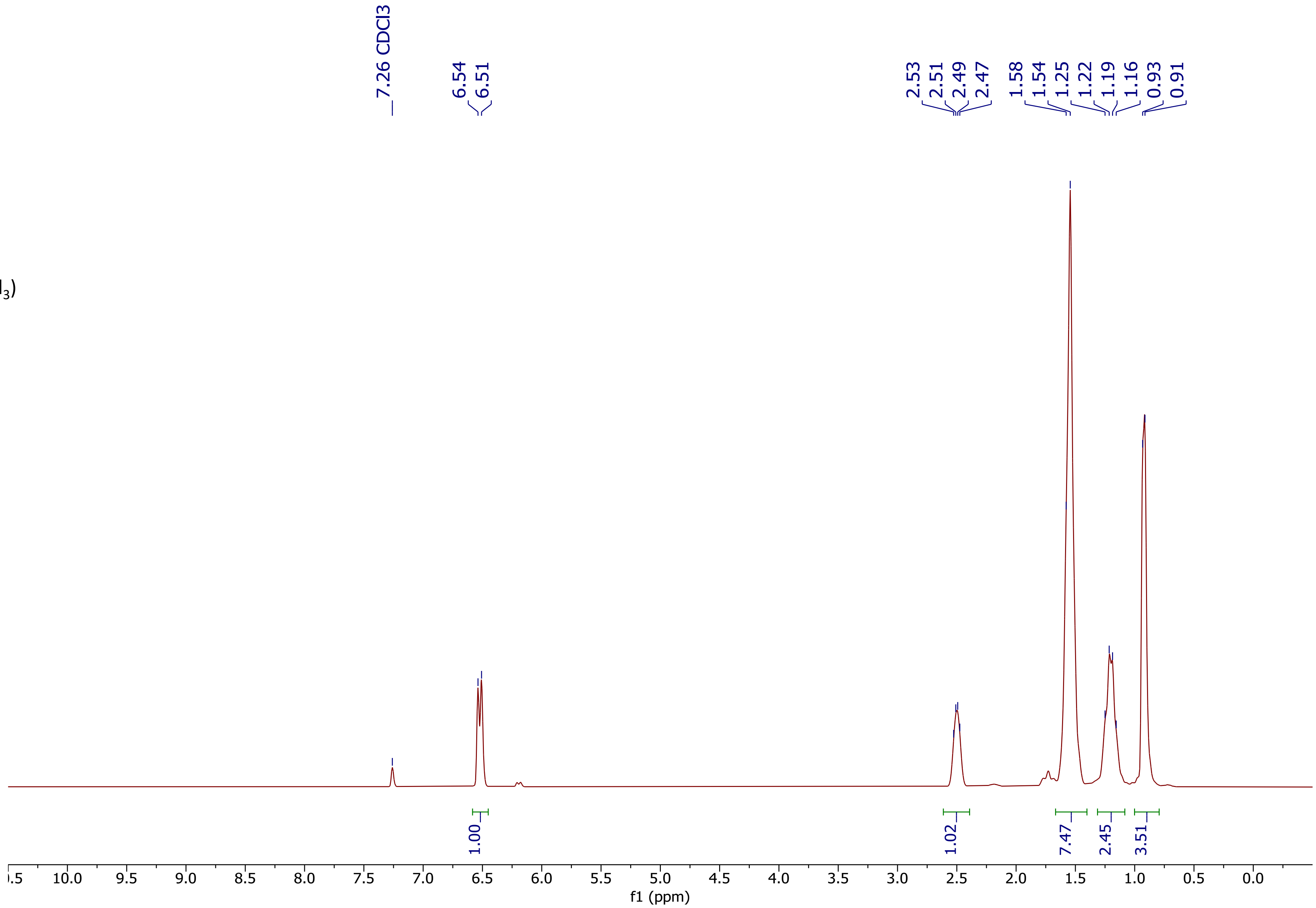

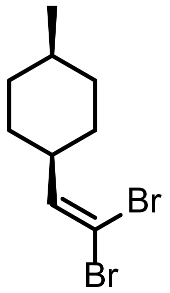

^{Me}*cis*-**1e-CBr$_2$**

^{13}C NMR (75 MHz, $CDCl_3$)

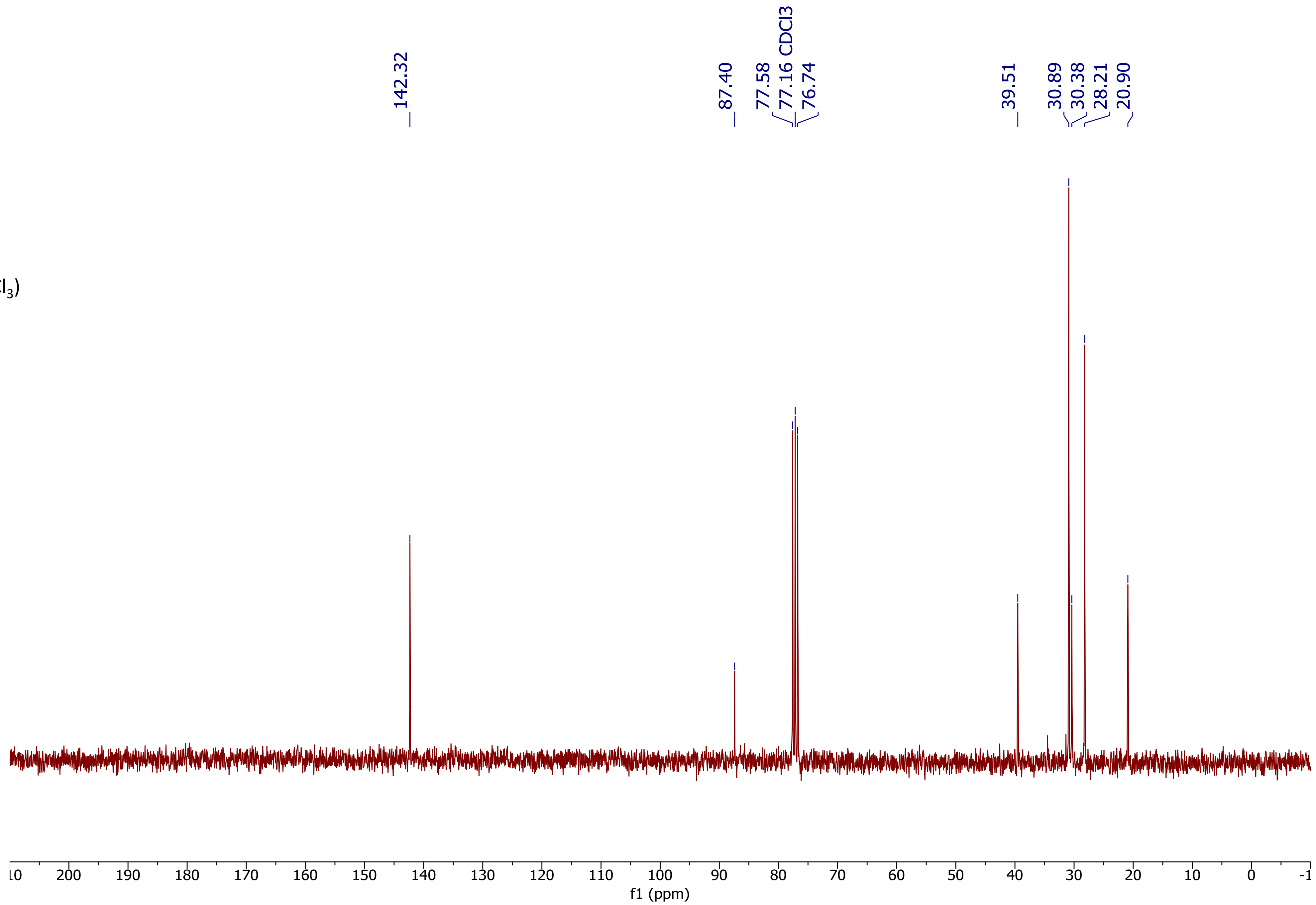

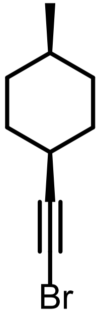

Me cis-**1e**

¹H NMR(300 MHz, $CDCl_3$)

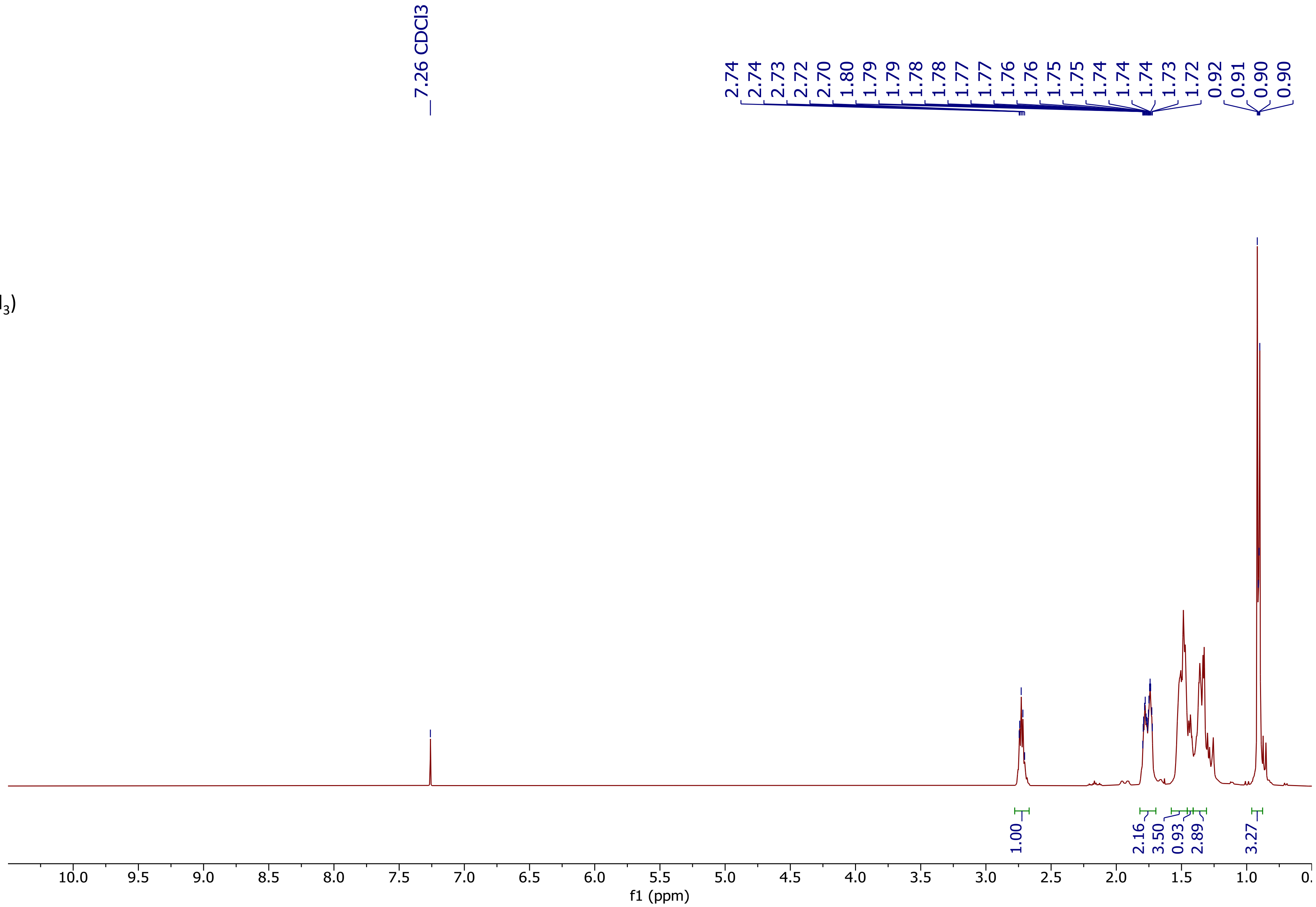

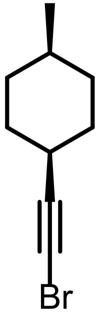

*Me**cis*-1e**

^{13}C NMR (75 MHz, $CDCl_3$)

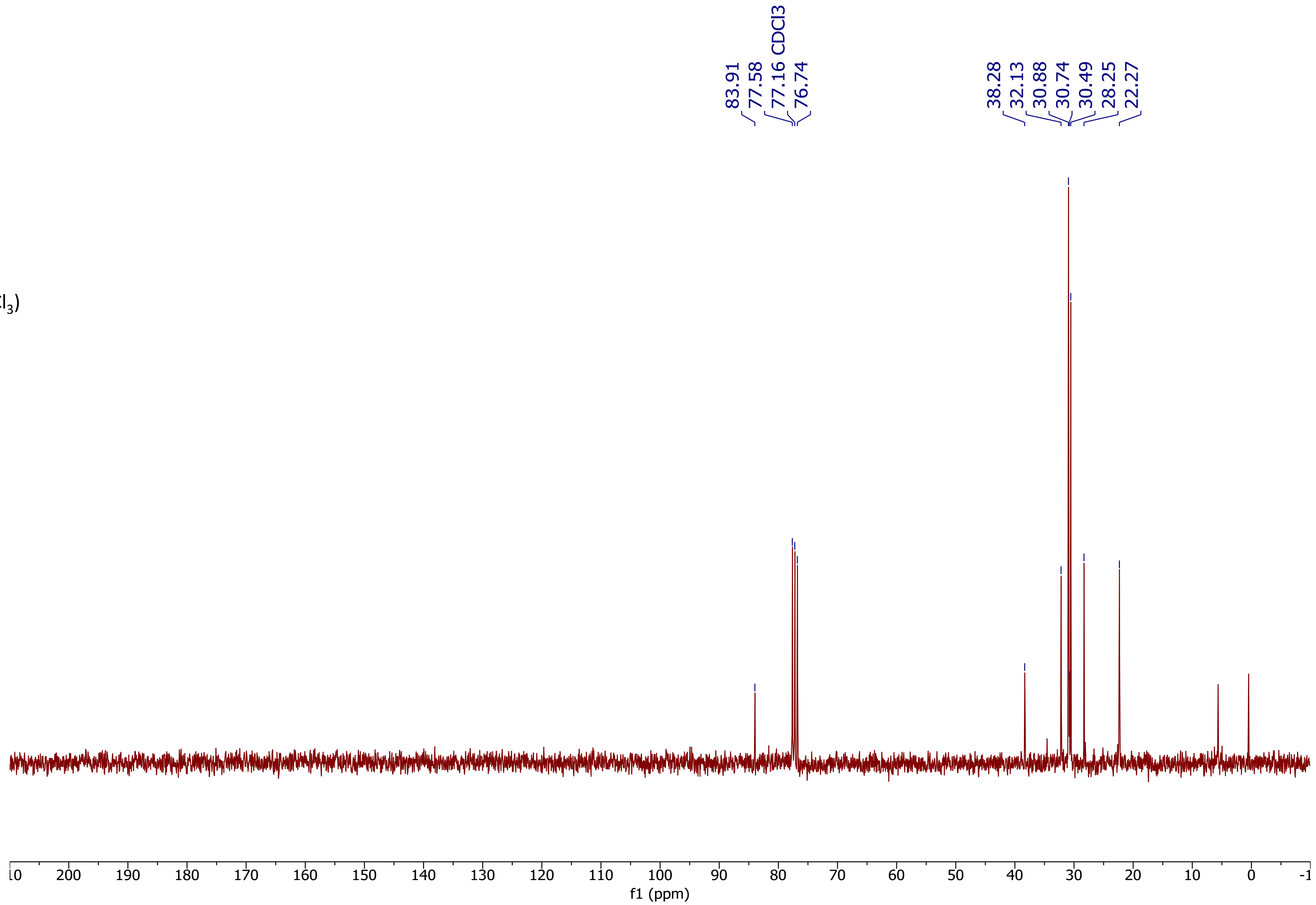

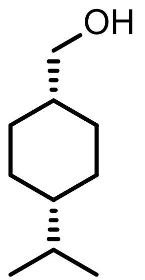

iPr cis-**1e-OH**

-crude-

^{1}H NMR(300 MHz, $CDCl_3$)

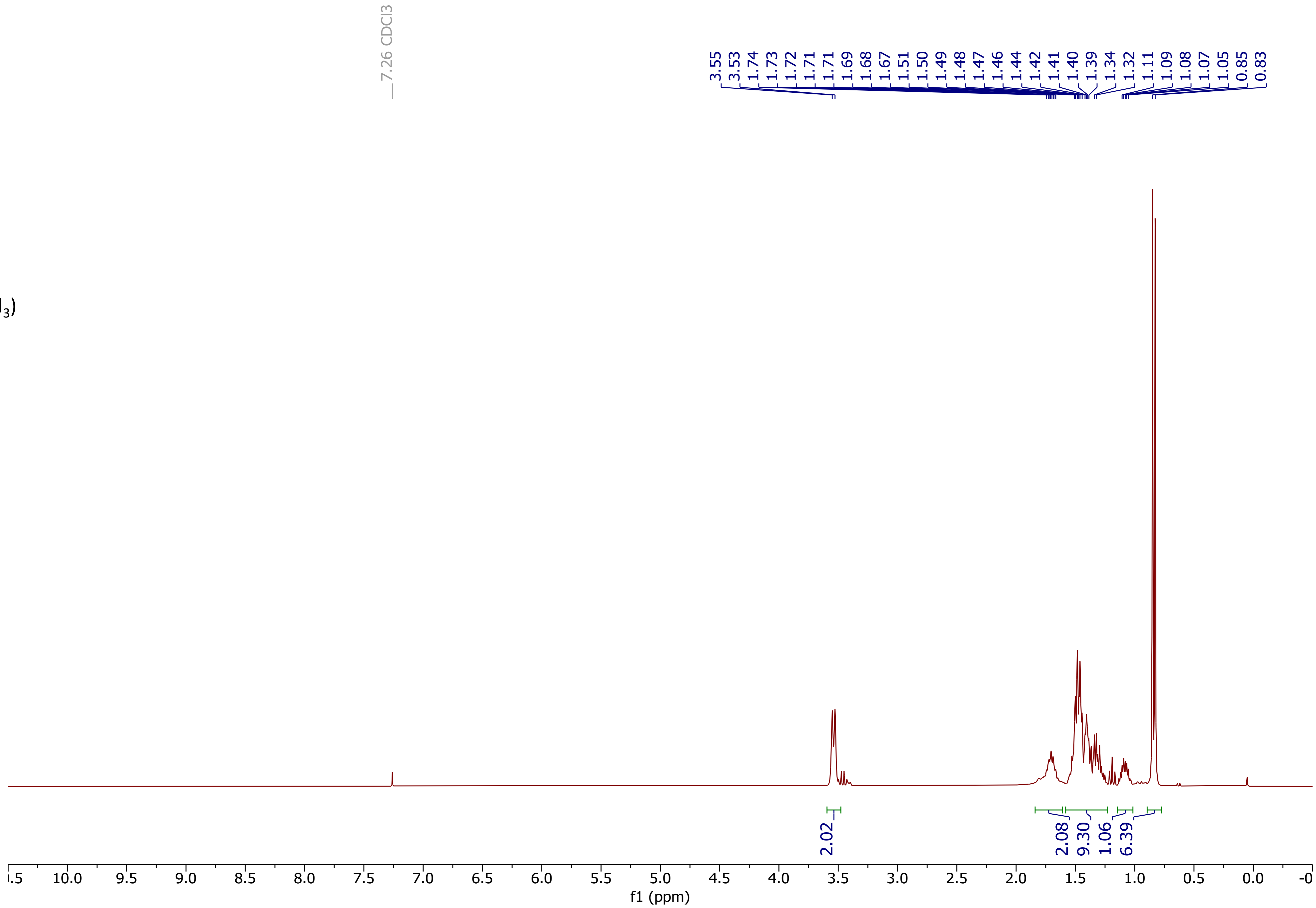

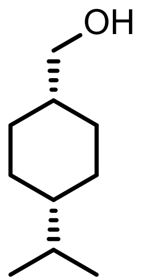

^{iPr}***cis*-1e-OH**

-crude-

^{13}C NMR (75 MHz, $CDCl_3$)

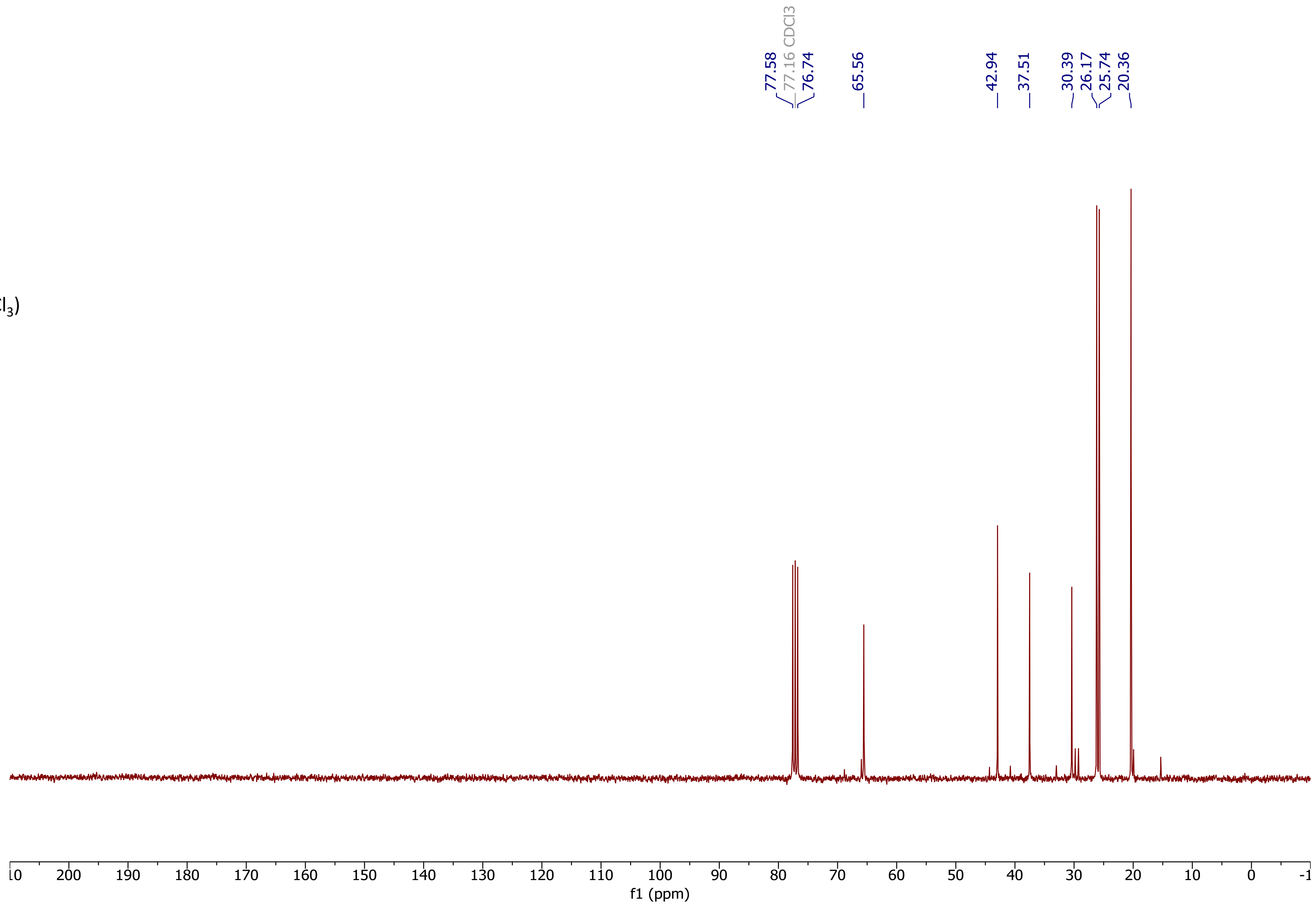

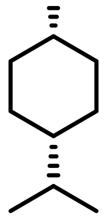

^{iPr}cis-**1e-CHO**

-crude-

^{1}H NMR(300 MHz, $CDCl_3$)

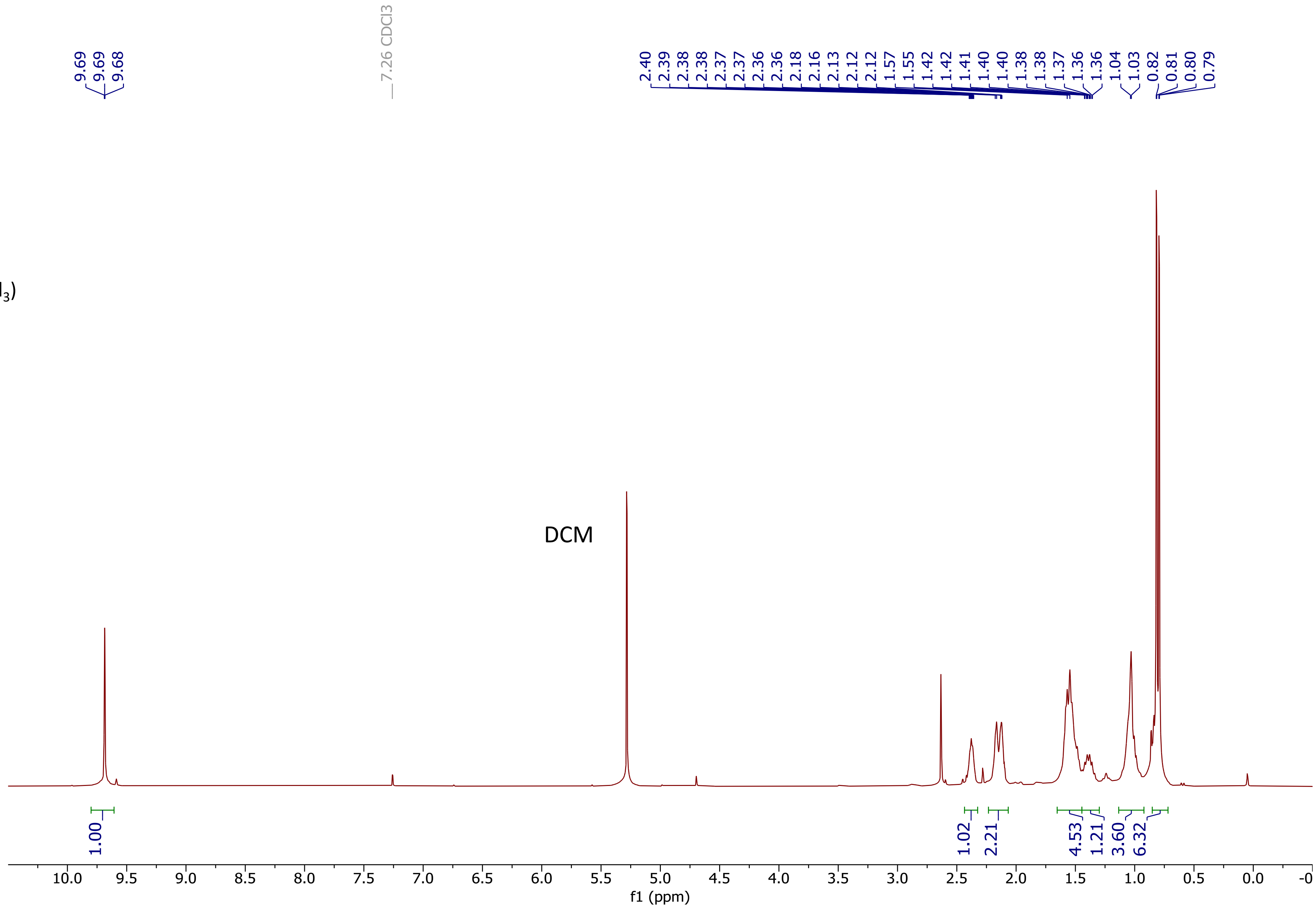

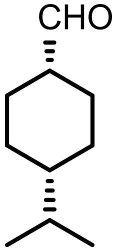

^{iPr}***cis*-1e-CHO**

-crude-

^{13}C NMR (75 MHz, $CDCl_3$)

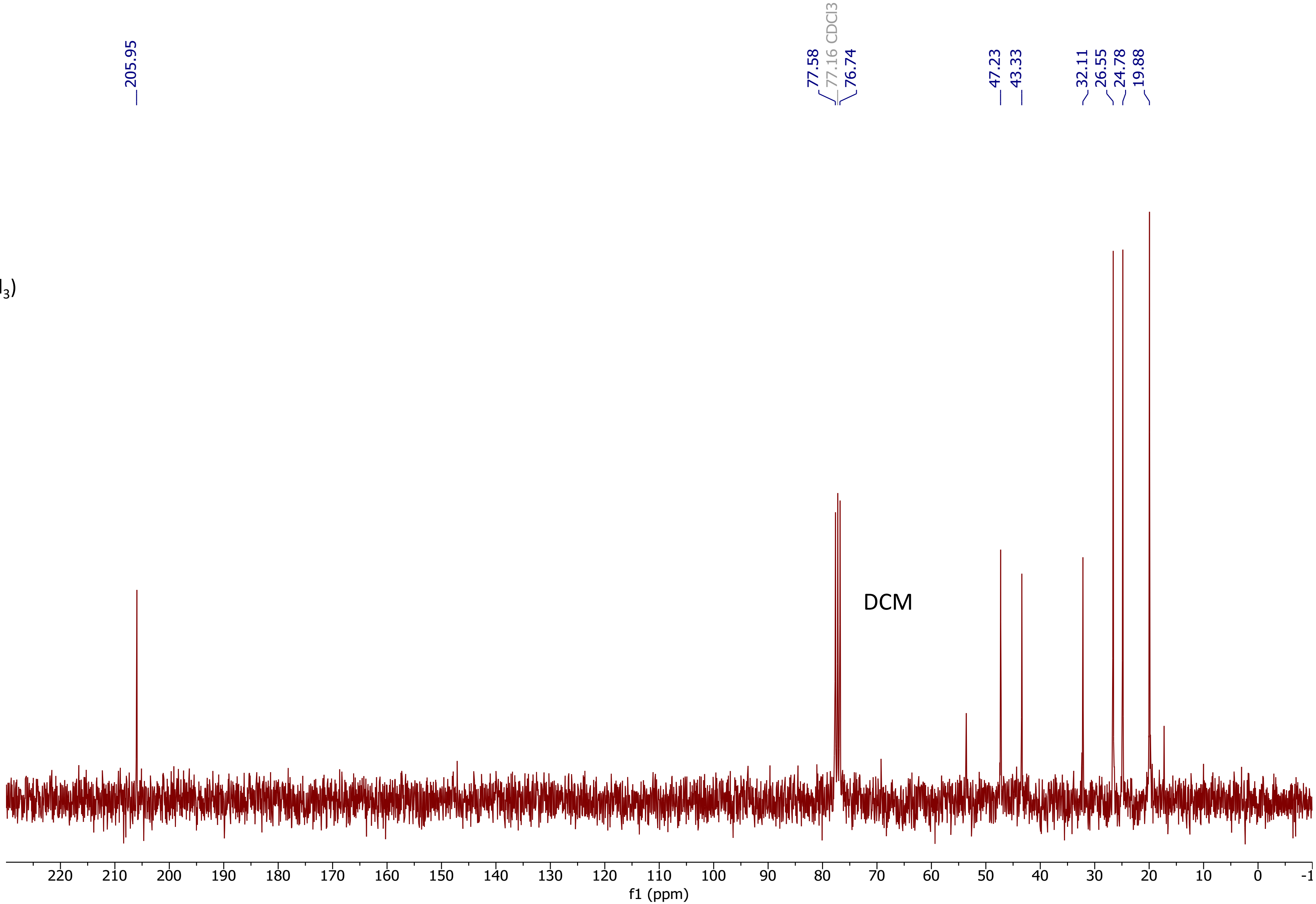

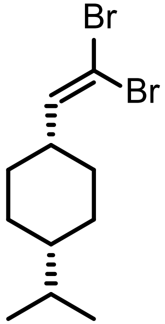

*iPr**cis*-1e-CBr$_2$

^{1}H NMR(300 MHz, $CDCl_3$)

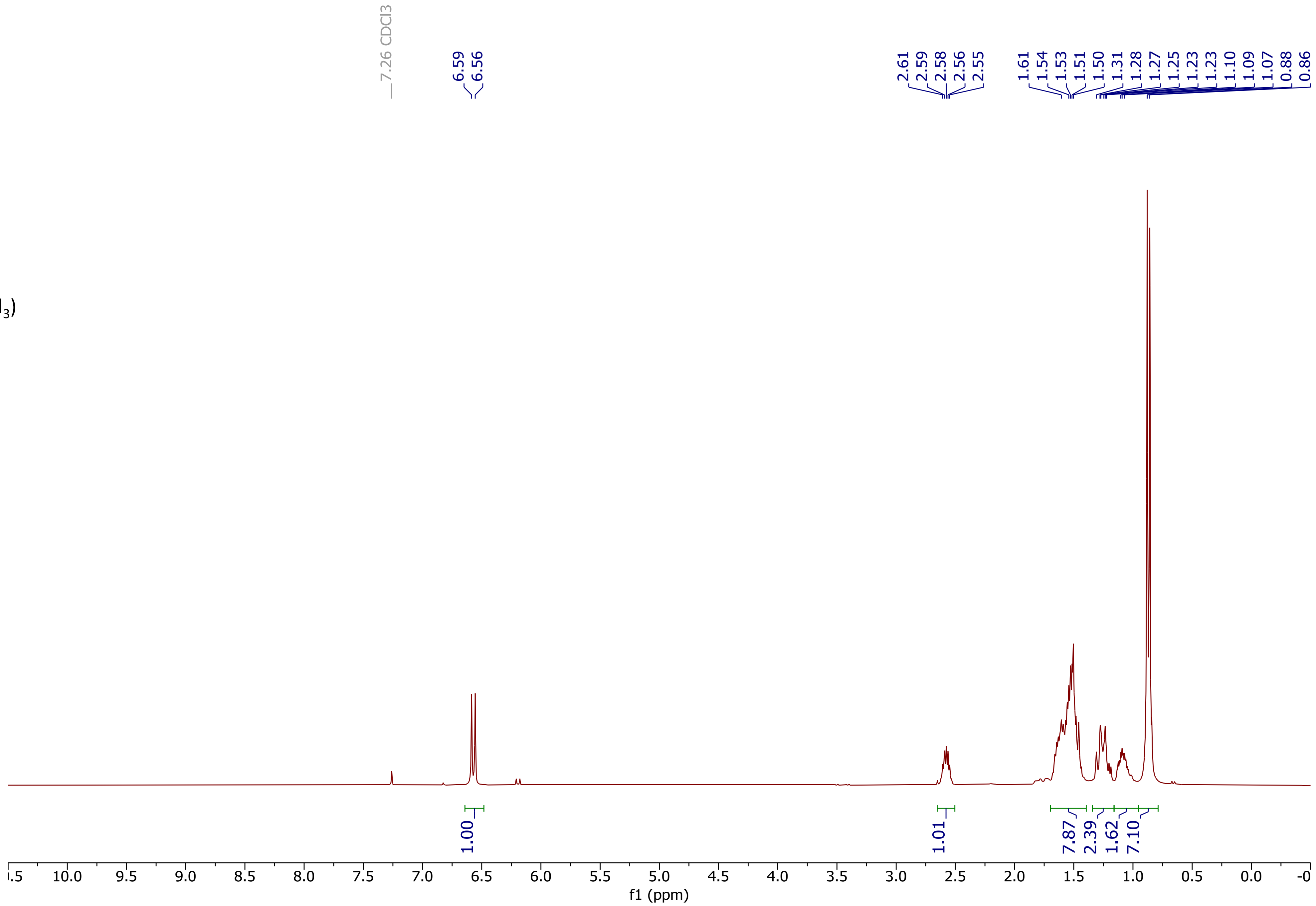

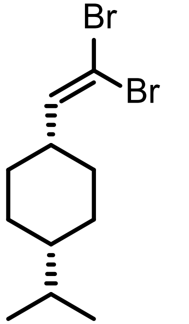

***iPr*cis-1e-CBr$_2$**

^{13}C NMR (75 MHz, $CDCl_3$)

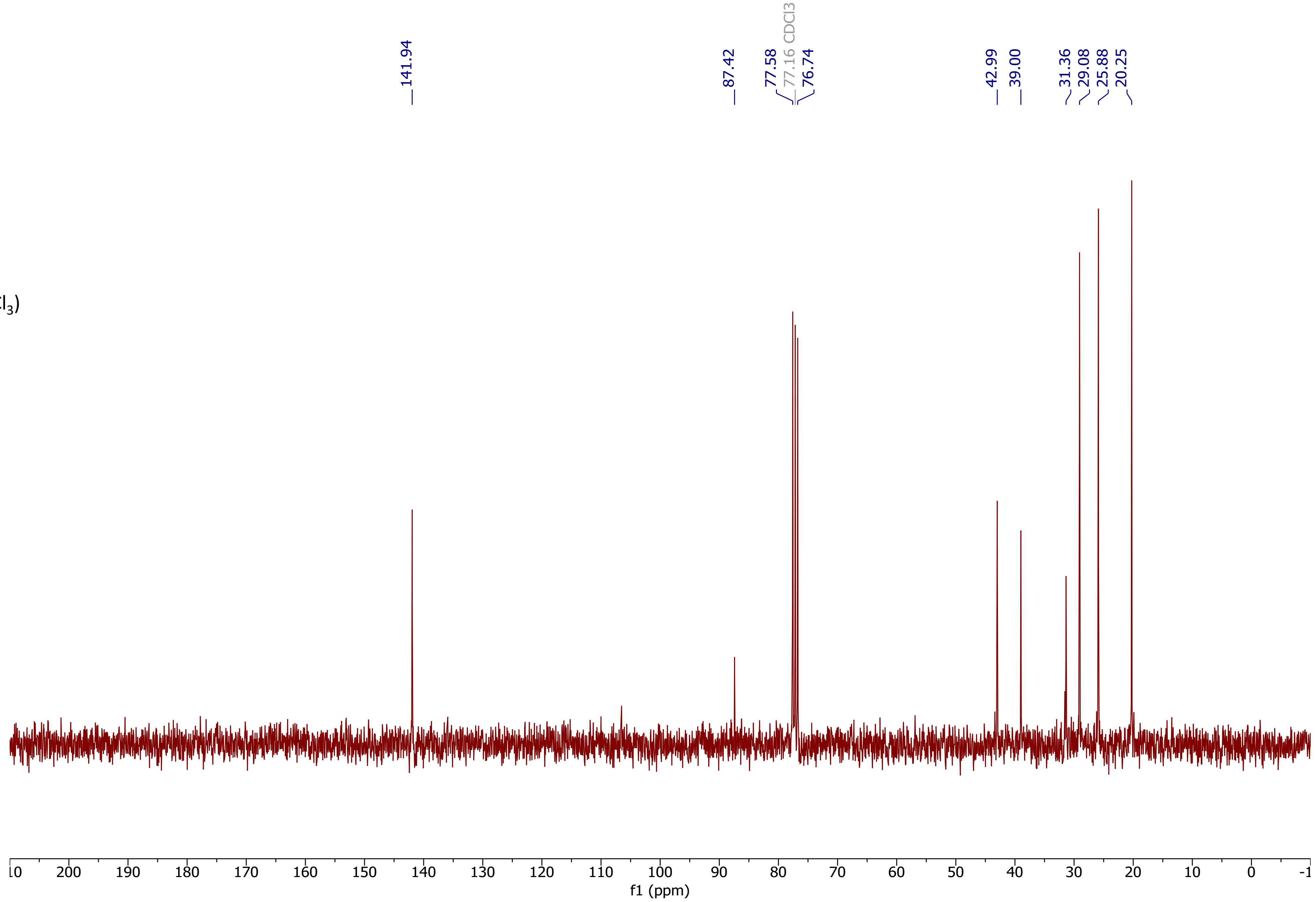

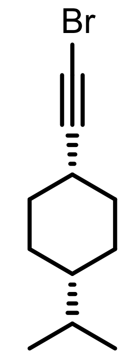

***iPr*cis-1e**

^{1}H NMR(300 MHz, $CDCl_3$)

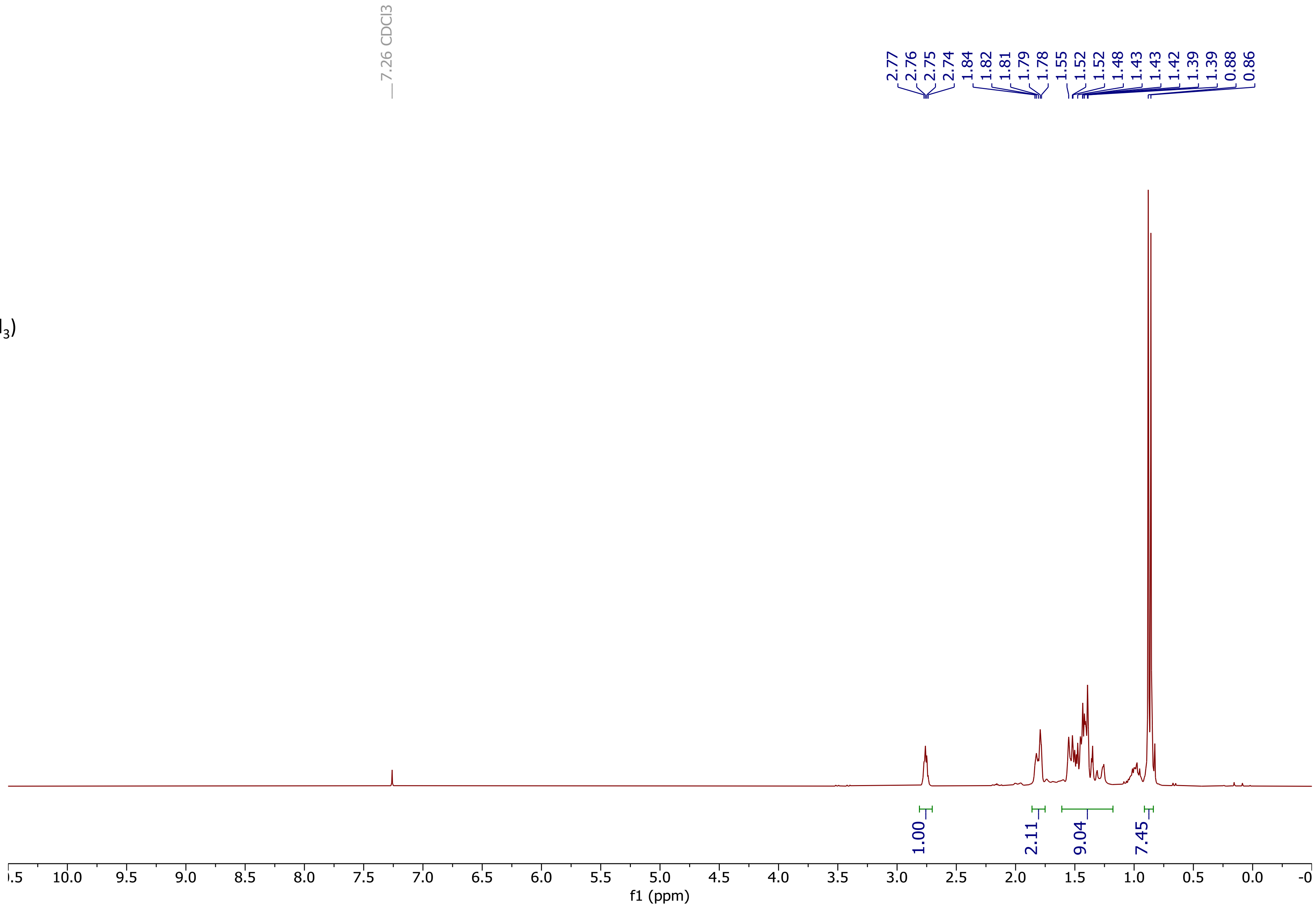

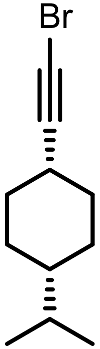

iPr cis-**1e**

^{13}C NMR (75 MHz, $CDCl_3$)

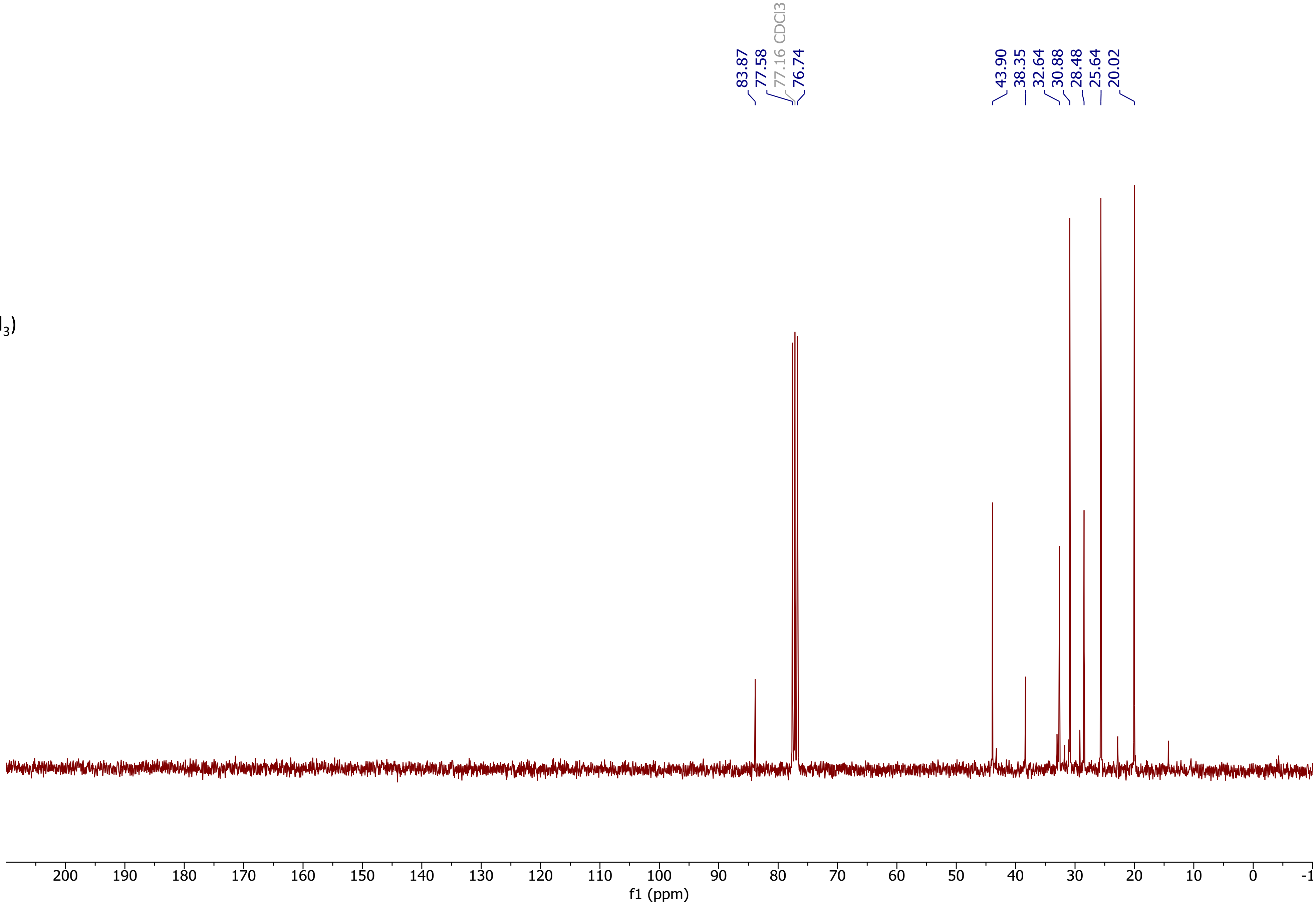

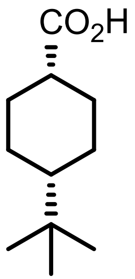

^{tBu}*cis*-1e-CO_2H

^{1}H NMR(300 MHz, $CDCl_3$)

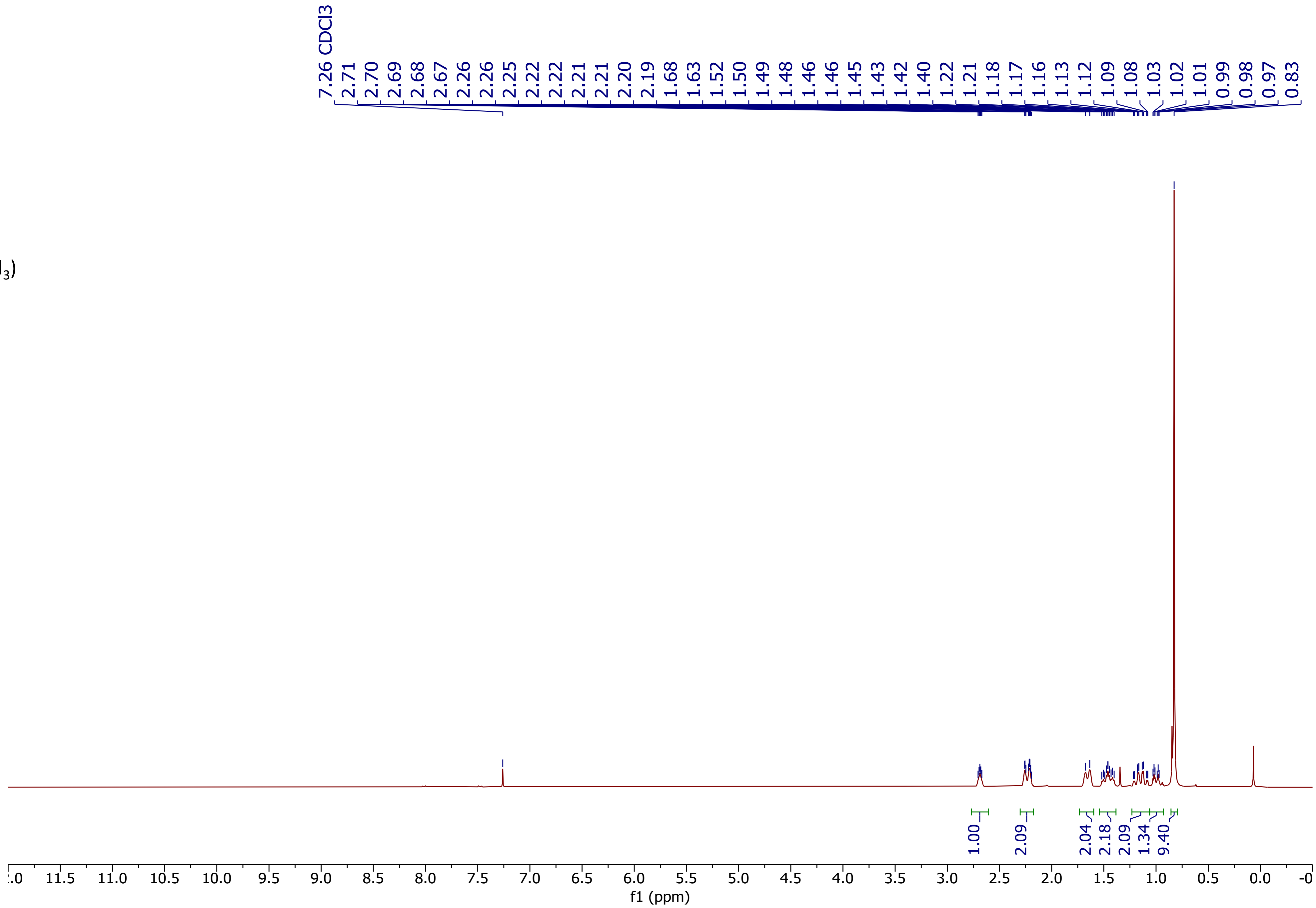

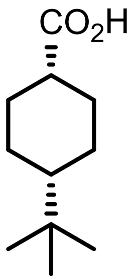

^{tBu}*cis*-1e-CO$_2$H

^{13}C NMR (75 MHz, CDCl$_3$)

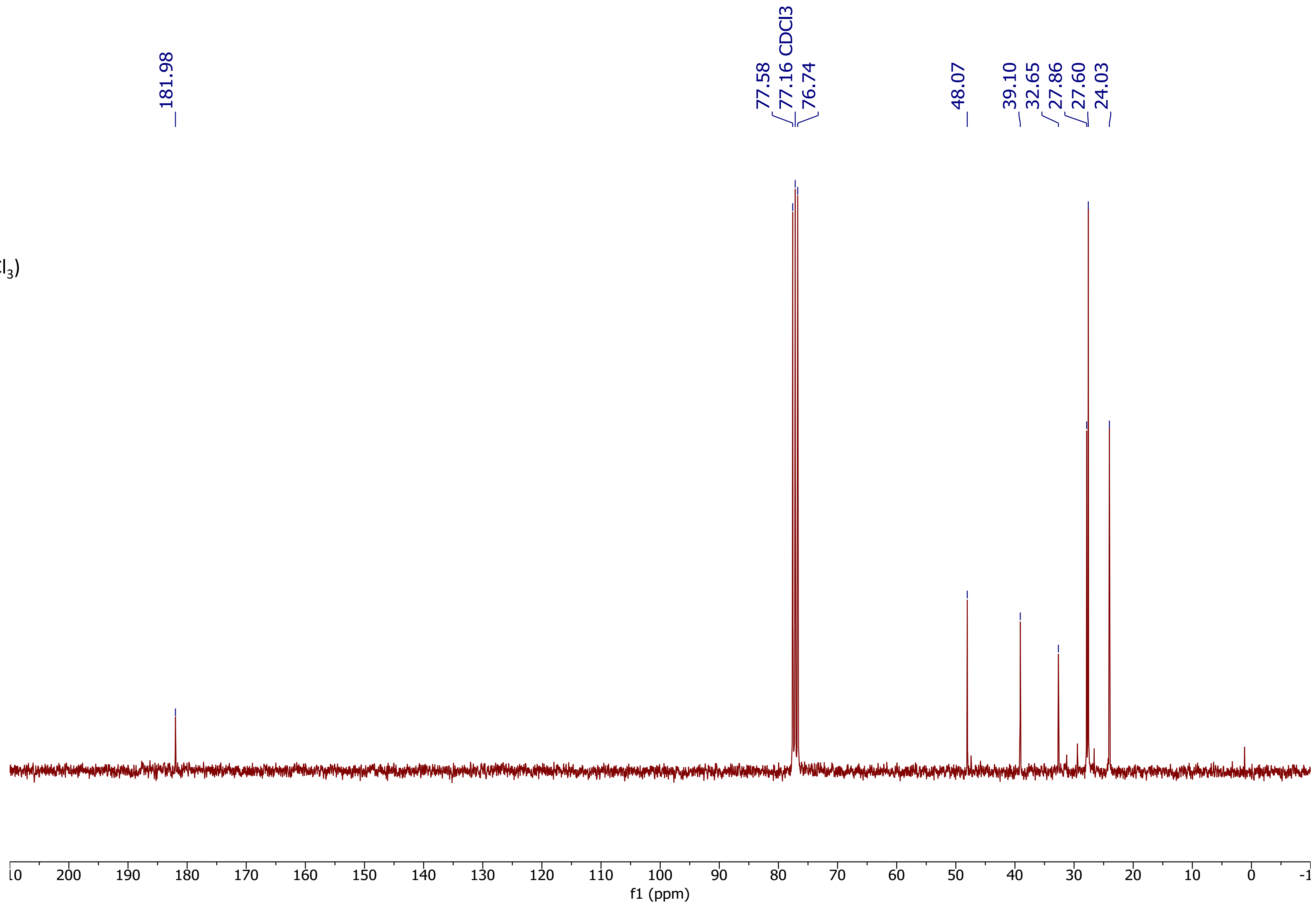

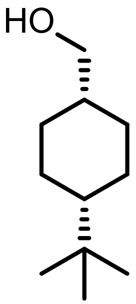

*tBu**cis*-1e-OH

-crude-

¹H NMR(300 MHz, $CDCl_3$)

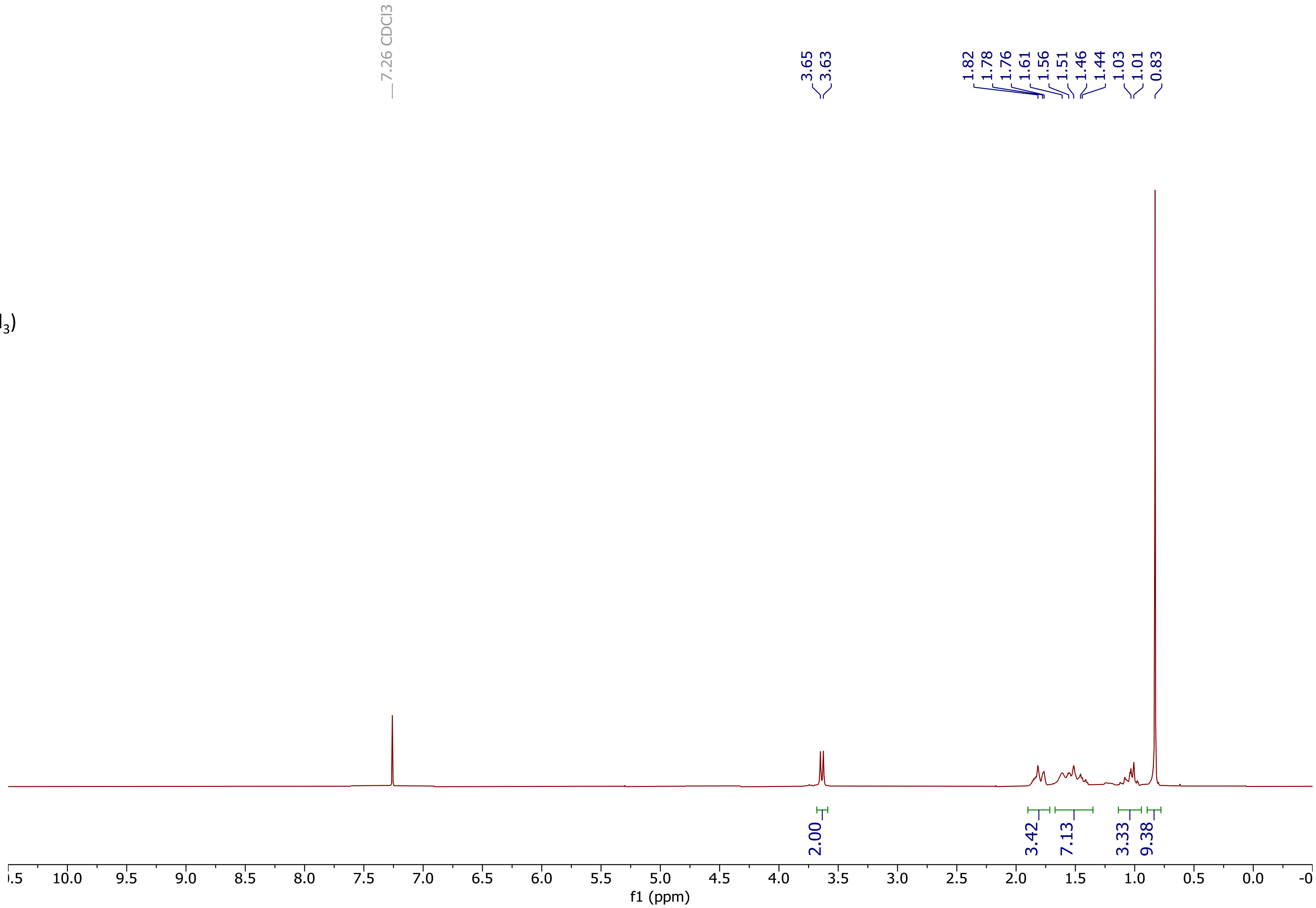

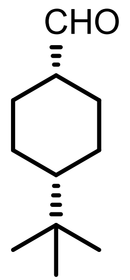

^{tBu}*cis*-**1e-OH**

-crude-

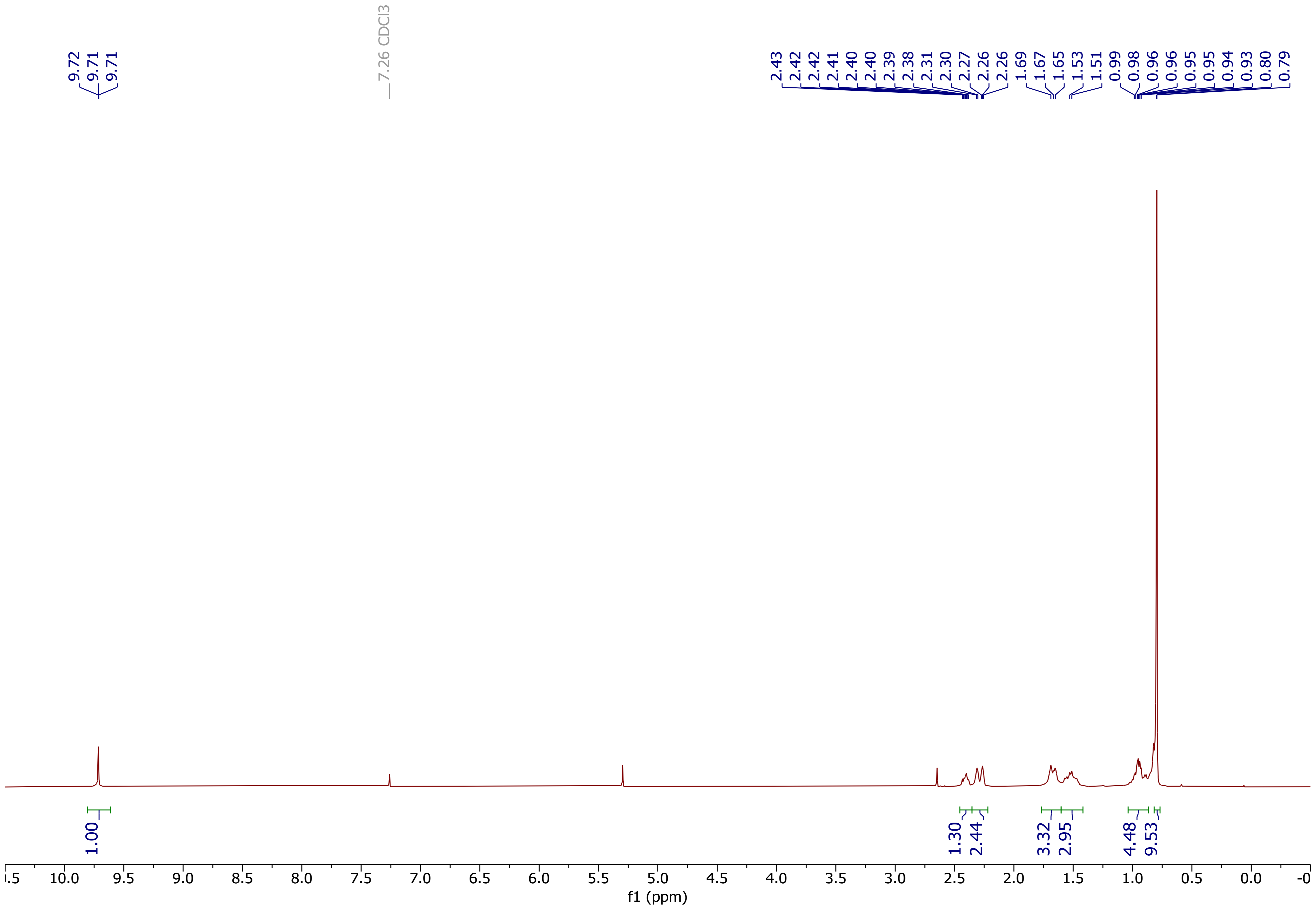

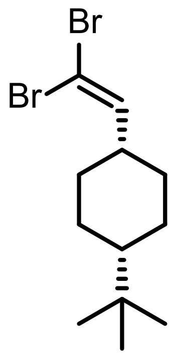

tBu-*cis*-1e-CBr$_2$

^{1}H NMR(300 MHz, $CDCl_3$)

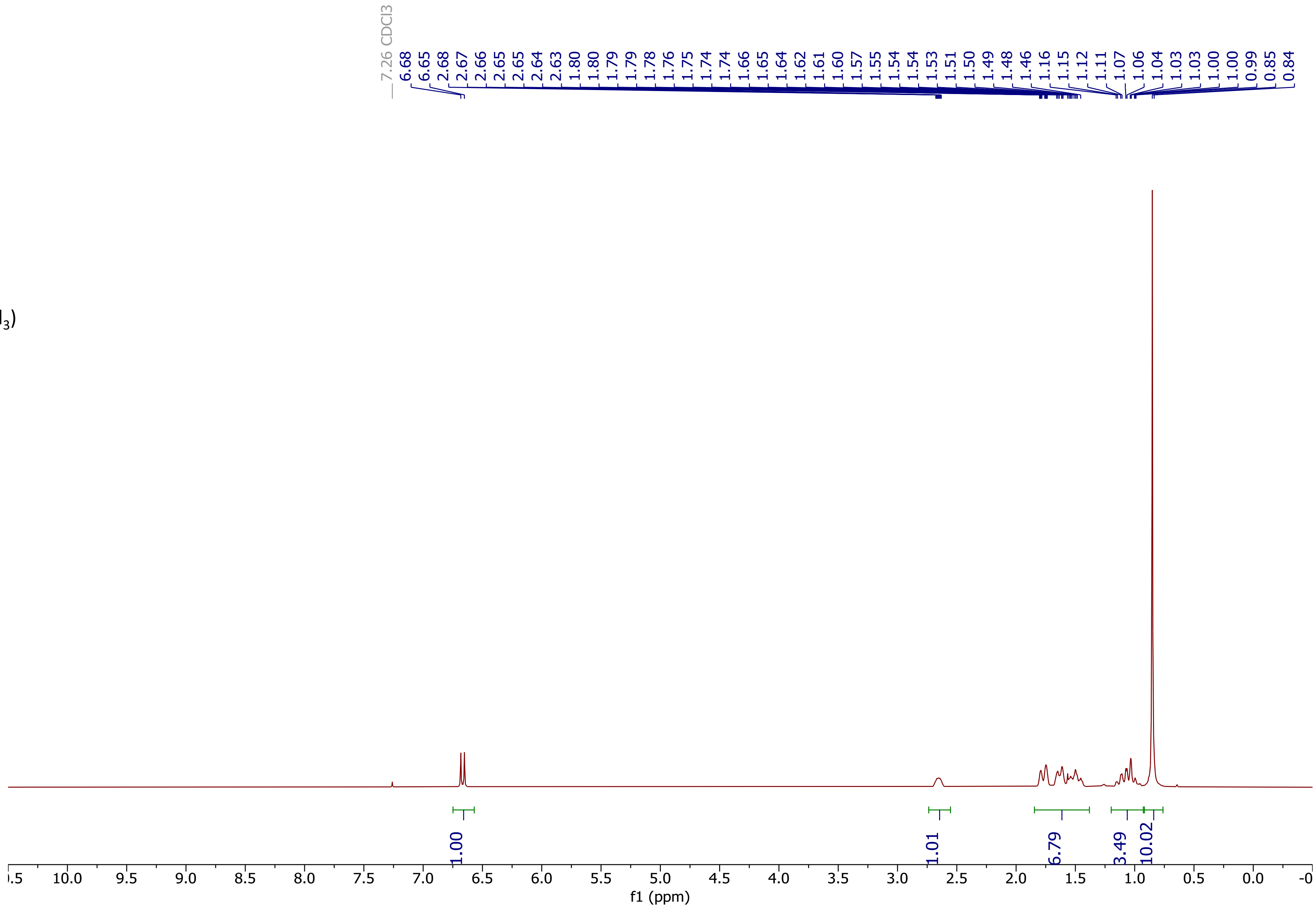

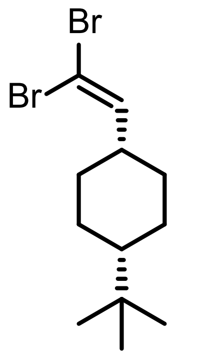

^{tBu}*cis*-**1e-CBr$_2$**

^{13}C NMR (75 MHz, $CDCl_3$)

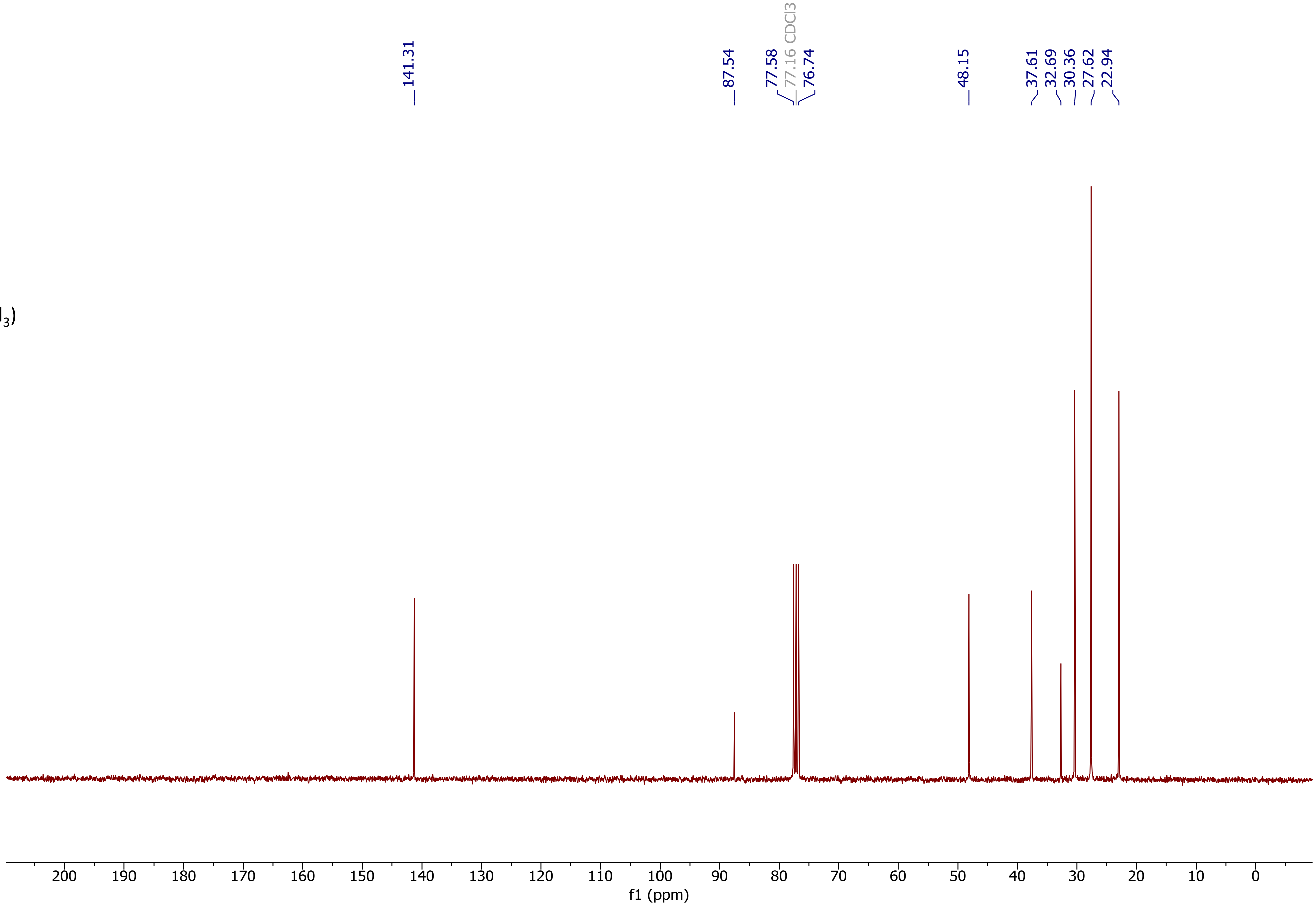

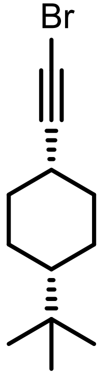

^{tBu}*cis*-1e

^{1}H NMR(300 MHz, $CDCl_3$)

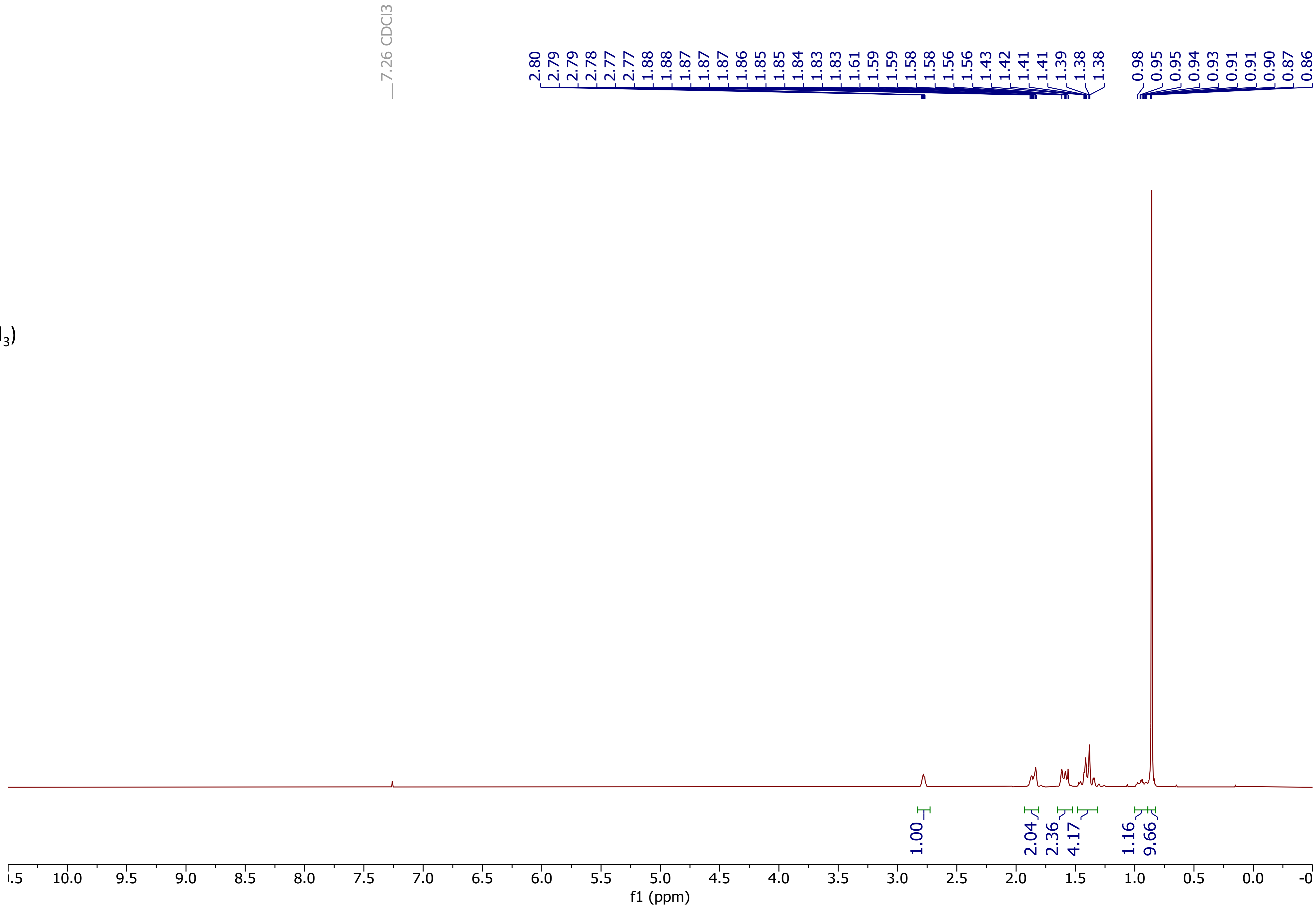

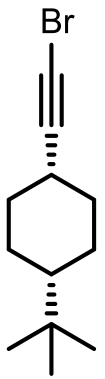

***tBu*cis-1e**

^{13}C NMR (75 MHz, $CDCl_3$)

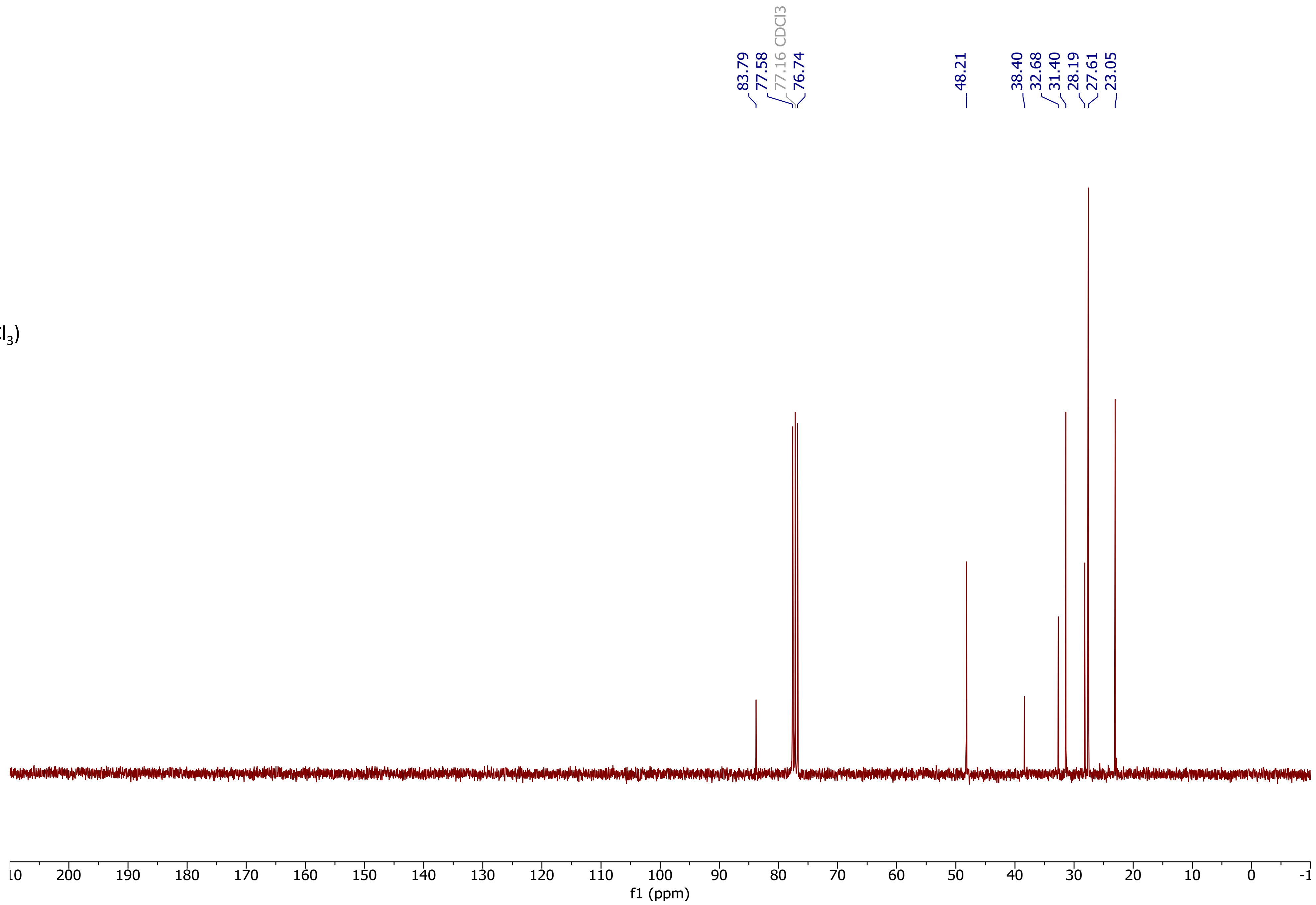

***trans*-1e**

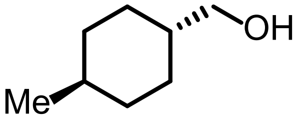

***Me*trans-1e-OH**

-crude-

^{1}H NMR(300 MHz, $CDCl_3$)

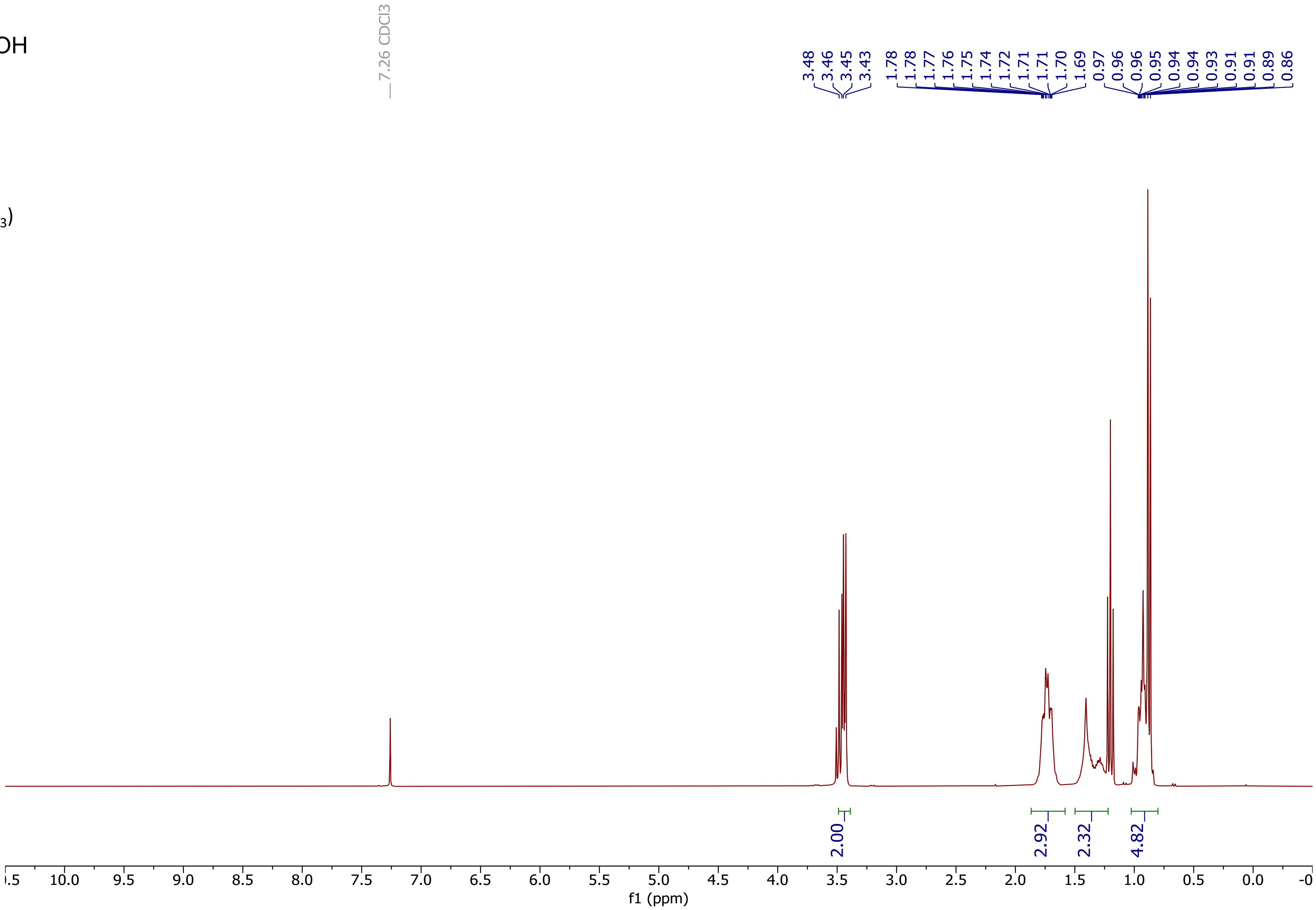

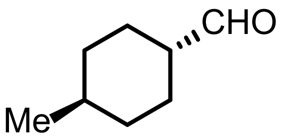

^{Me}***trans*-1e-CHO**

-crude-

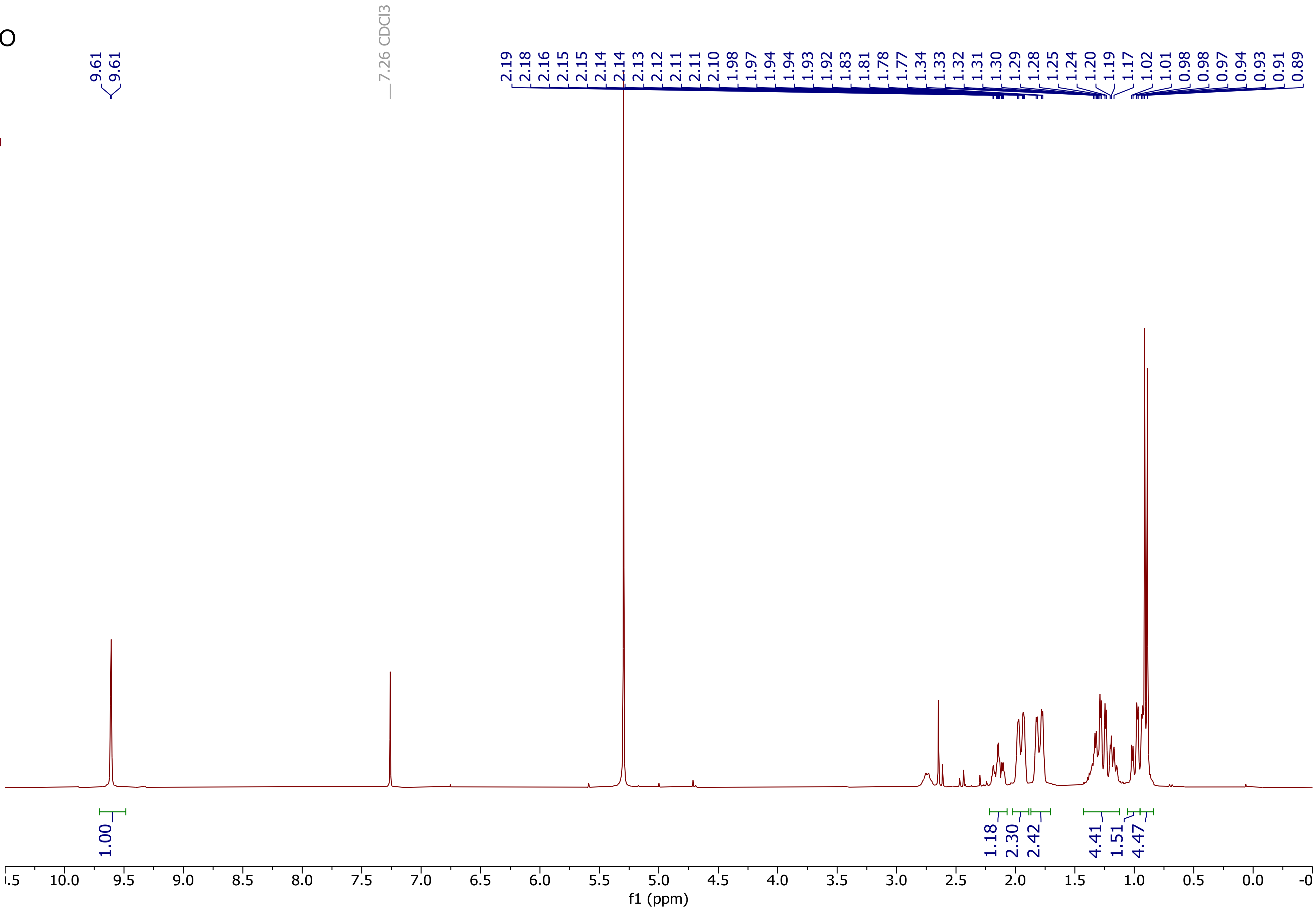

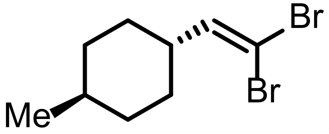

^{Me}***trans*-1e-CBr$_2$**

^{1}H NMR(300 MHz, $CDCl_3$)

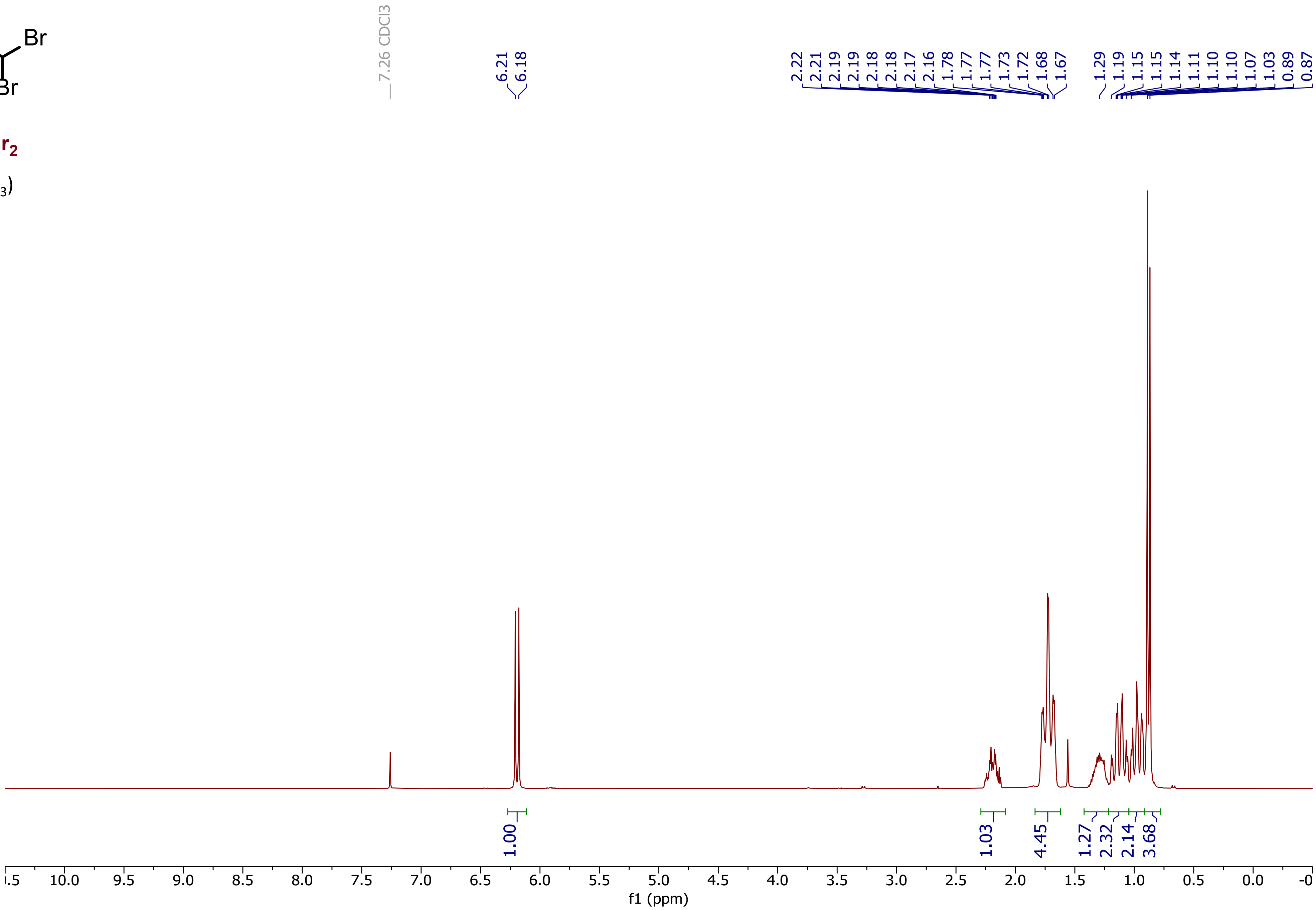

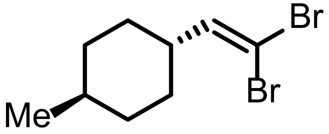

Me**trans*-1e-CBr$_2$**

^{13}C NMR (75 MHz, $CDCl_3$)

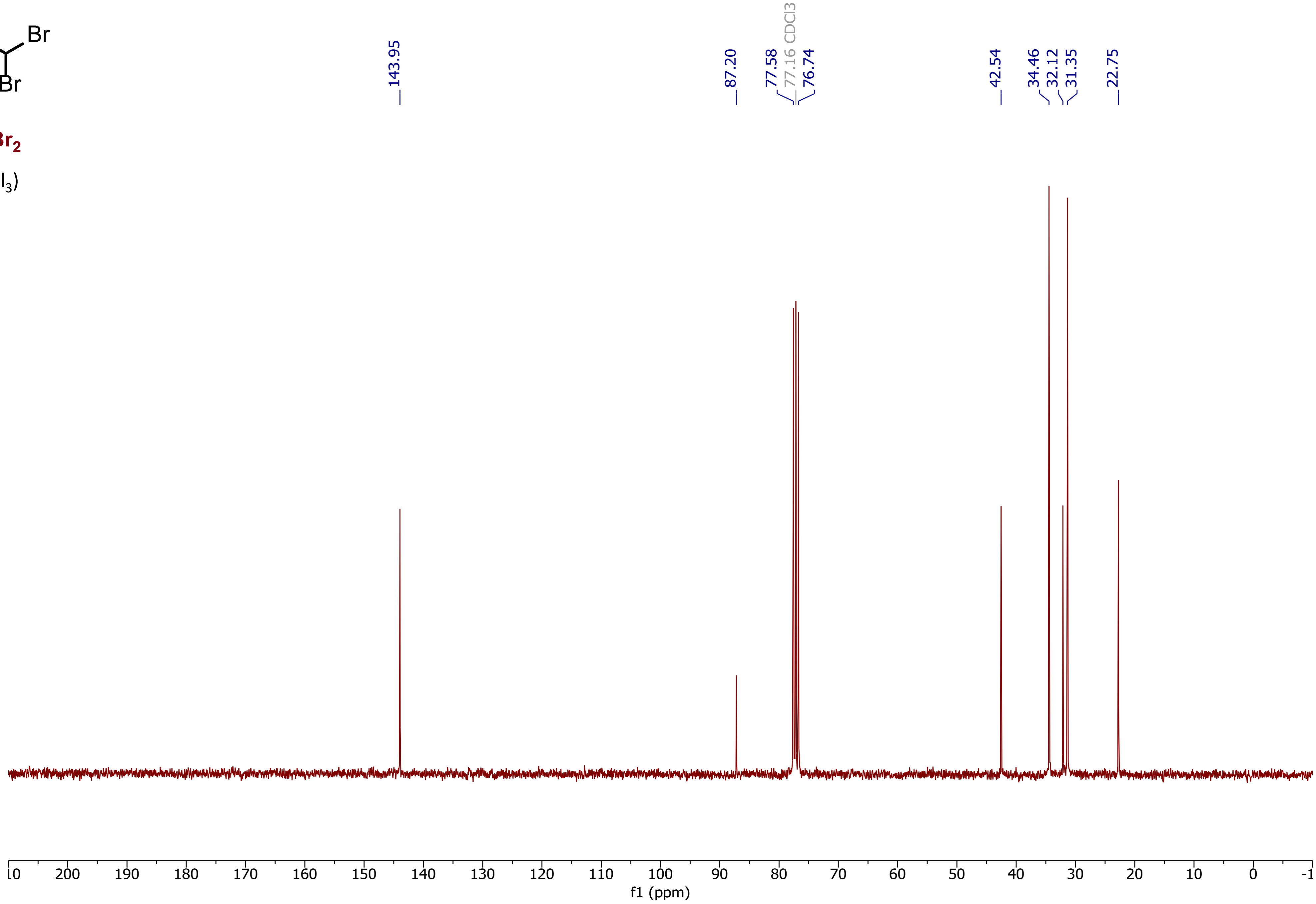

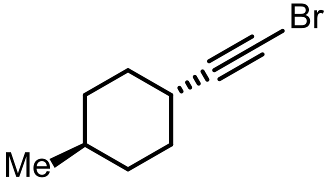

^{Me}*trans*-1e

^{1}H NMR(300 MHz, $CDCl_3$)

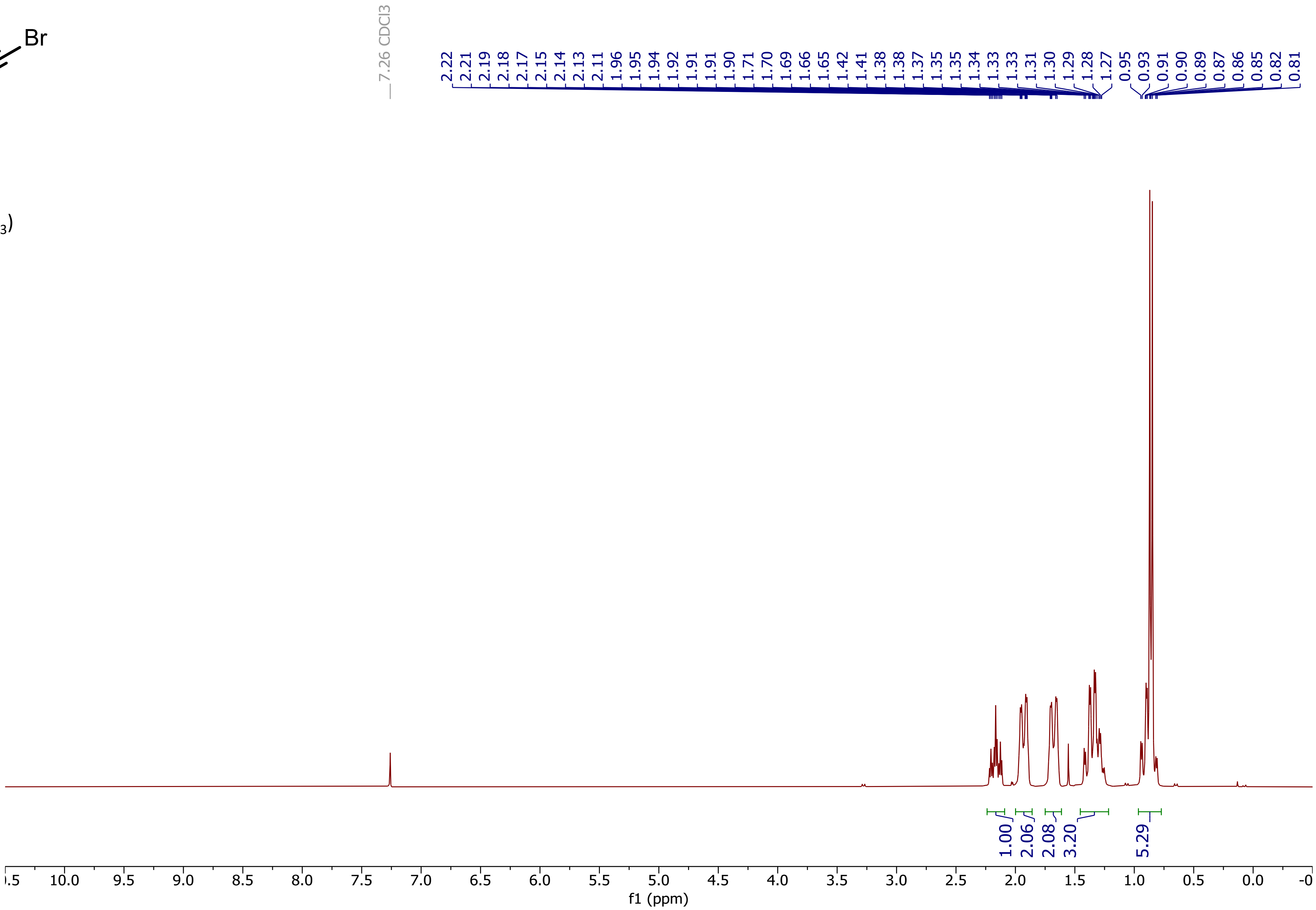

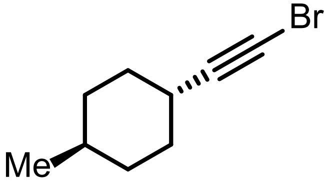

^{Me}***trans*-1e**

^{13}C NMR (75 MHz, $CDCl_3$)

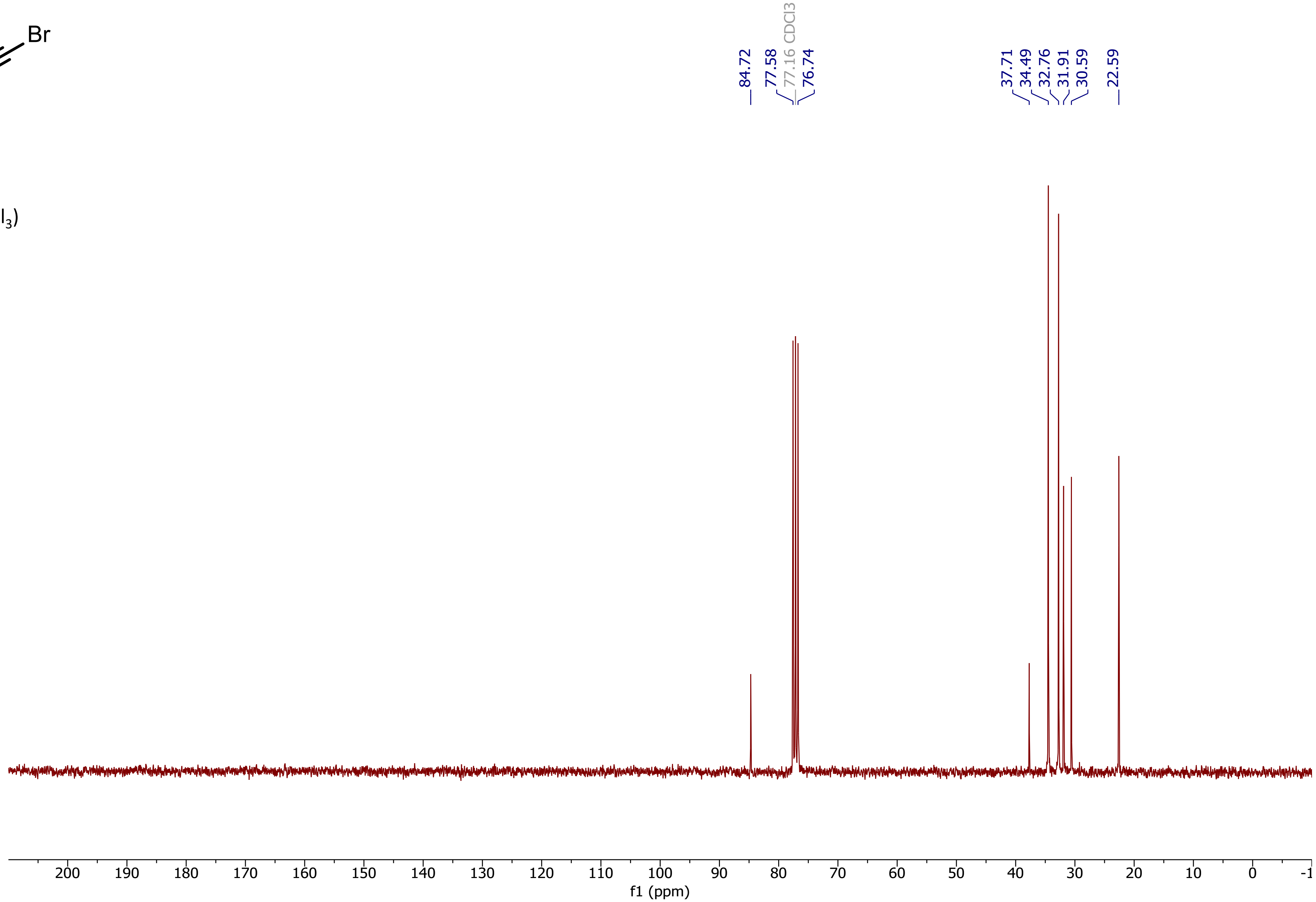

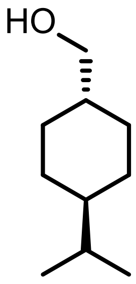

^{iPr}***trans*-1e-OH**

-crude-

^{1}H NMR(300 MHz, $CDCl_3$)

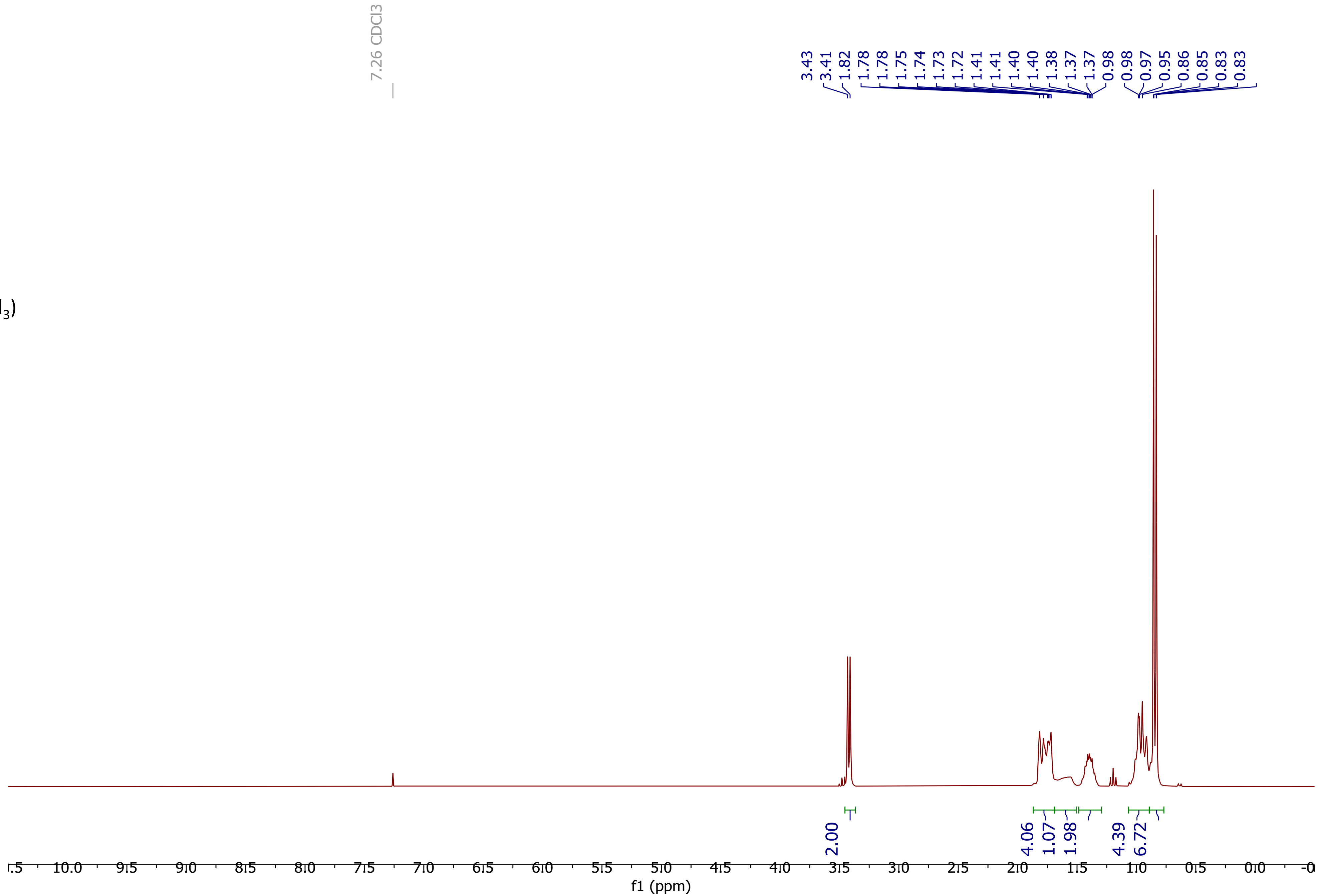

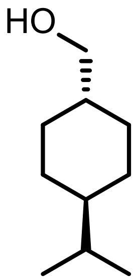

^{iPr}***trans*-1e-OH**

-crude-

^{13}C NMR (75 MHz, $CDCl_3$)

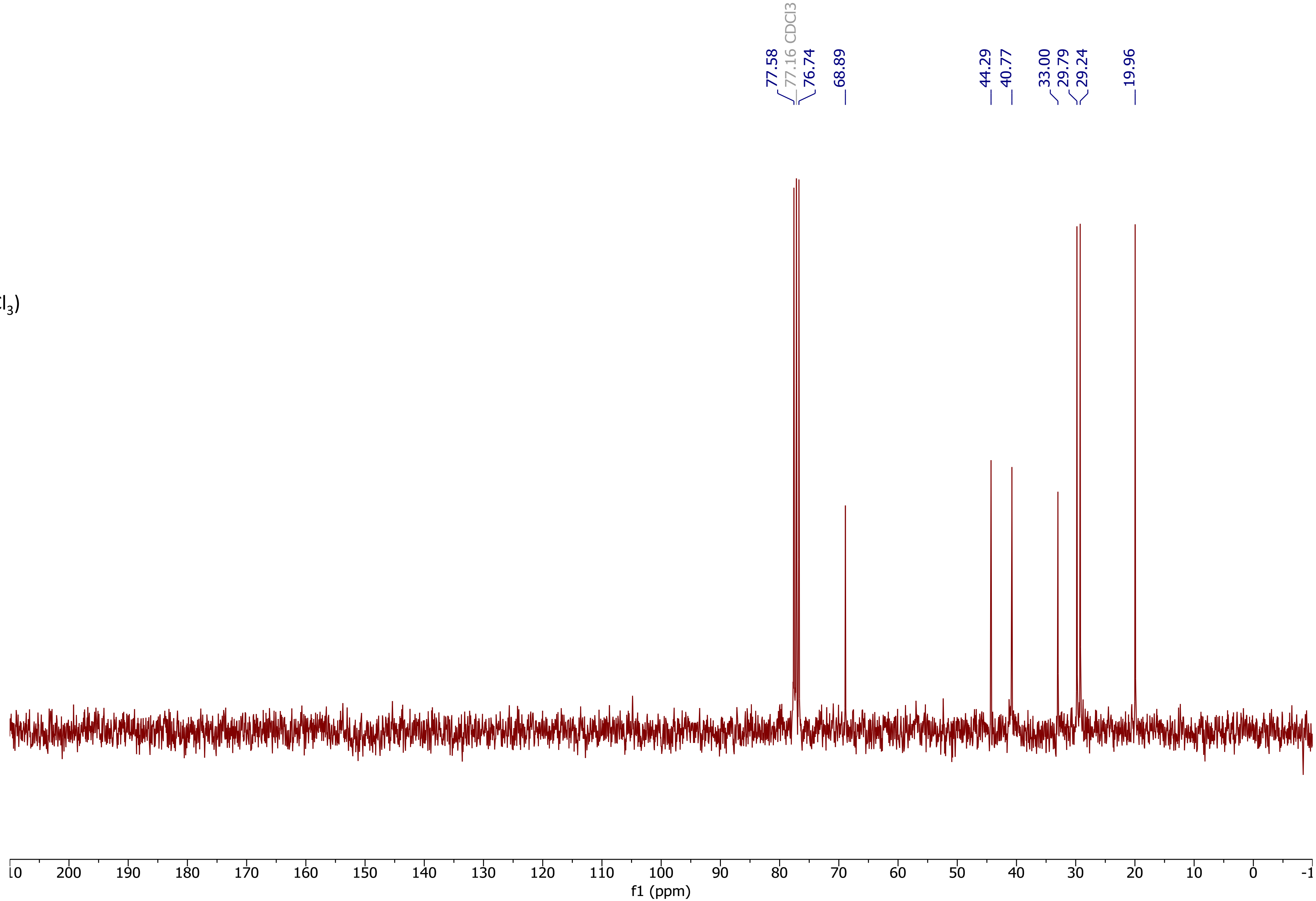

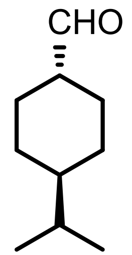

^{iPr}*trans*-1e-**CHO**

-crude-

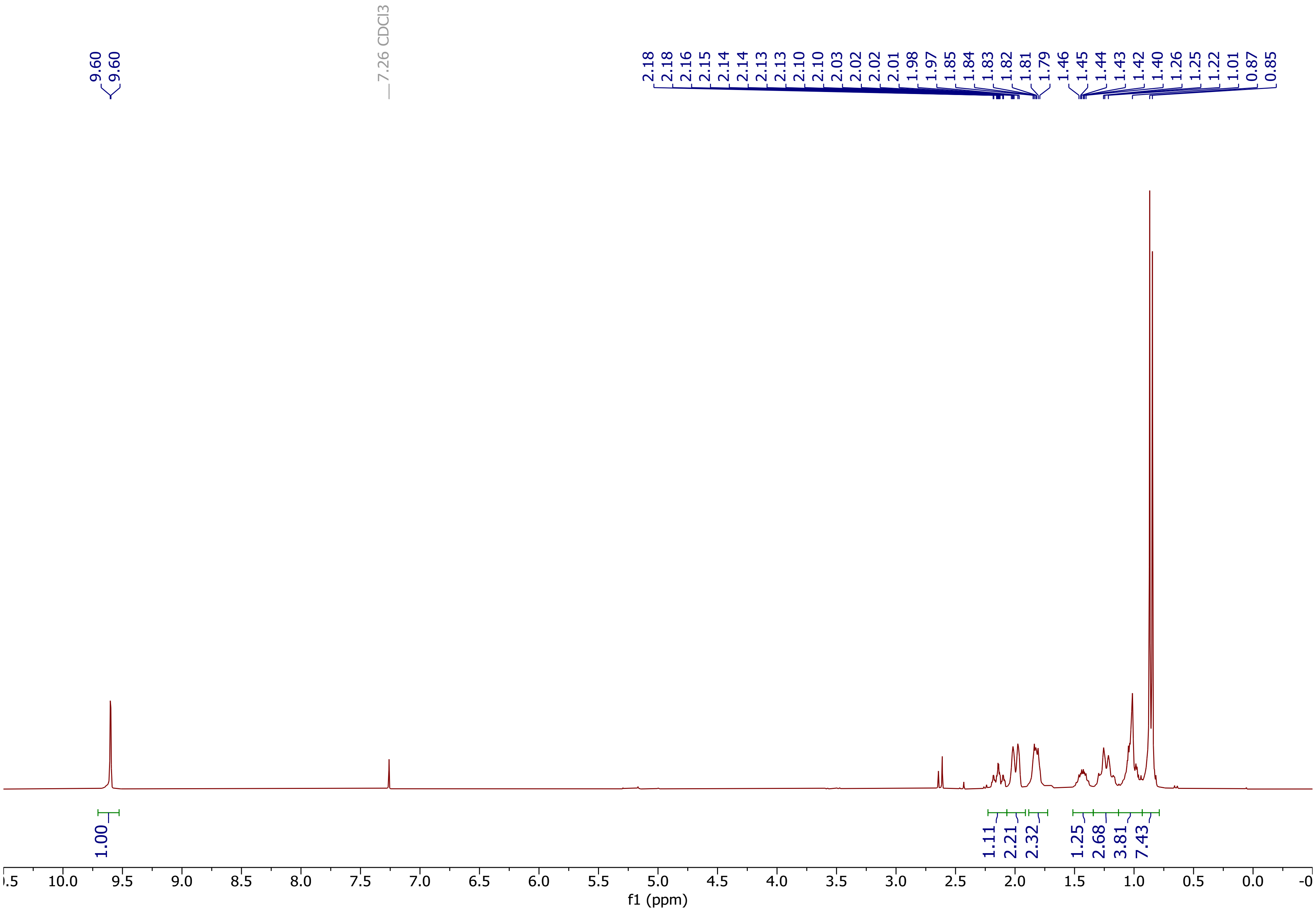

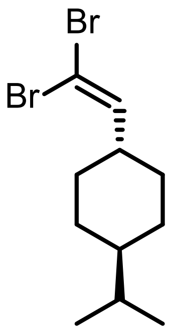

^{iPr}***trans*-1e-CBr$_2$**

^{1}H NMR(300 MHz, $CDCl_3$)

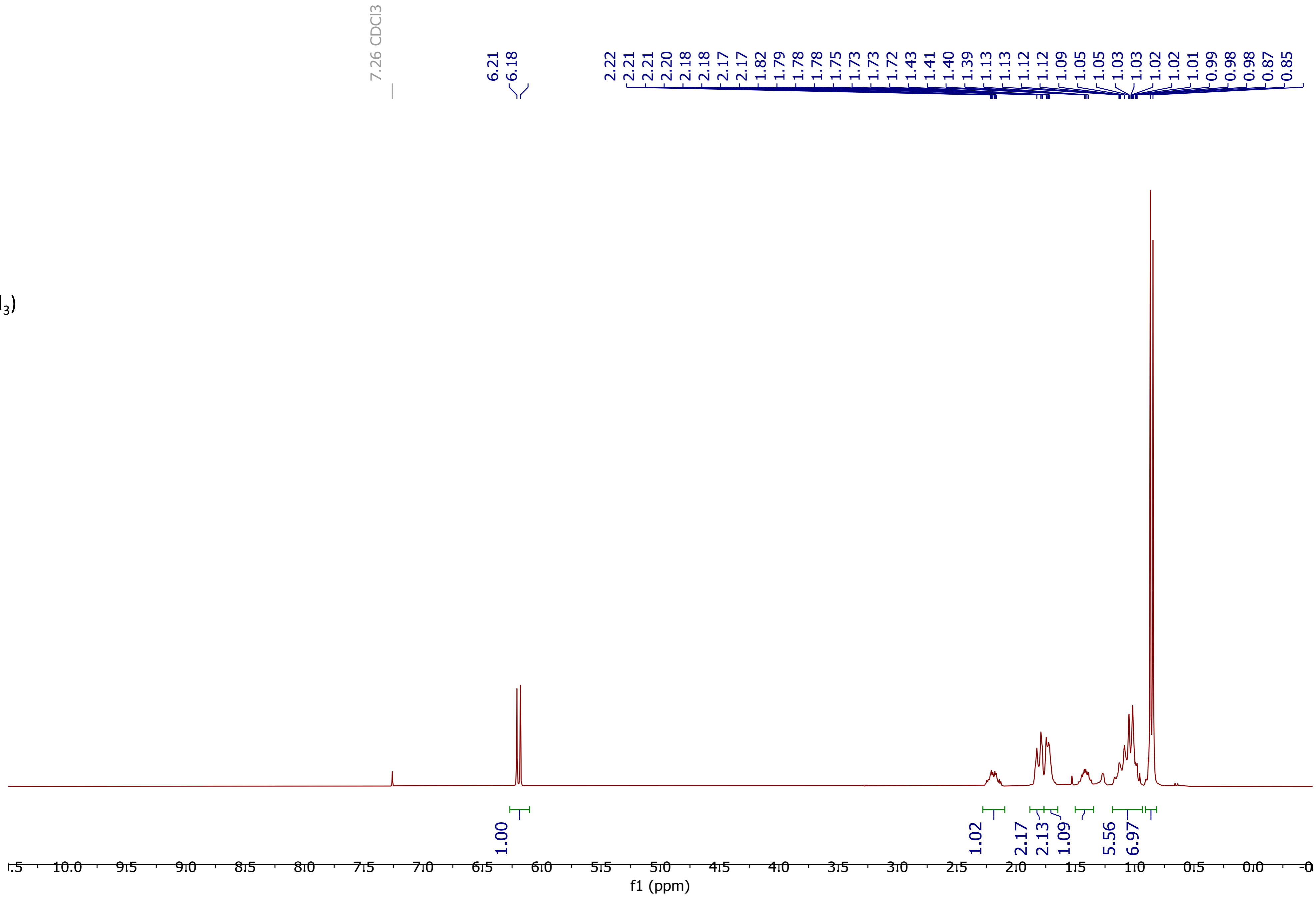

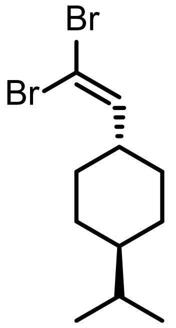

^{iPr}***trans*-1e-CBr$_2$**

^{13}C NMR (75 MHz, CDCl$_3$)

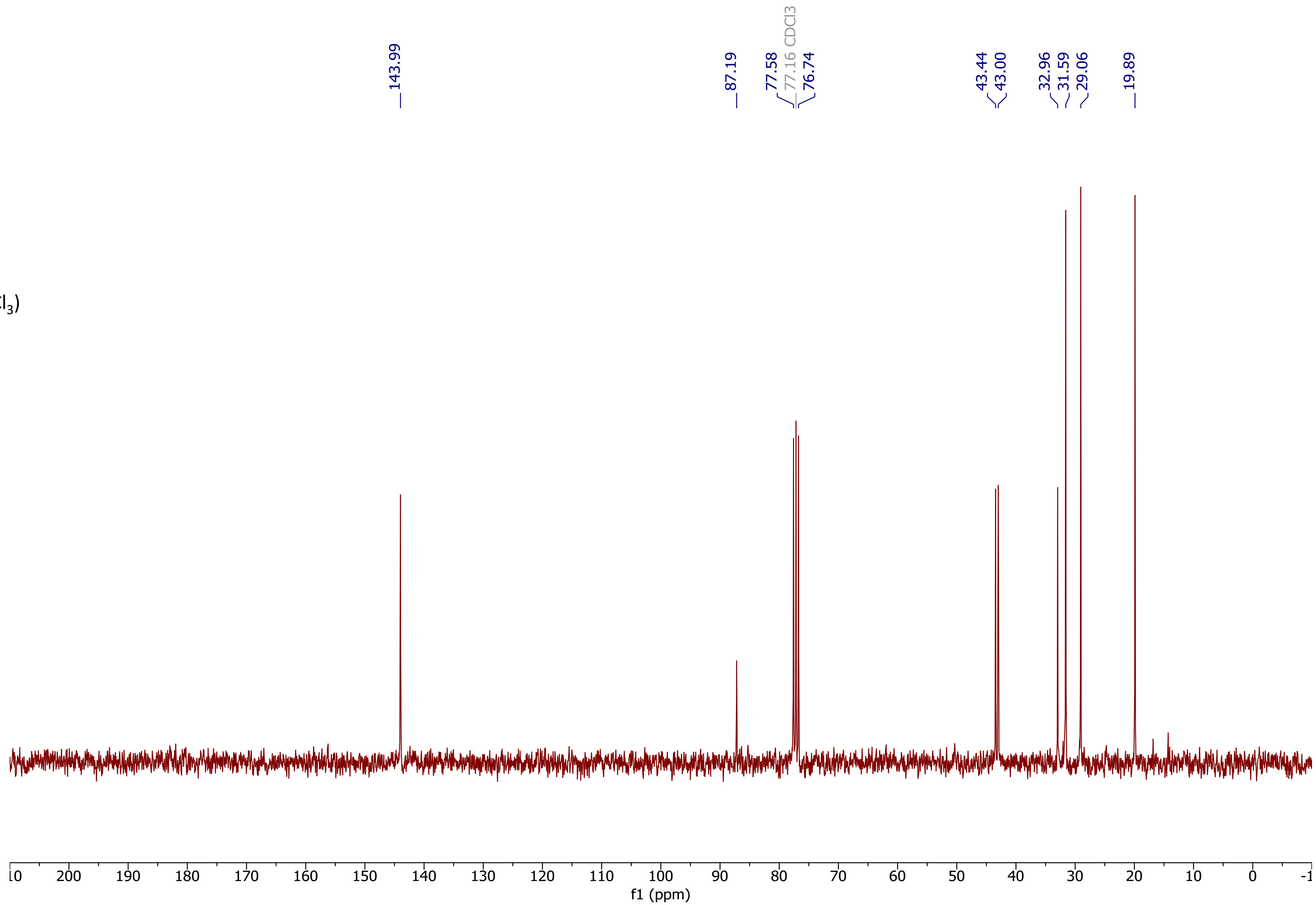

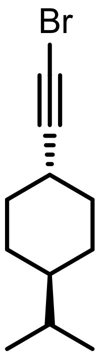

^{iPr}trans-**1e**

^{1}H NMR(300 MHz, $CDCl_3$)

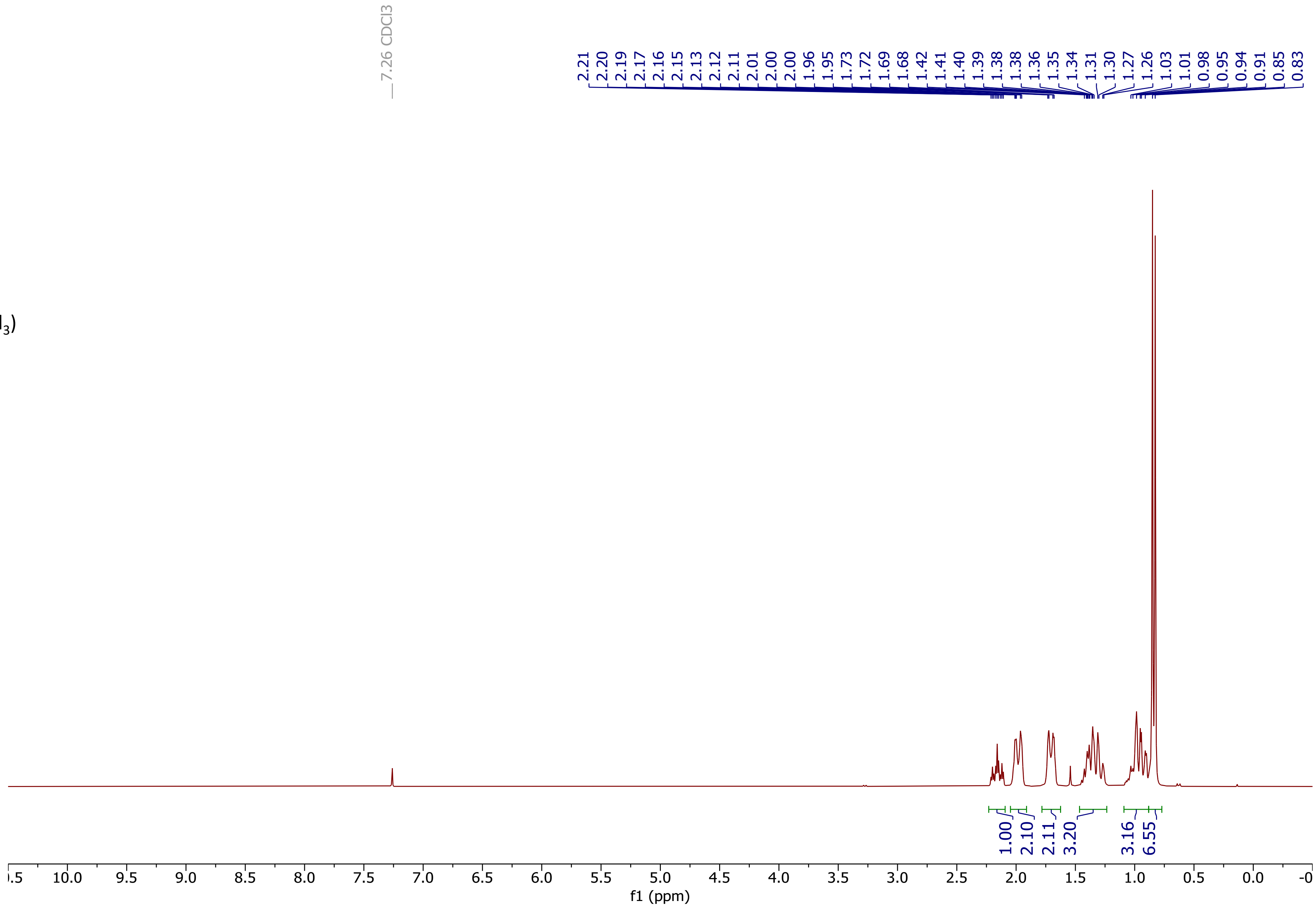

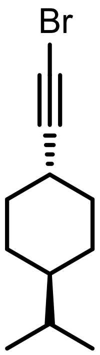

[iPr]trans-**1e**

^{13}C NMR (75 MHz, $CDCl_3$)

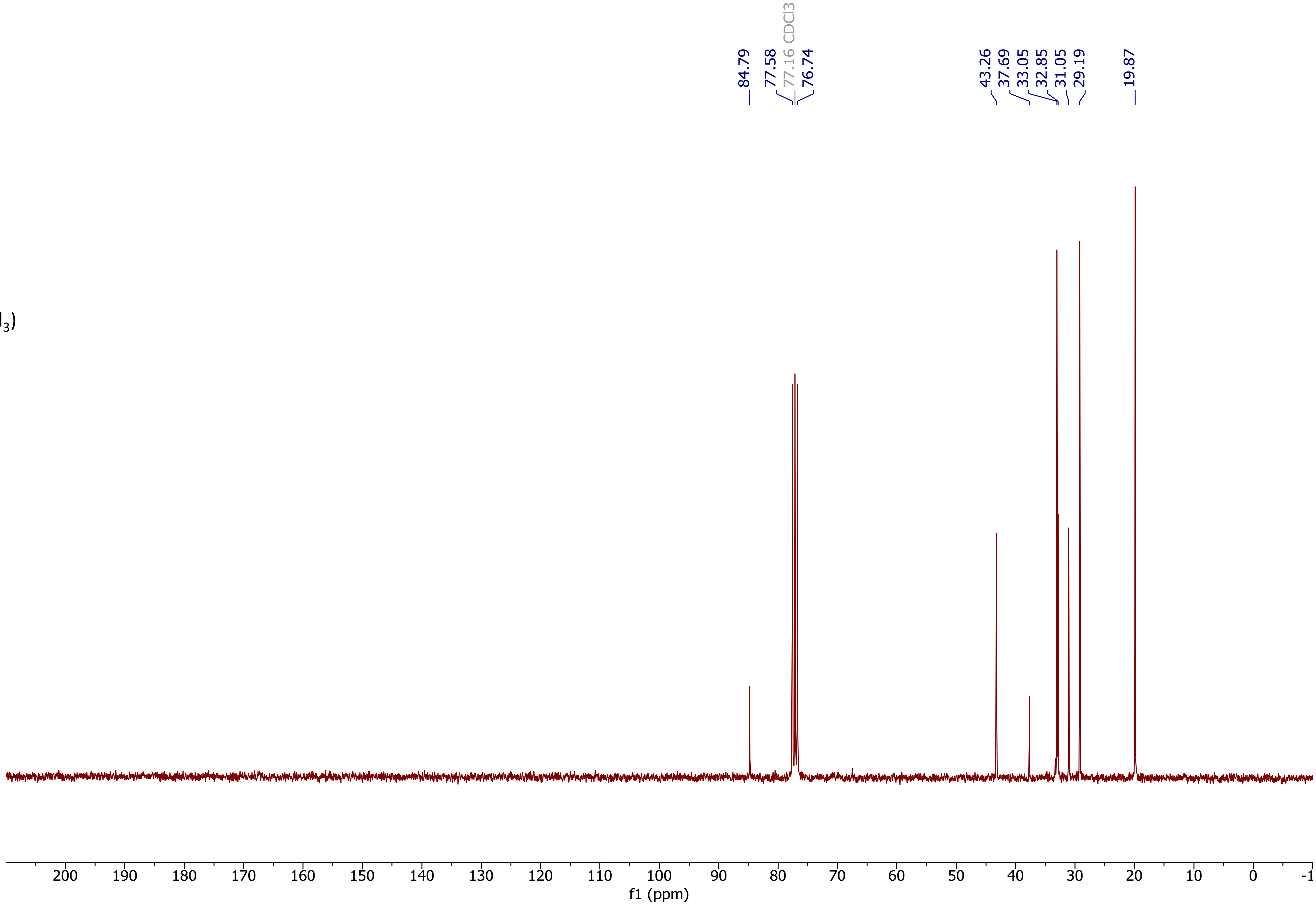

3b

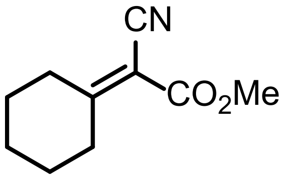

3b-CN, CO_2Me

^{1}H NMR(300 MHz, $CDCl_3$)

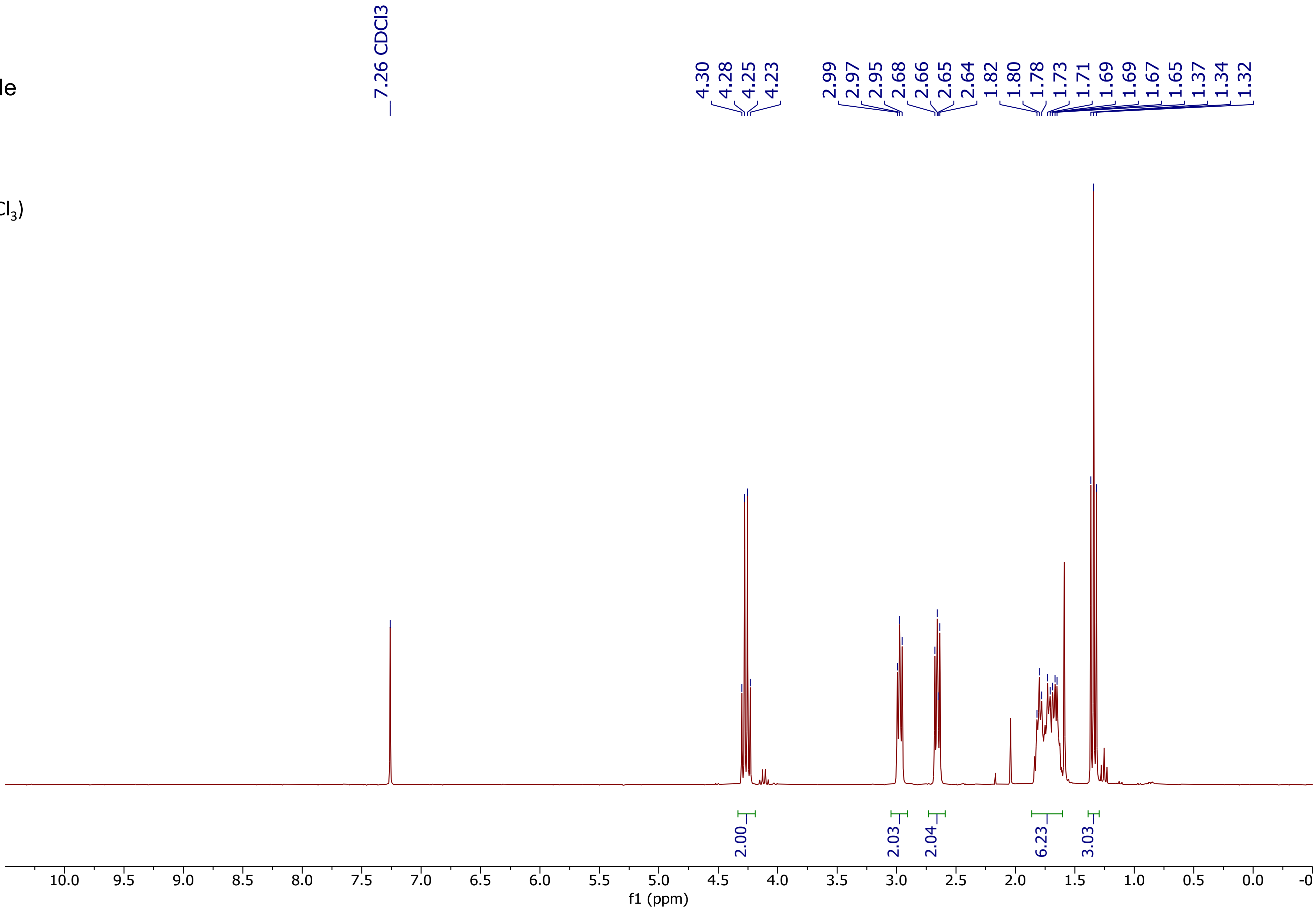

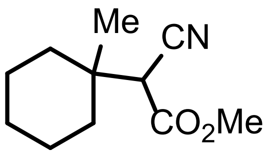

3b-CN, CO_2Me'

-crude-

^{1}H NMR(300 MHz, $CDCl_3$)

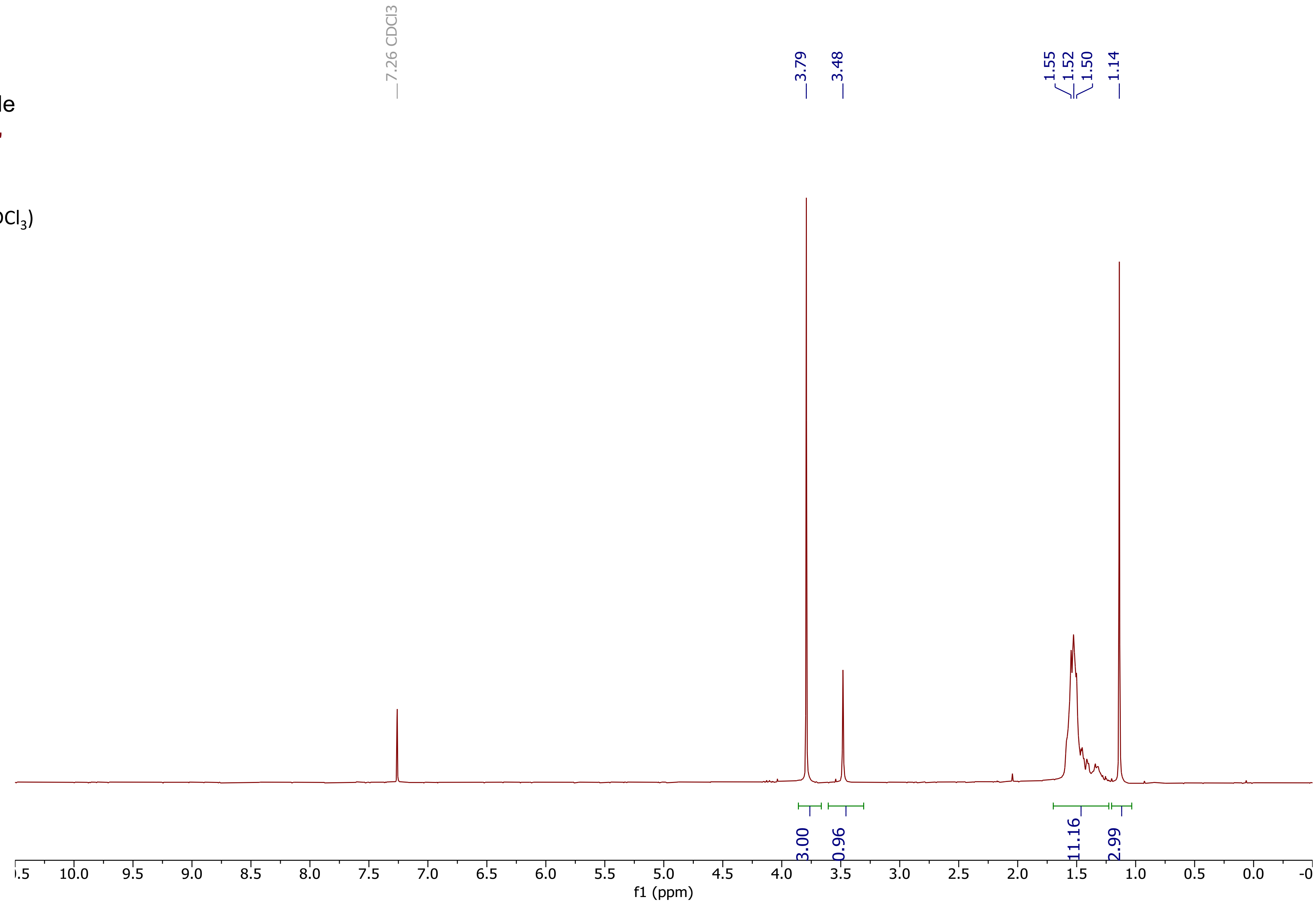

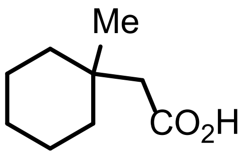

3b-CO_2H

-crude-

^{1}H NMR(300 MHz, $CDCl_3$)

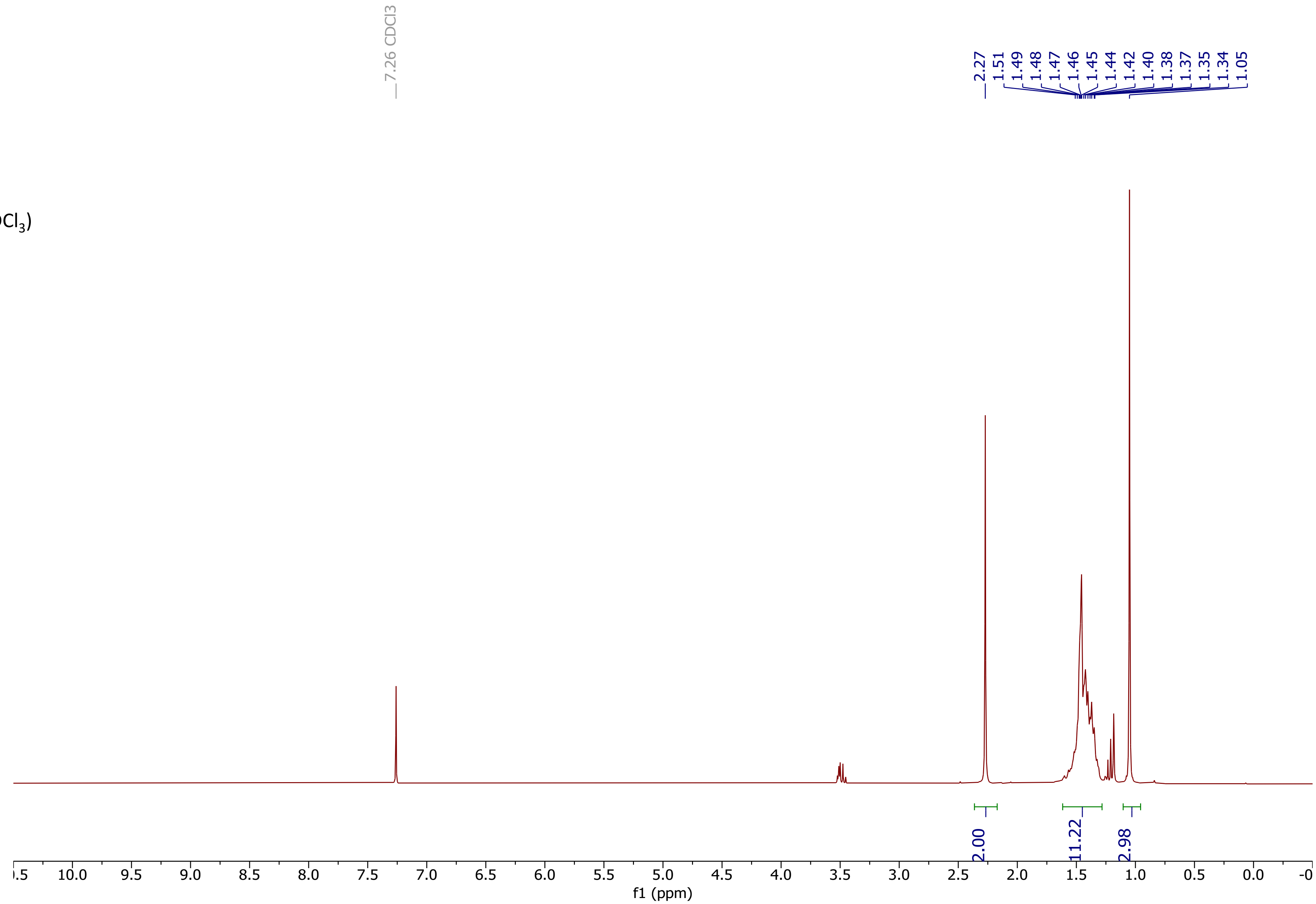

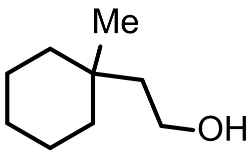

3b-OH

-crude-

^{1}H NMR(300 MHz, $CDCl_3$)

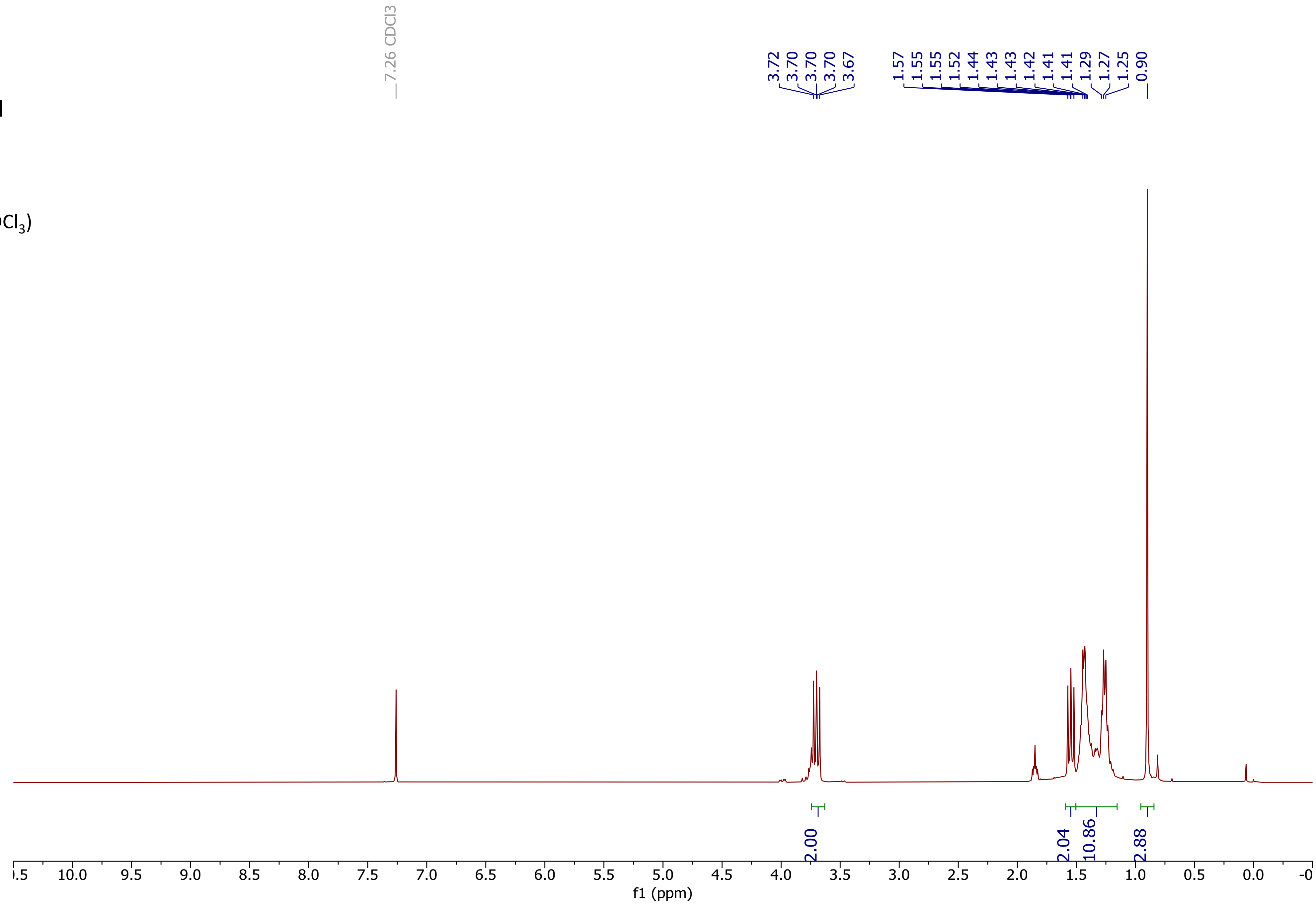

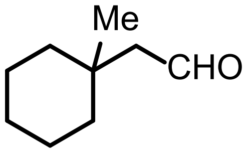

3b-CHO

-crude-

^{1}H NMR(300 MHz, $CDCl_3$)

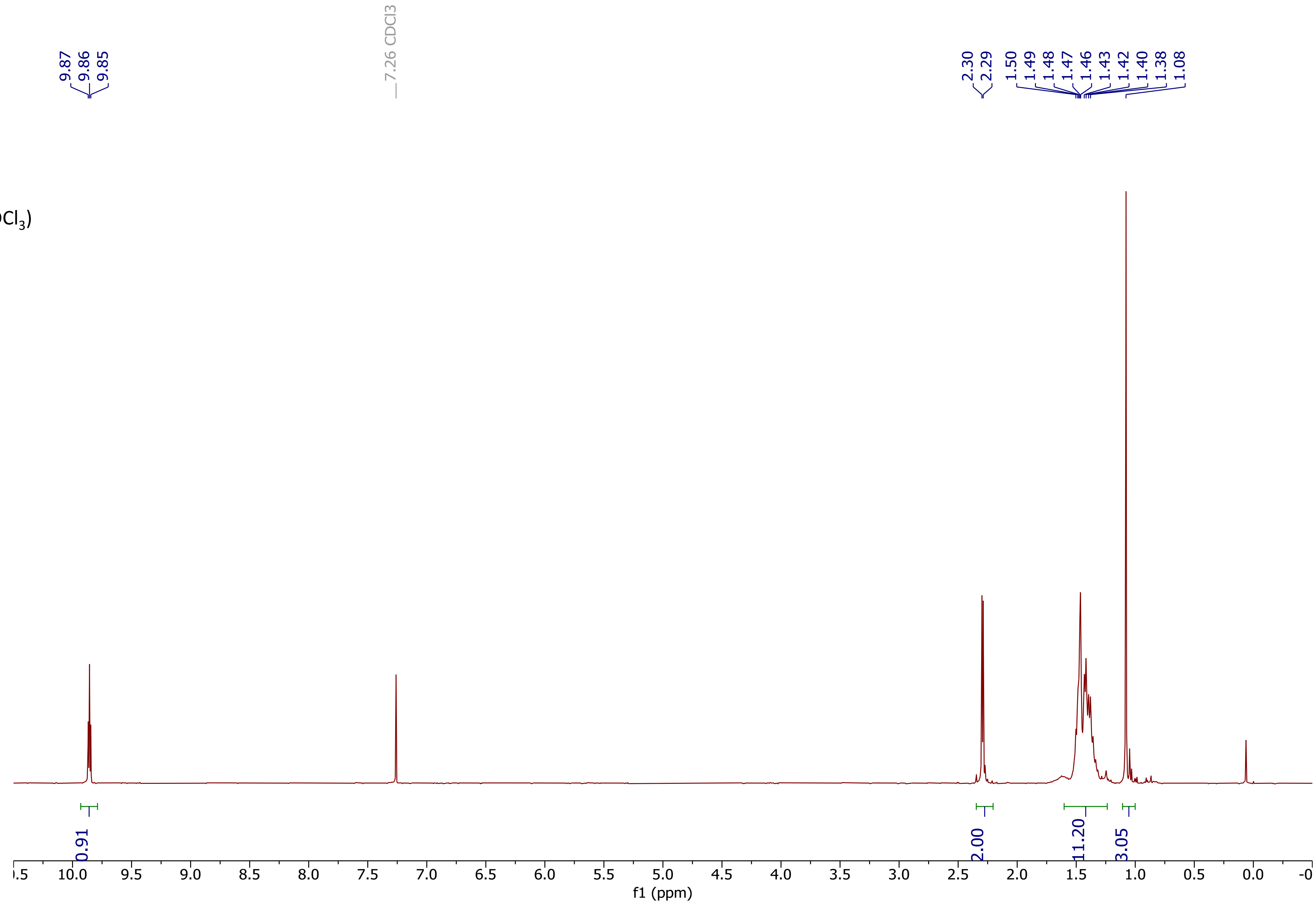

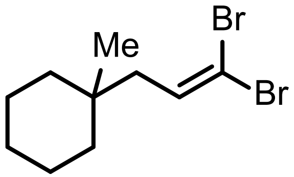

3b-CBr$_2$

^{1}H NMR(300 MHz, $CDCl_3$)

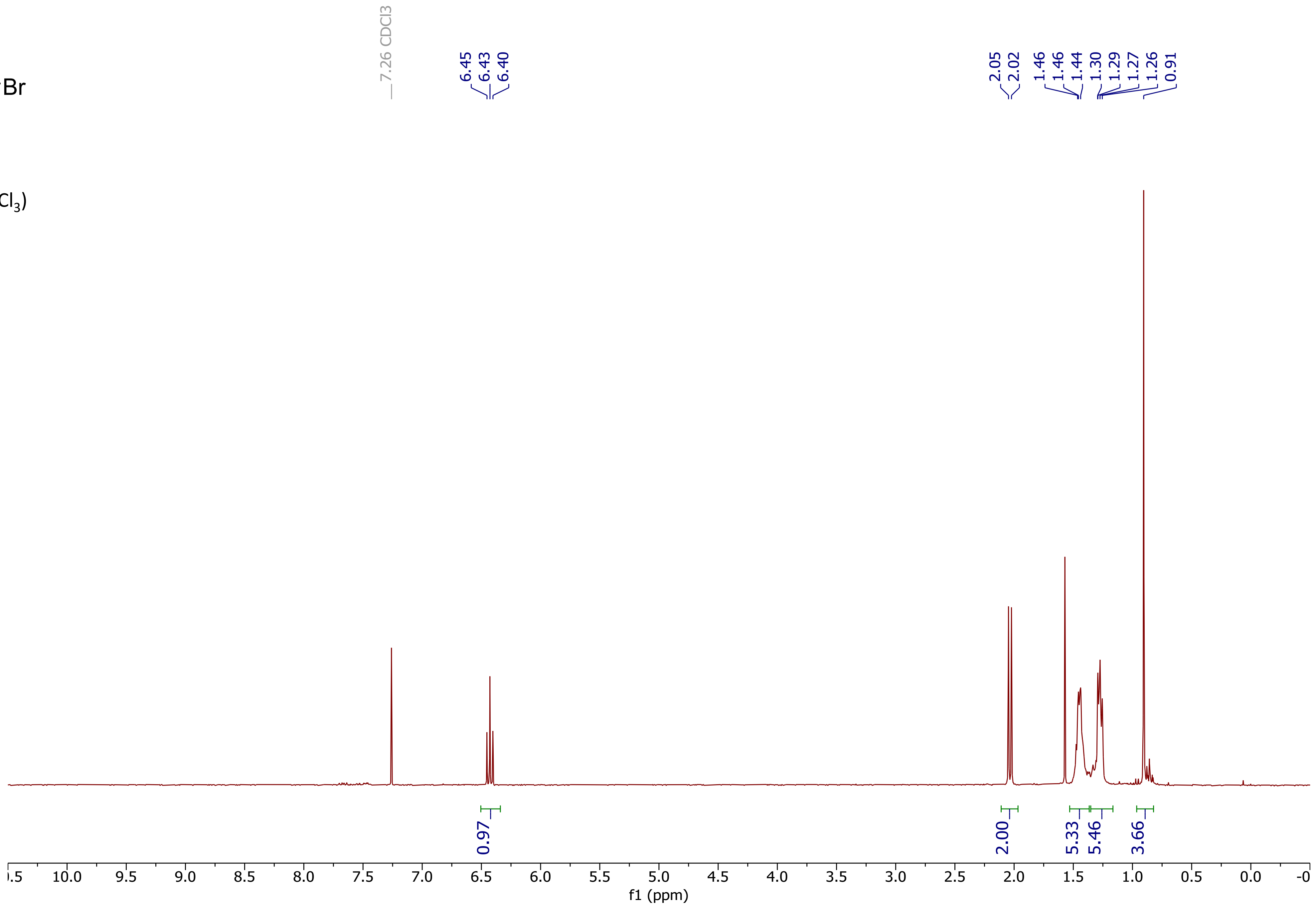

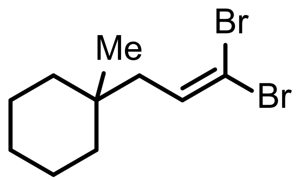

3b-CBr$_2$

^{13}C NMR (75 MHz, $CDCl_3$)

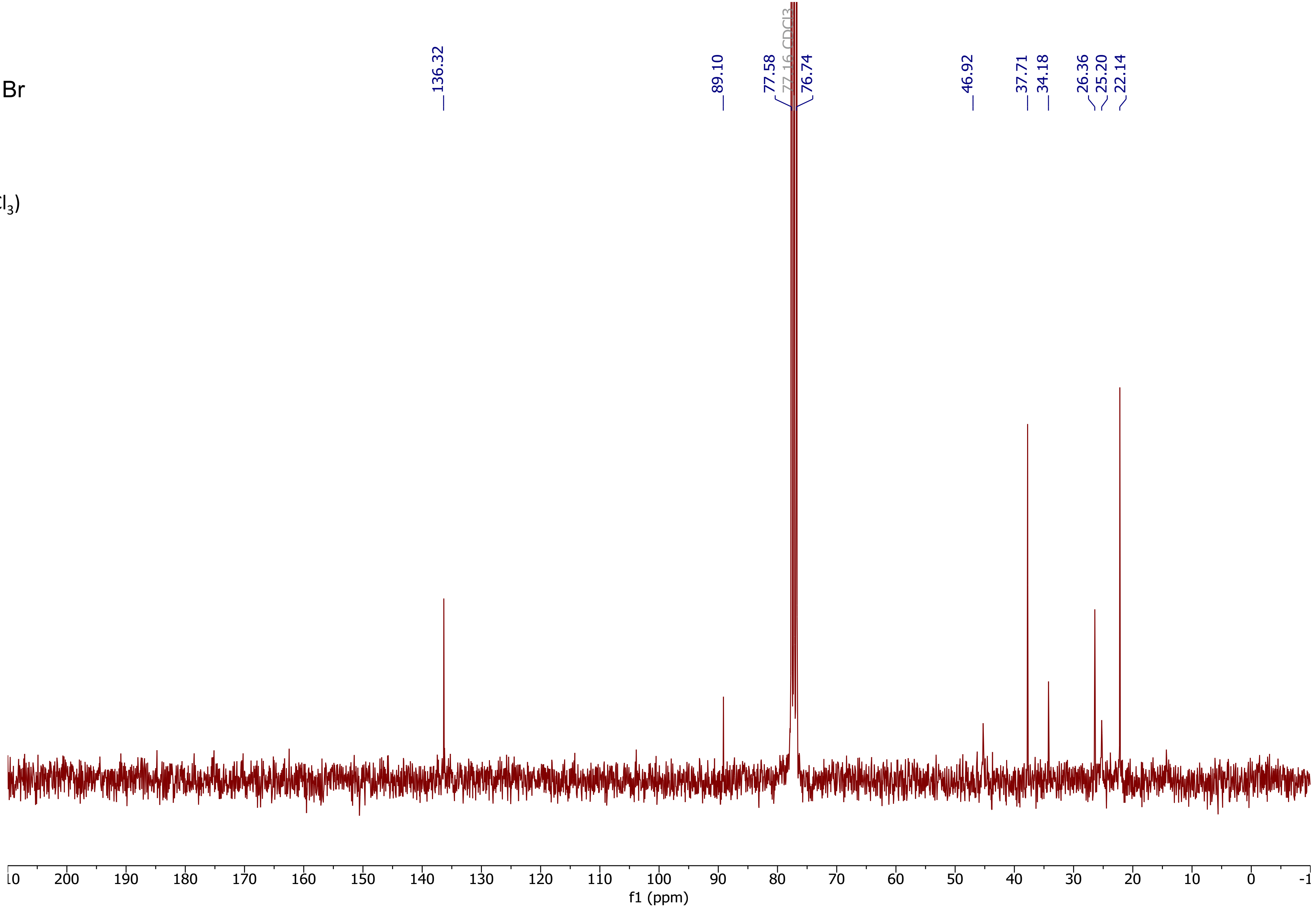

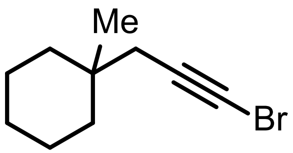

3b

^{1}H NMR(300 MHz, $CDCl_3$)

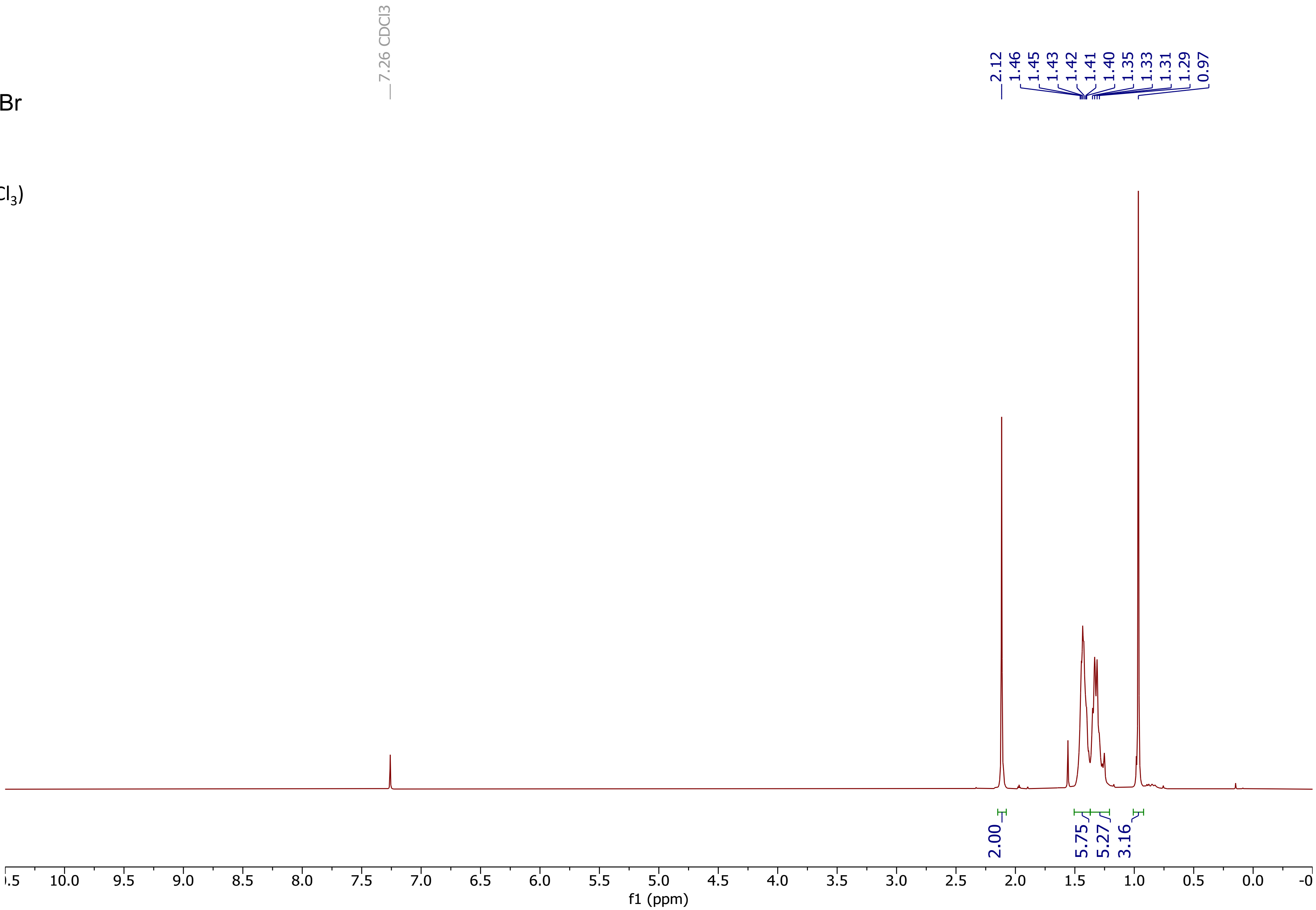

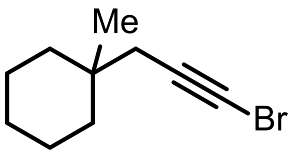

3b

^{13}C NMR (75 MHz, $CDCl_3$)

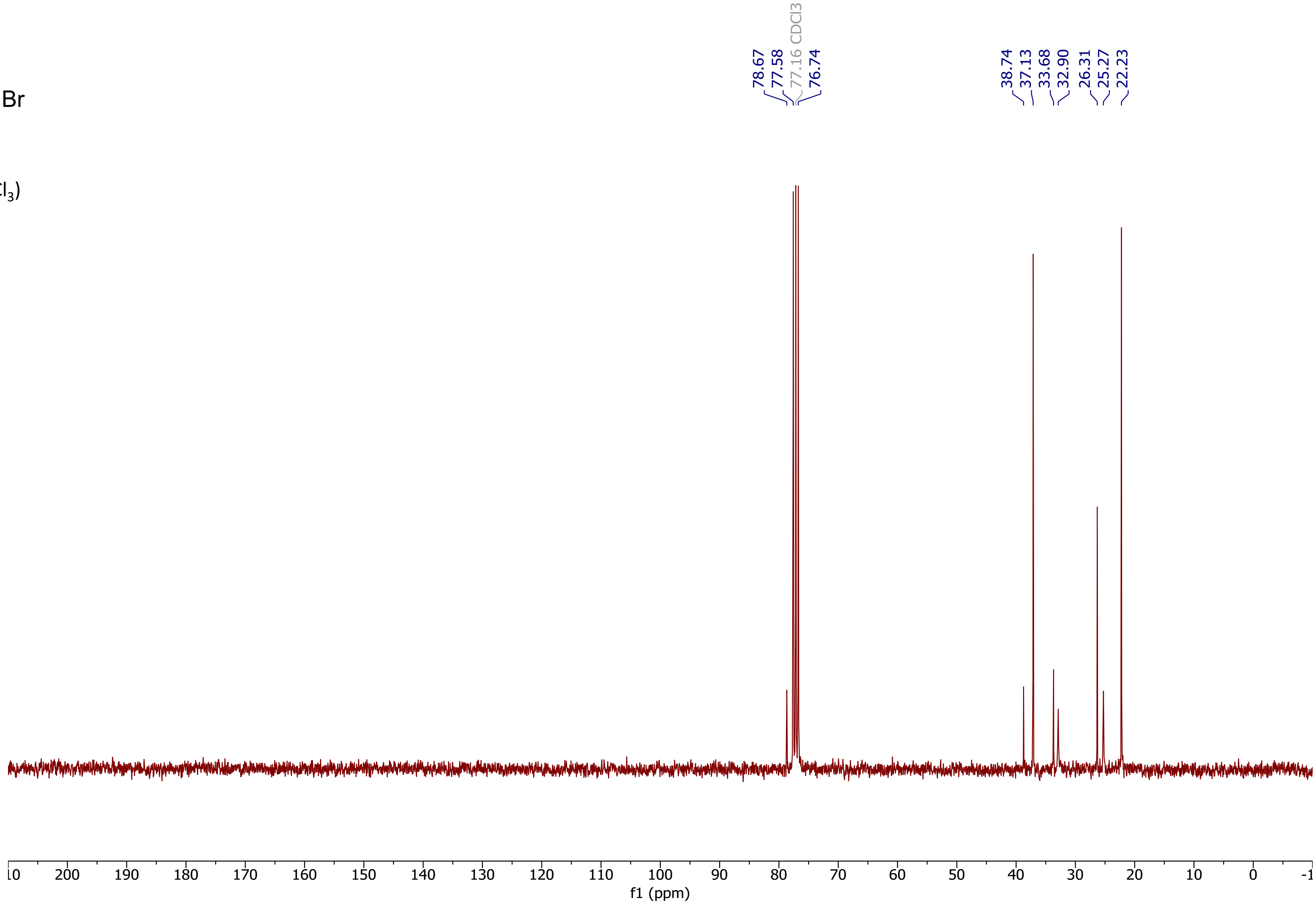

***cis*-3c**

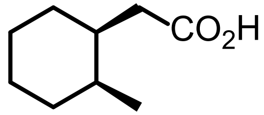

cis-3c-CO_2H

-crude-

^{1}H NMR(300 MHz, $CDCl_3$)

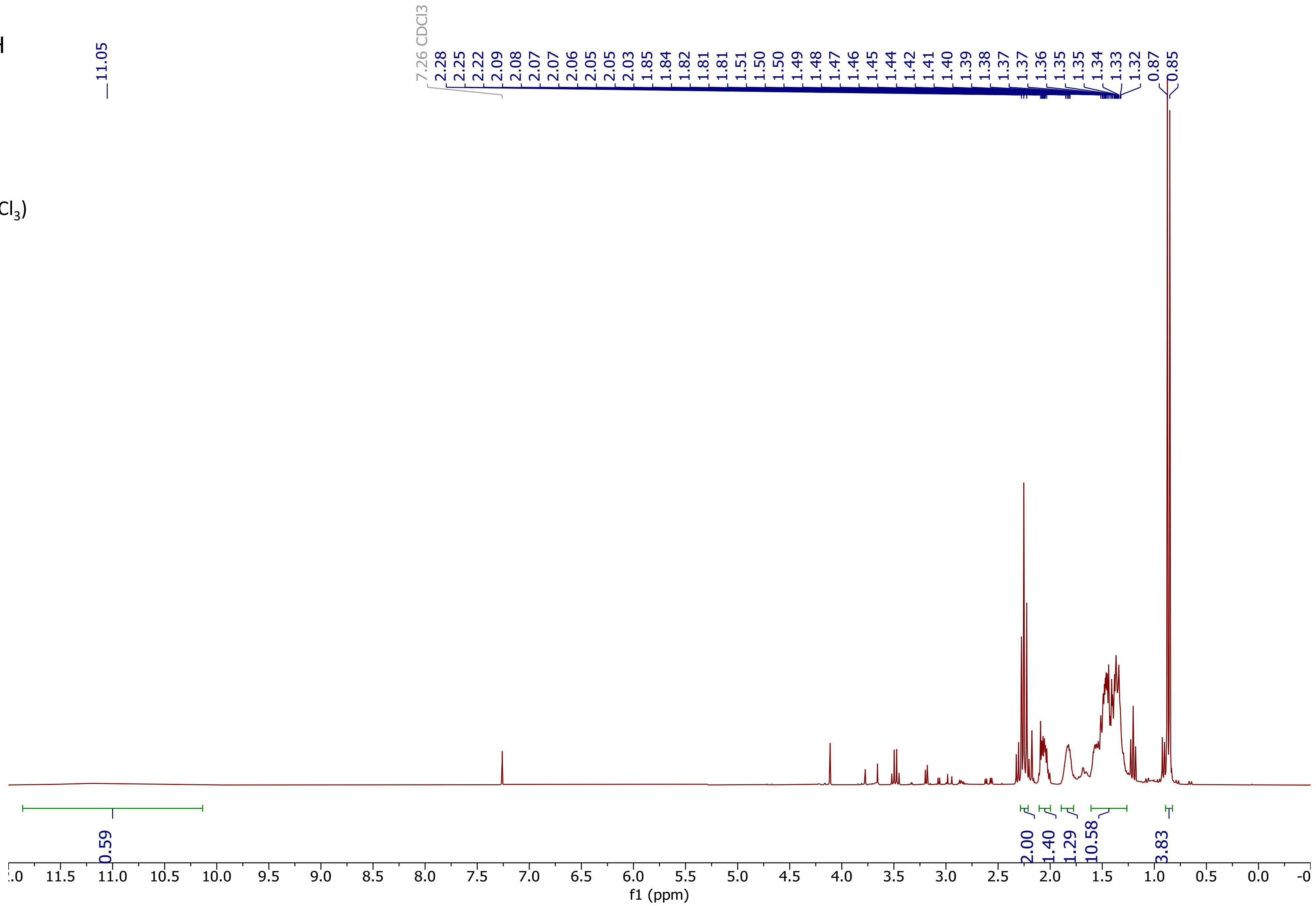

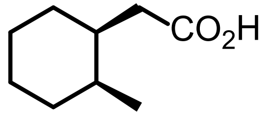

cis-3c-CO_2H

-crude-

^{13}C NMR (75 MHz, $CDCl_3$)

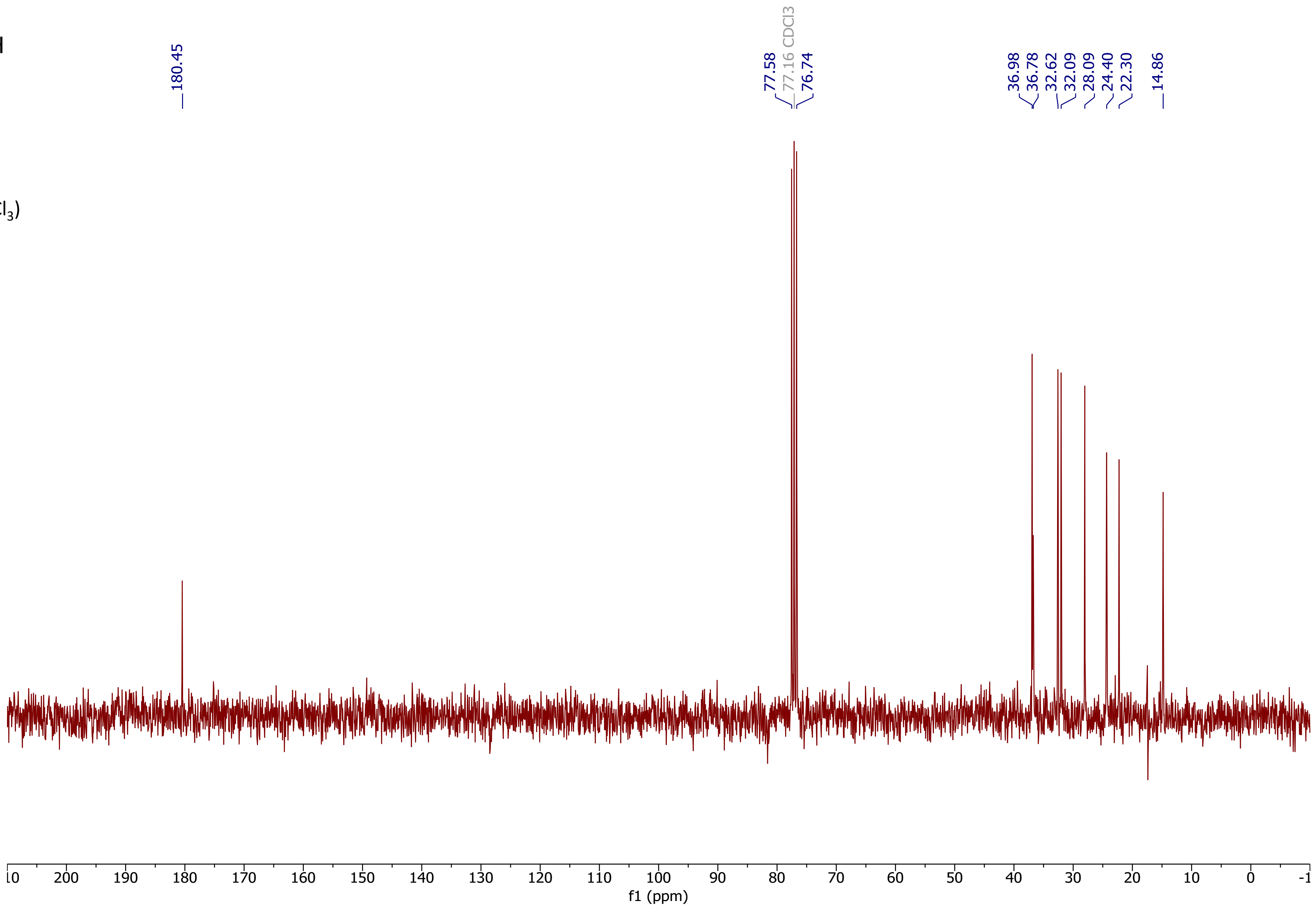

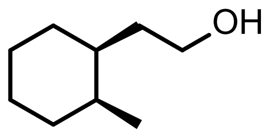

cis-3c-OH

-crude-

¹H NMR(300 MHz, $CDCl_3$)

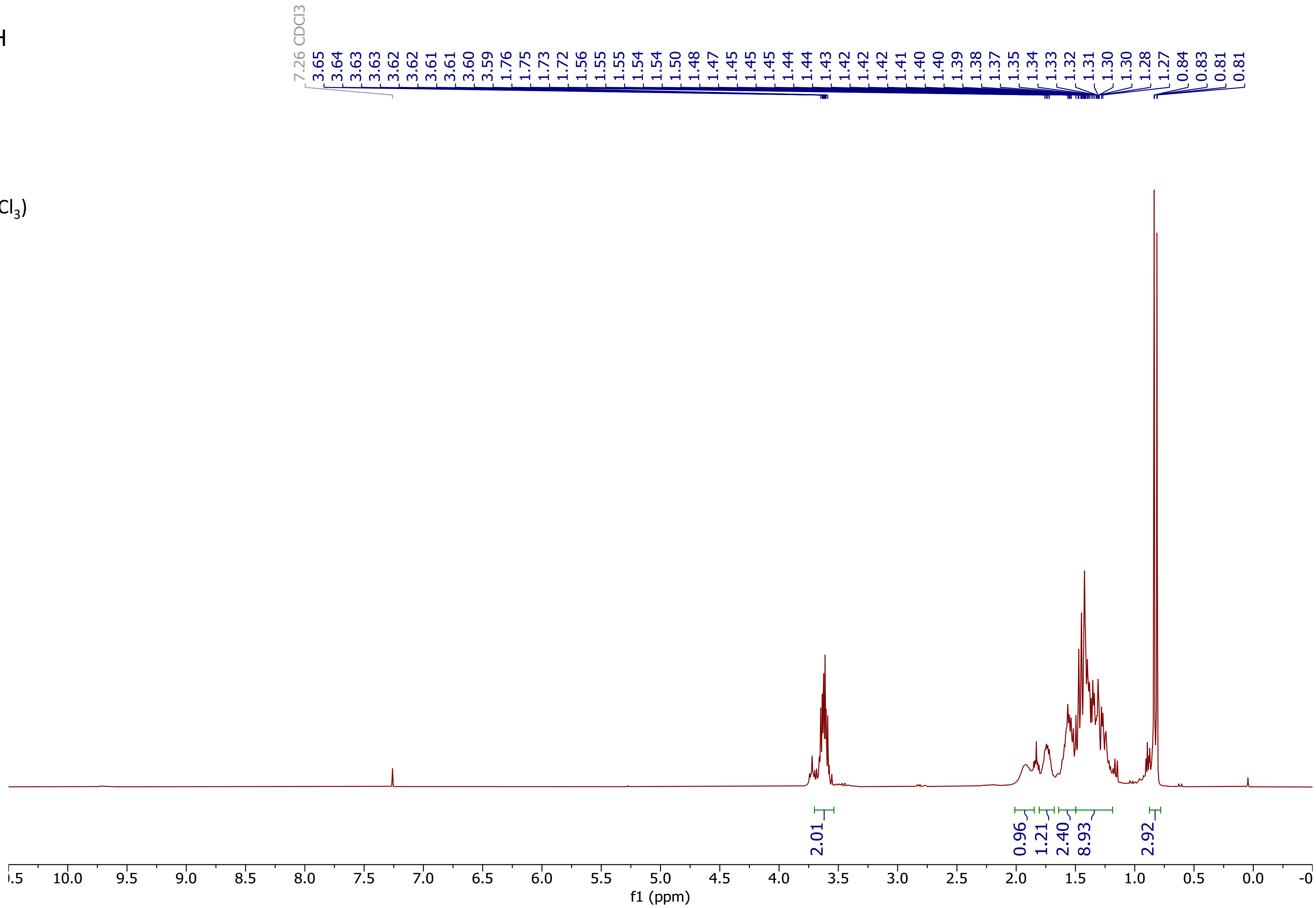

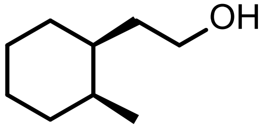

cis-3c-OH

-crude-

^{13}C NMR (75 MHz, $CDCl_3$)

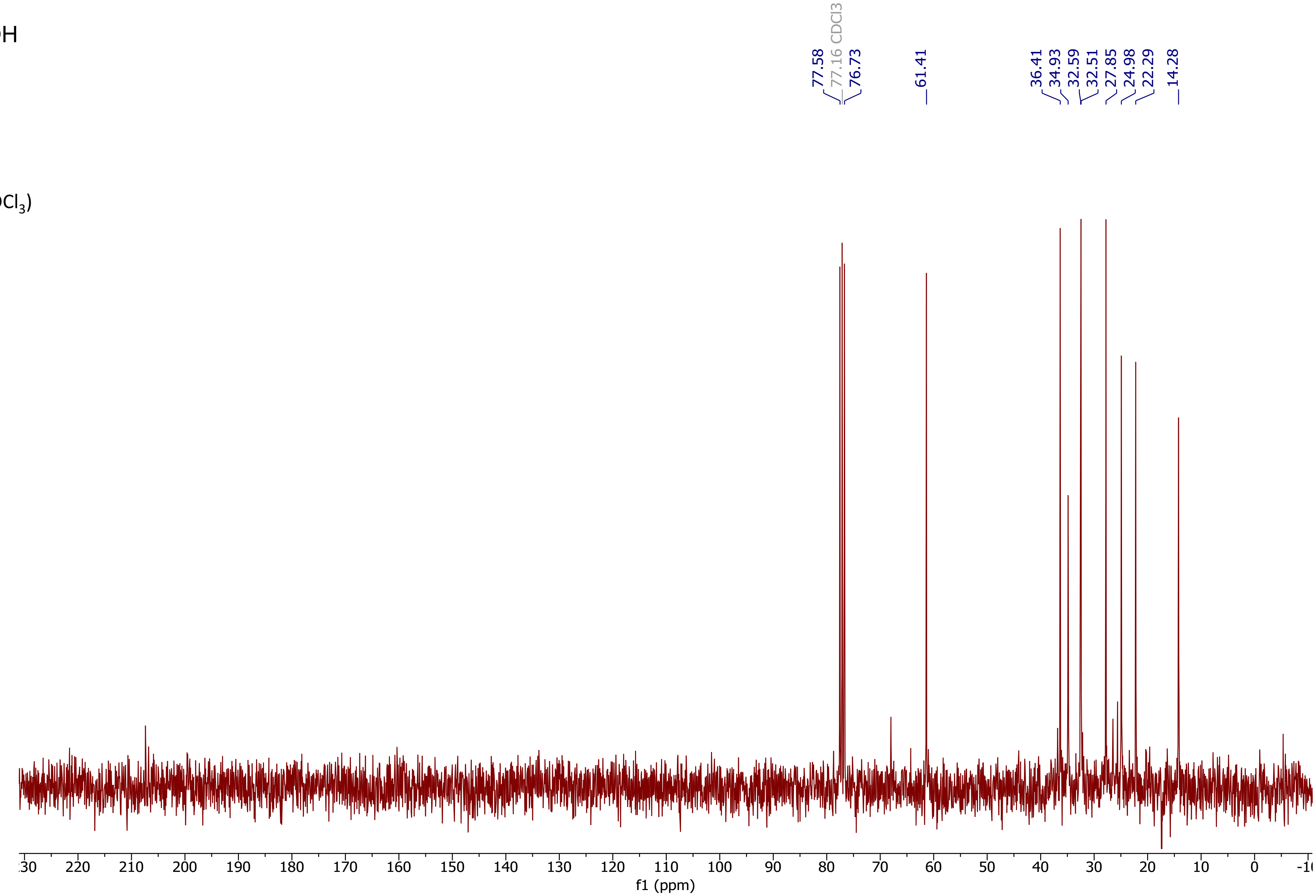

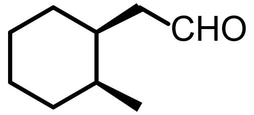

cis-3c-CHO

-crude-

[1]**H NMR**(300 MHz, $CDCl_3$)

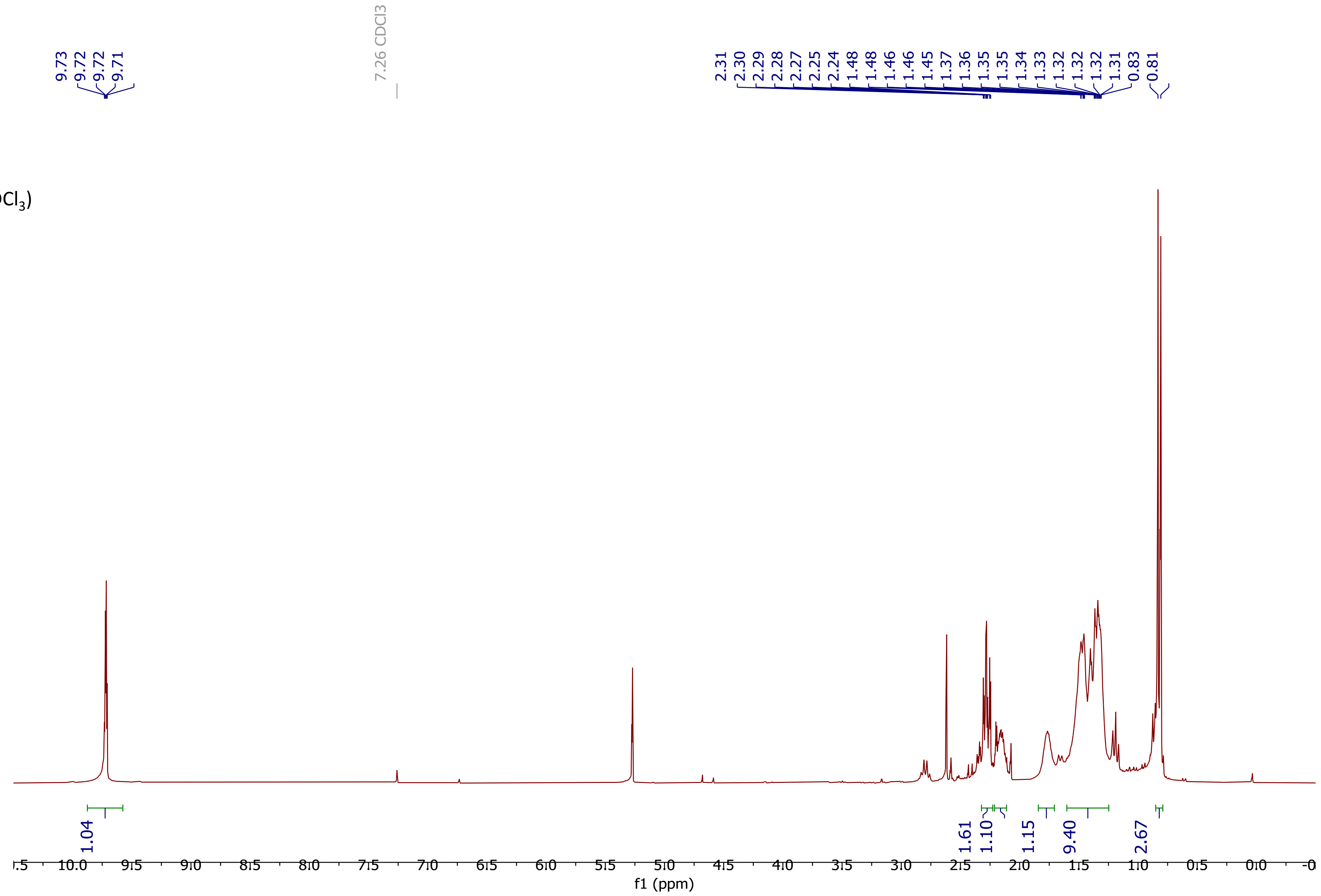

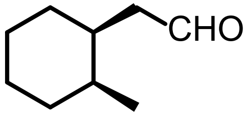

cis-3c-CHO

-crude-

^{13}C NMR (75 MHz, $CDCl_3$)

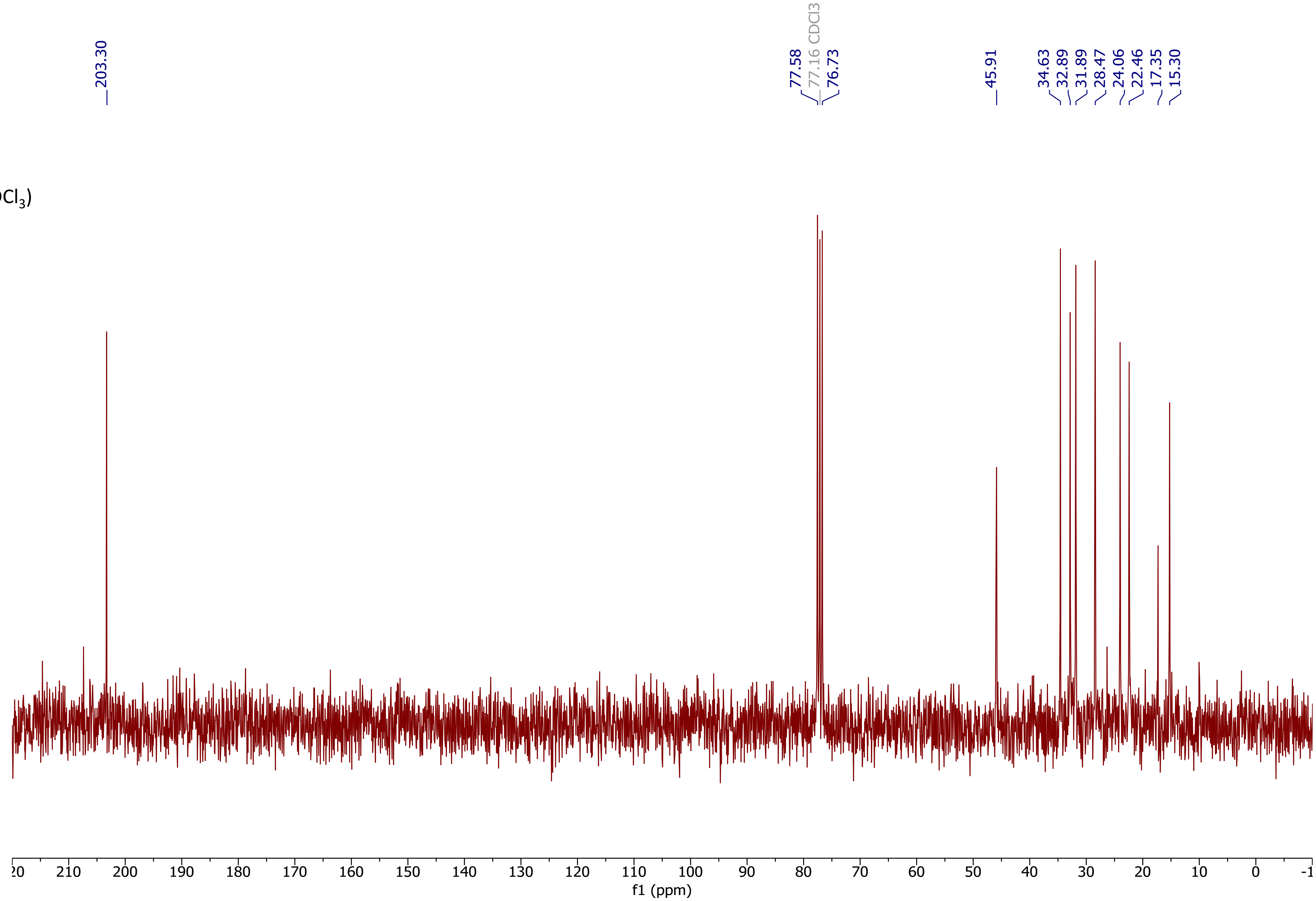

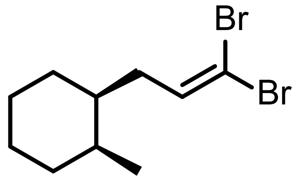

cis-3c-CBr$_2$

^{1}H NMR(300 MHz, $CDCl_3$)

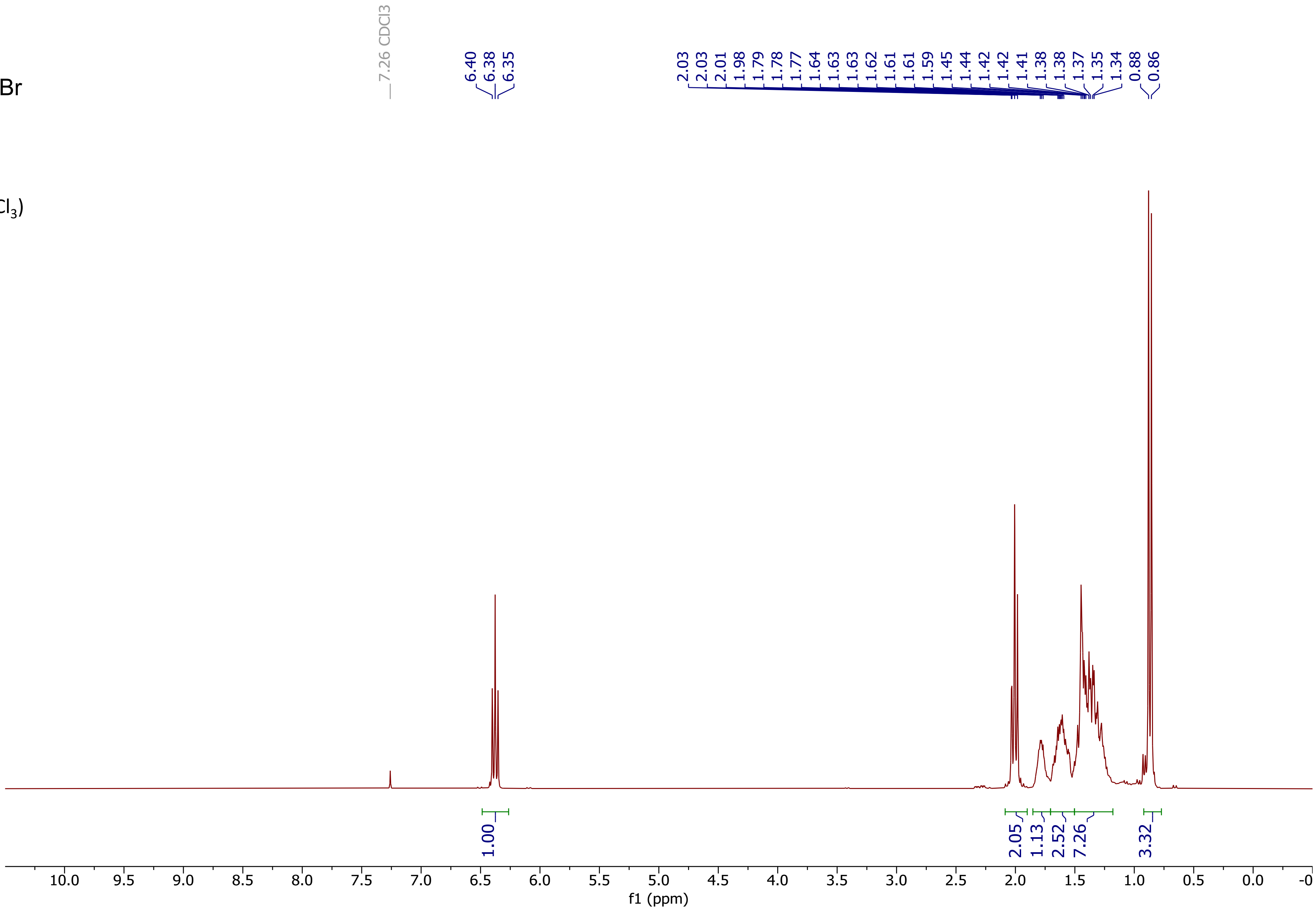

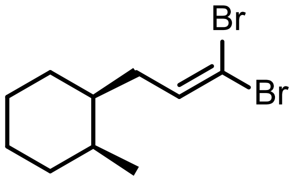

cis-3c-CBr_2

^{13}C NMR (75 MHz, $CDCl_3$)

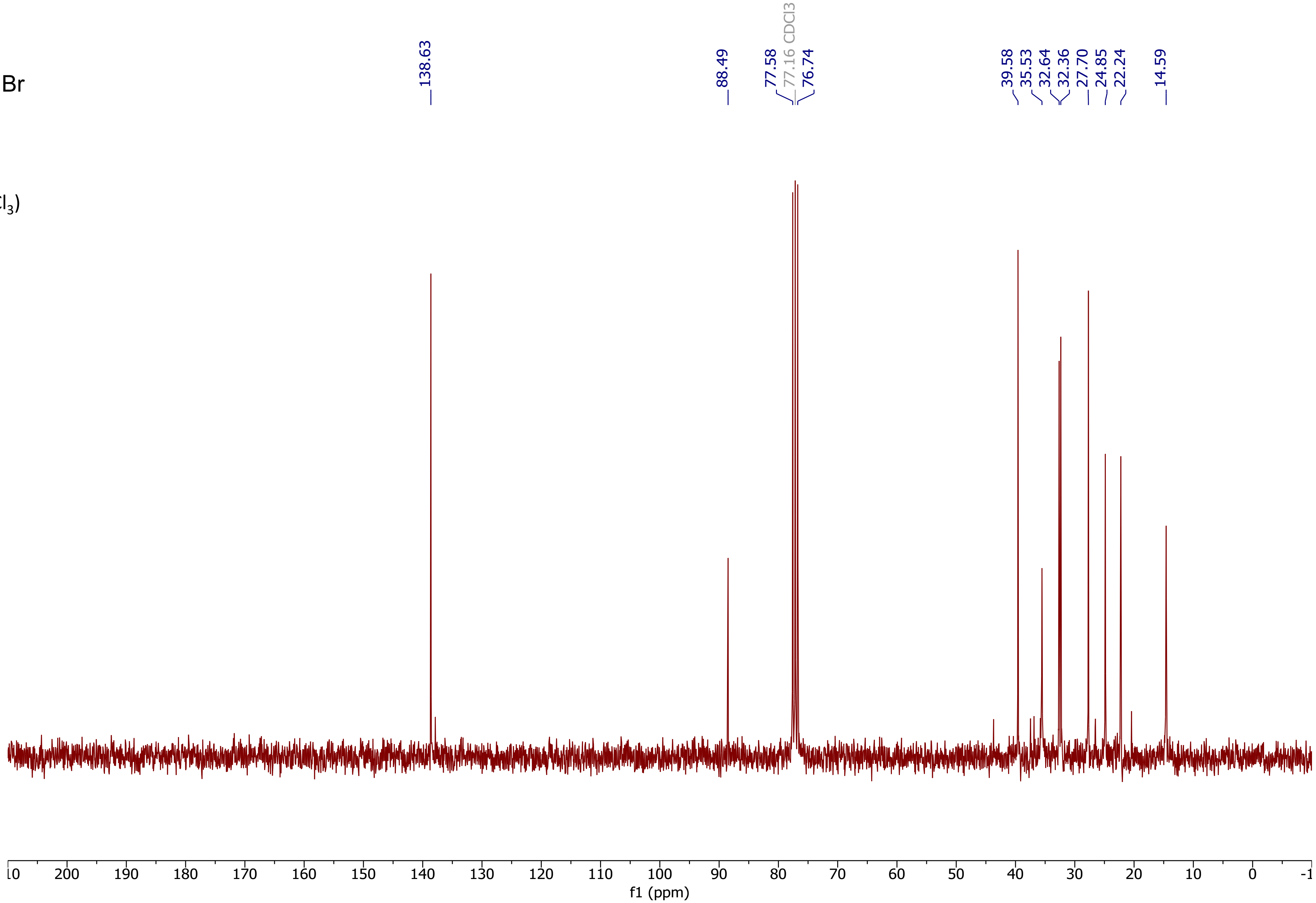

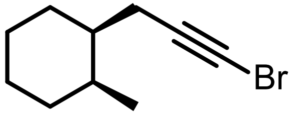

cis-3c

^{1}H NMR(300 MHz, $CDCl_3$)

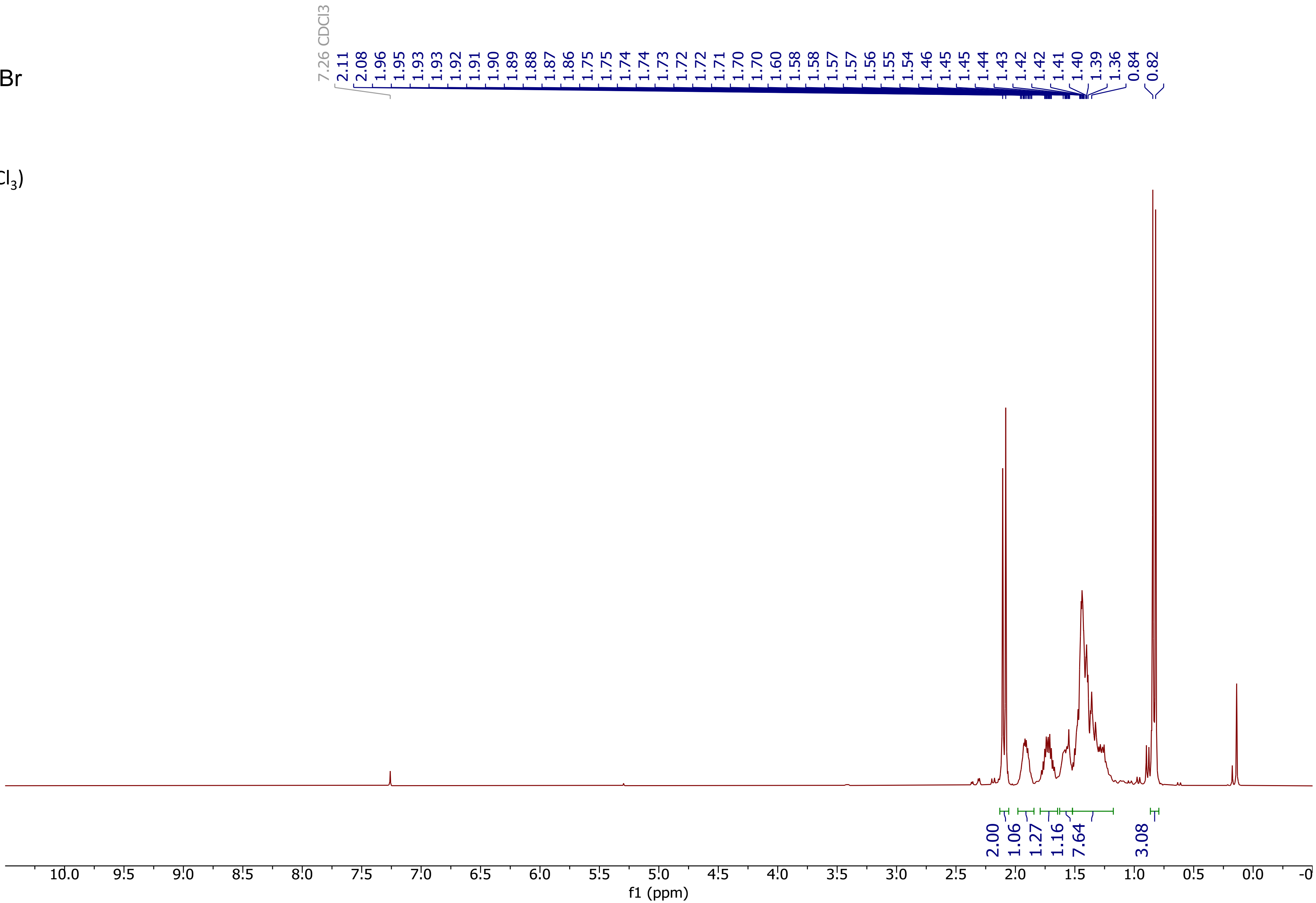

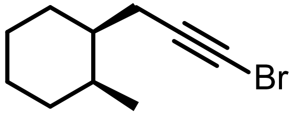

cis-3c

^{13}C NMR (75 MHz, $CDCl_3$)

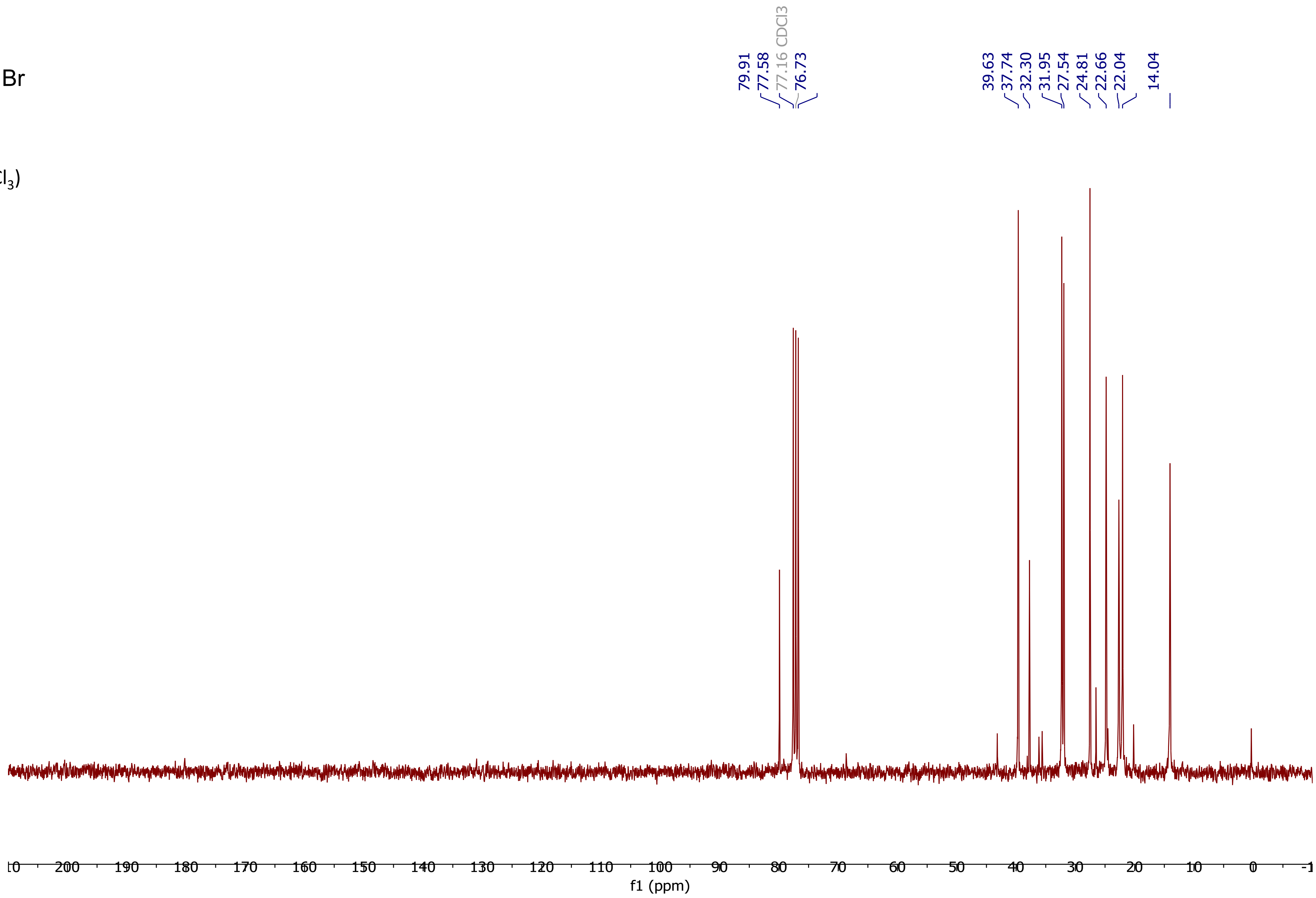

***trans*-3c**

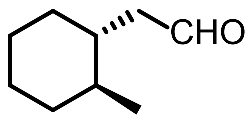

trans-3c-CHO

^{1}H NMR(300 MHz, $CDCl_3$)

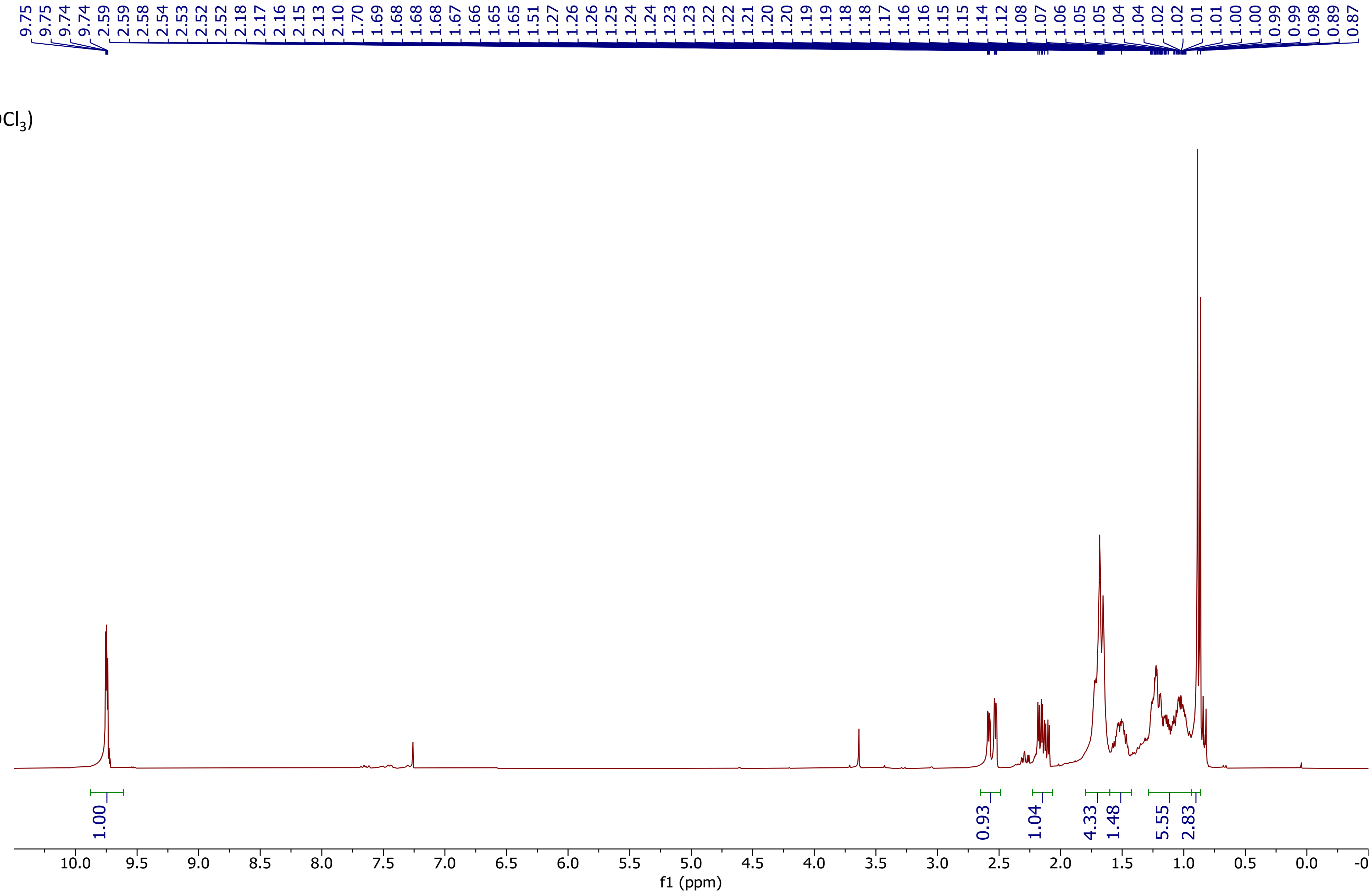

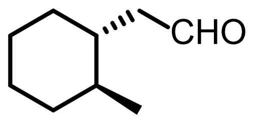

trans-3c-CHO

^{13}C NMR (75 MHz, $CDCl_3$)

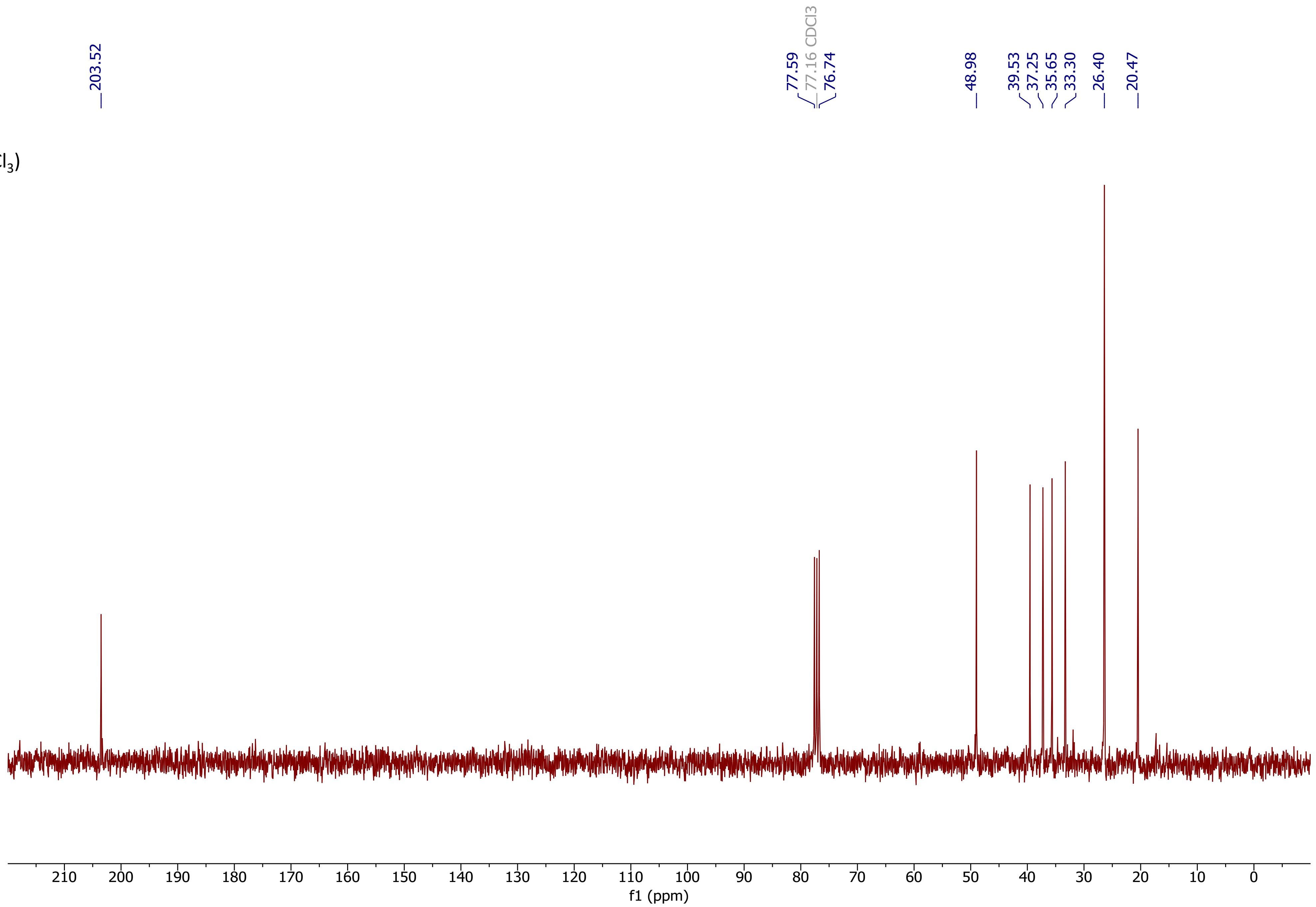

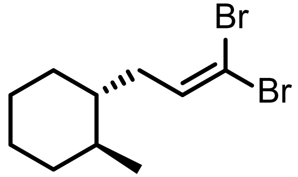

trans-3c-CBr_2

^{1}H NMR(300 MHz, $CDCl_3$)

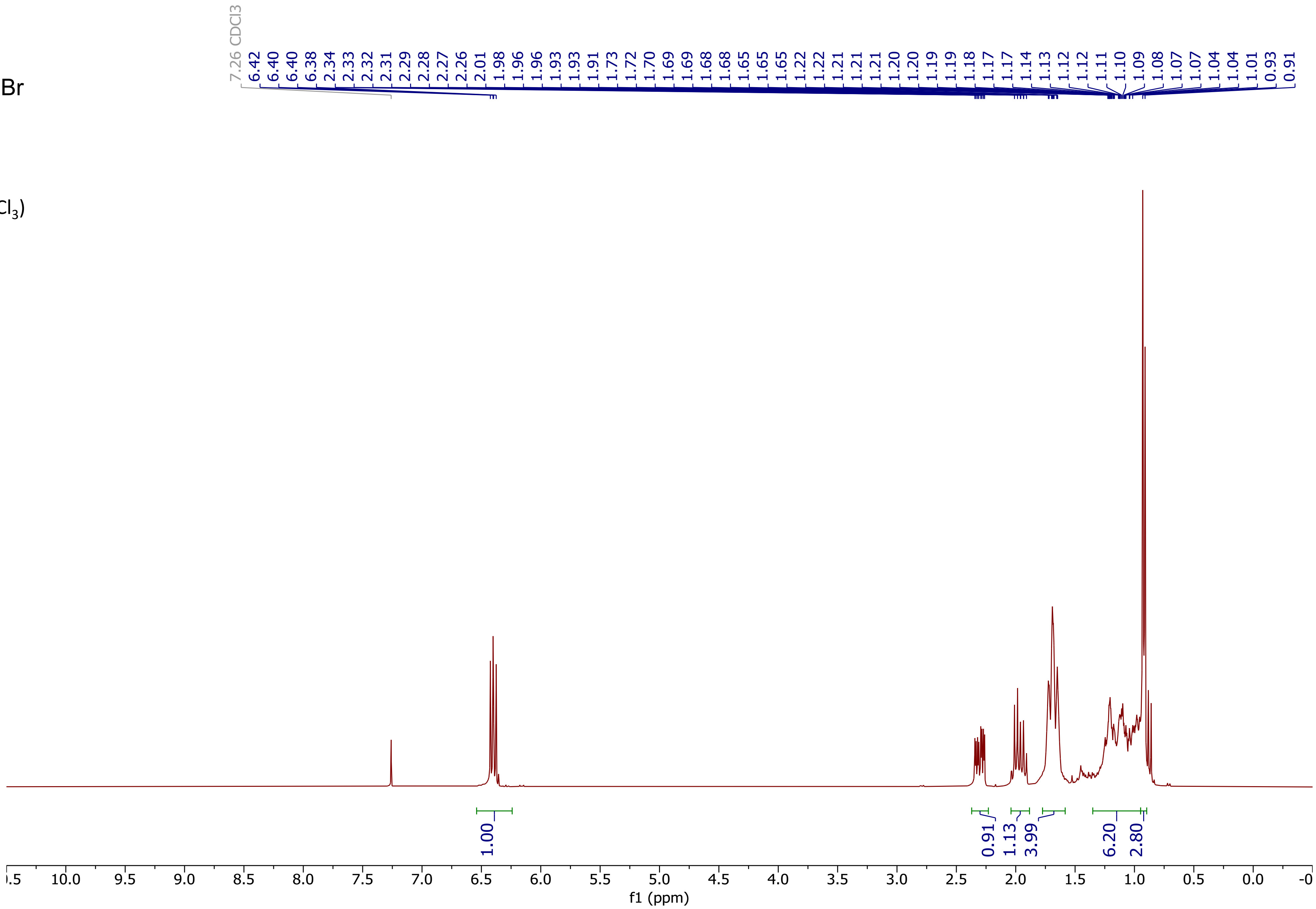

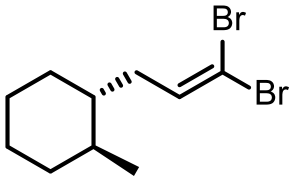

trans-3c-CBr$_2$

^{13}C NMR (75 MHz, $CDCl_3$)

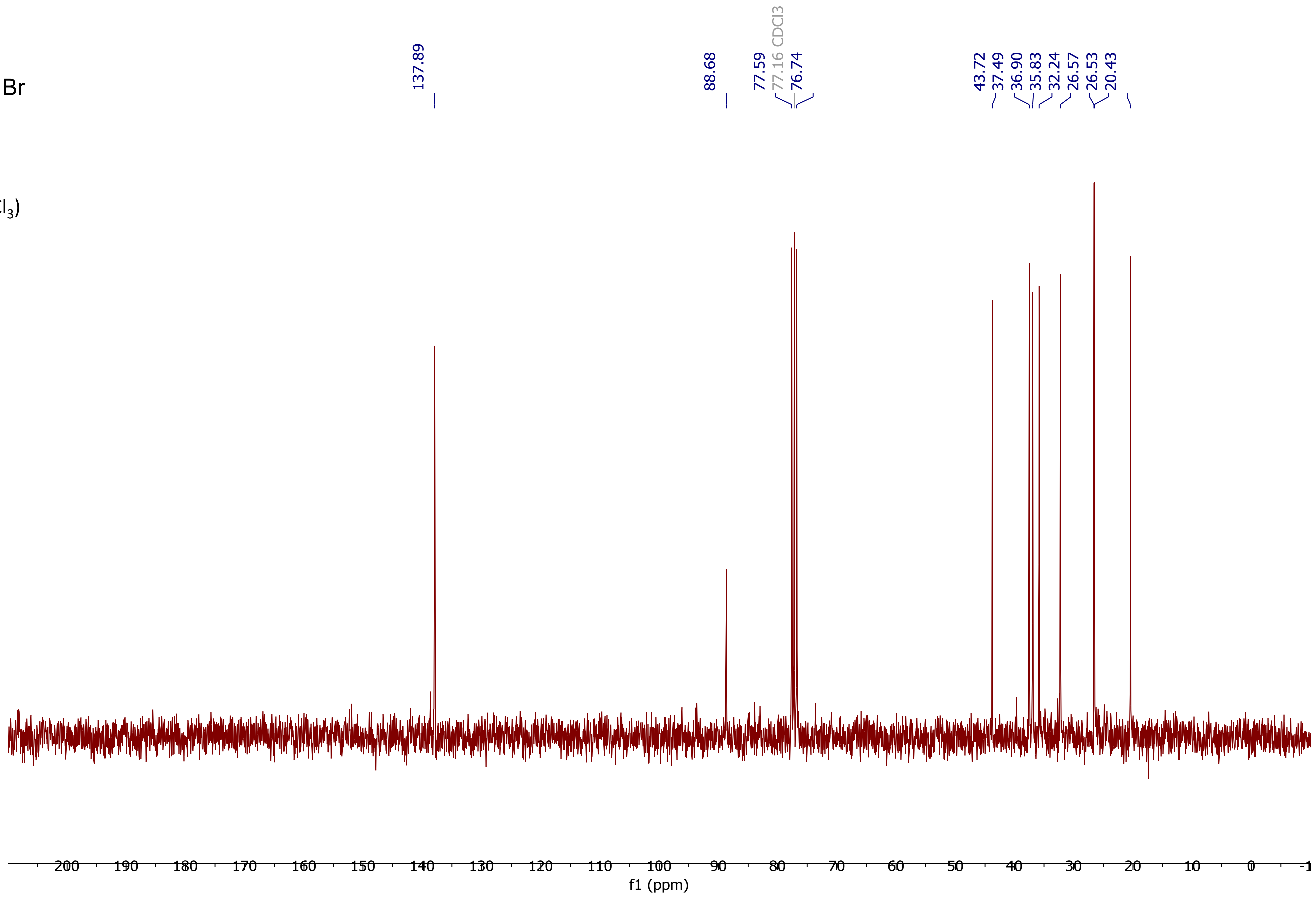

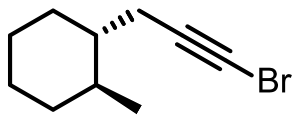

trans-3c

^{1}H NMR(300 MHz, $CDCl_3$)

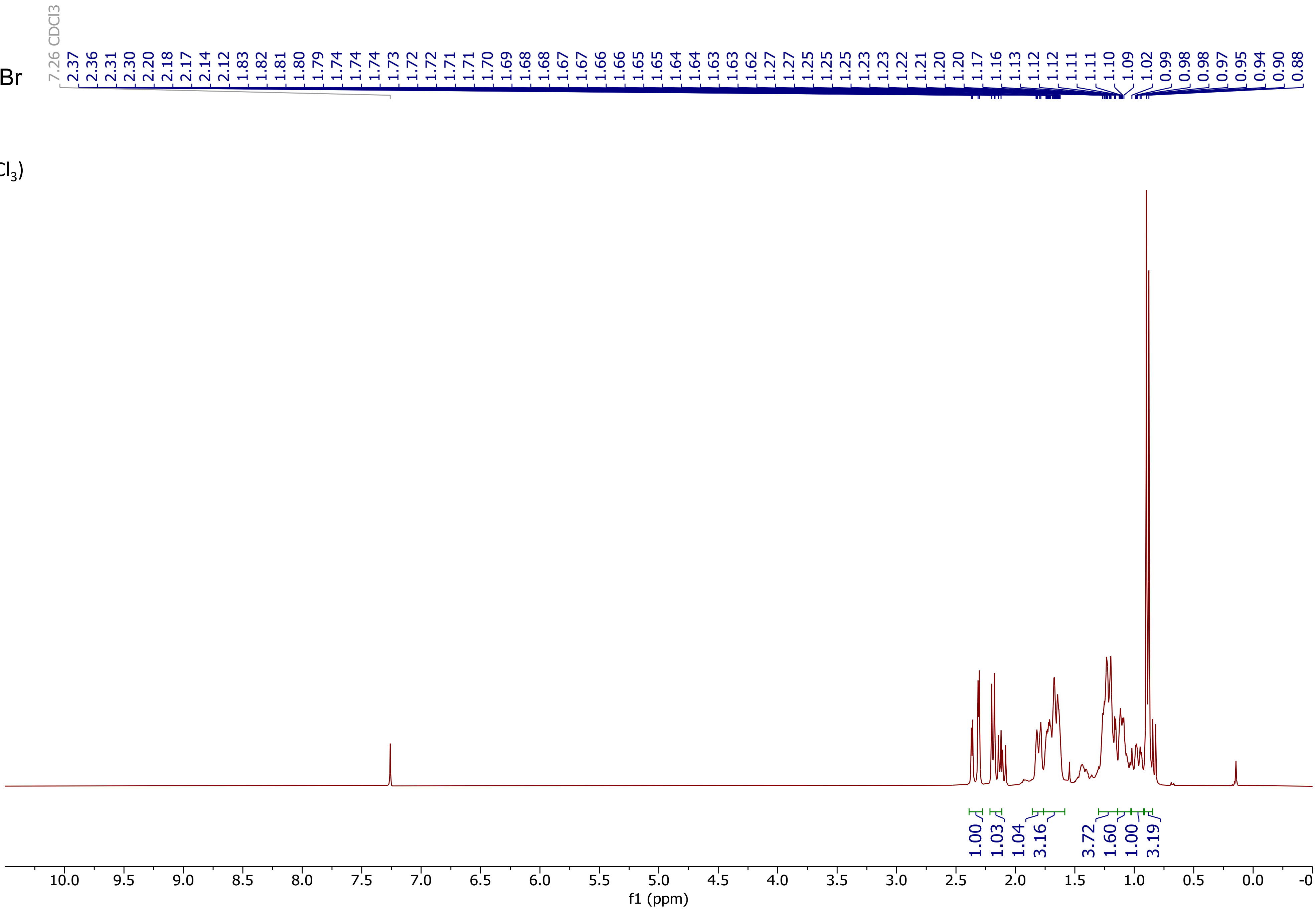

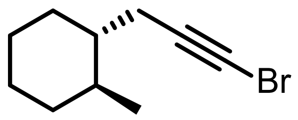

trans-3c

^{13}C NMR (75 MHz, $CDCl_3$)

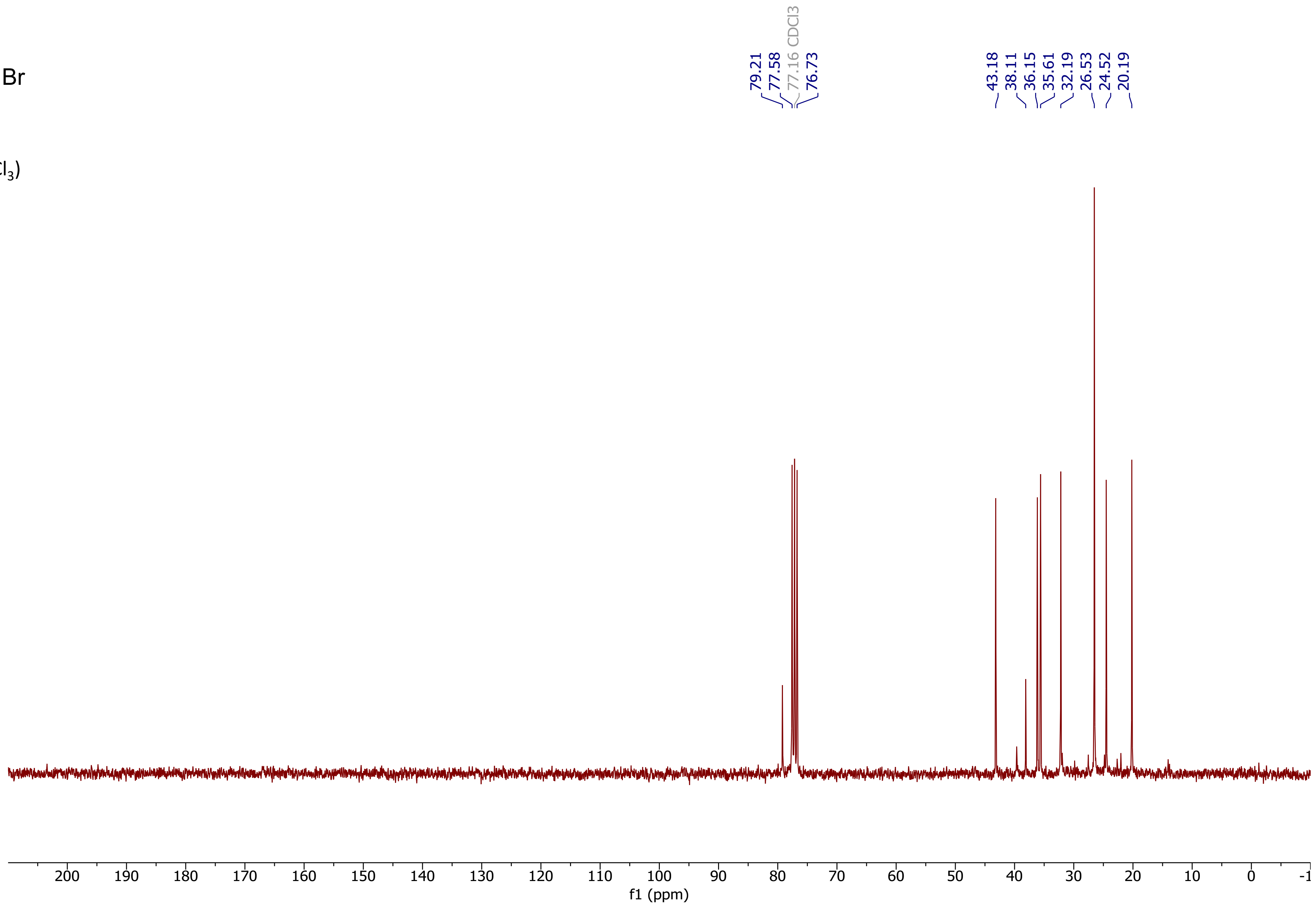

***cis*-3d**

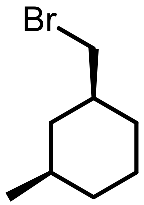

cis-**3d-Br**

^{1}H NMR(300 MHz, $CDCl_3$)

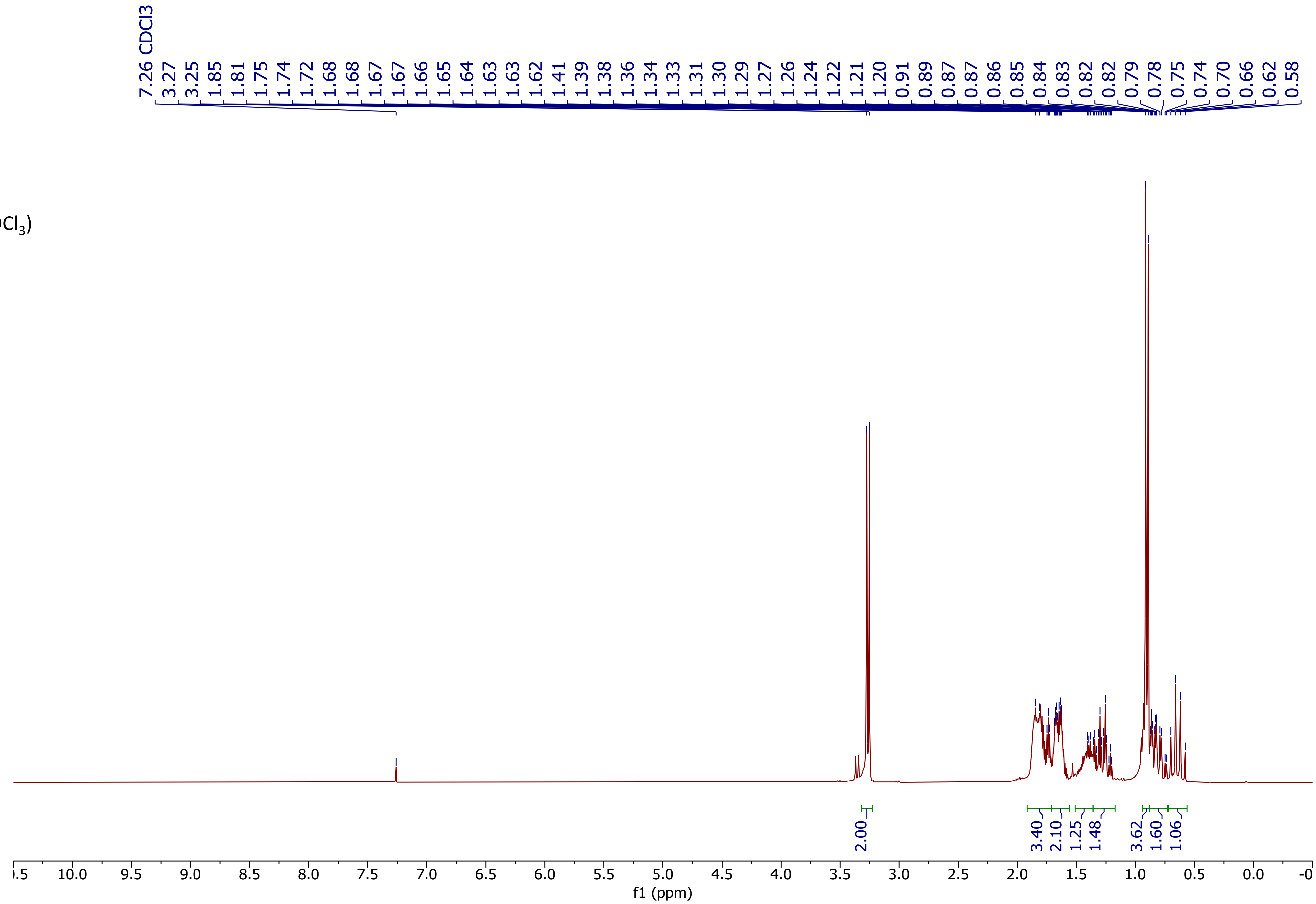

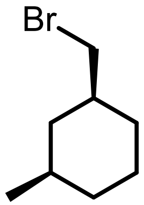

***cis*-3d-Br**

^{13}C NMR (75 MHz, $CDCl_3$)

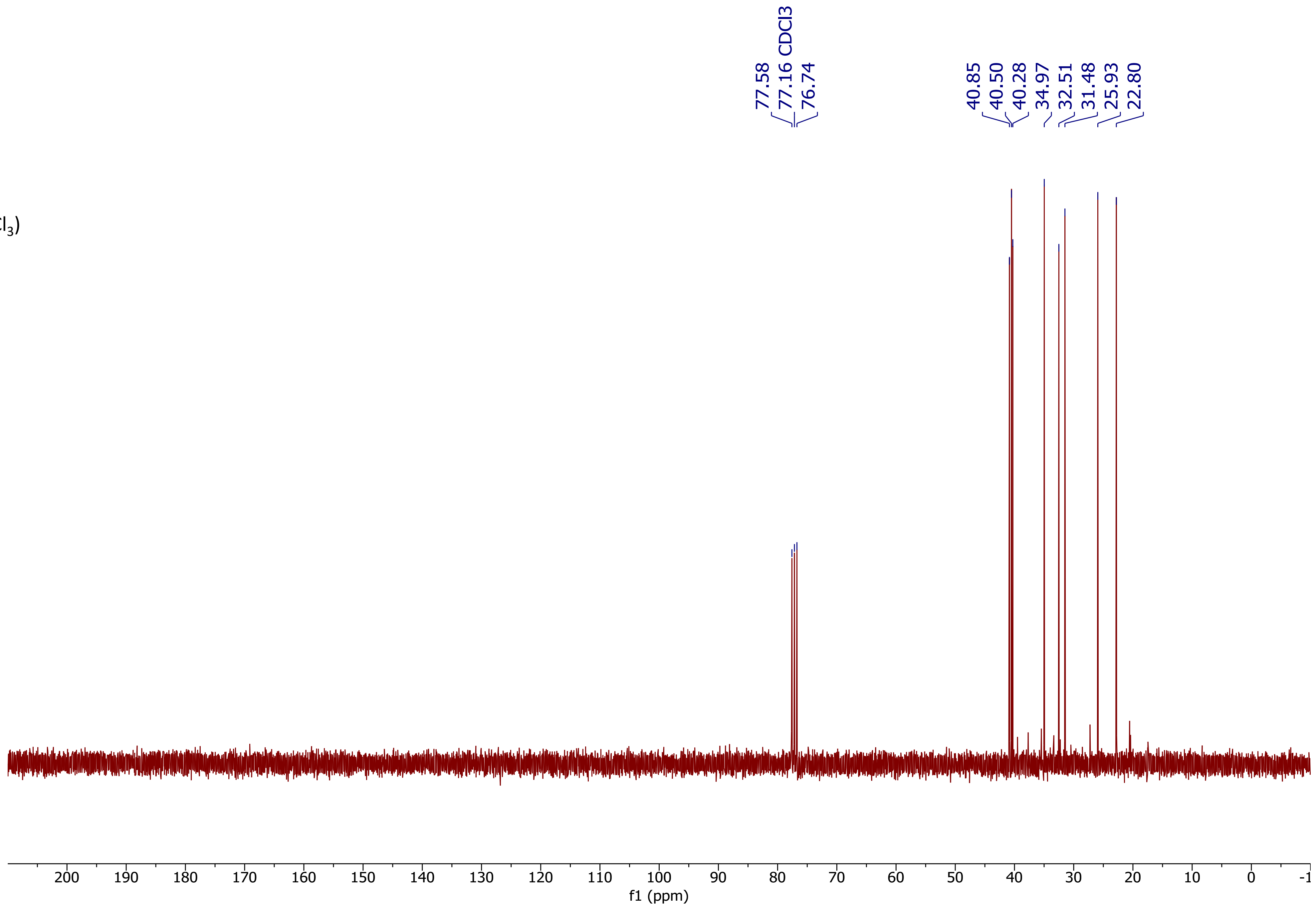

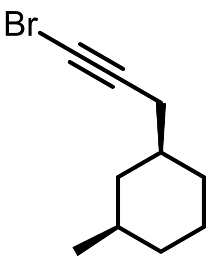

***cis*-3d**

^{1}H NMR(300 MHz, $CDCl_3$)

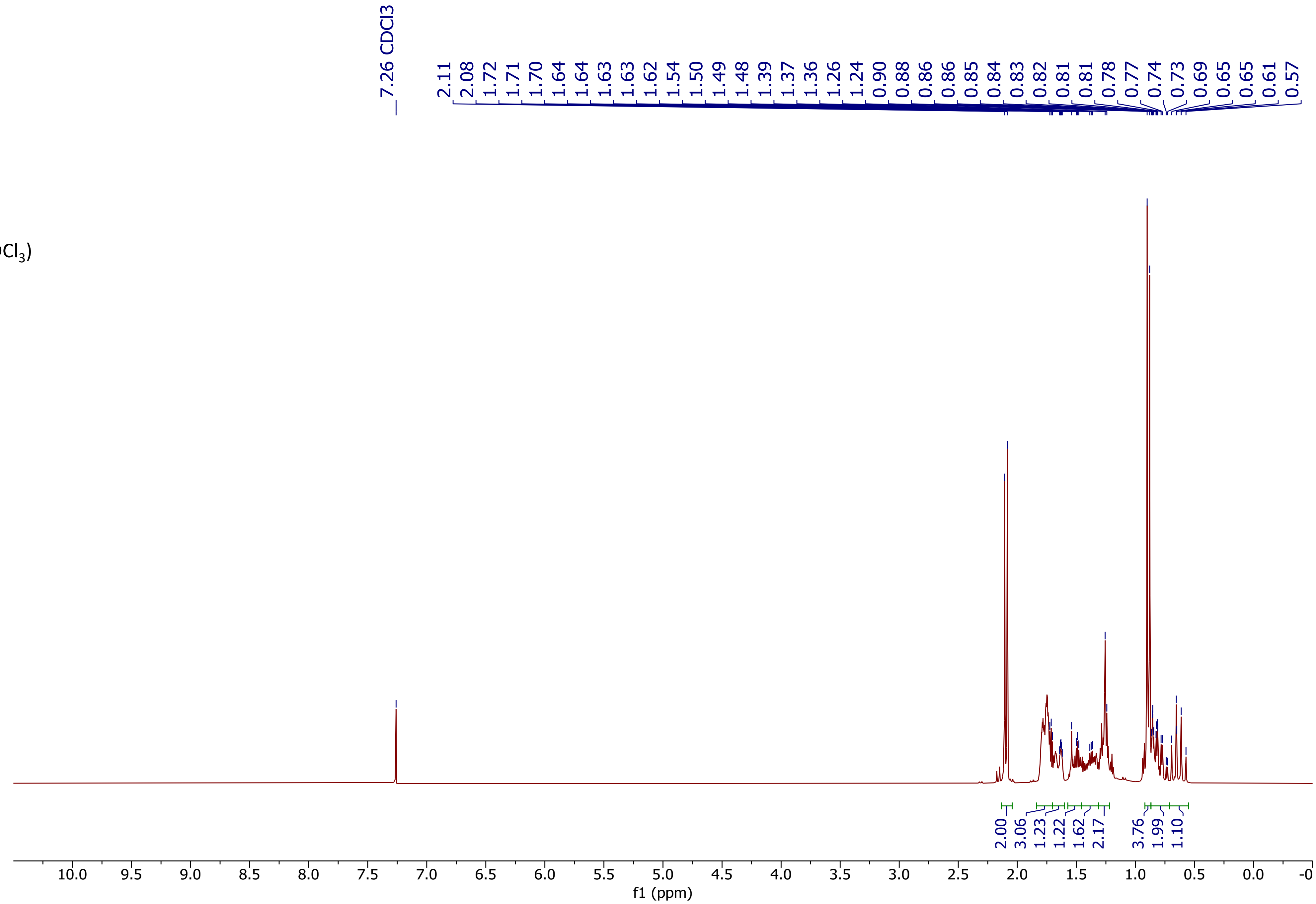

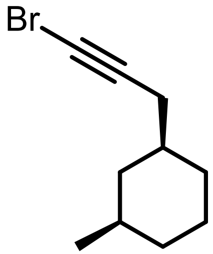

***cis*-3d**

^{13}C NMR (75 MHz, $CDCl_3$)

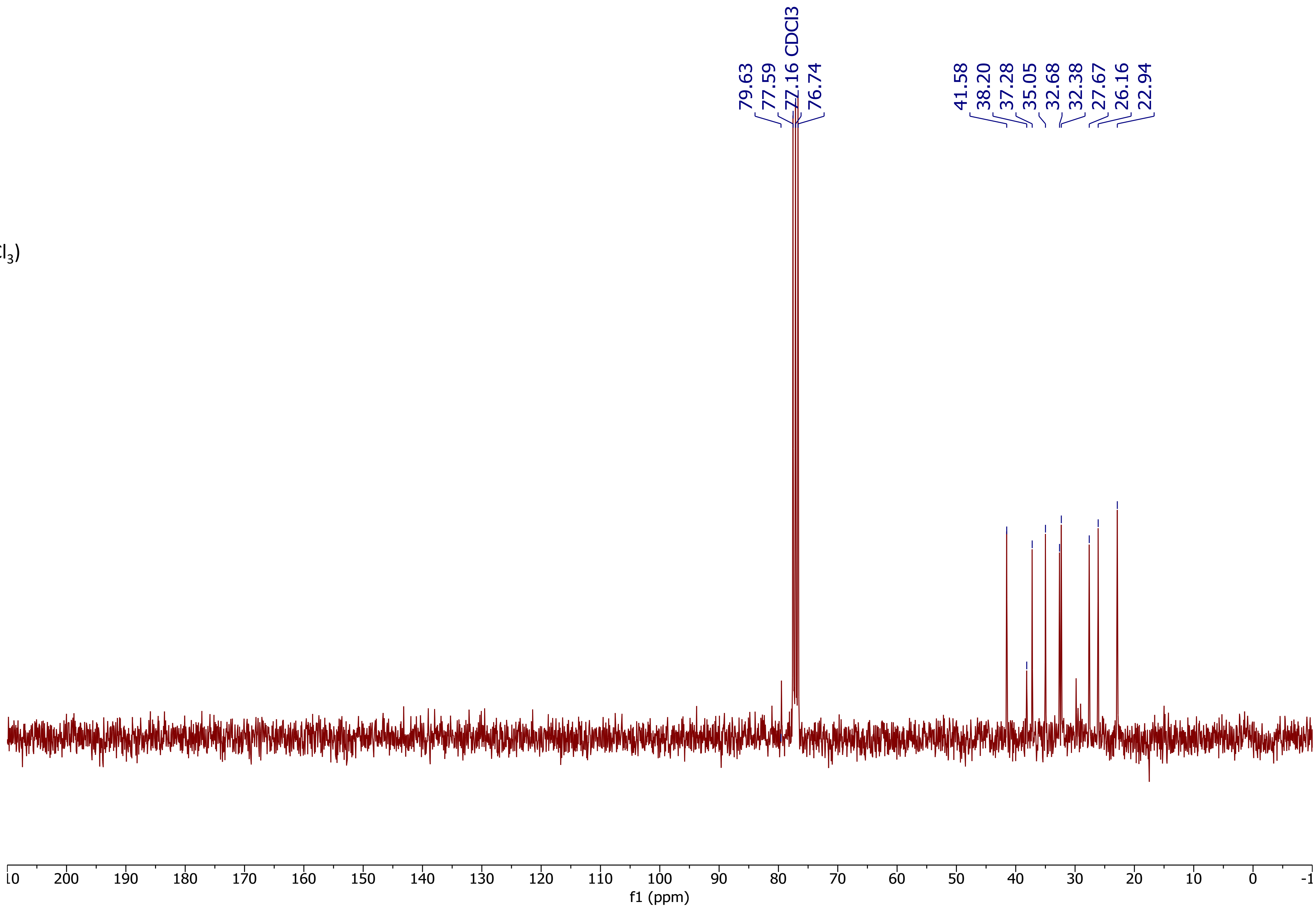

***trans*-3d**

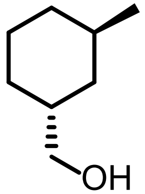

***trans*-3d-OH**

^{1}H NMR(300 MHz, $CDCl_3$)

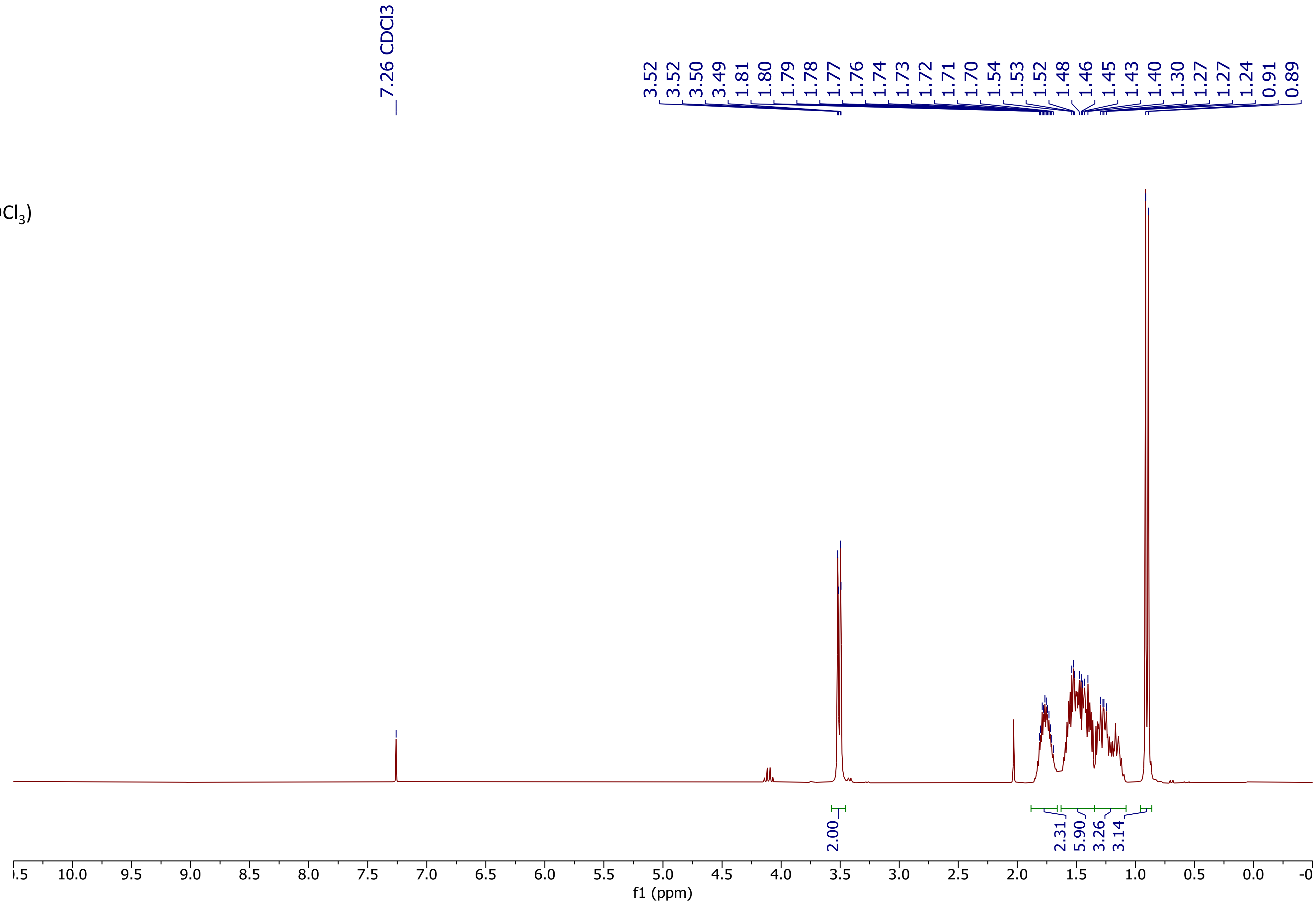

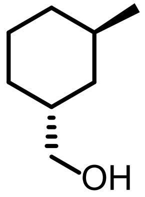

***trans*-3d-OH**

^{13}C NMR (75 MHz, $CDCl_3$)

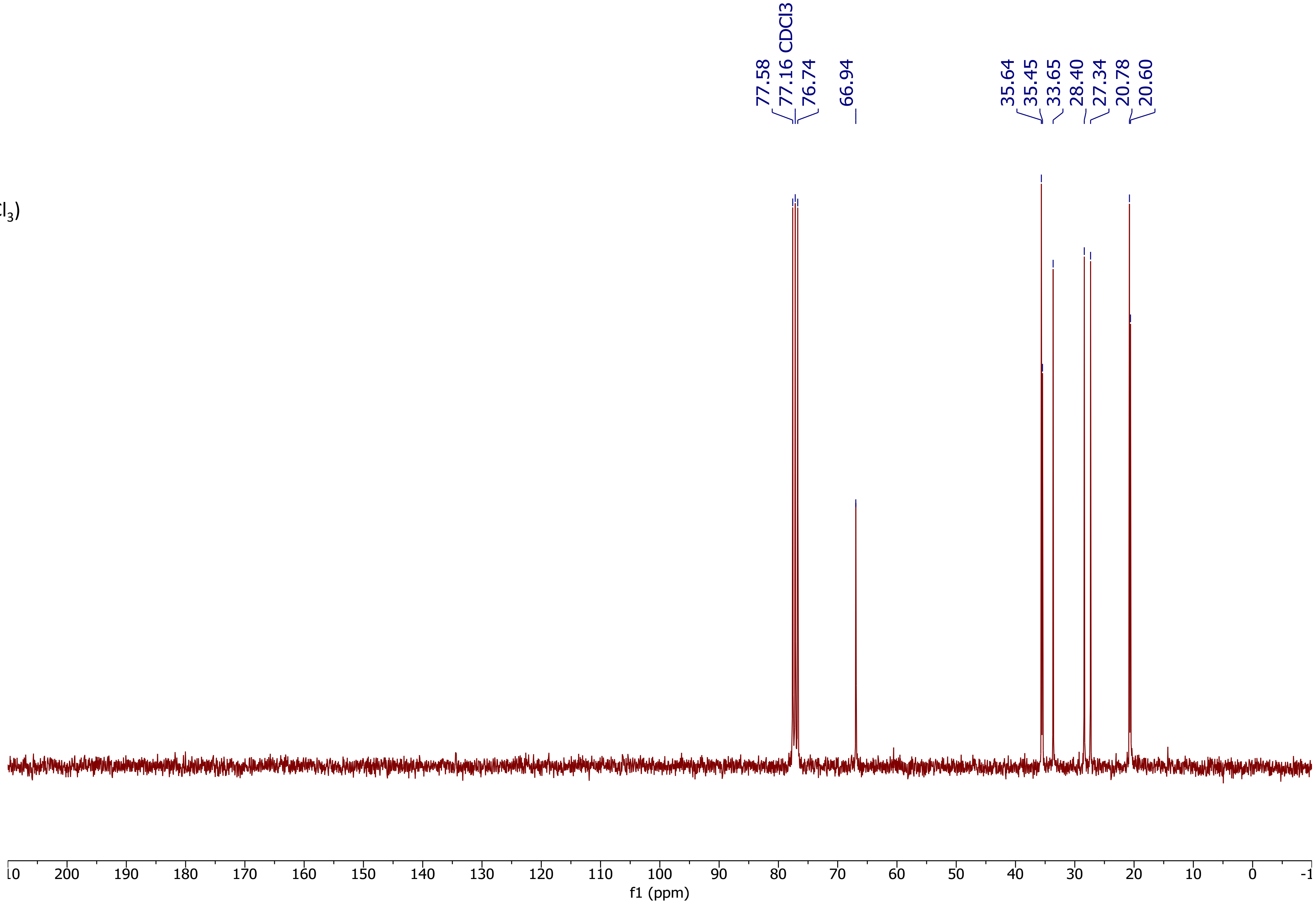

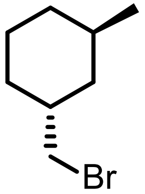

***trans*-3d-Br**

^{1}H NMR(300 MHz, $CDCl_3$)

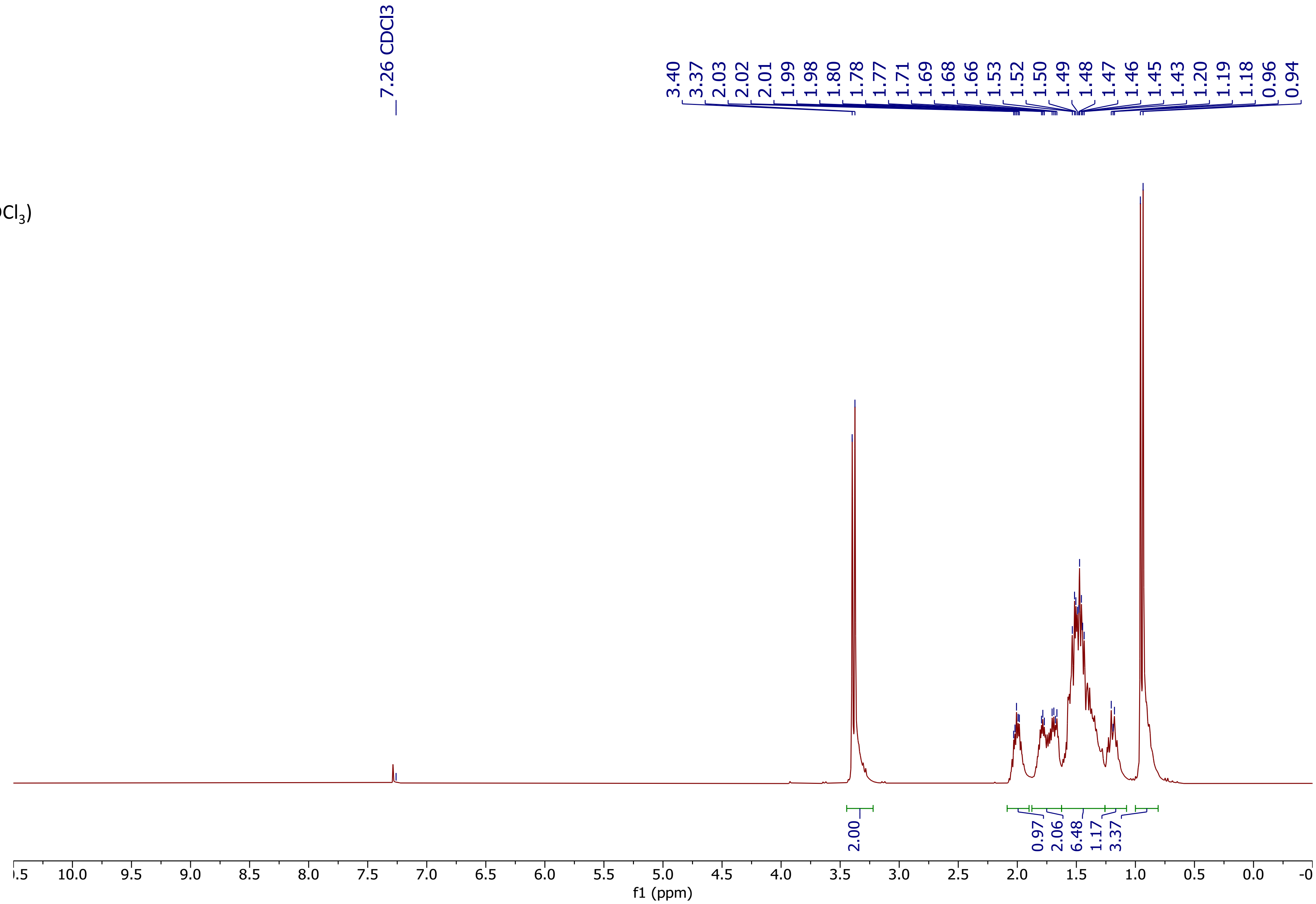

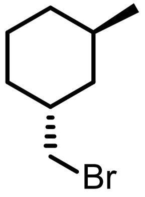

***trans*-3d-Br**

^{13}C NMR (75 MHz, $CDCl_3$)

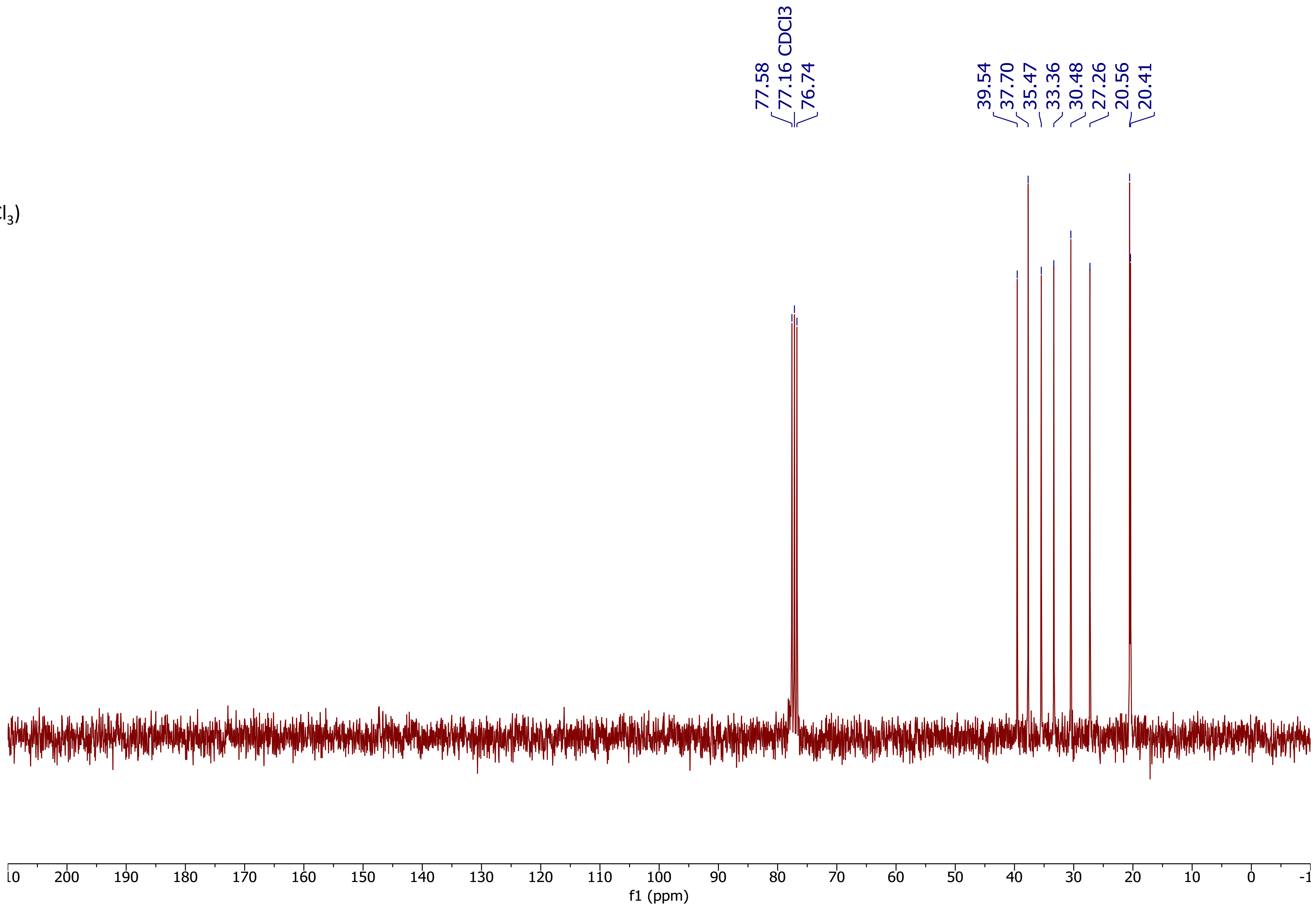

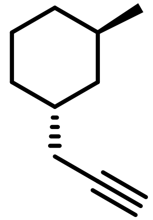

***trans*-3d-CCH**

^{1}H NMR(300 MHz, $CDCl_3$)

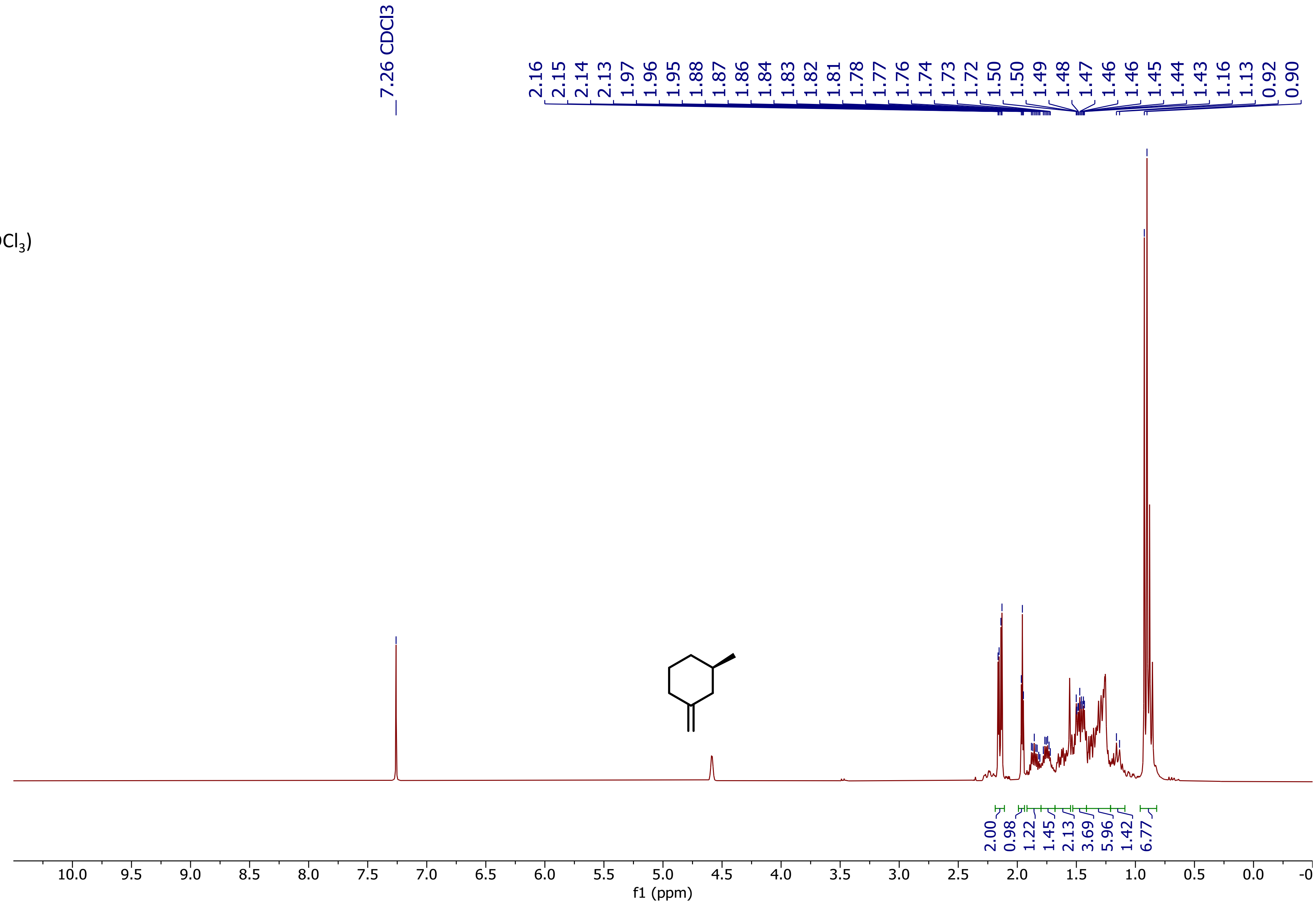

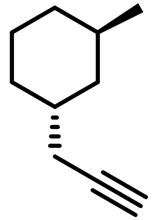

***trans*-3d-CCH**

^{13}C NMR (75 MHz, $CDCl_3$)

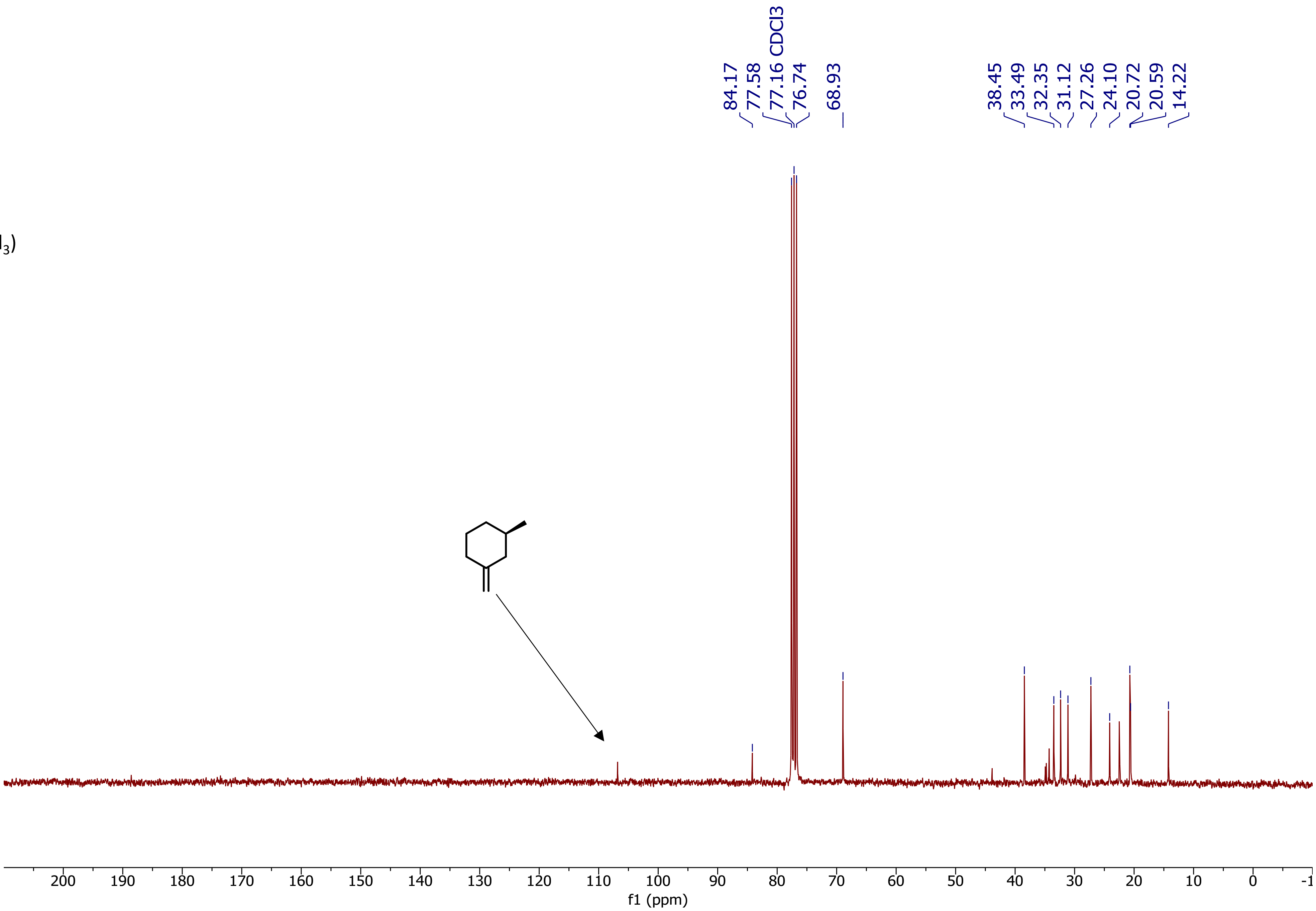

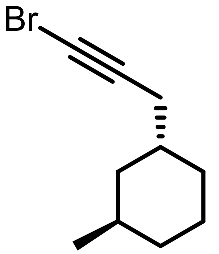

***trans*-3d**

^{1}H NMR(400 MHz, $CDCl_3$)

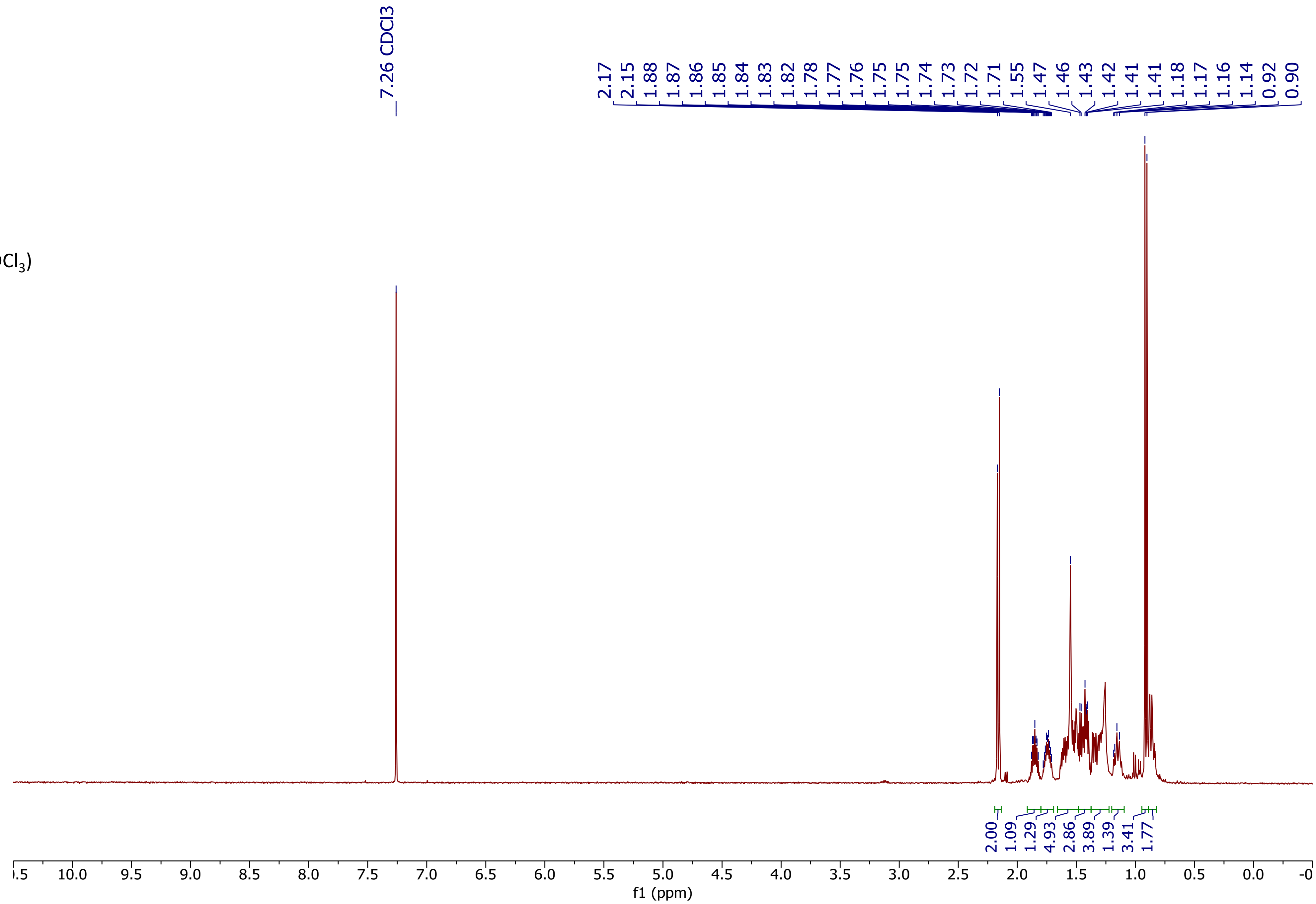

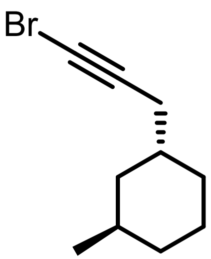

***trans*-3d**

^{13}C NMR (101 MHz, $CDCl_3$)

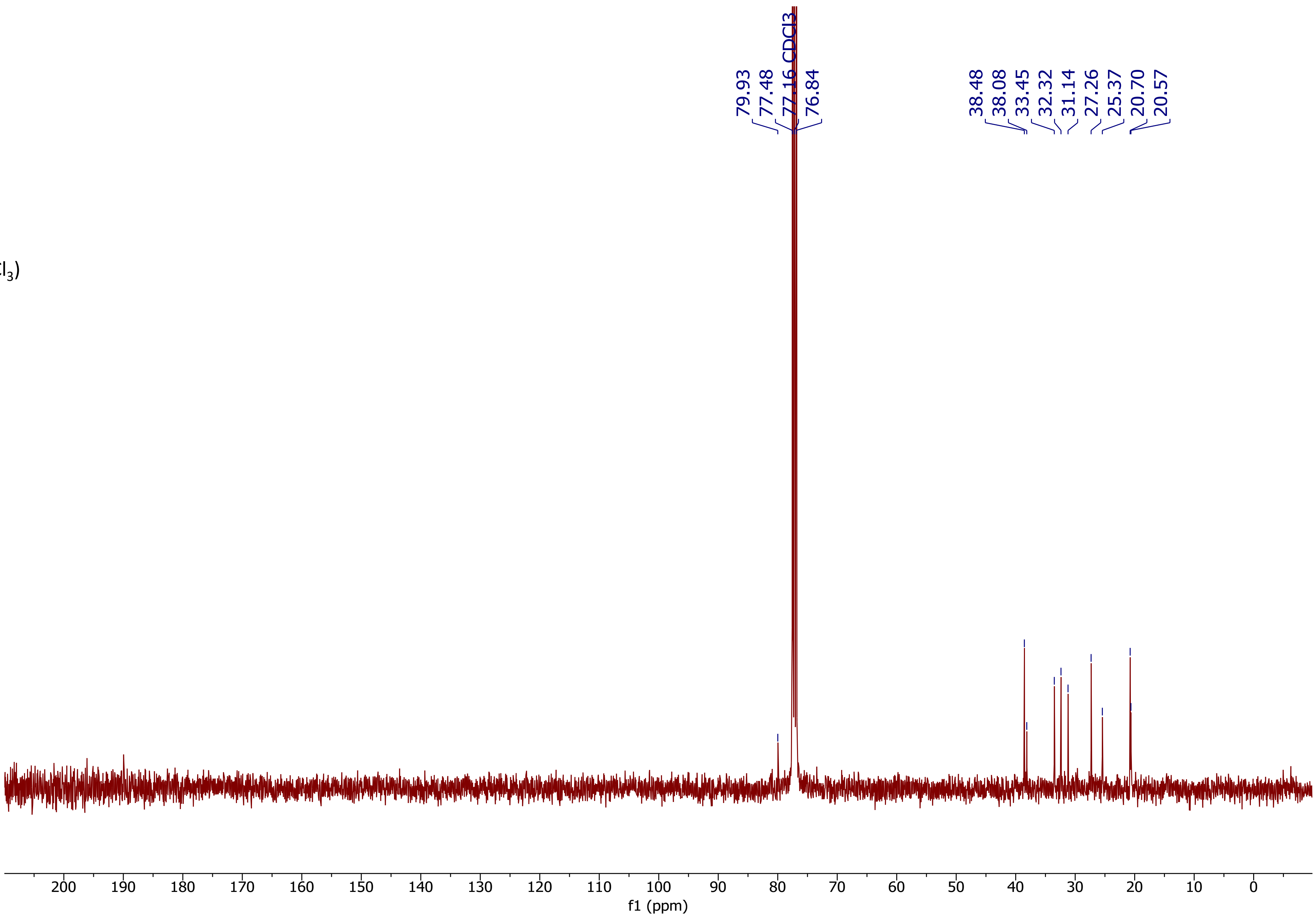

***cis*-3e**

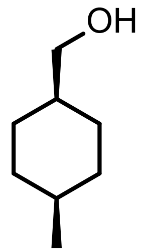

^{Me}***cis*-3e-OH**

-crude-

^{1}H NMR(300 MHz, $CDCl_3$)

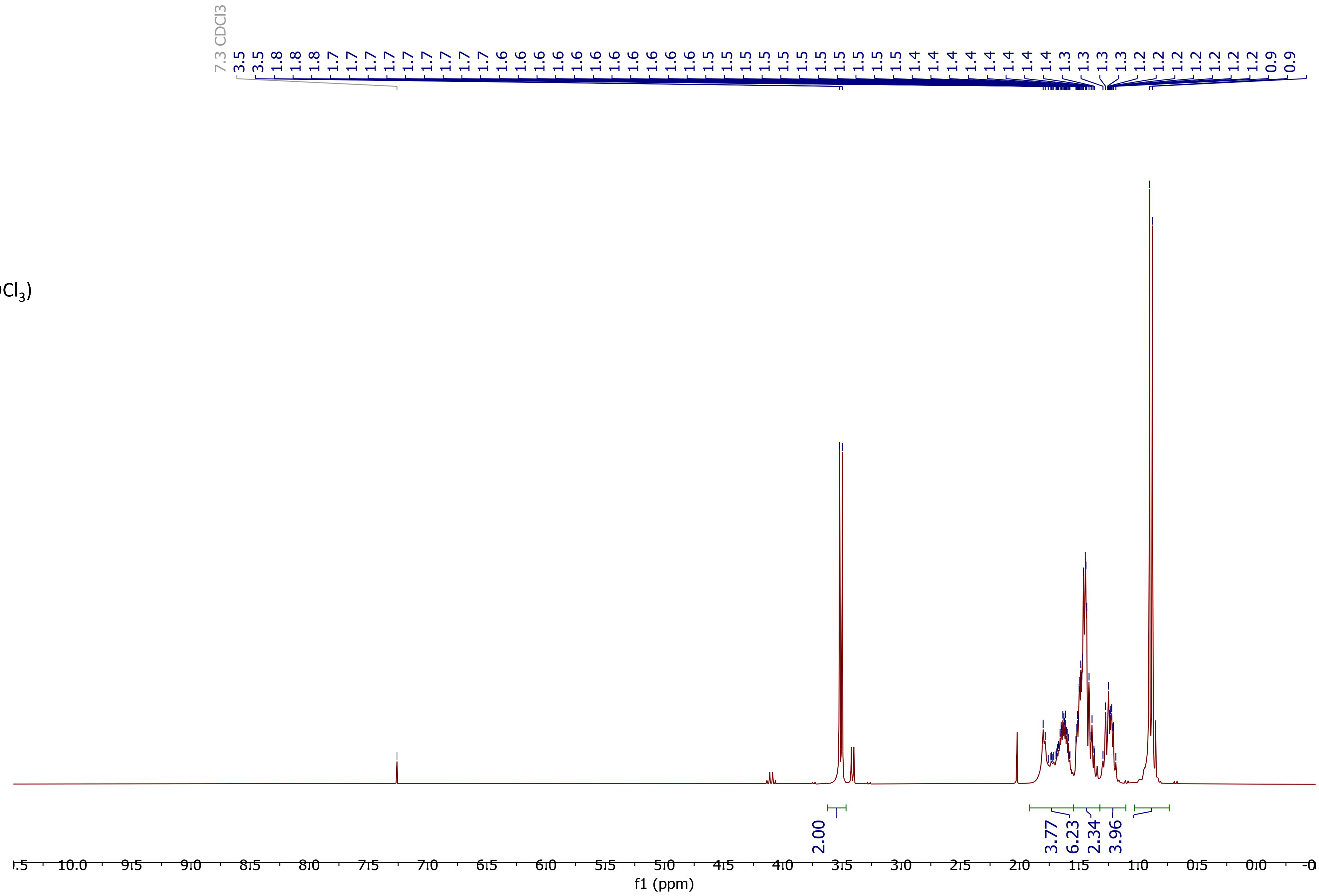

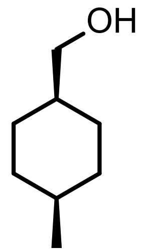

Me ***cis*-3e-OH**

-crude-

^{13}C NMR (75 MHz, $CDCl_3$)

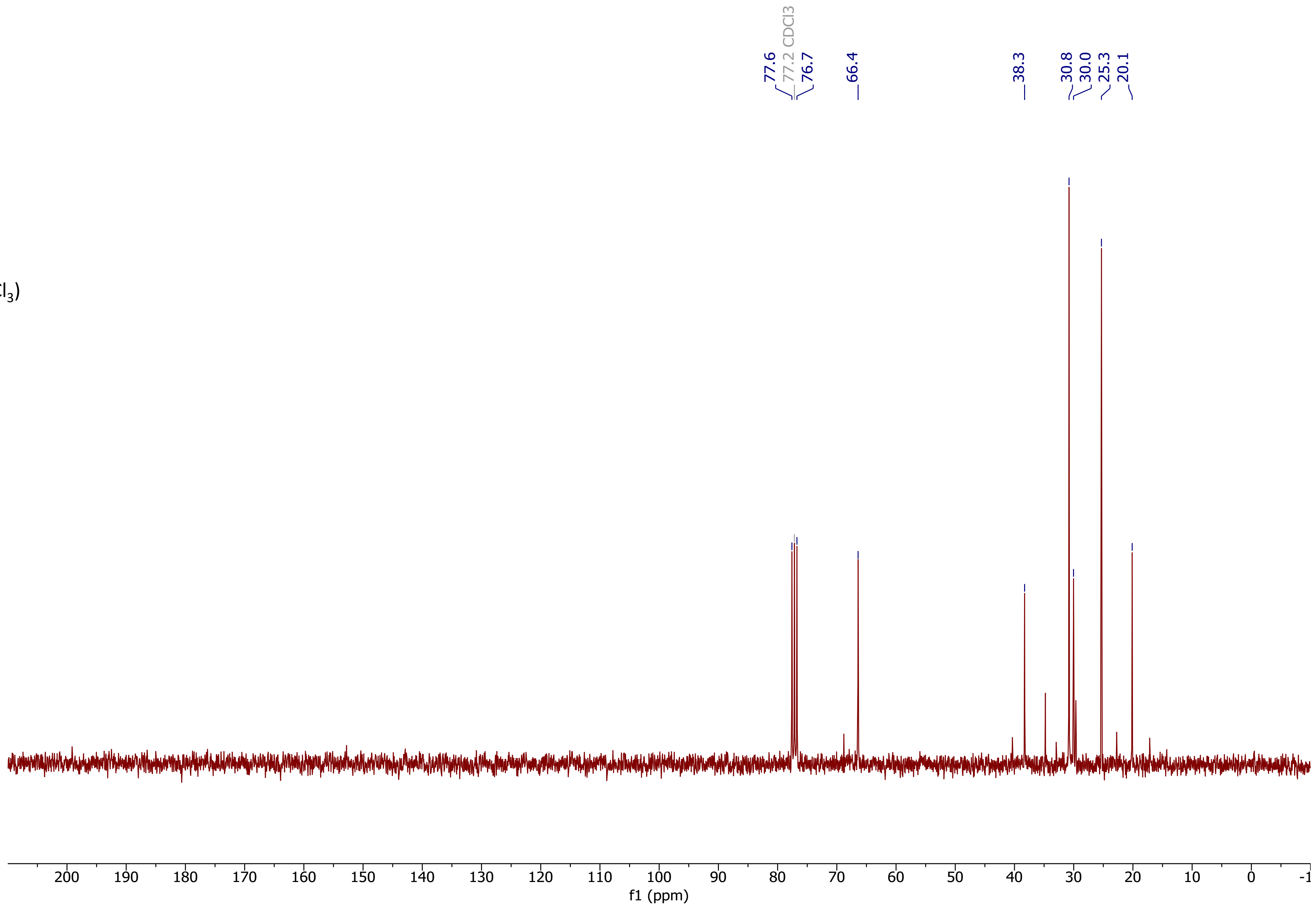

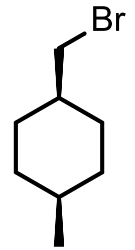

^{Me}*cis*-**3e-Br**

^{1}H NMR(300 MHz, $CDCl_3$)

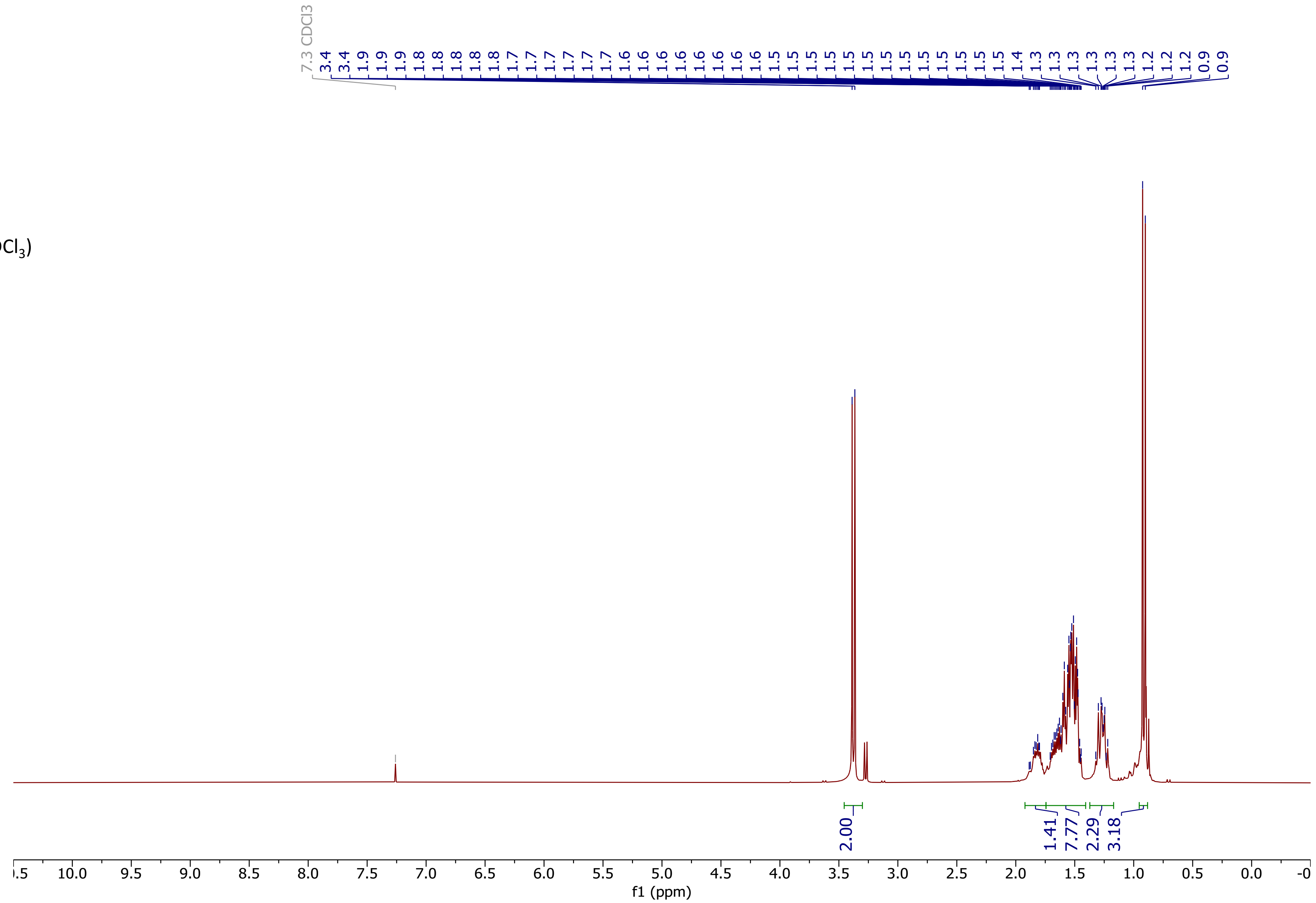

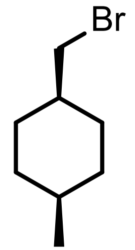

*Me**cis*-3e-Br**

^{13}C NMR (75 MHz, $CDCl_3$)

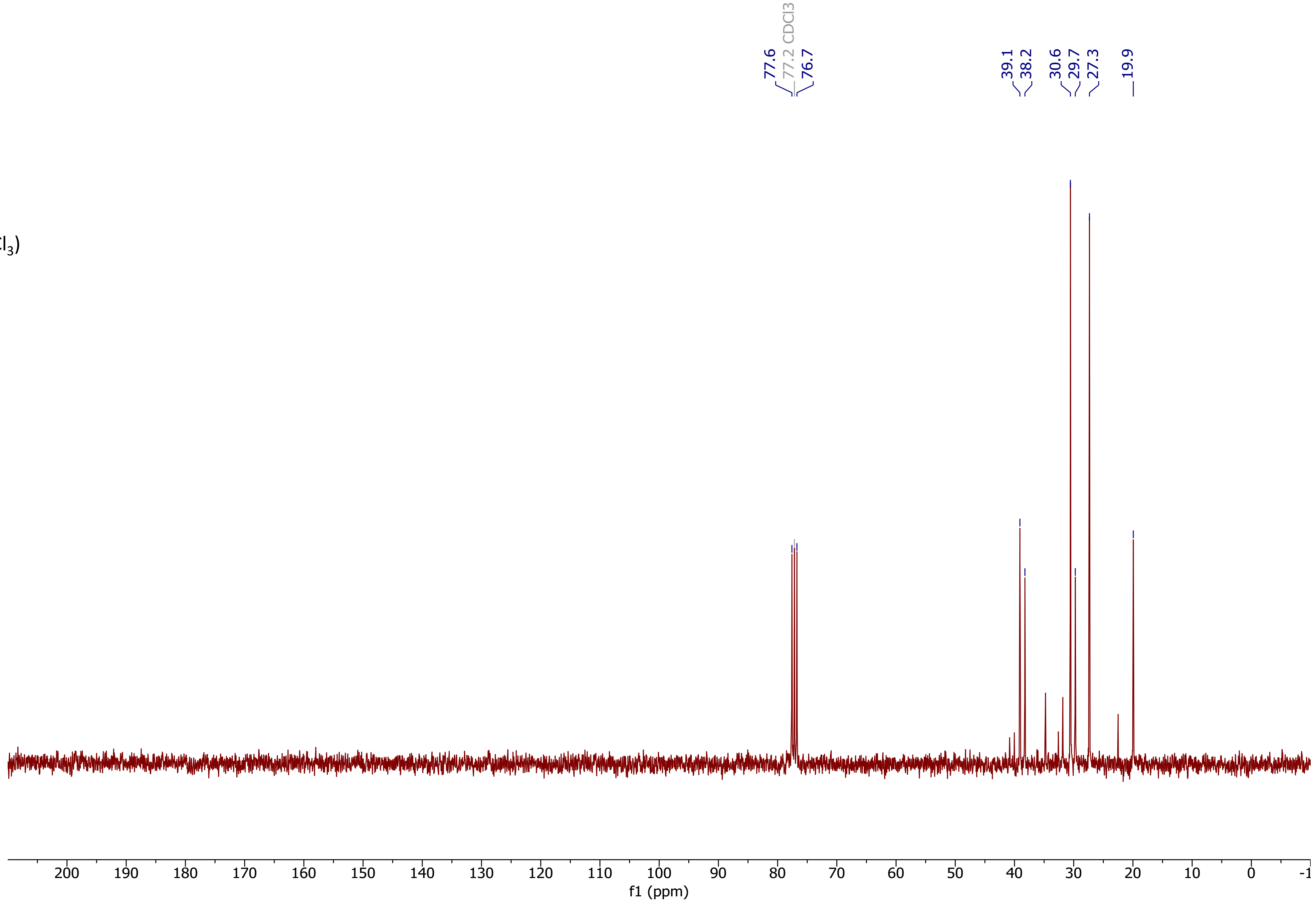

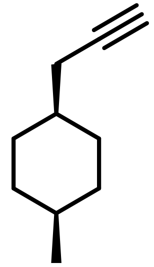

^{Me}*cis*-3e-CCH

^{1}H NMR(300 MHz, $CDCl_3$)

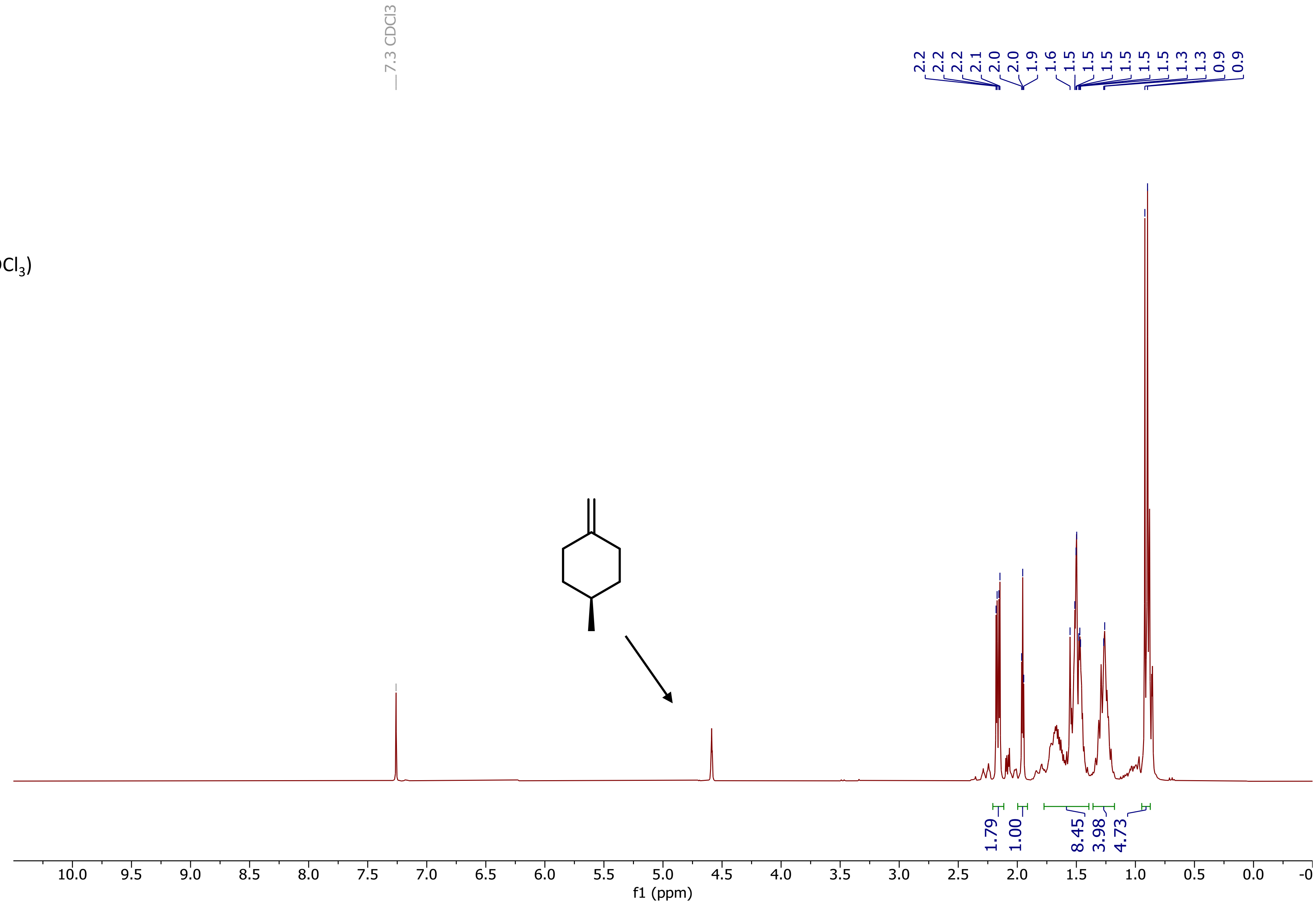

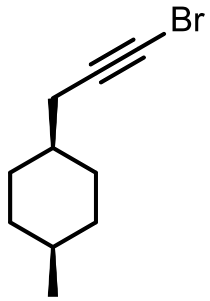

***Me*cis-3e**

^{1}H NMR(300 MHz, $CDCl_3$)

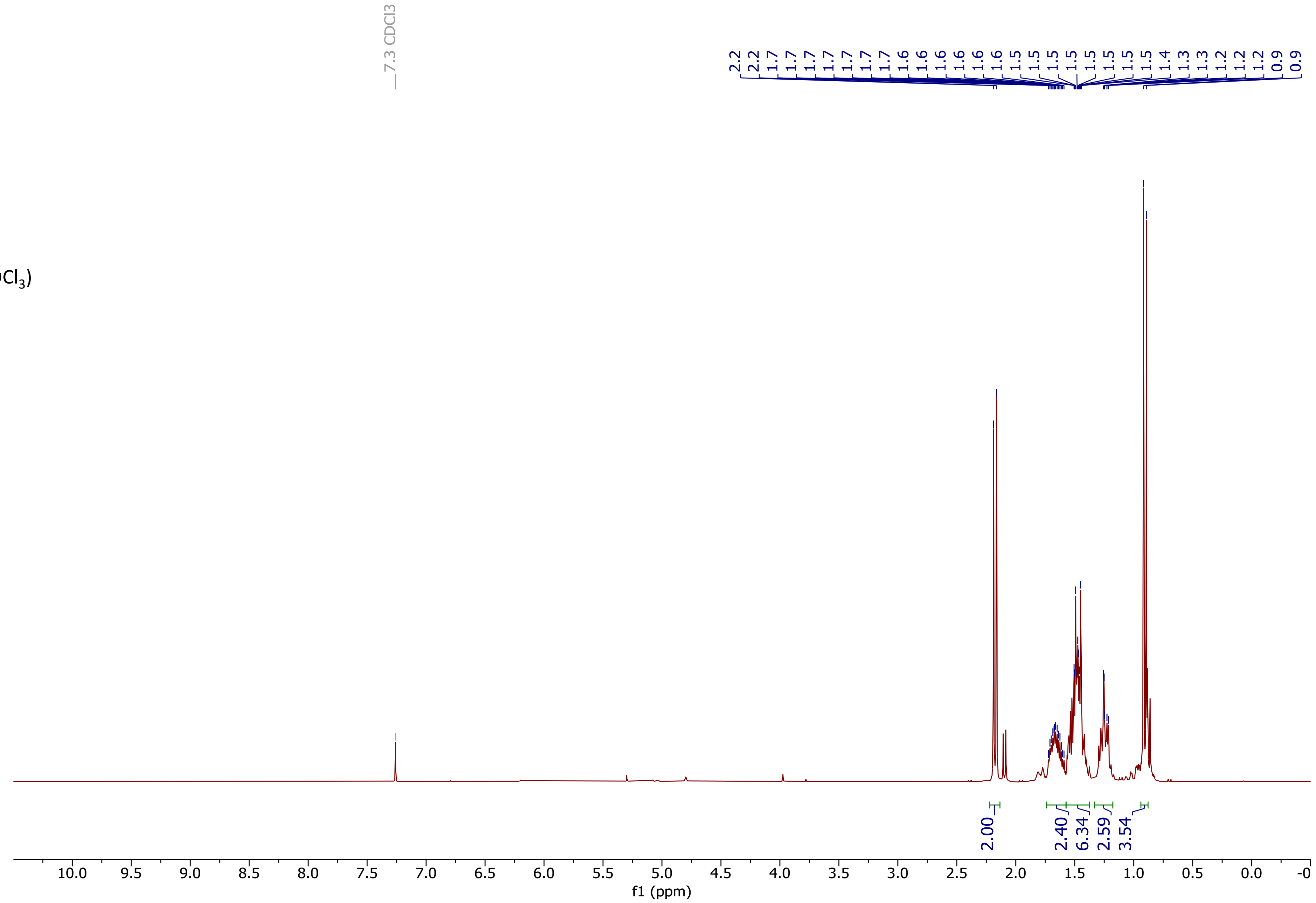

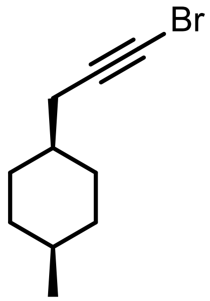

***Me*cis-3e**

^{13}C NMR (75 MHz, $CDCl_3$)

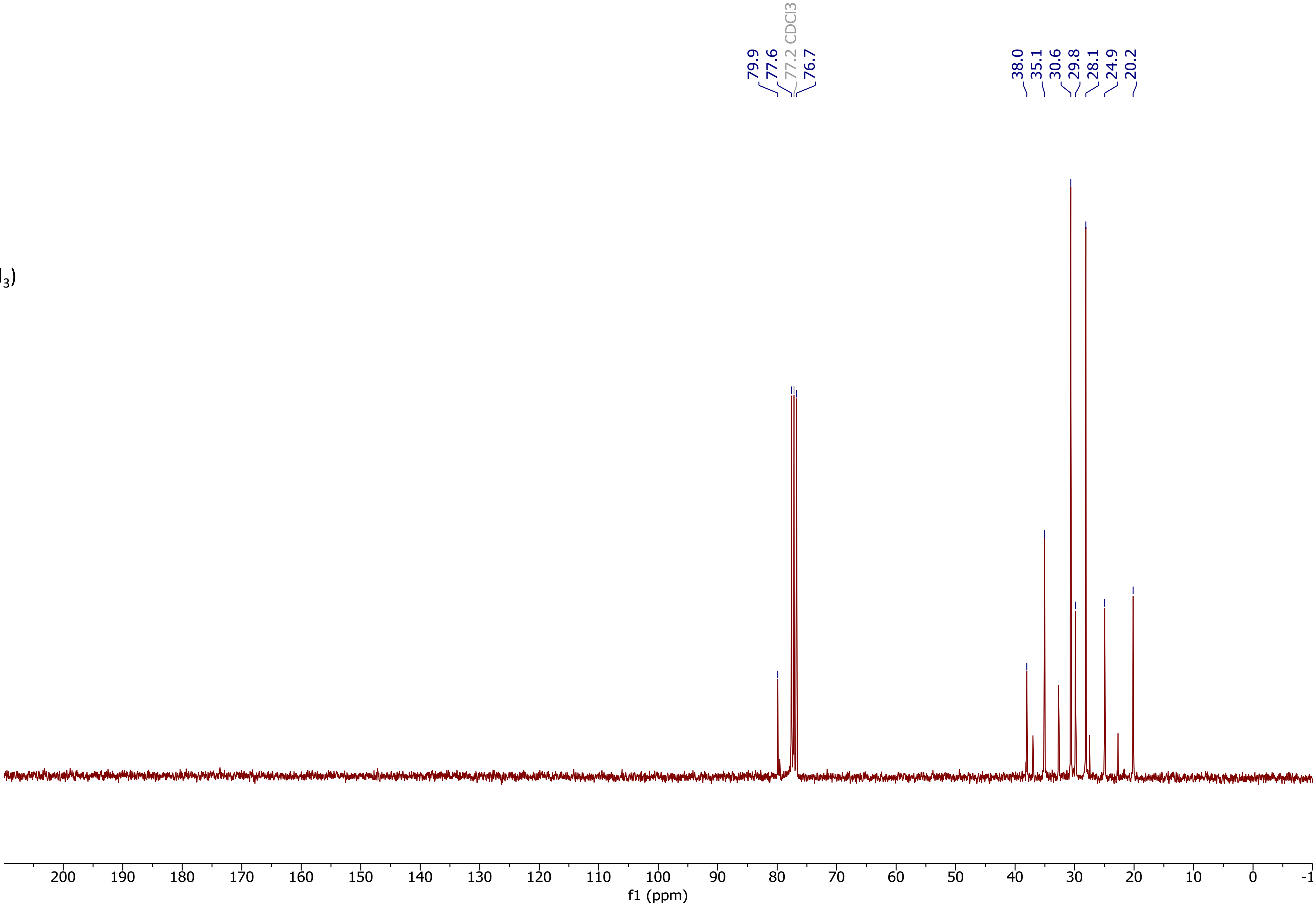

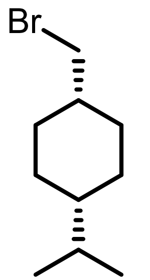

iPr cis-**3e-Br**

^{1}H NMR(300 MHz, $CDCl_3$)

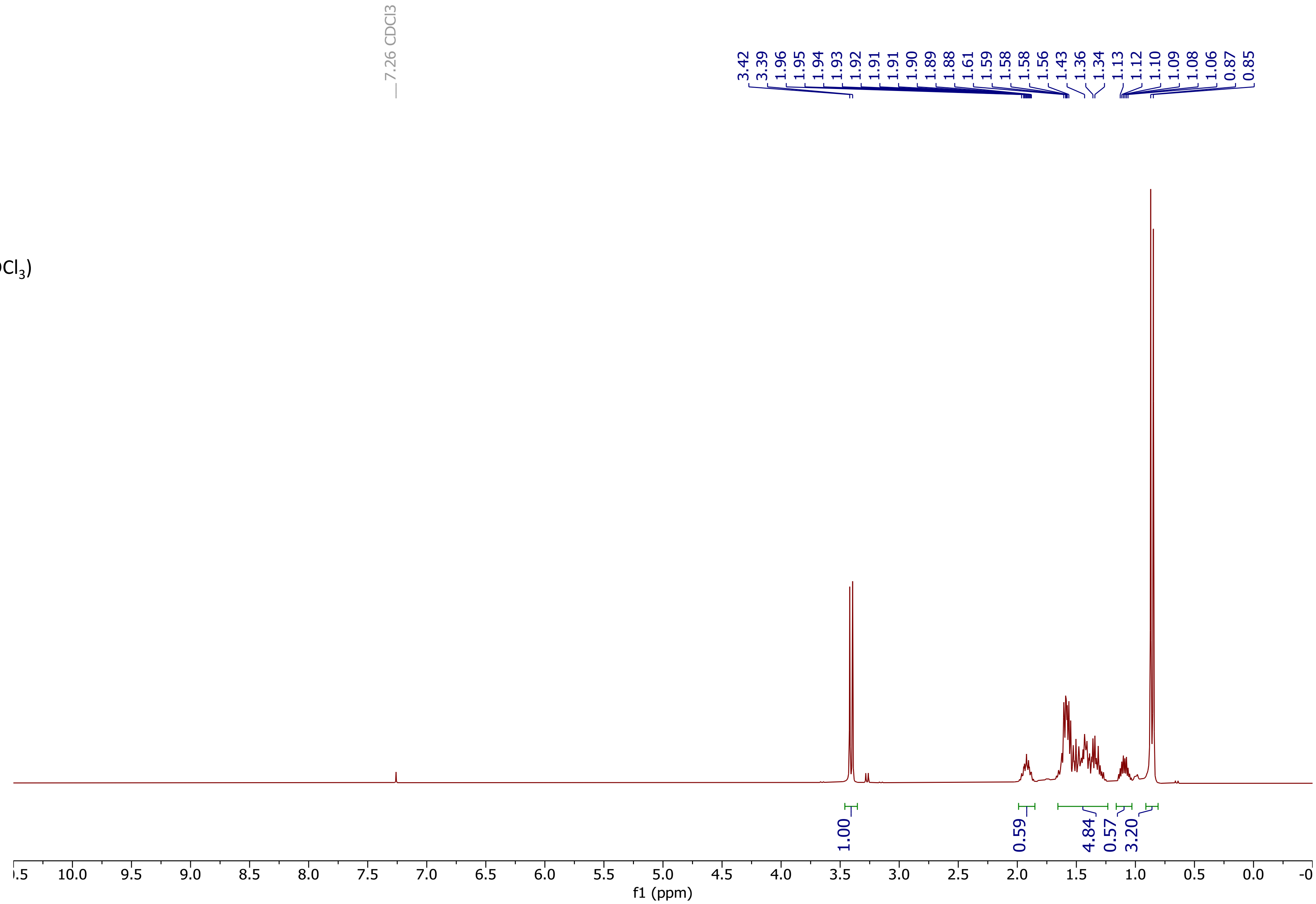

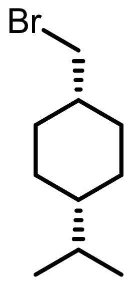

^{iPr}*cis*-3e-Br

^{13}C NMR (75 MHz, $CDCl_3$)

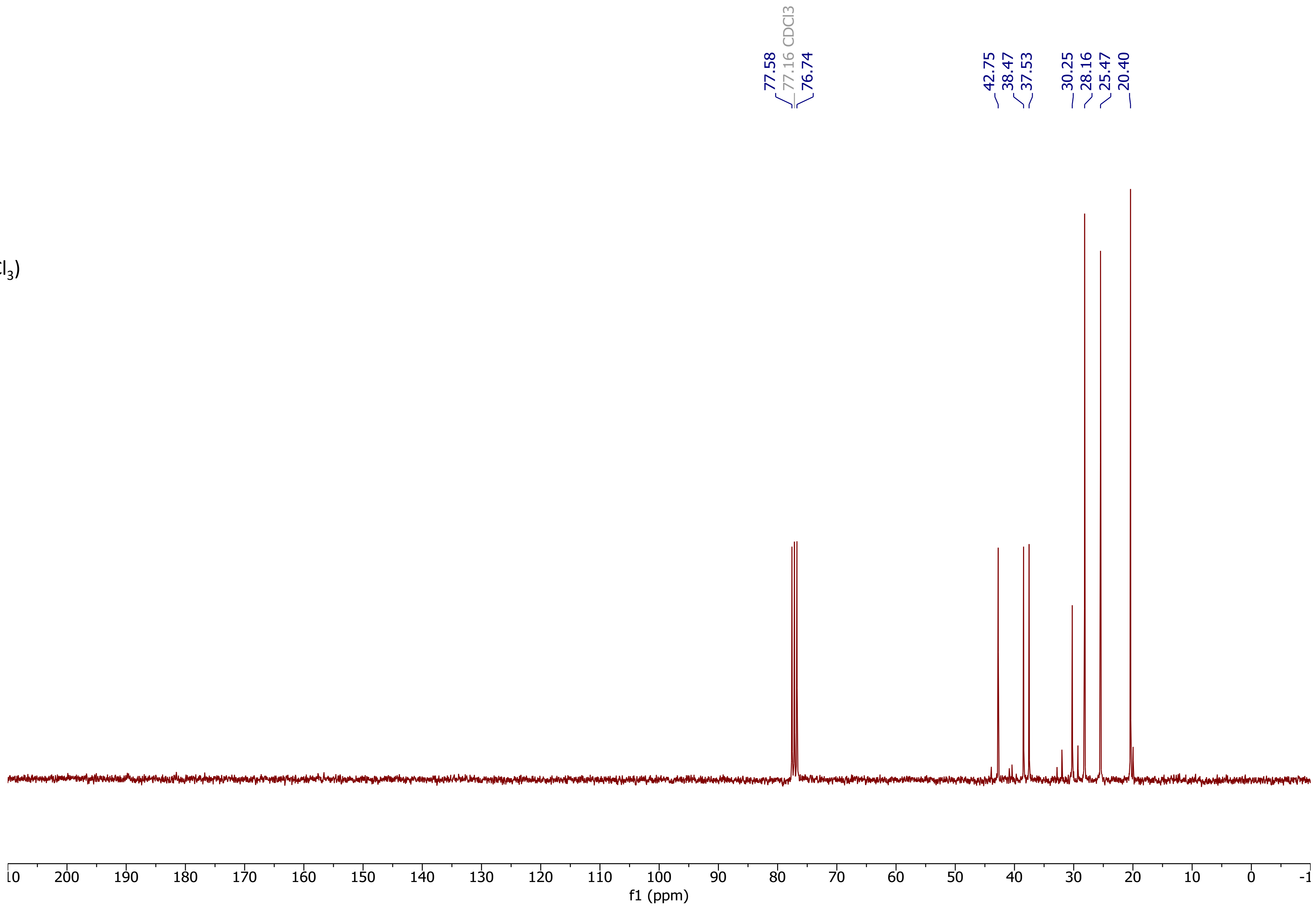

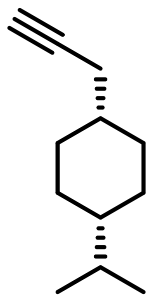

*iPr*cis-3e-CCH

¹H NMR(300 MHz, $CDCl_3$)

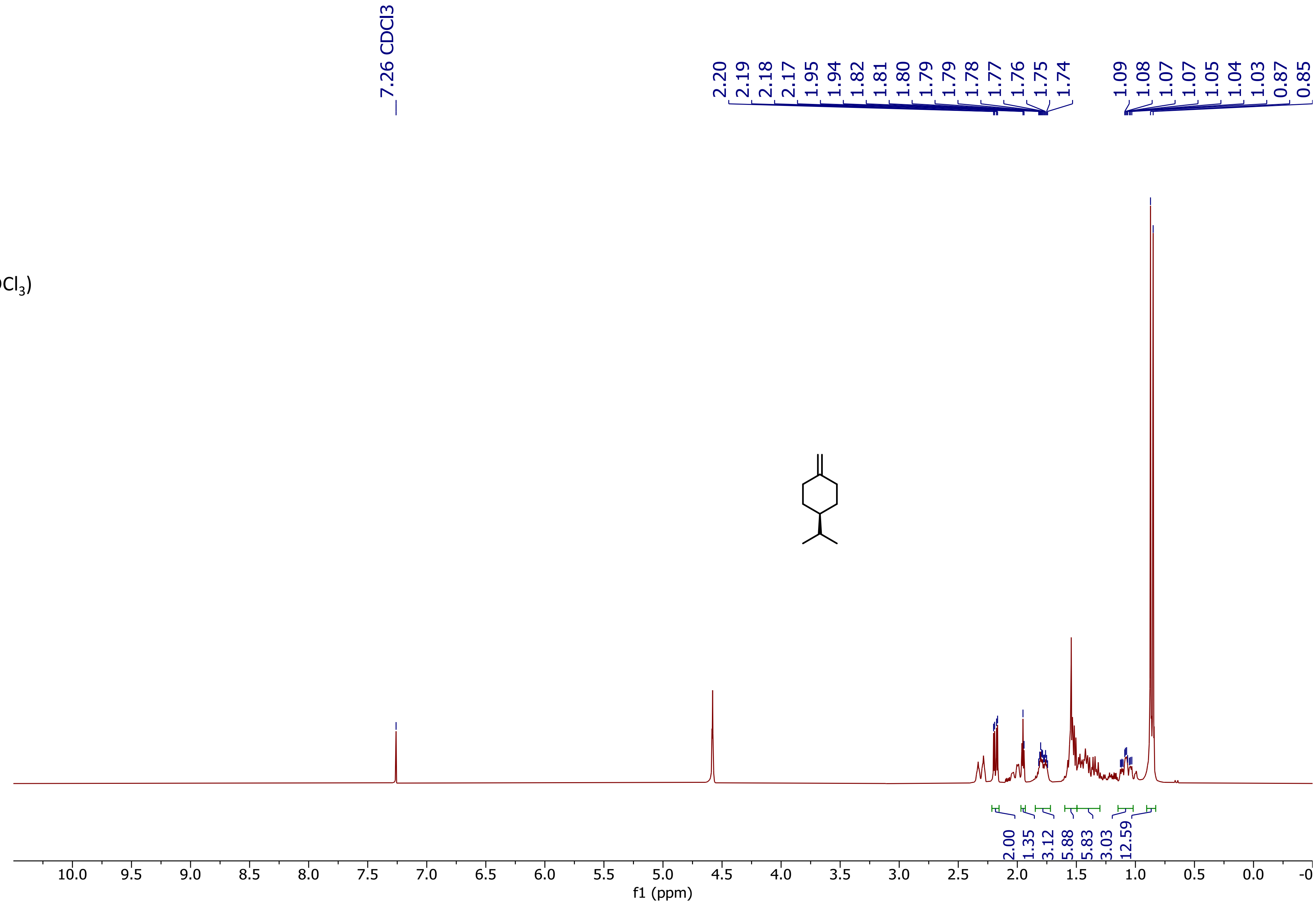

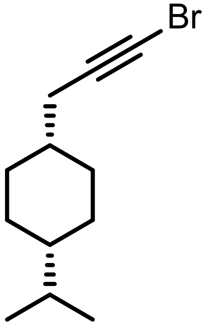

^{iPr}*cis*-**3e**

^{1}H NMR(300 MHz, $CDCl_3$)

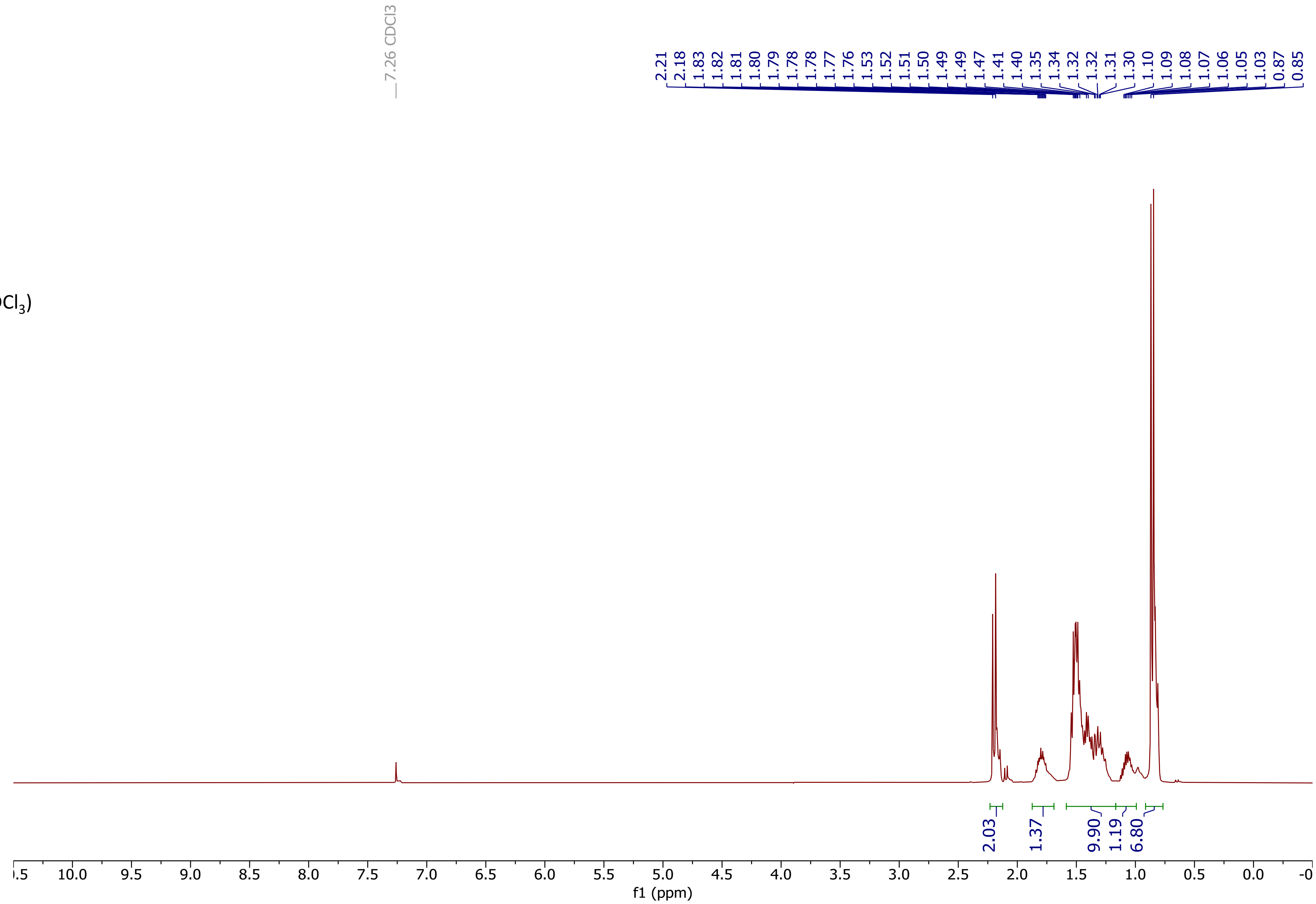

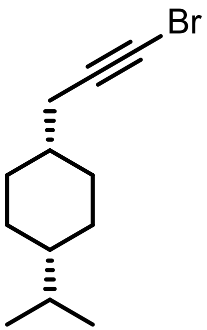

^{iPr}*cis*-**3e**

^{13}C NMR (75 MHz, $CDCl_3$)

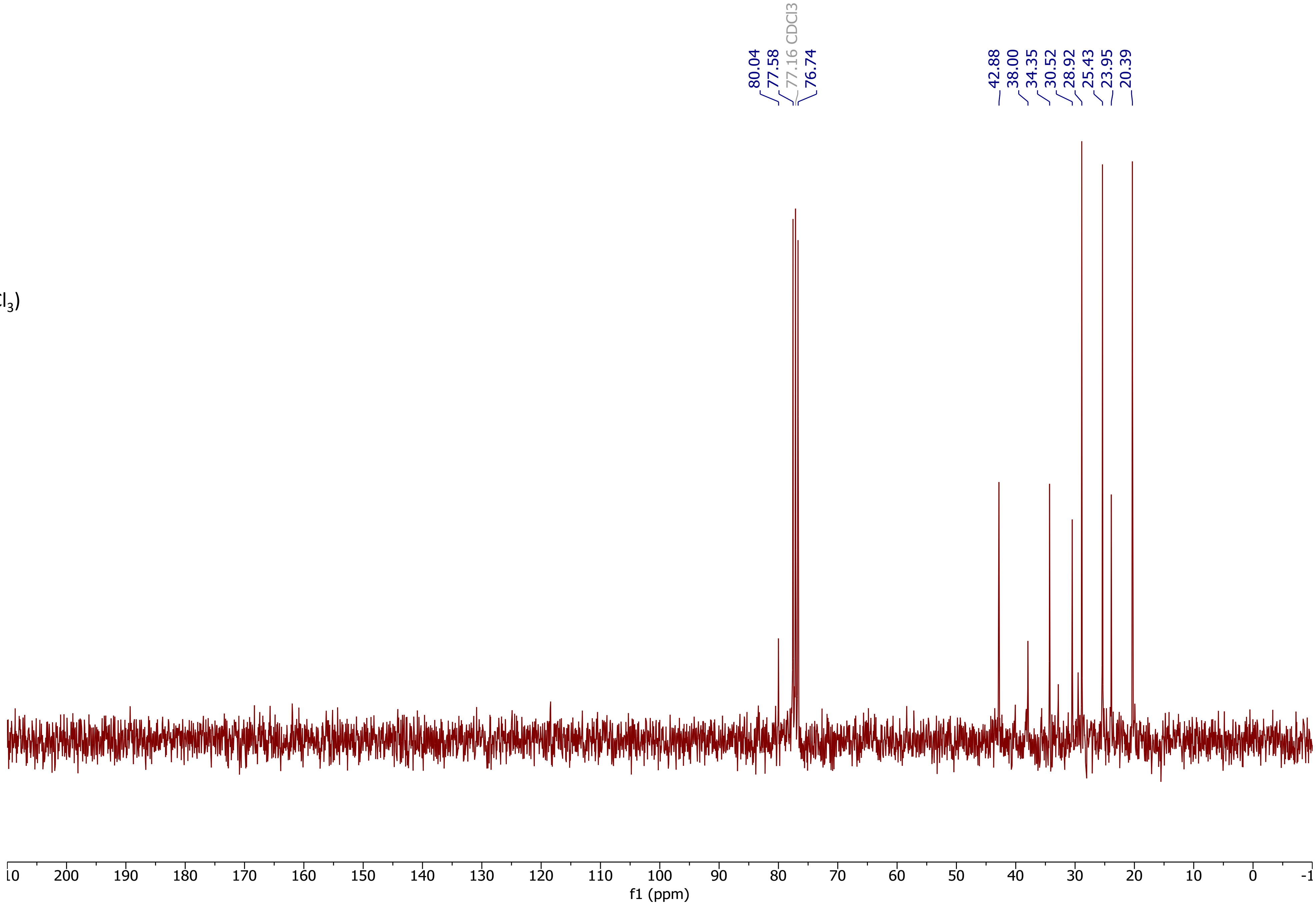

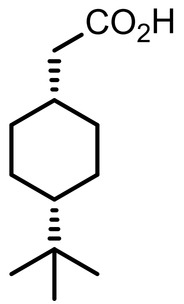

^{tBu}*cis*-3e-CO_2H

-crude-

^{1}H NMR(300 MHz, $CDCl_3$)

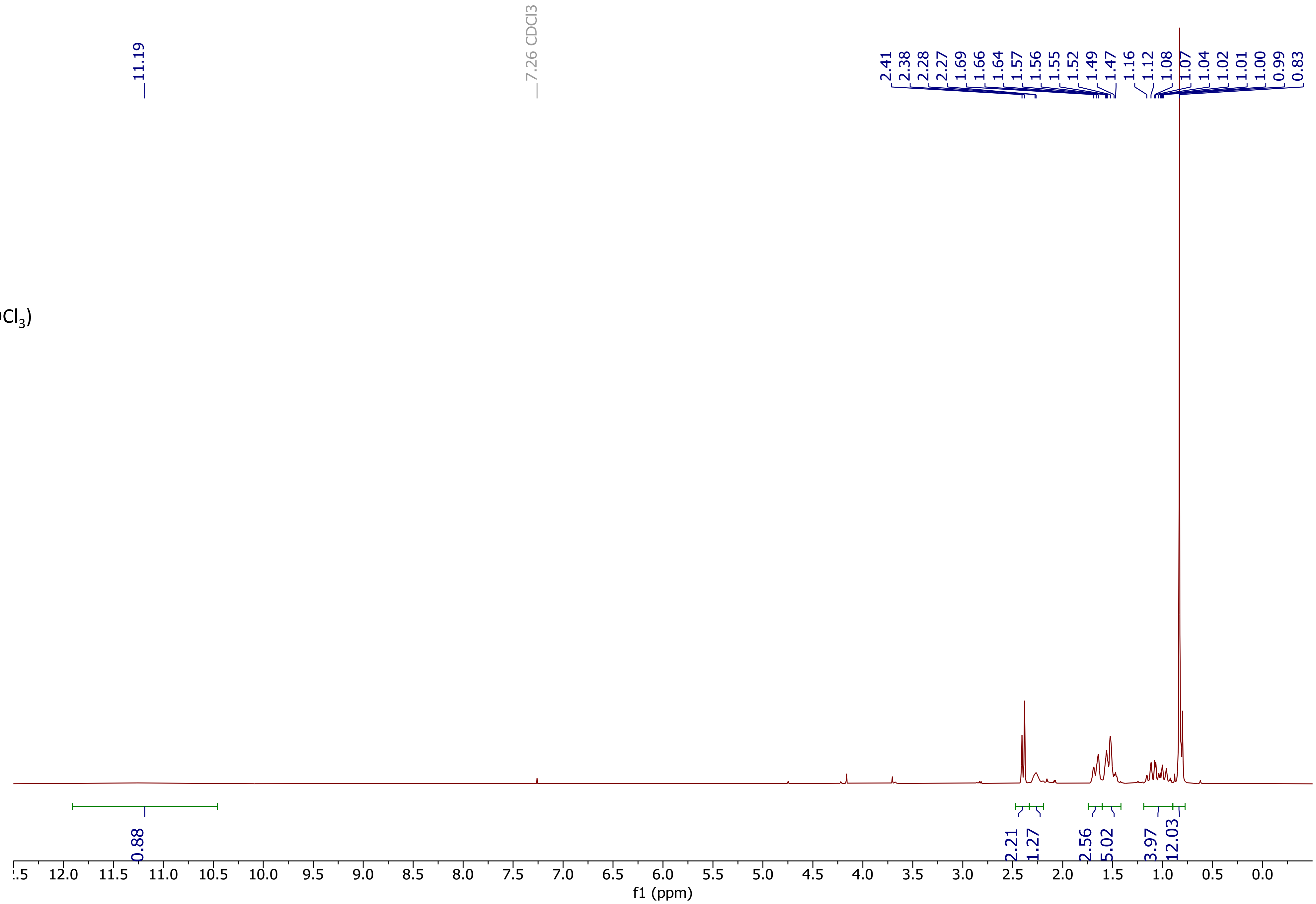

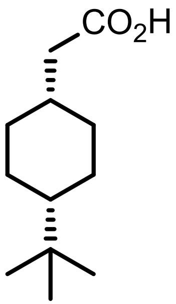

^{tBu}*cis*-**3e-CO_2H**

-crude-

^{13}C NMR (75 MHz, $CDCl_3$)

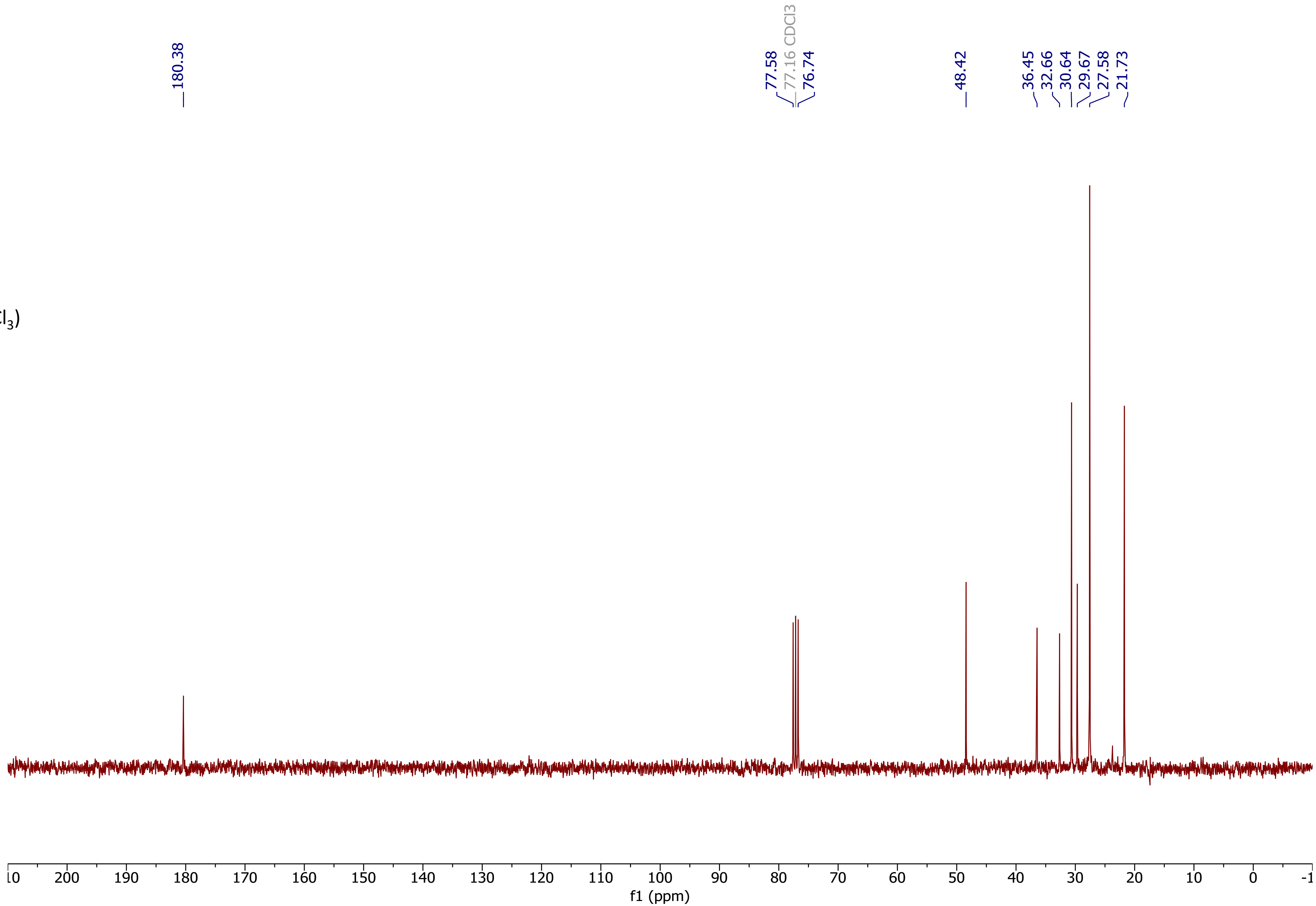

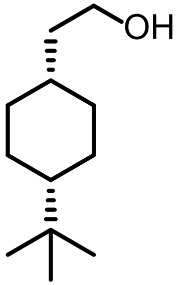

^{tBu}*cis*-3e-OH

-crude-

^{1}H NMR(300 MHz, $CDCl_3$)

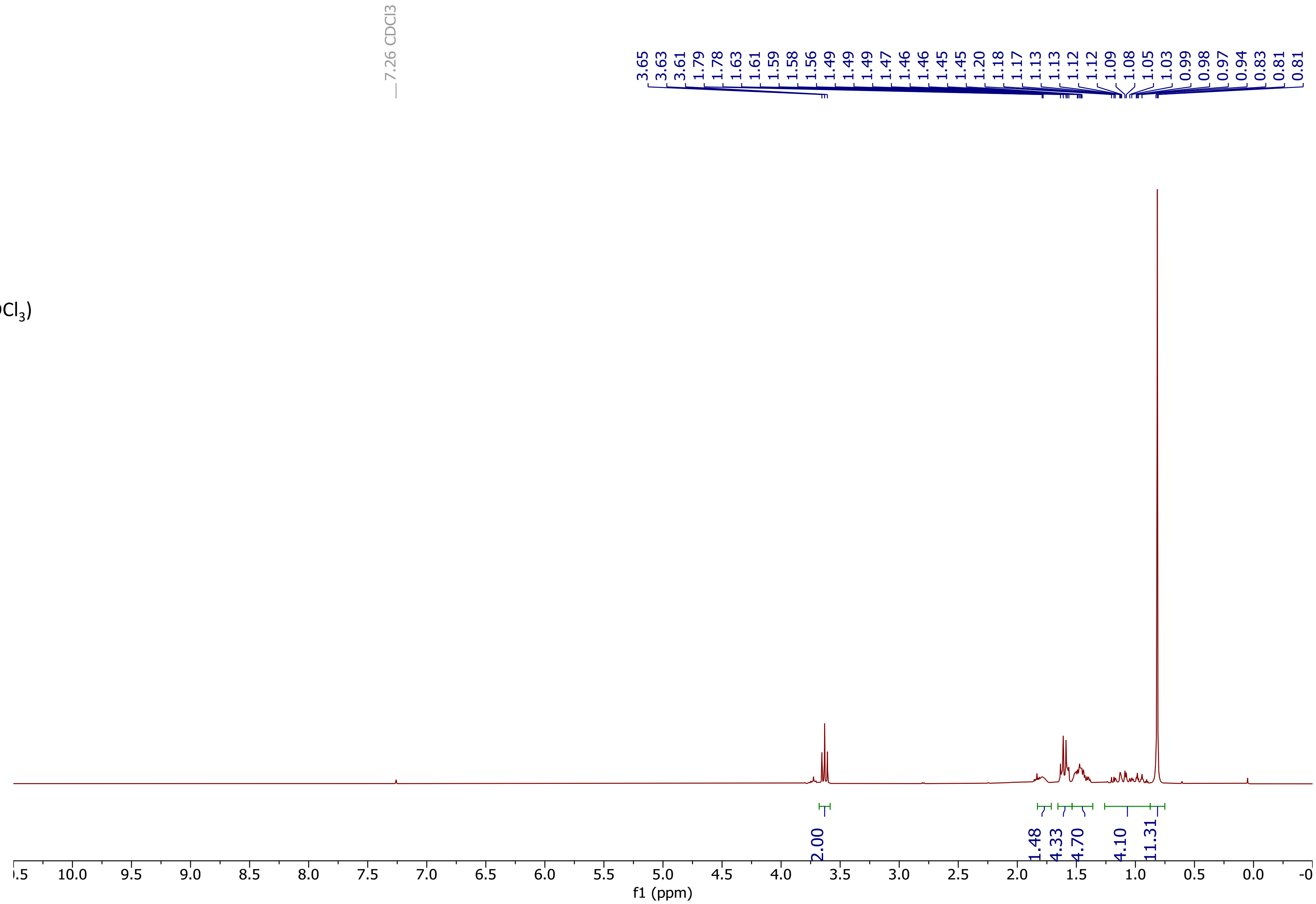

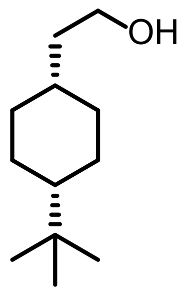

^{tBu}*cis*-3e-OH

-crude-

^{13}C NMR (75 MHz, $CDCl_3$)

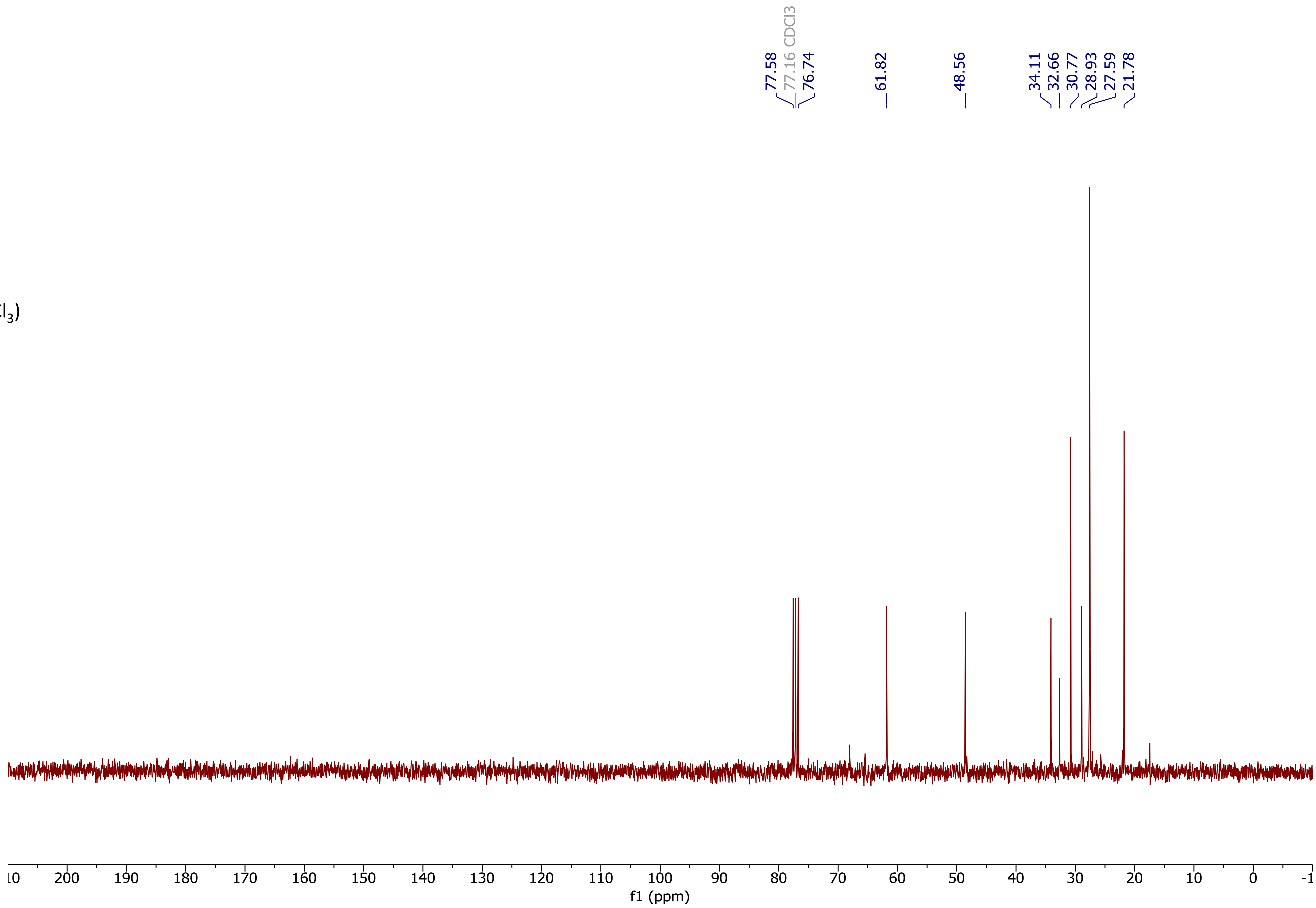

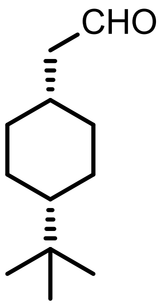

^{tBu}*cis*-3e-OH

-crude-

^{1}H NMR(300 MHz, $CDCl_3$)

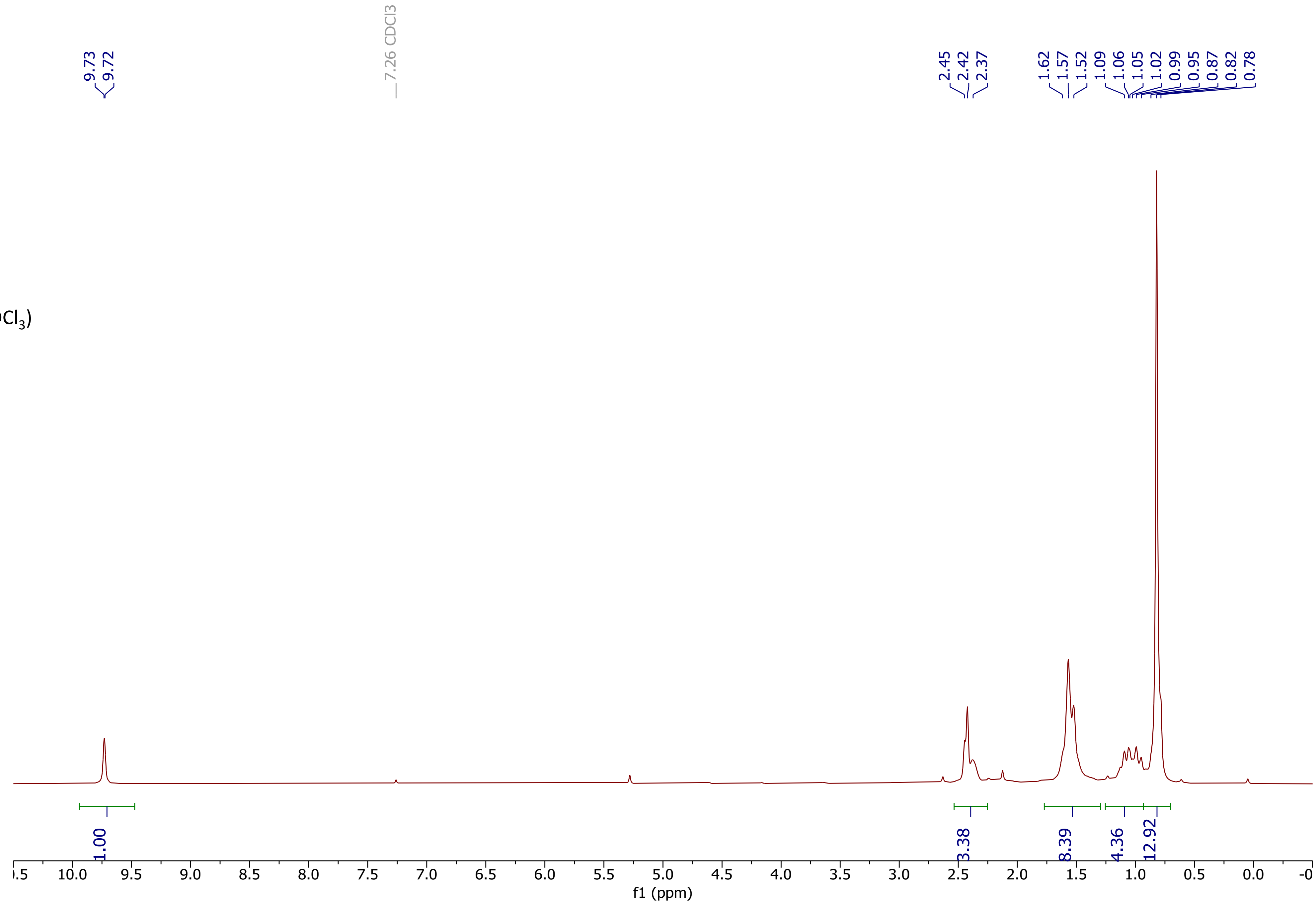

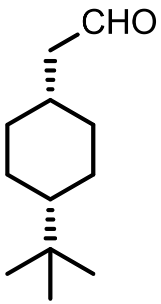

^{tBu}*cis*-3e-OH

-crude-

^{13}C NMR (75 MHz, $CDCl_3$)

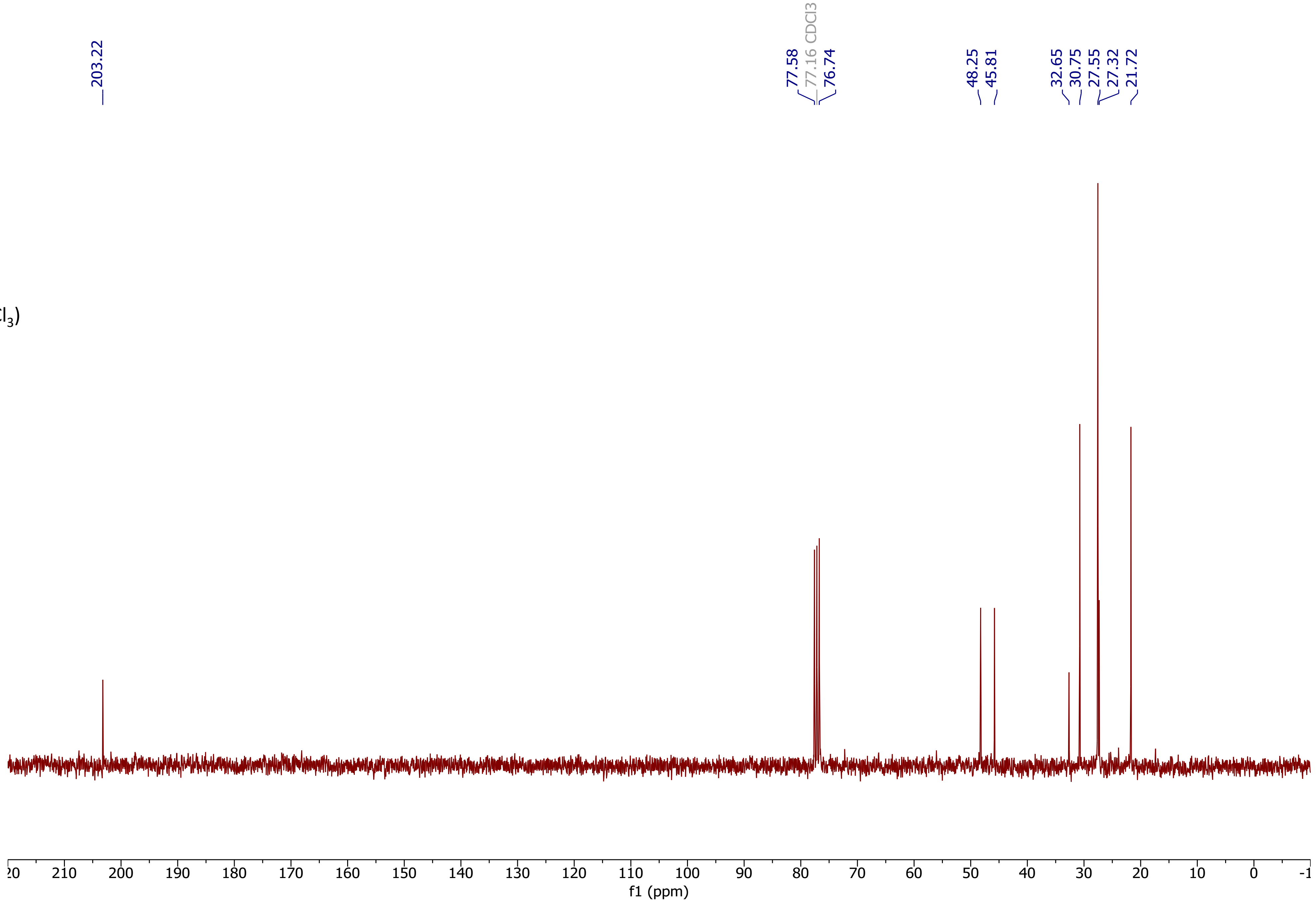

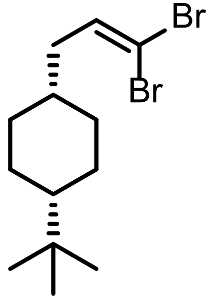

^{tBu}*cis*-3e-CBr$_2$

^{1}H NMR(300 MHz, $CDCl_3$)

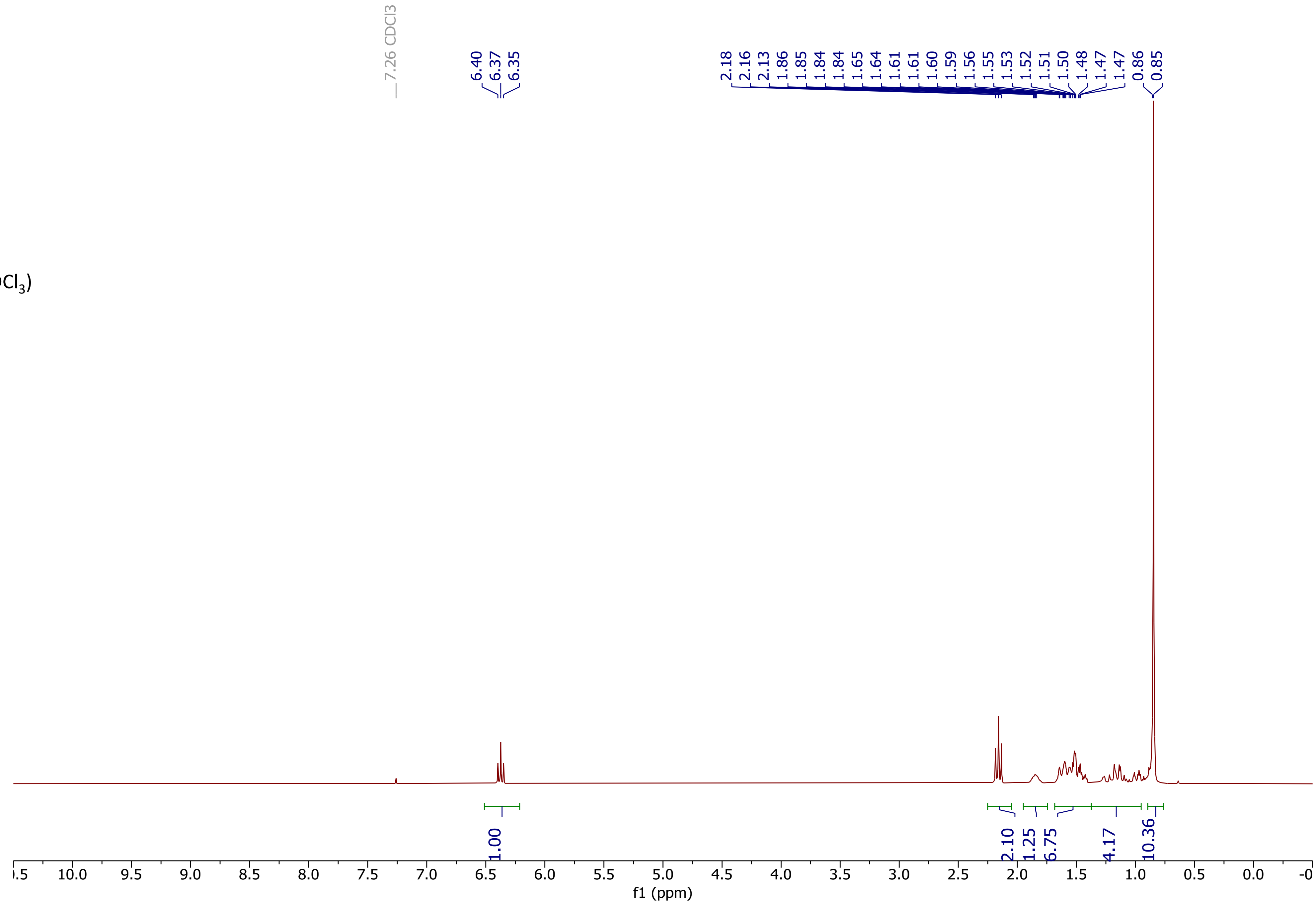

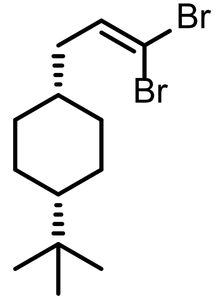

^{tBu}*cis*-3e-CBr$_2$

^{13}C NMR (75 MHz, $CDCl_3$)

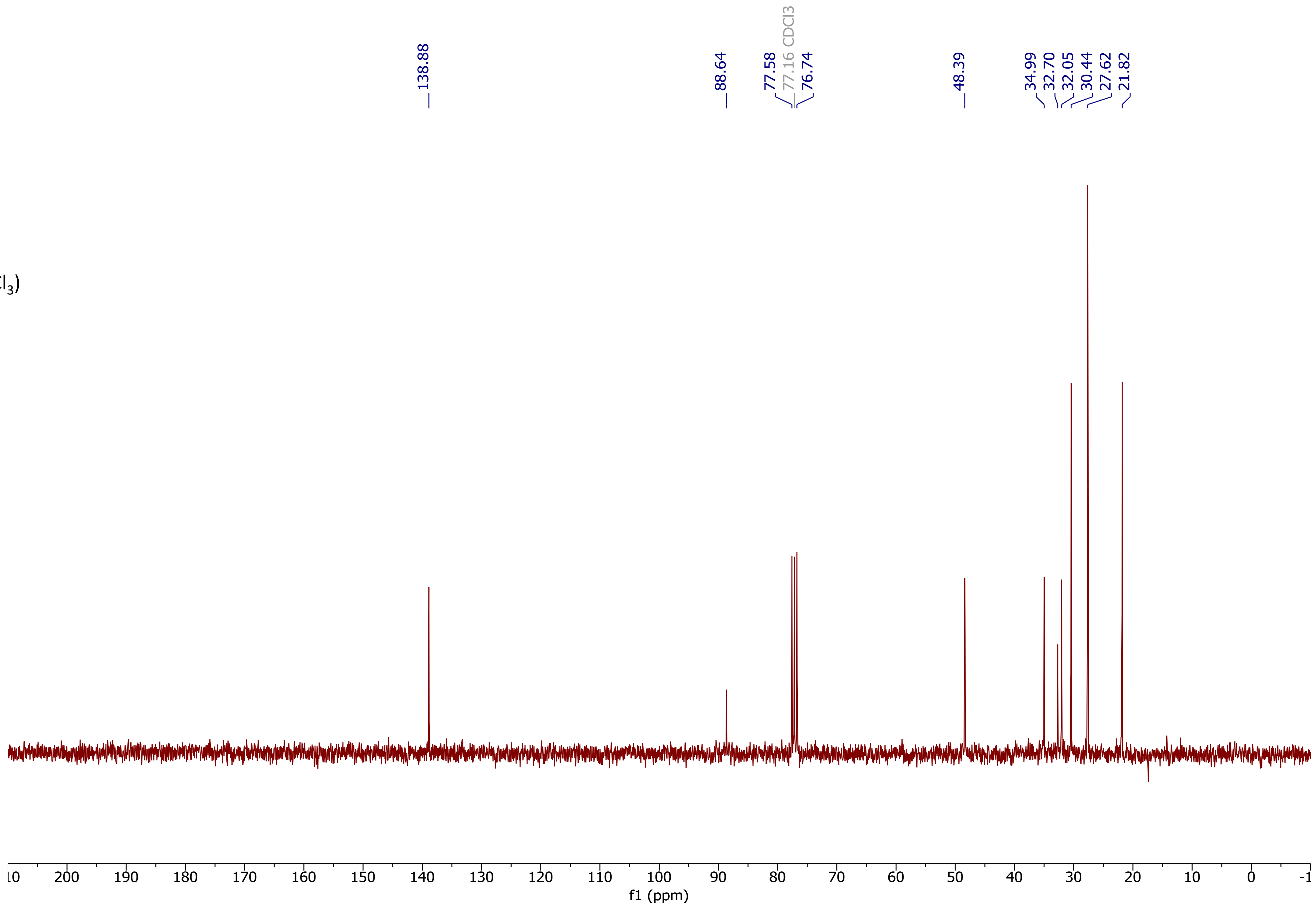

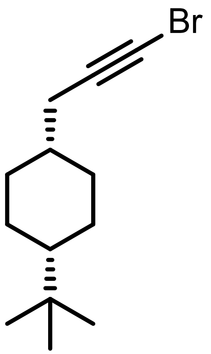

^{1}H NMR(300 MHz, $CDCl_3$)

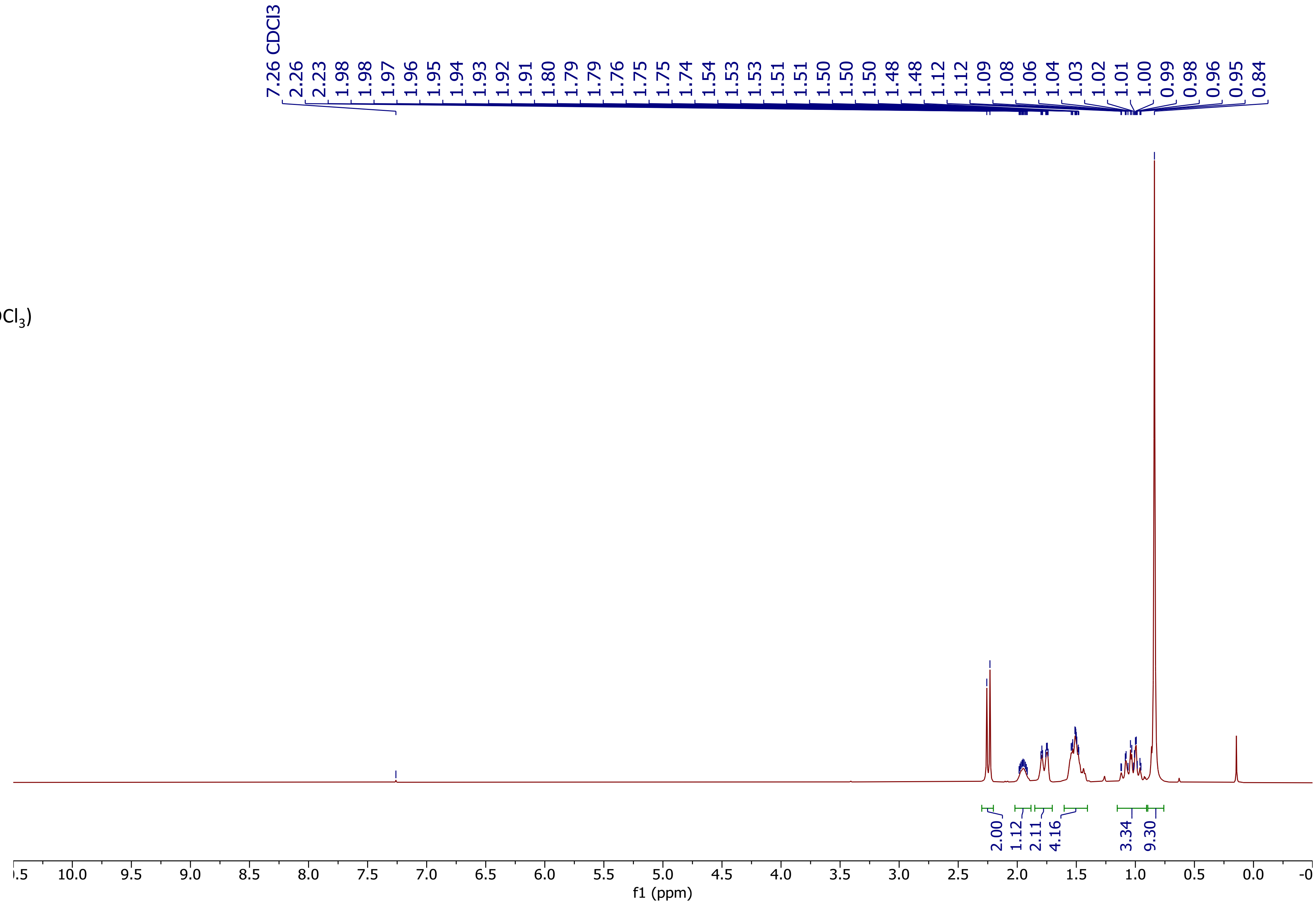

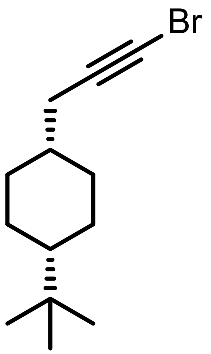

^{tBu}*cis*-**3e**

^{13}C NMR (75 MHz, $CDCl_3$)

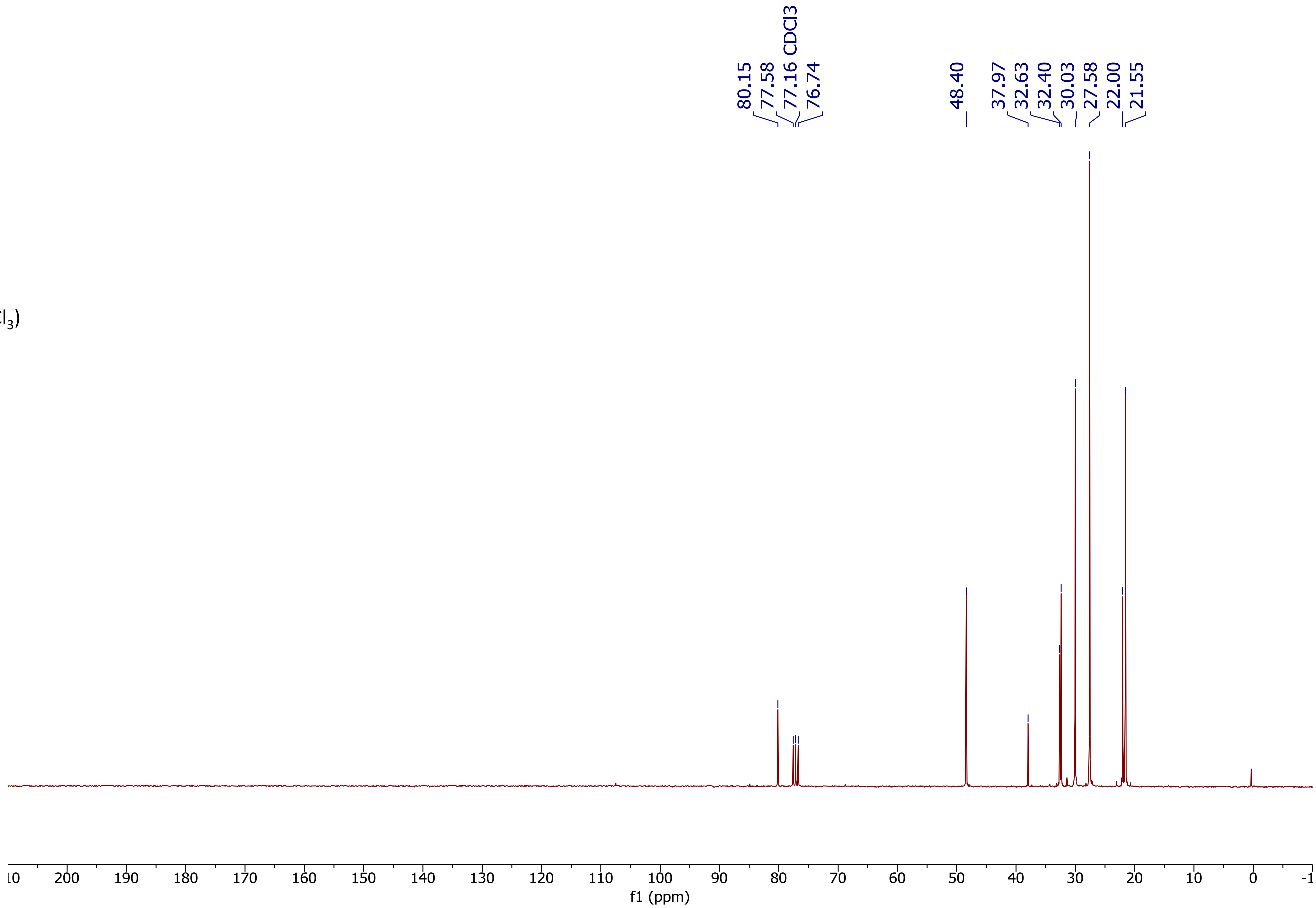

***trans*-3e**

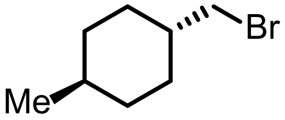

***Me*trans-3e-Br**

¹H NMR(300 MHz, $CDCl_3$)

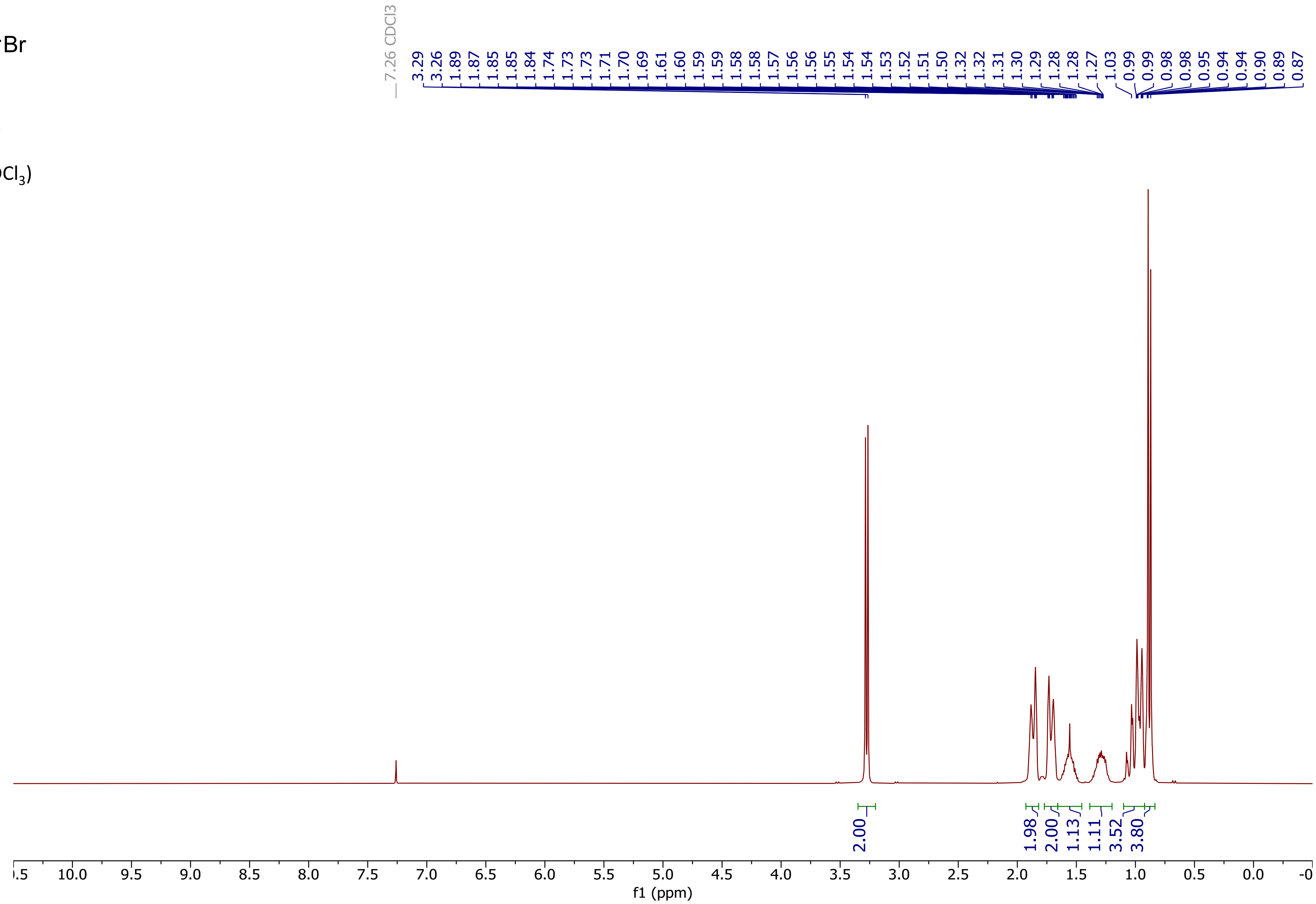

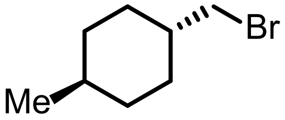

^{Me}*trans*-3e-Br

^{13}C NMR (75 MHz, $CDCl_3$)

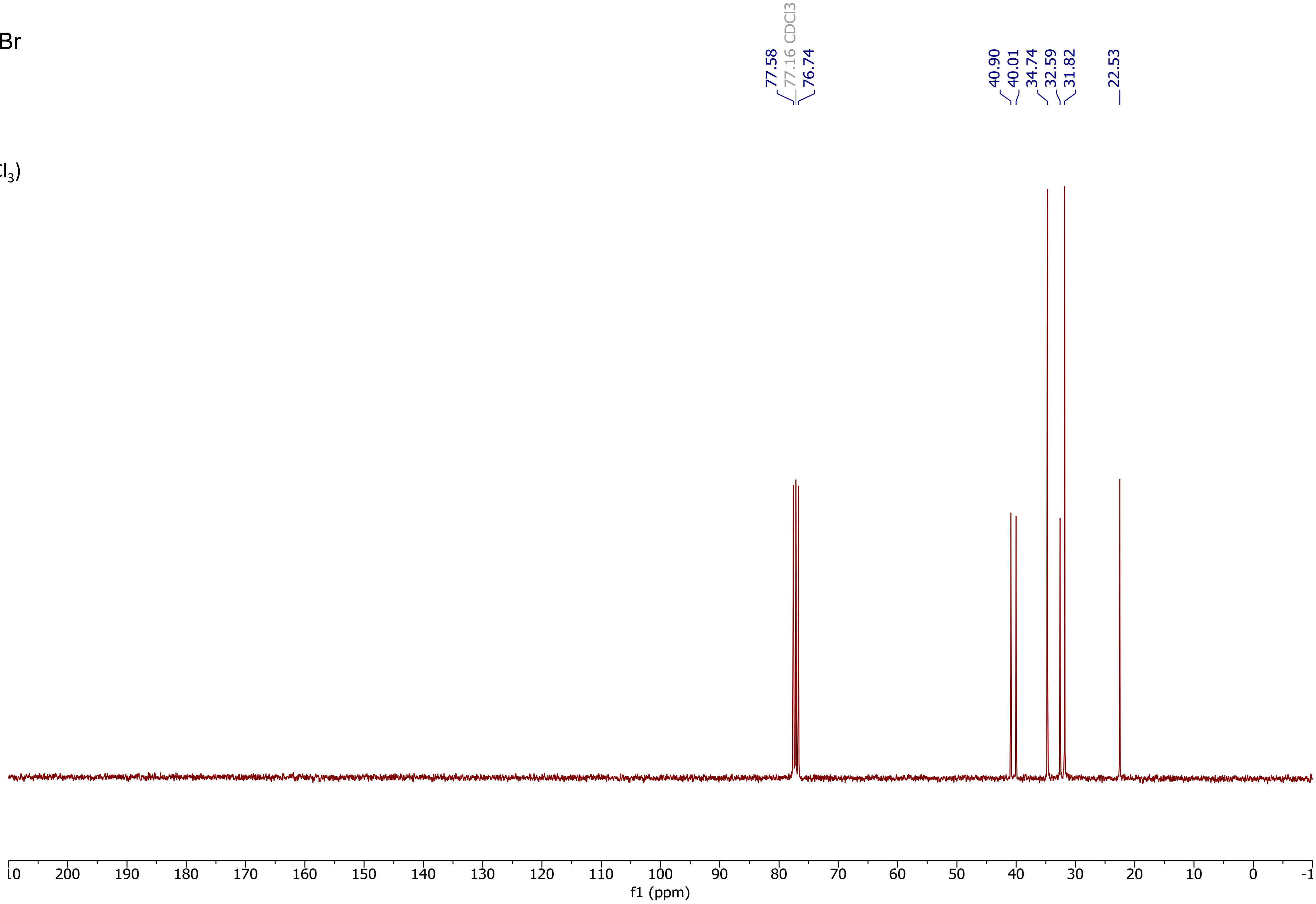

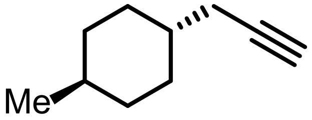

^Me^trans-3e-CCH

^{1}H NMR(300 MHz, $CDCl_3$)

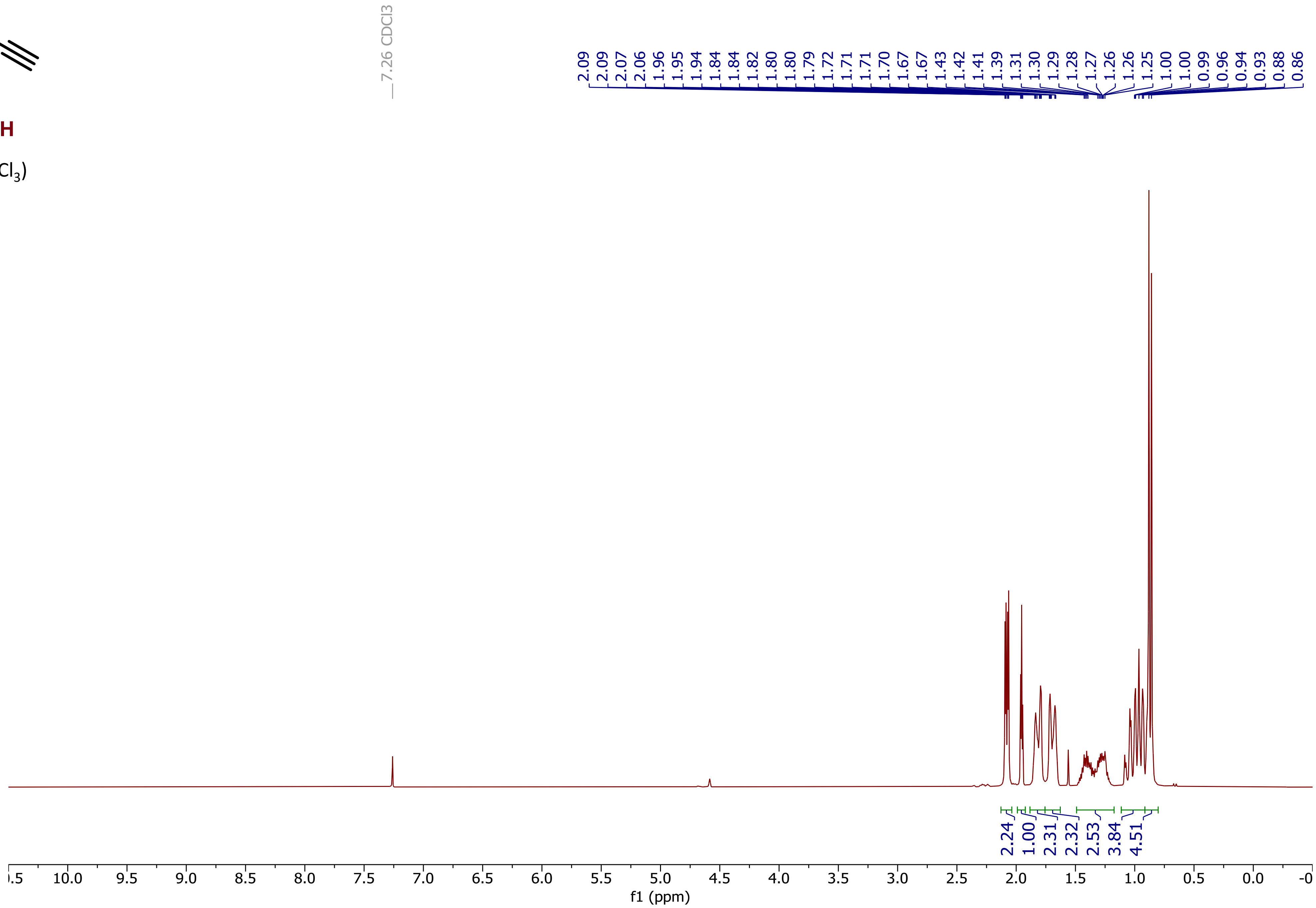

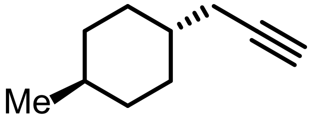

***Me*trans-3e-CCH**

^{13}C NMR (75 MHz, $CDCl_3$)

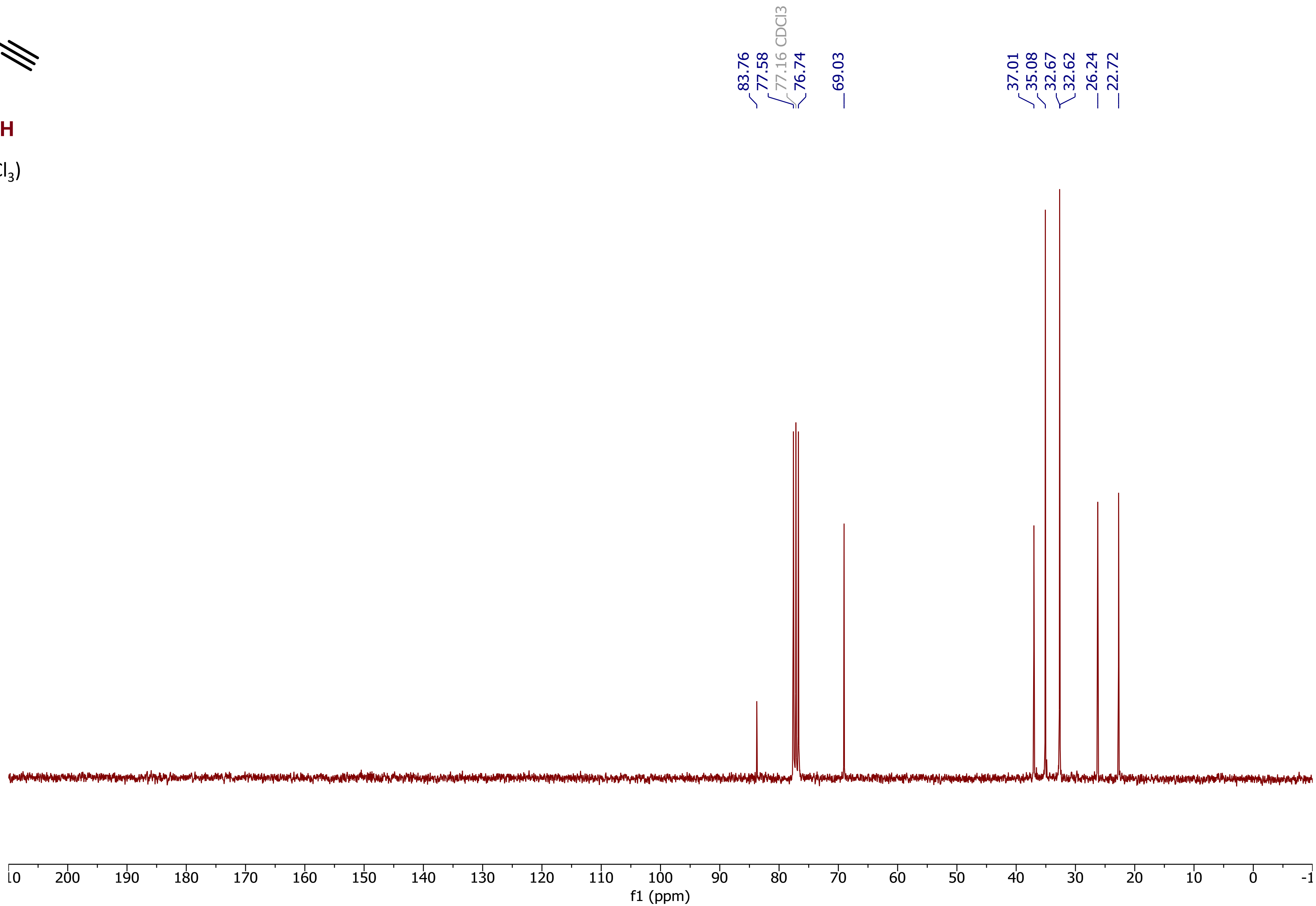

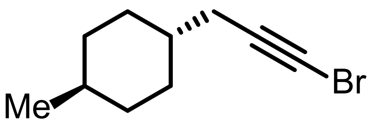

***Me*trans-3e**

¹H NMR(300 MHz, $CDCl_3$)

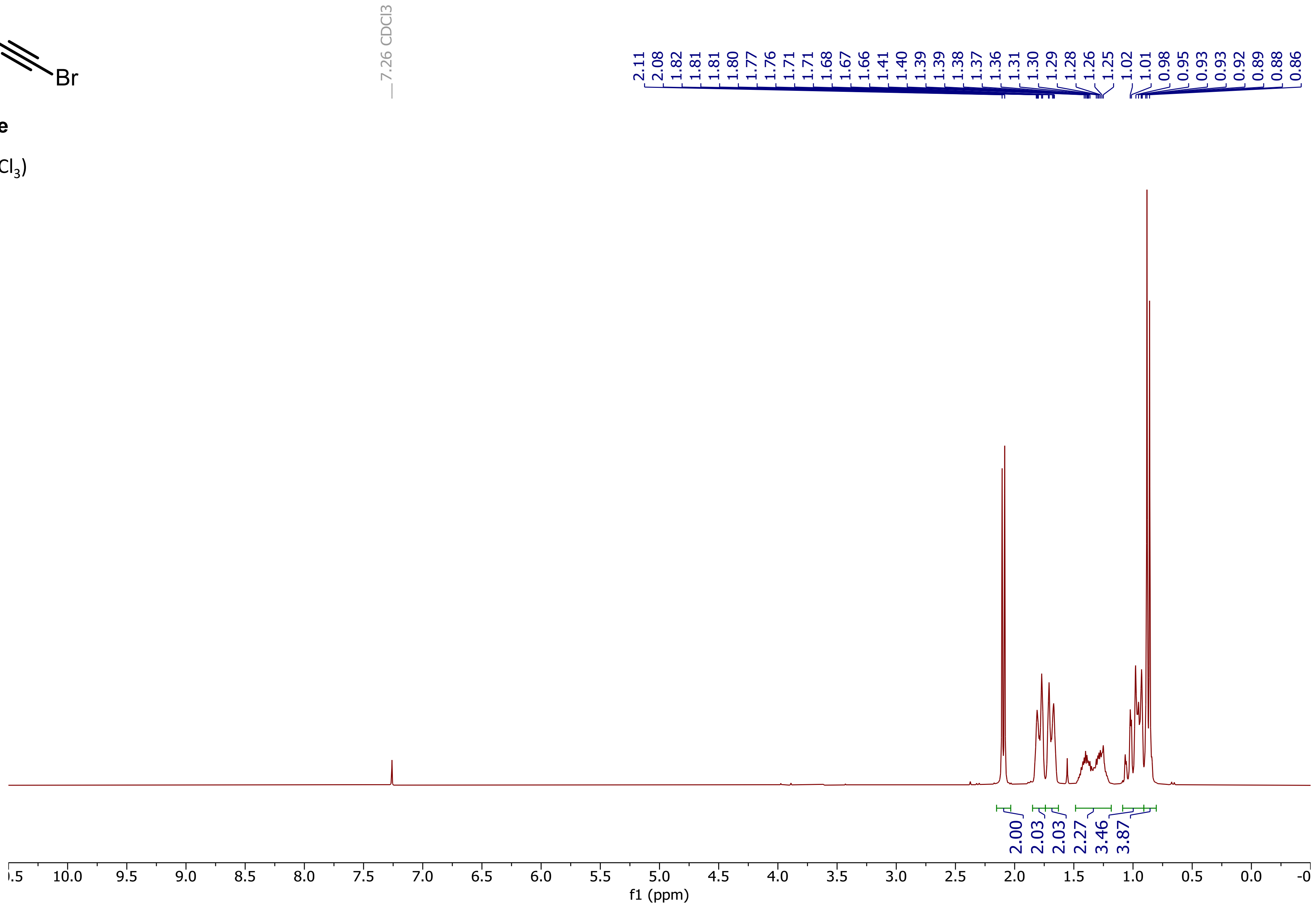

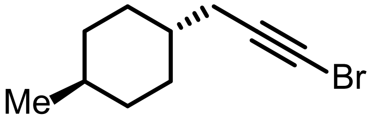

***Me*trans-3e**

^{13}C NMR (75 MHz, $CDCl_3$)

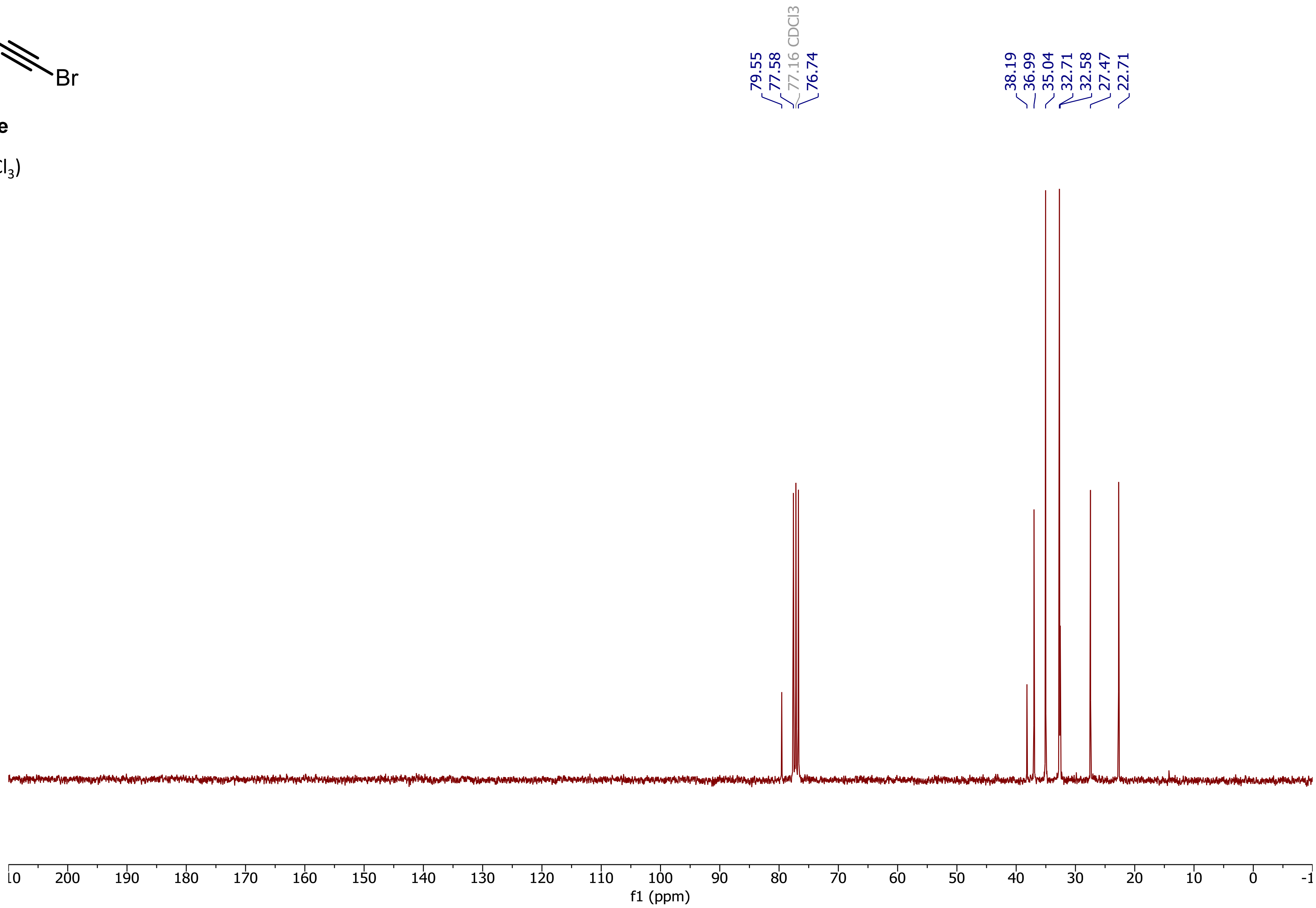

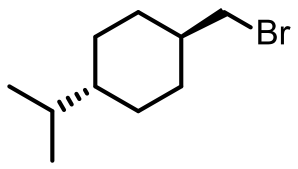

^{iPr}***trans*-3e-Br**

^{1}H NMR(300 MHz, $CDCl_3$)

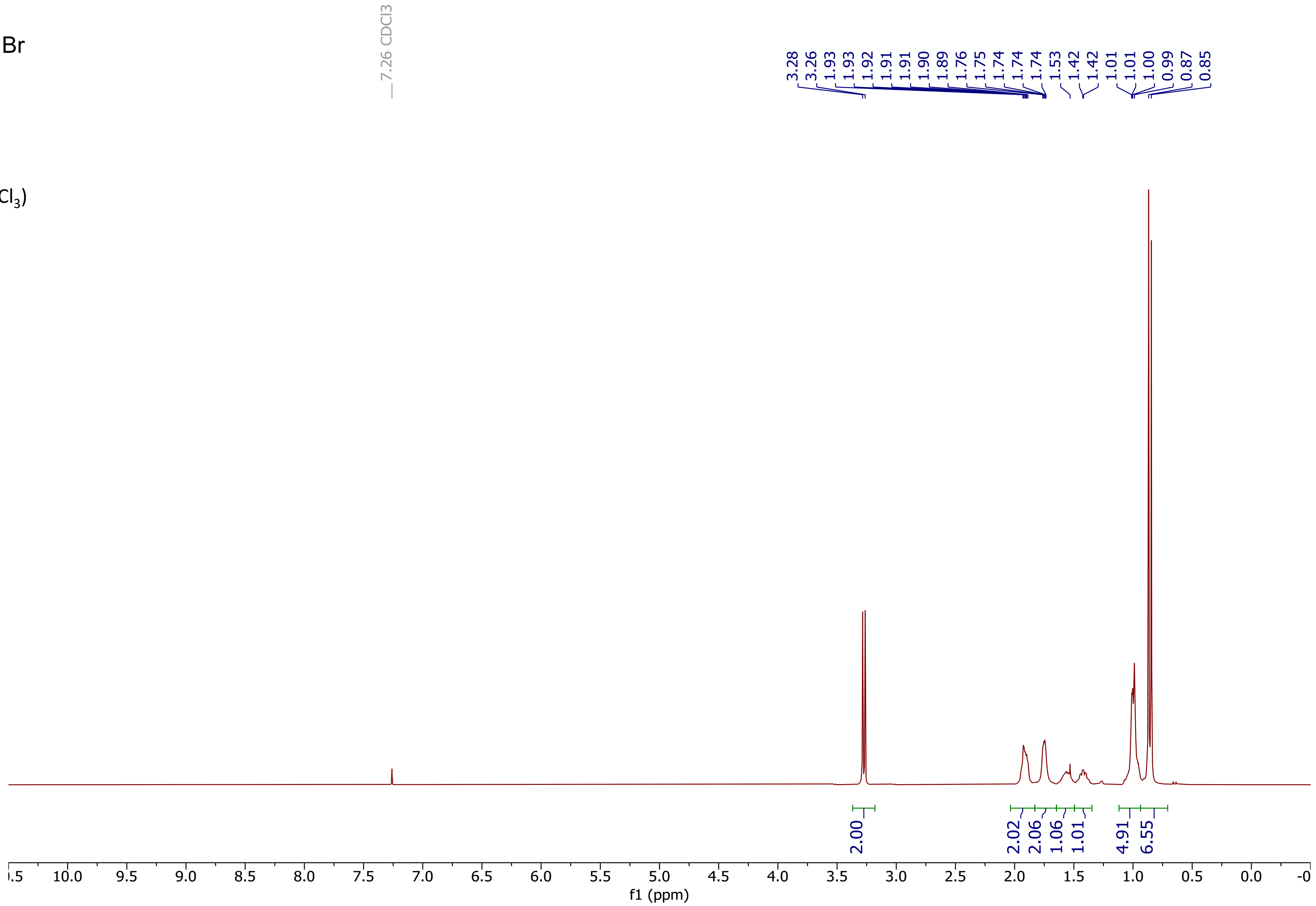

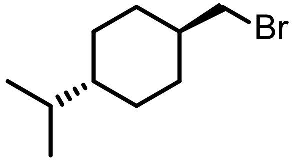

^{iPr}***trans*-3e-Br**

^{13}C NMR (75 MHz, $CDCl_3$)

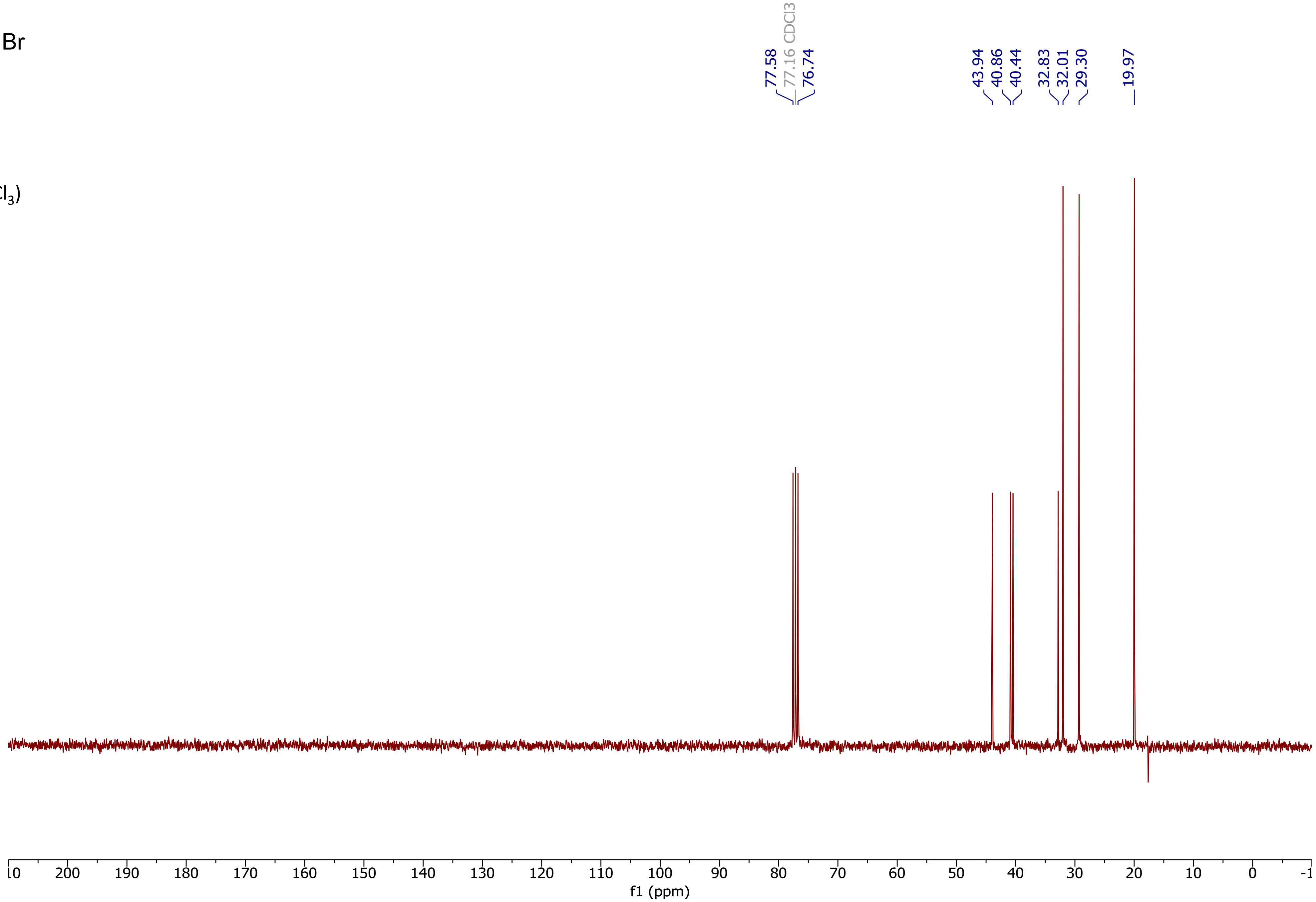

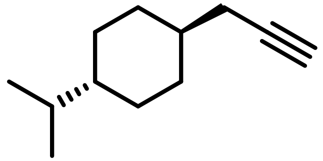

^{iPr}*trans*-3e-CCH

^{1}H NMR(300 MHz, $CDCl_3$)

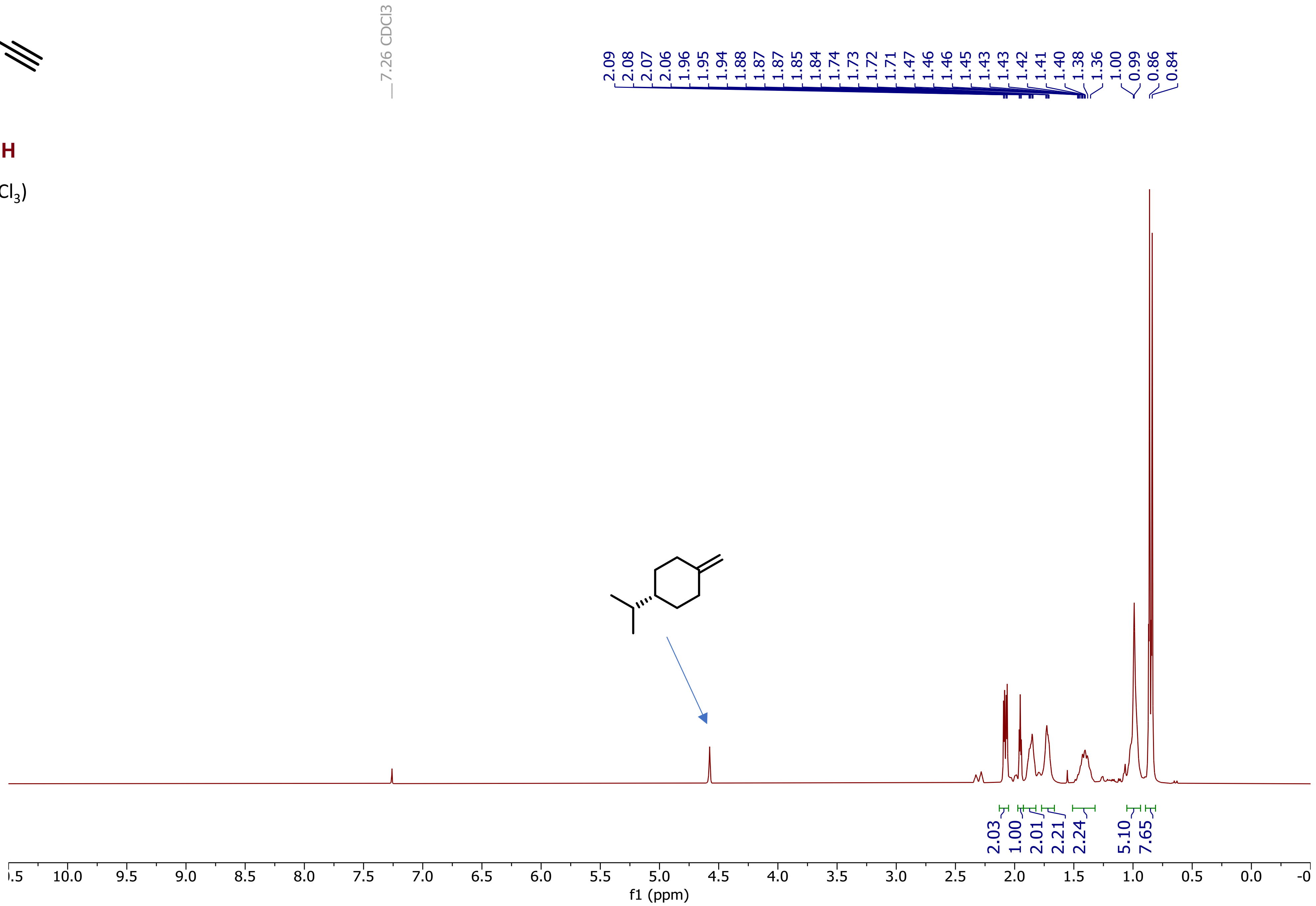

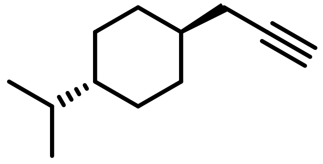

iPr trans-3e-CCH

^{13}C NMR (75 MHz, $CDCl_3$)

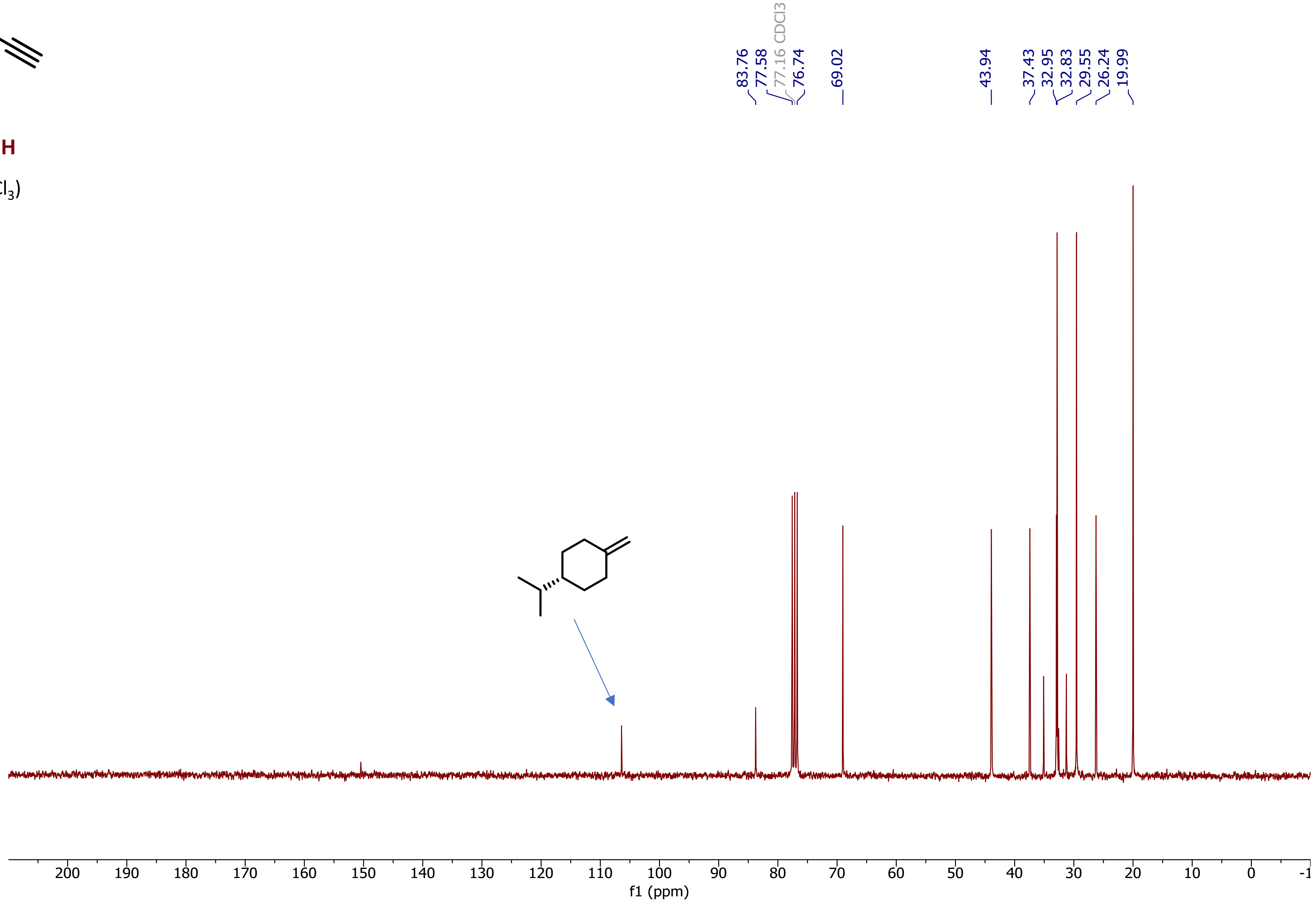

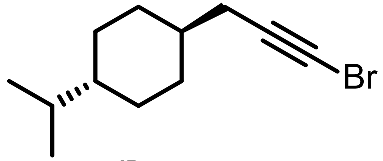

***iPr*trans-3e**

^{1}H NMR(300 MHz, $CDCl_3$)

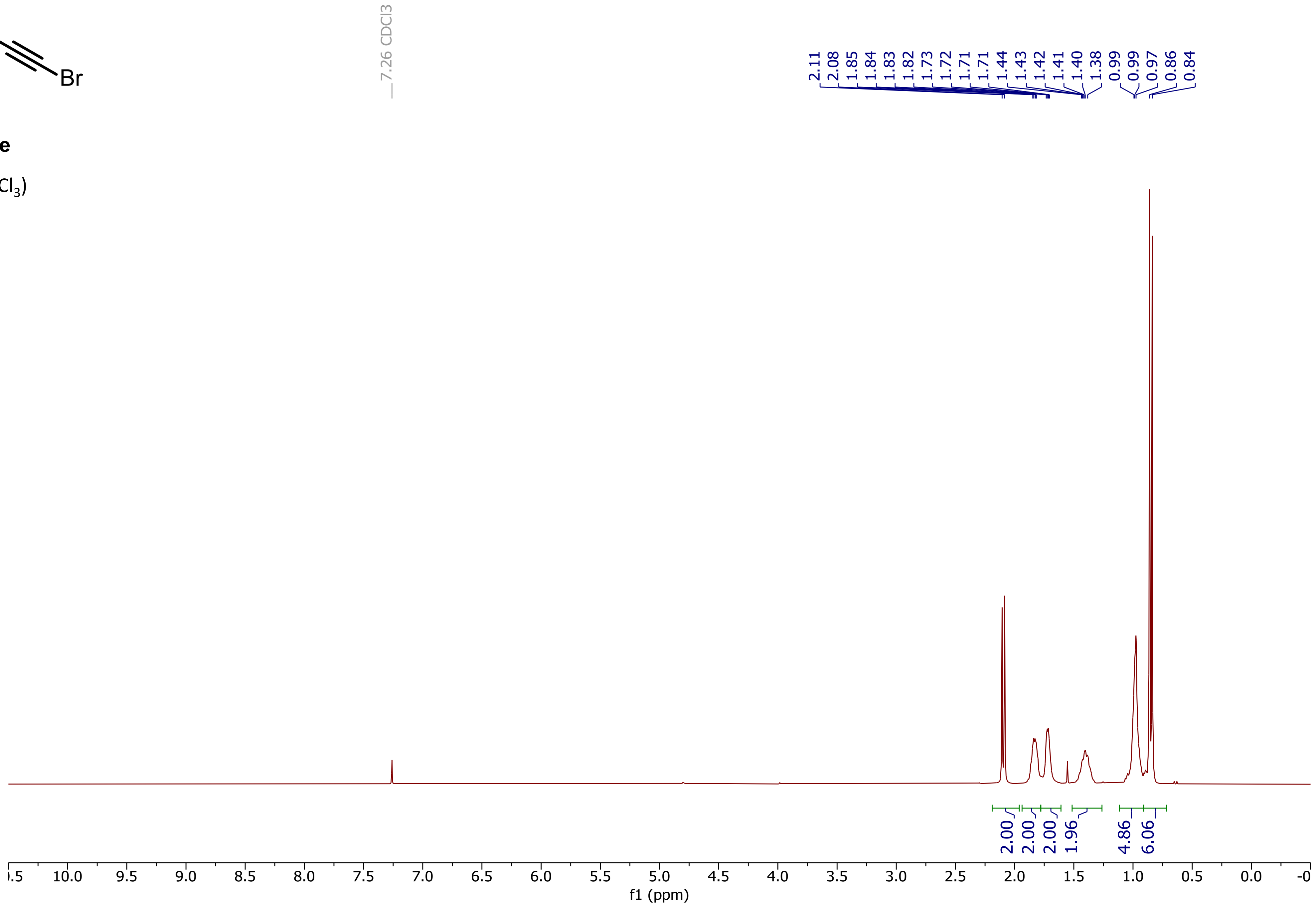

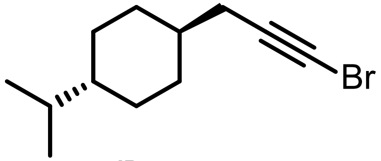

^{iPr}***trans*-3e**

^{13}C NMR (75 MHz, $CDCl_3$)

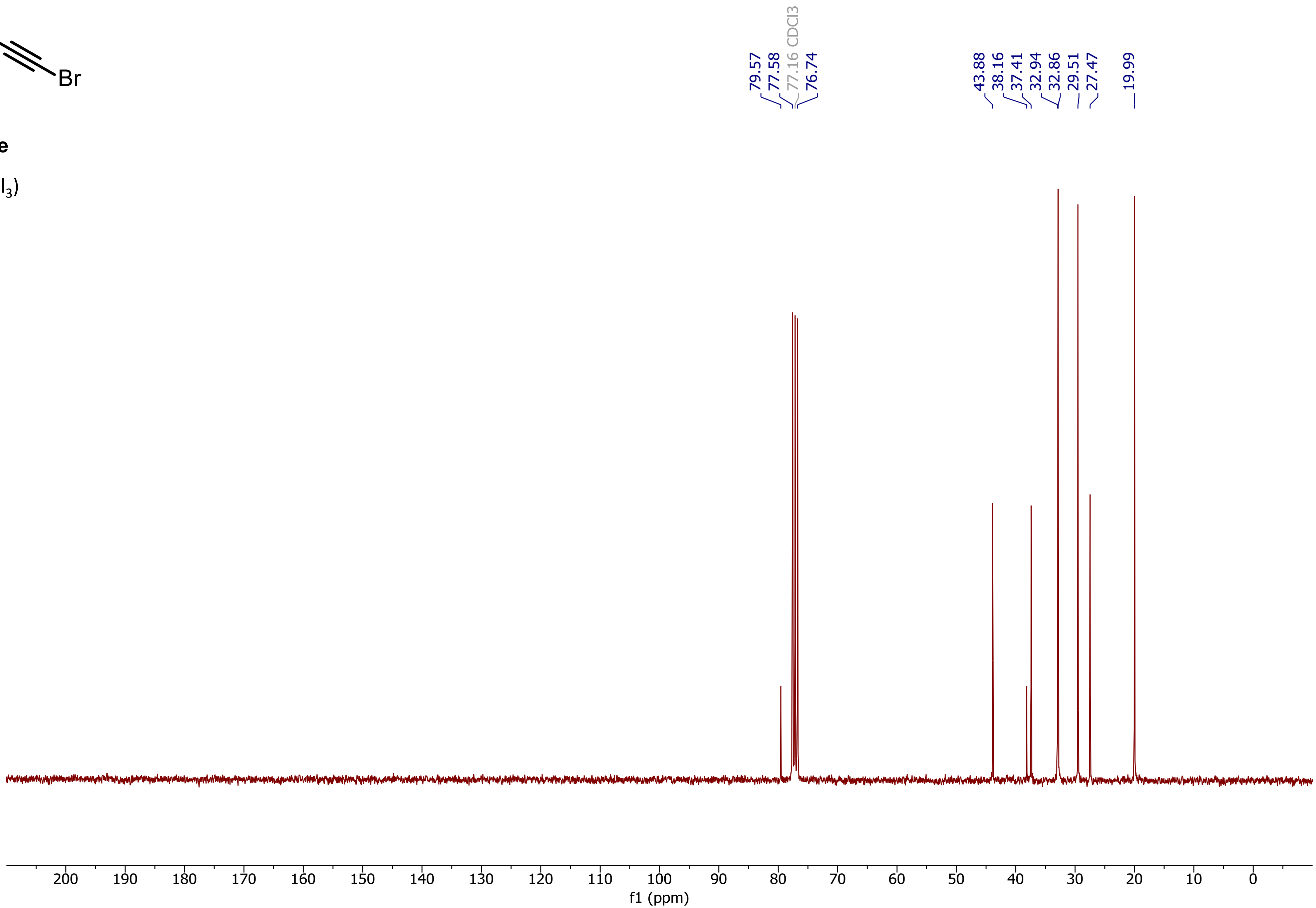

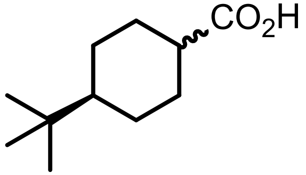

*tBu*3e-CO2Me

-crude-

1H NMR(300 MHz, CDCl3)

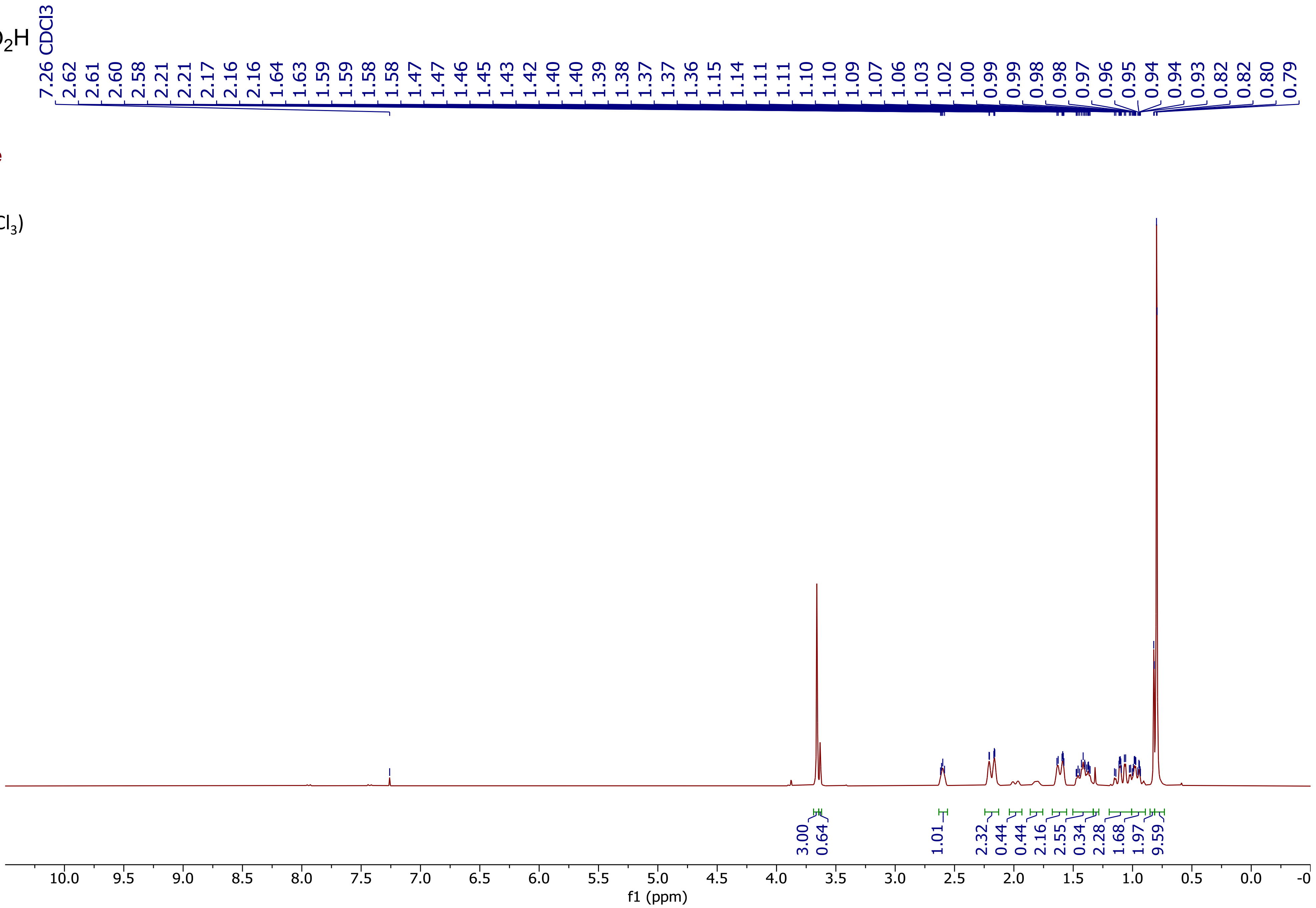

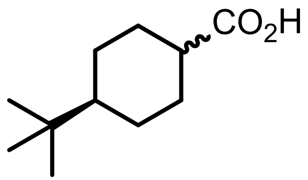

^{tBu}**3e-CO$_2$Me**

-crude-

^{1}H NMR(300 MHz, $CDCl_3$)

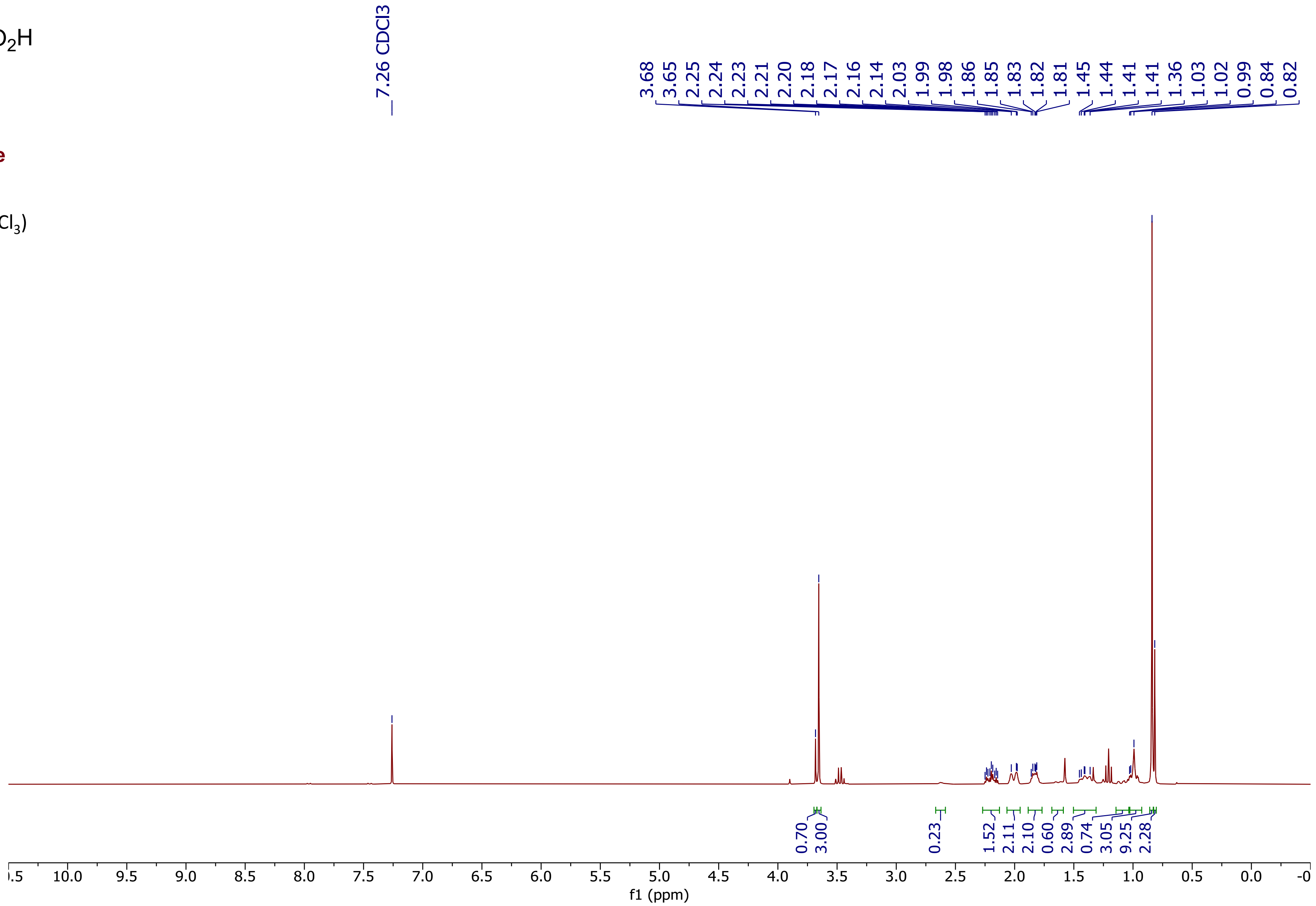

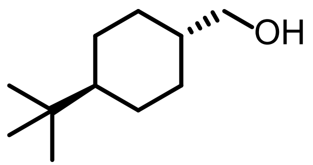

^{tBu}**trans-3e-OH**

^{1}H NMR(300 MHz, $CDCl_3$)

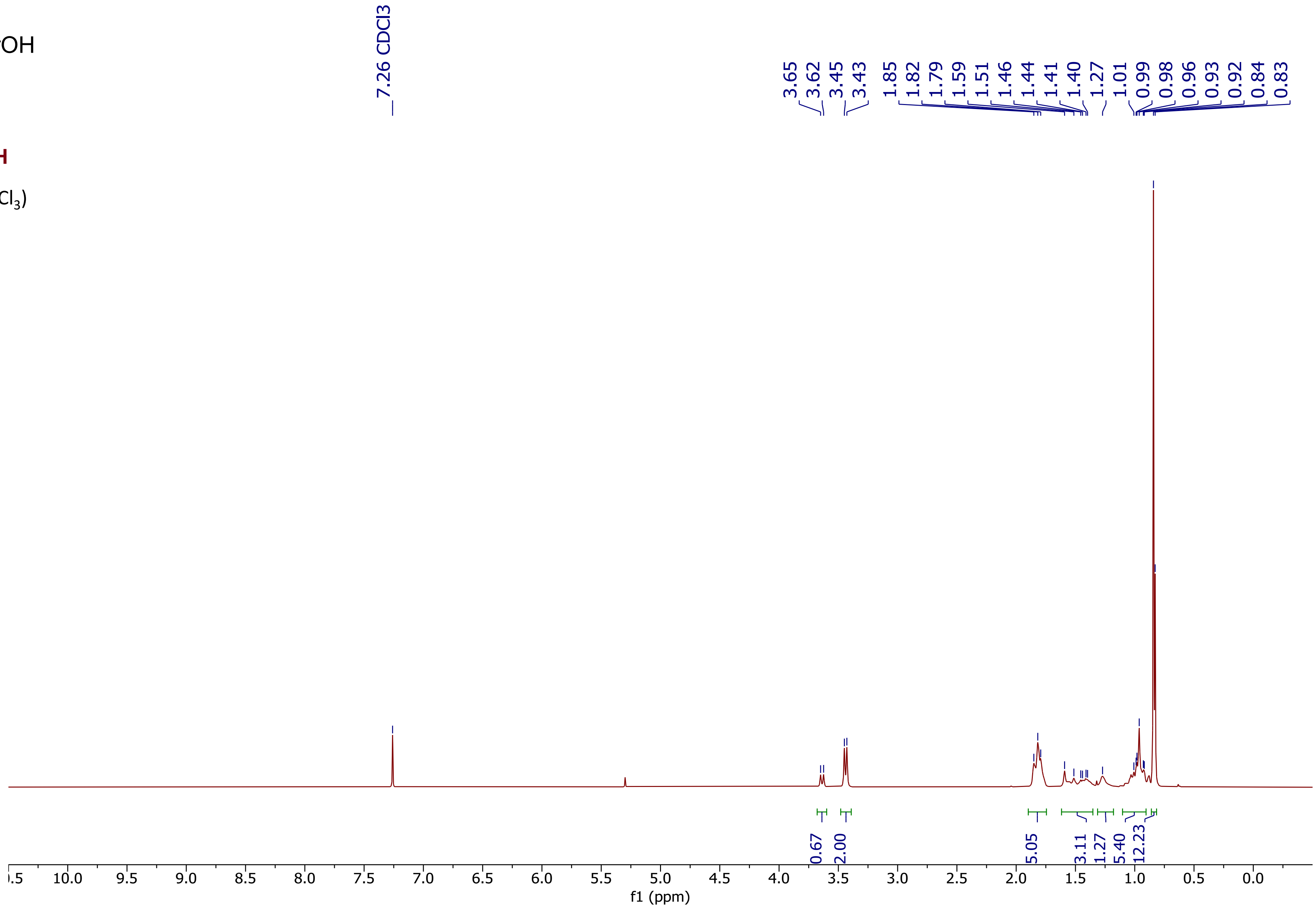

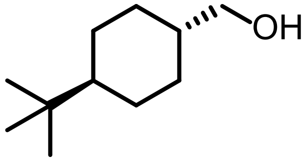

*tBu*trans-3e-OH

^{13}C NMR (75 MHz, $CDCl_3$)

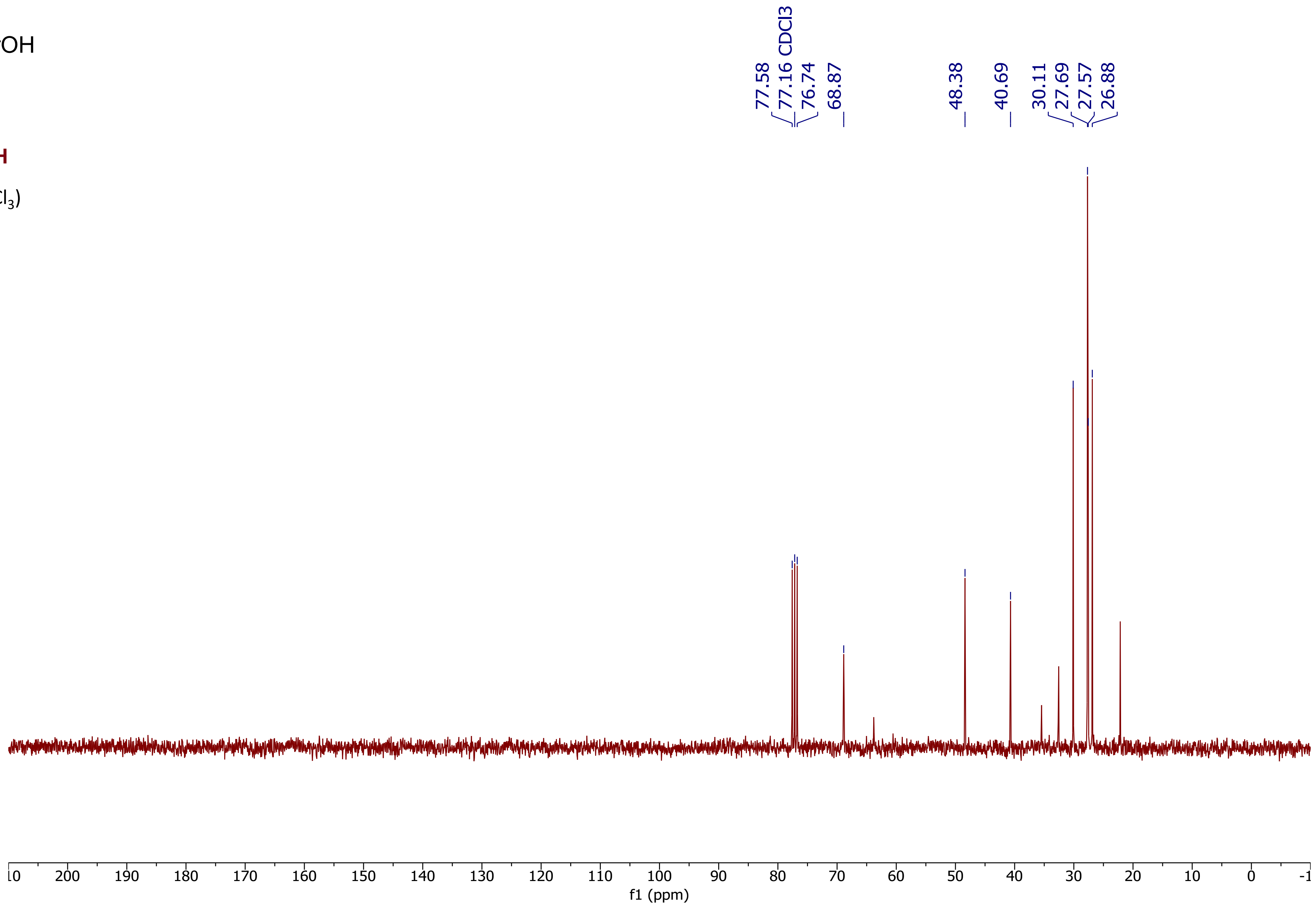

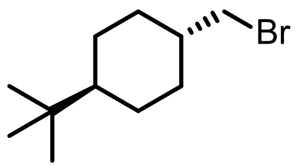

^{tBu}**trans-3e-Br**

^{1}H NMR(300 MHz, $CDCl_3$)

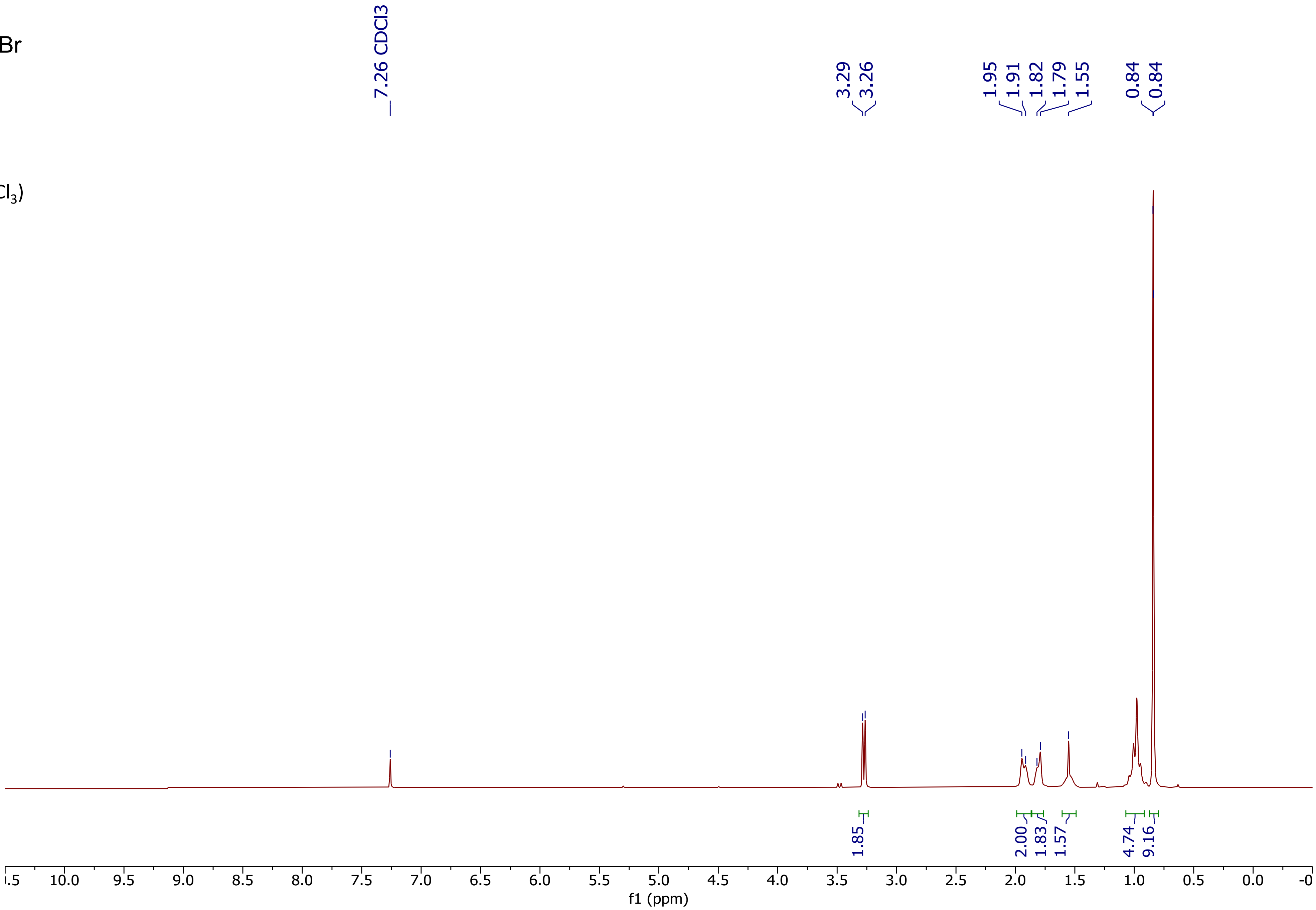

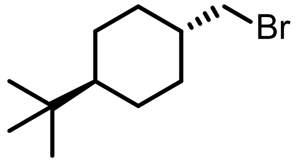

^{tBu}**trans-3e-Br**

^{13}C NMR (75 MHz, $CDCl_3$)

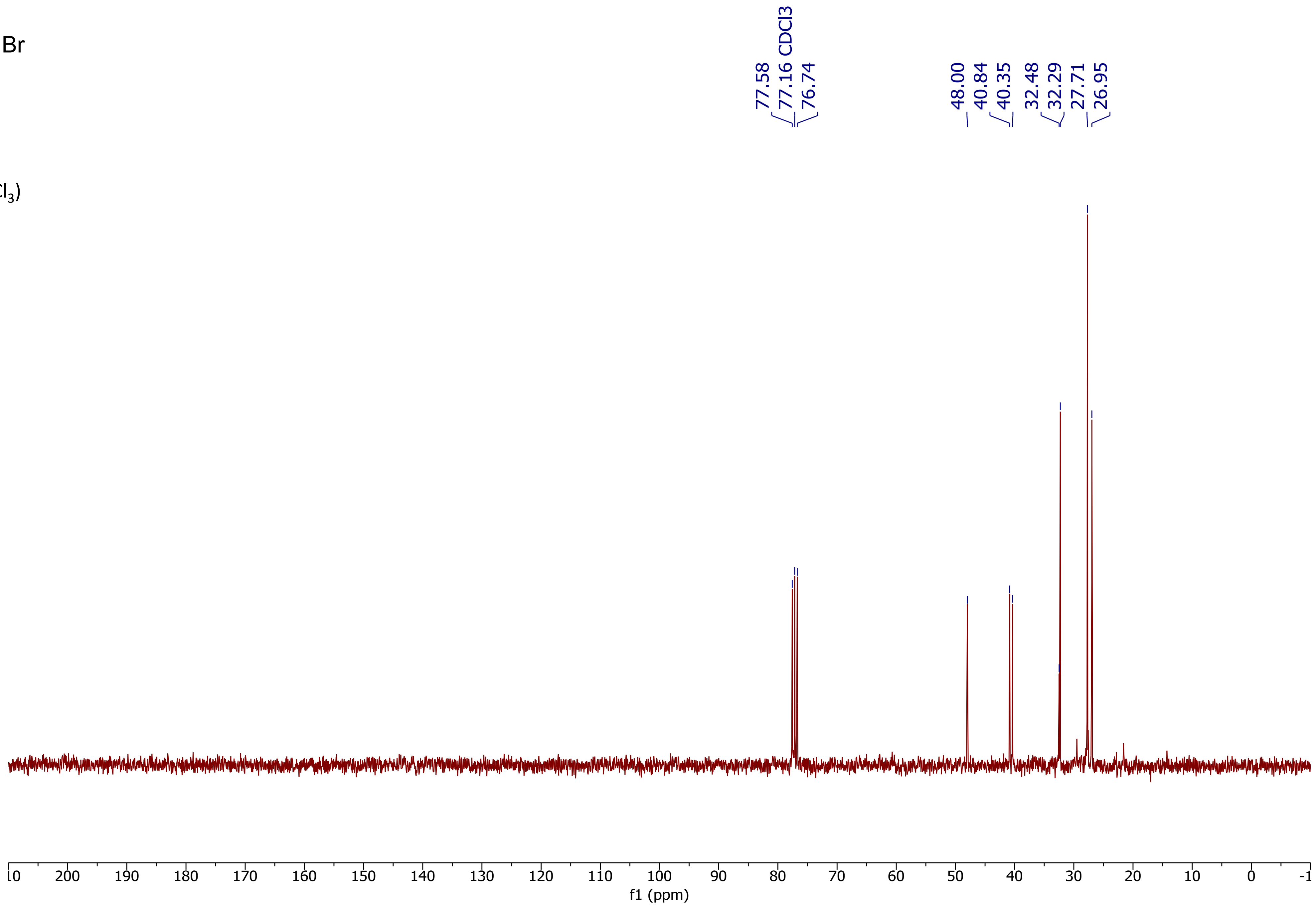

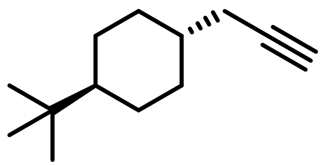

^{tBu}**trans-3e-CCH**

^{1}**H NMR**(300 MHz, $CDCl_3$)

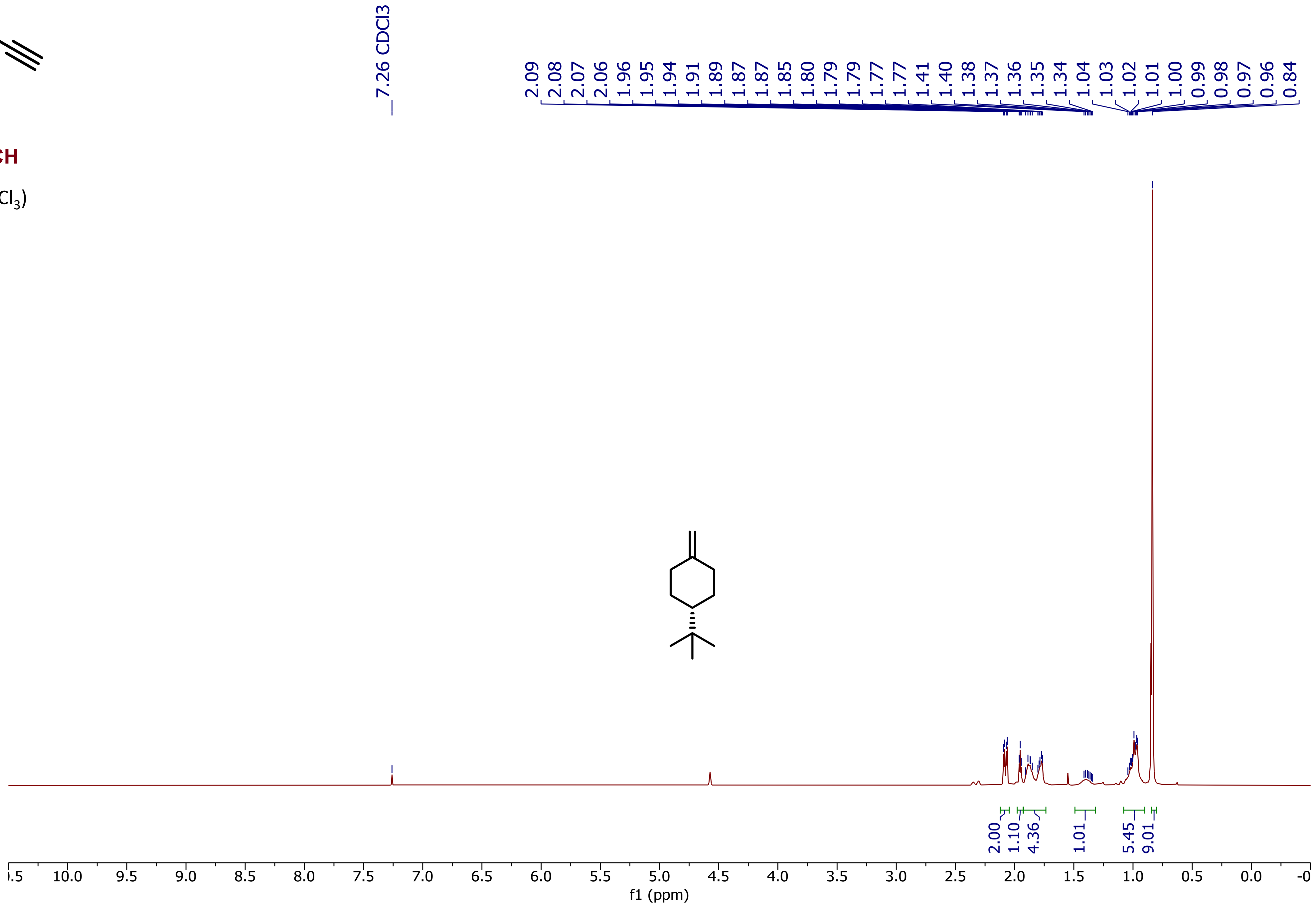

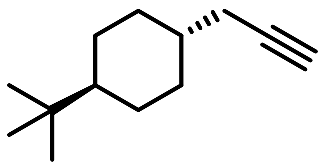

^{tBu}**trans-3e-CCH**

^{13}C NMR (75 MHz, $CDCl_3$)

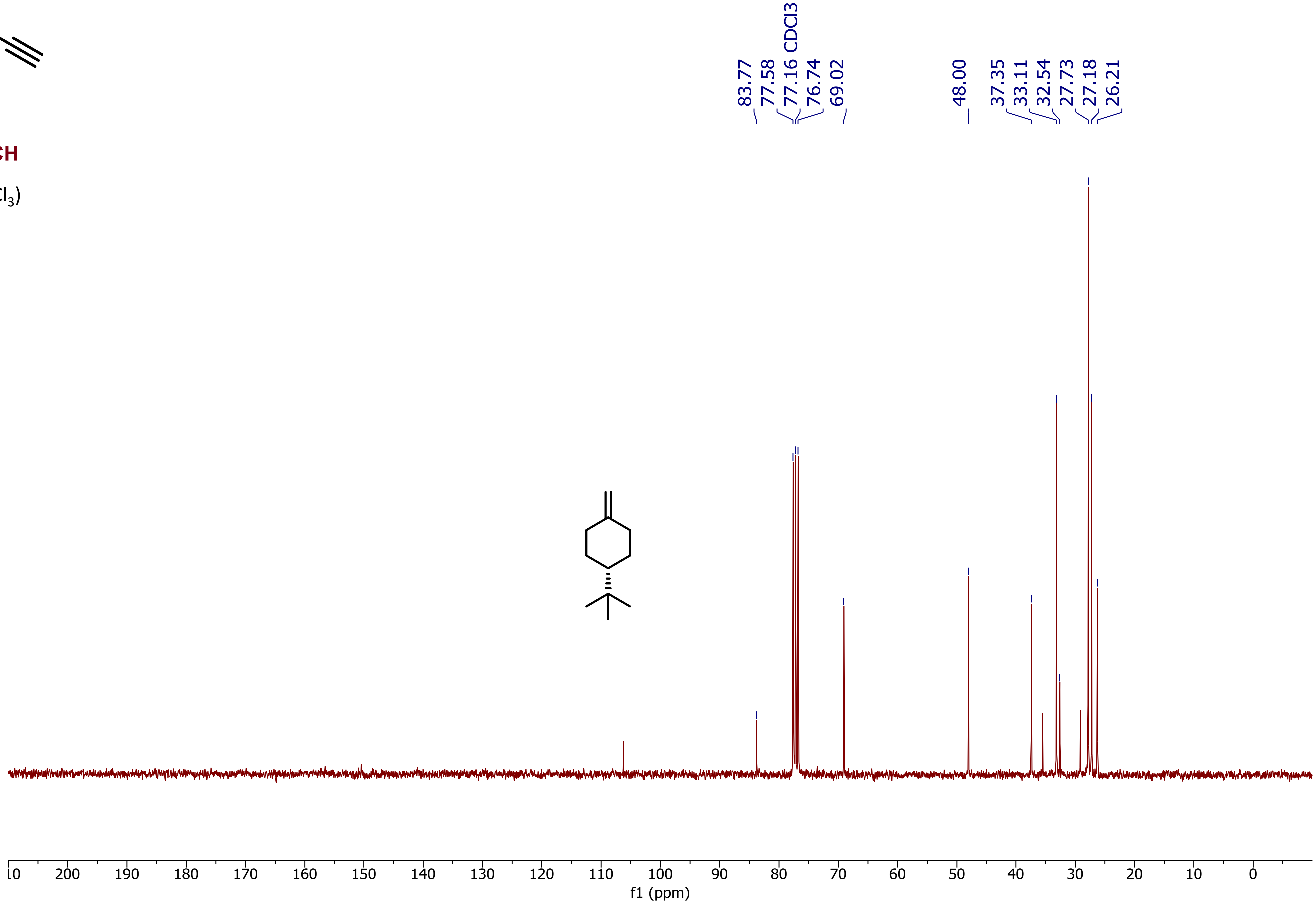

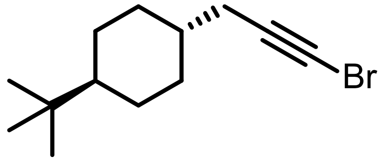

^{tBu}**trans-3e**

^{1}H NMR(300 MHz, $CDCl_3$)

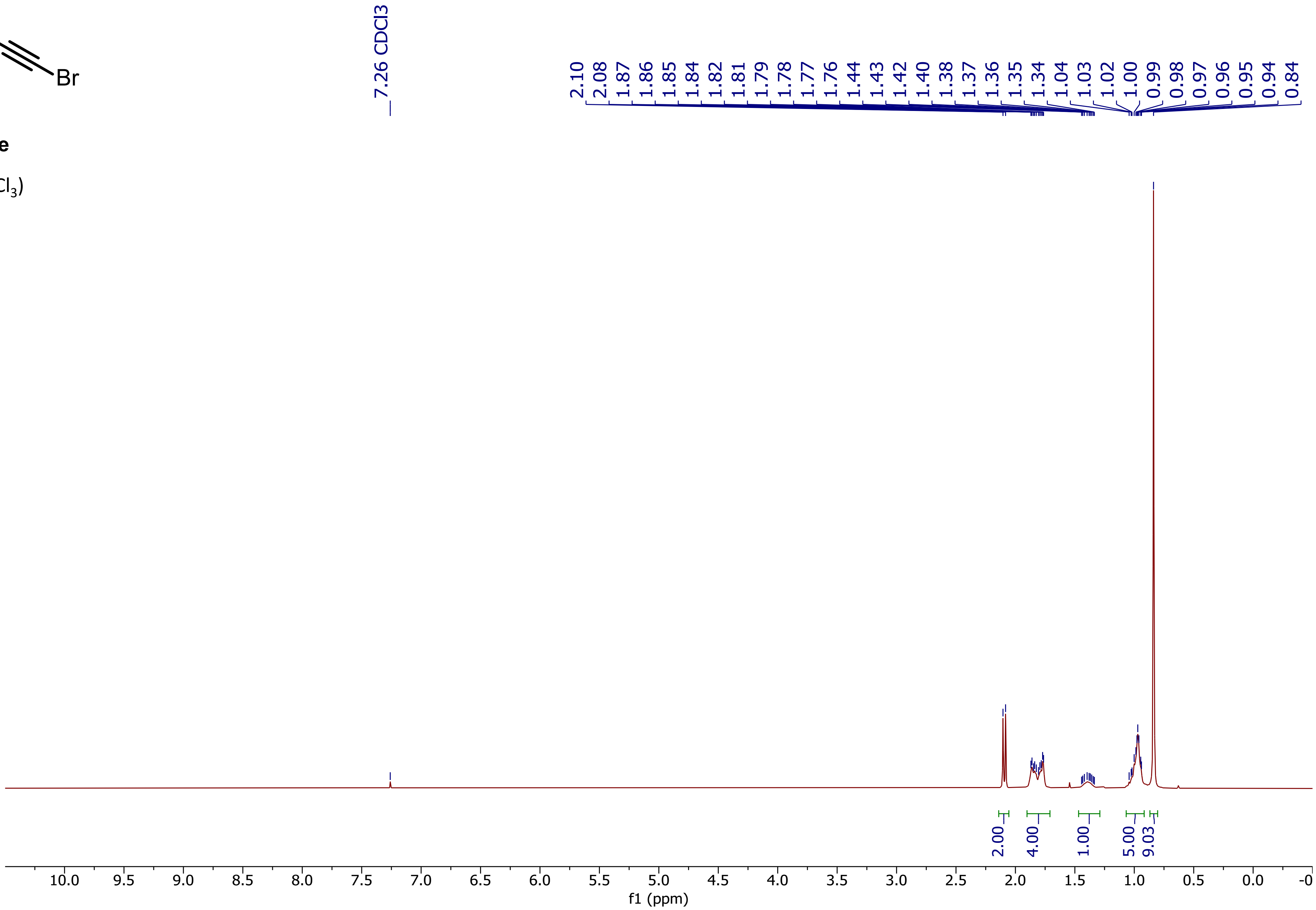

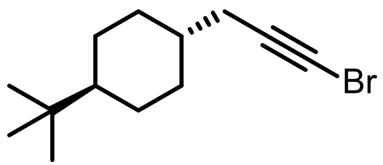

^{tBu}**trans-3e**

^{13}C NMR (75 MHz, $CDCl_3$)

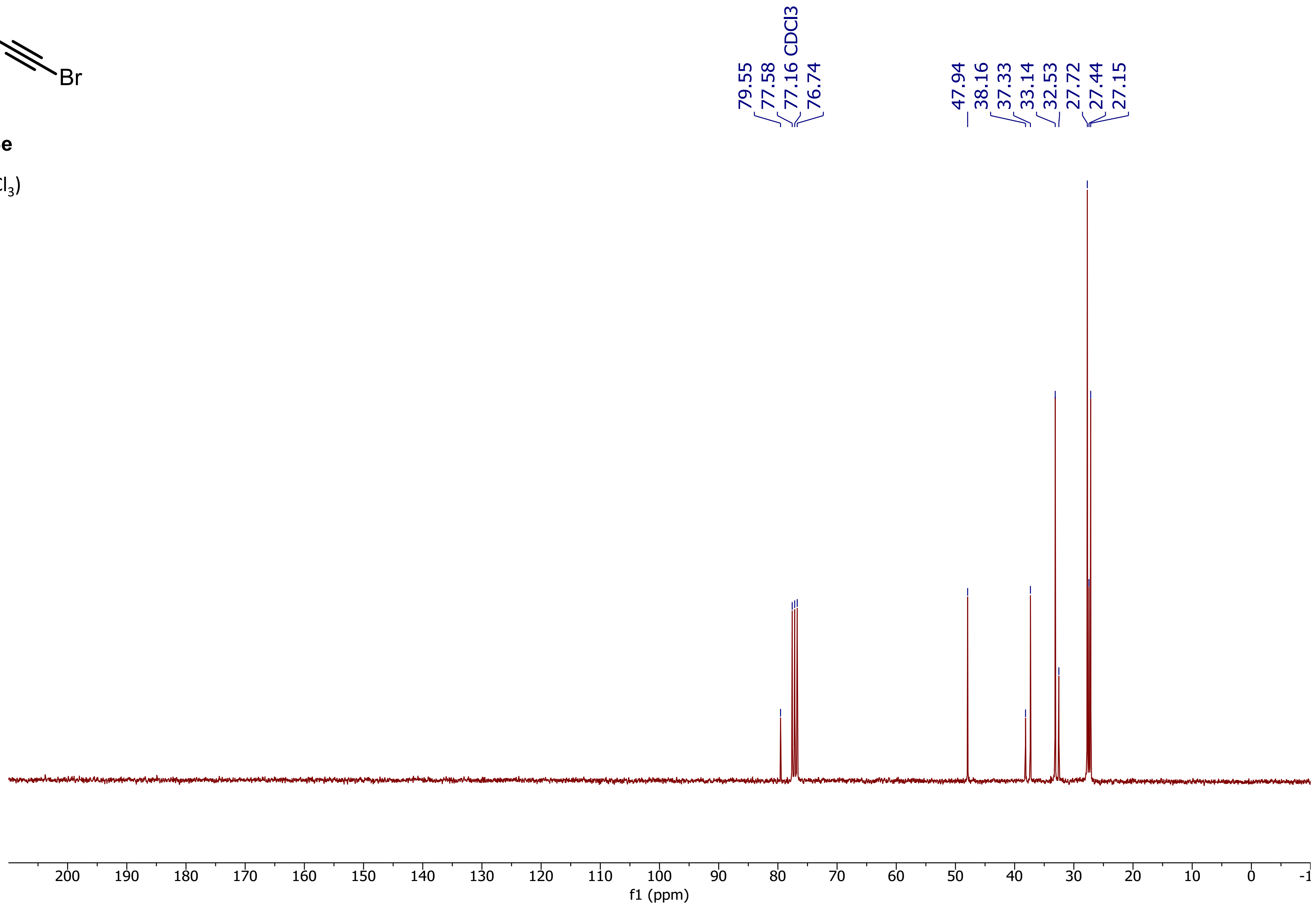

CHARACTERIZATION OF PRODUCTS

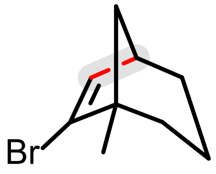

2b

^{1}H NMR(300 MHz, $CDCl_3$)

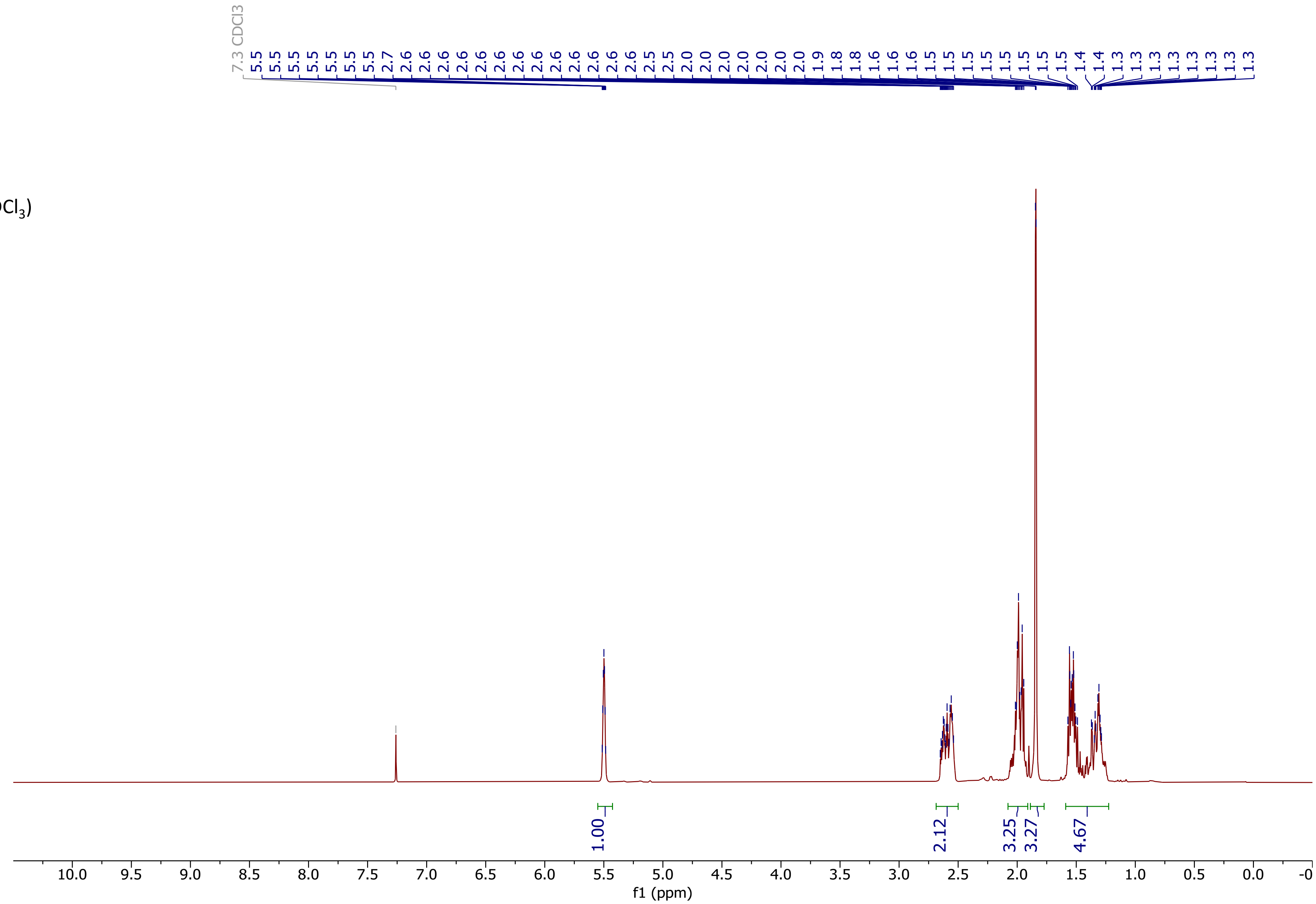

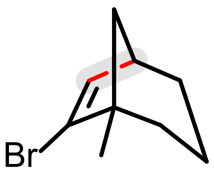

2b

^{13}C NMR (75 MHz, $CDCl_3$)

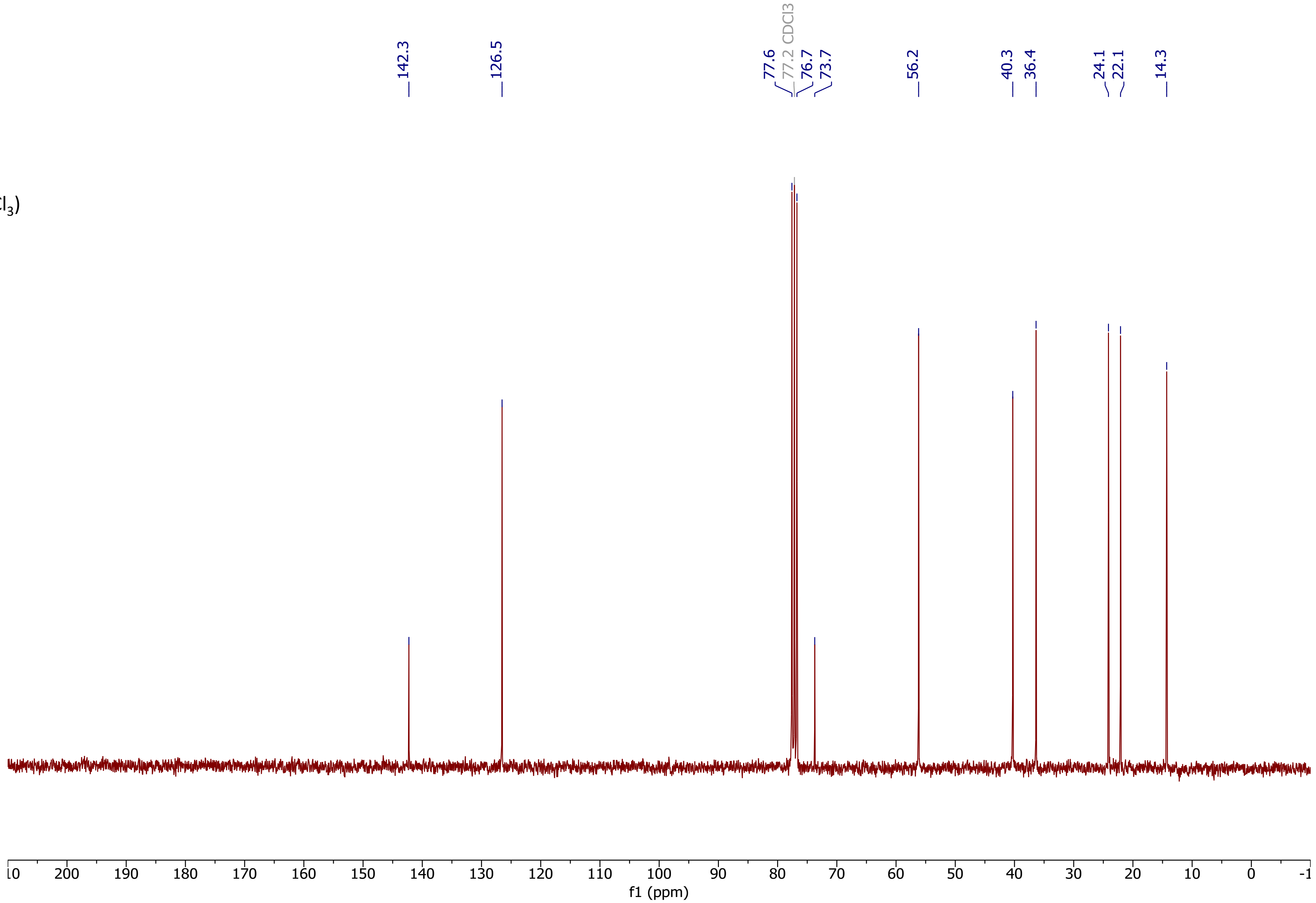

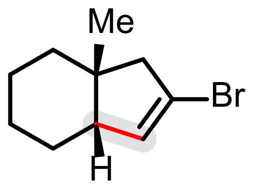

4b

^{1}H NMR(300 MHz, $CDCl_3$)

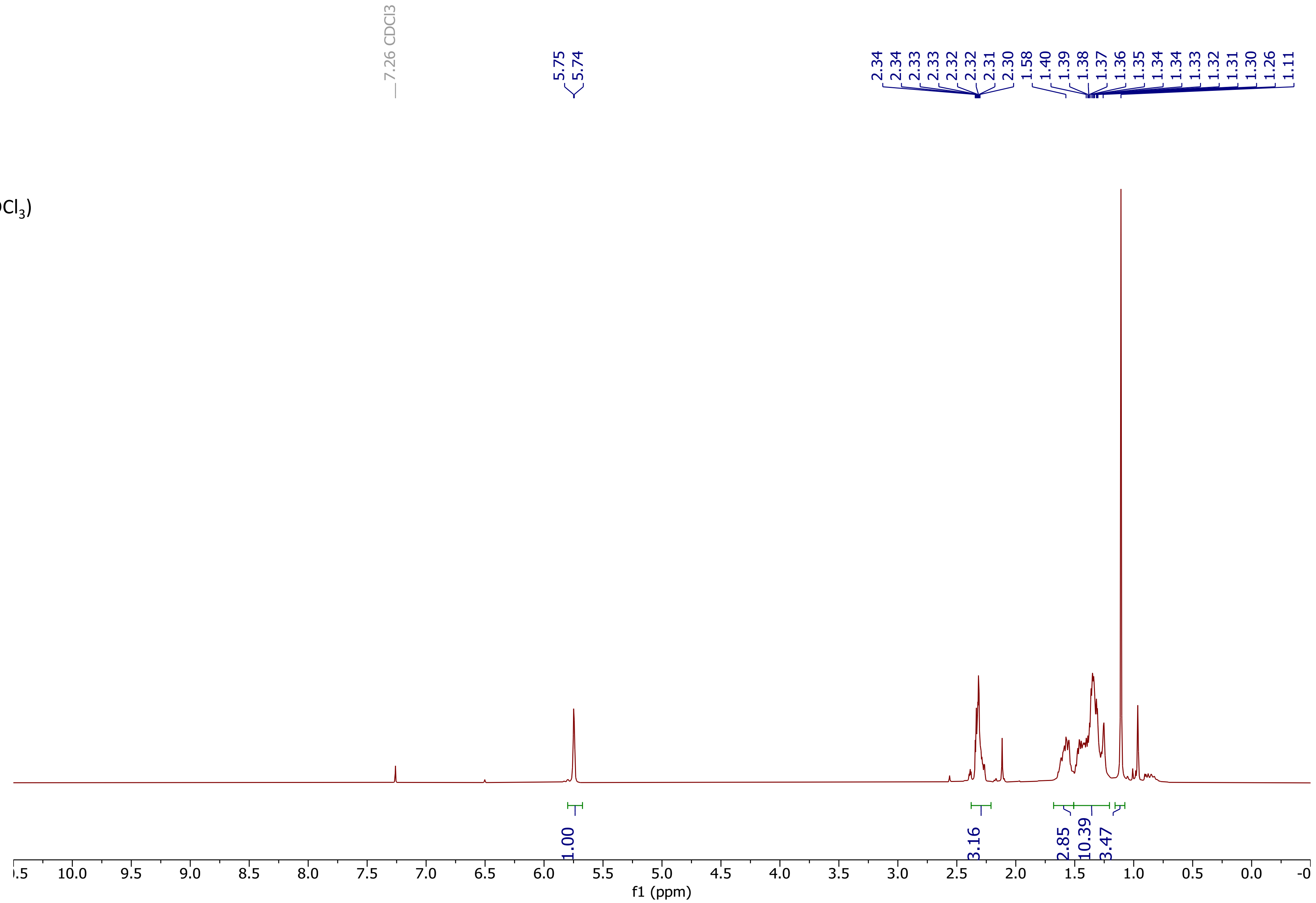

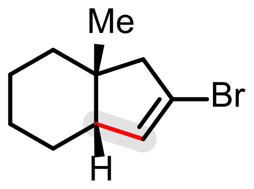

4b

^{13}C NMR (75 MHz, $CDCl_3$)

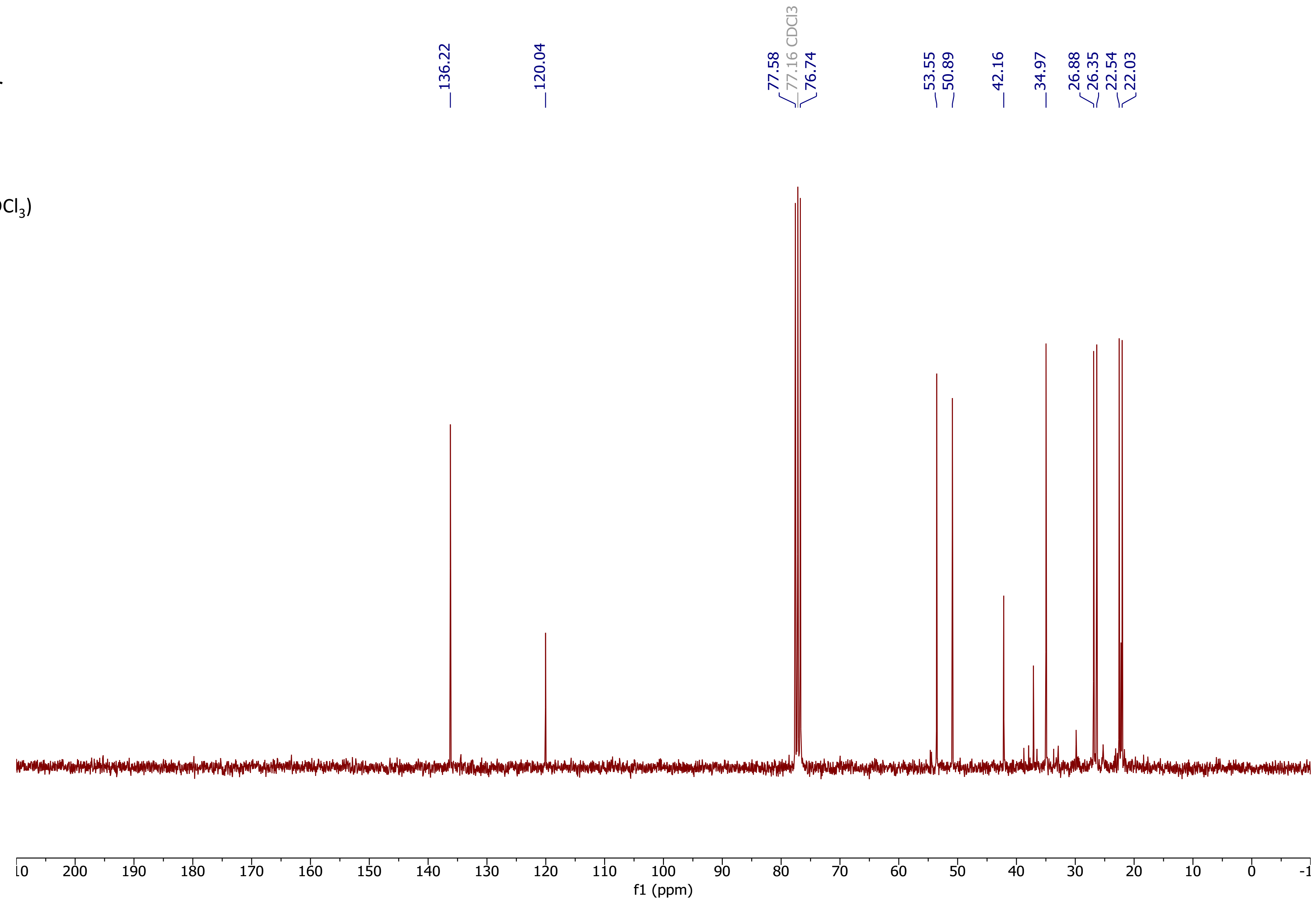

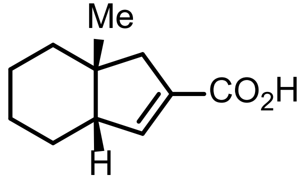

4b-CO_2H

^{1}H NMR(300 MHz, $CDCl_3$)

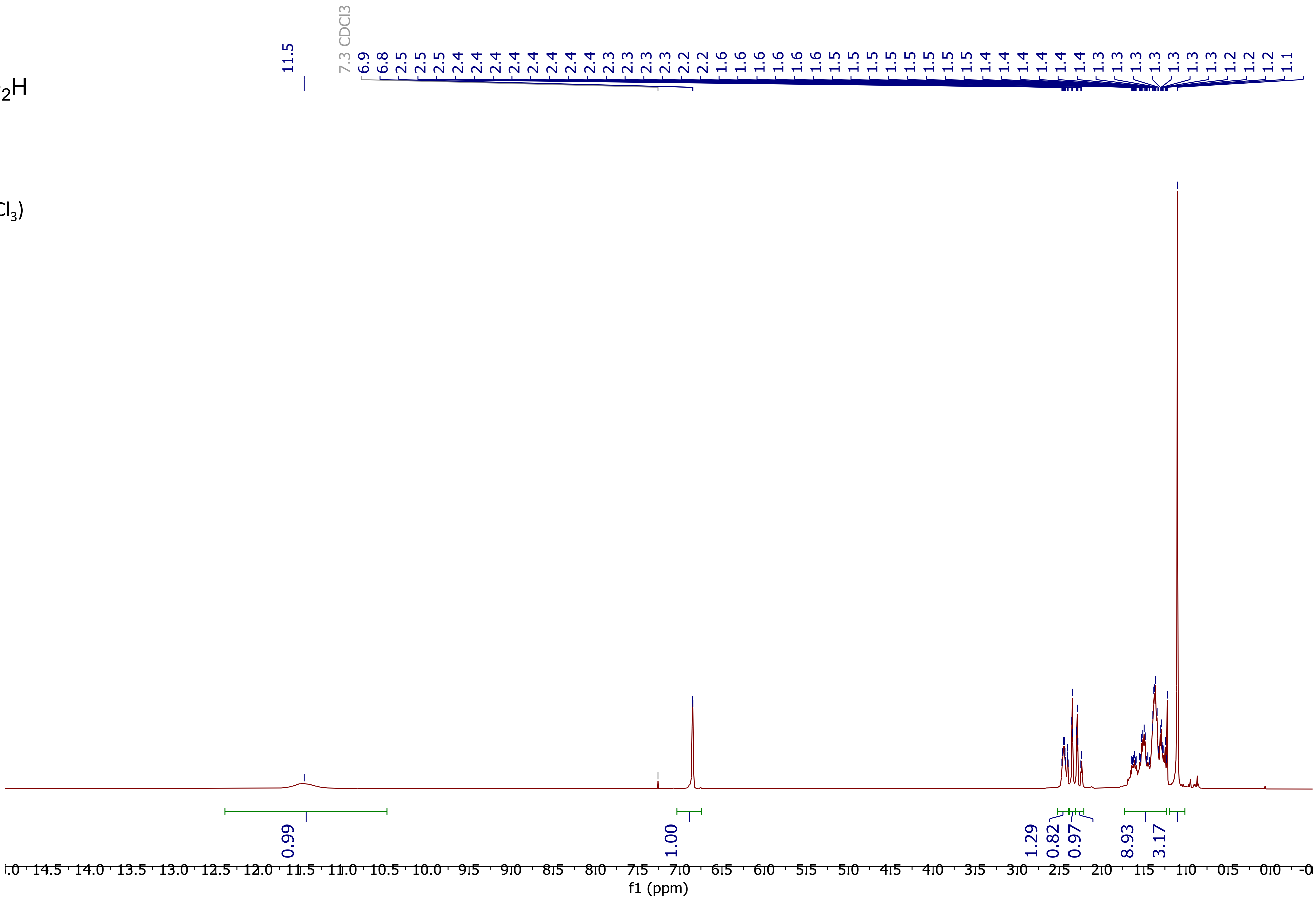

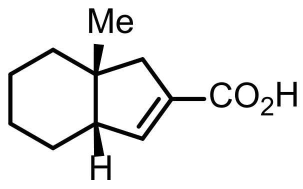

4b-CO_2H

^{13}C NMR (75 MHz, $CDCl_3$)

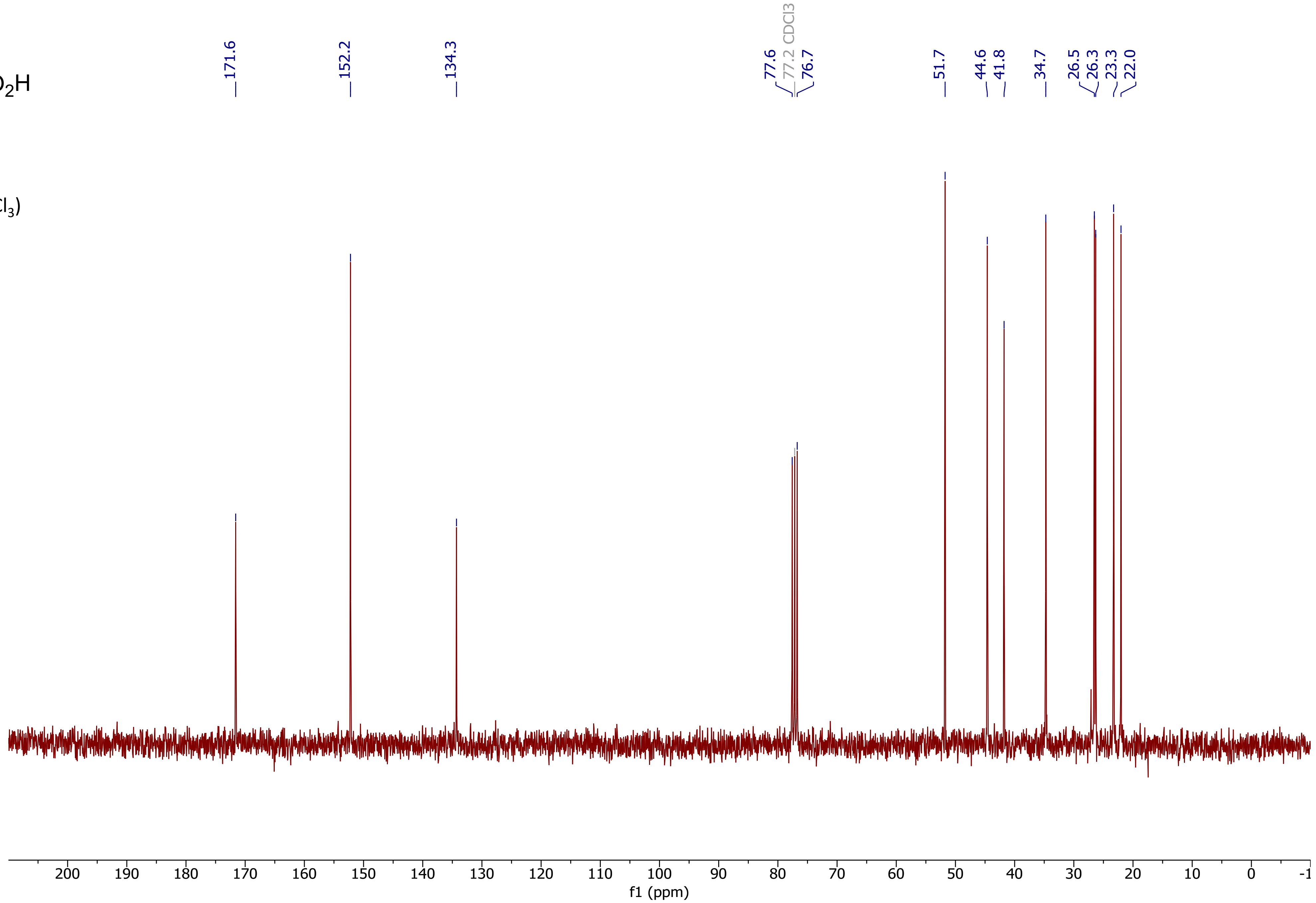

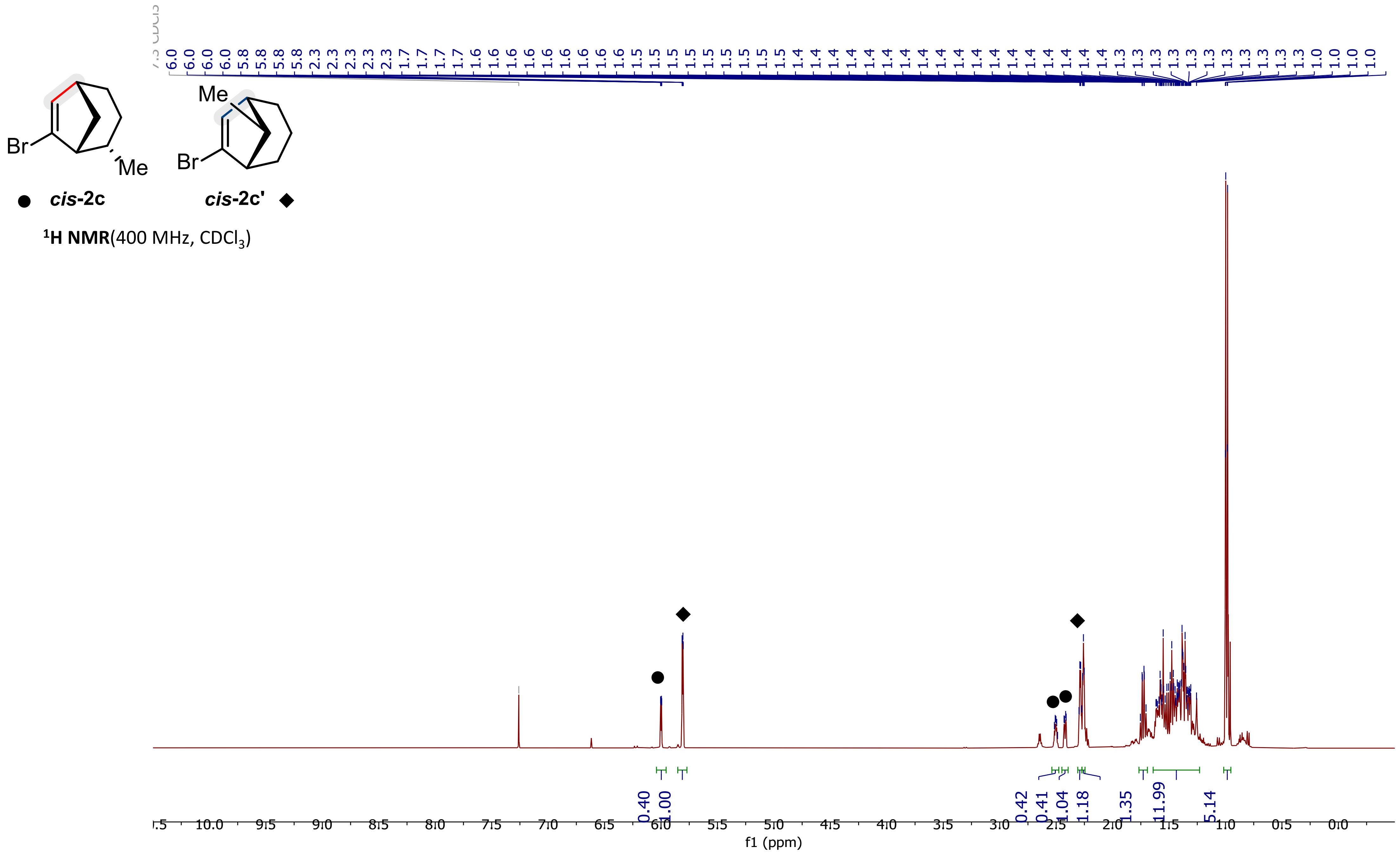

cis-2c
cis-2c'
¹H NMR(400 MHz, CDCl3)
f1 (ppm)

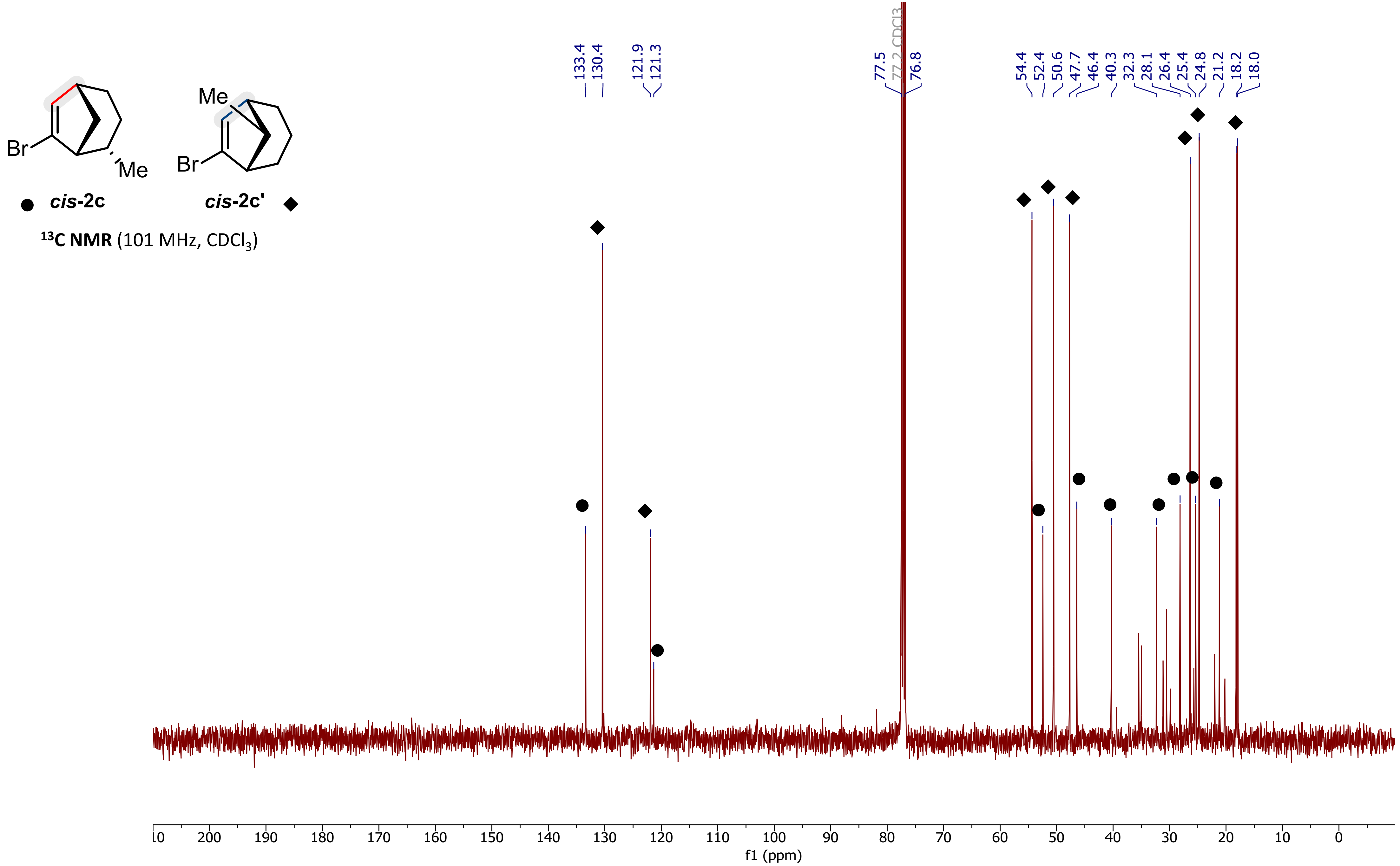
cis-2c
cis-2c'
13C NMR (101 MHz, CDCl3)
133.4
130.4
121.9
121.3
77.5
77.2 CDCl3
76.8
54.4
52.4
50.6
47.7
46.4
40.3
32.3
28.1
26.4
25.4
24.8
21.2
18.2
18.0
f1 (ppm)

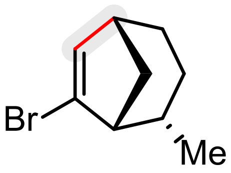

***cis*-2c**

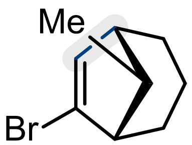

***cis*-2c'**

COSY NMR([400, 400] MHz, $CDCl_3$)

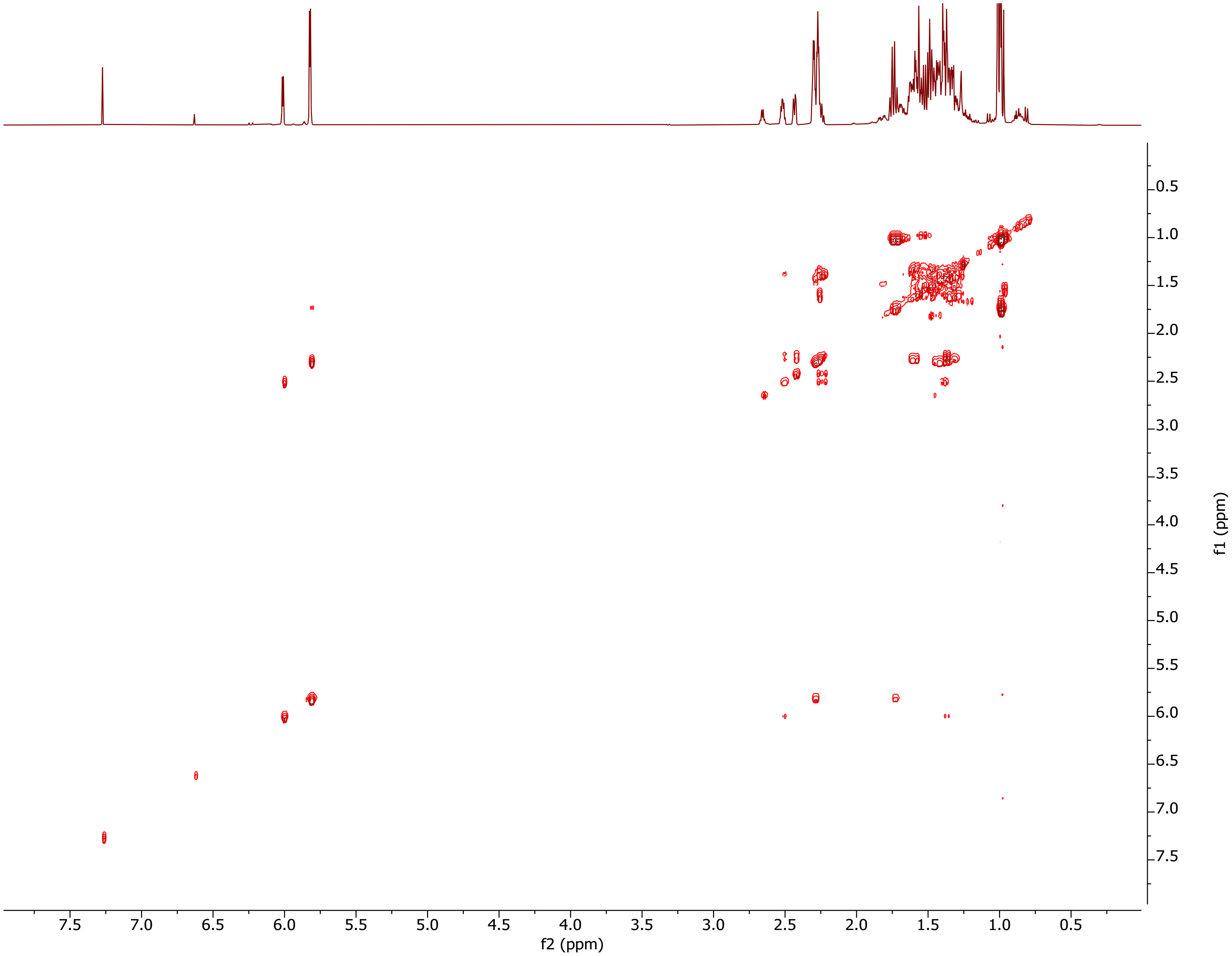

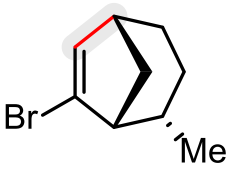

***cis*-2c**

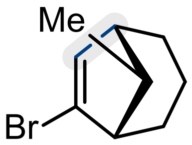

***cis*-2c'**

HSQC NMR([400, 101] MHz, $CDCl_3$)

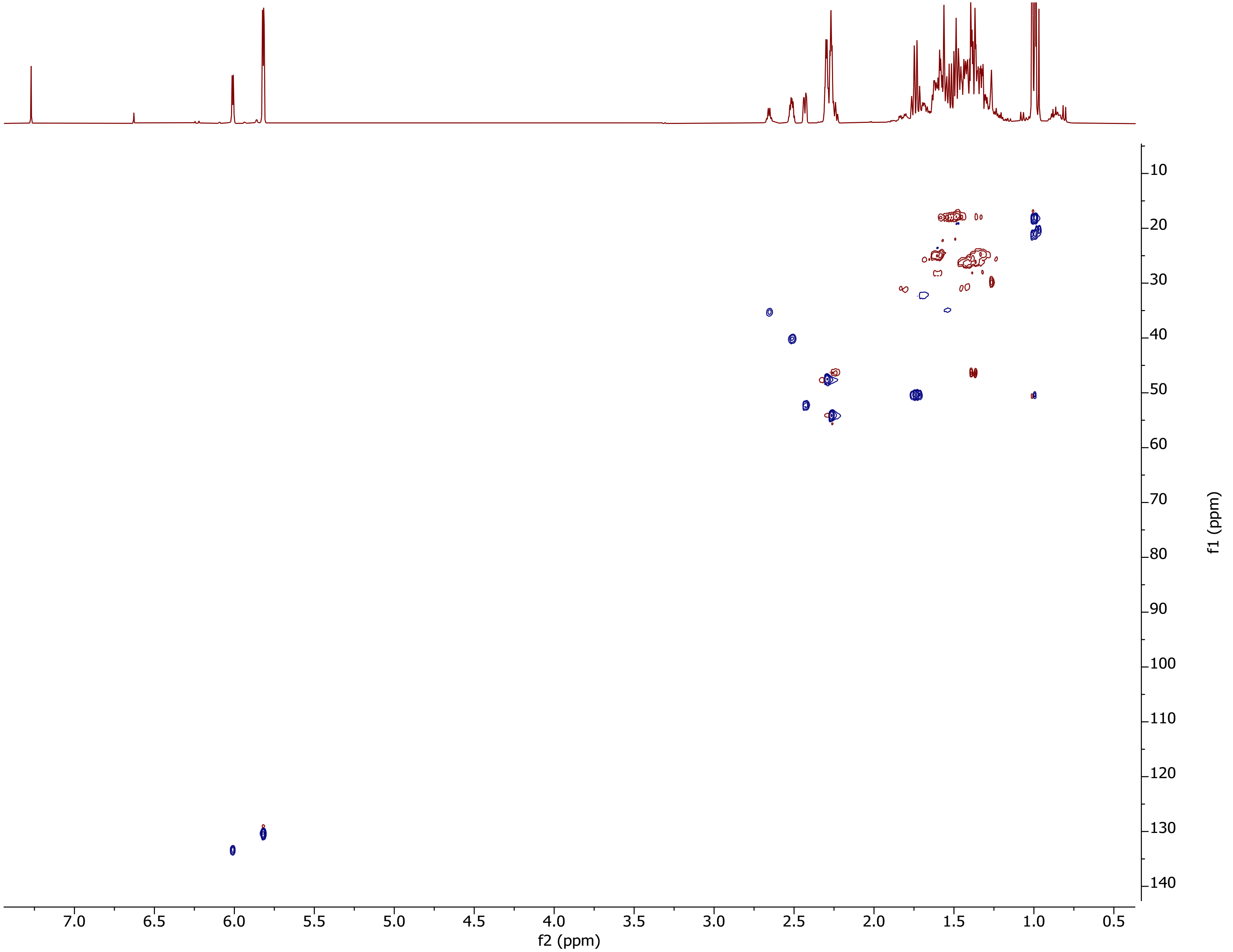

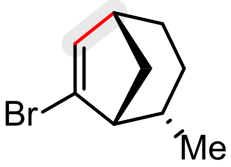

***cis*-2c**

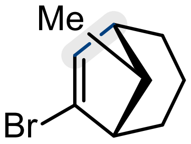

***cis*-2c'**

HMBC NMR([400, 101] MHz, $CDCl_3$)

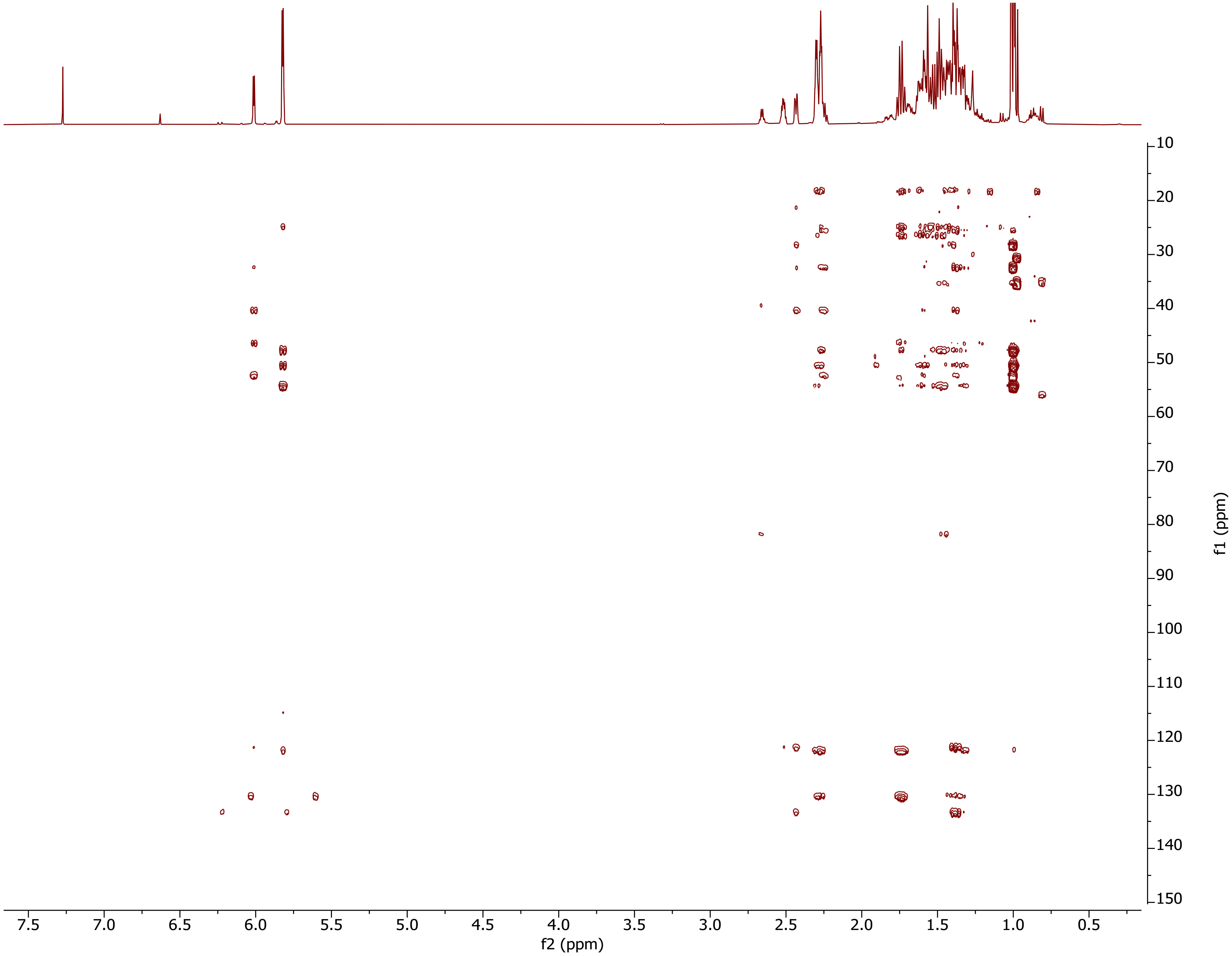

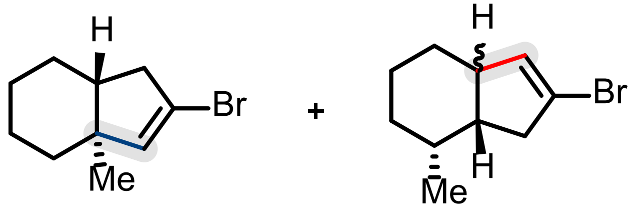

cis-4c **cis-4c'**

^{1}H NMR(400 MHz, $CDCl_3$)

● **cis-4c**

◆ **cis-4c'-cis**

▲ **cis-4c-trans**

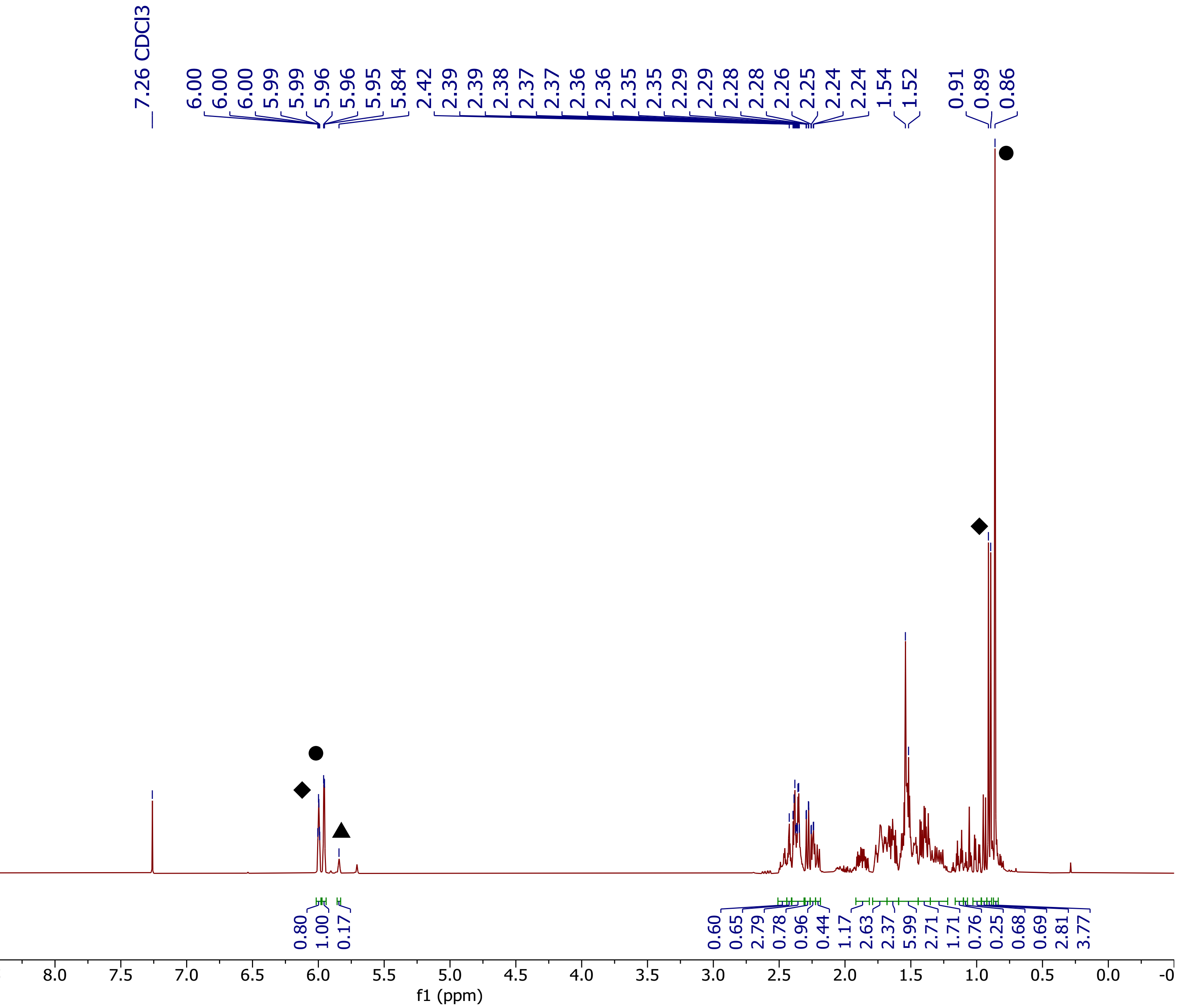

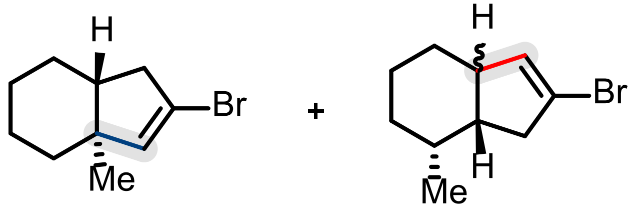

cis-4c **cis-4c'**

^{13}C NMR (101 MHz, $CDCl_3$)

● **cis-4c**

◆ **cis-4c'-cis**

▲ **cis-4c-trans**

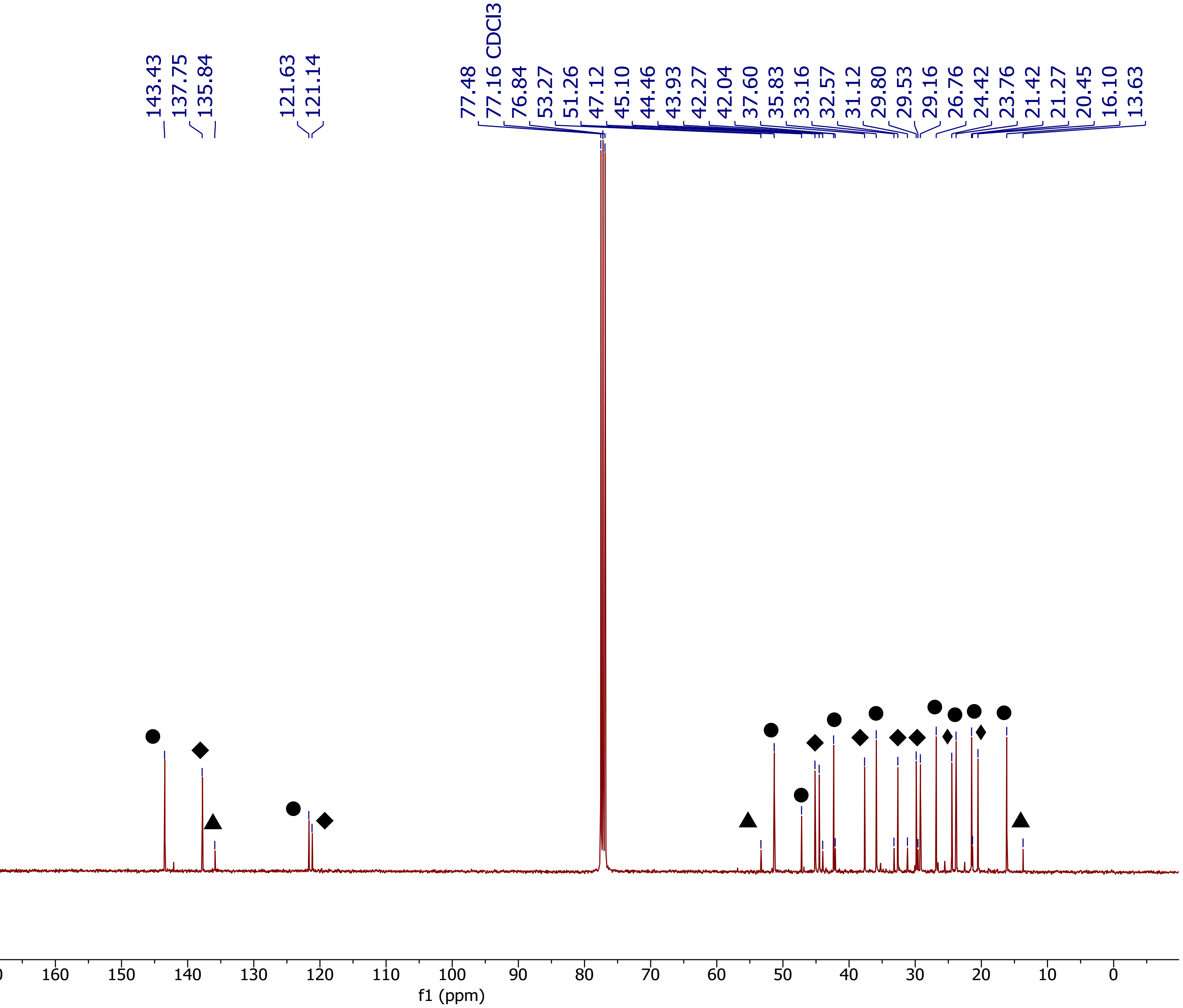

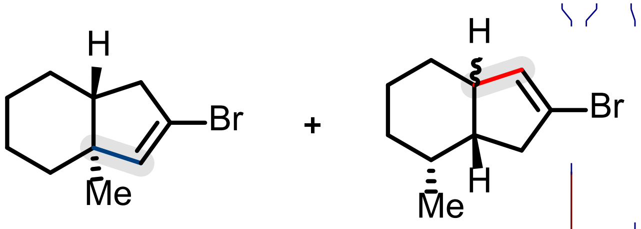

cis-4c **cis-4c'**

DEPT-135 NMR (101 MHz, $CDCl_3$)

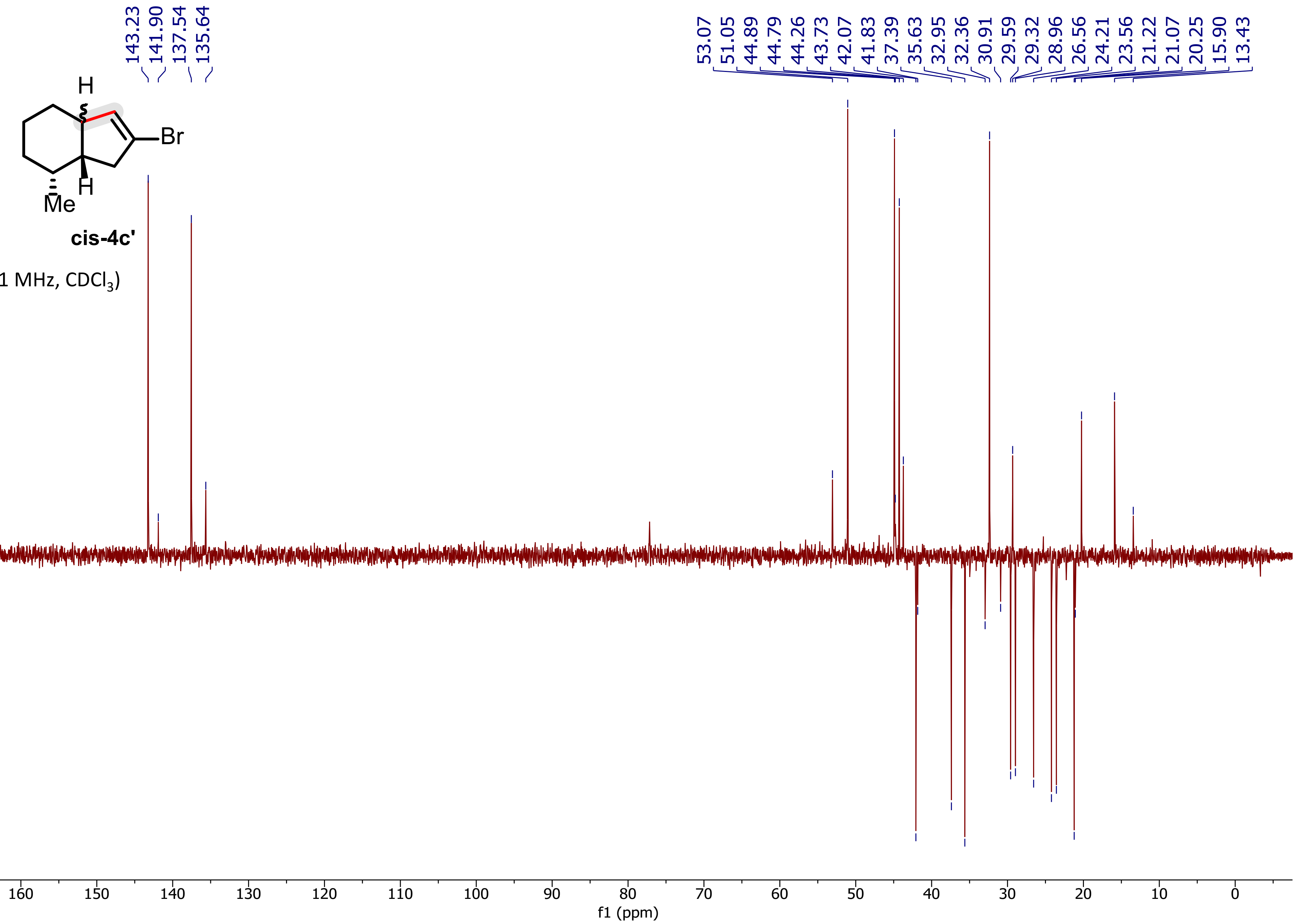

1H RMN AV600

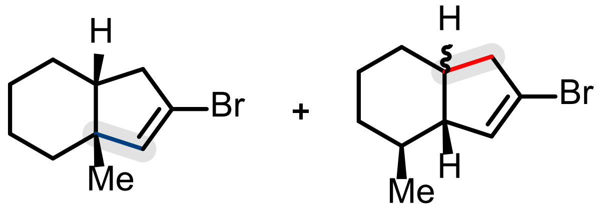

trans-4c **trans-4c'**

^{1}**H NMR**(600 MHz, $CDCl_3$)

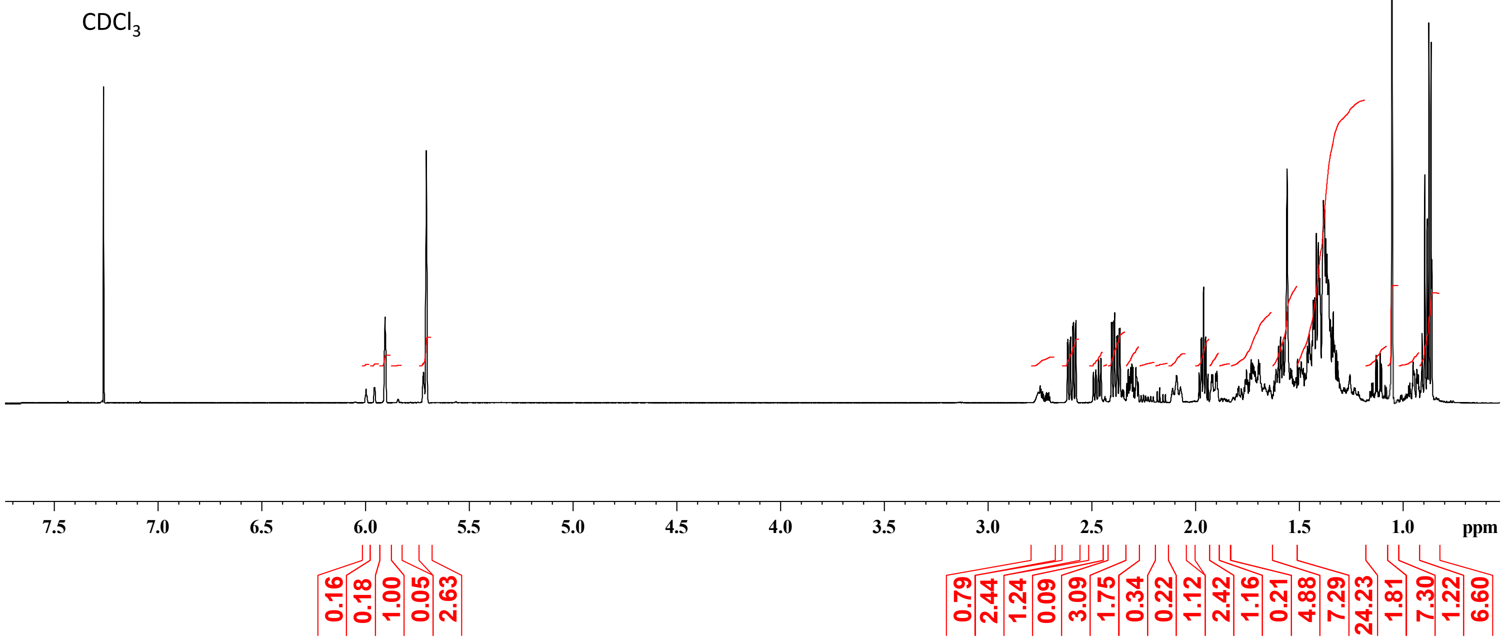


```
NAME            RM-1719F1
EXPNO                   4
PROCNO                  1
Date_             2024062
Time                    0
INSTRUM             12.59
PROBHD              spect
PULPROG    5 mm PATXI
TD                   1H/
SOLVENT             zg30
NS                  32768
DS                  CDCl3
SWH                   1 6
FIDRES                  0
AQ             6510.417 Hz
RG             0.198682 Hz
DW            2.5167091
DE                    sec
TE                    114
D1              76.800 usec
TD0                 6.00
                    usec
======== CHANNEL f298.2 K====
NUC1          1.00000000 1H
P1                 8.60 sec usec
PL1                2.00 1 dB
PL1W        15.84893227 W
SFO1        600.1529943
SI                    MHz
SF                  32768
WDW         600.1500154 MHz
SSB                    GM
LB                      0
GB                -0.30 Hz
PC                    0.3
                     1.00
```

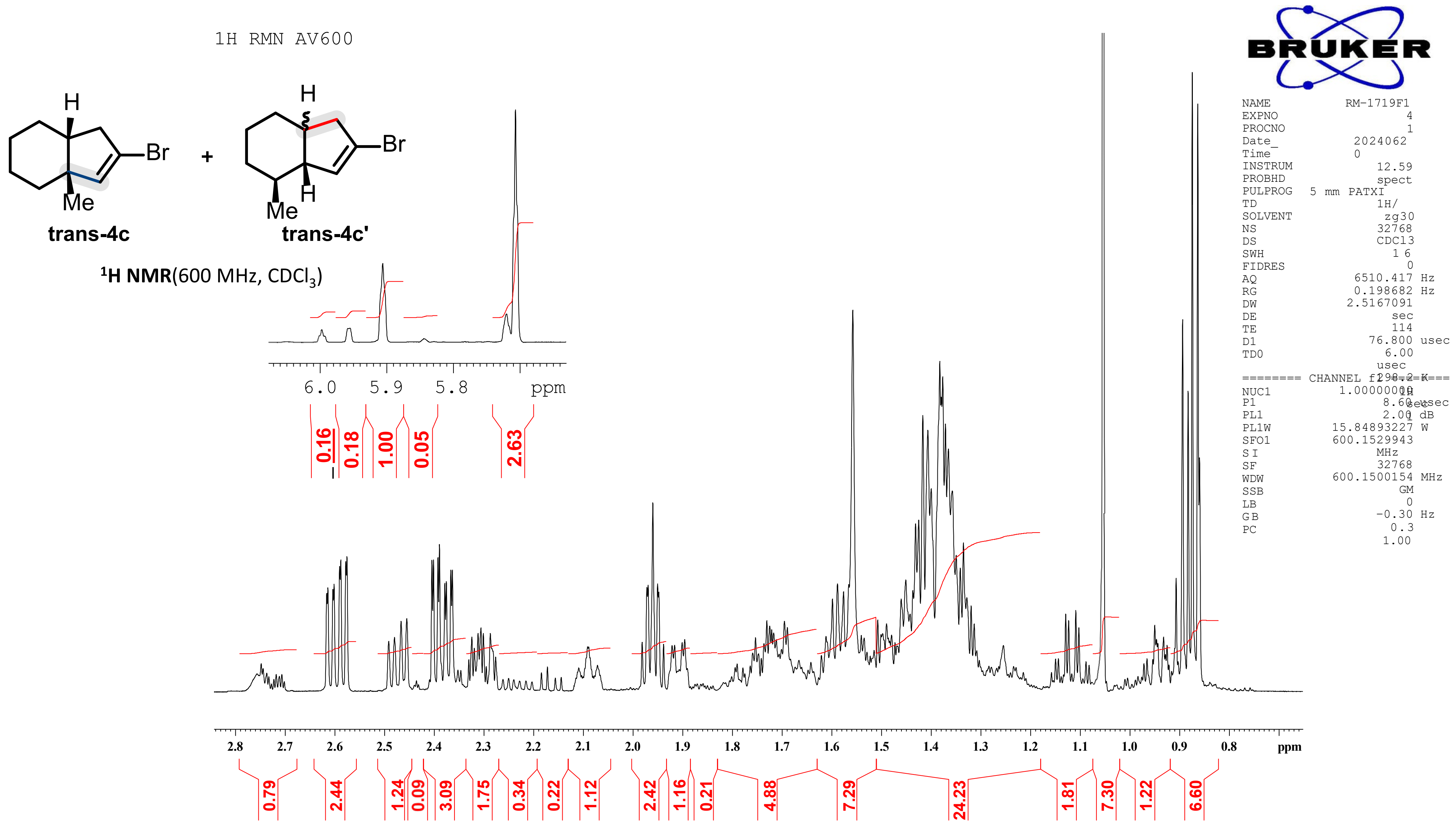
1H RMN AV600
H
Br
+
Me
trans-4c
trans-4c'
^{1}H NMR(600 MHz, $CDCl_3$)
BRUKER
NAME RM-1719F1
EXPNO 4
PROCNO 1
ppm

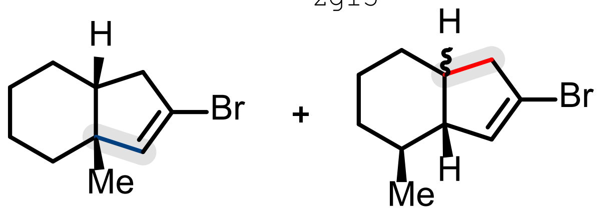

trans-4c trans-4c'

^{1}H NMR(600 MHz, $CDCl_3$)

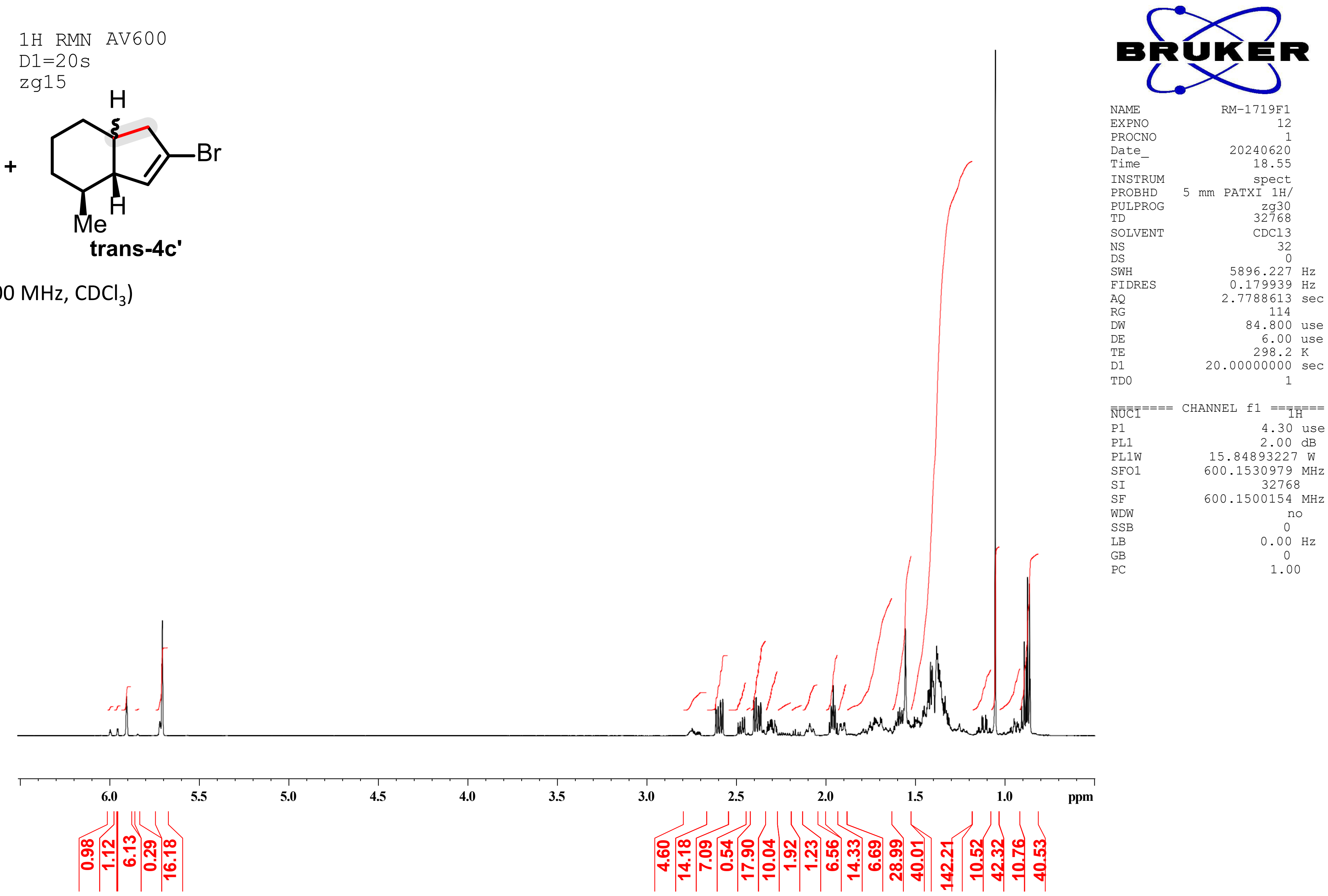

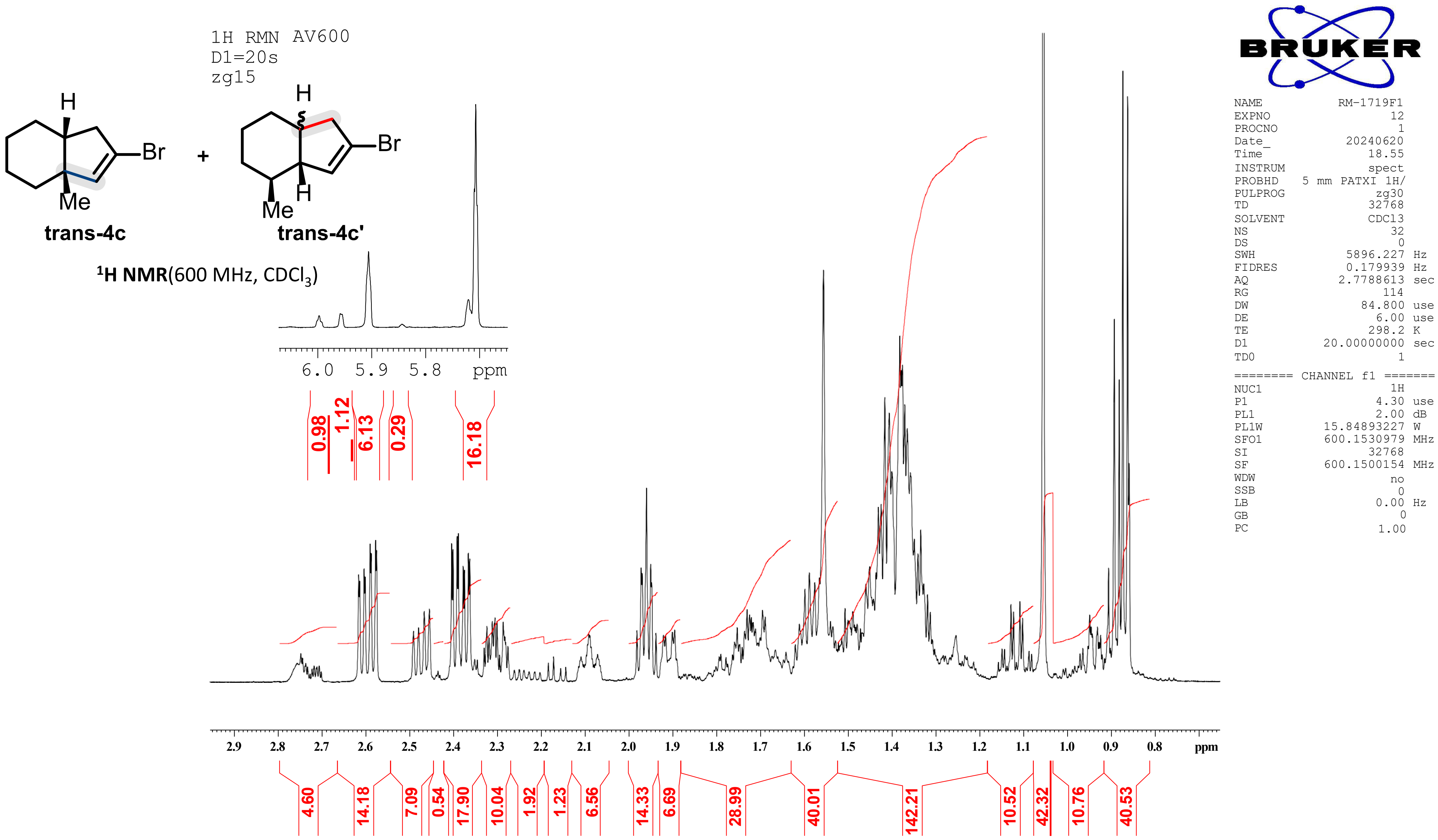
1H RMN AV600
D1=20s
zg15
H
Br
Me
+
H
Br
H
Me
trans-4c
trans-4c'
1H NMR(600 MHz, CDCl3)
6.0 5.9 5.8 ppm
0.98 1.12 6.13 0.29 16.18
2.9 2.8 2.7 2.6 2.5 2.4 2.3 2.2 2.1 2.0 1.9 1.8 1.7 1.6 1.5 1.4 1.3 1.2 1.1 1.0 0.9 0.8 ppm
4.60 14.18 7.09 0.54 17.90 10.04 1.92 1.23 6.56 14.33 6.69 28.99 40.01 142.21 10.52 42.32 10.76 40.53
BRUKER
NAME RM-1719F1
EXPNO 12
PROCNO 1
Date_ 20240620
Time 18.55
INSTRUM spect
PROBHD 5 mm PATXI 1H/
PULPROG zg30
TD 32768
SOLVENT CDCl3
NS 32
DS 0
SWH 5896.227 Hz
FIDRES 0.179939 Hz
AQ 2.7788613 sec
RG 114
DW 84.800 use
DE 6.00 use
TE 298.2 K
D1 20.00000000 sec
TD0 1
======== CHANNEL f1 =======
NUC1 1H
P1 4.30 use
PL1 2.00 dB
PL1W 15.84893227 W
SFO1 600.1530979 MHz
SI 32768
SF 600.1500154 MHz
WDW no
SSB 0
LB 0.00 Hz
GB 0
PC 1.00

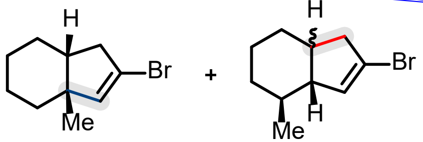

trans-4c + trans-4c'

13C NMR (150 MHz, $CDCl_3$)

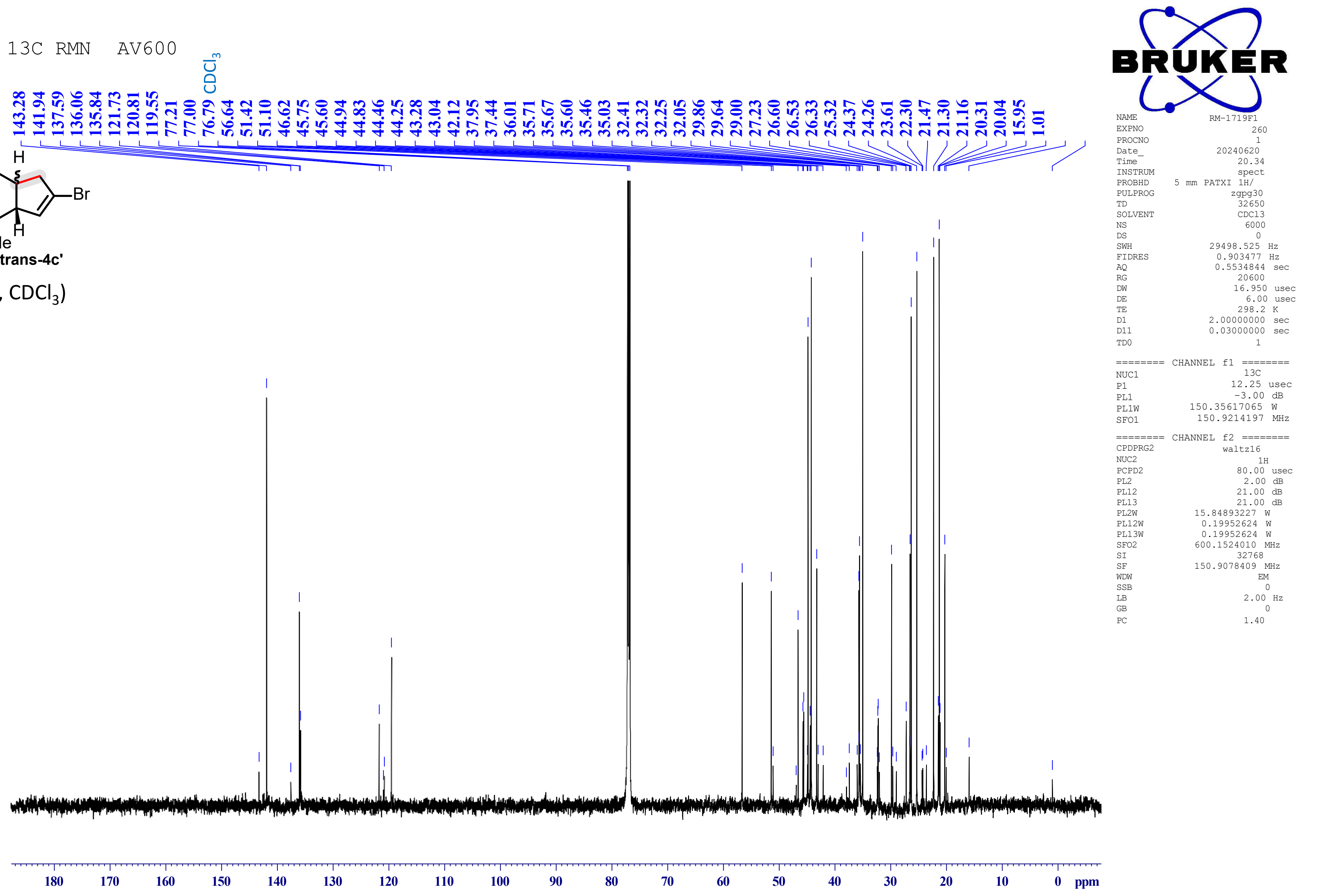

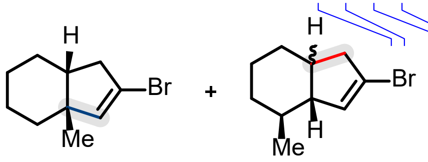

trans-4c + **trans-4c'**

^{13}C NMR (150 MHz, $CDCl_3$)

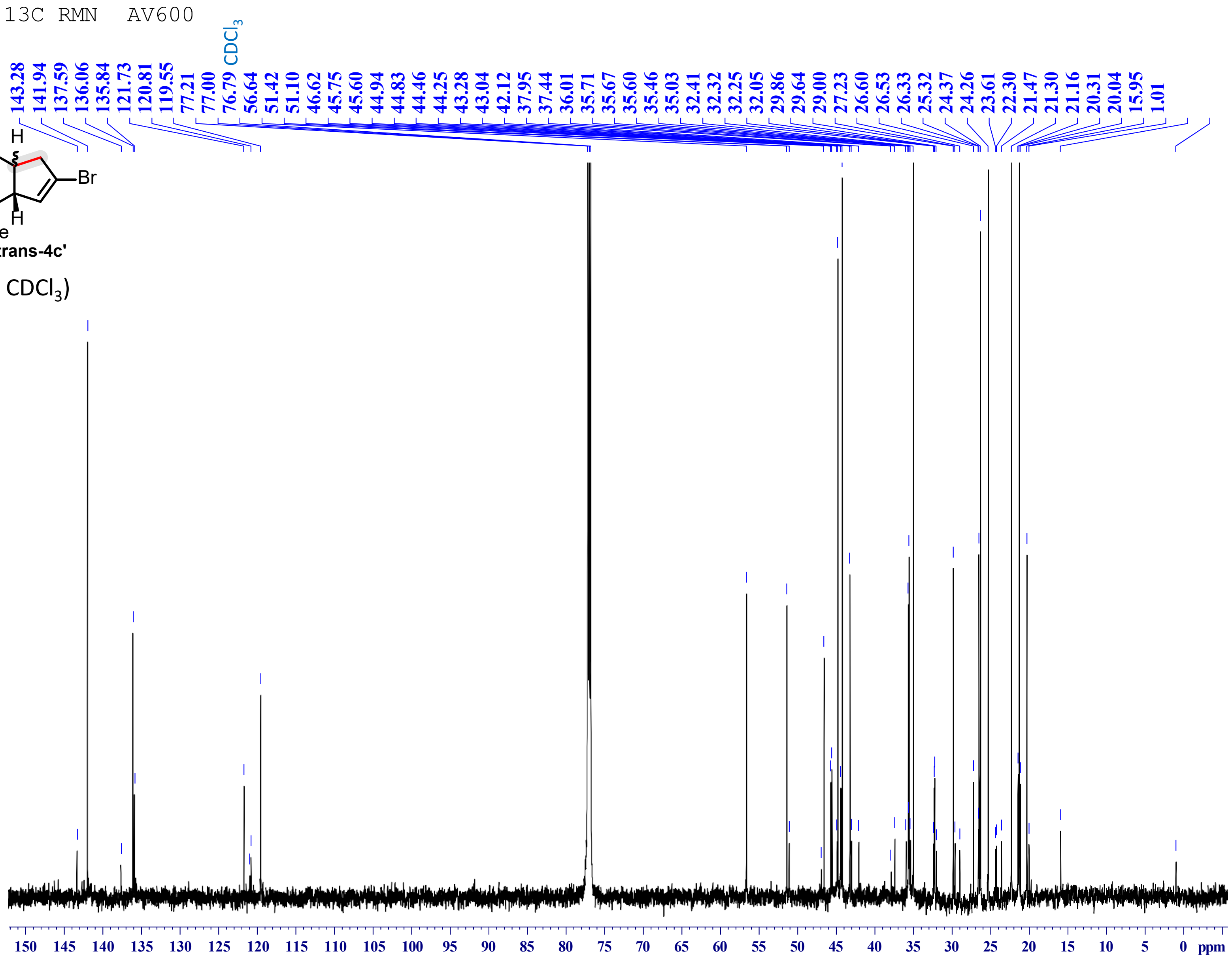


```
NAME            RM-1719F1
EXPNO                 260
PROCNO                  1
Date_            20240620
Time                20.34
INSTRUM             spect
PROBHD   5 mm PATXI 1H/
PULPROG           zgpg30
TD                  32650
SOLVENT             CDCl3
NS                   6000
DS                      0
SWH             29498.525 Hz
FIDRES           0.903477 Hz
AQ              0.5534844 sec
RG                  20600
DW                 16.950 usec
DE                   6.00 usec
TE                  298.2 K
D1             2.00000000 sec
D11            0.03000000 sec
TD0                     1

======== CHANNEL f1 ========
NUC1                  13C
P1                  12.25 usec
PL1                 -3.00 dB
PL1W         150.35617065 W
SFO1          150.9214197 MHz

======== CHANNEL f2 ========
CPDPRG2           waltz16
NUC2                   1H
PCPD2               80.00 usec
PL2                  2.00 dB
PL12                21.00 dB
PL13                21.00 dB
PL2W          15.84893227 W
PL12W          0.19952624 W
PL13W          0.19952624 W
SFO2          600.1524010 MHz
SI                  32768
SF            150.9078409 MHz
WDW                    EM
SSB                     0
LB                   2.00 Hz
GB                      0
PC                   1.40
```

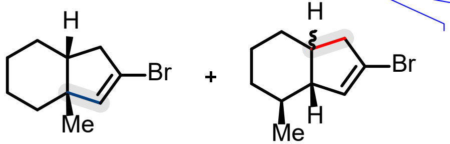

trans-4c **trans-4c'**

^{13}C NMR (150 MHz, $CDCl_3$)

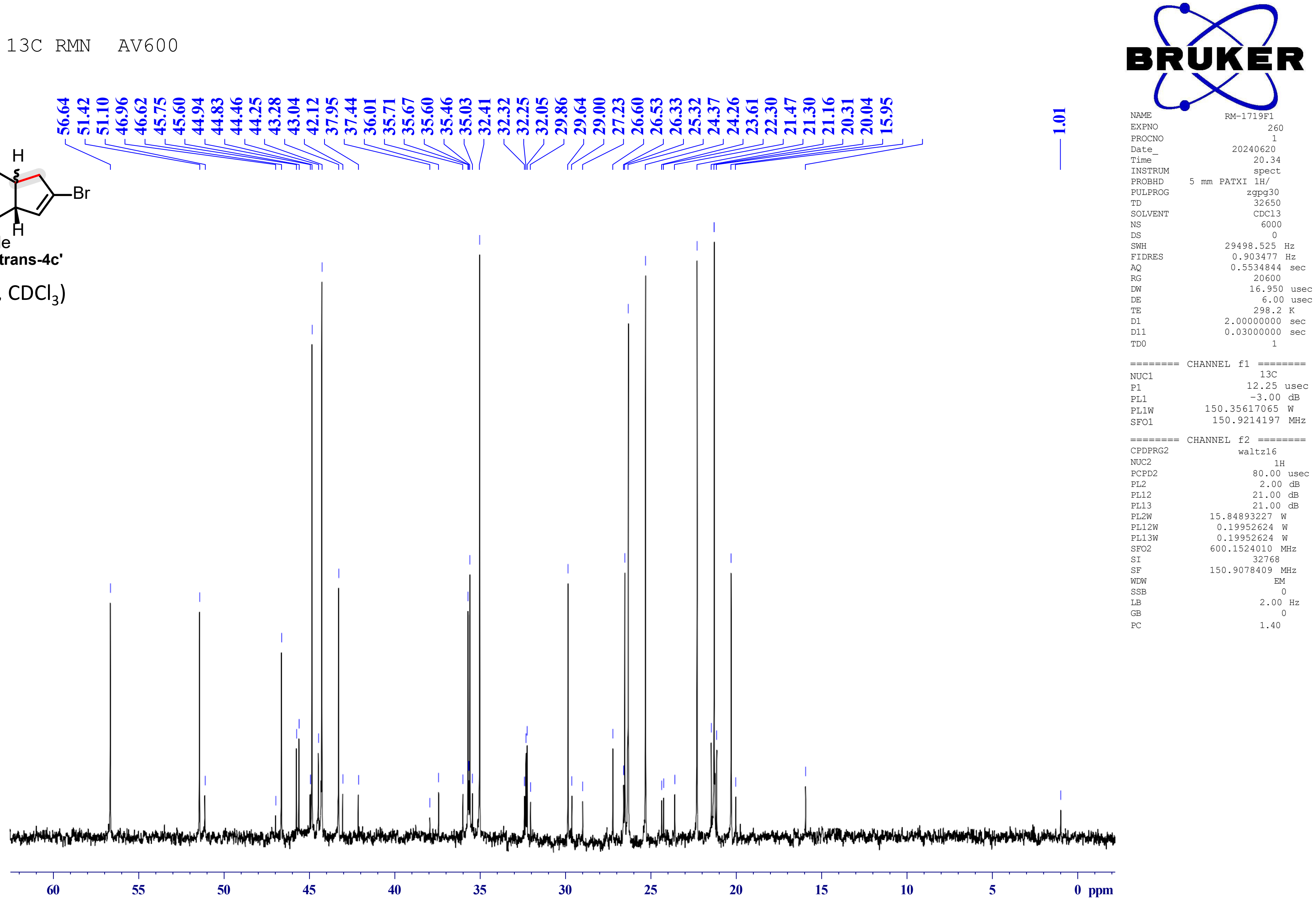

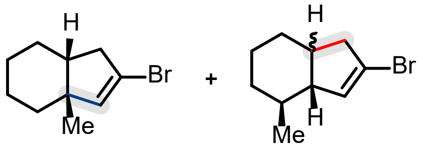

trans-4c + trans-4c'

COSY NMR([600, 600] MHz, $CDCl_3$)

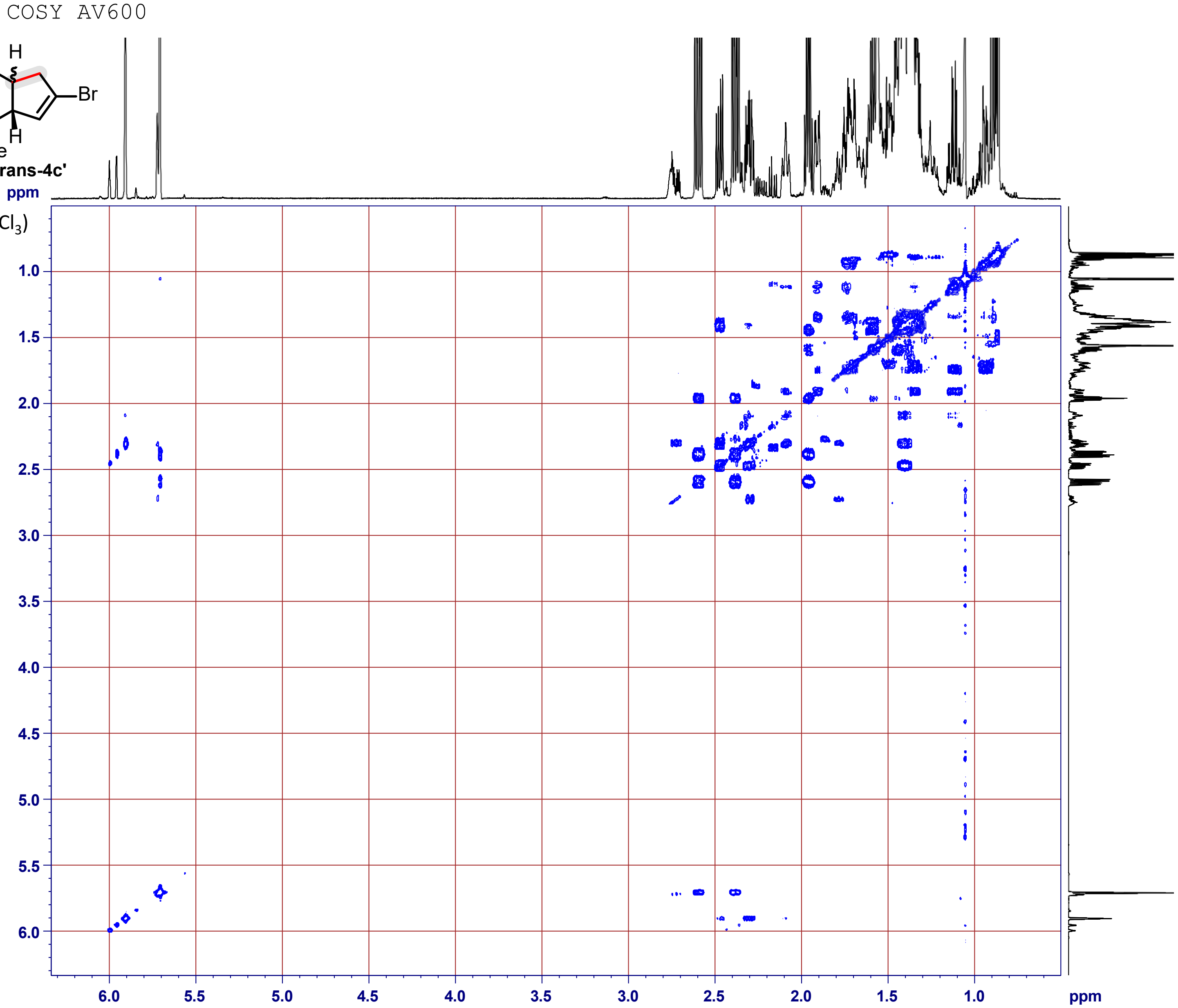

BRUKER

```
NAME              RM-1719F1
EXPNO                     6
PROCNO                    1
Date_              20240620
Time                  14.05
INSTRUM               spect
PROBHD   5 mm PATXI 1H/
PULPROG            cosygpqf
TD                     2048
SOLVENT               CDCl3
NS                        2
DS                       12
SWH                3501.401 Hz
FIDRES             1.709668 Hz
AQ                0.2926472
sec RG                  362
DW                  142.800 usec
DE                     6.00 usec
TE                    298.2 K
D0               0.00000300 sec
D1               1.00000000 sec
D13              0.00000400 sec
D16              0.00015000 sec
IN0              0.00028560 sec

======== CHANNEL f1 ========
NUC1                     1H
P0                     8.18 usec
P1                     8.18 usec
PL1                    2.00 dB
PL1W            15.84893227 W
SFO1            600.1520674 MHz

====== GRADIENT CHANNEL =====
GPNAM1             SINE.100
GPZ1                  10.00 %
P16                 1000.00
usec ND0                      1
TD                      256
SFO1               600.1521
MHz FIDRES        13.677372
Hz SW                 5.834
ppm FnMODE               QF
SI                     1024
SF              600.1500154 MHz
WDW                    SINE
SSB                       0
LB                     0.00 Hz
GB                        0
PC                     1.00
SI                     1024
MC2                      QF
SF              600.1500154 MHz
WDW                    SINE
SSB                       0
LB                     0.00 Hz
GB                        0
```

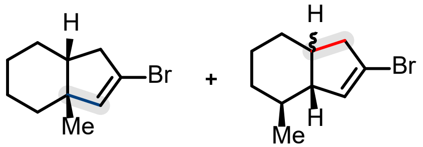

trans-4c + **trans-4c'**

COSY NMR([600, 600] MHz, $CDCl_3$)

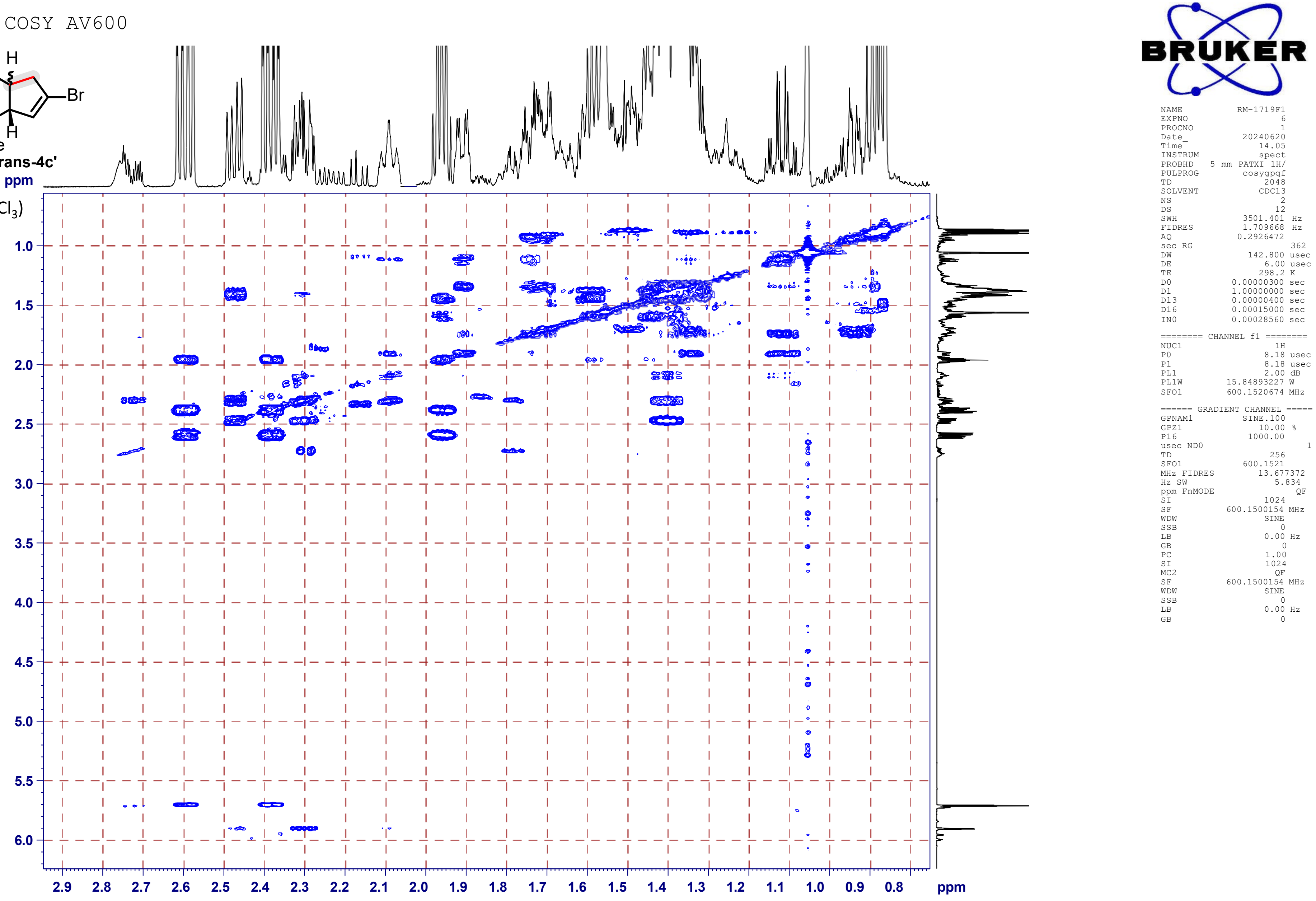

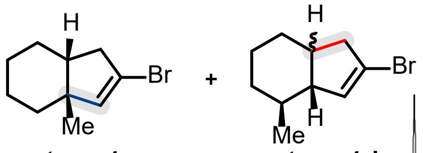

trans-4c **trans-4c'**

TOCSY NMR([600, 600] MHz, $CDCl_3$)

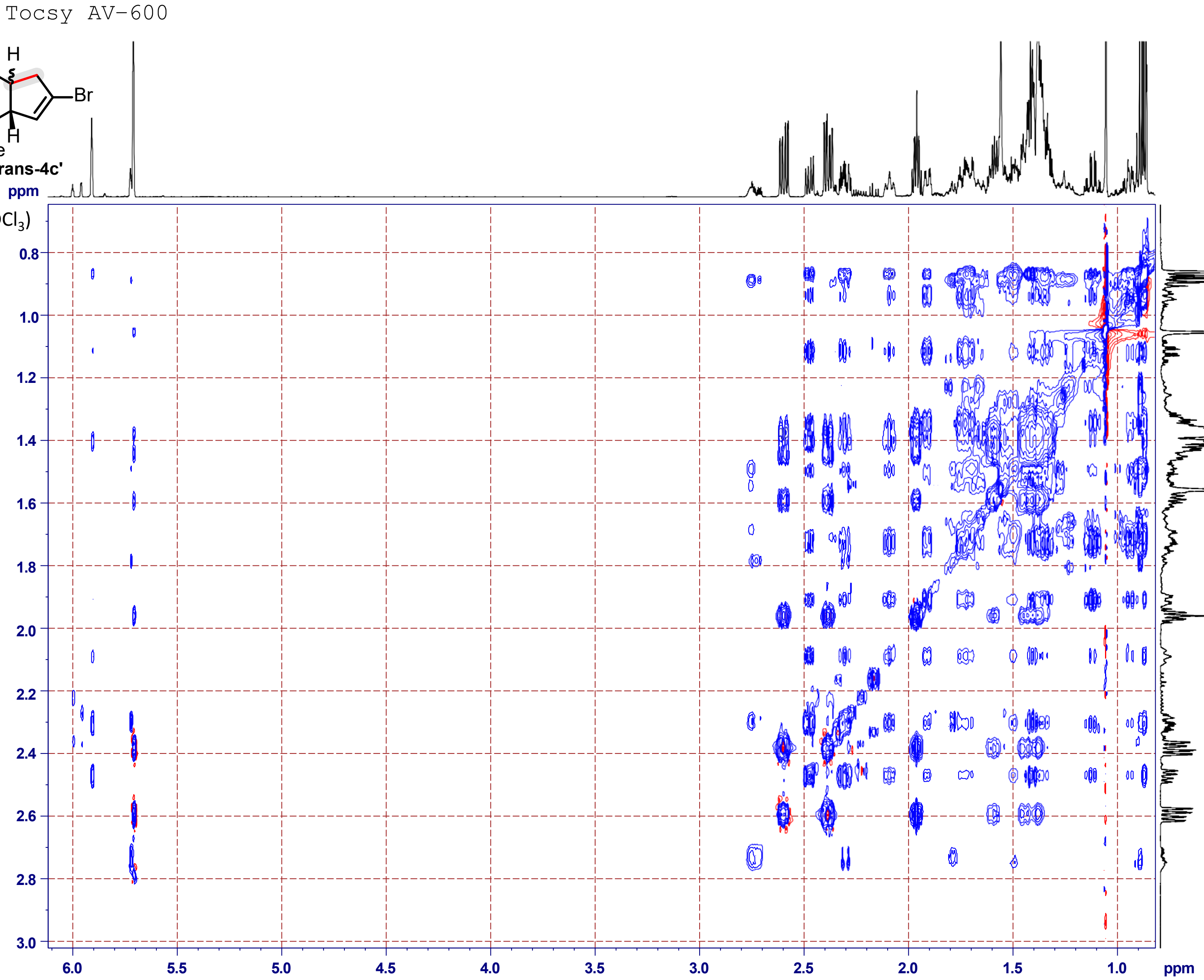


```
NAME          RM-1719F1
EXPN          9
O
PROCNO        1
Date_         20240620
Time          15.06
INSTRUM       spect
PROBHD        5 mm PATXI 1H/
PULPROG       dipsi2etgpsi
TD            2048
SOLVENT       CDCl3
NS            4
DS            8
SWH           3501.401 Hz
FIDRES        1.709668 Hz
AQ            0.2926472 sec
RG            71.8
DW            142.800 use
DE            6.50 use
TE            298.2 K
D0            0.00000300 sec
D1            1.00000000 sec
D9            0.06000000 sec
D11           0.03000000 sec
D16           0.00015000 sec
D20           0.00001000 sec
L1            18
D21           0.00001000 sec
IN0           0.00028560 sec
======== CHANNEL f1 ========
NUC1          1H
P1            8.18 use
P2            16.36 use
P6            30.00 use
PL1           2.00 dB
PL10          13.29 dB
PL1W          15.84893227 W
PL10W         1.17832196 W
SFO1          600.1520674 MHz

====== GRADIENT CHANNEL ===
GPNAM1        SINE.100
GPNAM2        SINE.100
GPZ1          30.00 %
GPZ2          30.00 %
P16           1000.00 use
ND0           1
TD            256
SFO1          600.1521 MHz
FIDRES        13.677372 Hz
SW            5.834 ppm
FnMODE        Echo-Antiecho
SI            1024
SF            600.1500154 MHz
WDW           QSINE
SSB           2
LB            0.00 Hz
GB            0
MC2           echo-antiecho
SF            600.1500154 MHz
WDW           QSINE
PC            1.00
SSB           2
SI            1024
LB            0.00 Hz
GB            0
```

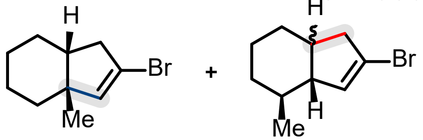

trans-4c + trans-4c'

TOCSY NMR([600, 600] MHz, $CDCl_3$)

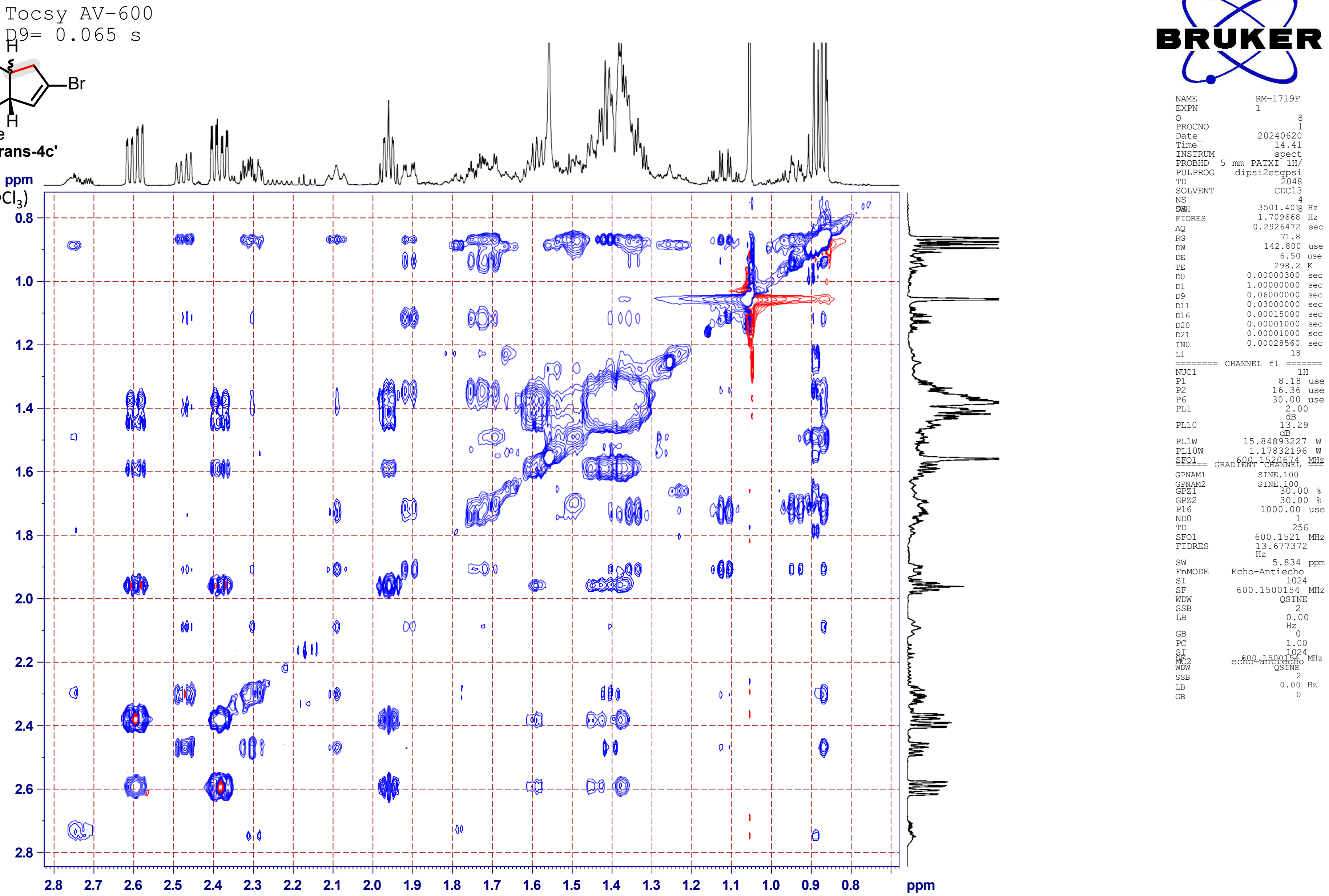

AV600 seltocsy D9= 90 ms
minor: CH 2.75ppm +CH2 2.70ppm

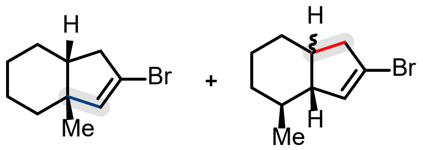

trans-4c trans-4c'

SEL-TOCSY NMR(600 MHz, $CDCl_3$)

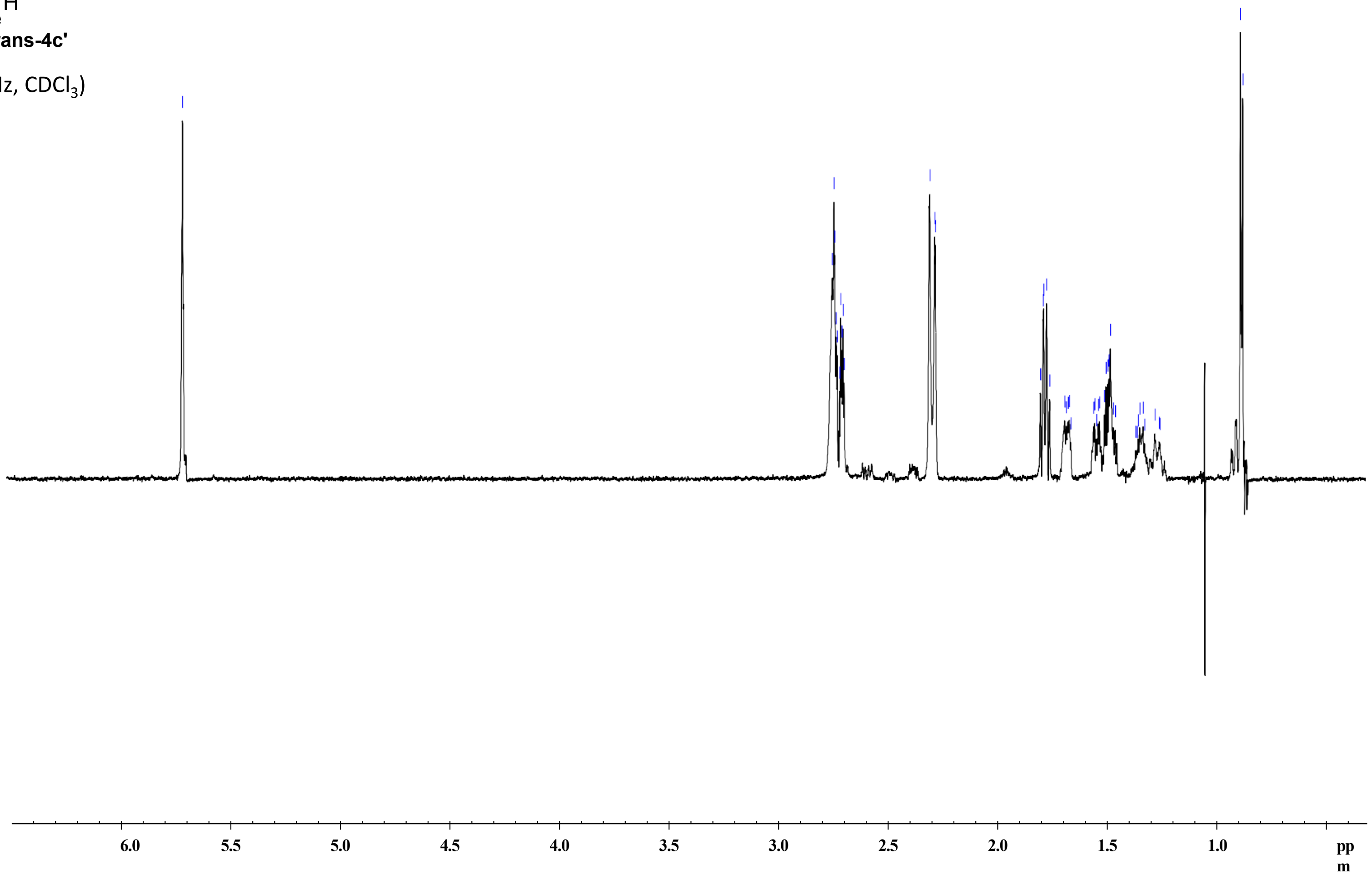

NAME RM-1719F1
EXPNO 18
PROCNO 1
Date_ 20240620
Time 19.21
INSTRUM spect
PROBHD 5 mm PATXI 1H/
PULPROG selmlgp
TD 16384
SOLVENT CDCl3
NS 32
DS 4
SWH 3720.238 Hz
FIDRES 0.227065 Hz
AQ 2.2021940 sec
RG 2300
DW 134.400 usec
DE 6.00 usec
TE 298.2 K
D1 1.00000000 sec
D9 0.09000000 sec
D16 0.00015000 sec
L1 48
TD0 1

======== CHANNEL f1 ========
NUC1 1H
P1 8.60 usec
P5 20.01 usec
P6 30.00 usec
P7 60.00 usec
P12 25317.70 usec
P17 1000.00 usec
PL0 120.00 dB
PL1 2.00 dB
PL10 12.85 dB
PL0W 0.00000000 W
PL1W 15.84893227 W
PL10W 1.30316663 W
SFO1 600.1520690 MHz
SP2 58.01 dB
SPNAM2 Gaus1.1000
SPOAL2 0.500
SPOFFS2 -417.99 Hz

====== GRADIENT CHANNEL ====
GPNAM1 SINE.100
GPZ1 15.00 %
P16 1000.00 usec
SI 32768
SF 600.1500154 MHz
WDW EM
SSB 0
LB 0.50 Hz
GB 0
PC 1.00

AV600 seltocsy D9= 90 ms
minor: CH 2.75ppm +CH2 2.70ppm

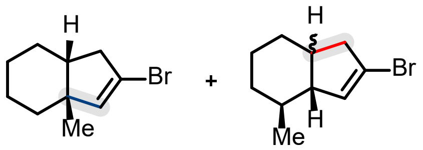

trans-4c + trans-4c'

SEL-TOCSY NMR(600 MHz, $CDCl_3$)

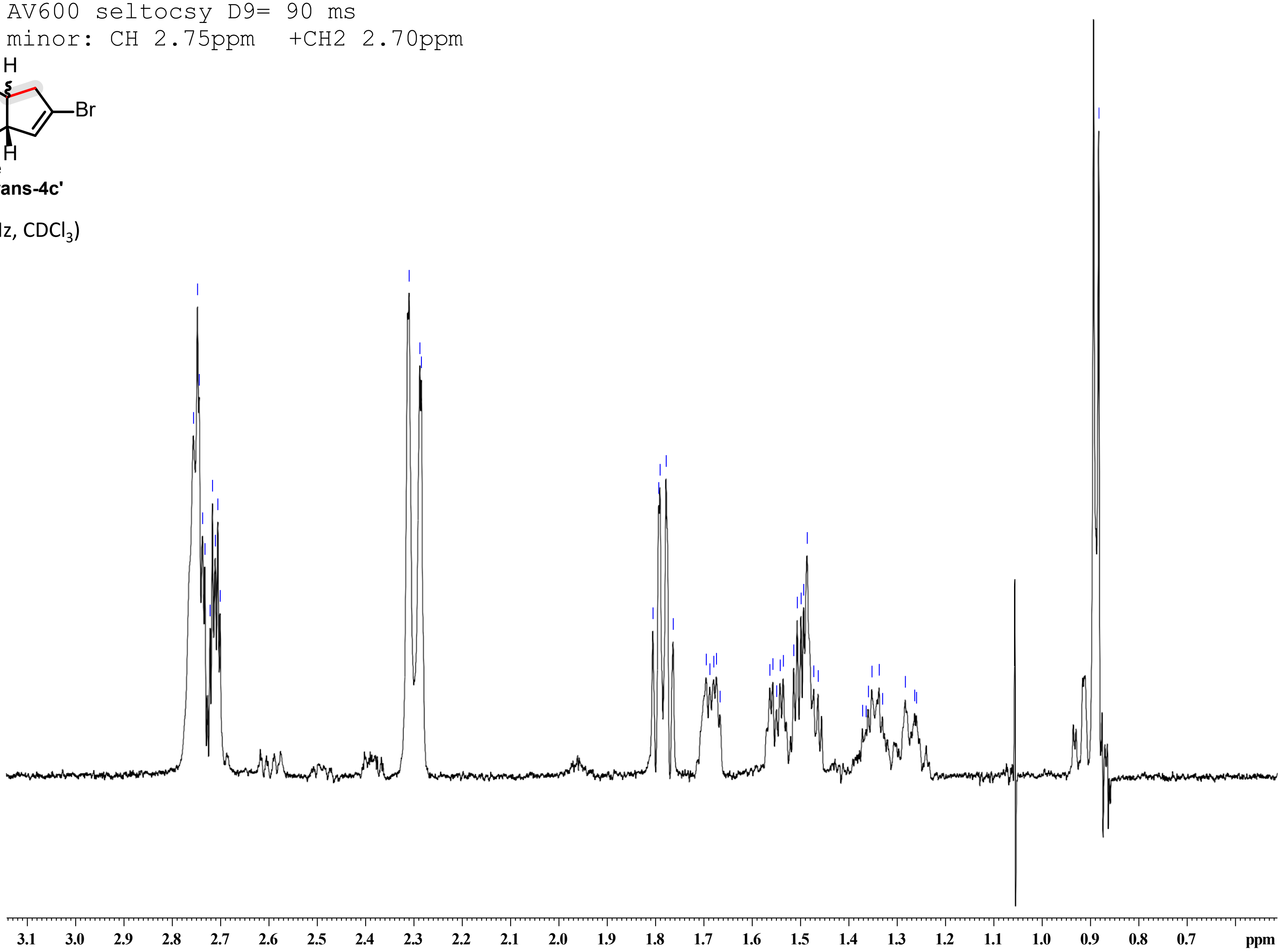

NAME	RM-1719F1
EXPNO	18
PROCNO	1
Date_	20240620
Time	19.21
INSTRUM	spect
PROBHD	5 mm PATXI 1H/
PULPROG	selmlgp
TD	16384
SOLVENT	CDCl3
NS	32
DS	4
SWH	3720.238 Hz
FIDRES	0.227065 Hz
AQ	2.2021940 sec
RG	2300
DW	134.400 usec
DE	6.00 usec
TE	298.2 K
D1	1.00000000 sec
D9	0.09000000 sec
D16	0.00015000 sec
L1	48
TD0	1

======== CHANNEL f1 ========

NUC1	1H
P1	8.60 usec
P5	20.01 usec
P6	30.00 usec
P7	60.00 usec
P12	25317.70 usec
P17	1000.00 usec
PL0	120.00 dB
PL1	2.00 dB
PL10	12.85 dB
PL0W	0.00000000 W
PL1W	15.84893227 W
PL10W	1.30316663 W
SFO1	600.1520690 MHz
SP2	58.01 dB
SPNAM2	Gaus1.1000
SPOAL2	0.500
SPOFFS2	-417.99 Hz

====== GRADIENT CHANNEL ====

GPNAM1	SINE.100
GPZ1	15.00 %
P16	1000.00 usec
SI	32768
SF	600.1500154 MHz
WDW	EM
SSB	0
LB	0.50 Hz
GB	0
PC	1.00

AV600 selTOCSY D9 = 90ms
CH 2.09 ppm

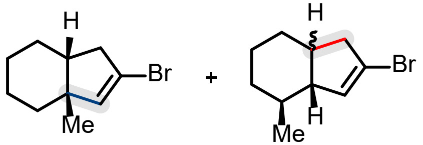

trans-4c   trans-4c'

SEL-TOCSY NMR(600 MHz, $CDCl_3$)

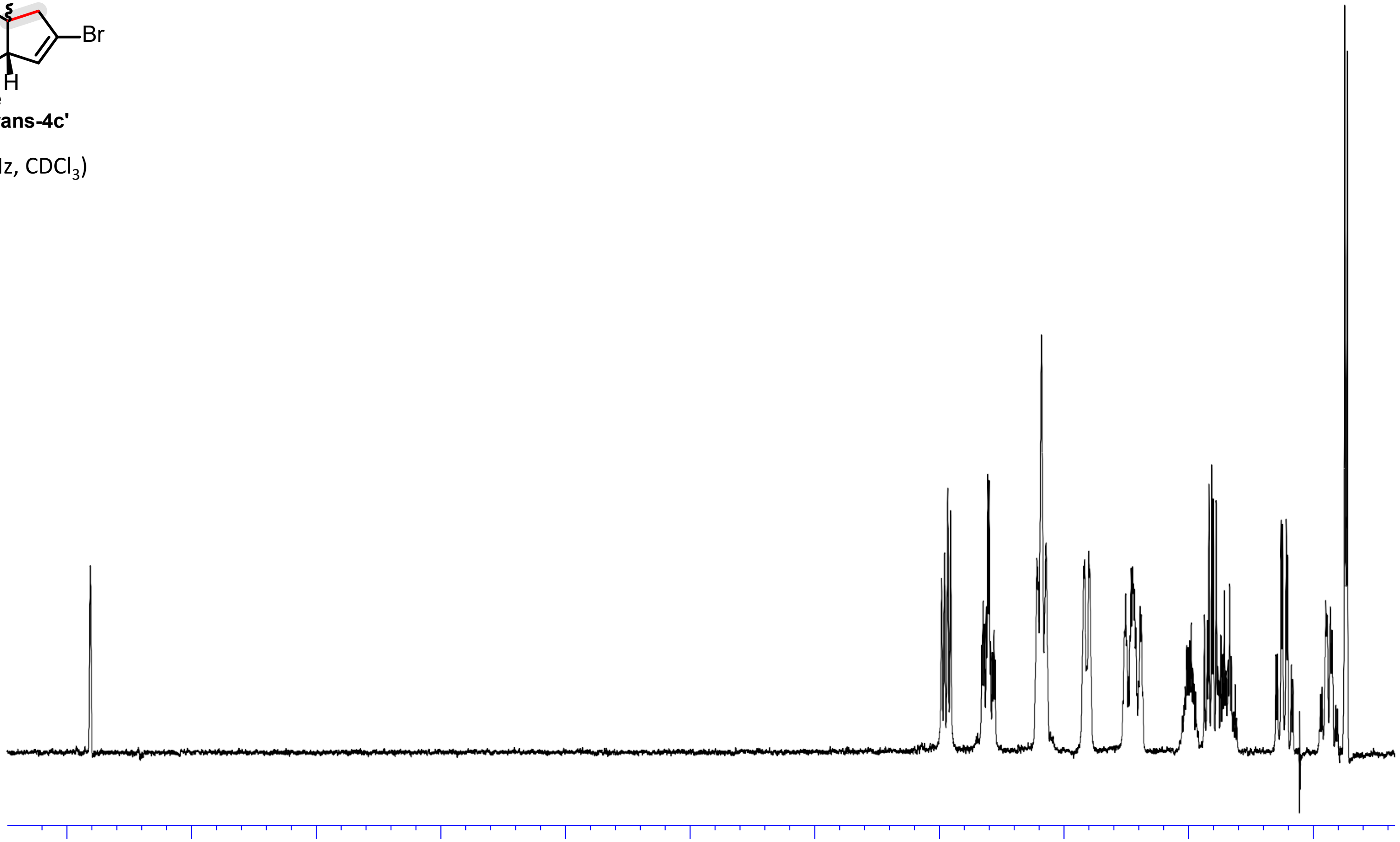

NAME	RM-1719F1
EXPNO	21
PROCNO	1
Date_	20240620
Time	19.28
INSTRUM	spect
PROBHD	5 mm PATXI 1H/
PULPROG	selmlgp
TD	16384
SOLVENT	CDCl3
NS	32
DS	4
SWH	3720.238 Hz
FIDRES	0.227065 Hz
AQ	2.2021940 sec
RG	2580
DW	134.400 usec
DE	6.00 usec
TE	298.2 K
D1	1.00000000 sec
D9	0.09000000 sec
D16	0.00015000 sec
L1	48
TD0	1

======== CHANNEL f1 ========

1H

8.60 usec

20.01 usec

30.00 usec

60.00 usec

42325.01 usec

1000.00 usec

120.00 dB

2.00 dB

12.85 dB

0.00000000 W

15.84893227 W

1.30316663 W

600.1520690 MHz

AV600
seltocsy D9=90ms
CH 2.09 ppm

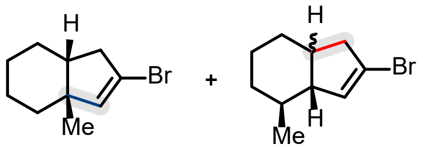

trans-4c trans-4c'

SEL-TOCSY NMR(600 MHz, $CDCl_3$)

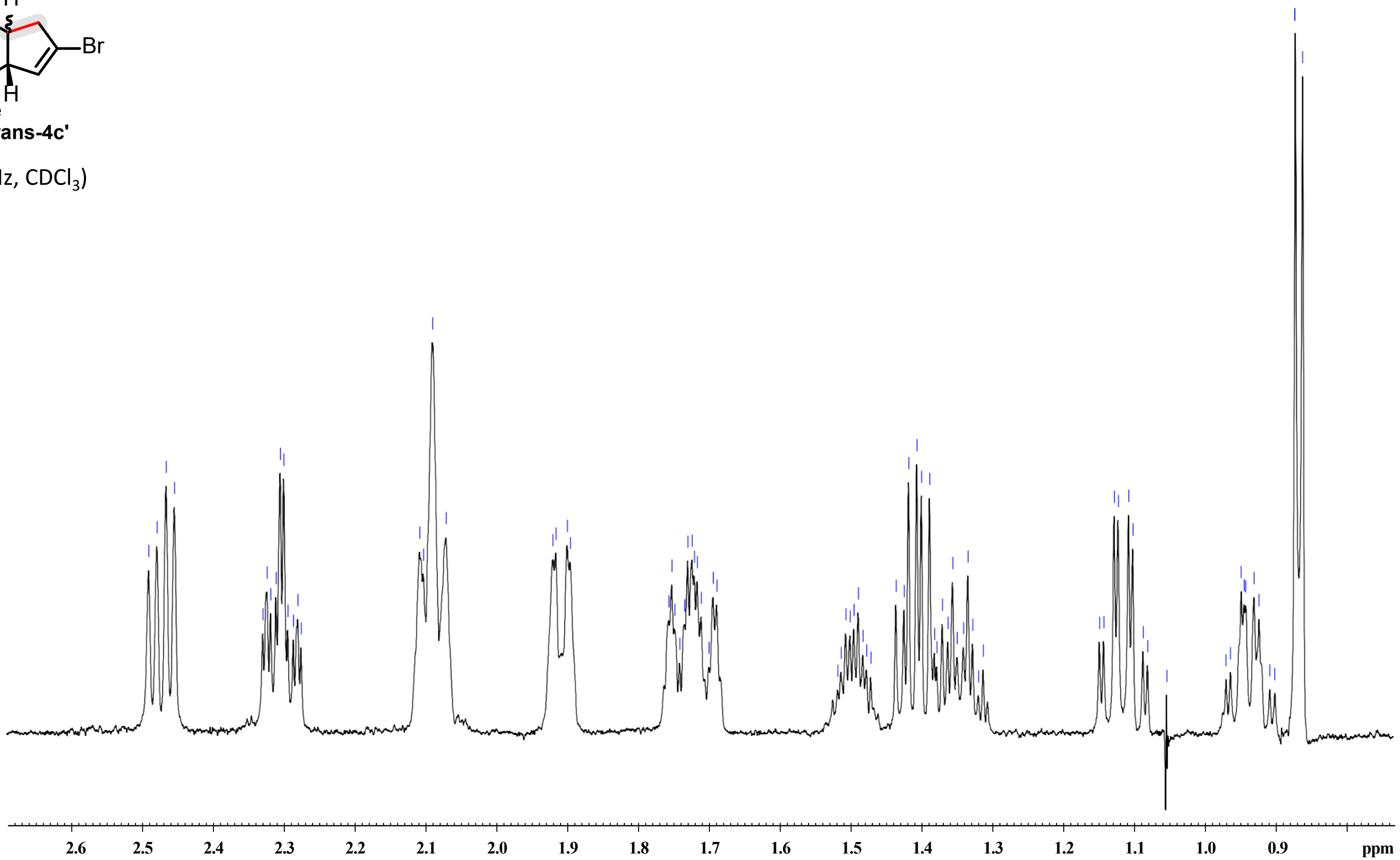

NAME RM-1719F1
EXPNO 21
PROCNO 1
Date_ 20240620
Time 19.28
INSTRUM spect
PROBHD 5 mm PATXI 1H/
PULPROG selmlgp
TD 16384
SOLVENT CDCl3
NS 32
DS 4
SWH 3720.238 Hz
FIDRES 0.227065 Hz
AQ 2.2021940 sec
RG 2580
DW 134.400 usec
DE 6.00 usec
TE 298.2 K
D1 1.00000000 sec
D9 0.09000000 sec
D16 0.00015000 sec
L1 48
TD0 1

======== CHANNEL f1 ========
NUC1 1H
P1 8.60 usec
P5 20.01 usec
P6 30.00 usec
P7 60.00 usec
P12 42325.01 usec
P17 1000.00 usec
PL0 120.00 dB
PL1 2.00 dB
PL10 12.85 dB
PL0W 0.00000000 W
PL1W 15.84893227 W
PL10W 1.30316663 W
SFO1 600.1520690 MHz
SP2 62.47 dB
SPNAM2 Gaus1.1000
SPOAL2 0.500
SPOFFS2 -798.41 Hz

====== GRADIENT CHANNEL ====
GPNAM1 SINE.100
GPZ1 15.00 %
P16 1000.00 usec
SI 32768
SF 600.1500154 MHz
WDW EM
SSB 0
LB 0.50 Hz
GB 0
PC 1.00

AV600 seltocsy D9 = 90 ms
CH 1.96 ppm

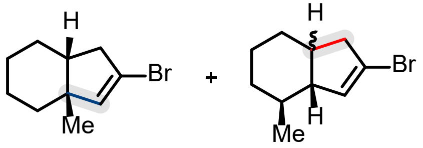

trans-4c **trans-4c'**

SEL-TOCSY NMR(600 MHz, $CDCl_3$)

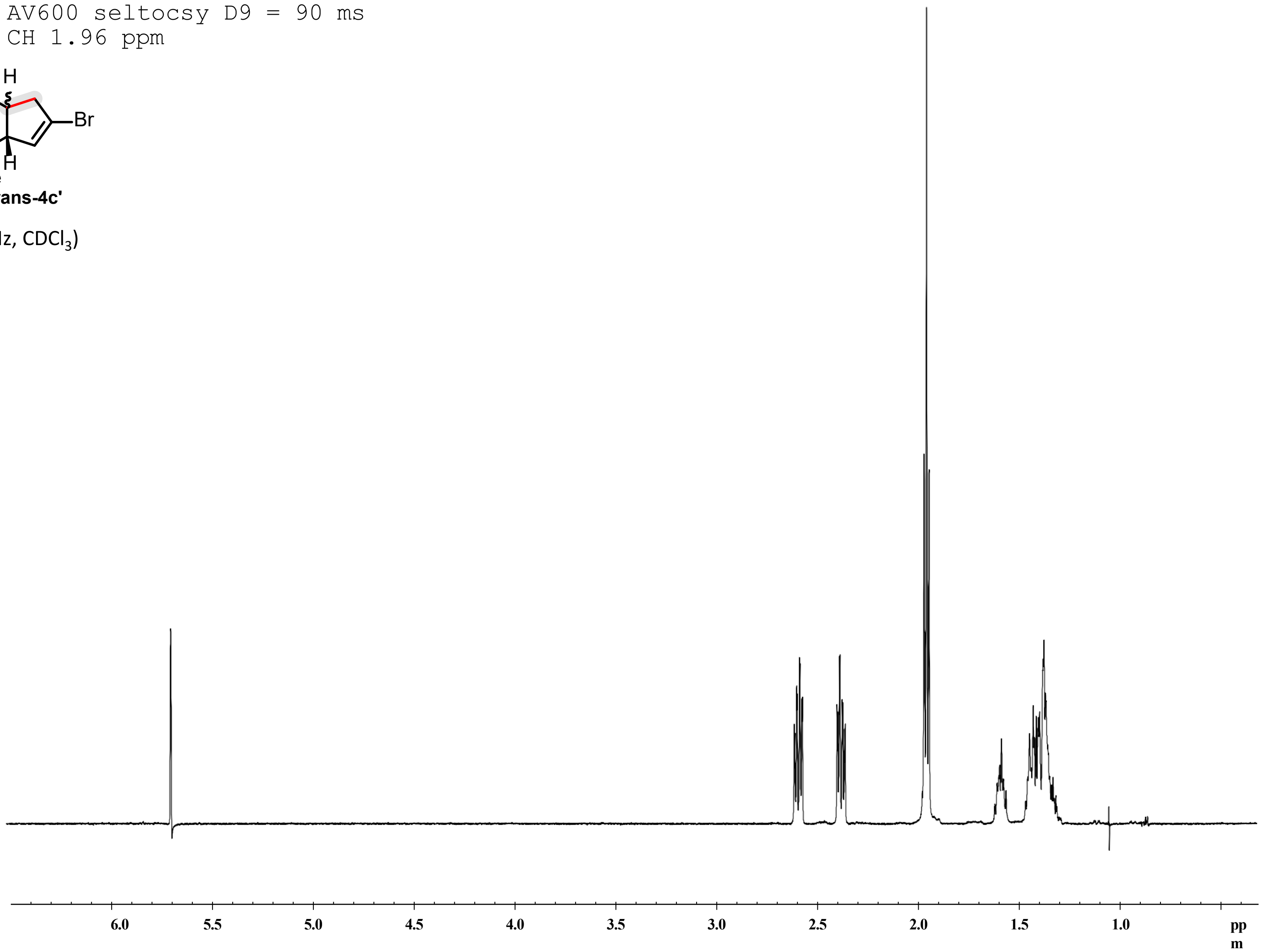

BRUKER

```
NAME           RM-1719F1
EXPNO                 22
PROCNO                 1
Date_           20240620
Time               19.42
INSTRUM            spect
PROBHD   5 mm PATXI 1H/
PULPROG          selmlgp
TD                 16384
SOLVENT            CDCl3
NS                    32
DS                     4
SWH             3720.238 Hz
FIDRES          0.227065 Hz
AQ             2.2021940 sec
RG                  3640
DW               134.400 usec
DE                  6.00 usec
TE                 298.2 K
D1            1.00000000 sec
D9            0.09000000 sec
D16           0.00015000 sec
L1                    48
TD0                    1

======== CHANNEL f1 ========
NUC1                  1H
P1                  8.60 usec
P5                 20.01 usec
P6                 30.00 usec
P7                 60.00 usec
P12             60347.83 usec
P17              1000.00 usec
PL0               120.00 dB
PL1                 2.00 dB
PL10               12.85 dB
PL0W          0.00000000 W
PL1W         15.84893227 W
PL10W         1.30316663 W
SFO1         600.1520690 MHz
SP2                65.55 dB
SPNAM2        Gaus1.1000
SPOAL2             0.500
SPOFFS2          -876.09 Hz

====== GRADIENT CHANNEL ====
GPNAM1          SINE.100
GPZ1               15.00 %
P16              1000.00 usec
SI                 32768
SF           600.1500154 MHz
WDW                   EM
SSB                    0
LB                  0.30 Hz
GB                     0
PC                  1.00
```

AV600 seltocsy D9 = 90 ms
CH 1.96 ppm

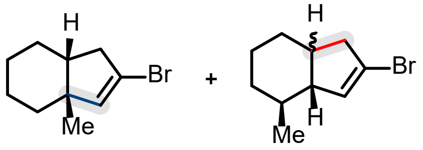

trans-4c **trans-4c'**

SEL-TOCSY NMR(600 MHz, $CDCl_3$)

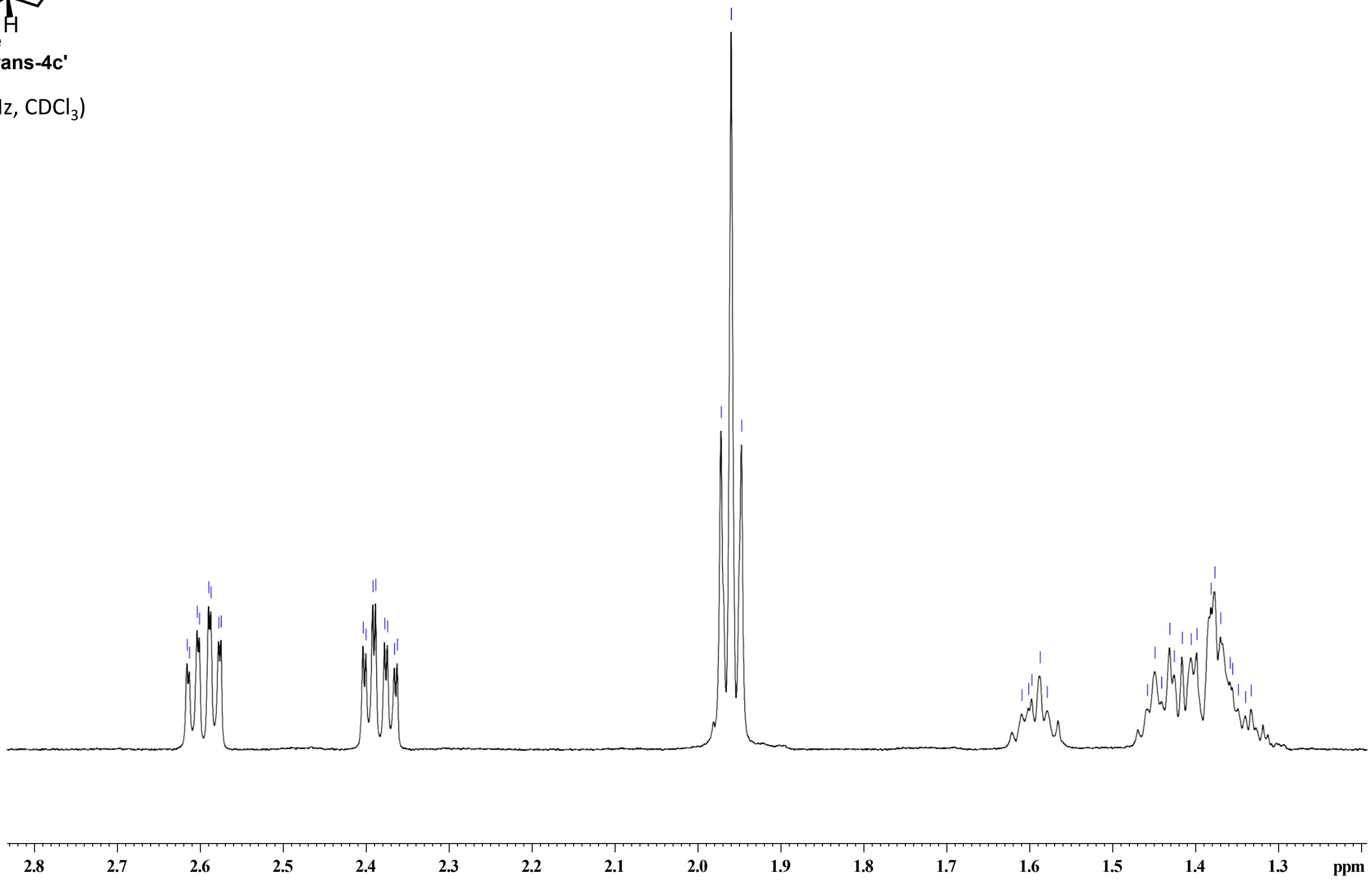

BRUKER

```
NAME            RM-1719F1
EXPNO                  22
PROCNO                  1
Date_            20240620
Time                19.42
INSTRUM             spect
PROBHD   5 mm PATXI 1H/
PULPROG           selmlgp
TD                  16384
SOLVENT             CDCl3
NS                     32
DS                      4
SWH             3720.238 Hz
FIDRES          0.227065 Hz
AQ             2.2021940 sec
RG                   3640
DW               134.400 usec
DE                  6.00 usec
TE                 298.2 K
D1            1.00000000 sec
D9            0.09000000 sec
D16           0.00015000 sec
L1                     48
TD0                     1

======== CHANNEL f1 ========
NUC1                   1H
P1                   8.60 usec
P5                  20.01 usec
P6                  30.00 usec
P7                  60.00 usec
P12             60347.83 usec
P17              1000.00 usec
PL0               120.00 dB
PL1                 2.00 dB
PL10               12.85 dB
PL0W          0.00000000 W
PL1W         15.84893227 W
PL10W         1.30316663 W
SFO1         600.1520690 MHz
SP2                65.55 dB
SPNAM2        Gaus1.1000
SPOAL2             0.500
SPOFFS2          -876.09 Hz

====== GRADIENT CHANNEL ====
GPNAM1          SINE.100
GPZ1               15.00 %
P16              1000.00 usec
SI                 32768
SF           600.1500154 MHz
WDW                   EM
SSB                    0
LB                  0.30 Hz
GB                     0
PC                  1.00
```

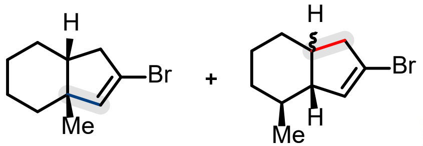

trans-4c **trans-4c'**

SEL-TOCSYs NMR(600 MHz, $CDCl_3$)

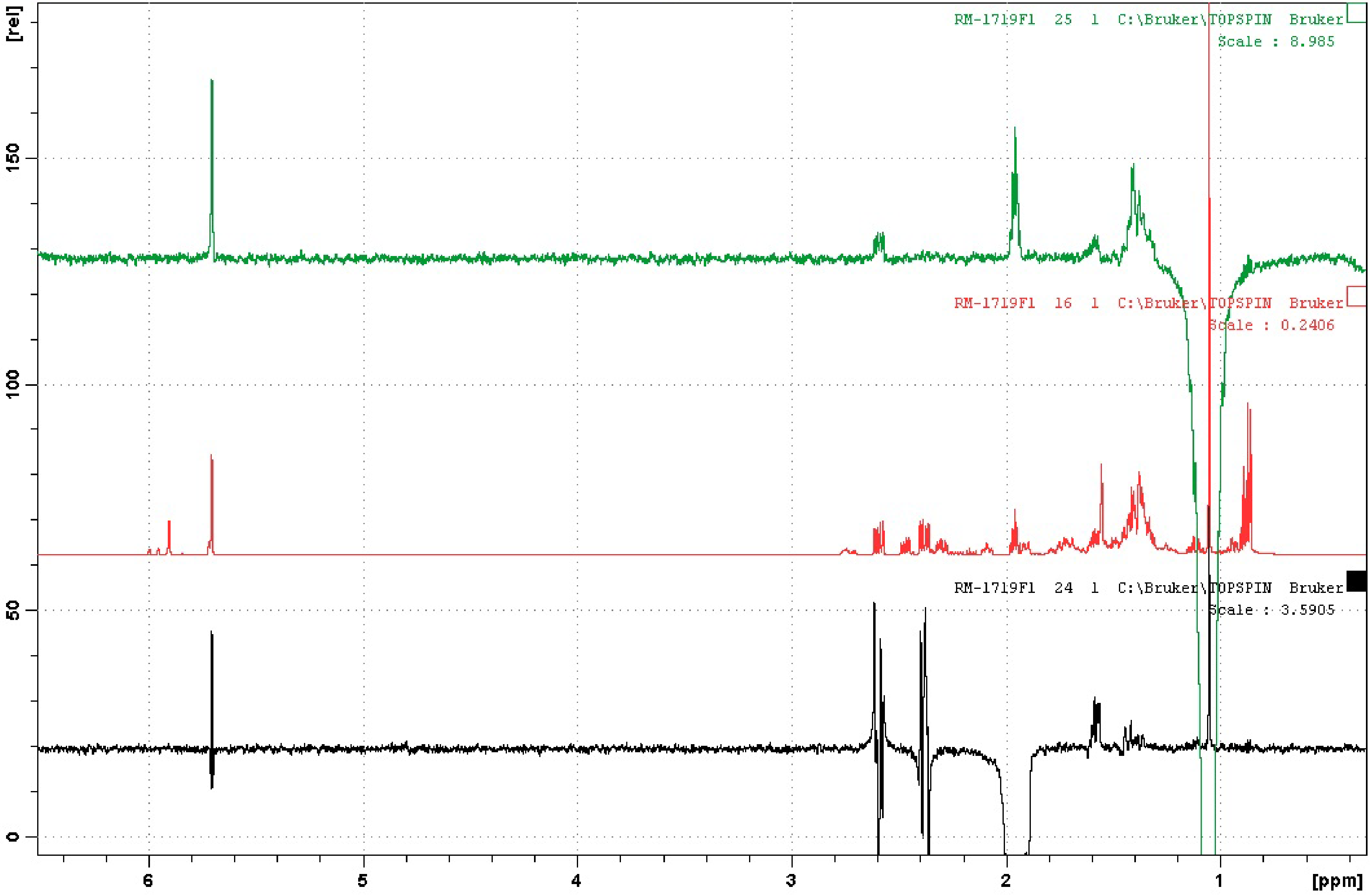

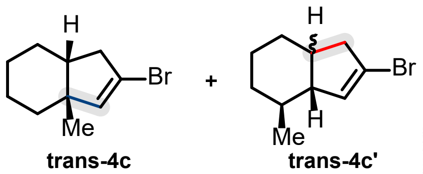

SEL-TOCSYs NMR(600 MHz, $CDCl_3$)

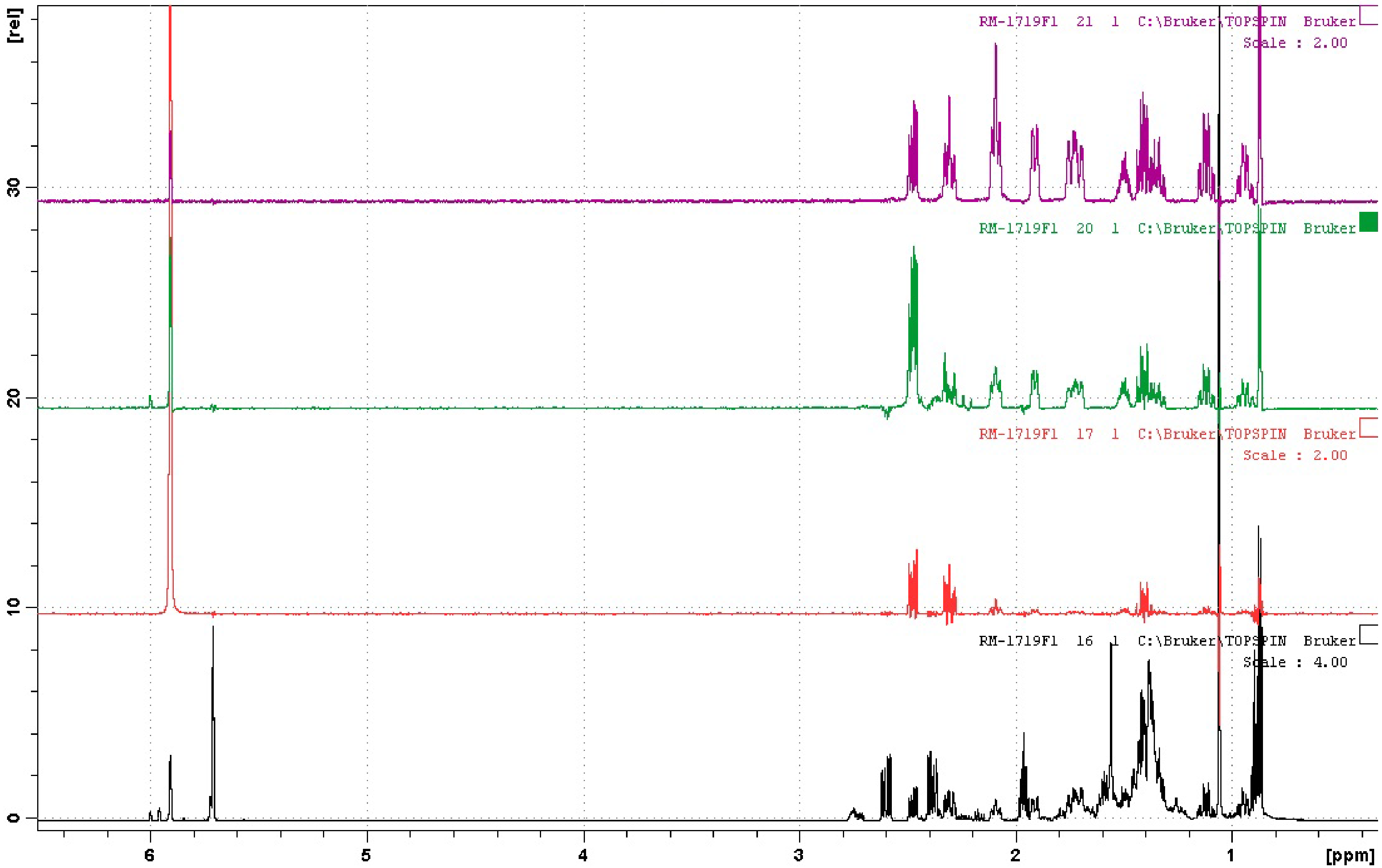

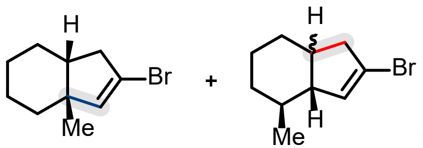

trans-4c + **trans-4c'**

SEL-TOCSYs NMR(600 MHz, $CDCl_3$)

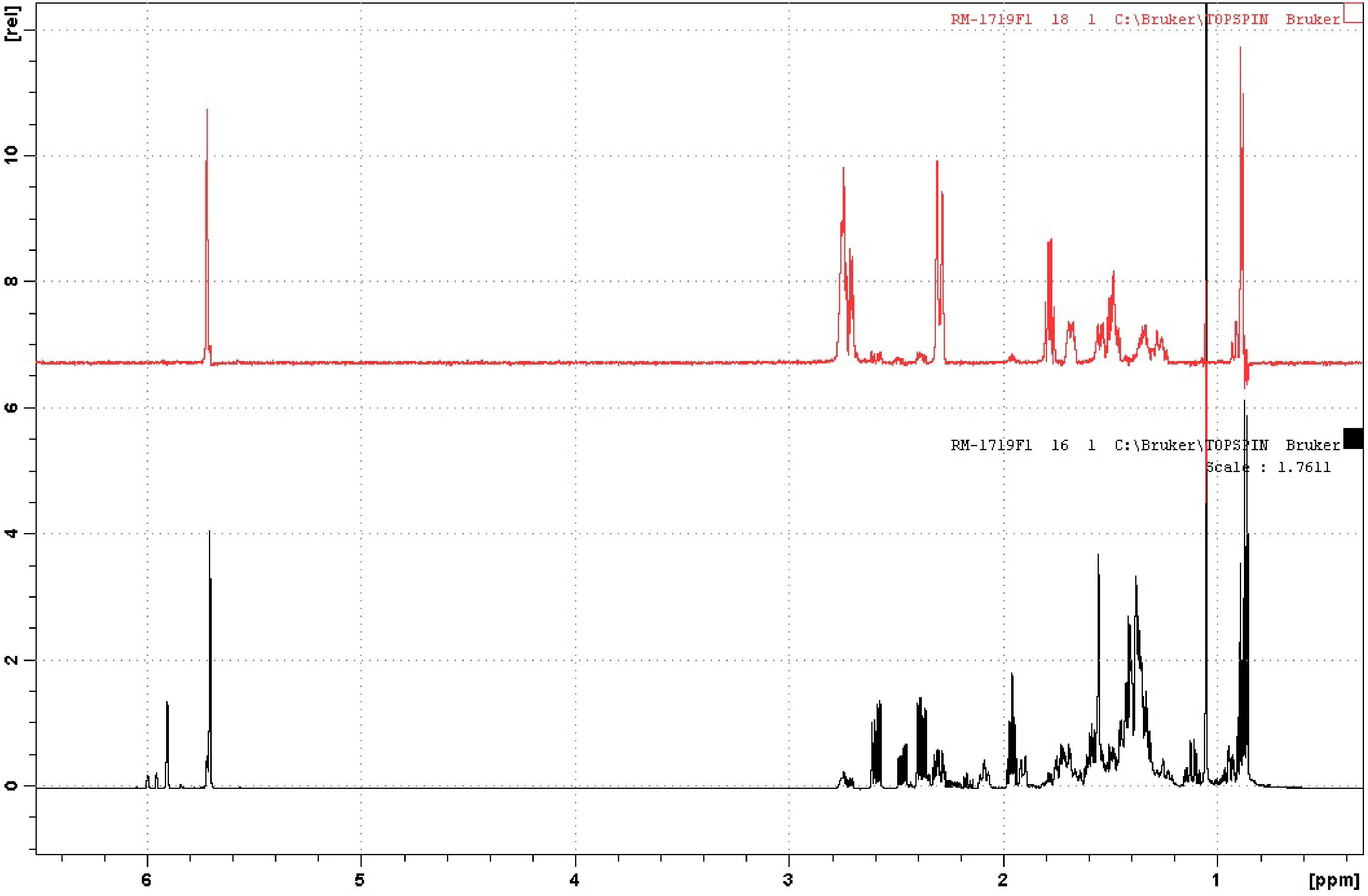

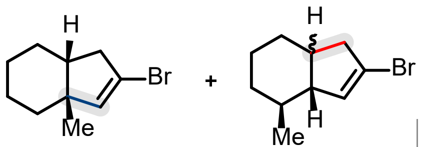

trans-4c + trans-4c'

SEL-TOCSYs NMR(600 MHz, $CDCl_3$)

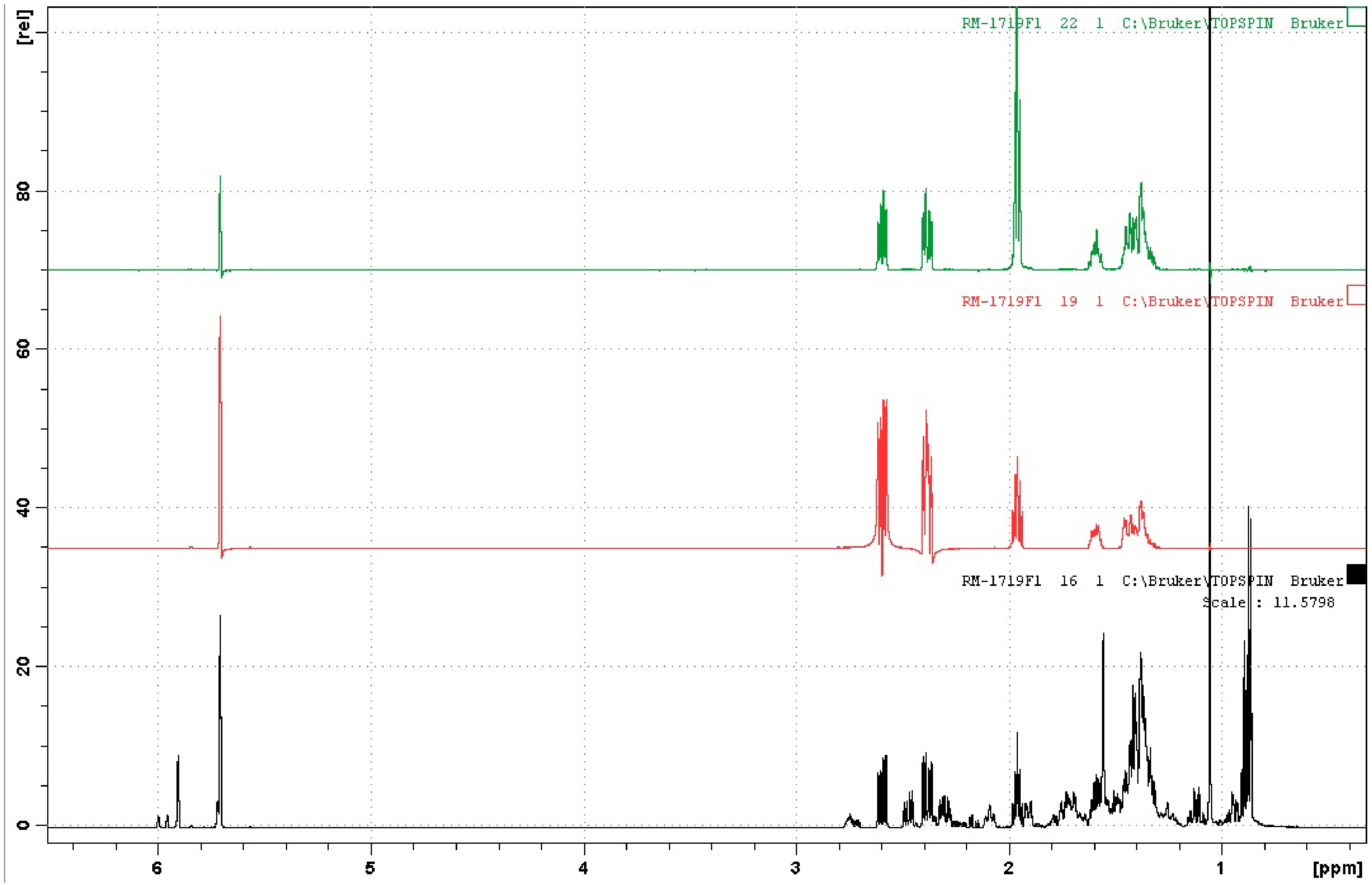

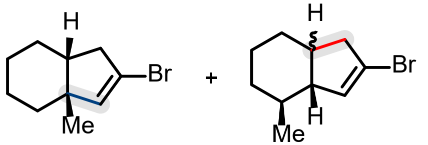

trans-4c **trans-4c'**

HSQC/SEL-TOCSY NMR(600 MHz, $CDCl_3$)

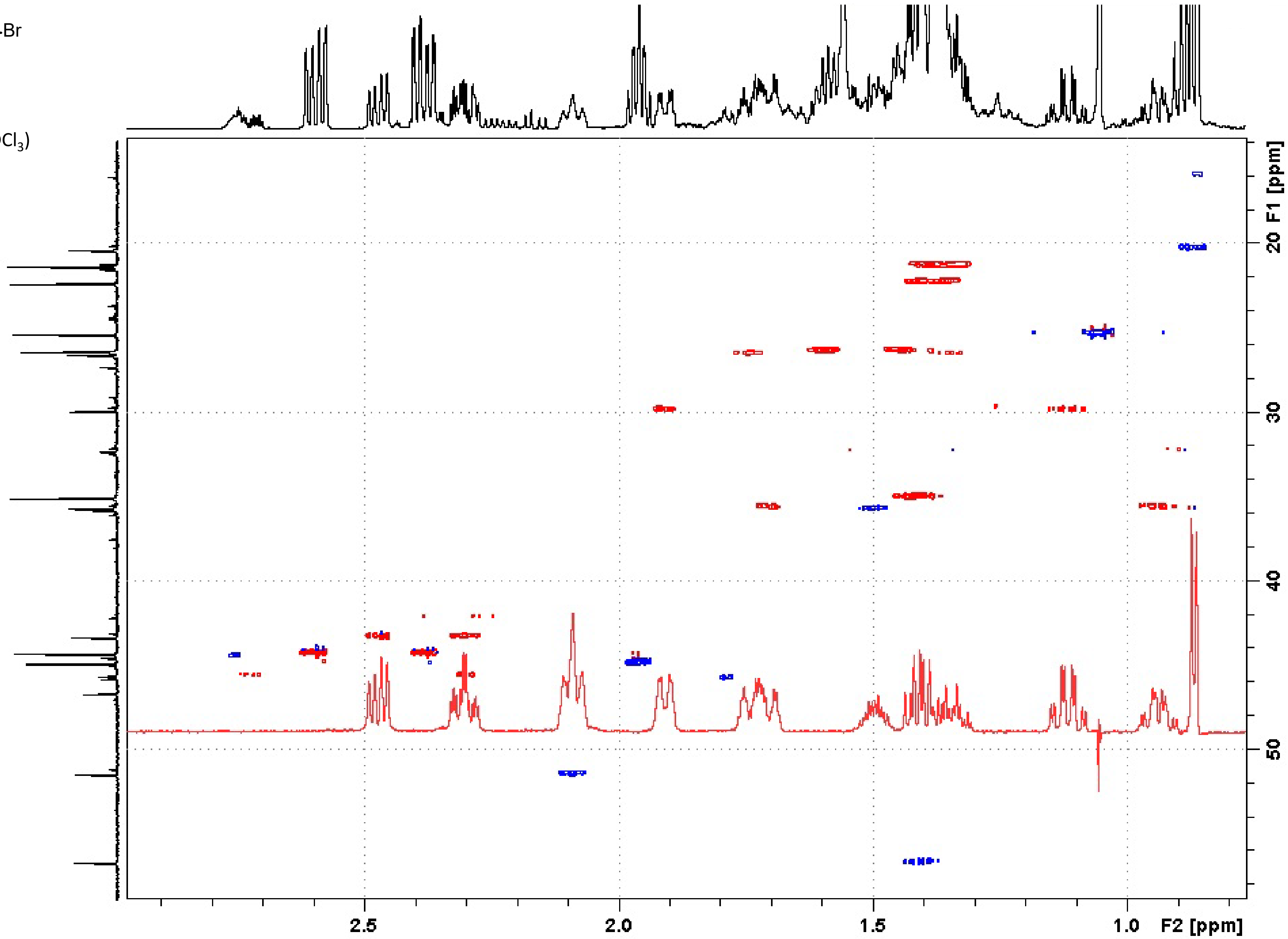

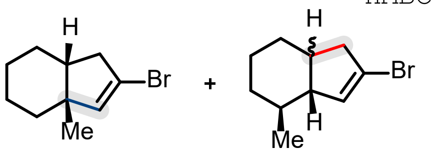

trans-4c trans-4c'

HMBC NMR([600, 150] MHz, $CDCl_3$)

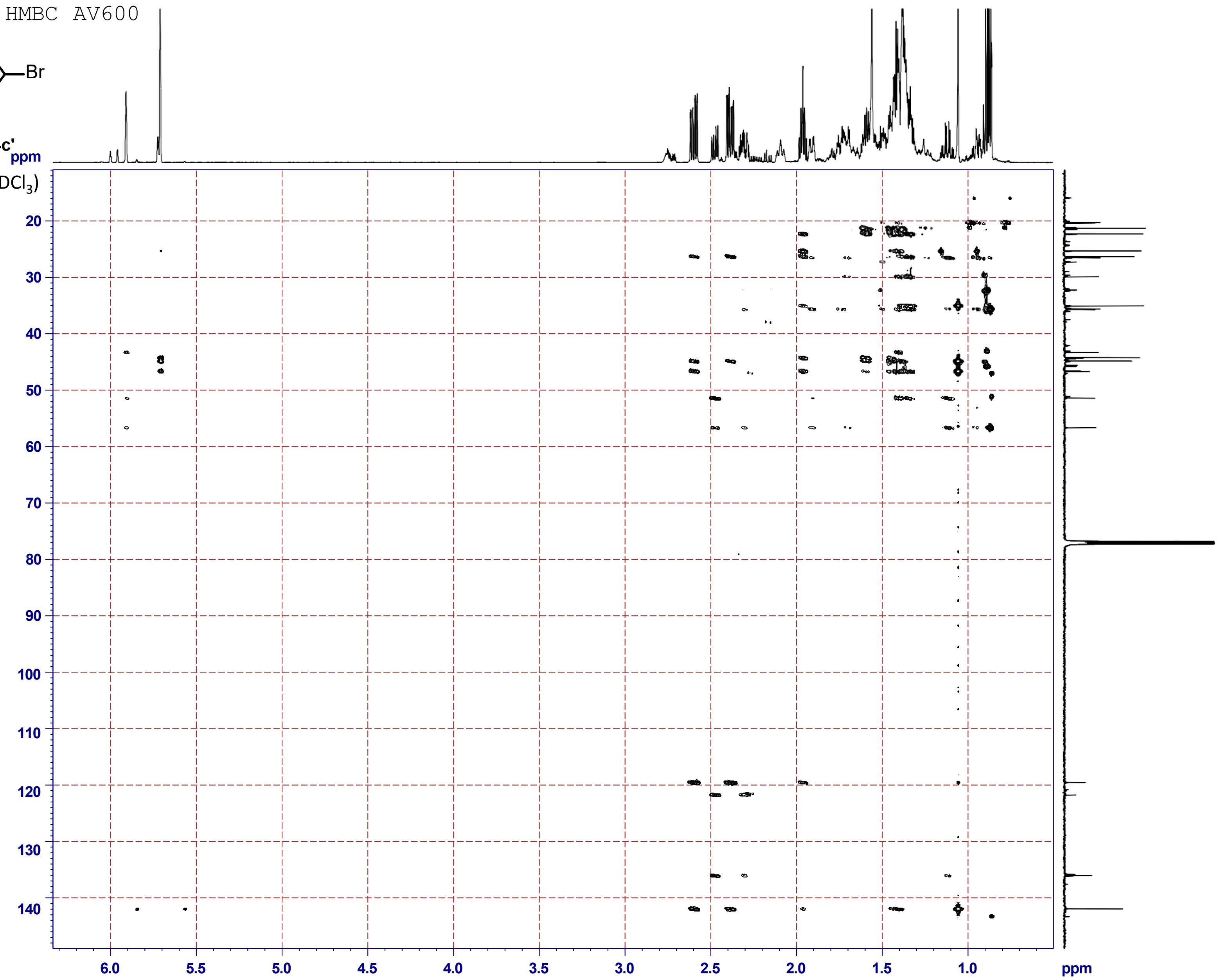

NAME	RM-1719F1
EXPNO	27
PROCNO	1
Date_	20240621
Time	0.49
INSTRUM	spect
PROBHD	5 mm PATXI 1H/
PULPROG	hmbcgplpndqf
TD	2048
SOLVENT	CDCl3
NS	20
DS	2
SWH	3501.401 Hz
FIDRES	1.709668 Hz
AQ	0.2926472 se
RG	26000
DW	142.800 us
DE	6.00 us
TE	298.2 K
CNST2	148.0000000
CNST13	8.0000000
D0	0.00000300 se
D1	1.00000000 se
D2	0.00337838 se
D6	0.06250000 se
D16	0.00015000 se
IN0	0.00002400 se

======== CHANNEL f1 ======

NUC1	1H
P1	8.60 us
P2	17.20 us
PL1	2.00 dB
PL1W	15.84893227 W
SFO1	600.1520674 MH

======== CHANNEL f2 ======

NUC2	13C
P3	12.50 us
PL2	-3.00 dB
PL2W	150.35617065 W
SFO2	150.9199024 MH

====== GRADIENT CHANNEL ==

GPNAM1	SINE.10
GPNAM2	0
GPNAM3	SINE.10
	0
	SINE.10
	0
GPZ1	50.00 %
GPZ2	30.00 %
GPZ3	40.10 %
P16	1000.00 us
ND0	2
TD	400
SFO1	150.9199 MH
FIDRES	52.082458 Hz
SW	138.040 pp
FnMODE	QF
SI	1024
SF	600.1500154 MH
WDW	SINE
SSB	0
LB	0.00 Hz
GB	0
PC	1.00
SI	1024
MC2	QF
SF	150.9078409 MH
WDW	SINE
SSB	0
LB	0.00 Hz
GB	0

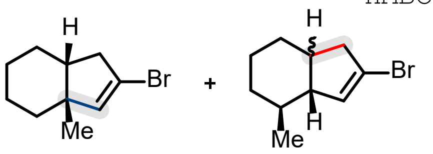

trans-4c + trans-4c'

HMBC NMR([600, 150] MHz, $CDCl_3$)

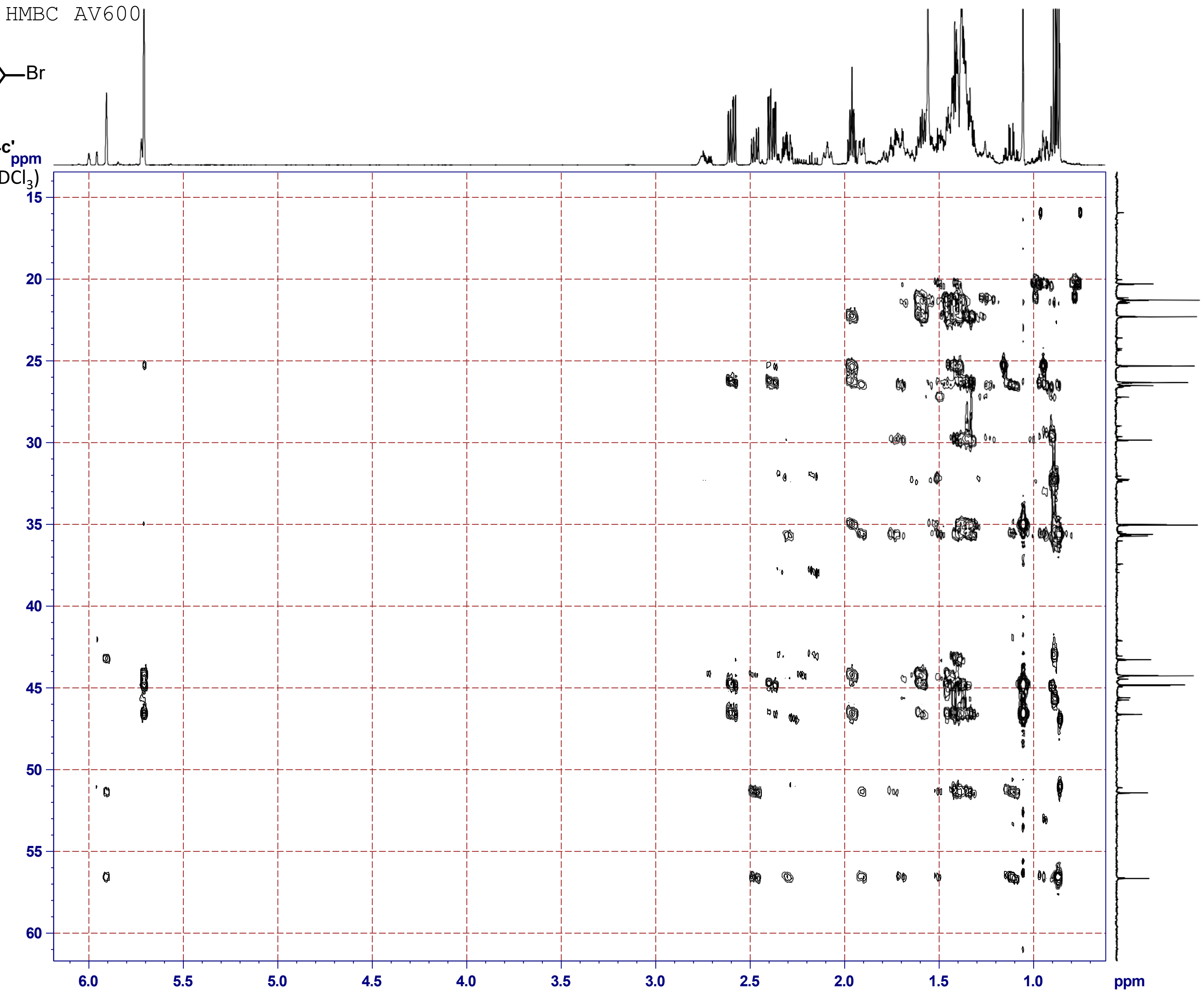

BRUKER

```
NAME          RM-1719F1
EXPNO                27
PROCNO                1
Date_          20240621
Time               0.49
INSTRUM           spect
PROBHD   5 mm PATXI 1H/
PULPROG    hmbcgplpndqf
TD                 2048
SOLVENT           CDCl3
NS                   20
DS                    2
SWH            3501.401 Hz
FIDRES         1.709668 Hz
AQ            0.2926472 se
RG                26000
DW              142.800 us
DE                 6.00 us
TE                298.2 K
CNST2       148.0000000
CNST13        8.0000000
D0           0.00000300 se
D1           1.00000000 se
D2           0.00337838 se
D6           0.06250000 se
D16          0.00015000 se
IN0          0.00002400 se
======== CHANNEL f1 ======
NUC1                 1H
P1                 8.60 us
P2                17.20 us
PL1                2.00 dB
PL1W        15.84893227 W
SFO1        600.1520674 MH
======== CHANNEL f2 ======
NUC2                13C
P3                12.50 us
PL2               -3.00 dB
PL2W       150.35617065 W
SFO2        150.9199024 MH
====== GRADIENT CHANNEL ==
GPNAM          SINE.10
1              0
GPNAM          SINE.10
2              0
GPNAM          SINE.10
3              0
GPZ1              50.00 %
GPZ2              30.00 %
[illegible]
SI                 1024
SF          600.1500154 MH
WDW                SINE
SSB                   0
LB                 0.00 Hz
GB                    0
PC                 1.00
SI                 1024
MC2                  QF
SF          150.9078409 MH
WDW                SINE
SSB                   0
LB                 0.00 Hz
```

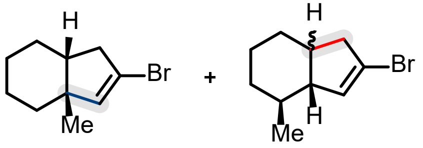

trans-4c trans-4c'

HMBC NMR([600, 150] MHz, $CDCl_3$)

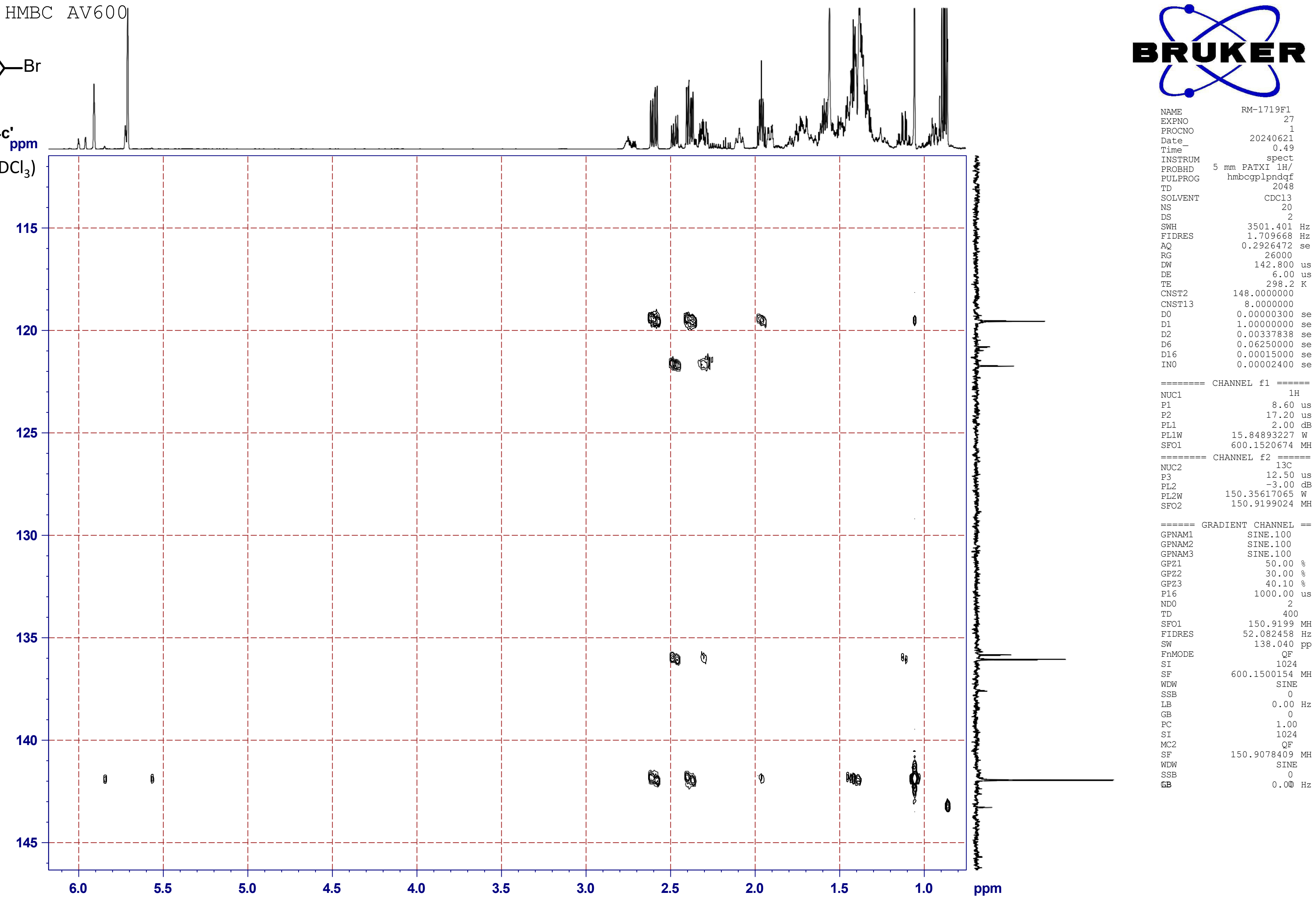

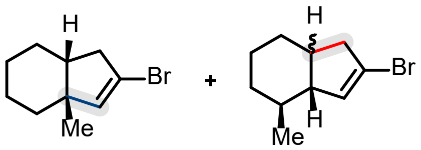

trans-4c + **trans-4c'**

NOESY NMR([600, 600] MHz, $CDCl_3$)

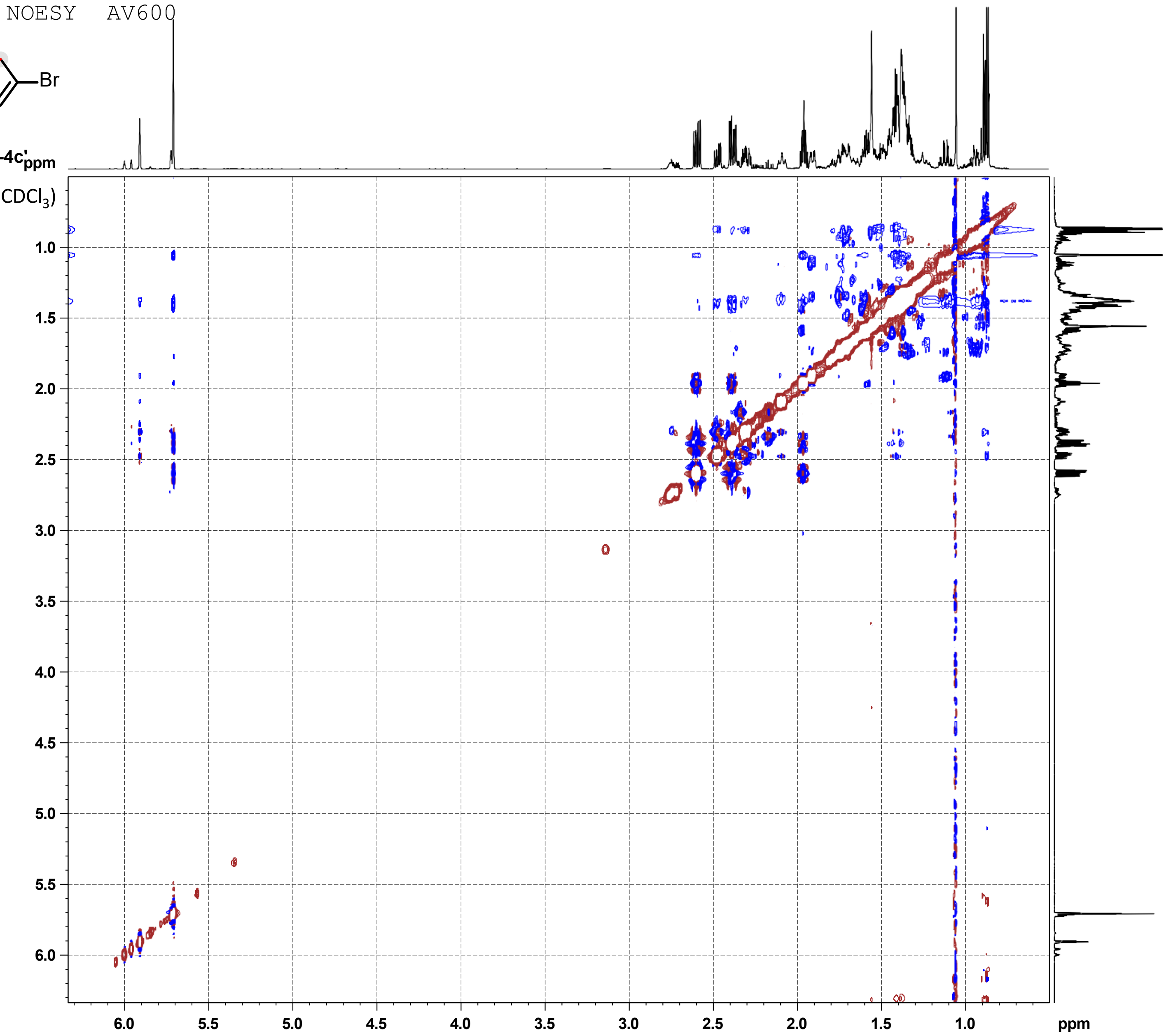


```
NAME          RM-1719F1
EXPNO                28
PROCNO                1
Date_          20240621
Time               3.53
INSTRUM           spect
PROBHD   5 mm PATXI 1H/
PULPROG       noesygpph
TD                 2048
SOLVENT           CDCl3
NS                   24
DS                    8
SWH          3501.401 Hz
FIDRES       1.709668 Hz
AQ          0.2926472 sec
RG                  322
DW            142.800 usec
DE              6.00 usec
TE              298.2 K
D0        0.00013185 sec
D1        1.00000000 sec
D8        0.50000000 sec
D16       0.00015000 sec
IN0       0.00028560 sec

======== CHANNEL f1 ========
NUC1                 1H
P1                 8.60 usec
P2                17.20 usec
PL1                2.00 dB
PL1W        15.84893227 W
SFO1        600.1520674 MHz

====== GRADIENT CHANNEL =====
GPNAM1         SINE.100
GPZ1              40.00 %
P16             1000.00 usec
ND0                   1
TD                  256
SFO1           600.1521 MHz
FIDRES        13.677372 Hz
SW                5.834 ppm
FnMODE       States-TPPI
SI                 2048
SF          600.1500154 MHz
WDW               QSINE
SSB                   2
LB                 0.00 Hz

GB                    0
PC                 1.00
SI                 1024
MC2          States-TPPI
SF          600.1500154 MHz
WDW               QSINE
SSB                   2
LB                 0.00 Hz
GB                    0
```

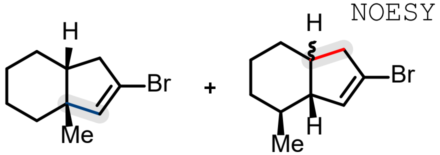

trans-4c + trans-4c'

NOESY NMR([600, 600] MHz, $CDCl_3$)

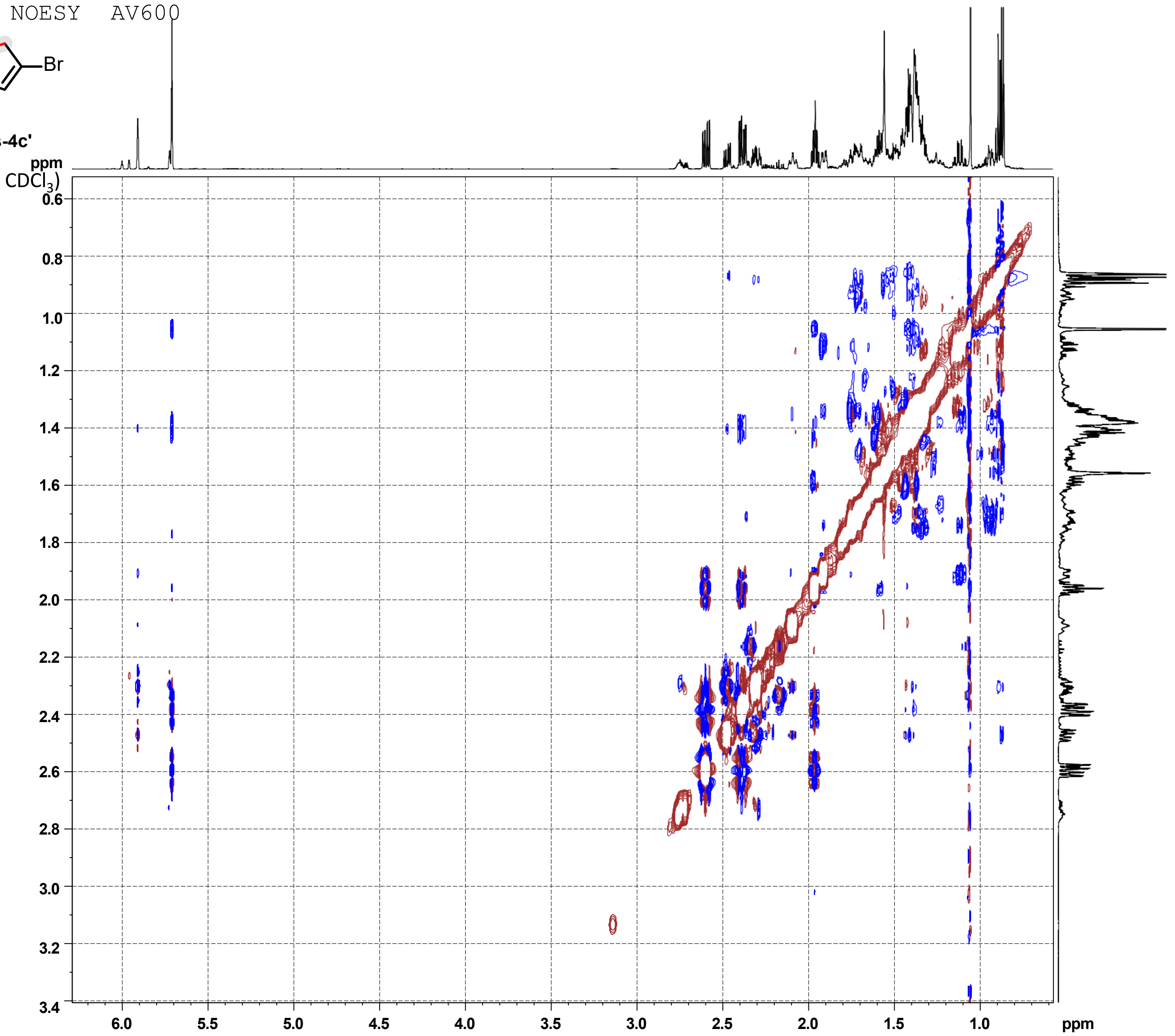

BRUKER

NAME RM-1719F1
EXPNO 28
PROCNO 1
Date_ 20240621
Time 3.53
INSTRUM spect
PROBHD 5 mm PATXI 1H/
PULPROG noesygpph
TD 2048
SOLVENT CDCl3
NS 24
DS 8
SWH 3501.401 Hz
FIDRES 1.709668 Hz
AQ 0.2926472 sec
RG 322
DW 142.800 usec
DE 6.00 usec
TE 298.2 K
D0 0.00013185 sec
D1 1.00000000 sec
D8 0.50000000 sec
D16 0.00015000 sec
IN0 0.00028560 sec

======== CHANNEL f1 ========
NUC1 1H
P1 8.60 usec
P2 17.20 usec
PL1 2.00 dB
PL1W 15.84893227 W
SFO1 600.1520674 MHz

====== GRADIENT CHANNEL =====
GPNAM1 SINE.100
GPZ1 40.00 %
P16 1000.00 usec
ND0 1
TD 256
SFO1 600.1521 MHz
FIDRES 13.677372 Hz
SW 5.834 ppm
FnMODE States-TPPI
SI 2048
SF 600.1500154 MHz
WDW QSINE
SSB 2
LB 0.00 Hz
GB 0
PC 1.00
SI 1024
MC2 States-TPPI
SF 600.1500154 MHz
WDW QSINE
SSB 2
LB 0.00 Hz
GB 0

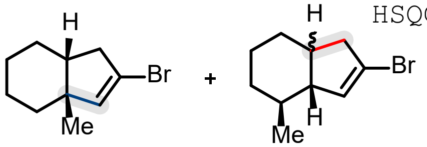

trans-4c **trans-4c'**

HSQC NMR([600, 150] MHz, $CDCl_3$)

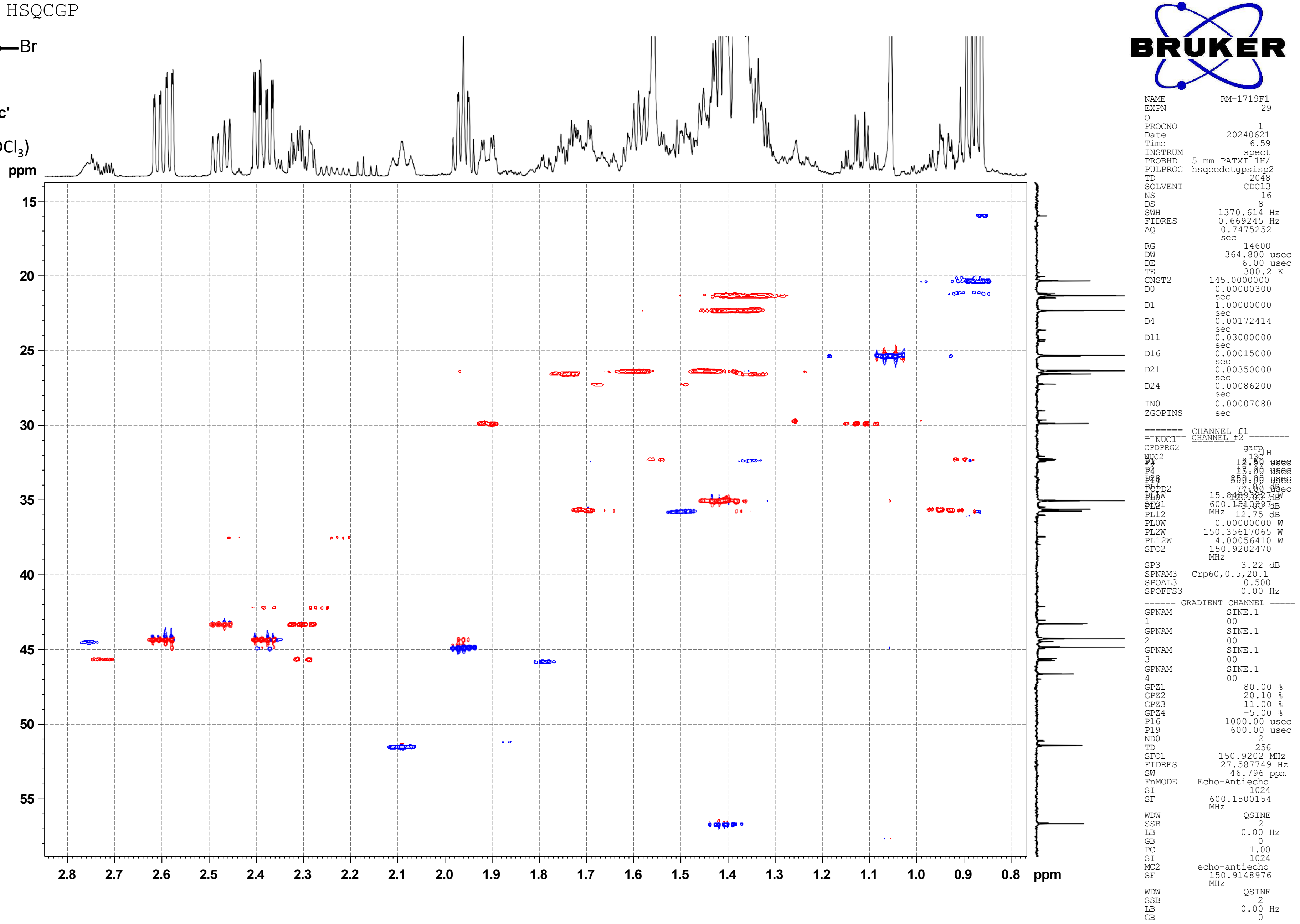

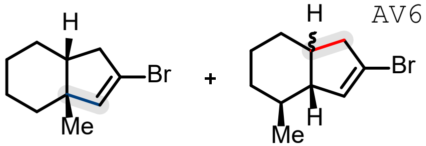

trans-4c **trans-4c'**

HSQC NMR([600, 150] MHz, $CDCl_3$)

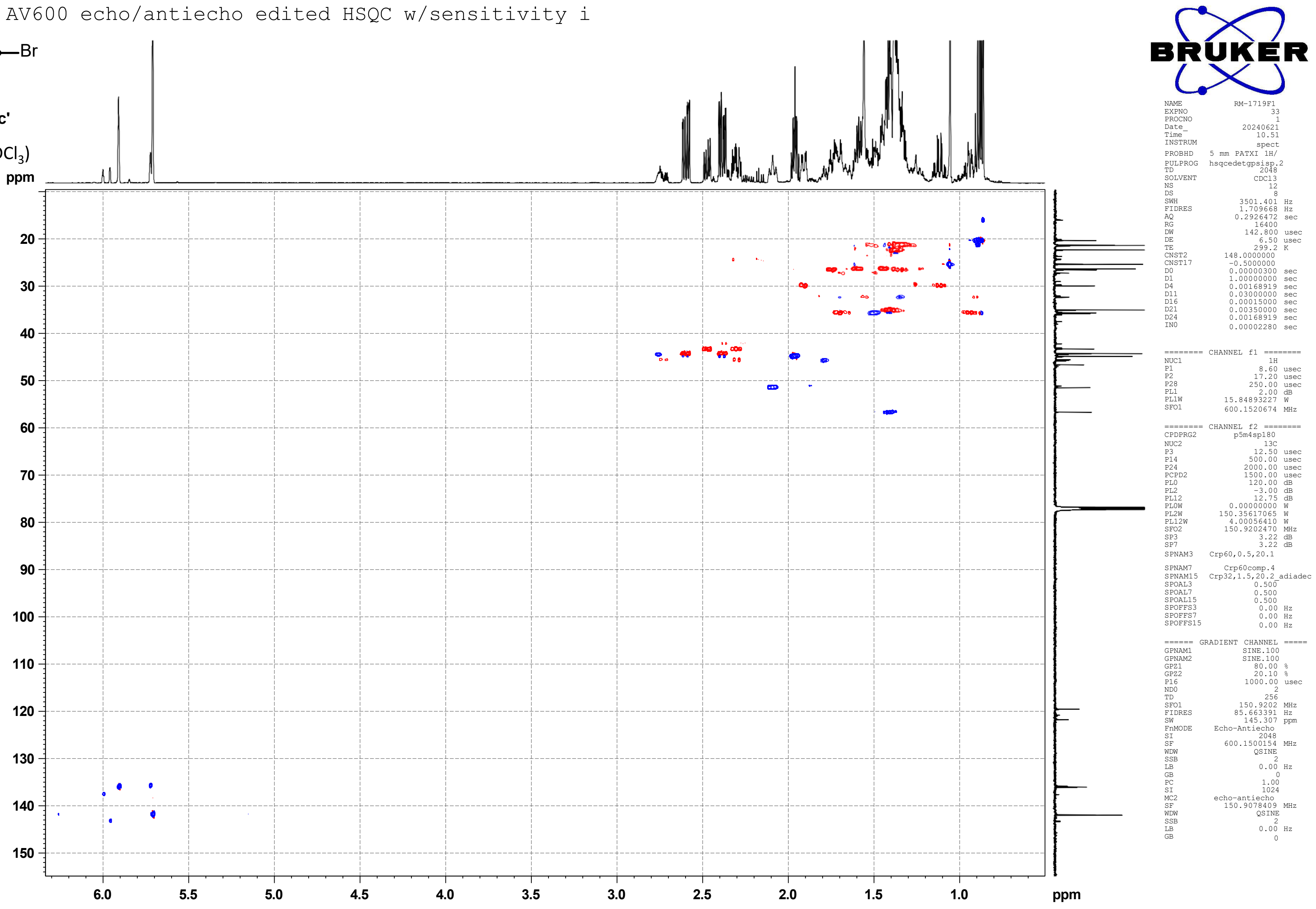

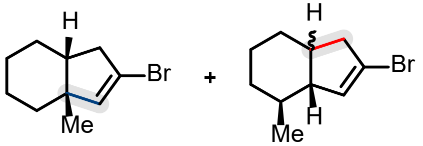

HSQC NMR([600, 150] MHz, $CDCl_3$)

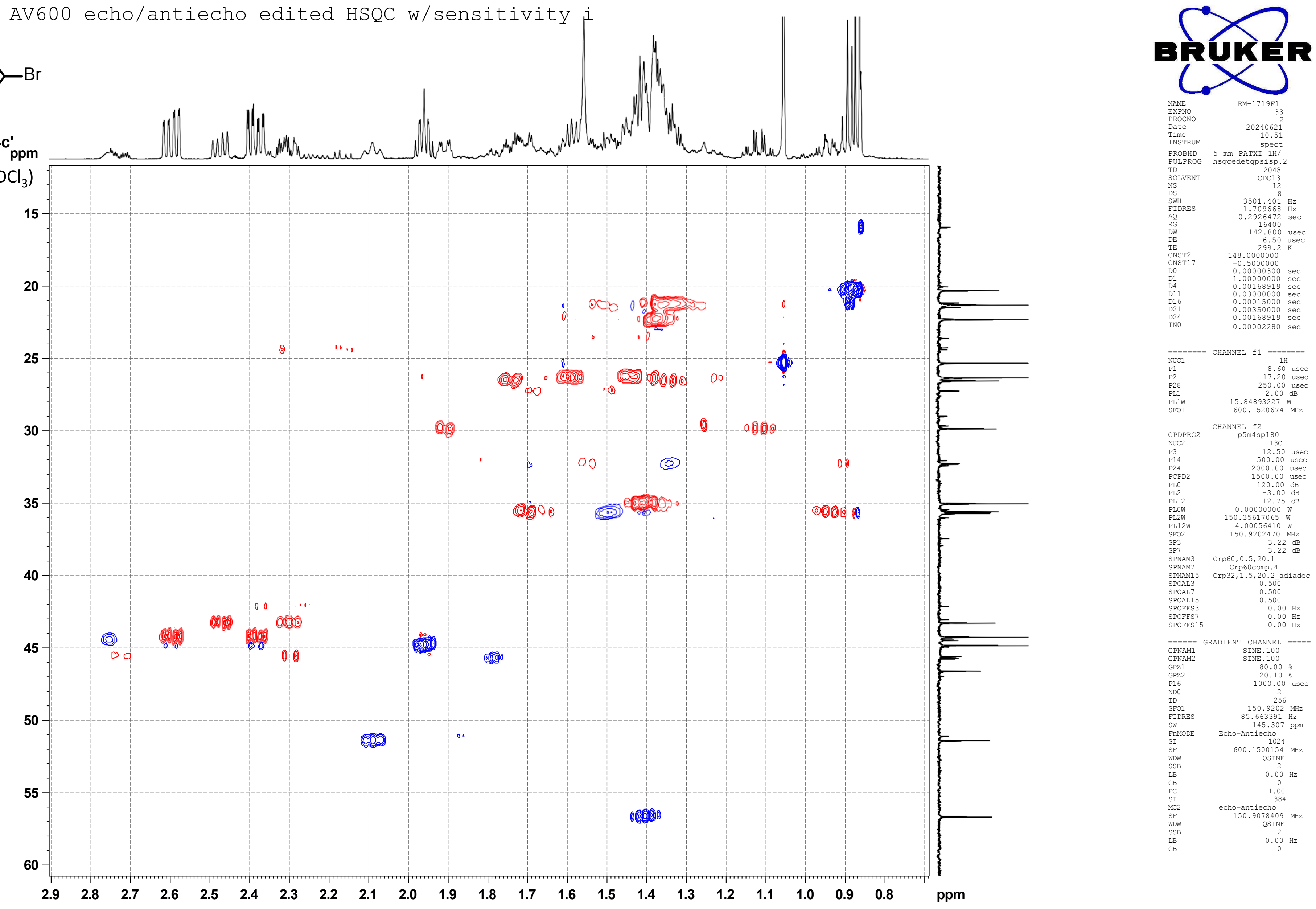

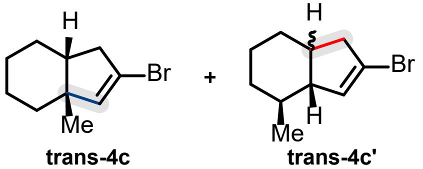

HSQC NMR([600, 150] MHz, $CDCl_3$)

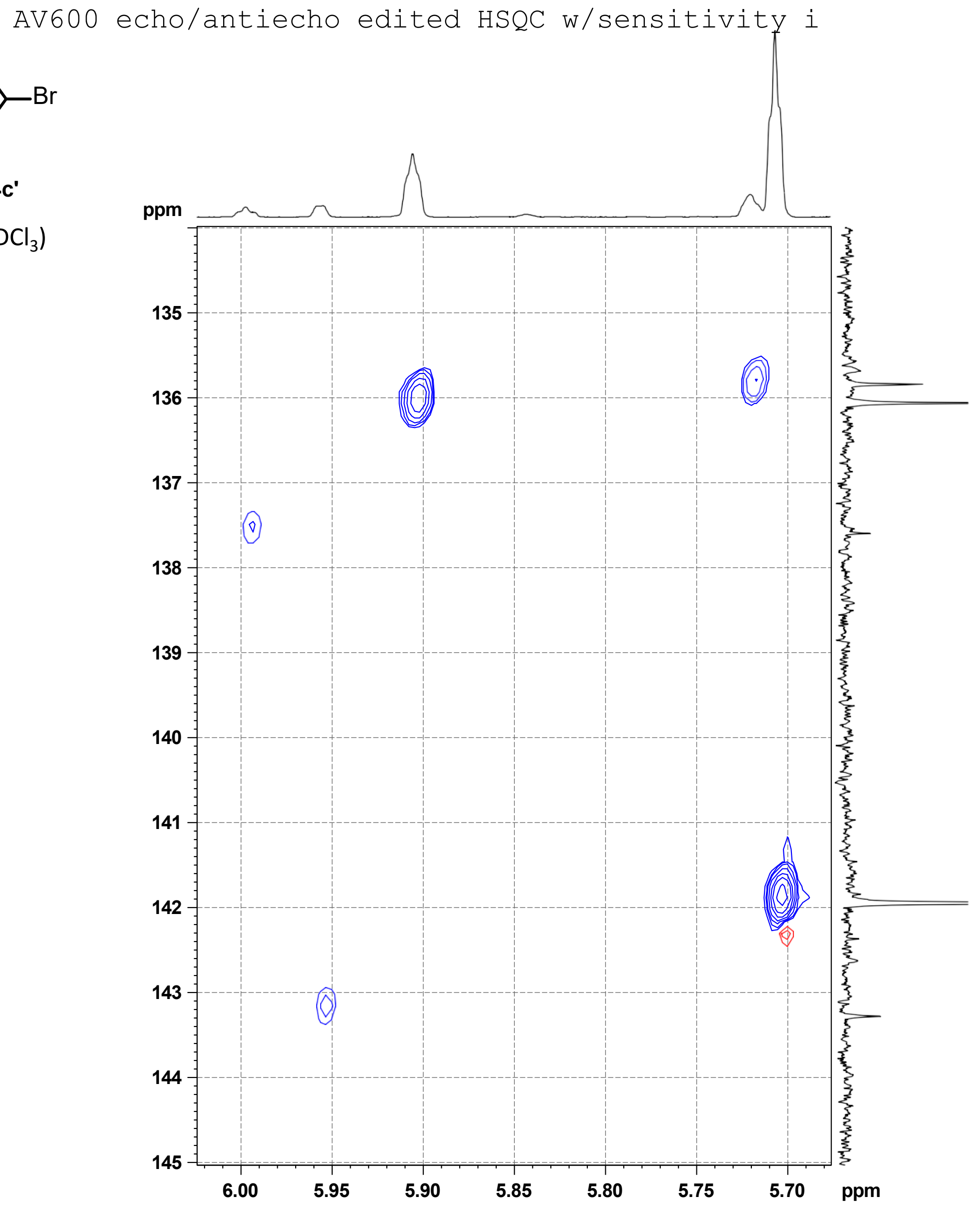


```
NAME            RM-1719F1
EXPNO                  33
PROCNO                  3
Date_           20240621
Time               10.51
INSTRUM            spect
PROBHD    5 mm PATXI 1H/
PULPROG   hsqcedetgpsisp.2
TD                  2048
SOLVENT            CDCl3
NS                    12
DS                     8
SWH             3501.401 Hz
FIDRES          1.709668 Hz
AQ             0.2926472 sec
RG                 16400
DW               142.800 usec
DE                  6.50 usec
TE                 299.2 K
CNST2        148.0000000
CNST17        -0.5000000
D0            0.00000300 sec
D1            1.00000000 sec
D4            0.00168919 sec
D11           0.03000000 sec
D16           0.00015000 sec
D21           0.00350000 sec
D24           0.00168919 sec
IN0           0.00002280 sec

======== CHANNEL f1 ========
NUC1                  1H
P1                  8.60 usec
P2                 17.20 usec
P28               250.00 usec
PL1                 2.00 dB
PL1W         15.84893227 W
SFO1         600.1520674 MHz

======== CHANNEL f2 ========
CPDPRG2        p5m4sp180
NUC2                 13C
P3                 12.50 usec
P14               500.00 usec
P24              2000.00 usec
PCPD2            1500.00 usec
PL0               120.00 dB
PL2                -3.00 dB
PL12               12.75 dB
PL0W          0.00000000 W
PL2W        150.35617065 W
PL12W         4.00056410 W
SFO2         150.9202470 MHz
SP3                 3.22 dB
SP7                 3.22 dB
SPNAM3    Crp60,0.5,20.1
SPNAM7      Crp60comp.4
SPNAM15   Crp32,1.5,20.2_adiadec
SPOAL3             0.500
SPOAL7             0.500
SPOAL15            0.500
SPOFFS3             0.00 Hz
SPOFFS7             0.00 Hz
SPOFFS15            0.00 Hz

====== GRADIENT CHANNEL =====
GPNAM1          SINE.100
GPNAM2          SINE.100
GPZ1               80.00 %
GPZ2               20.10 %
P16              1000.00 usec
ND0                    2
TD                   256
SFO1            150.9202 MHz
FIDRES         85.663391 Hz
SW               145.307 ppm
FnMODE     Echo-Antiecho
SI                  2048
SF           600.1500154 MHz
WDW                QSINE
SSB                    2
LB                  0.00 Hz
GB                     0
PC                  1.00
SI                  1024
MC2        echo-antiecho
SF           150.9078409 MHz
WDW                QSINE
SSB                    2
LB                  0.00 Hz
GB                     0
```

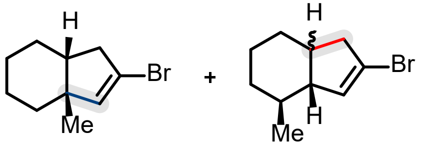

trans-4c + trans-4c'

HSQC NMR([600, 150] MHz, $CDCl_3$)

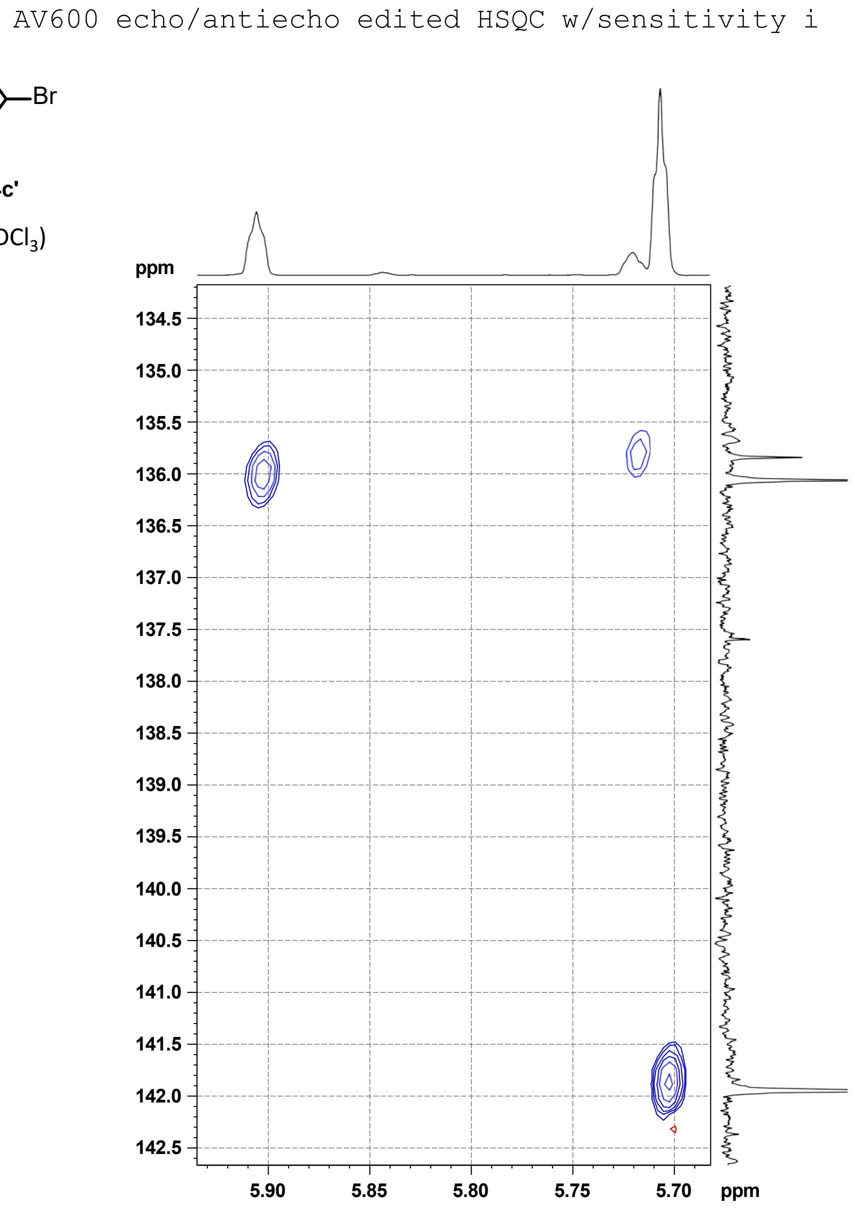


```
NAME          RM-1719F1
EXPNO                33
PROCNO                3
Date_          20240621
Time              10.51
INSTRUM           spect
PROBHD    5 mm PATXI 1H/
PULPROG   hsqcedetgpsisp.2
TD                 2048
SOLVENT           CDCl3
NS                   12
DS                    8
SWH            3501.401 Hz
FIDRES         1.709668 Hz
AQ            0.2926472 sec
RG                16400
DW              142.800 usec
DE                 6.50 usec
TE                299.2 K
CNST2       148.0000000
CNST17       -0.5000000
D0           0.00000300 sec
D1           1.00000000 sec
D4           0.00168919 sec
D11          0.03000000 sec
D16          0.00015000 sec
D21          0.00350000 sec
D24          0.00168919 sec
IN0          0.00002280 sec

======== CHANNEL f1 ========
NUC1                 1H
P1                 8.60 usec
P2                17.20 usec
P28              250.00 usec
PL1                2.00 dB
PL1W        15.84893227 W
SFO1        600.1520674 MHz

======== CHANNEL f2 ========
CPDPRG2        p5m4sp180
NUC2                13C
P3                12.50 usec
P14              500.00 usec
P24             2000.00 usec
PCPD2           1500.00 usec
PL0              120.00 dB
PL2               -3.00 dB
PL12              12.75 dB
PL0W         0.00000000 W
PL2W       150.35617065 W
PL12W        4.00056410 W
SFO2        150.9202470 MHz
SP3                3.22 dB
SP7                3.22 dB
SPNAM3    Crp60,0.5,20.1
SPNAM7       Crp60comp.4
SPNAM15   Crp32,1.5,20.2_adiadec
SPOAL3            0.500
SPOAL7            0.500
SPOAL15           0.500
SPOFFS3            0.00 Hz
SPOFFS7            0.00 Hz
SPOFFS15           0.00 Hz

====== GRADIENT CHANNEL =====
GPNAM1         SINE.100
GPNAM2         SINE.100
GPZ1              80.00 %
GPZ2              20.10 %
P16             1000.00 usec
ND0                   2
TD                  256
SFO1           150.9202 MHz
FIDRES        85.663391 Hz
SW              145.307 ppm
FnMODE     Echo-Antiecho
SI                 2048
SF          600.1500154 MHz
WDW               QSINE
SSB                   2
LB                 0.00 Hz
GB                    0
PC                 1.00
SI                 1024
MC2        echo-antiecho
SF          150.9078409 MHz
WDW               QSINE
SSB                   2
LB                 0.00 Hz
GB                    0
```

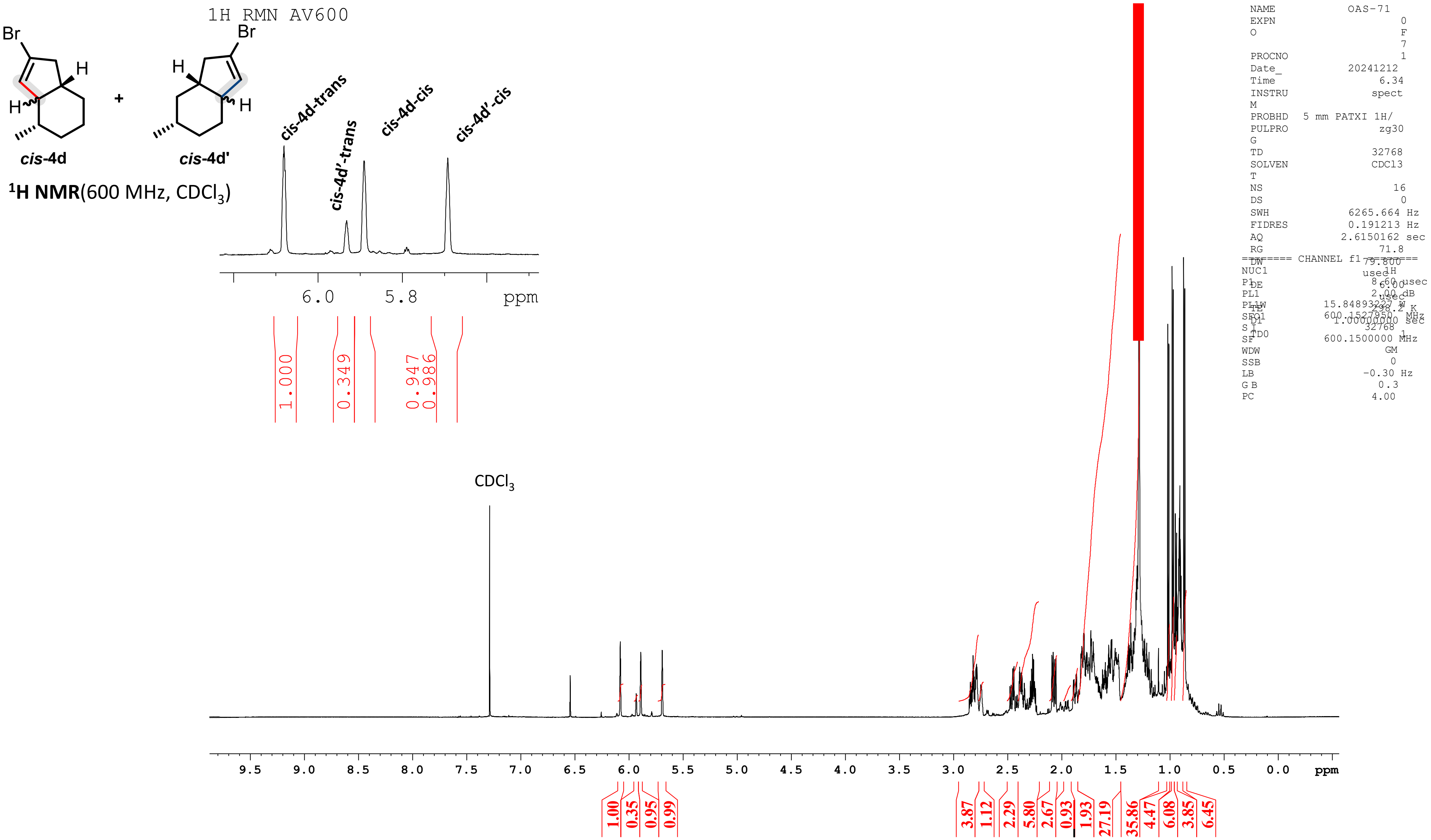

cis-4d
cis-4d'
^{1}H NMR(600 MHz, $CDCl_3$)
1H RMN AV600
cis-4d-trans
cis-4d'-trans
cis-4d-cis
cis-4d'-cis
$CDCl_3$
NAME OAS-71
PROCNO 1
Date_ 20241212
Time 6.34
INSTRUM spect
PROBHD 5 mm PATXI 1H/
PULPROG zg30
TD 32768
SOLVENT CDCl3
NS 16
DS 0
SWH 6265.664 Hz
FIDRES 0.191213 Hz
AQ 2.6150162 sec
RG 71.8
SF 600.1500000 MHz
WDW GM
SSB 0
LB -0.30 Hz
GB 0.3
PC 4.00
ppm

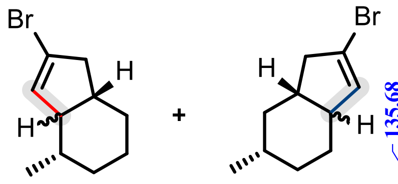

cis-**4d** *cis*-**4d'**

^{13}C NMR (150 MHz, $CDCl_3$)

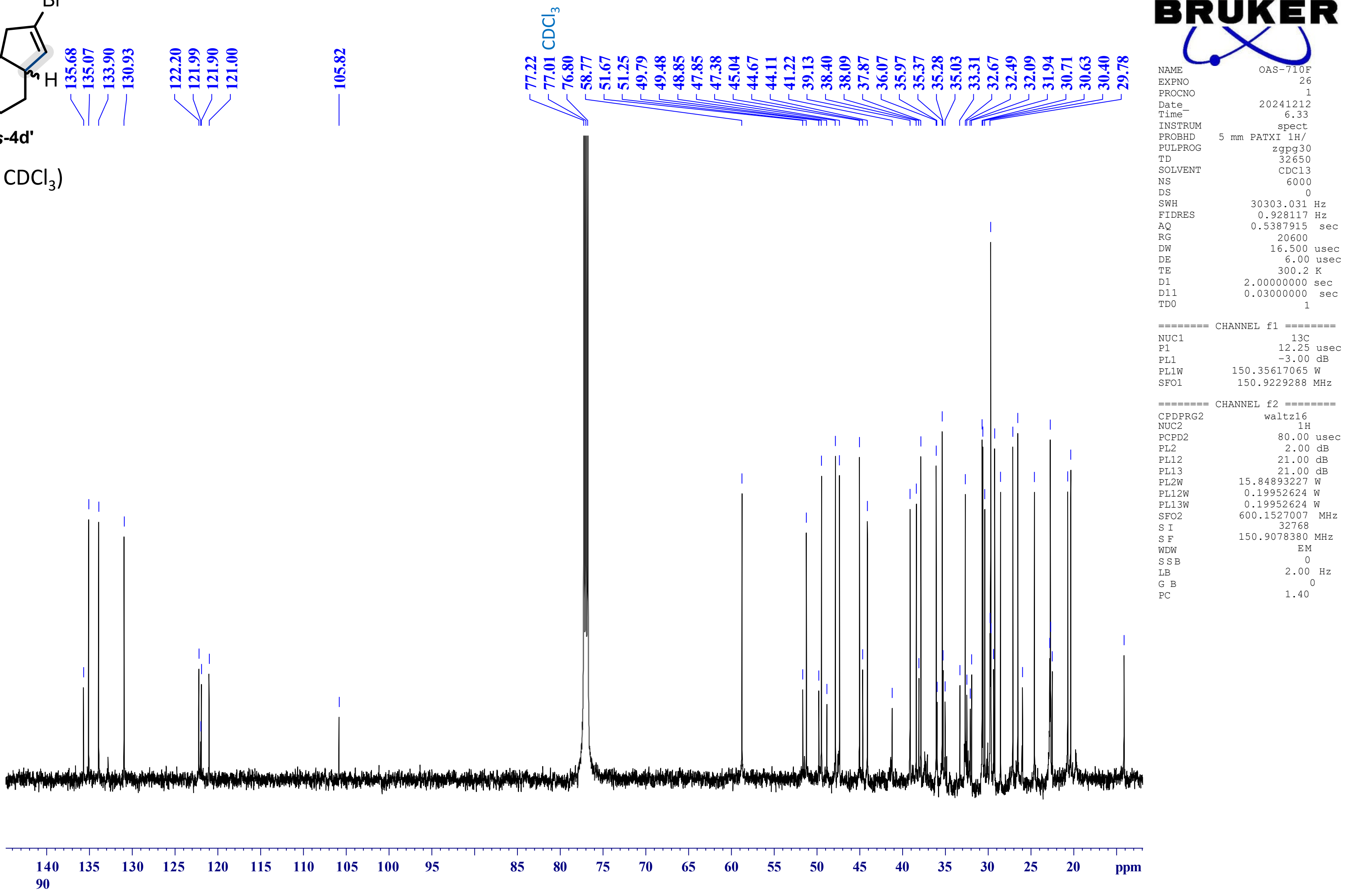

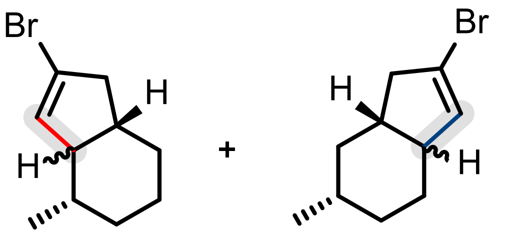

cis-4d *cis*-4d'

DEPT-135 NMR (150 MHz, $CDCl_3$)

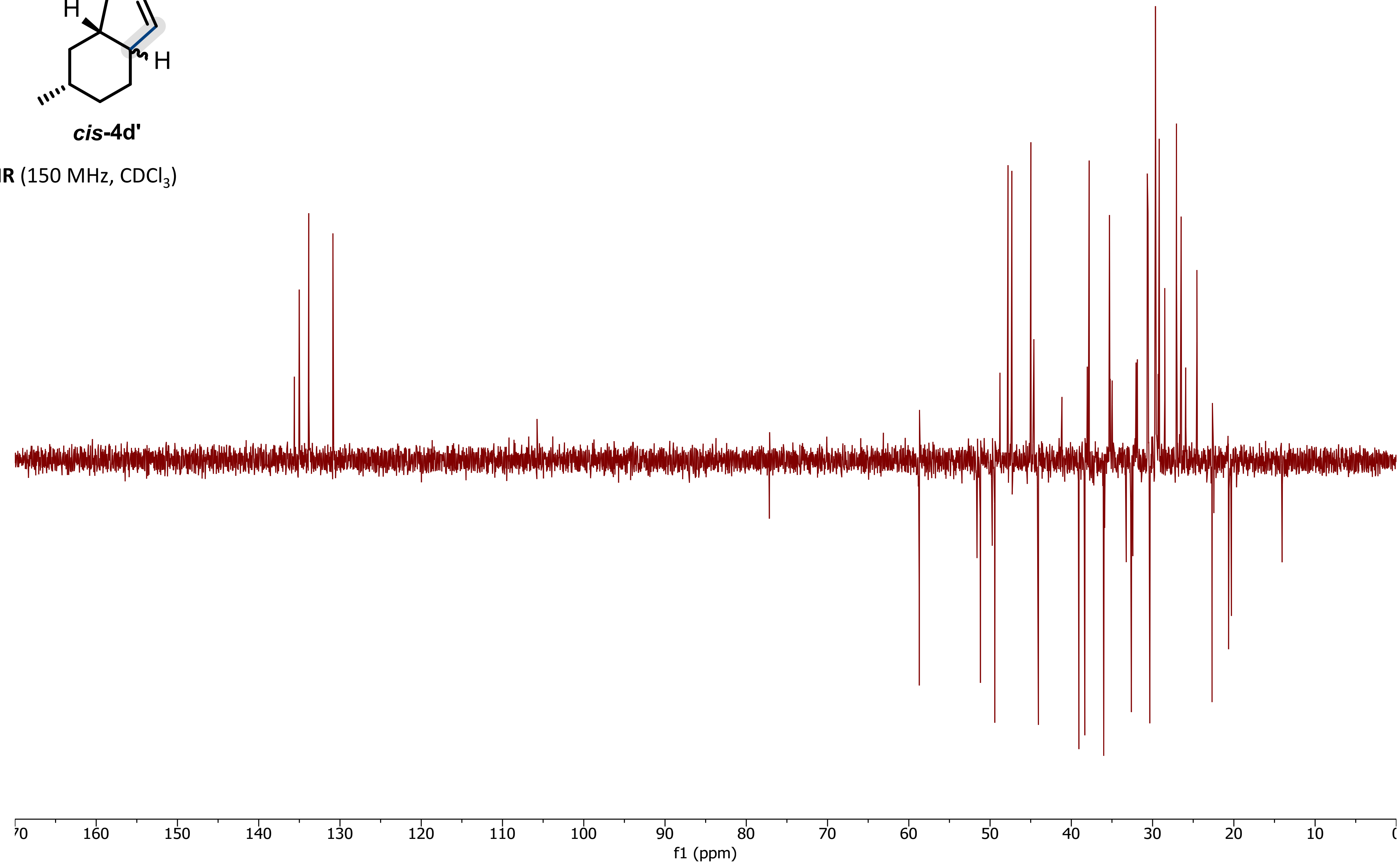

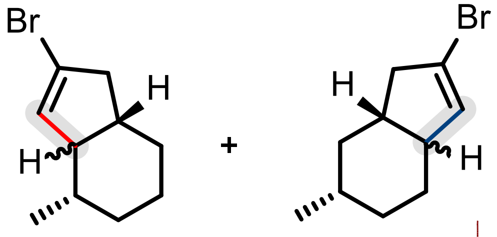

***cis*-4d** ***cis*-4d'**

COSY NMR([600, 600] MHz, $CDCl_3$)

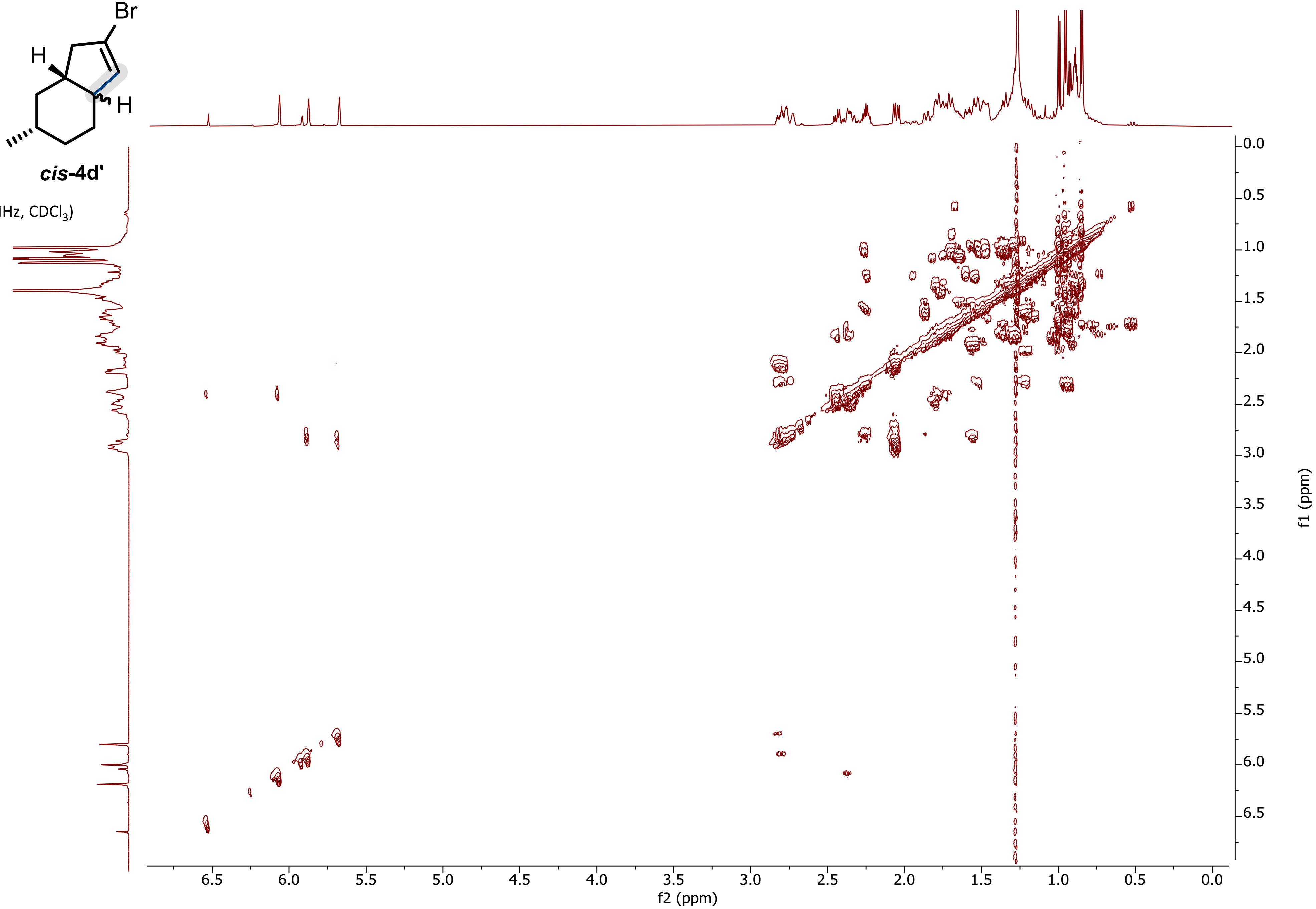

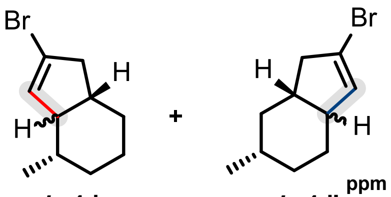

cis-4d + *cis*-4d'

HSQC NMR([600, 150] MHz, $CDCl_3$)

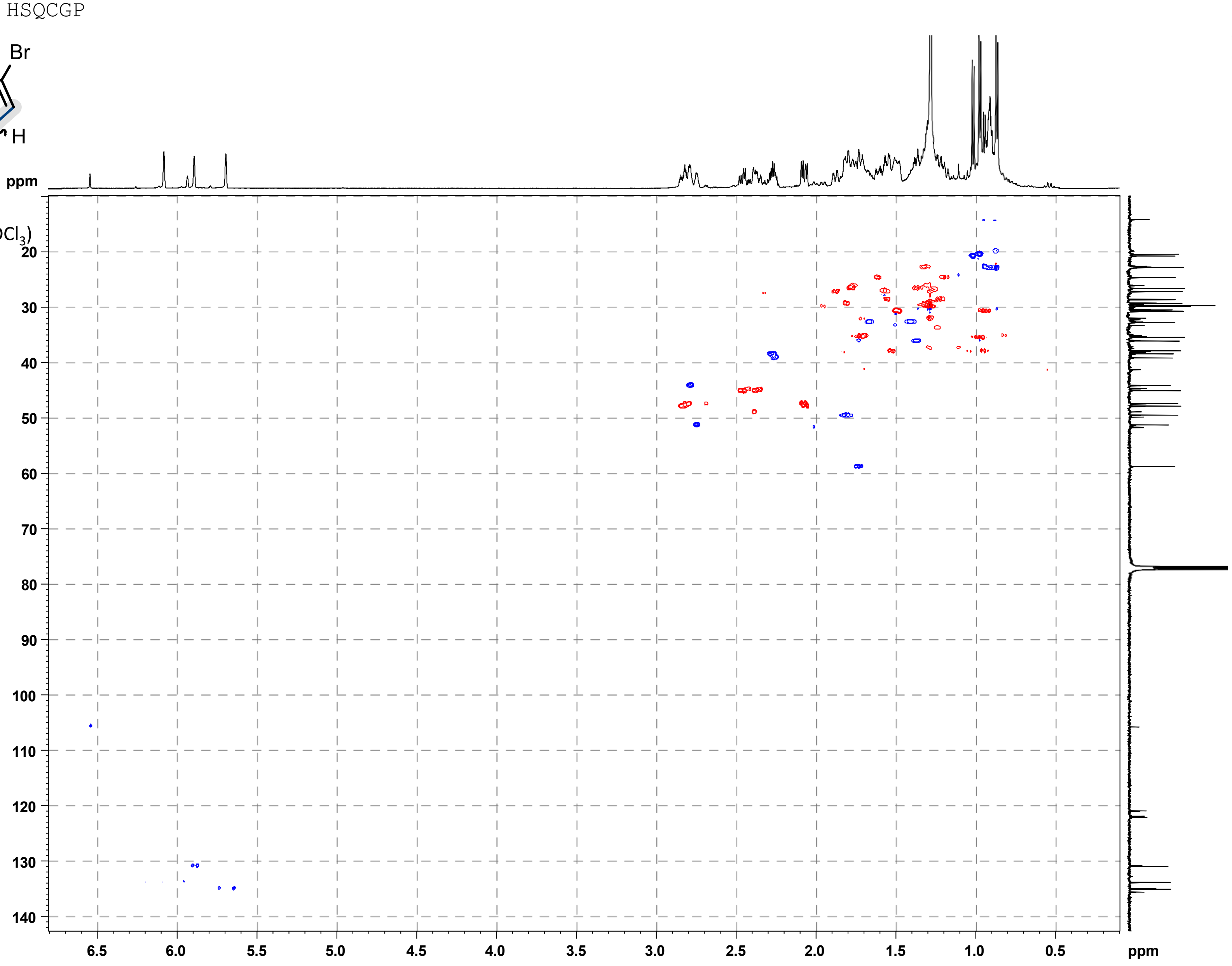


```
NAME                OAS-710F
EXPNO
4
PROCNO                     1
Date_               20241211
Time                   22.47
INSTRUM                spect
PROBHD    5 mm PATXI 1H/
PULPROG
hsqcedetgpsisp2 TD
2048
SOLVENT                CDCl3
NS                        12
DS                         8
SWH                 4222.973 Hz
FIDRES              2.061999 Hz
AQ                 0.2426516 sec
RG                     14600
DW                   118.400 usec
DE                      6.00 usec
TE                     299.2 K
CNST2            145.0000000
D0                0.00000300 sec
D1                1.00000000 sec
D4                0.00172414 sec
D11               0.03000000 sec
D16               0.00015000 sec
D21               0.00350000 sec
D24               0.00086200 sec
IN0               0.00002210 sec

ZGOPTNS

======== CHANNEL f1 ========
NUC1                      1H
P1                      8.60 usec
P2                     17.20 usec
P28                   250.00 usec
PL1                     2.00 dB
PL1W             15.84893227 W
SFO1             600.1520450 MHz

======== CHANNEL f2 ========
CPDPRG2                 garp
NUC2                     13C
P3                     12.50 usec
P4                     25.00 usec
P14                   500.00 usec
PCPD2                  77.00 usec
PL0                   120.00 dB
PL2                    -3.00 dB
PL12                   12.75 dB
PL0W              0.00000000 W
PL2W            150.35617065 W
PL12W             4.00056410 W
SFO2             150.9191561 MHz
SP3                     3.22 dB
SPNAM3     Crp60,0.5,20.1
SPOAL3                 0.500
SPOFFS3                 0.00 Hz

====== GRADIENT CHANNEL =====
GPNAM1             SINE.100
GPNAM2             SINE.100
GPNAM3             SINE.100
GPNAM4             SINE.100
GPZ1                   80.00 %
GPZ2                   20.10 %
GPZ3                   11.00 %
GPZ4                   -5.00 %
P16                  1000.00
usec P19
600.00 usec ND0
2
TD                       280
SFO1                150.9192
MHz FIDRES
80.849548 Hz SW
150.000 ppm FnMODE
Echo-Antiecho
SI                      1024
SF               600.1500000
MHz WDW                QSINE
SSB                        2
LB                      0.00 Hz
GB                         0
PC                      4.00
SI                      1024
MC2           echo-antiecho
SF               150.9078380
MHz WDW                QSINE
SSB                        2
LB                      0.00 Hz
GB                         0
```

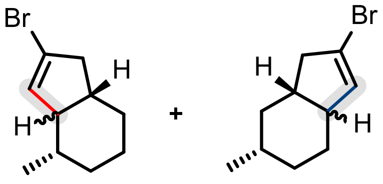

***cis*-4d** + ***cis*-4d'**

HSQC NMR([600, 150] MHz, $CDCl_3$)

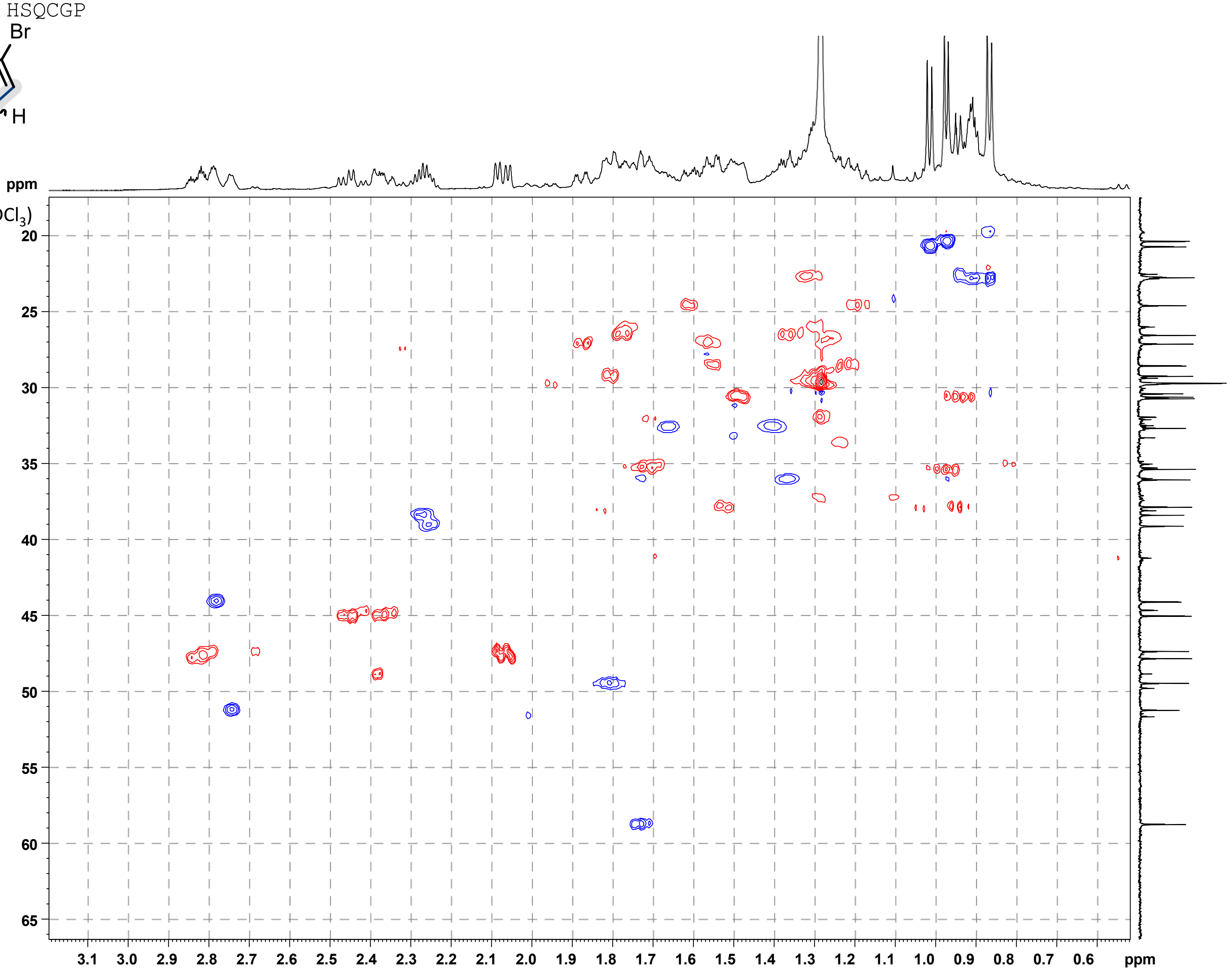


```
NAME              OAS-710F
EXPNO
4
PROCNO                   1
Date_             20241211
Time                 22.47
INSTRUM              spect
PROBHD   5 mm PATXI 1H/
PULPROG
hsqcedetgpsisp2 TD
2048
SOLVENT              CDCl3
NS                      12
DS                       8
SWH               4222.973 Hz
FIDRES            2.061999 Hz
AQ               0.2426516 sec
RG                   14600
DW                 118.400 usec
DE                    6.00 usec
TE                   299.2 K
CNST2        145.0000000
D0              0.00000300 sec
D1              1.00000000 sec
D4              0.00172414 sec
D11             0.03000000 sec
D16             0.00015000 sec
D21             0.00350000 sec
D24             0.00086200 sec
IN0             0.00002210 sec

ZGOPTNS

======== CHANNEL f1 ========
NUC1                    1H
P1                    8.60 usec
P2                   17.20 usec
P28                 250.00 usec
PL1                   2.00 dB
PL1W           15.84893227 W
SFO1           600.1520450 MHz

======== CHANNEL f2 ========
CPDPRG2               garp
NUC2                   13C
P3                   12.50 usec
P4                   25.00 usec
P14                 500.00 usec
PCPD2                77.00 usec
PL0                 120.00 dB
PL2                  -3.00 dB
PL12                 12.75 dB
PL0W            0.00000000 W
PL2W          150.35617065 W
PL12W           4.00056410 W
SFO2           150.9191561 MHz
SP3                   3.22 dB
SPNAM3    Crp60,0.5,20.1
SPOAL3               0.500
SPOFFS3               0.00 Hz

====== GRADIENT CHANNEL =====
GPNAM1            SINE.100
GPNAM2            SINE.100
GPNAM3            SINE.100
GPNAM4            SINE.100
GPZ1                 80.00 %
GPZ2                 20.10 %
GPZ3                 11.00 %
GPZ4                 -5.00 %
P16                1000.00
usec P19
600.00 usec ND0
2
TD                     280
SFO1              150.9192
MHz FIDRES
80.849548 Hz SW
150.000 ppm FnMODE
Echo-Antiecho
SI                    1024
SF             600.1500000
MHz WDW              QSINE
SSB                      2
LB                    0.00 Hz
GB                       0
PC                    4.00
SI                    1024
MC2         echo-antiecho
SF             150.9078380
MHz WDW              QSINE
SSB                      2
LB                    0.00 Hz
GB                       0
```

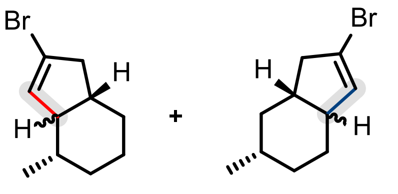

***cis*-4d** ***cis*-4d'**

HSQC NMR([600, 150] MHz, $CDCl_3$)

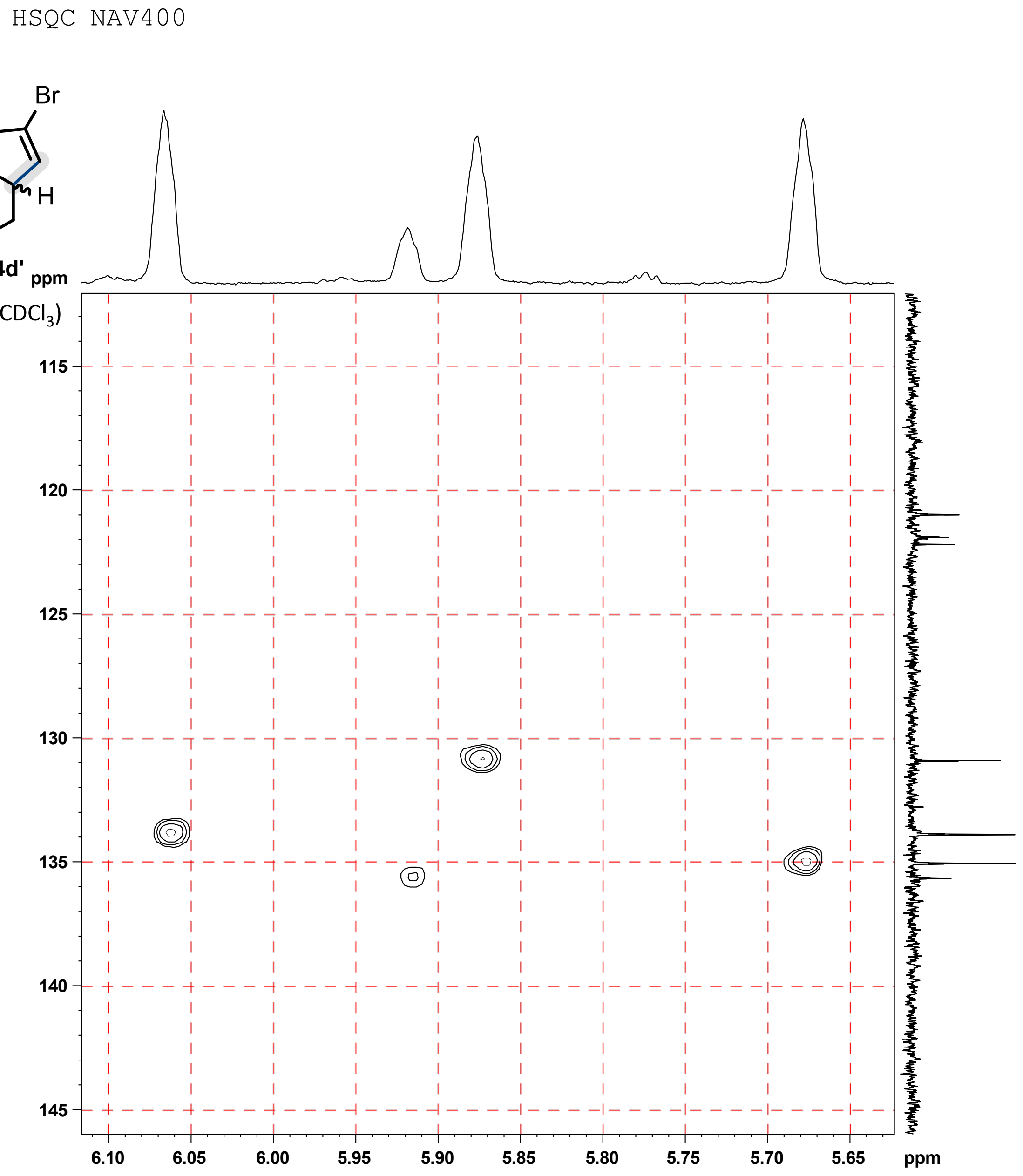


```
NAME              OAS710F
EXPNO                   6
PROCNO                  1
Date_            20241130
Time                 6.02
INSTRUM             spect
PROBHD   5 mm TBO
BB-1H PULPROG
hsqcetgpsisp TD
2048
SOLVENT             CDCl3
NS                      8
DS                     16
SWH            2796.421 Hz
FIDRES         1.365440 Hz
AQ            0.3662324 sec
RG              23170.5
DW              178.800 usec
DE                 6.00 usec
TE                298.0 K
CNST2       145.0000000
D0           0.00000300 sec
D1           1.00000000 sec
D4           0.00172414 sec
D11          0.03000000 sec
D16          0.00010000 sec
D24          0.00086207 sec
IN0          0.00002610 sec
ZGOPTNS

======== CHANNEL f1 ========
NUC1                   1H
P1                  11.00 usec
P2                  22.00 usec
P28               2000.00 usec
PL1                 -2.50 dB
SFO1         400.1313986 MHz

======== CHANNEL f2 ========
CPDPRG2              garp
NUC2                  13C
P3                   9.00 usec
P4                  18.00 usec
P14                500.00 usec
PCPD2               80.00 usec
PL0                120.00 dB
PL2                  3.00 dB
PL12                21.50 dB
SFO2         100.6223307 MHz
SP3                 12.07 dB
SPNAM3   Crp60,0.5,20.1
SPOAL3              0.500
SPOFFS3              0.00 Hz

====== GRADIENT CHANNEL =====
GPNAM1           SINE.100
GPNAM2           SINE.100
GPZ1                80.00 %
GPZ2                20.10 %
P16               1000.00
usec ND0                       2
TD                    512
SFO1             100.6223  MHz
FIDRES          37.416939  Hz
SW                190.390  ppm
FnMODE      Echo-Antiecho
SI                   2048
SF            400.1300000  MHz
WDW                 QSINE
SSB                     2
LB                   0.00 Hz
GB                      0
PC                   1.00
SI                   1024
MC2         echo-antiecho
SF            100.6127690  MHz
WDW                 QSINE
SSB                     2
LB                   0.00 Hz
GB                      0
```

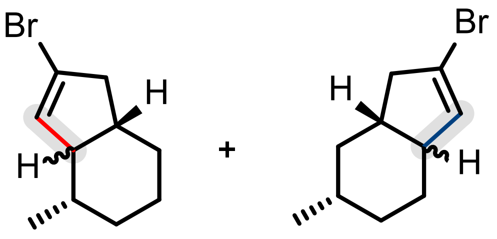

cis-4d *cis*-4d'

HMBC NMR([600, 150] MHz, $CDCl_3$)

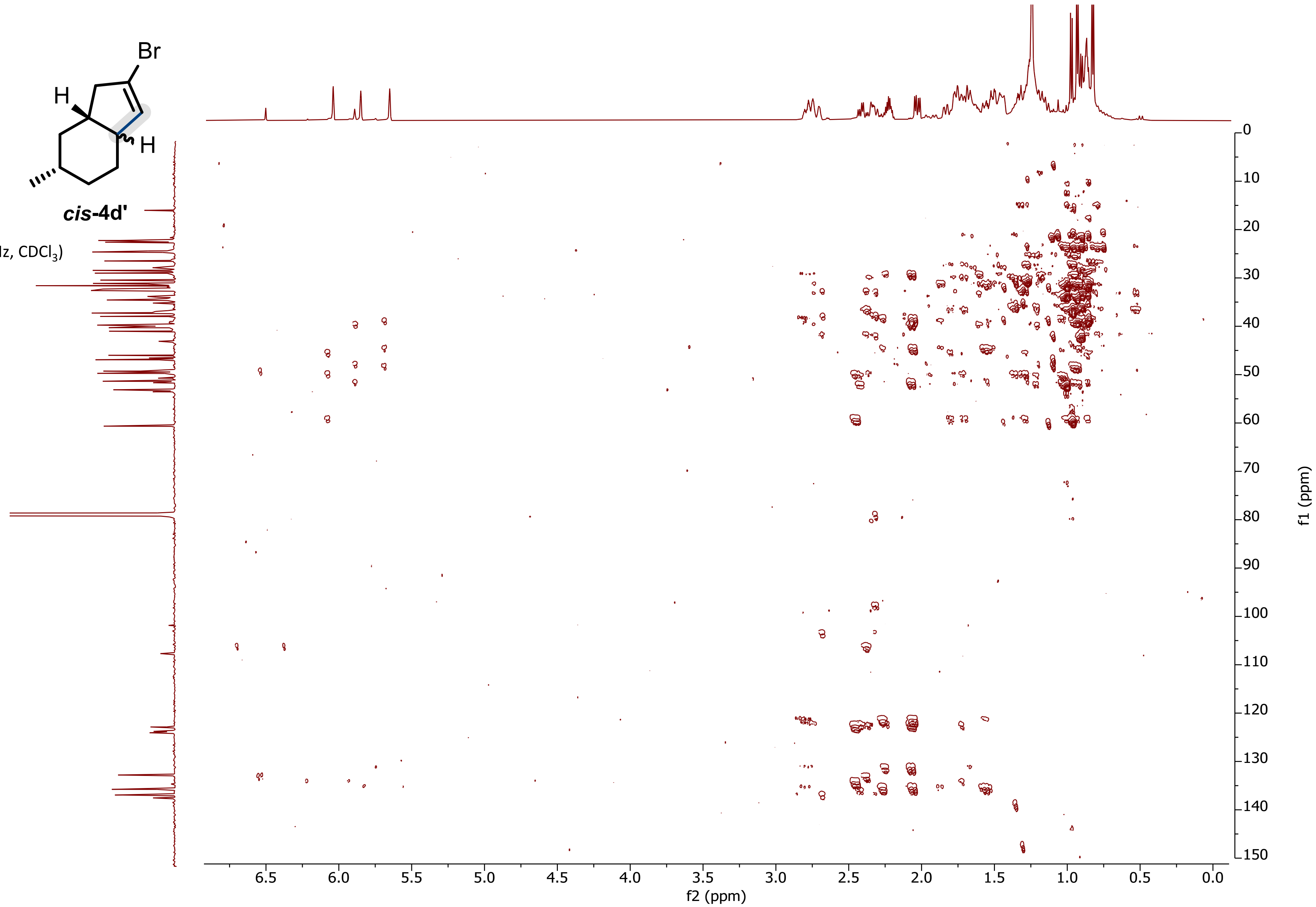

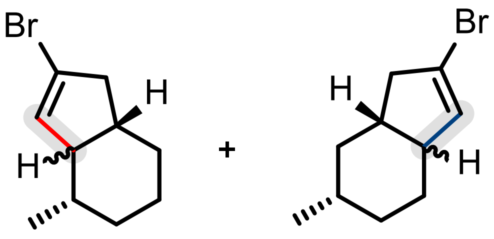

***cis*-4d** ***cis*-4d'**

HMQC NMR([600, 150] MHz, $CDCl_3$)

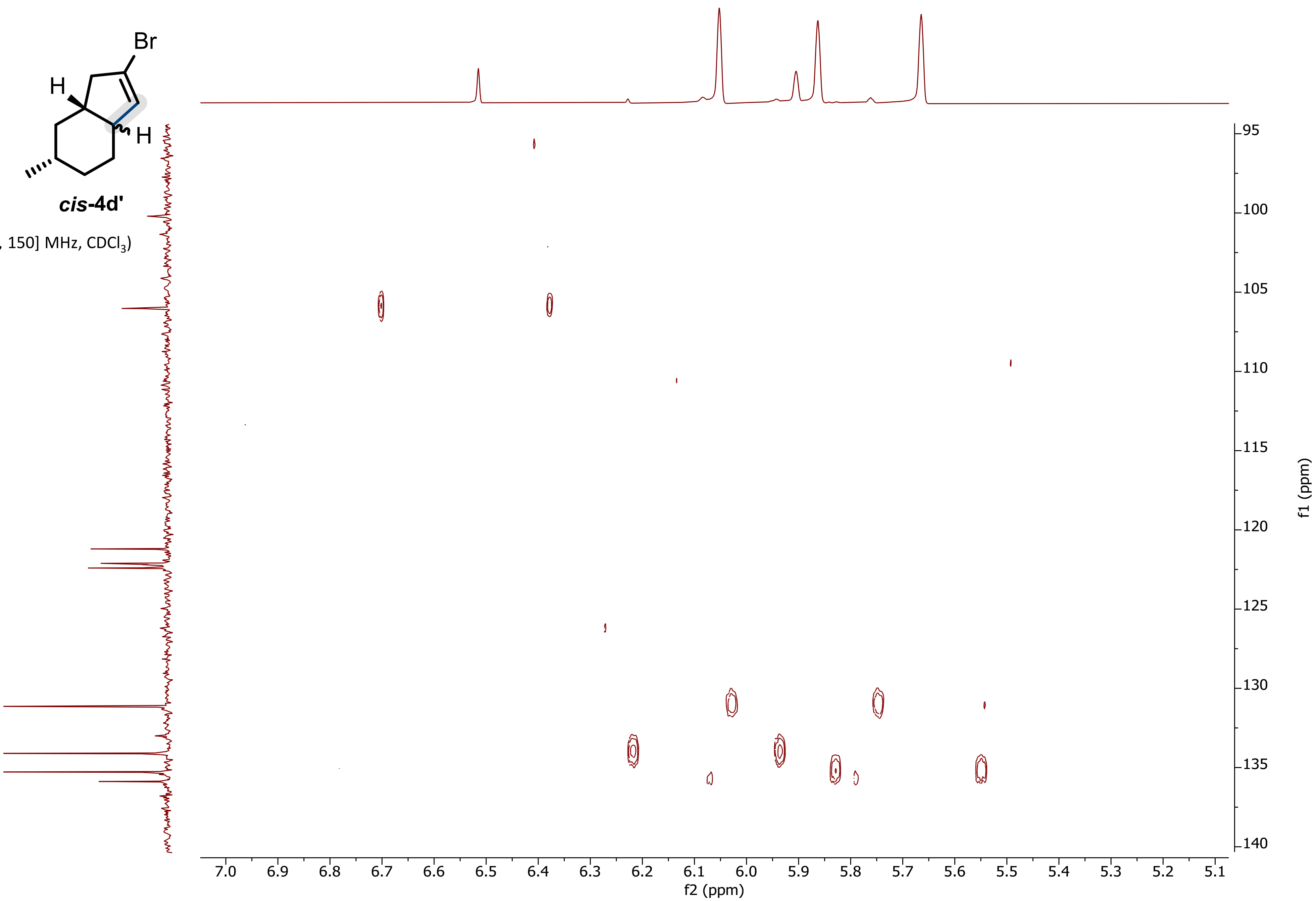

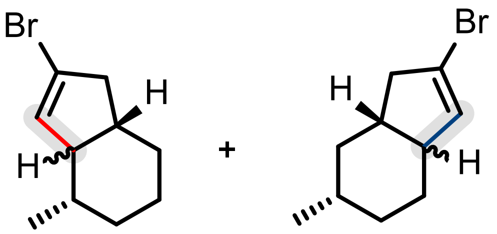

cis-4d *cis*-4d'

SEL-TOCSY NMR(600 MHz, $CDCl_3$)

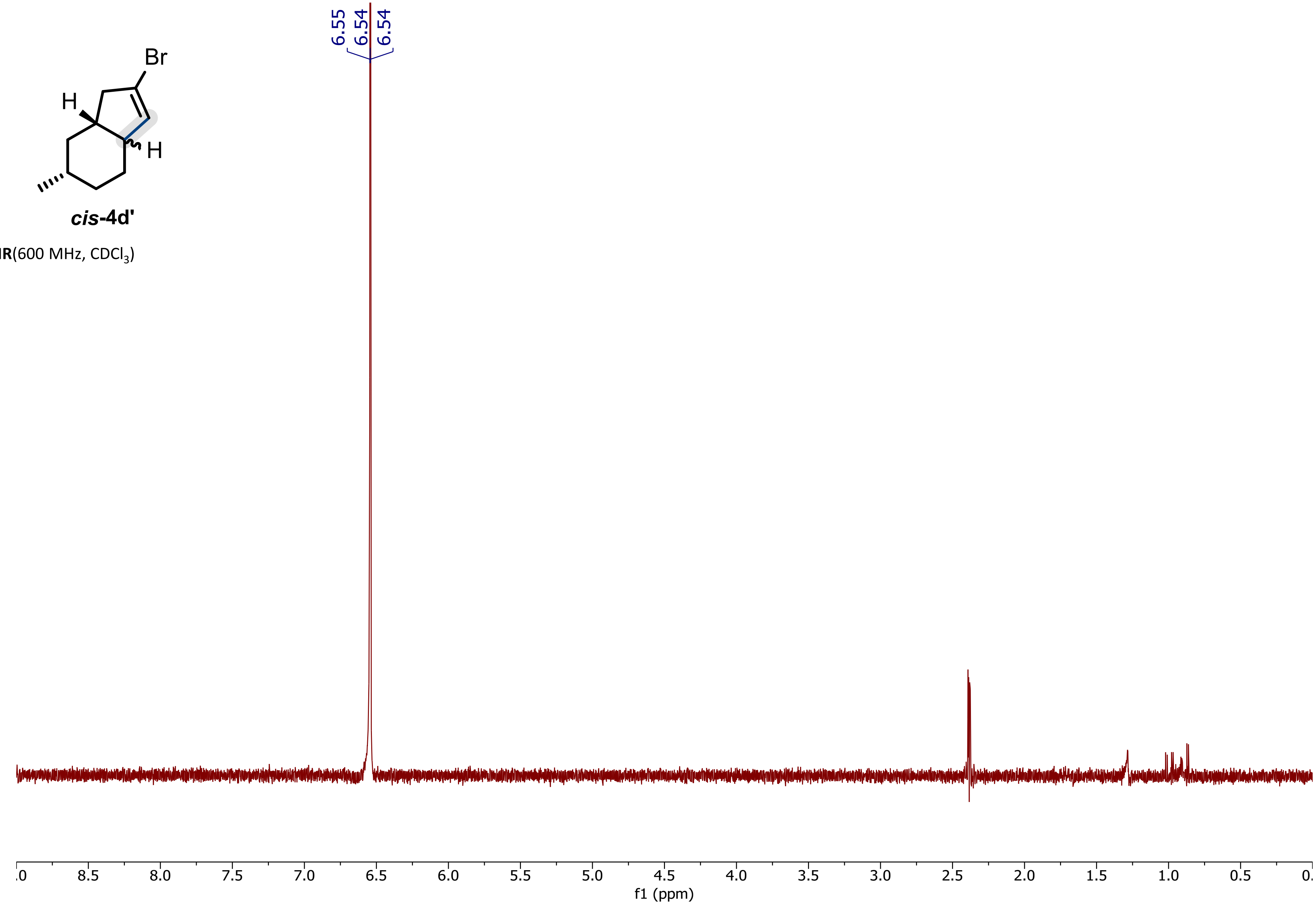

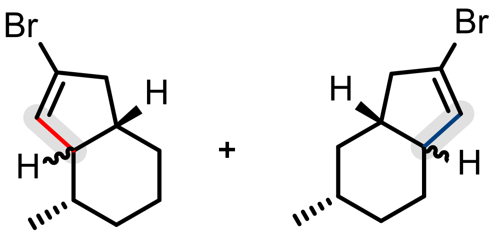

cis-4d *cis*-4d'

SEL-TOCSY NMR(600 MHz, $CDCl_3$)

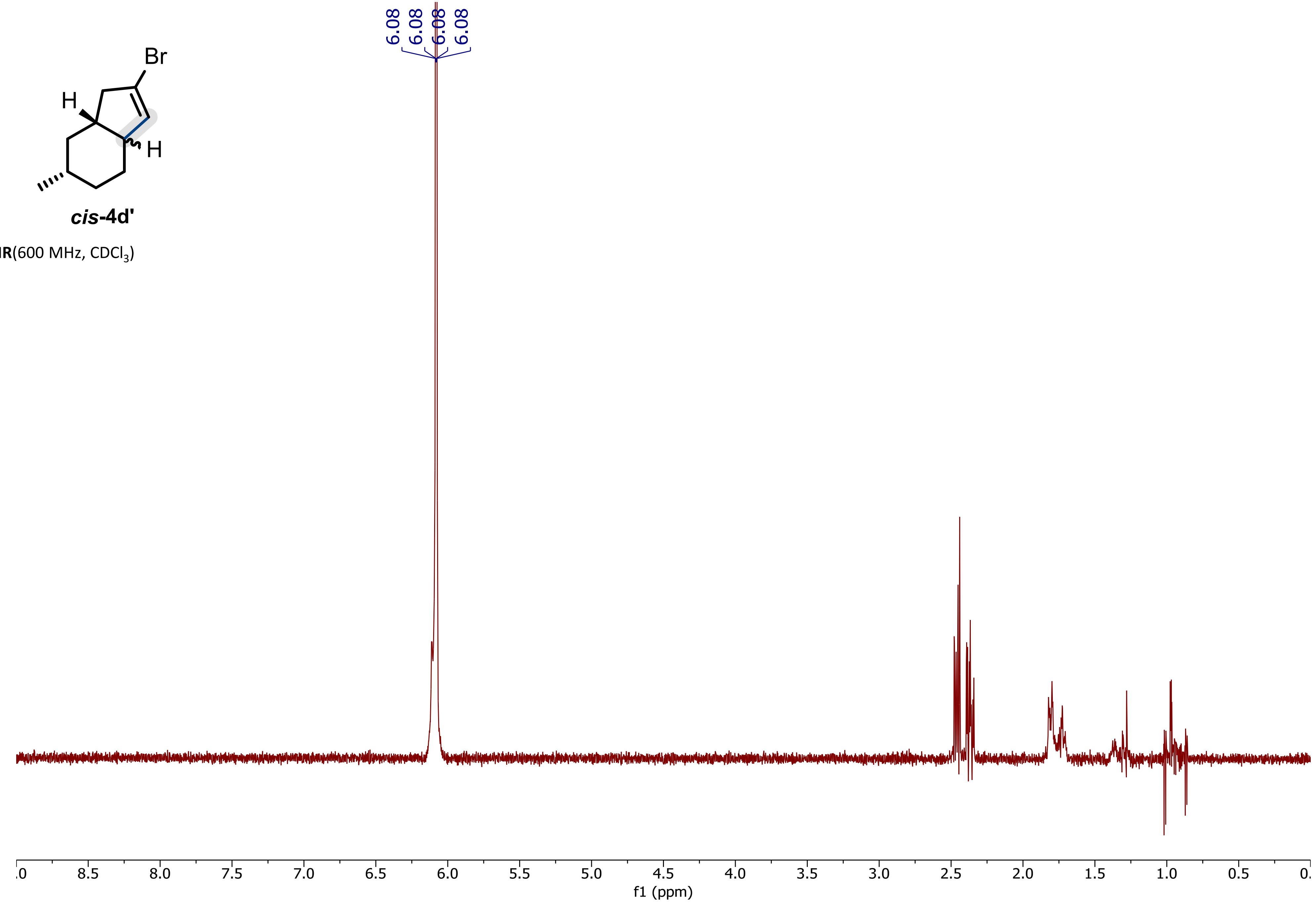

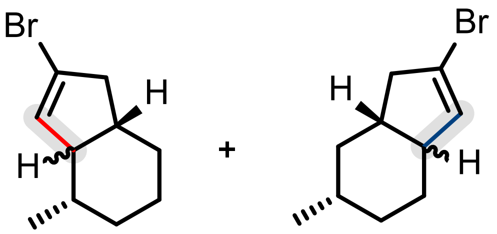

***cis*-4d** + ***cis*-4d'**

SEL-TOCSY NMR(600 MHz, $CDCl_3$)

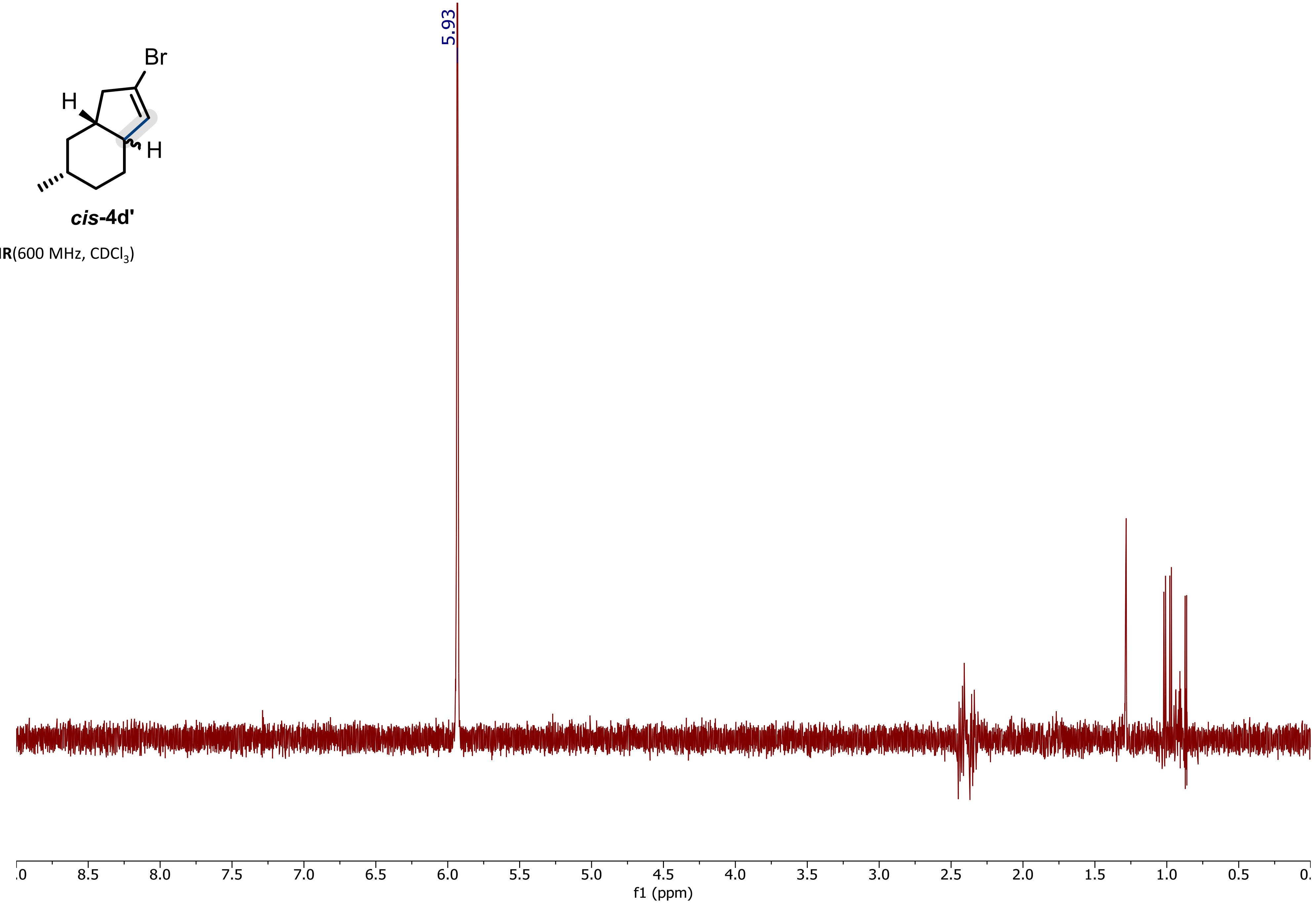

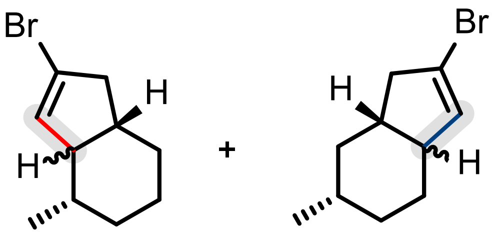

***cis*-4d** ***cis*-4d'**

SEL-TOCSY NMR(600 MHz, $CDCl_3$)

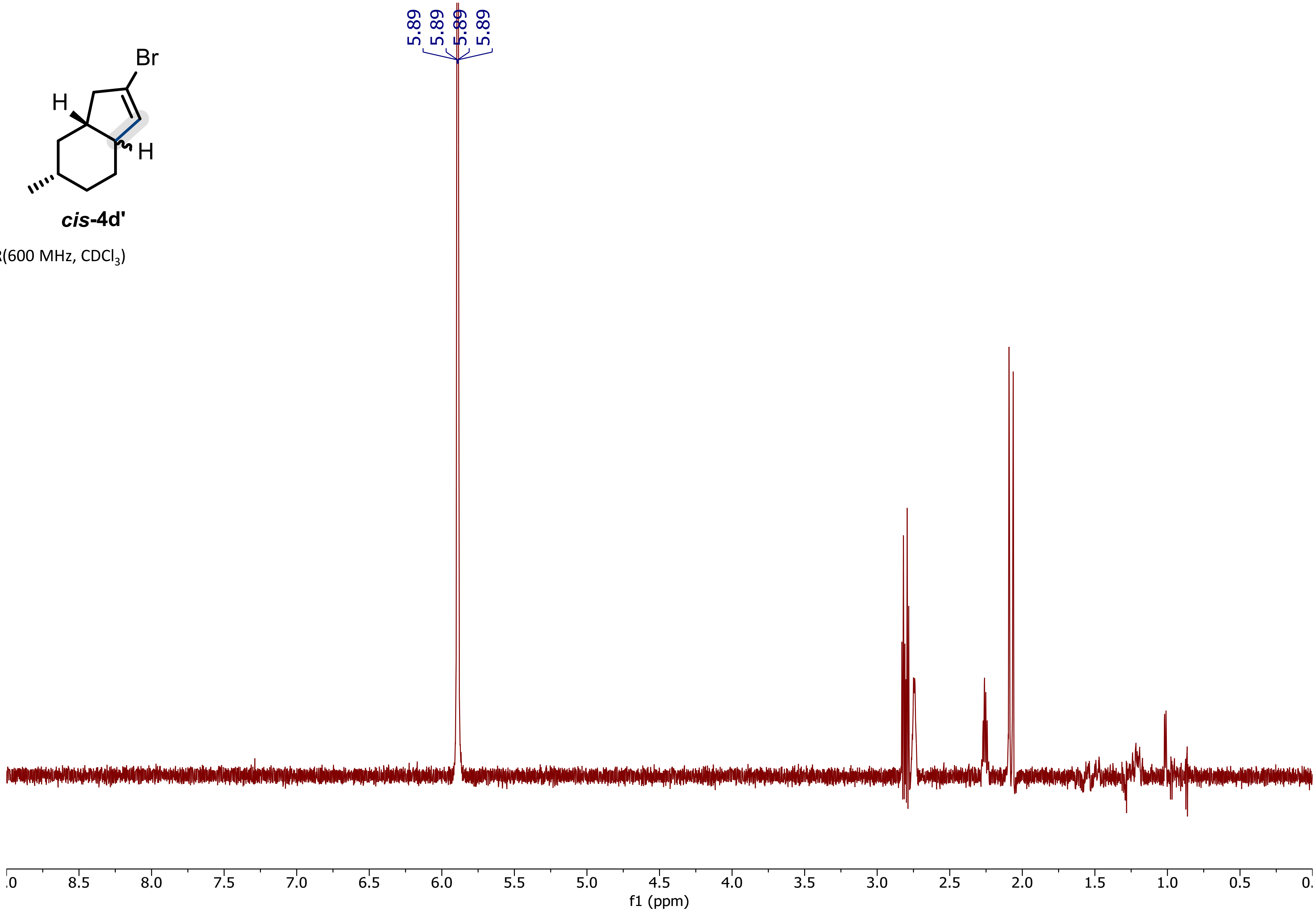

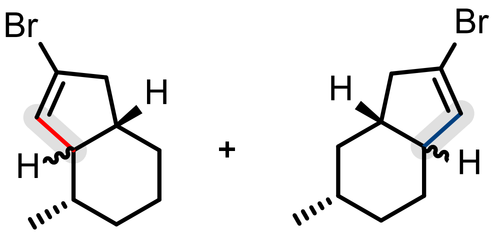

***cis*-4d** ***cis*-4d'**

SEL-TOCSY NMR(600 MHz, $CDCl_3$)

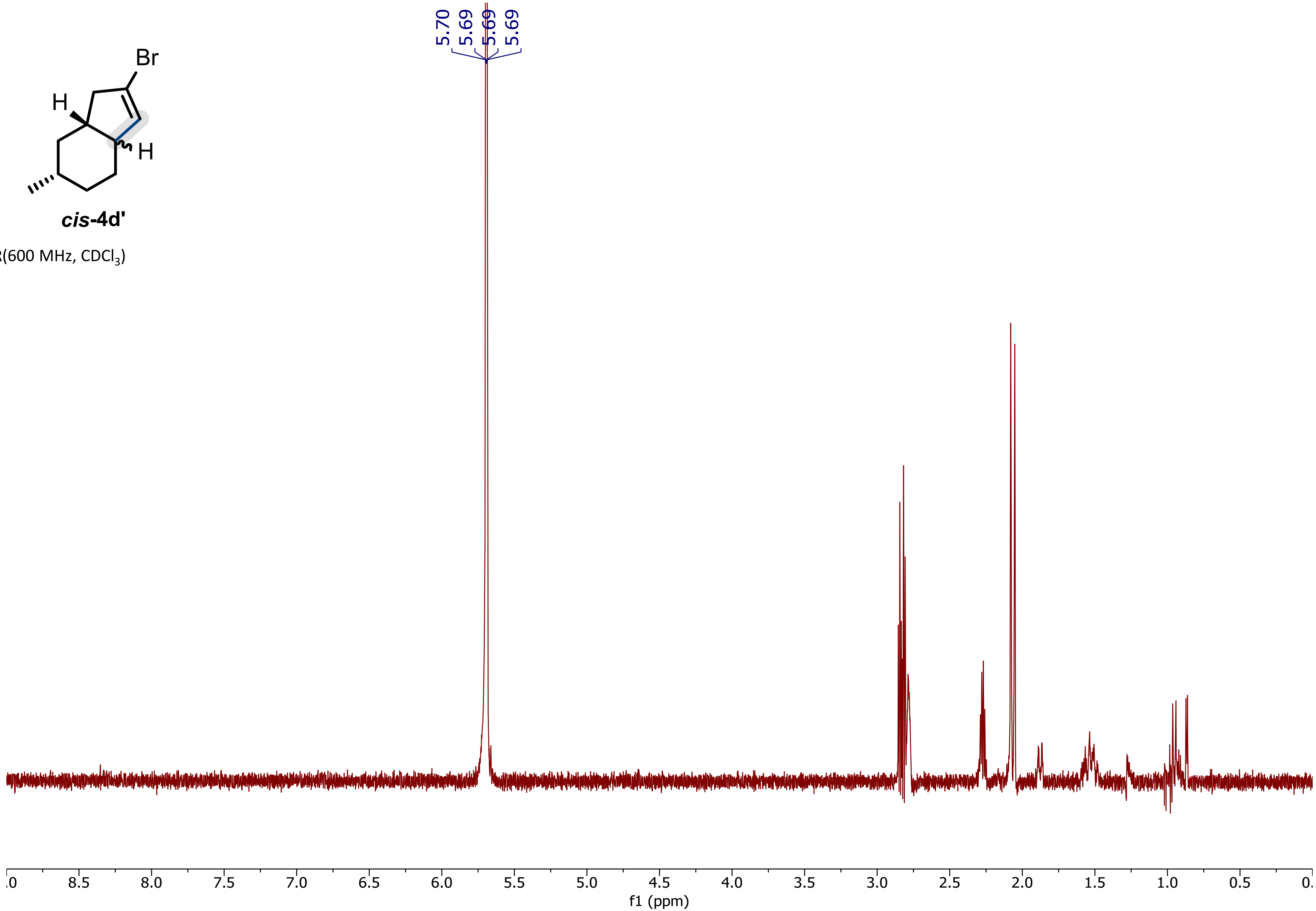

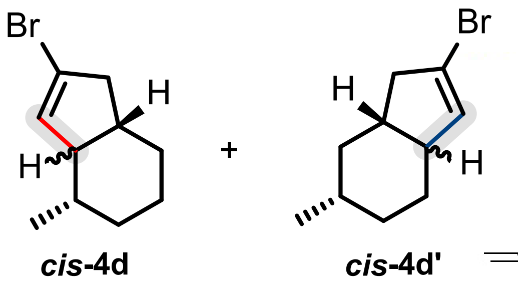

HSQC/SEL-NOE NMR([600, 150] MHz, $CDCl_3$)

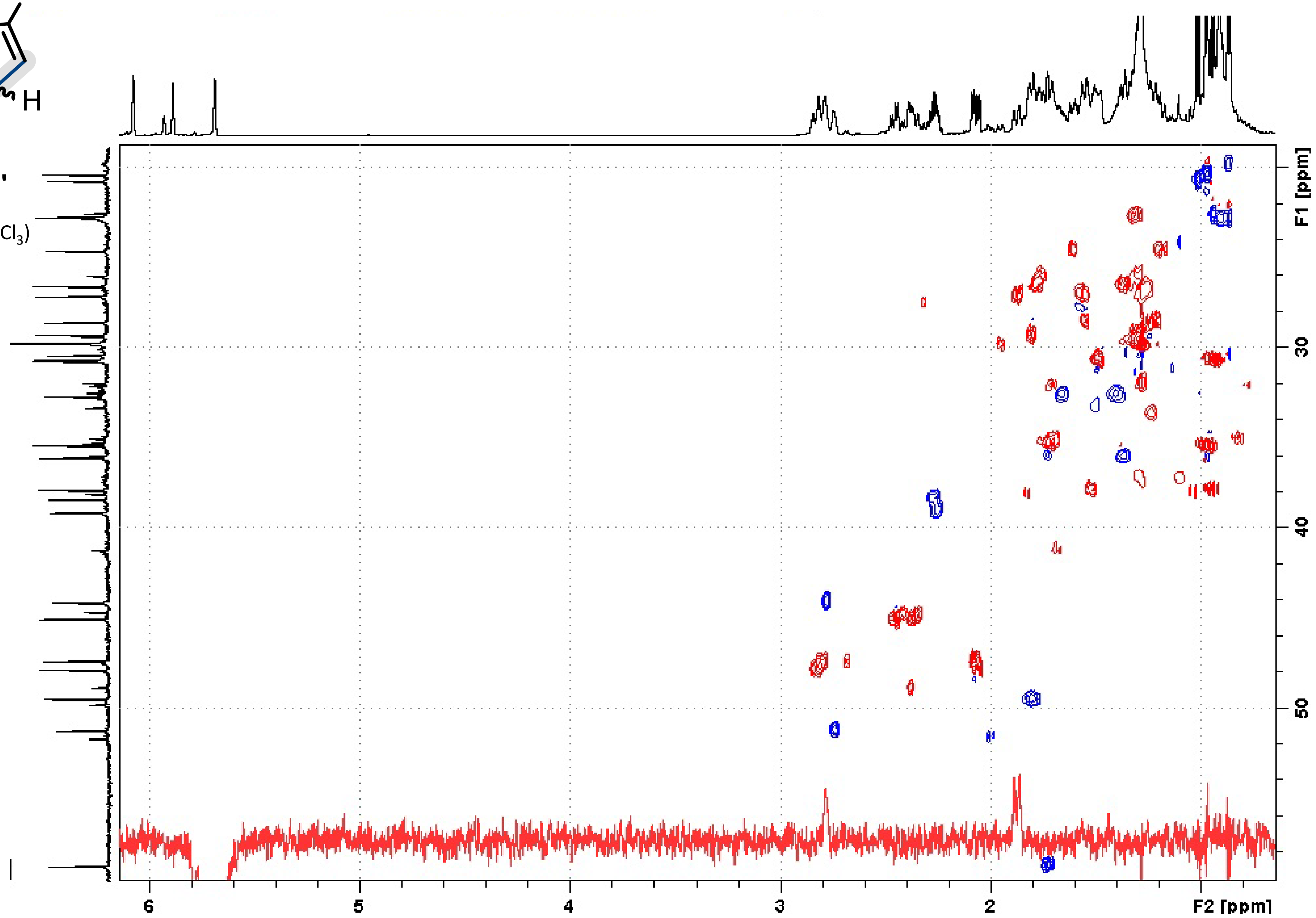

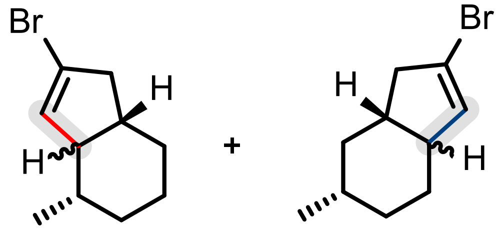

***cis*-4d** ***cis*-4d'**

HSQC/SEL-NOE/SEL-TOCSY NMR([600, 150] MHz, $CDCl_3$)

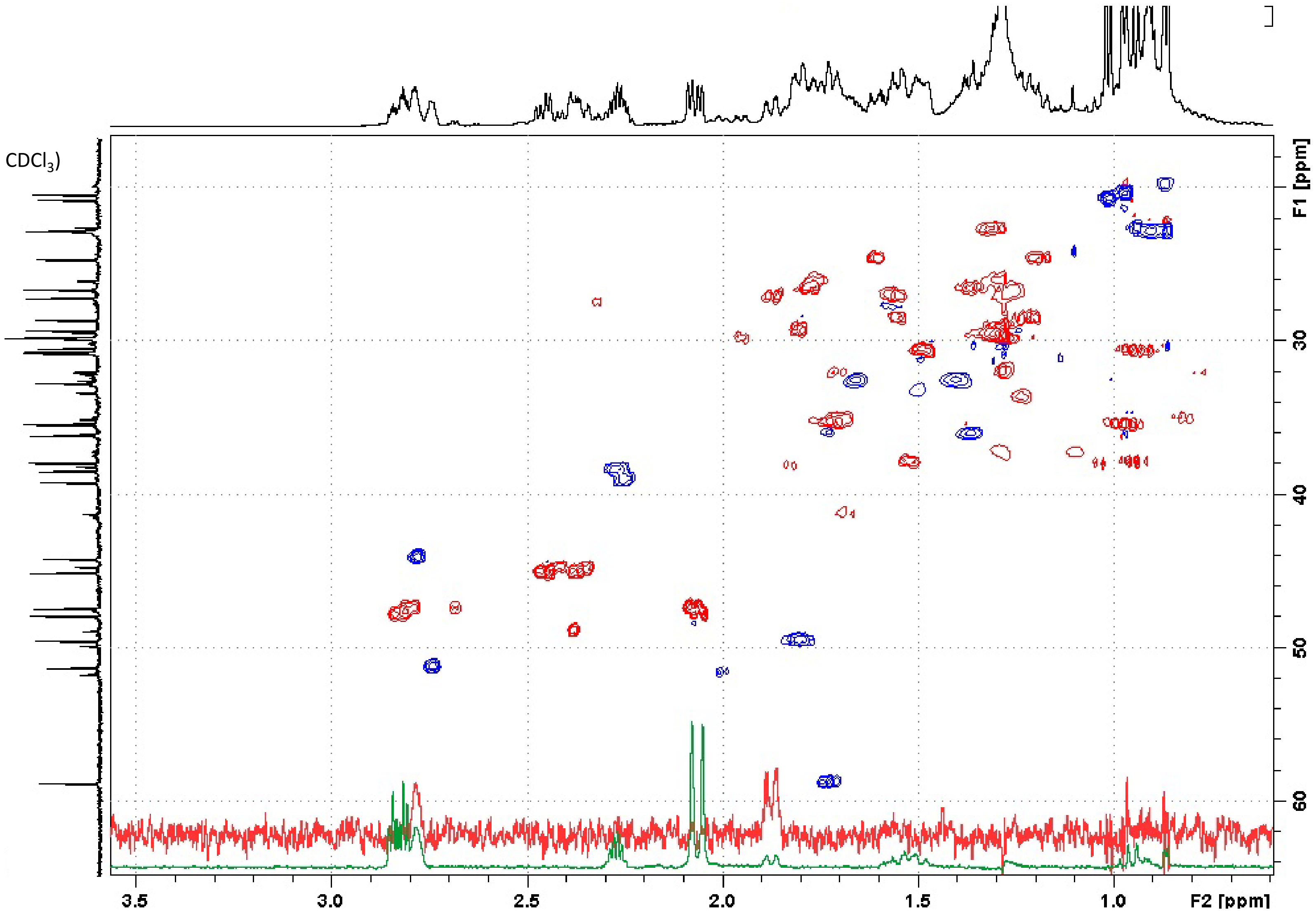

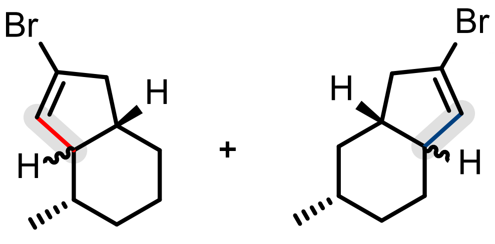

SEL-NOEs NMR(600 MHz, $CDCl_3$)

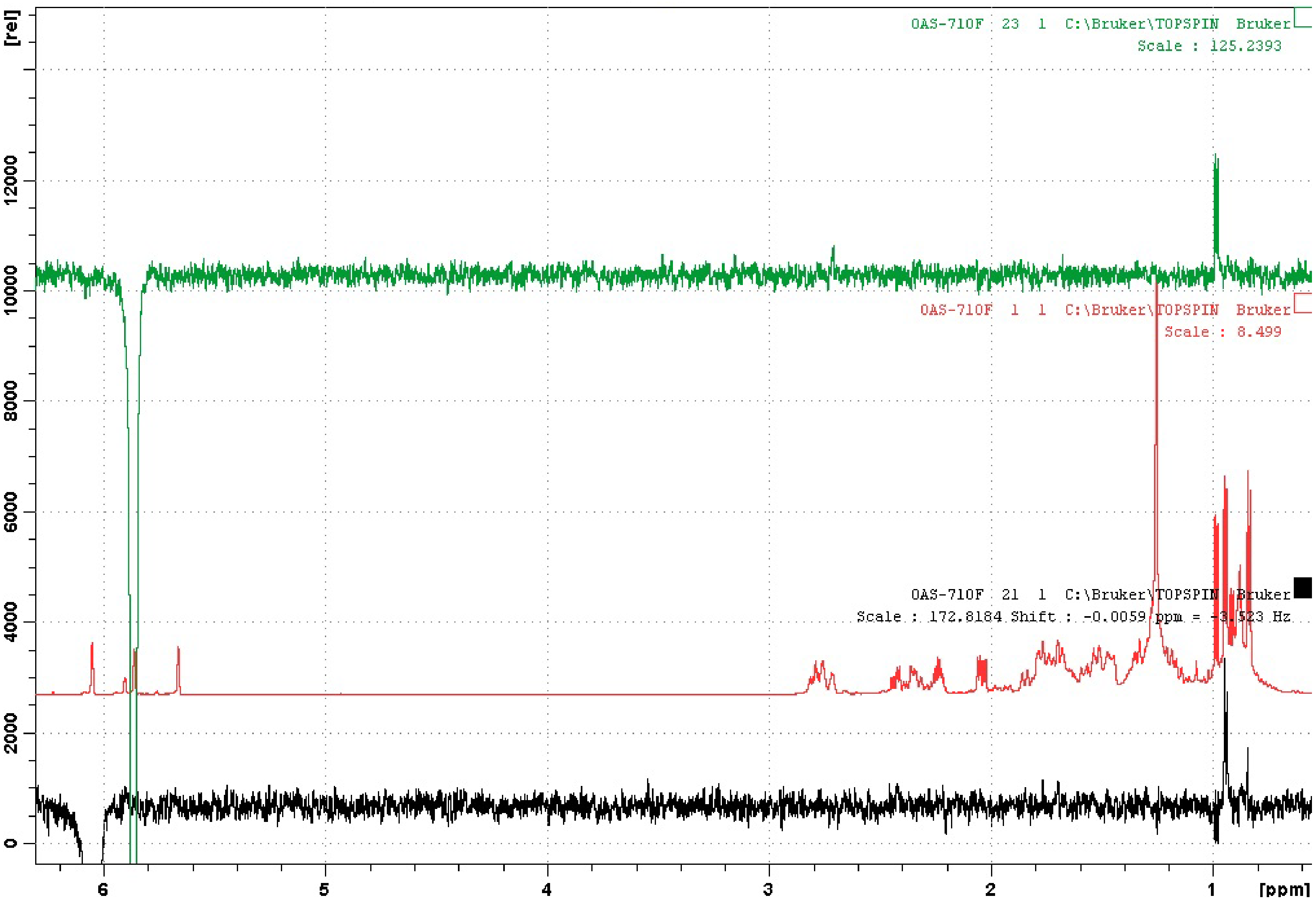

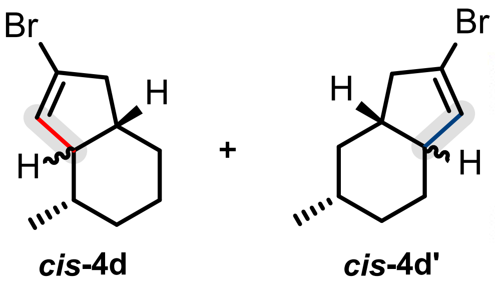

SEL-TOCSYs NMR(600 MHz, $CDCl_3$)

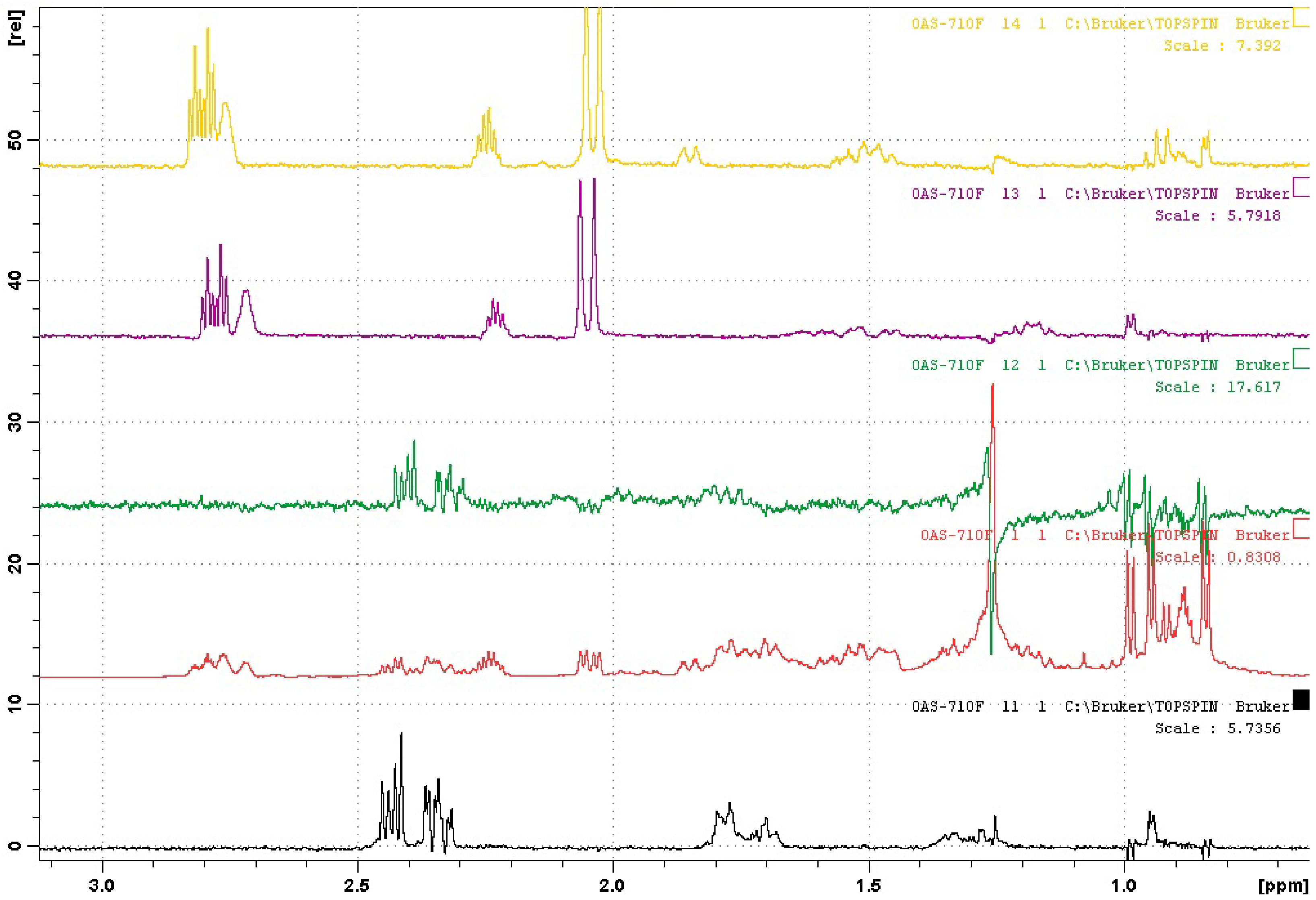

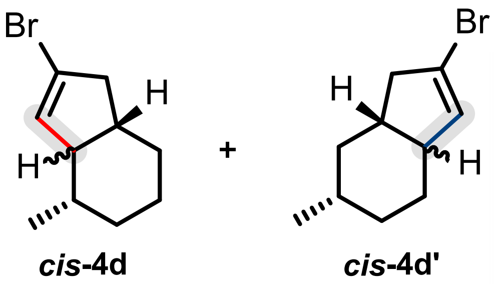

SEL-NOE/SEL-TOCSY NMR(600 MHz, $CDCl_3$)

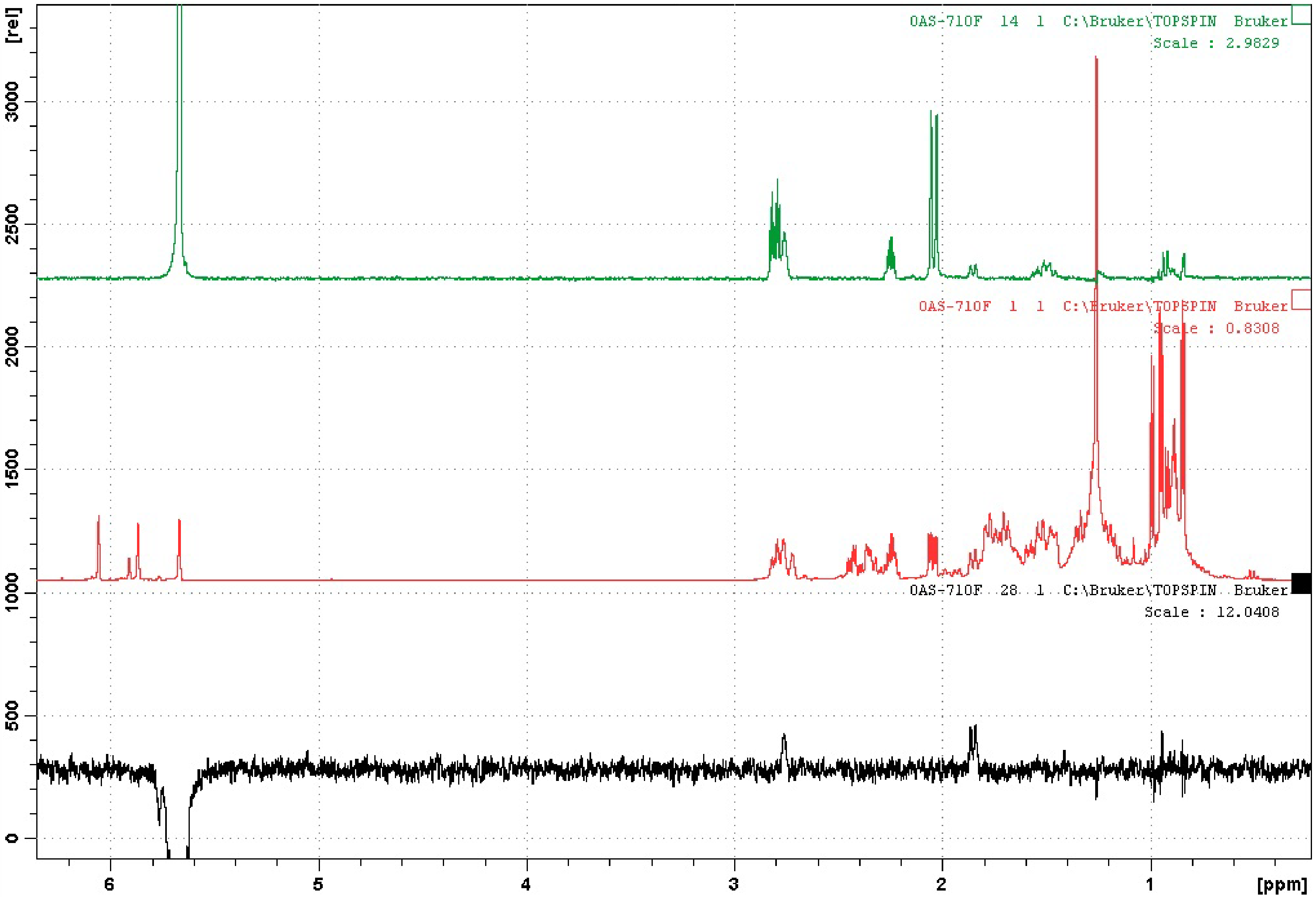

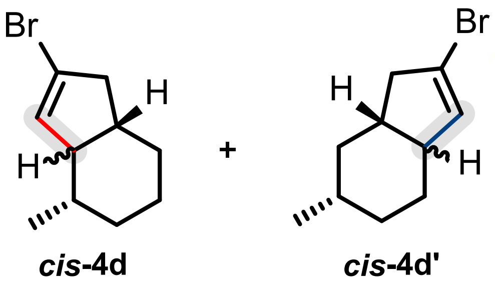

SEL-NOEs NMR(600 MHz, $CDCl_3$)

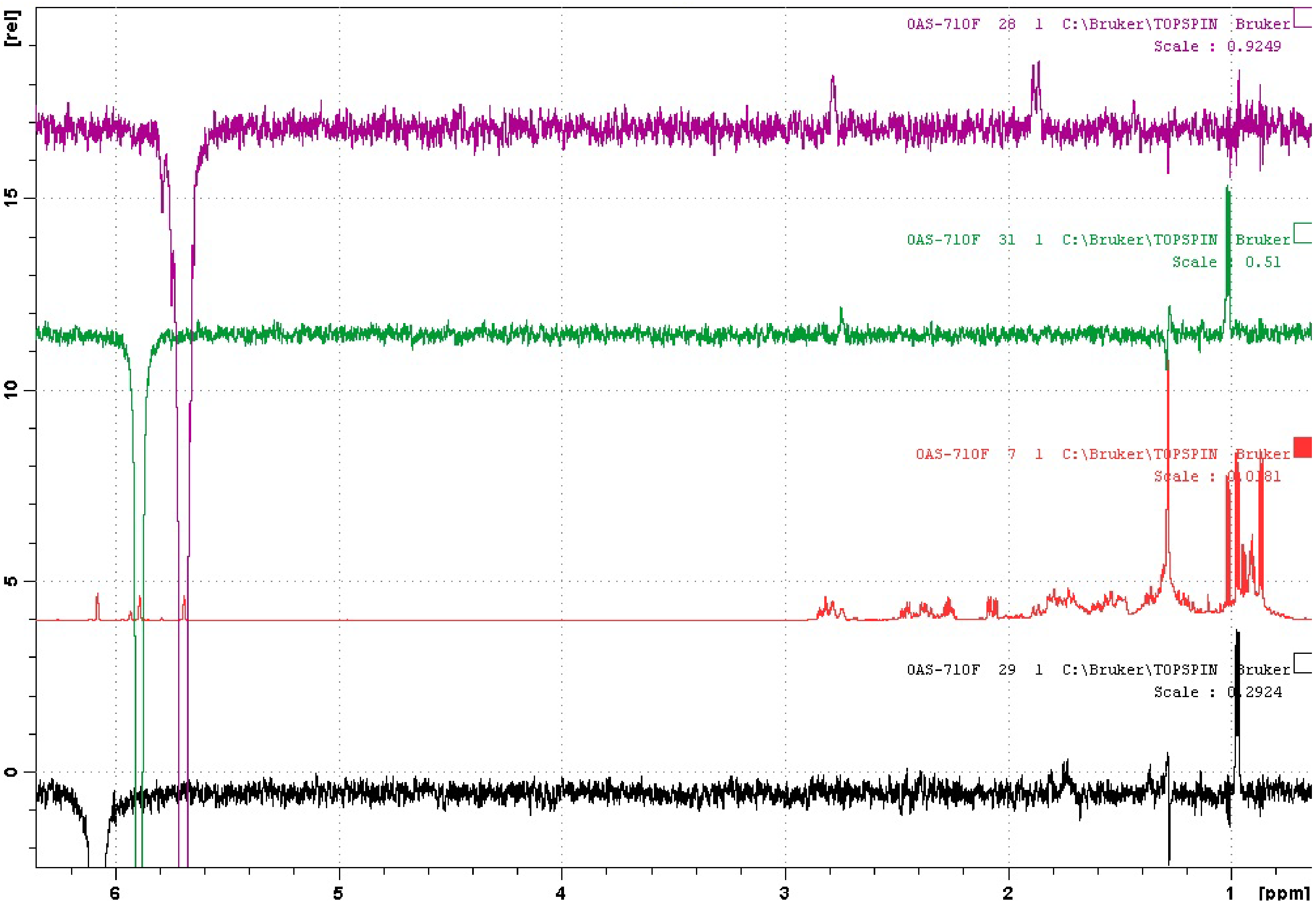

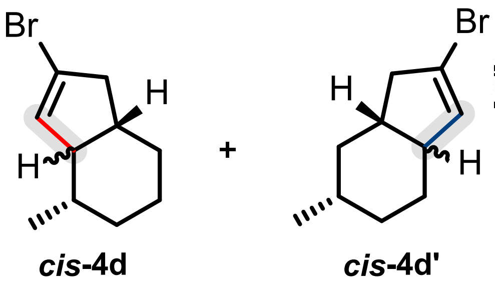

SEL-NOEs NMR(600 MHz, $CDCl_3$)

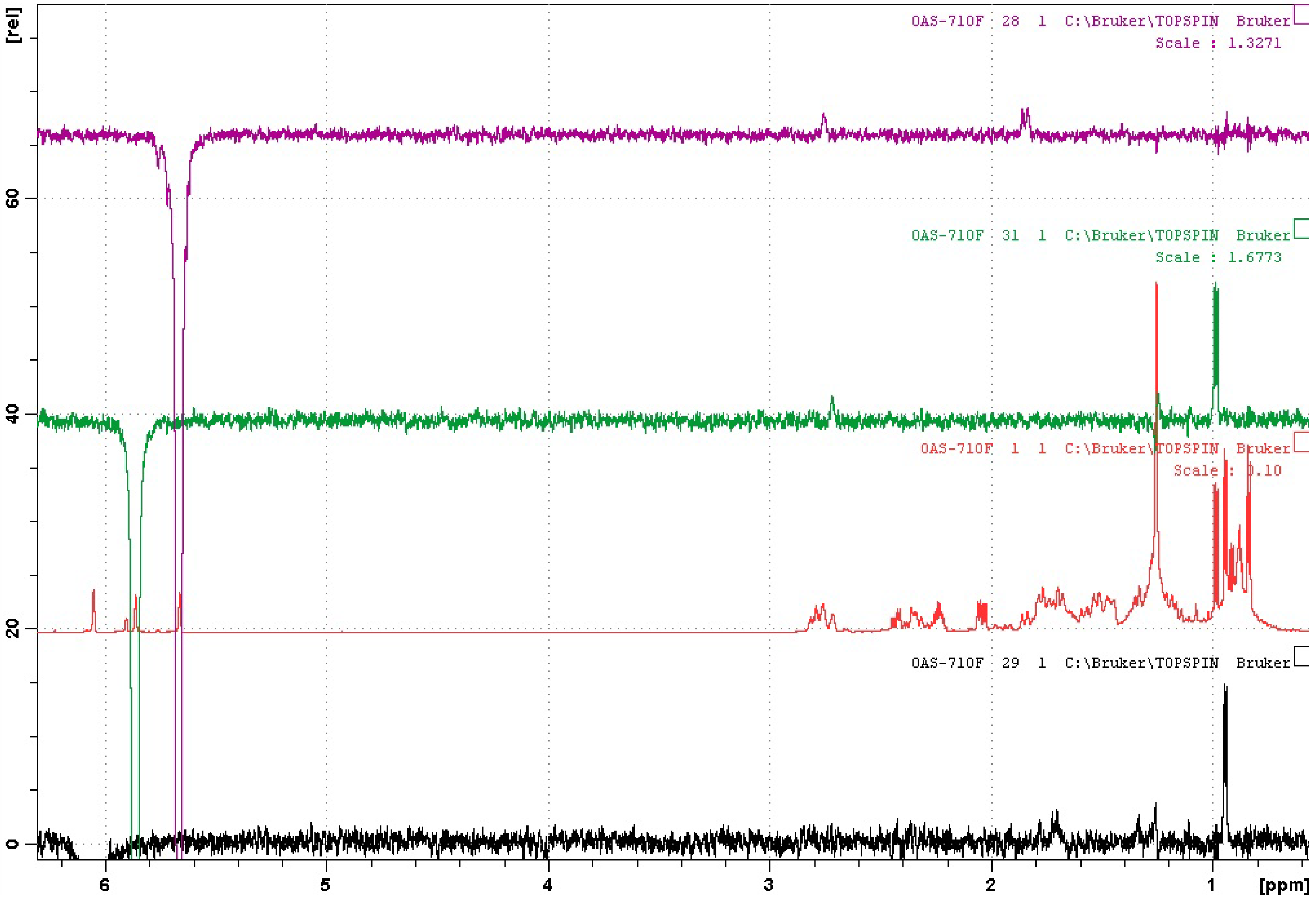

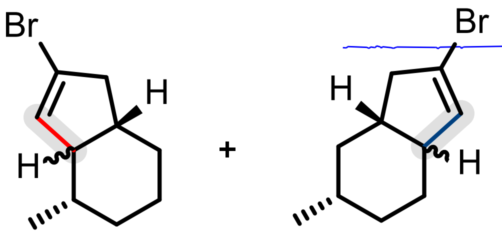

cis-4d *cis*-4d'

SEL-NOEs NMR(600 MHz, $CDCl_3$)

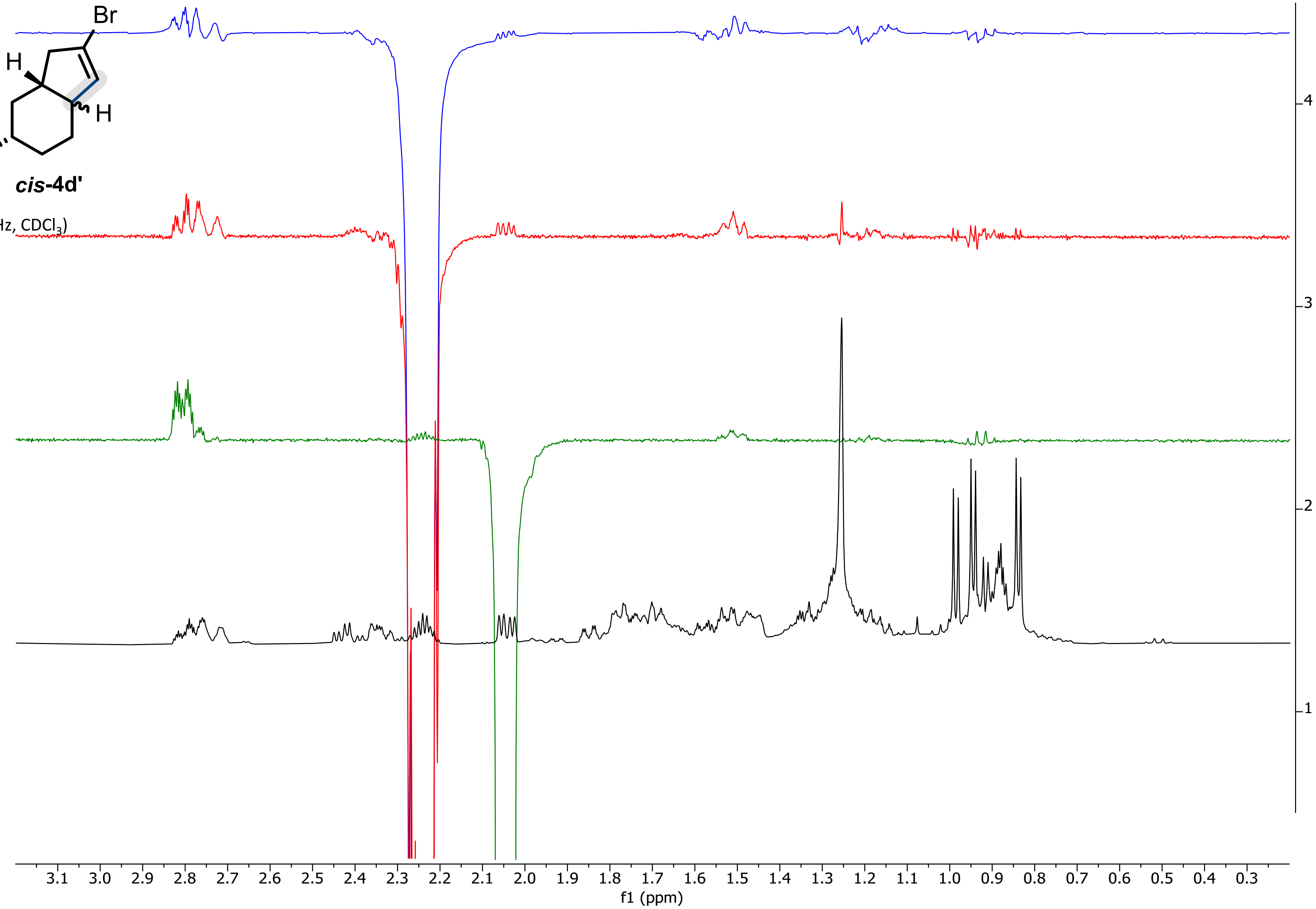

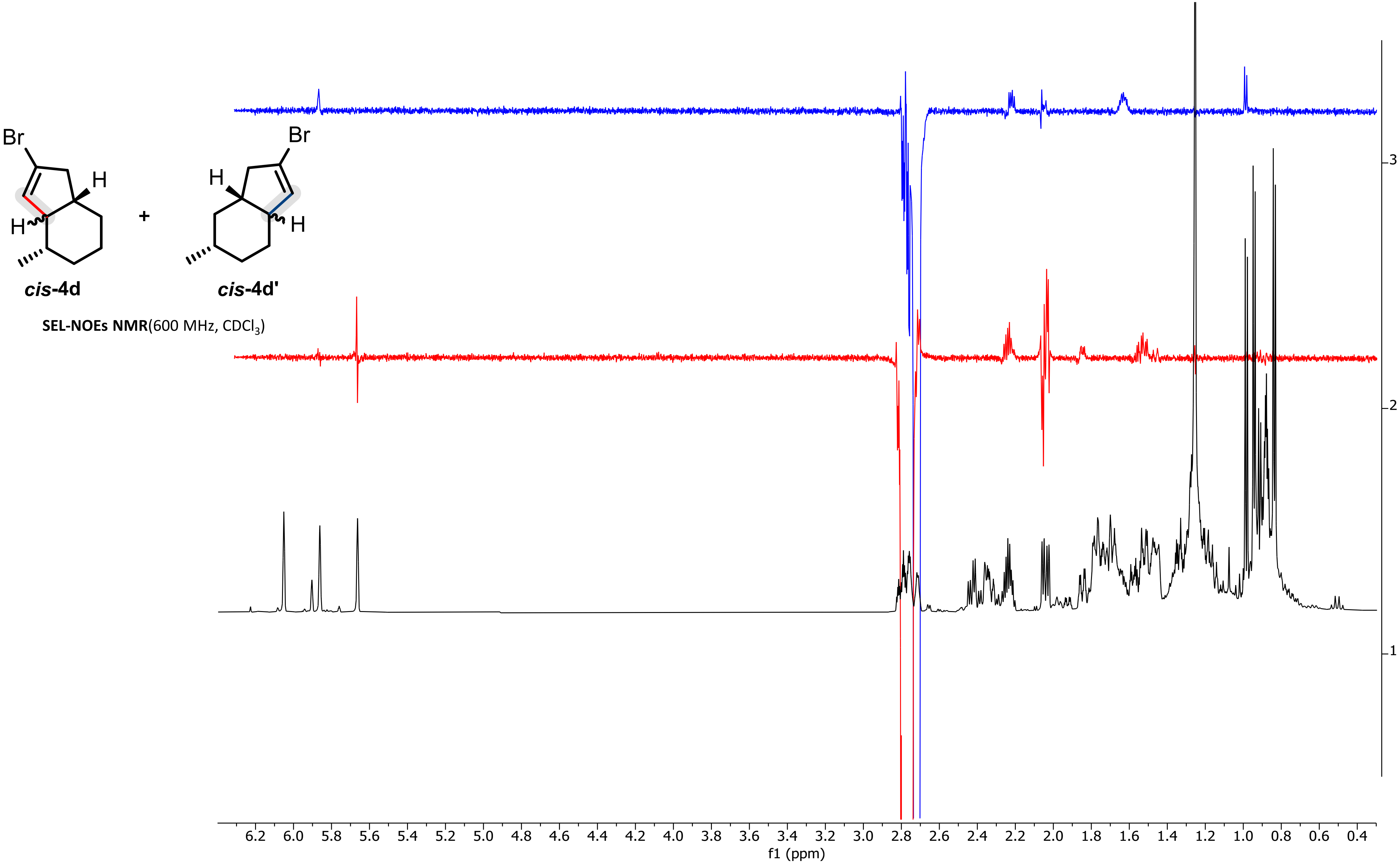
Br
H
H
cis-4d
+
Br
H
H
cis-4d'
SEL-NOEs NMR(600 MHz, CDCl3)
3
2
1
6.2
6.0
5.8
5.6
5.4
5.2
5.0
4.8
4.6
4.4
4.2
4.0
3.8
3.6
3.4
3.2
3.0
2.8
2.6
2.4
2.2
2.0
1.8
1.6
1.4
1.2
1.0
0.8
0.6
0.4
f1 (ppm)

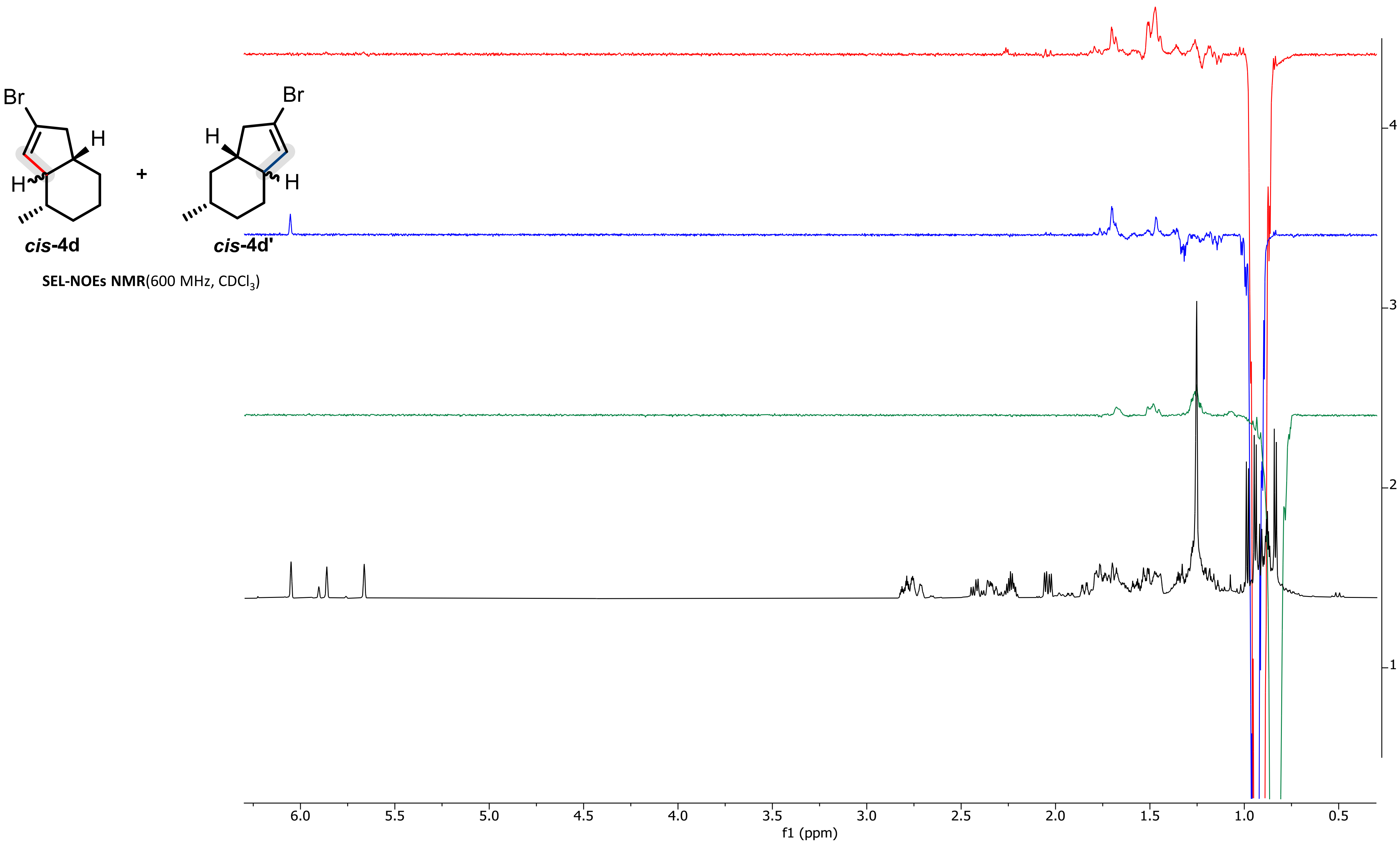

Br
H
H
cis-4d
+
Br
H
H
cis-4d'
SEL-NOEs NMR(600 MHz, CDCl3)
6.0
5.5
5.0
4.5
4.0
3.5
3.0
2.5
2.0
1.5
1.0
0.5
f1 (ppm)
4
3
2
1

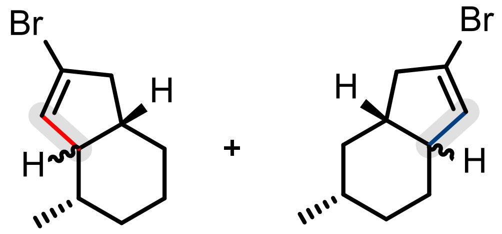

cis-4d *cis*-4d'

HSQC/SEL-TOCSY NMR

HSQC/SEL-TOCSY NMR([600, 150] MHz, $CDCl_3$)

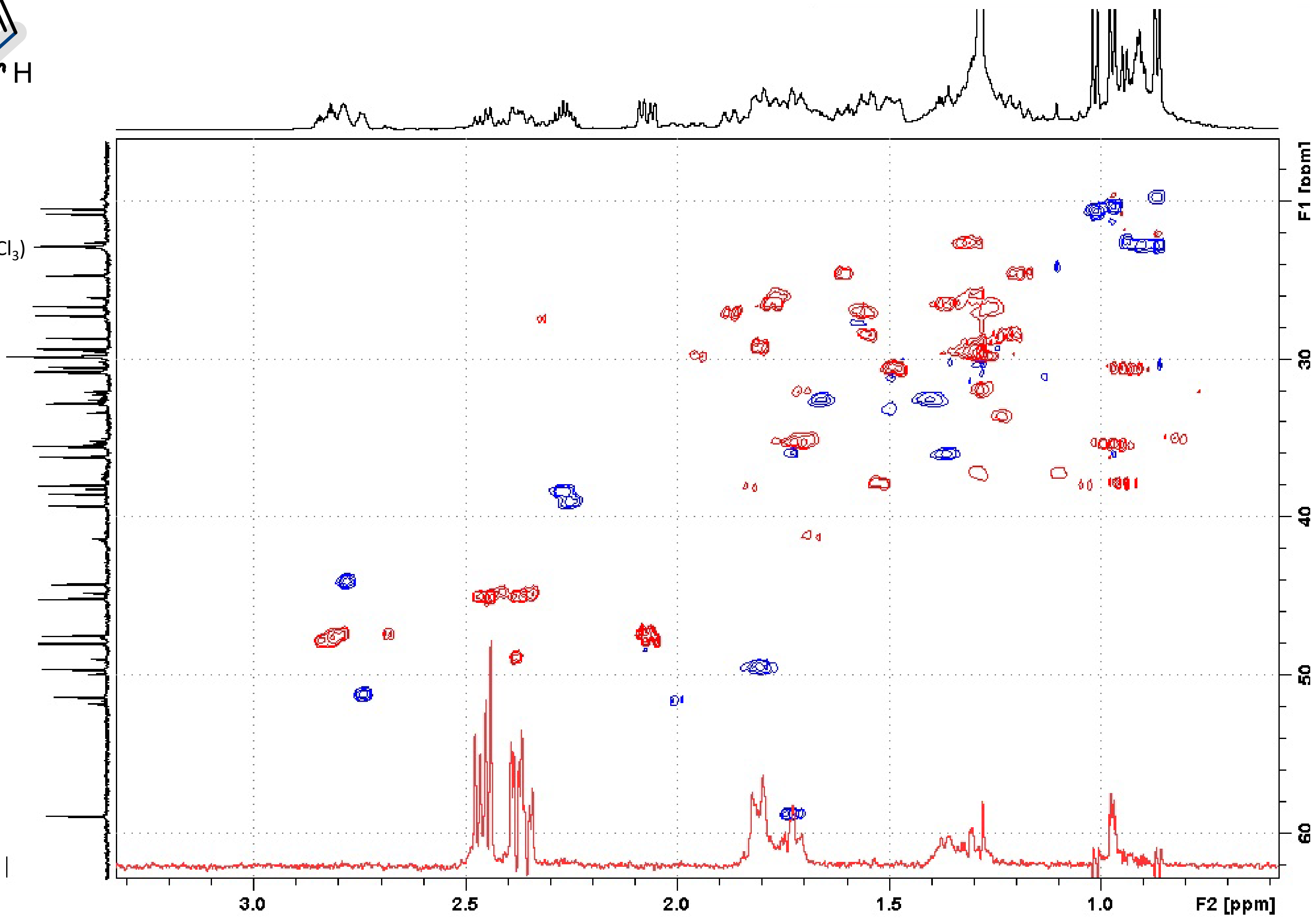

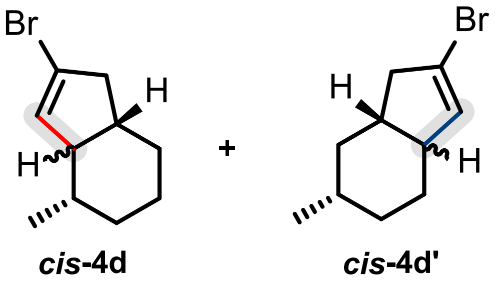

HSQC/SEL-TOCSY NMR

HSQC/SEL-TOCSY NMR([600, 150] MHz, $CDCl_3$)

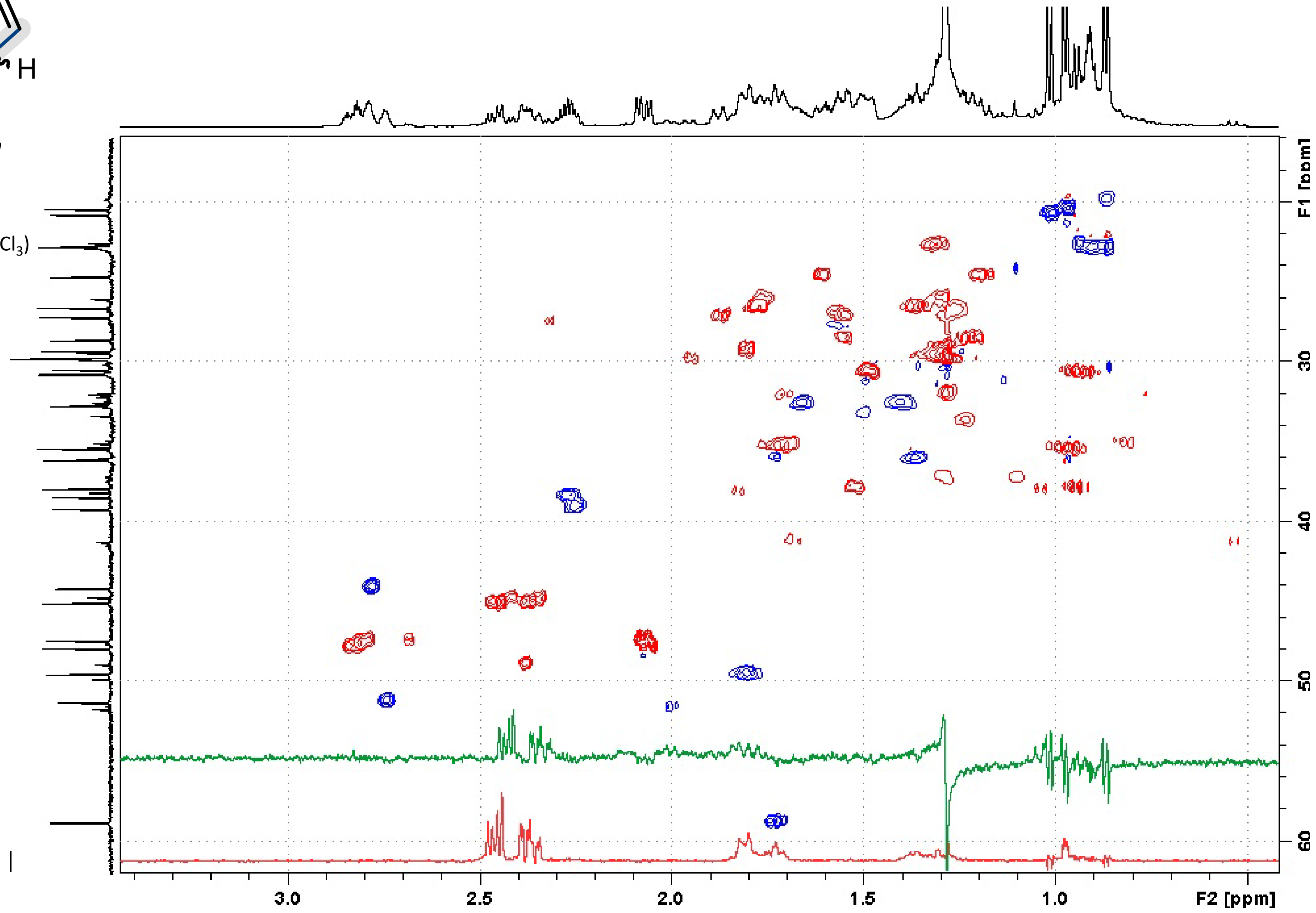

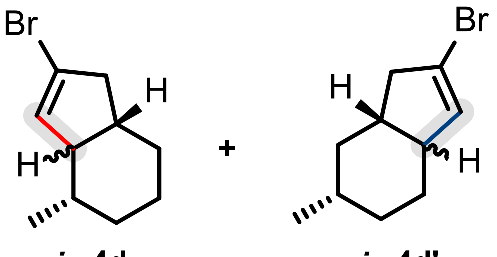

HSQC/SEL-TOCSY NMR

HSQC/SEL-TOCSY NMR([600, 150] MHz, $CDCl_3$)

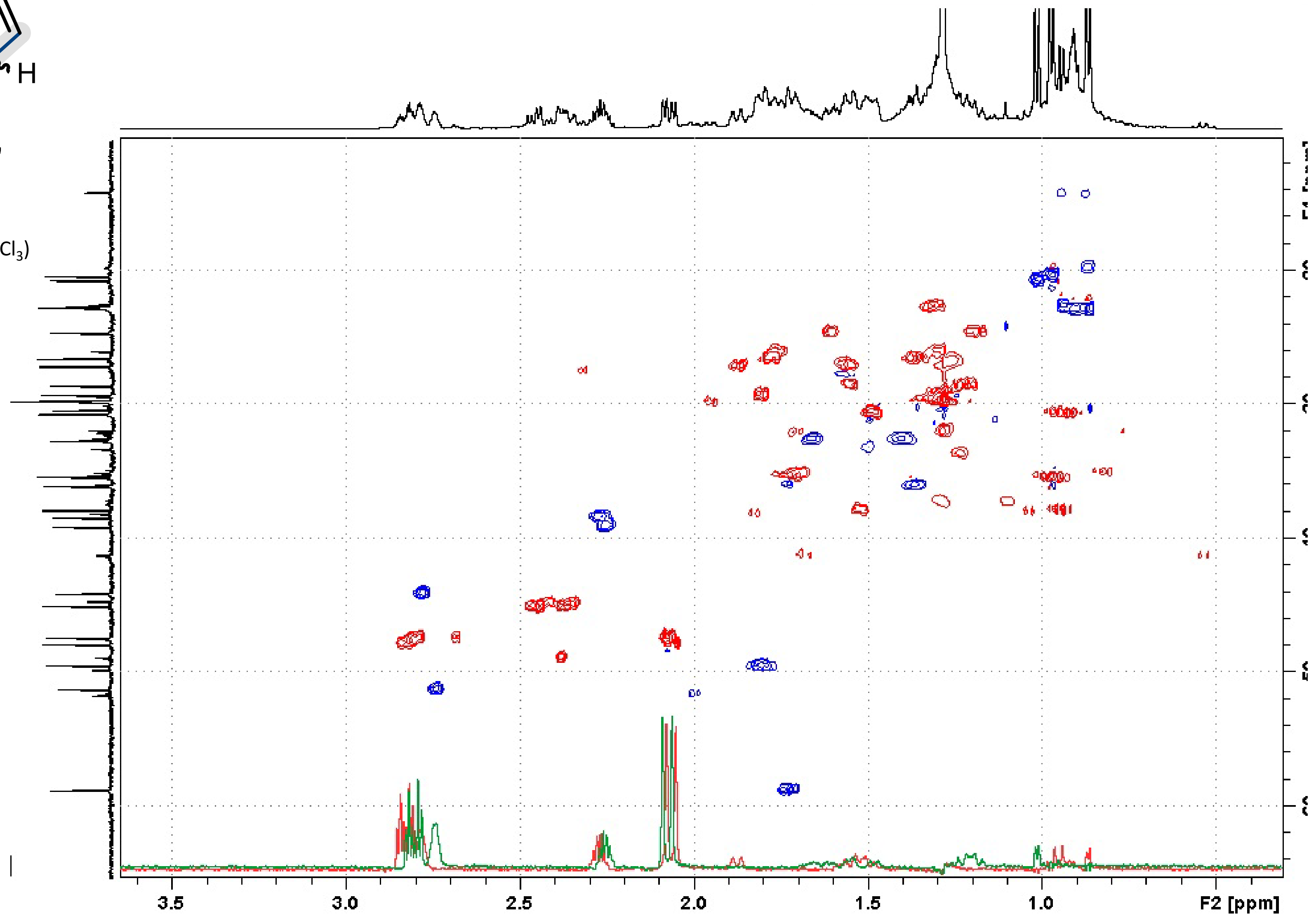

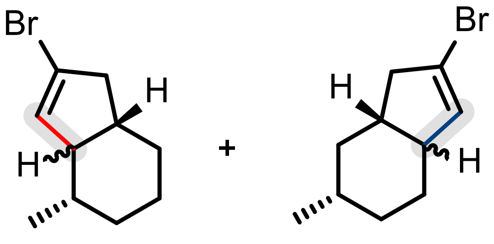

HSQC/SEL-TOCSY NMR

HSQC/SEL-TOCSY NMR([600, 150] MHz, $CDCl_3$)

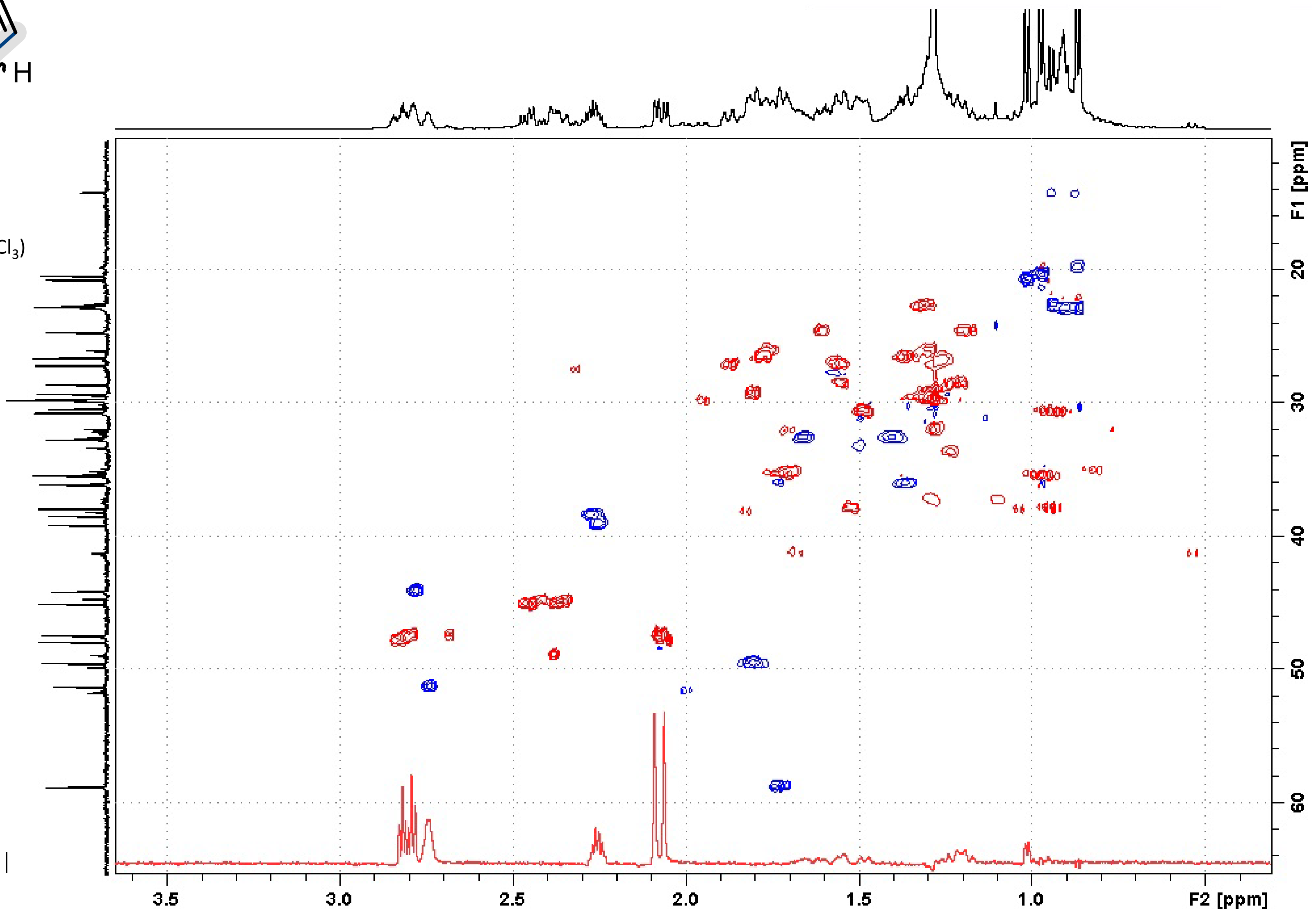

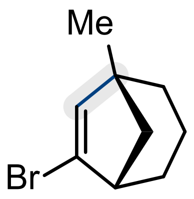

***trans*-2d**

^{1}H NMR(400 MHz, $CDCl_3$)

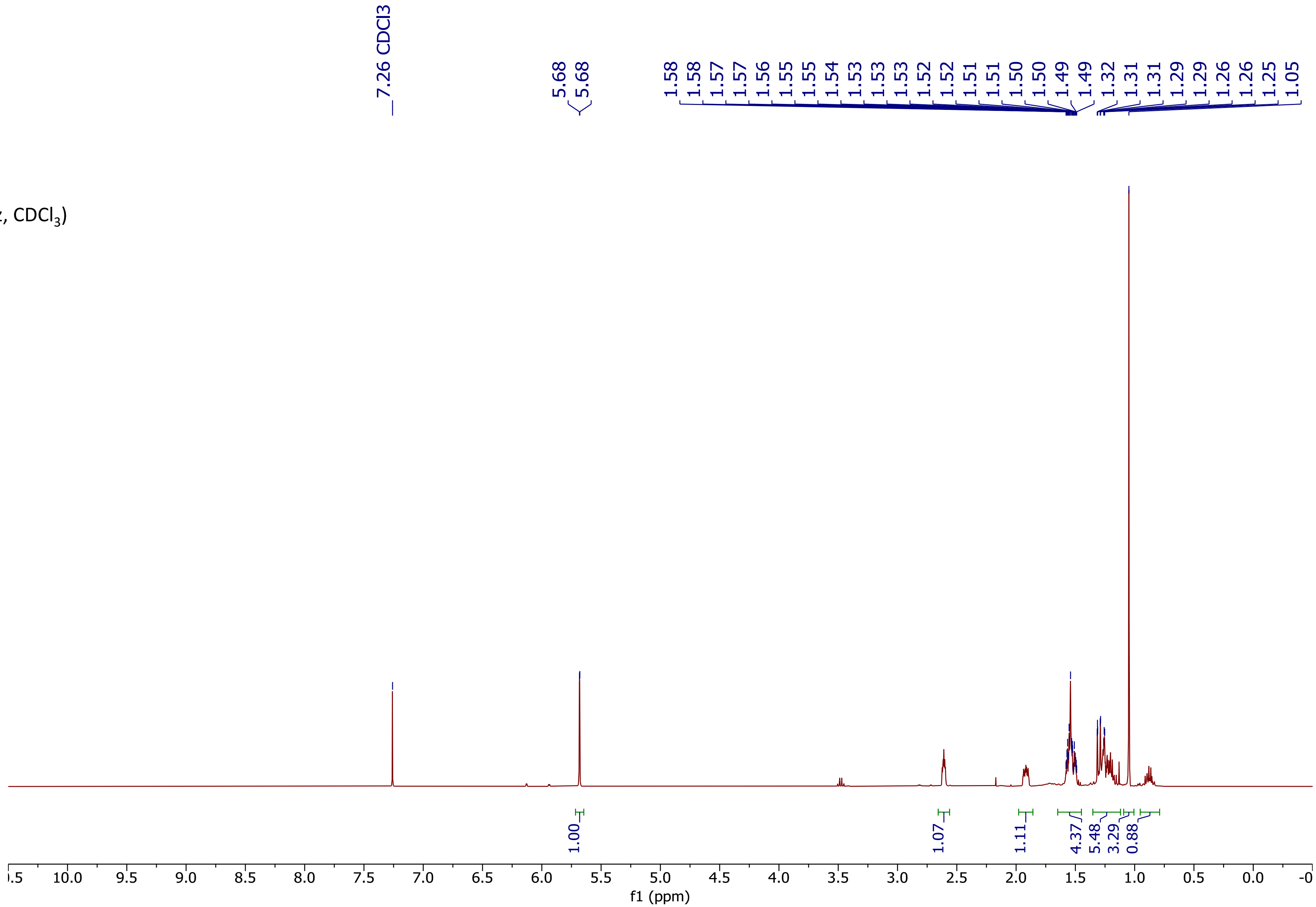

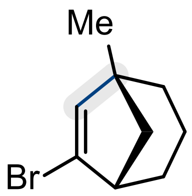

***trans*-2d**

^{13}C NMR (101 MHz, $CDCl_3$)

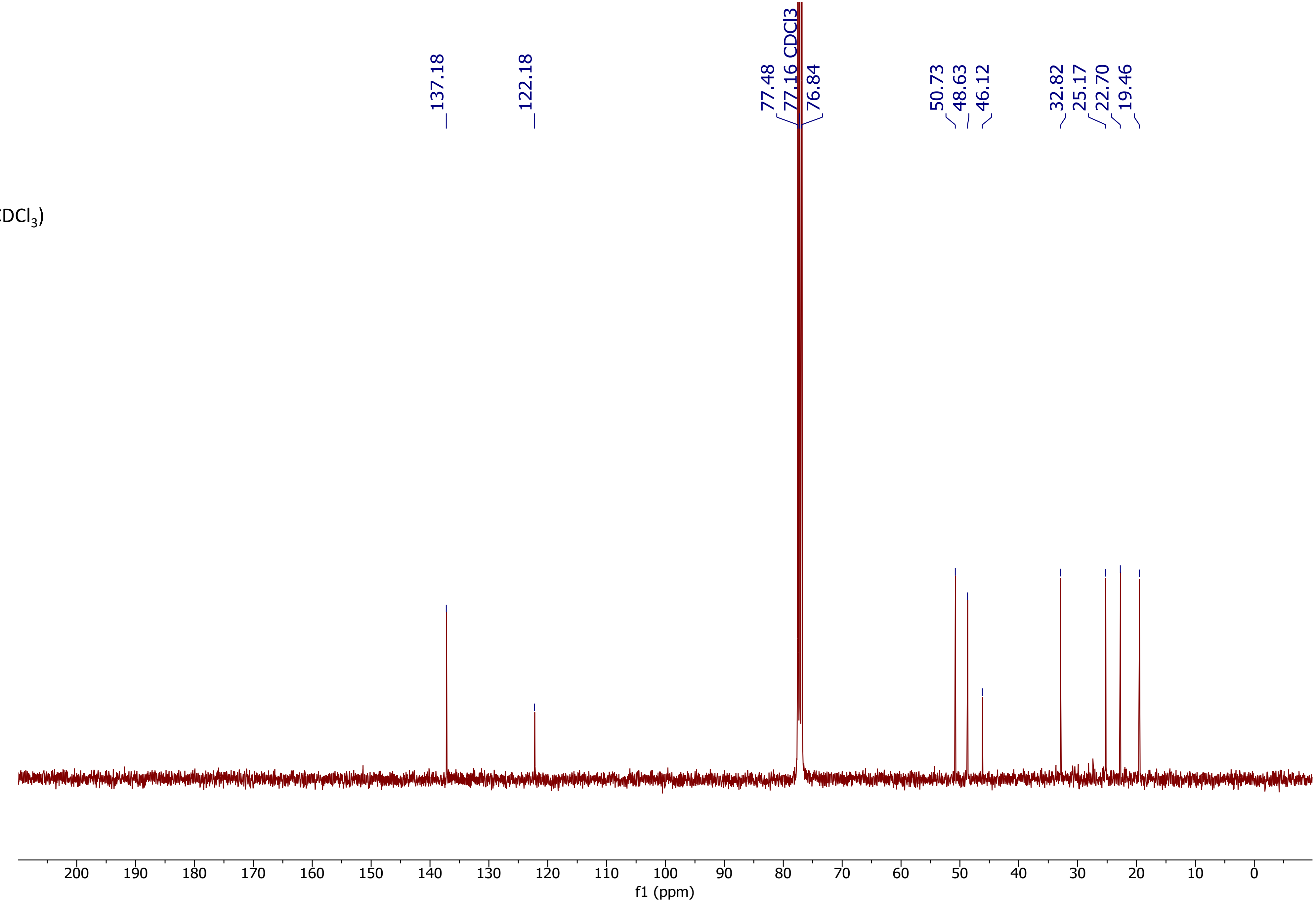

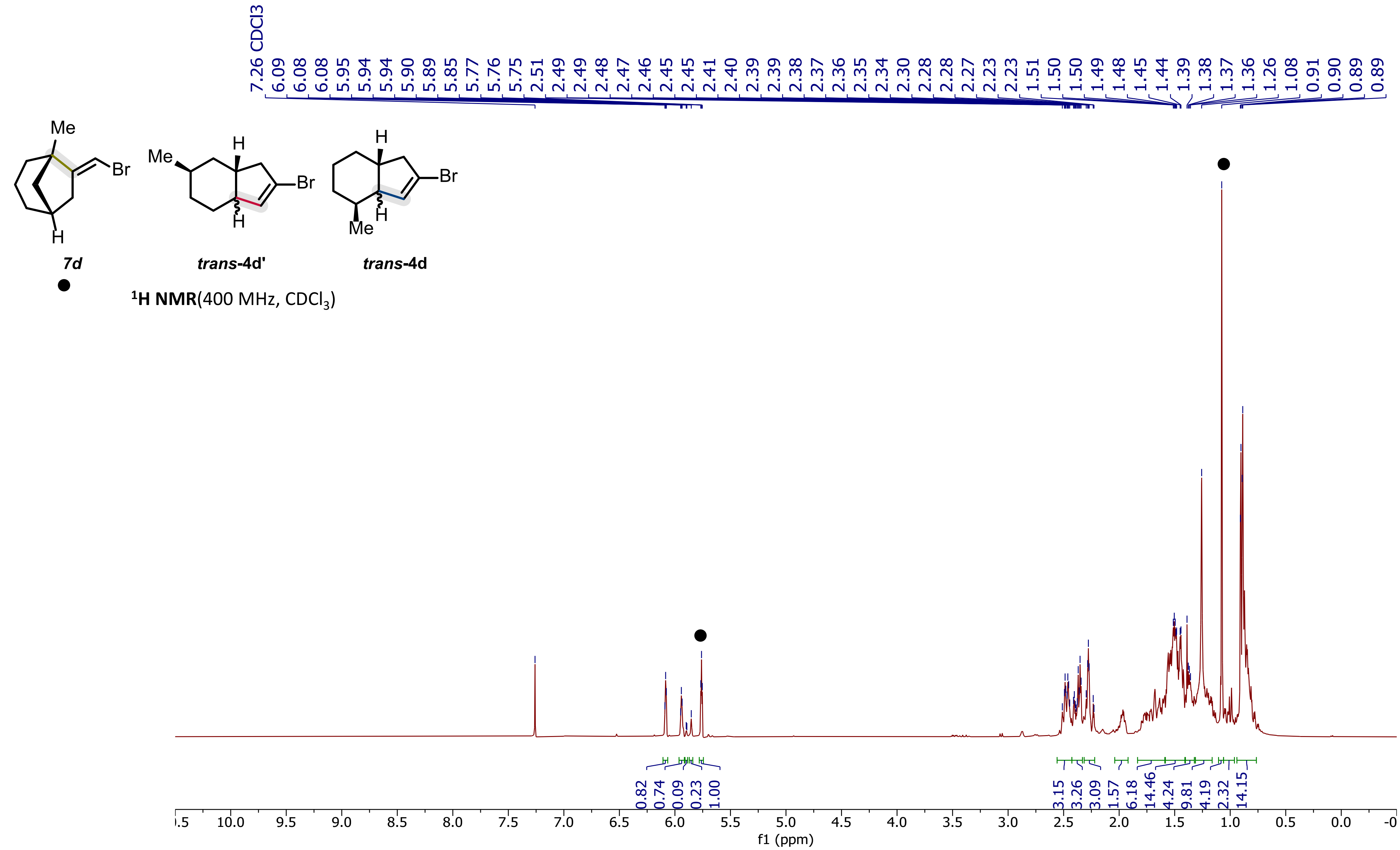
7d
trans-4d'
trans-4d
Me
Br
H
¹H NMR(400 MHz, CDCl3)
7.26 CDCl3
6.09 6.08 6.08 5.95 5.94 5.94 5.90 5.89 5.85 5.77 5.76 5.75 2.51 2.49 2.49 2.48 2.47 2.46 2.45 2.45 2.41 2.40 2.39 2.39 2.38 2.37 2.36 2.35 2.34 2.30 2.28 2.28 2.27 2.23 2.23 1.51 1.50 1.50 1.49 1.48 1.45 1.44 1.39 1.38 1.37 1.36 1.26 1.08 0.91 0.90 0.89 0.89
0.82 0.74 0.09 0.23 1.00
3.15 3.26 3.09 1.57 6.18 14.46 4.24 9.81 4.19 2.32 14.15
f1 (ppm)

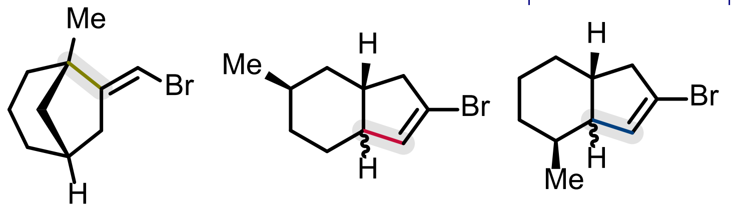

7d *trans*-**4d'** *trans*-**4d**

●

^{13}C NMR (101 MHz, $CDCl_3$)

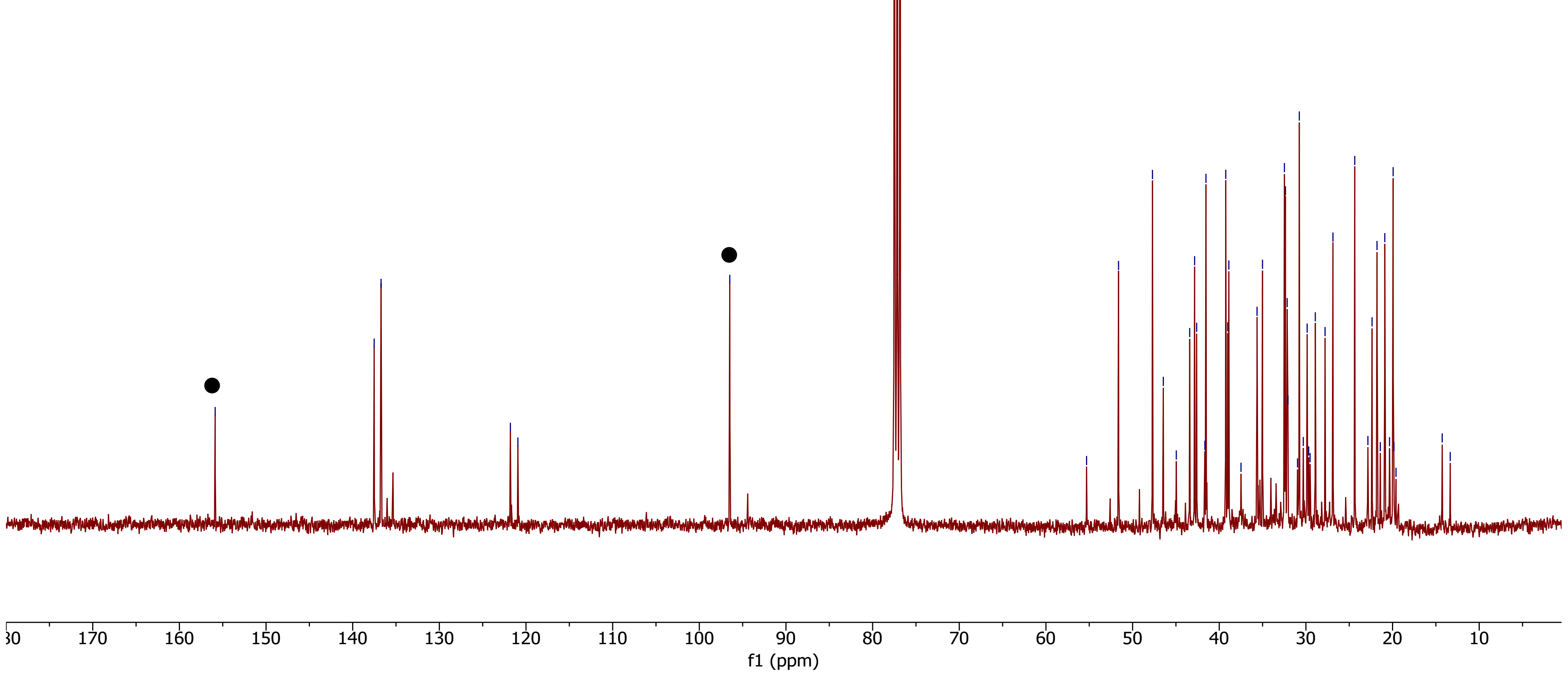

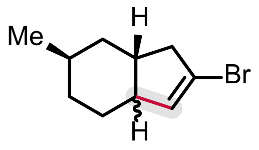

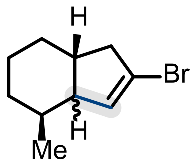

***trans*-4d'** ***trans*-4d**

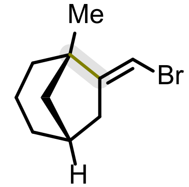

7d

COSY NMR([400, 400] MHz, $CDCl_3$)

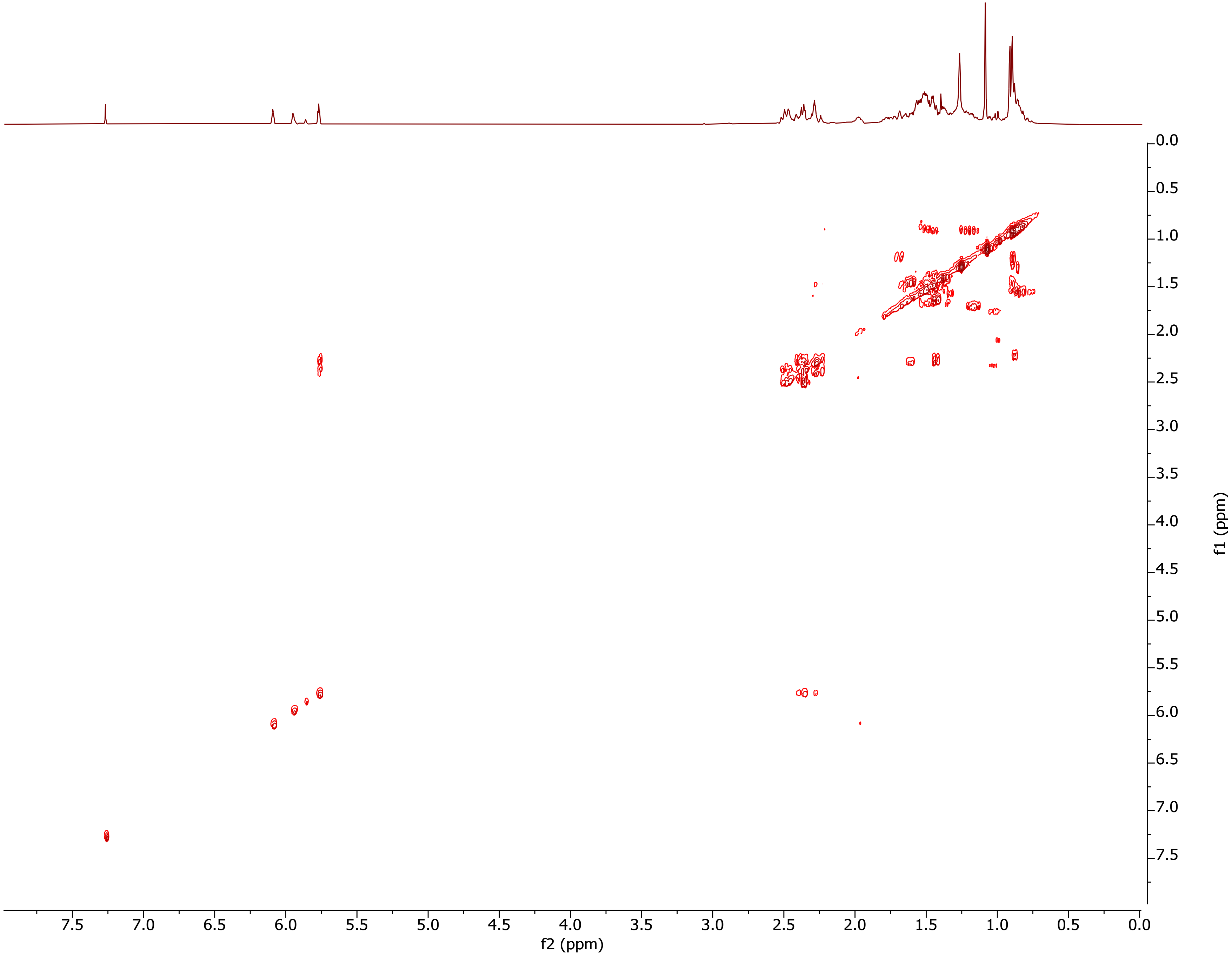

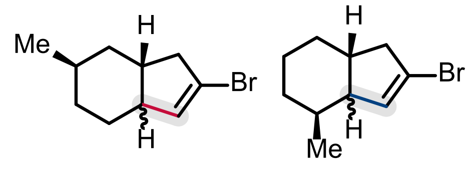

trans-4d' *trans*-4d

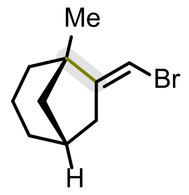

7d

HMBC NMR

HMBC NMR([400, 101] MHz, $CDCl_3$)

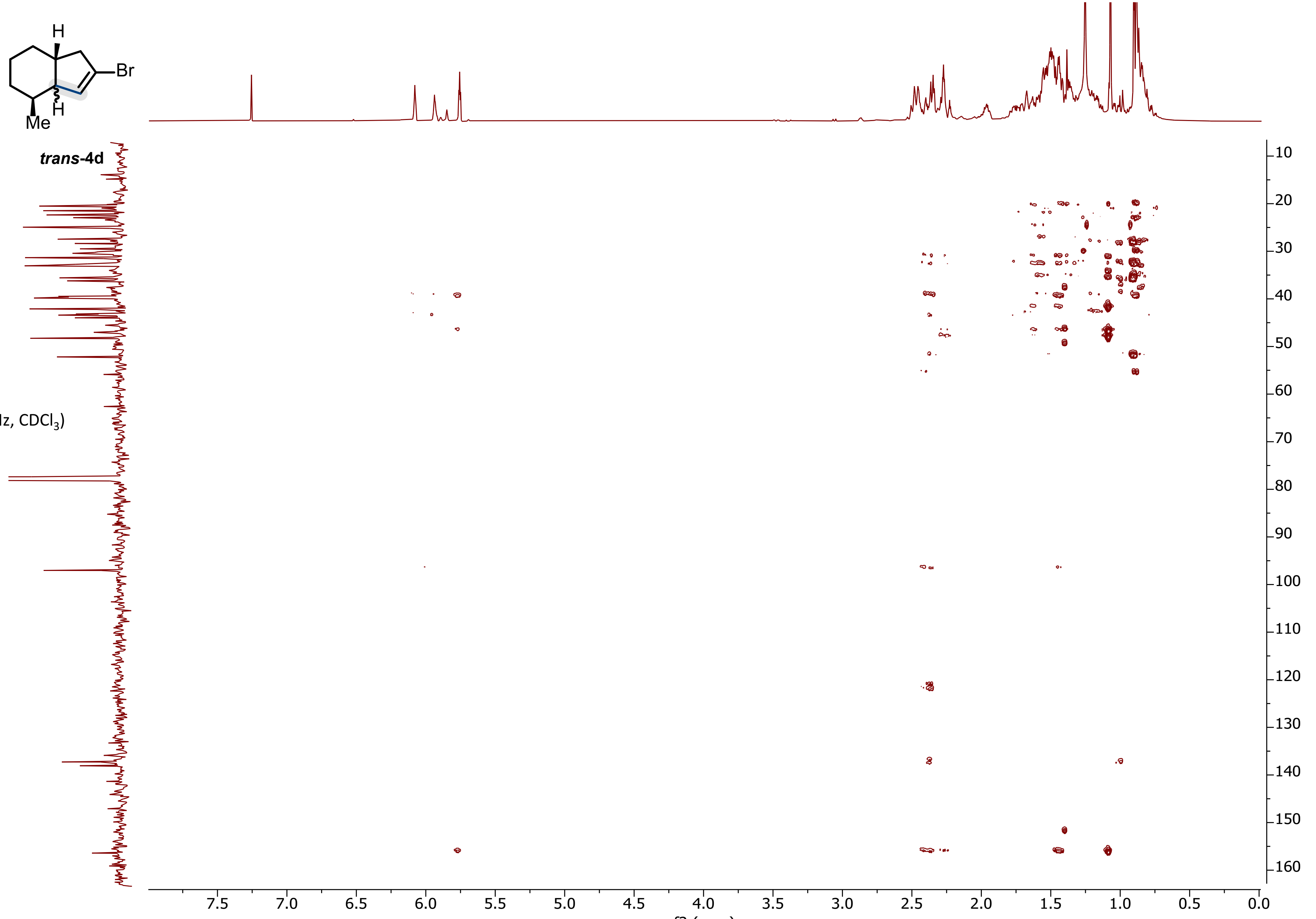

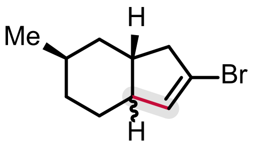

***trans*-4d'**

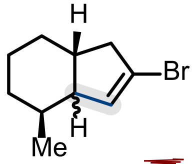

***trans*-4d**

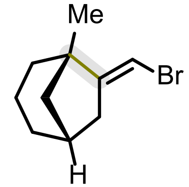

7d

HSQC NMR

HSQC NMR([400, 101] MHz, $CDCl_3$)

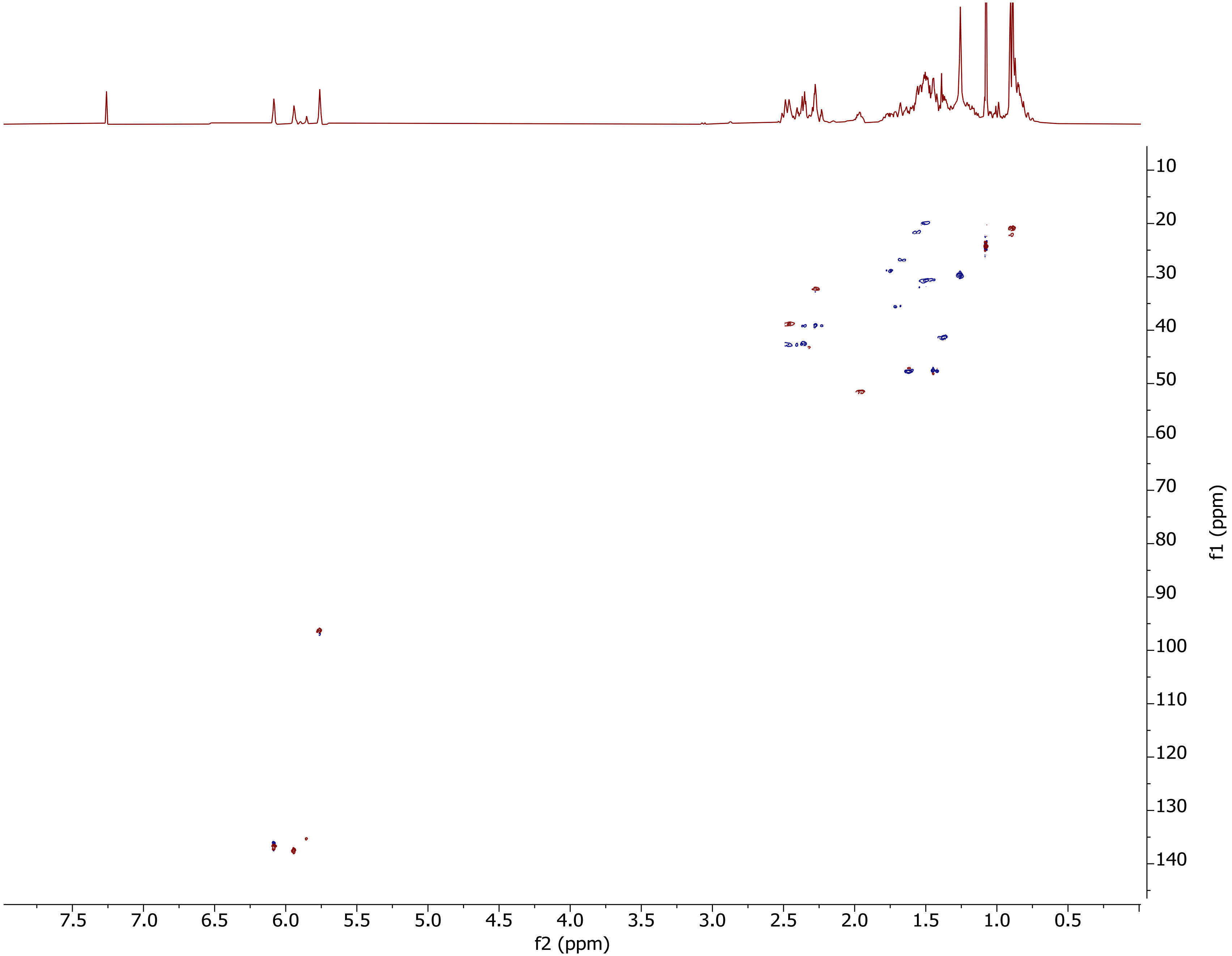

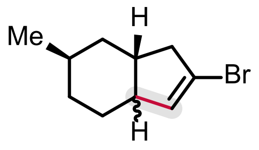

***trans*-4d'**

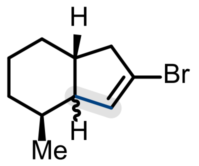

***trans*-4d**

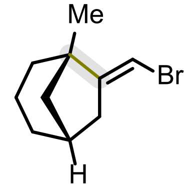

7d

TOCSY NMR

TOCSY NMR([400, 400] MHz, $CDCl_3$)

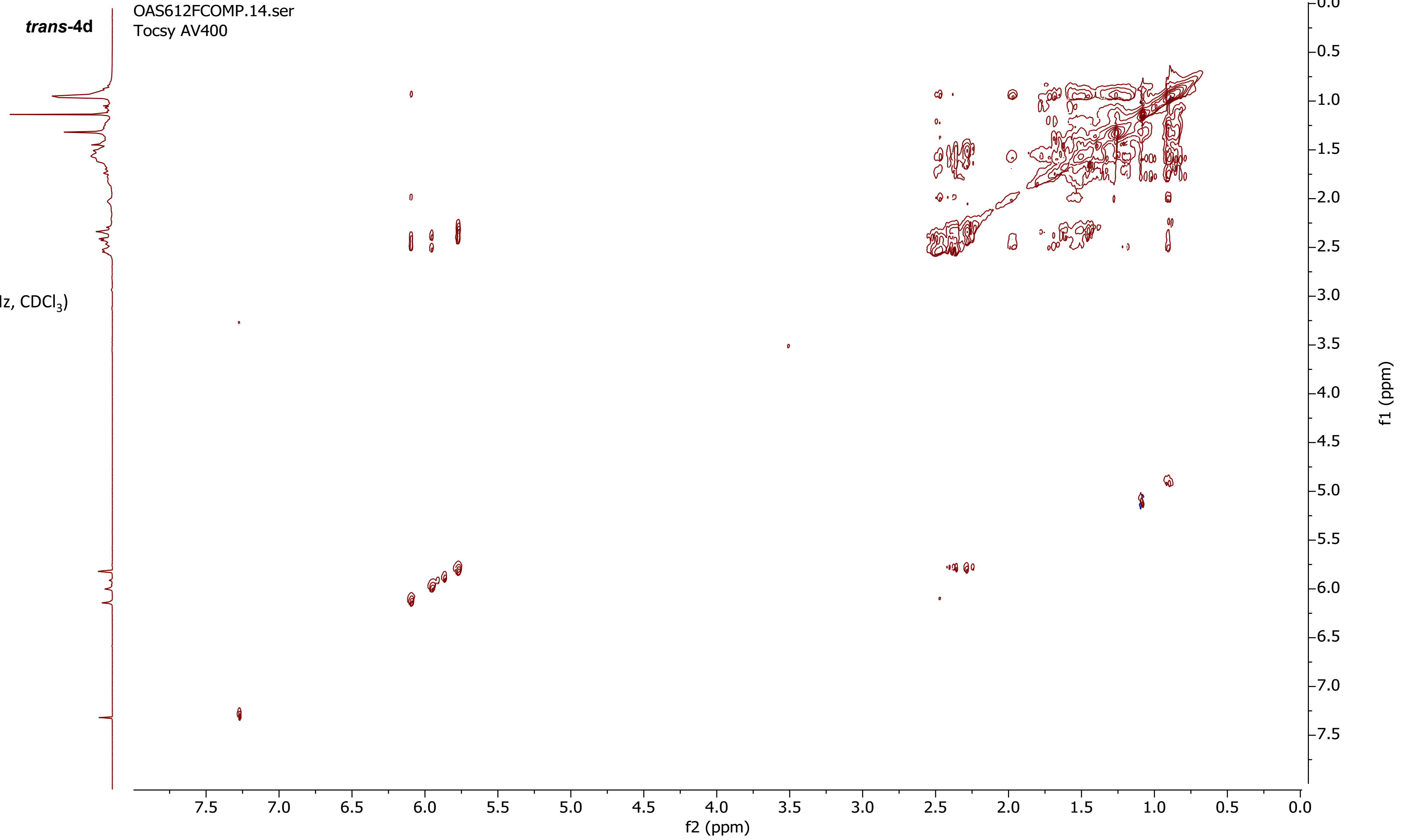

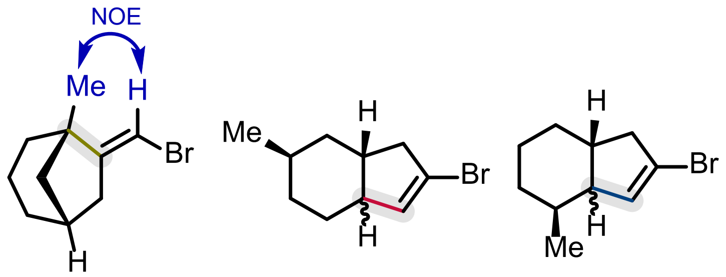

7d *trans*-**4d'** *trans*-**4d**

^{1}H NMR/SEL-NOE(400 MHz, $CDCl_3$)

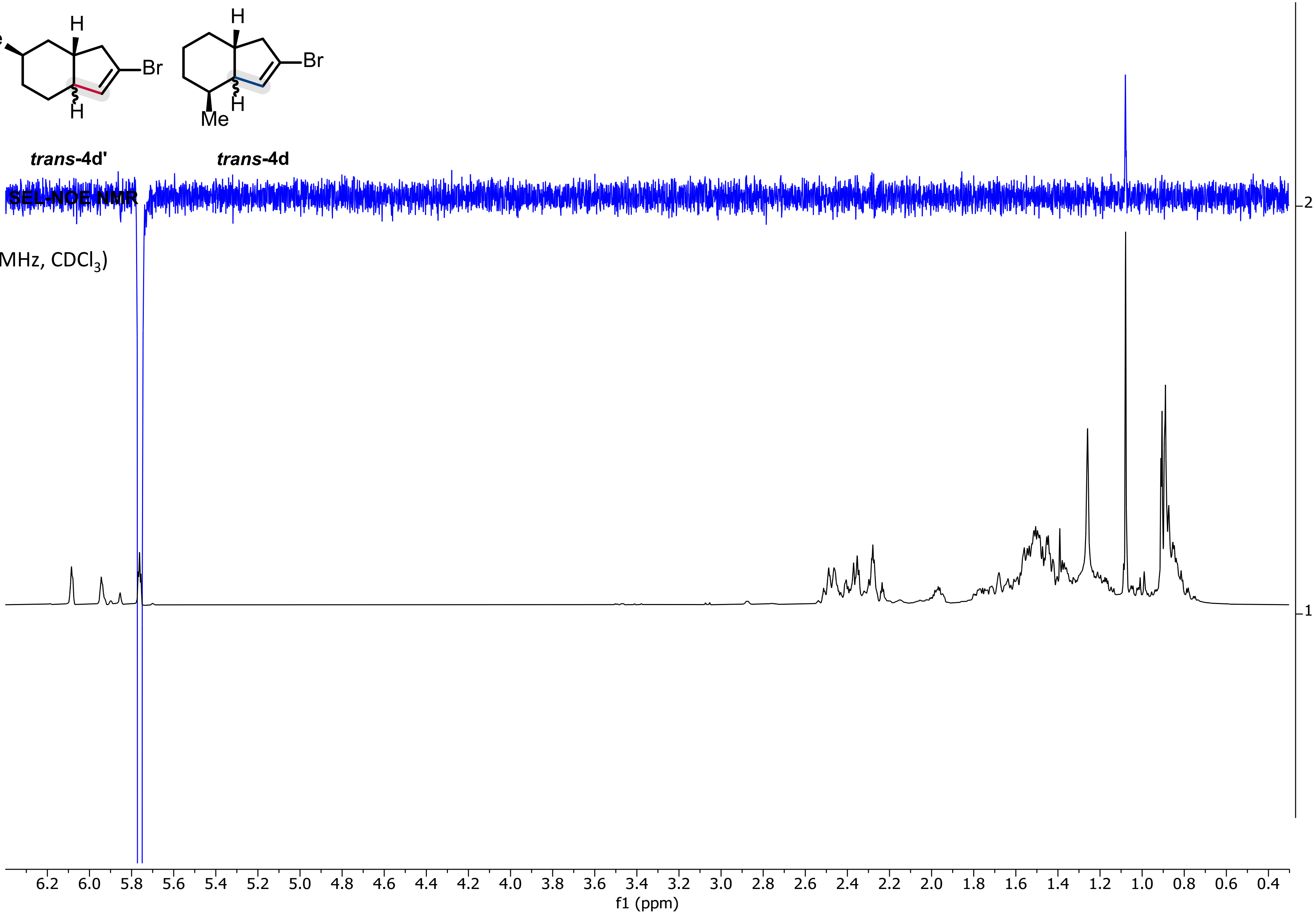

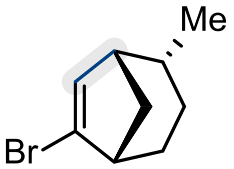

***Me*cis-2e**

¹H NMR(400 MHz, $CDCl_3$)

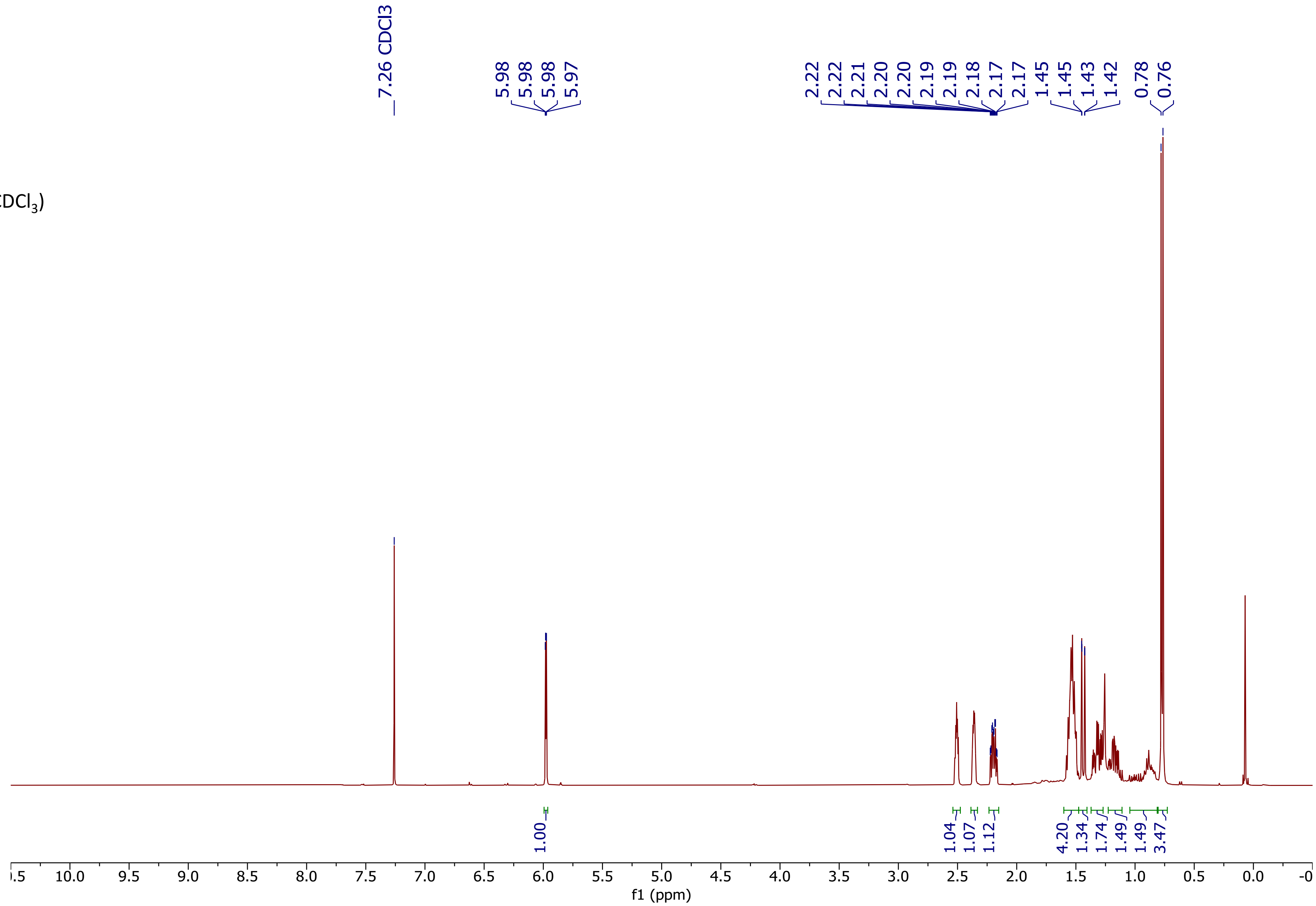

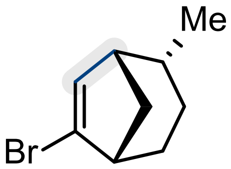

^{Me}***cis*-2e**

^{13}C NMR (101 MHz, $CDCl_3$)

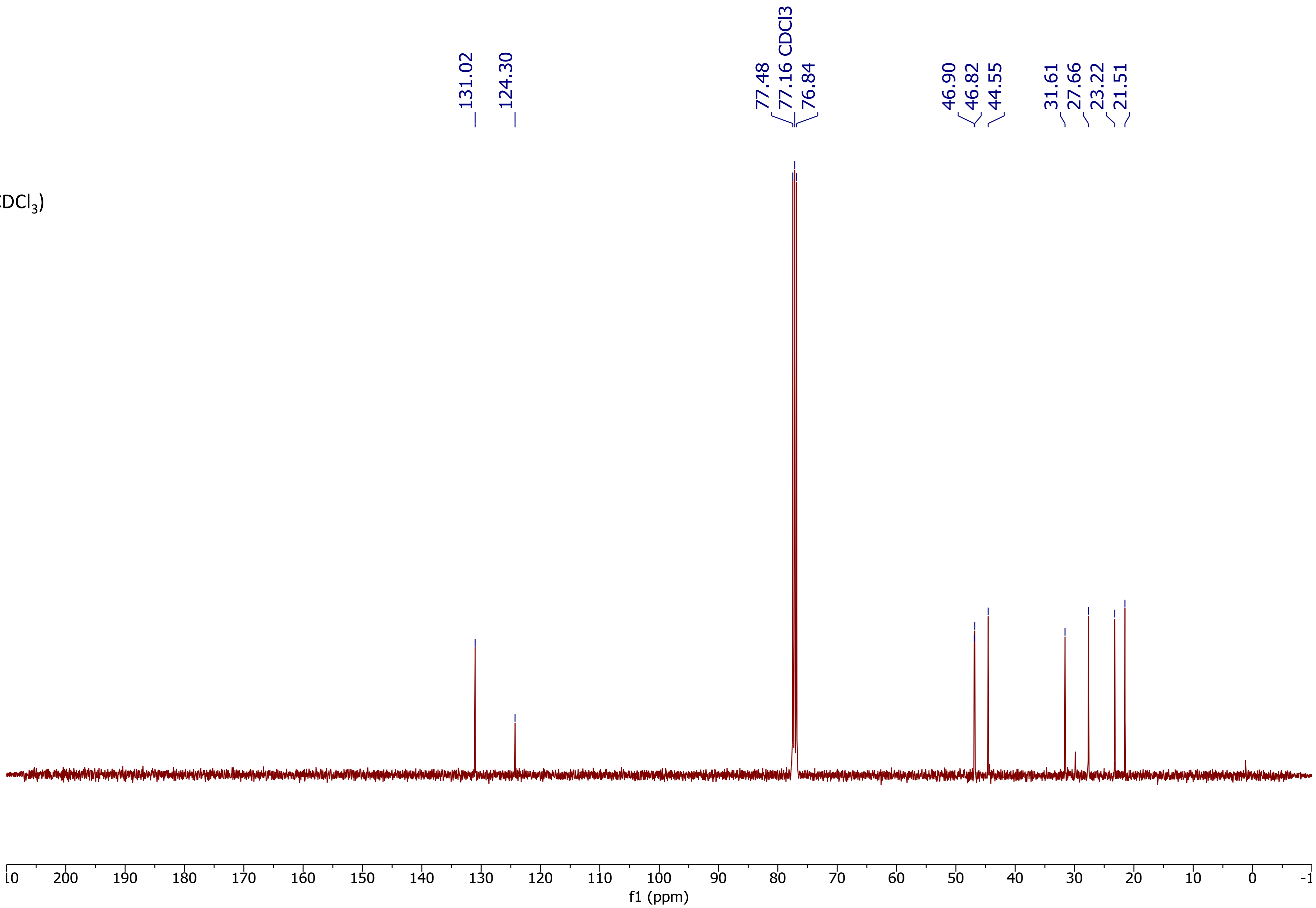

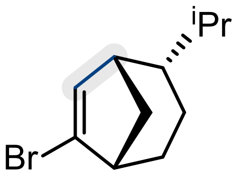

^{iPr}cis-**2e**

^{1}H NMR(400 MHz, $CDCl_3$)

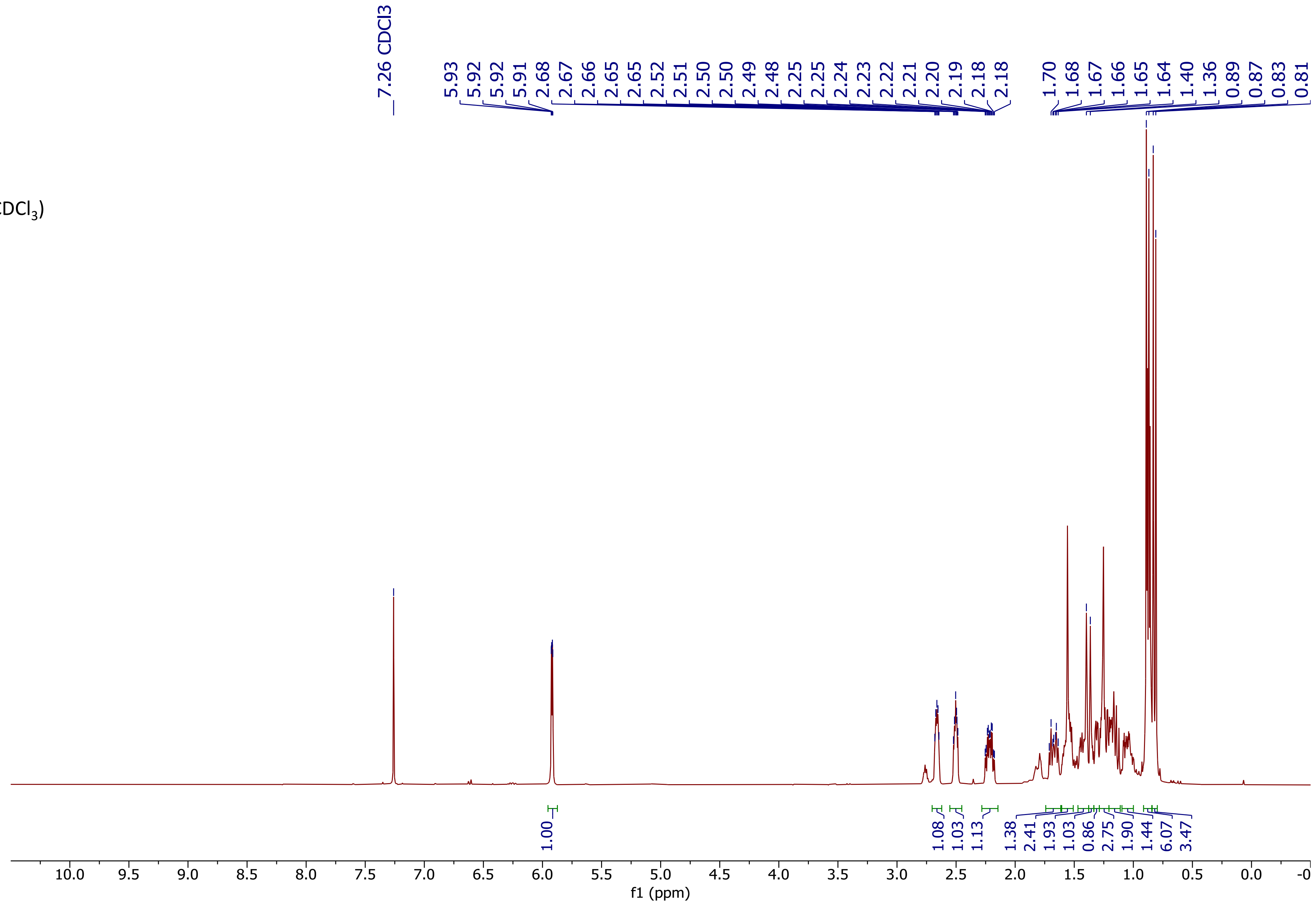

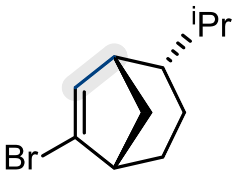

*iPr**cis*-**2e**

^{13}C NMR (101 MHz, $CDCl_3$)

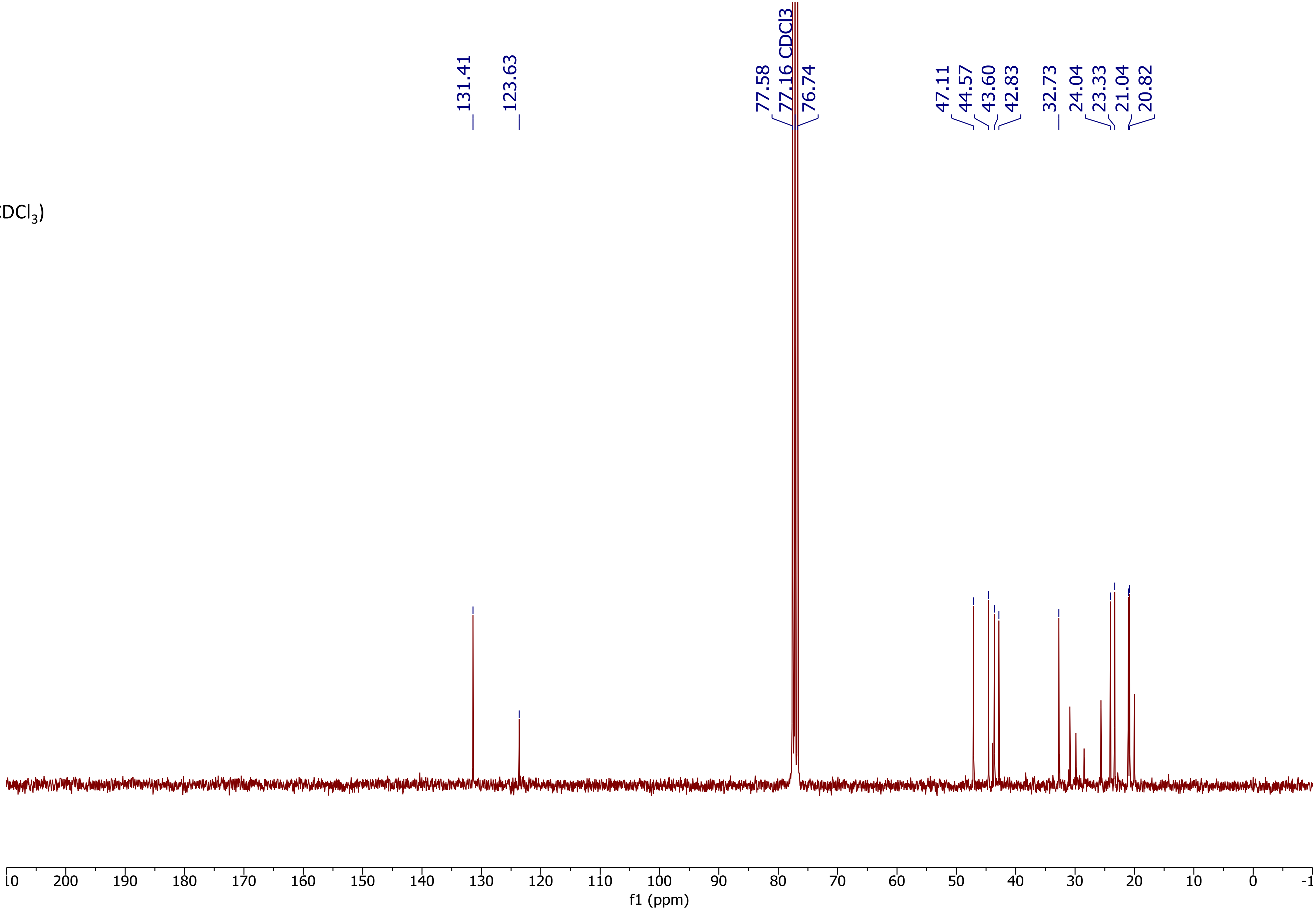

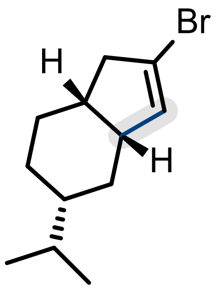

^{iPr}***cis*-4e**

^{1}**H NMR**(300 MHz, $CDCl_3$)

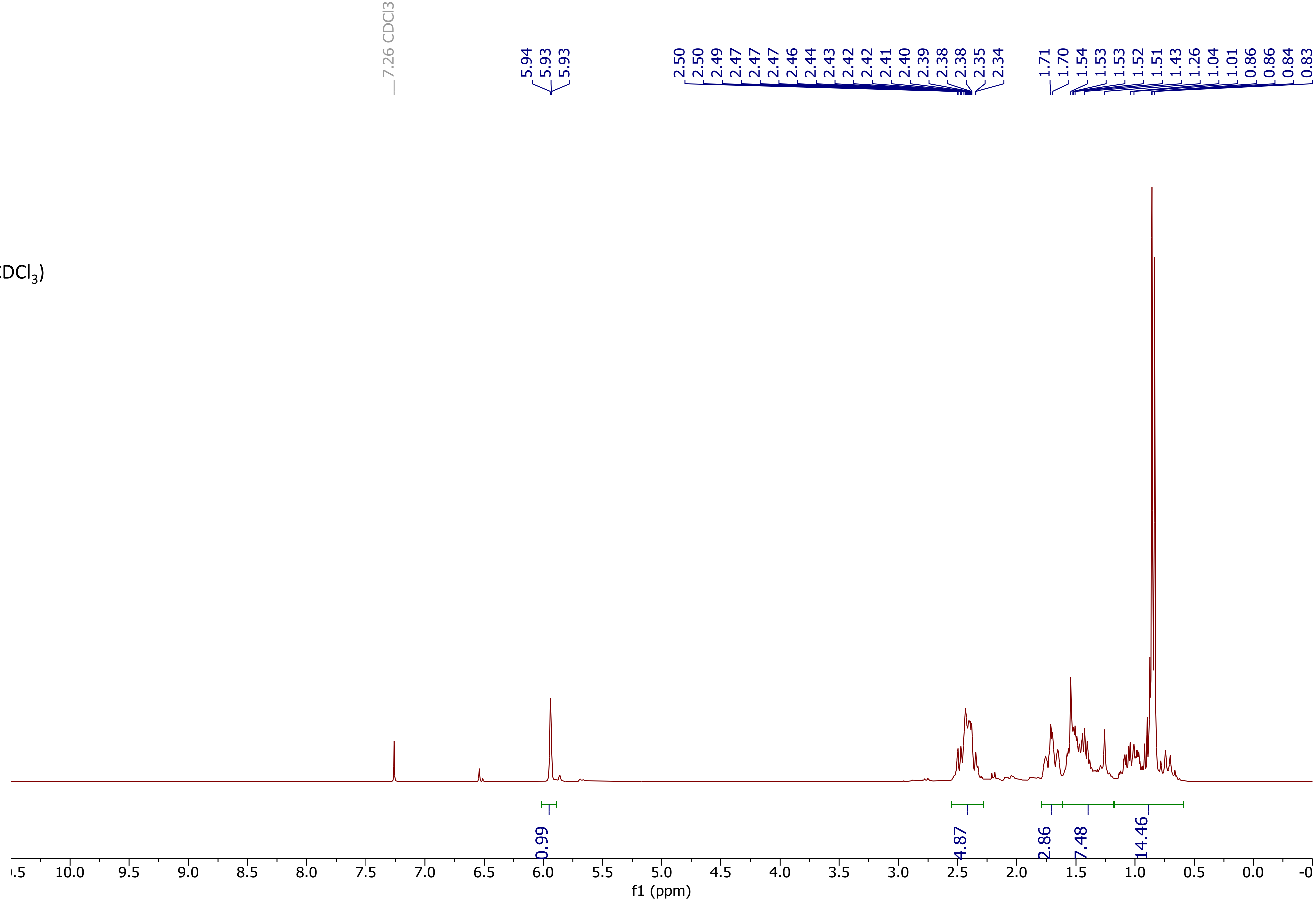

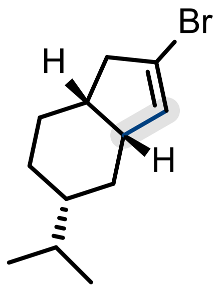

*iPr**cis*-4e**

^{13}C NMR (75 MHz, $CDCl_3$)

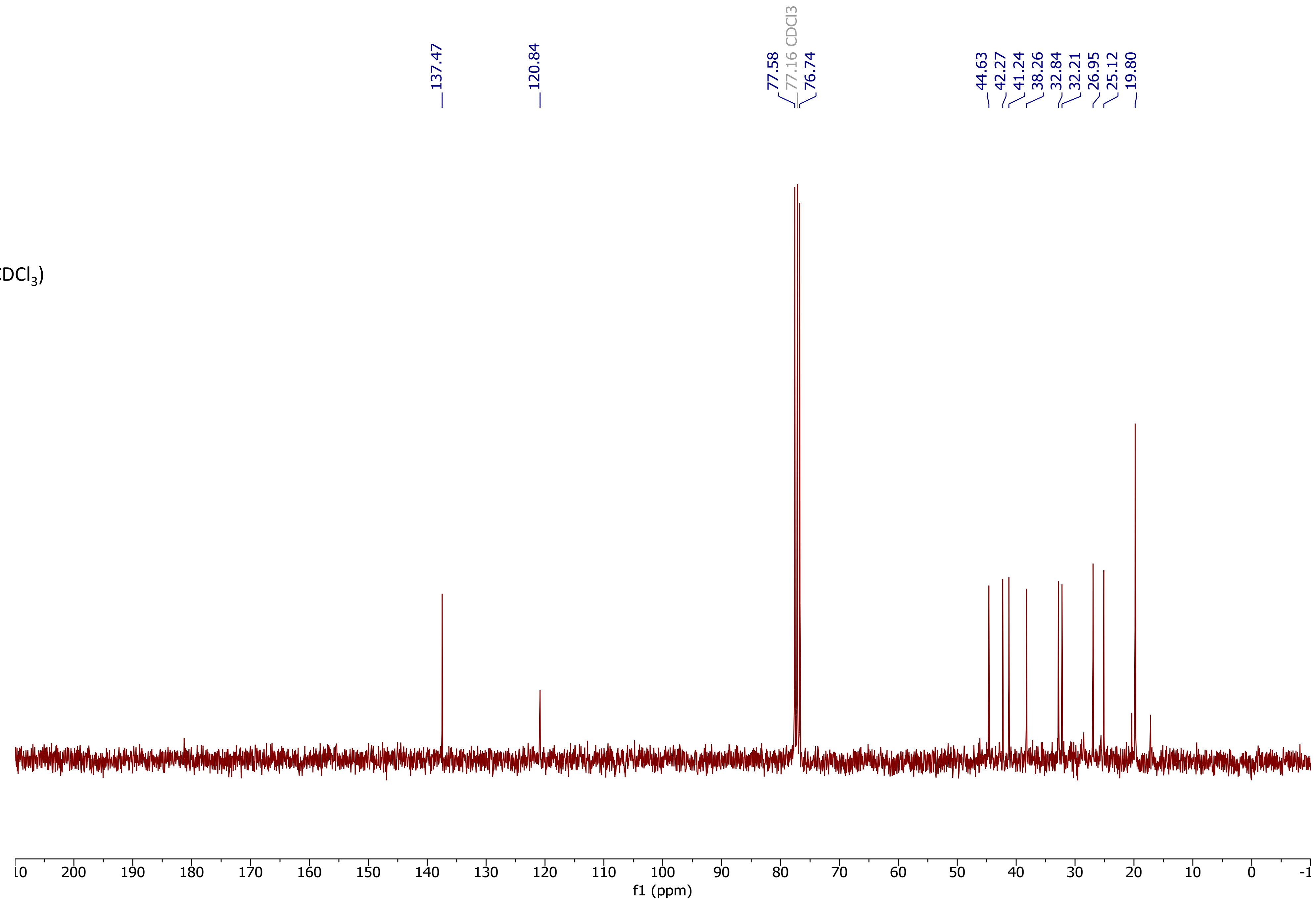

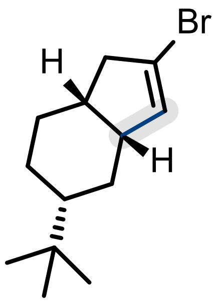

^{tBu}*cis*-**4e**

^{1}H NMR(300 MHz, $CDCl_3$)

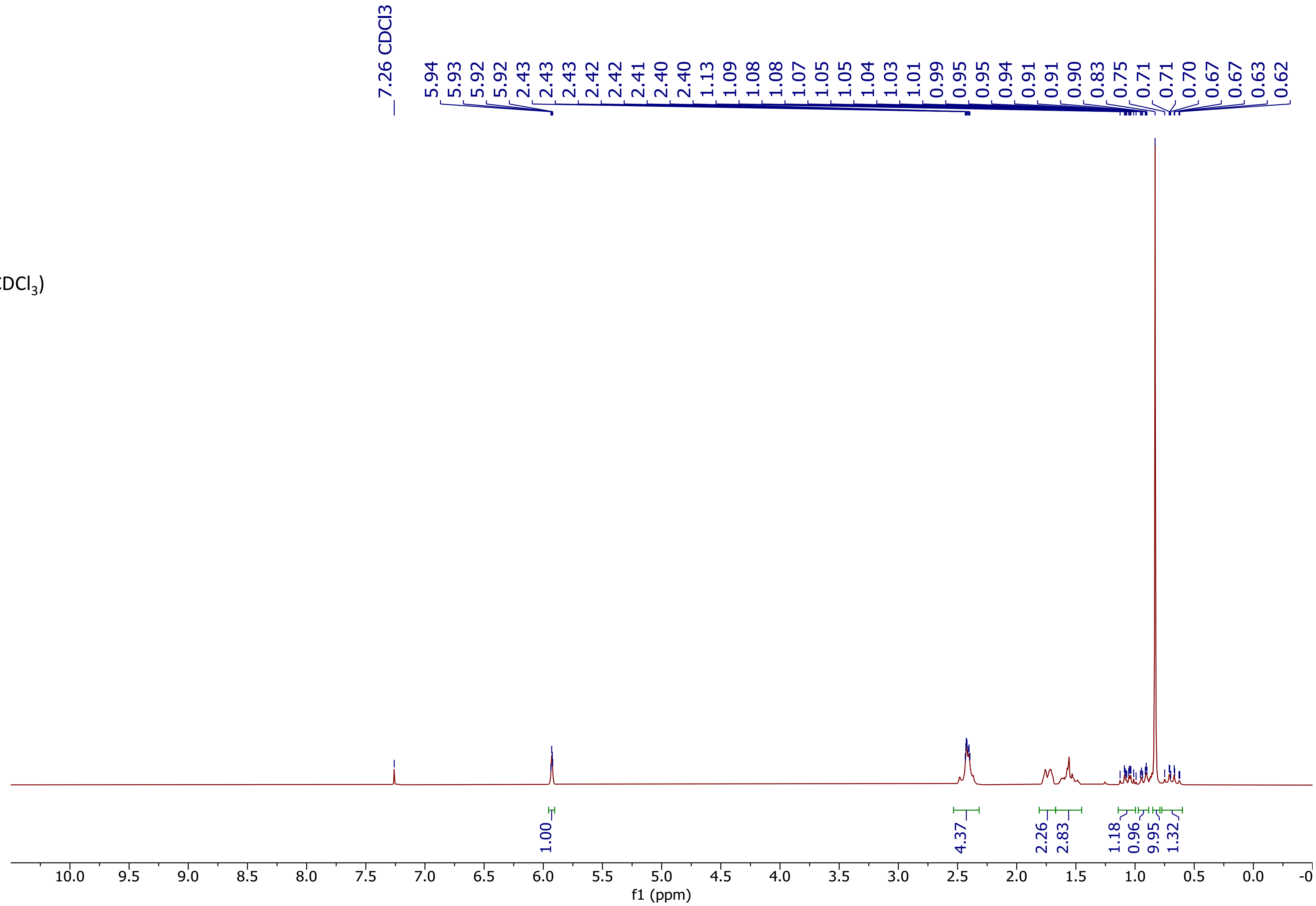

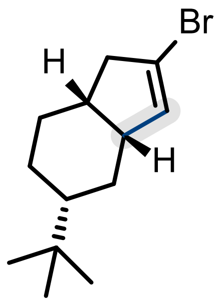

^{tBu}***cis*-4e**

^{13}C NMR (75 MHz, $CDCl_3$)

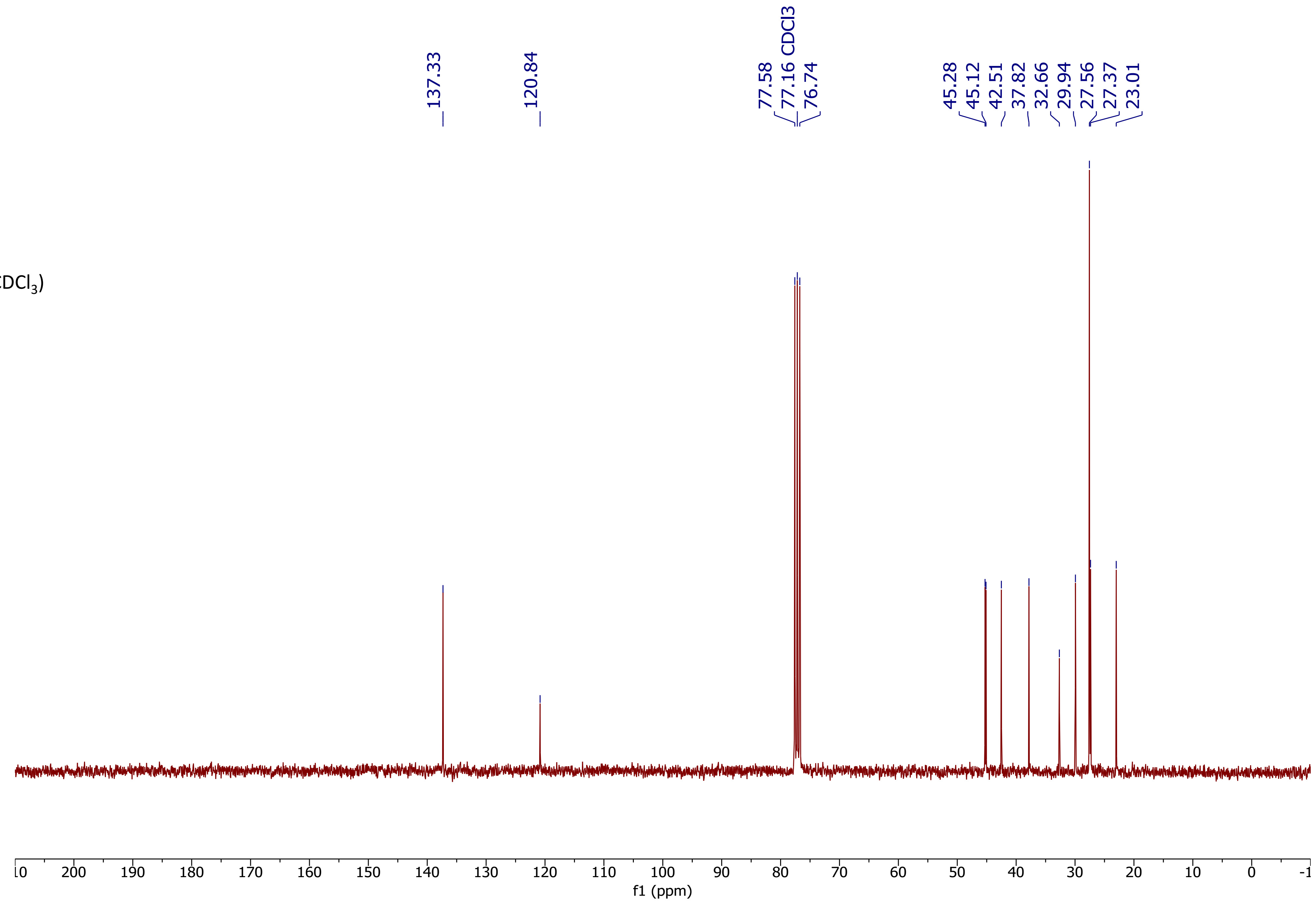

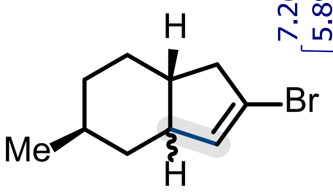

***Me*trans-4e**

^{1}H NMR(400 MHz, $CDCl_3$)

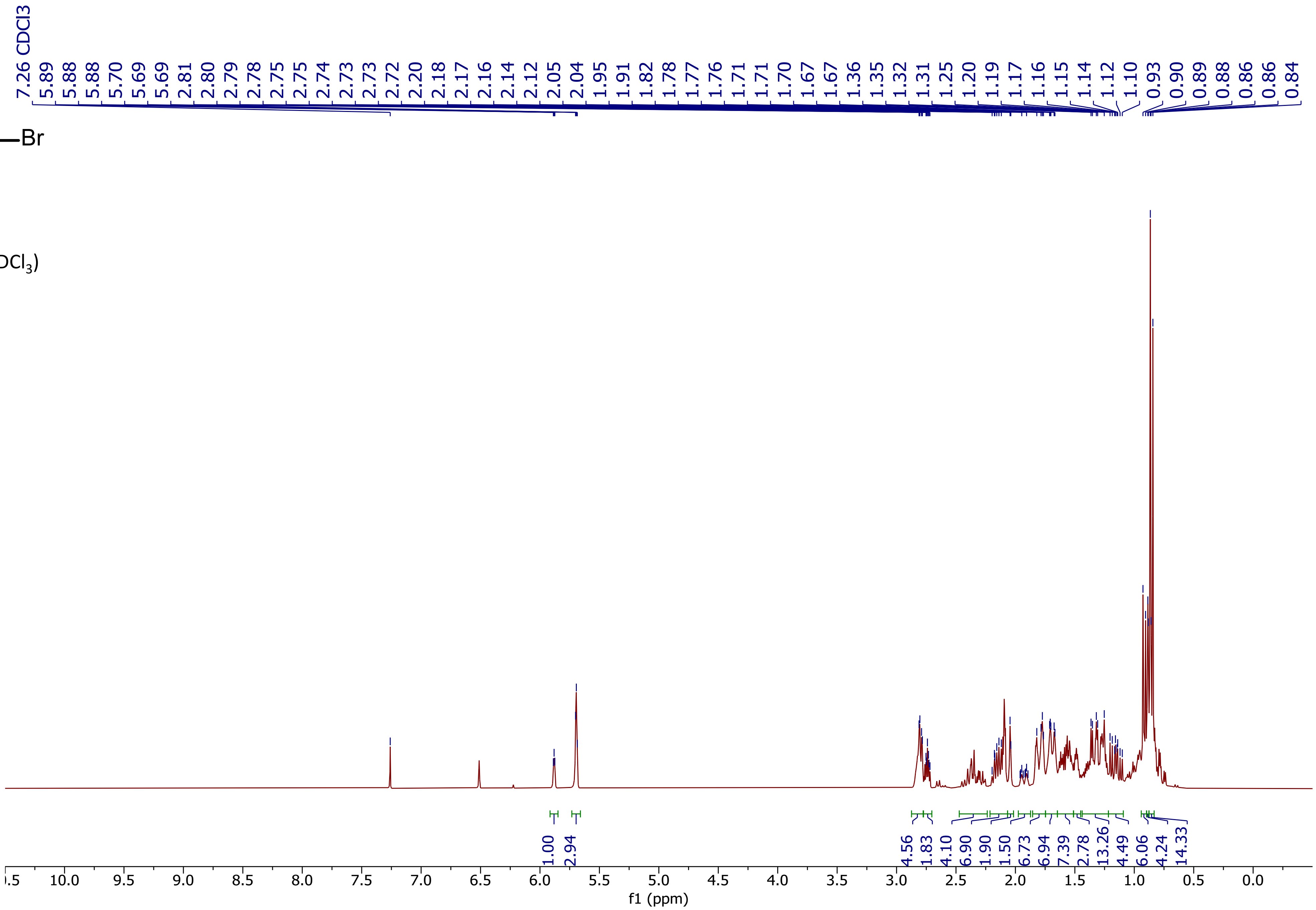

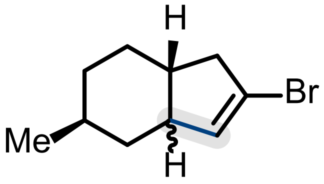

^{Me}*trans*-**4e**

^{13}C NMR (101 MHz, $CDCl_3$)

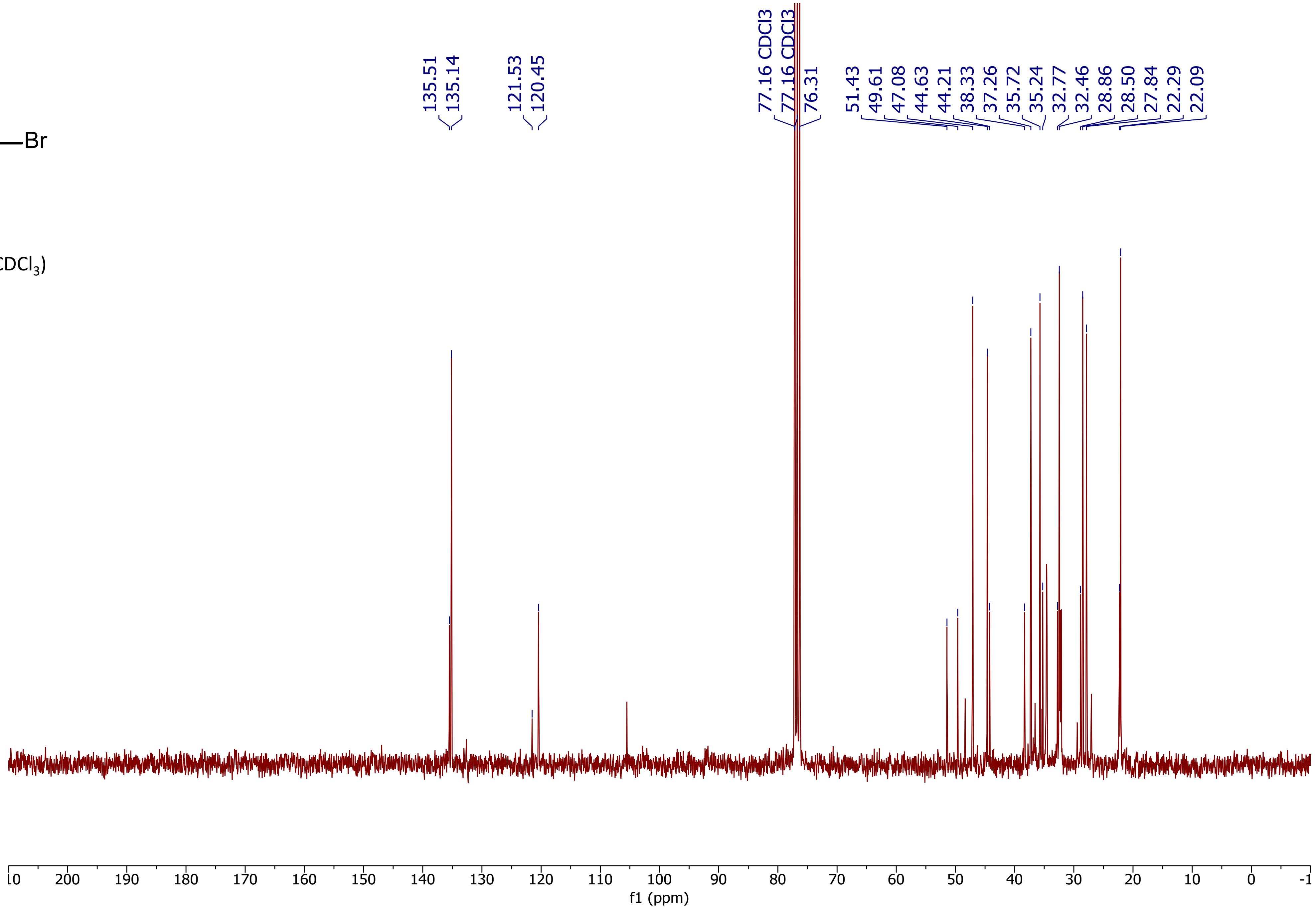

CRUDES

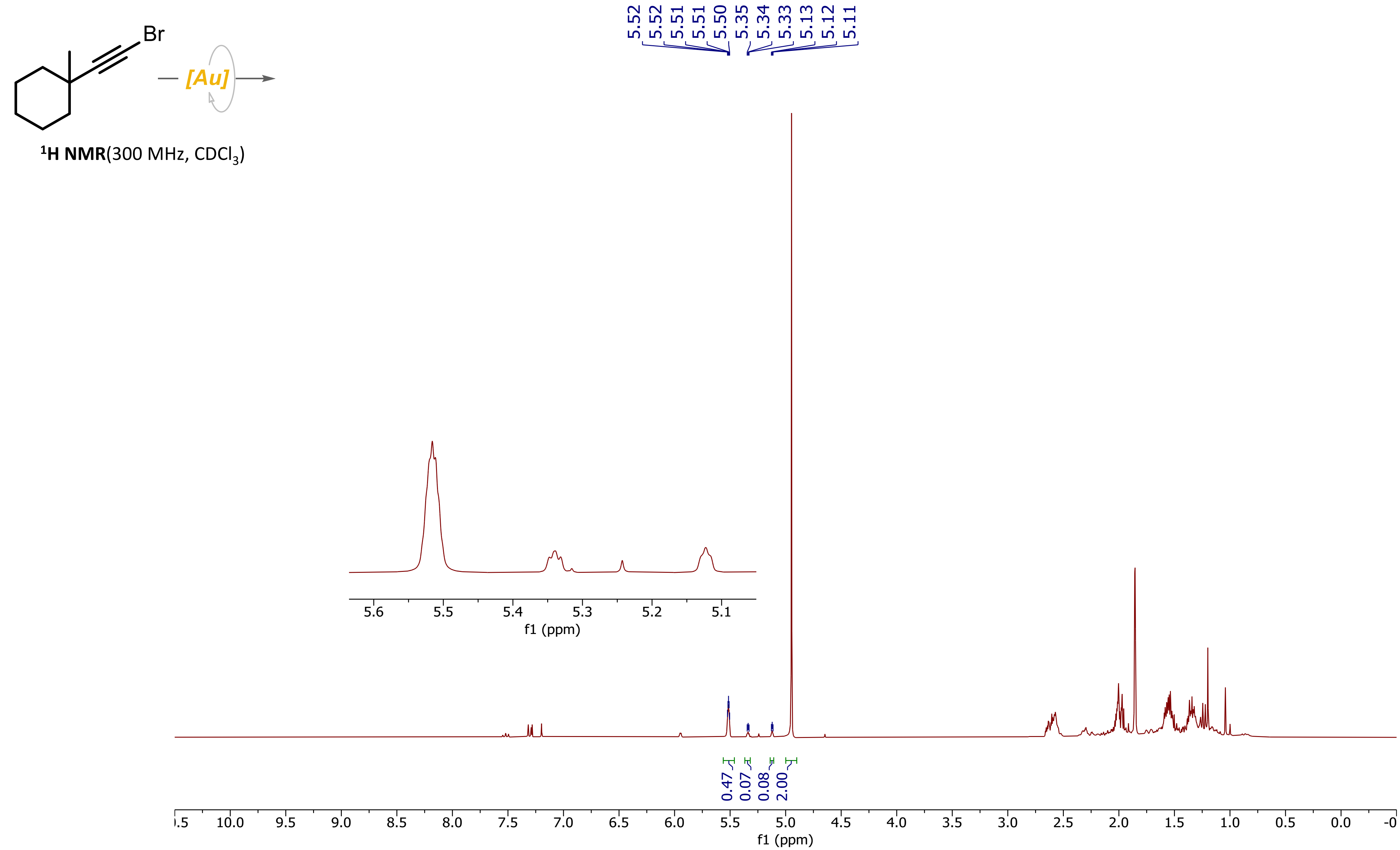
Br
[Au]
^{1}H NMR(300 MHz, CDCl$_3$)
5.52
5.52
5.51
5.51
5.50
5.35
5.34
5.33
5.13
5.12
5.11
0.47
0.07
0.08
2.00
f1 (ppm)

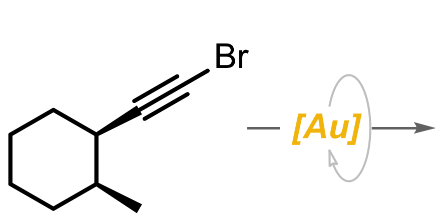

^{1}**H NMR**(300 MHz, $CDCl_3$)

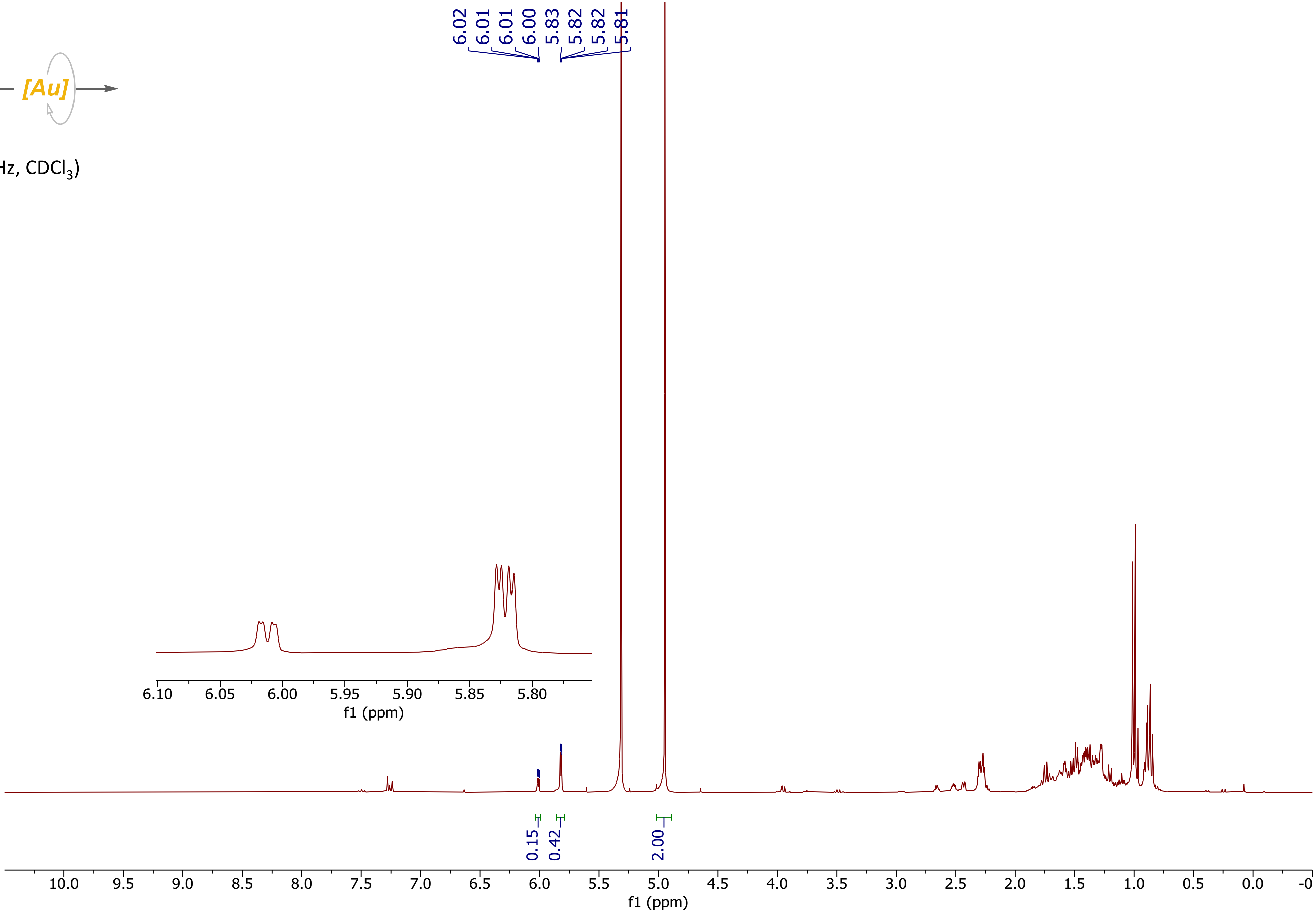

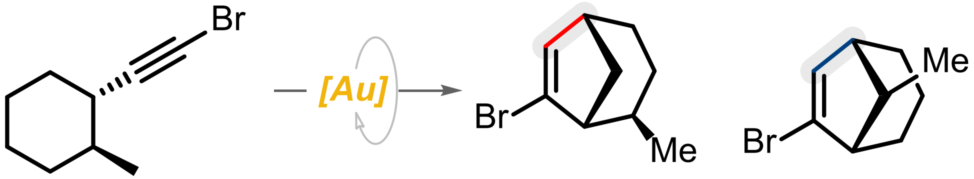

***trans*-2c** ***trans*-2c'**

^{1}H NMR(300 MHz, $CDCl_3$)

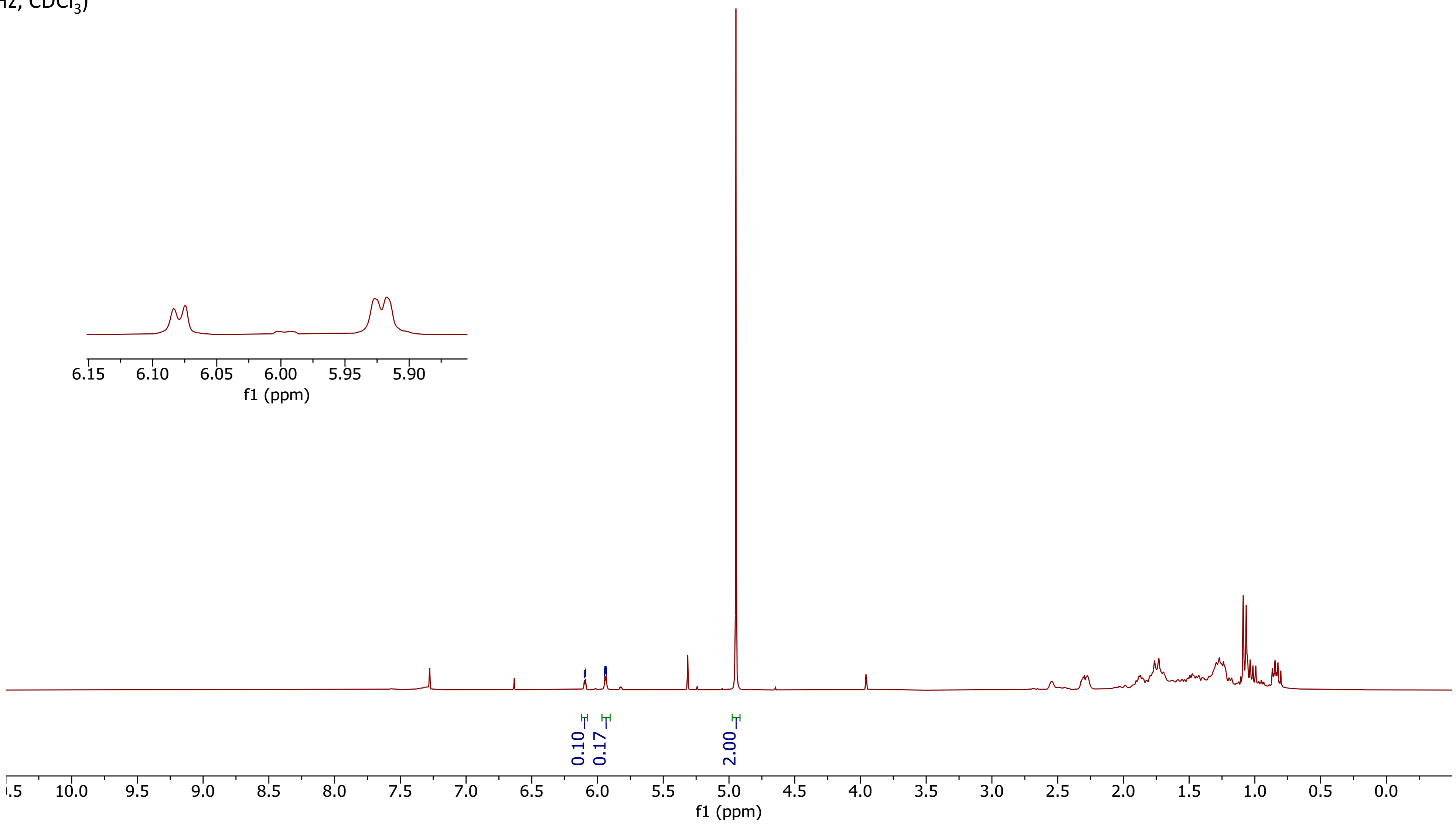

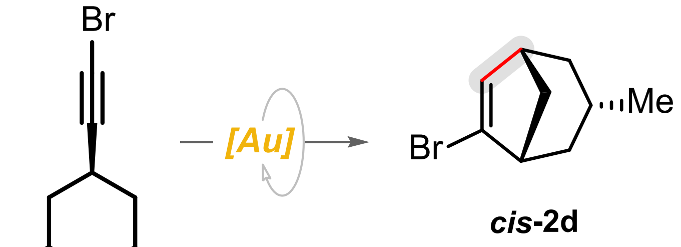

[1]**H NMR**(300 MHz, $CDCl_3$)

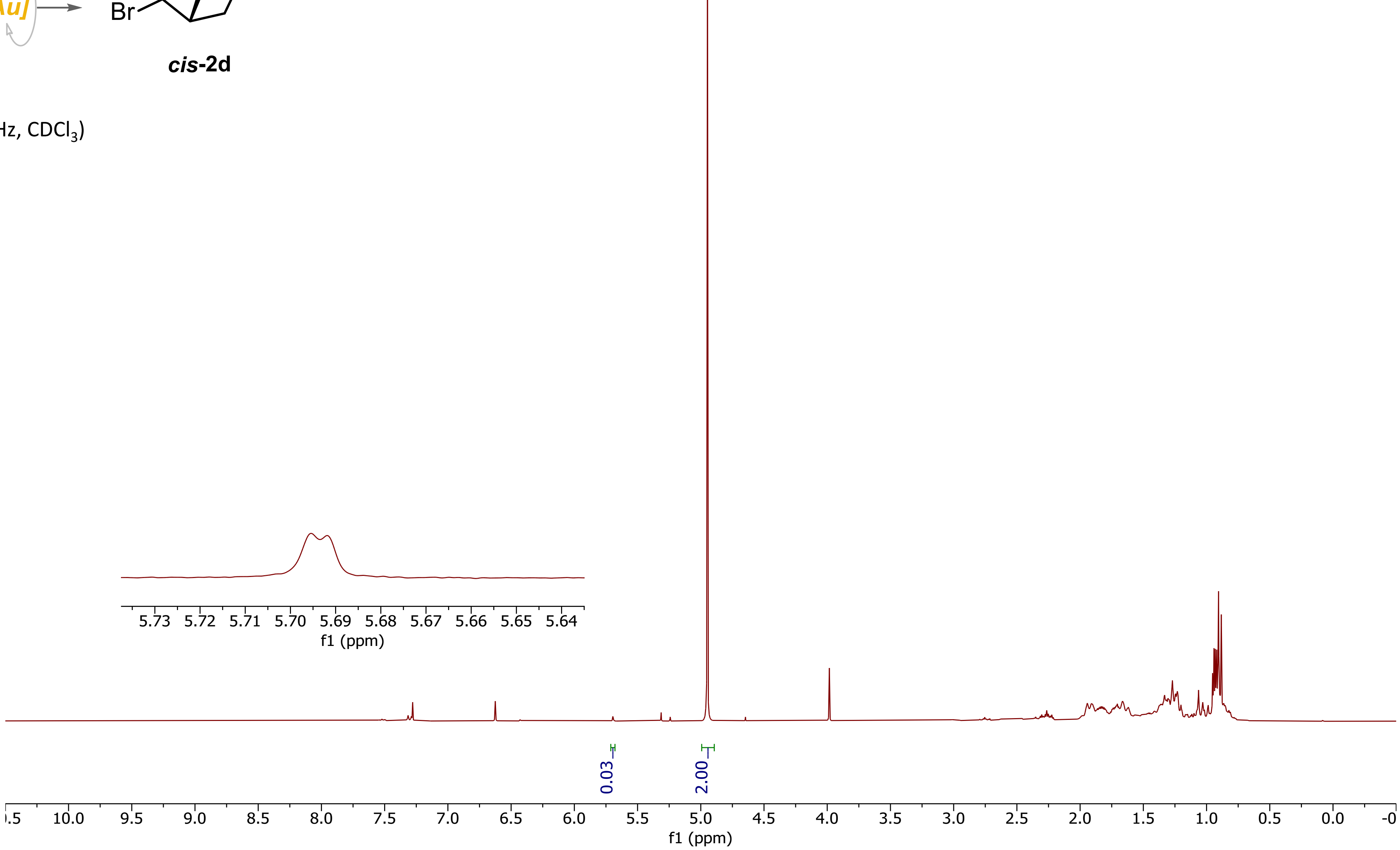

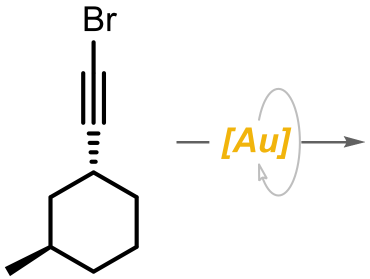

^{1}**H NMR**(300 MHz, $CDCl_3$)

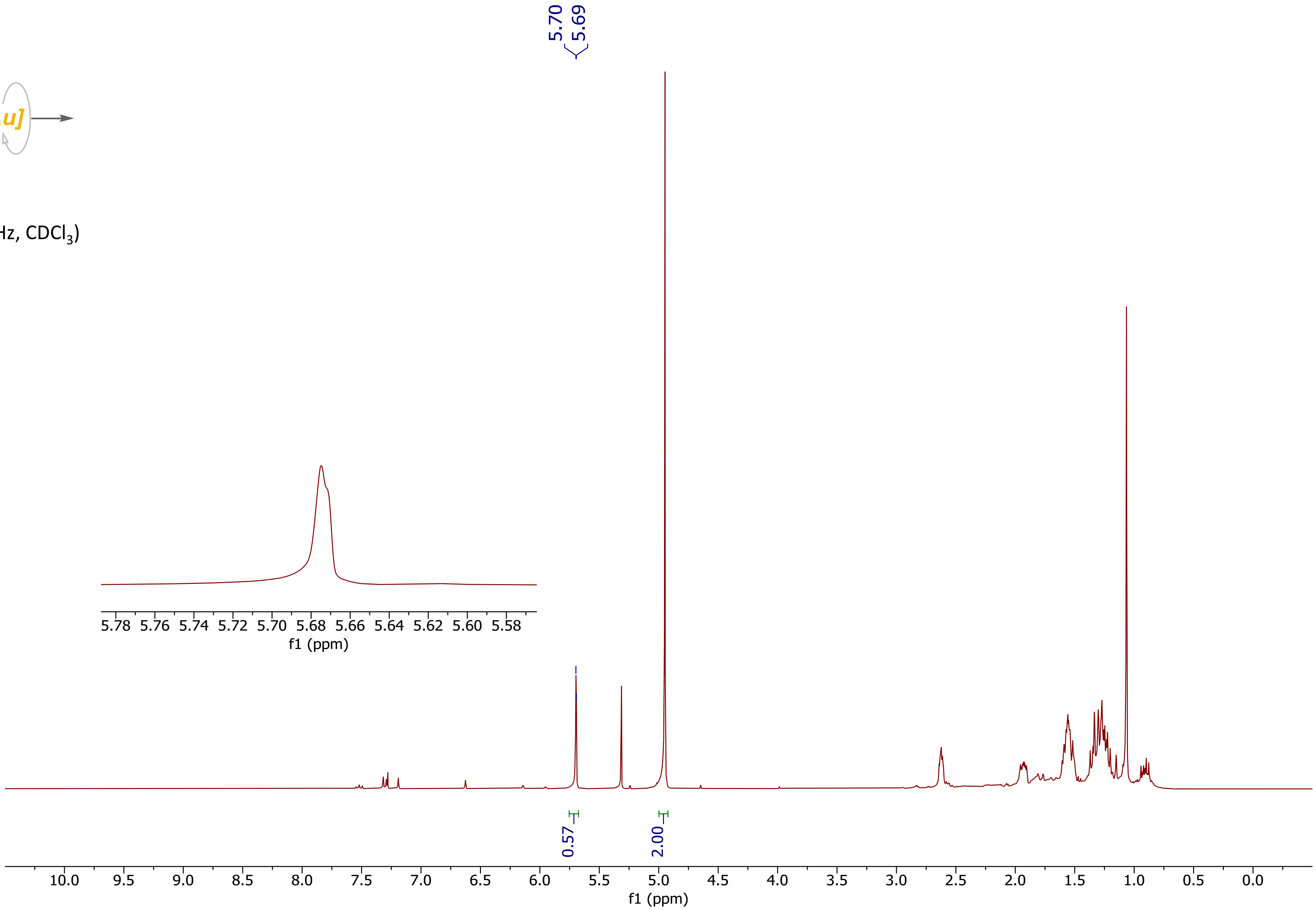

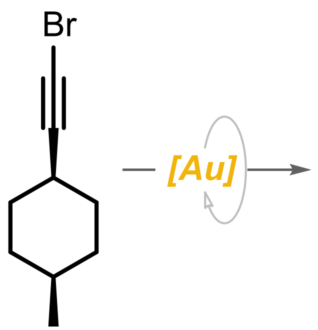

^{1}H **NMR**(300 MHz, $CDCl_3$)

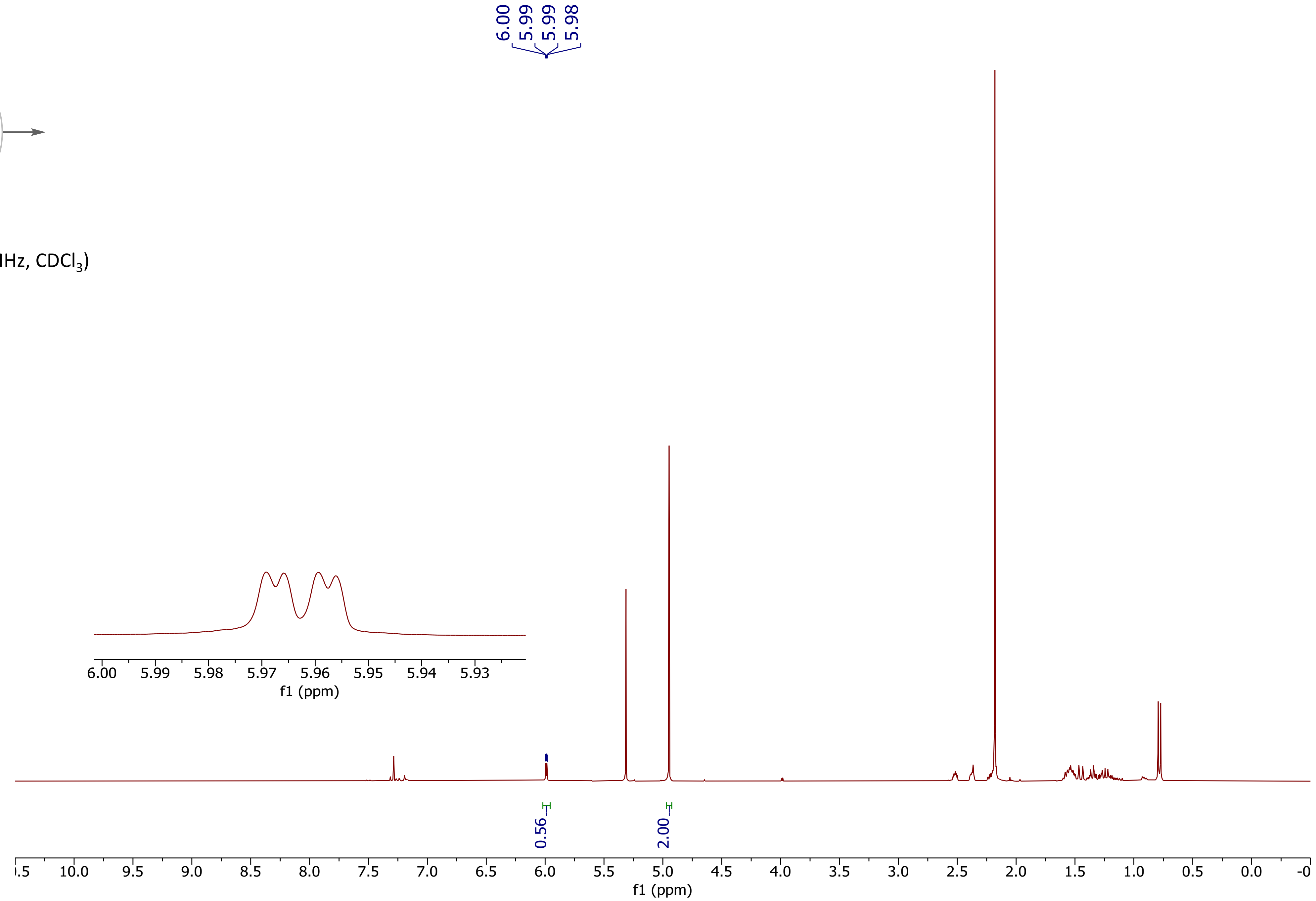

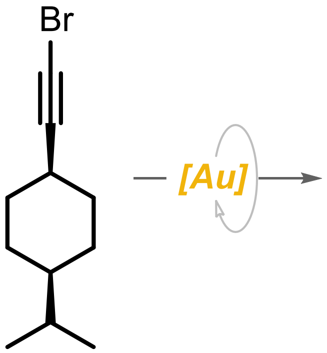

^{1}H NMR(300 MHz, $CDCl_3$)

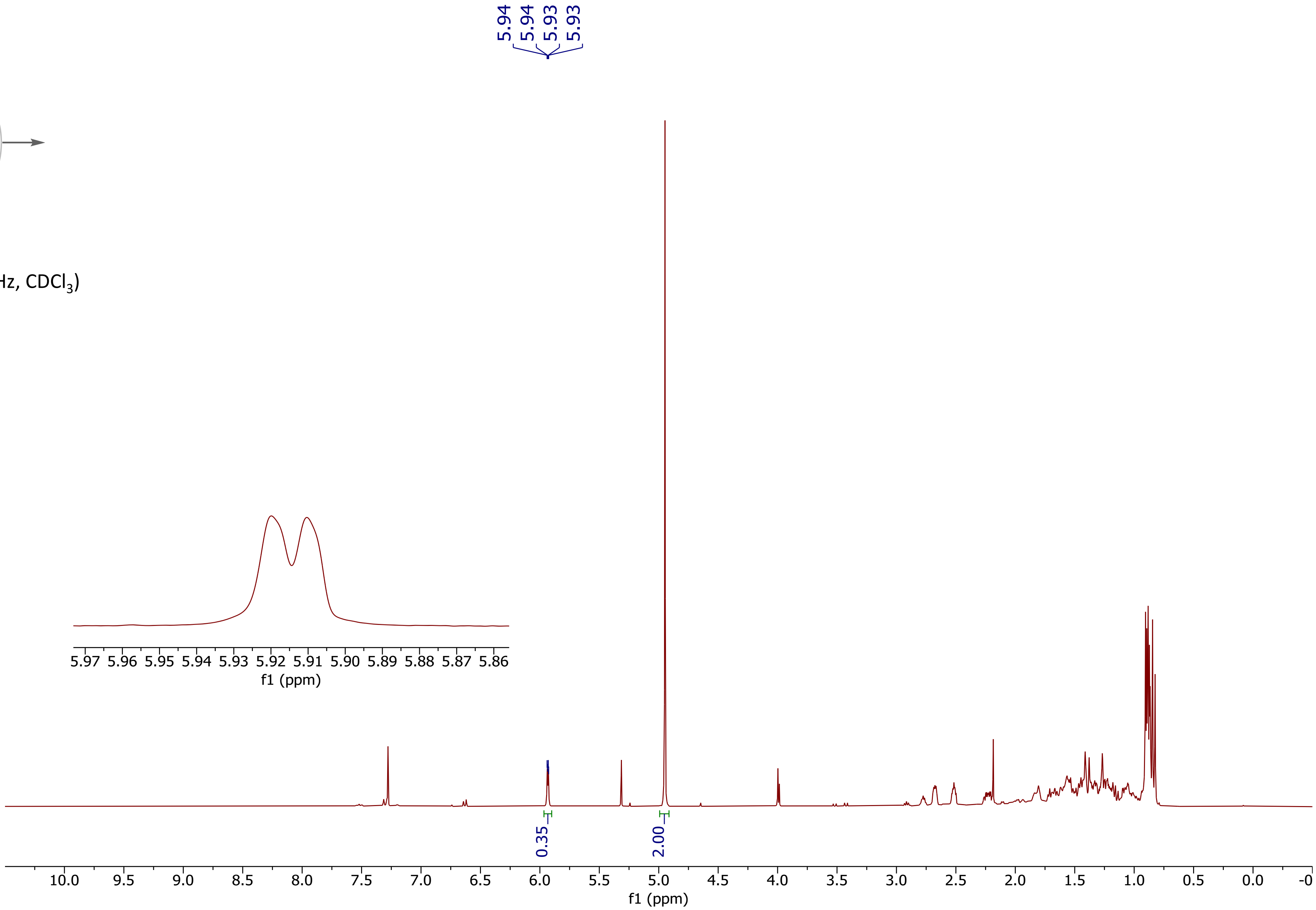

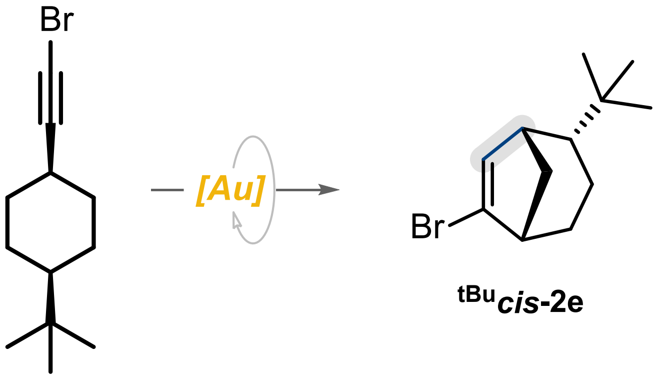

tBu*cis*-2e

^{1}H NMR(300 MHz, $CDCl_3$)

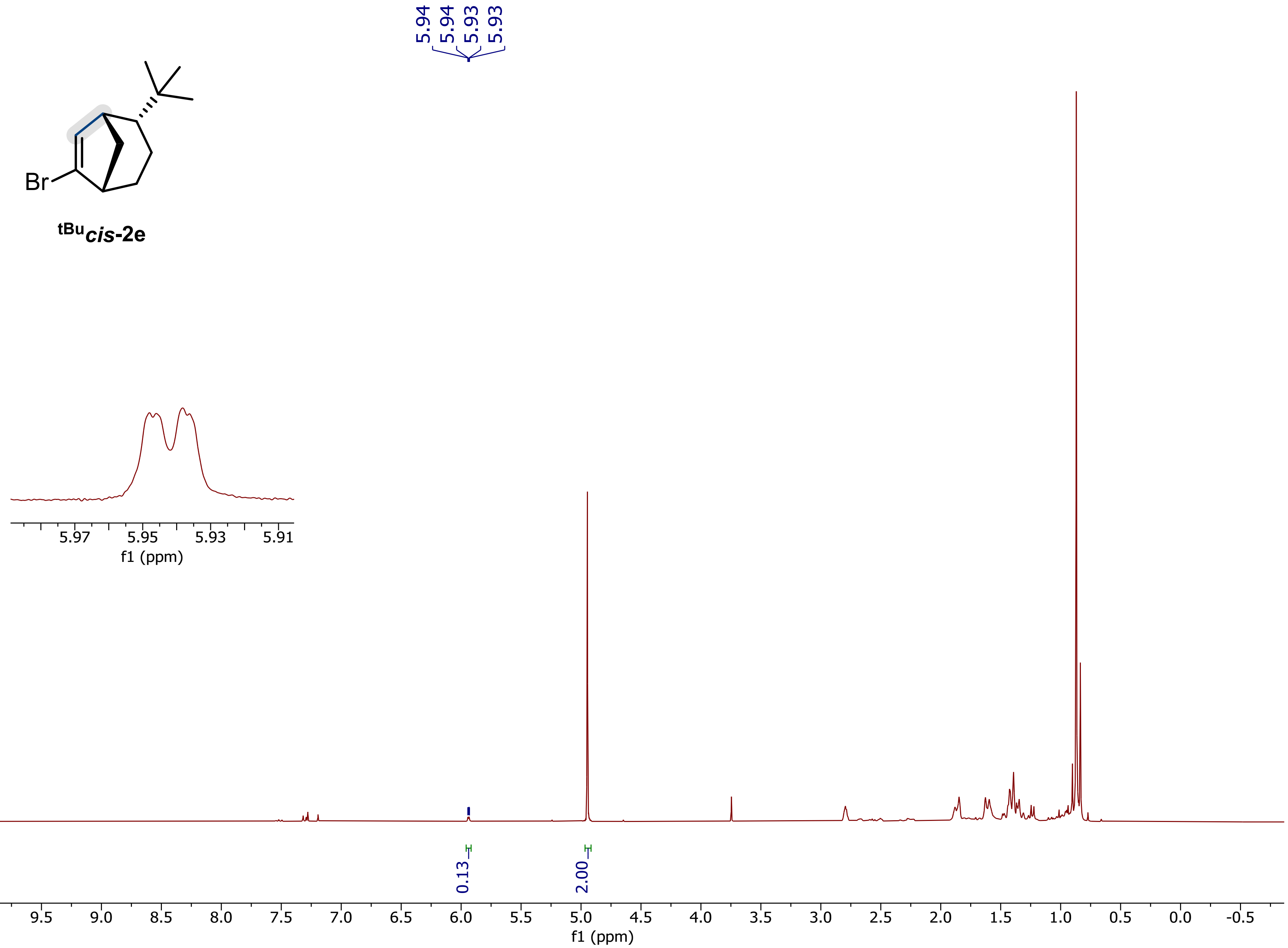

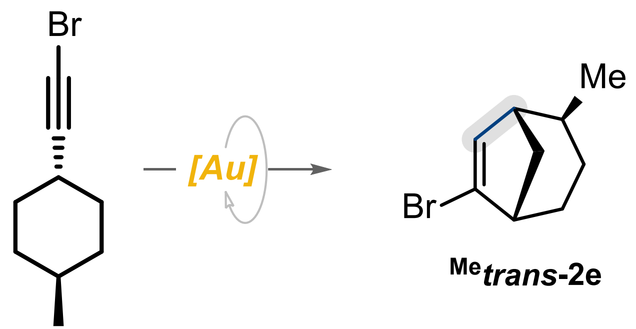

^{1}H NMR(300 MHz, $CDCl_3$)

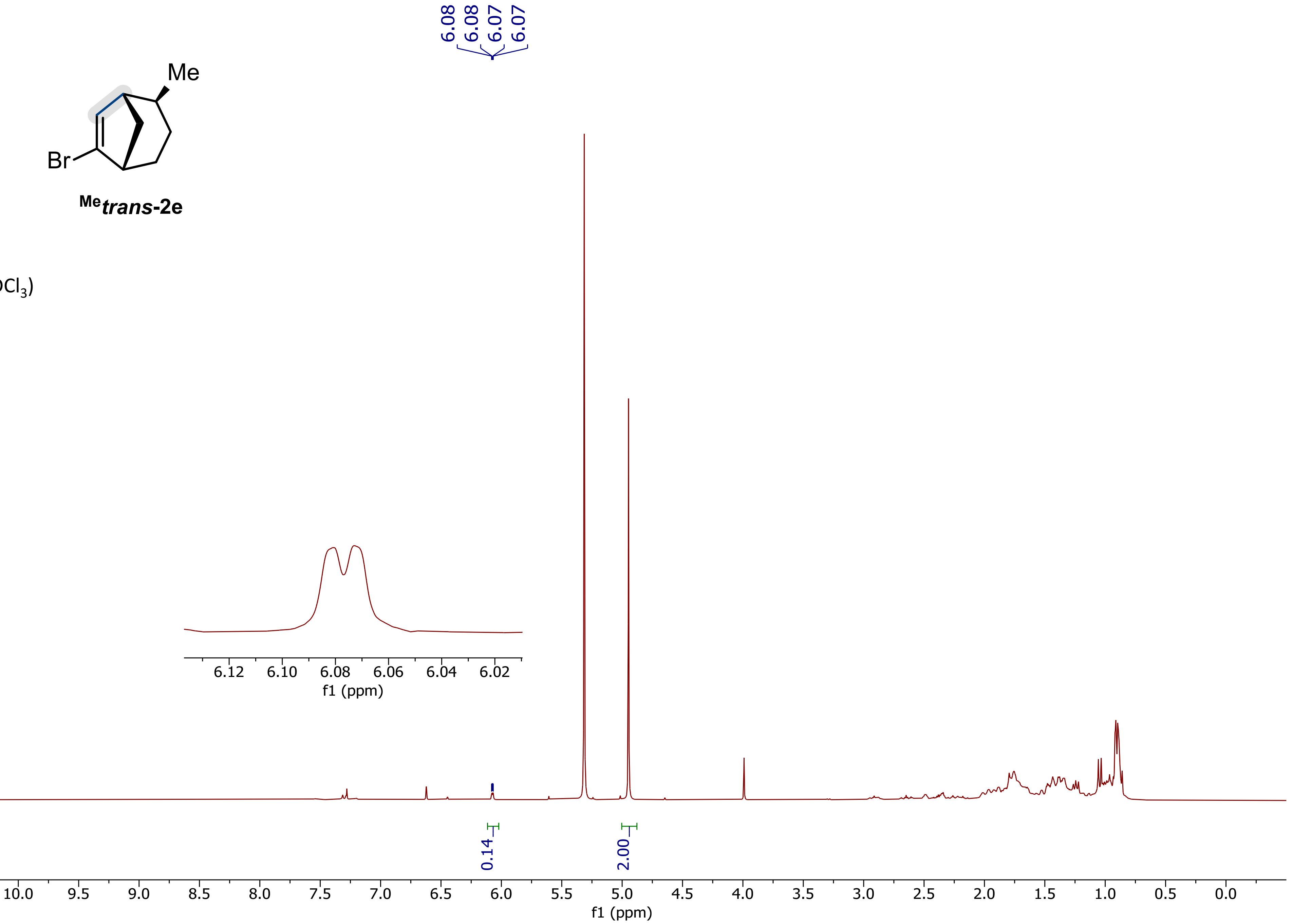

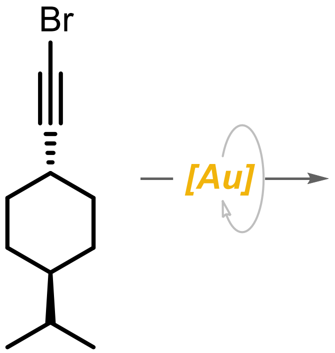

^{1}**H NMR**(300 MHz, $CDCl_3$)

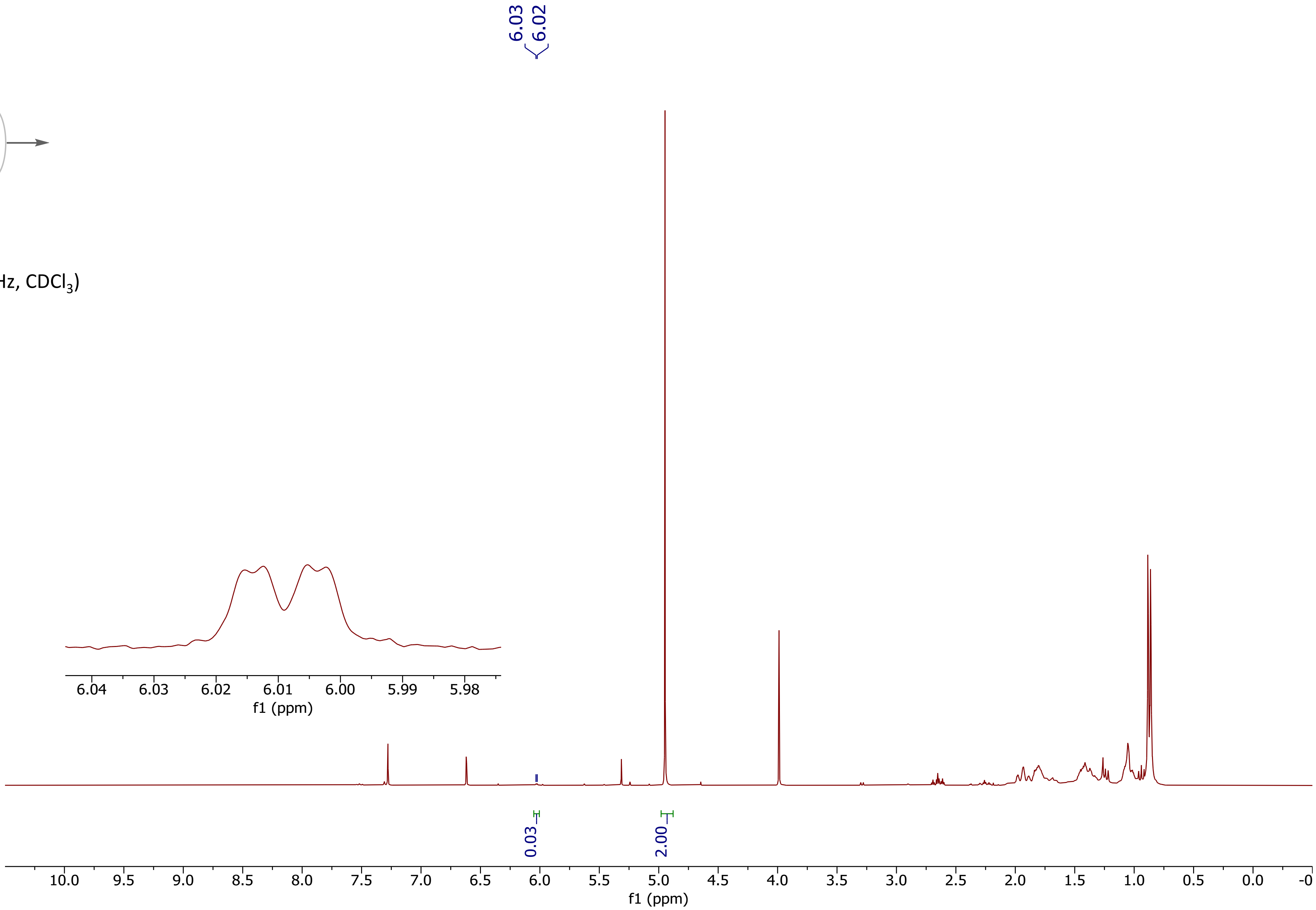

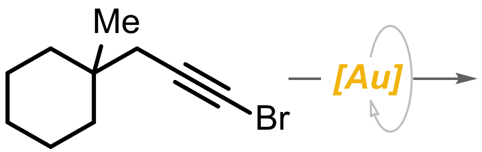

^{1}H NMR(300 MHz, $CDCl_3$)

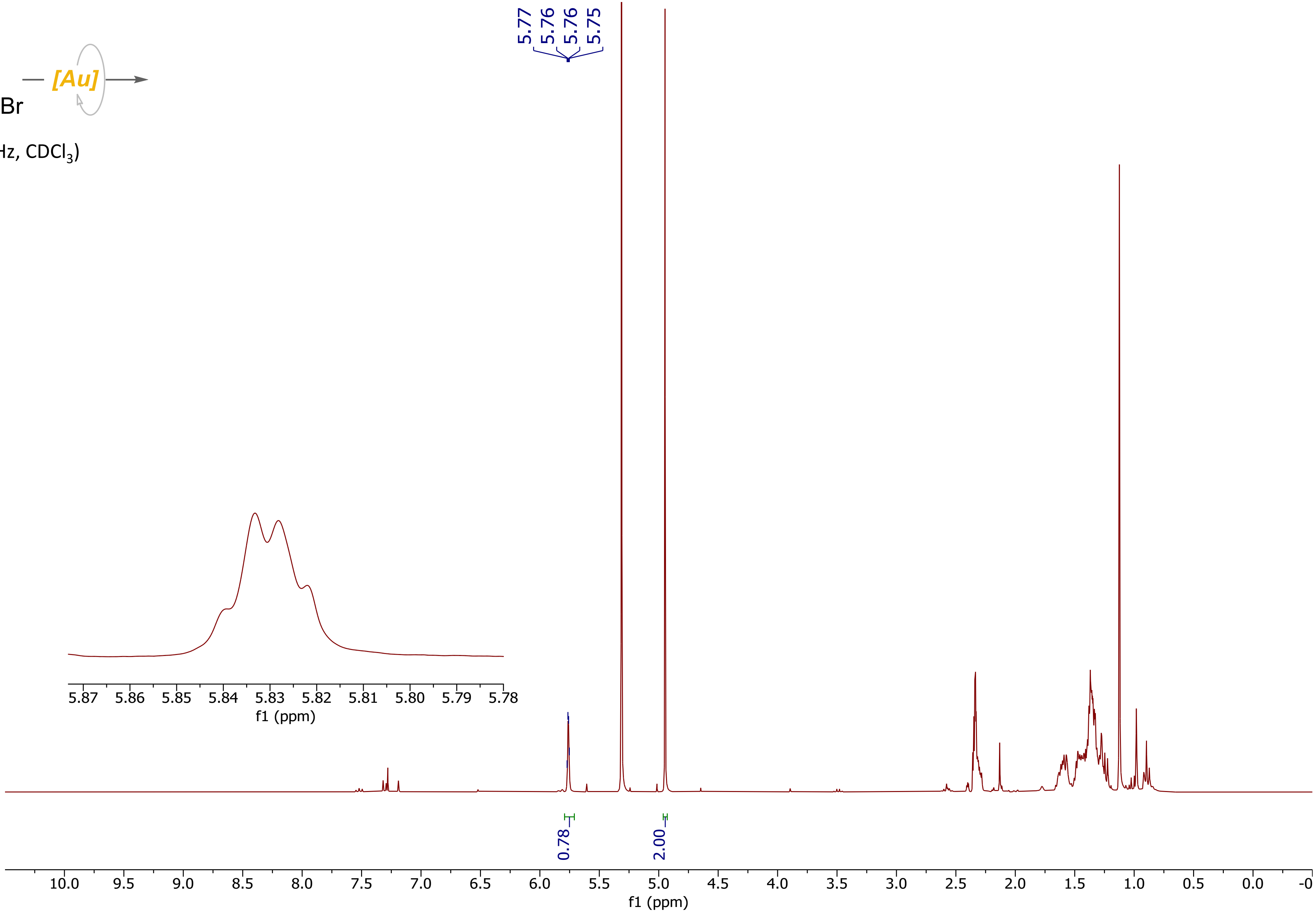

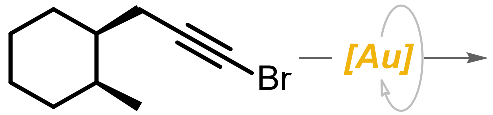

^{1}**H NMR**(300 MHz, $CDCl_3$)

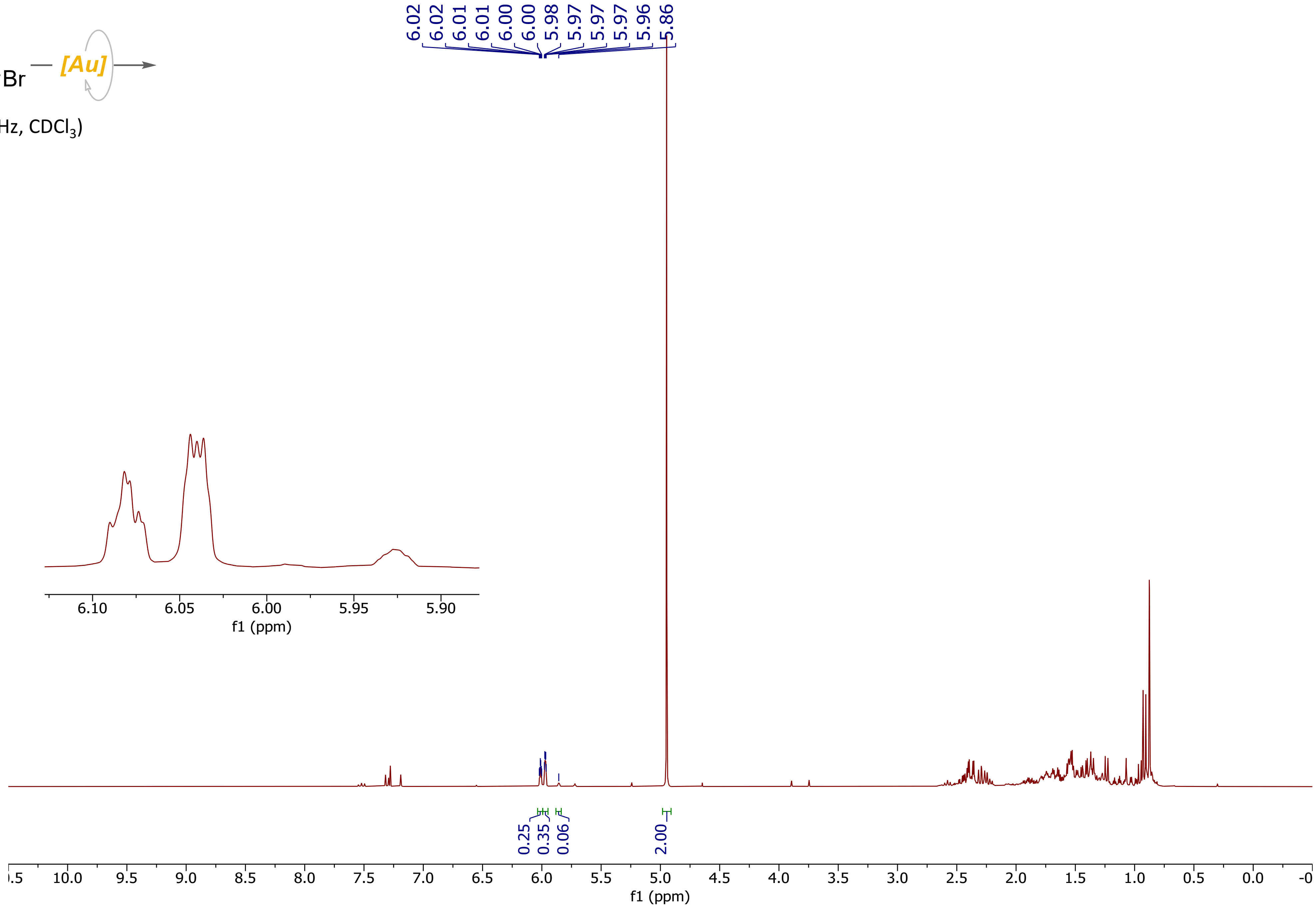

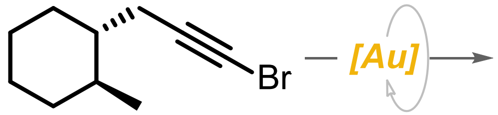

^{1}H NMR(300 MHz, $CDCl_3$)

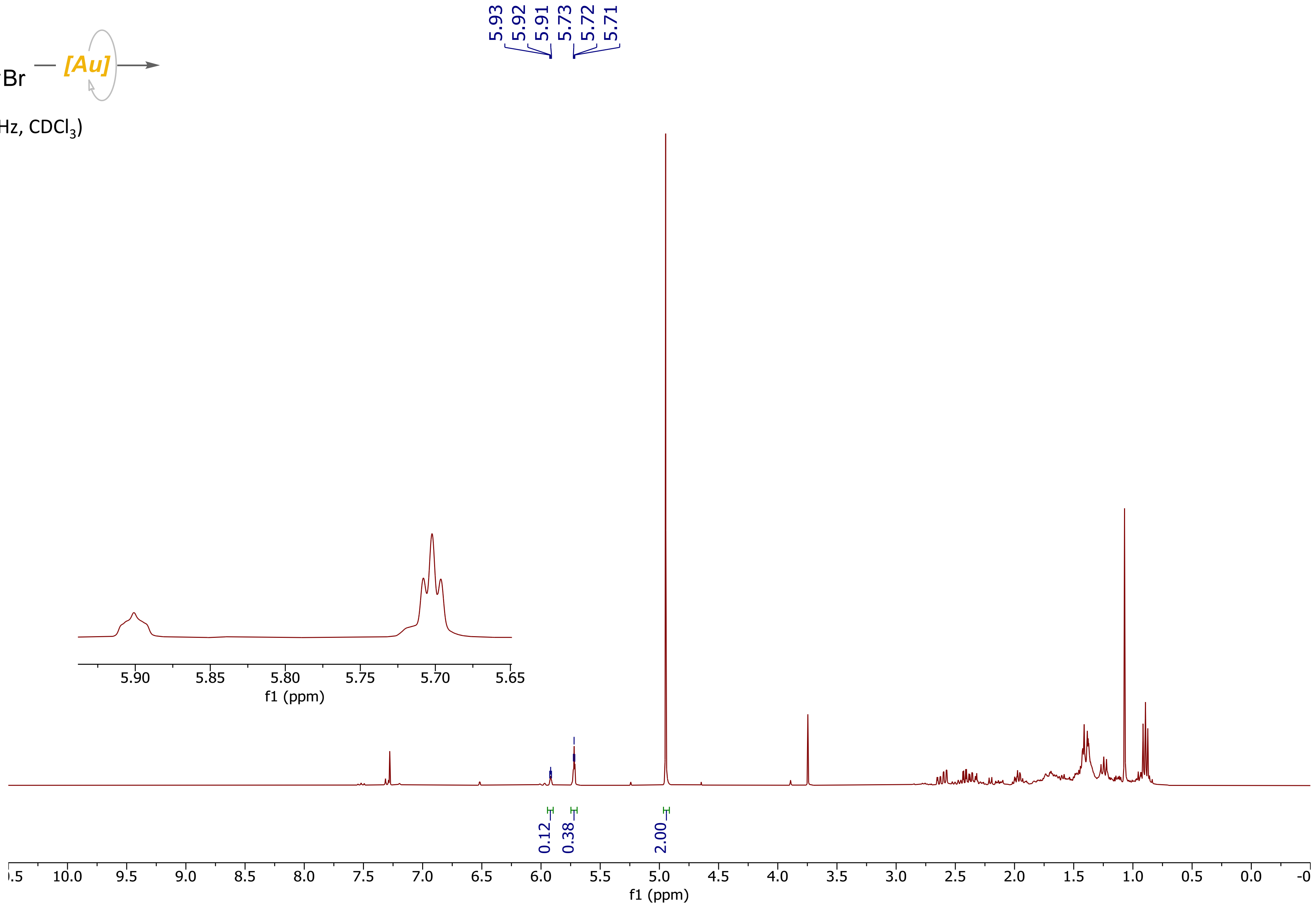

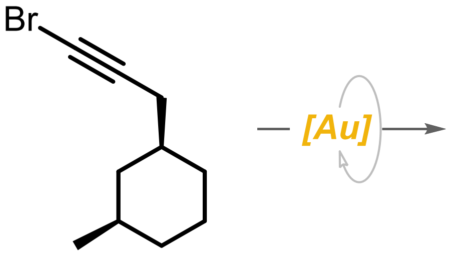

¹H NMR(300 MHz, $CDCl_3$)

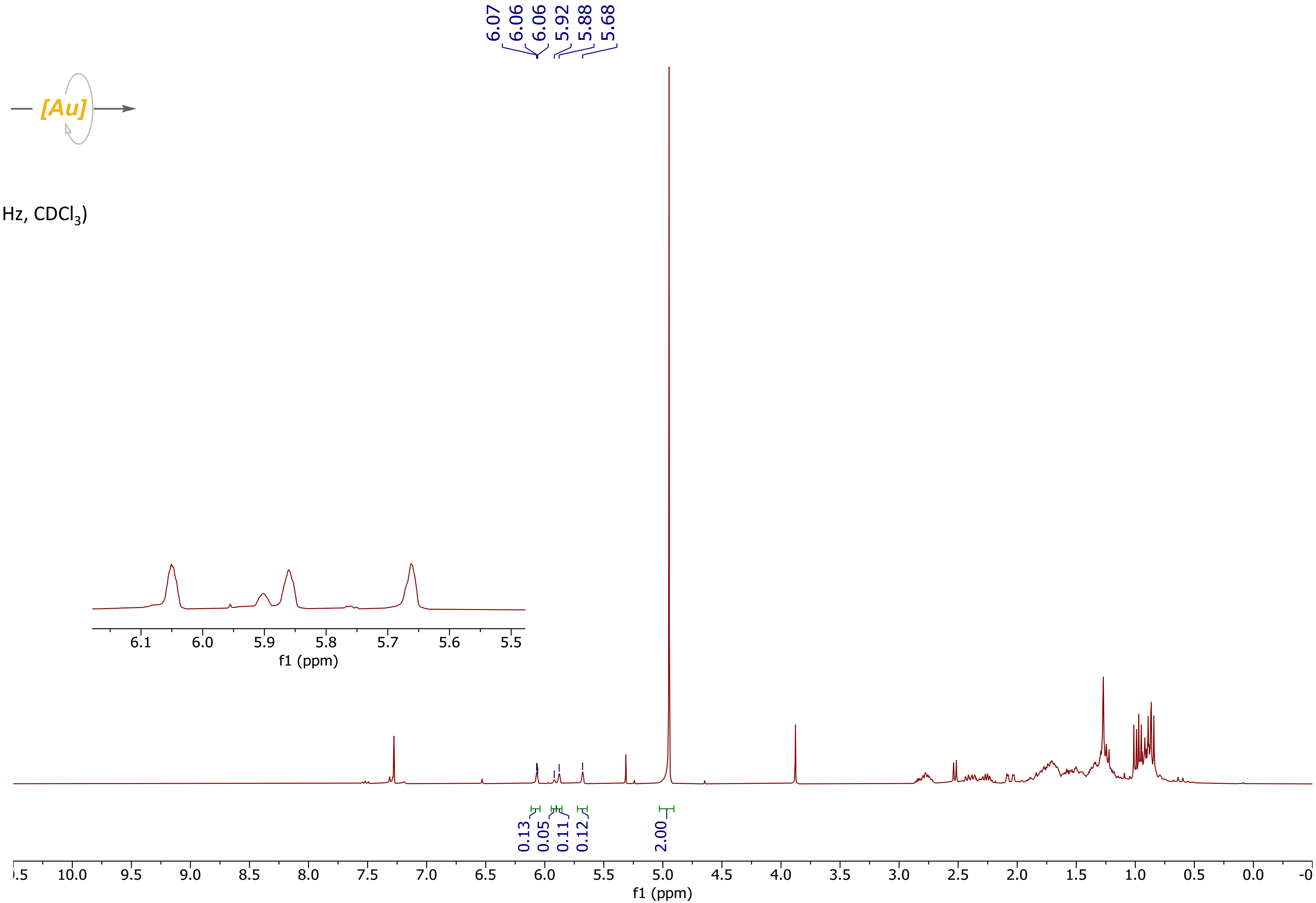

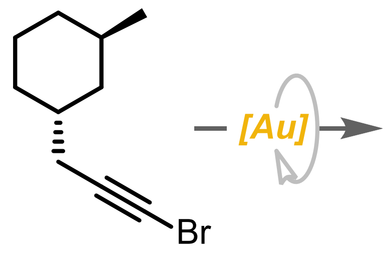

¹H NMR(300 MHz, $CDCl_3$)

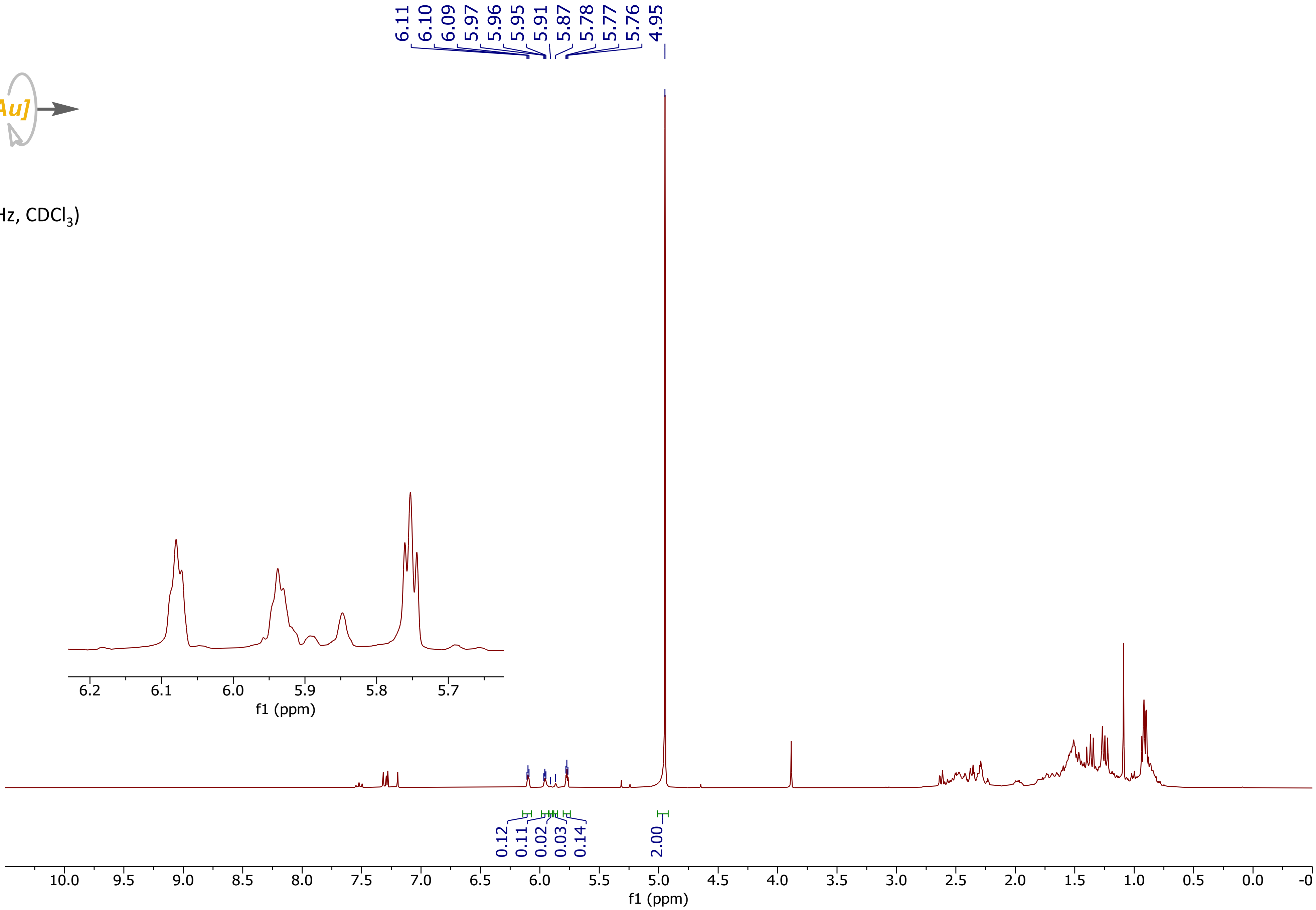

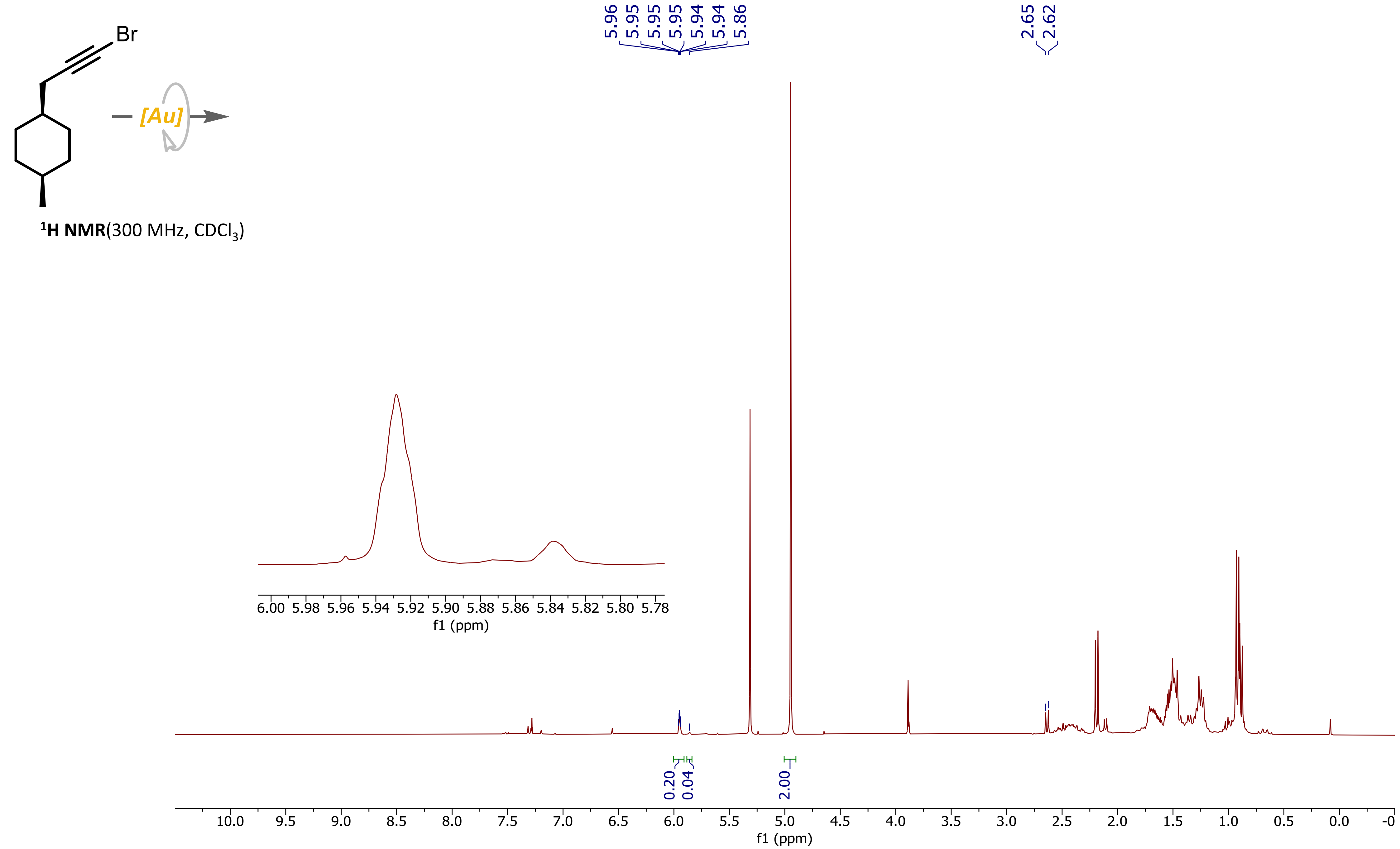

^{1}H NMR(300 MHz, $CDCl_3$)

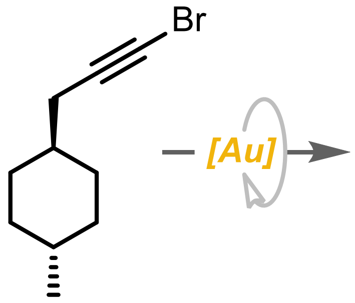

^{1}H NMR(300 MHz, $CDCl_3$)

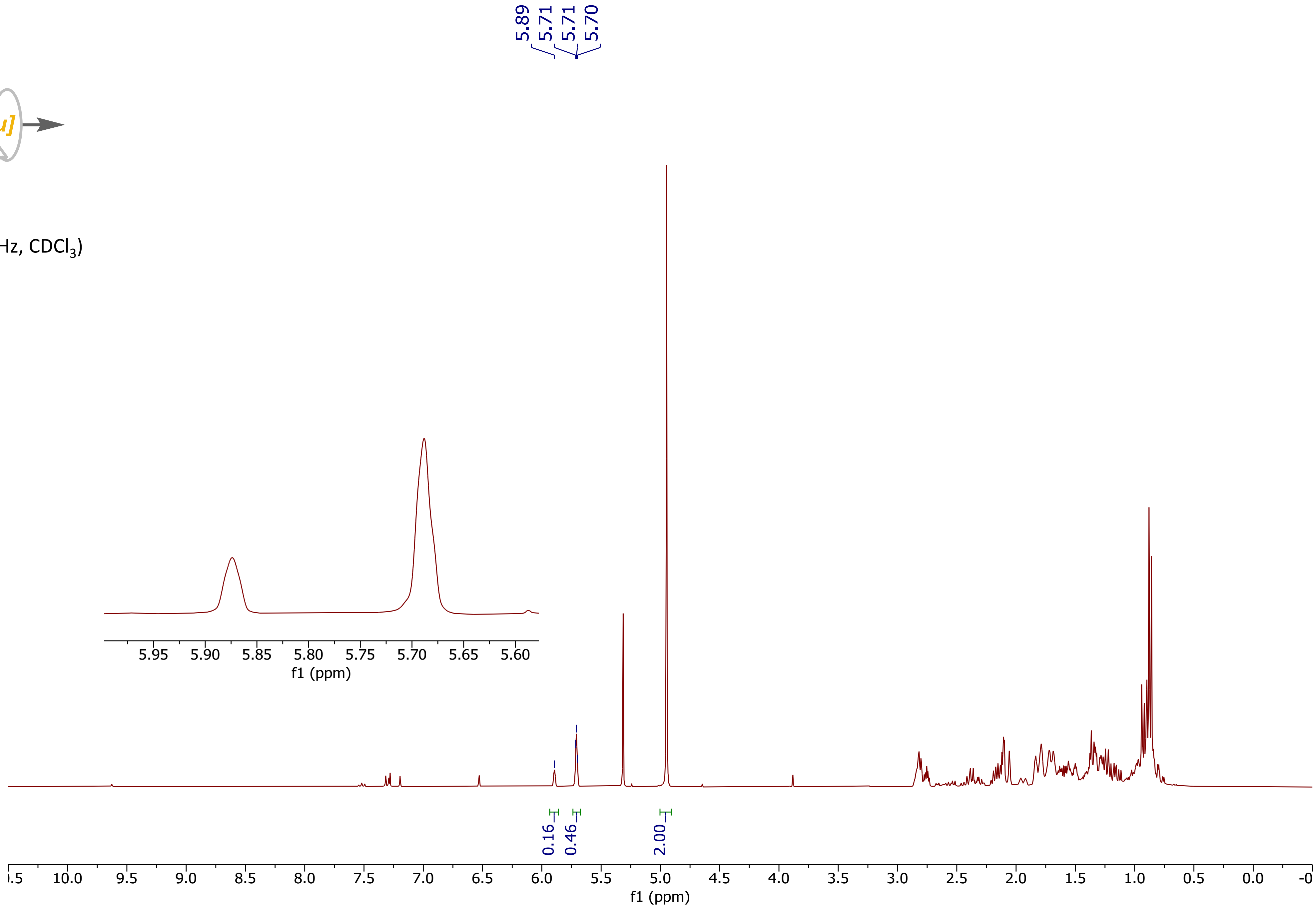

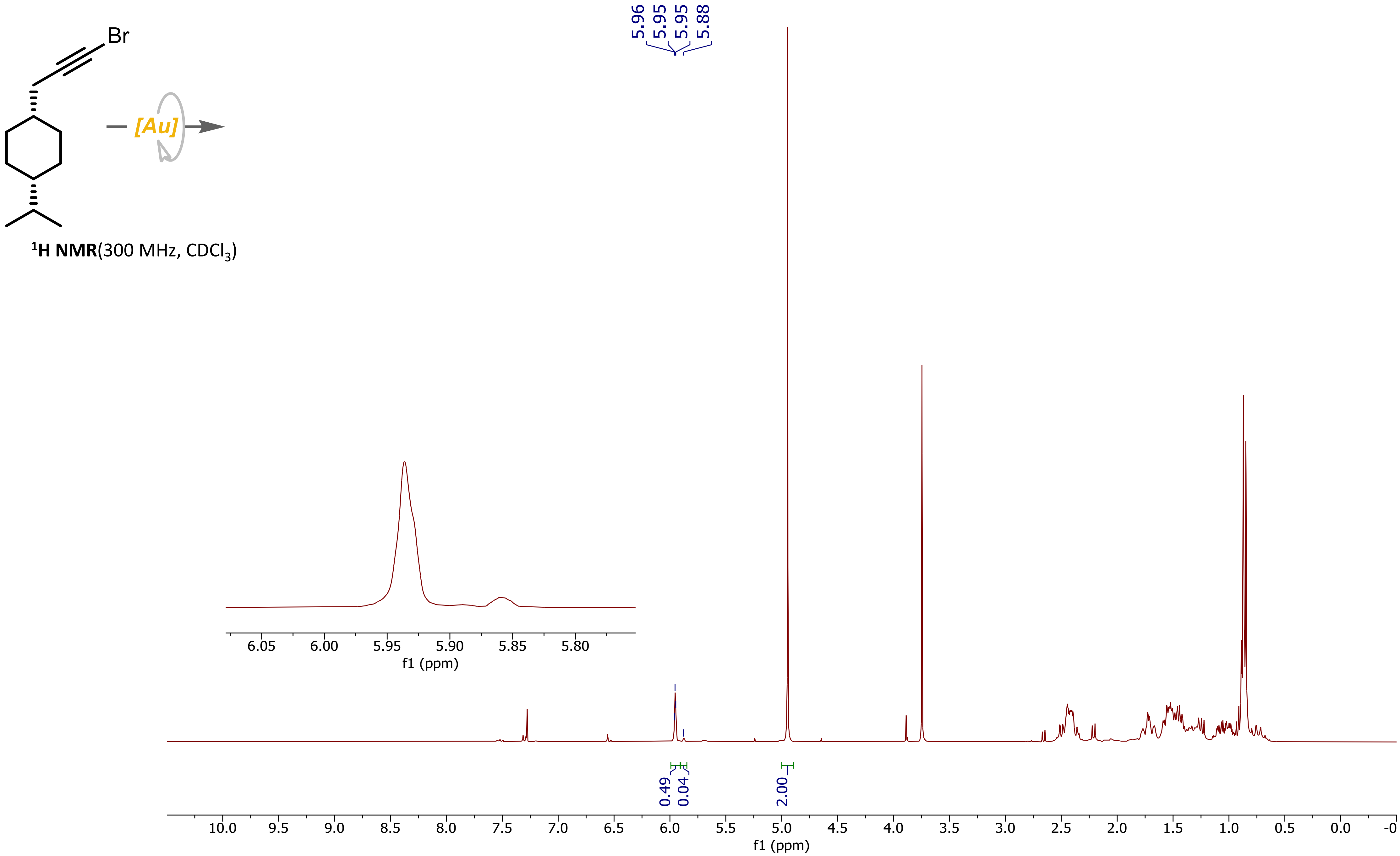
Br
[Au]
¹H NMR(300 MHz, CDCl3)
5.96
5.95
5.95
5.88
6.05
6.00
5.95
5.90
5.85
5.80
f1 (ppm)
0.49
0.04
2.00
10.0
9.5
9.0
8.5
8.0
7.5
7.0
6.5
6.0
5.5
5.0
4.5
4.0
3.5
3.0
2.5
2.0
1.5
1.0
0.5
0.0
-0
f1 (ppm)

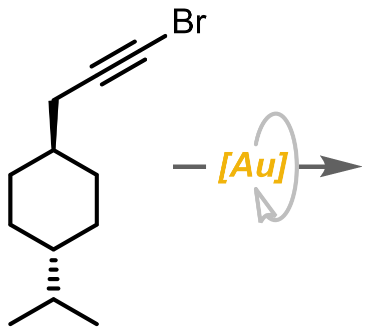

^{1}H NMR(300 MHz, $CDCl_3$)

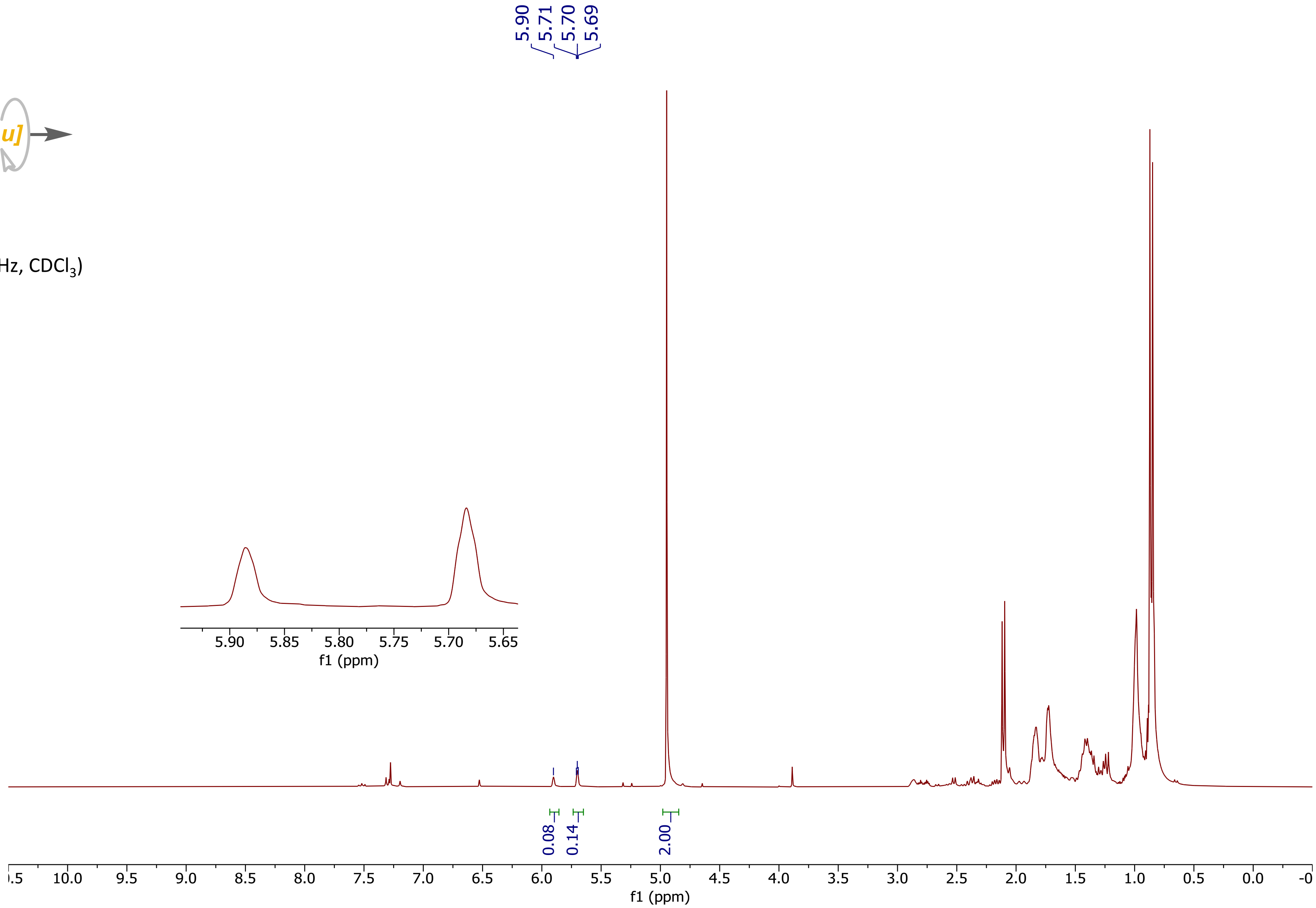

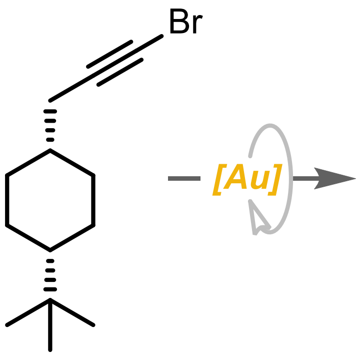

^{1}H NMR(300 MHz, $CDCl_3$)

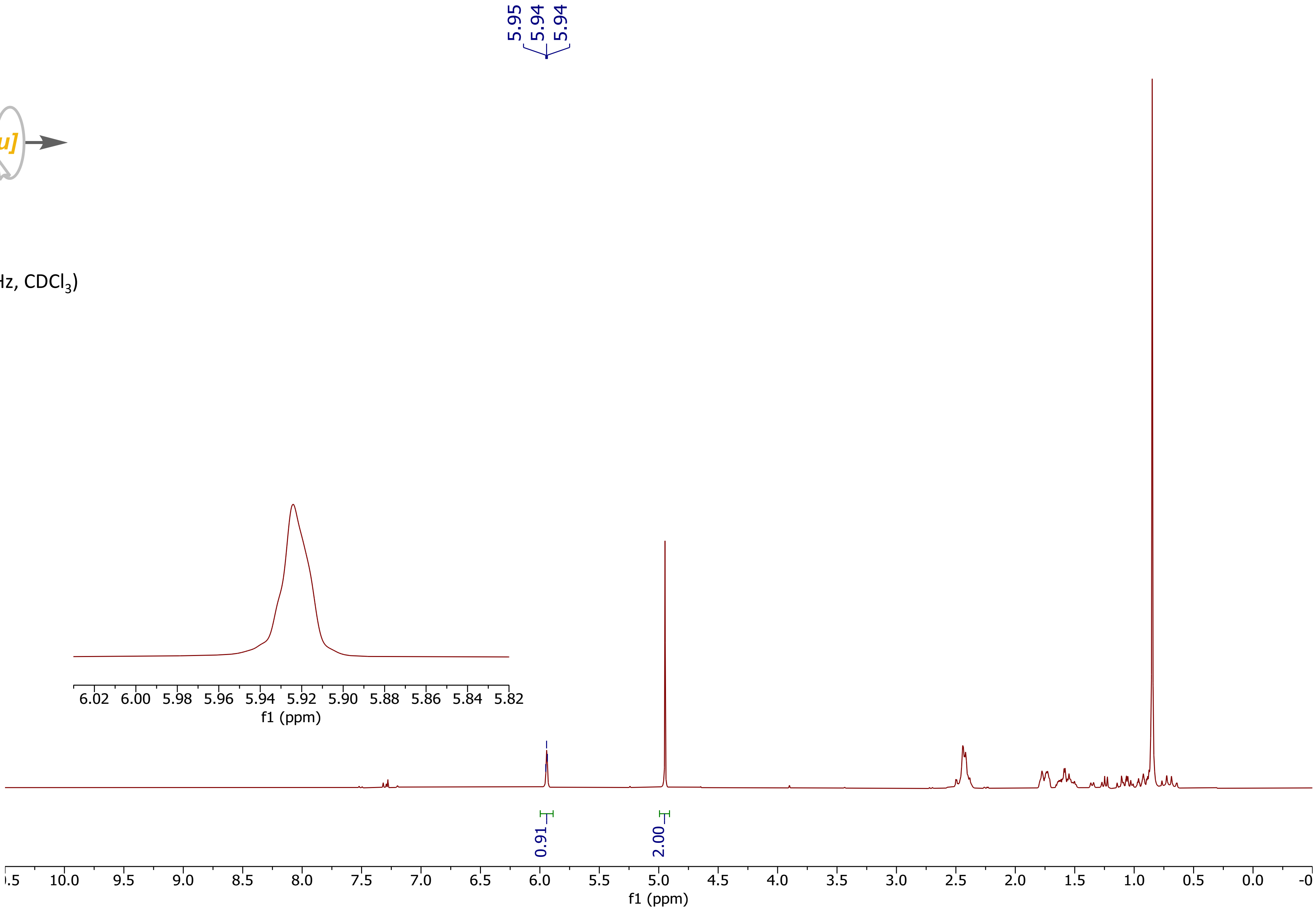

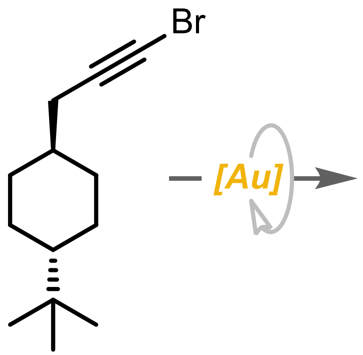

^{1}**H NMR**(300 MHz, $CDCl_3$)

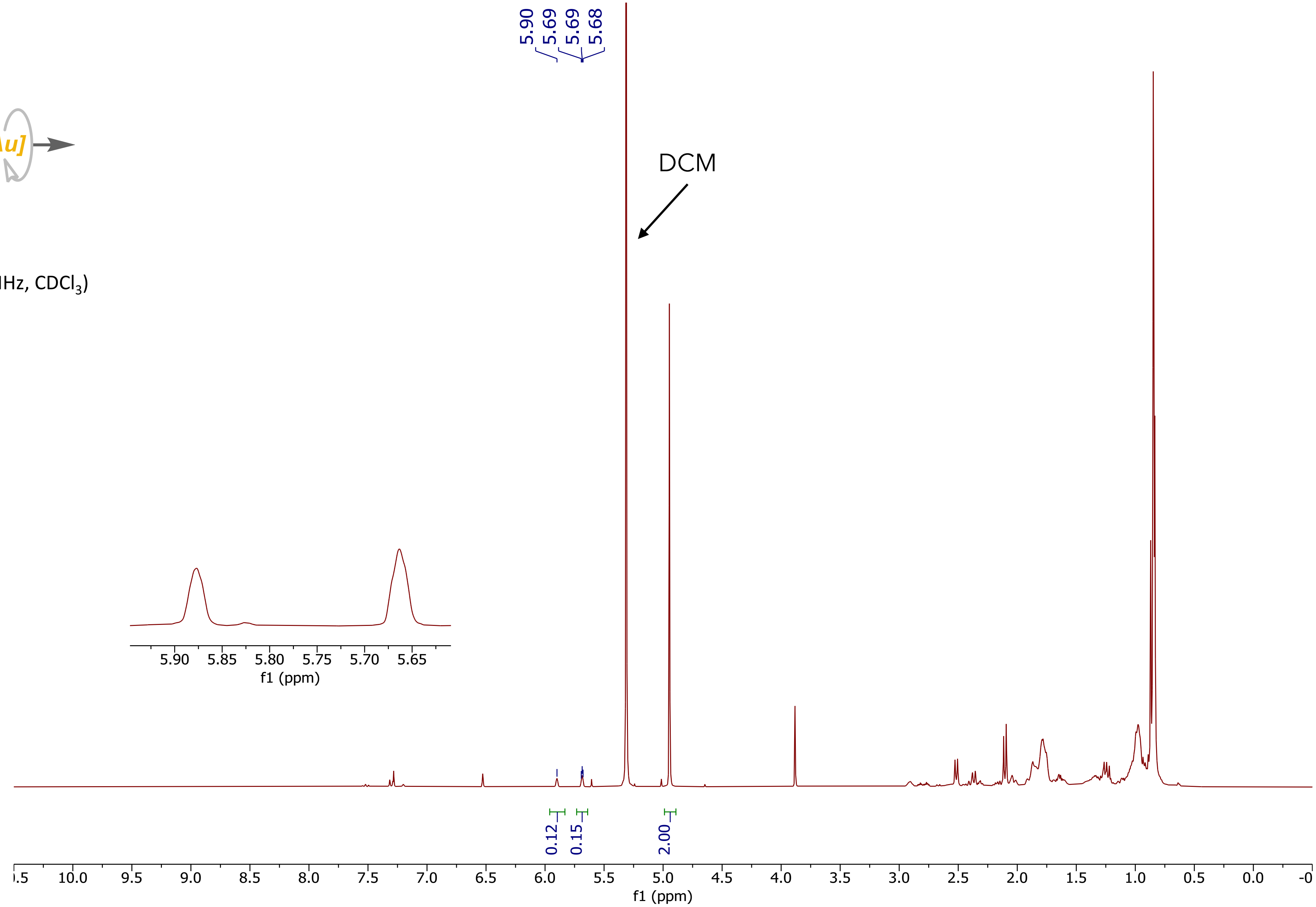

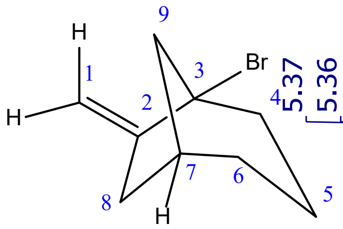

5

[1]**H NMR**(600 MHz, $CDCl_3$)

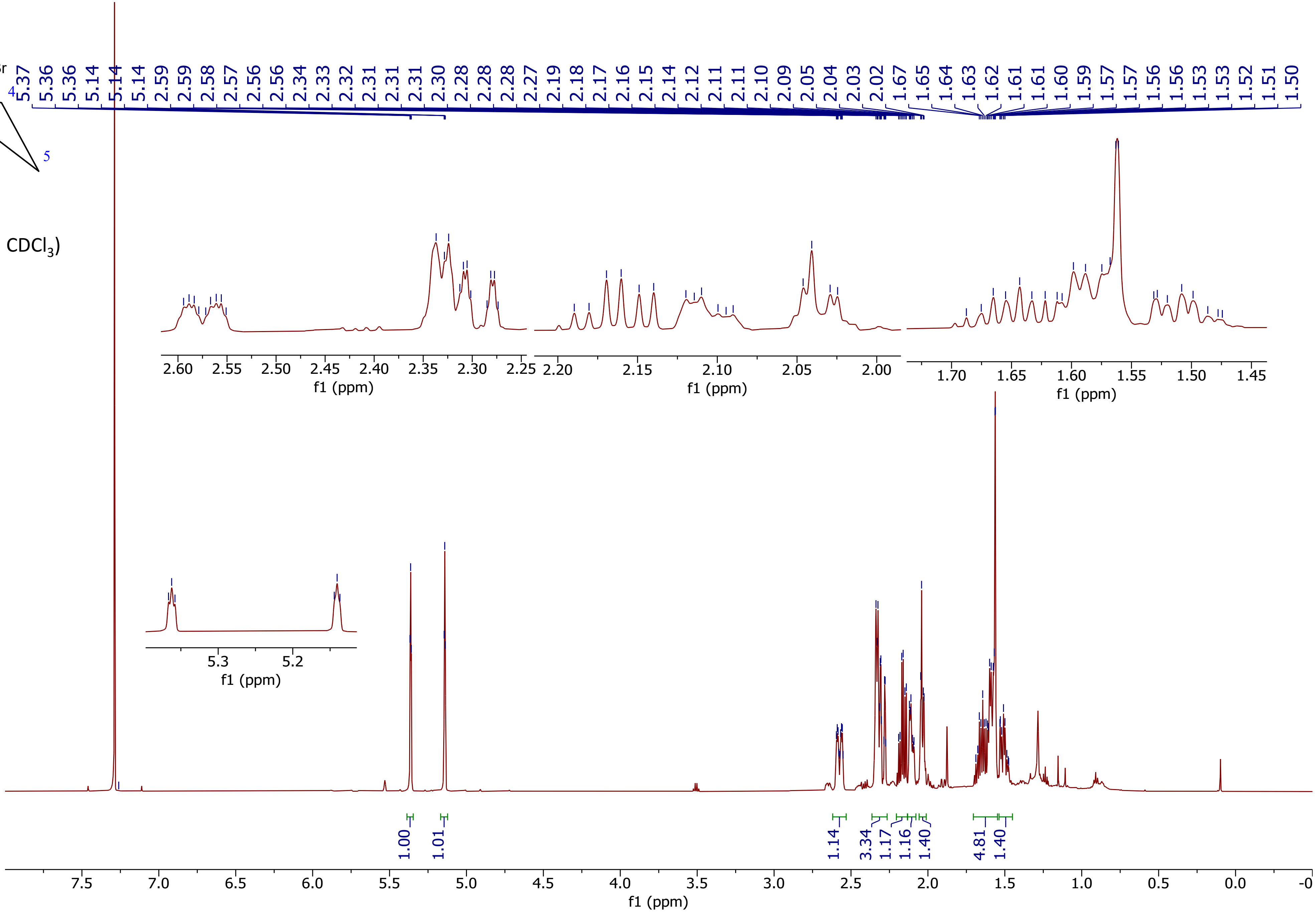

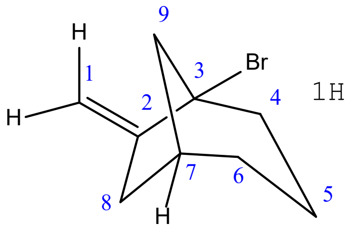

5

^{1}H NMR(600 MHz, $CDCl_3$)

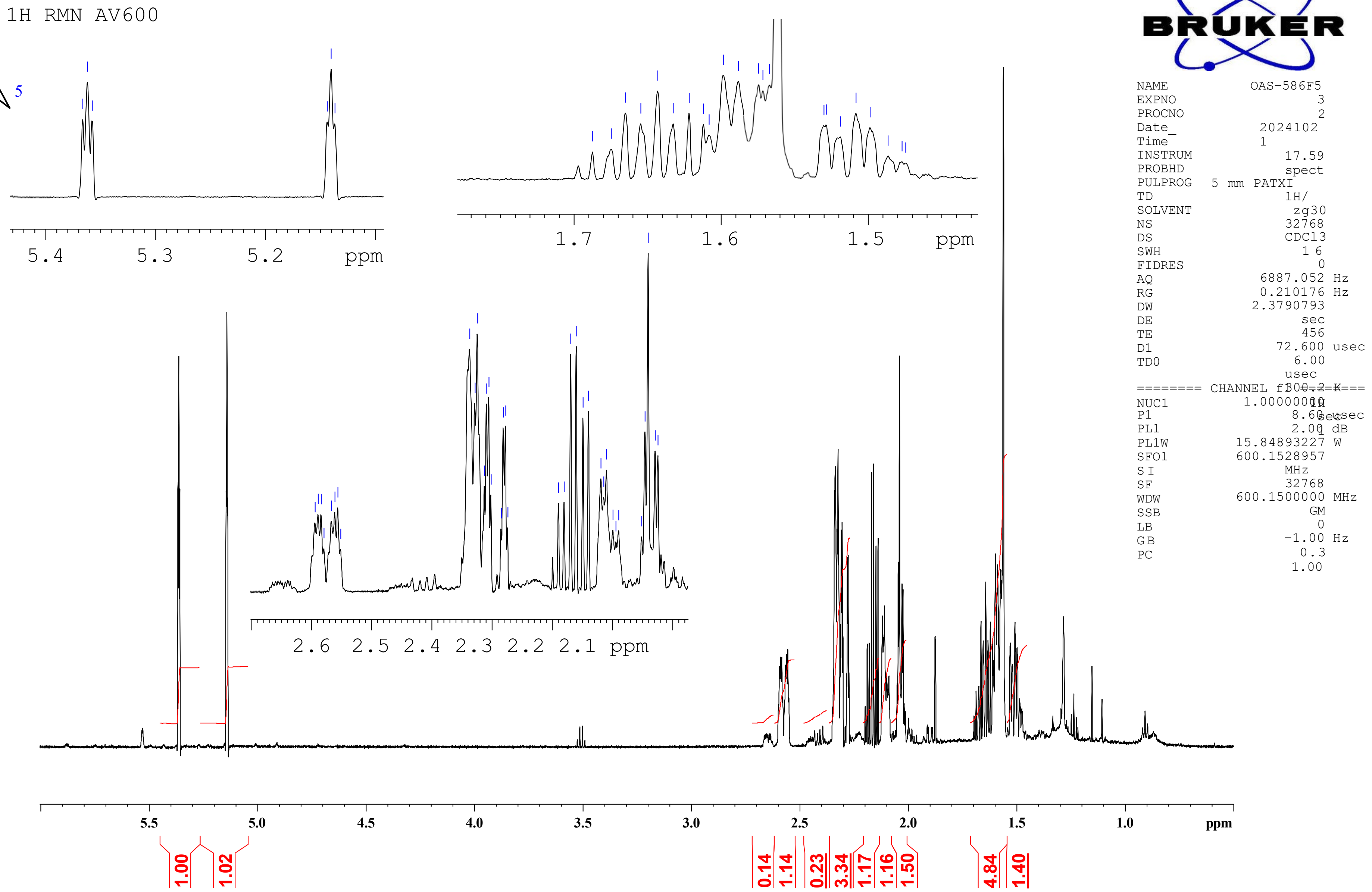

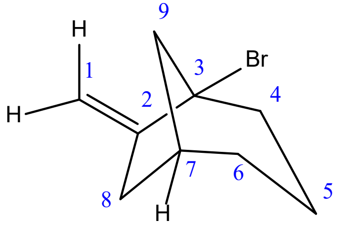

5

^{13}**C NMR** (150 MHz, $CDCl_3$)

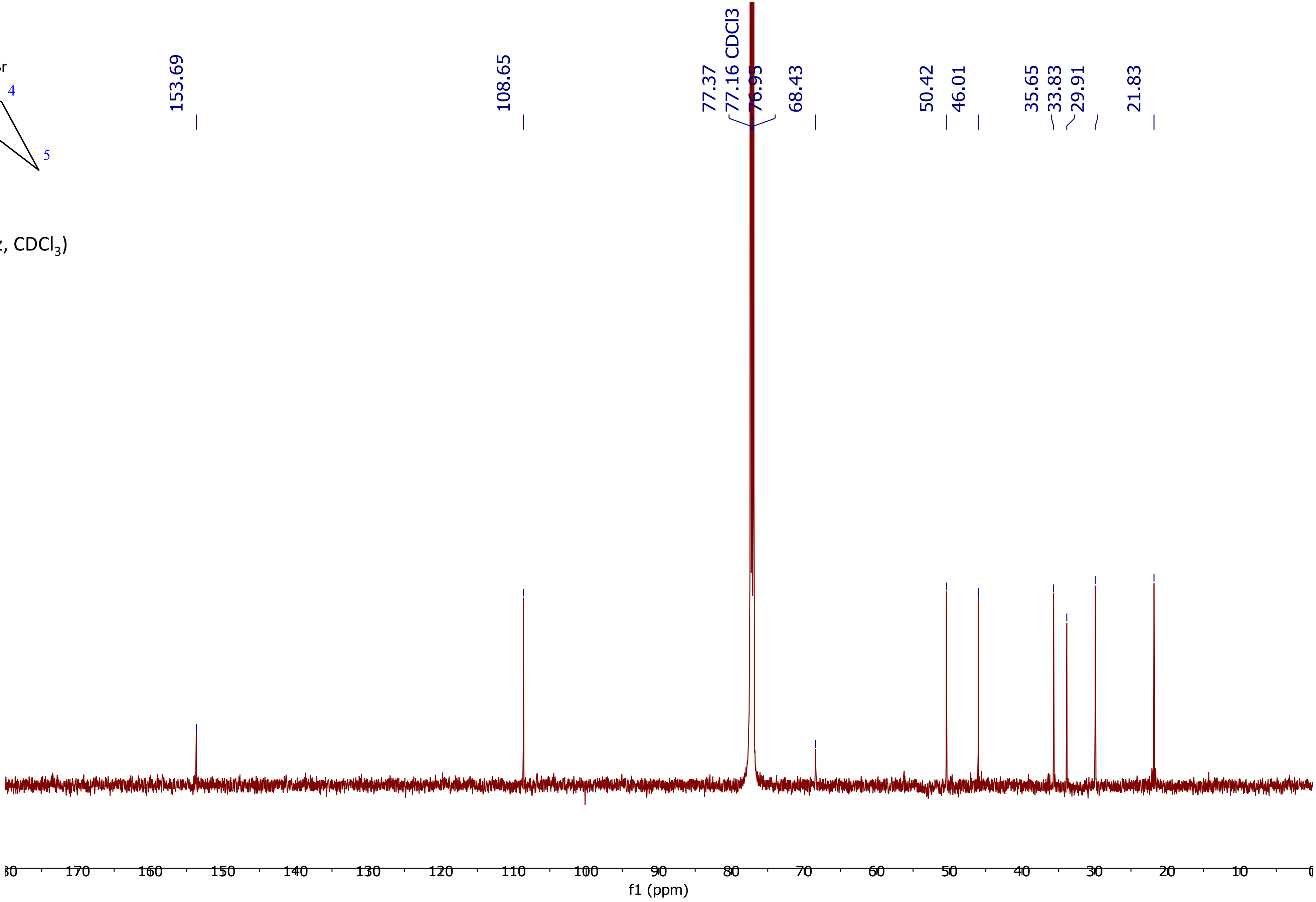

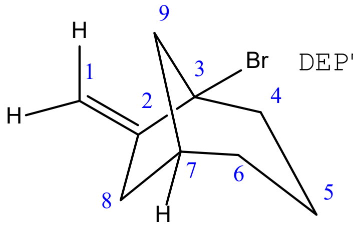

5

DEPT3 AV600

DEPT-135 NMR (150 MHz, $CDCl_3$)

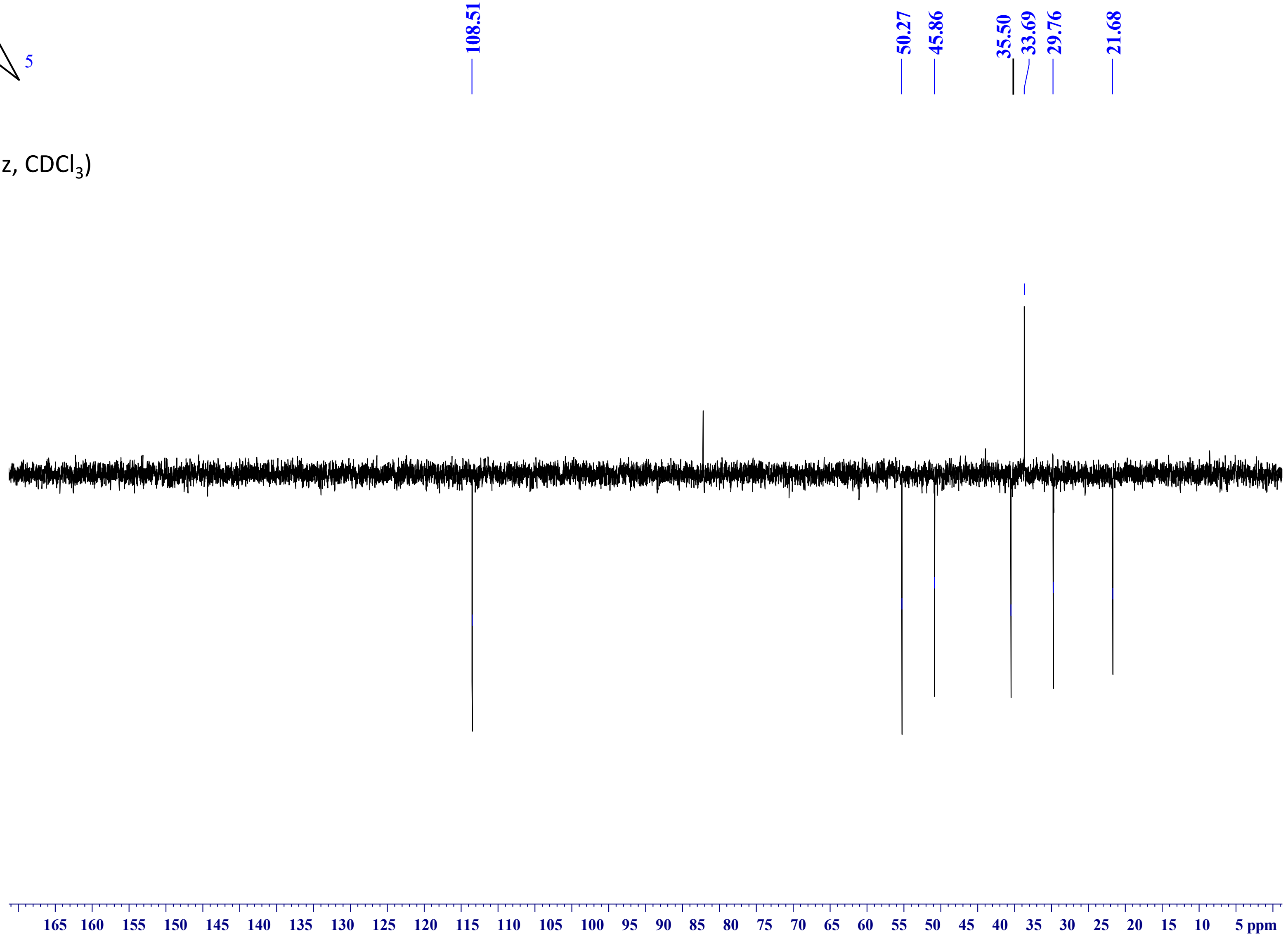

NAME	OAS-586F5
EXPNO	10
PROCNO	1
Date_	20241022
Time	10.16
INSTRUM	spect
PROBHD	5 mm PATXI 1H/
PULPROG	dept135
TD	32768
SOLVENT	CDCl3
NS	4000
DS	0
SWH	26041.666 Hz
FIDRES	0.794729 Hz
AQ	0.6292148 sec
RG	20600
DW	19.200 usec
DE	6.00 usec
TE	299.2 K
CNST2	145.0000000
D1	2.00000000 sec
D2	0.00344828 sec
D12	0.00002000 sec
TD0	1

======== CHANNEL f1 ========

NUC1	13C
P1	12.25 usec
P2	24.50 usec
PL1	-3.00 dB
PL1W	150.35617065 W
SFO1	150.9206652 MHz

======== CHANNEL f2 ========

CPDPRG2	waltz16
NUC2	1H
P3	9.00 usec
P4	18.00 usec
PCPD2	80.00 usec
PL2	2.00 dB
PL12	21.00 dB
PL2W	15.84893227 W
PL12W	0.19952624 W
SFO2	600.1524010 MHz
SI	32768
SF	150.9078380 MHz
WDW	EM
SSB	0
LB	2.00 Hz
GB	0
PC	1.40

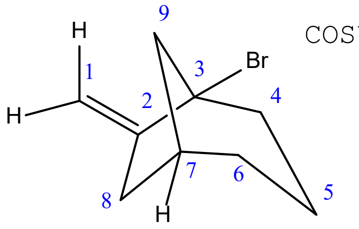

5

COSY NMR([600, 600] MHz, $CDCl_3$)

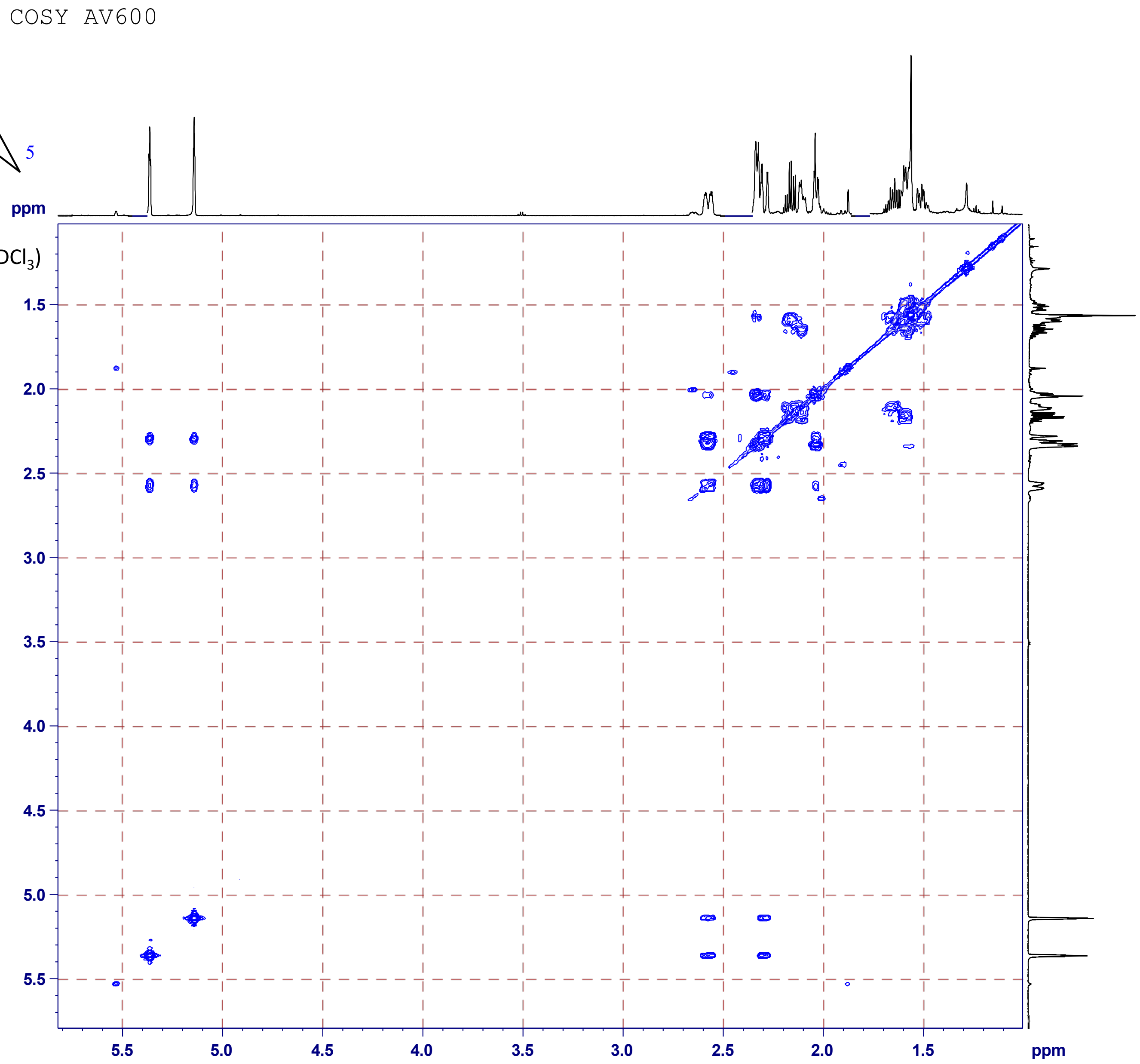


```
NAME            OAS-586F5
EXPNO                   4
PROCNO                  1
Date_            20241021
Time                18.00
INSTRUM             spect
PROBHD   5 mm PATXI 1H/
PULPROG          cosygpqf
TD                   1024
SOLVENT             CDCl3
NS                      2
DS                     12
SWH              3443.526 Hz
FIDRES           3.362818 Hz
AQ              0.1488800
sec RG                    1620
DW                145.200 usec
DE                   6.00 usec
TE                  299.2 K
D0             0.00000300 sec
D1             1.00000000 sec
D13            0.00000400 sec
D16            0.00015000 sec
IN0            0.00029040 sec

======== CHANNEL f1 ========
NUC1                   1H
P0                   8.60 usec
P1                   8.60 usec
PL1                  2.00 dB
PL1W          15.84893227 W
SFO1          600.1519389 MHz

====== GRADIENT CHANNEL =====
GPNAM1           SINE.100
GPZ1                10.00 %
P16               1000.00
usec ND0                      1
TD                    256
SFO1             600.1519
MHz FIDRES          13.451374
Hz SW                  5.738
ppm FnMODE                QF
SI                   1024
SF            600.1500000 MHz
WDW                  SINE
SSB                     0
LB                   0.00 Hz
GB                      0
PC                   4.00
SI                   1024
MC2                    QF
SF            600.1500000 MHz
WDW                  SINE
SSB                     0
LB                   0.00 Hz
GB                      0
```

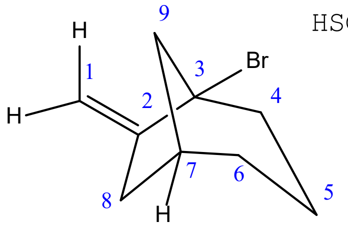

5

HSQC NMR([600, 150] MHz, $CDCl_3$)

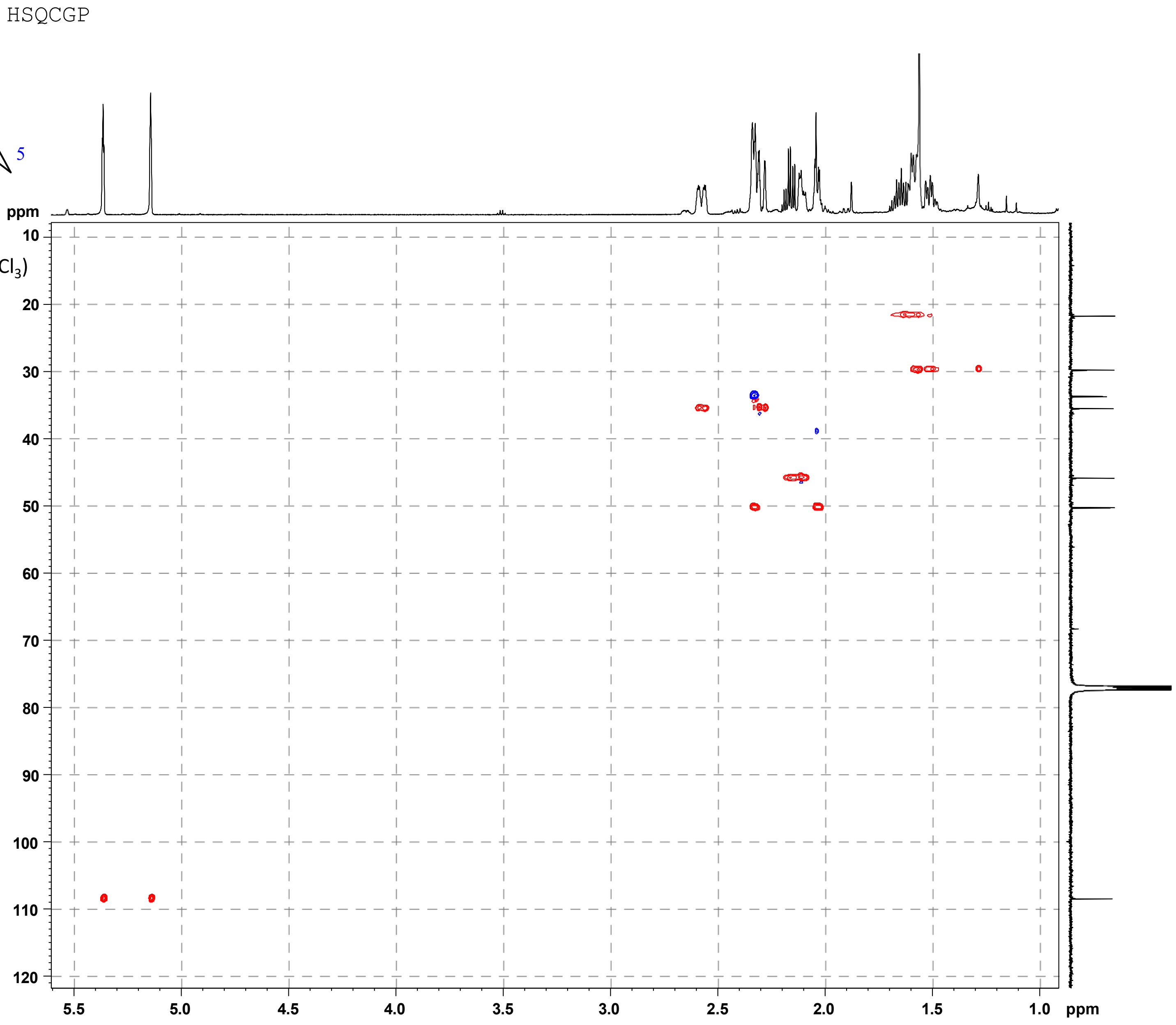


```
NAME          OAS-586F5
EXPNO
5
PROCNO                1
Date_          20241021
Time              18.10
INSTRUM           spect
PROBHD   5 mm PATXI 1H/
PULPROG
hsqcedetgpsisp2 TD
1024
SOLVENT           CDCl3
NS                   12
DS                    8
SWH          3443.526 Hz
FIDRES       3.362818 Hz
AQ          0.1488800 sec
RG                29100
DW            145.200 usec
DE               6.00 usec
TE              299.2 K
CNST2     145.0000000
D0         0.00000300 sec
D1         1.00000000 sec
D4         0.00172414 sec
D11        0.03000000 sec
D16        0.00015000 sec
D21        0.00350000 sec
D24        0.00086200 sec
IN0        0.00002045 sec
ZGOPTNS

======== CHANNEL f1 ========
NUC1                 1H
P1                 8.60 usec
P2                17.20 usec
P28              250.00 usec
PL1                2.00 dB
PL1W        15.84893227 W
SFO1        600.1519389 MHz

======== CHANNEL f2 ========
CPDPRG2            garp
NUC2                13C
P3                12.50 usec
P4                25.00 usec
P14              500.00 usec
PCPD2             77.00 usec
PL0              120.00 dB
PL2               -3.00 dB
PL12              12.75 dB
PL0W         0.00000000 W
PL2W       150.35617065 W
PL12W        4.00056410 W
SFO2        150.9199106 MHz
SP3                3.22 dB
SPNAM3   Crp60,0.5,20.1
SPOAL3            0.500
SPOFFS3            0.00 Hz
```

[illegible]

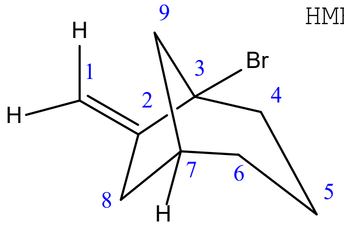

5

HMBC NMR([600, 150] MHz, $CDCl_3$)

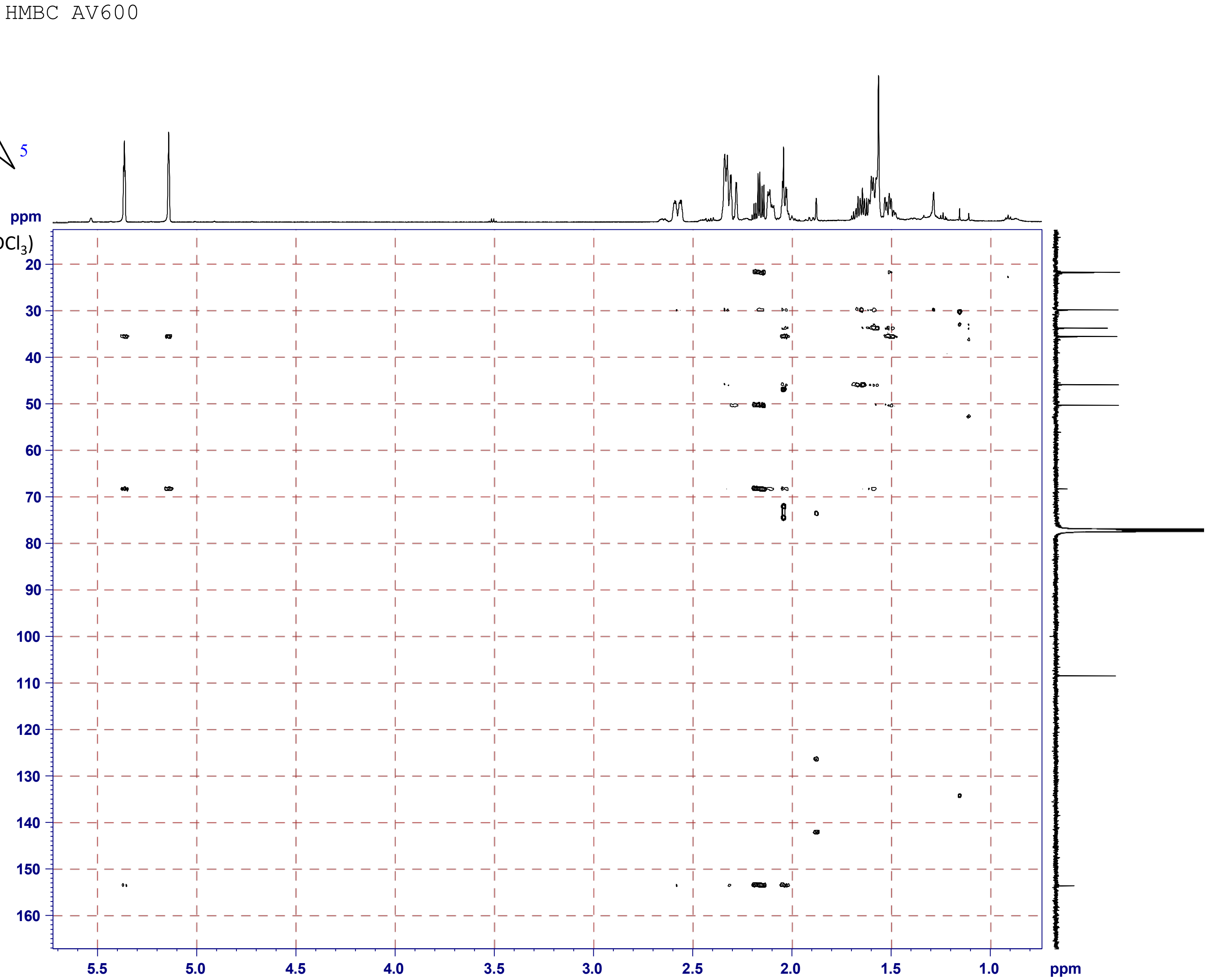

NAME OAS-586F5
EXPNO 6
PROCNO 1
Date_ 2024102
Time 1
INSTRUM 19.11
PROBHD spect
PULPROG 5 mm PATXI 1H/
TD hmbcgplpndq
SOLVENT f
NS 2048
DS CDCl
SWH 3
FIDRES 24
AQ 8
RG 3443.526 Hz
DW 1.681409 Hz
DE 0.2975648
TE sec
CNST2 26000
CNST13 145.200 usec
D0 6.00 usec
D1 298.2 K
D2 145.000000
D6 0
D16 8.0000000
IN0 0.00000300 sec
1.00000000 sec

======== CHANNEL f1 ======== [illegible]
NUC1 0.06250000 sec
P1 0.00015[illegible] usec
P2 0.0000[illegible] usec
PL1 2.00 dB
PL1W 15.84893227 W
SFO1 600.1519389 MHz

======== CHANNEL f2 ========
NUC2 13C
P3 12.50 usec
PL2 -3.00 dB
PL2W 150.35617065 W
SFO2 150.9214197 MHz

====== GRADIENT CHANNEL =====
GPNAM1 SINE.100
GPNAM2 SINE.100
GPNAM3 SINE.10
GPZ1 0
GPZ2 50.00
GPZ3 %
P16 30.00
ND0 %
TD 40.10 %
SFO1 1000.00 usec
FIDRES 2
SW 320
FnMODE 150.9214 MHz
SI 84.893295 Hz
SF 180.000
WDW ppm
SSB QF
LB 1024
GB 600.1500000 MHz
PC SINE
SI 0
MC2 0.00 Hz
SF 0
WDW 4.0
SSB 0
LB 1024
GB QF
150.9078380 MHz
SIN
E
0
0.00
Hz
0

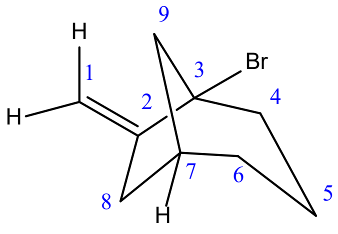

5

NOESY NMR([600, 600] MHz, $CDCl_3$)

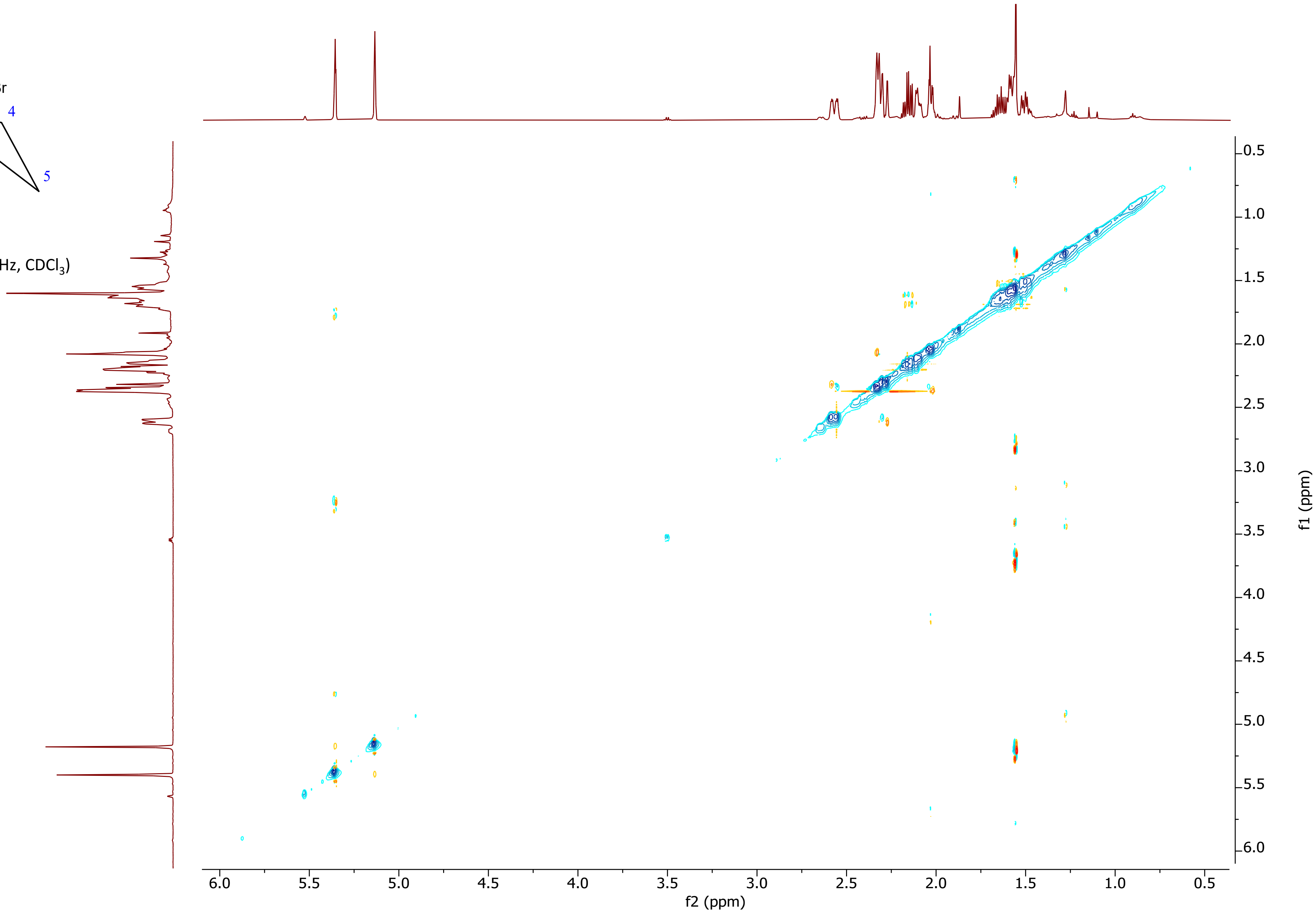

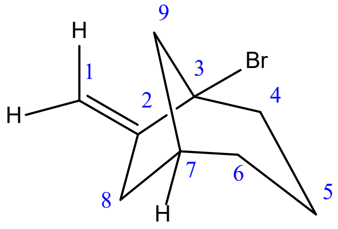

5

SEL-TOCSY NMR(600 MHz, $CDCl_3$)

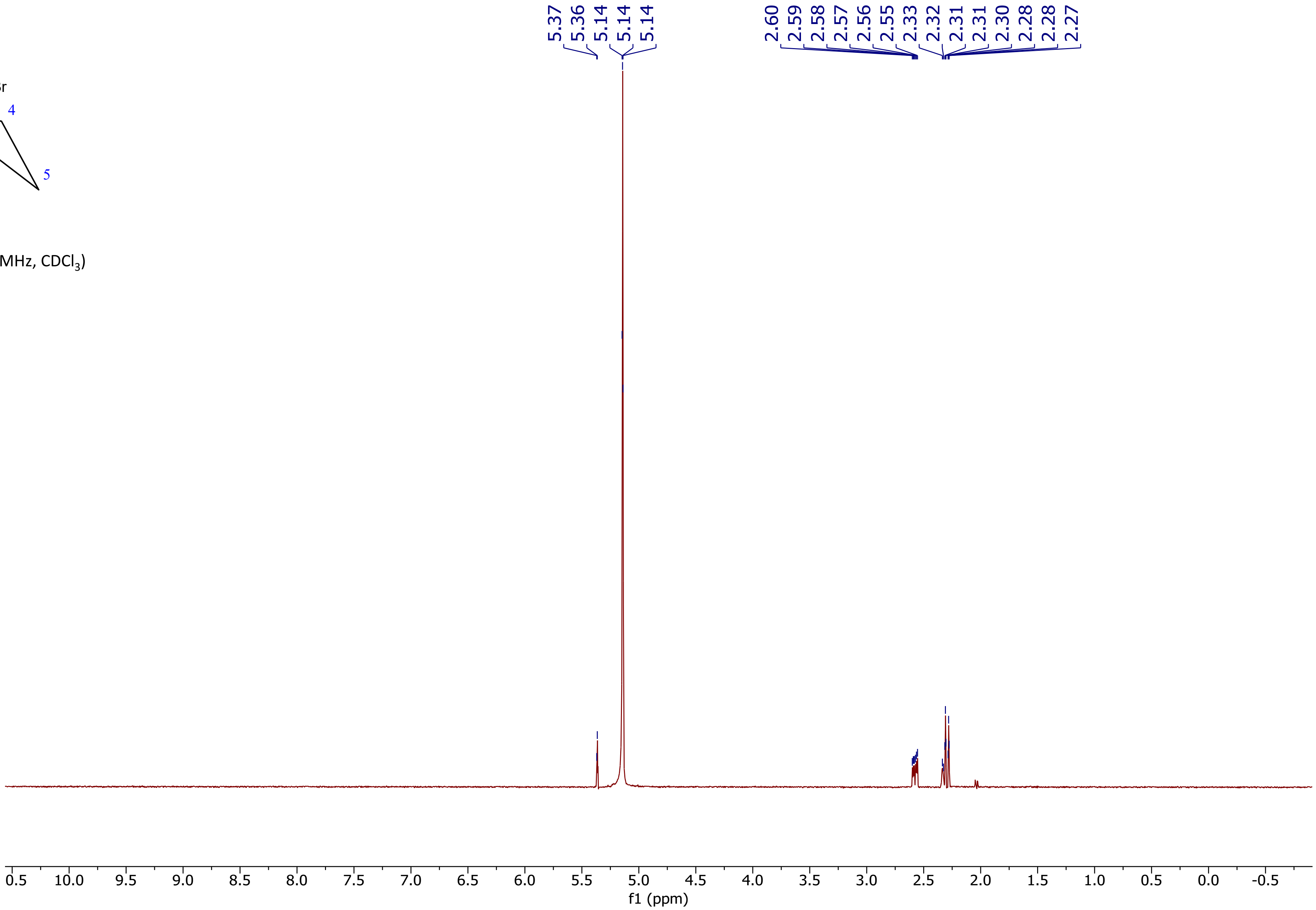

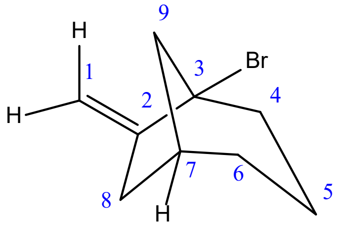

5

SEL-TOCSYs NMR(600 MHz, $CDCl_3$)

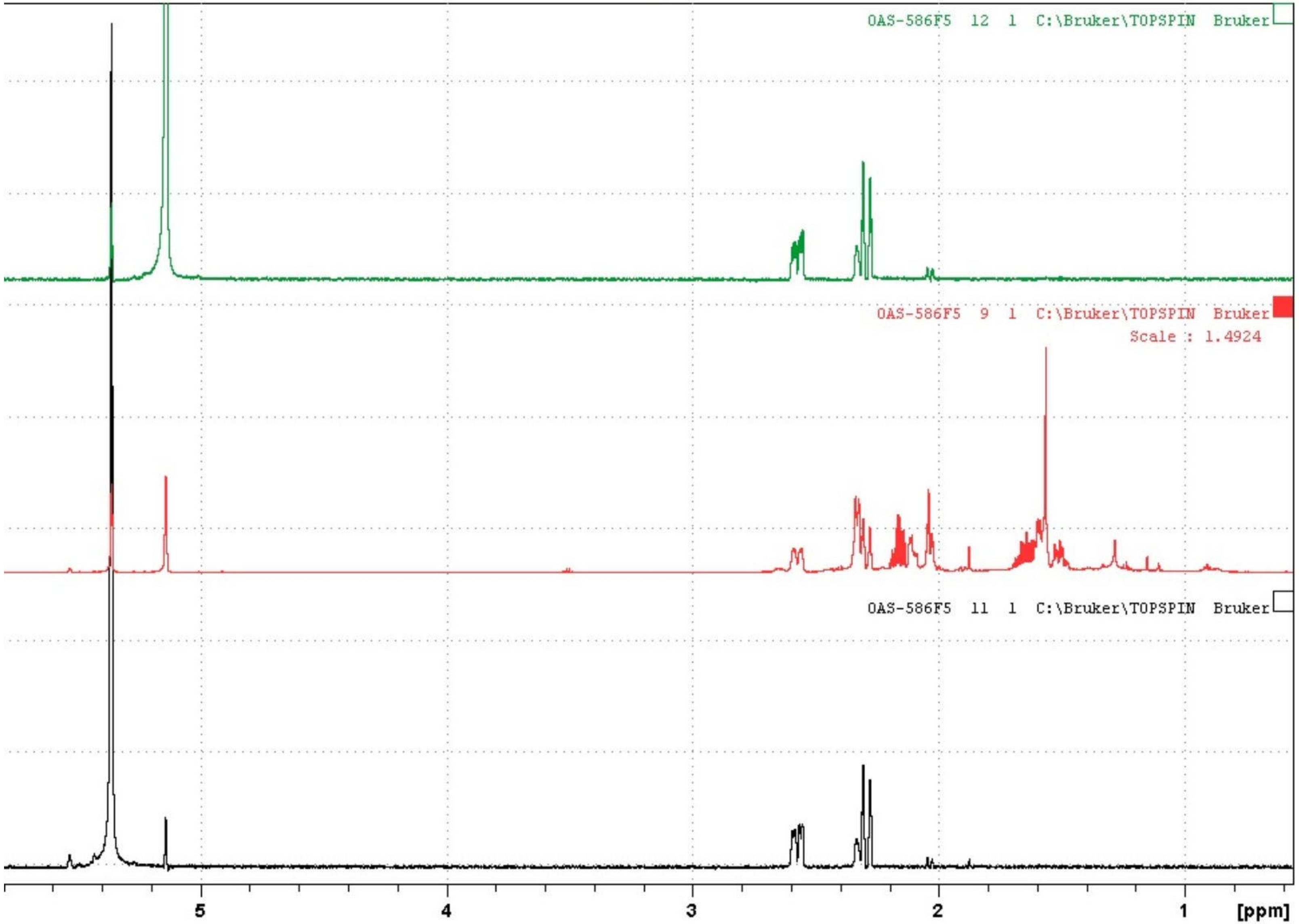

Supplement: Supplementary file 3 [file ol5c03430_si_003.pdf]
